# Lupus dermal fibroblasts are proinflammatory and exhibit a profibrotic phenotype in scarring skin disease

**DOI:** 10.1172/jci.insight.173437

**Published:** 2024-02-15

**Authors:** Suzanne K. Shoffner-Beck, Lisa Abernathy-Close, Stephanie Lazar, Feiyang Ma, Mehrnaz Gharaee-Kermani, Amy Hurst, Craig Dobry, Deepika Pandian, Rachael Wasikowski, Amanda Victory, Kelly Arnold, Johann E. Gudjonsson, Lam C. Tsoi, J. Michelle Kahlenberg

**Affiliations:** 1 Department of Biomedical Engineering,; 2 Department of Internal Medicine, Division of Rheumatology,; 3 Department of Dermatology, and; 4 Department of Computational Medicine and Bioinformatics, Department of Biostatistics, University of Michigan, Ann Arbor, Michigan, USA.

**Keywords:** Autoimmunity, Dermatology, Autoimmune diseases, Collagens, Skin

## Abstract

Fibroblasts are stromal cells known to regulate local immune responses important for wound healing and scar formation; however, the cellular mechanisms driving damage and scarring in patients with cutaneous lupus erythematosus (CLE) remain poorly understood. Dermal fibroblasts in patients with systemic lupus erythematosus (SLE) experience increased cytokine signaling in vivo, but the effect of inflammatory mediators on fibroblast responses in nonscarring versus scarring CLE subtypes is unclear. Here, we examined responses to cytokines in dermal fibroblasts from nonlesional skin of 22 patients with SLE and CLE and 34 individuals acting as healthy controls. Notably, inflammatory cytokine responses were exaggerated in SLE fibroblasts compared with those from individuals acting as healthy controls. In lesional CLE biopsies, these same inflammatory profiles were reflected in single-cell RNA-Seq of SFRP2^+^ and inflammatory fibroblast subsets, and TGF-β was identified as a critical upstream regulator for inflammatory fibroblasts in scarring discoid lupus lesions. In vitro cytokine stimulation of nonlesional fibroblasts from patients who scar from CLE identified an upregulation of collagens, particularly in response to TGF-β, whereas inflammatory pathways were more prominent in nonscarring patients. Our study revealed that SLE fibroblasts are poised to hyperrespond to inflammation, with differential responses among patients with scarring versus nonscarring disease, providing a potential skin-specific target for mitigating damage.

## Abstract 2

Lupus patient-derived fibroblasts are hyperresponsive to inflammatory stimuli, and fibroblasts from patients with cutaneous lupus erythematosus differentially respond to TGF-&beta; by enacting collagen upregulation.

## Introduction

Systemic lupus erythematosus (SLE) is an autoimmune disease driven by aberrant activation of innate and adaptive immune pathways that results in organ damage. Cutaneous lupus erythematosus (CLE), a dermatologic manifestation of SLE that also occurs as an isolated skin disease, affects approximately 70% of patients with SLE and can cause patient distress and disfiguration secondary to scar (1). Discoid lupus erythematosus (DLE) is the most prevalent CLE subtype, with skin lesions that typically heal with scarring (2). Conversely, subacute CLE (SCLE) is characterized by nonscarring disease (3). The heterogeneity accompanying cutaneous and systemic manifestations of lupus continues to be a challenge. Identifying cellular and molecular differences associated with either DLE or SCLE will likely lead to better interventions for managing SLE and scar-forming CLE lesions.

Studies have shown that type I IFNs are central to pathogenesis in SLE and CLE, and the level of IFNs correlates with disease severity, including in the skin (4). Recently, we identified in vivo that fibroblasts in SLE nonlesional and lesional skin exhibit evidence of exposure to inflammatory cytokine stimulation, including TNF-α, IL-1β, and type I and type II IFNs (5). Importantly, on the basis of receptor-ligand predictions, type I IFN–exposed fibroblasts are robust communicators with inflammatory cells in SLE skin (5). As fibroblasts are important regulators of the extracellular matrix, including collagen production, and play a role in regulation of inflammation and wound healing, it is critical to understand their role in SLE and CLE pathogenesis.

In this study, we first compared the inflammatory phenotype in fibroblasts isolated from individuals acting as healthy controls and from nonlesional skin of patients with SLE and CLE to explore differences in gene expression that occur in the disease. After identifying inflammatory profiles upregulated in lupus fibroblasts upon stimulation, we examined single-cell RNA-Seq (scRNA-Seq) data from lesional skin from patients with SCLE and DLE and looked for upstream regulators driving the differences between scarring and nonscarring disease. We then investigated the differences in gene expression between those with scarring cutaneous lupus and those with nonscarring disease in vitro. Finally, our data revealed that SLE dermal fibroblasts exhibit exaggerated cytokine responses, with collagen pathways predominating in those from patients with scarring skin disease. Thus, dermal fibroblasts in SLE skin are abnormal and exhibit differential responses based on the propensity for CLE lesions to heal with a scar.

## Results

### SLE dermal fibroblasts exhibit hyperinflammatory responses.

**Figure 1:**
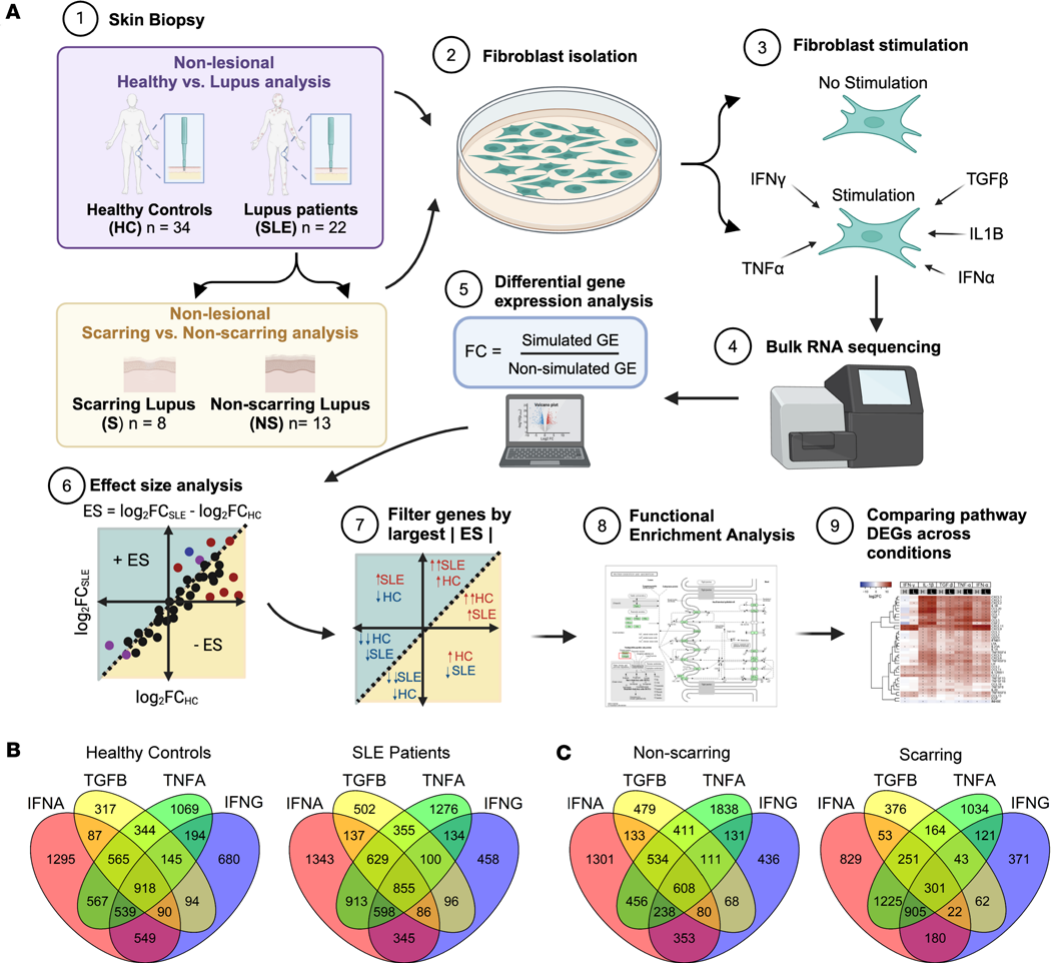
Schematic of fibroblast isolation, culture, and differential gene expression analysis workflow. (**A**) Two parallel studies were performed: one study compared nonlesional biopsies from individuals acting as healthy controls and patients with lupus, and another study that subdivided the patients with lupus into patients with damaged or scarring skin disease and patients with nondamaged or nonscarring skin disease and examined their fibroblasts from nonlesional biopsies. Fibroblasts were subjected to RNA-Seq at baseline (no cytokine stimulation) and stimulated with IFN-γ (IFNG), IFN-α (IFNA), TNF-α (TNFA), TGF-β (TGFB), and IL-1β (IL1B). After sequencing, gene expression fold change was determined for the stimulated versus unstimulated state. Effect size (ES) is plotted and calculated by taking the difference in fold change across disease groups. Pathway analysis was performed to identify pathways shared across cytokine stimulations and unique to individual stimulations. The genes in those pathways were plotted in heatmaps for all conditions. (**B** and **C**) The number of differentially expressed genes across stimulations in (**B**) healthy individuals and patients with lupus and (**C**) nonscarring and scarring patients.

In order to examine differences in fibroblast function, fibroblast cultures were grown from punch biopsy samples from healthy control skin and nonlesional lupus skin isolated from upper thigh (non-sun-exposed) biopsies. Patient characteristics are shown in Supplemental Table 1 (supplemental material available online with this article; https://doi.org/10.1172/jci.insight.173437DS1). At passage 2, they were treated with or without IFN-γ, IFN-ɑ, TNF-ɑ, TGF-β, or IL-1β followed by harvesting of RNA at 6 hours. These cytokines were chosen based on important upstream regulators of fibroblast differences in SLE identified by scRNA-Seq (5). The experimental setup is outlined in Figure 1A, with a similar protocol used for examining patients who do not scar and patients who scar with lesional healing. The number of differentially expressed genes across conditions is shown in Figure 1, B and C. We confirmed that similar fibroblast populations were present in healthy control and SLE cultures before and after cytokine stimulation (Supplemental Figure 1, A and B).

**Figure 2:**
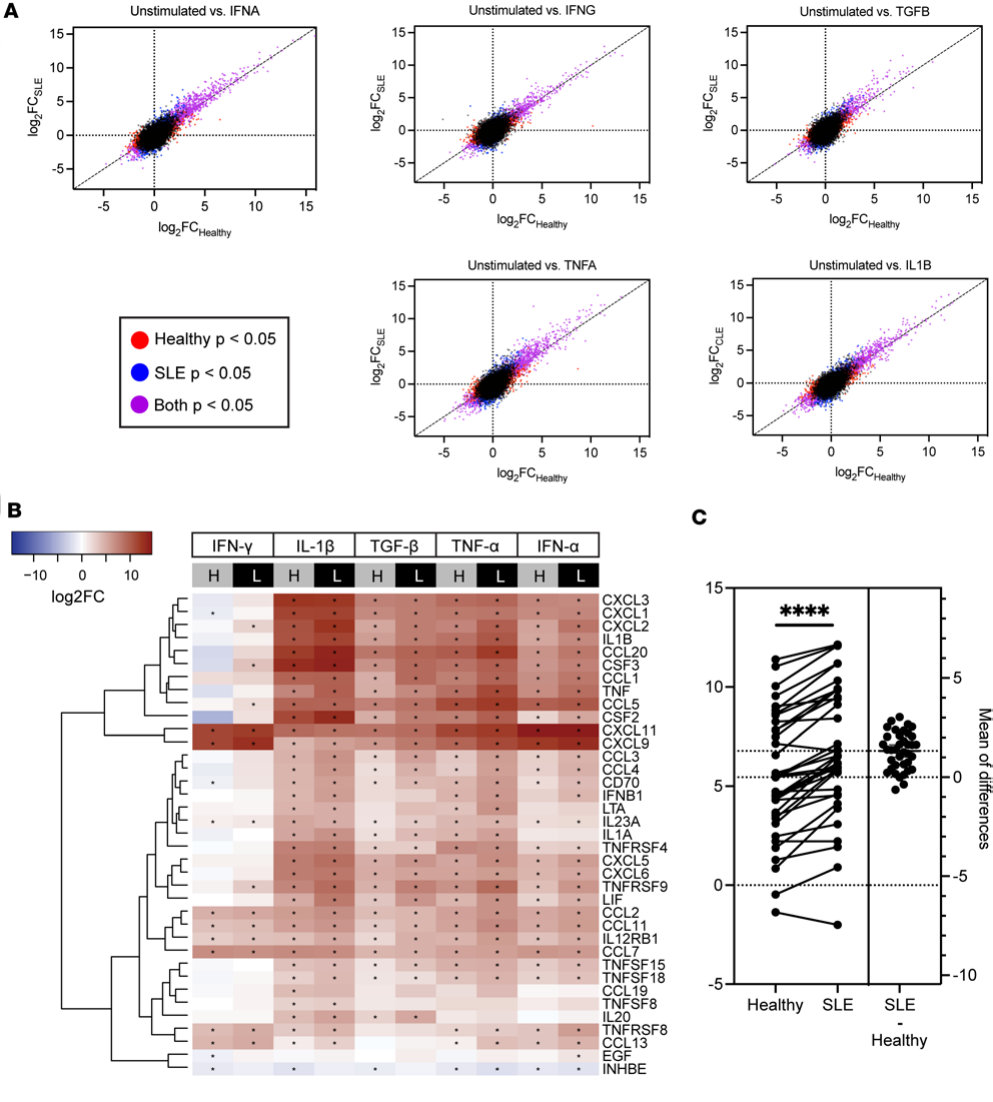
Fibroblasts from SLE skin are hyperresponsive to cytokine stimulations. (**A**) The effect sizes (log_2_FC in stimulated versus unstimulated conditions) under indicated cytokine stimulations of healthy (*x* axis) and SLE (*y* axis) fibroblasts. Diagonal lines represent what would be expected if there was no difference in gene expression between patients with SLE and individuals acting as healthy controls. (**B**) Heatmap illustrating the relatively higher upregulation of cytokine-cytokine receptor pathway genes in SLE fibroblasts (L) compared with those from individuals acting as healthy controls (H). Asterisks indicate significant log_2_FC in stimulated versus unstimulated conditions, with Wald’s test adjusted *P* < 0.05. (**C**) Quantification of the change in log_2_FC gene expression for each of the genes in the heatmap in **B** after TGF-β exposure for healthy individuals and patients with lupus using a paired *t* test for each gene. ****P < 0.0001.

As expected, we found differential gene expression after cytokine stimulation for both healthy controls and patients with SLE, with the largest effect size (ES) differences between the 2 patient groups for TGF-β, TNF-α, IFN-γ, and IFN-α stimulations (Figure 2A). As fibroblasts are important communicators with inflammatory cells in the dermis in SLE (5), we then focused on cytokine-cytokine receptor signaling pathway genes in each of the treatment conditions. As noted in Figure 2B, similar significant upregulation of chemokine (C-X-C motif ligand) CXCL family, chemokine (C-C motif) ligand family, IL, and TNF genes was induced across all treatment conditions, but a larger degree of upregulation was detected in SLE fibroblasts compared with healthy controls (FDR < 1 × 10^–5^). Intriguingly, the upregulation was exaggerated after exposure to TGF-β, with a paired *t* test for each gene showing a significant increase (*P* < 0.0001) in fold change (FC) between healthy and lupus (Figure 2C). Supplemental Table 2 lists all differentially expressed genes for each condition. These results indicate that while exposure to inflammatory cytokines upregulates inflammatory pathways in both healthy and SLE fibroblasts, the effect is magnified in SLE.

### TGF-β is an upstream regulator of inflammatory fibroblasts in scarring CLE.

**Figure 3:**
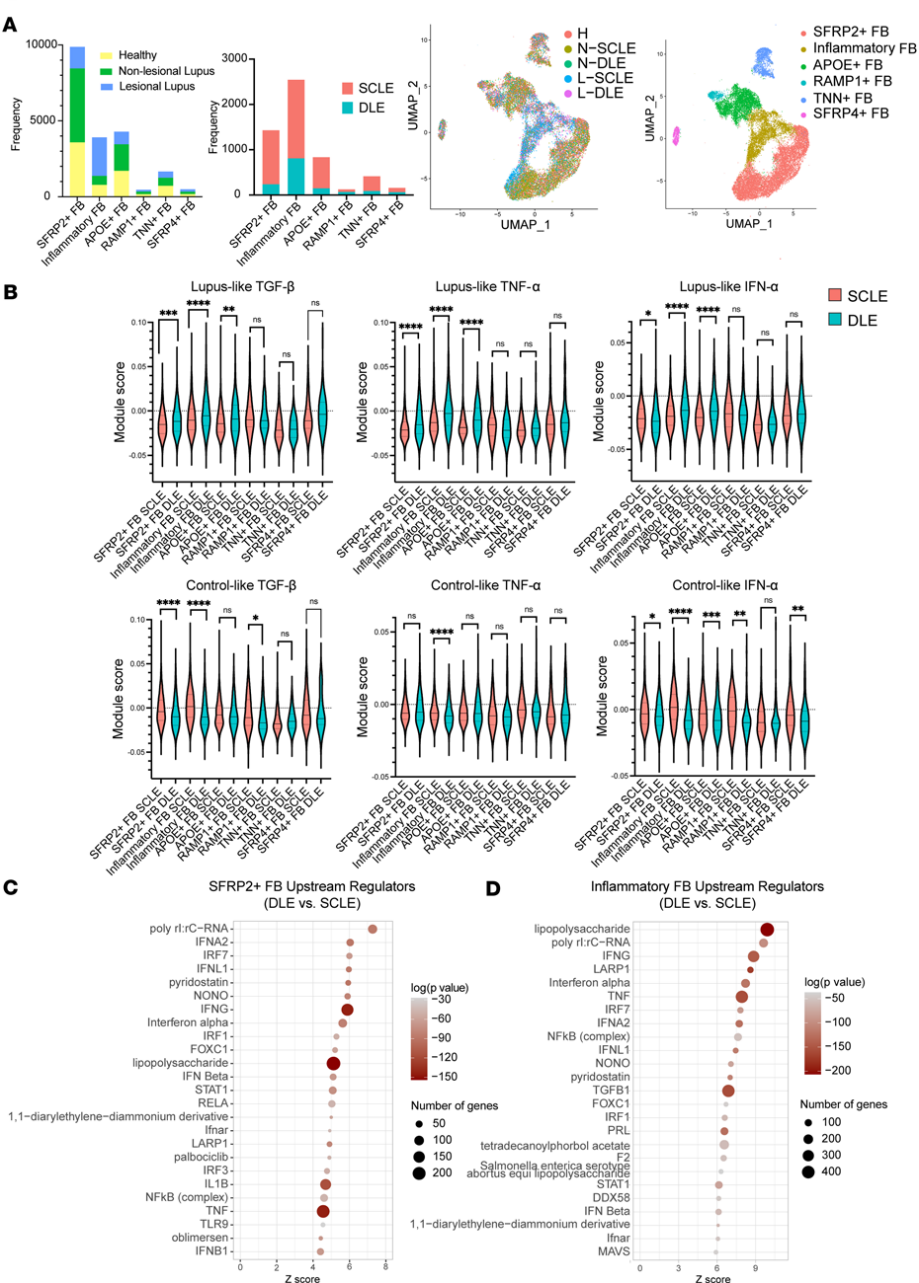
Cutaneous lupus lesional skin single-cell module scores and upstream regulators of dermal fibroblasts from patients with DLE (scarring disease) or SCLE (nonscarring disease). (**A**) Bar plots showing breakdown of samples by sample type (healthy, nonlesional lupus, and lesional lupus) and by disease type (SCLE and DLE). UMAP showing fibroblast clusters based on cell origin and disease type, including healthy control skin (H), nonlesional lupus skin (NLE), or lesional lupus skin (LLE) and by fibroblast cell subtype. (**B**) Module scores calculated using inflammatory profiles from control/lupus analysis of dermal fibroblasts. Significance level is denoted by asterisks (*P < 0.05, **P < 0.01, ***P < 0.001, ****P < 0.0001). (**C** and **D**) Top 25 upstream regulators plotted with the number of genes represented by dot size and colored by log(*P* value) for (**C**) the SFRP2^+^ fibroblast subtype and (**D**) the inflammatory fibroblast subtype.

To better understand the roles of cytokines in the upstream regulation of CLE disease pathogenesis and validate our findings, we then studied another set of samples using scRNA-Seq data acquired from nonlesional and lesional skin from patients with DLE (*n* = 5) versus SCLE (*n* = 8) (workflow schematic depicted in Supplemental Figure 2). Bar charts and UMAPs of the fibroblasts shown by disease type and cluster identification are shown in Figure 3A. Of note, a large inflammatory fibroblast population was identified that was derived primarily from lesional biopsies with smaller contributions from nonlesional skin. To asses how our skewed cytokine production in vitro may reflect the in vivo production of cytokines and chemokines in the skin, we generated modules reflecting the cytokine stimulations of the cultured fibroblasts in Figure 2, using genes that had a cytokine-induced effect that skewed higher for SLE samples (when compared with healthy controls) or higher for healthy control samples (when compared with SLE) for TGF-β, IFN-α, and TNF-α (Supplemental Table 3). We then overlaid these SLE-favored or healthy control–favored scores onto scRNA-Seq data from fibroblast subclusters in DLE and SCLE lesions, as shown in Figure 3B. Remarkably, the DLE fibroblast subclusters exhibited a skewing toward the SLE-like gene changes (Figure 3B), whereas the SCLE fibroblast subclusters exhibited a response more in line with genes favored in healthy control responses (Figure 3B). This was most prominent in SFRP2^+^, APOE^+^, and inflammatory fibroblasts. This is of interest as SFRP2^+^ fibroblasts are known to differentiate into myofibroblasts, which are critical for proper wound healing but can also be skewed to promote fibrosis and scar formation (6, 7), especially in inflammatory signaling environments (8). Indeed, when we performed upstream regulatory analyses using Ingenuity Pathway Analysis to determine the critical signals driving changes in SFRP2^+^ and inflammatory fibroblast subclusters in cells derived from patients with DLE compared with those from patients with SCLE, there was significant enrichment for IFN-γ and TNF as upstream regulators for DLE versus SCLE SFRP2^+^ fibroblasts (Figure 3, C and D) and more significant enrichment by TGF-β as an upstream regulator for inflammatory fibroblasts (Figure 3D). Importantly, this skewing toward a more robust inflammatory response in DLE was also replicated in bulk RNA-Seq of lesional skin biopsies (Supplemental Figure 3). These results suggest that TGF-β, TNF-α, and IFN-γ are important regulatory cytokines in SLE skin, and their presence may contribute to fibroblast activation, especially in DLE lesions. Furthermore, fibroblast interactions were visualized using CellChat (9), and numerous significant interactions were noted between SFRP2^+^ fibroblasts and inflammatory fibroblasts, especially after exposure to TGF-β (Supplemental Figure 4). We used pseudotime analysis to study the trajectory and relation between different fibroblast subtypes. We observed that inflammatory fibroblasts fall on the pseudotime trajectory between SRFP2^+^ and APOE^+^ populations (Supplemental Figure 5A). Specifically, our results illustrated higher expression of IRF1, STAT1, and IRF7 in inflammatory and SFRP2^+^ fibroblasts (Supplemental Figure 5B). All together, these data suggest that inflammatory fibroblast populations are likely derived from SRFP2^+^ fibroblasts by signals that promote activation of IRF1, STAT1, and IRF7.

### Dermal fibroblasts in DLE skin exhibit increased cytokine signaling and TGF-β–induced collagen responses.

**Figure 4:**
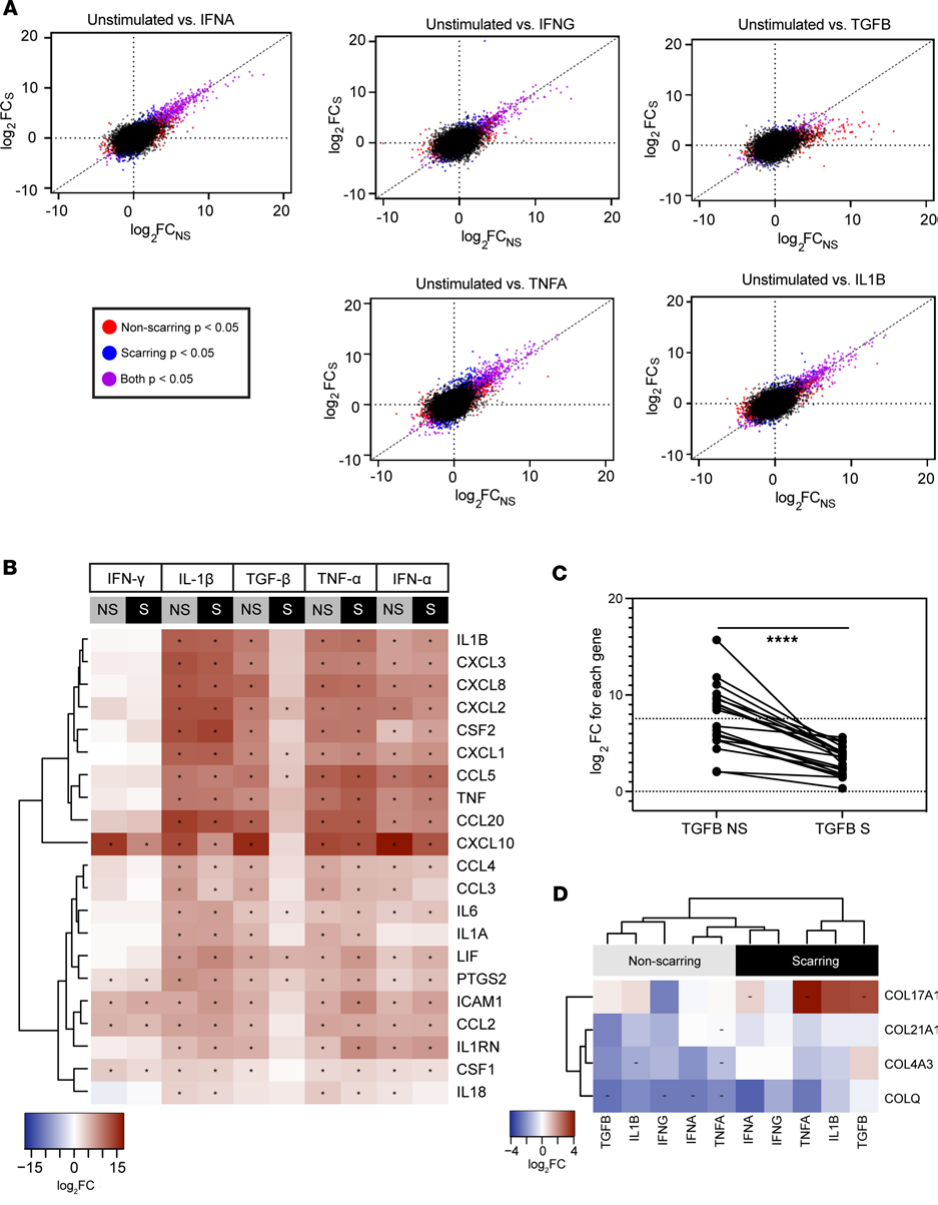
Dermal fibroblasts from patients with SLE with scarring CLE upregulate inflammatory and collagen transcriptional programs in response to stimulation. (**A**) The effect sizes (log_2_FC) in response to indicated cytokine treatments of fibroblasts from nonlesional skin of patients with SLE with nonscarring (NS, *x* axis) or scarring (S, *y* axis) CLE. Diagonal lines represent what would be expected if there is no difference in gene expression between scarring and nonscarring CLE. (**B**) Heatmaps illustrating the relatively higher upregulation of inflammatory pathway genes. (**C**) Quantification of differences in log_2_FC of differential gene expression for each gene between the nonscarring and scarring states using a paired *t* test. ****P < 0.0001. (**D**) The upregulation of collagen trimer pathway genes in scarring compared with nonscarring CLE. Asterisks indicate Wald’s test adjusted *P* < 0.05.

Given the differences observed between DLE and SCLE lesional fibroblasts, we then reexamined the bulk nonlesional fibroblast data used in Figure 2 to look at the differences in gene expression between fibroblasts isolated from patients with scarring and nonscarring CLE. Using the Cutaneous Lupus Erythematosus Disease Area and Severity Index (CLASI), a validated metric for CLE disease activity and damage (10), patients with SLE were separated into 2 categories: those that had evidence of scar from their CLE and those that resolved lesions without scarring. Comparisons in cytokine stimulation effect between scarring and nonscarring individuals underscored differences in ES (Supplemental Table 3), with the most notable difference showing a skewed ES in response to TGF-β stimulation in patients with CLE with scar-forming disease (Figure 4A). Comparisons of differentially regulated chemokines, cytokines, and other signaling molecules are shown as a heatmap in Figure 4B, where similar responses were denoted for most cytokines except for a robust difference that was apparent between nonscarring and scarring responses to TGF-β, a known fibrotic factor involved in myofibroblast activation (11). In nonscarring disease, TGF-β upregulated inflammatory genes to a greater extent. This suggests that upregulation of inflammatory genes upon TGF-β stimulation actually may induce potential protective effects against scarring. This skewing of response is quantified in Figure 4C using a paired *t* test for each gene in the heatmap by comparing log_2_FC in fibroblasts from nonscarring versus scarring disease and is significant, with *P* < 0.0001.

Further examination of the pathways dysregulated in scarring versus nonscarring patients following stimulation with our cytokine panel highlighted significant differences in the collagen trimer pathway (12, 13). In particular, *COL17A1*, a collagen found at the dermal-epidermal basement membrane zone and upregulated in fibrosing disorders (14), was upregulated in patients who scar, especially after stimulation with TGF-β (ES = 2.79, FDR = 4.19 × 10^–2^), TNF-α (ES = 4.01, FDR = 1.52 × 10^–4^), and IL-1β (ES = 2.62, FDR = 1.13 × 10^–4^) (Figure 4D). Similarly, subtle (nonsignificant) repression of potentially profibrotic collagens *COLQ*, *COL21A1*, and *COL4A3* (ES = 2.49, FDR = 9.10 × 10^–1^, ES = 2.02, FDR = 6.56 × 10^–1^, ES = 3.01, FDR = 7.49 × 10^–1^ respectively, see Supplemental Table 3) (15, 16) was noted in the nonscarring states across most inflammatory stimulations. Bulk RNA-Seq analysis of lesional biopsies supported a skewing of collagen upregulation in scarring versus nonscarring lesions (Supplemental Figure 3).

**Figure 5:**
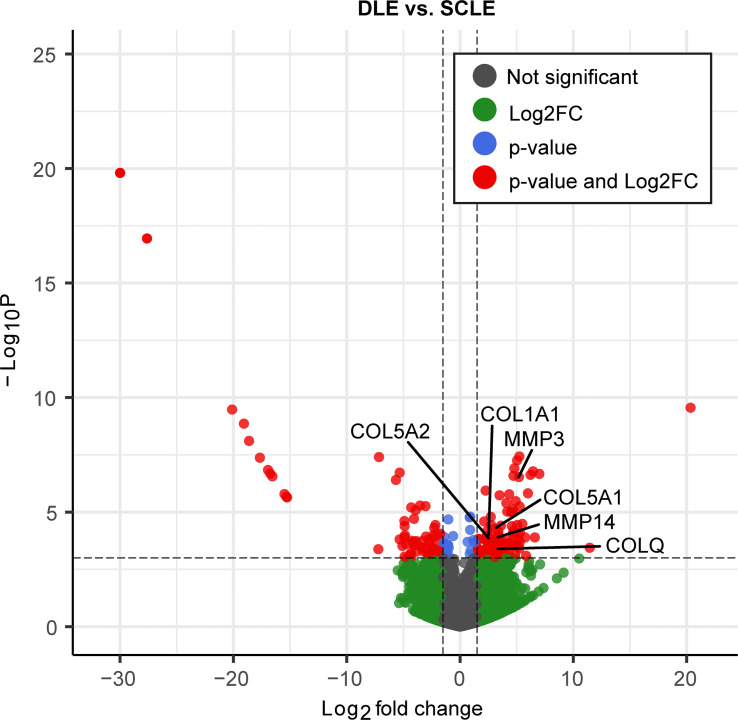
Differentially expressed genes identified via bulk RNA-Seq of lesional skin between scarring (DLE) and nonscarring disease (SCLE). Significance marked as a cutoff *P* value of < 10 × 10^–4^ and log_2_FC > 1.5. Highlighted genes are involved in collagen catabolic processes and fibril organization.

**Figure 6:**
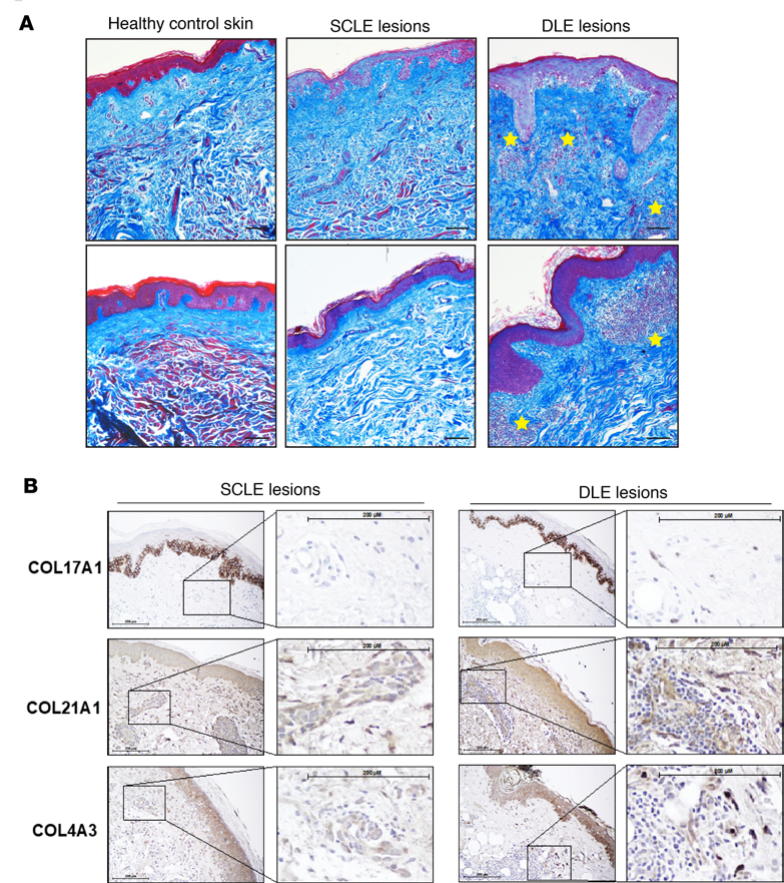
In situ detection of collagens in SCLE and DLE lesions. Formalin-fixed, paraffin-embedded tissue sections from skin were evaluated. (**A**) Masson’s trichrome staining of healthy skin and SCLE and DLE lesions revealed the in situ deposition and organization of collagen (blue) and muscle tissue (red), with inflammatory cell infiltration in DLE lesions marked with yellow stars. Scale bar: 100 μm. (**B**) Immunostaining for COL17A1, COL21A1, and COL4A3 in lesional skin from patients with lupus with DLE (scarring disease) or SCLE (nonscarring disease). Scale bar: 200 μm. Representative images from 3 to 5 patients from each subtype are shown (original magnification, ×200).

We then sought to confirm differences in collagen expression in lesional skin sections from patients with nonscarring SCLE (*n* = 20) and patients with DLE (*n* = 9), a phenotype of CLE associated with postlesional skin scarring using bulk RNA-Seq (Figure 5 and Supplemental Figure 3). Transcriptome comparisons revealed 153 significantly differentiated expressed genes (92 up- and 61 downregulated genes in individuals with scarring, see Supplemental Table 4). Consistent with our previous data, collagen catabolic processes and fibril organization were among the most significant functions enriched among the upregulated genes in DLE lesions (*P* < 1 × 10^–5^), including *MMP3*, *MMP14*, *COL5A2*, *COL1A1*, *COL5A1*, and particularly *COLQ* (FC = 3.27; FDR = 7.44 × 10^–2^) (Figure 5). Features of skin collagens were visualized by nonspecific and specific histological staining in order to corroborate these transcriptional changes. Indeed, increased collagen deposition, disordered collagen bundles, and inflammatory cell infiltration, indicative of a coinciding inflammatory and profibrotic wound healing process, were observed in DLE but not in SCLE lesions or healthy skin via Masson’s trichrome staining (Figure 6A). Specific confirmation and localization of *COL17A1*, *COL21A1*, and *COL4A3* was then performed by immunohistochemistry (Figure 6B). Increased staining for COL17A1, COL21A1, and COL4A3 was observed in the dermis of DLE lesional skin, compared with that of SCLE skin (Figure 6B, Supplemental Figure 6, and Supplemental Figure 7A). Of note, staining of these collagens was prominent in dermal fibroinflammatory infiltrates associated with DLE lesions, while this phenomenon was not observed in SCLE lesions (Figure 6B, inset). Unfortunately, no antibody was found that would reliably stain for COLQ. All together, these data suggest that, while dermal SLE fibroblasts exhibit skewed responses to inflammatory cytokines, skin-damaging DLE lesions are associated with TGF-β induction of profibrotic collagen pathways, whereas nonscarring SCLE lesions exhibit an inflammatory response.

## Discussion

Chronic skin inflammation is a unifying pathologic feature of SLE and CLE, yet scarring cutaneous lesions occur in only a subset of patients (17). The basis of divergent healing outcomes of CLE lesions is not clear. Abnormal proinflammatory interactions between epidermal and dermal cells in healthy-appearing lupus skin are critical in shaping the development of cutaneous lesions (5). While recent studies revealed altered production of, and responses to, cytokines and growth factors in SLE skin, the effect of these changes on local fibroblast responses and lupus-specific skin inflammation and damage is unclear.

Our study identifies increased upregulation of inflammatory responses in SLE dermal fibroblasts compared with those from individuals acting as healthy controls, indicating that these cells may be more susceptible to pathologic, overexaggerated inflammation. Intriguingly, we observed that dermal fibroblast subpopulations received increased inflammatory signals from IFN-γ, TNF-α, and TGF-β in scar-prone DLE skin, when compared with patients with nonscarring SCLE, and exhibited a differential response to TGF-β by upregulating collagen transcriptional profiles rather than inflammatory pathways. Increased presence of type XVII, type XXI, and type IV collagens was confirmed in the dermis of DLE lesions, with the most prominent staining localized to fibroinflammatory infiltrates. These data suggest a role for crosstalk between dermal fibroblasts and inflammatory cells, potentially mediated by TGF-β, TNF-α, or IFNs. Indeed, activation of cytotoxic lymphocytes in DLE but not SCLE has been reported (18), and our own previous data have also identified an important role for myeloid activation and fibroblast crosstalk in nonlesional SLE skin (5). These data are congruous with that in reports that scarless versus scar-forming healing in the skin in response to injury or damage hinges on suppression or activation of inflammatory signals and myeloid recruitment, respectively (19).

Skewed fibrosis is not unique to the skin of patients with SLE. In the kidney, fibroblast activation drives renal scar (20). Intriguingly, the IFN signature that exists in the skin may reflect the fibrosis progression in the kidney (21), suggesting that type I IFN exposure may modify fibroblasts in many organs to promote impaired wound healing and fibrosis in SLE. While inflammatory signaling is known to be important in the initiation of wound healing responses, the downregulation of this response and the correct balance between subtypes of TGF-β exposure may be critical for the avoidance of scar (8). Here, we found that patients with DLE have increased inflammatory signatures in their SFRP2^+^ and inflammatory fibroblasts when compared with those from patients with SCLE. In scleroderma, where skin fibrosis is a predominant feature, activation of transcription factors driven by IFNs and TGF-β contribute to the transition of SFRP2^+^ fibroblasts to a myofibroblast phenotype (6, 7). In DLE skin, these same cytokine drivers may also be skewing the wound healing response to promote excessive matrix deposition and scar formation rather than scarless healing, especially after TGF-β exposure. These data suggest that collagen pathway upregulation in dermal fibroblasts could be an important target for mitigating scars resulting from CLE.

A limitation of our study is that the examination of fibroblasts using 2-dimensional cell culture may not fully reflect the physiologically relevant behaviors of these stromal cells. For example, in addition to cytokines and other soluble mediators, the skin provides additional substrates and mechanical cues sensed by dermal fibroblasts (22). Our experimental conditions also are unable to ascertain the contribution of additional immunologic, epidermal, and stromal cells in the inflammatory skin milieu. In this context, it is reassuring that our single-cell data reflect our in vitro findings and that we don’t identify a shift in fibroblast subtype gene expression in vitro. However, future studies of fibroblasts in a 3-dimensional culture system and mapping of inflammatory cell interactions are warranted and will be the foci of further studies.

Together, our data provide important insight into the heterogeneity of dermal fibroblast responses in patients with SLE and identify dermal fibroblasts as a relevant cell population in modulating divergent healing outcomes among CLE subtypes.

## Methods

### Demographic information.

In this study, punch biopsies were obtained from University of Michigan patients enrolled in the Taubman Institute Innovative Program PerMIPA cohort with SLE (*n* = 22), divided into patients with scarring skin lesions (*n* = 8) and nonscarring skin lesions (*n* = 13), as well as a healthy control group (*n* = 34). Scarring disease status was determined using the damage score of CLASI, with a damage score of ≥1 indicating scarring disease (10). Male and female patients were part of the study (Supplemental Table 1 and Supplemental Table 5). Sex was not considered as a biological variable.

### Isolation and culture of human dermal fibroblasts.

Six mm punch biopsies were obtained from non-sun-exposed skin in individuals acting as healthy controls and nonlesional skin was obtained from patients with lupus, specifically from the upper thigh in both populations. Fibroblasts were isolated from biopsies via digestion of the dermis in 0.2% collagenase type II for 1 hour at 37°C, cultured in RPMI 1640 medium supplemented with 10% FBS until passage 2, and stimulated with various cytokines for 6 hours followed by harvest in TriPure for RNA-Seq. Cytokine concentrations were as follows: IFN-γ 5 ng/μL, IFN-ɑ 5 ng/μL, TNF-ɑ 10 ng/μL, TGF-β 10 ng/μL, and IL-1β 10 ng/μL. Confirmation that all subtypes of fibroblasts were present without skewing toward a particular subtype between healthy and lupus samples after culture and before stimulation in the nonlesional bulk RNA-Seq analysis is shown in Supplemental Figure 1A by examining the normalized counts for the top 10 cell markers for each fibroblast subtype. Supplemental Figure 1B shows that there was also no skewing toward a particular subtype between the control and lupus populations after stimulation.

### Lesional skin biopsy RNA-Seq.

Patients enrolled in the Taubman Institute Innovative Program PerMIPA cohort donated lesional skin biopsies that were bisected, and one-half was placed in RNA later (Thermo Fisher Scientific) and stored at –80^o^C until use. RNA was isolated using Zymo Direct-Zol kits, and RNA-Seq was performed by the University of Michigan Advanced Genomics Core on the Novaseq 6000 platform, with an average of >50 million read pairs per sample.

### Masson’s trichrome and immunohistochemical staining.

CLE skin biopsies from lesions of patients with SCLE or DLE were collected and fixed in formalin. Formalin-fixed, paraffin-embedded skin biopsy sections were evaluated for collagen deposition by Masson’s trichrome staining (Poly Scientific), while specific collagen protein levels were assayed for by chromogenic immunostaining with anti-COL17A1 (clone 2C3, Invitrogen), anti-COL21A1 (polyclonal, Invitrogen), and anti-COL4A3 (polyclonal, Invitrogen). Antigen retrieval was achieved by heating sections in sodium citrate buffer (pH 6.0) prior to antibody incubation. A minimum of 3 patients per disease status group were assayed, and representative images are shown.

### scRNA-Seq.

Skin biopsies were digested and libraries were prepared using the 10x Genomics platform as we have previously reported (5). The R package Seurat (v4.1.1) was used to cluster the cells. Cells with fewer than 100 genes or 500 transcripts or more than 50,000 transcripts or 10% of mitochondrial expression were first filtered out as low-quality cells. The NormalizeData function was used to normalize the expression level for each cell with default parameters. The FindVariableFeatures function was used to select variable genes with default parameters. The ScaleData function was used to scale and center the counts in the data set. Principal component analysis was performed on the variable genes. The RunHarmony function from the Harmony package was applied to remove potential batch effects among samples processed in different batches or from different donors. Uniform Manifold Approximation and Projection (UMAP) dimensional reduction was performed using the RunUMAP function. The clusters were obtained using the FindNeighbors and FindClusters functions with the resolution set to 0.5. The cluster marker genes were found using the FindAllMarkers function. Differential expression analysis was performed using the FindMarkers function. Module scores were calculated using the AddModuleScore function with default parameters.

### Gene expression analysis.

Genes were filtered for an average abundance of at least 1 read across all samples. Samples included in each analysis are shown in Supplemental Table 1. DEseq2 was used for expression normalization and to determine differentially expressed genes with FC of stimulated versus unstimulated | log_2_FC | > 0.6 and Benjamini-Hochberg adjusted *P* value with FDR < 0.05.

### Pathway analysis.

Pathway analysis was performed by filtering the genes with the largest positive and negative ES differences (log_2_FC_CLE_ – log_2_FC_healthy control_) to identify pathways shared across all stimulations and unique to individual stimulations. Positive ES differences in genes were either more upregulated in the disease group compared with the control group or less downregulated in the disease group compared with the control group (Figure 1A). Negative ES differences in genes were either more upregulated in the control group than the disease group or more downregulated in the disease group compared with the control group (Figure 1A). An ES difference of magnitude >1.5 was considered of interest. Using the hypergeometric test, we conducted functional enrichment analysis and examined the enrichment for pathways or functions compiled from Gene Ontology, Reactome, Biocarta, and KEGG (12, 13, 23–27). Out of 9,166 pathways, we investigated the functions that had fewer than 200 annotated genes and at least 3 annotated genes of interest expressed in our data set, with an adjusted *P* < 0.05. We then identified pathways that appeared to be significantly enriched across multiple stimulations as well as pathways unique to individual stimulations. The log_2_FC of stimulated versus unstimulated genes associated with these pathways was then plotted in a heatmap across stimulations and conditions.

### qPCR of TGFB1-stimulated dermal fibroblasts.

Primary dermal fibroblasts isolated from the nonlesional skin of patients with nonscarring (SCLE, *n* = 3) or scarring disease (DLE, *n* = 4), as well as from the skin of individuals acting as healthy controls (*n* = 3), was stimulated with 10 ng/mL TGFB1 (R&D Systems). RNA was extracted from cells using the Qiagen RNeasy Plus Kit after 24 hours of TGFB1 stimulation. cDNA libraries were generated using iScript (Bio-Rad), and real-time PCR was performed with SYBR Green Master Mix (Thermo Fisher Scientific) via the QuantStudio 12K Flex Real-Time PCR System (Thermo Fisher Scientific). The following primers were used: COL1A1 forward primer, 5′-AGTGGTTTGGATGGTGCCAA-3′; COL1A1 reverse primer, 5′-GCACCATCATTTCCACGAGC-3′; β-actin forward primer, 5′-CCTCGCCTTTGCCGATCC-3′; β-actin reverse primer, 5′-GCGCGGCGATATCATCATCC-3′. COL1A1 gene expression levels were normalized to β-actin, and FC relative to unstimulated healthy controls was calculated using the ΔΔCt method (28). Samples were compared via 1-way ANOVA.

### Statistics.

Negative binomial was used to model the read count data, and differential gene expression analysis was performed with DESeq2 using the Wald’s test (29). Multiple testing correction was conducted using the Benjamini-Hochberg method (30) that controls FDR < 0.05. Comparison of gene expression before and after treatment with TGF-β was completed using paired, 2-tailed Student’s *t* test. All analyses were performed in R. *P* values of less than 0.05 were considered significant.

### Study approval.

The studies described in this manuscript were reviewed and approved by the Michigan Medicine IRBMED (HUM00151834). Prior to participation in the study, all patients gave written, informed consent and were treated according to the Declaration of Helsinki.

### Data availability.

Fibroblast RNA-Seq data have been deposited in GEO (GSE237690). Values for all data points in graphs are reported in the Supporting Data Values file.

## Author contributions

Design of the study was performed by SKSB, JEG, LCT, and JMK. Sample and clinical data collection was performed by SL and AH. Conduct of experiments and data acquirement were done by SKSB, LAC, MGK, CD, DP, and AV. Data and bioinformatics analyses were performed by SKSB, FM, RW, and LCT. Interpretation of data and writing of drafts were performed by SKSB, LAC, KA, RW, LCT, JEG, and JMK. All authors read and edited the manuscript and agree with its contents.

## Supplementary Material

Supplemental data

Supplemental table 2

Supplemental table 3

Supplemental table 4

Supporting data values

## Version 1 02/15/2024

In-Press Preview

## Version 2 03/22/2024

Electronic publication

## References

[1] Mikita N (2011). Recent advances in cytokines in cutaneous and systemic lupus erythematosus. J Dermatol.

[2] Eastham AB, Vleugels RA (2014). Cutaneous lupus erythematosus. JAMA Dermatol.

[3] Stavropoulos PG (2008). Pathogenesis of subacute cutaneous lupus erythematosus. J Eur Acad Dermatol Venereol.

[4] Braunstein I (2012). The interferon-regulated gene signature is elevated in subacute cutaneous lupus erythematosus and discoid lupus erythematosus and correlates with the cutaneous lupus area and severity index score. Br J Dermatol.

[5] Billi AC (2022). Nonlesional lupus skin contributes to inflammatory education of myeloid cells and primes for cutaneous inflammation. Sci Transl Med.

[6] Tabib T (2021). Myofibroblast transcriptome indicates SFRP2^hi^ fibroblast progenitors in systemic sclerosis skin. Nat Commun.

[7] Feng Y (2020). Targeted apoptosis of myofibroblasts by elesclomol inhibits hypertrophic scar formation. eBioMedicine.

[8] Moretti L (2022). The interplay of fibroblasts, the extracellular matrix, and inflammation in scar formation. J Biol Chem.

[9] Jin S (2021). Inference and analysis of cell-cell communication using CellChat. Nat Commun.

[10] Albrecht J (2005). The CLASI (cutaneous lupus erythematosus disease area and severity index): an outcome instrument for cutaneous lupus erythematosus. J Invest Dermatol.

[11] Biernacka A (2011). TGF-β signaling in fibrosis. Growth Factors.

[12] Ashburner M (2000). Gene ontology: tool for the unification of biology. The gene ontology consortium. Nat Genet.

[13] The Gene Ontology Consortium (2021). The gene ontology resource: enriching a GOld mine. Nucleic Acids Res.

[14] Li D (2020). Identification of transcriptomic markers for developing idiopathic pulmonary fibrosis: an integrative analysis of gene expression profiles. Int J Clin Exp Pathol.

[15] Galindo CL (2014). Anti-remodeling and anti-fibrotic effects of the neuregulin-1β glial growth factor 2 in a large animal model of heart failure. J Am Heart Assoc.

[16] Motegi S-I (2017). Possible association of elevated serum collagen type IV level with skin sclerosis in systemic sclerosis. J Dermatol.

[17] Stull C (2023). Cutaneous involvement in systemic lupus erythematosus: a review for the rheumatologist. J Rheumatol.

[18] Wenzel J (2005). Scarring skin lesions of discoid lupus erythematosus are characterized by high numbers of skin-homing cytotoxic lymphocytes associated with strong expression of the type I interferon-induced protein MxA. Br J Dermatol.

[19] Sinha S (2022). Fibroblast inflammatory priming determines regenerative versus fibrotic skin repair in reindeer. Cell.

[20] Sato Y, Yanagita M (2017). Resident fibroblasts in the kidney: a major driver of fibrosis and inflammation. Inflamm Regen.

[21] Der E (2019). Tubular cell and keratinocyte single-cell transcriptomics applied to lupus nephritis reveal type I IFN and fibrosis relevant pathways. Nat Immunol.

[22] Smithmyer ME (2019). Bridging 2D and 3D culture: probing impact of extracellular environment on fibroblast activation in layered hydrogels. AIChE J.

[23] https://www.liebertpub.com/doi/abs/10.1089/152791601750294344.

[24] Kanehisa M (2023). KEGG for taxonomy-based analysis of pathways and genomes. Nucleic Acids Res.

[25] Kanehisa M, Goto S (2000). KEGG: kyoto encyclopedia of genes and genomes. Nucleic Acids Res.

[26] Gillespie M (2022). The reactome pathway knowledgebase 2022. Nucleic Acids Res.

[27] Kanehisa M (2019). Toward understanding the origin and evolution of cellular organisms. Protein Sci.

[28] Livak KJ, Schmittgen TD (2001). Analysis of relative gene expression data using real-time quantitative PCR and the 2(-Delta Delta C(T)) method. Methods.

[29] Love MI (2014). Moderated estimation of fold change and dispersion for RNA-seq data with DESeq2. Genome Biol.

[30] Benjamini Y, Hochberg Y (1995). Controlling the false discovery rate: a practical and powerful approach to multiple testing. J R Stat Soc Series B Stat Methodol.

