# Supplementary material for: Lupus dermal fibroblasts are proinflammatory and exhibit a profibrotic phenotype in scarring skin disease
Source: JCI Insight. 2024 Feb 15;9(6):e173437. doi: 10.1172/jci.insight.173437 (PMC10972618; doi:10.1172/jci.insight.173437)
Supplement: Supplemental data [file jciinsight-9-173437-s136.pdf]

Supplemental Figure 1. Fibroblast subtype populations present before (a) and after (b) cytokine stimulation. Visualization of raw normalized counts (a) and $\log_2$FC for DEGs (b) using the top 10 cell markers for each fibroblast subtype cluster identified in the scRNA-seq dataset.

A

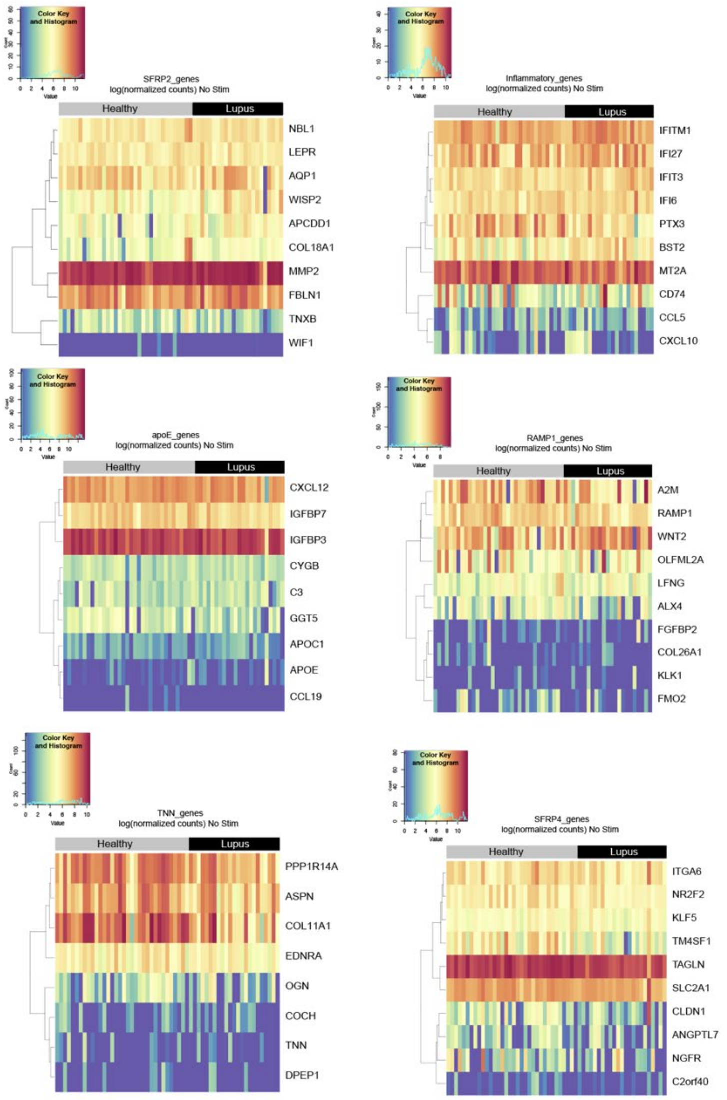

B

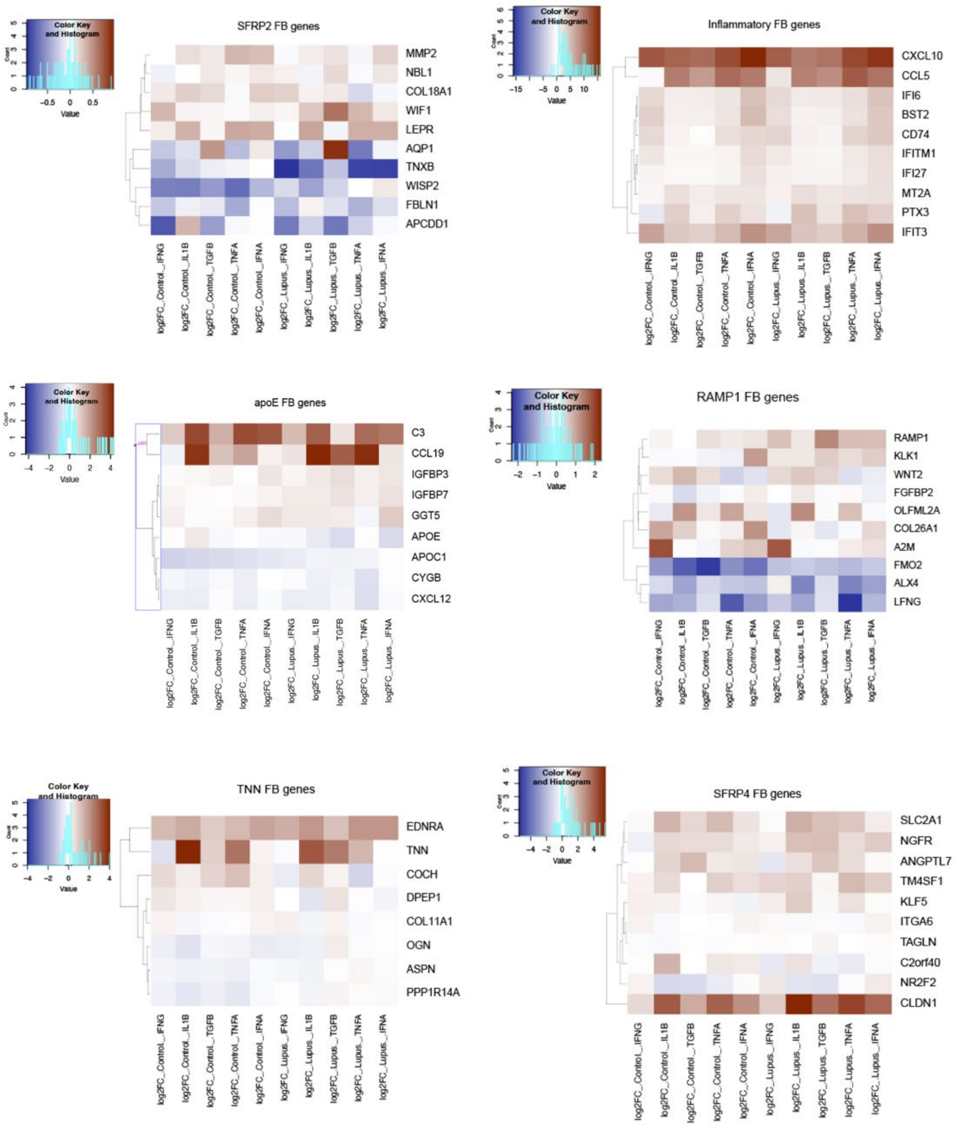
SFRP2 FB genes
MMP2
NBL1
COL18A1
WIF1
LEPR
AQP1
TNXB
WISP2
FBLN1
APCDD1
Inflammatory FB genes
CXCL10
CCL5
IFI6
BST2
CD74
IFITM1
IFI27
MT2A
PTX3
IFIT3
apoE FB genes
C3
CCL19
IGFBP3
IGFBP7
GGT5
APOE
APOC1
CYGB
CXCL12
RAMP1 FB genes
RAMP1
KLK1
WNT2
FGFBP2
OLFML2A
COL26A1
A2M
FMO2
ALX4
LFNG
TNN FB genes
EDNRA
TNN
COCH
DPEP1
COL11A1
OGN
ASPN
PPP1R14A
SFRP4 FB genes
SLC2A1
NGFR
ANGPTL7
TM4SF1
KLF5
ITGA6
TAGLN
C2orf40
NR2F2
CLDN1
Color Key and Histogram
Count
Value
log2FC_Control_IFNG
log2FC_Control_IL1B
log2FC_Control_TGFB
log2FC_Control_TNFA
log2FC_Control_IFNA
log2FC_Lupus_IFNG
log2FC_Lupus_IL1B
log2FC_Lupus_TGFB
log2FC_Lupus_TNFA
log2FC_Lupus_IFNA

Supplemental Figure 2: Workflow schematic for SCLE vs. DLE single cell RNA sequencing analysis.

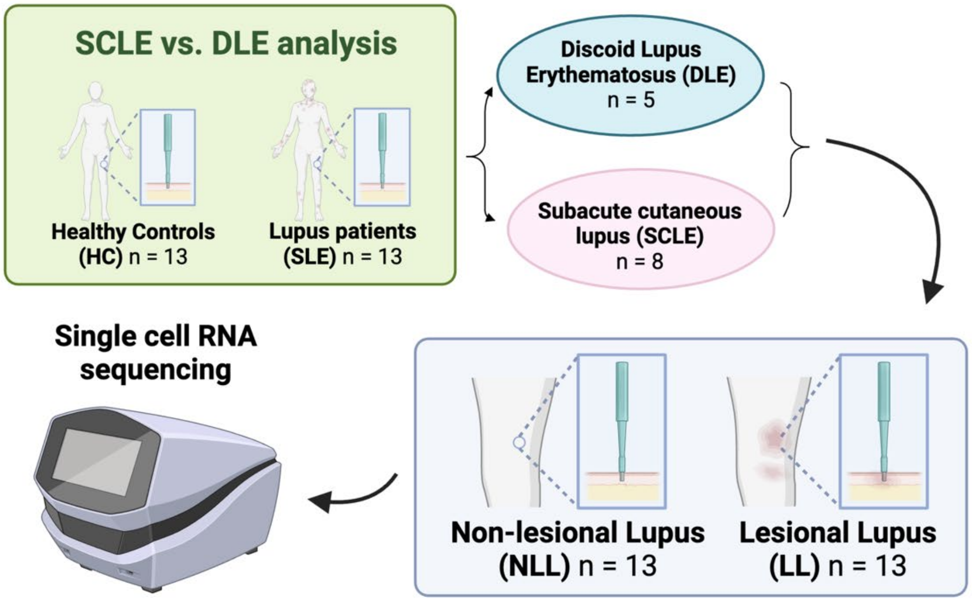

Supplemental Figure 3: Upregulation of inflammatory pathway and collagen genes skewed in fibroblasts are found in lesional biopsies from scarring or non-scarring CLE. Heatmaps illustrating the relatively higher upregulation of **a)** cytokine-cytokine receptor pathway, **b)** collagen pathway, and **c)** inflammatory pathwaygenes in lesional bulk RNA-seq data from patients with DLE (scarring) vs. SCLE (non-scarring).

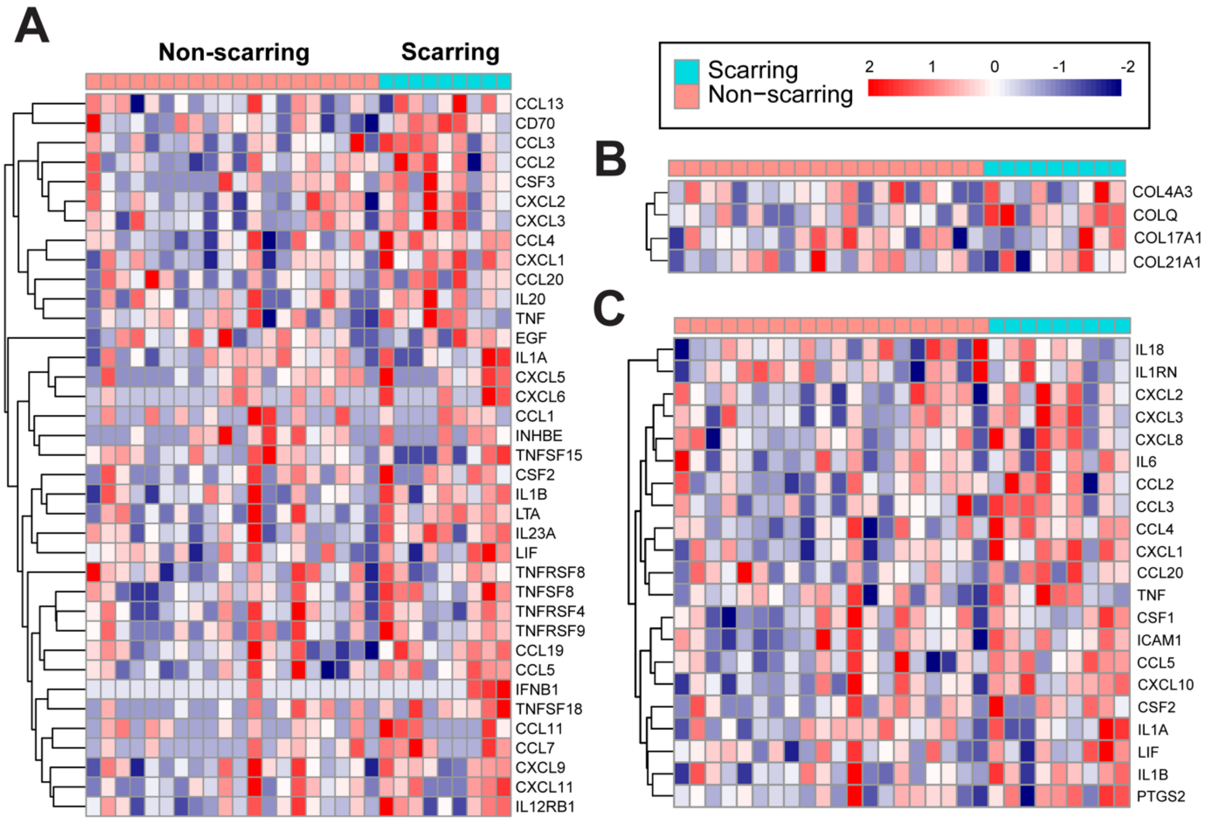

Supplemental Figure 4: Cell-cell interactions. Visualization of cell-cell communications from the single-cell data using CellChat (9). Node size indicates number of interactions and edge weight denotes strength of interaction. Numerous strong interactions are noted between inflammatory fibroblasts and SFRP2+ fibroblasts.

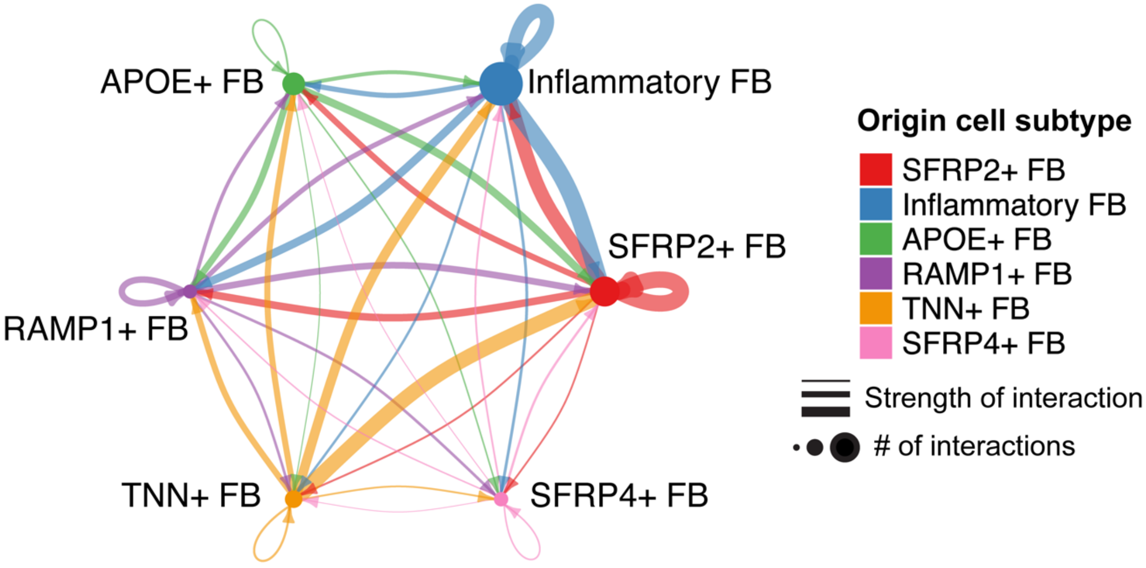

Supplemental Figure 5: Pseudotime analysis. Pseudotime trajectory between the various subtypes of fibroblasts (A) and heatmap showing gene expression for each of the subtypes across pseudotime (B).

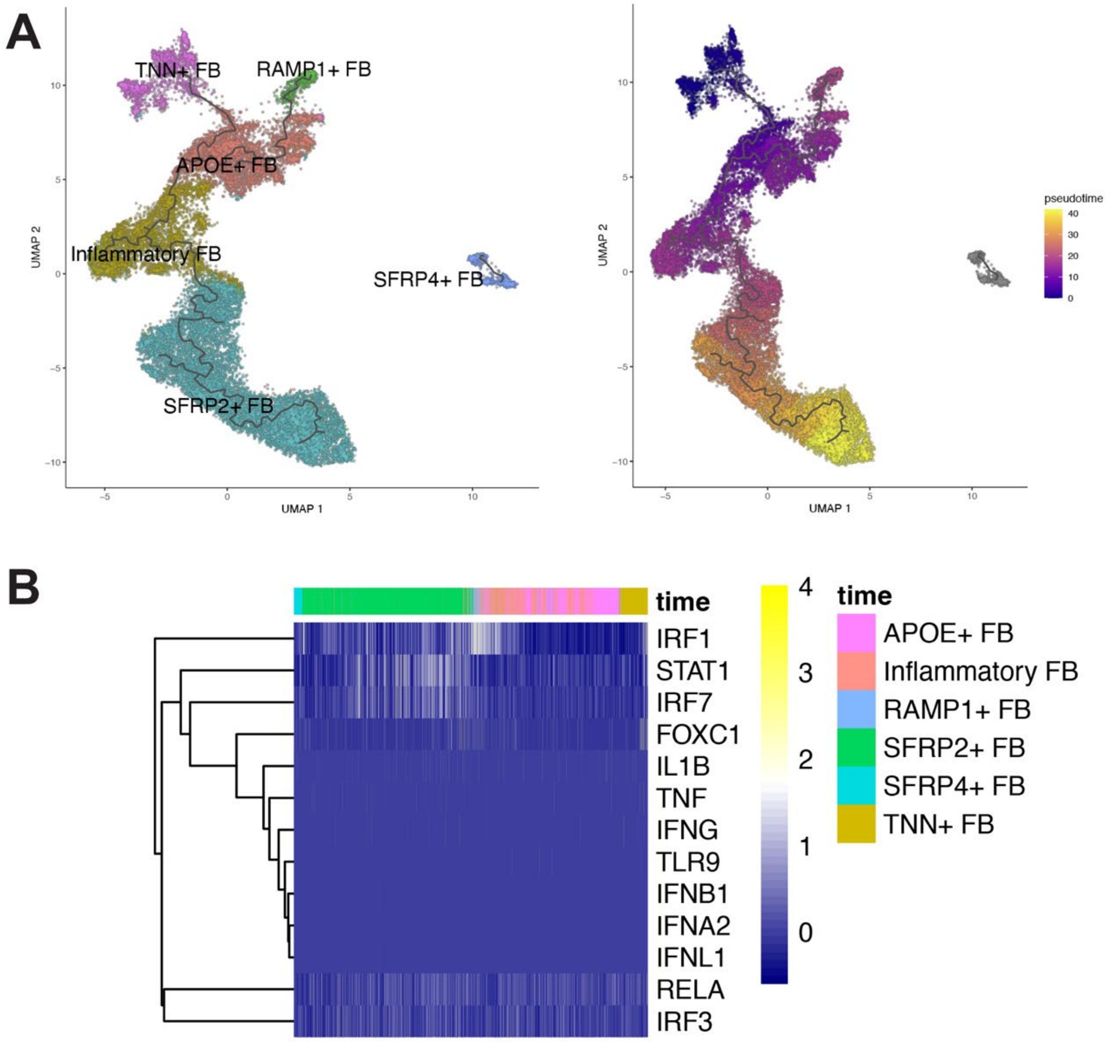

Supplemental Figure 6. Additional immunostaining for specific collagens in lesional skin sections from additional lupus patients with SCLE or DLE. Formalin-fixed paraffin-embedded tissue sections from lesional skin were stained for COL17A1, COL21A1, and COL4A3. Representative images from different patients of each subtype are shown at 200X magnification with a scale bar of 200 μm.

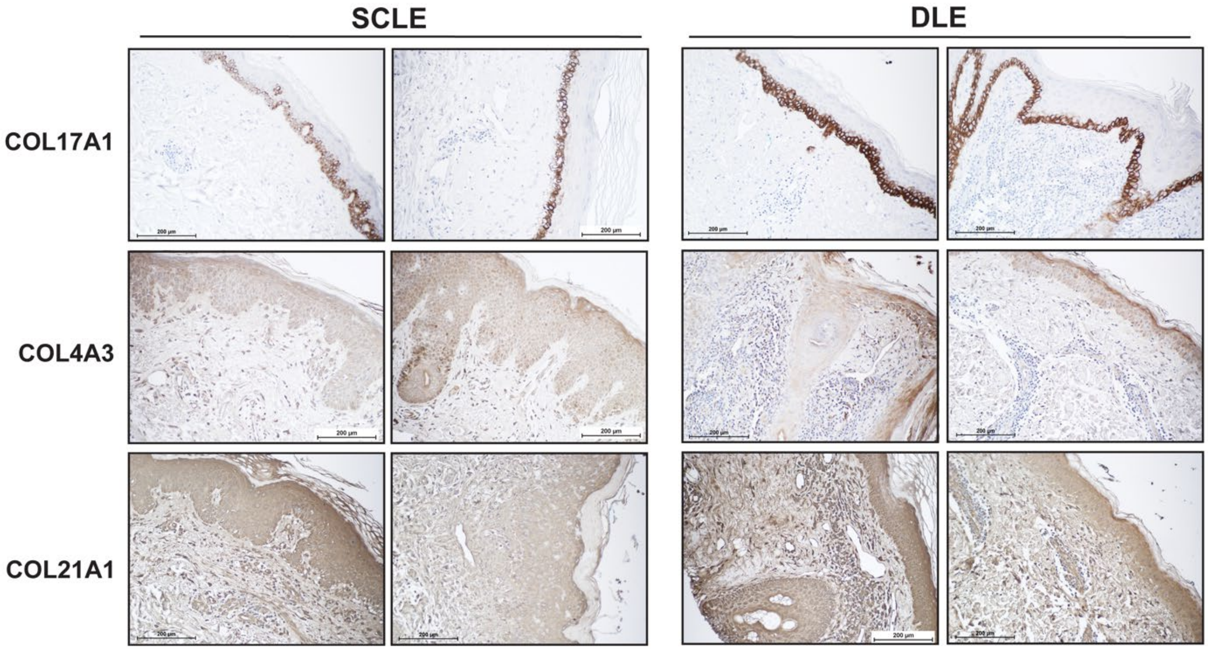

Supplemental Figure 7. TGF-β induces significantly high levels of COL1A1 expression in dermal fibroblasts from SLE patients with scarring skin disease. (A) Quantification of immunostaining for COL21A1, COL4A3, and COL17A1 from **Figure 6**. (B) Dermal fibroblasts isolated from healthy control (HC, n=3) volunteers or from the non-lesional skin of patients with non-scarring (SCLE, n=3) or scarring (DLE, n=4) CLE were treated with vehicle or stimulated with 10 ng/mL TGF-β for 24 hours. Collagen type I alpha 1 (*COL1A1*) expression was quantified by qPCR. Expression was normalized to β-actin and compared to untreated HC. Data were analyzed by one-way ANOVA; p-values for all comparisons are shown, and the statistically significant response is denoted by asterisks (**p-value < 0.01).

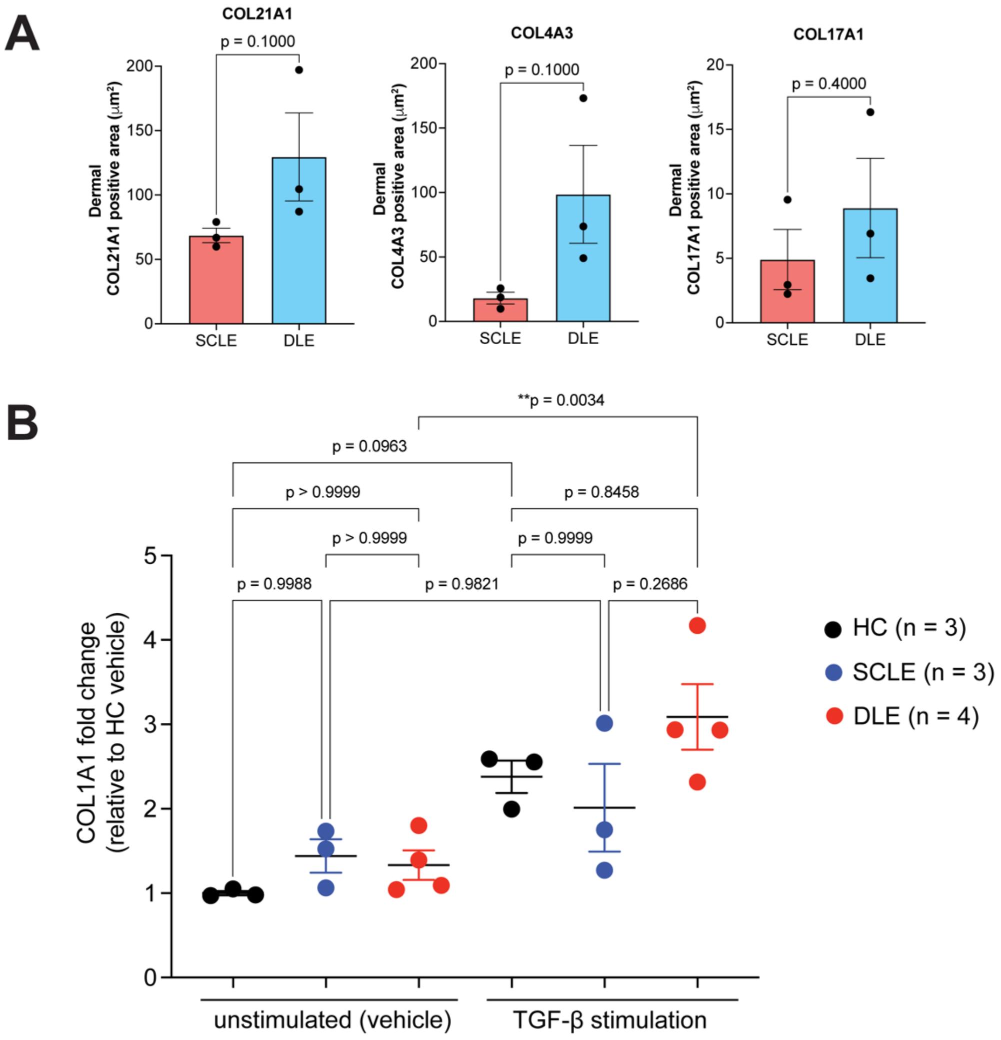

Supplementary Table 1: Demographic data for study population

	Control (*n* = 33)	Non-damage (*n* = 13)	Damage (*n* = 8)
**Age at skin biopsy, years, mean (SD)**	27.5 (19.8)	47.6 (13.0)	49.7 (15.0)
**Sex *(n)***			
Female	24 (73%)	11 (85%)	8 (100%)
Male	9 (27%)	2 (15%)	0 (0%)
**Ethnicity *(n)***			
Caucasian	31 (94%)	13 (100%)	3 (38%)
Black	3 (9%)	0 (0%)	6 (75%)
Hispanic	0 (0%)	1 (8%)	0 (0%)
Other/Unspecified	1 (3%)	0 (0%)	0 (0%)
**BMI, mean (SD)**	27.4 (5.5)	32.2 (7.2)	26.9 (6.0)
**Autoantibodies** *(n)***			
dsDNA (21)		5 (38%)	5 (63%)
Smith (21)		2 (15%)	3 (38%)
SSA/SSB (16)		2 (22%)	2 (29%)
RNP (15)		3 (33%)	0 (0%)
Chromatin (15)		3 (33%)	1 (17%)
Beta2-glycoprotein, anti-cardiolipin (15)		0 (0%)	3 (50%)
**Low C3 and/or C4 *(n)***		6 (46%)	5 (63%)
**Type of skin lesion* *(n)***			
**ACLE**		9 (69%)	4 (50%)
**SCLE**		3 (23%)	2 (25%)
**DLE**		3 (23%)	5 (63%)
**Lupus panniculitis**		0 (0%)	1 (13%)
**Lupus tumidis**		0 (0%)	1 (13%)
**CLASI-Activity, mean (SD)**		6.9 (13.3)	7.0 (6.0)
**CLASI-Damage, mean (SD)**		0 (0)	7.5 (3.6)
**Lupus nephritis class III or IV *(n)***		1 (8%)	5 (63%)
**SLEDAI, mean (SD)**		5.1 (5.5)	6.9 (5.5)

*Some patients had more than one lesion subtype in their rash history, all patients with ACLE in the "damaged" subgroup had concurrent hx of DLE.

**Some antibody data was not available for each patient

Gene	log2FoldChange	padj
CXCL10	10.96312251	1.20698E-38
CX3CL1	8.987904923	8.43699E-17
CXCL8	8.918660889	1.38175E-79
CXCL11	8.531203901	4.86983E-16
CCL20	8.384389483	1.40164E-21
OASL	8.270019379	1.80921E-34
RSAD2	7.985986838	2.88898E-18
GPR84	7.910249847	1.39939E-16
CXCL3	7.767424611	7.40624E-39
CCL5	7.676087616	6.54854E-30
SELE	7.612795184	3.6729E-06
CXCL1	7.089087118	7.96446E-53
CCL8	7.052575603	8.14375E-13
OAS1	6.681889017	2.30374E-24
CMPK2	6.67813158	3.52338E-17
CD69	6.666172172	4.72871E-08
BCL2A1	6.54846124	6.36706E-17
CSF3	6.537483717	3.50699E-06
LINC01539	6.345032827	7.06158E-10
MRGPRX3	6.328679599	1.34374E-07
CXCL9	6.322765801	3.1765E-06
G24578	6.16579548	1.19795E-07
TLR2	6.020946263	5.08478E-14
IL1B	5.898025243	2.144E-22
KYNU	5.872770065	4.04523E-17
GBP1P1	5.84305287	2.38692E-24
GCH1	5.701138106	1.94228E-27
GPR37L1	5.625879938	3.79436E-18
KDR	5.389528672	2.78092E-18
AC022509.2	5.383604904	1.8293E-23
SSTR2	5.348856024	1.04911E-12
NOD2	5.322246378	7.84288E-15
CXCL2	5.292846997	8.12304E-20
MEOX1	5.281162754	9.88496E-18
DCLK3	5.277687047	2.19845E-21
GBP4	5.192848744	6.08537E-23
KCNK9	5.053905143	6.99698E-06
CXCL6	5.015349453	5.87415E-30
IL33	4.98707369	2.19424E-29
CSF2	4.980017154	5.94518E-12
IDO1	4.978205243	1.13222E-11
TNF	4.967471711	1.91645E-15
AC022509.1	4.959964363	0.000822975

TNFAIP6	4.937679489	6.38778E-37
IFIT2	4.834214274	3.71492E-20
CCL1	4.827579427	0.004465009
CXCL5	4.816486208	7.6107E-19
TNFRSF9	4.780620698	5.07945E-13
ACOD1	4.742449412	0.004702949
AC083837.1	4.72277589	1.10285E-08
IL4I1	4.620181962	3.49778E-16
SLAMF8	4.605386806	2.37474E-14
AL078604.2	4.53321298	1.8911E-05
RASGEF1B	4.523143092	2.34102E-16
RCSD1	4.518070308	5.61012E-11
ICAM1	4.507297466	5.87415E-30
CCL7	4.486516554	5.53081E-27
AL356234.3	4.48002288	1.81582E-15
G26207	4.474432021	1.24413E-21
EXOC3L1	4.441205658	4.7613E-18
ELOVL7	4.414177544	3.03775E-07
KPNA7	4.406930628	5.85021E-07
SLC7A2	4.361174768	1.6314E-09
TNFSF10	4.351138434	3.07971E-11
AL356414.1	4.346354803	0.000323786
EFNA1	4.195145018	2.77895E-09
CLDN14	4.18791239	2.1334E-23
AC105450.1	4.181059334	0.000274432
MIR3142HG	4.167551636	8.17811E-05
TNFSF13B	4.163115431	4.55572E-13
XLOC_005900	4.158681825	7.56519E-14
NPTX1	4.135289622	2.73795E-18
ELF3	4.080586883	1.37997E-06
TNFAIP2	4.079698675	7.42037E-31
IFI44L	4.061561943	1.58234E-20
SAA2	3.994823374	4.56259E-14
C15orf48	3.989667392	7.35015E-25
CCL2	3.947305474	2.85629E-30
TNFAIP3	3.916791755	9.54228E-31
HERC5	3.896463055	1.49596E-10
MX1	3.88722442	1.95876E-14
ODAPH	3.861804952	3.93253E-06
CYP7B1	3.858690723	3.61555E-22
TCIM	3.822441673	4.83639E-37
HAS1	3.815299586	4.39589E-25
CD274	3.788344042	1.28007E-30

SPATA20P1	3.780978853	1.24303E-10
SOD2	3.764409135	3.6577E-29
CD38	3.761597687	0.001453888
IFIT3	3.72847741	4.03713E-15
IL1RN	3.721948854	3.06955E-11
AL135818.2	3.711012723	3.26983E-07
GBP7	3.700178364	0.024006421
EGR2	3.695921554	9.62156E-47
LINC02156	3.598833776	4.24078E-12
HSPD1P6	3.597072152	2.57147E-09
KCNF1	3.569926793	0.000244778
PHOSPHO1	3.563229943	5.47083E-13
GNA15	3.557291083	0.000769098
LINC02392	3.550898499	4.46019E-05
FTLP15	3.542168534	4.22466E-06
TRAF1	3.532713213	7.41634E-22
LIF	3.505275811	1.44381E-29
CXCR5	3.493772516	1.57219E-05
RORB	3.472429437	0.000688371
AC011511.5	3.462895055	0.000304597
EREG	3.443763245	1.42679E-07
IFIH1	3.436003566	1.25141E-14
FAM177B	3.417088869	1.65807E-21
GBP5	3.415070397	3.64992E-12
TTC4P1	3.409963062	1.95271E-11
AP001434.1	3.37534852	1.03471E-09
PIK3AP1	3.359806163	2.69778E-06
AC068205.1	3.35437823	0.035134347
DNAAF1	3.347431256	0.000174246
NR4A3	3.341296478	7.49727E-13
LCP2	3.337315567	0.036997889
EXOC3L4	3.337277749	3.88584E-08
AL135818.1	3.326635234	2.30343E-13
PTGS2	3.304103782	6.83683E-22
AP003119.3	3.282910234	2.00882E-11
LINC00944	3.27775924	5.30311E-09
GPR68	3.260683	1.34469E-59
G0S2	3.245831277	3.35047E-15
VCAM1	3.23805057	1.05723E-15
BIRC3	3.213451778	4.60727E-19
TP63	3.21272748	1.14596E-06
CCL11	3.210979664	1.85401E-14
CHI3L2	3.203996522	7.87479E-09

IL6	3.20109248	5.84778E-30
ICOSLG	3.196382596	4.57903E-09
BATF2	3.186900389	1.16229E-07
AC025580.2	3.182927423	9.02719E-10
ESM1	3.166569224	4.63724E-32
DDX58	3.154250467	4.25397E-16
TRIM36	3.149162683	1.24597E-09
CAVIN4	3.141320762	1.50482E-22
XLOC_000133	3.132042686	0.000107233
NLRP3	3.110633181	8.28915E-05
LRRC4C	3.10053176	7.79124E-11
ANGPTL4	3.095724855	3.12182E-39
NKX3-1	3.089469885	7.30079E-39
MX2	3.069154437	3.26549E-10
IFIT1	3.063515154	9.02023E-11
EGOT	3.04873265	0.010883476
PKP1	3.045356977	5.32888E-09
LIPG	3.043786581	4.01844E-22
TRPA1	3.041437675	1.30721E-23
AC022816.1	3.041002609	0.005749887
HRASLS2	3.032318293	0.00702696
TNFRSF4	3.001897344	0.000501277
AKR1B15	2.994339745	3.21072E-05
AC126178.1	2.991179813	2.78692E-07
AC083837.2	2.978255409	7.49465E-15
HOXD11	2.967687096	2.7842E-06
CFAP161	2.950839629	5.18339E-07
HSD11B1	2.915161065	9.2083E-09
TGFA	2.907163916	4.10503E-09
AC099489.1	2.903037665	0.00066502
CFB	2.885734842	1.18808E-12
SP6	2.878731077	2.8803E-05
RTP4	2.878568266	1.19176E-10
DNER	2.867876182	2.81104E-05
IER3	2.840528128	1.49926E-44
ZC3H12A	2.833132644	1.08856E-22
AP000640.2	2.829011778	0.000561127
NOTUM	2.82130535	0.008758794
SOST	2.820516651	3.13822E-09
PCDH17	2.813432657	0.000999879
AC026461.3	2.813251836	0.019419242
CHRNA9	2.799141222	1.53457E-13
G25440	2.793762755	1.29733E-07

AC009950.1	2.789592695	5.66728E-06
SPTBN5	2.785823936	8.07875E-05
SLFN12L	2.782919379	0.002827269
NKD1	2.780724156	6.10057E-08
KANK4	2.780284251	1.16874E-19
AC004988.1	2.774874747	9.5649E-05
GRIP2	2.767642152	6.08491E-06
TMEM52B	2.761007119	6.74835E-06
RGS16	2.737454352	1.05052E-16
SLC12A1	2.736036353	3.27124E-06
USP18	2.735536146	5.55774E-12
UNC13A	2.719045232	4.69176E-08
MMP13	2.71492744	0.00094444
AL355304.1	2.708413586	2.67718E-09
PDZK1IP1	2.705771332	1.5351E-06
RHBDL3	2.700551777	0.000157952
HBEGF	2.693384021	9.30009E-29
OAS3	2.675803923	4.49259E-11
AC099552.1	2.670472113	0.001679802
BTBD11	2.665738305	2.90512E-11
ACTN2	2.64789284	0.025696598
LINC01629	2.626242066	0.000284049
OAS2	2.624808022	1.3331E-10
FAM107A	2.622317878	0.008265495
BDKRB1	2.615845633	3.01938E-20
WNT1	2.611628006	0.047043295
AC005549.3	2.608471826	0.000145767
EBI3	2.603645679	2.68816E-06
PEG13	2.596357193	1.19601E-05
KLHL38	2.596328143	5.4644E-06
FOSB	2.589365014	5.37429E-07
USP30-AS1	2.585550347	8.45552E-05
PKN2-AS1	2.576199052	7.07692E-07
P2RY6	2.575757312	2.26677E-21
IL20	2.573359548	8.19463E-08
PLAC4	2.571647535	3.59327E-05
NIPAL4	2.564147339	5.55899E-12
AC012020.2	2.563508314	0.004278543
AC131649.2	2.562832965	0.005266034
STRA6	2.560396975	5.21469E-12
IL22RA1	2.558872278	5.90985E-08
SERPINA9	2.54418348	4.45107E-08
RASL11B	2.524889904	1.60113E-20

ITGB6	2.520826869	0.003609155
CLIC6	2.519292026	9.0147E-06
HERC6	2.511939093	9.07828E-09
IFI44	2.504442082	4.63998E-16
CFAP58-DT	2.497930002	1.21415E-09
AL162274.1	2.494572376	1.36496E-05
CCL3	2.476537004	0.001614972
IL32	2.476253301	5.10239E-21
P2RY2	2.469398319	5.72473E-06
XLOC_002530	2.46894345	6.31816E-05
OLR1	2.464999994	8.43699E-17
BTC	2.464201702	0.006581804
RELB	2.460876056	1.67221E-29
DRAXIN	2.453494886	4.00805E-05
AL445490.1	2.44921877	0.036336377
XLOC_011331	2.445296535	0.000472029
AC044781.1	2.442018227	0.000458879
PPP4R4	2.440099078	1.44408E-07
AIM2	2.439858277	0.004554534
IFI30	2.436493682	0.01428142
PAIP1P1	2.435686348	0.001628626
G11966	2.433718191	0.01568741
ROBO4	2.432561162	0.000292789
LST1	2.424792215	9.81545E-05
GBP1	2.423402271	4.26129E-12
S1PR5	2.421977096	2.89821E-16
EPSTI1	2.419112108	1.25916E-10
HELZ2	2.417103243	5.42817E-10
USP43	2.413964192	8.66053E-05
CACNA1G	2.412535024	2.07703E-07
PRAL	2.409148225	0.018538747
AL158166.1	2.405633556	5.77024E-05
XLOC_012049	2.402864286	1.15107E-09
IL15RA	2.399991842	5.83242E-13
CTSS	2.396492051	6.70085E-11
HTR1D	2.385199187	9.69836E-07
PGBD5	2.384395095	0.001064451
XLOC_000358	2.382780594	4.41202E-07
DCSTAMP	2.374211501	3.24946E-05
IRAK2	2.369207476	1.51295E-19
XLOC_000283	2.366950329	0.009341297
AL033397.2	2.344356466	0.004438834
AC110015.1	2.340690236	1.99936E-08

AC099494.3	2.339840237	8.24066E-06
AL049825.1	2.339562376	4.66667E-09
LINC02470	2.323875292	0.002033755
DIRC1	2.321170101	8.72448E-07
IL11	2.316059046	1.68998E-18
AL391056.1	2.313407988	2.46394E-13
FAM124A	2.311535754	1.12135E-07
TMEM51	2.311043899	5.96319E-12
IL12RB1	2.309915788	0.000103463
IL10RB-DT	2.30984346	1.32496E-06
TSPAN2	2.307301719	1.64137E-39
LINC01828	2.304717642	0.008739801
IRF7	2.298016996	1.7458E-13
TMEM88	2.276614254	0.027032293
SLCO5A1	2.276390446	2.93039E-05
RND1	2.274079082	0.000230714
XLOC_003146	2.274011237	6.92815E-28
LMO2	2.27189239	0.009680923
MMP12	2.271115411	3.02251E-09
AC009152.3	2.26589135	7.98831E-10
AC018644.1	2.262273022	2.19647E-06
KIAA0040	2.26114438	4.55338E-20
TBILA	2.260064345	0.006198066
PRRG4	2.259275596	0.011231705
AC005840.3	2.25760646	0.003293904
CLDN1	2.248359157	1.00268E-10
SMAD7	2.238934414	7.82202E-29
AC005899.8	2.229094592	0.000509566
TLR5	2.215778845	7.17766E-06
XIRP1	2.21529732	0.008338425
AC006249.1	2.210869666	8.74562E-05
LDLRAD4	2.205298832	1.60727E-14
AL357673.2	2.19736469	0.005576723
AC097451.1	2.19193582	2.09642E-10
ST6GAL2	2.190629416	0.001981018
AC099494.1	2.188402112	6.68324E-06
SNX10	2.185119187	0.00889055
TSLP	2.182901957	3.92015E-07
PTHLH	2.182348416	5.23327E-17
CD83	2.180449491	1.19176E-10
AC004817.3	2.178917113	0.000149371
KCTD11	2.178612167	2.58618E-34
PLA1A	2.175199485	0.029617134

BCL2L14	2.172946517	0.007742678
AC016397.2	2.169861519	5.69109E-08
C1QTNF1	2.156774519	4.31776E-14
CACNA1G-AS1	2.155806738	0.016728276
TRABD2B	2.154847781	0.02737114
CD70	2.154080949	4.97388E-10
TRAPPC3L	2.146115164	6.93042E-05
AC007686.1	2.13763389	0.000105618
RASL12	2.137204347	0.001014711
NFATC2	2.130009704	9.04457E-13
HCK	2.124511675	2.10533E-08
C3AR1	2.122297791	0.001069092
PARP12	2.119661942	8.12304E-20
BHLHE40	2.117330543	2.53109E-22
DHX58	2.115753499	3.50405E-10
AC080014.1	2.109240554	1.22034E-05
ETV7	2.109027486	3.54396E-08
ATF3	2.10842272	1.76757E-12
NFKB2	2.105493957	1.00736E-18
NFKBIZ	2.103254516	6.43113E-27
MMP1	2.102868901	3.35214E-12
IL34	2.099572768	3.05595E-09
TYMP	2.095776796	5.76036E-14
SOCS1	2.093054482	8.96317E-05
TLR3	2.092155666	4.5932E-07
LEFTY2	2.084457438	0.001300834
AP002852.1	2.07318675	9.32897E-06
QRFP	2.072144773	7.85824E-05
P2RY1	2.071823231	0.000370851
TFPI2	2.071737506	3.02351E-21
BEST3	2.069088114	0.001815527
AC131009.2	2.058635218	0.009359025
THEMIS2	2.055051406	1.47985E-11
NR4A2	2.051277352	4.57903E-09
CYP27B1	2.049310962	3.27546E-06
AL024508.1	2.034282807	0.021811603
SLC39A14	2.021941604	6.12529E-20
CFAP45	2.019920411	1.33734E-07
HLA-DOB	2.019240476	0.005574733
DDX60	2.014326966	1.8565E-12
MFSD2A	2.008836076	3.38853E-12
SEMA4D	1.993248155	4.3651E-07
KCNJ15	1.990874123	8.68017E-24

C1orf147	1.986919038	0.024289653
RELT	1.983531379	1.58065E-20
AC009152.1	1.982819793	0.027280807
SLC22A2	1.982574551	0.026714397
XAF1	1.981437924	1.46418E-16
FYB1	1.979500895	0.005468718
FAM19A3	1.976727424	4.42265E-05
CLEC1A	1.971668233	8.66053E-05
FOXP1-IT1	1.970214862	5.80274E-07
PARP14	1.967827556	7.34789E-12
44258	1.966698881	1.08865E-23
AC008083.1	1.966551819	0.000106236
RN7SL368P	1.964762231	0.013374582
IFNE	1.963226455	0.000102409
PMEPA1	1.958450413	6.84333E-32
IRF1	1.956186029	1.69986E-08
SLC12A7	1.946743894	4.38916E-10
RN7SL834P	1.944622742	0.024774205
TNFAIP8	1.943095395	1.03557E-18
AC020913.3	1.942236861	0.006473472
AMPD3	1.937572107	2.92397E-14
PHACTR1	1.93702651	1.46534E-16
NFKBIE	1.935540978	4.13199E-11
XXYLT1-AS2	1.935522674	0.000874584
CD34	1.934329729	1.14712E-08
TRIB1	1.934188455	1.33562E-06
GFPT2	1.933733238	2.00055E-32
RNF19B	1.933218294	1.23022E-14
ADAP1	1.93301867	3.79939E-11
SLC19A2	1.932621764	1.54294E-29
SAMD9L	1.924003048	3.04428E-11
BMP2	1.92240212	1.03296E-05
CSRNP1	1.920267225	8.92345E-28
AL592546.1	1.919878816	0.011618051
ISG20	1.919340198	1.25003E-07
CCL4	1.917584213	0.019684448
LINC00640	1.916386349	0.000156834
LINC00622	1.916313347	0.003845738
LINC02086	1.912103294	1.58903E-05
WWC1	1.911666268	1.14447E-10
SAA1	1.909163708	5.822E-09
TNIP3	1.907662886	1.52129E-06
IL18BP	1.905076858	5.99773E-07

RASSF5	1.904486754	1.7818E-05
HSPA6	1.903769114	0.025382144
MSC	1.903633749	7.71179E-19
VNN1	1.90086761	0.003198674
RHOH	1.899934519	0.001669661
AC009949.1	1.898303789	0.009572677
AL158166.2	1.896554044	0.032924027
XLOC_007602	1.89357291	7.77086E-05
ENC1	1.892765284	3.13151E-37
LRRC8C	1.892495783	1.07559E-30
SERPINB2	1.890564154	1.50007E-11
LRIG1	1.890234739	1.70727E-19
PROX1	1.889678881	0.000139541
TBX3	1.887721742	1.9816E-39
AL356234.1	1.884014154	0.004232781
DLL1	1.882239545	0.000162148
APOL4	1.879974567	0.003180022
SPACA6P-AS	1.877491501	0.011755896
JUNB	1.875264973	5.60712E-27
PTGER2	1.874199412	4.53451E-09
AP003555.2	1.872245731	0.000361982
AC243571.2	1.871163476	0.003484615
TNIP1	1.86945669	7.99814E-15
XLOC_013952	1.866841842	4.22466E-06
AC083949.1	1.860475204	0.022575665
CLEC12A-AS1	1.860193252	6.68471E-08
CAMK2A	1.859688267	1.92833E-05
ADAMTS4	1.856134174	3.73439E-31
SERPINA1	1.851090363	0.000260481
RNU6-60P	1.849171304	6.76981E-05
AL021408.2	1.843823182	0.000169667
AC034223.1	1.836027425	0.004329066
ANGPTL7	1.833357424	1.04144E-07
AL139807.1	1.83089513	0.004657015
ITGA2	1.826442103	3.11625E-24
AC013451.2	1.821820624	0.000244395
MMP8	1.821060337	0.003893202
SAMD9	1.820814124	9.32558E-13
PDGFB	1.814703433	0.012479274
HMOX1	1.812769583	9.28994E-35
MTSS1	1.805513279	5.66739E-08
AL355102.4	1.803808484	0.005188722
AL355102.1	1.802626004	0.014138228

ZNF259P1	1.799985722	1.83506E-23
DDX60L	1.798012251	5.58135E-13
ARHGEF16	1.796617887	0.004655234
CEACAM1	1.795524913	0.020632062
NFKBIA	1.791899538	2.11049E-16
IL1A	1.790819655	0.013807572
G41319	1.789753114	1.76321E-06
DGKI	1.789072102	4.31513E-26
AL139405.1	1.788599937	0.000546989
LINC01140	1.786293525	9.38324E-07
EDN1	1.784728269	2.32431E-24
HMGN2P46	1.783696304	6.19568E-05
SLC16A6	1.780446318	0.01465372
GBP2	1.779669249	5.80421E-09
RBM47	1.769185976	5.42322E-07
NCOA7	1.767846969	2.1083E-09
AL357992.1	1.767384765	4.1101E-08
SLC16A9	1.766869625	0.001830939
SRSF12	1.765143946	7.32974E-10
NFKB1	1.757661604	3.84587E-23
NCF2	1.753271527	5.87467E-11
XLOC_007190	1.748734499	0.003016546
SMOX	1.747426625	1.06711E-20
APOL1	1.746593323	4.09635E-09
PIK3CD	1.746567431	3.44663E-24
RN7SL683P	1.741483228	0.002018757
XLOC_009415	1.739102002	0.015068922
AC003092.1	1.736304487	4.21321E-07
GPR183	1.73420417	1.66022E-07
RIPK2	1.732121585	5.4202E-23
CCDC184	1.730205801	1.80207E-11
AL157937.1	1.728166079	0.001838434
LINC00222	1.725963825	0.010867031
AC004974.1	1.725768488	0.027015828
AC079174.1	1.723321246	0.003612929
AC004832.5	1.714071261	1.02346E-05
LINC00515	1.712697847	0.000155968
ITPRIP	1.711002552	2.53492E-26
MYO10	1.709813241	1.29139E-38
C5orf56	1.707374554	1.16229E-07
AC097634.1	1.706902971	0.000287647
BATF3	1.705930146	0.005436214
P3H2	1.701054434	2.22742E-14

G14029	1.700493817	0.001582668
L1TD1	1.698209337	2.24088E-12
MCC	1.694516672	1.47358E-22
SNORD39	1.69420235	2.60677E-05
SUSD6	1.693534805	3.89393E-34
LAMC2	1.69347441	3.66151E-14
AC109347.2	1.692525117	0.002201757
LINC02085	1.692229206	0.01161262
PLSCR1	1.688511745	2.1819E-09
RRAD	1.679101925	5.75518E-13
PFKFB3	1.676070511	4.83639E-37
RPL35AP32	1.674124269	0.041482577
NR4A1	1.673995624	2.16289E-11
HOXC13-AS	1.66393762	9.95252E-06
EFR3B	1.662094385	5.6851E-13
DHRS2	1.661722051	8.94979E-07
ISG15	1.661539474	3.98715E-08
LINC01711	1.661144323	0.002371503
HIVEP3	1.659408863	8.20287E-19
C3orf52	1.656741883	1.3182E-28
AP000695.1	1.655373463	3.40355E-06
AP004609.1	1.653344269	0.02139178
ADGRG1	1.652944114	7.20902E-07
XLOC_001195	1.648933607	0.002361967
ACKR3	1.648278413	4.56434E-10
WTAPP1	1.648064369	1.31687E-06
NPAS2	1.646981166	3.09642E-14
AP001970.1	1.646030016	0.012704651
CALHM6	1.64361074	4.66589E-06
APOL3	1.64180581	2.24016E-05
CSF1R	1.636772681	1.1695E-09
C3	1.636053111	1.22944E-07
TAP1	1.635269515	2.51368E-10
TNFSF15	1.624246023	0.004523095
LGALS9	1.619260088	1.20513E-07
WNT10B	1.614500467	0.000795108
AC116366.1	1.610869454	0.002647358
MFSD4A	1.607927022	0.034986649
LINC01359	1.605631146	0.039084399
AC025186.1	1.603891235	0.036420907
XYLT1	1.601953702	3.38289E-33
AC107021.2	1.597744093	2.93178E-15
POU3F2	1.59477258	0.014298248

GBP3	1.59131831	5.37602E-09
CETP	1.590956293	0.019196943
PDPN	1.588381617	5.36639E-17
AC007728.2	1.58835272	0.001311952
APOL2	1.586949705	2.35585E-08
AC093567.1	1.581639891	0.000385273
NECTIN4	1.580269662	0.000563281
AL691420.1	1.579310859	3.23218E-06
PTPRE	1.576167442	1.36475E-13
STX11	1.575208169	4.46019E-05
HS3ST3B1	1.572921649	2.82218E-31
AQP9	1.572319627	0.000108767
IL23A	1.567984911	1.80021E-05
GCSAM	1.566863452	0.03604777
LRRTM2	1.565712193	3.28652E-06
RAP1GAP2	1.565260853	3.02117E-06
MMP10	1.565190664	0.000793282
KIAA1755	1.564044951	1.61955E-07
SSBP3-AS1	1.562901004	0.006588639
LAMB3	1.560157217	9.53886E-09
TRIM25	1.555453883	1.81625E-10
ZNFX1	1.547428516	1.6479E-12
LINC00525	1.546899267	0.010828602
TNFRSF18	1.545256542	0.000689408
DLX2	1.544684222	1.33734E-07
COL27A1	1.544615743	7.47498E-38
XLOC_014038	1.54340843	0.000429764
LINC01686	1.543399853	0.00090075
ANO1	1.540051094	0.001260862
LRRN3	1.539747739	9.50103E-36
IBSP	1.538570119	0.00151914
IFI6	1.538543088	2.27836E-07
RPA4	1.536104005	0.022784124
ANGPTL1	1.533620459	1.14992E-05
AC087164.1	1.532618881	0.007737571
ARL9	1.531662668	8.98043E-05
CHST15	1.527470555	2.38914E-17
PTGES	1.527366897	9.08179E-10
STAT5A	1.526008289	6.79634E-11
AC090617.2	1.522833924	0.020713549
AL357060.1	1.515944727	5.58917E-05
ACHE	1.510440761	0.011073096
AC124319.4	1.507401463	0.0016223

FMNL3	1.504862661	3.08466E-28
SECTM1	1.504220516	0.000193021
TRAF3	1.504039041	1.36212E-29
AL021707.6	1.502316366	7.54931E-06
TRIM21	1.500501904	4.89707E-10
KDM6B	1.491692804	1.05723E-15
NUAK2	1.491415833	1.44847E-10
NGF	1.491279603	9.80927E-28
AC068860.1	1.489995739	0.002969704
NOCT	1.488492242	3.44829E-30
LEF1	1.483087833	3.70586E-10
CLDN4	1.480292381	2.71673E-06
GPR146	1.478779067	5.93353E-06
LHX8	1.478297181	0.002860033
NAMPTP1	1.47571304	1.48622E-10
AL078590.2	1.470570052	0.016859308
KIF5C	1.467824425	0.000323717
ZC3HAV1	1.467092725	5.80834E-10
TNFSF18	1.466422722	6.99712E-07
AL136295.2	1.466128548	0.000230399
AC022079.1	1.465867582	0.023855137
AL359752.1	1.464945355	3.85589E-08
ARHGAP27	1.460008162	0.000438581
GIMAP2	1.459163976	0.013974009
AC067751.1	1.455136547	5.2087E-05
AC099506.1	1.455008671	0.009382298
FZD8	1.454526581	1.11808E-09
L3MBTL4	1.452360043	0.036421887
CNTN1	1.451953712	5.98246E-05
NTN1	1.448226991	0.000198199
POU2F2	1.446786172	1.28229E-10
MAFF	1.445266479	2.32314E-15
MIR155HG	1.444595849	1.32442E-06
AC109588.1	1.441291375	0.004877989
MT2A	1.439144719	3.44831E-15
ZSWIM4	1.438323847	1.58629E-07
G38640	1.438025571	4.55158E-06
MYOCD	1.437067689	3.1073E-08
RARRES3	1.436047385	6.25264E-07
AL157702.2	1.435904215	0.002223149
PMAIP1	1.435822318	1.05826E-08
RNA5SP385	1.433748812	0.000439308
SAT1	1.431723503	1.49663E-14

TDRD7	1.431328936	4.7487E-05
AL356273.3	1.431218415	0.000313209
SLFNL1	1.430494598	0.024552892
GZMK	1.426962117	0.000229636
NGF-AS1	1.426642208	0.018487185
CSF1	1.425888714	7.9446E-07
SAMHD1	1.424691454	1.57646E-08
REL	1.422279118	2.42942E-17
CHST11	1.412176573	7.09411E-31
MOB3B	1.41176882	0.001492996
SNRPGP5	1.40655628	0.032915653
PCDH19	1.404485246	3.69457E-06
SLC41A2	1.403739823	3.48197E-24
FGFR3	1.403197251	0.022470964
SLC25A28	1.402189534	1.05349E-08
CACHD1	1.399359165	5.06536E-16
TIFA	1.397108811	1.91189E-12
YEATS2-AS1	1.397105028	0.042628258
PRG4	1.394552881	0.001375144
RIPOR3	1.392998865	1.00844E-07
GLDN	1.392678808	0.00010876
TMOD1	1.391577492	0.032278221
RFLNA	1.391186262	1.64909E-12
PPP1R14C	1.390789286	0.001371817
FGF18	1.390072945	0.007372273
HCG20	1.388683125	0.027936536
THBS4	1.38269311	5.0516E-05
PID1	1.377627077	4.91161E-13
AC022509.3	1.373961861	0.00773412
PAK3	1.367000101	0.015415399
AL603832.1	1.366452615	0.007040188
GPRC5B	1.365197239	5.45691E-06
PLEK2	1.362215506	9.63145E-21
EDARADD	1.360995913	2.03932E-06
SLC7A8	1.360405862	3.99521E-17
CIITA	1.35856302	0.00362064
CD82	1.356030038	2.34562E-07
SUSD4	1.353867951	0.001182927
ANXA13	1.349585167	0.025189318
INHBA	1.342967979	2.75258E-17
HIVEP2	1.341547352	1.97171E-28
AGAP9	1.335702958	0.022066953
SEC14L2	1.333190525	7.24726E-25

AC124276.2	1.331332939	8.43699E-17
SP110	1.329401035	4.48642E-07
AFF2	1.328467873	3.98259E-05
IL24	1.327350488	0.005917797
WWC2-AS2	1.325876567	0.000458748
RSPO3	1.325156407	1.69012E-05
MIR503	1.3237037	1.0393E-06
RNU6-1208P	1.323386205	0.039374511
BDKRB2	1.322538075	3.90431E-08
C5AR2	1.321385738	5.72084E-05
XLOC_008812	1.32044775	0.037098282
SLC22A3	1.316751967	0.002249188
CTGF	1.316431312	1.15415E-05
TSKU	1.313801811	1.99658E-21
KIAA1024	1.31265074	4.34843E-11
HDX	1.309678059	2.85403E-12
NIPAL1	1.309118335	0.0043344
AC012462.1	1.307258534	9.837E-05
SNCAIP	1.306330425	8.77949E-08
PLK3	1.305572787	3.38992E-20
MGAM	1.305570477	0.001611294
PTX3	1.298036958	1.80747E-05
NAMPT	1.297364869	8.27141E-14
CHRNA1	1.294513871	0.018718606
RNU6-301P	1.289738949	0.012725256
AF117829.1	1.288334943	2.54337E-08
SPHK1	1.288136725	6.15129E-19
BST2	1.287428224	0.000328366
AC073410.1	1.287312363	0.002213805
KDM7A	1.287138105	3.73024E-13
ART3	1.287082664	0.049987404
GLIS3	1.284060734	1.72315E-20
PNPT1	1.283349567	1.29266E-12
ITGB3	1.280800072	5.46082E-09
EDNRB	1.279819931	0.000325375
KBTBD8	1.279624524	0.000633526
ABTB2	1.277481087	3.28472E-09
SLFN5	1.276796166	1.22963E-09
TAF4B	1.275129852	2.88871E-06
SLCO3A1	1.274891588	1.02291E-13
DGKG	1.274698725	0.014208388
AC092800.1	1.270967886	0.030248509
AC104389.4	1.269757902	0.038999883

XLOC_011309	1.268128538	0.006978202
BHLHE40-AS1	1.2671572	0.000218445
MIR503HG	1.266854764	5.67001E-07
PARP10	1.266177993	1.91692E-05
LINC01411	1.265744846	0.014643438
MYOM3	1.263663127	0.002581167
AC097059.2	1.261474324	0.005141691
AP000442.2	1.260513328	0.029733421
AC129507.1	1.258604183	0.009036948
RHBDF2	1.258583301	1.15414E-09
PRDM16	1.258081347	0.007098564
EPHB2	1.257975398	1.4795E-09
CBFB	1.255591177	1.64791E-16
PARP15	1.255078003	0.005766412
DTX3L	1.254882669	2.02747E-06
LNCSRLR	1.25322702	4.16673E-05
SLC43A2	1.251977024	5.17751E-08
ERVMER34-1	1.250843542	0.013089362
MSX2	1.248864343	0.040702769
HLA-F	1.248543209	2.77741E-07
SLC1A3	1.247558611	9.91733E-11
NEDD9	1.247063042	1.85905E-40
FGF2	1.246492579	3.77116E-25
BEAN1	1.244864871	0.011110195
NINJ1	1.244169172	1.33737E-08
CAPN3	1.240779401	0.038368369
SLC2A6	1.237799475	6.39001E-14
AC009404.1	1.23640763	1.54634E-15
AC092718.3	1.235635441	0.000772456
LINC01943	1.234633846	7.19612E-16
ZNF267	1.234633674	9.52364E-23
RPL7L1P9	1.233921369	0.003751053
PANX1	1.233151643	5.55774E-12
IL15	1.233148105	4.92231E-09
RASD2	1.230259954	9.61456E-05
AC105105.3	1.229350628	0.027112583
AL158050.1	1.228860529	0.042871197
NKILA	1.223343148	1.2658E-06
IFNAR2	1.221016151	3.41337E-11
PRICKLE2-AS3	1.217772494	0.004985575
SLC39A8	1.217445179	1.69839E-06
LAP3	1.216794077	8.72794E-09
ESR1	1.214954432	0.017014999

AC087623.1	1.213742701	0.04645006
RNF122	1.213537399	2.00882E-11
PRDM1	1.21225153	2.21929E-34
FOXO3	1.210871757	2.00055E-32
AC037459.2	1.209406106	0.0032037
GPR75	1.209355173	0.015931804
AC017002.1	1.207671093	1.59461E-10
TLR1	1.206603173	0.00016837
SYTL3	1.206285304	1.752E-08
SLC28A3	1.201442832	0.019433212
TMEM217	1.199177888	7.63654E-07
G12163	1.198234718	0.007737571
AL355355.1	1.19767066	0.017976091
ARRDC2	1.197153321	5.04115E-10
SHISAL1	1.196736209	2.42161E-18
NAV2	1.19624729	3.97902E-35
AL078644.1	1.195748082	0.015594967
SQOR	1.194682022	1.05261E-11
RASD1	1.194629014	1.58175E-06
CNGA3	1.194484121	0.014479283
LAMA3	1.191959113	9.63231E-07
AL596325.2	1.191928336	4.90341E-05
LINC01239	1.190255658	0.01508876
AC018628.1	1.187214415	2.94801E-05
LYPD3	1.187146214	0.00087767
POLH-AS1	1.186980037	0.030914942
AC017002.3	1.185933481	3.10312E-07
PML	1.185599695	3.684E-09
CGAS	1.185184047	3.76452E-11
AC107021.1	1.185119532	0.001420226
TJP2	1.184239331	4.70581E-05
XLOC_008957	1.181967683	0.017362169
NOX4	1.178548062	1.81141E-15
MYO7B	1.175288918	3.60401E-05
COL22A1	1.175040918	0.029165999
FAM49A	1.174352462	0.003112239
FRMD4A	1.173902321	6.10047E-11
PPARD	1.171364159	2.74619E-14
ERN1	1.170783225	3.03424E-16
PLXNA2	1.170590033	7.86392E-09
PRICKLE2	1.1704811	1.85795E-20
RHEBL1	1.168957871	3.02453E-05
ARNTL2	1.166702282	3.06851E-08

AL139022.1	1.165373623	0.020474906
ZCCHC2	1.164896694	4.7654E-09
SPSB1	1.164749962	1.95638E-10
JAK2	1.164478246	5.55729E-06
PSMB9	1.162509549	1.03075E-06
AL672207.1	1.161687353	0.049662319
DRP2	1.161503786	0.000742296
AC093908.1	1.16022588	0.003528975
AC024909.2	1.159783565	0.048387635
CIART	1.15624655	7.31017E-15
AL132656.4	1.15458836	2.68997E-05
CFLAR-AS1	1.154420216	0.003154203
APOL6	1.153488462	2.00311E-05
APOBEC3G	1.15182425	1.32297E-05
G36605	1.15081278	0.00060876
OGFR	1.149014962	3.77848E-06
AC245100.8	1.145901062	4.31334E-08
PSTPIP2	1.144474594	7.4794E-16
ANGPT4	1.144137602	0.011230153
CDKN2B	1.143295829	1.56272E-15
PCDH9	1.140021083	3.26068E-15
S1PR3	1.138923127	4.03713E-15
AL354732.1	1.135974416	0.000989137
CCDC88C	1.134267912	0.002965571
AL359711.2	1.133272178	0.038963263
AC078785.1	1.132745083	0.010961066
TAP2	1.131580508	1.94428E-09
TBXAS1	1.131408228	1.85581E-05
RELN	1.131177939	0.045026579
AC005224.3	1.131086575	0.000501006
CD7	1.129776988	0.026168021
STARD4	1.129144716	6.08537E-23
BAK1P1	1.128132503	0.032680002
AP005136.2	1.126177439	0.032612245
WNK4	1.124922122	1.74112E-09
KY	1.123773254	0.020672165
TRIM14	1.122516297	8.58643E-07
KDM7A-DT	1.122238771	9.30897E-08
SNAI1	1.117272659	1.2928E-08
ST3GAL1	1.113418533	3.957E-20
IL18	1.113184796	0.001360705
FAM71F1	1.112165083	0.023940535
IRF2	1.111765306	1.1323E-05

SHANK3	1.111391468	0.020587698
ZNF295-AS1	1.111200375	0.001674513
AL357054.4	1.108801718	0.030273334
CFLAR	1.108386033	1.36475E-13
SLC2A1	1.105832934	2.59402E-13
ACOX1	1.105650398	1.09878E-22
FOXP1	1.104516574	6.50281E-26
DIRAS3	1.103689341	0.000111331
IFI35	1.100247529	4.39573E-06
NMI	1.094645179	1.94361E-06
CCIN	1.09355351	1.82613E-12
PPM1K	1.092885552	1.77007E-05
RNF152	1.09202367	1.31421E-07
CD3EAP	1.091181307	1.96149E-11
AC010343.3	1.091073604	0.000126257
RIMS2	1.090850771	0.023866814
AC103702.2	1.090133106	0.002305293
APCDD1L-DT	1.089502949	1.45271E-13
AL162727.3	1.088770246	0.047177493
CPN2	1.088596694	0.044235804
ZFPM2	1.088100453	0.000260481
NET1	1.08776958	9.36019E-14
GMPR	1.087219531	0.002421226
MT2P1	1.085252287	5.03433E-05
MOB3C	1.083804196	9.94683E-07
CPNE5	1.08312706	0.01089305
BMPR1B	1.080709512	1.06514E-08
HIVEP1	1.079141029	1.62121E-19
SYNDIG1	1.079069968	3.90431E-08
AC021205.1	1.076117782	7.23277E-05
TMEM92	1.074820294	0.000637418
MYD88	1.073645229	6.09255E-06
B4GALT1	1.070382853	7.97615E-54
MT1X	1.069982389	6.62139E-11
LRRC4	1.069395496	0.030751704
LINC00908	1.067250989	0.041216836
CLEC2A	1.066093624	3.62716E-10
CLEC2B	1.064731311	6.88901E-11
XLOC_003077	1.06374649	0.023142538
PARP9	1.06234772	0.000110619
AC079298.3	1.061057671	2.25079E-06
AC009549.1	1.060988979	0.016668123
ZNF469	1.06061522	1.12508E-10

ZC3H12C	1.059845876	5.94199E-07
TGM2	1.059281585	1.21497E-15
CASP1	1.058715897	9.91451E-06
SHB	1.056501231	4.45107E-08
PLAT	1.056172302	3.38914E-10
IRAK3	1.056005672	1.29212E-05
RHBDL2	1.055210716	9.32199E-14
AL031595.3	1.053669442	0.014572899
AL390719.1	1.053221201	0.038874395
BTN3A3	1.052402958	2.07193E-09
LCP1	1.052390286	0.008695822
DIO3OS	1.051968053	0.025798206
ELOVL3	1.05165594	1.04514E-05
FLT1	1.049000615	1.26763E-07
SIRPAP1	1.048414355	0.02035033
AL136295.5	1.047879258	0.042318966
CCNJ	1.047081292	1.63227E-14
HLA-J	1.047005472	0.041482577
MARCKSL1	1.046306704	9.19226E-06
SLC25A37	1.046140529	1.68546E-11
A4GALT	1.044076437	1.82909E-07
CDYL2	1.043562784	2.58474E-11
ASPHD2	1.043082811	0.000242925
NKX2-6	1.042641773	0.014087552
DUSP5	1.042559519	5.70276E-07
IFNGR2	1.042020397	9.90421E-11
LINC01060	1.041892189	3.97444E-05
HSPD1P4	1.041337929	0.010512708
RUNX3	1.040815495	5.62611E-11
AC034223.2	1.040795684	0.011179324
PDZD2	1.040622037	0.001430301
CGNL1	1.03886367	1.71952E-05
PGM2L1	1.038023096	5.56272E-27
AC093724.1	1.037856614	0.009720977
SLFNL1-AS1	1.035830709	0.008692635
FAM168A	1.034597869	3.56331E-21
FRMD6	1.033901323	1.53175E-23
ADAMTS9	1.032833269	0.001602297
AL138724.1	1.032183147	0.000198132
GEM	1.031662079	3.2548E-17
HK2	1.031611616	4.07703E-17
TET3	1.031395406	1.41582E-05
C19orf66	1.030390061	1.94828E-05

KLF10	1.029971692	3.39982E-18
BCOR	1.029322567	4.68838E-13
BEND5	1.028993354	0.003377912
SERPINE1	1.028911325	3.86282E-14
RAB20	1.028504432	0.00477087
MSANTD3	1.027717101	7.32872E-19
EDNRA	1.026521724	1.42679E-07
TMEM140	1.024614511	0.001453377
HOMER1	1.021292943	8.94545E-12
LINC01561	1.021198043	0.039084399
XLOC_004803	1.018007225	1.2055E-06
SKIL	1.012321281	3.3361E-15
IPO7P2	1.010702637	0.009473173
PKNOX2	1.010584235	0.012571928
SLC7A5	1.009861707	3.08546E-13
C21orf91	1.003408207	2.40184E-09
PHACTR4	1.003277174	1.16139E-08
APOBEC3D	1.003272992	0.014489129
LRRC49	1.00310989	1.80703E-12
AC090197.1	1.000053348	2.88601E-05
EVA1A	0.999045463	1.2802E-19
AK3P3	0.998278149	0.033649704
KCNN4	0.998021319	0.000105296
CCDC144CP	0.997748285	0.017811721
SPRY4	0.997740626	1.82745E-14
GRB14	0.994569154	0.047032398
RAI14	0.993591884	7.71818E-27
TRAFD1	0.993063367	1.72538E-05
DUSP3	0.989864735	2.4769E-25
DCP1A	0.989280531	1.59112E-12
CLEC2D	0.98860142	0.000140515
FSTL3	0.986979818	3.18492E-06
RTTN	0.986442411	2.23966E-19
LINC01013	0.986162698	2.96081E-09
ADAM19	0.986136534	3.32195E-24
MSX1	0.984715946	4.72007E-08
CDC42SE1	0.980898785	9.74777E-20
KCNG1	0.980303631	3.38353E-07
IL18R1	0.979797709	0.044814528
PDCD1LG2	0.979612457	1.24413E-21
LINC00519	0.979611695	0.000100892
KLF9	0.978171842	8.14238E-10
SLC11A2	0.976449559	3.24362E-15

ABLIM3	0.975055154	5.08679E-18
NOTCH2	0.974729442	4.83639E-37
MIRLET7A2	0.974462176	0.002045607
AL358115.1	0.973543357	0.014713775
CASP7	0.971160185	3.07573E-07
HIPK2	0.968488048	2.10818E-23
MLKL	0.967712522	1.53995E-05
AUTS2	0.965441645	3.95999E-13
RNF213	0.964762421	4.36215E-06
AC112721.2	0.962647804	0.014694392
AMZ1	0.961312248	9.00383E-14
TPM1-AS	0.960509078	0.014087552
AL355355.2	0.957865188	0.006501506
PFKFB4	0.956186367	5.05941E-11
MMP25-AS1	0.95410835	4.005E-06
STK38L	0.954000382	1.45952E-11
MTFP1	0.953505102	8.36708E-09
ATP2B1	0.953338364	4.76163E-11
ARID5A	0.952935775	1.97916E-08
STAT4	0.952800736	3.40478E-05
CYLD	0.951933609	2.4941E-13
TICAM1	0.951230885	7.95228E-11
CREB3L2	0.949315992	9.39224E-33
DRAM1	0.947365759	2.27557E-09
TMEM63B	0.946118861	3.45211E-09
G17127	0.944458494	0.008505828
MAFB	0.944446738	0.000485058
AC060766.4	0.943223246	0.002883105
NGFR	0.942262924	0.031635005
CD40	0.941750896	8.32698E-08
MORC3	0.940991635	1.04361E-25
ZNF710	0.9409768	8.78066E-05
C15orf39	0.940373817	2.46909E-08
AC040162.1	0.937762055	0.012009722
AL157394.1	0.937208778	0.00052635
HLX	0.936847823	2.91372E-09
RNF114	0.93566526	9.09558E-11
NAB1	0.935545519	2.87744E-13
HIST2H2BC	0.93553211	0.001838851
ITPR3	0.932940865	7.08778E-10
ARHGAP31	0.932687712	1.00654E-22
IL1RL1	0.932263393	0.000380509
C17orf49	0.931823208	0.017215831

DENND5A	0.931283226	1.02665E-20
TMEM41B	0.930314438	1.53346E-17
IFIT5	0.928916542	3.88758E-07
ZNF107	0.928317449	3.40676E-07
BIRC2	0.926741028	8.33193E-19
XLOC_006335	0.92597185	1.99275E-06
XLOC_007116	0.925595735	4.89742E-05
FOXP4	0.925002478	1.4163E-08
AC105105.1	0.924733065	0.04645006
PRSS35	0.923235284	0.031095647
ENTPD7	0.920816921	4.60134E-16
ETS1	0.920740054	7.62677E-13
BMT2	0.920582603	5.64571E-16
AC124798.1	0.919783521	0.002526697
LINC00842	0.919387097	0.0019605
FUT4	0.919032651	0.000107214
SMURF1	0.918630073	1.13452E-20
SPRY2	0.918463152	5.23894E-12
C2orf27A	0.916315699	2.84131E-13
XLOC_006303	0.915799908	3.14292E-06
IL4R	0.915386115	2.61318E-17
CTPS1	0.914511923	4.03437E-21
PLAUR	0.913456885	2.89468E-24
DNAJB5	0.912588267	5.78163E-14
AC091806.1	0.911307477	0.006139756
AC004816.2	0.909916031	0.000110741
NRIP2	0.909660657	0.008282821
B3GNT2	0.909457057	1.4806E-07
TTC39B	0.907551068	8.98142E-09
N4BP1	0.907405038	2.82739E-10
AMIGO2	0.906592951	5.33838E-21
AL645929.1	0.906253887	0.033811242
TMEM132A	0.904988533	1.8835E-05
XLOC_000523	0.90492542	0.013329223
NFATC1	0.904543209	6.05565E-10
AP000866.6	0.901851984	0.045989516
HS3ST3A1	0.901623963	1.7446E-10
AL139393.2	0.90150755	0.000259315
TLNRD1	0.900651725	4.41172E-08
RAPGEF2	0.900242168	6.97654E-12
TRAF2	0.899919478	2.25811E-08
ARHGEF40	0.898924345	3.42633E-15
MIR1245A	0.897614587	4.57397E-05

AC090607.2	0.895455649	0.02921111
CALB2	0.895223362	0.000326945
GASAL1	0.895105258	2.30972E-06
GPR137C	0.895073902	0.035444045
AC004832.4	0.89397091	0.036148472
TRIM38	0.893733934	8.01555E-07
NLRC5	0.892725824	1.86279E-05
EGFR	0.892493531	1.14362E-15
IKBKE	0.891286233	8.2443E-07
PPP1R3B	0.890157329	2.71584E-20
USP42	0.886259194	1.83165E-10
WDFY2	0.885069203	2.63485E-16
KCNT2	0.884891375	0.00773604
FERMT1	0.884098706	0.004172749
PELI1	0.883471355	6.91971E-09
UAP1	0.882999995	1.41414E-21
PNP	0.882863977	6.04895E-14
G7242	0.881991565	0.008470709
SH3PXD2A	0.881733043	2.04828E-28
CRISPLD2	0.881543819	2.39438E-20
BAZ1A	0.880495071	1.19299E-10
ACSL5	0.879397792	0.001033395
BHLHE41	0.879207407	4.07432E-07
APBB2	0.879114238	8.85584E-22
AC060766.5	0.878118623	0.044990507
FRMD3	0.877805251	0.035405544
MIR4482-1	0.875308191	0.018802766
NPTX2	0.875266977	0.017010445
ELK3	0.873792051	8.72637E-34
AC093801.1	0.873488437	1.74788E-06
BTN2A2	0.872946593	4.40866E-10
GFRA2	0.872363306	0.019160835
GPR3	0.872303384	0.016361088
MAML2	0.869796626	4.40866E-10
MOV10L1	0.868250914	0.012812545
MAP3K5	0.868142793	0.000144062
PTPRJ	0.867983466	2.53019E-05
VEGFC	0.867127136	5.11607E-13
TPST1	0.866341153	4.34845E-17
TNS1	0.865523729	2.63734E-12
PREX1	0.864625372	0.000633526
ADAMTSL4-AS1	0.864512437	0.036198033
PARP8	0.863736469	1.62838E-11

LHFPL2	0.863328739	2.52085E-13
TSHZ3	0.862206773	8.51086E-13
ADTRP	0.861873181	5.89243E-07
SYT15	0.860518266	0.000442569
LRCH1	0.860274399	3.48523E-11
UBASH3B	0.860064857	1.64557E-14
ACSL4	0.860012423	3.85795E-21
SIRPA	0.859923118	1.51609E-25
BNC1	0.859259838	0.009812504
MICB	0.858418799	1.21111E-07
SLFN13	0.857571404	0.000355929
NCR3LG1	0.857443719	0.004675098
G14979	0.857273017	0.022453362
CDK6	0.857139843	9.13856E-13
SAMD4A	0.857082351	2.38895E-15
GTPBP1	0.857028078	1.55702E-06
CXCL16	0.856393679	6.58383E-05
SPATA13	0.854020952	9.32597E-05
PPP3CC	0.85331742	1.98132E-06
AL157871.3	0.852415501	0.000565522
ANKRD44	0.852123881	5.26829E-12
RUNX1	0.851369244	1.79955E-14
E2F7	0.850737136	8.76244E-09
CDKL5	0.849226344	0.000495859
PODXL	0.848481296	4.38508E-07
TCAF2	0.847768048	2.71053E-05
SALL1	0.847464291	0.00021465
KRT16	0.84702008	0.017765807
AC108010.1	0.846599891	0.009209543
RPSAP52	0.846396907	6.46431E-11
HECW2	0.846233081	2.27034E-05
PALM2	0.845163262	0.000224486
PCNX1	0.845120564	1.39967E-21
BMPR2	0.843900633	1.04031E-18
PLEKHA4	0.842002069	0.000154491
TBC1D1	0.840759246	6.8044E-11
ARNTL	0.837898506	3.4459E-23
LINC01592	0.837840304	2.24271E-05
PRKCZ	0.837790401	0.000881683
MSC-AS1	0.83771117	2.93801E-23
DLX5	0.837705346	0.029156563
EIF2AK2	0.837608583	8.86477E-08
RP11-640M9.1	0.837286139	0.000198132

TRIM26	0.835914038	4.59892E-06
TMEFF1	0.83546501	0.041196031
POPDC2	0.834828367	0.000615759
WTAP	0.834686901	1.77528E-11
WARS	0.834340538	1.28339E-06
ITPRIPL2	0.832785438	2.57554E-10
GSAP	0.831185358	0.002526683
SH2B3	0.830939194	3.43049E-13
IRS2	0.830673089	1.27497E-09
BTN3A1	0.830344647	8.23421E-05
ADCY4	0.829579836	6.58383E-05
COL24A1	0.829345195	3.22614E-06
RORA	0.829285047	5.82116E-12
AL121603.2	0.829285012	0.028491043
LINC01137	0.828514555	4.30275E-10
CBR3	0.827281352	2.86085E-07
SMURF2	0.826898287	2.79245E-31
RNF31	0.82674881	9.54718E-05
B4GALT5	0.825872424	1.6479E-12
XLOC_013420	0.825257002	0.010709564
SSBP3	0.82462253	1.2397E-16
TNFAIP8L3	0.823916889	0.014754481
SOX11	0.82238261	0.000149162
LINC01426	0.822371653	2.05598E-05
FAS	0.821783471	5.58135E-13
GAS7	0.820594306	7.67698E-09
ZBED6	0.820115639	0.024395647
ABL1	0.818884674	4.01877E-10
AC245297.2	0.81834337	0.011958909
SHROOM4	0.817657799	0.000184312
AC004987.2	0.816671883	0.007517006
TMEM106A	0.816354705	1.50985E-06
IL21R	0.81495282	0.037150537
TSPAN13	0.814673072	6.52995E-10
SRRM3	0.813809125	0.000154796
ARL5B	0.813714337	3.41828E-07
ROS1	0.813160218	0.015188123
ADAMTS6	0.812596427	7.17372E-15
EHD1	0.812297217	1.00744E-06
RNF126	0.811982139	2.78734E-09
VEGFA	0.811863857	2.74136E-09
ANKLE2	0.811359174	3.15373E-19
TBC1D10A	0.810564562	2.85259E-09

DNAJA1	0.810451443	8.35062E-12
MASTL	0.810325192	3.54354E-05
SLC22A23	0.808320046	3.58365E-08
CYP51A1P2	0.806832219	0.049462435
TBC1D2B	0.805623303	3.70452E-11
AC108463.3	0.805454955	0.039472924
SLC35G1	0.804887171	3.79119E-10
PLAU	0.802102784	7.12678E-05
MDGA1	0.801769309	0.000268795
RGS20	0.80137851	2.34613E-05
NUDCD1	0.799840839	2.30406E-12
OTUD4	0.798704277	3.17082E-09
APCDD1L	0.796594118	5.60865E-09
RARRES1	0.796333517	0.044784535
ZNF470	0.796035705	4.88468E-10
SERPINB8	0.795467338	1.16223E-23
ADPRHL2	0.793152815	3.73954E-10
UBE2L6	0.79242529	0.000192916
UCK2	0.792227089	3.71728E-19
TMEM171	0.791409721	0.020639194
LRP4	0.790775137	0.001339769
KSR1	0.788280201	6.88099E-06
PLXNA4	0.788230314	0.003141401
RASGRP1	0.788217941	0.005460082
GALNT16	0.787337323	0.002062104
CLMAT3	0.786284359	7.35174E-06
TP53BP2	0.785803992	6.90439E-10
ZNF670	0.785400853	5.4184E-07
SERTAD1	0.784338976	8.0406E-13
PVR	0.783686736	1.79251E-16
INSIG1	0.78332929	3.0746E-06
AC068491.4	0.783239331	0.000248312
TNFRSF10B	0.783136158	2.63764E-10
EHD4	0.782406086	1.37217E-06
S1PR2	0.78117158	1.78371E-09
AC060766.7	0.780332098	0.00188583
ETV6	0.778343482	7.3307E-27
RGPD5	0.778333015	0.001716529
LINC00624	0.775342417	0.004626942
PIK3R3	0.774139965	0.025991653
GPR180	0.773098988	3.54274E-11
APAF1	0.772127441	6.66611E-09
CEP350	0.771733082	1.16139E-08

CASP10	0.770574081	0.000664506
ANO7L1	0.770270609	0.002212371
NCK2	0.768652724	1.3849E-11
CARD16	0.768001197	0.000325375
GALNT3	0.765536837	0.00280799
EMP2	0.764399169	1.28151E-08
NRG1	0.763684388	3.67641E-20
AC002056.1	0.762113255	0.020827916
SH3PXD2B	0.76199619	2.17813E-11
CEP170P1	0.761953132	2.29082E-07
CYTH1	0.761890878	1.56743E-08
TIPARP	0.761528015	2.19264E-12
CEP170	0.761000928	1.42892E-23
CEMIP2	0.759997657	1.19641E-19
MAP2	0.756760052	0.000176697
KLF7	0.756645461	9.48441E-19
SP100	0.754087652	2.54926E-09
RBM24	0.753526706	1.03296E-05
RCAN1	0.752649179	1.16378E-05
SLFN11	0.752371476	4.90456E-09
NRP2	0.75067964	1.2988E-08
SPECC1	0.750587363	2.34586E-21
SDC1	0.750229211	3.75741E-09
CDH2	0.747994378	5.75248E-23
GSTO2	0.747037617	2.02057E-06
ZNF597	0.746614787	0.00019399
TRIM22	0.74633393	2.12951E-06
CYTOR	0.74473144	4.01449E-16
CXorf38	0.744707044	2.5398E-08
C17orf67	0.744320745	0.00330035
NUS1P1	0.744078147	0.012911656
BTG3	0.742530662	4.16191E-11
TRIM5	0.742448402	5.68768E-05
AC008147.2	0.742299891	0.045987156
RAP2C	0.742000341	1.17265E-09
XLOC_004362	0.73929487	0.001780732
SPEN	0.73861683	7.37485E-11
HHIP	0.738545988	0.000913568
CCDC93	0.738376784	4.00949E-26
RCL1	0.73816465	6.30519E-16
ELL	0.738092821	3.06477E-07
NAV3	0.737998008	4.0808E-18
PNRC2P1	0.737965027	0.038365928

SAMD11	0.737833898	0.004016247
XRN1	0.736924737	4.3651E-07
TLE3	0.735929079	1.20011E-07
STX1B	0.735767652	0.033253322
HIF1A	0.735657445	3.27168E-18
GABARAPL1	0.735609098	1.6431E-12
DGKH	0.735575215	5.10047E-07
TIAM2	0.734828246	1.32609E-11
AC021205.3	0.734461353	0.020683078
ADGRD1	0.73302062	6.53903E-07
BLZF1	0.732968979	1.48531E-09
HELB	0.732625759	0.002101641
MT1E	0.729067501	1.0444E-07
ABL2	0.728921274	4.36941E-18
PRKD2	0.727877692	0.000513017
AL356417.2	0.727818614	6.40817E-05
ZBTB21	0.727313053	1.61099E-14
TAPBP	0.727261757	1.09333E-06
NBN	0.727134292	2.83415E-11
SDC4	0.726768679	3.41828E-07
PTGIR	0.725431905	1.4237E-05
CACNA1A	0.725380081	0.002352183
LIMD2	0.723761146	2.96075E-05
G6708	0.723395523	4.78916E-05
ANKFY1	0.722057594	6.22461E-08
TMEM100	0.721076496	0.045955859
UNC5B	0.720714547	1.32911E-08
LACTB	0.720328201	3.83815E-11
PHLDB1	0.720276293	2.10608E-06
TRIP10	0.719498006	1.12537E-08
GPD2	0.719381394	2.1205E-07
PDLIM4	0.719303579	4.09009E-11
PAX8	0.717443897	0.000584844
AC243964.3	0.717065507	3.59327E-05
PIM3	0.71565267	0.000671172
TBC1D16	0.712505035	4.14274E-10
MMP3	0.710554246	1.21472E-06
AMOTL1	0.71043221	3.58782E-20
TLE4	0.709049013	2.87358E-10
RBMS1P1	0.708352128	0.011336586
MAP3K8	0.708213857	0.000398156
TENT4A	0.706122623	1.33281E-08
JMJD6	0.705949542	3.0244E-28

DTX4	0.705579441	0.002030486
UXS1	0.705161929	2.9734E-10
ARHGAP42	0.704649974	0.000408299
RNU2-59P	0.70418575	0.006981274
CHST2	0.703774222	0.004240005
XIAP	0.701687676	2.8163E-16
CD44	0.701078401	2.37591E-13
TRIM47	0.700307009	0.008641295
ETV3	0.698486597	8.44568E-07
PLA2G4A	0.697778179	0.000865768
AC005229.1	0.696045059	0.000104612
MCL1	0.695728861	6.31149E-11
ERRFI1	0.694102089	1.72538E-05
CSRNP2	0.693884167	3.62841E-08
HLA-L	0.693271098	2.91495E-06
FIBCD1	0.689302444	0.000208983
PIM1	0.688942709	0.000418263
CFH	0.688338397	9.20959E-05
LRRC15	0.686994678	2.9262E-07
GLIPR2	0.685936049	1.07637E-14
STIL	0.685877262	1.54783E-10
1-Dec	0.685517801	0.034830714
SYTL4	0.685340065	0.003948068
OSGIN2	0.684976951	2.48022E-06
MICAL3	0.684385836	2.66873E-05
GTF2B	0.684227817	3.36911E-08
ATP10A	0.683677954	5.77267E-15
ATP2B1-AS1	0.683434038	0.008492438
WWC3	0.682872603	9.70459E-15
FAM83G	0.682093486	0.000462123
SGK1	0.680917768	1.45848E-11
ZBTB11-AS1	0.68070167	0.002655419
SEMA7A	0.679324928	3.30303E-08
PSD4	0.679112135	0.005790968
PSMB10	0.679088514	4.4431E-05
CD58	0.67889121	6.53937E-12
TRANK1	0.676104606	0.002110212
PRKCD	0.676088728	2.15887E-07
MAP1S	0.676071995	0.000405958
MEF2A	0.675198045	8.14919E-20
FILIP1L	0.675022185	1.60274E-18
IFITM1	0.674371906	0.000275924
TRPM3	0.673480964	0.004646126

RELA	0.673178186	2.49461E-06
AC112220.2	0.673010748	1.54091E-07
AP000525.9	0.672692226	0.000164318
SLC7A7	0.672172231	9.97562E-05
MYOSLID	0.671979846	2.01075E-06
CCDC150	0.671399265	0.008354064
CHIC2	0.670866634	2.20895E-16
TENM4	0.669662537	1.10832E-06
IFNWP19	0.669399601	3.28985E-05
EPB41L4A	0.668046888	0.009999524
NUP58	0.667582214	5.53141E-19
HILPDA	0.667524844	5.83014E-13
NHS	0.666944836	0.004193735
NUP62	0.666518046	1.10854E-06
DOT1L	0.663568485	1.26109E-05
PCGF5	0.663541183	3.94863E-06
ANKRD1	0.663047805	0.001536647
CTDP1	0.662598828	0.00256266
BAHCC1	0.662438154	1.1961E-05
SPP1	0.662390516	3.90139E-05
ADAMTS14	0.661354731	0.033778872
BCL10	0.659416236	1.41414E-21
WSB1	0.657249384	1.78813E-19
MEX3B	0.656005275	0.000800786
SERTAD2	0.654313614	4.21069E-11
SP140L	0.653594652	7.98114E-10
DNAJB9	0.653172465	7.32872E-19
UHRF1BP1	0.651538376	0.000185944
BCL7A	0.651151856	2.18089E-05
BPGM	0.650980854	9.47569E-16
PSMB8	0.650430597	0.000156834
FAM110A	0.650414187	0.045632352
SLC35F2	0.650288715	6.02212E-09
SEC24A	0.650275864	1.30324E-10
FAM210A	0.649649295	9.69675E-11
KLHL21	0.649476365	3.19856E-08
PPP1R13L	0.648247967	9.26693E-07
OGFRL1	0.64802255	1.31846E-05
LDLRAD3	0.646931336	2.42953E-09
NUB1	0.645498165	5.51977E-07
FAM208B	0.645205528	2.08046E-16
LIMS1	0.644314121	6.66118E-21
FAM155A	0.644137263	0.012359759

BAK1	0.643243979	3.57655E-05
LUZP1	0.643186753	1.24631E-21
PLPP4	0.643161873	2.00025E-08
AC080013.4	0.643058088	0.033461519
HASPIN	0.642876542	0.004037559
PPAN	0.641058092	0.034140821
STBD1	0.639565011	4.78128E-08
PDE4D	0.63950758	0.005758342
ABCC6	0.638903827	0.007956343
SGPL1	0.63821017	2.77476E-16
TBX2	0.638035334	0.014432243
ZNF618	0.636353621	0.000484833
SLC30A7	0.636111454	3.74045E-11
ARSI	0.635915608	0.00095054
NNMT	0.635394525	9.28222E-11
FUT11	0.633917508	7.07304E-15
TBC1D9	0.633411993	4.78255E-07
GADD45B	0.632481953	8.91832E-09
MPP3	0.632104414	0.000634862
WDR66	0.631493192	0.001986539
SPTLC2	0.63143925	7.68887E-32
DUSP6	0.630898149	4.43348E-06
TNFAIP1	0.630604891	3.11213E-08
JAK3	0.629203344	0.000237776
MLXIP	0.628952482	7.4559E-11
ROBO1	0.62859838	6.48678E-10
ARHGAP22	0.628218917	3.64115E-07
USP31	0.628044763	5.30389E-06
RP6-206I17.1	0.627287434	1.39519E-05
PSMD6-AS2	0.626990194	0.029231487
ZNF697	0.626932297	0.00019236
SLFN12	0.626699368	3.41828E-07
HIST2H2AA3	0.626377057	0.021742204
PUS7	0.62634421	7.19867E-13
DENND2C	0.626271377	4.02061E-05
KDM6A	0.625788998	9.94683E-07
BICDL2	0.625262083	0.044385585
FMR1	0.624901428	1.07568E-06
SELENOI	0.624851893	6.47805E-09
DOCK9	0.624426699	8.44136E-06
PIGA	0.623638194	5.9381E-11
CNP	0.623564076	2.68997E-05
STARD13-AS	0.623432928	0.001453888

NOD1	0.622431764	0.004902418
MOV10	0.621651226	3.34308E-05
VHL	0.62079477	4.75618E-07
CLCF1	0.620659464	2.27821E-07
LINC01705	0.620503217	0.004337897
KBTBD2	0.620479145	8.32257E-18
JCAD	0.619416731	6.67101E-14
APLF	0.619136081	2.20842E-05
PABPC4L	0.618606814	0.011222348
SERPINB9	0.61858739	0.010651741
ODF2L	0.618270059	1.56983E-08
IRF9	0.617675425	0.014486126
G33445	0.615919792	0.001558171
MICAL2	0.615848396	1.53457E-13
MICALL2	0.614571491	0.000168762
NOTCH2NLA	0.614440187	8.27141E-14
ADAMTS9-AS2	0.613681722	0.001152329
TMEM189	0.613460391	3.57149E-09
EPHB1	0.613245474	0.008492288
SPAG1	0.612980884	0.019597545
JADE3	0.611505263	5.72544E-06
RNF149	0.610585066	2.1572E-05
APOBEC3F	0.610564314	0.002535223
ADO	0.609497634	9.56676E-10
PHF11	0.609380428	1.03122E-06
CPEB4	0.60906477	3.46284E-08
BTN2A1	0.60895264	6.15058E-11
WISP1	0.608695216	1.13644E-06
GNPNAT1	0.607779229	2.86018E-22
BAZ2A	0.607700512	2.31166E-06
SOX4	0.607464011	1.26572E-05
SMG6	0.606213714	2.00638E-07
BLOC1S3	0.605368305	5.96655E-11
STARD13	0.604732457	2.39694E-09
FGF5	0.604434837	1.90137E-08
P2RY11	0.603781467	0.039333034
CHST3	0.602801524	1.97351E-09
ULK1	0.602332177	2.32307E-05
MFHAS1	0.601670754	0.000121654
YWHAG	0.601168759	3.00203E-15
NANOS3	0.600986944	0.000461202
SDK2	0.600603112	1.99014E-05
BID	0.600256466	7.43653E-08

SENP8	-0.600063622	0.000148404
AC008946.1	-0.600417468	0.027551936
AL135925.1	-0.600515083	9.7793E-05
BLOC1S1-RDH5	-0.60065305	0.017701818
SHOX2	-0.600793617	5.32186E-06
ZNF503	-0.600841183	9.88132E-05
AC005736.1	-0.601986887	7.74073E-06
NHLRC1	-0.602037541	0.00015502
OXLD1	-0.602061362	2.84352E-07
ACBD4	-0.602998009	0.000230403
SRP14-AS1	-0.604311831	0.000778865
AKR1C3	-0.604756508	5.48748E-07
LINC00475	-0.605401038	0.006710551
DDX47	-0.605504393	0.000315922
AC133552.5	-0.605860445	0.007176541
ZFP36	-0.606150467	1.92738E-05
COQ8B	-0.606768872	4.93978E-06
PLEKHF1	-0.607772324	0.010752677
OTUD1	-0.607806271	0.02035821
SMAD3	-0.607918319	2.22944E-08
ARHGAP18	-0.608081241	7.22073E-14
ZNF248	-0.608648932	6.80574E-06
C20orf96	-0.608711298	1.00846E-06
VSIR	-0.608943901	8.28937E-05
FAM24B	-0.609352473	0.000203424
ARHGAP20	-0.609494903	0.045990751
ARMC7	-0.609593369	5.03125E-05
TMEM53	-0.610371938	3.36891E-08
DKK2	-0.610400594	0.000253053
KBTBD7	-0.610526811	1.00246E-06
AL031320.2	-0.610699873	0.012571001
AL450998.1	-0.610782164	0.003818433
LIN7A	-0.610863324	0.000165063
FANCE	-0.610912218	0.012834499
MINCR	-0.611555093	3.67359E-06
TGFBR3L	-0.614262108	0.000724537
ZFP36L2	-0.61482527	1.49326E-06
ABHD11-AS1	-0.615078412	0.036264602
ARMCX7P	-0.615622494	0.014450069
GALM	-0.615832179	9.04457E-13
AC134043.2	-0.616195681	4.90421E-05
FAM133CP	-0.616583581	0.040538887
THRB-IT1	-0.617096539	0.047192568

INTS6-AS1	-0.617764906	0.007517006
HSPB3	-0.618032856	2.01619E-09
KCCAT198	-0.618150277	0.000218704
CCDC9B	-0.618503513	2.38178E-08
TTLL7	-0.618771717	0.000197259
CASC2	-0.619810519	0.008716171
THAP11	-0.619811265	3.75531E-07
ORAI3	-0.620742182	2.68707E-05
LDB2	-0.621271432	4.18935E-08
DOCK11	-0.621758572	5.57378E-05
AC016065.1	-0.622334675	4.31156E-05
BAIAP2-DT	-0.623342527	6.88249E-05
ZNF90	-0.623629259	0.012028907
NUDT7	-0.623741379	0.001113774
C1orf134	-0.624327901	0.00347433
TNS2	-0.624506576	4.47659E-05
TXNRD3	-0.624759102	0.002724402
PRR16	-0.624973507	4.95663E-06
BDNF-AS	-0.625155176	0.007026491
GLYATL2	-0.625512235	0.005330971
PXMP2	-0.625755809	0.001562558
ZSCAN16	-0.625875263	0.000339947
FAM86EP	-0.626633251	0.022197526
GNAI1	-0.626639696	1.03E-09
CEMIP	-0.627969794	1.03213E-06
EVA1C	-0.627982027	0.00178064
TSHZ1	-0.628009548	0.000169834
CUEDC1	-0.628301008	0.000199713
AL096870.2	-0.628314542	0.025551301
CCDC125	-0.629295588	1.79177E-05
SNPH	-0.629386054	0.00470412
NDUFA7	-0.629926529	0.038984221
CUTALP	-0.631441521	5.45556E-10
RHOBTB3	-0.631557127	3.79579E-13
CTDSPL	-0.631675746	1.07466E-12
GS1-124K5.4	-0.631724113	6.93749E-06
AC090515.2	-0.631729685	0.009295907
CEBPD	-0.631985094	2.29823E-05
ZNF630	-0.63257285	0.000619221
ZNF563	-0.633071359	0.000295925
AC016582.3	-0.634556263	0.043657992
PRIM1	-0.634906822	1.67375E-05
PGPEP1	-0.63503803	2.12654E-11

ELOVL4	-0.635610048	7.83102E-07
RANGRF	-0.636219563	0.000901416
SNORA84	-0.636435668	0.012501921
AL928921.2	-0.636981353	0.027138018
CC2D1A	-0.637374047	1.74881E-06
TSEN54	-0.637654714	7.86278E-07
TMEM177	-0.638159396	6.23567E-09
ST6GALNAC5	-0.63906809	2.37347E-05
SSX2IP	-0.639424268	0.002389129
CRIP1	-0.640260186	0.005966604
LRRC43	-0.640516924	0.034103313
TRIM6	-0.642517807	0.007546992
PLEKHH3	-0.643173477	0.000264099
CAPS2	-0.643666017	0.0148964
PER3	-0.644213842	2.01601E-07
ZNF793-AS1	-0.644471526	0.027165317
ATP6V1E2	-0.645606345	0.005917797
EHBP1	-0.646883959	3.71385E-15
FAM27A	-0.647345791	0.014765801
AC026979.2	-0.647552082	0.003290398
RSRP1	-0.648296802	1.60727E-14
FAM117B	-0.648497331	0.026732791
AC138207.8	-0.648844985	1.13352E-05
NEIL1	-0.649097012	0.002651113
LRRC1	-0.649941568	0.042579925
NTHL1	-0.649971634	1.43665E-13
PEX11A	-0.650049683	7.06158E-10
JRK	-0.650085019	5.31578E-07
SERAC1	-0.650323857	1.75794E-06
INMT	-0.651178979	9.55866E-05
TOLLIP-AS1	-0.652310501	0.002291203
RTL10	-0.653047951	8.85617E-08
OSGEPL1	-0.654417639	3.67275E-06
TRAM1L1	-0.655129607	0.0006686
FRMPD4	-0.655333022	0.033705333
GAD1	-0.655598362	0.014453019
CSKMT	-0.656679494	6.37729E-10
AC244153.1	-0.657820668	1.47131E-10
EID2B	-0.65833884	0.01176338
MYLK-AS1	-0.658466218	0.03839149
BTBD3	-0.658486338	1.75883E-07
HCP5	-0.658601246	0.042579925
AL031599.1	-0.659691766	0.003472186

AL137782.1	-0.659972037	0.034196696
AL021707.2	-0.660169035	0.01943463
AC068338.2	-0.660357673	0.04516727
PHLPP1	-0.660882117	0.00260229
NDRG4	-0.660908199	0.001685393
AC132192.2	-0.661248214	0.047177493
CASTOR3	-0.661427629	0.00681856
MAP3K1	-0.661804749	9.83797E-06
AP002360.1	-0.662083326	0.000314286
RDH5	-0.663325	0.000151005
LCA5	-0.664967183	1.56566E-06
NR2F2-AS1	-0.665234281	0.007146212
EHHADH	-0.665360434	8.50296E-08
IRF6	-0.666053993	0.025660733
AL158206.1	-0.666325821	0.00285224
ZNF517	-0.66784925	0.007413248
TCEA3	-0.668535144	1.03122E-06
CEP126	-0.668955113	6.51356E-07
AC138409.2	-0.669052559	0.023487292
LGR4	-0.669455772	1.26009E-08
CCDC89	-0.669584234	3.00829E-06
KRT19	-0.67003365	8.24066E-06
BCDIN3D	-0.670176649	1.88153E-11
ARID3B	-0.670494196	0.044219117
LRIG3	-0.670767804	1.18269E-06
ZNF395	-0.671605527	9.20355E-06
TGFB3	-0.672072407	0.000166499
BICRAL	-0.673463284	3.92901E-07
PCYT2	-0.67400981	1.91444E-07
FAM214A	-0.674322233	1.19855E-09
CASP9	-0.67454759	8.40115E-09
AC041040.1	-0.674914184	0.03135861
FZD4	-0.675005128	1.96728E-07
FGD4	-0.675314773	0.000619853
UBE3D	-0.675349369	1.01286E-06
KANTR	-0.675741621	0.000990275
TM4SF20	-0.676122687	7.07447E-05
AHRR	-0.676872263	4.40023E-10
AL136116.3	-0.677483147	3.04475E-05
HIST1H2AG	-0.678124096	0.002775724
SERTAD4	-0.678379253	9.45911E-07
SNX21	-0.679172147	5.0155E-07
LINC02202	-0.679519755	1.67894E-05

LINC00899	-0.679622623	1.41768E-05
ZNF573	-0.680534173	5.54741E-05
HDHD3	-0.680942184	3.68845E-09
PSG1	-0.681422674	6.75546E-05
THEM6	-0.681482289	2.70709E-05
SNHG7	-0.682654996	0.000160485
PLAC8	-0.682683218	2.11278E-06
TRIP6	-0.682824125	1.27674E-06
MVK	-0.682833453	1.40803E-06
NABP1	-0.683205219	1.46519E-12
AC084033.3	-0.683466029	3.77849E-15
FRY	-0.68409024	3.75621E-07
RIN1	-0.684240483	2.70585E-05
SLC43A1	-0.684721475	8.44568E-07
ATP1A1-AS1	-0.684820347	0.00547937
FAM86HP	-0.685909583	0.0021324
SHROOM3	-0.686028015	7.73363E-08
B3GALT4	-0.686643937	0.000361982
LINC01336	-0.686658813	0.039514544
CASC10	-0.686720276	0.002598607
HNMT	-0.686753323	7.31422E-14
TFAP2C	-0.686932198	1.12574E-09
XLOC_001583	-0.686946677	0.024307929
AC002456.1	-0.687768183	0.011346785
ZNF436-AS1	-0.687791547	2.04068E-06
AP002387.1	-0.68862254	0.002892288
AC002066.1	-0.689025665	0.000559274
PJVK	-0.6892418	0.030661632
LZTS1	-0.689294654	0.033746835
EFNA5	-0.68936662	0.000410888
ZNF30	-0.690153114	0.000732303
MIR4754	-0.690172389	0.005406507
RDH10	-0.690833437	3.32305E-08
LINC01534	-0.690928657	0.043381403
SLC25A35	-0.692727079	0.0003734
IDH1-AS1	-0.692990194	0.022062293
PWWP2B	-0.693640973	0.010212817
CDK15	-0.693729566	1.22495E-06
TMTC1	-0.693998807	0.000253396
NEAT1	-0.69629009	5.69109E-08
EPHB3	-0.69650959	0.005388458
TRIM2	-0.696685296	8.75452E-11
CACFD1	-0.696930054	0.000265791

PARD6G	-0.69816479	0.041556515
GPR1	-0.699066408	2.82143E-16
CATSPER1	-0.699258756	0.000293828
DLEU1	-0.699463072	3.40536E-07
AL163051.1	-0.700430751	0.041006466
CFL1P1	-0.701482569	0.043277797
EFCAB7	-0.701835793	3.59679E-06
MTERF2	-0.702010418	2.42183E-09
FAM111B	-0.702436754	0.002879333
AVPI1	-0.702548076	1.46151E-08
E2F8	-0.706114864	0.014999597
LINC01778	-0.706290158	0.025874732
RAB3D	-0.706331769	3.94378E-08
AC010245.2	-0.706648371	0.043392342
FAT4	-0.707348374	1.77623E-11
CDON	-0.70747496	0.012725256
HOXA10-AS	-0.708137803	0.000216315
XLOC_002344	-0.708385603	0.024522012
AC008035.1	-0.708597329	0.006997633
STAMBPL1	-0.711519628	5.70061E-08
AC080023.1	-0.71218893	0.000118809
ZNF503-AS2	-0.714166335	8.26013E-05
CCDC171	-0.715087585	0.000363842
SSC4D	-0.716741254	0.024363543
FAM20A	-0.716846754	0.001331259
RASGRP2	-0.716898498	0.007356612
YPEL3	-0.717694518	1.30027E-08
PDZD7	-0.720642698	0.014542422
KIF13B	-0.721457662	0.001272847
GHR	-0.721693966	0.02609798
ARHGAP35	-0.723496809	1.04373E-12
PPFIA3	-0.723551028	0.034196696
ROR1	-0.724190227	5.76902E-05
GDPD5	-0.724332806	6.60815E-05
RCCD1	-0.724553146	1.59473E-08
AC010864.1	-0.725114039	0.035457561
HTR7P1	-0.726106114	0.001939989
HOXC4	-0.726170875	5.15208E-07
PPIEL	-0.726571615	0.000708975
LACTB2	-0.727638442	8.07924E-25
TMEM131L	-0.728232555	0.00012733
XLOC_010145	-0.731688447	9.61949E-06
HSPB2	-0.731724231	2.31923E-06

DLGAP1	-0.731974162	0.040983966
ISCA2	-0.732961883	4.39275E-20
XLOC_004049	-0.733624125	2.04846E-11
SOCS3	-0.733643624	0.000143923
PAXIP1-AS1	-0.736162102	0.00289157
XLOC_008704	-0.737045271	0.0129536
LYPD6B	-0.737501132	3.20496E-14
SBF2	-0.737527102	6.31149E-11
MYH2	-0.737616355	0.026626627
XLOC_001188	-0.739581316	5.84514E-05
FAM86C2P	-0.739665351	0.000651869
LBH	-0.739973415	1.50928E-07
AC097534.2	-0.740011903	3.35214E-12
LINC01956	-0.745110308	0.005945065
VMAC	-0.746341521	2.20524E-05
AMIGO1	-0.746606517	0.041805576
AC106782.2	-0.746701913	0.009063589
CDHR3	-0.747226486	0.000265418
HRAT17	-0.747956655	0.001263045
LY6G5C	-0.748794169	0.004409928
SLC46A1	-0.750386068	5.26036E-05
RAET1E	-0.750544308	0.038757122
GTF2IP20	-0.752147964	0.018862317
AC011451.3	-0.752456767	0.011306126
NR1D2	-0.754375967	4.26042E-14
ZBED3	-0.755129946	0.049884554
IQCC	-0.755426082	0.00860637
CCDC163	-0.7561183	0.014095799
SH2D4A	-0.756480004	5.03719E-16
HAND2	-0.75652784	0.00152773
SEMA6A	-0.757133549	0.005523038
LURAP1	-0.757601102	1.16079E-05
XLOC_007462	-0.758726375	0.005848051
SHROOM2	-0.760549154	0.00895396
S100A5	-0.761541558	0.013574522
GYG2	-0.762022525	0.008367385
AC139530.1	-0.762351167	0.000127228
EIF3J-DT	-0.764222759	8.31799E-14
AP001160.2	-0.764793547	0.025755978
TMEM155	-0.765931613	1.27289E-05
C16orf86	-0.766549358	0.000163837
AL035563.1	-0.766637254	0.021966271
ZNF497	-0.766686573	0.011984889

LINC00476	-0.766856328	4.80962E-08
ABHD6	-0.767920117	0.00014693
PRKG2	-0.769176648	0.041919775
ZNF540	-0.770635177	0.003554333
CCDC121	-0.771604137	0.013115173
ADAMTS1	-0.774414788	4.66301E-13
ATP6V1G3	-0.775420725	0.000290283
AL121672.3	-0.776013998	0.025712056
SLC27A3	-0.7763212	0.004446034
VWA5A	-0.776941278	1.22099E-09
ARIH2OS	-0.777560511	0.000174882
LINC00342	-0.778917084	0.002537916
LGR5	-0.779118115	0.000420271
SREBF2-AS1	-0.78016545	2.41899E-07
EPHA5	-0.780916648	0.043105276
KRTAP2-3	-0.780995346	0.000124452
AC009061.2	-0.781472343	0.012406184
AC137767.1	-0.78220301	0.000140321
TIMP4	-0.782562016	0.013584073
LINC00265	-0.782651423	0.035732218
AC093323.1	-0.782767723	0.007754024
C14orf93	-0.78285102	7.85515E-07
HOXC8	-0.782855856	4.10339E-09
SPSB2	-0.783060085	6.12459E-13
GPRC5A	-0.783322739	4.59771E-09
LINC00886	-0.783822349	3.37264E-05
KCNK2	-0.783956823	6.54688E-15
ETV1	-0.784756675	6.16798E-05
KIF18A	-0.784831488	1.43056E-05
AL356515.1	-0.78517096	9.11172E-06
XLOC_009668	-0.786689843	0.014331954
GPAT3	-0.790481183	0.010846329
LINC01089	-0.790612167	8.12515E-07
LINC00346	-0.790783043	0.005350717
AC007114.1	-0.791007028	0.012581995
RPP25	-0.79125934	0.000217037
AGFG2	-0.791727239	0.000688371
EFNB2	-0.791897161	0.000109603
RGS14	-0.792258797	0.0093993
CCDC88B	-0.792518304	0.005287797
CCDC30	-0.79295691	0.002167746
AC007098.1	-0.793541719	5.10963E-05
AC107294.2	-0.794815619	0.00160942

CPLANE2	-0.795244761	0.000781064
NRK	-0.796260084	0.006523386
ALDH3A1	-0.796864188	0.044003542
ZNF214	-0.799869337	0.001358808
AJUBA	-0.801321885	2.82527E-09
AC108488.1	-0.801717457	1.76077E-11
AKAP1	-0.802917144	1.13615E-06
THAP7-AS1	-0.804464805	3.10925E-05
AL358472.4	-0.804994181	0.001059119
SAMD3	-0.805040751	0.007668652
AL355338.1	-0.805245747	1.79087E-09
SAMD12	-0.805465708	6.11313E-05
FAM185BP	-0.805872935	0.035913881
SCOC-AS1	-0.80618001	0.004747244
AL354920.1	-0.808384357	2.43656E-06
NCK1-DT	-0.808389683	4.54667E-06
METTL21A	-0.808477247	2.43768E-20
FLJ31356	-0.811553366	0.009048908
STAM-AS1	-0.812106724	0.011163605
SATB2-AS1	-0.812817218	0.012249987
AL390955.2	-0.815144173	5.29053E-05
MINDY1	-0.815223959	5.58379E-14
AC138696.2	-0.816004229	0.003101845
LINC01301	-0.816815502	0.006202589
MGARP	-0.817044834	7.07004E-21
APOC1	-0.817988249	0.038743982
TBX15	-0.819146432	2.59459E-13
C15orf65	-0.81942498	4.07278E-09
SAC3D1	-0.8202915	0.011355076
SETDB2	-0.820938142	2.0185E-08
AP001189.5	-0.821122611	0.049400449
ZSCAN23	-0.821772774	0.011456483
SNX18	-0.822367772	5.46754E-13
BFSP1	-0.822433929	0.003503731
TMEM246	-0.822463673	2.17176E-16
PXN-AS1	-0.823308487	1.38673E-05
HSD11B1L	-0.823445478	1.30587E-07
TACC2	-0.823510721	2.97925E-11
RHOBTB1	-0.824295815	4.51542E-12
RAB11FIP2	-0.826982692	5.7839E-13
XLOC_002951	-0.827287392	0.037479007
LINC01788	-0.827395037	0.002625983
TRIM45	-0.827977616	0.00183724

CDKN1C	-0.830112399	1.72195E-10
LINC00884	-0.833370296	0.010883549
TMCC1-AS1	-0.833390928	0.017661255
SYTL2	-0.833982893	8.43699E-17
ARHGAP28	-0.834285676	0.000322527
XLOC_000294	-0.835440158	0.03017744
RENBP	-0.837340322	0.000278611
KRT80	-0.837628824	0.006254427
TRIM46	-0.838332829	0.012379389
AP001107.5	-0.839418471	0.011078199
KIZ	-0.840802381	2.09676E-15
ARHGEF26	-0.841261087	0.02451578
ID2	-0.8413653	5.017E-11
BDH1	-0.842000853	1.62149E-07
AL137779.2	-0.842136413	0.015311745
HOXA13	-0.842566673	0.014496365
PHF7	-0.843231293	6.46661E-06
COQ10A	-0.843240827	2.15886E-10
NDUFA6-DT	-0.843469967	0.020071466
CAMK2G	-0.844437634	5.40685E-22
MITF	-0.844618845	7.56906E-05
LMOD1	-0.844738704	1.04733E-19
AP001372.2	-0.844748904	0.030967265
MKNK2	-0.844881119	1.07921E-12
TCF7L2	-0.845405951	6.86391E-13
CHAC1	-0.845728309	1.56643E-06
TMEM37	-0.845910732	0.00175395
ANO3	-0.847067722	0.001646931
CHN2	-0.847162175	0.029750915
ARHGEF4	-0.847649773	8.95705E-06
MC1R	-0.847656837	0.045026579
RP11-436G20.1	-0.849647502	0.044779716
AL355472.1	-0.849707601	0.000889651
SFTA1P	-0.850710713	1.24745E-05
GUCY1B1	-0.851030554	1.43521E-05
CABCOCO1	-0.852782268	1.25014E-06
AKAP6	-0.854143586	1.20121E-08
AP002026.1	-0.854614299	3.26686E-07
ID2-AS1	-0.855263576	0.034202293
FSIP1	-0.85661365	1.08873E-06
KLRA1P	-0.858997515	0.014001157
ZNF385D	-0.862484451	6.22663E-08
NR1D1	-0.862946721	8.67489E-07

XLOC_004198	-0.862962681	0.002210393
XLOC_005361	-0.86341112	0.012693628
AC023509.4	-0.865018591	0.029989646
AP001107.3	-0.865832486	0.02586996
MYO1H	-0.865956056	0.009116343
FAM218A	-0.865959298	2.06359E-06
SYP	-0.866519963	0.00807879
BAG2	-0.867885869	6.17962E-18
AXIN2	-0.868835468	1.40804E-05
ZBTB18	-0.869209626	7.55165E-11
AC244258.1	-0.871261884	5.80421E-09
AC098820.1	-0.873181189	0.027782465
XLOC_001460	-0.873243075	0.022641977
AP002884.1	-0.873868462	1.85343E-16
SLC25A42	-0.873944338	7.31247E-06
MKX	-0.874353895	6.21985E-11
ABHD15	-0.874837478	7.44357E-05
XLOC_011753	-0.874895089	0.032756479
LINC01118	-0.87491022	0.000106355
OXCT2P1	-0.875048175	0.000801824
CDKN2C	-0.877433046	1.63638E-16
AL157402.2	-0.877845914	2.73822E-16
XLOC_002900	-0.87817176	2.78155E-08
MISP3	-0.878251503	0.002668761
NRXN2	-0.879541313	9.82758E-08
PLPP6	-0.879815324	1.22097E-07
C15orf61	-0.8801347	1.10635E-15
IQCH-AS1	-0.880224077	2.97218E-07
ZKSCAN7	-0.88142366	1.33097E-05
MDH1B	-0.882182705	0.042021131
FAHD2CP	-0.883039969	1.09511E-07
AL353626.2	-0.883666898	0.044528996
ZC2HC1C	-0.885697335	0.007809414
RIMS1	-0.887883261	1.32417E-07
RAB3IL1	-0.887901547	0.000684415
AC012615.1	-0.889935318	0.000190731
WDR63	-0.890102555	0.000316471
AL359513.1	-0.890103179	0.005833954
PLEKHG3	-0.891418008	0.006966686
IQCD	-0.891726028	0.001609153
KIAA0895	-0.892112159	0.038479398
AL136164.3	-0.893964155	0.01294168
ZNF815P	-0.895430476	0.007763345

KIAA1958	-0.895524315	0.000674418
USP44	-0.896152774	0.044301237
G35570	-0.89646643	0.001241643
SYCP2	-0.897168885	0.000444079
G23966	-0.897574548	0.000782897
GSN-AS1	-0.899396457	0.000239132
AC063944.1	-0.899563544	0.00813381
AL356277.3	-0.899687698	0.000413427
LINC01094	-0.900358967	2.13258E-09
FAM84A	-0.902982474	1.07926E-06
AC110597.1	-0.904157984	0.013380865
AL139156.2	-0.904885235	0.018892389
MIR616	-0.90582639	0.000410927
G4361	-0.907831014	0.032187434
NUDT6	-0.90796311	1.71345E-13
KCTD21-AS1	-0.908075418	0.002965797
HAGLROS	-0.908080907	0.027729914
C21orf62-AS1	-0.908129078	0.003530895
TMEM144	-0.908316708	0.000118245
AC016355.1	-0.91117495	0.038823618
USP3-AS1	-0.912642222	0.037957302
SPAAR	-0.912765173	0.044140463
USP51	-0.912952819	0.032278221
AC245060.5	-0.913126563	0.013489645
PSTPIP1	-0.914347223	0.047873718
C11orf45	-0.914829626	0.016754306
FRMD4B	-0.914981177	0.012720912
AP001033.1	-0.915447775	0.023487292
BMF	-0.915644853	0.039865205
EBF1	-0.915917425	8.12189E-15
LINC01011	-0.91648847	0.005669855
TMEM169	-0.917259217	0.04516727
CYP26B1	-0.917818363	0.007675751
LINC00663	-0.920384814	0.018487185
TRIB3	-0.9206727	2.48997E-09
HOXA6	-0.922856318	0.033784986
RASSF7	-0.923072642	5.23482E-07
DEPDC4	-0.923522765	0.001796549
EIF1B-AS1	-0.925567262	0.000429105
MAN1C1	-0.927705584	4.75216E-07
SYDE2	-0.927845411	0.000690365
PDE1A	-0.928450675	1.14498E-06
AC022400.1	-0.929588701	0.043596506

DKFZp779M0652	-0.929767026	0.009805367
AC103702.1	-0.929999435	5.30167E-07
PHOSPHO2	-0.93034273	3.73318E-08
GCHFR	-0.931257788	0.009912406
AC004812.2	-0.931460007	0.023789423
GDF15	-0.93148091	2.57818E-07
FAM221A	-0.931993322	0.029511828
ABCA7	-0.932169006	0.042118768
PRR15	-0.932296851	0.000949644
LRRC8B	-0.932414338	0.024541097
ITGA9-AS1	-0.932459842	3.9392E-07
AC009414.2	-0.932708642	4.62299E-06
ZSCAN31	-0.933223485	0.000851002
IRAK1BP1	-0.933960505	1.05005E-09
ANKRD65	-0.935220002	0.046199521
AP003469.4	-0.935721853	0.003264447
AL109918.1	-0.936284386	3.86297E-09
NEURL2	-0.937300186	0.029518278
MAF	-0.937674257	4.26289E-12
XLOC_009060	-0.937998246	2.98429E-06
ANKRD33B	-0.938154291	0.017377516
ZFP2	-0.938693649	0.000288602
SEL1L2	-0.939479133	4.07278E-09
AL512329.2	-0.942088373	0.048666594
AC004477.1	-0.94273478	0.001773915
ABHD8	-0.944329457	0.00261225
RAB11FIP1	-0.944700605	8.75767E-08
HOXB-AS3	-0.947258104	5.10239E-21
AC114763.1	-0.948631012	0.013448242
HESX1	-0.949278787	0.01837994
AC116914.2	-0.950256614	0.036111804
HOXB3	-0.951076145	1.2324E-10
C2orf81	-0.951684314	6.22461E-08
XLOC_003471	-0.952837181	0.010396866
KITLG	-0.953761529	1.74878E-11
GACAT2	-0.956318339	5.16588E-05
MAP3K12	-0.956521365	0.000732587
LINC00517	-0.957041075	0.000243217
TMEM86A	-0.957869795	2.03899E-07
FAM86FP	-0.958911382	1.0687E-10
G39927	-0.959496437	0.006910938
KCNMB4	-0.960848342	0.020069244
HOXC5	-0.961950036	7.2711E-05

LHX9	-0.962302262	3.01932E-08
AC007541.1	-0.962322801	0.00831917
SH2D5	-0.96241334	0.007356612
C9orf170	-0.965247789	0.021882738
CNNM1	-0.96561532	0.045069715
AC023794.3	-0.966515903	1.03775E-05
AL021368.2	-0.97007977	0.001303101
IL20RA	-0.970141802	0.001420226
XLOC_002952	-0.970620586	1.93538E-05
LINC01637	-0.97175423	5.52209E-08
AL138756.1	-0.973524206	0.041360921
ANKRD35	-0.974040092	5.55281E-11
MTSS1L	-0.975060261	9.0803E-06
RIMKLBP2	-0.975784511	0.040099155
NOSTRIN	-0.976127394	0.001977932
AL022476.1	-0.97673831	0.027327475
CDC20P1	-0.976811001	0.010608274
MIR99AHG	-0.978023336	2.2973E-09
AL049838.1	-0.980468204	0.000868124
LINC00211	-0.981971769	0.010890619
PPP1R26-AS1	-0.982478045	0.035379753
LIPT2	-0.982842749	2.30513E-05
PITPNA-AS1	-0.983385496	3.04975E-09
ARRDC4	-0.983599359	5.3674E-13
DNAJC28	-0.98607899	1.1323E-05
AC107884.1	-0.987978639	0.002259003
XLOC_000566	-0.988243716	0.021733457
HOXC6	-0.989364513	4.57949E-10
ATP8B1	-0.989635046	3.0521E-25
ALG1L9P	-0.991044314	0.031688303
RCAN2	-0.991118772	2.48424E-13
PLD1	-0.991140592	2.30491E-05
CTXN1	-0.994470884	0.005917797
AL022157.1	-0.994762333	0.036031243
ZMYND10	-0.99547286	0.003567155
NUDT18	-0.996892606	2.43694E-06
TMC3-AS1	-0.997222397	1.72384E-07
BX537318.1	-0.99884777	0.000154485
PTPRH	-0.999778771	0.038197526
SLC26A1	-1.001291437	0.04674688
TSPAN10	-1.00267807	0.000225742
SPEG	-1.005415987	2.24923E-11
LINC01936	-1.00655512	0.027547013

AC106791.1	-1.006856371	0.036921981
AP003068.2	-1.009817161	0.018931179
ZNF781	-1.011628742	0.015780603
TRIM7	-1.013502541	0.001212727
AC025181.2	-1.013940829	0.000931547
AC007383.2	-1.016147614	5.89687E-05
CBX8	-1.016295969	3.53276E-07
SLC11A1	-1.016940096	0.000517189
ELOA-AS1	-1.020062828	3.67245E-06
AC105383.1	-1.020466483	0.046841624
DAPK1	-1.02237943	3.77405E-06
KCTD4	-1.025438919	2.49461E-06
CPEB1	-1.025650796	0.009085457
TTC28-AS1	-1.025730444	9.30897E-08
CCDC136	-1.026775957	2.00692E-07
AC037486.1	-1.027148816	0.027685051
PRCD	-1.027202945	0.049504644
AP005264.7	-1.028589916	0.036233955
LINC01134	-1.029999513	0.036264602
PLD6	-1.034558699	5.04427E-05
SNORA77	-1.035331206	0.012634529
CNKSR3	-1.035571853	2.38769E-05
MRVI1	-1.03797081	2.86832E-27
TIGD2	-1.038624078	7.60107E-08
AL590822.1	-1.039508295	0.002386738
GDPGP1	-1.040318772	1.67719E-05
ZNF589	-1.041951992	9.38324E-07
AC089983.1	-1.04199544	1.34491E-06
GIPR	-1.042126078	0.02628216
PKIB	-1.043014249	0.017189526
RASIP1	-1.046414328	0.002040537
HOXA1	-1.046423544	0.026991344
GVQW3	-1.04679849	0.011512939
HDAC2-AS2	-1.046909294	0.000239249
MORN3	-1.048105232	0.00250706
LINC02019	-1.050153023	0.001361097
LINC01563	-1.052137735	0.027978674
ZNF33B	-1.053995757	1.87652E-06
AL365214.2	-1.054288195	0.023772086
AMOT	-1.054392239	6.07707E-07
CHDH	-1.054774858	0.027206849
NR2F2	-1.055130346	2.9614E-21
ANKRD30B	-1.056646276	0.001962295

PWAR6	-1.057138553	0.011147712
SEPSECS-AS1	-1.057261592	0.001054828
XLOC_000403	-1.05787708	0.006783137
THAP8	-1.060036163	5.00973E-16
SBF2-AS1	-1.060043623	9.36284E-21
Z99289.1	-1.060102651	0.037456146
TGFBR3	-1.061119512	4.03713E-15
TEF	-1.061630602	2.78678E-06
PURPL	-1.064638193	0.000619362
ARMC4	-1.065421969	8.74379E-06
L3MBTL1	-1.068161798	0.00110191
RABGAP1	-1.071916924	3.19061E-19
ZIC4	-1.072342917	0.002628988
MEIS2	-1.076251595	2.16809E-21
GPR39	-1.077576397	0.025924055
MYCBPAP	-1.080908378	0.031218507
MTCL1	-1.082386142	6.78287E-12
44264	-1.084357207	0.001206749
AC013394.1	-1.084446813	0.028714319
CHRM2	-1.085668222	0.001262182
AC023632.2	-1.087019418	0.040849543
DPH6-DT	-1.087142951	0.002965262
ERICH5	-1.087711211	0.000308575
FAM217B	-1.088220568	1.39652E-11
BDNF	-1.08869033	4.50097E-09
FAM198B-AS1	-1.088745663	4.22397E-06
AC138150.2	-1.088797083	0.000665662
ANKEF1	-1.089500906	1.36391E-05
AC114284.1	-1.090475139	3.91428E-10
SPIN2A	-1.091508241	0.000205315
XLOC_011981	-1.092225833	0.009637666
AQP11	-1.09333246	1.07306E-05
FAM13C	-1.095811974	0.018967684
LENG9	-1.097748576	0.020175093
PKP3	-1.099658461	0.007097608
SUSD3	-1.0999014	0.018866272
XLOC_002888	-1.100801377	0.025374567
NR3C2	-1.100853377	0.002697617
STON2	-1.101466132	0.001264632
SLC38A4	-1.103765272	0.000410365
AP001273.1	-1.105210807	0.017504753
AL035448.1	-1.110071104	0.011856883
SALL2	-1.11057036	0.042550495

SSR4P1	-1.110763352	0.028188896
LINC02595	-1.110897762	8.23078E-11
AC016757.1	-1.113593734	0.034581575
NXNL2	-1.114911639	3.31386E-07
BX284668.4	-1.114950385	9.13599E-08
MAOA	-1.115236482	7.23277E-05
CYYR1-AS1	-1.115437156	0.004610615
AL355001.2	-1.117258393	0.000546989
LINC01569	-1.120540004	0.012479274
LINC00840	-1.12242339	4.37757E-05
SEC31B	-1.12289173	0.002571863
ARL4D	-1.123356682	2.75739E-15
RUNX1T1	-1.126520541	2.73645E-18
AC254633.1	-1.127896921	0.000253053
KCNJ2	-1.130315958	8.13404E-07
TFAP4	-1.133464571	1.70087E-07
AL161911.1	-1.133613692	0.031635005
FAM86JP	-1.134513298	0.000101014
RPARP-AS1	-1.136548935	0.002342909
AC090877.1	-1.136567077	0.043343505
CPA3	-1.137012759	0.017085902
METTL7B	-1.137033956	1.60697E-05
ANXA9	-1.137900641	0.00037363
PRICKLE1	-1.138043681	9.21855E-12
DNAJC9-AS1	-1.138173344	1.3493E-06
LINC00565	-1.139230606	3.71059E-07
ADRA2A	-1.139317782	1.81756E-07
INHBE	-1.139400651	3.3232E-07
CCNO	-1.14167881	0.001914147
SOCS2	-1.144370526	3.718E-16
LNX2	-1.144943717	4.05141E-07
OSR2	-1.146931448	1.94744E-05
XLOC_001374	-1.147502073	0.038792507
LINC01546	-1.147585879	0.005540263
AC005532.1	-1.150718926	0.031166283
PPARG	-1.152746939	5.00705E-06
FALEC	-1.156591	0.004353811
SIM1	-1.158209345	5.76036E-14
AL133355.1	-1.158668446	0.04105462
THRB	-1.160948262	1.01345E-14
IGSF10	-1.16148162	0.001954413
LINC00547	-1.163174292	4.52475E-05
ZIC1	-1.164022089	2.60823E-06

HOXA10	-1.164116017	2.99276E-18
MYO15B	-1.164712302	0.032064615
AC092171.5	-1.165499517	1.47518E-11
PSCA	-1.167374878	1.27014E-06
AL357146.1	-1.170949176	0.000421063
CCDC54	-1.171063589	0.023696789
PPL	-1.17111461	0.000289508
SOX12	-1.171234238	2.04846E-11
STXBP6	-1.173415249	6.9119E-05
BCL11B	-1.174173717	0.007770965
DDIT3	-1.17420705	5.9677E-23
SIRT4	-1.175898709	2.6898E-06
AC108062.1	-1.176400753	0.000175143
CPAMD8	-1.177707053	0.009041246
IMPA2	-1.179568347	1.04529E-05
LINC02550	-1.180017686	0.034524516
AC007906.2	-1.181214525	0.022415258
GRPR	-1.182537686	0.000674359
SEMA3B-AS1	-1.184851764	0.005926325
AC006273.1	-1.184882451	0.018487185
44256	-1.18613382	0.003221907
AP001092.1	-1.187466584	0.006587922
AF186192.2	-1.189112694	0.024301739
LRRK2	-1.190293102	1.83468E-05
RXFP1	-1.190574726	0.000919351
ACSS1	-1.191440691	0.001136813
AL162231.2	-1.194143852	4.86327E-06
NATD1	-1.19439317	1.0959E-13
ZNF192P1	-1.195107355	0.003510284
FLRT3	-1.196335554	0.00090811
ARHGEF37	-1.196819367	0.000354988
HOXA5	-1.206437287	0.002607947
LKAAEAR1	-1.20715171	0.039788644
LACTB2-AS1	-1.210242319	0.001452393
PCSK4	-1.211182088	0.011019589
ZMAT4	-1.211530809	0.020479943
SSTR1	-1.211637805	4.94006E-09
STX18-AS1	-1.212828157	1.19572E-05
PALMD	-1.212893317	1.03279E-07
GAS1RR	-1.213876211	2.207E-15
EMX2	-1.214432859	6.26435E-30
AC011389.1	-1.216927119	0.005417336
AC011444.1	-1.220346481	0.003712723

SUCLA2-AS1	-1.221107056	0.005577287
TWIST1	-1.221667862	8.19917E-25
DEF6	-1.222618662	0.017794417
AC090360.1	-1.225079766	0.041241039
ID4	-1.22639994	4.53949E-09
SNORA26	-1.226759604	0.001453377
AC023908.3	-1.227953203	0.000901416
PCDH18	-1.237755142	2.05733E-14
NBEAL2	-1.237798661	0.007258249
A2M-AS1	-1.238965548	0.031104571
TNFRSF19	-1.240348528	9.28348E-19
AC007953.1	-1.243066662	0.006492534
SLC14A1	-1.244632249	0.000104897
XLOC_002925	-1.24548986	0.011462165
RGMA	-1.246317492	6.14848E-08
CALCRL	-1.248165453	3.63552E-05
AL023581.2	-1.2482637	0.027979542
AP001412.1	-1.250296544	0.042403595
LINC01085	-1.252138175	0.000185684
LINC01111	-1.252834949	0.007819261
AC004130.1	-1.253112449	0.000155135
HRCT1	-1.253845054	1.6314E-09
MAMSTR	-1.254939098	2.49977E-08
AC010173.1	-1.25642481	0.00406321
DEPTOR	-1.256621317	5.71298E-18
PDE3A	-1.256878286	5.58574E-07
TNKS2-AS1	-1.258798111	0.02638949
ZNF713	-1.259181952	0.005483368
XLOC_007348	-1.26450942	1.50343E-09
AC005674.2	-1.266858386	0.027411929
TRERF1	-1.2717067	2.40817E-12
AC005696.1	-1.272262338	0.0012896
G7528	-1.272917632	0.034821337
AL353141.1	-1.27339789	0.047045984
DLK2	-1.273424894	0.039358621
CCDC96	-1.275144303	0.003970952
ANKRD34A	-1.277581039	1.55386E-08
ELL3	-1.278052008	0.014021182
AP005264.1	-1.281527954	0.001974208
LINC01305	-1.283612775	1.5715E-05
HOXA11-AS	-1.286940851	3.00054E-06
FBXO16	-1.290353832	0.000100396
EBF2	-1.291059221	3.36913E-06

XLOC_000263	-1.291479687	0.029519468
AL080317.2	-1.29516111	0.021349577
TAF1A-AS1	-1.297774537	0.003631062
G6313	-1.29897857	2.86128E-05
XLOC_007506	-1.301791686	0.016160002
PWAR5	-1.302604002	0.018931179
AC012470.1	-1.304372764	0.029238485
LYPLAL1-DT	-1.306359938	0.03872289
MYLIP	-1.309475154	6.55288E-05
MRGPRF	-1.309525407	4.28042E-18
PANO1	-1.311659227	0.016965981
IRX5	-1.312806679	4.4431E-10
LINC01550	-1.313916698	0.001743483
AC092171.4	-1.314113137	0.037954614
TET1	-1.320402084	3.29733E-12
ANKRD20A5P	-1.32086814	0.003299484
HOXB8	-1.321955087	0.000716231
AC131235.3	-1.322274018	0.037456146
XLOC_005587	-1.323058217	0.004706824
SYNPO2L	-1.330538919	0.042309216
SLC9A3-AS1	-1.332779992	0.001527858
HOXA9	-1.334845738	6.82424E-13
XLOC_002552	-1.335119212	0.000201844
KHK	-1.335309287	0.00015579
SEMA6D	-1.338568166	5.1423E-08
AC008507.1	-1.339438335	0.016626359
SLC25A27	-1.34405467	3.78391E-06
RASSF9	-1.345143459	0.000198132
AC026250.1	-1.345727299	3.9159E-10
RASL11A	-1.350051192	0.045705126
XLOC_001527	-1.352516993	0.001423856
LRRC20	-1.354077354	0.001059145
AC092171.3	-1.363357358	0.023081874
MBOAT1	-1.368972227	0.000296014
AC011365.1	-1.369595325	0.013516566
XLOC_011016	-1.370734149	0.00099751
AC107398.2	-1.370749754	0.038551899
MTUS2	-1.37210199	2.40364E-05
AC011389.3	-1.374487587	0.001486547
AC145285.2	-1.37790048	0.009064527
WEE1	-1.381307152	4.54831E-20
TBKBP1	-1.382737814	2.05811E-07
FSBP	-1.383243769	0.024349039

S1PR1	-1.387507116	7.49465E-15
SAMD5	-1.387917581	0.006349874
ADAMTS5	-1.390902593	8.03715E-21
GPR162	-1.390994432	0.012686152
OSBPL7	-1.391340031	0.002386051
AC092535.4	-1.391745587	0.027965401
XLOC_004822	-1.391864459	3.21248E-09
NUDT16P1	-1.392741241	8.67395E-06
FLJ46284	-1.393937023	0.016661465
ADAMTS15	-1.400064394	0.042409385
C19orf73	-1.402382969	5.27262E-08
INKA2-AS1	-1.404374436	0.001477802
TMEM26	-1.407484682	0.000856087
AC051619.5	-1.40927144	0.006701838
CASS4	-1.409749689	0.008034768
FGF10	-1.411569865	1.91176E-06
XLOC_012692	-1.411586523	0.017411906
AC087477.2	-1.411871097	0.000324907
ACTL10	-1.416338836	0.005954013
AL592158.1	-1.416929285	0.003525352
B4GALNT4	-1.417780924	0.029107763
NOG	-1.419981269	3.59864E-08
Z97634.1	-1.4222595	0.030904729
C11orf88	-1.422813217	0.020278972
AARD	-1.424706854	1.75883E-07
AL590560.1	-1.425448363	0.027165317
MYHAS	-1.426365346	0.001750641
XLOC_002543	-1.426551105	0.012238273
CITED2	-1.427106996	1.48687E-23
CISH	-1.433114521	0.000144642
FIGN	-1.434400395	7.93807E-07
NLRP10	-1.434551653	1.11951E-05
AC003102.1	-1.436993697	1.14992E-05
TRIM17	-1.438045663	0.01456706
AC022154.1	-1.439726307	0.017533269
AL359504.2	-1.439861462	2.07962E-08
AP000813.1	-1.440254616	0.019626776
REM2	-1.441222039	0.010296105
GCK	-1.444918493	1.01056E-05
MME-AS1	-1.444934144	0.019924155
LIPE	-1.44661604	0.008180329
AP000662.1	-1.45368562	0.003893202
SH3TC2	-1.454211182	0.001593103

LINC00484	-1.457904898	0.046939286
HOXA7	-1.460175581	2.0474E-09
INKA2	-1.460934838	3.71852E-05
ADIRF-AS1	-1.465642039	8.11588E-06
GLIPR1L1	-1.46715802	0.005687953
MATN1-AS1	-1.47050419	0.01857694
VWCE	-1.474423506	0.003394424
IL6R	-1.479596661	6.41119E-11
GDPD1	-1.480391239	0.000532485
CAVIN2	-1.48281466	1.04826E-30
KBTBD3	-1.483539833	2.16651E-23
AC026801.2	-1.483553588	0.03600968
LINC02361	-1.48370976	0.015244586
DDIT4L	-1.484888191	2.68925E-15
AP003071.4	-1.485485017	0.012485713
HOXA11	-1.485818613	1.16371E-11
MEOX2	-1.487537438	1.2582E-20
AC120036.3	-1.487545711	4.69961E-05
EYA4	-1.487973808	0.01143045
G8154	-1.491404595	0.007957955
FAM78A	-1.491760167	0.004424423
ADM	-1.492910739	1.68297E-16
AC245060.6	-1.494611624	0.018444691
LINC01273	-1.497644402	0.001416476
XLOC_002440	-1.505309373	1.57251E-08
MBP	-1.505458259	5.74151E-08
XLOC_009535	-1.505853634	0.001279337
AL844908.1	-1.505897969	0.000691447
FZD2	-1.509760478	1.58234E-20
SAMD13	-1.516248283	0.027165317
BX470102.1	-1.518523626	4.92086E-06
XLOC_006829	-1.520318138	0.029131808
G2112	-1.521515185	0.020278972
CYSRT1	-1.524246503	1.58355E-05
ARL4C	-1.526176944	2.03756E-19
GRTP1	-1.530602874	3.21768E-05
XLOC_000917	-1.532865969	0.000896389
AC144652.1	-1.537161471	0.013974009
CSRNP3	-1.538540701	0.023605932
RPS3AP24	-1.545224473	0.032174214
AL358176.4	-1.546375001	0.002336734
AL109924.2	-1.547207783	0.000105107
AL590428.1	-1.549504265	0.000153285

PLCE1-AS1	-1.550928673	1.652E-11
XLOC_003703	-1.553081253	7.85523E-06
AC027237.3	-1.558084577	4.14648E-08
AL353593.1	-1.558883428	0.010053038
RCOR2	-1.564103113	0.043915812
LMO7DN	-1.56563152	1.68133E-05
SPRY1	-1.566251173	8.82345E-11
AC021054.1	-1.570313832	4.55442E-06
OPRL1	-1.584329298	8.43624E-06
AC003986.2	-1.585633031	0.000110619
LINC01444	-1.591080751	1.4327E-07
AL132780.2	-1.591132037	1.42834E-05
G28383	-1.594072824	9.7793E-05
SIPA1L2	-1.598197718	7.8722E-10
KCNS1	-1.598389131	0.004586579
TMC3	-1.618019003	0.010789275
FRAT1	-1.61879922	0.011439477
AC138207.3	-1.621957624	0.004033288
LINC00327	-1.624130515	2.58016E-14
MPP7	-1.626796107	3.18576E-12
PDE7B	-1.626801454	2.9705E-10
AC009093.1	-1.630538425	3.37264E-05
RIPK4	-1.630932476	5.8638E-05
RERG	-1.633485444	0.046034705
FLNB-AS1	-1.634832449	0.001926661
AL109741.1	-1.636706975	0.002337983
STOX2	-1.63816427	0.041956739
SLC40A1	-1.641847028	6.91607E-06
CCDC102B	-1.64594685	3.70586E-10
AC007920.2	-1.647696016	5.26248E-13
TTC34	-1.650472424	0.019924155
DICER1-AS1	-1.653837369	5.88957E-07
FUT1	-1.654335254	0.022667757
GPER1	-1.65714707	4.174E-20
SLC7A4	-1.664735224	0.01705507
RTN4RL1	-1.666735084	0.013291181
PRSS36	-1.669974158	4.45754E-05
MKX-AS1	-1.672648862	2.40817E-12
LURAP1L	-1.672750513	6.09396E-30
BRINP1	-1.677183091	6.99717E-26
AC078881.1	-1.677768588	0.019807031
LINC01443	-1.682905536	0.005758342
AP000911.1	-1.684338462	0.002077454

PAN3-AS1	-1.6847473	0.002409611
RGCC	-1.686753305	8.086E-05
FAXDC2	-1.690560008	2.53492E-26
AP006623.1	-1.690986244	0.037372495
FAM84B	-1.697447168	9.45069E-05
XLOC_007038	-1.700684487	0.023886446
ZNF385B	-1.709701064	0.025509535
ZNF608	-1.713337164	2.42957E-05
IRX6	-1.717014901	0.043181435
GAS6-AS1	-1.725133982	0.017931757
KRTAP1-1	-1.730564957	3.0961E-06
AC011369.2	-1.743767188	0.015036448
AC120036.4	-1.746198127	3.18492E-06
JAKMIP3	-1.74665811	7.60885E-05
EYA1	-1.748882995	0.027362009
KCNE3	-1.749571839	0.000101768
AC040160.1	-1.752481711	2.10503E-05
RTN4R	-1.755364234	0.00048933
NALT1	-1.758796667	0.027394718
LINC00857	-1.759485838	5.76275E-08
PNMA8B	-1.76499889	0.003827091
XLOC_003261	-1.767231106	0.000396377
AC018755.1	-1.777920898	0.014593358
EEPD1	-1.781169605	1.10203E-12
TSHR	-1.786227935	0.000689719
AC010997.3	-1.791218088	0.006804196
AC110048.2	-1.791548733	0.004043537
SH3RF2	-1.793307494	1.30336E-06
EXPH5	-1.793404697	5.54155E-14
PDK4	-1.793844437	1.34115E-12
AC005550.2	-1.795790634	0.000189826
TRPC6	-1.796378301	7.59764E-10
LINC01750	-1.809459194	7.58052E-08
NAPSA	-1.81276325	0.001531978
ZNF396	-1.815101415	2.58242E-10
TRIM55	-1.826207737	0.007087627
AL138689.2	-1.830648932	0.041587793
EVI2B	-1.847125303	4.11815E-09
KCNJ8	-1.857484141	3.56412E-12
DBP	-1.869271391	1.16852E-16
ANKFN1	-1.874301854	0.001319422
AL109924.4	-1.878406583	0.046573446
FBXL22	-1.884397409	9.13868E-05

AC090152.1	-1.884493091	5.71298E-18
AC117395.1	-1.89773283	0.005497111
XLOC_010311	-1.901885094	2.8713E-08
GAS1	-1.910148582	1.1123E-27
XLOC_001457	-1.915781216	0.001012438
ADH1B	-1.922241573	0.002625983
TOB1-AS1	-1.92681378	0.0001969
KLRG1	-1.929078424	0.000296014
XLOC_007920	-1.937323581	0.004742653
AC009093.2	-1.943308068	1.25938E-06
PIK3C2B	-1.953239889	7.72456E-10
CNKSR2	-1.970492415	1.4748E-14
AC069224.1	-1.973256044	0.049094653
ZNF233	-1.990381231	9.12559E-07
GSC	-1.991824694	3.43682E-05
SP9	-1.999144757	9.28913E-05
LINC02172	-2.007830919	0.0030816
YPEL4	-2.011563136	0.000412343
AC008708.2	-2.039011998	5.32888E-09
AC010359.1	-2.047363773	0.000814818
AL355596.1	-2.075613467	0.000287942
SMILR	-2.087971148	3.68968E-15
EVI2A	-2.098648902	1.26992E-16
XLOC_006390	-2.100197489	1.83658E-06
SIX2	-2.110235649	0.004532586
LINC00398	-2.118392335	0.007844735
AP001596.1	-2.1214768	0.000137469
TENT5B	-2.12615059	1.61366E-06
AC027808.2	-2.14118382	0.002505471
PRRT1	-2.145006029	3.51084E-06
ZSWIM2	-2.145422287	0.001481182
LINC01836	-2.148525529	1.00108E-07
MAP2K6	-2.174568184	5.51368E-16
KRTAP1-5	-2.210495627	3.6577E-29
LMO3	-2.233045316	7.46013E-05
PKD1L2	-2.23927017	0.003762941
Z99289.3	-2.242589841	0.000597202
INHA	-2.248247253	2.21747E-06
LINC00954	-2.262919426	0.000265418
AC124947.1	-2.270682709	8.45552E-05
AL513534.1	-2.271011413	7.39714E-14
XLOC_003463	-2.30103428	0.011439477
TOX	-2.310244324	9.52266E-23

AC022395.1	-2.391897891	4.65052E-06
AC116351.1	-2.408181223	0.027073897
APELA	-2.411004331	4.70716E-07
LINC02587	-2.449977344	1.33448E-05
PPIAP39	-2.464545868	1.62363E-09
XLOC_008955	-2.496153972	5.04369E-14
PPARGC1A	-2.516750265	3.43067E-06
FGF9	-2.565233241	3.34861E-07
C11orf87	-2.567340875	7.02787E-26
OSR1	-2.601458138	6.34025E-23
MSTN	-2.608313892	5.60377E-07
AC008705.1	-2.667132363	0.000635744
LUARIS	-2.681604987	0.000506972
BMP4	-2.722998531	1.93844E-14
CTB-178M22.2	-2.755919325	1.53337E-07
CYP39A1	-2.757698204	2.97218E-07
AC109779.1	-2.760497508	4.25139E-13
FILNC1	-2.942359466	1.55485E-13
NFE2	-3.252764318	0.001389324
GDF5	-3.616668978	1.28459E-19
G30615	-3.639980837	5.59589E-05
MC4R	-3.831470184	4.47401E-17
KIT	-5.182154278	6.28255E-26

Gene	log2FC_.Non.Damaged._.IFNA	padj_.Non.Damaged._.IFNA	log2FC_.Damaged._.IFNA	padj_.Damaged._.IFNA	ES_.Non.Damaged._.Damaged._.IFNA
AL356235.1	-3.252784586	0.001808032	2.997573667	0.070192072	6.250358253
HIST1H2BF	-3.879539696	0.03265849	0.616911346	1	4.496451043
KCNC3	-4.104026598	0.00168848	0.304173144	1	4.408199742
HSD11B1	2.667288225	0.016913299	6.504779704	2.89126E-06	3.837491479
MC5R	-3.741957199	0.002572382	-0.108736753	0.977664147	3.633220446
FRMD7	1.841659082	0.506012021	5.41763982	0.011305089	3.575980739
DNAAF1	2.410420024	0.09561123	5.922981996	5.36397E-08	3.512561972
IFNB1	2.675924633	0.594964589	6.151725631	1.17146E-05	3.475800998
AC112128.1	-3.632502668	0.021295641	-0.207369346	0.932561265	3.425133322
SLC38A3	-4.113524806	0.006103354	-0.794184116	0.777773973	3.31934069
AC008074.2	0.16735707	0.93482762	3.398556497	0.003300616	3.231199427
LRRC37A7P	-3.218216023	0.010412766	-0.007517731	1	3.210698292
ARAP2	1.183948736	0.070618399	4.359090144	7.71796E-06	3.175141407
AL592424.1	-4.308249068	0.018926016	-1.152088641	1	3.156160426
BCL2A1	5.864785934	1.01265E-07	8.95722323	2.12164E-10	3.092437296
XLOC_002041	0.792353886	0.693348162	3.874878031	0.049041492	3.082524144
PLEKHG6	1.203422663	0.575704453	4.260828573	0.022113953	3.05740591
CYP27B1	2.705859086	6.26016E-05	5.736384569	1.40119E-09	3.030525484
ZNF366	2.058329682	1	5.039028221	0.024345777	2.980698539
HNF1B	1.976503371	0.101498132	4.934858398	0.001579226	2.958355028
XLOC_005458	-3.470683964	0.044223482	-0.513416358	0.887511219	2.957267606
NT5CP1	0.849671465	0.382965969	3.789464019	0.00506264	2.939792555
AC092296.2	-4.516170659	0.000453877	-1.615738609	0.521457435	2.900432051
SERPINB4	2.554000394	0.025177794	5.450816212	0.035204432	2.896815818
PTGER4	0.025212706	0.985759756	2.916628669	0.004434244	2.891415963
BMP3	0.291286494	0.870908801	3.122170915	0.043464471	2.830884421
AC069224.1	0.474650225	0.881568246	3.263065123	0.045829551	2.788414898
ENPEP	0.836433159	0.259312807	3.607287365	8.05871E-05	2.770854206
IRF8	2.716097719	0.587423133	5.450134473	4.52606E-06	2.734036754
AC020915.1	-3.821685072	0.003117886	-1.104070722	0.534382985	2.71761435
DHCR24-DT	-4.189127372	0.022541381	-1.471732336	1	2.717395036
XLOC_004201	1.926159242	1	4.59875979	0.004327602	2.672600549
C6orf163	-1.364275236	0.044037021	1.243289318	0.202062486	2.607564554
NIP7P3	-3.380926143	0.043994559	-0.786220928	1	2.594705216
AL022345.4	0.207586091	0.921740217	2.80129267	0.027110752	2.593706579
CXCL5	5.002820266	1.45869E-11	7.595621908	5.02138E-09	2.592801642
RP11-380M21.4	-2.369411194	0.042975321	0.206481232	1	2.575892426

PWAR6	-3.822487409	1.63007E-05	-1.277333395	0.341050357	2.545154014
ZNF474	-2.040563606	0.025350595	0.5033644	0.634320329	2.543928006
FER1L5	-2.298658581	0.008934604	0.244641714	0.893935621	2.543300296
RPA4	0.627170147	0.55978711	3.167157477	0.00104072	2.53998733
HLA-V	1.893737568	0.026733514	4.425774287	0.000615457	2.53203672
AC010168.2	0.140837366	0.916477975	2.668064635	1.0575E-06	2.52722727
AL161891.1	-2.693660468	0.013980711	-0.171352735	0.946490198	2.522307734
PCA3	-4.380376657	0.004601009	-1.858397084	0.298550509	2.521979574
PRKCQ	2.393317097	0.551422233	4.904215455	0.038974304	2.510898358
CXCL6	4.344253333	1.67704E-11	6.852754334	5.62202E-10	2.508501
AC092919.2	-3.833529593	0.03061351	-1.334063917	0.368776489	2.499465676
AC005899.8	2.004171564	0.047811321	4.471906154	0.003792882	2.467734591
AC083837.1	6.287528902	3.94639E-18	8.738783533	9.36288E-11	2.451254631
CD69	6.50905389	0.001050035	8.935467487	5.69158E-24	2.426413597
AL683813.1	-2.897737697	0.028317948	-0.473600632	0.854402805	2.424137065
RASGEF1B	3.675914607	2.23589E-06	6.090363611	3.96292E-11	2.414449004
AL137186.1	2.017669563	0.36468201	4.421241343	0.005108376	2.40357178
TTC4P1	2.342250379	0.02764942	4.74351205	0.002136323	2.401261672
LYZ	-2.738113646	0.042883347	-0.344366851	0.895324696	2.393746795
AL356234.3	4.482086879	4.01293E-06	6.857959946	8.77098E-11	2.375873067
AP001596.1	-3.88451995	0.000377701	-1.541624054	0.154754645	2.342895896
AC022816.1	4.194360229	0.000229451	6.517222807	2.95708E-05	2.322862578
IFI30	5.525938332	1.59071E-14	7.840767211	1.12449E-14	2.314828879
AC073862.4	2.941635934	0.127631304	5.256076532	0.000295869	2.314440598
XIRP1	6.071039823	4.56537E-11	8.376890602	6.41647E-08	2.305850779
C19orf38	0.71845491	0.448056057	3.024120231	0.02222518	2.305665321
AC004988.1	3.7649853	1.24512E-07	6.025225595	2.70831E-07	2.260240296
CFAP161	1.54886102	0.042283448	3.799499761	0.001175729	2.250638742
FRRS1	2.056901819	0.022017285	4.292938516	0.002611256	2.236036697
AC131649.2	2.279147603	0.22743498	4.507863003	0.012022724	2.228715399
AC067838.1	-2.629127298	0.00218226	-0.406480145	0.661791496	2.222647153
CCDC162P	1.233583381	0.104600197	3.440785912	0.020349244	2.207202531

HIPK1-AS1	-2.701725386	0.006314184	-0.517348582	0.889303512	2.184376804
NR4A3	3.165645271	3.20016E-09	5.343820213	2.12895E-23	2.178174943
P2RY1	3.605897846	0.00012682	5.781544058	0.000106227	2.175646212
G7822	-2.905402123	0.011407177	-0.730913262	0.703784278	2.174488861
XLOC_011313	-2.350770616	0.00084852	-0.204483144	0.893514283	2.146287472
EGFLAM	0.575599814	0.461366981	2.72168513	0.001909232	2.146085316
AL132780.4	-2.520220404	0.000256715	-0.376224904	1	2.143995501
IBSP	2.808392967	0.005889724	4.949976445	0.002604198	2.141583479
CSF2	4.648181362	2.00459E-06	6.788580082	8.64702E-10	2.14039872
AC008147.2	0.135669625	0.91634768	2.253320665	0.000441731	2.11765104
STARD8	-0.12840416	0.920612569	1.98030379	0.000697554	2.10870795
CD79A	1.056701304	0.571715473	3.163116985	0.038172035	2.10641568
HEATR9	3.94630816	0.041746489	6.051541624	1.33093E-05	2.105233464
AL137009.1	0.939399816	0.637513269	3.024017272	0.011517537	2.084617456
COL4A3	-2.099597207	0.03821952	-0.016457893	0.993730228	2.083139314
PPARGC1A	-4.391824778	3.78936E-05	-2.331422362	0.033021098	2.060402415
AC099489.1	4.009747474	4.73628E-07	6.070084092	4.54528E-15	2.060336618
HCK	3.145264043	5.11152E-05	5.204802791	0.000223326	2.059538747
FAM107A	7.600952027	7.48589E-23	9.659334098	3.48654E-13	2.058382071
B3GNT5	1.033491403	0.212131262	3.082970439	0.005187705	2.049479036
AD000864.1	0.194057221	0.869530723	2.232129863	0.015959811	2.038072642
CP	1.348268782	0.282650577	3.38443452	9.59452E-05	2.036165738
TP63	3.148654041	4.83422E-05	5.183223378	3.98219E-08	2.034569337
KBTBD11	-0.948523472	0.174624816	1.071443523	0.00792838	2.019966995
KRT18P63	-3.313029082	0.049947387	-1.294722671	1	2.018306411
LINC02392	4.181212077	0.022626066	6.1941042	8.24737E-05	2.012892122
ADD2	0.978611101	0.154527309	2.987839994	0.00381357	2.009228893
MEOX1	4.444867378	4.13659E-08	6.452881268	3.43543E-16	2.00801389
AC010524.1	0.245084675	1	2.25144637	0.045399678	2.006361695
AC108673.2	1.053226506	0.366430596	3.054276152	0.010143353	2.001049646
AC016590.3	-2.802614243	0.016567086	-0.808637361	1	1.993976883
BANF1P1	1.107813711	0.250047119	3.101650972	0.018563449	1.993837262

SLC12A1	2.539106741	0.09565917	4.52387191	0.001124535	1.984765169
RP11-87H9.3	-2.322856648	0.031950678	-0.343236369	1	1.979620279
AL590648.3	3.904897541	0.000325417	5.855178729	0.000243897	1.950281188
DNER	4.97173059	4.93772E-06	6.918612749	9.27863E-09	1.946882159
FTLP15	1.584398643	0.243552879	3.528286056	0.011827968	1.943887412
DUSP8	-3.152274691	0.026663093	-1.211445333	0.680111958	1.940829358
FBXL22	-3.31041336	0.001319133	-1.391585025	0.254575447	1.918828335
AL136382.1	1.041987787	0.384762448	2.957777299	0.021729368	1.915789512
RHBDL3	2.251145593	0.121115516	4.165611742	0.016781181	1.914466149
RRAD	1.165906123	0.0076278	3.071282028	6.73057E-05	1.905375906
LGI4	3.245380334	1.27138E-06	5.147266019	4.66438E-11	1.901885685
AC103923.1	0.559296265	0.660022364	2.458329387	0.02922274	1.899033121
SHISA9	-1.525140211	0.007360317	0.37210946	0.808923183	1.897249671
RNU6-26P	-1.920829768	0.003707789	-0.046891208	0.981866698	1.87393856
RBPMS2	-2.500139709	0.001220924	-0.638953002	0.125302055	1.861186706
TRHDE-AS1	-2.072305913	0.036135509	-0.212708238	0.886374841	1.859597675
LINC00865	-2.28417729	0.049024051	-0.431273709	0.772585337	1.852903582
KIAA1671	3.412496129	2.55275E-05	5.258780808	1.30814E-11	1.846284679
CCL4L1	2.92447228	0.550349772	4.767574633	0.024395822	1.843102353
DCLK3	2.662277858	0.041830414	4.500756825	0.011795673	1.838478968
PTPRB	-2.150399634	0.000204861	-0.321422536	0.723494516	1.828977098
PROX1	2.287598856	0.000307555	4.113537799	2.9222E-05	1.825938943
EXOC3L4	3.762627567	0.000951301	5.582790231	3.41597E-10	1.820162663
XLOC_012442	3.268435978	0.005950534	5.084103947	0.001116261	1.815667969
FZD5	0.677371674	0.440705216	2.485069316	0.000161626	1.807697643
LINC01140	2.125034786	0.000508203	3.912294066	7.59628E-07	1.78725928
TNFSF18	2.884167721	9.78008E-05	4.649865655	1.89904E-07	1.765697934
AC116003.1	-2.797583966	0.024398849	-1.036421058	1	1.761162908
CREB5	-1.910495296	7.60617E-05	-0.157117002	0.825098071	1.753378294
AL592429.1	4.193251277	1.50142E-05	5.942358598	1.30508E-06	1.749107321
AC004917.1	0.126927143	0.934666646	1.853721765	0.03133383	1.726794622
BAK1P1	0.857877455	0.440366805	2.579660637	0.00247165	1.721783182

CCNA1	3.800746508	0.045012799	5.519902174	0.032810742	1.719155665
LIF	3.970241249	7.65441E-05	5.689335524	4.88457E-09	1.719094275
SLC14A1	-1.901673581	0.002775307	-0.204695363	0.820577052	1.696978218
MAP3K12	-1.782102324	0.003760098	-0.094654991	0.90347525	1.687447334
LEP	-2.043295732	0.005834531	-0.358336652	0.595275724	1.684959081
AC005703.6	1.147290122	0.122291806	2.829792127	0.000875643	1.682502005
AC006160.1	1.6383527	0.252671849	3.318347675	0.021182644	1.679994975
AC073548.1	1.745540134	0.043880999	3.421787159	0.013805658	1.676247025
NEFM	-1.632782129	0.002026261	0.037192549	0.967301166	1.669974678
AL035071.2	-2.52411758	0.045693778	-0.858364309	1	1.665753271
SAA2	5.590723123	1.50744E-35	7.254101745	2.39038E-52	1.663378621
TFAP2A-AS2	-1.098761806	0.008837646	0.53529416	0.610431071	1.634055966
SLAMF8	6.273937072	1.78641E-34	7.907255398	6.96337E-25	1.633318326
RUNDC3B	-1.886371677	0.03652963	-0.270134437	0.811567111	1.61623724
LTB	1.724531409	0.227630061	3.331628834	0.002112713	1.607097425
AP000924.1	2.967024578	0.392177266	4.571802058	0.009455051	1.604777481
NEFL	-1.49438483	0.008785537	0.107503916	0.833539586	1.601888746
SAA1	3.119352879	2.13483E-18	4.717681857	7.41678E-20	1.598328978
CARD10	-2.315305988	0.006948581	-0.717045136	0.33937623	1.598260852
CAMK2B	-2.148968425	0.021295641	-0.554315559	0.693079249	1.594652866
NPL	0.387394824	0.288144599	1.975071357	0.013191554	1.587676533
PPP1R2P1	2.318728707	0.035214813	3.856745795	0.009732336	1.538017087
SP140	3.307113723	0.000304389	4.843436234	0.004676363	1.536322512
AL021707.7	2.726891828	0.001218721	4.259736599	0.000125358	1.532844771
PAX5	5.526369263	9.53396E-14	7.058639285	1.47911E-12	1.532270022
PCSK9	-2.382464671	0.002671259	-0.855653161	0.559264521	1.52681151
AC011498.7	-1.977830203	0.017884358	-0.468807045	1	1.509023159
ZNF205	-1.885692588	0.012037413	-0.379888348	0.846000048	1.505804241

	skin_biopsy_nonlesional.lo	sy_nonlesion	psy_nonlesio	_lesional.log2	opsy_lesional	skin_biopsy_lesional.padj
**XLOC_010133**	NA	NA	NA	-20.15	3.08E-10	7.55E-06
**IGKJ1**	0.48	8.19E-01	1.00E+00	5.23	3.74E-08	3.19E-04
**CYP1A2**	-0.62	5.36E-01	1.00E+00	-7.15	3.91E-08	3.19E-04
**IGKC**	4.31	4.14E-11	2.61E-08	5.03	5.67E-08	3.47E-04
**CHRDL2**	-4.31	1.63E-02	8.04E-01	4.80	1.25E-07	6.15E-04
**IGLV3-25**	5.01	1.31E-01	1.00E+00	6.44	1.69E-07	6.50E-04
**LINC00668**	-1.37	3.89E-01	1.00E+00	-5.33	1.86E-07	6.50E-04
**IGHV4-4**	1.74	6.05E-01	1.00E+00	7.00	2.18E-07	6.50E-04
**IGHV4-34**	-2.12	4.29E-01	1.00E+00	6.22	2.39E-07	6.50E-04
**IGHA2**	8.73	1.29E-26	3.92E-22	4.71	2.66E-07	6.51E-04
**MMP3**	-1.56	1.61E-01	1.00E+00	5.20	3.00E-07	6.67E-04
**G30725**	-0.14	8.94E-01	1.00E+00	-5.66	3.92E-07	8.00E-04
**SFRP2**	0.74	2.19E-01	1.00E+00	2.26	1.18E-06	2.23E-03
**IGLV2-23**	4.30	5.69E-02	1.00E+00	5.99	1.53E-06	2.68E-03
**IGHA1**	4.85	4.91E-10	2.80E-07	4.33	1.69E-06	2.76E-03
**CCL18**	0.82	3.70E-01	1.00E+00	3.49	1.88E-06	2.88E-03
**CACNA1G-AS1**	-0.45	6.94E-01	1.00E+00	4.91	3.35E-06	4.84E-03
**HMGA2**	-0.43	7.90E-01	1.00E+00	4.09	4.09E-06	5.57E-03
**PLS3-AS1**	1.29	6.05E-02	1.00E+00	-3.53	5.12E-06	6.55E-03
**HIST1H1E**	-0.61	3.17E-01	1.00E+00	-3.05	5.34E-06	6.55E-03
**IGHG2**	4.13	1.17E-02	6.70E-01	5.27	5.76E-06	6.72E-03
**SNORD3B-2**	-1.05	3.30E-01	1.00E+00	-4.31	6.14E-06	6.84E-03
**IGHV3-23**	3.51	1.10E-02	6.46E-01	4.67	7.62E-06	8.11E-03
**RNASEH2A**	-2.38	2.23E-01	1.00E+00	-3.92	8.19E-06	8.36E-03
**IGLV1-51**	2.68	9.79E-03	6.13E-01	4.17	9.59E-06	9.22E-03
**HTR3A**	0.31	7.78E-01	1.00E+00	4.49	1.00E-05	9.22E-03
**ARL14**	-4.52	9.91E-02	1.00E+00	4.64	1.02E-05	9.22E-03
**GLMP**	0.12	4.76E-01	1.00E+00	0.90	1.62E-05	1.38E-02
**C1QTNF6**	-0.77	2.75E-01	1.00E+00	2.71	1.63E-05	1.38E-02
**RAP2B**	-0.23	3.68E-01	1.00E+00	0.85	1.78E-05	1.45E-02
**RPS19P7**	0.66	8.49E-01	1.00E+00	-4.07	1.93E-05	1.52E-02

**GTF2IP1**	-0.10	6.81E-01	1.00E+00	-1.03	2.05E-05	1.57E-02
**CBLN1**	-0.93	5.69E-01	1.00E+00	-4.91	2.43E-05	1.81E-02
**INHBA**	0.11	8.60E-01	1.00E+00	2.13	2.58E-05	1.86E-02
**IGLV2-8**	6.14	1.94E-03	2.39E-01	5.49	3.32E-05	2.32E-02
**RAC1P2**	-0.26	5.43E-01	1.00E+00	-2.18	3.60E-05	2.41E-02
**IGKV3D-20**	3.70	1.05E-02	6.33E-01	5.05	3.69E-05	2.41E-02
**PRSS27**	-0.80	7.81E-02	1.00E+00	2.74	3.74E-05	2.41E-02
**MZB1**	4.49	9.95E-04	1.63E-01	3.61	3.97E-05	2.46E-02
**OOEP**	1.20	3.00E-01	1.00E+00	-4.89	4.02E-05	2.46E-02
**IGKV3-20**	6.32	3.38E-08	1.48E-05	4.57	4.11E-05	2.46E-02
**MTRNR2L8**	-2.71	3.15E-02	1.00E+00	-2.28	4.84E-05	2.82E-02
**COL5A1**	-0.55	3.78E-01	1.00E+00	3.09	5.37E-05	3.06E-02
**FKBP11**	-0.24	4.73E-01	1.00E+00	0.89	6.17E-05	3.44E-02
**IGKV2-30**	5.77	9.10E-04	1.54E-01	4.72	6.53E-05	3.53E-02
**STMN2**	-0.46	4.46E-01	1.00E+00	2.41	6.62E-05	3.53E-02
**LINC02223**	0.51	5.97E-01	1.00E+00	3.38	7.12E-05	3.71E-02
**IGKV3D-15**	4.19	1.88E-03	2.39E-01	5.29	8.12E-05	4.06E-02
**AC006372.2**	-0.77	7.96E-01	1.00E+00	5.12	8.13E-05	4.06E-02
**IGKV1D-39**	0.63	7.94E-01	1.00E+00	4.68	8.32E-05	4.08E-02
**MT-RNR2**	0.09	8.44E-01	1.00E+00	-1.76	8.56E-05	4.11E-02
**MZT2B**	-0.06	8.47E-01	1.00E+00	-1.96	9.38E-05	4.42E-02
**G826**	0.14	9.27E-01	1.00E+00	-2.35	9.88E-05	4.57E-02
**AL078595.1**	NA	NA	NA	-4.86	1.02E-04	4.61E-02
**GUF1**	-0.02	9.11E-01	1.00E+00	-0.61	1.03E-04	4.61E-02
**MMP14**	-0.22	4.21E-01	1.00E+00	3.41	1.06E-04	4.61E-02
**COL1A1**	0.51	6.23E-01	1.00E+00	2.62	1.07E-04	4.61E-02
**LINC01705**	NA	NA	NA	4.67	1.13E-04	4.64E-02
**CD37**	-0.19	6.13E-01	1.00E+00	2.33	1.13E-04	4.64E-02
**PTPN20B**	-0.72	3.90E-01	1.00E+00	-2.22	1.14E-04	4.64E-02
**RPL3L**	-0.49	5.38E-01	1.00E+00	-3.01	1.16E-04	4.64E-02
**SLC16A3**	-0.39	2.76E-01	1.00E+00	3.38	1.19E-04	4.65E-02
**CRNDE**	-0.20	4.69E-01	1.00E+00	-1.39	1.20E-04	4.65E-02

**AL033527.5**	-0.91	4.73E-01	1.00E+00	-4.91	1.24E-04	4.65E-02
**EPGN**	-1.76	6.30E-02	1.00E+00	3.40	1.28E-04	4.65E-02
**PTHLH**	0.02	9.80E-01	1.00E+00	2.35	1.28E-04	4.65E-02
**IGHV4-28**	0.56	8.71E-01	1.00E+00	6.60	1.29E-04	4.65E-02
**IGHJ6**	1.29	7.08E-01	1.00E+00	5.74	1.29E-04	4.65E-02
**COL5A2**	-0.24	6.92E-01	1.00E+00	2.63	1.47E-04	5.07E-02
**CXCL1**	-1.25	1.18E-01	1.00E+00	3.16	1.48E-04	5.07E-02
**EVI2B**	-0.15	7.24E-01	1.00E+00	1.59	1.50E-04	5.07E-02
**MTND2P28**	0.33	6.21E-01	1.00E+00	-2.76	1.52E-04	5.07E-02
**MDK**	0.11	8.37E-01	1.00E+00	1.71	1.52E-04	5.07E-02
**AC091488.1**	1.73	4.46E-01	1.00E+00	-5.33	1.53E-04	5.07E-02
**CCL4L1**	-3.00	3.02E-01	1.00E+00	5.17	1.58E-04	5.17E-02
**SIGLEC10**	-0.54	3.43E-01	1.00E+00	2.95	1.71E-04	5.38E-02
**LGALS2**	0.48	3.49E-01	1.00E+00	2.27	1.72E-04	5.38E-02
**SLC7A8**	-0.01	9.75E-01	1.00E+00	1.16	1.74E-04	5.38E-02
**MTRNR2L10**	2.28	5.03E-01	1.00E+00	-4.26	1.77E-04	5.38E-02
**NCF1C**	-0.19	8.12E-01	1.00E+00	3.48	1.79E-04	5.38E-02
**XLOC_010219**	-1.39	6.89E-01	1.00E+00	-3.35	1.82E-04	5.38E-02
**PPP1R14D**	-1.29	3.72E-01	1.00E+00	-3.87	1.82E-04	5.38E-02
**RETN**	-2.24	2.01E-01	1.00E+00	3.99	1.83E-04	5.38E-02
**ISG15**	-0.14	8.99E-01	1.00E+00	2.77	1.85E-04	5.38E-02
**FNDC1**	-0.84	5.66E-02	1.00E+00	2.76	1.89E-04	5.40E-02
**NDP**	-0.66	4.42E-01	1.00E+00	2.48	1.90E-04	5.40E-02
**XLOC_000595**	-0.91	3.06E-01	1.00E+00	-4.05	1.95E-04	5.49E-02
**FKBP1B**	-0.42	5.66E-01	1.00E+00	-2.71	2.03E-04	5.61E-02
**TREM1**	0.14	8.73E-01	1.00E+00	3.70	2.04E-04	5.61E-02
**TNFRSF12A**	-0.76	1.05E-01	1.00E+00	1.25	2.08E-04	5.66E-02
**AC006994.2**	-0.06	9.48E-01	1.00E+00	-4.15	2.13E-04	5.66E-02
**FHL2**	-0.04	8.37E-01	1.00E+00	0.66	2.14E-04	5.66E-02
**AC108449.2**	-0.24	6.88E-01	1.00E+00	-2.03	2.17E-04	5.66E-02
**GPR15**	2.52	2.90E-01	1.00E+00	5.26	2.17E-04	5.66E-02
**AC126768.2**	-0.38	6.69E-01	1.00E+00	-1.43	2.23E-04	5.76E-02

**AC007608.3**	-2.58	7.34E-02	1.00E+00	2.44	2.31E-04	5.88E-02
**LCE3A**	0.61	6.92E-01	1.00E+00	4.33	2.35E-04	5.95E-02
**AL132780.4**	-4.16	2.76E-03	2.72E-01	-4.75	2.49E-04	6.23E-02
**AP002748.3**	-0.02	9.58E-01	1.00E+00	-1.44	2.58E-04	6.33E-02
**FCRL5**	2.76	1.21E-01	1.00E+00	5.04	2.59E-04	6.33E-02
**AC093510.1**	0.02	9.90E-01	1.00E+00	-4.10	2.63E-04	6.33E-02
**AL355338.1**	-0.31	3.81E-01	1.00E+00	-1.05	2.63E-04	6.33E-02
**SETD9**	0.09	8.24E-01	1.00E+00	-1.07	2.72E-04	6.46E-02
**AC007038.1**	1.38	5.23E-01	1.00E+00	-3.99	2.81E-04	6.61E-02
**AC009949.1**	-3.06	2.54E-01	1.00E+00	4.56	2.91E-04	6.73E-02
**LCE3E**	-0.80	1.97E-01	1.00E+00	2.72	2.91E-04	6.73E-02
**KRT16**	-1.18	4.34E-03	3.73E-01	3.36	2.95E-04	6.75E-02
**AC011451.3**	0.03	9.91E-01	1.00E+00	-5.08	2.98E-04	6.75E-02
**IGLC2**	5.35	3.39E-13	2.50E-10	3.97	3.06E-04	6.88E-02
**AC008691.1**	0.37	7.97E-01	1.00E+00	3.85	3.17E-04	7.06E-02
**ABHD6**	0.28	3.85E-01	1.00E+00	-1.13	3.28E-04	7.21E-02
**IGKV3D-11**	4.01	1.82E-01	1.00E+00	5.05	3.30E-04	7.21E-02
**MTRNR2L1**	-1.45	6.71E-01	1.00E+00	-3.38	3.44E-04	7.44E-02
**TMEM271**	-0.66	1.69E-01	1.00E+00	-3.38	3.51E-04	7.44E-02
**PM20D2**	0.05	8.85E-01	1.00E+00	-0.99	3.53E-04	7.44E-02
**ZNF469**	-0.66	1.97E-01	1.00E+00	3.36	3.55E-04	7.44E-02
**COLQ**	0.38	7.51E-01	1.00E+00	3.27	3.55E-04	7.44E-02
**XLOC_007264**	2.08	2.48E-01	1.00E+00	-2.43	3.58E-04	7.44E-02
**AC040174.1**	0.05	9.36E-01	1.00E+00	-3.01	3.66E-04	7.54E-02
**FKBP10**	0.22	6.20E-01	1.00E+00	2.50	3.76E-04	7.68E-02
**NCF1**	-0.27	6.29E-01	1.00E+00	3.33	3.82E-04	7.75E-02
**B3GNT7**	-0.84	7.98E-02	1.00E+00	1.56	3.88E-04	7.79E-02
**IGHV1-8**	1.75	4.44E-01	1.00E+00	5.23	3.95E-04	7.86E-02
**PKIA**	0.13	7.57E-01	1.00E+00	-2.34	4.00E-04	7.90E-02
**RCN3**	0.13	7.93E-01	1.00E+00	1.57	4.13E-04	8.01E-02
**IFIT1**	0.01	9.95E-01	1.00E+00	2.28	4.15E-04	8.01E-02
**XLOC_004545**	1.89	1.14E-01	1.00E+00	-7.22	4.15E-04	8.01E-02

**CCDC121**	0.53	1.63E-01	1.00E+00	-1.36	4.31E-04	8.25E-02
**IGFL1**	-1.86	4.76E-02	1.00E+00	3.40	4.43E-04	8.42E-02
**XLOC_002675**	0.66	5.08E-01	1.00E+00	2.47	4.48E-04	8.44E-02
**ANKRD30BL**	-1.19	5.23E-01	1.00E+00	-3.03	4.56E-04	8.53E-02
**STAP1**	1.63	6.90E-02	1.00E+00	2.59	4.65E-04	8.58E-02
**NDUFA7**	0.37	7.69E-01	1.00E+00	-1.95	4.66E-04	8.58E-02
**AP002986.1**	0.90	5.16E-01	1.00E+00	-2.34	4.70E-04	8.58E-02
**AL359182.1**	0.15	8.75E-01	1.00E+00	2.12	4.73E-04	8.58E-02
**AP001043.1**	0.22	7.91E-01	1.00E+00	1.88	4.76E-04	8.58E-02
**ECM1**	0.25	3.88E-01	1.00E+00	0.93	4.84E-04	8.66E-02
**FAM20C**	-0.45	7.60E-02	1.00E+00	2.35	4.90E-04	8.71E-02
**AC112491.1**	NA	NA	NA	-3.26	5.03E-04	8.87E-02
**AC138207.8**	-0.34	3.52E-01	1.00E+00	-1.16	5.11E-04	8.95E-02
**LYZ**	-0.44	3.06E-01	1.00E+00	3.13	5.19E-04	9.01E-02
**DERL3**	0.07	9.50E-01	1.00E+00	2.79	5.23E-04	9.03E-02
**CFAP65**	-3.02	2.76E-02	9.71E-01	-3.87	5.28E-04	9.05E-02
**GALNT5**	-0.44	4.30E-01	1.00E+00	2.30	5.36E-04	9.11E-02
**AC007952.4**	-0.27	8.30E-01	1.00E+00	-2.57	5.58E-04	9.36E-02
**FAM89B**	-0.45	6.28E-01	1.00E+00	-2.06	5.60E-04	9.36E-02
**AC004130.1**	0.42	4.42E-01	1.00E+00	-1.88	5.62E-04	9.36E-02
**CD14**	-0.15	7.38E-01	1.00E+00	1.92	5.79E-04	9.53E-02
**MIR3936HG**	0.32	6.95E-01	1.00E+00	-1.88	5.81E-04	9.53E-02
**AC026356.2**	0.48	6.96E-01	1.00E+00	4.26	5.83E-04	9.53E-02
**MTCO1P40**	-0.86	5.66E-01	1.00E+00	2.66	5.88E-04	9.54E-02
**CAMP**	1.85	1.25E-01	1.00E+00	3.54	6.01E-04	9.68E-02
**TTC3P1**	-0.40	6.15E-02	1.00E+00	-1.08	6.04E-04	9.68E-02
**AC010198.1**	-3.72	3.82E-02	1.00E+00	-4.34	6.13E-04	9.76E-02
**PLAUR**	-0.57	6.84E-02	1.00E+00	1.74	6.23E-04	9.82E-02
**IGKV2-28**	4.39	6.75E-04	1.24E-01	3.99	6.28E-04	9.82E-02
**JCHAIN**	4.73	1.58E-10	9.19E-08	2.66	6.29E-04	9.82E-02
**SNORA12**	0.56	8.71E-01	1.00E+00	-2.75	6.33E-04	9.82E-02
**HHIP-AS1**	-3.05	2.08E-02	8.94E-01	-2.85	6.43E-04	9.91E-02

**ADAM12**	1.63	1.01E-01	1.00E+00	2.90	6.50E-04	9.96E-02
**SEMA6B**	0.04	9.32E-01	1.00E+00	3.37	6.58E-04	1.00E-01
**AGTRAP**	0.13	7.36E-01	1.00E+00	0.86	6.95E-04	1.05E-01
**HMGN1P36**	-0.41	6.18E-01	1.00E+00	-1.43	7.03E-04	1.05E-01
**CEMIP**	-0.57	2.89E-01	1.00E+00	2.74	7.06E-04	1.05E-01
**RP11-292F22.7**	-2.21	9.74E-02	1.00E+00	-4.40	7.14E-04	1.06E-01
**IGKV1-27**	-2.80	2.81E-01	1.00E+00	4.85	7.16E-04	1.06E-01
**C10orf99**	-0.69	2.84E-01	1.00E+00	3.01	7.30E-04	1.07E-01
**AC012146.1**	-0.63	5.48E-01	1.00E+00	-2.16	7.66E-04	1.12E-01
**ARMC10P1**	0.12	8.63E-01	1.00E+00	-2.43	7.72E-04	1.12E-01
**LILRB4**	0.12	8.24E-01	1.00E+00	3.41	7.96E-04	1.15E-01
**LINC01050**	NA	NA	NA	5.83	8.08E-04	1.16E-01
**AP001362.2**	-0.35	5.54E-01	1.00E+00	-1.41	8.40E-04	1.20E-01
**PDPN**	-0.16	6.58E-01	1.00E+00	1.37	8.52E-04	1.21E-01
**TRDN-AS1**	2.23	2.38E-01	1.00E+00	-4.89	8.69E-04	1.22E-01
**MMP13**	0.61	5.65E-01	1.00E+00	3.85	8.77E-04	1.23E-01
**KRT6B**	-0.08	8.83E-01	1.00E+00	3.03	8.82E-04	1.23E-01
**AL020996.1**	-0.31	6.64E-01	1.00E+00	-1.89	9.20E-04	1.26E-01
**SAMD9**	-0.50	5.05E-01	1.00E+00	1.84	9.21E-04	1.26E-01
**VAV1**	0.02	9.74E-01	1.00E+00	3.08	9.22E-04	1.26E-01
**CDK8P2**	-3.65	1.79E-02	8.39E-01	-4.34	9.23E-04	1.26E-01
**NCF1B**	0.24	7.27E-01	1.00E+00	2.73	9.28E-04	1.26E-01
**PPT2-EGFL8**	1.27	7.13E-01	1.00E+00	-4.64	9.64E-04	1.30E-01
**HIST1H4E**	-0.15	8.00E-01	1.00E+00	-2.66	9.90E-04	1.32E-01
**HEBP2**	0.12	6.25E-01	1.00E+00	-1.94	9.91E-04	1.32E-01
**GBP3**	-0.01	9.84E-01	1.00E+00	1.41	1.00E-03	1.32E-01
**AC022784.1**	0.28	5.42E-01	1.00E+00	-1.90	1.00E-03	1.32E-01
**SH3PXD2B**	-0.25	4.61E-01	1.00E+00	1.85	1.01E-03	1.32E-01
**LINC02030**	-0.85	5.83E-01	1.00E+00	-4.57	1.02E-03	1.33E-01
**CKB**	-0.49	6.27E-04	1.18E-01	-0.84	1.02E-03	1.33E-01
**ZNF66**	0.04	9.37E-01	1.00E+00	-1.45	1.04E-03	1.34E-01
**XLOC_013542**	-1.20	5.69E-01	1.00E+00	4.05	1.05E-03	1.35E-01

**IGHV1-18**	2.63	1.22E-01	1.00E+00	4.68	1.06E-03	1.35E-01
**AK4**	0.10	6.94E-01	1.00E+00	-0.52	1.06E-03	1.35E-01
**TRIM25**	0.68	7.33E-02	1.00E+00	1.24	1.07E-03	1.35E-01
**IGHV3-33**	2.96	1.86E-02	8.56E-01	4.90	1.08E-03	1.36E-01
**AL139289.2**	-0.09	8.39E-01	1.00E+00	-1.78	1.12E-03	1.40E-01
**ACP5**	0.01	9.69E-01	1.00E+00	1.44	1.14E-03	1.42E-01
**IL6R**	0.36	3.16E-01	1.00E+00	1.11	1.18E-03	1.45E-01
**CCL3L3**	0.25	9.42E-01	1.00E+00	4.19	1.18E-03	1.45E-01
**LINC01579**	0.63	5.14E-01	1.00E+00	-2.69	1.18E-03	1.45E-01
**OASL**	-0.39	7.56E-01	1.00E+00	2.76	1.19E-03	1.45E-01
**OSCAR**	-0.21	5.15E-01	1.00E+00	2.23	1.21E-03	1.46E-01
**ANAPC4**	0.16	4.95E-01	1.00E+00	-0.87	1.22E-03	1.47E-01
**AL445205.1**	-2.20	9.25E-02	1.00E+00	3.14	1.22E-03	1.47E-01
**LINC01186**	-1.29	6.84E-01	1.00E+00	-2.41	1.26E-03	1.51E-01
**SLC14A1**	0.46	7.05E-01	1.00E+00	-2.17	1.29E-03	1.52E-01
**SERPINA1**	-0.48	3.23E-01	1.00E+00	2.69	1.29E-03	1.52E-01
**GRID1**	0.05	8.96E-01	1.00E+00	1.88	1.30E-03	1.53E-01
**IFIT2**	-1.10	3.24E-01	1.00E+00	1.92	1.31E-03	1.53E-01
**RNU2-59P**	0.53	7.32E-01	1.00E+00	-2.62	1.32E-03	1.53E-01
**MSR1**	0.08	8.45E-01	1.00E+00	2.37	1.33E-03	1.53E-01
**APLN**	0.60	3.41E-01	1.00E+00	2.60	1.33E-03	1.53E-01
**NOG**	0.07	8.78E-01	1.00E+00	2.04	1.34E-03	1.53E-01
**AL121601.1**	0.13	9.70E-01	1.00E+00	-3.05	1.34E-03	1.53E-01
**IGHV3-74**	6.41	6.21E-11	3.76E-08	3.39	1.35E-03	1.53E-01
**FAM133CP**	0.25	8.12E-01	1.00E+00	1.77	1.35E-03	1.53E-01
**MFSD2B**	1.78	2.54E-01	1.00E+00	2.16	1.35E-03	1.53E-01
**IGHV3-15**	4.33	3.33E-04	7.10E-02	3.94	1.37E-03	1.54E-01
**IGLV3-19**	8.44	3.08E-04	6.69E-02	6.11	1.38E-03	1.54E-01
**C11orf87**	-6.05	1.07E-04	2.56E-02	3.69	1.39E-03	1.54E-01
**AGXT**	1.57	3.69E-01	1.00E+00	-4.58	1.44E-03	1.59E-01
**IGHV4-31**	1.20	6.55E-01	1.00E+00	4.58	1.44E-03	1.59E-01
**SLC11A1**	0.39	3.51E-01	1.00E+00	2.14	1.46E-03	1.60E-01

**MFAP2**	0.09	8.30E-01	1.00E+00	1.16	1.46E-03	1.60E-01
**CD79A**	1.17	8.54E-02	1.00E+00	3.23	1.47E-03	1.60E-01
**PXMP2**	-0.12	8.94E-01	1.00E+00	-1.94	1.49E-03	1.61E-01
**FGFR2**	0.08	7.23E-01	1.00E+00	-1.26	1.50E-03	1.62E-01
**AL365361.1**	-2.22	9.73E-02	1.00E+00	3.75	1.51E-03	1.62E-01
**IGSF6**	-0.86	1.20E-01	1.00E+00	1.57	1.52E-03	1.63E-01
**IGKV1-39**	4.05	1.18E-03	1.81E-01	4.32	1.53E-03	1.63E-01
**IGLL5**	3.96	7.94E-02	1.00E+00	4.53	1.53E-03	1.63E-01
**PAPPA**	0.11	8.03E-01	1.00E+00	2.21	1.54E-03	1.63E-01
**MSH3**	0.15	4.44E-01	1.00E+00	-0.44	1.57E-03	1.65E-01
**KCNC3**	-0.61	1.81E-01	1.00E+00	2.82	1.57E-03	1.65E-01
**MMP11**	-0.65	2.72E-01	1.00E+00	2.75	1.62E-03	1.69E-01
**CPXM1**	-1.64	2.99E-03	2.87E-01	2.55	1.63E-03	1.69E-01
**SPANXA2-OT1**	2.93	1.09E-01	1.00E+00	-4.50	1.64E-03	1.70E-01
**RFX8**	-1.37	3.84E-01	1.00E+00	3.24	1.65E-03	1.70E-01
**CD48**	-0.32	6.26E-01	1.00E+00	2.70	1.68E-03	1.72E-01
**ATOH7**	-1.61	4.51E-01	1.00E+00	-3.93	1.70E-03	1.74E-01
**AC100771.2**	1.56	7.65E-02	1.00E+00	-3.05	1.73E-03	1.76E-01
**SPINK9**	-2.18	3.84E-01	1.00E+00	3.50	1.74E-03	1.76E-01
**S100A8**	-0.95	1.90E-01	1.00E+00	2.79	1.74E-03	1.76E-01
**CTSA**	-0.09	6.60E-01	1.00E+00	0.37	1.77E-03	1.76E-01
**AP000580.1**	1.85	4.48E-01	1.00E+00	-2.70	1.78E-03	1.76E-01
**G5850**	-0.09	9.62E-01	1.00E+00	-4.98	1.78E-03	1.76E-01
**IFNG-AS1**	-1.63	6.25E-01	1.00E+00	4.25	1.80E-03	1.76E-01
**PCCA**	0.09	7.36E-01	1.00E+00	-0.73	1.81E-03	1.76E-01
**NLGN4Y**	-0.86	2.46E-01	1.00E+00	3.96	1.82E-03	1.76E-01
**CERNA2**	-1.96	2.04E-01	1.00E+00	4.40	1.83E-03	1.76E-01
**GREM1**	0.06	9.14E-01	1.00E+00	2.00	1.83E-03	1.76E-01
**THBS1**	0.21	7.17E-01	1.00E+00	1.61	1.84E-03	1.76E-01
**ALPL**	0.26	6.64E-01	1.00E+00	2.27	1.84E-03	1.76E-01
**FHAD1**	0.50	4.55E-01	1.00E+00	2.79	1.85E-03	1.76E-01
**B4GALT1-AS1**	0.46	3.91E-01	1.00E+00	-1.76	1.85E-03	1.76E-01

**IGLV6-57**	1.72	2.68E-01	1.00E+00	4.11	1.86E-03	1.76E-01
**CP**	0.54	2.99E-01	1.00E+00	1.98	1.86E-03	1.76E-01
**G10215**	2.28	3.75E-01	1.00E+00	-3.12	1.87E-03	1.76E-01
**AC097534.1**	-0.94	3.70E-01	1.00E+00	-2.38	1.88E-03	1.76E-01
**RXYLT1**	-0.27	1.89E-01	1.00E+00	-0.80	1.88E-03	1.76E-01
**PLPP4**	-1.17	2.12E-01	1.00E+00	1.65	1.89E-03	1.76E-01
**XLOC_002813**	-1.09	6.15E-01	1.00E+00	4.45	1.89E-03	1.76E-01
**DPYSL4**	-1.28	1.84E-02	8.51E-01	3.01	1.89E-03	1.76E-01
**XLOC_002925**	-2.26	6.71E-02	1.00E+00	-2.29	1.92E-03	1.77E-01
**LINC00273**	-0.80	8.18E-01	1.00E+00	-3.10	1.92E-03	1.77E-01
**IGHV3-48**	2.20	5.15E-01	1.00E+00	3.82	1.93E-03	1.77E-01
**NELL2**	-0.14	8.24E-01	1.00E+00	1.68	1.93E-03	1.77E-01
**SLC13A5**	-0.61	7.33E-01	1.00E+00	3.12	1.94E-03	1.77E-01
**NLGN2**	-0.30	4.23E-01	1.00E+00	2.49	1.95E-03	1.77E-01
**PAK5**	-0.32	6.02E-01	1.00E+00	-2.81	1.95E-03	1.77E-01
**RNU2-61P**	NA	NA	NA	-3.17	1.96E-03	1.77E-01
**C1QC**	0.15	7.72E-01	1.00E+00	2.10	1.98E-03	1.78E-01
**C5orf64**	3.60	5.09E-02	1.00E+00	-4.27	1.99E-03	1.79E-01
**AC131392.1**	0.79	4.85E-01	1.00E+00	-2.07	2.00E-03	1.79E-01
**IGHV3-20**	1.52	6.57E-01	1.00E+00	6.05	2.01E-03	1.79E-01
**RENBP**	0.16	6.45E-01	1.00E+00	2.55	2.01E-03	1.79E-01
**AL160006.1**	1.04	5.30E-02	1.00E+00	1.07	2.04E-03	1.80E-01
**LINC01094**	-0.50	3.57E-01	1.00E+00	1.11	2.05E-03	1.80E-01
**HMGB1P24**	-0.88	8.01E-01	1.00E+00	-2.33	2.07E-03	1.80E-01
**C19orf66**	-0.20	6.75E-01	1.00E+00	1.10	2.07E-03	1.80E-01
**GPSM3**	-0.23	5.09E-01	1.00E+00	1.80	2.07E-03	1.80E-01
**IGKV1D-33**	6.69	2.98E-02	9.97E-01	4.45	2.08E-03	1.80E-01
**TNFSF13B**	-0.19	7.41E-01	1.00E+00	1.71	2.09E-03	1.80E-01
**POU2F2**	-0.19	6.86E-01	1.00E+00	2.26	2.09E-03	1.80E-01
**AC079313.2**	-0.70	6.26E-01	1.00E+00	-2.49	2.09E-03	1.80E-01
**AC144831.1**	0.55	3.08E-01	1.00E+00	1.53	2.10E-03	1.80E-01
**ABCA1**	0.68	2.13E-02	8.94E-01	1.00	2.12E-03	1.80E-01

**COL27A1**	-0.37	3.64E-01	1.00E+00	2.01	2.12E-03	1.80E-01
**PABPC1P4**	-0.14	7.87E-01	1.00E+00	0.98	2.13E-03	1.80E-01
**AKNA**	0.17	6.13E-01	1.00E+00	2.61	2.16E-03	1.82E-01
**WISP1**	0.44	4.70E-01	1.00E+00	2.30	2.16E-03	1.82E-01
**HAMP**	0.07	9.58E-01	1.00E+00	2.56	2.18E-03	1.83E-01
**SVIP**	0.27	1.82E-01	1.00E+00	-1.32	2.19E-03	1.83E-01
**TSGA10IP**	0.86	3.97E-01	1.00E+00	2.22	2.19E-03	1.83E-01
**PPM1B**	0.23	4.55E-01	1.00E+00	-1.12	2.20E-03	1.83E-01
**ABL2**	-0.05	8.24E-01	1.00E+00	1.15	2.25E-03	1.86E-01
**AL662884.3**	-1.60	6.43E-01	1.00E+00	-2.54	2.27E-03	1.86E-01
**CFB**	-0.66	4.71E-01	1.00E+00	1.69	2.28E-03	1.86E-01
**IGLC3**	4.73	3.28E-11	2.11E-08	3.17	2.28E-03	1.86E-01
**UVRAG-DT**	1.22	5.47E-03	4.37E-01	-0.98	2.29E-03	1.86E-01
**SLC38A5**	-0.11	7.07E-01	1.00E+00	1.06	2.29E-03	1.86E-01
**DMXL2**	0.04	9.26E-01	1.00E+00	1.77	2.29E-03	1.86E-01
**AKR1B15**	0.18	7.92E-01	1.00E+00	2.39	2.32E-03	1.87E-01
**PGBD5**	-0.24	7.11E-01	1.00E+00	2.72	2.33E-03	1.88E-01
**ACTG1P14**	-0.51	8.83E-01	1.00E+00	4.01	2.35E-03	1.88E-01
**EHHADH**	0.32	3.95E-01	1.00E+00	-0.90	2.35E-03	1.88E-01
**SPRR1A**	-0.45	5.30E-01	1.00E+00	2.55	2.36E-03	1.88E-01
**SLC35E2A**	-0.95	5.26E-02	1.00E+00	-0.88	2.37E-03	1.88E-01
**AC112243.1**	-1.23	4.04E-01	1.00E+00	-4.81	2.43E-03	1.92E-01
**EXOSC6**	-0.22	3.03E-01	1.00E+00	-0.66	2.43E-03	1.92E-01
**LGALS9**	0.20	7.14E-01	1.00E+00	1.73	2.47E-03	1.95E-01
**AP001107.9**	-0.81	3.68E-02	1.00E+00	-1.23	2.51E-03	1.97E-01
**TP53I3**	-0.18	6.49E-01	1.00E+00	0.97	2.54E-03	1.98E-01
**B4GALT1**	-0.08	6.93E-01	1.00E+00	1.19	2.54E-03	1.98E-01
**AC009275.1**	-0.87	6.81E-01	1.00E+00	3.21	2.56E-03	1.99E-01
**DDX60L**	0.00	9.96E-01	1.00E+00	1.47	2.58E-03	2.00E-01
**AL512343.2**	6.06	2.48E-02	9.28E-01	-3.20	2.59E-03	2.00E-01
**AC063960.1**	0.11	9.28E-01	1.00E+00	-4.00	2.60E-03	2.00E-01
**MT-TY**	0.83	4.47E-01	1.00E+00	-2.23	2.61E-03	2.01E-01

**BCL7C**	0.15	5.19E-01	1.00E+00	-1.50	2.64E-03	2.02E-01
**TCERG1L**	-0.12	8.97E-01	1.00E+00	-2.94	2.66E-03	2.03E-01
**AC007098.1**	-0.52	5.65E-01	1.00E+00	-1.32	2.68E-03	2.03E-01
**TMPRSS11D**	-3.69	1.11E-02	6.49E-01	4.23	2.68E-03	2.03E-01
**CTPS2**	0.33	2.92E-01	1.00E+00	-0.60	2.71E-03	2.04E-01
**AC110995.1**	0.48	5.04E-01	1.00E+00	1.64	2.71E-03	2.04E-01
**STC2**	-0.30	4.92E-01	1.00E+00	1.90	2.72E-03	2.05E-01
**AL031728.1**	0.00	1.00E+00	1.00E+00	3.11	2.73E-03	2.05E-01
**SH3GL2**	-6.02	8.99E-09	4.25E-06	-4.48	2.77E-03	2.05E-01
**SLAMF8**	-1.22	5.62E-02	1.00E+00	2.16	2.77E-03	2.05E-01
**ZNF295-AS1**	0.08	9.65E-01	1.00E+00	-2.39	2.78E-03	2.05E-01
**AC005034.4**	-0.98	6.95E-01	1.00E+00	-4.24	2.79E-03	2.05E-01
**AC008105.2**	4.45	4.67E-04	9.32E-02	-1.77	2.79E-03	2.05E-01
**IRF7**	-0.37	6.00E-01	1.00E+00	1.72	2.80E-03	2.05E-01
**GPAA1P1**	1.17	7.31E-01	1.00E+00	3.13	2.81E-03	2.05E-01
**LINC01605**	-0.57	1.98E-01	1.00E+00	1.95	2.82E-03	2.05E-01
**CD86**	0.03	9.41E-01	1.00E+00	1.41	2.82E-03	2.05E-01
**BBS10**	0.16	5.44E-01	1.00E+00	-0.73	2.83E-03	2.05E-01
**AC064807.4**	-0.26	8.11E-01	1.00E+00	-3.12	2.83E-03	2.05E-01
**PTCRA**	-0.12	9.73E-01	1.00E+00	3.93	2.85E-03	2.06E-01
**RPL12P14**	-1.48	6.61E-01	1.00E+00	-2.63	2.87E-03	2.07E-01
**ID4**	0.13	6.24E-01	1.00E+00	-1.51	2.90E-03	2.07E-01
**NOC2LP1**	-1.33	5.91E-02	1.00E+00	-1.54	2.91E-03	2.07E-01
**BST2**	-0.48	5.33E-01	1.00E+00	2.02	2.92E-03	2.07E-01
**KLHL6**	0.08	8.82E-01	1.00E+00	2.94	2.92E-03	2.07E-01
**C5AR2**	-0.62	1.75E-01	1.00E+00	2.33	2.93E-03	2.07E-01
**GRN**	-0.25	2.14E-01	1.00E+00	0.90	2.93E-03	2.07E-01
**LCE3D**	-0.23	7.28E-01	1.00E+00	2.30	2.94E-03	2.07E-01
**AL683887.1**	-3.03	2.77E-02	9.71E-01	-1.88	2.95E-03	2.07E-01
**ABHD10**	0.21	3.81E-01	1.00E+00	-1.11	2.95E-03	2.07E-01
**REEP3**	0.16	4.84E-01	1.00E+00	-0.64	2.96E-03	2.07E-01
**FYB1**	-0.12	7.86E-01	1.00E+00	1.67	2.98E-03	2.08E-01

**FPR3**	-0.04	9.42E-01	1.00E+00	2.01	2.98E-03	2.08E-01
**PKD1P5**	0.07	9.29E-01	1.00E+00	3.21	2.99E-03	2.08E-01
**SPRR1B**	-0.96	1.93E-01	1.00E+00	2.40	3.00E-03	2.08E-01
**COL12A1**	0.47	4.01E-01	1.00E+00	1.71	3.03E-03	2.09E-01
**FBXL3**	0.30	2.66E-01	1.00E+00	-1.00	3.13E-03	2.15E-01
**IRF9**	0.59	6.92E-01	1.00E+00	1.74	3.17E-03	2.18E-01
**NFAM1**	0.01	9.86E-01	1.00E+00	2.29	3.20E-03	2.19E-01
**HIST1H1B**	-1.71	1.81E-01	1.00E+00	-3.11	3.22E-03	2.19E-01
**PLA2R1**	-0.24	6.31E-01	1.00E+00	-1.06	3.22E-03	2.19E-01
**BMF**	-0.07	8.05E-01	1.00E+00	2.05	3.23E-03	2.19E-01
**SDS**	-1.05	2.34E-01	1.00E+00	2.67	3.23E-03	2.19E-01
**IGKV1D-17**	2.02	5.50E-01	1.00E+00	6.14	3.24E-03	2.19E-01
**SAMM50**	0.13	6.24E-01	1.00E+00	-0.51	3.26E-03	2.19E-01
**NCOA7**	0.00	9.98E-01	1.00E+00	0.96	3.28E-03	2.20E-01
**PDE6G**	-3.30	3.03E-03	2.89E-01	1.68	3.29E-03	2.20E-01
**SHISAL1**	0.24	6.63E-01	1.00E+00	2.42	3.32E-03	2.22E-01
**IGKV4-1**	6.71	2.34E-02	9.15E-01	3.51	3.33E-03	2.22E-01
**SCNN1D**	-1.20	6.82E-02	1.00E+00	3.41	3.37E-03	2.24E-01
**RPS15AP17**	4.56	1.74E-01	1.00E+00	-2.54	3.38E-03	2.24E-01
**CLEC2D**	0.02	9.76E-01	1.00E+00	1.50	3.39E-03	2.24E-01
**SIAH1**	0.13	6.06E-01	1.00E+00	-1.08	3.41E-03	2.25E-01
**IGF2BP3**	-0.96	5.18E-01	1.00E+00	2.84	3.44E-03	2.26E-01
**ALDH2**	0.50	7.10E-02	1.00E+00	-0.59	3.45E-03	2.26E-01
**VWFP1**	2.93	1.60E-02	7.91E-01	2.11	3.47E-03	2.26E-01
**LINC02421**	0.26	9.40E-01	1.00E+00	-5.50	3.48E-03	2.26E-01
**RPLP0P2**	-0.84	4.82E-01	1.00E+00	1.60	3.49E-03	2.26E-01
**RPS2P7**	-1.29	2.98E-01	1.00E+00	-1.97	3.51E-03	2.26E-01
**U62317.1**	1.10	7.38E-01	1.00E+00	2.39	3.51E-03	2.26E-01
**HLA-F**	-0.09	8.17E-01	1.00E+00	1.16	3.53E-03	2.26E-01
**RALYL**	-1.26	7.16E-01	1.00E+00	-4.96	3.53E-03	2.26E-01
**CLEC7A**	-1.10	1.68E-01	1.00E+00	1.59	3.54E-03	2.26E-01
**SDHAF4**	0.09	7.50E-01	1.00E+00	-0.67	3.55E-03	2.26E-01

**PQLC2L**	0.84	2.91E-01	1.00E+00	-1.82	3.55E-03	2.26E-01
**AC107956.1**	-1.96	2.85E-01	1.00E+00	-3.28	3.55E-03	2.26E-01
**P3H3**	-0.13	7.36E-01	1.00E+00	1.69	3.56E-03	2.26E-01
**BGN**	-0.21	6.72E-01	1.00E+00	1.44	3.56E-03	2.26E-01
**AL031847.1**	-1.23	7.24E-01	1.00E+00	-4.00	3.58E-03	2.26E-01
**BRSK1**	-1.12	9.64E-02	1.00E+00	2.53	3.60E-03	2.26E-01
**CST3**	0.12	6.94E-01	1.00E+00	0.76	3.61E-03	2.26E-01
**XLOC_005405**	-1.47	6.70E-01	1.00E+00	-3.41	3.61E-03	2.26E-01
**SCGB2B2**	-0.94	3.68E-01	1.00E+00	-1.92	3.61E-03	2.26E-01
**AMDHD2**	-0.18	5.20E-01	1.00E+00	0.83	3.62E-03	2.26E-01
**MUC5B**	-1.88	4.86E-02	1.00E+00	4.08	3.63E-03	2.26E-01
**FNBP1P1**	-0.08	9.18E-01	1.00E+00	-1.84	3.64E-03	2.26E-01
**TMEM86A**	0.24	6.38E-01	1.00E+00	1.14	3.66E-03	2.26E-01
**EIF3C**	-0.44	1.06E-01	1.00E+00	-0.71	3.68E-03	2.27E-01
**LY6E**	0.16	7.75E-01	1.00E+00	1.23	3.68E-03	2.27E-01
**DRD4**	0.28	7.01E-01	1.00E+00	3.50	3.71E-03	2.27E-01
**IGHV1-3**	-0.50	8.54E-01	1.00E+00	4.13	3.72E-03	2.27E-01
**CDH12**	-0.51	3.66E-01	1.00E+00	-1.91	3.72E-03	2.27E-01
**PLAU**	-0.30	5.78E-01	1.00E+00	1.13	3.72E-03	2.27E-01
**IGKV2-29**	3.87	2.51E-01	1.00E+00	6.29	3.75E-03	2.27E-01
**FADS2**	0.88	2.61E-01	1.00E+00	-2.27	3.75E-03	2.27E-01
**POLR3G**	-0.08	8.23E-01	1.00E+00	1.23	3.76E-03	2.27E-01
**MSTN**	-1.83	2.10E-01	1.00E+00	-2.89	3.77E-03	2.27E-01
**COL1A2**	0.83	4.70E-01	1.00E+00	1.77	3.78E-03	2.27E-01
**ACOT4**	0.55	4.90E-01	1.00E+00	-1.27	3.78E-03	2.27E-01
**KCNJ6**	0.22	9.49E-01	1.00E+00	4.13	3.79E-03	2.27E-01
**HES5**	0.66	2.25E-01	1.00E+00	-2.76	3.80E-03	2.27E-01
**KRT16P2**	-3.42	1.06E-02	6.36E-01	2.58	3.80E-03	2.27E-01
**ANKRD1**	-1.96	5.61E-01	1.00E+00	3.43	3.83E-03	2.27E-01
**G40988**	-0.35	4.78E-01	1.00E+00	1.28	3.83E-03	2.27E-01
**IL7R**	0.43	5.10E-01	1.00E+00	2.67	3.85E-03	2.28E-01
**AL031316.1**	0.06	9.85E-01	1.00E+00	-2.55	3.85E-03	2.28E-01

**PTGER2**	0.35	4.14E-01	1.00E+00	1.35	3.91E-03	2.30E-01
**METTL21EP**	0.57	8.33E-01	1.00E+00	-4.04	3.91E-03	2.30E-01
**AC011008.2**	0.04	9.68E-01	1.00E+00	-3.16	3.98E-03	2.32E-01
**ANKLE1**	0.82	5.67E-01	1.00E+00	1.73	3.98E-03	2.32E-01
**PLA2G4C**	0.66	2.13E-01	1.00E+00	1.37	3.98E-03	2.32E-01
**PRSS23**	-0.15	7.18E-01	1.00E+00	1.15	3.99E-03	2.32E-01
**GALNT1**	0.39	2.71E-01	1.00E+00	-1.27	4.04E-03	2.35E-01
**RBM26-AS1**	-1.35	1.59E-01	1.00E+00	-1.67	4.06E-03	2.35E-01
**PRKY**	0.68	3.30E-01	1.00E+00	3.47	4.07E-03	2.35E-01
**QSOX1**	-0.09	7.18E-01	1.00E+00	1.27	4.09E-03	2.35E-01
**SULT1C2**	1.83	2.88E-01	1.00E+00	3.69	4.09E-03	2.35E-01
**SMIM18**	-1.24	7.19E-01	1.00E+00	2.32	4.10E-03	2.35E-01
**BX284668.5**	-0.79	3.03E-01	1.00E+00	-1.59	4.10E-03	2.35E-01
**CRISP3**	-0.04	9.76E-01	1.00E+00	-2.37	4.13E-03	2.36E-01
**TLR2**	0.15	7.18E-01	1.00E+00	1.69	4.16E-03	2.37E-01
**HIST1H2BJ**	-0.54	5.39E-01	1.00E+00	1.54	4.17E-03	2.37E-01
**PCDH17**	-0.41	4.48E-01	1.00E+00	1.94	4.18E-03	2.37E-01
**HAS2**	0.42	2.77E-01	1.00E+00	1.29	4.20E-03	2.38E-01
**FAIM2**	0.25	7.91E-01	1.00E+00	1.41	4.24E-03	2.39E-01
**AL121790.1**	-0.09	9.66E-01	1.00E+00	-3.02	4.24E-03	2.39E-01
**UPP1**	-0.14	7.35E-01	1.00E+00	1.36	4.26E-03	2.39E-01
**IGHV3-64**	NA	NA	NA	5.84	4.26E-03	2.39E-01
**DDTL**	0.29	6.98E-01	1.00E+00	1.46	4.27E-03	2.39E-01
**KCNJ1**	0.35	7.78E-01	1.00E+00	3.13	4.27E-03	2.39E-01
**EEF1A1P4**	-1.04	5.95E-01	1.00E+00	-1.65	4.28E-03	2.39E-01
**THEMIS2**	0.53	2.30E-01	1.00E+00	1.92	4.30E-03	2.39E-01
**AC020928.1**	-2.53	1.81E-01	1.00E+00	-4.58	4.32E-03	2.39E-01
**COL3A1**	1.28	3.83E-01	1.00E+00	1.93	4.32E-03	2.39E-01
**ALOX5**	-0.71	1.77E-01	1.00E+00	1.48	4.34E-03	2.40E-01
**EEF1A1P6**	-0.01	9.75E-01	1.00E+00	-0.59	4.37E-03	2.40E-01
**CERCAM**	0.39	5.58E-01	1.00E+00	1.55	4.38E-03	2.41E-01
**CD33**	-0.19	6.85E-01	1.00E+00	1.49	4.40E-03	2.41E-01

**HERC5**	0.23	6.98E-01	1.00E+00	1.59	4.43E-03	2.42E-01
**NT5C3A**	0.01	9.67E-01	1.00E+00	0.73	4.45E-03	2.42E-01
**PLD4**	-0.37	5.00E-01	1.00E+00	1.93	4.45E-03	2.42E-01
**AC090236.2**	NA	NA	NA	-5.13	4.49E-03	2.42E-01
**XLOC_005595**	-0.37	5.00E-01	1.00E+00	-1.17	4.49E-03	2.42E-01
**GJB2**	-0.41	2.87E-01	1.00E+00	2.07	4.49E-03	2.42E-01
**MUC21**	1.03	6.03E-01	1.00E+00	-5.06	4.53E-03	2.42E-01
**CDH22**	-0.65	6.38E-02	1.00E+00	-2.30	4.54E-03	2.42E-01
**KRT20**	-3.85	2.14E-02	8.94E-01	-4.45	4.54E-03	2.42E-01
**AC138207.5**	0.69	2.52E-01	1.00E+00	2.05	4.54E-03	2.42E-01
**HIST1H1C**	-0.01	9.69E-01	1.00E+00	-0.95	4.55E-03	2.42E-01
**AL109613.1**	0.45	7.18E-01	1.00E+00	2.10	4.55E-03	2.42E-01
**LINC02544**	-3.01	9.13E-02	1.00E+00	2.53	4.56E-03	2.42E-01
**ZNF396**	0.11	7.83E-01	1.00E+00	-1.61	4.56E-03	2.42E-01
**AL583722.1**	0.92	3.58E-01	1.00E+00	3.65	4.58E-03	2.43E-01
**PRB1**	-1.82	1.11E-01	1.00E+00	-2.71	4.59E-03	2.43E-01
**LMAN2L**	0.20	3.97E-01	1.00E+00	-0.80	4.61E-03	2.43E-01
**AC004069.1**	0.68	8.04E-01	1.00E+00	-2.63	4.62E-03	2.43E-01
**AP000925.1**	0.01	9.98E-01	1.00E+00	4.18	4.62E-03	2.43E-01
**RNU1-60P**	0.32	7.53E-01	1.00E+00	-2.07	4.65E-03	2.44E-01
**MIR635**	0.94	2.91E-01	1.00E+00	2.88	4.68E-03	2.45E-01
**TSLP**	1.17	6.85E-02	1.00E+00	1.22	4.69E-03	2.45E-01
**SLC7A7**	0.35	4.27E-01	1.00E+00	1.87	4.70E-03	2.45E-01
**OR1N1**	NA	NA	NA	-4.38	4.72E-03	2.45E-01
**MPG**	-0.20	4.12E-01	1.00E+00	0.59	4.76E-03	2.47E-01
**VMO1**	-0.07	8.56E-01	1.00E+00	1.38	4.80E-03	2.48E-01
**NTN1**	0.35	4.06E-01	1.00E+00	2.49	4.82E-03	2.49E-01
**TPTE2P5**	-0.06	9.66E-01	1.00E+00	-2.49	4.84E-03	2.50E-01
**FNTA**	0.19	2.99E-01	1.00E+00	-1.00	4.86E-03	2.50E-01
**XLOC_000600**	0.68	6.90E-01	1.00E+00	-3.07	4.88E-03	2.50E-01
**AC093908.1**	-0.20	7.55E-01	1.00E+00	1.99	4.88E-03	2.50E-01
**G36296**	-3.52	7.35E-02	1.00E+00	2.06	4.91E-03	2.51E-01

**PBRM1**	-0.05	8.47E-01	1.00E+00	-0.84	4.91E-03	2.51E-01
**LGMN**	0.29	3.79E-01	1.00E+00	1.00	4.92E-03	2.51E-01
**SIRPB2**	-0.01	9.87E-01	1.00E+00	1.91	4.94E-03	2.51E-01
**AC239799.1**	-0.65	4.34E-01	1.00E+00	1.39	4.95E-03	2.51E-01
**ENC1**	-0.45	2.96E-01	1.00E+00	1.41	4.95E-03	2.51E-01
**AC012313.8**	0.09	9.14E-01	1.00E+00	-3.48	5.00E-03	2.53E-01
**MAPKAPK2**	-0.04	8.05E-01	1.00E+00	1.75	5.05E-03	2.54E-01
**AC090517.5**	0.18	7.89E-01	1.00E+00	-0.99	5.06E-03	2.54E-01
**AP003680.1**	-0.06	9.86E-01	1.00E+00	-4.65	5.07E-03	2.54E-01
**AL132780.2**	0.22	6.77E-01	1.00E+00	-1.33	5.08E-03	2.54E-01
**FRZB**	-0.54	3.37E-01	1.00E+00	-1.10	5.08E-03	2.54E-01
**ZHX1**	-0.02	9.17E-01	1.00E+00	-0.45	5.12E-03	2.55E-01
**WAS**	-0.70	1.31E-01	1.00E+00	1.97	5.20E-03	2.58E-01
**TIMP1**	0.09	8.33E-01	1.00E+00	1.02	5.20E-03	2.58E-01
**MT1G**	-0.46	2.79E-01	1.00E+00	-1.52	5.21E-03	2.58E-01
**TRPM2**	0.23	7.01E-01	1.00E+00	2.57	5.21E-03	2.58E-01
**LINC00996**	-0.61	7.71E-01	1.00E+00	3.97	5.23E-03	2.58E-01
**AC107032.2**	-0.41	6.53E-01	1.00E+00	-1.58	5.28E-03	2.60E-01
**AC104237.2**	2.62	1.19E-01	1.00E+00	-4.29	5.30E-03	2.61E-01
**TRIM52-AS1**	-0.29	3.27E-01	1.00E+00	-0.93	5.34E-03	2.62E-01
**G42895**	-0.13	8.83E-01	1.00E+00	-2.93	5.35E-03	2.62E-01
**TTC33**	0.32	3.32E-01	1.00E+00	-1.02	5.42E-03	2.65E-01
**TOMM20L**	-0.28	7.78E-01	1.00E+00	-2.12	5.43E-03	2.65E-01
**PTAFR**	-0.34	3.13E-01	1.00E+00	1.76	5.47E-03	2.66E-01
**AC138474.1**	-0.92	5.77E-01	1.00E+00	-3.28	5.53E-03	2.69E-01
**MTATP8P1**	-1.26	7.13E-01	1.00E+00	-2.63	5.54E-03	2.69E-01
**KATNAL2**	-0.23	6.64E-01	1.00E+00	-1.68	5.56E-03	2.69E-01
**Z99129.3**	-0.80	8.18E-01	1.00E+00	-4.06	5.60E-03	2.70E-01
**AC004233.2**	0.56	6.74E-01	1.00E+00	-3.75	5.60E-03	2.70E-01
**HTR2A**	-0.56	5.27E-01	1.00E+00	1.81	5.62E-03	2.70E-01
**SPCS2P4**	-0.35	4.27E-01	1.00E+00	-1.16	5.62E-03	2.70E-01
**SPATA31D5P**	-0.05	9.88E-01	1.00E+00	-2.11	5.65E-03	2.70E-01

**SYNGAP1**	-0.63	4.73E-02	1.00E+00	2.34	5.67E-03	2.70E-01
**LINC02156**	0.56	8.71E-01	1.00E+00	4.02	5.67E-03	2.70E-01
**OLFML3**	0.61	2.24E-01	1.00E+00	1.24	5.68E-03	2.70E-01
**IFITM1**	0.24	6.41E-01	1.00E+00	1.21	5.69E-03	2.70E-01
**AC046158.1**	1.13	7.44E-01	1.00E+00	-4.09	5.70E-03	2.70E-01
**SLC2A9**	-0.36	2.73E-01	1.00E+00	0.89	5.70E-03	2.70E-01
**LCP2**	0.26	5.72E-01	1.00E+00	1.58	5.73E-03	2.70E-01
**MXD1**	0.05	8.95E-01	1.00E+00	0.87	5.73E-03	2.70E-01
**AC008972.2**	-0.05	9.66E-01	1.00E+00	1.99	5.73E-03	2.70E-01
**ISG20**	-0.49	4.00E-01	1.00E+00	1.64	5.75E-03	2.71E-01
**LINC01152**	0.56	5.69E-01	1.00E+00	-2.24	5.77E-03	2.71E-01
**GK-IT1**	0.56	8.71E-01	1.00E+00	4.41	5.81E-03	2.72E-01
**SULF1**	-0.28	5.74E-01	1.00E+00	1.68	5.83E-03	2.72E-01
**COL4A4**	-1.48	1.33E-02	7.22E-01	2.96	5.85E-03	2.72E-01
**PAQR3**	0.24	3.88E-01	1.00E+00	-1.10	5.86E-03	2.72E-01
**LINC00871**	0.59	5.45E-01	1.00E+00	-3.83	5.86E-03	2.72E-01
**AC187652.1**	-1.19	2.21E-01	1.00E+00	3.71	5.87E-03	2.72E-01
**AL390037.1**	-0.79	8.13E-01	1.00E+00	-3.69	5.90E-03	2.73E-01
**CHN1**	-0.64	3.11E-01	1.00E+00	1.62	5.94E-03	2.74E-01
**NEXMIF**	-0.86	8.12E-03	5.56E-01	-1.68	5.94E-03	2.74E-01
**PRSS22**	-0.28	6.29E-01	1.00E+00	2.44	5.99E-03	2.76E-01
**DLGAP1-AS5**	-0.91	5.12E-01	1.00E+00	-3.52	6.01E-03	2.76E-01
**XLOC_000916**	0.33	7.82E-01	1.00E+00	-2.85	6.03E-03	2.77E-01
**MNDA**	-0.13	8.05E-01	1.00E+00	1.19	6.04E-03	2.77E-01
**SLC47A1**	-0.36	1.78E-01	1.00E+00	-0.98	6.07E-03	2.77E-01
**BMP8A**	0.32	7.82E-01	1.00E+00	3.87	6.17E-03	2.82E-01
**H19**	1.24	1.32E-02	7.22E-01	1.55	6.22E-03	2.83E-01
**TTYH3**	-0.77	9.66E-03	6.09E-01	2.13	6.22E-03	2.83E-01
**AKR1B10**	-0.98	1.71E-02	8.25E-01	2.06	6.24E-03	2.83E-01
**RNU2-64P**	NA	NA	NA	-3.04	6.24E-03	2.83E-01
**IRAK1BP1**	-0.23	2.90E-01	1.00E+00	-0.95	6.26E-03	2.83E-01
**AC010168.1**	0.44	5.77E-01	1.00E+00	-2.20	6.30E-03	2.84E-01

**IGKV2D-30**	3.66	5.54E-02	1.00E+00	3.82	6.38E-03	2.87E-01
**HLA-V**	0.03	9.74E-01	1.00E+00	1.35	6.40E-03	2.87E-01
**SGCA**	0.64	3.12E-01	1.00E+00	1.44	6.40E-03	2.87E-01
**ACADL**	0.34	3.50E-01	1.00E+00	-1.62	6.44E-03	2.87E-01
**MGST1**	2.40	3.37E-02	1.00E+00	-1.84	6.45E-03	2.87E-01
**AC007608.4**	1.44	4.13E-01	1.00E+00	-3.47	6.46E-03	2.87E-01
**ADAM23**	-0.47	3.68E-01	1.00E+00	2.15	6.47E-03	2.87E-01
**TMSB4XP8**	-0.15	6.23E-01	1.00E+00	-1.15	6.48E-03	2.87E-01
**HSD17B1**	-0.40	1.58E-01	1.00E+00	-1.52	6.49E-03	2.87E-01
**PROSER2-AS1**	-0.19	7.86E-01	1.00E+00	-2.04	6.50E-03	2.87E-01
**OLFML2B**	-0.26	6.06E-01	1.00E+00	1.87	6.51E-03	2.87E-01
**CREB3L1**	0.11	8.50E-01	1.00E+00	1.31	6.52E-03	2.87E-01
**FAM173B**	0.12	7.17E-01	1.00E+00	-0.96	6.52E-03	2.87E-01
**CMPK1**	0.10	5.98E-01	1.00E+00	-0.66	6.55E-03	2.88E-01
**TENM3**	0.95	2.02E-01	1.00E+00	2.10	6.57E-03	2.89E-01
**ITGAM**	0.10	8.38E-01	1.00E+00	2.16	6.64E-03	2.91E-01
**C1QB**	0.34	5.45E-01	1.00E+00	1.93	6.65E-03	2.91E-01
**TNFAIP8L2**	-0.44	5.29E-01	1.00E+00	1.33	6.71E-03	2.92E-01
**RP1**	-0.44	6.49E-01	1.00E+00	-2.34	6.71E-03	2.92E-01
**KRT37**	-2.12	1.69E-01	1.00E+00	3.69	6.72E-03	2.92E-01
**ZNF683**	-0.02	9.88E-01	1.00E+00	2.64	6.75E-03	2.93E-01
**RNU2-63P**	-10.56	2.06E-03	2.39E-01	-2.58	6.76E-03	2.93E-01
**RBFOX2**	0.15	6.59E-01	1.00E+00	0.42	6.79E-03	2.93E-01
**XLOC_007053**	-2.22	3.25E-01	1.00E+00	1.66	6.79E-03	2.93E-01
**IGKJ5**	NA	NA	NA	4.76	6.80E-03	2.93E-01
**HMGN2P6**	-1.02	7.65E-01	1.00E+00	-1.73	6.81E-03	2.93E-01
**SCAMP5**	0.91	2.42E-01	1.00E+00	1.98	6.86E-03	2.94E-01
**AC005696.4**	-0.62	1.86E-01	1.00E+00	-2.48	6.87E-03	2.94E-01
**FOLH1B**	-1.60	6.41E-01	1.00E+00	2.94	6.87E-03	2.94E-01
**STEAP1B**	-0.76	2.02E-01	1.00E+00	1.70	6.87E-03	2.94E-01
**MCUR1**	0.13	7.03E-01	1.00E+00	-0.73	6.88E-03	2.94E-01
**MYO1G**	-0.55	2.58E-01	1.00E+00	2.37	6.91E-03	2.94E-01

**AC009950.1**	0.45	5.86E-01	1.00E+00	1.50	6.92E-03	2.95E-01
**CASP4**	-0.18	5.10E-01	1.00E+00	0.74	6.95E-03	2.95E-01
**PCOLCE**	0.32	5.91E-01	1.00E+00	1.43	7.01E-03	2.97E-01
**ZNF471**	0.40	2.90E-01	1.00E+00	-1.49	7.02E-03	2.97E-01
**SCD**	1.04	7.44E-01	1.00E+00	-2.16	7.02E-03	2.97E-01
**AC241584.1**	-1.38	6.85E-01	1.00E+00	-2.87	7.07E-03	2.97E-01
**EFCAB11**	0.04	9.01E-01	1.00E+00	-0.67	7.08E-03	2.97E-01
**DNAJC27-AS1**	0.15	7.33E-01	1.00E+00	-1.13	7.09E-03	2.97E-01
**TAGAP**	0.32	4.78E-01	1.00E+00	1.65	7.12E-03	2.97E-01
**AL356056.1**	NA	NA	NA	3.19	7.12E-03	2.97E-01
**C16orf54**	-0.03	9.67E-01	1.00E+00	1.50	7.12E-03	2.97E-01
**YBX3**	-0.09	7.61E-01	1.00E+00	-1.44	7.13E-03	2.97E-01
**CD300A**	-0.22	5.79E-01	1.00E+00	1.46	7.18E-03	2.99E-01
**AC006511.3**	0.20	8.51E-01	1.00E+00	-1.78	7.22E-03	2.99E-01
**AC009902.3**	2.95	7.53E-02	1.00E+00	-3.26	7.23E-03	2.99E-01
**LYPD1**	-0.46	6.46E-01	1.00E+00	3.05	7.23E-03	2.99E-01
**AVL9**	0.09	6.31E-01	1.00E+00	0.75	7.26E-03	2.99E-01
**KLK12**	-0.27	9.04E-01	1.00E+00	2.60	7.27E-03	2.99E-01
**TF**	2.32	1.38E-01	1.00E+00	-2.26	7.28E-03	2.99E-01
**AC068282.1**	-2.39	1.93E-02	8.74E-01	-1.42	7.29E-03	2.99E-01
**AC012618.3**	-1.87	4.74E-01	1.00E+00	2.52	7.29E-03	2.99E-01
**THBS2**	0.03	9.31E-01	1.00E+00	0.84	7.31E-03	2.99E-01
**SMIM22**	2.84	2.23E-02	8.98E-01	-1.87	7.31E-03	2.99E-01
**CCDC91**	-0.10	6.43E-01	1.00E+00	-0.65	7.31E-03	2.99E-01
**RNU5A-1**	-2.88	2.90E-01	1.00E+00	-2.22	7.35E-03	3.00E-01
**PPIAP59**	0.65	7.76E-01	1.00E+00	2.87	7.36E-03	3.00E-01
**MVB12A**	-0.29	2.40E-01	1.00E+00	0.31	7.37E-03	3.00E-01
**CTSL**	0.16	6.80E-01	1.00E+00	1.37	7.41E-03	3.01E-01
**AL139156.2**	-0.35	7.05E-01	1.00E+00	-1.37	7.43E-03	3.01E-01
**CCL11**	-1.39	6.89E-01	1.00E+00	3.41	7.44E-03	3.01E-01
**AL359317.2**	0.53	5.90E-01	1.00E+00	-2.29	7.48E-03	3.02E-01
**RGS4**	-1.43	1.72E-01	1.00E+00	3.07	7.48E-03	3.02E-01

**AEN**	-0.56	9.25E-02	1.00E+00	1.34	7.50E-03	3.02E-01
**RN7SL138P**	-0.80	4.97E-01	1.00E+00	1.86	7.51E-03	3.02E-01
**PTK7**	-0.10	7.15E-01	1.00E+00	1.34	7.52E-03	3.02E-01
**MIR5689HG**	0.98	7.78E-01	1.00E+00	3.87	7.54E-03	3.02E-01
**LY9**	0.18	8.04E-01	1.00E+00	2.07	7.55E-03	3.02E-01
**ADIPOR2**	0.43	1.01E-01	1.00E+00	0.89	7.56E-03	3.02E-01
**MIR181A1HG**	NA	NA	NA	3.04	7.60E-03	3.03E-01
**AC108673.2**	-0.89	3.13E-01	1.00E+00	3.37	7.62E-03	3.03E-01
**ZSCAN16-AS1**	0.51	4.60E-01	1.00E+00	-1.12	7.62E-03	3.03E-01
**TRAF3IP2**	-0.09	7.04E-01	1.00E+00	0.57	7.64E-03	3.04E-01
**AL121672.2**	1.39	2.72E-01	1.00E+00	-2.69	7.66E-03	3.04E-01
**B3GAT1**	0.17	9.46E-01	1.00E+00	3.41	7.68E-03	3.04E-01
**PTPN1**	-0.07	7.53E-01	1.00E+00	0.92	7.76E-03	3.06E-01
**C1QA**	0.20	6.78E-01	1.00E+00	1.74	7.76E-03	3.06E-01
**SHMT1**	-0.06	8.09E-01	1.00E+00	-0.86	7.84E-03	3.08E-01
**DUXAP10**	-0.63	4.16E-01	1.00E+00	2.28	7.84E-03	3.08E-01
**GKAP1**	0.08	8.55E-01	1.00E+00	-1.67	7.87E-03	3.09E-01
**GPR183**	-1.03	1.31E-01	1.00E+00	1.22	7.94E-03	3.11E-01
**AL034376.1**	-2.51	2.45E-02	9.24E-01	1.99	7.99E-03	3.13E-01
**G30347**	-1.36	5.86E-02	1.00E+00	3.40	8.02E-03	3.13E-01
**NARS2**	0.05	7.83E-01	1.00E+00	-0.76	8.03E-03	3.13E-01
**G25302**	-3.09	1.74E-03	2.31E-01	-3.13	8.04E-03	3.13E-01
**GAL**	0.15	9.25E-01	1.00E+00	-2.63	8.07E-03	3.14E-01
**AC083829.2**	0.43	9.03E-01	1.00E+00	-2.66	8.13E-03	3.15E-01
**TIMD4**	-0.12	9.17E-01	1.00E+00	2.19	8.14E-03	3.15E-01
**TYROBP**	-0.29	4.74E-01	1.00E+00	1.31	8.15E-03	3.15E-01
**LRRC37A**	0.42	4.75E-01	1.00E+00	1.04	8.20E-03	3.16E-01
**PLD3**	-0.14	6.00E-01	1.00E+00	1.06	8.20E-03	3.16E-01
**PAX6**	-0.49	5.57E-01	1.00E+00	-2.40	8.20E-03	3.16E-01
**G39617**	-0.97	6.36E-01	1.00E+00	-2.36	8.25E-03	3.18E-01
**LINC02446**	-1.24	3.80E-01	1.00E+00	3.70	8.29E-03	3.18E-01
**LL22NC03-N14H11.1**	2.34	1.80E-01	1.00E+00	4.30	8.31E-03	3.19E-01

**RAD51-AS1**	0.77	9.20E-02	1.00E+00	-1.05	8.36E-03	3.20E-01
**RPL9P29**	-0.24	8.91E-01	1.00E+00	2.95	8.37E-03	3.20E-01
**NLGN4X**	0.21	5.11E-01	1.00E+00	1.52	8.39E-03	3.20E-01
**AL357833.1**	1.32	1.55E-01	1.00E+00	1.93	8.39E-03	3.20E-01
**ADAMTS16**	-1.75	1.60E-01	1.00E+00	2.91	8.45E-03	3.21E-01
**AC105094.1**	-0.18	8.68E-01	1.00E+00	-2.16	8.46E-03	3.21E-01
**RTP4**	-0.41	6.19E-01	1.00E+00	1.63	8.47E-03	3.21E-01
**WNT5A**	-0.61	4.45E-02	1.00E+00	1.62	8.50E-03	3.21E-01
**TYW1B**	2.01	5.73E-03	4.50E-01	1.24	8.51E-03	3.21E-01
**ADA2**	-0.39	2.55E-01	1.00E+00	1.60	8.54E-03	3.21E-01
**POC1B-AS1**	0.03	9.56E-01	1.00E+00	-1.06	8.54E-03	3.21E-01
**EFNA3**	-0.06	8.99E-01	1.00E+00	-1.42	8.54E-03	3.21E-01
**MC5R**	0.29	7.83E-01	1.00E+00	-3.15	8.58E-03	3.21E-01
**IGSF5**	-0.89	4.17E-01	1.00E+00	-2.55	8.59E-03	3.21E-01
**EYA2**	0.08	8.83E-01	1.00E+00	1.18	8.60E-03	3.21E-01
**ZNF565**	-0.20	5.89E-01	1.00E+00	-0.87	8.61E-03	3.21E-01
**CCDC62**	-0.23	7.70E-01	1.00E+00	-2.42	8.64E-03	3.21E-01
**LGALS3BP**	0.13	8.34E-01	1.00E+00	1.31	8.64E-03	3.21E-01
**PLA2G5**	0.91	1.40E-01	1.00E+00	1.55	8.65E-03	3.21E-01
**CTD-2328D6.1**	0.06	8.49E-01	1.00E+00	-1.69	8.67E-03	3.21E-01
**AC018638.7**	2.98	3.78E-01	1.00E+00	5.70	8.67E-03	3.21E-01
**EPSTI1**	-0.22	7.61E-01	1.00E+00	1.80	8.67E-03	3.21E-01
**CEP70**	-0.03	9.16E-01	1.00E+00	-0.75	8.68E-03	3.21E-01
**B4GALT5**	0.39	2.08E-01	1.00E+00	1.50	8.72E-03	3.22E-01
**FAM53B-AS1**	1.44	6.73E-01	1.00E+00	-3.91	8.74E-03	3.23E-01
**FCGR3A**	-0.55	3.75E-01	1.00E+00	2.31	8.78E-03	3.23E-01
**IGKV1D-27**	1.43	6.78E-01	1.00E+00	4.09	8.78E-03	3.23E-01
**IL10**	0.06	9.48E-01	1.00E+00	2.21	8.79E-03	3.23E-01
**AL109628.1**	NA	NA	NA	-3.42	8.83E-03	3.24E-01
**AP005717.1**	-3.62	2.83E-01	1.00E+00	-3.82	8.86E-03	3.24E-01
**XLOC_011805**	0.55	7.73E-01	1.00E+00	-3.64	8.86E-03	3.24E-01
**LATS2**	-0.16	5.16E-01	1.00E+00	1.24	8.95E-03	3.27E-01

**RIN2**	-0.18	2.78E-01	1.00E+00	0.65	8.98E-03	3.27E-01
**GATC**	0.08	7.57E-01	1.00E+00	-0.49	8.99E-03	3.27E-01
**G38234**	-0.80	6.75E-01	1.00E+00	-3.12	9.04E-03	3.28E-01
**ACP2**	0.01	9.59E-01	1.00E+00	0.80	9.06E-03	3.28E-01
**COL5A3**	-0.62	2.55E-02	9.29E-01	1.90	9.08E-03	3.28E-01
**MAP4K5**	0.07	8.23E-01	1.00E+00	-0.59	9.08E-03	3.28E-01
**AC011473.4**	-0.45	4.82E-01	1.00E+00	1.70	9.09E-03	3.28E-01
**KPNA2**	0.04	8.77E-01	1.00E+00	0.57	9.09E-03	3.28E-01
**C5AR1**	0.34	4.18E-01	1.00E+00	1.92	9.12E-03	3.28E-01
**C1QL4**	0.00	9.97E-01	1.00E+00	-3.11	9.14E-03	3.28E-01
**SZRD1**	-0.01	9.38E-01	1.00E+00	0.66	9.14E-03	3.28E-01
**LINC01203**	-1.93	5.74E-01	1.00E+00	-3.92	9.15E-03	3.28E-01
**TRBV7-6**	4.10	1.04E-01	1.00E+00	2.26	9.16E-03	3.28E-01
**AP001024.1**	-2.04	5.52E-01	1.00E+00	-2.16	9.20E-03	3.29E-01
**TFP1**	1.97	2.73E-01	1.00E+00	-3.49	9.20E-03	3.29E-01
**KRT6A**	-0.57	2.13E-01	1.00E+00	2.48	9.26E-03	3.29E-01
**CATSPER2**	-0.15	8.14E-01	1.00E+00	-1.18	9.27E-03	3.29E-01
**AL109917.1**	0.00	9.95E-01	1.00E+00	-2.19	9.27E-03	3.29E-01
**SLFNL1-AS1**	2.59	8.72E-02	1.00E+00	2.34	9.27E-03	3.29E-01
**CCDC3**	-0.41	1.30E-01	1.00E+00	-1.10	9.28E-03	3.29E-01
**CCDC28B**	-0.25	6.35E-01	1.00E+00	0.84	9.28E-03	3.29E-01
**AC093157.1**	-0.67	1.49E-01	1.00E+00	-1.10	9.31E-03	3.29E-01
**AC068733.3**	1.63	1.85E-01	1.00E+00	-2.43	9.32E-03	3.29E-01
**STARD3**	-0.02	9.01E-01	1.00E+00	0.43	9.35E-03	3.30E-01
**STAB1**	0.24	6.36E-01	1.00E+00	1.87	9.39E-03	3.30E-01
**C1orf162**	-0.46	1.99E-01	1.00E+00	1.11	9.41E-03	3.30E-01
**LCE3C**	0.00	1.00E+00	1.00E+00	3.72	9.41E-03	3.30E-01
**LIPC**	0.89	6.30E-02	1.00E+00	1.40	9.43E-03	3.31E-01
**AL356056.2**	0.08	9.07E-01	1.00E+00	1.60	9.46E-03	3.31E-01
**XLOC_008786**	0.22	8.94E-01	1.00E+00	-3.51	9.49E-03	3.32E-01
**YBX1P2**	-0.05	9.59E-01	1.00E+00	-0.81	9.53E-03	3.32E-01
**PTPRO**	-0.04	9.60E-01	1.00E+00	2.87	9.53E-03	3.32E-01

**LIMD2**	-0.51	1.57E-01	1.00E+00	1.47	9.54E-03	3.32E-01
**CXCL3**	-1.50	2.56E-01	1.00E+00	2.45	9.56E-03	3.32E-01
**10-Sep**	0.13	3.73E-01	1.00E+00	-0.37	9.59E-03	3.32E-01
**G20080**	-1.80	4.42E-01	1.00E+00	3.78	9.59E-03	3.32E-01
**LONRF1**	0.39	2.53E-01	1.00E+00	-0.98	9.61E-03	3.32E-01
**EIF1AXP1**	0.46	3.45E-01	1.00E+00	-0.85	9.61E-03	3.32E-01
**PLXND1**	0.07	8.63E-01	1.00E+00	1.94	9.65E-03	3.33E-01
**IL4I1**	-1.21	1.13E-01	1.00E+00	2.34	9.66E-03	3.33E-01
**AC099066.2**	-0.20	9.02E-01	1.00E+00	1.99	9.69E-03	3.33E-01
**MX2**	-0.04	9.59E-01	1.00E+00	1.93	9.72E-03	3.34E-01
**PPP1R9B**	-0.46	6.99E-02	1.00E+00	1.90	9.77E-03	3.35E-01
**FBXO25**	0.10	5.41E-01	1.00E+00	-0.46	9.77E-03	3.35E-01
**WBP2NL**	-0.39	6.31E-01	1.00E+00	-1.51	9.81E-03	3.36E-01
**COPZ2**	0.01	9.72E-01	1.00E+00	-1.72	9.82E-03	3.36E-01
**HELZ2**	-0.40	4.80E-01	1.00E+00	2.25	9.87E-03	3.36E-01
**P3H1**	-0.19	6.28E-01	1.00E+00	1.06	9.87E-03	3.36E-01
**AP003392.4**	-0.47	2.59E-01	1.00E+00	-1.21	9.88E-03	3.36E-01
**RPRML**	0.76	5.08E-01	1.00E+00	-2.10	9.90E-03	3.36E-01
**PGRMC2**	0.06	8.31E-01	1.00E+00	-0.95	9.91E-03	3.36E-01
**AP003774.3**	-0.16	9.62E-01	1.00E+00	4.47	9.92E-03	3.36E-01
**TSTD3**	-0.19	7.45E-01	1.00E+00	-1.18	9.94E-03	3.36E-01
**FAM20A**	-0.02	9.69E-01	1.00E+00	1.91	9.95E-03	3.36E-01
**CCL5**	-1.34	4.21E-02	1.00E+00	1.82	9.97E-03	3.36E-01
**DLSTP1**	-0.45	8.42E-01	1.00E+00	1.88	9.99E-03	3.36E-01
**TRAV30**	1.33	6.99E-01	1.00E+00	3.65	1.00E-02	3.36E-01
**LST1**	-0.92	4.06E-02	1.00E+00	0.92	1.00E-02	3.36E-01
**IFITM3**	0.27	5.94E-01	1.00E+00	0.93	1.00E-02	3.36E-01
**MRC2**	0.30	5.13E-01	1.00E+00	1.66	1.00E-02	3.37E-01
**RPTN**	-0.43	5.79E-01	1.00E+00	2.18	1.01E-02	3.38E-01
**RRP7BP**	-0.46	3.38E-01	1.00E+00	1.14	1.01E-02	3.38E-01
**RASSF3**	0.53	3.25E-01	1.00E+00	1.30	1.01E-02	3.38E-01
**G33755**	-1.65	6.32E-01	1.00E+00	3.85	1.01E-02	3.38E-01

**ZBTB37**	0.11	7.07E-01	1.00E+00	0.47	1.02E-02	3.38E-01
**ARRB2**	-0.29	1.80E-01	1.00E+00	1.09	1.02E-02	3.39E-01
**PFN1P8**	0.76	6.83E-01	1.00E+00	-2.99	1.02E-02	3.39E-01
**NMI**	-0.29	5.47E-01	1.00E+00	0.79	1.03E-02	3.39E-01
**FBXO10**	-0.02	9.57E-01	1.00E+00	1.16	1.03E-02	3.39E-01
**ENOPH1**	0.00	9.99E-01	1.00E+00	-0.29	1.03E-02	3.39E-01
**TLR7**	-0.28	6.06E-01	1.00E+00	2.22	1.03E-02	3.39E-01
**SLC36A1**	0.06	7.98E-01	1.00E+00	1.10	1.03E-02	3.39E-01
**CXCR4**	0.18	6.48E-01	1.00E+00	1.82	1.03E-02	3.39E-01
**PLXDC1**	-0.13	7.44E-01	1.00E+00	1.31	1.03E-02	3.39E-01
**AC022167.3**	-2.19	3.48E-01	1.00E+00	-2.20	1.03E-02	3.39E-01
**RNU4-2**	-0.17	8.89E-01	1.00E+00	-2.43	1.03E-02	3.39E-01
**NSRP1P1**	0.88	3.19E-01	1.00E+00	1.42	1.04E-02	3.40E-01
**HIST1H1D**	-0.71	4.48E-01	1.00E+00	-2.02	1.04E-02	3.40E-01
**CASP16P**	-1.76	2.32E-01	1.00E+00	3.22	1.04E-02	3.40E-01
**LIMK1**	-0.23	3.75E-01	1.00E+00	1.74	1.04E-02	3.40E-01
**AGRN**	-0.07	8.22E-01	1.00E+00	2.04	1.06E-02	3.45E-01
**SIGLEC1**	0.70	2.19E-01	1.00E+00	2.31	1.06E-02	3.46E-01
**AL161457.2**	-1.86	5.37E-01	1.00E+00	-2.07	1.06E-02	3.46E-01
**NGF-AS1**	1.98	4.79E-01	1.00E+00	3.38	1.07E-02	3.46E-01
**FNDC3A**	0.17	5.22E-01	1.00E+00	0.56	1.07E-02	3.46E-01
**CNPY3**	-0.04	8.80E-01	1.00E+00	0.73	1.07E-02	3.46E-01
**IGKV1-33**	0.93	6.15E-01	1.00E+00	3.63	1.07E-02	3.46E-01
**AC092171.2**	-0.12	7.85E-01	1.00E+00	1.15	1.07E-02	3.46E-01
**GDF11**	-0.24	5.71E-01	1.00E+00	1.27	1.08E-02	3.48E-01
**AL162412.1**	1.27	5.02E-01	1.00E+00	-3.63	1.08E-02	3.48E-01
**TTC19**	0.05	7.80E-01	1.00E+00	-1.33	1.08E-02	3.48E-01
**DDX3Y**	0.91	2.83E-01	1.00E+00	2.78	1.09E-02	3.50E-01
**LAMA5-AS1**	-1.00	7.53E-01	1.00E+00	-2.72	1.09E-02	3.50E-01
**FBXL4**	0.19	5.87E-01	1.00E+00	-0.53	1.09E-02	3.50E-01
**PRMT8**	-1.86	3.07E-01	1.00E+00	2.81	1.09E-02	3.50E-01
**FRA10AC1**	0.32	2.92E-01	1.00E+00	-0.85	1.09E-02	3.50E-01

**KRT16P1**	-1.13	6.02E-01	1.00E+00	1.81	1.10E-02	3.50E-01
**AL157829.1**	-2.50	1.93E-01	1.00E+00	2.91	1.10E-02	3.50E-01
**DNAJC5**	0.01	9.64E-01	1.00E+00	1.23	1.10E-02	3.50E-01
**FAM172A**	0.36	1.81E-01	1.00E+00	-0.70	1.11E-02	3.51E-01
**GALK1**	-0.03	8.59E-01	1.00E+00	0.63	1.11E-02	3.51E-01
**LAMC2**	0.22	4.40E-01	1.00E+00	1.05	1.11E-02	3.52E-01
**SCT**	-2.90	3.45E-01	1.00E+00	3.70	1.11E-02	3.53E-01
**BMP1**	-0.44	1.40E-01	1.00E+00	1.67	1.12E-02	3.53E-01
**KRT4**	2.27	6.45E-02	1.00E+00	2.43	1.12E-02	3.53E-01
**ARMC10**	0.25	2.97E-01	1.00E+00	-1.50	1.12E-02	3.53E-01
**CALR4P**	-0.80	8.18E-01	1.00E+00	-4.20	1.13E-02	3.53E-01
**CFP**	-0.03	9.49E-01	1.00E+00	1.45	1.13E-02	3.53E-01
**AC002429.2**	-1.59	5.17E-01	1.00E+00	-3.98	1.13E-02	3.53E-01
**BTF3L4P2**	0.61	4.38E-01	1.00E+00	-1.11	1.13E-02	3.53E-01
**HIST2H2AC**	-0.24	8.11E-01	1.00E+00	-1.35	1.13E-02	3.53E-01
**CTSS**	0.00	9.90E-01	1.00E+00	1.40	1.13E-02	3.53E-01
**CYP19A1**	-0.17	7.74E-01	1.00E+00	2.06	1.13E-02	3.54E-01
**GRIA1**	-1.18	1.90E-01	1.00E+00	2.11	1.13E-02	3.54E-01
**ITGB3BP**	-0.41	2.03E-01	1.00E+00	-0.85	1.14E-02	3.54E-01
**ERVMER61-1**	2.43	4.64E-02	1.00E+00	-2.71	1.14E-02	3.54E-01
**RELT**	-0.43	2.75E-01	1.00E+00	1.68	1.14E-02	3.54E-01
**SIRPAP1**	-0.39	8.99E-01	1.00E+00	-2.68	1.14E-02	3.54E-01
**AC008147.3**	1.39	2.06E-01	1.00E+00	2.24	1.14E-02	3.54E-01
**ARNT2**	-0.38	4.55E-01	1.00E+00	1.45	1.14E-02	3.54E-01
**AP001627.1**	1.35	2.62E-01	1.00E+00	-2.72	1.14E-02	3.54E-01
**FCGR1B**	-1.21	3.48E-01	1.00E+00	2.33	1.14E-02	3.54E-01
**LINC01615**	-0.22	6.52E-01	1.00E+00	1.44	1.15E-02	3.54E-01
**SIGLEC8**	1.71	1.08E-01	1.00E+00	2.75	1.15E-02	3.54E-01
**AL157700.1**	-1.11	4.99E-01	1.00E+00	-2.74	1.15E-02	3.54E-01
**LINC01305**	-0.34	7.33E-01	1.00E+00	2.53	1.15E-02	3.54E-01
**AL158212.5**	0.59	3.93E-01	1.00E+00	1.87	1.16E-02	3.55E-01
**G10489**	-0.91	2.39E-01	1.00E+00	-3.46	1.16E-02	3.55E-01

**HIBADH**	0.39	3.21E-01	1.00E+00	-0.91	1.16E-02	3.55E-01
**AC018755.4**	0.32	7.98E-01	1.00E+00	2.54	1.16E-02	3.55E-01
**GCSH**	0.86	4.30E-01	1.00E+00	-1.20	1.16E-02	3.55E-01
**PKD1L3**	-1.93	4.48E-01	1.00E+00	2.73	1.17E-02	3.55E-01
**LINC01002**	-0.93	4.31E-01	1.00E+00	2.81	1.17E-02	3.55E-01
**SP110**	-0.14	7.71E-01	1.00E+00	1.00	1.17E-02	3.55E-01
**MT-TE**	NA	NA	NA	-4.50	1.17E-02	3.55E-01
**KCP**	0.42	5.38E-01	1.00E+00	1.88	1.17E-02	3.55E-01
**AL355353.1**	-0.86	1.68E-01	1.00E+00	-1.39	1.17E-02	3.55E-01
**EPHA5**	-1.03	1.14E-01	1.00E+00	-2.79	1.18E-02	3.55E-01
**PAX5**	-2.30	4.82E-01	1.00E+00	2.36	1.18E-02	3.55E-01
**AC015910.1**	0.56	5.86E-01	1.00E+00	-2.00	1.18E-02	3.55E-01
**CD180**	0.58	5.83E-01	1.00E+00	2.43	1.18E-02	3.55E-01
**SLC26A7**	0.15	8.80E-01	1.00E+00	-2.14	1.18E-02	3.55E-01
**CBX6**	0.06	8.77E-01	1.00E+00	0.66	1.18E-02	3.55E-01
**PODNL1**	0.05	9.00E-01	1.00E+00	1.19	1.18E-02	3.55E-01
**SYCP3**	-1.22	5.66E-01	1.00E+00	-2.06	1.18E-02	3.55E-01
**OAS1**	0.48	5.40E-01	1.00E+00	1.41	1.18E-02	3.55E-01
**MXI1**	0.07	7.91E-01	1.00E+00	-0.88	1.19E-02	3.55E-01
**SMPD1**	-0.09	7.26E-01	1.00E+00	0.79	1.19E-02	3.55E-01
**LINC02386**	-3.00	1.21E-01	1.00E+00	-3.63	1.19E-02	3.55E-01
**AC009093.1**	-1.79	1.20E-01	1.00E+00	1.60	1.19E-02	3.55E-01
**IGLC7**	1.76	6.07E-01	1.00E+00	3.03	1.19E-02	3.55E-01
**CYP3A5**	1.00	1.14E-01	1.00E+00	1.54	1.20E-02	3.56E-01
**STXBP5L**	-1.65	1.60E-01	1.00E+00	-3.38	1.20E-02	3.56E-01
**TTC6**	0.51	7.81E-01	1.00E+00	-2.55	1.20E-02	3.56E-01
**AC036176.1**	-0.04	9.54E-01	1.00E+00	-1.14	1.20E-02	3.57E-01
**SLC7A4**	-0.12	7.89E-01	1.00E+00	-1.54	1.21E-02	3.58E-01
**CDC42P6**	2.78	2.07E-02	8.94E-01	1.30	1.22E-02	3.59E-01
**COX6CP1**	0.56	8.71E-01	1.00E+00	2.44	1.22E-02	3.59E-01
**BICC1**	0.31	4.94E-01	1.00E+00	0.93	1.22E-02	3.59E-01
**NACC1**	-0.18	5.96E-01	1.00E+00	2.03	1.22E-02	3.61E-01

**MARCKS**	-0.03	8.64E-01	1.00E+00	0.83	1.23E-02	3.61E-01
**AL353807.3**	0.44	5.43E-01	1.00E+00	1.33	1.23E-02	3.62E-01
**CCL4L2**	0.71	8.09E-01	1.00E+00	3.38	1.23E-02	3.62E-01
**ARMC9**	0.28	2.47E-01	1.00E+00	0.84	1.23E-02	3.62E-01
**CRAT37**	-1.24	7.19E-01	1.00E+00	4.98	1.24E-02	3.62E-01
**OR7E13P**	NA	NA	NA	-3.92	1.24E-02	3.62E-01
**MDM2**	0.07	7.78E-01	1.00E+00	0.50	1.24E-02	3.62E-01
**AL137186.2**	0.35	6.95E-01	1.00E+00	1.37	1.24E-02	3.62E-01
**DBP**	-0.97	6.76E-03	4.99E-01	-1.47	1.25E-02	3.63E-01
**ZNF747**	-0.02	9.52E-01	1.00E+00	-0.68	1.26E-02	3.66E-01
**IFIT1B**	-2.44	2.96E-01	1.00E+00	3.86	1.26E-02	3.66E-01
**SLC16A1**	-0.14	6.62E-01	1.00E+00	0.61	1.26E-02	3.66E-01
**SNX3**	0.10	5.31E-01	1.00E+00	-0.97	1.26E-02	3.67E-01
**AC011330.1**	0.40	7.91E-01	1.00E+00	-2.69	1.27E-02	3.68E-01
**NCF2**	0.27	3.79E-01	1.00E+00	1.06	1.27E-02	3.68E-01
**LRRC4**	-0.54	3.31E-01	1.00E+00	2.24	1.27E-02	3.68E-01
**ISLR**	0.76	9.21E-02	1.00E+00	1.11	1.27E-02	3.68E-01
**NPFFR2**	-0.51	6.32E-01	1.00E+00	2.92	1.28E-02	3.68E-01
**LENG1**	0.04	8.56E-01	1.00E+00	0.75	1.28E-02	3.68E-01
**NCF4**	-0.09	7.82E-01	1.00E+00	1.24	1.28E-02	3.68E-01
**ADH7**	-0.74	3.68E-01	1.00E+00	1.89	1.28E-02	3.69E-01
**ACSS2**	0.26	4.34E-01	1.00E+00	-0.98	1.29E-02	3.69E-01
**S1PR2**	0.20	5.13E-01	1.00E+00	0.80	1.29E-02	3.69E-01
**PDGFA**	-0.58	2.21E-02	8.96E-01	1.07	1.29E-02	3.69E-01
**HEPHL1**	-1.00	6.71E-01	1.00E+00	2.42	1.29E-02	3.70E-01
**DCAF4L1**	-1.32	6.98E-01	1.00E+00	-3.00	1.29E-02	3.70E-01
**SORCS2**	0.07	8.96E-01	1.00E+00	2.29	1.29E-02	3.70E-01
**BLK**	-2.28	1.67E-01	1.00E+00	3.78	1.30E-02	3.70E-01
**AL845321.1**	-0.79	6.96E-01	1.00E+00	-3.58	1.30E-02	3.71E-01
**XLOC_000992**	1.01	1.45E-01	1.00E+00	-1.94	1.30E-02	3.71E-01
**SNF8**	-0.24	2.77E-01	1.00E+00	-1.13	1.31E-02	3.72E-01
**SND1-IT1**	-1.95	4.05E-01	1.00E+00	-2.31	1.31E-02	3.72E-01

**CTSK**	0.46	3.16E-01	1.00E+00	1.06	1.31E-02	3.72E-01
**TRGC1**	0.44	7.68E-01	1.00E+00	2.64	1.31E-02	3.72E-01
**G14986**	-0.79	5.92E-01	1.00E+00	-3.29	1.32E-02	3.72E-01
**DKK4**	-2.40	5.83E-02	1.00E+00	-3.07	1.32E-02	3.72E-01
**AL355312.3**	0.35	9.19E-01	1.00E+00	2.33	1.32E-02	3.72E-01
**MCCC1**	0.18	4.59E-01	1.00E+00	-0.62	1.32E-02	3.72E-01
**AC080013.1**	-0.39	6.28E-01	1.00E+00	-1.83	1.32E-02	3.72E-01
**CPZ**	0.76	7.99E-01	1.00E+00	2.54	1.32E-02	3.73E-01
**TRIM38**	-0.09	8.08E-01	1.00E+00	0.69	1.33E-02	3.73E-01
**AC093227.1**	-0.08	9.07E-01	1.00E+00	-1.33	1.33E-02	3.73E-01
**CASR**	-2.97	2.79E-01	1.00E+00	3.85	1.33E-02	3.73E-01
**PECR**	1.22	8.50E-02	1.00E+00	-1.27	1.33E-02	3.73E-01
**AC007389.5**	-0.07	9.59E-01	1.00E+00	-2.23	1.34E-02	3.74E-01
**RTCA-AS1**	0.20	7.64E-01	1.00E+00	-1.09	1.34E-02	3.76E-01
**SNX18**	0.00	9.79E-01	1.00E+00	1.01	1.35E-02	3.76E-01
**FBLIM1**	-0.44	2.13E-01	1.00E+00	1.22	1.35E-02	3.76E-01
**CBWD3**	0.08	8.55E-01	1.00E+00	-0.95	1.35E-02	3.76E-01
**CD4**	-0.01	9.87E-01	1.00E+00	1.25	1.35E-02	3.76E-01
**AC100872.1**	-2.62	1.88E-01	1.00E+00	-3.63	1.35E-02	3.76E-01
**HPR**	2.74	1.01E-01	1.00E+00	2.76	1.36E-02	3.77E-01
**PCDHGB9P**	-2.35	2.07E-01	1.00E+00	3.04	1.36E-02	3.77E-01
**CYTH4**	-0.04	9.31E-01	1.00E+00	1.82	1.36E-02	3.77E-01
**RIPOR3**	0.67	1.93E-01	1.00E+00	2.26	1.36E-02	3.77E-01
**AC009237.14**	0.02	9.71E-01	1.00E+00	-1.42	1.37E-02	3.77E-01
**AC069307.1**	1.32	2.00E-01	1.00E+00	-1.85	1.37E-02	3.77E-01
**ASIP**	-0.15	8.33E-01	1.00E+00	1.16	1.38E-02	3.79E-01
**ACOX1**	0.39	2.42E-02	9.19E-01	-0.33	1.38E-02	3.79E-01
**SPTLC2**	0.04	8.62E-01	1.00E+00	0.60	1.38E-02	3.79E-01
**DNAJC12**	2.14	7.97E-02	1.00E+00	2.20	1.38E-02	3.80E-01
**AACS**	0.09	7.44E-01	1.00E+00	-0.82	1.39E-02	3.80E-01
**FCGR1CP**	0.07	9.80E-01	1.00E+00	3.42	1.39E-02	3.80E-01
**IGHV4-55**	NA	NA	NA	4.93	1.39E-02	3.80E-01

**AC132192.2**	1.34	4.04E-01	1.00E+00	-3.30	1.39E-02	3.81E-01
**CTSD**	0.16	4.67E-01	1.00E+00	0.97	1.40E-02	3.81E-01
**AC018653.3**	1.32	3.12E-01	1.00E+00	2.51	1.40E-02	3.82E-01
**OLA1**	-0.18	3.37E-01	1.00E+00	-0.28	1.40E-02	3.82E-01
**SPATA13**	0.00	9.97E-01	1.00E+00	0.90	1.40E-02	3.82E-01
**ST8SIA4**	-0.25	5.91E-01	1.00E+00	1.26	1.41E-02	3.82E-01
**CD22**	0.75	5.91E-01	1.00E+00	2.32	1.41E-02	3.82E-01
**TRBV7-3**	-0.13	9.57E-01	1.00E+00	1.89	1.41E-02	3.82E-01
**AC004865.2**	-1.96	1.82E-01	1.00E+00	2.15	1.41E-02	3.82E-01
**RBP7**	0.39	5.16E-01	1.00E+00	-1.21	1.41E-02	3.82E-01
**RPS2P55**	-3.41	5.85E-04	1.11E-01	-1.51	1.42E-02	3.83E-01
**SLAMF7**	-0.40	3.25E-01	1.00E+00	1.82	1.42E-02	3.83E-01
**CYBB**	0.04	9.32E-01	1.00E+00	1.86	1.42E-02	3.83E-01
**ARHGEF25**	0.22	6.64E-01	1.00E+00	1.57	1.42E-02	3.83E-01
**NBPF16**	0.76	2.43E-01	1.00E+00	1.08	1.42E-02	3.83E-01
**CAPN7**	0.14	4.29E-01	1.00E+00	-0.41	1.42E-02	3.83E-01
**G2520**	0.51	4.46E-01	1.00E+00	-2.46	1.43E-02	3.84E-01
**AC100847.1**	1.41	2.58E-01	1.00E+00	-1.70	1.43E-02	3.84E-01
**PRR34-AS1**	0.22	6.48E-01	1.00E+00	-1.63	1.44E-02	3.85E-01
**RNF213**	0.06	8.64E-01	1.00E+00	1.40	1.44E-02	3.85E-01
**CXCL12**	0.42	4.40E-01	1.00E+00	1.16	1.44E-02	3.85E-01
**LINC01206**	-0.56	8.73E-01	1.00E+00	3.88	1.44E-02	3.85E-01
**G36839**	-0.85	3.81E-01	1.00E+00	-2.48	1.45E-02	3.85E-01
**RPL24P2**	-4.27	1.54E-01	1.00E+00	-1.78	1.45E-02	3.85E-01
**GAPDHP2**	-0.79	8.17E-01	1.00E+00	3.61	1.45E-02	3.85E-01
**ANKDD1B**	-0.55	5.78E-01	1.00E+00	-2.34	1.45E-02	3.85E-01
**XLOC_008785**	3.74	2.76E-02	9.71E-01	-2.87	1.45E-02	3.85E-01
**H3F3AP6**	-0.10	7.85E-01	1.00E+00	-0.86	1.45E-02	3.85E-01
**CHRNA9**	-1.91	5.67E-01	1.00E+00	2.31	1.45E-02	3.85E-01
**SLC25A18**	0.32	6.32E-01	1.00E+00	-1.79	1.45E-02	3.85E-01
**ZNF662**	0.09	7.97E-01	1.00E+00	0.84	1.46E-02	3.85E-01
**C1orf109**	0.27	2.69E-01	1.00E+00	-0.66	1.46E-02	3.86E-01

**AL391005.1**	-1.06	2.74E-01	1.00E+00	-4.31	1.46E-02	3.86E-01
**JAML**	-0.36	5.42E-01	1.00E+00	1.34	1.47E-02	3.86E-01
**LRP1**	0.76	1.43E-01	1.00E+00	1.64	1.47E-02	3.86E-01
**CD2BP2**	-0.01	9.20E-01	1.00E+00	0.50	1.47E-02	3.86E-01
**DSG1-AS1**	-0.86	5.83E-01	1.00E+00	1.65	1.47E-02	3.86E-01
**CKM**	-2.05	5.29E-01	1.00E+00	-2.92	1.47E-02	3.86E-01
**RNF125**	-0.10	6.15E-01	1.00E+00	-0.57	1.47E-02	3.86E-01
**GPATCH11**	0.31	2.61E-01	1.00E+00	-1.05	1.47E-02	3.86E-01
**TMEM115**	-0.06	7.80E-01	1.00E+00	0.42	1.47E-02	3.86E-01
**AP000894.4**	0.15	8.41E-01	1.00E+00	-1.52	1.48E-02	3.86E-01
**AC122718.1**	0.10	8.64E-01	1.00E+00	1.14	1.48E-02	3.86E-01
**AGAP2**	-0.54	3.19E-01	1.00E+00	1.87	1.48E-02	3.86E-01
**IQCH-AS1**	-0.02	9.57E-01	1.00E+00	-1.28	1.48E-02	3.86E-01
**RNF14**	0.53	1.02E-01	1.00E+00	-0.74	1.48E-02	3.86E-01
**MUCL1**	0.71	5.70E-01	1.00E+00	-1.98	1.49E-02	3.86E-01
**GLIS2**	0.01	9.78E-01	1.00E+00	1.74	1.49E-02	3.86E-01
**ADAM8**	-0.98	8.05E-02	1.00E+00	2.53	1.49E-02	3.86E-01
**LPIN3**	-0.27	3.55E-01	1.00E+00	0.69	1.49E-02	3.86E-01
**PCNX1**	0.31	2.24E-01	1.00E+00	1.06	1.49E-02	3.86E-01
**ALG14**	0.02	9.35E-01	1.00E+00	-0.69	1.49E-02	3.86E-01
**PSPC1**	-0.27	7.47E-02	1.00E+00	0.45	1.49E-02	3.86E-01
**PANX1**	-0.13	7.19E-01	1.00E+00	0.89	1.50E-02	3.86E-01
**AC012569.1**	-1.55	6.45E-01	1.00E+00	2.69	1.50E-02	3.86E-01
**FAF1**	-0.14	5.55E-01	1.00E+00	-1.18	1.50E-02	3.86E-01
**CDH3**	-0.65	2.28E-02	9.09E-01	1.45	1.50E-02	3.86E-01
**CFAP97**	-0.01	9.64E-01	1.00E+00	-0.52	1.50E-02	3.86E-01
**MTCL1**	-0.96	4.92E-02	1.00E+00	1.91	1.50E-02	3.86E-01
**USP2**	-0.40	5.44E-01	1.00E+00	1.04	1.50E-02	3.86E-01
**AL354920.1**	-0.80	8.64E-02	1.00E+00	-1.03	1.51E-02	3.86E-01
**ATF5**	0.00	9.94E-01	1.00E+00	1.32	1.51E-02	3.86E-01
**CATSPER2P1**	-0.33	4.73E-01	1.00E+00	-0.84	1.51E-02	3.86E-01
**MIR4653**	-3.27	2.82E-02	9.75E-01	1.39	1.52E-02	3.86E-01

**LHFPL2**	0.19	6.67E-01	1.00E+00	1.50	1.52E-02	3.86E-01
**SRSF2**	-0.04	8.67E-01	1.00E+00	-0.57	1.52E-02	3.86E-01
**FOXI3**	-1.04	4.46E-01	1.00E+00	-3.70	1.52E-02	3.86E-01
**NFYC-AS1**	-2.58	1.28E-01	1.00E+00	-2.60	1.52E-02	3.86E-01
**RAPGEF2**	0.07	8.51E-01	1.00E+00	0.98	1.52E-02	3.86E-01
**YRDC**	0.00	9.92E-01	1.00E+00	-1.14	1.53E-02	3.89E-01
**UBE2L6**	-0.15	7.67E-01	1.00E+00	0.93	1.53E-02	3.89E-01
**MYO9B**	-0.59	3.57E-02	1.00E+00	1.77	1.53E-02	3.89E-01
**TSPAN9**	-0.02	9.36E-01	1.00E+00	1.25	1.54E-02	3.90E-01
**AL627309.1**	-0.52	6.63E-01	1.00E+00	2.61	1.54E-02	3.90E-01
**ABI3**	-0.27	4.31E-01	1.00E+00	1.01	1.54E-02	3.90E-01
**XLOC_013370**	-2.78	2.17E-01	1.00E+00	-3.61	1.54E-02	3.90E-01
**PIAS2**	0.08	7.62E-01	1.00E+00	-0.74	1.55E-02	3.90E-01
**G16357**	-1.24	7.22E-03	5.15E-01	2.73	1.55E-02	3.90E-01
**OR52K3P**	0.29	8.48E-01	1.00E+00	2.79	1.55E-02	3.90E-01
**NFATC4**	0.23	5.40E-01	1.00E+00	1.46	1.56E-02	3.91E-01
**PTGIR**	0.06	8.90E-01	1.00E+00	1.26	1.56E-02	3.92E-01
**RPS4XP22**	0.54	8.31E-01	1.00E+00	-2.60	1.56E-02	3.92E-01
**PSMB9**	-0.31	4.91E-01	1.00E+00	0.92	1.57E-02	3.92E-01
**SNX29**	-0.11	6.17E-01	1.00E+00	0.84	1.57E-02	3.92E-01
**FBN2**	0.57	4.64E-01	1.00E+00	3.00	1.57E-02	3.92E-01
**MUT**	0.07	7.81E-01	1.00E+00	-0.59	1.57E-02	3.92E-01
**NSUN6**	-0.11	7.47E-01	1.00E+00	-0.77	1.57E-02	3.92E-01
**TIMP2**	0.38	4.21E-01	1.00E+00	1.38	1.57E-02	3.92E-01
**ALKBH3-AS1**	0.37	7.31E-01	1.00E+00	2.18	1.58E-02	3.92E-01
**MIR31HG**	-1.88	5.82E-01	1.00E+00	3.33	1.58E-02	3.93E-01
**MYD88**	-0.28	4.27E-01	1.00E+00	0.86	1.58E-02	3.93E-01
**VCAN**	0.12	7.70E-01	1.00E+00	1.05	1.59E-02	3.93E-01
**DYNC1I2**	0.16	3.05E-01	1.00E+00	0.41	1.59E-02	3.93E-01
**C1RL**	-0.01	9.80E-01	1.00E+00	1.23	1.59E-02	3.93E-01
**LYSMD3**	0.13	6.85E-01	1.00E+00	-0.77	1.59E-02	3.93E-01
**NREP**	-0.25	5.77E-01	1.00E+00	0.76	1.59E-02	3.93E-01

**AC012360.1**	-1.60	5.08E-01	1.00E+00	-3.64	1.60E-02	3.93E-01
**AF064858.2**	0.98	5.90E-01	1.00E+00	3.22	1.60E-02	3.93E-01
**APOBR**	-0.91	6.08E-02	1.00E+00	2.49	1.60E-02	3.93E-01
**FLJ42969**	-3.54	1.20E-01	1.00E+00	3.29	1.60E-02	3.93E-01
**ZNF300P1**	0.87	1.14E-01	1.00E+00	-1.66	1.60E-02	3.93E-01
**AK7**	-0.11	8.38E-01	1.00E+00	-1.12	1.60E-02	3.93E-01
**TMEM229B**	-0.73	6.52E-02	1.00E+00	1.69	1.61E-02	3.93E-01
**VN1R20P**	-0.39	7.99E-01	1.00E+00	-2.92	1.61E-02	3.93E-01
**SCARNA21**	-2.18	4.76E-01	1.00E+00	-2.20	1.61E-02	3.93E-01
**WNT9A**	0.02	9.63E-01	1.00E+00	1.74	1.61E-02	3.93E-01
**PARVG**	-0.27	5.60E-01	1.00E+00	1.39	1.61E-02	3.93E-01
**AC093700.1**	-0.80	8.18E-01	1.00E+00	2.66	1.61E-02	3.93E-01
**HRASLS**	0.05	9.05E-01	1.00E+00	-1.56	1.62E-02	3.93E-01
**RAB21**	0.02	9.03E-01	1.00E+00	-0.58	1.62E-02	3.93E-01
**EMP3**	-0.02	9.62E-01	1.00E+00	0.64	1.62E-02	3.93E-01
**GDNF-AS1**	-1.04	5.16E-01	1.00E+00	2.47	1.62E-02	3.93E-01
**STAM-AS1**	0.79	7.65E-01	1.00E+00	1.90	1.62E-02	3.93E-01
**AL109840.2**	-0.87	7.94E-01	1.00E+00	2.46	1.62E-02	3.93E-01
**CSPG4P11**	-0.27	6.65E-01	1.00E+00	2.08	1.62E-02	3.93E-01
**IL2RG**	-1.04	2.08E-01	1.00E+00	1.08	1.62E-02	3.93E-01
**AL359921.2**	-0.34	6.66E-01	1.00E+00	1.61	1.63E-02	3.94E-01
**RAB40B**	0.05	7.73E-01	1.00E+00	-0.83	1.63E-02	3.94E-01
**TCEA3**	0.05	7.73E-01	1.00E+00	-0.83	1.63E-02	3.94E-01
**DHRS4-AS1**	-0.21	3.99E-01	1.00E+00	-0.60	1.63E-02	3.94E-01
**MRAP2**	-0.71	7.57E-02	1.00E+00	-1.68	1.64E-02	3.94E-01
**PON3**	-0.63	1.66E-01	1.00E+00	-1.55	1.64E-02	3.94E-01
**AOAH**	0.23	5.95E-01	1.00E+00	1.77	1.64E-02	3.94E-01
**TMEM202-AS1**	0.07	9.17E-01	1.00E+00	-1.23	1.64E-02	3.94E-01
**ESD**	0.06	7.35E-01	1.00E+00	-0.50	1.64E-02	3.94E-01
**PLPBP**	0.18	4.47E-01	1.00E+00	-0.60	1.64E-02	3.94E-01
**KCNJ5**	-0.09	8.51E-01	1.00E+00	2.01	1.64E-02	3.94E-01
**MASP1**	0.39	4.98E-01	1.00E+00	1.55	1.64E-02	3.94E-01

**TRIM60P18**	0.23	9.14E-01	1.00E+00	-2.73	1.65E-02	3.94E-01
**RNU6-37P**	0.78	4.28E-01	1.00E+00	2.50	1.65E-02	3.94E-01
**TCEA1P2**	-0.11	8.24E-01	1.00E+00	-0.72	1.65E-02	3.94E-01
**NAALADL2**	0.30	3.97E-01	1.00E+00	-0.77	1.66E-02	3.95E-01
**CCR1**	0.56	3.90E-01	1.00E+00	2.10	1.66E-02	3.95E-01
**BHLHB9**	-0.15	5.24E-01	1.00E+00	-0.98	1.66E-02	3.95E-01
**U2AF2**	-0.23	2.29E-01	1.00E+00	1.74	1.66E-02	3.95E-01
**G41661**	0.08	8.85E-01	1.00E+00	1.95	1.66E-02	3.95E-01
**ALPK2**	-0.34	7.04E-01	1.00E+00	2.30	1.66E-02	3.95E-01
**AC139100.1**	-0.53	6.10E-01	1.00E+00	-1.69	1.67E-02	3.95E-01
**LCAT**	0.13	7.78E-01	1.00E+00	1.22	1.67E-02	3.95E-01
**PLAC8**	0.33	7.45E-01	1.00E+00	1.79	1.67E-02	3.95E-01
**P2RY2**	-0.43	2.78E-01	1.00E+00	1.28	1.68E-02	3.96E-01
**GLCCI1**	0.04	9.07E-01	1.00E+00	-0.86	1.68E-02	3.97E-01
**SMAD5**	0.24	2.89E-01	1.00E+00	-0.45	1.68E-02	3.97E-01
**KANSL1-AS1**	0.11	8.50E-01	1.00E+00	-1.16	1.69E-02	3.97E-01
**DSC2**	-0.54	6.10E-02	1.00E+00	0.95	1.69E-02	3.97E-01
**IVL**	-0.14	7.97E-01	1.00E+00	2.06	1.69E-02	3.97E-01
**LINC02298**	-0.25	6.14E-01	1.00E+00	-1.37	1.69E-02	3.97E-01
**FOXS1**	0.06	9.23E-01	1.00E+00	1.15	1.69E-02	3.97E-01
**CCER2**	-2.22	1.20E-02	6.79E-01	-2.12	1.69E-02	3.97E-01
**AP001160.3**	-1.15	4.45E-01	1.00E+00	-2.60	1.69E-02	3.97E-01
**SERPINH1**	-0.16	6.99E-01	1.00E+00	1.08	1.69E-02	3.97E-01
**HS3ST1**	0.03	9.58E-01	1.00E+00	1.09	1.70E-02	3.97E-01
**EIF2S2P3**	3.05	1.18E-01	1.00E+00	1.83	1.71E-02	3.99E-01
**FBLN7**	0.97	1.12E-03	1.75E-01	1.37	1.71E-02	3.99E-01
**TRBV9**	-1.62	3.86E-01	1.00E+00	2.36	1.72E-02	4.01E-01
**LAPTM5**	-0.17	4.62E-01	1.00E+00	1.29	1.72E-02	4.02E-01
**LNCAROD**	1.37	1.85E-01	1.00E+00	2.75	1.72E-02	4.02E-01
**AC005618.1**	-0.19	7.91E-01	1.00E+00	-2.09	1.73E-02	4.02E-01
**CYP1B1**	-0.22	6.36E-01	1.00E+00	0.78	1.73E-02	4.02E-01
**FN1**	-0.14	7.75E-01	1.00E+00	1.53	1.73E-02	4.02E-01

**ARHGAP9**	-0.73	2.53E-01	1.00E+00	1.16	1.73E-02	4.02E-01
**AC067945.1**	0.28	8.74E-01	1.00E+00	2.04	1.74E-02	4.02E-01
**MMP2**	0.31	5.71E-01	1.00E+00	1.46	1.74E-02	4.02E-01
**AC073335.2**	-0.82	4.09E-01	1.00E+00	2.03	1.74E-02	4.02E-01
**AL078590.2**	-0.36	8.13E-01	1.00E+00	2.14	1.74E-02	4.02E-01
**ARSA**	-0.26	4.75E-01	1.00E+00	1.01	1.74E-02	4.02E-01
**AC018523.1**	30.00	6.59E-18	5.54E-15	-1.78	1.75E-02	4.02E-01
**APOBEC3C**	-0.36	1.98E-01	1.00E+00	0.51	1.75E-02	4.02E-01
**REST**	0.26	1.34E-01	1.00E+00	-0.68	1.75E-02	4.03E-01
**ZBTB20-AS4**	1.16	3.59E-01	1.00E+00	-1.97	1.75E-02	4.03E-01
**FAM89A**	0.36	3.24E-01	1.00E+00	-1.47	1.75E-02	4.03E-01
**HLA-DPB2**	-1.03	7.43E-01	1.00E+00	1.88	1.76E-02	4.04E-01
**SLC38A7**	-0.19	4.48E-01	1.00E+00	0.73	1.76E-02	4.04E-01
**RP11-423O2.5**	-0.20	7.56E-01	1.00E+00	-0.96	1.76E-02	4.04E-01
**IGKV6-21**	2.81	8.25E-02	1.00E+00	2.70	1.76E-02	4.04E-01
**AC005831.1**	-0.92	6.60E-01	1.00E+00	-2.41	1.76E-02	4.04E-01
**KRT17**	-0.84	1.44E-01	1.00E+00	1.72	1.77E-02	4.04E-01
**KCNK2**	0.01	9.86E-01	1.00E+00	1.47	1.77E-02	4.04E-01
**AC104986.2**	0.18	7.61E-01	1.00E+00	-1.09	1.77E-02	4.04E-01
**TGFB2-AS1**	-0.57	3.33E-01	1.00E+00	1.26	1.78E-02	4.05E-01
**CD163**	-0.25	6.03E-01	1.00E+00	1.81	1.78E-02	4.05E-01
**STAC3**	-0.78	9.33E-02	1.00E+00	2.40	1.78E-02	4.05E-01
**AP001206.1**	-1.10	7.53E-01	1.00E+00	-3.88	1.78E-02	4.05E-01
**BISPR**	-0.92	1.78E-01	1.00E+00	1.46	1.79E-02	4.05E-01
**CHST11**	-0.46	5.46E-02	1.00E+00	2.03	1.79E-02	4.05E-01
**UBE2SP1**	-0.98	6.75E-01	1.00E+00	-2.61	1.79E-02	4.05E-01
**AL021155.2**	-3.54	1.09E-02	6.43E-01	-1.88	1.79E-02	4.05E-01
**ZNF697**	0.25	5.57E-01	1.00E+00	1.48	1.79E-02	4.06E-01
**TRIM21**	-0.13	7.98E-01	1.00E+00	0.90	1.80E-02	4.06E-01
**AL355994.2**	-2.69	4.91E-02	1.00E+00	-3.18	1.80E-02	4.06E-01
**DLG4**	-0.44	3.79E-01	1.00E+00	2.54	1.80E-02	4.06E-01
**MNX1**	-0.30	8.21E-01	1.00E+00	-3.27	1.81E-02	4.06E-01

**SMOX**	0.01	9.90E-01	1.00E+00	1.14	1.81E-02	4.06E-01
**AL157838.1**	0.19	8.62E-01	1.00E+00	2.61	1.81E-02	4.06E-01
**IGF2BP2**	-0.18	6.80E-01	1.00E+00	1.21	1.81E-02	4.06E-01
**PIGX**	0.08	7.13E-01	1.00E+00	-1.00	1.81E-02	4.06E-01
**PLA2G12AP1**	-0.63	6.66E-01	1.00E+00	-1.94	1.81E-02	4.06E-01
**ZNF420**	0.33	3.85E-01	1.00E+00	-0.97	1.81E-02	4.06E-01
**SNX12**	0.08	6.23E-01	1.00E+00	1.03	1.81E-02	4.06E-01
**ALDH3B1**	0.13	7.34E-01	1.00E+00	0.97	1.82E-02	4.07E-01
**KCNAB2**	-0.27	2.00E-01	1.00E+00	1.45	1.82E-02	4.08E-01
**AL451060.1**	-1.43	2.00E-01	1.00E+00	-1.34	1.83E-02	4.08E-01
**SDR42E2**	-2.94	6.71E-02	1.00E+00	-2.24	1.83E-02	4.08E-01
**PDE6A**	0.97	5.15E-01	1.00E+00	-2.35	1.83E-02	4.08E-01
**TGFBI**	-0.08	7.62E-01	1.00E+00	0.79	1.83E-02	4.08E-01
**AC128709.2**	-0.92	6.47E-01	1.00E+00	-2.73	1.83E-02	4.08E-01
**GRINA**	-0.09	7.14E-01	1.00E+00	1.07	1.83E-02	4.08E-01
**COL6A3**	-0.40	3.98E-01	1.00E+00	1.64	1.84E-02	4.08E-01
**AGPAT4**	0.10	7.97E-01	1.00E+00	1.08	1.84E-02	4.08E-01
**GPSM2**	0.00	9.91E-01	1.00E+00	-1.11	1.84E-02	4.08E-01
**SPG21**	0.17	3.17E-01	1.00E+00	-0.85	1.85E-02	4.08E-01
**IFIT3**	-0.40	6.13E-01	1.00E+00	1.61	1.85E-02	4.08E-01
**SHC1**	-0.01	9.68E-01	1.00E+00	0.81	1.85E-02	4.08E-01
**RHOJ**	0.09	8.18E-01	1.00E+00	0.92	1.85E-02	4.08E-01
**PPP1R14BP3**	0.25	6.44E-01	1.00E+00	-1.13	1.85E-02	4.09E-01
**PDK2**	0.07	7.85E-01	1.00E+00	-0.63	1.85E-02	4.09E-01
**TFPT**	0.07	7.54E-01	1.00E+00	0.69	1.86E-02	4.09E-01
**XLOC_000271**	-1.45	2.44E-01	1.00E+00	1.83	1.86E-02	4.09E-01
**EGFEM1P**	0.26	9.39E-01	1.00E+00	-4.49	1.87E-02	4.10E-01
**ECHS1**	0.30	4.41E-01	1.00E+00	-0.71	1.87E-02	4.10E-01
**SLC30A5**	0.32	2.32E-01	1.00E+00	-0.81	1.87E-02	4.10E-01
**TRMT12**	0.20	3.09E-01	1.00E+00	0.59	1.87E-02	4.10E-01
**NDUFB1**	-0.05	8.70E-01	1.00E+00	-1.05	1.87E-02	4.10E-01
**AC005014.2**	-1.69	6.19E-01	1.00E+00	-3.71	1.88E-02	4.10E-01

**MDFI**	0.30	6.36E-01	1.00E+00	1.81	1.88E-02	4.10E-01
**BTN3A3**	0.03	9.55E-01	1.00E+00	1.11	1.88E-02	4.10E-01
**NEFL**	-2.60	4.20E-01	1.00E+00	1.85	1.88E-02	4.10E-01
**SGIP1**	0.13	8.71E-01	1.00E+00	1.75	1.88E-02	4.10E-01
**NNMT**	0.30	4.21E-01	1.00E+00	1.26	1.88E-02	4.10E-01
**AC140479.7**	-0.27	9.38E-01	1.00E+00	3.92	1.88E-02	4.10E-01
**MBD1**	-0.04	8.74E-01	1.00E+00	0.58	1.89E-02	4.10E-01
**PPIAP22**	-0.25	2.81E-01	1.00E+00	-0.51	1.89E-02	4.11E-01
**CCR7**	-0.65	3.55E-01	1.00E+00	1.99	1.91E-02	4.15E-01
**ZNF394**	-0.05	8.32E-01	1.00E+00	0.30	1.91E-02	4.15E-01
**RNLS**	-0.07	7.17E-01	1.00E+00	-0.67	1.92E-02	4.16E-01
**NDUFAF3**	0.06	8.07E-01	1.00E+00	-0.70	1.92E-02	4.16E-01
**HSF2**	-0.26	3.59E-01	1.00E+00	-0.78	1.93E-02	4.17E-01
**FOXD4**	-1.89	3.43E-01	1.00E+00	3.59	1.93E-02	4.17E-01
**AC012236.1**	0.93	7.24E-01	1.00E+00	2.87	1.93E-02	4.17E-01
**DNAH8**	-3.77	2.35E-02	9.15E-01	-2.91	1.93E-02	4.17E-01
**AC104692.1**	-0.13	9.70E-01	1.00E+00	3.46	1.94E-02	4.18E-01
**CD53**	-0.05	9.09E-01	1.00E+00	1.41	1.94E-02	4.18E-01
**USF2**	-0.16	4.38E-01	1.00E+00	1.36	1.94E-02	4.18E-01
**CD72**	-1.88	1.16E-02	6.66E-01	2.04	1.95E-02	4.20E-01
**AL033397.2**	0.50	6.64E-01	1.00E+00	-2.31	1.96E-02	4.21E-01
**MRPL33**	0.24	3.57E-01	1.00E+00	-0.99	1.96E-02	4.21E-01
**MFGE8**	-0.10	8.11E-01	1.00E+00	0.93	1.97E-02	4.22E-01
**CLEC11A**	-0.27	5.15E-01	1.00E+00	0.78	1.97E-02	4.22E-01
**HFE**	0.14	6.78E-01	1.00E+00	0.86	1.98E-02	4.24E-01
**AC010809.1**	-2.19	2.48E-01	1.00E+00	2.27	1.98E-02	4.24E-01
**MYO1F**	-0.14	6.89E-01	1.00E+00	1.77	1.99E-02	4.24E-01
**AL359915.2**	0.20	6.91E-01	1.00E+00	0.74	1.99E-02	4.24E-01
**RAD9A**	-0.15	5.21E-01	1.00E+00	0.58	2.00E-02	4.24E-01
**SSH1**	0.18	2.47E-01	1.00E+00	1.16	2.00E-02	4.24E-01
**PIK3CD**	0.11	7.66E-01	1.00E+00	1.58	2.00E-02	4.24E-01
**AL392172.1**	-0.05	9.14E-01	1.00E+00	-0.90	2.00E-02	4.24E-01

**LUARIS**	0.43	9.01E-01	1.00E+00	4.19	2.01E-02	4.24E-01
**TRGV7**	-1.73	4.61E-01	1.00E+00	2.11	2.01E-02	4.24E-01
**THEMIS**	-0.73	3.47E-01	1.00E+00	2.14	2.01E-02	4.24E-01
**AC138951.1**	-2.70	8.37E-02	1.00E+00	-1.83	2.01E-02	4.24E-01
**WISP2**	1.37	1.29E-02	7.15E-01	1.26	2.01E-02	4.24E-01
**AC004706.3**	-0.49	4.96E-01	1.00E+00	4.07	2.01E-02	4.24E-01
**CAMTA1**	-0.20	3.99E-01	1.00E+00	-0.70	2.01E-02	4.24E-01
**UBXN11**	0.20	4.10E-01	1.00E+00	0.54	2.01E-02	4.24E-01
**AC069213.3**	NA	NA	NA	-2.06	2.01E-02	4.24E-01
**KCNJ10**	-4.26	8.52E-04	1.48E-01	3.17	2.01E-02	4.24E-01
**PTPRE**	-0.23	3.45E-01	1.00E+00	0.84	2.01E-02	4.24E-01
**RPL23AP58**	0.85	8.00E-01	1.00E+00	-3.47	2.02E-02	4.25E-01
**TNFSF13**	0.44	2.77E-01	1.00E+00	0.90	2.02E-02	4.25E-01
**LUCAT1**	0.69	2.88E-01	1.00E+00	1.61	2.02E-02	4.25E-01
**WDR45**	-0.08	7.10E-01	1.00E+00	0.38	2.02E-02	4.25E-01
**NRXN2**	0.14	8.59E-01	1.00E+00	1.98	2.03E-02	4.25E-01
**ADGRF5P1**	-2.90	2.91E-02	9.89E-01	-2.54	2.03E-02	4.26E-01
**XLOC_012579**	-0.18	8.46E-01	1.00E+00	1.57	2.03E-02	4.27E-01
**LTBP3**	0.23	5.55E-01	1.00E+00	1.04	2.04E-02	4.27E-01
**PEX7**	-0.01	9.83E-01	1.00E+00	-0.84	2.04E-02	4.27E-01
**ZBED5**	0.13	5.94E-01	1.00E+00	-0.47	2.05E-02	4.27E-01
**THOC7**	-0.13	5.05E-01	1.00E+00	-0.52	2.05E-02	4.27E-01
**XLOC_008417**	-0.44	7.48E-01	1.00E+00	-3.42	2.05E-02	4.27E-01
**MTRNR2L3**	1.36	6.93E-01	1.00E+00	2.99	2.05E-02	4.27E-01
**AC012181.1**	-2.44	2.02E-01	1.00E+00	2.16	2.05E-02	4.27E-01
**TRBV5-1**	-1.94	1.79E-01	1.00E+00	2.18	2.05E-02	4.27E-01
**ODF3B**	-0.15	8.06E-01	1.00E+00	0.91	2.06E-02	4.28E-01
**MGAT5**	0.03	9.19E-01	1.00E+00	1.31	2.06E-02	4.28E-01
**XLOC_008020**	-0.24	7.43E-01	1.00E+00	1.86	2.06E-02	4.28E-01
**LLGL1**	-0.18	4.51E-01	1.00E+00	1.75	2.06E-02	4.28E-01
**PTPRJ**	0.22	5.72E-01	1.00E+00	1.54	2.06E-02	4.28E-01
**AC007216.4**	-1.59	3.63E-01	1.00E+00	2.53	2.07E-02	4.28E-01

**AC103691.1**	0.25	8.96E-01	1.00E+00	-1.76	2.07E-02	4.28E-01
**EIF3CL**	-0.24	5.89E-01	1.00E+00	-0.61	2.07E-02	4.28E-01
**AREG**	-0.78	4.24E-01	1.00E+00	1.22	2.08E-02	4.28E-01
**RNF180**	-0.18	4.60E-01	1.00E+00	-0.86	2.08E-02	4.28E-01
**RSAD2**	-0.08	9.50E-01	1.00E+00	1.95	2.08E-02	4.28E-01
**IGKV3-7**	NA	NA	NA	5.46	2.08E-02	4.28E-01
**FMO5**	0.33	4.47E-01	1.00E+00	-0.93	2.08E-02	4.28E-01
**RPS19P3**	-0.11	9.58E-01	1.00E+00	-1.51	2.09E-02	4.28E-01
**G895**	0.34	6.37E-01	1.00E+00	2.31	2.09E-02	4.28E-01
**LCT**	-0.26	9.27E-01	1.00E+00	-3.38	2.09E-02	4.28E-01
**SCOC-AS1**	0.26	7.57E-01	1.00E+00	-1.14	2.09E-02	4.28E-01
**AC026412.1**	-0.53	1.08E-01	1.00E+00	-1.16	2.09E-02	4.29E-01
**PLCXD2**	0.66	1.29E-01	1.00E+00	1.66	2.10E-02	4.30E-01
**IGLV3-10**	30.00	3.93E-19	5.54E-15	3.17	2.10E-02	4.30E-01
**4-Mar**	-1.26	2.80E-01	1.00E+00	3.62	2.10E-02	4.30E-01
**NPC1**	0.32	1.93E-01	1.00E+00	0.64	2.10E-02	4.30E-01
**ATRNL1**	-0.47	6.62E-01	1.00E+00	-2.49	2.11E-02	4.30E-01
**MPV17L**	-0.24	6.40E-01	1.00E+00	-1.10	2.11E-02	4.31E-01
**KRT77**	0.32	4.45E-01	1.00E+00	-1.55	2.11E-02	4.31E-01
**G10734**	0.15	7.43E-01	1.00E+00	-1.81	2.12E-02	4.31E-01
**FHOD1**	0.01	9.84E-01	1.00E+00	1.33	2.12E-02	4.32E-01
**NDNF**	0.16	7.78E-01	1.00E+00	-1.50	2.12E-02	4.32E-01
**AP2S1**	-0.13	4.93E-01	1.00E+00	0.53	2.14E-02	4.35E-01
**CHCHD7**	0.05	8.15E-01	1.00E+00	-0.62	2.15E-02	4.36E-01
**RASA4DP**	-0.42	6.41E-01	1.00E+00	1.59	2.15E-02	4.36E-01
**C9orf139**	-2.16	1.10E-01	1.00E+00	1.55	2.15E-02	4.36E-01
**REEP6**	-0.22	5.46E-01	1.00E+00	-0.76	2.15E-02	4.36E-01
**FAM83A**	-0.87	6.23E-02	1.00E+00	2.01	2.15E-02	4.36E-01
**KPNA5**	0.31	2.47E-01	1.00E+00	-0.68	2.16E-02	4.36E-01
**IGLV4-60**	1.83	5.90E-01	1.00E+00	5.75	2.16E-02	4.36E-01
**SEPSECS-AS1**	0.68	2.32E-01	1.00E+00	-1.10	2.16E-02	4.36E-01
**TOR1B**	0.21	3.05E-01	1.00E+00	0.52	2.17E-02	4.36E-01

**ZNF618**	-0.48	6.66E-02	1.00E+00	1.15	2.17E-02	4.36E-01
**AK3**	0.14	4.72E-01	1.00E+00	-0.58	2.17E-02	4.36E-01
**GPR62**	-0.49	5.32E-01	1.00E+00	-2.21	2.17E-02	4.36E-01
**CSNK1G1**	0.03	8.91E-01	1.00E+00	0.60	2.17E-02	4.36E-01
**COL14A1**	0.03	9.53E-01	1.00E+00	1.43	2.17E-02	4.36E-01
**FAM120AOS**	0.17	3.61E-01	1.00E+00	-0.48	2.18E-02	4.36E-01
**CCZ1**	-0.43	1.72E-01	1.00E+00	-1.19	2.18E-02	4.36E-01
**RCHY1**	0.44	1.37E-01	1.00E+00	-0.81	2.18E-02	4.37E-01
**AL162726.3**	-0.16	9.62E-01	1.00E+00	2.36	2.18E-02	4.37E-01
**ACVRL1**	0.49	2.01E-01	1.00E+00	1.50	2.19E-02	4.38E-01
**GLIPR1**	-0.35	4.03E-01	1.00E+00	0.77	2.19E-02	4.38E-01
**KLK6**	-2.15	4.07E-01	1.00E+00	1.99	2.19E-02	4.38E-01
**AL035563.1**	-0.39	5.34E-01	1.00E+00	-1.43	2.20E-02	4.38E-01
**DLL3**	-0.82	7.37E-01	1.00E+00	-2.67	2.20E-02	4.38E-01
**CARNMT1**	-0.11	7.25E-01	1.00E+00	-0.65	2.20E-02	4.38E-01
**PEAK3**	1.41	8.44E-02	1.00E+00	2.21	2.20E-02	4.38E-01
**PBX3**	-0.08	7.56E-01	1.00E+00	-0.55	2.20E-02	4.38E-01
**CFHR5**	-0.80	8.18E-01	1.00E+00	4.42	2.21E-02	4.39E-01
**PTPRD**	-0.69	2.36E-01	1.00E+00	1.07	2.21E-02	4.39E-01
**LINC02580**	-0.03	9.70E-01	1.00E+00	-1.08	2.21E-02	4.39E-01
**MS4A6A**	0.25	6.41E-01	1.00E+00	0.96	2.22E-02	4.40E-01
**ZNF534**	-2.91	1.66E-01	1.00E+00	-2.99	2.22E-02	4.40E-01
**RETREG1**	0.35	3.38E-01	1.00E+00	-0.65	2.22E-02	4.40E-01
**LRRC25**	-0.10	8.76E-01	1.00E+00	1.53	2.23E-02	4.40E-01
**RHOH**	0.20	7.33E-01	1.00E+00	1.44	2.23E-02	4.40E-01
**METTL27**	0.14	8.51E-01	1.00E+00	1.16	2.23E-02	4.40E-01
**CCNH**	0.01	9.52E-01	1.00E+00	-0.83	2.23E-02	4.40E-01
**AC011595.1**	0.75	6.41E-01	1.00E+00	-2.64	2.23E-02	4.40E-01
**TRIM5**	-0.27	5.37E-01	1.00E+00	0.77	2.24E-02	4.40E-01
**COL4A1**	0.19	7.72E-01	1.00E+00	1.82	2.24E-02	4.41E-01
**EME2**	-0.21	6.54E-01	1.00E+00	1.49	2.24E-02	4.41E-01
**NEK10**	-0.18	8.85E-01	1.00E+00	-2.17	2.25E-02	4.41E-01

**PNRC1**	-0.39	2.09E-01	1.00E+00	-1.24	2.25E-02	4.41E-01
**ADAP2**	-0.23	5.27E-01	1.00E+00	0.85	2.25E-02	4.41E-01
**TRBC2**	-0.52	3.52E-01	1.00E+00	1.37	2.25E-02	4.41E-01
**TIPRL**	-0.06	7.82E-01	1.00E+00	-0.38	2.25E-02	4.41E-01
**SELPLG**	-0.25	5.49E-01	1.00E+00	1.23	2.25E-02	4.41E-01
**XLOC_001823**	-0.74	8.29E-01	1.00E+00	-2.57	2.25E-02	4.41E-01
**TRAV20**	-1.64	6.25E-01	1.00E+00	3.27	2.26E-02	4.41E-01
**RNASE6**	-0.30	5.21E-01	1.00E+00	0.83	2.26E-02	4.41E-01
**MXRA5**	-0.37	2.12E-01	1.00E+00	1.34	2.26E-02	4.42E-01
**CXCL8**	-2.83	6.89E-02	1.00E+00	2.13	2.27E-02	4.42E-01
**FAT1**	-0.37	2.42E-01	1.00E+00	1.03	2.27E-02	4.42E-01
**DECR1**	-0.02	9.44E-01	1.00E+00	-0.54	2.27E-02	4.42E-01
**CAV2**	0.26	4.83E-01	1.00E+00	-0.87	2.27E-02	4.42E-01
**MGAT3**	0.03	9.45E-01	1.00E+00	1.74	2.28E-02	4.42E-01
**NUTM2A-AS1**	-0.19	5.82E-01	1.00E+00	-0.55	2.28E-02	4.42E-01
**AC009299.2**	0.90	5.63E-01	1.00E+00	1.71	2.28E-02	4.42E-01
**FXYD5**	-0.33	2.16E-01	1.00E+00	0.77	2.28E-02	4.42E-01
**SIGLEC14**	-0.08	9.31E-01	1.00E+00	2.25	2.28E-02	4.43E-01
**SERTAD4**	-0.03	9.05E-01	1.00E+00	-0.80	2.29E-02	4.43E-01
**MX1**	-0.02	9.77E-01	1.00E+00	1.51	2.29E-02	4.43E-01
**SPHK1**	-0.29	8.39E-02	1.00E+00	1.40	2.29E-02	4.43E-01
**IGLV7-43**	-0.15	9.36E-01	1.00E+00	3.90	2.29E-02	4.43E-01
**TDRD5**	-0.14	8.98E-01	1.00E+00	-1.97	2.30E-02	4.43E-01
**SNX29P1**	1.74	4.08E-01	1.00E+00	2.56	2.30E-02	4.43E-01
**PROX1**	-0.16	7.37E-01	1.00E+00	1.20	2.30E-02	4.43E-01
**HIST2H3D**	-1.60	1.71E-01	1.00E+00	-2.69	2.30E-02	4.43E-01
**DCST2**	-0.09	8.73E-01	1.00E+00	1.21	2.30E-02	4.43E-01
**RLN2**	1.16	1.29E-01	1.00E+00	-1.73	2.31E-02	4.43E-01
**AC026310.2**	NA	NA	NA	4.91	2.31E-02	4.43E-01
**FBXO27**	0.37	3.12E-01	1.00E+00	-0.92	2.31E-02	4.43E-01
**ADGRB2**	-0.39	5.78E-01	1.00E+00	1.75	2.31E-02	4.43E-01
**POU6F2**	1.13	3.40E-01	1.00E+00	-2.40	2.31E-02	4.43E-01

**AL355309.1**	-2.56	4.53E-01	1.00E+00	-1.99	2.31E-02	4.43E-01
**SLC15A3**	0.16	7.44E-01	1.00E+00	2.05	2.32E-02	4.43E-01
**AC073569.1**	0.67	7.10E-01	1.00E+00	2.05	2.32E-02	4.43E-01
**MDH1**	0.04	7.99E-01	1.00E+00	-0.85	2.32E-02	4.43E-01
**AL359258.3**	-1.28	5.21E-01	1.00E+00	-2.28	2.32E-02	4.43E-01
**AC090587.1**	-1.81	2.51E-01	1.00E+00	-1.56	2.32E-02	4.43E-01
**AC098820.1**	-0.85	8.05E-01	1.00E+00	-2.87	2.33E-02	4.44E-01
**ALOX15B**	-0.14	7.38E-01	1.00E+00	-1.43	2.33E-02	4.44E-01
**ACP6**	-0.20	5.94E-01	1.00E+00	-1.00	2.34E-02	4.45E-01
**LRRC1**	-0.01	9.79E-01	1.00E+00	-0.83	2.34E-02	4.45E-01
**GGT1**	0.48	4.55E-01	1.00E+00	1.10	2.35E-02	4.45E-01
**NT5E**	0.38	3.69E-01	1.00E+00	1.06	2.35E-02	4.45E-01
**GCSHP5**	-0.46	6.19E-01	1.00E+00	-1.24	2.35E-02	4.45E-01
**ACOT11**	-0.74	1.07E-01	1.00E+00	1.62	2.35E-02	4.45E-01
**ACRV1**	-1.95	2.18E-01	1.00E+00	1.94	2.35E-02	4.45E-01
**NLRP7**	0.90	7.96E-01	1.00E+00	3.48	2.35E-02	4.45E-01
**HMGB1P51**	0.56	8.71E-01	1.00E+00	2.60	2.36E-02	4.45E-01
**CHMP4B**	-0.11	6.21E-01	1.00E+00	1.69	2.36E-02	4.45E-01
**AC023509.4**	0.83	4.10E-01	1.00E+00	-1.00	2.36E-02	4.45E-01
**PILRA**	-0.98	4.69E-02	1.00E+00	1.59	2.36E-02	4.46E-01
**FIGNL2**	0.21	7.88E-01	1.00E+00	3.15	2.37E-02	4.47E-01
**IGHJ5**	1.29	7.08E-01	1.00E+00	3.38	2.37E-02	4.47E-01
**LINC00237**	-1.87	2.37E-01	1.00E+00	-2.80	2.38E-02	4.47E-01
**CR392039.2**	1.86	3.59E-02	1.00E+00	-2.20	2.38E-02	4.47E-01
**AL359397.1**	-1.86	5.88E-01	1.00E+00	-2.43	2.38E-02	4.47E-01
**G42437**	1.80	6.00E-01	1.00E+00	-3.60	2.39E-02	4.47E-01
**TNFAIP6**	0.14	8.06E-01	1.00E+00	1.25	2.39E-02	4.47E-01
**JOSD2**	-0.20	4.79E-01	1.00E+00	0.57	2.39E-02	4.47E-01
**AL049597.2**	0.20	7.37E-01	1.00E+00	-1.04	2.39E-02	4.47E-01
**KLHL31**	2.28	2.25E-02	9.03E-01	-1.75	2.39E-02	4.47E-01
**SHISA5**	0.17	6.44E-01	1.00E+00	0.64	2.39E-02	4.47E-01
**LINC00239**	-0.17	8.38E-01	1.00E+00	-1.60	2.39E-02	4.47E-01

**CRLS1**	0.24	2.03E-01	1.00E+00	-1.50	2.39E-02	4.47E-01
**ROCR**	-0.99	3.51E-01	1.00E+00	-1.66	2.40E-02	4.47E-01
**TRAV29DV5**	0.59	7.33E-01	1.00E+00	2.09	2.40E-02	4.47E-01
**HCK**	-0.52	2.45E-01	1.00E+00	1.26	2.40E-02	4.47E-01
**HDDC2**	0.00	9.91E-01	1.00E+00	-0.49	2.40E-02	4.47E-01
**CDH11**	-0.24	5.26E-01	1.00E+00	1.02	2.40E-02	4.47E-01
**SUCLG1**	0.06	6.58E-01	1.00E+00	-0.41	2.40E-02	4.47E-01
**ADAMTS7**	-0.10	8.80E-01	1.00E+00	1.93	2.41E-02	4.47E-01
**AL139147.1**	0.92	7.41E-01	1.00E+00	4.03	2.41E-02	4.48E-01
**XLOC_011313**	-0.35	5.52E-01	1.00E+00	1.33	2.42E-02	4.48E-01
**MT-TC**	0.91	6.67E-01	1.00E+00	-1.74	2.42E-02	4.48E-01
**SLC24A2**	2.19	5.12E-01	1.00E+00	2.74	2.42E-02	4.49E-01
**FAM96AP2**	-0.98	2.76E-01	1.00E+00	1.70	2.43E-02	4.49E-01
**SYN2**	0.01	9.90E-01	1.00E+00	-1.50	2.43E-02	4.49E-01
**TNFRSF17**	1.63	5.20E-01	1.00E+00	3.08	2.43E-02	4.49E-01
**AC098828.1**	-1.10	7.53E-01	1.00E+00	-2.62	2.43E-02	4.49E-01
**ANTXR1**	0.04	8.72E-01	1.00E+00	0.92	2.43E-02	4.49E-01
**OSER1-DT**	-0.14	6.44E-01	1.00E+00	-0.85	2.44E-02	4.49E-01
**HSP90AB4P**	0.30	8.86E-01	1.00E+00	1.92	2.44E-02	4.49E-01
**FAM78A**	0.15	7.73E-01	1.00E+00	2.18	2.45E-02	4.51E-01
**PTGDS**	-1.98	8.16E-02	1.00E+00	1.26	2.45E-02	4.51E-01
**STAT1**	-0.06	9.06E-01	1.00E+00	1.23	2.46E-02	4.51E-01
**PNRC2P1**	-2.31	2.42E-01	1.00E+00	-1.06	2.46E-02	4.51E-01
**UBA7**	0.14	7.63E-01	1.00E+00	1.12	2.46E-02	4.51E-01
**HLTF**	0.26	4.72E-01	1.00E+00	-0.60	2.46E-02	4.51E-01
**ELOVL5**	0.98	1.12E-01	1.00E+00	-1.34	2.46E-02	4.51E-01
**FOXN3**	0.06	7.81E-01	1.00E+00	-0.54	2.46E-02	4.51E-01
**AEBP1**	0.24	6.54E-01	1.00E+00	1.55	2.47E-02	4.52E-01
**FBXL22**	-0.45	3.40E-01	1.00E+00	-1.11	2.47E-02	4.52E-01
**SDHD**	0.03	9.05E-01	1.00E+00	-1.08	2.47E-02	4.52E-01
**TBXAS1**	-0.10	8.49E-01	1.00E+00	1.29	2.48E-02	4.52E-01
**AC097059.2**	1.15	6.28E-01	1.00E+00	3.62	2.48E-02	4.52E-01

**SNX20**	-0.70	3.10E-01	1.00E+00	1.73	2.48E-02	4.52E-01
**GABRB1**	-0.82	6.71E-01	1.00E+00	-2.87	2.48E-02	4.52E-01
**INKA2-AS1**	-2.86	4.00E-01	1.00E+00	-2.65	2.49E-02	4.52E-01
**PNP**	-0.86	7.93E-02	1.00E+00	0.73	2.49E-02	4.52E-01
**AC116651.1**	-1.04	7.46E-01	1.00E+00	-2.68	2.49E-02	4.52E-01
**CLCF1**	-1.25	2.88E-02	9.88E-01	1.49	2.49E-02	4.52E-01
**DIO2**	-0.28	4.78E-01	1.00E+00	1.19	2.49E-02	4.52E-01
**NID1**	0.47	3.02E-01	1.00E+00	1.32	2.49E-02	4.52E-01
**HAVCR2**	0.49	3.81E-01	1.00E+00	1.38	2.50E-02	4.52E-01
**PKD1P1**	-0.50	2.20E-01	1.00E+00	2.01	2.50E-02	4.52E-01
**PRRT3-AS1**	1.67	1.41E-01	1.00E+00	-1.52	2.50E-02	4.52E-01
**CCDC58**	0.00	9.93E-01	1.00E+00	-1.16	2.50E-02	4.52E-01
**AC011005.1**	-0.30	8.40E-01	1.00E+00	-1.31	2.51E-02	4.54E-01
**TBC1D16**	0.36	4.11E-01	1.00E+00	0.96	2.52E-02	4.54E-01
**TRAV13-2**	1.38	5.93E-01	1.00E+00	2.81	2.52E-02	4.54E-01
**TNFSF10**	-0.09	8.42E-01	1.00E+00	0.79	2.52E-02	4.54E-01
**1-Mar**	-0.06	8.92E-01	1.00E+00	1.16	2.53E-02	4.54E-01
**G39123**	1.57	6.35E-01	1.00E+00	-2.41	2.53E-02	4.54E-01
**PLPPR4**	-0.32	4.80E-01	1.00E+00	0.91	2.53E-02	4.54E-01
**AC231533.2**	-1.37	6.93E-01	1.00E+00	-1.59	2.53E-02	4.54E-01
**AC104758.2**	-0.50	7.47E-01	1.00E+00	2.59	2.53E-02	4.54E-01
**USP3-AS1**	-0.64	6.85E-01	1.00E+00	-1.95	2.53E-02	4.55E-01
**AC020763.3**	-2.46	4.32E-02	1.00E+00	2.14	2.54E-02	4.55E-01
**JTB**	-0.24	6.39E-01	1.00E+00	-1.38	2.54E-02	4.55E-01
**AP003390.1**	0.26	6.45E-01	1.00E+00	1.45	2.54E-02	4.55E-01
**STX5**	-0.04	7.57E-01	1.00E+00	0.34	2.54E-02	4.55E-01
**AC211433.1**	1.29	7.02E-01	1.00E+00	2.26	2.54E-02	4.55E-01
**ZNF529-AS1**	0.12	8.29E-01	1.00E+00	-0.93	2.54E-02	4.55E-01
**TRAV12-2**	-2.36	1.69E-01	1.00E+00	2.40	2.55E-02	4.55E-01
**IL15RA**	0.34	3.55E-01	1.00E+00	0.82	2.55E-02	4.55E-01
**AC009078.3**	2.08	7.70E-02	1.00E+00	-3.08	2.55E-02	4.55E-01
**PSMB10**	-0.52	1.09E-01	1.00E+00	0.77	2.55E-02	4.55E-01

**XLOC_006336**	-0.69	4.24E-01	1.00E+00	1.33	2.56E-02	4.55E-01
**IFI35**	0.14	7.81E-01	1.00E+00	1.03	2.56E-02	4.55E-01
**PIK3AP1**	0.44	4.54E-01	1.00E+00	1.65	2.56E-02	4.55E-01
**TRAV21**	0.60	6.17E-01	1.00E+00	2.05	2.56E-02	4.55E-01
**ZSCAN4**	1.16	3.36E-01	1.00E+00	-3.48	2.56E-02	4.55E-01
**AP001972.5**	1.03	4.40E-02	1.00E+00	0.82	2.57E-02	4.56E-01
**HEPH**	0.29	5.02E-01	1.00E+00	1.23	2.57E-02	4.56E-01
**MEGF8**	0.08	8.68E-01	1.00E+00	1.45	2.57E-02	4.56E-01
**NCALD**	0.50	5.10E-01	1.00E+00	-0.91	2.58E-02	4.56E-01
**PABPC4**	-0.05	8.21E-01	1.00E+00	0.85	2.58E-02	4.56E-01
**GIPC2**	0.24	4.90E-01	1.00E+00	-0.88	2.58E-02	4.56E-01
**CMIP**	-0.32	2.13E-01	1.00E+00	1.70	2.58E-02	4.56E-01
**XLOC_006166**	-1.18	5.97E-01	1.00E+00	-2.02	2.58E-02	4.56E-01
**MTND5P2**	NA	NA	NA	2.79	2.59E-02	4.57E-01
**MTURN**	0.35	5.05E-01	1.00E+00	-0.94	2.59E-02	4.57E-01
**MYO15B**	-0.21	6.52E-01	1.00E+00	1.87	2.59E-02	4.57E-01
**CEMIP2**	0.32	4.47E-01	1.00E+00	1.32	2.60E-02	4.57E-01
**PEX3**	-0.26	4.05E-01	1.00E+00	-0.73	2.60E-02	4.58E-01
**FGD3**	-0.59	1.22E-01	1.00E+00	1.22	2.60E-02	4.58E-01
**UBE2E2**	-0.09	7.16E-01	1.00E+00	-0.58	2.61E-02	4.58E-01
**GRAMD1B**	-0.73	2.50E-01	1.00E+00	2.51	2.61E-02	4.58E-01
**MAL2**	0.28	5.27E-01	1.00E+00	-1.11	2.61E-02	4.58E-01
**PLTP**	0.20	6.17E-01	1.00E+00	0.88	2.62E-02	4.58E-01
**CADM2**	-0.36	4.89E-01	1.00E+00	-1.41	2.62E-02	4.58E-01
**AL049838.1**	-0.86	2.30E-01	1.00E+00	-1.55	2.62E-02	4.58E-01
**SEC14L4**	1.49	2.18E-01	1.00E+00	-2.50	2.62E-02	4.58E-01
**SIDT2**	0.13	5.45E-01	1.00E+00	0.84	2.63E-02	4.58E-01
**CCDC154**	-2.55	1.38E-01	1.00E+00	3.90	2.63E-02	4.58E-01
**ALDOC**	0.33	3.86E-01	1.00E+00	-0.90	2.63E-02	4.58E-01
**HAGHL**	-0.60	2.24E-01	1.00E+00	1.41	2.63E-02	4.58E-01
**AC053503.2**	-1.53	6.53E-01	1.00E+00	3.49	2.63E-02	4.58E-01
**CCDC122**	-0.42	2.99E-01	1.00E+00	-0.66	2.63E-02	4.58E-01

**CD74**	-0.56	7.31E-02	1.00E+00	0.80	2.64E-02	4.59E-01
**LIPT1**	0.10	7.64E-01	1.00E+00	-0.90	2.64E-02	4.59E-01
**CSNK1E**	0.10	6.94E-01	1.00E+00	0.51	2.64E-02	4.59E-01
**ADGRE2**	-0.09	8.65E-01	1.00E+00	1.27	2.65E-02	4.60E-01
**SNHG9**	0.56	5.11E-01	1.00E+00	-1.32	2.65E-02	4.60E-01
**RABL3**	0.11	5.97E-01	1.00E+00	-0.63	2.66E-02	4.60E-01
**LINC01607**	-0.14	8.98E-01	1.00E+00	-1.90	2.66E-02	4.60E-01
**UBE2E3**	-0.02	9.28E-01	1.00E+00	-0.60	2.66E-02	4.60E-01
**AC016727.1**	-0.20	6.88E-01	1.00E+00	-0.90	2.66E-02	4.60E-01
**AL359317.1**	-2.57	1.68E-01	1.00E+00	-2.18	2.67E-02	4.61E-01
**SMAD1**	0.03	8.94E-01	1.00E+00	-0.49	2.67E-02	4.61E-01
**XLOC_013276**	0.25	7.47E-01	1.00E+00	-1.81	2.67E-02	4.61E-01
**EVL**	-0.36	1.54E-01	1.00E+00	1.43	2.67E-02	4.61E-01
**AC243829.4**	-2.94	3.87E-01	1.00E+00	3.44	2.67E-02	4.61E-01
**MYBPHL**	-0.14	9.68E-01	1.00E+00	2.78	2.68E-02	4.62E-01
**ZCCHC2**	-0.10	7.99E-01	1.00E+00	1.00	2.69E-02	4.62E-01
**PAX1**	0.09	9.12E-01	1.00E+00	2.91	2.69E-02	4.62E-01
**USP18**	0.02	9.77E-01	1.00E+00	1.34	2.69E-02	4.63E-01
**RALGPS1**	-0.10	6.06E-01	1.00E+00	-0.80	2.71E-02	4.65E-01
**AC104083.1**	-0.47	3.74E-01	1.00E+00	1.03	2.71E-02	4.65E-01
**CSMD2**	-1.02	2.13E-01	1.00E+00	1.69	2.71E-02	4.65E-01
**IKBKG**	0.05	8.05E-01	1.00E+00	0.49	2.72E-02	4.65E-01
**AC004687.1**	1.00	6.32E-01	1.00E+00	3.95	2.72E-02	4.65E-01
**RIN3**	-0.37	2.19E-01	1.00E+00	1.33	2.72E-02	4.65E-01
**RUNX1**	-0.21	6.28E-01	1.00E+00	1.38	2.72E-02	4.65E-01
**VAMP3**	-0.13	4.21E-01	1.00E+00	-0.43	2.72E-02	4.65E-01
**ZNF204P**	0.38	2.86E-01	1.00E+00	-1.02	2.72E-02	4.65E-01
**LY86**	-1.10	1.35E-02	7.28E-01	1.06	2.73E-02	4.67E-01
**AC104692.2**	NA	NA	NA	4.27	2.74E-02	4.67E-01
**AC068831.1**	0.42	8.50E-01	1.00E+00	2.43	2.74E-02	4.67E-01
**SEZ6L2**	-1.80	1.43E-05	4.42E-03	1.46	2.75E-02	4.68E-01
**MASTL**	-0.45	1.62E-01	1.00E+00	0.78	2.75E-02	4.68E-01

**TPRG1-AS1**	1.88	5.87E-02	1.00E+00	-1.55	2.76E-02	4.69E-01
**KIF3B**	-0.01	9.69E-01	1.00E+00	0.63	2.76E-02	4.69E-01
**SCGB1D2**	-0.40	7.74E-01	1.00E+00	-2.04	2.76E-02	4.69E-01
**ANKRD36BP2**	1.14	1.15E-01	1.00E+00	-2.40	2.77E-02	4.70E-01
**UBXN6**	0.05	8.36E-01	1.00E+00	1.02	2.77E-02	4.70E-01
**FCN1**	0.93	1.21E-01	1.00E+00	1.65	2.77E-02	4.70E-01
**CKLF**	-0.28	9.34E-01	1.00E+00	-1.84	2.78E-02	4.70E-01
**ELFN1**	-1.26	3.11E-05	8.71E-03	2.20	2.78E-02	4.70E-01
**LCMT2**	-0.06	7.74E-01	1.00E+00	0.56	2.78E-02	4.70E-01
**AC078883.2**	-2.59	4.27E-01	1.00E+00	-2.50	2.78E-02	4.70E-01
**COL6A1**	-0.14	7.67E-01	1.00E+00	1.58	2.78E-02	4.70E-01
**ULBP2**	-1.25	3.45E-01	1.00E+00	1.70	2.79E-02	4.70E-01
**GDNF**	-0.63	3.16E-01	1.00E+00	1.79	2.79E-02	4.70E-01
**HSD17B13**	2.25	7.59E-04	1.35E-01	-1.86	2.79E-02	4.70E-01
**FAM83A-AS1**	-0.93	3.77E-01	1.00E+00	2.51	2.79E-02	4.70E-01
**COL11A1**	0.43	7.53E-01	1.00E+00	1.91	2.79E-02	4.70E-01
**AC109826.1**	2.58	1.32E-01	1.00E+00	2.55	2.79E-02	4.70E-01
**XLOC_012881**	-0.31	8.92E-01	1.00E+00	2.59	2.79E-02	4.70E-01
**AL591806.1**	-0.70	5.06E-01	1.00E+00	-2.32	2.80E-02	4.70E-01
**TLR4**	0.57	2.00E-01	1.00E+00	1.22	2.80E-02	4.70E-01
**AL137802.2**	1.31	7.05E-01	1.00E+00	-2.65	2.80E-02	4.70E-01
**C2**	0.00	9.95E-01	1.00E+00	1.39	2.80E-02	4.70E-01
**ST20**	-2.19	5.19E-01	1.00E+00	-1.38	2.80E-02	4.70E-01
**PPID**	0.25	3.85E-01	1.00E+00	-0.43	2.80E-02	4.70E-01
**CBLB**	0.54	1.04E-01	1.00E+00	0.80	2.81E-02	4.70E-01
**ECHDC3**	-0.25	5.12E-01	1.00E+00	-1.45	2.81E-02	4.70E-01
**ATP1B3**	-0.35	6.93E-02	1.00E+00	-0.67	2.81E-02	4.70E-01
**DUS4L**	-0.11	7.25E-01	1.00E+00	-0.54	2.82E-02	4.70E-01
**OAT**	-0.07	6.99E-01	1.00E+00	-0.47	2.82E-02	4.71E-01
**RGCC**	0.48	3.16E-01	1.00E+00	-1.45	2.82E-02	4.71E-01
**TRAM2**	0.25	3.82E-01	1.00E+00	1.00	2.82E-02	4.71E-01
**CETN2**	0.08	7.10E-01	1.00E+00	-0.43	2.83E-02	4.71E-01

**AP002360.1**	0.19	6.52E-01	1.00E+00	-0.82	2.83E-02	4.71E-01
**GOLT1A**	-0.51	6.02E-01	1.00E+00	-2.22	2.83E-02	4.71E-01
**CD5L**	0.59	8.51E-01	1.00E+00	4.16	2.83E-02	4.71E-01
**XLOC_009145**	0.11	7.86E-01	1.00E+00	-0.67	2.84E-02	4.71E-01
**CALCA**	-0.87	8.02E-01	1.00E+00	3.97	2.84E-02	4.71E-01
**CR381653.1**	-0.01	9.84E-01	1.00E+00	-0.87	2.84E-02	4.71E-01
**MAFK**	-0.36	1.31E-01	1.00E+00	2.14	2.84E-02	4.71E-01
**SLC45A3**	-0.17	6.36E-01	1.00E+00	1.22	2.85E-02	4.72E-01
**NFS1**	0.03	9.12E-01	1.00E+00	-0.73	2.85E-02	4.72E-01
**MMP10**	-1.37	6.93E-01	1.00E+00	3.68	2.86E-02	4.74E-01
**AL591485.1**	-0.16	6.37E-01	1.00E+00	-1.11	2.87E-02	4.74E-01
**NMNAT3**	0.46	1.93E-01	1.00E+00	-0.73	2.87E-02	4.74E-01
**ZNF804A**	-1.73	2.83E-01	1.00E+00	3.17	2.87E-02	4.74E-01
**PLEKHG7**	-2.19	5.23E-01	1.00E+00	2.89	2.87E-02	4.74E-01
**PREX1**	-0.01	9.84E-01	1.00E+00	1.63	2.88E-02	4.74E-01
**NOA1**	-0.32	1.68E-01	1.00E+00	0.42	2.88E-02	4.74E-01
**GALNS**	-0.11	7.52E-01	1.00E+00	0.92	2.88E-02	4.74E-01
**DDC**	-1.74	2.19E-01	1.00E+00	-3.85	2.88E-02	4.74E-01
**FAM241A**	0.03	9.17E-01	1.00E+00	-1.21	2.89E-02	4.74E-01
**FUZ**	0.11	6.94E-01	1.00E+00	0.55	2.89E-02	4.74E-01
**PRAM1**	-0.80	3.58E-01	1.00E+00	2.53	2.89E-02	4.74E-01
**ABCG4**	-1.63	8.44E-02	1.00E+00	2.86	2.89E-02	4.74E-01
**RTN4IP1**	-0.11	6.86E-01	1.00E+00	-0.60	2.90E-02	4.74E-01
**AC084809.2**	1.86	2.50E-01	1.00E+00	1.78	2.90E-02	4.74E-01
**EGR2**	-0.32	5.61E-01	1.00E+00	0.99	2.90E-02	4.74E-01
**RTN4R**	-0.30	3.65E-01	1.00E+00	-1.15	2.90E-02	4.74E-01
**IFI16**	-0.34	3.85E-01	1.00E+00	0.77	2.90E-02	4.74E-01
**G6339**	0.48	6.77E-01	1.00E+00	-2.22	2.90E-02	4.74E-01
**ARTN**	-0.93	2.29E-01	1.00E+00	2.61	2.90E-02	4.74E-01
**AL358852.1**	-2.84	7.88E-02	1.00E+00	-1.39	2.90E-02	4.74E-01
**LINC00906**	3.96	3.42E-02	1.00E+00	3.26	2.91E-02	4.75E-01
**CR769775.1**	-0.97	2.17E-01	1.00E+00	-1.37	2.91E-02	4.75E-01

**USF1**	0.14	5.90E-01	1.00E+00	2.04	2.92E-02	4.75E-01
**GAPT**	1.06	2.01E-01	1.00E+00	1.16	2.92E-02	4.75E-01
**AL049637.1**	1.18	7.26E-01	1.00E+00	-3.47	2.92E-02	4.75E-01
**ATP5ME**	0.31	3.36E-01	1.00E+00	-1.50	2.92E-02	4.75E-01
**IFI6**	0.29	7.87E-01	1.00E+00	1.68	2.93E-02	4.75E-01
**RAB17**	0.04	9.55E-01	1.00E+00	-1.12	2.93E-02	4.76E-01
**CD93**	0.13	8.13E-01	1.00E+00	1.39	2.93E-02	4.76E-01
**SH3D21**	-0.64	2.91E-02	9.89E-01	1.45	2.94E-02	4.77E-01
**JOSD1**	-0.15	4.17E-01	1.00E+00	1.06	2.94E-02	4.77E-01
**MPC1**	0.02	9.45E-01	1.00E+00	-0.67	2.94E-02	4.77E-01
**CD44-AS1**	-0.87	6.95E-01	1.00E+00	1.73	2.95E-02	4.77E-01
**AC092135.3**	2.28	1.19E-01	1.00E+00	3.39	2.96E-02	4.78E-01
**MIR3687**	-0.33	5.73E-01	1.00E+00	-3.33	2.96E-02	4.78E-01
**AC135178.1**	-0.83	4.10E-01	1.00E+00	2.01	2.96E-02	4.78E-01
**YBX3P1**	0.15	9.35E-01	1.00E+00	-1.71	2.96E-02	4.78E-01
**SUGT1P2**	-0.80	8.18E-01	1.00E+00	2.74	2.96E-02	4.78E-01
**PXDN**	0.79	2.43E-01	1.00E+00	1.42	2.97E-02	4.79E-01
**HEXA-AS1**	-1.25	1.41E-01	1.00E+00	-1.78	2.97E-02	4.79E-01
**TAF11**	0.38	2.01E-01	1.00E+00	0.30	2.98E-02	4.79E-01
**CDK5R1**	-1.17	1.91E-03	2.39E-01	1.66	2.98E-02	4.79E-01
**CATSPER1**	-0.45	6.17E-01	1.00E+00	1.38	2.98E-02	4.79E-01
**FAM184B**	-0.42	5.20E-01	1.00E+00	-1.37	2.98E-02	4.79E-01
**AC080080.1**	-0.43	5.52E-01	1.00E+00	-2.01	2.98E-02	4.79E-01
**DDX39B**	0.24	6.69E-01	1.00E+00	1.24	2.99E-02	4.79E-01
**CCSER1**	-0.75	1.12E-01	1.00E+00	-1.01	2.99E-02	4.79E-01
**NFYB**	0.32	2.59E-01	1.00E+00	-0.47	2.99E-02	4.79E-01
**SUN1**	-0.04	8.23E-01	1.00E+00	-0.35	2.99E-02	4.79E-01
**ORAI2**	0.03	9.42E-01	1.00E+00	1.37	3.00E-02	4.80E-01
**ZNF92**	-0.07	8.62E-01	1.00E+00	-0.94	3.00E-02	4.80E-01
**LINC02487**	0.58	7.24E-01	1.00E+00	2.83	3.01E-02	4.80E-01
**CCL3L1**	0.49	8.88E-01	1.00E+00	2.14	3.01E-02	4.82E-01
**IQSEC2**	-0.49	6.77E-02	1.00E+00	0.95	3.02E-02	4.83E-01

**NALCN**	-0.73	2.94E-01	1.00E+00	-1.69	3.03E-02	4.83E-01
**SYK**	0.11	6.97E-01	1.00E+00	0.80	3.03E-02	4.83E-01
**TRANK1**	-0.29	6.69E-01	1.00E+00	1.60	3.04E-02	4.84E-01
**AC130371.2**	-0.03	9.53E-01	1.00E+00	1.93	3.04E-02	4.84E-01
**MAPK6**	-0.11	7.76E-01	1.00E+00	0.56	3.04E-02	4.84E-01
**ATAD1**	0.21	3.30E-01	1.00E+00	-0.41	3.04E-02	4.84E-01
**HMOX1**	-0.34	5.56E-01	1.00E+00	1.16	3.04E-02	4.84E-01
**AL121820.2**	-1.77	6.24E-02	1.00E+00	-1.28	3.05E-02	4.84E-01
**AL050327.1**	2.74	4.20E-01	1.00E+00	-3.10	3.05E-02	4.84E-01
**G4615**	-14.72	1.70E-05	5.09E-03	-1.86	3.06E-02	4.84E-01
**AC017104.5**	1.03	4.54E-01	1.00E+00	-1.97	3.06E-02	4.84E-01
**TMEM68**	0.24	4.59E-01	1.00E+00	-0.76	3.06E-02	4.84E-01
**TRDV2**	-0.67	8.47E-01	1.00E+00	3.91	3.06E-02	4.84E-01
**ZC3H7B**	0.03	8.95E-01	1.00E+00	1.83	3.07E-02	4.86E-01
**BNC2-AS1**	-2.19	9.60E-03	6.08E-01	-1.43	3.07E-02	4.86E-01
**XLOC_008233**	-0.23	8.17E-01	1.00E+00	-1.16	3.08E-02	4.86E-01
**FAM210B**	-0.07	6.83E-01	1.00E+00	-0.88	3.08E-02	4.86E-01
**KIAA1024**	-0.06	8.97E-01	1.00E+00	2.10	3.09E-02	4.86E-01
**LILRB3**	-0.59	4.79E-01	1.00E+00	1.97	3.09E-02	4.86E-01
**PTPA**	-0.12	5.62E-01	1.00E+00	0.79	3.09E-02	4.86E-01
**XLOC_003146**	-1.80	6.02E-01	1.00E+00	3.73	3.09E-02	4.86E-01
**AL158825.2**	-1.05	3.19E-01	1.00E+00	1.43	3.09E-02	4.86E-01
**RFX1**	-0.27	3.88E-01	1.00E+00	1.37	3.09E-02	4.86E-01
**TMC8**	-0.95	8.19E-02	1.00E+00	1.51	3.09E-02	4.86E-01
**TGDS**	0.36	1.88E-01	1.00E+00	-0.71	3.10E-02	4.86E-01
**GBGT1**	0.18	7.62E-01	1.00E+00	1.67	3.10E-02	4.86E-01
**IFI27**	0.14	8.41E-01	1.00E+00	1.25	3.10E-02	4.86E-01
**RIPOR1**	-0.08	8.19E-01	1.00E+00	1.23	3.10E-02	4.86E-01
**G33524**	-1.32	4.53E-01	1.00E+00	2.13	3.10E-02	4.86E-01
**PVT1**	-0.95	6.32E-02	1.00E+00	1.13	3.11E-02	4.86E-01
**AL391421.1**	0.12	9.09E-01	1.00E+00	2.26	3.11E-02	4.86E-01
**TMEM256**	0.15	8.24E-01	1.00E+00	-1.39	3.11E-02	4.86E-01

**PPARG**	2.13	1.16E-02	6.66E-01	-1.44	3.11E-02	4.86E-01
**SLC35C2**	-0.17	2.92E-01	1.00E+00	0.23	3.12E-02	4.87E-01
**CMKLR1**	0.31	4.55E-01	1.00E+00	1.34	3.13E-02	4.88E-01
**ICAM3**	-0.84	3.84E-01	1.00E+00	1.21	3.13E-02	4.88E-01
**NRROS**	0.12	8.29E-01	1.00E+00	1.92	3.13E-02	4.88E-01
**HESX1**	-1.43	6.67E-02	1.00E+00	1.15	3.13E-02	4.88E-01
**AP1S1**	-0.22	5.12E-01	1.00E+00	0.90	3.13E-02	4.88E-01
**ACOT7**	-0.48	1.15E-01	1.00E+00	0.67	3.14E-02	4.89E-01
**PRR3**	-0.02	9.60E-01	1.00E+00	0.78	3.15E-02	4.89E-01
**AC024361.2**	0.94	5.79E-01	1.00E+00	-2.44	3.15E-02	4.90E-01
**AL031123.1**	-1.61	5.10E-02	1.00E+00	-1.37	3.15E-02	4.90E-01
**GJB6**	-0.74	6.17E-02	1.00E+00	1.51	3.16E-02	4.90E-01
**TMEM176B**	0.14	6.95E-01	1.00E+00	1.12	3.16E-02	4.90E-01
**RNF139-AS1**	-2.29	3.65E-02	1.00E+00	1.98	3.16E-02	4.90E-01
**HADH**	0.35	2.75E-01	1.00E+00	-0.57	3.16E-02	4.90E-01
**TNFRSF21**	0.34	4.19E-01	1.00E+00	1.01	3.16E-02	4.90E-01
**LINC00632**	1.02	1.30E-01	1.00E+00	-1.17	3.17E-02	4.90E-01
**AC119427.1**	0.21	6.78E-01	1.00E+00	-1.50	3.17E-02	4.90E-01
**AL365356.5**	-1.56	2.28E-01	1.00E+00	2.71	3.18E-02	4.91E-01
**SALL4**	-3.00	2.02E-02	8.85E-01	2.44	3.18E-02	4.91E-01
**MT1L**	-0.27	6.79E-01	1.00E+00	-0.80	3.18E-02	4.91E-01
**TNFRSF10B**	-0.09	7.44E-01	1.00E+00	1.16	3.19E-02	4.92E-01
**SIRPA**	-0.02	9.26E-01	1.00E+00	1.15	3.19E-02	4.92E-01
**SCFD2**	-0.21	3.68E-01	1.00E+00	-0.43	3.20E-02	4.92E-01
**CYP24A1**	-0.29	7.84E-01	1.00E+00	2.27	3.20E-02	4.92E-01
**SUMO4**	-3.51	2.65E-02	9.49E-01	1.78	3.20E-02	4.92E-01
**HPCA**	-3.54	2.47E-01	1.00E+00	2.76	3.21E-02	4.93E-01
**NME7**	-0.09	7.81E-01	1.00E+00	-0.71	3.21E-02	4.93E-01
**AL353648.1**	1.72	2.93E-01	1.00E+00	-4.55	3.21E-02	4.93E-01
**TRIM22**	0.21	7.05E-01	1.00E+00	1.14	3.21E-02	4.93E-01
**HACL1**	-0.09	7.43E-01	1.00E+00	-0.64	3.23E-02	4.94E-01
**AIF1**	-0.31	4.40E-01	1.00E+00	0.76	3.23E-02	4.94E-01

**AC068860.1**	-0.80	7.03E-01	1.00E+00	-2.90	3.23E-02	4.94E-01
**LINC01644**	-0.42	9.02E-01	1.00E+00	3.98	3.23E-02	4.94E-01
**G8687**	-2.56	8.27E-02	1.00E+00	-2.27	3.24E-02	4.94E-01
**GZMK**	-3.06	7.46E-02	1.00E+00	1.95	3.24E-02	4.94E-01
**XLOC_003809**	-2.01	2.62E-01	1.00E+00	2.92	3.24E-02	4.94E-01
**POLR2J3**	0.34	5.81E-01	1.00E+00	0.79	3.24E-02	4.94E-01
**ZNF865**	-0.54	1.08E-01	1.00E+00	1.96	3.24E-02	4.94E-01
**HTR2B**	0.84	4.67E-01	1.00E+00	1.63	3.24E-02	4.94E-01
**ZC3H11A**	-1.10	7.53E-01	1.00E+00	2.09	3.24E-02	4.94E-01
**F8A1**	-0.21	5.29E-01	1.00E+00	-1.31	3.24E-02	4.94E-01
**AC068338.3**	NA	NA	NA	-1.97	3.25E-02	4.94E-01
**TGM1**	0.12	8.46E-01	1.00E+00	1.27	3.25E-02	4.94E-01
**IDH1**	0.63	1.31E-01	1.00E+00	-0.84	3.26E-02	4.94E-01
**AC026367.1**	0.43	6.43E-01	1.00E+00	2.19	3.26E-02	4.94E-01
**COLGALT1**	-0.12	5.65E-01	1.00E+00	0.61	3.26E-02	4.94E-01
**AC105250.1**	-0.87	7.99E-01	1.00E+00	-1.52	3.27E-02	4.94E-01
**SNHG12**	0.25	7.18E-01	1.00E+00	1.14	3.27E-02	4.94E-01
**SRC**	0.09	7.00E-01	1.00E+00	1.38	3.27E-02	4.94E-01
**ATP5PDP4**	-0.27	9.38E-01	1.00E+00	-1.60	3.27E-02	4.94E-01
**ERFE**	-0.26	8.58E-01	1.00E+00	2.84	3.27E-02	4.94E-01
**CCDC153**	-0.27	5.01E-01	1.00E+00	-0.80	3.28E-02	4.94E-01
**SH3RF3**	0.10	7.08E-01	1.00E+00	1.39	3.28E-02	4.94E-01
**PDK3**	0.25	6.65E-01	1.00E+00	-0.76	3.28E-02	4.94E-01
**FKBP15**	-0.14	4.50E-01	1.00E+00	0.79	3.28E-02	4.94E-01
**ITGA4**	-0.67	5.05E-02	1.00E+00	1.18	3.28E-02	4.94E-01
**KRT222**	0.65	6.83E-01	1.00E+00	-2.07	3.28E-02	4.94E-01
**CEBPZOS**	0.02	9.21E-01	1.00E+00	-0.45	3.28E-02	4.94E-01
**GLIS3**	-0.36	4.61E-01	1.00E+00	1.61	3.28E-02	4.94E-01
**LYRM1**	0.26	1.83E-01	1.00E+00	-0.63	3.29E-02	4.94E-01
**RUNX2**	-0.21	5.35E-01	1.00E+00	1.21	3.29E-02	4.94E-01
**COQ5**	0.40	5.57E-02	1.00E+00	-0.51	3.29E-02	4.94E-01
**IGHV3-49**	6.57	2.25E-07	8.84E-05	3.88	3.29E-02	4.94E-01

**KRT8P36**	-0.07	9.74E-01	1.00E+00	-1.92	3.30E-02	4.95E-01
**NRP2**	0.08	8.41E-01	1.00E+00	1.45	3.30E-02	4.95E-01
**AC007619.1**	0.37	8.57E-01	1.00E+00	1.71	3.30E-02	4.95E-01
**GPLD1**	0.34	5.23E-01	1.00E+00	-0.90	3.30E-02	4.95E-01
**KRT18P63**	-3.71	7.70E-03	5.38E-01	2.22	3.31E-02	4.95E-01
**PSTPIP1**	-0.32	5.01E-01	1.00E+00	1.15	3.31E-02	4.95E-01
**CYB5A**	0.08	6.81E-01	1.00E+00	-0.87	3.31E-02	4.95E-01
**COG8**	-0.20	5.68E-01	1.00E+00	-0.55	3.31E-02	4.95E-01
**G26894**	-1.69	4.16E-01	1.00E+00	3.88	3.32E-02	4.96E-01
**KIAA0513**	-0.24	4.99E-01	1.00E+00	0.89	3.32E-02	4.96E-01
**GON7**	-0.14	4.93E-01	1.00E+00	-0.56	3.32E-02	4.96E-01
**TNN**	-0.96	2.71E-01	1.00E+00	-1.83	3.33E-02	4.96E-01
**IGSF22**	0.32	4.23E-01	1.00E+00	0.95	3.33E-02	4.96E-01
**CSK**	-0.14	5.29E-01	1.00E+00	1.19	3.33E-02	4.96E-01
**GPR85**	-0.17	8.40E-01	1.00E+00	1.63	3.34E-02	4.97E-01
**ITGB2**	-0.20	5.43E-01	1.00E+00	1.59	3.34E-02	4.97E-01
**HIPK2**	0.34	4.99E-01	1.00E+00	0.58	3.35E-02	4.98E-01
**MICA**	0.04	8.60E-01	1.00E+00	0.47	3.35E-02	4.98E-01
**MN1**	-0.40	2.79E-01	1.00E+00	0.95	3.35E-02	4.98E-01
**AC011239.2**	-2.17	1.34E-01	1.00E+00	-2.18	3.36E-02	4.98E-01
**CDKN2AIPNL**	-0.01	9.61E-01	1.00E+00	-0.61	3.36E-02	4.98E-01
**G9105**	0.11	9.39E-01	1.00E+00	-2.12	3.36E-02	4.98E-01
**PPP4R1**	-0.24	3.81E-01	1.00E+00	0.71	3.37E-02	4.98E-01
**NPM3**	-0.83	4.71E-04	9.32E-02	0.87	3.37E-02	4.98E-01
**BTK**	0.03	9.49E-01	1.00E+00	1.25	3.37E-02	4.98E-01
**MTCO1P12**	-0.77	8.10E-01	1.00E+00	-1.59	3.37E-02	4.98E-01
**XLOC_004054**	0.92	7.17E-01	1.00E+00	2.62	3.37E-02	4.98E-01
**AC092167.1**	0.43	9.03E-01	1.00E+00	2.88	3.37E-02	4.98E-01
**TMBIM4**	0.21	4.23E-01	1.00E+00	-0.88	3.38E-02	4.98E-01
**LINC01431**	-1.24	2.06E-01	1.00E+00	-1.32	3.38E-02	4.98E-01
**LRRC15**	-0.47	5.48E-01	1.00E+00	1.59	3.38E-02	4.98E-01
**TTLL10**	0.21	8.06E-01	1.00E+00	-2.58	3.38E-02	4.98E-01

**COL16A1**	0.00	9.98E-01	1.00E+00	1.38	3.38E-02	4.98E-01
**AC011247.1**	0.32	6.90E-01	1.00E+00	-1.13	3.39E-02	4.98E-01
**SLC34A1**	-2.41	5.03E-02	1.00E+00	2.14	3.39E-02	4.99E-01
**AC008556.1**	-0.07	8.83E-01	1.00E+00	-1.35	3.39E-02	4.99E-01
**CD2**	-0.92	1.41E-01	1.00E+00	1.61	3.40E-02	5.00E-01
**CD84**	0.41	4.77E-01	1.00E+00	1.25	3.41E-02	5.00E-01
**AL357079.2**	-0.35	8.51E-01	1.00E+00	-2.16	3.41E-02	5.00E-01
**HOXB13**	1.84	4.69E-01	1.00E+00	3.37	3.41E-02	5.00E-01
**GLRX5**	-0.01	9.68E-01	1.00E+00	-1.35	3.42E-02	5.00E-01
**AGFG2**	0.15	4.51E-01	1.00E+00	-0.60	3.42E-02	5.00E-01
**AC092953.1**	1.99	3.92E-01	1.00E+00	2.12	3.42E-02	5.00E-01
**LINC01506**	-0.03	9.92E-01	1.00E+00	1.95	3.43E-02	5.01E-01
**TNK2**	-0.22	2.83E-01	1.00E+00	1.46	3.43E-02	5.01E-01
**PPIF**	0.02	9.55E-01	1.00E+00	0.71	3.43E-02	5.01E-01
**CDH18**	-0.19	8.42E-01	1.00E+00	-3.18	3.43E-02	5.01E-01
**DDA1**	-0.05	8.50E-01	1.00E+00	0.29	3.43E-02	5.01E-01
**ARSJ**	-0.09	8.37E-01	1.00E+00	0.97	3.43E-02	5.01E-01
**CHRAC1**	-0.15	3.92E-01	1.00E+00	-0.41	3.44E-02	5.01E-01
**PHOSPHO2**	0.11	8.49E-01	1.00E+00	-1.31	3.44E-02	5.01E-01
**LHFPL3-AS1**	0.95	4.85E-01	1.00E+00	2.27	3.44E-02	5.01E-01
**ARHGAP30**	0.02	9.54E-01	1.00E+00	1.54	3.44E-02	5.01E-01
**DDX41**	-0.35	7.71E-02	1.00E+00	0.44	3.45E-02	5.01E-01
**ORAI1**	-0.21	5.44E-01	1.00E+00	-1.05	3.45E-02	5.01E-01
**AC137723.1**	0.04	9.86E-01	1.00E+00	-1.81	3.45E-02	5.01E-01
**AC084036.1**	-0.33	7.14E-01	1.00E+00	-1.37	3.45E-02	5.01E-01
**SRGN**	0.51	3.27E-01	1.00E+00	1.04	3.46E-02	5.01E-01
**SERPINB9**	0.87	7.18E-02	1.00E+00	1.19	3.46E-02	5.02E-01
**ADPGK**	-0.06	7.61E-01	1.00E+00	0.46	3.46E-02	5.02E-01
**CHRNA7**	1.41	8.75E-02	1.00E+00	1.21	3.46E-02	5.02E-01
**BCL2L1**	0.19	3.59E-01	1.00E+00	0.64	3.47E-02	5.02E-01
**FGFR3**	-0.15	6.99E-01	1.00E+00	-1.73	3.47E-02	5.02E-01
**ITGA11**	0.29	6.51E-01	1.00E+00	1.11	3.47E-02	5.02E-01

**FBXO9**	0.18	4.28E-01	1.00E+00	-0.55	3.48E-02	5.03E-01
**ACADM**	0.47	1.73E-01	1.00E+00	-0.68	3.48E-02	5.03E-01
**TRBV7-8**	0.22	8.83E-01	1.00E+00	2.49	3.49E-02	5.03E-01
**MMP2-AS1**	0.40	8.51E-01	1.00E+00	2.29	3.49E-02	5.03E-01
**NPAS1**	-0.28	6.31E-01	1.00E+00	-1.68	3.49E-02	5.03E-01
**CDC25B**	0.03	9.17E-01	1.00E+00	1.18	3.49E-02	5.03E-01
**CD83**	0.01	9.82E-01	1.00E+00	0.67	3.49E-02	5.03E-01
**RUBCN**	-0.06	8.06E-01	1.00E+00	0.52	3.50E-02	5.03E-01
**CXorf21**	0.51	4.35E-01	1.00E+00	1.70	3.50E-02	5.03E-01
**PNPLA5**	1.87	2.94E-01	1.00E+00	-2.34	3.50E-02	5.03E-01
**LUZP1**	-0.24	2.40E-01	1.00E+00	0.41	3.50E-02	5.03E-01
**MPEG1**	-0.29	5.64E-01	1.00E+00	1.35	3.50E-02	5.03E-01
**AC002091.2**	-1.31	6.67E-01	1.00E+00	2.81	3.51E-02	5.03E-01
**AC083799.1**	-0.32	3.42E-01	1.00E+00	0.66	3.51E-02	5.03E-01
**CBX5**	0.39	2.80E-01	1.00E+00	-0.42	3.51E-02	5.03E-01
**AL353708.3**	-1.47	6.70E-01	1.00E+00	-2.92	3.52E-02	5.03E-01
**SNX29P2**	0.37	6.80E-01	1.00E+00	1.73	3.52E-02	5.03E-01
**NEK7**	0.92	3.83E-02	1.00E+00	-1.03	3.52E-02	5.03E-01
**MED28P3**	-0.76	4.35E-01	1.00E+00	-1.64	3.52E-02	5.03E-01
**SLC25A1**	0.17	6.05E-01	1.00E+00	-1.02	3.53E-02	5.03E-01
**CGGBP1**	0.18	3.90E-01	1.00E+00	-0.37	3.53E-02	5.03E-01
**AP000911.2**	-1.09	7.12E-01	1.00E+00	-3.39	3.53E-02	5.03E-01
**RPL30P11**	-3.52	2.96E-01	1.00E+00	-3.40	3.54E-02	5.03E-01
**RGS19**	0.02	9.50E-01	1.00E+00	0.85	3.54E-02	5.03E-01
**SNHG6**	-0.27	4.46E-01	1.00E+00	-0.85	3.54E-02	5.03E-01
**IL33**	-0.33	4.59E-01	1.00E+00	-0.73	3.54E-02	5.03E-01
**YIPF6**	0.36	2.59E-02	9.40E-01	-0.56	3.54E-02	5.03E-01
**OPN3**	0.85	5.41E-02	1.00E+00	-1.45	3.54E-02	5.03E-01
**RBMS1P1**	-1.38	4.41E-01	1.00E+00	-1.02	3.55E-02	5.03E-01
**ADAMTS2**	0.16	8.17E-01	1.00E+00	1.78	3.55E-02	5.03E-01
**CCDC51**	0.02	9.18E-01	1.00E+00	0.43	3.55E-02	5.03E-01
**SALL1**	-2.49	1.23E-02	6.93E-01	2.99	3.55E-02	5.03E-01

**AL731569.1**	-0.11	8.24E-01	1.00E+00	-1.86	3.55E-02	5.03E-01
**SP100**	0.09	8.20E-01	1.00E+00	0.52	3.55E-02	5.03E-01
**AL669831.5**	0.03	9.75E-01	1.00E+00	1.07	3.55E-02	5.03E-01
**AC080023.2**	2.05	3.23E-01	1.00E+00	-1.23	3.56E-02	5.04E-01
**SUCLA2**	0.26	2.87E-01	1.00E+00	-0.63	3.57E-02	5.06E-01
**ADCY2**	-0.31	2.99E-01	1.00E+00	-1.02	3.58E-02	5.06E-01
**TIGD7**	0.20	7.86E-01	1.00E+00	-1.11	3.58E-02	5.06E-01
**AL022069.1**	-2.51	1.01E-01	1.00E+00	-2.93	3.59E-02	5.06E-01
**AC011503.1**	2.64	1.08E-01	1.00E+00	-3.11	3.59E-02	5.07E-01
**CLECL1**	0.00	9.98E-01	1.00E+00	1.57	3.59E-02	5.07E-01
**TMEM26**	-2.55	4.56E-02	1.00E+00	2.73	3.59E-02	5.07E-01
**NOV**	-0.19	5.57E-01	1.00E+00	0.82	3.60E-02	5.07E-01
**SMURF2P1**	-0.34	3.19E-01	1.00E+00	1.48	3.60E-02	5.07E-01
**HSH2D**	0.17	8.37E-01	1.00E+00	2.33	3.61E-02	5.08E-01
**ZNF687**	-0.26	1.57E-01	1.00E+00	1.41	3.61E-02	5.08E-01
**ANKRD20A4**	-4.32	5.78E-03	4.52E-01	-1.76	3.62E-02	5.08E-01
**RPS4XP2**	NA	NA	NA	1.54	3.62E-02	5.08E-01
**SLC30A8**	-3.17	2.14E-01	1.00E+00	-4.51	3.62E-02	5.08E-01
**AL354928.1**	-2.22	1.36E-01	1.00E+00	2.53	3.62E-02	5.08E-01
**TLR8**	-0.32	6.51E-01	1.00E+00	2.14	3.62E-02	5.08E-01
**IGDCC4**	0.27	6.68E-01	1.00E+00	1.30	3.63E-02	5.08E-01
**ARMCX7P**	1.70	3.76E-01	1.00E+00	-1.11	3.63E-02	5.08E-01
**GPX7**	0.26	5.30E-01	1.00E+00	0.60	3.63E-02	5.08E-01
**YIF1B**	-0.39	6.19E-02	1.00E+00	0.41	3.63E-02	5.08E-01
**XLOC_001953**	0.65	3.73E-01	1.00E+00	1.39	3.63E-02	5.08E-01
**ZNF208**	0.28	7.36E-01	1.00E+00	-2.05	3.63E-02	5.08E-01
**SSC5D**	0.01	9.81E-01	1.00E+00	1.59	3.64E-02	5.08E-01
**LINC02211**	4.85	4.83E-03	4.03E-01	2.59	3.64E-02	5.08E-01
**AP000439.2**	0.64	1.50E-01	1.00E+00	-2.07	3.64E-02	5.08E-01
**HIST1H2BE**	-1.85	1.88E-01	1.00E+00	-2.19	3.64E-02	5.08E-01
**PIEZO2**	0.84	1.04E-01	1.00E+00	1.31	3.65E-02	5.08E-01
**AC034243.1**	-1.10	6.86E-01	1.00E+00	-2.13	3.65E-02	5.08E-01

**PIK3R5**	-0.34	4.08E-01	1.00E+00	1.75	3.65E-02	5.08E-01
**WFDC12**	-0.88	1.83E-01	1.00E+00	1.66	3.66E-02	5.09E-01
**DZIP1**	0.08	8.73E-01	1.00E+00	1.00	3.66E-02	5.09E-01
**BSN**	-1.67	1.61E-02	7.94E-01	2.42	3.67E-02	5.10E-01
**COL2A1**	-1.96	2.13E-02	8.94E-01	-2.76	3.67E-02	5.10E-01
**AC007546.1**	3.41	2.95E-01	1.00E+00	-2.91	3.67E-02	5.10E-01
**STAG2**	0.20	3.50E-01	1.00E+00	0.30	3.67E-02	5.10E-01
**PNPLA6**	-0.16	3.89E-01	1.00E+00	1.18	3.68E-02	5.10E-01
**FLVCR2**	-0.02	9.71E-01	1.00E+00	1.00	3.68E-02	5.10E-01
**AC068620.1**	-0.20	8.41E-01	1.00E+00	-1.36	3.68E-02	5.10E-01
**XIST**	-0.88	1.36E-01	1.00E+00	1.24	3.68E-02	5.10E-01
**CORO1A**	-0.36	3.12E-01	1.00E+00	1.29	3.68E-02	5.10E-01
**CNTNAP2**	-2.43	1.11E-02	6.52E-01	1.81	3.69E-02	5.10E-01
**XLOC_014097**	-1.54	5.99E-01	1.00E+00	2.72	3.69E-02	5.10E-01
**LINC01443**	-0.13	9.24E-01	1.00E+00	2.01	3.69E-02	5.10E-01
**SLC20A2**	-0.11	6.47E-01	1.00E+00	0.52	3.69E-02	5.10E-01
**PPP4R1-AS1**	-1.76	5.63E-01	1.00E+00	-2.05	3.70E-02	5.10E-01
**ITPA**	-0.17	4.35E-01	1.00E+00	0.37	3.70E-02	5.10E-01
**RIMS1**	-0.54	5.90E-01	1.00E+00	-1.48	3.70E-02	5.10E-01
**XLOC_009498**	1.92	1.01E-01	1.00E+00	-1.76	3.71E-02	5.10E-01
**KLRB1**	-0.64	4.50E-01	1.00E+00	1.49	3.71E-02	5.10E-01
**AC116407.1**	-0.29	7.52E-01	1.00E+00	-2.25	3.71E-02	5.10E-01
**ARPP19**	0.38	1.06E-01	1.00E+00	-0.41	3.71E-02	5.10E-01
**AC004812.2**	-0.39	3.09E-01	1.00E+00	-1.12	3.72E-02	5.10E-01
**PLIN3**	-0.32	3.12E-01	1.00E+00	0.45	3.72E-02	5.10E-01
**SLC44A1**	0.15	4.67E-01	1.00E+00	-0.52	3.72E-02	5.10E-01
**HIC1**	0.50	3.15E-01	1.00E+00	1.48	3.72E-02	5.10E-01
**TOR2A**	-0.18	4.90E-01	1.00E+00	0.54	3.72E-02	5.10E-01
**PNOC**	-1.22	7.08E-01	1.00E+00	3.27	3.72E-02	5.10E-01
**AP001021.2**	-0.20	9.01E-01	1.00E+00	-1.95	3.72E-02	5.10E-01
**AC093912.1**	-2.92	3.90E-01	1.00E+00	-4.05	3.73E-02	5.10E-01
**XLOC_006617**	-0.93	7.87E-01	1.00E+00	5.16	3.73E-02	5.11E-01

**AC017104.3**	1.92	1.17E-01	1.00E+00	2.02	3.74E-02	5.11E-01
**RUBCNL**	0.61	3.43E-01	1.00E+00	1.10	3.74E-02	5.11E-01
**AC007318.2**	-3.34	9.79E-02	1.00E+00	1.73	3.74E-02	5.11E-01
**DPEP2**	0.33	5.84E-01	1.00E+00	1.19	3.75E-02	5.11E-01
**AC099552.3**	1.23	4.56E-01	1.00E+00	-2.89	3.75E-02	5.11E-01
**LTBP2**	0.36	4.58E-01	1.00E+00	1.20	3.75E-02	5.12E-01
**AL035530.2**	0.00	9.98E-01	1.00E+00	-0.77	3.76E-02	5.12E-01
**MRPL19**	-0.10	6.13E-01	1.00E+00	-0.47	3.76E-02	5.12E-01
**ARL15**	-0.06	8.25E-01	1.00E+00	-0.82	3.77E-02	5.12E-01
**FCER1G**	0.20	6.29E-01	1.00E+00	0.91	3.77E-02	5.12E-01
**KIAA0391**	0.28	2.91E-01	1.00E+00	0.72	3.77E-02	5.12E-01
**PIP5KL1**	-0.04	9.11E-01	1.00E+00	0.79	3.77E-02	5.12E-01
**LINC01269**	2.53	2.88E-01	1.00E+00	2.24	3.77E-02	5.12E-01
**ATP5MC3**	0.15	5.38E-01	1.00E+00	-0.42	3.77E-02	5.12E-01
**HNRNPDL**	0.13	5.54E-01	1.00E+00	0.35	3.78E-02	5.12E-01
**DBNL**	-0.12	5.87E-01	1.00E+00	0.38	3.78E-02	5.12E-01
**KLF12**	-0.08	8.28E-01	1.00E+00	0.58	3.78E-02	5.12E-01
**DRAM2**	0.17	5.07E-01	1.00E+00	-0.71	3.79E-02	5.12E-01
**C9orf116**	-0.02	9.76E-01	1.00E+00	0.75	3.79E-02	5.12E-01
**NACA4P**	-0.13	9.52E-01	1.00E+00	-1.47	3.79E-02	5.12E-01
**G29073**	-4.34	1.23E-01	1.00E+00	2.59	3.80E-02	5.12E-01
**ACTR3C**	0.10	8.43E-01	1.00E+00	-0.95	3.80E-02	5.12E-01
**AC006449.5**	-0.24	8.28E-01	1.00E+00	-2.02	3.80E-02	5.12E-01
**IGFBP2**	-0.13	7.42E-01	1.00E+00	1.54	3.80E-02	5.12E-01
**AC009542.1**	0.59	8.65E-01	1.00E+00	2.90	3.80E-02	5.12E-01
**XLOC_012551**	-1.27	7.09E-01	1.00E+00	-2.92	3.80E-02	5.12E-01
**OTOGL**	-1.17	2.58E-01	1.00E+00	-2.06	3.80E-02	5.12E-01
**TMEM260**	-0.08	7.85E-01	1.00E+00	-0.57	3.80E-02	5.12E-01
**PTER**	-0.27	2.74E-01	1.00E+00	-0.54	3.81E-02	5.12E-01
**BX293535.1**	-3.36	1.62E-01	1.00E+00	-2.18	3.81E-02	5.12E-01
**SELL**	0.14	7.95E-01	1.00E+00	1.69	3.81E-02	5.12E-01
**ANO1**	-0.21	3.94E-01	1.00E+00	-0.58	3.82E-02	5.12E-01

**FER1L5**	1.03	5.52E-01	1.00E+00	-3.02	3.82E-02	5.12E-01
**NUDT16**	0.01	9.62E-01	1.00E+00	0.68	3.82E-02	5.12E-01
**TUBB2A**	-0.23	5.65E-01	1.00E+00	0.64	3.83E-02	5.12E-01
**SEC14L1P1**	-1.02	2.57E-01	1.00E+00	1.46	3.83E-02	5.12E-01
**CD3D**	-0.38	5.98E-01	1.00E+00	1.30	3.83E-02	5.12E-01
**CCDC78**	-1.24	4.39E-01	1.00E+00	2.67	3.83E-02	5.12E-01
**FRMD6-AS1**	0.02	9.68E-01	1.00E+00	0.92	3.83E-02	5.12E-01
**FAM45BP**	0.07	9.27E-01	1.00E+00	-0.83	3.83E-02	5.12E-01
**XLOC_000567**	-0.35	7.03E-01	1.00E+00	-1.55	3.84E-02	5.12E-01
**CSF3R**	-0.41	4.24E-01	1.00E+00	2.04	3.84E-02	5.12E-01
**ANKRD2**	0.23	7.44E-01	1.00E+00	1.30	3.84E-02	5.12E-01
**SDK2**	-1.15	1.65E-02	8.08E-01	1.29	3.84E-02	5.12E-01
**MAFTRR**	0.14	8.53E-01	1.00E+00	-1.35	3.85E-02	5.12E-01
**TRPT1**	0.06	7.87E-01	1.00E+00	-0.46	3.85E-02	5.12E-01
**GIMAP2**	0.24	5.90E-01	1.00E+00	1.01	3.85E-02	5.12E-01
**BMI1**	0.20	5.22E-01	1.00E+00	-0.63	3.85E-02	5.12E-01
**PCSK4**	0.02	9.83E-01	1.00E+00	1.81	3.85E-02	5.12E-01
**RHOQP1**	1.73	2.09E-01	1.00E+00	-2.12	3.86E-02	5.12E-01
**YJU2**	-0.33	2.12E-01	1.00E+00	0.40	3.86E-02	5.12E-01
**TMEM17**	0.66	4.84E-02	1.00E+00	-0.64	3.86E-02	5.12E-01
**RAB5C**	0.06	7.79E-01	1.00E+00	0.54	3.86E-02	5.12E-01
**DOK3**	-0.04	9.43E-01	1.00E+00	1.80	3.86E-02	5.12E-01
**FNBP1L**	0.03	9.36E-01	1.00E+00	-0.56	3.86E-02	5.12E-01
**GPS2**	-3.40	3.55E-02	1.00E+00	-0.94	3.86E-02	5.12E-01
**C8orf34**	0.30	6.44E-01	1.00E+00	-1.55	3.86E-02	5.12E-01
**KYNU**	-0.70	9.42E-02	1.00E+00	1.26	3.87E-02	5.13E-01
**TMEM14B**	0.29	2.25E-01	1.00E+00	-0.82	3.87E-02	5.13E-01
**HCLS1**	0.07	8.40E-01	1.00E+00	1.26	3.87E-02	5.13E-01
**MRPL28**	-0.26	2.14E-01	1.00E+00	0.36	3.88E-02	5.13E-01
**ZNFX1**	-0.03	9.43E-01	1.00E+00	0.92	3.88E-02	5.13E-01
**CLDND2**	-3.08	1.15E-01	1.00E+00	-1.41	3.88E-02	5.13E-01
**BCAR3**	-0.01	9.72E-01	1.00E+00	-0.83	3.89E-02	5.13E-01

**CRYBB2P1**	-0.11	8.46E-01	1.00E+00	0.67	3.89E-02	5.13E-01
**SLC7A2**	0.37	2.18E-01	1.00E+00	0.86	3.89E-02	5.13E-01
**IGKV2D-40**	NA	NA	NA	3.97	3.89E-02	5.13E-01
**OSBPL8**	0.15	6.20E-01	1.00E+00	-0.38	3.89E-02	5.13E-01
**PRNCR1**	2.11	5.33E-01	1.00E+00	-1.63	3.89E-02	5.13E-01
**AP000347.1**	0.29	8.10E-01	1.00E+00	1.93	3.89E-02	5.13E-01
**SLC16A7**	0.17	5.82E-01	1.00E+00	-0.59	3.90E-02	5.13E-01
**DDHD2**	0.30	4.45E-01	1.00E+00	-0.63	3.90E-02	5.13E-01
**ANAPC13**	0.22	2.38E-01	1.00E+00	-0.77	3.91E-02	5.14E-01
**TMSB10**	0.00	1.00E+00	1.00E+00	0.66	3.91E-02	5.14E-01
**LINC01871**	0.44	7.66E-01	1.00E+00	1.19	3.91E-02	5.14E-01
**ALKAL1**	1.62	3.33E-01	1.00E+00	-3.23	3.92E-02	5.15E-01
**Z94721.3**	-2.71	3.15E-02	1.00E+00	2.27	3.93E-02	5.15E-01
**PRKRIP1**	0.16	4.57E-01	1.00E+00	0.41	3.94E-02	5.16E-01
**PTN**	0.08	8.66E-01	1.00E+00	0.94	3.94E-02	5.16E-01
**AP002026.1**	0.11	9.15E-01	1.00E+00	-1.32	3.94E-02	5.16E-01
**P2RX6P**	0.85	3.76E-01	1.00E+00	-1.94	3.95E-02	5.17E-01
**DNAJC25**	-0.14	6.97E-01	1.00E+00	-0.97	3.95E-02	5.17E-01
**MMP19**	0.20	6.15E-01	1.00E+00	1.35	3.95E-02	5.17E-01
**TYSND1**	-0.09	6.76E-01	1.00E+00	-0.48	3.96E-02	5.17E-01
**CANT1**	0.00	9.81E-01	1.00E+00	0.75	3.97E-02	5.18E-01
**SLCO2A1**	0.09	6.84E-01	1.00E+00	1.24	3.97E-02	5.19E-01
**PRAG1**	-0.49	5.50E-02	1.00E+00	1.49	3.98E-02	5.19E-01
**ANKRD45**	-0.74	7.21E-01	1.00E+00	2.38	3.98E-02	5.19E-01
**GBAP1**	0.48	1.96E-01	1.00E+00	0.96	3.98E-02	5.19E-01
**SPON2**	-0.25	5.15E-01	1.00E+00	0.59	3.99E-02	5.19E-01
**SLC22A3**	0.47	1.28E-01	1.00E+00	-0.75	3.99E-02	5.19E-01
**MTIF2**	-0.09	6.55E-01	1.00E+00	-0.26	3.99E-02	5.19E-01
**ZSCAN26**	0.11	8.17E-01	1.00E+00	-0.79	4.00E-02	5.19E-01
**SPINK7**	-2.17	6.86E-02	1.00E+00	1.90	4.00E-02	5.19E-01
**HRH2**	-0.88	3.46E-02	1.00E+00	1.57	4.00E-02	5.19E-01
**RETREG3**	0.28	1.35E-01	1.00E+00	-0.46	4.00E-02	5.19E-01

**PGM1**	0.25	4.27E-01	1.00E+00	-0.69	4.00E-02	5.19E-01
**AL161431.1**	-1.04	4.00E-01	1.00E+00	1.90	4.00E-02	5.19E-01
**SMTNL1**	-2.03	7.10E-02	1.00E+00	2.18	4.01E-02	5.19E-01
**SLC3A2**	-0.25	2.67E-01	1.00E+00	0.29	4.01E-02	5.19E-01
**DYNC2H1**	0.03	9.31E-01	1.00E+00	-0.42	4.01E-02	5.19E-01
**HIST1H2AJ**	-2.52	2.11E-01	1.00E+00	-2.30	4.02E-02	5.19E-01
**XLOC_007038**	1.68	4.35E-01	1.00E+00	-2.39	4.02E-02	5.19E-01
**P2RX5**	-1.00	6.80E-01	1.00E+00	3.84	4.02E-02	5.19E-01
**AC010245.2**	0.91	1.30E-01	1.00E+00	-1.74	4.02E-02	5.19E-01
**ZCCHC4**	0.20	5.51E-01	1.00E+00	-0.64	4.02E-02	5.19E-01
**ERGIC3**	-0.14	5.21E-01	1.00E+00	0.29	4.02E-02	5.19E-01
**THUMPD2**	0.25	2.97E-01	1.00E+00	-0.49	4.03E-02	5.19E-01
**PARP9**	-0.10	8.71E-01	1.00E+00	0.99	4.03E-02	5.19E-01
**PTGFRN**	-0.11	5.64E-01	1.00E+00	0.84	4.04E-02	5.20E-01
**APOBEC3G**	-0.09	8.43E-01	1.00E+00	1.42	4.04E-02	5.20E-01
**PPP1R18**	-0.30	1.94E-01	1.00E+00	1.26	4.04E-02	5.20E-01
**PITX1**	-1.74	1.23E-03	1.86E-01	2.29	4.04E-02	5.20E-01
**LINC02067**	1.03	2.96E-01	1.00E+00	-2.62	4.05E-02	5.20E-01
**TMEM198B**	0.34	2.00E-01	1.00E+00	0.65	4.06E-02	5.22E-01
**AP000864.1**	-0.80	8.18E-01	1.00E+00	3.45	4.07E-02	5.22E-01
**UQCRQ**	-0.02	9.13E-01	1.00E+00	-1.09	4.07E-02	5.22E-01
**SLFN12**	0.12	7.90E-01	1.00E+00	0.83	4.07E-02	5.22E-01
**BCHE**	-0.56	1.35E-01	1.00E+00	-0.98	4.08E-02	5.22E-01
**AC115284.2**	-0.25	7.59E-01	1.00E+00	-1.93	4.08E-02	5.22E-01
**CDR2L**	0.09	8.49E-01	1.00E+00	0.93	4.08E-02	5.22E-01
**MIR3945HG**	NA	NA	NA	2.54	4.08E-02	5.22E-01
**IL16**	0.91	2.73E-02	9.66E-01	0.73	4.08E-02	5.22E-01
**XLOC_013547**	1.53	5.58E-01	1.00E+00	3.40	4.08E-02	5.22E-01
**HLF**	-0.39	1.71E-01	1.00E+00	-0.86	4.09E-02	5.22E-01
**AKR1C2**	0.74	3.20E-01	1.00E+00	-1.22	4.09E-02	5.22E-01
**RFX5**	0.13	6.27E-01	1.00E+00	0.60	4.09E-02	5.22E-01
**AC073508.3**	-0.22	6.68E-01	1.00E+00	-0.88	4.10E-02	5.22E-01

**RUFY2**	-0.23	4.10E-01	1.00E+00	-0.31	4.10E-02	5.22E-01
**PPP1R14C**	0.45	2.85E-01	1.00E+00	-1.41	4.10E-02	5.22E-01
**TUBB3**	-0.80	5.96E-01	1.00E+00	1.55	4.10E-02	5.22E-01
**TEX29**	-0.21	9.51E-01	1.00E+00	2.96	4.10E-02	5.22E-01
**AC110609.1**	3.31	3.27E-01	1.00E+00	-3.66	4.10E-02	5.22E-01
**AC092868.1**	-2.03	2.47E-01	1.00E+00	2.51	4.11E-02	5.22E-01
**AC091868.1**	1.41	6.79E-01	1.00E+00	-2.96	4.11E-02	5.22E-01
**COL8A1**	1.00	1.90E-01	1.00E+00	1.09	4.11E-02	5.22E-01
**AL513548.1**	0.00	9.99E-01	1.00E+00	2.51	4.12E-02	5.22E-01
**LINC00689**	-3.98	9.50E-03	6.06E-01	-2.33	4.12E-02	5.22E-01
**OVOL3**	1.74	3.60E-01	1.00E+00	-3.43	4.12E-02	5.22E-01
**RNA5-8SP2**	-1.53	2.53E-01	1.00E+00	-1.89	4.12E-02	5.23E-01
**GSTO1**	-0.12	4.56E-01	1.00E+00	0.45	4.12E-02	5.23E-01
**IPO5P1**	0.33	4.79E-01	1.00E+00	-0.66	4.13E-02	5.24E-01
**C17orf51**	0.21	6.38E-01	1.00E+00	0.72	4.14E-02	5.24E-01
**TMEM59L**	-1.25	6.55E-01	1.00E+00	2.07	4.14E-02	5.24E-01
**PSIP1**	0.06	7.86E-01	1.00E+00	-0.69	4.14E-02	5.24E-01
**AC005410.1**	0.44	8.14E-01	1.00E+00	-2.18	4.14E-02	5.24E-01
**SLC9A3-AS1**	-0.71	5.63E-02	1.00E+00	1.65	4.14E-02	5.24E-01
**TSPAN11**	0.09	8.16E-01	1.00E+00	1.50	4.15E-02	5.24E-01
**EP300-AS1**	-0.80	2.86E-01	1.00E+00	0.82	4.15E-02	5.24E-01
**AC004951.2**	-1.44	1.71E-01	1.00E+00	1.41	4.16E-02	5.25E-01
**STYX**	-0.06	8.09E-01	1.00E+00	-0.50	4.16E-02	5.25E-01
**GPR35**	0.57	3.27E-01	1.00E+00	2.06	4.16E-02	5.25E-01
**BRMS1**	-0.27	2.89E-01	1.00E+00	0.35	4.16E-02	5.25E-01
**LINC01376**	-0.39	5.31E-01	1.00E+00	-1.34	4.17E-02	5.25E-01
**AC005498.3**	0.30	6.29E-01	1.00E+00	-1.44	4.17E-02	5.25E-01
**SFMBT2**	0.27	6.68E-01	1.00E+00	1.80	4.17E-02	5.25E-01
**C1QL2**	-3.96	1.11E-03	1.75E-01	-3.10	4.17E-02	5.25E-01
**LGALSL**	0.27	5.79E-01	1.00E+00	-0.84	4.18E-02	5.25E-01
**AC010422.8**	0.28	9.25E-01	1.00E+00	1.71	4.18E-02	5.25E-01
**PTPN6**	-0.39	7.42E-02	1.00E+00	0.74	4.18E-02	5.25E-01

**ZNF807**	-0.34	8.55E-01	1.00E+00	-3.32	4.18E-02	5.25E-01
**BDKRB2**	0.11	7.67E-01	1.00E+00	0.85	4.19E-02	5.25E-01
**PRXL2A**	0.50	7.12E-02	1.00E+00	-0.63	4.20E-02	5.27E-01
**SPINDOC**	-0.13	6.53E-01	1.00E+00	1.61	4.20E-02	5.27E-01
**NGEF**	-0.34	3.57E-01	1.00E+00	-1.11	4.21E-02	5.27E-01
**ISCA1P1**	0.84	8.06E-01	1.00E+00	-1.05	4.21E-02	5.27E-01
**CLEC2L**	-1.38	1.96E-01	1.00E+00	-2.33	4.21E-02	5.27E-01
**BEX1**	-1.68	7.64E-02	1.00E+00	-1.06	4.21E-02	5.27E-01
**P4HA3**	-0.02	9.84E-01	1.00E+00	2.12	4.21E-02	5.27E-01
**VIPAS39**	0.07	7.14E-01	1.00E+00	0.31	4.22E-02	5.27E-01
**NDUFA5**	0.02	9.24E-01	1.00E+00	-0.86	4.22E-02	5.27E-01
**XLOC_004672**	0.14	8.40E-01	1.00E+00	2.17	4.22E-02	5.27E-01
**AC026765.2**	-2.28	5.01E-01	1.00E+00	-2.55	4.22E-02	5.27E-01
**ANKRD18A**	-0.23	7.31E-01	1.00E+00	-1.32	4.23E-02	5.27E-01
**UGT2B4**	0.76	8.27E-01	1.00E+00	-3.31	4.23E-02	5.27E-01
**AP001610.1**	-2.32	4.96E-01	1.00E+00	2.33	4.23E-02	5.27E-01
**CAMSAP3**	-0.35	5.27E-01	1.00E+00	-1.56	4.23E-02	5.27E-01
**ZNF441**	0.04	9.03E-01	1.00E+00	-0.82	4.24E-02	5.27E-01
**CXCR6**	0.31	7.46E-01	1.00E+00	2.13	4.24E-02	5.27E-01
**AC112191.2**	NA	NA	NA	-1.50	4.24E-02	5.27E-01
**HPSE**	0.39	4.09E-01	1.00E+00	0.88	4.24E-02	5.27E-01
**PYROXD1**	-0.04	8.74E-01	1.00E+00	-0.50	4.25E-02	5.27E-01
**MTRNR2L6**	1.02	7.67E-01	1.00E+00	-1.93	4.25E-02	5.27E-01
**DOCK4**	0.17	6.47E-01	1.00E+00	1.27	4.25E-02	5.27E-01
**PPIL6**	-0.07	8.80E-01	1.00E+00	-0.87	4.26E-02	5.27E-01
**HMGN2P3**	-0.18	6.49E-01	1.00E+00	-0.81	4.26E-02	5.27E-01
**AAK1**	-0.15	6.10E-01	1.00E+00	0.61	4.26E-02	5.27E-01
**CD27**	-0.96	3.18E-01	1.00E+00	1.53	4.26E-02	5.27E-01
**DAB2IP**	-0.01	9.85E-01	1.00E+00	1.29	4.26E-02	5.28E-01
**S1PR3**	0.03	9.19E-01	1.00E+00	1.02	4.27E-02	5.28E-01
**SCCPDH**	0.44	1.70E-01	1.00E+00	-0.77	4.27E-02	5.28E-01
**GBA**	0.14	5.21E-01	1.00E+00	0.50	4.28E-02	5.29E-01

**BTG1**	0.14	5.45E-01	1.00E+00	-0.75	4.28E-02	5.29E-01
**DENND4C**	0.11	6.10E-01	1.00E+00	-0.47	4.29E-02	5.30E-01
**RRS1-AS1**	-2.16	2.58E-01	1.00E+00	-2.60	4.30E-02	5.30E-01
**LCTL**	-0.18	9.59E-01	1.00E+00	2.54	4.30E-02	5.30E-01
**PCDHA6**	-3.09	9.31E-03	6.05E-01	-2.28	4.30E-02	5.30E-01
**FCHSD1**	0.10	7.67E-01	1.00E+00	1.01	4.30E-02	5.30E-01
**XLOC_008151**	-2.02	5.57E-01	1.00E+00	-1.96	4.31E-02	5.30E-01
**AL669831.4**	0.21	7.68E-01	1.00E+00	-1.29	4.31E-02	5.30E-01
**TXNDC15**	0.12	6.25E-01	1.00E+00	0.43	4.31E-02	5.30E-01
**AC126773.1**	1.33	6.25E-01	1.00E+00	2.84	4.31E-02	5.30E-01
**TMEM164**	0.04	8.96E-01	1.00E+00	0.73	4.31E-02	5.30E-01
**LYRM7**	-0.02	9.39E-01	1.00E+00	-0.69	4.32E-02	5.30E-01
**AZGP1P1**	0.61	2.72E-01	1.00E+00	1.01	4.32E-02	5.30E-01
**SRGAP1**	0.52	3.29E-01	1.00E+00	0.53	4.32E-02	5.30E-01
**SLC12A8**	0.19	7.62E-01	1.00E+00	0.99	4.32E-02	5.30E-01
**PKM**	-0.22	9.80E-02	1.00E+00	0.46	4.33E-02	5.30E-01
**AC005000.1**	-0.18	8.71E-01	1.00E+00	-0.87	4.33E-02	5.30E-01
**ATPAF1**	0.16	6.25E-01	1.00E+00	-1.23	4.33E-02	5.30E-01
**ZNF616**	-0.01	9.86E-01	1.00E+00	-0.57	4.33E-02	5.30E-01
**ZNF773**	0.29	2.94E-01	1.00E+00	-0.47	4.34E-02	5.30E-01
**UBALD2**	-0.05	8.89E-01	1.00E+00	1.60	4.34E-02	5.30E-01
**PPIAP29**	0.74	2.16E-01	1.00E+00	-0.71	4.34E-02	5.30E-01
**GOLGA2P7**	0.00	9.99E-01	1.00E+00	1.30	4.34E-02	5.30E-01
**COMP**	-0.43	5.48E-01	1.00E+00	1.40	4.35E-02	5.31E-01
**AUNIP**	-1.39	4.39E-03	3.74E-01	1.38	4.36E-02	5.31E-01
**PLEKHG2**	-0.24	5.30E-01	1.00E+00	1.57	4.36E-02	5.31E-01
**GRIP2**	0.34	5.64E-01	1.00E+00	1.53	4.36E-02	5.31E-01
**RDH14**	0.13	6.01E-01	1.00E+00	-0.65	4.36E-02	5.31E-01
**PCGF6**	0.18	5.07E-01	1.00E+00	-0.49	4.36E-02	5.31E-01
**SLC44A3-AS1**	-0.41	5.97E-01	1.00E+00	0.79	4.38E-02	5.33E-01
**PALB2**	-0.07	7.65E-01	1.00E+00	0.48	4.38E-02	5.33E-01
**LINC02478**	-0.90	5.29E-01	1.00E+00	-2.41	4.38E-02	5.33E-01

**MMD**	2.47	2.53E-03	2.58E-01	-1.23	4.39E-02	5.33E-01
**NOTCH2NLA**	0.28	4.07E-01	1.00E+00	0.57	4.39E-02	5.33E-01
**AL645933.2**	-0.51	4.54E-01	1.00E+00	1.21	4.39E-02	5.33E-01
**TOP1MT**	0.14	7.14E-01	1.00E+00	0.58	4.40E-02	5.33E-01
**VASH1**	0.29	6.26E-01	1.00E+00	1.70	4.40E-02	5.34E-01
**AL513550.1**	-1.10	1.45E-03	2.09E-01	-0.96	4.40E-02	5.34E-01
**TRBV2**	-1.20	5.66E-01	1.00E+00	2.80	4.41E-02	5.34E-01
**LPXN**	-0.24	5.36E-01	1.00E+00	0.91	4.41E-02	5.34E-01
**IL10RA**	-0.22	6.35E-01	1.00E+00	1.42	4.41E-02	5.34E-01
**DNASE1L1**	-0.03	9.33E-01	1.00E+00	0.73	4.42E-02	5.34E-01
**IFIH1**	-0.05	9.44E-01	1.00E+00	1.11	4.43E-02	5.35E-01
**MMRN1**	0.38	3.76E-01	1.00E+00	1.01	4.43E-02	5.35E-01
**WDR26**	-0.10	7.88E-01	1.00E+00	0.72	4.43E-02	5.35E-01
**GPR68**	-0.75	3.58E-02	1.00E+00	1.45	4.44E-02	5.35E-01
**AC092164.1**	0.26	7.39E-01	1.00E+00	-1.14	4.44E-02	5.35E-01
**AC096642.1**	0.17	9.20E-01	1.00E+00	-3.92	4.44E-02	5.35E-01
**FOXQ1**	0.01	9.70E-01	1.00E+00	-1.59	4.44E-02	5.35E-01
**XLOC_009272**	-0.74	4.37E-01	1.00E+00	-1.92	4.44E-02	5.35E-01
**ZNF503**	0.28	4.34E-01	1.00E+00	1.55	4.44E-02	5.35E-01
**IGHV3-72**	1.65	6.27E-01	1.00E+00	4.06	4.44E-02	5.35E-01
**STXBP5-AS1**	0.00	9.91E-01	1.00E+00	1.30	4.45E-02	5.35E-01
**G20694**	0.63	5.11E-01	1.00E+00	-1.48	4.45E-02	5.35E-01
**AC090206.1**	1.91	5.79E-01	1.00E+00	-2.15	4.45E-02	5.35E-01
**LILRB2**	-0.21	6.92E-01	1.00E+00	1.81	4.45E-02	5.35E-01
**XLOC_012945**	-2.16	3.22E-01	1.00E+00	-3.16	4.45E-02	5.35E-01
**PREPL**	0.00	9.89E-01	1.00E+00	-0.51	4.46E-02	5.35E-01
**HIF1A-AS2**	1.25	6.51E-01	1.00E+00	1.59	4.47E-02	5.36E-01
**SLC12A2**	0.11	8.84E-01	1.00E+00	-0.96	4.47E-02	5.36E-01
**SLC17A9**	-0.16	7.81E-01	1.00E+00	1.86	4.48E-02	5.37E-01
**SPRY4**	0.53	2.93E-01	1.00E+00	1.12	4.48E-02	5.37E-01
**PSTK**	-0.11	7.20E-01	1.00E+00	-0.93	4.49E-02	5.38E-01
**ZNF707**	-0.22	5.36E-01	1.00E+00	1.49	4.49E-02	5.38E-01

**SCAF1**	-0.24	3.15E-01	1.00E+00	1.54	4.50E-02	5.38E-01
**GPR18**	-0.90	5.74E-01	1.00E+00	1.46	4.50E-02	5.38E-01
**CLDN11**	0.09	8.54E-01	1.00E+00	1.13	4.51E-02	5.38E-01
**STRADB**	0.96	1.42E-01	1.00E+00	-1.02	4.51E-02	5.38E-01
**AC145625.1**	0.73	4.18E-01	1.00E+00	1.78	4.51E-02	5.38E-01
**AC125603.3**	-0.89	7.07E-01	1.00E+00	2.27	4.51E-02	5.38E-01
**TMEM206**	-0.05	8.95E-01	1.00E+00	0.69	4.52E-02	5.39E-01
**ARRDC1**	-0.31	3.49E-01	1.00E+00	0.34	4.52E-02	5.39E-01
**MYPOP**	-0.43	1.24E-01	1.00E+00	1.45	4.52E-02	5.39E-01
**RPE65**	0.55	7.01E-01	1.00E+00	2.53	4.53E-02	5.39E-01
**NUGGC**	1.21	2.38E-01	1.00E+00	2.17	4.53E-02	5.39E-01
**SYDE2**	-0.03	9.37E-01	1.00E+00	-1.35	4.53E-02	5.39E-01
**SULT4A1**	-2.25	3.27E-01	1.00E+00	2.67	4.54E-02	5.39E-01
**XLOC_014409**	-1.06	4.70E-01	1.00E+00	-3.87	4.54E-02	5.40E-01
**SAMSN1**	0.57	3.16E-01	1.00E+00	1.41	4.56E-02	5.41E-01
**ADAMTS12**	0.16	7.89E-01	1.00E+00	1.37	4.56E-02	5.41E-01
**XLOC_003993**	0.88	6.54E-01	1.00E+00	-1.98	4.56E-02	5.41E-01
**GDPD3**	0.14	8.36E-01	1.00E+00	1.12	4.56E-02	5.41E-01
**AC008946.1**	1.37	2.31E-01	1.00E+00	-1.28	4.56E-02	5.41E-01
**CETN3**	0.30	2.82E-01	1.00E+00	-0.61	4.57E-02	5.41E-01
**LINC01482**	-0.60	7.29E-01	1.00E+00	1.96	4.57E-02	5.41E-01
**CAVIN3**	-0.02	9.56E-01	1.00E+00	0.73	4.57E-02	5.41E-01
**CTSB**	-0.02	9.46E-01	1.00E+00	0.94	4.57E-02	5.41E-01
**TMEM64**	0.39	1.24E-01	1.00E+00	-0.69	4.57E-02	5.41E-01
**PTPRH**	-1.26	3.00E-02	9.97E-01	1.87	4.58E-02	5.41E-01
**XLOC_008971**	0.68	5.34E-01	1.00E+00	-2.19	4.58E-02	5.41E-01
**TMEM252**	0.27	6.54E-01	1.00E+00	-2.23	4.58E-02	5.41E-01
**FOXP1-IT1**	0.18	9.53E-01	1.00E+00	-2.11	4.58E-02	5.41E-01
**EIF4E3**	0.17	5.39E-01	1.00E+00	-0.76	4.58E-02	5.41E-01
**ATP5F1E**	0.13	4.33E-01	1.00E+00	-0.71	4.59E-02	5.41E-01
**EIF2AK1**	-0.10	5.58E-01	1.00E+00	-0.82	4.59E-02	5.41E-01
**LNX2**	-0.14	6.31E-01	1.00E+00	-0.57	4.59E-02	5.41E-01

**RAB4A**	0.06	8.20E-01	1.00E+00	-0.77	4.60E-02	5.41E-01
**AC005476.2**	-2.40	3.30E-02	1.00E+00	-1.30	4.60E-02	5.41E-01
**CIC**	-0.56	8.55E-03	5.69E-01	1.65	4.61E-02	5.42E-01
**KRBOX1**	0.20	9.12E-01	1.00E+00	-1.63	4.61E-02	5.42E-01
**CACNA1E**	-1.97	5.60E-01	1.00E+00	3.01	4.61E-02	5.42E-01
**GABARAPL2**	0.22	3.60E-01	1.00E+00	-0.62	4.61E-02	5.42E-01
**AC022540.1**	-0.32	8.61E-01	1.00E+00	2.19	4.62E-02	5.42E-01
**PUDP**	0.12	6.76E-01	1.00E+00	0.48	4.62E-02	5.42E-01
**P3H4**	-0.16	5.11E-01	1.00E+00	0.58	4.62E-02	5.42E-01
**AC005392.2**	NA	NA	NA	3.32	4.62E-02	5.42E-01
**CISD3**	-0.19	3.45E-01	1.00E+00	-0.46	4.63E-02	5.43E-01
**RRBP1**	-0.33	2.60E-01	1.00E+00	1.12	4.63E-02	5.43E-01
**FGD2**	-0.87	2.10E-02	8.94E-01	1.08	4.64E-02	5.43E-01
**MCEMP1**	NA	NA	NA	3.11	4.64E-02	5.43E-01
**AC239800.1**	-1.12	6.38E-01	1.00E+00	-1.85	4.64E-02	5.43E-01
**WDR72**	-0.69	2.75E-01	1.00E+00	-1.40	4.64E-02	5.43E-01
**PDGFRL**	0.88	8.72E-02	1.00E+00	1.16	4.65E-02	5.43E-01
**FMNL1**	0.11	7.57E-01	1.00E+00	1.40	4.65E-02	5.43E-01
**NUDT22**	-0.22	4.12E-01	1.00E+00	0.44	4.65E-02	5.43E-01
**AC016925.1**	NA	NA	NA	-1.57	4.65E-02	5.43E-01
**PARS2**	0.19	4.92E-01	1.00E+00	-0.50	4.65E-02	5.43E-01
**DHX58**	-0.10	8.55E-01	1.00E+00	1.10	4.66E-02	5.43E-01
**PPM1E**	-4.32	3.24E-04	6.99E-02	2.25	4.66E-02	5.43E-01
**AC138969.1**	1.35	4.60E-01	1.00E+00	2.10	4.66E-02	5.43E-01
**RGS20**	-0.38	5.57E-01	1.00E+00	0.90	4.67E-02	5.43E-01
**FBXO7**	0.12	5.60E-01	1.00E+00	-0.31	4.67E-02	5.43E-01
**AGL**	-0.08	7.77E-01	1.00E+00	-0.41	4.67E-02	5.43E-01
**PCAT18**	0.10	9.52E-01	1.00E+00	-2.07	4.67E-02	5.43E-01
**CSTF2T**	0.05	8.04E-01	1.00E+00	0.46	4.68E-02	5.43E-01
**SLC25A40**	0.09	7.26E-01	1.00E+00	-0.63	4.68E-02	5.43E-01
**CCDC71L**	0.10	7.47E-01	1.00E+00	1.48	4.68E-02	5.43E-01
**NKIRAS1**	0.47	1.35E-01	1.00E+00	-0.76	4.68E-02	5.43E-01

**AKR1D1**	-1.67	6.27E-01	1.00E+00	-4.17	4.68E-02	5.43E-01
**FGF5**	-3.56	5.01E-02	1.00E+00	3.86	4.69E-02	5.43E-01
**HLA-DMB**	-0.62	1.57E-01	1.00E+00	0.75	4.69E-02	5.43E-01
**RAB6A**	0.12	5.77E-01	1.00E+00	-0.34	4.69E-02	5.43E-01
**PLCB4**	0.22	6.92E-01	1.00E+00	-0.92	4.69E-02	5.43E-01
**CCDC74B**	0.17	7.69E-01	1.00E+00	0.71	4.69E-02	5.43E-01
**PLA1A**	-0.37	6.04E-01	1.00E+00	1.99	4.69E-02	5.43E-01
**ANAPC2**	-0.22	3.07E-01	1.00E+00	1.14	4.70E-02	5.43E-01
**TLNRD1**	0.01	9.79E-01	1.00E+00	1.37	4.70E-02	5.43E-01
**ANO3**	1.71	5.68E-02	1.00E+00	-1.53	4.70E-02	5.43E-01
**TCN2**	0.47	2.59E-01	1.00E+00	1.04	4.71E-02	5.43E-01
**CCDC114**	-0.19	7.55E-01	1.00E+00	-2.16	4.71E-02	5.43E-01
**SETP14**	0.16	8.70E-01	1.00E+00	-0.78	4.71E-02	5.43E-01
**SNX8**	-0.04	8.83E-01	1.00E+00	0.72	4.72E-02	5.44E-01
**MINDY1**	-0.11	6.08E-01	1.00E+00	0.41	4.72E-02	5.44E-01
**LOXL3**	0.12	8.00E-01	1.00E+00	1.36	4.72E-02	5.44E-01
**SDHAF3**	-0.01	9.68E-01	1.00E+00	-0.93	4.72E-02	5.44E-01
**GIMAP5**	1.39	3.05E-01	1.00E+00	1.54	4.73E-02	5.45E-01
**UBE2FP1**	0.94	2.29E-01	1.00E+00	1.30	4.74E-02	5.45E-01
**AC093503.1**	-1.86	5.23E-01	1.00E+00	-2.18	4.74E-02	5.45E-01
**TMEM183B**	-0.62	2.69E-01	1.00E+00	-0.83	4.74E-02	5.45E-01
**FSD1**	1.64	3.79E-01	1.00E+00	2.97	4.75E-02	5.45E-01
**ANAPC16**	0.09	5.87E-01	1.00E+00	-0.52	4.75E-02	5.45E-01
**G4643**	1.00	3.63E-01	1.00E+00	1.70	4.75E-02	5.45E-01
**FGR**	0.42	2.51E-01	1.00E+00	1.55	4.76E-02	5.45E-01
**TRBV20-1**	-1.75	8.87E-02	1.00E+00	1.54	4.76E-02	5.45E-01
**Z73979.1**	1.10	1.44E-01	1.00E+00	-1.56	4.76E-02	5.45E-01
**TNFRSF13B**	-1.15	5.72E-01	1.00E+00	3.31	4.76E-02	5.45E-01
**ACAP1**	-0.03	9.39E-01	1.00E+00	1.06	4.77E-02	5.45E-01
**G19426**	-4.10	6.89E-02	1.00E+00	-2.61	4.77E-02	5.45E-01
**KMT5B**	0.07	7.05E-01	1.00E+00	-0.55	4.77E-02	5.45E-01
**GNGT2**	0.02	9.70E-01	1.00E+00	1.13	4.77E-02	5.45E-01

**FAM81A**	-0.21	6.22E-01	1.00E+00	-0.97	4.77E-02	5.45E-01
**DTX2P1-UPK3BP1-PMS2P11**	-1.87	2.39E-01	1.00E+00	-1.37	4.78E-02	5.45E-01
**EGOT**	0.60	5.20E-01	1.00E+00	1.38	4.78E-02	5.45E-01
**DOK4**	0.16	6.89E-01	1.00E+00	-0.83	4.78E-02	5.45E-01
**AC025280.3**	-2.24	3.17E-01	1.00E+00	1.76	4.78E-02	5.46E-01
**ARL4C**	-0.32	3.34E-01	1.00E+00	1.00	4.79E-02	5.46E-01
**CHRM4**	-0.03	9.71E-01	1.00E+00	-2.11	4.79E-02	5.46E-01
**OLIG1**	-0.53	5.95E-01	1.00E+00	-1.69	4.80E-02	5.47E-01
**ASNA1**	-0.08	7.02E-01	1.00E+00	0.23	4.80E-02	5.47E-01
**IDI1**	-0.03	8.99E-01	1.00E+00	-0.75	4.81E-02	5.47E-01
**GRM5**	-1.24	5.75E-01	1.00E+00	-3.52	4.81E-02	5.47E-01
**PSMC3IP**	0.02	9.41E-01	1.00E+00	0.49	4.82E-02	5.47E-01
**CXCR1**	-1.92	4.28E-02	1.00E+00	1.29	4.82E-02	5.47E-01
**PATL1**	-0.11	6.02E-01	1.00E+00	0.92	4.82E-02	5.47E-01
**QPRT**	0.12	8.05E-01	1.00E+00	0.85	4.82E-02	5.47E-01
**PGM5P4**	-0.74	4.11E-02	1.00E+00	-1.31	4.82E-02	5.48E-01
**PEBP1**	0.09	6.44E-01	1.00E+00	-0.39	4.83E-02	5.48E-01
**FMNL2**	-0.11	7.93E-01	1.00E+00	1.04	4.83E-02	5.48E-01
**IGLJ2**	NA	NA	NA	6.22	4.84E-02	5.48E-01
**STAG3L5P**	-0.23	8.66E-01	1.00E+00	1.29	4.84E-02	5.48E-01
**LEMD1**	-0.45	6.78E-01	1.00E+00	1.31	4.85E-02	5.49E-01
**FOSL1**	-3.10	2.83E-02	9.75E-01	1.23	4.86E-02	5.50E-01
**SLC38A9**	0.28	4.79E-01	1.00E+00	0.65	4.86E-02	5.50E-01
**PLEKHO1**	-0.51	1.63E-01	1.00E+00	1.43	4.86E-02	5.50E-01
**NIPSNAP3B**	1.00	1.45E-01	1.00E+00	-1.28	4.87E-02	5.50E-01
**AC022306.2**	0.61	6.23E-01	1.00E+00	-1.25	4.87E-02	5.50E-01
**XLOC_002113**	-0.36	5.84E-01	1.00E+00	-1.14	4.87E-02	5.50E-01
**RGS10**	-0.35	1.22E-01	1.00E+00	0.51	4.87E-02	5.50E-01
**PLK2**	-0.61	9.82E-02	1.00E+00	0.60	4.88E-02	5.50E-01
**AL691432.2**	-0.40	1.90E-01	1.00E+00	-0.96	4.88E-02	5.50E-01
**RNF128**	-0.33	1.69E-01	1.00E+00	-0.90	4.89E-02	5.50E-01
**COMMD10**	-0.07	7.32E-01	1.00E+00	-0.80	4.89E-02	5.50E-01

**AC015908.3**	-0.78	5.84E-01	1.00E+00	-1.54	4.89E-02	5.50E-01
**RBMS2**	0.59	1.13E-01	1.00E+00	0.83	4.90E-02	5.50E-01
**ROM1**	-0.11	7.42E-01	1.00E+00	0.52	4.90E-02	5.50E-01
**ARHGAP4**	-0.10	7.50E-01	1.00E+00	1.46	4.90E-02	5.50E-01
**IGF2**	0.71	2.69E-01	1.00E+00	1.18	4.90E-02	5.50E-01
**G33837**	-0.08	9.45E-01	1.00E+00	-2.36	4.90E-02	5.50E-01
**GSG1L**	0.05	9.52E-01	1.00E+00	-2.12	4.90E-02	5.50E-01
**AC004943.1**	1.58	2.28E-01	1.00E+00	2.33	4.91E-02	5.50E-01
**AC004233.3**	-1.92	5.78E-01	1.00E+00	-2.52	4.91E-02	5.50E-01
**LINC00467**	0.08	8.44E-01	1.00E+00	-0.63	4.91E-02	5.50E-01
**OVCH1**	0.69	6.49E-01	1.00E+00	-2.98	4.91E-02	5.50E-01
**GARS-DT**	0.17	6.26E-01	1.00E+00	-0.59	4.92E-02	5.50E-01
**VWA3B**	-1.11	5.61E-01	1.00E+00	2.16	4.92E-02	5.50E-01
**TRBV27**	0.43	8.26E-01	1.00E+00	2.54	4.92E-02	5.50E-01
**CCDC173**	-1.18	1.82E-01	1.00E+00	1.40	4.92E-02	5.50E-01
**AL031733.2**	1.78	1.83E-01	1.00E+00	1.69	4.92E-02	5.50E-01
**LCORL**	0.05	8.82E-01	1.00E+00	-0.57	4.92E-02	5.50E-01
**AP001372.2**	-0.11	7.38E-01	1.00E+00	-0.92	4.92E-02	5.50E-01
**AC004471.1**	0.27	8.79E-01	1.00E+00	-2.33	4.93E-02	5.50E-01
**LILRA6**	-0.65	3.05E-01	1.00E+00	1.62	4.93E-02	5.50E-01
**NDST1**	0.02	9.10E-01	1.00E+00	0.88	4.93E-02	5.50E-01
**ZNF626**	0.29	3.60E-01	1.00E+00	-0.89	4.93E-02	5.50E-01
**G8228**	0.16	8.63E-01	1.00E+00	1.78	4.93E-02	5.50E-01
**PAWR**	-0.04	9.11E-01	1.00E+00	-0.54	4.93E-02	5.50E-01
**GINM1**	0.15	3.72E-01	1.00E+00	-0.38	4.93E-02	5.50E-01
**MMAB**	0.02	9.32E-01	1.00E+00	-0.40	4.95E-02	5.50E-01
**AL357143.1**	-1.24	7.19E-01	1.00E+00	3.26	4.95E-02	5.50E-01
**NUBP1**	-0.18	2.97E-01	1.00E+00	0.46	4.95E-02	5.50E-01
**EIF3G**	-0.13	4.68E-01	1.00E+00	0.44	4.95E-02	5.50E-01
**FAH**	1.08	1.27E-01	1.00E+00	-1.05	4.95E-02	5.50E-01
**CHRM1**	-0.36	5.36E-01	1.00E+00	-1.34	4.95E-02	5.50E-01
**FCGR2C**	-0.65	4.43E-01	1.00E+00	1.42	4.96E-02	5.50E-01

**CXCR2**	-0.47	2.42E-01	1.00E+00	0.69	4.96E-02	5.50E-01
**LINC01497**	0.67	6.97E-01	1.00E+00	-2.47	4.96E-02	5.50E-01
**PRMT6**	-0.34	2.81E-01	1.00E+00	0.35	4.97E-02	5.51E-01
**POU5F1P5**	0.19	9.55E-01	1.00E+00	4.33	4.97E-02	5.51E-01
**OAZ3**	-0.12	8.99E-01	1.00E+00	-0.68	4.98E-02	5.51E-01
**HNRNPCP1**	1.19	1.46E-01	1.00E+00	1.89	4.98E-02	5.51E-01
**MAPKAPK5**	-0.17	4.11E-01	1.00E+00	-0.37	4.98E-02	5.51E-01
**KLK10**	-0.50	2.75E-01	1.00E+00	1.20	4.98E-02	5.51E-01
**LINC01215**	-0.54	8.74E-01	1.00E+00	3.35	4.99E-02	5.51E-01
**ADAMDEC1**	-0.87	2.87E-01	1.00E+00	2.80	5.00E-02	5.52E-01
**AL138820.1**	0.13	9.65E-01	1.00E+00	-2.74	5.00E-02	5.52E-01
**AC015911.8**	-0.44	7.81E-01	1.00E+00	3.61	5.00E-02	5.52E-01
**AC079944.2**	-0.34	6.64E-01	1.00E+00	1.31	5.00E-02	5.52E-01
**UBE3A**	0.11	5.47E-01	1.00E+00	-0.52	5.00E-02	5.52E-01
**MYH4**	NA	NA	NA	4.42	5.01E-02	5.52E-01
**RFLNA**	-0.05	9.37E-01	1.00E+00	2.24	5.01E-02	5.52E-01
**AL158063.1**	-1.24	7.19E-01	1.00E+00	-3.61	5.01E-02	5.52E-01
**JAK3**	-0.03	9.47E-01	1.00E+00	1.36	5.02E-02	5.53E-01
**MKNK1**	-0.32	1.48E-01	1.00E+00	0.50	5.02E-02	5.53E-01
**HLA-DPB1**	-0.63	6.90E-02	1.00E+00	0.83	5.02E-02	5.53E-01
**METTL15P1**	-2.06	2.36E-01	1.00E+00	1.27	5.03E-02	5.53E-01
**TECPR1**	-0.09	6.69E-01	1.00E+00	1.08	5.03E-02	5.53E-01
**FH**	-0.04	7.77E-01	1.00E+00	-0.25	5.03E-02	5.53E-01
**ITM2A**	0.21	6.55E-01	1.00E+00	-0.98	5.04E-02	5.53E-01
**RP11-193H22.2**	NA	NA	NA	-4.01	5.04E-02	5.53E-01
**EI24**	0.12	4.24E-01	1.00E+00	-0.79	5.04E-02	5.53E-01
**TXNL4A**	-0.03	8.56E-01	1.00E+00	-0.91	5.04E-02	5.53E-01
**TMEM218**	-0.20	4.58E-01	1.00E+00	-0.68	5.05E-02	5.53E-01
**USP45**	-0.09	8.01E-01	1.00E+00	-0.41	5.05E-02	5.53E-01
**TMEM203**	0.14	5.13E-01	1.00E+00	-1.09	5.05E-02	5.53E-01
**NRTN**	-0.63	2.14E-01	1.00E+00	-1.34	5.05E-02	5.53E-01
**INF2**	-0.16	5.49E-01	1.00E+00	1.24	5.06E-02	5.53E-01

**POLL**	0.35	2.99E-01	1.00E+00	0.75	5.07E-02	5.54E-01
**DPEP1**	-1.24	1.23E-01	1.00E+00	1.90	5.07E-02	5.54E-01
**AL354892.2**	-0.32	7.07E-01	1.00E+00	-1.18	5.07E-02	5.54E-01
**KCNJ8**	0.45	3.86E-01	1.00E+00	0.89	5.07E-02	5.54E-01
**LKAAEAR1**	-1.14	1.87E-01	1.00E+00	-1.77	5.07E-02	5.54E-01
**SDK1**	-0.39	3.57E-01	1.00E+00	1.70	5.08E-02	5.54E-01
**IKZF1**	0.07	8.39E-01	1.00E+00	1.22	5.09E-02	5.55E-01
**AL391422.1**	1.23	1.65E-01	1.00E+00	1.73	5.09E-02	5.55E-01
**MIR34AHG**	0.65	4.28E-01	1.00E+00	1.75	5.09E-02	5.55E-01
**TRIM66**	-0.15	7.23E-01	1.00E+00	1.20	5.09E-02	5.55E-01
**PPIAP11**	1.15	3.50E-01	1.00E+00	-0.81	5.10E-02	5.55E-01
**NEUROD2**	-0.92	1.17E-01	1.00E+00	-1.38	5.10E-02	5.55E-01
**MIR4458HG**	-0.60	2.87E-02	9.87E-01	-0.98	5.11E-02	5.55E-01
**AL121749.1**	-1.86	1.83E-01	1.00E+00	-2.18	5.11E-02	5.55E-01
**KLF13**	0.16	6.21E-01	1.00E+00	1.11	5.11E-02	5.55E-01
**XLOC_004581**	-0.10	8.25E-01	1.00E+00	1.93	5.11E-02	5.55E-01
**SASH3**	-0.22	6.68E-01	1.00E+00	1.53	5.11E-02	5.55E-01
**AMZ2**	0.08	6.52E-01	1.00E+00	-0.63	5.12E-02	5.55E-01
**PON2**	-0.29	2.47E-01	1.00E+00	-0.71	5.12E-02	5.55E-01
**DPH6-DT**	-2.07	2.18E-01	1.00E+00	1.74	5.13E-02	5.55E-01
**DENND6B**	-0.02	9.40E-01	1.00E+00	1.05	5.13E-02	5.55E-01
**CLYBL**	-0.33	1.94E-01	1.00E+00	-0.55	5.13E-02	5.55E-01
**SLC4A5**	1.16	2.65E-01	1.00E+00	1.72	5.13E-02	5.55E-01
**SPATA3**	-2.93	2.25E-01	1.00E+00	-2.63	5.13E-02	5.55E-01
**SERPINB3**	-1.29	2.71E-01	1.00E+00	1.78	5.13E-02	5.55E-01
**AC134407.2**	0.31	6.01E-01	1.00E+00	1.90	5.14E-02	5.55E-01
**PEX19**	0.43	1.41E-01	1.00E+00	-0.65	5.14E-02	5.55E-01
**LMTK3**	-0.59	6.86E-02	1.00E+00	1.24	5.14E-02	5.55E-01
**ARMC1**	0.29	3.52E-01	1.00E+00	-0.36	5.14E-02	5.55E-01
**TSSC4**	-0.04	8.72E-01	1.00E+00	0.40	5.14E-02	5.55E-01
**AC090877.2**	-0.69	4.32E-01	1.00E+00	2.03	5.14E-02	5.55E-01
**ZBTB25**	-0.29	3.00E-01	1.00E+00	-0.45	5.15E-02	5.55E-01

**XLOC_012065**	-0.24	8.17E-01	1.00E+00	-1.70	5.15E-02	5.55E-01
**HTT**	0.19	3.24E-01	1.00E+00	0.89	5.15E-02	5.55E-01
**HILS1**	0.53	6.42E-01	1.00E+00	2.88	5.15E-02	5.55E-01
**SNX7**	0.09	7.23E-01	1.00E+00	-0.95	5.15E-02	5.55E-01
**STXBP6**	0.25	5.09E-01	1.00E+00	-0.68	5.16E-02	5.55E-01
**NEDD1**	0.25	3.86E-01	1.00E+00	-0.42	5.16E-02	5.55E-01
**HLA-B**	0.04	9.18E-01	1.00E+00	0.90	5.16E-02	5.55E-01
**NPIPB6**	-1.02	3.36E-01	1.00E+00	-1.61	5.17E-02	5.55E-01
**WIPF1**	0.28	4.15E-01	1.00E+00	0.99	5.17E-02	5.55E-01
**ITGAD**	0.24	8.66E-01	1.00E+00	2.99	5.17E-02	5.56E-01
**DACH1**	-0.33	4.98E-01	1.00E+00	-1.01	5.18E-02	5.56E-01
**POU2F3**	-0.12	7.67E-01	1.00E+00	-1.40	5.18E-02	5.56E-01
**DNMT3A**	-0.21	5.21E-01	1.00E+00	0.75	5.18E-02	5.56E-01
**ZNF319**	-0.12	6.71E-01	1.00E+00	1.04	5.18E-02	5.56E-01
**SPATA4**	-1.86	4.50E-01	1.00E+00	-3.56	5.19E-02	5.56E-01
**POU2F1**	0.22	4.35E-01	1.00E+00	0.45	5.19E-02	5.56E-01
**HMCES**	-0.11	4.99E-01	1.00E+00	-0.30	5.19E-02	5.56E-01
**CDKL4**	-1.08	7.35E-01	1.00E+00	-3.26	5.19E-02	5.56E-01
**PRKCE**	-0.06	8.52E-01	1.00E+00	0.88	5.20E-02	5.56E-01
**GRIN2D**	-0.20	6.33E-01	1.00E+00	2.02	5.20E-02	5.56E-01
**AC092142.1**	-2.66	3.93E-02	1.00E+00	-1.35	5.20E-02	5.56E-01
**HOXB-AS2**	-0.97	2.70E-01	1.00E+00	-1.82	5.21E-02	5.56E-01
**DLEU7**	-1.19	2.14E-01	1.00E+00	1.73	5.21E-02	5.56E-01
**SMIM4**	-0.15	6.33E-01	1.00E+00	-0.81	5.21E-02	5.56E-01
**RNU4-62P**	-0.74	6.43E-01	1.00E+00	2.51	5.21E-02	5.56E-01
**LIPT2**	-0.35	4.08E-01	1.00E+00	-0.75	5.21E-02	5.56E-01
**KMT2B**	-0.33	2.69E-01	1.00E+00	1.45	5.21E-02	5.56E-01
**IGLV3-27**	0.56	8.71E-01	1.00E+00	4.75	5.21E-02	5.56E-01
**TMEM56-RWDD3**	-0.34	8.28E-01	1.00E+00	-1.69	5.22E-02	5.56E-01
**DIS3L**	0.30	1.62E-01	1.00E+00	-0.47	5.22E-02	5.56E-01
**IL24**	-0.62	4.02E-01	1.00E+00	2.05	5.22E-02	5.56E-01
**PSME2**	-0.08	8.22E-01	1.00E+00	0.58	5.23E-02	5.56E-01

**SNTB2**	0.27	1.51E-01	1.00E+00	-0.54	5.23E-02	5.56E-01
**ATP2A1**	1.18	4.27E-01	1.00E+00	2.64	5.23E-02	5.56E-01
**AP001056.2**	0.01	9.90E-01	1.00E+00	2.04	5.24E-02	5.57E-01
**HSD17B8**	0.08	6.99E-01	1.00E+00	-0.70	5.24E-02	5.57E-01
**IL32**	-0.36	5.07E-01	1.00E+00	1.13	5.25E-02	5.58E-01
**ZMYM3**	-0.09	7.10E-01	1.00E+00	0.79	5.26E-02	5.58E-01
**PRB2**	-0.79	3.49E-01	1.00E+00	-1.69	5.26E-02	5.58E-01
**NPIPA1**	0.04	9.41E-01	1.00E+00	1.18	5.26E-02	5.58E-01
**ARID3A**	-0.08	8.56E-01	1.00E+00	1.28	5.26E-02	5.58E-01
**GVINP1**	0.48	5.20E-01	1.00E+00	1.61	5.26E-02	5.58E-01
**RNF122**	-0.39	4.20E-01	1.00E+00	1.64	5.27E-02	5.58E-01
**SYCE1L**	0.08	7.99E-01	1.00E+00	-0.69	5.27E-02	5.58E-01
**TMEM178A**	-0.16	7.43E-01	1.00E+00	-1.49	5.28E-02	5.58E-01
**PRPS1**	0.07	7.47E-01	1.00E+00	0.45	5.28E-02	5.58E-01
**RHBDD3**	-0.15	6.45E-01	1.00E+00	0.58	5.28E-02	5.58E-01
**CD3G**	-1.04	1.51E-01	1.00E+00	0.96	5.29E-02	5.58E-01
**AC106820.3**	2.85	1.96E-01	1.00E+00	-1.68	5.29E-02	5.58E-01
**ING1**	0.15	4.16E-01	1.00E+00	0.31	5.29E-02	5.58E-01
**AL122017.1**	2.12	5.34E-01	1.00E+00	-2.09	5.29E-02	5.58E-01
**AC007383.2**	-0.18	6.02E-01	1.00E+00	-0.87	5.30E-02	5.58E-01
**TIAL1**	0.13	3.79E-01	1.00E+00	0.40	5.30E-02	5.58E-01
**SGPP1**	-0.29	3.10E-01	1.00E+00	-1.01	5.30E-02	5.58E-01
**CENPBD1P1**	0.07	7.82E-01	1.00E+00	0.59	5.30E-02	5.58E-01
**AC104170.1**	1.62	1.89E-01	1.00E+00	-1.22	5.30E-02	5.58E-01
**RGS7BP**	-1.82	1.50E-02	7.62E-01	-1.46	5.30E-02	5.58E-01
**MECP2**	0.19	5.60E-01	1.00E+00	0.64	5.30E-02	5.58E-01
**CAMK2B**	-1.60	1.15E-02	6.65E-01	-1.41	5.31E-02	5.59E-01
**AC026412.3**	-0.30	6.42E-01	1.00E+00	1.21	5.31E-02	5.59E-01
**COX5A**	-0.24	1.87E-01	1.00E+00	-0.45	5.32E-02	5.59E-01
**OXCT1-AS1**	1.87	6.18E-02	1.00E+00	-1.58	5.33E-02	5.59E-01
**ACTR3B**	0.20	7.03E-01	1.00E+00	-0.85	5.33E-02	5.59E-01
**PLEKHA2**	0.25	3.31E-01	1.00E+00	0.94	5.33E-02	5.59E-01

**LY6G5C**	0.03	9.62E-01	1.00E+00	-0.92	5.33E-02	5.59E-01
**NAGA**	-0.22	2.85E-01	1.00E+00	0.49	5.33E-02	5.59E-01
**INPP5D**	-0.30	3.65E-01	1.00E+00	1.22	5.33E-02	5.59E-01
**UPB1**	1.25	2.47E-01	1.00E+00	-2.19	5.33E-02	5.59E-01
**FAM225A**	-1.23	5.39E-01	1.00E+00	2.19	5.34E-02	5.59E-01
**LINC01963**	0.67	1.38E-01	1.00E+00	-1.05	5.34E-02	5.59E-01
**PODN**	1.18	3.03E-02	1.00E+00	1.13	5.34E-02	5.59E-01
**AC011939.1**	1.19	7.31E-01	1.00E+00	-1.61	5.35E-02	5.59E-01
**YIPF2**	0.04	8.59E-01	1.00E+00	0.46	5.35E-02	5.59E-01
**PAFAH1B1**	0.14	4.50E-01	1.00E+00	0.55	5.35E-02	5.59E-01
**AC008592.3**	-0.83	1.42E-01	1.00E+00	0.73	5.36E-02	5.59E-01
**AC106047.1**	-0.31	8.37E-01	1.00E+00	-1.37	5.36E-02	5.59E-01
**XLOC_005754**	-1.34	5.84E-01	1.00E+00	-2.61	5.36E-02	5.59E-01
**TRAV9-2**	-1.22	6.45E-01	1.00E+00	1.90	5.36E-02	5.59E-01
**NIT1**	0.04	8.21E-01	1.00E+00	0.34	5.37E-02	5.59E-01
**AMZ1**	-2.98	4.80E-02	1.00E+00	1.96	5.37E-02	5.59E-01
**ZNF283**	0.22	3.65E-01	1.00E+00	-0.62	5.37E-02	5.60E-01
**SLC25A10**	0.23	5.13E-01	1.00E+00	-0.74	5.38E-02	5.60E-01
**MTMR3**	0.10	6.89E-01	1.00E+00	0.52	5.38E-02	5.60E-01
**ZNF84**	0.08	7.92E-01	1.00E+00	-0.64	5.38E-02	5.60E-01
**PQLC2**	-0.18	5.77E-01	1.00E+00	0.55	5.39E-02	5.61E-01
**LINC01242**	NA	NA	NA	-4.08	5.39E-02	5.61E-01
**HLA-DRB6**	-0.86	3.07E-01	1.00E+00	0.99	5.40E-02	5.61E-01
**SMIM19**	0.10	5.61E-01	1.00E+00	-0.83	5.40E-02	5.61E-01
**MAOA**	1.03	3.16E-03	2.95E-01	-0.85	5.40E-02	5.61E-01
**ALDH1L2**	-0.05	9.32E-01	1.00E+00	1.02	5.41E-02	5.62E-01
**RBM12**	-0.10	4.77E-01	1.00E+00	0.44	5.42E-02	5.62E-01
**TMEM52**	-1.10	2.41E-02	9.18E-01	-1.06	5.42E-02	5.62E-01
**DHX33**	-0.10	5.79E-01	1.00E+00	0.73	5.42E-02	5.62E-01
**DKK3**	-0.07	8.27E-01	1.00E+00	0.51	5.42E-02	5.62E-01
**SCML2P2**	-2.45	9.98E-02	1.00E+00	2.36	5.43E-02	5.62E-01
**TMEM161B-AS1**	-0.16	6.54E-01	1.00E+00	-0.86	5.44E-02	5.63E-01

**LINC02384**	-0.08	9.66E-01	1.00E+00	1.34	5.44E-02	5.63E-01
**G10488**	-1.79	1.48E-01	1.00E+00	-2.46	5.44E-02	5.63E-01
**AC027307.2**	0.07	8.62E-01	1.00E+00	0.82	5.44E-02	5.63E-01
**CFAP43**	-1.38	2.82E-02	9.75E-01	-1.60	5.45E-02	5.63E-01
**TRAV13-1**	0.05	9.70E-01	1.00E+00	1.95	5.45E-02	5.63E-01
**AC022210.1**	-0.86	7.00E-01	1.00E+00	-1.71	5.45E-02	5.63E-01
**WASH7P**	0.14	6.60E-01	1.00E+00	1.17	5.45E-02	5.63E-01
**TNS3**	0.66	1.88E-01	1.00E+00	1.10	5.46E-02	5.64E-01
**SPNS1**	0.36	7.69E-01	1.00E+00	1.90	5.46E-02	5.64E-01
**HMGN2P5**	0.15	6.35E-01	1.00E+00	-0.72	5.47E-02	5.64E-01
**TRBV4-1**	-2.12	3.86E-01	1.00E+00	2.39	5.47E-02	5.64E-01
**IGHV3-11**	6.41	5.39E-05	1.42E-02	4.27	5.48E-02	5.64E-01
**CNTNAP1**	-0.28	5.62E-01	1.00E+00	1.69	5.48E-02	5.64E-01
**TPD52L1**	0.08	8.11E-01	1.00E+00	-0.64	5.50E-02	5.66E-01
**SVBP**	-0.09	7.60E-01	1.00E+00	-0.78	5.51E-02	5.67E-01
**OXGR1**	0.09	9.21E-01	1.00E+00	-1.24	5.51E-02	5.67E-01
**XLOC_011298**	0.09	9.80E-01	1.00E+00	2.82	5.52E-02	5.67E-01
**TREX2**	-0.10	8.72E-01	1.00E+00	1.30	5.52E-02	5.67E-01
**IMPA2**	0.07	8.26E-01	1.00E+00	-0.97	5.52E-02	5.67E-01
**TMEM237**	0.14	3.81E-01	1.00E+00	-0.65	5.52E-02	5.67E-01
**CENPB**	-0.23	3.21E-01	1.00E+00	1.22	5.52E-02	5.67E-01
**CEND1**	-0.93	4.31E-01	1.00E+00	2.02	5.53E-02	5.67E-01
**ST8SIA2**	-3.67	2.15E-02	8.94E-01	2.95	5.54E-02	5.68E-01
**DCHS1**	0.28	4.76E-01	1.00E+00	1.63	5.54E-02	5.68E-01
**XLOC_009092**	-2.62	7.99E-02	1.00E+00	-1.84	5.55E-02	5.68E-01
**NOP9**	-0.01	9.42E-01	1.00E+00	1.05	5.55E-02	5.68E-01
**HLA-H**	-0.39	5.89E-01	1.00E+00	0.86	5.55E-02	5.68E-01
**GOLM1**	0.06	8.90E-01	1.00E+00	0.97	5.55E-02	5.68E-01
**MLLT1**	-0.21	3.92E-01	1.00E+00	1.09	5.55E-02	5.68E-01
**CNTLN**	-0.33	2.80E-01	1.00E+00	-0.33	5.56E-02	5.68E-01
**MBD6**	-0.53	6.81E-02	1.00E+00	1.41	5.56E-02	5.68E-01
**FCGR2A**	0.21	5.05E-01	1.00E+00	1.08	5.57E-02	5.68E-01

**CASC10**	-0.46	1.90E-01	1.00E+00	-0.76	5.57E-02	5.68E-01
**COX6A1P2**	-0.29	7.34E-01	1.00E+00	-1.12	5.57E-02	5.68E-01
**AL157373.2**	-0.12	9.14E-01	1.00E+00	-2.10	5.57E-02	5.68E-01
**LINC01943**	-2.78	7.45E-02	1.00E+00	1.59	5.58E-02	5.69E-01
**ZNF667-AS1**	0.51	1.59E-01	1.00E+00	-0.98	5.58E-02	5.69E-01
**SYT17**	0.39	3.41E-01	1.00E+00	-0.92	5.59E-02	5.69E-01
**HOGA1**	0.65	3.23E-01	1.00E+00	-1.24	5.59E-02	5.69E-01
**LUM**	0.63	1.99E-01	1.00E+00	0.96	5.59E-02	5.69E-01
**NFU1**	0.15	6.12E-01	1.00E+00	-0.56	5.59E-02	5.69E-01
**CHPT1**	0.44	1.70E-01	1.00E+00	-1.09	5.59E-02	5.69E-01
**DTX3**	0.15	7.19E-01	1.00E+00	0.73	5.59E-02	5.69E-01
**STRIP2**	-0.56	3.95E-01	1.00E+00	1.91	5.60E-02	5.69E-01
**DPH6**	-0.22	4.80E-01	1.00E+00	-0.58	5.60E-02	5.69E-01
**AL122058.1**	-1.56	8.10E-02	1.00E+00	-1.65	5.62E-02	5.71E-01
**AP001596.1**	0.15	9.29E-01	1.00E+00	-2.26	5.63E-02	5.71E-01
**KIF26B**	-0.38	3.99E-01	1.00E+00	1.43	5.63E-02	5.71E-01
**TTC9C**	0.05	8.30E-01	1.00E+00	0.40	5.63E-02	5.71E-01
**SMAD2**	0.09	5.28E-01	1.00E+00	-0.35	5.63E-02	5.71E-01
**NLRP3**	0.23	4.66E-01	1.00E+00	1.32	5.64E-02	5.71E-01
**XAF1**	-0.28	7.20E-01	1.00E+00	1.31	5.65E-02	5.72E-01
**RLN1**	0.09	9.56E-01	1.00E+00	-1.71	5.66E-02	5.73E-01
**LDHAP4**	-0.43	6.74E-01	1.00E+00	-1.21	5.66E-02	5.73E-01
**DYNLL2**	0.15	4.47E-01	1.00E+00	1.21	5.67E-02	5.73E-01
**ADGRG2**	0.93	2.05E-01	1.00E+00	-1.18	5.68E-02	5.74E-01
**FAM200B**	0.29	2.67E-01	1.00E+00	-0.74	5.68E-02	5.74E-01
**SCIN**	-0.36	1.94E-01	1.00E+00	-0.73	5.69E-02	5.75E-01
**GPR162**	-0.88	1.81E-01	1.00E+00	2.07	5.70E-02	5.75E-01
**ITIH3**	-1.30	1.54E-01	1.00E+00	2.24	5.70E-02	5.75E-01
**CLDN17**	1.76	4.01E-01	1.00E+00	2.28	5.70E-02	5.75E-01
**LMCD1-AS1**	-0.78	4.04E-01	1.00E+00	1.12	5.70E-02	5.75E-01
**ZSWIM7**	0.23	2.88E-01	1.00E+00	-0.49	5.70E-02	5.75E-01
**ICAM5**	-2.29	1.50E-02	7.62E-01	2.75	5.70E-02	5.75E-01

**ALDH9A1**	0.25	2.87E-01	1.00E+00	-0.29	5.71E-02	5.75E-01
**JHY**	-0.07	8.03E-01	1.00E+00	-0.68	5.71E-02	5.75E-01
**RAB44**	0.70	1.59E-01	1.00E+00	1.72	5.72E-02	5.76E-01
**PTPRC**	-0.44	4.43E-01	1.00E+00	0.99	5.72E-02	5.76E-01
**VGLL1**	1.73	4.03E-01	1.00E+00	3.88	5.72E-02	5.76E-01
**EAF1**	0.15	5.95E-01	1.00E+00	0.47	5.73E-02	5.76E-01
**ZNF138**	-0.57	1.51E-01	1.00E+00	-0.79	5.74E-02	5.77E-01
**ARL11**	0.55	2.66E-01	1.00E+00	1.26	5.75E-02	5.77E-01
**FOLH1**	-0.56	3.53E-01	1.00E+00	0.86	5.76E-02	5.78E-01
**LAMB1**	-0.09	7.74E-01	1.00E+00	1.04	5.76E-02	5.78E-01
**SRP9**	0.19	4.33E-01	1.00E+00	-0.73	5.76E-02	5.78E-01
**PEMT**	0.18	6.53E-01	1.00E+00	-0.73	5.77E-02	5.79E-01
**CCDC159**	0.11	7.71E-01	1.00E+00	0.37	5.78E-02	5.79E-01
**MERTK**	0.22	6.22E-01	1.00E+00	1.06	5.78E-02	5.79E-01
**GLT8D2**	0.39	3.63E-01	1.00E+00	0.68	5.78E-02	5.79E-01
**HNRNPA1P50**	-0.25	9.41E-01	1.00E+00	-1.97	5.78E-02	5.79E-01
**AKAP13**	-0.10	6.09E-01	1.00E+00	0.40	5.79E-02	5.79E-01
**C11orf1**	-0.22	3.33E-01	1.00E+00	-0.71	5.79E-02	5.79E-01
**C14orf28**	0.15	6.21E-01	1.00E+00	-0.64	5.79E-02	5.79E-01
**FLJ46906**	-0.28	6.86E-01	1.00E+00	0.64	5.80E-02	5.79E-01
**DESI1**	-0.24	3.96E-01	1.00E+00	0.62	5.80E-02	5.80E-01
**SLC43A3**	-0.08	7.32E-01	1.00E+00	0.49	5.81E-02	5.80E-01
**FAM47E**	-0.85	3.63E-01	1.00E+00	-1.29	5.81E-02	5.80E-01
**AC068491.4**	1.25	7.09E-01	1.00E+00	1.60	5.81E-02	5.80E-01
**ATG9B**	0.04	9.51E-01	1.00E+00	0.70	5.82E-02	5.81E-01
**LMO7-AS1**	-0.93	5.10E-01	1.00E+00	-1.24	5.83E-02	5.81E-01
**XLOC_001341**	-0.19	9.56E-01	1.00E+00	-1.98	5.83E-02	5.81E-01
**MS4A1**	-1.40	3.62E-01	1.00E+00	2.54	5.83E-02	5.81E-01
**IKBKE**	-0.03	9.28E-01	1.00E+00	0.71	5.83E-02	5.81E-01
**PYCR1**	-0.79	4.95E-02	1.00E+00	0.62	5.84E-02	5.81E-01
**HCG22**	0.92	3.26E-01	1.00E+00	2.65	5.84E-02	5.81E-01
**AC124276.1**	-0.71	5.86E-01	1.00E+00	-2.75	5.85E-02	5.81E-01

**CYP2E1**	-0.05	9.47E-01	1.00E+00	-1.28	5.85E-02	5.81E-01
**SMIM34B**	-0.89	4.77E-01	1.00E+00	1.57	5.85E-02	5.81E-01
**TMEM119**	-0.05	8.94E-01	1.00E+00	1.21	5.85E-02	5.81E-01
**LINC00672**	-1.19	3.54E-01	1.00E+00	1.77	5.85E-02	5.81E-01
**BCL9L**	-0.55	3.88E-02	1.00E+00	1.65	5.85E-02	5.81E-01
**DENND3**	0.55	1.85E-01	1.00E+00	1.44	5.86E-02	5.81E-01
**PIK3CG**	0.33	5.11E-01	1.00E+00	1.32	5.86E-02	5.81E-01
**TCEA1**	0.16	4.45E-01	1.00E+00	-0.34	5.86E-02	5.81E-01
**CRNN**	-0.69	3.14E-01	1.00E+00	1.56	5.86E-02	5.81E-01
**AL390036.1**	-0.50	7.70E-01	1.00E+00	1.36	5.87E-02	5.81E-01
**ISOC1**	0.13	5.40E-01	1.00E+00	-0.79	5.87E-02	5.81E-01
**SURF1**	-0.08	7.58E-01	1.00E+00	-0.90	5.87E-02	5.81E-01
**XLOC_004431**	0.78	4.02E-01	1.00E+00	-2.41	5.87E-02	5.81E-01
**MPC2**	0.10	6.58E-01	1.00E+00	-0.85	5.87E-02	5.81E-01
**SLC14A2**	2.27	5.18E-02	1.00E+00	-1.97	5.88E-02	5.81E-01
**AKAP3**	-0.78	4.82E-02	1.00E+00	-1.13	5.89E-02	5.81E-01
**AC023301.1**	-0.37	8.08E-01	1.00E+00	-2.60	5.89E-02	5.81E-01
**AC010624.1**	-2.85	3.19E-01	1.00E+00	2.62	5.89E-02	5.81E-01
**CBFB**	-0.02	9.33E-01	1.00E+00	-0.56	5.89E-02	5.81E-01
**HADHAP2**	-0.46	7.75E-01	1.00E+00	-1.08	5.90E-02	5.81E-01
**SKIDA1**	0.25	6.65E-01	1.00E+00	-0.82	5.90E-02	5.81E-01
**AC012360.2**	2.38	3.04E-01	1.00E+00	-2.25	5.90E-02	5.81E-01
**AL139353.1**	-0.24	8.23E-01	1.00E+00	-1.12	5.90E-02	5.81E-01
**HIST2H3DP1**	0.88	6.25E-01	1.00E+00	1.86	5.91E-02	5.81E-01
**FUT4**	0.08	8.38E-01	1.00E+00	1.48	5.91E-02	5.81E-01
**ZNF677**	0.02	9.34E-01	1.00E+00	-0.61	5.91E-02	5.81E-01
**IRS3P**	1.20	5.68E-01	1.00E+00	2.36	5.91E-02	5.81E-01
**PIWIL2**	0.66	3.16E-01	1.00E+00	-2.02	5.91E-02	5.81E-01
**DHTKD1**	0.07	6.87E-01	1.00E+00	-0.42	5.91E-02	5.81E-01
**NOCT**	-1.06	2.07E-02	8.94E-01	1.08	5.92E-02	5.81E-01
**PCDHGA12**	0.05	8.33E-01	1.00E+00	1.18	5.92E-02	5.82E-01
**AC018644.1**	0.41	8.06E-01	1.00E+00	2.50	5.93E-02	5.82E-01

**G33706**	-0.14	9.38E-01	1.00E+00	-1.08	5.94E-02	5.82E-01
**XPR1**	-0.13	5.97E-01	1.00E+00	0.62	5.94E-02	5.82E-01
**GPR107**	0.13	5.60E-01	1.00E+00	0.71	5.94E-02	5.82E-01
**ZNF91**	0.17	5.24E-01	1.00E+00	-0.65	5.94E-02	5.82E-01
**HADHB**	-0.04	8.50E-01	1.00E+00	-0.32	5.94E-02	5.82E-01
**CCDC144B**	0.90	3.60E-01	1.00E+00	-1.06	5.95E-02	5.82E-01
**MTSS1L**	-0.31	2.96E-01	1.00E+00	1.31	5.95E-02	5.82E-01
**AL450322.2**	-1.79	2.16E-01	1.00E+00	1.78	5.95E-02	5.82E-01
**XLOC_004625**	0.38	9.14E-01	1.00E+00	-3.04	5.95E-02	5.82E-01
**CLDN10**	0.18	8.87E-01	1.00E+00	-1.42	5.96E-02	5.82E-01
**BTN3A1**	0.07	8.59E-01	1.00E+00	0.79	5.96E-02	5.82E-01
**KLK14**	-0.31	5.92E-01	1.00E+00	1.22	5.96E-02	5.82E-01
**RPS15AP10**	-0.31	8.67E-01	1.00E+00	-1.86	5.97E-02	5.83E-01
**SARAF**	0.13	4.06E-01	1.00E+00	-0.37	5.97E-02	5.83E-01
**ZNF143**	0.03	8.71E-01	1.00E+00	-0.31	5.97E-02	5.83E-01
**RP11-782C8.1**	-0.63	4.57E-01	1.00E+00	-1.04	5.97E-02	5.83E-01
**ITGAX**	-0.24	7.04E-01	1.00E+00	1.70	5.98E-02	5.83E-01
**NAMPT**	-0.32	2.52E-01	1.00E+00	0.76	5.98E-02	5.83E-01
**COL6A2**	-0.02	9.54E-01	1.00E+00	1.46	5.98E-02	5.83E-01
**RAB43**	-0.34	3.91E-01	1.00E+00	1.70	5.99E-02	5.83E-01
**RBMX2**	0.06	6.87E-01	1.00E+00	0.45	5.99E-02	5.83E-01
**PRELID3A**	-0.69	1.77E-01	1.00E+00	1.41	5.99E-02	5.83E-01
**SOWAHD**	-1.48	1.70E-02	8.22E-01	2.34	5.99E-02	5.83E-01
**ANO8**	-0.26	5.91E-01	1.00E+00	1.72	6.00E-02	5.83E-01
**MGRN1**	-0.21	3.06E-01	1.00E+00	1.05	6.00E-02	5.83E-01
**UBE2R2-AS1**	1.25	3.35E-01	1.00E+00	2.52	6.01E-02	5.83E-01
**TXLNB**	0.14	7.48E-01	1.00E+00	1.27	6.01E-02	5.83E-01
**ARMH4**	-0.31	4.59E-01	1.00E+00	-0.53	6.01E-02	5.83E-01
**GULP1**	-0.07	7.91E-01	1.00E+00	-0.67	6.01E-02	5.83E-01
**CDC37L1**	0.14	4.77E-01	1.00E+00	-0.48	6.01E-02	5.83E-01
**TEKT1**	-0.89	7.93E-01	1.00E+00	2.03	6.01E-02	5.83E-01
**AL355355.1**	0.53	5.67E-01	1.00E+00	-1.66	6.02E-02	5.84E-01

**AC135977.1**	0.82	7.53E-01	1.00E+00	-1.90	6.03E-02	5.84E-01
**AL118508.1**	0.01	9.94E-01	1.00E+00	-1.60	6.03E-02	5.84E-01
**DUT**	0.19	3.38E-01	1.00E+00	-0.74	6.03E-02	5.84E-01
**KCNK12**	-0.41	6.67E-01	1.00E+00	2.27	6.04E-02	5.84E-01
**CRMP1**	0.13	7.48E-01	1.00E+00	1.09	6.04E-02	5.84E-01
**OCRL**	0.09	6.80E-01	1.00E+00	0.58	6.04E-02	5.85E-01
**MSL3**	0.39	2.27E-01	1.00E+00	0.40	6.04E-02	5.85E-01
**REEP1**	0.25	5.57E-01	1.00E+00	-1.13	6.05E-02	5.85E-01
**MEIG1**	-2.77	1.54E-01	1.00E+00	-2.20	6.06E-02	5.85E-01
**MIIP**	-0.49	8.27E-02	1.00E+00	0.39	6.06E-02	5.85E-01
**NDST1-AS1**	0.69	7.61E-01	1.00E+00	-1.48	6.06E-02	5.85E-01
**SEMA7A**	-0.26	5.94E-01	1.00E+00	1.51	6.06E-02	5.85E-01
**TMX2P1**	0.25	5.23E-01	1.00E+00	1.08	6.06E-02	5.85E-01
**WSCD2**	-0.09	8.87E-01	1.00E+00	-1.61	6.06E-02	5.85E-01
**GSPT1**	0.15	3.50E-01	1.00E+00	-0.71	6.07E-02	5.85E-01
**ABCD1**	-0.33	4.37E-01	1.00E+00	1.36	6.08E-02	5.85E-01
**SOX9-AS1**	-1.00	1.18E-01	1.00E+00	-1.12	6.08E-02	5.85E-01
**NRARP**	-1.74	2.16E-02	8.94E-01	-1.35	6.08E-02	5.85E-01
**CD300LG**	1.07	2.12E-01	1.00E+00	-1.40	6.08E-02	5.85E-01
**ZFPM2-AS1**	1.76	4.11E-01	1.00E+00	-2.33	6.08E-02	5.85E-01
**FAM111A**	0.00	9.96E-01	1.00E+00	0.41	6.08E-02	5.85E-01
**STIM2**	-0.13	5.37E-01	1.00E+00	-0.58	6.08E-02	5.85E-01
**GPR160**	0.57	3.33E-01	1.00E+00	-0.91	6.09E-02	5.85E-01
**FER1L6**	1.75	4.51E-01	1.00E+00	3.36	6.09E-02	5.85E-01
**RAB1C**	-0.58	8.67E-01	1.00E+00	1.51	6.09E-02	5.85E-01
**TMEM70**	0.08	7.54E-01	1.00E+00	-0.46	6.10E-02	5.85E-01
**AL603756.1**	1.27	4.02E-01	1.00E+00	1.53	6.10E-02	5.85E-01
**MIR4435-2HG**	0.70	7.12E-02	1.00E+00	0.94	6.10E-02	5.85E-01
**SUCLG2-AS1**	0.30	6.90E-01	1.00E+00	-1.14	6.10E-02	5.85E-01
**PCDH9**	0.38	6.08E-01	1.00E+00	-1.12	6.11E-02	5.85E-01
**TRIM34**	-1.09	6.50E-01	1.00E+00	1.46	6.11E-02	5.85E-01
**PDHA1**	0.05	8.57E-01	1.00E+00	-0.44	6.11E-02	5.85E-01

**PARVB**	0.33	3.27E-01	1.00E+00	0.73	6.11E-02	5.85E-01
**RHOT1**	0.27	4.60E-01	1.00E+00	-0.58	6.12E-02	5.85E-01
**IRS1**	0.14	7.02E-01	1.00E+00	1.00	6.12E-02	5.85E-01
**IGF2R**	0.43	1.47E-01	1.00E+00	1.18	6.12E-02	5.85E-01
**RNF13**	-0.13	4.38E-01	1.00E+00	-0.50	6.12E-02	5.85E-01
**MKRN3**	-0.24	8.05E-01	1.00E+00	-2.09	6.13E-02	5.86E-01
**DOK5**	0.78	3.31E-01	1.00E+00	0.86	6.14E-02	5.86E-01
**ALDH1A2**	0.66	3.01E-01	1.00E+00	-1.02	6.14E-02	5.86E-01
**ROS1**	0.08	9.76E-01	1.00E+00	-3.75	6.15E-02	5.86E-01
**AP001830.1**	-2.28	4.71E-02	1.00E+00	-1.43	6.15E-02	5.86E-01
**BHLHE22**	-0.55	3.30E-01	1.00E+00	1.53	6.15E-02	5.86E-01
**WASHC1**	-0.36	3.17E-01	1.00E+00	0.58	6.15E-02	5.86E-01
**XLOC_014271**	1.17	5.59E-01	1.00E+00	-2.86	6.16E-02	5.86E-01
**FOLR3**	-0.20	8.48E-01	1.00E+00	1.52	6.16E-02	5.86E-01
**PAIP1**	0.05	8.10E-01	1.00E+00	-0.57	6.16E-02	5.86E-01
**APH1A**	0.18	3.16E-01	1.00E+00	0.57	6.16E-02	5.86E-01
**SCGB3A1**	1.24	2.02E-01	1.00E+00	-1.08	6.16E-02	5.86E-01
**NKX2-5**	-3.60	3.58E-02	1.00E+00	1.39	6.17E-02	5.86E-01
**DAPK3**	-0.21	3.68E-01	1.00E+00	0.62	6.17E-02	5.86E-01
**TLR3**	0.09	8.42E-01	1.00E+00	0.67	6.17E-02	5.86E-01
**ZFHX4**	0.96	1.75E-01	1.00E+00	0.87	6.17E-02	5.86E-01
**HLA-DQA2**	-1.92	1.45E-03	2.09E-01	1.54	6.18E-02	5.87E-01
**SLC25A4**	0.02	9.65E-01	1.00E+00	-0.57	6.18E-02	5.87E-01
**AL031432.4**	-1.11	3.01E-01	1.00E+00	-1.78	6.19E-02	5.87E-01
**PMS2P1**	-0.06	8.42E-01	1.00E+00	-0.53	6.19E-02	5.87E-01
**LINC02551**	-0.97	5.61E-01	1.00E+00	2.48	6.20E-02	5.87E-01
**HLA-DOA**	-0.79	3.63E-02	1.00E+00	1.12	6.20E-02	5.87E-01
**G32185**	-0.94	7.85E-01	1.00E+00	2.67	6.20E-02	5.87E-01
**YIPF3**	-0.09	6.30E-01	1.00E+00	0.22	6.20E-02	5.87E-01
**PSD3**	0.06	8.66E-01	1.00E+00	0.54	6.20E-02	5.87E-01
**AC005041.3**	-0.73	6.46E-01	1.00E+00	1.76	6.20E-02	5.87E-01
**TRIO**	0.11	6.28E-01	1.00E+00	1.14	6.21E-02	5.87E-01

**TNRC18**	-0.50	9.07E-02	1.00E+00	1.66	6.21E-02	5.87E-01
**RRAGC**	0.13	5.43E-01	1.00E+00	0.40	6.21E-02	5.87E-01
**HYPK**	2.08	3.06E-03	2.89E-01	-1.00	6.22E-02	5.87E-01
**PAG1**	0.05	8.73E-01	1.00E+00	0.56	6.22E-02	5.87E-01
**TCTA**	0.09	7.68E-01	1.00E+00	-0.46	6.22E-02	5.87E-01
**STON2**	-0.59	2.47E-02	9.28E-01	0.50	6.22E-02	5.87E-01
**G22722**	0.47	5.21E-01	1.00E+00	-2.38	6.24E-02	5.88E-01
**G43442**	-2.32	4.29E-01	1.00E+00	2.28	6.24E-02	5.88E-01
**EEF1A1P14**	-2.72	4.11E-01	1.00E+00	1.77	6.24E-02	5.88E-01
**RMI2**	-0.09	8.18E-01	1.00E+00	-1.27	6.25E-02	5.88E-01
**IGFBP4**	0.47	2.01E-01	1.00E+00	1.33	6.25E-02	5.88E-01
**CSF1R**	-0.19	6.69E-01	1.00E+00	1.02	6.25E-02	5.88E-01
**ZNF256**	-0.36	3.11E-01	1.00E+00	-0.81	6.26E-02	5.88E-01
**FXYD2**	-0.91	6.17E-01	1.00E+00	1.58	6.26E-02	5.88E-01
**ZNF426-DT**	0.43	5.40E-01	1.00E+00	-1.21	6.26E-02	5.88E-01
**AC025034.1**	0.19	8.73E-01	1.00E+00	1.84	6.26E-02	5.88E-01
**NUDT12**	-0.04	9.02E-01	1.00E+00	-0.51	6.27E-02	5.88E-01
**CALCR**	-1.29	5.09E-01	1.00E+00	2.54	6.27E-02	5.88E-01
**MAP4K3**	0.26	3.20E-01	1.00E+00	-0.66	6.27E-02	5.88E-01
**XLOC_007094**	0.58	8.01E-01	1.00E+00	-2.53	6.27E-02	5.88E-01
**AC092881.1**	2.87	1.92E-01	1.00E+00	-2.62	6.27E-02	5.88E-01
**MTATP6P1**	0.07	9.21E-01	1.00E+00	-1.07	6.27E-02	5.88E-01
**ABCG1**	-0.01	9.68E-01	1.00E+00	0.88	6.28E-02	5.88E-01
**AC015802.3**	-2.55	1.83E-01	1.00E+00	-1.54	6.28E-02	5.89E-01
**STK16**	-0.14	4.61E-01	1.00E+00	0.39	6.29E-02	5.89E-01
**TANC2**	-0.35	1.34E-01	1.00E+00	0.89	6.29E-02	5.89E-01
**NDUFB2**	0.27	2.12E-01	1.00E+00	-1.03	6.30E-02	5.89E-01
**HNRNPKP2**	-1.68	6.27E-01	1.00E+00	-0.95	6.30E-02	5.89E-01
**CCDC18-AS1**	0.14	7.69E-01	1.00E+00	0.61	6.30E-02	5.89E-01
**ZNF772**	0.49	2.25E-01	1.00E+00	-0.85	6.31E-02	5.89E-01
**AC087501.4**	-0.69	4.45E-01	1.00E+00	0.93	6.31E-02	5.89E-01
**CCDC88B**	-0.15	7.71E-01	1.00E+00	1.70	6.31E-02	5.89E-01

**ZNF676**	-1.28	2.66E-01	1.00E+00	-1.52	6.31E-02	5.89E-01
**ACKR2**	0.05	9.21E-01	1.00E+00	1.13	6.31E-02	5.89E-01
**ZNF879**	0.26	4.82E-01	1.00E+00	-0.74	6.32E-02	5.90E-01
**NMRK1**	0.25	1.78E-01	1.00E+00	-0.45	6.33E-02	5.90E-01
**NANOS3**	0.00	9.97E-01	1.00E+00	-1.40	6.34E-02	5.91E-01
**CCL4**	-0.94	2.33E-01	1.00E+00	1.22	6.34E-02	5.91E-01
**SHLD2P1**	1.43	5.10E-01	1.00E+00	1.23	6.34E-02	5.91E-01
**SUSD6**	0.00	9.93E-01	1.00E+00	0.61	6.35E-02	5.91E-01
**TRADD**	-0.28	3.05E-01	1.00E+00	0.38	6.35E-02	5.91E-01
**MCCC2**	0.02	9.17E-01	1.00E+00	-0.34	6.35E-02	5.91E-01
**LAPTM4B**	-0.05	8.20E-01	1.00E+00	-0.88	6.36E-02	5.91E-01
**AC112220.2**	0.18	5.57E-01	1.00E+00	0.50	6.36E-02	5.91E-01
**NDUFAF8**	-0.34	1.10E-01	1.00E+00	-0.60	6.36E-02	5.91E-01
**AC092140.2**	-2.28	3.16E-01	1.00E+00	-2.10	6.37E-02	5.91E-01
**RANBP1**	-0.22	2.67E-01	1.00E+00	-0.58	6.37E-02	5.91E-01
**TPI1**	-0.13	2.70E-01	1.00E+00	0.32	6.37E-02	5.91E-01
**HMCN1**	-0.15	6.86E-01	1.00E+00	1.17	6.37E-02	5.91E-01
**RPS10**	-1.93	3.78E-01	1.00E+00	-1.16	6.37E-02	5.91E-01
**ZNF22**	0.38	1.77E-01	1.00E+00	-0.68	6.38E-02	5.91E-01
**TRAF3IP2-AS1**	0.55	1.81E-01	1.00E+00	-0.81	6.38E-02	5.91E-01
**LINC01235**	0.55	5.78E-01	1.00E+00	1.06	6.38E-02	5.91E-01
**IL4R**	-0.25	4.62E-01	1.00E+00	1.27	6.39E-02	5.91E-01
**PLSCR2**	0.68	7.24E-01	1.00E+00	1.84	6.39E-02	5.91E-01
**C1R**	0.52	1.96E-01	1.00E+00	0.96	6.39E-02	5.91E-01
**CHST14**	0.21	5.00E-01	1.00E+00	1.18	6.39E-02	5.91E-01
**STARD4-AS1**	-0.64	4.66E-01	1.00E+00	1.23	6.40E-02	5.91E-01
**CTSH**	-0.46	1.74E-01	1.00E+00	0.65	6.40E-02	5.91E-01
**DDX25**	-0.13	8.26E-01	1.00E+00	-1.22	6.40E-02	5.91E-01
**ANKRD31**	0.00	9.98E-01	1.00E+00	1.53	6.42E-02	5.93E-01
**RAB30-AS1**	-0.02	9.65E-01	1.00E+00	-0.87	6.43E-02	5.93E-01
**APBA2**	-0.94	3.78E-02	1.00E+00	1.44	6.43E-02	5.93E-01
**TAPBPL**	-0.19	4.93E-01	1.00E+00	0.48	6.43E-02	5.93E-01

**TTLL3**	0.52	2.61E-01	1.00E+00	0.74	6.44E-02	5.93E-01
**NTSR1**	-2.25	1.55E-02	7.76E-01	2.26	6.44E-02	5.93E-01
**AL133243.3**	-0.39	6.29E-01	1.00E+00	0.95	6.44E-02	5.93E-01
**CHRFAM7A**	1.41	1.43E-01	1.00E+00	1.40	6.44E-02	5.93E-01
**C3orf86**	-2.36	3.95E-01	1.00E+00	2.01	6.45E-02	5.93E-01
**HACD3**	0.16	5.58E-01	1.00E+00	-0.40	6.45E-02	5.93E-01
**AL360181.2**	-2.03	1.89E-01	1.00E+00	1.83	6.45E-02	5.93E-01
**AC125807.2**	-0.30	3.95E-01	1.00E+00	1.34	6.45E-02	5.93E-01
**XLOC_008935**	-0.18	8.31E-01	1.00E+00	1.77	6.45E-02	5.93E-01
**PAXIP1-AS2**	0.04	9.27E-01	1.00E+00	0.48	6.45E-02	5.93E-01
**XLOC_010389**	2.47	4.68E-01	1.00E+00	-2.81	6.45E-02	5.93E-01
**EPM2A**	0.03	9.31E-01	1.00E+00	-0.77	6.46E-02	5.93E-01
**KIF5C**	-1.26	4.09E-02	1.00E+00	1.16	6.46E-02	5.93E-01
**THG1L**	0.06	8.22E-01	1.00E+00	0.53	6.47E-02	5.94E-01
**AHRR**	0.11	7.79E-01	1.00E+00	1.28	6.47E-02	5.94E-01
**RAB42**	0.46	4.34E-01	1.00E+00	1.39	6.47E-02	5.94E-01
**AC099850.3**	-0.37	6.00E-01	1.00E+00	1.69	6.48E-02	5.94E-01
**NCKAP5**	-0.23	2.63E-01	1.00E+00	0.58	6.48E-02	5.94E-01
**CAHM**	-1.24	1.66E-01	1.00E+00	-1.50	6.49E-02	5.94E-01
**MTCO2P2**	-1.34	6.97E-01	1.00E+00	-1.57	6.49E-02	5.94E-01
**AL162586.1**	-0.86	4.93E-01	1.00E+00	1.69	6.49E-02	5.94E-01
**MIR3142HG**	0.32	8.52E-01	1.00E+00	1.98	6.50E-02	5.94E-01
**KYAT1**	-0.01	9.73E-01	1.00E+00	-0.86	6.50E-02	5.94E-01
**IFI44**	-0.11	8.85E-01	1.00E+00	1.23	6.50E-02	5.94E-01
**AL157996.1**	0.26	7.58E-01	1.00E+00	-2.51	6.50E-02	5.94E-01
**FAM227B**	-0.15	5.84E-01	1.00E+00	-0.75	6.50E-02	5.94E-01
**AP000229.1**	-2.47	9.29E-02	1.00E+00	-1.78	6.50E-02	5.94E-01
**IL1A**	0.15	8.61E-01	1.00E+00	1.89	6.51E-02	5.94E-01
**FAM122A**	-0.02	8.71E-01	1.00E+00	-0.61	6.51E-02	5.94E-01
**AC139495.3**	-0.46	7.72E-01	1.00E+00	1.95	6.51E-02	5.94E-01
**AL513283.1**	-1.61	1.29E-02	7.15E-01	-0.74	6.51E-02	5.94E-01
**ST3GAL1**	0.30	5.75E-01	1.00E+00	0.89	6.52E-02	5.94E-01

**ZNF812P**	1.12	5.22E-01	1.00E+00	1.84	6.52E-02	5.94E-01
**GTF2A2**	0.16	3.51E-01	1.00E+00	-0.46	6.52E-02	5.94E-01
**CORO7**	-0.30	6.36E-01	1.00E+00	0.88	6.52E-02	5.94E-01
**MISP3**	-0.25	3.43E-01	1.00E+00	-0.70	6.53E-02	5.95E-01
**PIM2**	-0.17	5.45E-01	1.00E+00	0.70	6.53E-02	5.95E-01
**UNC5B**	-0.52	2.31E-01	1.00E+00	0.89	6.54E-02	5.95E-01
**AC009318.1**	1.00	1.67E-01	1.00E+00	1.56	6.54E-02	5.95E-01
**ACO1**	0.66	2.63E-01	1.00E+00	-0.93	6.54E-02	5.95E-01
**USP35**	-0.27	4.18E-01	1.00E+00	1.53	6.54E-02	5.95E-01
**ENPP4**	0.38	5.17E-01	1.00E+00	-0.79	6.54E-02	5.95E-01
**FAAH**	-0.12	6.56E-01	1.00E+00	-1.04	6.55E-02	5.95E-01
**KIF16B**	-0.03	8.96E-01	1.00E+00	-0.31	6.57E-02	5.96E-01
**SOD2**	0.37	2.62E-01	1.00E+00	0.89	6.57E-02	5.96E-01
**APPL1**	-0.01	9.51E-01	1.00E+00	-0.36	6.57E-02	5.96E-01
**KCTD12**	0.30	4.29E-01	1.00E+00	0.90	6.58E-02	5.97E-01
**CSF2RB**	-0.30	4.84E-01	1.00E+00	1.48	6.58E-02	5.97E-01
**PRELID2P1**	2.53	3.41E-01	1.00E+00	-1.48	6.59E-02	5.97E-01
**CRYGN**	-2.22	2.54E-01	1.00E+00	2.71	6.59E-02	5.97E-01
**AC009407.1**	0.17	9.12E-01	1.00E+00	-2.21	6.59E-02	5.97E-01
**RPAP3**	-0.10	7.00E-01	1.00E+00	-0.44	6.59E-02	5.97E-01
**TRABD2A**	0.63	3.10E-01	1.00E+00	1.19	6.60E-02	5.97E-01
**TMPRSS4**	-2.01	1.11E-01	1.00E+00	1.40	6.60E-02	5.97E-01
**HTRA4**	-2.30	2.36E-01	1.00E+00	3.64	6.60E-02	5.97E-01
**AC093668.2**	-0.93	3.80E-01	1.00E+00	0.93	6.61E-02	5.97E-01
**AC000061.1**	-1.72	1.32E-01	1.00E+00	-2.04	6.61E-02	5.97E-01
**SLC25A34**	0.40	6.52E-01	1.00E+00	-1.76	6.61E-02	5.97E-01
**MLIP**	-0.42	2.90E-01	1.00E+00	-0.58	6.62E-02	5.97E-01
**GPN3**	0.27	1.49E-01	1.00E+00	-0.62	6.62E-02	5.98E-01
**CALN1**	-0.18	6.59E-01	1.00E+00	-1.00	6.63E-02	5.98E-01
**SDHAP3**	1.67	6.45E-04	1.20E-01	0.98	6.63E-02	5.98E-01
**CYTIP**	-0.33	5.35E-01	1.00E+00	0.92	6.63E-02	5.98E-01
**DOK2**	-0.02	9.61E-01	1.00E+00	1.17	6.64E-02	5.98E-01

**AL390198.1**	0.39	9.00E-01	1.00E+00	-1.58	6.65E-02	5.98E-01
**ITGAE**	0.20	4.61E-01	1.00E+00	-0.83	6.65E-02	5.98E-01
**EIF2D**	-0.16	5.13E-01	1.00E+00	-0.24	6.65E-02	5.98E-01
**ZBTB20-AS1**	-0.80	8.18E-01	1.00E+00	-2.34	6.65E-02	5.98E-01
**ROR2**	-0.35	2.84E-01	1.00E+00	1.07	6.65E-02	5.98E-01
**CRABP1**	-1.29	9.52E-03	6.06E-01	-1.46	6.65E-02	5.98E-01
**MRPS35**	0.03	8.42E-01	1.00E+00	-0.34	6.66E-02	5.98E-01
**G5386**	-1.33	1.03E-01	1.00E+00	0.89	6.66E-02	5.98E-01
**AC008906.1**	-2.59	1.02E-01	1.00E+00	1.38	6.66E-02	5.98E-01
**STAG3L4**	0.41	4.49E-01	1.00E+00	-0.66	6.67E-02	5.98E-01
**COMMD7**	0.20	3.78E-01	1.00E+00	-0.67	6.67E-02	5.98E-01
**XPNPEP1**	0.05	7.81E-01	1.00E+00	0.28	6.67E-02	5.98E-01
**TSPAN13**	0.50	1.84E-01	1.00E+00	-0.60	6.67E-02	5.98E-01
**AC005884.2**	NA	NA	NA	-2.25	6.67E-02	5.98E-01
**THPO**	-0.23	8.23E-01	1.00E+00	1.91	6.67E-02	5.98E-01
**TMEM169**	-0.31	5.46E-01	1.00E+00	1.91	6.68E-02	5.99E-01
**SLC29A3**	0.06	8.73E-01	1.00E+00	0.92	6.70E-02	5.99E-01
**RAB6B**	-0.26	7.12E-01	1.00E+00	0.80	6.70E-02	5.99E-01
**LRRC57**	0.14	5.37E-01	1.00E+00	-0.53	6.70E-02	5.99E-01
**B3GNT3**	-0.09	8.91E-01	1.00E+00	1.49	6.70E-02	5.99E-01
**SLA**	-0.21	6.57E-01	1.00E+00	1.12	6.70E-02	5.99E-01
**AL359504.2**	1.54	5.51E-01	1.00E+00	-2.09	6.71E-02	5.99E-01
**CACNA1B**	-0.29	9.07E-01	1.00E+00	2.18	6.71E-02	5.99E-01
**C22orf39**	0.23	4.16E-01	1.00E+00	-0.59	6.71E-02	5.99E-01
**LINC02577**	-0.58	8.67E-01	1.00E+00	3.06	6.71E-02	5.99E-01
**IKZF2**	0.05	9.23E-01	1.00E+00	-0.88	6.72E-02	5.99E-01
**AL356968.2**	NA	NA	NA	-1.75	6.72E-02	5.99E-01
**AREGB**	-1.52	6.60E-01	1.00E+00	2.20	6.72E-02	5.99E-01
**CFAP221**	-0.30	7.56E-01	1.00E+00	-1.70	6.73E-02	5.99E-01
**BAX**	-0.25	3.07E-01	1.00E+00	0.87	6.73E-02	5.99E-01
**GOT2**	-0.18	2.39E-01	1.00E+00	-0.28	6.73E-02	5.99E-01
**KPRP**	0.28	6.85E-01	1.00E+00	1.46	6.73E-02	5.99E-01

**HBG2**	0.00	1.00E+00	NA	2.31	6.74E-02	5.99E-01
**AC060766.4**	-0.53	6.34E-01	1.00E+00	1.58	6.74E-02	5.99E-01
**RIT1**	0.13	5.46E-01	1.00E+00	0.45	6.75E-02	5.99E-01
**FADS1**	1.01	1.60E-01	1.00E+00	-1.19	6.76E-02	5.99E-01
**FLJ42351**	-0.96	7.61E-01	1.00E+00	2.47	6.77E-02	5.99E-01
**LINC00345**	1.42	4.53E-01	1.00E+00	2.05	6.77E-02	5.99E-01
**IMPACT**	-0.08	6.56E-01	1.00E+00	-0.40	6.77E-02	5.99E-01
**NOL12**	0.72	4.91E-01	1.00E+00	0.76	6.77E-02	5.99E-01
**ECI2**	0.21	5.75E-01	1.00E+00	-0.78	6.77E-02	5.99E-01
**CCDC88A**	-0.25	5.05E-01	1.00E+00	0.71	6.78E-02	5.99E-01
**MPO**	0.18	8.88E-01	1.00E+00	1.94	6.78E-02	5.99E-01
**TMCC1**	0.02	9.37E-01	1.00E+00	0.32	6.78E-02	5.99E-01
**ACTR1B**	0.09	6.33E-01	1.00E+00	0.53	6.78E-02	5.99E-01
**SDC3**	-0.17	6.31E-01	1.00E+00	1.42	6.78E-02	5.99E-01
**GALR1**	1.68	1.00E-01	1.00E+00	-2.18	6.78E-02	5.99E-01
**SIRT6**	-0.15	5.26E-01	1.00E+00	1.11	6.78E-02	5.99E-01
**PBX2**	-0.17	5.40E-01	1.00E+00	1.30	6.79E-02	5.99E-01
**PDCD4**	-0.21	4.02E-01	1.00E+00	-0.53	6.79E-02	5.99E-01
**PEX11A**	0.67	1.23E-01	1.00E+00	-0.89	6.79E-02	5.99E-01
**IGLV1-36**	2.17	3.93E-01	1.00E+00	4.37	6.79E-02	5.99E-01
**SMIM25**	0.21	7.56E-01	1.00E+00	1.27	6.80E-02	5.99E-01
**BTN3A2**	-0.09	8.53E-01	1.00E+00	0.90	6.80E-02	5.99E-01
**HSPA1A**	0.06	8.04E-01	1.00E+00	-0.72	6.80E-02	5.99E-01
**REEP5**	0.12	4.20E-01	1.00E+00	-0.42	6.80E-02	5.99E-01
**CHI3L2**	0.19	8.29E-01	1.00E+00	1.17	6.80E-02	5.99E-01
**PRKCQ**	0.06	9.30E-01	1.00E+00	2.25	6.80E-02	5.99E-01
**MEX3B**	0.20	6.76E-01	1.00E+00	1.65	6.80E-02	5.99E-01
**RPSAP52**	0.05	9.89E-01	1.00E+00	2.73	6.81E-02	5.99E-01
**CDK11B**	-0.12	6.00E-01	1.00E+00	0.82	6.81E-02	5.99E-01
**PSME4**	0.06	7.62E-01	1.00E+00	0.72	6.81E-02	5.99E-01
**IL11**	1.18	3.49E-01	1.00E+00	2.32	6.81E-02	5.99E-01
**DBI**	0.26	2.64E-01	1.00E+00	-0.72	6.81E-02	5.99E-01

**AC090559.1**	-0.56	2.23E-01	1.00E+00	1.09	6.81E-02	5.99E-01
**TNIK**	-0.24	5.02E-01	1.00E+00	1.40	6.82E-02	5.99E-01
**GMPR**	0.22	5.96E-01	1.00E+00	0.76	6.82E-02	5.99E-01
**ITGB2-AS1**	-2.17	3.50E-02	1.00E+00	1.25	6.82E-02	5.99E-01
**HACD2**	0.41	1.57E-01	1.00E+00	-0.77	6.82E-02	5.99E-01
**FOXO1**	0.02	9.41E-01	1.00E+00	-0.74	6.82E-02	5.99E-01
**G23007**	-1.31	2.23E-01	1.00E+00	-2.62	6.83E-02	5.99E-01
**NCKAP1L**	0.08	8.72E-01	1.00E+00	1.19	6.83E-02	5.99E-01
**DPF1**	-2.05	8.84E-04	1.50E-01	1.63	6.83E-02	5.99E-01
**XKR8**	0.03	9.07E-01	1.00E+00	0.64	6.83E-02	5.99E-01
**AC012615.6**	-0.94	3.90E-01	1.00E+00	3.43	6.84E-02	5.99E-01
**TLCD1**	-0.37	4.11E-01	1.00E+00	-1.00	6.84E-02	5.99E-01
**IGHV2-26**	3.55	2.90E-01	1.00E+00	3.76	6.84E-02	5.99E-01
**ZNF740**	0.51	9.62E-02	1.00E+00	1.17	6.84E-02	5.99E-01
**TRAF3IP3**	-0.71	2.50E-01	1.00E+00	1.35	6.84E-02	5.99E-01
**AC005343.1**	2.75	3.77E-01	1.00E+00	-1.79	6.85E-02	5.99E-01
**C4orf48**	-0.34	4.45E-01	1.00E+00	0.64	6.85E-02	5.99E-01
**ZNF284**	0.32	7.16E-01	1.00E+00	-0.96	6.85E-02	5.99E-01
**TNFRSF14**	-0.14	6.44E-01	1.00E+00	1.02	6.85E-02	5.99E-01
**AC104232.2**	NA	NA	NA	-3.78	6.85E-02	5.99E-01
**CLUL1**	-1.33	1.16E-01	1.00E+00	-1.85	6.86E-02	5.99E-01
**EIF1AX**	0.16	5.42E-01	1.00E+00	-0.32	6.86E-02	5.99E-01
**SLC2A1-AS1**	-0.12	8.90E-01	1.00E+00	-1.06	6.86E-02	5.99E-01
**DTX3L**	0.16	7.58E-01	1.00E+00	0.80	6.86E-02	5.99E-01
**SCAND1**	-0.12	6.89E-01	1.00E+00	0.40	6.87E-02	5.99E-01
**CCL3**	-0.59	8.28E-01	1.00E+00	1.79	6.88E-02	6.00E-01
**RNF219**	0.21	5.18E-01	1.00E+00	-0.48	6.88E-02	6.00E-01
**NEURL1B**	0.30	2.29E-01	1.00E+00	0.88	6.89E-02	6.00E-01
**LINC01532**	2.53	4.50E-01	1.00E+00	-4.11	6.90E-02	6.00E-01
**ACYP2**	-0.04	9.09E-01	1.00E+00	-0.69	6.90E-02	6.00E-01
**ADGRE4P**	-1.74	5.16E-01	1.00E+00	3.21	6.90E-02	6.00E-01
**KIFC3**	-0.26	2.94E-01	1.00E+00	0.64	6.90E-02	6.00E-01

**GTF2H5**	0.27	2.67E-01	1.00E+00	-0.52	6.90E-02	6.00E-01
**BIN3**	-0.10	6.45E-01	1.00E+00	0.38	6.91E-02	6.00E-01
**TGFB3**	-0.29	5.57E-01	1.00E+00	0.82	6.91E-02	6.00E-01
**ARMCX5-GPRASP2**	0.11	7.95E-01	1.00E+00	-0.69	6.91E-02	6.00E-01
**GPR182**	0.52	5.63E-01	1.00E+00	-2.21	6.92E-02	6.00E-01
**PIGG**	-0.22	2.60E-01	1.00E+00	0.36	6.92E-02	6.00E-01
**LINC02325**	-0.45	8.08E-01	1.00E+00	2.28	6.92E-02	6.00E-01
**EVA1A**	-0.26	6.80E-01	1.00E+00	0.76	6.92E-02	6.00E-01
**C2orf81**	0.64	4.71E-01	1.00E+00	0.84	6.92E-02	6.00E-01
**ALKAL2**	0.59	3.58E-01	1.00E+00	-1.43	6.92E-02	6.00E-01
**GRAMD1A**	-0.24	4.76E-01	1.00E+00	1.22	6.92E-02	6.00E-01
**MRPS30-DT**	0.41	4.19E-01	1.00E+00	-1.07	6.93E-02	6.00E-01
**DPP8**	0.00	9.93E-01	1.00E+00	-0.33	6.93E-02	6.00E-01
**HK3**	0.39	6.12E-01	1.00E+00	2.14	6.93E-02	6.00E-01
**PRRC2A**	-0.38	1.01E-01	1.00E+00	1.34	6.93E-02	6.00E-01
**CCL7**	3.99	1.97E-01	1.00E+00	3.19	6.94E-02	6.00E-01
**RP11-439A17.9**	-0.62	4.08E-01	1.00E+00	1.95	6.94E-02	6.00E-01
**VKORC1L1**	0.91	4.25E-02	1.00E+00	-0.72	6.94E-02	6.00E-01
**NEBL**	-0.08	7.50E-01	1.00E+00	-0.72	6.95E-02	6.00E-01
**AXL**	0.44	2.87E-01	1.00E+00	0.70	6.95E-02	6.00E-01
**AC023024.2**	1.01	3.25E-01	1.00E+00	2.10	6.95E-02	6.00E-01
**CD226**	0.08	9.07E-01	1.00E+00	1.86	6.96E-02	6.00E-01
**AC106738.1**	0.90	4.77E-01	1.00E+00	-1.43	6.96E-02	6.00E-01
**CWF19L2**	0.41	1.16E-01	1.00E+00	0.42	6.96E-02	6.00E-01
**TMED9**	-0.05	7.80E-01	1.00E+00	0.59	6.96E-02	6.00E-01
**EIF4A1**	0.51	6.37E-01	1.00E+00	0.89	6.96E-02	6.00E-01
**CLEC4D**	3.27	3.32E-01	1.00E+00	2.60	6.96E-02	6.00E-01
**P2RX4**	-0.05	8.68E-01	1.00E+00	0.64	6.97E-02	6.01E-01
**WBP1L**	0.32	1.48E-01	1.00E+00	0.50	6.98E-02	6.01E-01
**PDCD2**	-0.11	5.31E-01	1.00E+00	-0.56	6.98E-02	6.01E-01
**G25988**	-2.54	1.89E-01	1.00E+00	2.20	6.98E-02	6.01E-01
**CAPZB**	-0.11	4.43E-01	1.00E+00	0.33	6.98E-02	6.01E-01

**WRB**	0.46	1.95E-01	1.00E+00	-0.90	6.99E-02	6.01E-01
**SNHG25**	-1.43	3.50E-01	1.00E+00	-1.10	6.99E-02	6.01E-01
**CCDC86**	-0.21	5.17E-01	1.00E+00	0.45	6.99E-02	6.01E-01
**TSHZ3**	-0.33	3.82E-01	1.00E+00	1.15	7.00E-02	6.01E-01
**AC079298.3**	0.16	9.12E-01	1.00E+00	-1.73	7.00E-02	6.01E-01
**JPT1**	-0.20	5.37E-01	1.00E+00	0.40	7.00E-02	6.01E-01
**AC106882.1**	-0.64	8.54E-01	1.00E+00	-3.03	7.01E-02	6.01E-01
**CD47**	0.17	5.09E-01	1.00E+00	0.36	7.01E-02	6.01E-01
**AP000356.1**	0.08	9.35E-01	1.00E+00	1.34	7.01E-02	6.01E-01
**NR1H2**	-0.01	9.50E-01	1.00E+00	0.40	7.02E-02	6.02E-01
**BCYRN1**	-0.03	9.75E-01	1.00E+00	1.36	7.03E-02	6.02E-01
**NR2E1**	-4.07	3.22E-02	1.00E+00	-2.48	7.04E-02	6.03E-01
**AP001062.1**	-0.03	9.72E-01	1.00E+00	-0.98	7.04E-02	6.04E-01
**XLOC_000974**	-2.17	9.27E-02	1.00E+00	-2.34	7.05E-02	6.04E-01
**ADAM32**	0.42	5.87E-01	1.00E+00	-1.70	7.06E-02	6.04E-01
**SIGLEC17P**	0.28	6.90E-01	1.00E+00	1.27	7.06E-02	6.04E-01
**AC239804.1**	0.31	8.20E-01	1.00E+00	1.91	7.06E-02	6.04E-01
**TNC**	-1.19	1.60E-02	7.93E-01	1.25	7.06E-02	6.04E-01
**ATP5PF**	0.18	4.39E-01	1.00E+00	-0.99	7.07E-02	6.04E-01
**DNAJC5B**	-0.93	7.87E-01	1.00E+00	2.79	7.07E-02	6.04E-01
**CERS3-AS1**	-0.78	1.21E-01	1.00E+00	-0.82	7.07E-02	6.04E-01
**SLC39A11**	0.05	8.13E-01	1.00E+00	0.38	7.08E-02	6.05E-01
**AC097534.2**	0.18	6.88E-01	1.00E+00	-0.77	7.08E-02	6.05E-01
**SLC26A4-AS1**	-2.87	1.52E-01	1.00E+00	2.70	7.09E-02	6.05E-01
**ZNF85**	0.19	5.71E-01	1.00E+00	-0.60	7.09E-02	6.05E-01
**RAB20**	-0.01	9.83E-01	1.00E+00	1.26	7.09E-02	6.05E-01
**AC073332.1**	-1.04	3.31E-01	1.00E+00	-0.98	7.11E-02	6.06E-01
**TRBV24-1**	0.26	9.39E-01	1.00E+00	1.95	7.11E-02	6.06E-01
**RPS24P8**	-0.58	7.95E-01	1.00E+00	-0.98	7.11E-02	6.06E-01
**ANPEP**	0.15	6.68E-01	1.00E+00	1.20	7.11E-02	6.06E-01
**TRBV6-5**	-2.95	3.84E-02	1.00E+00	1.97	7.12E-02	6.06E-01
**AL135791.1**	1.27	2.60E-01	1.00E+00	-2.06	7.12E-02	6.06E-01

**URI1**	0.11	5.56E-01	1.00E+00	-0.57	7.12E-02	6.06E-01
**XLOC_005552**	-0.61	5.96E-01	1.00E+00	2.05	7.13E-02	6.06E-01
**HMOX2**	-0.26	1.44E-01	1.00E+00	0.28	7.14E-02	6.06E-01
**ASPH**	0.72	1.56E-01	1.00E+00	-0.79	7.14E-02	6.06E-01
**DNAJB14**	0.05	8.62E-01	1.00E+00	-0.32	7.14E-02	6.06E-01
**PRADC1**	-0.06	7.85E-01	1.00E+00	-0.54	7.14E-02	6.06E-01
**CTSC**	-0.27	4.35E-01	1.00E+00	0.74	7.14E-02	6.06E-01
**XLOC_003221**	-0.27	6.17E-01	1.00E+00	-1.48	7.14E-02	6.06E-01
**SLC12A5**	-0.80	8.09E-01	1.00E+00	1.66	7.14E-02	6.06E-01
**CDC73**	0.12	5.71E-01	1.00E+00	-0.38	7.15E-02	6.06E-01
**AL139125.2**	-0.49	8.43E-01	1.00E+00	3.02	7.15E-02	6.06E-01
**CHD3**	-0.11	7.38E-01	1.00E+00	1.04	7.15E-02	6.06E-01
**QDPR**	0.53	2.58E-01	1.00E+00	-0.60	7.16E-02	6.06E-01
**8-Sep**	0.01	9.87E-01	1.00E+00	0.62	7.16E-02	6.06E-01
**ZNF791**	0.18	3.47E-01	1.00E+00	-0.29	7.16E-02	6.06E-01
**AL133346.1**	-2.73	4.13E-02	1.00E+00	-1.76	7.17E-02	6.06E-01
**C1S**	0.53	2.07E-01	1.00E+00	0.92	7.17E-02	6.06E-01
**XAB2**	0.02	8.93E-01	1.00E+00	0.61	7.17E-02	6.06E-01
**PRKCSH**	-0.18	4.53E-01	1.00E+00	0.69	7.17E-02	6.07E-01
**RNU2-6P**	NA	NA	NA	-1.61	7.18E-02	6.07E-01
**HLA-C**	0.02	9.53E-01	1.00E+00	0.73	7.18E-02	6.07E-01
**RGN**	0.80	1.86E-01	1.00E+00	-1.01	7.19E-02	6.07E-01
**AC064836.3**	-0.09	7.83E-01	1.00E+00	-0.90	7.19E-02	6.07E-01
**PI4KAP1**	0.28	6.52E-01	1.00E+00	1.31	7.20E-02	6.07E-01
**PCGF2**	-0.40	2.19E-01	1.00E+00	1.25	7.20E-02	6.07E-01
**AGAP14P**	0.56	8.71E-01	1.00E+00	-2.39	7.20E-02	6.07E-01
**IMMP1L**	0.41	1.37E-01	1.00E+00	-0.67	7.20E-02	6.07E-01
**MKNK1-AS1**	0.09	9.76E-01	1.00E+00	-2.72	7.21E-02	6.07E-01
**BTN2A3P**	-0.37	6.20E-01	1.00E+00	0.95	7.21E-02	6.07E-01
**CLSTN3**	-0.22	5.61E-01	1.00E+00	0.94	7.21E-02	6.07E-01
**NHEG1**	0.31	7.16E-01	1.00E+00	-1.67	7.21E-02	6.07E-01
**CYP2F2P**	0.23	8.56E-01	1.00E+00	-1.10	7.21E-02	6.07E-01

**STRAP**	0.08	5.93E-01	1.00E+00	-0.31	7.22E-02	6.07E-01
**MBLAC2**	-0.12	5.74E-01	1.00E+00	-0.58	7.22E-02	6.07E-01
**AC011591.2**	-5.88	2.41E-02	9.18E-01	-1.68	7.22E-02	6.07E-01
**LGR4**	0.02	9.16E-01	1.00E+00	-0.69	7.22E-02	6.07E-01
**GALE**	0.02	9.30E-01	1.00E+00	0.37	7.22E-02	6.07E-01
**GPR132**	0.10	8.63E-01	1.00E+00	1.55	7.22E-02	6.07E-01
**AL442663.3**	-0.63	7.52E-01	1.00E+00	1.65	7.24E-02	6.07E-01
**KCNJ16**	1.18	4.32E-01	1.00E+00	-1.42	7.24E-02	6.07E-01
**AL590068.3**	0.36	9.17E-01	1.00E+00	3.80	7.24E-02	6.07E-01
**RAB40A**	-0.24	9.19E-01	1.00E+00	2.11	7.24E-02	6.07E-01
**AP1AR**	0.21	4.78E-01	1.00E+00	-0.87	7.24E-02	6.07E-01
**AC010343.3**	-4.04	1.74E-01	1.00E+00	-1.77	7.25E-02	6.07E-01
**LDHAP7**	-0.99	7.23E-01	1.00E+00	-1.42	7.25E-02	6.07E-01
**UBA5**	0.09	6.13E-01	1.00E+00	-0.69	7.25E-02	6.07E-01
**MVD**	-0.18	7.38E-01	1.00E+00	-0.56	7.25E-02	6.07E-01
**TRAV1-2**	-0.83	8.06E-01	1.00E+00	2.56	7.26E-02	6.08E-01
**KDELC1**	0.03	9.35E-01	1.00E+00	0.89	7.26E-02	6.08E-01
**APOM**	-0.41	4.44E-01	1.00E+00	-0.78	7.27E-02	6.08E-01
**ARMC8**	0.24	2.38E-01	1.00E+00	-0.41	7.27E-02	6.08E-01
**ATP5F1C**	-0.06	6.89E-01	1.00E+00	-0.57	7.27E-02	6.08E-01
**PDXDC2P-NPIPB14P**	-0.52	7.67E-01	1.00E+00	1.57	7.27E-02	6.08E-01
**XLOC_005448**	-1.14	6.30E-01	1.00E+00	-2.07	7.27E-02	6.08E-01
**SH3BP5**	0.35	4.83E-01	1.00E+00	1.13	7.27E-02	6.08E-01
**ECHDC1**	0.28	3.98E-01	1.00E+00	-0.70	7.28E-02	6.08E-01
**ZNF25**	0.30	3.19E-01	1.00E+00	-0.53	7.29E-02	6.08E-01
**AC011465.1**	-0.58	8.67E-01	1.00E+00	-2.81	7.29E-02	6.08E-01
**ZNF512**	0.08	8.42E-01	1.00E+00	-0.57	7.30E-02	6.08E-01
**RHOG**	-0.10	6.21E-01	1.00E+00	0.60	7.30E-02	6.08E-01
**MACROD1**	0.21	3.63E-01	1.00E+00	-0.92	7.30E-02	6.08E-01
**UBR3**	0.08	7.25E-01	1.00E+00	-0.50	7.31E-02	6.09E-01
**ENAH**	-0.40	8.18E-02	1.00E+00	0.48	7.31E-02	6.09E-01
**CACYBP**	0.08	6.73E-01	1.00E+00	-0.58	7.31E-02	6.09E-01

**DIRC2**	0.01	9.85E-01	1.00E+00	-0.83	7.32E-02	6.09E-01
**AC106795.2**	0.08	9.44E-01	1.00E+00	-1.20	7.32E-02	6.09E-01
**AJAP1**	-0.58	2.95E-01	1.00E+00	1.56	7.32E-02	6.09E-01
**SCN9A**	0.14	7.73E-01	1.00E+00	-0.79	7.32E-02	6.09E-01
**AC026362.2**	-1.45	3.38E-01	1.00E+00	-1.83	7.32E-02	6.09E-01
**CDC37L1-DT**	0.19	8.56E-01	1.00E+00	-1.17	7.32E-02	6.09E-01
**PSAP**	0.27	2.91E-01	1.00E+00	0.53	7.33E-02	6.09E-01
**XLOC_004229**	-0.28	6.80E-01	1.00E+00	-0.93	7.34E-02	6.09E-01
**NABP2**	-0.35	3.72E-02	1.00E+00	0.58	7.34E-02	6.09E-01
**TATDN3**	0.11	6.61E-01	1.00E+00	-0.50	7.35E-02	6.09E-01
**AC015819.2**	-0.30	9.19E-01	1.00E+00	2.03	7.35E-02	6.09E-01
**BYSL**	-0.34	1.08E-01	1.00E+00	0.53	7.35E-02	6.10E-01
**IL36G**	-0.12	8.62E-01	1.00E+00	1.43	7.36E-02	6.10E-01
**CALM2**	0.23	2.45E-01	1.00E+00	-0.76	7.36E-02	6.10E-01
**STK33**	-2.52	7.61E-02	1.00E+00	-2.20	7.36E-02	6.10E-01
**ERCC8**	0.19	5.00E-01	1.00E+00	-0.57	7.37E-02	6.10E-01
**CNDP1**	-2.05	5.35E-01	1.00E+00	-2.18	7.37E-02	6.10E-01
**AL451085.2**	-2.29	2.70E-01	1.00E+00	-1.93	7.37E-02	6.10E-01
**RIMS3**	0.09	8.87E-01	1.00E+00	0.87	7.38E-02	6.10E-01
**RNF25**	-0.05	8.40E-01	1.00E+00	0.42	7.38E-02	6.10E-01
**RTRAF**	-0.01	9.73E-01	1.00E+00	-0.36	7.38E-02	6.10E-01
**SRD5A2**	2.43	8.72E-02	1.00E+00	2.95	7.39E-02	6.10E-01
**AP003352.1**	2.02	2.15E-02	8.94E-01	-1.05	7.39E-02	6.10E-01
**CCL20**	-3.31	2.39E-02	9.18E-01	1.87	7.39E-02	6.10E-01
**DHRS9**	-0.26	6.41E-01	1.00E+00	0.83	7.39E-02	6.10E-01
**AP005482.1**	-0.39	8.65E-01	1.00E+00	-2.19	7.40E-02	6.10E-01
**AL512791.1**	1.58	3.99E-01	1.00E+00	1.83	7.40E-02	6.10E-01
**AC127024.4**	0.37	8.71E-01	1.00E+00	-1.99	7.41E-02	6.10E-01
**SCML4**	1.02	4.20E-01	1.00E+00	1.52	7.41E-02	6.10E-01
**ISL1**	-0.25	7.27E-01	1.00E+00	-1.34	7.41E-02	6.10E-01
**MUL1**	0.04	8.09E-01	1.00E+00	0.18	7.41E-02	6.10E-01
**AC100810.3**	-1.13	6.68E-01	1.00E+00	-2.07	7.41E-02	6.10E-01

**9-Mar**	-0.25	2.81E-01	1.00E+00	1.13	7.41E-02	6.10E-01
**ADGRA2**	0.50	2.13E-01	1.00E+00	1.52	7.42E-02	6.10E-01
**VOPP1**	0.20	5.13E-01	1.00E+00	1.09	7.42E-02	6.10E-01
**FBRS**	-0.42	2.19E-01	1.00E+00	1.20	7.42E-02	6.10E-01
**SLC20A1**	-0.35	3.04E-01	1.00E+00	0.75	7.43E-02	6.10E-01
**ALDH6A1**	0.23	4.31E-01	1.00E+00	-0.69	7.43E-02	6.10E-01
**LINC00909**	0.01	9.46E-01	1.00E+00	-0.49	7.43E-02	6.10E-01
**GTF2E2**	0.45	2.77E-02	9.71E-01	-0.99	7.44E-02	6.10E-01
**RPL29P19**	-0.19	8.42E-01	1.00E+00	2.08	7.44E-02	6.10E-01
**MTFR1L**	-0.03	9.12E-01	1.00E+00	-0.58	7.44E-02	6.10E-01
**GDF15**	-0.80	1.25E-01	1.00E+00	1.44	7.45E-02	6.10E-01
**KCNK3**	-0.58	4.00E-01	1.00E+00	1.44	7.46E-02	6.10E-01
**AC022395.1**	1.19	7.32E-01	1.00E+00	-3.64	7.46E-02	6.10E-01
**OR7E14P**	0.57	6.82E-01	1.00E+00	-1.10	7.46E-02	6.10E-01
**VMA21**	0.27	2.98E-01	1.00E+00	-0.52	7.46E-02	6.10E-01
**DOT1L**	-0.29	4.22E-01	1.00E+00	1.19	7.46E-02	6.10E-01
**ZC3H6**	0.06	7.73E-01	1.00E+00	-0.56	7.46E-02	6.10E-01
**TNFRSF1A**	-0.17	4.71E-01	1.00E+00	0.55	7.46E-02	6.10E-01
**VPS9D1**	-0.32	2.20E-01	1.00E+00	0.97	7.47E-02	6.10E-01
**DOC2GP**	-2.86	5.06E-02	1.00E+00	1.90	7.47E-02	6.10E-01
**AL121658.1**	0.45	6.61E-01	1.00E+00	-1.53	7.47E-02	6.10E-01
**XLOC_010579**	-2.94	2.11E-02	8.94E-01	1.29	7.47E-02	6.10E-01
**CD244**	-0.96	2.76E-01	1.00E+00	1.75	7.47E-02	6.10E-01
**ATP5F1B**	-0.08	6.32E-01	1.00E+00	-0.27	7.48E-02	6.10E-01
**HELT**	-1.18	3.78E-01	1.00E+00	-1.96	7.48E-02	6.10E-01
**ZNF32-AS2**	-0.56	7.99E-01	1.00E+00	-1.82	7.49E-02	6.10E-01
**NPEPL1**	0.16	6.26E-01	1.00E+00	-0.38	7.49E-02	6.10E-01
**G13446**	-2.37	1.57E-01	1.00E+00	-3.10	7.49E-02	6.10E-01
**GOPC**	0.02	9.30E-01	1.00E+00	-0.42	7.49E-02	6.10E-01
**AC012181.2**	-1.65	3.79E-01	1.00E+00	1.53	7.49E-02	6.10E-01
**HIST1H2BG**	-1.41	5.02E-02	1.00E+00	-1.05	7.49E-02	6.10E-01
**WASH3P**	-0.11	6.83E-01	1.00E+00	0.71	7.50E-02	6.10E-01

**FUT8-AS1**	0.92	4.43E-01	1.00E+00	1.34	7.50E-02	6.10E-01
**RGPD3**	-0.41	6.07E-01	1.00E+00	1.04	7.50E-02	6.10E-01
**ULK4P2**	0.64	7.22E-01	1.00E+00	-1.61	7.51E-02	6.11E-01
**VSTM1**	NA	NA	NA	3.40	7.51E-02	6.11E-01
**CTTN**	-0.15	5.11E-01	1.00E+00	0.53	7.52E-02	6.11E-01
**ADGRL4**	0.28	6.25E-01	1.00E+00	-0.92	7.52E-02	6.11E-01
**AL135905.2**	-0.15	8.14E-01	1.00E+00	1.10	7.52E-02	6.11E-01
**CUL4B**	0.01	9.36E-01	1.00E+00	0.28	7.53E-02	6.11E-01
**SBNO2**	-0.51	4.26E-02	1.00E+00	1.76	7.53E-02	6.11E-01
**AC147651.3**	-1.74	8.88E-02	1.00E+00	1.15	7.53E-02	6.11E-01
**SCG5**	-1.00	2.80E-01	1.00E+00	0.85	7.53E-02	6.11E-01
**GZMM**	-0.60	3.35E-01	1.00E+00	1.60	7.53E-02	6.11E-01
**TMEM176A**	0.18	5.74E-01	1.00E+00	1.11	7.53E-02	6.11E-01
**EGLN2**	1.81	4.92E-02	1.00E+00	1.05	7.54E-02	6.11E-01
**TMEM87B**	0.03	8.90E-01	1.00E+00	0.38	7.54E-02	6.11E-01
**RSU1**	-0.13	4.56E-01	1.00E+00	-0.42	7.54E-02	6.11E-01
**TBX6**	0.49	2.13E-01	1.00E+00	1.11	7.54E-02	6.11E-01
**CRIP2**	-0.18	5.74E-01	1.00E+00	-0.40	7.55E-02	6.11E-01
**HSPE1P2**	NA	NA	NA	-1.47	7.55E-02	6.11E-01
**AC026355.2**	-0.38	7.93E-01	1.00E+00	2.10	7.55E-02	6.11E-01
**ERVK9-11**	-0.16	8.52E-01	1.00E+00	-1.41	7.55E-02	6.11E-01
**AC111170.3**	1.58	2.15E-01	1.00E+00	2.48	7.56E-02	6.11E-01
**ESM1**	-0.19	8.38E-01	1.00E+00	1.71	7.56E-02	6.11E-01
**RARRES1**	0.38	6.65E-01	1.00E+00	-1.19	7.56E-02	6.11E-01
**CAPN11**	0.45	2.73E-01	1.00E+00	-0.88	7.57E-02	6.12E-01
**LINC02099**	NA	NA	NA	3.34	7.58E-02	6.12E-01
**CTDSPL**	0.26	1.92E-01	1.00E+00	-0.52	7.58E-02	6.12E-01
**FRMD4B**	-0.01	9.65E-01	1.00E+00	0.39	7.58E-02	6.12E-01
**PCDHB16**	0.23	5.63E-01	1.00E+00	-0.84	7.59E-02	6.12E-01
**AC004076.2**	0.63	6.39E-01	1.00E+00	-1.57	7.59E-02	6.12E-01
**AL356750.1**	1.66	6.25E-01	1.00E+00	-2.49	7.59E-02	6.12E-01
**LINC01819**	-0.72	1.50E-01	1.00E+00	-1.22	7.59E-02	6.12E-01

**PNPT1**	0.10	8.03E-01	1.00E+00	0.51	7.59E-02	6.12E-01
**TMEM232**	0.19	7.60E-01	1.00E+00	-1.13	7.59E-02	6.12E-01
**TLR1**	0.25	6.52E-01	1.00E+00	0.86	7.60E-02	6.12E-01
**ZNF573**	0.17	6.37E-01	1.00E+00	-0.92	7.60E-02	6.12E-01
**RPA1**	-0.02	8.98E-01	1.00E+00	-0.18	7.61E-02	6.12E-01
**NID2**	-0.56	2.26E-01	1.00E+00	1.55	7.61E-02	6.12E-01
**ASB8**	0.02	9.24E-01	1.00E+00	-0.20	7.61E-02	6.12E-01
**AC127070.1**	1.23	3.34E-01	1.00E+00	-1.85	7.61E-02	6.12E-01
**CAPN13**	0.77	4.94E-01	1.00E+00	-1.92	7.61E-02	6.12E-01
**ZFYVE26**	0.04	8.48E-01	1.00E+00	0.98	7.62E-02	6.12E-01
**ABR**	0.01	9.59E-01	1.00E+00	0.89	7.62E-02	6.12E-01
**WEE1**	-0.09	8.63E-01	1.00E+00	-0.72	7.62E-02	6.12E-01
**AL158071.4**	1.18	2.45E-01	1.00E+00	2.04	7.62E-02	6.12E-01
**MRGPRX2**	1.59	3.17E-02	1.00E+00	1.62	7.62E-02	6.12E-01
**TOR4A**	-0.27	5.38E-01	1.00E+00	1.56	7.63E-02	6.12E-01
**SLFN5**	-0.21	6.83E-01	1.00E+00	0.89	7.63E-02	6.12E-01
**DLD**	0.02	9.41E-01	1.00E+00	-0.50	7.65E-02	6.13E-01
**PEX14**	-0.22	4.59E-01	1.00E+00	0.52	7.66E-02	6.13E-01
**CHST2**	-0.49	4.59E-01	1.00E+00	1.39	7.66E-02	6.13E-01
**SLC9A3**	-0.94	5.39E-02	1.00E+00	0.78	7.66E-02	6.13E-01
**XCL2**	-1.51	2.49E-01	1.00E+00	1.77	7.67E-02	6.13E-01
**MAP2K7**	-0.23	2.47E-01	1.00E+00	0.78	7.67E-02	6.13E-01
**RET**	0.51	4.86E-01	1.00E+00	1.30	7.68E-02	6.13E-01
**ACTG1P1**	0.00	9.99E-01	1.00E+00	-1.40	7.68E-02	6.13E-01
**FERMT3**	-0.14	6.81E-01	1.00E+00	1.29	7.68E-02	6.13E-01
**SMIM7**	0.42	9.16E-02	1.00E+00	-0.59	7.68E-02	6.13E-01
**OR2S1P**	-0.10	9.75E-01	1.00E+00	-3.83	7.68E-02	6.13E-01
**AC087343.1**	-0.11	9.44E-01	1.00E+00	-1.31	7.68E-02	6.13E-01
**TMEM175**	-0.04	8.78E-01	1.00E+00	0.34	7.68E-02	6.13E-01
**CKMT2**	0.43	5.48E-01	1.00E+00	-0.95	7.68E-02	6.13E-01
**SENP2**	0.19	3.64E-01	1.00E+00	0.31	7.68E-02	6.13E-01
**SMIM24**	-0.84	3.69E-01	1.00E+00	-1.41	7.69E-02	6.13E-01

**PFKFB4**	-0.34	2.38E-01	1.00E+00	0.98	7.69E-02	6.13E-01
**AC008892.1**	-0.86	8.05E-01	1.00E+00	-2.29	7.69E-02	6.13E-01
**PHLDA2**	-0.88	1.61E-01	1.00E+00	0.68	7.70E-02	6.13E-01
**GZMA**	-0.53	5.26E-01	1.00E+00	1.27	7.70E-02	6.13E-01
**AL360270.3**	-3.41	2.40E-01	1.00E+00	1.99	7.70E-02	6.13E-01
**IER3IP1**	0.18	5.09E-01	1.00E+00	-0.78	7.70E-02	6.13E-01
**RAC2**	-0.06	8.95E-01	1.00E+00	1.21	7.71E-02	6.13E-01
**XBP1**	-0.11	7.53E-01	1.00E+00	-0.82	7.71E-02	6.13E-01
**CD163L1**	0.72	1.42E-01	1.00E+00	0.65	7.71E-02	6.13E-01
**XLOC_009723**	0.16	9.21E-01	1.00E+00	-1.76	7.71E-02	6.13E-01
**ADAMTS9-AS1**	0.67	6.20E-01	1.00E+00	-1.40	7.72E-02	6.13E-01
**NRIR**	3.10	3.60E-01	1.00E+00	2.58	7.72E-02	6.13E-01
**PDLIM7**	-0.51	2.57E-01	1.00E+00	0.63	7.72E-02	6.13E-01
**AC087633.2**	-1.26	3.26E-01	1.00E+00	-1.05	7.73E-02	6.13E-01
**XLOC_002344**	-0.18	9.07E-01	1.00E+00	-1.57	7.73E-02	6.13E-01
**ADAM10**	0.21	4.53E-01	1.00E+00	0.30	7.73E-02	6.13E-01
**AC098484.4**	0.64	5.02E-01	1.00E+00	-1.15	7.73E-02	6.13E-01
**AC005829.2**	-1.16	6.40E-01	1.00E+00	-3.71	7.74E-02	6.14E-01
**DOCK9-DT**	-0.10	8.00E-01	1.00E+00	-0.90	7.74E-02	6.14E-01
**CUL2**	0.11	4.87E-01	1.00E+00	-0.30	7.74E-02	6.14E-01
**MIA**	NA	NA	NA	-1.39	7.74E-02	6.14E-01
**PSD**	-1.68	2.88E-03	2.79E-01	0.72	7.75E-02	6.14E-01
**FAM30A**	0.87	7.56E-01	1.00E+00	2.63	7.75E-02	6.14E-01
**C1orf74**	-0.25	6.04E-01	1.00E+00	0.60	7.75E-02	6.14E-01
**FP325330.3**	0.10	9.36E-01	1.00E+00	-3.08	7.76E-02	6.14E-01
**DCK**	0.32	3.22E-01	1.00E+00	-0.51	7.76E-02	6.14E-01
**PCDHA11**	-2.34	8.67E-02	1.00E+00	-2.70	7.76E-02	6.14E-01
**XLOC_010989**	-2.39	4.35E-01	1.00E+00	-3.27	7.76E-02	6.14E-01
**NDRG2**	-0.03	9.11E-01	1.00E+00	-0.43	7.76E-02	6.14E-01
**CARHSP1**	0.04	9.12E-01	1.00E+00	0.47	7.77E-02	6.14E-01
**EEF2KMT**	-0.31	2.16E-01	1.00E+00	0.40	7.78E-02	6.14E-01
**TM6SF2**	0.01	9.98E-01	1.00E+00	1.85	7.78E-02	6.14E-01

**G14339**	0.86	5.90E-01	1.00E+00	1.24	7.78E-02	6.14E-01
**LDHAP3**	1.02	7.67E-01	1.00E+00	-1.35	7.78E-02	6.14E-01
**CCL13**	0.17	7.91E-01	1.00E+00	0.97	7.78E-02	6.14E-01
**F8A3**	-0.04	9.59E-01	1.00E+00	-1.37	7.79E-02	6.14E-01
**RPL12P47**	NA	NA	NA	-1.16	7.79E-02	6.14E-01
**PGF**	-0.76	7.40E-03	5.23E-01	0.88	7.80E-02	6.14E-01
**IFI44L**	1.03	3.28E-01	1.00E+00	1.21	7.80E-02	6.14E-01
**TM9SF3**	0.14	4.45E-01	1.00E+00	-0.55	7.80E-02	6.14E-01
**AP001033.1**	-2.67	3.64E-01	1.00E+00	-2.23	7.81E-02	6.14E-01
**CHD2**	-0.09	7.19E-01	1.00E+00	0.55	7.81E-02	6.14E-01
**FOXA1**	0.07	9.55E-01	1.00E+00	-1.78	7.81E-02	6.14E-01
**ADH6**	-0.36	6.55E-01	1.00E+00	-1.53	7.81E-02	6.14E-01
**AC026271.1**	0.33	2.46E-01	1.00E+00	-0.61	7.81E-02	6.14E-01
**IQCA1**	-0.58	4.38E-01	1.00E+00	1.13	7.81E-02	6.14E-01
**THY1**	-0.48	4.74E-01	1.00E+00	1.10	7.81E-02	6.14E-01
**LSM12**	-0.26	1.70E-01	1.00E+00	-0.32	7.81E-02	6.14E-01
**NUDCD2**	0.02	9.19E-01	1.00E+00	-0.62	7.81E-02	6.14E-01
**XLOC_012894**	-0.33	4.34E-01	1.00E+00	0.56	7.82E-02	6.14E-01
**COMMD4**	-0.21	3.03E-01	1.00E+00	0.31	7.82E-02	6.14E-01
**MED13**	-0.05	8.29E-01	1.00E+00	0.58	7.82E-02	6.14E-01
**POM121B**	0.26	7.27E-01	1.00E+00	1.48	7.82E-02	6.14E-01
**SRPK2**	-0.03	8.62E-01	1.00E+00	0.32	7.83E-02	6.14E-01
**AC007496.1**	0.01	9.98E-01	1.00E+00	-2.08	7.83E-02	6.14E-01
**POLI**	0.22	5.07E-01	1.00E+00	-0.67	7.83E-02	6.14E-01
**WFDC3**	0.01	9.93E-01	1.00E+00	-1.34	7.84E-02	6.14E-01
**PIWIL1**	-2.88	1.60E-01	1.00E+00	3.29	7.84E-02	6.14E-01
**PCYT2**	-0.27	3.18E-01	1.00E+00	-0.94	7.85E-02	6.14E-01
**AL158211.5**	-0.09	9.53E-01	1.00E+00	-2.24	7.85E-02	6.14E-01
**ZNF652**	0.25	2.12E-01	1.00E+00	-0.53	7.85E-02	6.14E-01
**AC108097.1**	-2.85	1.12E-01	1.00E+00	-1.74	7.85E-02	6.14E-01
**AP003392.6**	-0.14	9.61E-01	1.00E+00	1.58	7.86E-02	6.14E-01
**ZNF563**	-0.13	6.37E-01	1.00E+00	-0.71	7.86E-02	6.14E-01

**SSU72**	-0.08	6.30E-01	1.00E+00	-0.44	7.86E-02	6.14E-01
**XLOC_009944**	1.46	4.52E-01	1.00E+00	1.60	7.87E-02	6.14E-01
**AC015914.1**	-0.22	9.21E-01	1.00E+00	-2.02	7.87E-02	6.14E-01
**LINC00629**	-1.78	6.06E-01	1.00E+00	-2.74	7.87E-02	6.14E-01
**AL136116.3**	0.35	5.47E-01	1.00E+00	-0.77	7.87E-02	6.14E-01
**AL139289.1**	-1.54	4.16E-01	1.00E+00	2.62	7.88E-02	6.15E-01
**ZBTB17**	-0.07	7.05E-01	1.00E+00	0.62	7.89E-02	6.15E-01
**OACYLP**	-0.93	5.85E-01	1.00E+00	2.80	7.89E-02	6.15E-01
**ZNF675**	-0.16	6.88E-01	1.00E+00	-0.69	7.89E-02	6.15E-01
**TGIF1**	-0.18	5.30E-01	1.00E+00	0.33	7.89E-02	6.15E-01
**ZFHX3**	-0.06	8.41E-01	1.00E+00	0.61	7.90E-02	6.15E-01
**ARL6IP5**	0.36	2.70E-01	1.00E+00	-0.63	7.90E-02	6.15E-01
**DZANK1**	0.01	9.85E-01	1.00E+00	-0.64	7.90E-02	6.15E-01
**WFDC21P**	0.92	1.21E-01	1.00E+00	-1.37	7.91E-02	6.15E-01
**LAT2**	-0.08	8.47E-01	1.00E+00	0.95	7.91E-02	6.15E-01
**DISC1**	0.49	2.54E-01	1.00E+00	1.14	7.92E-02	6.15E-01
**FUT3**	-1.21	8.94E-03	5.88E-01	1.40	7.92E-02	6.15E-01
**COLGALT2**	0.09	8.93E-01	1.00E+00	1.43	7.92E-02	6.15E-01
**KLRD1**	0.02	9.85E-01	1.00E+00	1.43	7.92E-02	6.16E-01
**HNRNPA1P54**	-0.51	8.83E-01	1.00E+00	-1.59	7.93E-02	6.16E-01
**FAM206A**	0.26	2.01E-01	1.00E+00	-0.61	7.94E-02	6.16E-01
**MTHFD2**	0.03	9.15E-01	1.00E+00	0.45	7.94E-02	6.16E-01
**LRTOMT**	0.52	2.36E-01	1.00E+00	-0.81	7.95E-02	6.16E-01
**USP44**	0.99	1.66E-01	1.00E+00	-1.22	7.95E-02	6.16E-01
**PTPN12**	0.06	7.85E-01	1.00E+00	0.57	7.96E-02	6.16E-01
**AC124319.2**	0.63	3.38E-01	1.00E+00	1.55	7.96E-02	6.16E-01
**RP11-277L2.3**	-2.02	3.96E-01	1.00E+00	1.22	7.96E-02	6.16E-01
**SLC28A1**	1.34	6.98E-01	1.00E+00	2.64	7.97E-02	6.16E-01
**GNAS-AS1**	-1.39	3.28E-01	1.00E+00	1.98	7.98E-02	6.16E-01
**SUGCT**	0.49	4.37E-01	1.00E+00	0.75	7.98E-02	6.16E-01
**TRAV8-6**	1.73	4.12E-01	1.00E+00	2.06	7.98E-02	6.16E-01
**FCGR2B**	0.09	8.50E-01	1.00E+00	0.66	7.98E-02	6.16E-01

**CCDC13-AS1**	0.79	6.87E-01	1.00E+00	-2.04	7.98E-02	6.16E-01
**EEF1A1P11**	-0.30	5.85E-01	1.00E+00	-0.68	7.98E-02	6.16E-01
**LCK**	-0.98	1.68E-01	1.00E+00	1.24	7.99E-02	6.16E-01
**APP**	0.03	9.14E-01	1.00E+00	0.87	7.99E-02	6.16E-01
**MRPS6**	0.07	7.63E-01	1.00E+00	-0.45	7.99E-02	6.16E-01
**G36288**	0.10	8.94E-01	1.00E+00	-1.10	7.99E-02	6.16E-01
**AC025580.1**	-2.29	2.33E-01	1.00E+00	1.81	7.99E-02	6.16E-01
**AC093724.1**	0.82	7.97E-02	1.00E+00	1.56	7.99E-02	6.16E-01
**MPP1**	-0.11	7.27E-01	1.00E+00	0.53	7.99E-02	6.16E-01
**VGLL4**	-0.02	8.93E-01	1.00E+00	0.39	8.00E-02	6.16E-01
**DPY19L2P2**	-0.43	4.41E-01	1.00E+00	1.19	8.00E-02	6.16E-01
**CACYBPP2**	-0.04	9.72E-01	1.00E+00	-0.97	8.00E-02	6.16E-01
**SUZ12**	0.08	7.73E-01	1.00E+00	-0.50	8.00E-02	6.16E-01
**SRP9P1**	0.50	2.85E-01	1.00E+00	-0.60	8.01E-02	6.16E-01
**LRRC8B**	-0.38	2.83E-01	1.00E+00	-0.54	8.01E-02	6.16E-01
**TRIM23**	0.14	7.05E-01	1.00E+00	-0.59	8.01E-02	6.16E-01
**IGLVI-70**	0.00	1.00E+00	NA	5.38	8.01E-02	6.16E-01
**CCDC65**	0.02	9.78E-01	1.00E+00	1.21	8.01E-02	6.16E-01
**AL035413.2**	-0.50	3.62E-01	1.00E+00	-0.95	8.02E-02	6.16E-01
**SLC25A20**	0.76	4.76E-02	1.00E+00	-0.62	8.02E-02	6.16E-01
**COL24A1**	-0.75	3.82E-01	1.00E+00	1.50	8.02E-02	6.16E-01
**C12orf77**	-1.40	3.93E-01	1.00E+00	-2.71	8.02E-02	6.16E-01
**NDEL1**	0.03	9.44E-01	1.00E+00	0.43	8.02E-02	6.16E-01
**XLOC_009217**	-0.24	7.15E-01	1.00E+00	-1.64	8.03E-02	6.16E-01
**BAALC-AS1**	-0.36	6.31E-01	1.00E+00	-0.60	8.03E-02	6.16E-01
**PCED1B-AS1**	-0.51	5.30E-01	1.00E+00	1.15	8.03E-02	6.16E-01
**LACC1**	-0.07	8.06E-01	1.00E+00	0.32	8.03E-02	6.16E-01
**TRIM14**	0.18	7.01E-01	1.00E+00	0.84	8.04E-02	6.16E-01
**AC007485.1**	-1.90	5.74E-01	1.00E+00	2.10	8.04E-02	6.16E-01
**SETD1A**	-0.61	2.54E-02	9.29E-01	1.30	8.06E-02	6.17E-01
**CDK12**	-0.28	2.18E-01	1.00E+00	0.68	8.06E-02	6.18E-01
**PSMB8**	-0.16	6.13E-01	1.00E+00	0.54	8.07E-02	6.18E-01

**MT-TD**	NA	NA	NA	-2.51	8.07E-02	6.18E-01
**LINC02547**	-0.21	8.75E-01	1.00E+00	-3.31	8.07E-02	6.18E-01
**AC019080.5**	2.52	4.04E-01	1.00E+00	2.58	8.08E-02	6.18E-01
**TPPP**	-0.25	5.57E-01	1.00E+00	-0.98	8.08E-02	6.18E-01
**FABP5**	0.11	7.52E-01	1.00E+00	0.65	8.08E-02	6.18E-01
**C20orf194**	0.68	1.25E-01	1.00E+00	0.79	8.08E-02	6.18E-01
**XLOC_006169**	-0.47	5.94E-01	1.00E+00	-1.34	8.09E-02	6.18E-01
**NGF**	-0.44	4.24E-01	1.00E+00	1.42	8.09E-02	6.18E-01
**JSRP1**	2.01	2.09E-01	1.00E+00	2.57	8.09E-02	6.18E-01
**RPL7P21**	-0.49	7.65E-01	1.00E+00	-1.09	8.10E-02	6.18E-01
**ZNF569**	0.05	8.72E-01	1.00E+00	-0.64	8.10E-02	6.18E-01
**FAM13A-AS1**	1.05	3.51E-01	1.00E+00	1.30	8.10E-02	6.18E-01
**HEATR4**	0.00	9.98E-01	1.00E+00	1.26	8.10E-02	6.18E-01
**AL627402.1**	-1.15	4.77E-01	1.00E+00	-0.85	8.11E-02	6.18E-01
**MROH2A**	-0.83	2.12E-01	1.00E+00	-1.56	8.11E-02	6.18E-01
**EVC**	-0.07	8.23E-01	1.00E+00	1.26	8.12E-02	6.18E-01
**IRF2**	0.00	9.98E-01	1.00E+00	0.45	8.12E-02	6.18E-01
**G19573**	-2.10	6.81E-02	1.00E+00	-1.67	8.12E-02	6.18E-01
**GGA3**	-0.17	4.81E-01	1.00E+00	0.62	8.12E-02	6.18E-01
**AC002306.1**	-1.71	6.18E-01	1.00E+00	-2.47	8.12E-02	6.18E-01
**AL390728.6**	0.62	3.12E-01	1.00E+00	-0.92	8.12E-02	6.18E-01
**TRAV12-3**	-0.30	9.01E-01	1.00E+00	1.85	8.13E-02	6.18E-01
**AC026254.2**	-1.86	2.01E-01	1.00E+00	2.05	8.13E-02	6.18E-01
**AC080188.2**	-3.16	6.62E-03	4.93E-01	1.29	8.14E-02	6.18E-01
**AL513318.2**	1.54	6.83E-02	1.00E+00	1.89	8.14E-02	6.18E-01
**RAPGEF1**	0.03	9.01E-01	1.00E+00	1.10	8.14E-02	6.18E-01
**TTC32**	-0.25	4.64E-01	1.00E+00	-0.76	8.14E-02	6.18E-01
**PDCD1LG2**	0.47	2.59E-01	1.00E+00	1.12	8.14E-02	6.18E-01
**RYK**	0.40	5.33E-02	1.00E+00	-0.63	8.15E-02	6.18E-01
**POR**	-0.06	7.90E-01	1.00E+00	0.55	8.15E-02	6.18E-01
**CDK18**	-0.62	1.17E-01	1.00E+00	1.26	8.15E-02	6.18E-01
**NFYC**	-0.06	7.21E-01	1.00E+00	-0.20	8.15E-02	6.18E-01

**VWF**	0.39	5.46E-01	1.00E+00	1.12	8.16E-02	6.19E-01
**G28914**	-0.49	6.25E-01	1.00E+00	-1.86	8.16E-02	6.19E-01
**AC010761.1**	0.14	8.55E-01	1.00E+00	1.36	8.17E-02	6.19E-01
**IGHV3-9**	7.72	5.32E-08	2.27E-05	2.23	8.17E-02	6.19E-01
**KDSR**	0.19	3.56E-01	1.00E+00	-0.42	8.18E-02	6.20E-01
**UBR4**	0.22	4.04E-01	1.00E+00	0.55	8.20E-02	6.20E-01
**ZNF689**	-0.07	7.75E-01	1.00E+00	0.54	8.20E-02	6.20E-01
**FAM129B**	0.00	9.86E-01	1.00E+00	1.29	8.20E-02	6.20E-01
**5-Sep**	-0.79	4.76E-01	1.00E+00	0.83	8.20E-02	6.20E-01
**KBTBD4**	-0.30	4.09E-01	1.00E+00	-0.35	8.20E-02	6.20E-01
**SH3PXD2A**	-0.21	4.62E-01	1.00E+00	0.87	8.20E-02	6.20E-01
**PPIAP41**	-1.20	5.22E-01	1.00E+00	-2.30	8.20E-02	6.20E-01
**FKBPL**	0.12	6.90E-01	1.00E+00	0.58	8.20E-02	6.20E-01
**VENTX**	0.46	4.54E-01	1.00E+00	2.01	8.21E-02	6.20E-01
**THAP12P7**	0.88	4.52E-01	1.00E+00	-1.51	8.21E-02	6.20E-01
**SMURF2P1-LRRC37BP1**	-0.56	8.08E-01	1.00E+00	1.15	8.21E-02	6.20E-01
**AP000695.1**	0.67	5.40E-01	1.00E+00	1.93	8.22E-02	6.20E-01
**OTUD7A**	-0.47	1.37E-01	1.00E+00	-0.79	8.23E-02	6.20E-01
**SPAG1**	0.11	8.52E-01	1.00E+00	-0.68	8.23E-02	6.20E-01
**TBCCD1**	-0.05	8.68E-01	1.00E+00	-0.50	8.23E-02	6.20E-01
**AC104596.1**	-0.20	7.71E-01	1.00E+00	1.39	8.23E-02	6.20E-01
**LGALS3**	-0.03	9.00E-01	1.00E+00	-0.45	8.23E-02	6.20E-01
**AC073264.3**	2.07	7.21E-02	1.00E+00	1.30	8.24E-02	6.20E-01
**ITGA5**	0.10	8.10E-01	1.00E+00	1.02	8.24E-02	6.20E-01
**SCGB2A2**	0.33	8.07E-01	1.00E+00	-1.62	8.25E-02	6.20E-01
**CNTN6**	1.53	6.44E-01	1.00E+00	2.45	8.25E-02	6.20E-01
**KRT8P3**	-0.27	8.41E-01	1.00E+00	-1.45	8.25E-02	6.20E-01
**AP000786.1**	0.98	6.87E-01	1.00E+00	-2.61	8.26E-02	6.20E-01
**XLOC_010145**	-0.08	9.50E-01	1.00E+00	1.49	8.26E-02	6.20E-01
**IGLV7-46**	-0.11	9.67E-01	1.00E+00	2.56	8.26E-02	6.20E-01
**AC099494.3**	1.27	7.13E-01	1.00E+00	-3.02	8.27E-02	6.20E-01
**SCDP1**	1.96	5.66E-01	1.00E+00	-2.32	8.27E-02	6.20E-01

**AC008895.1**	-0.25	9.28E-01	1.00E+00	1.56	8.27E-02	6.20E-01
**VPS53**	-0.11	6.80E-01	1.00E+00	0.49	8.27E-02	6.20E-01
**AC098613.1**	-1.40	5.76E-01	1.00E+00	1.52	8.27E-02	6.20E-01
**GTF2IRD2P1**	0.76	4.75E-01	1.00E+00	-1.40	8.27E-02	6.20E-01
**ERO1A**	-0.05	8.35E-01	1.00E+00	0.32	8.27E-02	6.20E-01
**DLK1**	-0.38	6.95E-01	1.00E+00	-2.12	8.28E-02	6.20E-01
**FTH1P5**	0.10	9.61E-01	1.00E+00	-1.21	8.28E-02	6.20E-01
**RAB34**	-0.14	6.10E-01	1.00E+00	0.33	8.28E-02	6.20E-01
**TRAV8-2**	1.59	4.53E-01	1.00E+00	1.61	8.29E-02	6.21E-01
**LINC02018**	-1.31	2.00E-01	1.00E+00	-0.99	8.29E-02	6.21E-01
**ADCY7**	0.10	7.25E-01	1.00E+00	1.20	8.29E-02	6.21E-01
**FBXO39**	-1.49	1.18E-01	1.00E+00	1.73	8.30E-02	6.21E-01
**FAS**	-0.34	3.82E-01	1.00E+00	0.47	8.30E-02	6.21E-01
**AC007342.9**	-1.69	2.46E-01	1.00E+00	-2.03	8.30E-02	6.21E-01
**AC131934.1**	1.85	7.17E-02	1.00E+00	1.69	8.30E-02	6.21E-01
**MANEA**	-0.13	7.30E-01	1.00E+00	-0.39	8.31E-02	6.21E-01
**INPPL1**	-0.06	8.29E-01	1.00E+00	1.12	8.32E-02	6.21E-01
**EPHB2**	-0.36	5.50E-01	1.00E+00	1.21	8.32E-02	6.21E-01
**ATXN1**	0.06	8.19E-01	1.00E+00	0.42	8.32E-02	6.21E-01
**XLOC_000190**	0.51	5.79E-01	1.00E+00	-1.35	8.33E-02	6.21E-01
**MIR663AHG**	-0.22	7.13E-01	1.00E+00	-1.66	8.33E-02	6.21E-01
**EDRF1**	0.09	7.93E-01	1.00E+00	-0.43	8.33E-02	6.21E-01
**COQ10A**	-0.41	3.76E-01	1.00E+00	-0.75	8.34E-02	6.21E-01
**USP30**	-0.09	6.41E-01	1.00E+00	-0.37	8.34E-02	6.21E-01
**RNASET2**	-0.28	3.23E-01	1.00E+00	0.74	8.34E-02	6.21E-01
**UBASH3B**	0.28	6.86E-01	1.00E+00	0.79	8.34E-02	6.21E-01
**GSDMD**	-0.43	2.14E-01	1.00E+00	0.87	8.34E-02	6.21E-01
**FGF1**	-0.10	9.03E-01	1.00E+00	-0.90	8.34E-02	6.21E-01
**FBXW11P1**	1.65	3.47E-01	1.00E+00	-2.42	8.35E-02	6.21E-01
**AC092634.4**	0.62	5.59E-01	1.00E+00	-1.36	8.35E-02	6.21E-01
**XLOC_011016**	0.78	2.82E-01	1.00E+00	-1.23	8.35E-02	6.21E-01
**AP001542.3**	0.04	8.65E-01	1.00E+00	-0.94	8.35E-02	6.21E-01

**AC005488.1**	0.69	4.22E-01	1.00E+00	1.24	8.36E-02	6.21E-01
**KLK13**	-0.66	2.20E-01	1.00E+00	1.41	8.37E-02	6.21E-01
**PRR33**	-0.26	7.65E-01	1.00E+00	1.93	8.37E-02	6.21E-01
**SH3BP1**	-0.24	5.97E-01	1.00E+00	0.99	8.37E-02	6.21E-01
**AC016877.3**	0.99	2.32E-01	1.00E+00	-1.83	8.38E-02	6.21E-01
**HMGXB4**	0.12	4.68E-01	1.00E+00	0.34	8.38E-02	6.21E-01
**EPAS1**	0.18	6.28E-01	1.00E+00	0.81	8.39E-02	6.21E-01
**AC007382.1**	0.01	9.71E-01	1.00E+00	0.67	8.39E-02	6.21E-01
**SLC1A6**	-0.71	1.76E-01	1.00E+00	-1.19	8.39E-02	6.21E-01
**INSIG1**	0.27	4.56E-01	1.00E+00	-0.92	8.39E-02	6.21E-01
**AP001623.1**	-0.41	4.41E-01	1.00E+00	1.04	8.39E-02	6.21E-01
**AKT3**	0.44	3.43E-01	1.00E+00	1.09	8.39E-02	6.21E-01
**AC007424.1**	0.60	6.87E-01	1.00E+00	-2.23	8.39E-02	6.21E-01
**BCAT1**	-0.01	9.82E-01	1.00E+00	1.06	8.39E-02	6.21E-01
**NADSYN1**	0.05	8.71E-01	1.00E+00	0.40	8.40E-02	6.22E-01
**PLXNC1**	-0.29	2.70E-01	1.00E+00	0.95	8.41E-02	6.22E-01
**HNRNPA1L2**	0.04	9.23E-01	1.00E+00	0.51	8.41E-02	6.22E-01
**CD24P4**	-0.15	6.36E-01	1.00E+00	1.06	8.41E-02	6.22E-01
**CA6**	-0.55	3.35E-01	1.00E+00	-1.33	8.42E-02	6.22E-01
**MADCAM1**	-0.08	9.36E-01	1.00E+00	1.38	8.43E-02	6.22E-01
**TAB1**	-0.07	6.89E-01	1.00E+00	0.66	8.43E-02	6.22E-01
**LPIN2**	0.44	2.02E-01	1.00E+00	1.00	8.43E-02	6.22E-01
**AC007388.1**	0.23	3.81E-01	1.00E+00	-0.64	8.43E-02	6.22E-01
**AP5M1**	-0.06	8.01E-01	1.00E+00	-0.42	8.44E-02	6.22E-01
**NHLRC2**	-0.12	6.74E-01	1.00E+00	-0.50	8.44E-02	6.22E-01
**GPSM1**	0.01	9.74E-01	1.00E+00	1.20	8.44E-02	6.22E-01
**TOB1-AS1**	0.21	7.43E-01	1.00E+00	-0.84	8.44E-02	6.22E-01
**HMGB1P14**	NA	NA	NA	-2.51	8.44E-02	6.22E-01
**SLC35F2**	-0.01	9.71E-01	1.00E+00	-0.43	8.44E-02	6.22E-01
**EIF3D**	-0.17	4.76E-01	1.00E+00	0.32	8.45E-02	6.22E-01
**RPL39P40**	0.98	3.46E-01	1.00E+00	-2.46	8.45E-02	6.22E-01
**AC090198.1**	0.46	2.61E-01	1.00E+00	1.05	8.45E-02	6.22E-01

**LPAL2**	-0.25	6.01E-01	1.00E+00	1.05	8.45E-02	6.22E-01
**NECAB1**	-0.37	4.40E-01	1.00E+00	-0.92	8.45E-02	6.22E-01
**FCAR**	0.38	9.14E-01	1.00E+00	2.33	8.45E-02	6.22E-01
**RIDA**	0.23	3.72E-01	1.00E+00	-0.71	8.45E-02	6.22E-01
**FEN1**	-0.31	8.46E-02	1.00E+00	0.33	8.45E-02	6.22E-01
**ZC4H2**	-0.06	8.40E-01	1.00E+00	0.62	8.46E-02	6.22E-01
**MS4A7**	0.71	6.91E-02	1.00E+00	0.90	8.46E-02	6.22E-01
**VWDE**	-0.69	4.48E-01	1.00E+00	-2.57	8.47E-02	6.22E-01
**POLD3**	-0.17	2.84E-01	1.00E+00	0.26	8.47E-02	6.22E-01
**CAP1**	-0.17	2.99E-01	1.00E+00	0.34	8.48E-02	6.22E-01
**DUOXA2**	0.43	5.79E-01	1.00E+00	2.77	8.48E-02	6.22E-01
**XLOC_003433**	2.65	1.07E-01	1.00E+00	1.87	8.48E-02	6.22E-01
**SRF**	-0.04	8.24E-01	1.00E+00	1.10	8.49E-02	6.22E-01
**CES1**	1.35	2.39E-02	9.18E-01	-1.26	8.49E-02	6.22E-01
**IRF3**	-0.27	3.37E-01	1.00E+00	0.48	8.49E-02	6.23E-01
**CCDC126**	0.34	3.55E-01	1.00E+00	-0.78	8.50E-02	6.23E-01
**MFF**	-0.01	9.40E-01	1.00E+00	-0.40	8.50E-02	6.23E-01
**PAPLN**	0.49	2.64E-01	1.00E+00	1.03	8.50E-02	6.23E-01
**GLS**	0.31	5.25E-01	1.00E+00	-0.66	8.50E-02	6.23E-01
**WNT5A-AS1**	-0.98	1.96E-01	1.00E+00	2.79	8.51E-02	6.23E-01
**SRSF8**	0.11	5.80E-01	1.00E+00	-0.51	8.51E-02	6.23E-01
**AL021068.2**	NA	NA	NA	2.57	8.51E-02	6.23E-01
**XLOC_008102**	0.90	7.96E-01	1.00E+00	-1.96	8.51E-02	6.23E-01
**AL158151.1**	-0.33	7.45E-01	1.00E+00	1.56	8.51E-02	6.23E-01
**NBPF10**	-0.34	4.99E-01	1.00E+00	0.72	8.53E-02	6.23E-01
**CALM3**	0.08	7.76E-01	1.00E+00	0.51	8.53E-02	6.23E-01
**GLUD1**	0.11	5.48E-01	1.00E+00	-0.62	8.53E-02	6.23E-01
**DLX6-AS1**	0.08	9.12E-01	1.00E+00	-1.20	8.54E-02	6.23E-01
**BAG6**	-0.11	5.75E-01	1.00E+00	0.81	8.54E-02	6.23E-01
**AC105101.1**	-0.97	7.80E-01	1.00E+00	2.22	8.54E-02	6.23E-01
**FRY-AS1**	0.39	9.10E-01	1.00E+00	-2.50	8.54E-02	6.23E-01
**AP000924.1**	-1.38	2.80E-01	1.00E+00	1.86	8.55E-02	6.23E-01

**AL360012.1**	1.54	2.56E-01	1.00E+00	-1.47	8.55E-02	6.23E-01
**MYADM**	0.15	5.51E-01	1.00E+00	0.64	8.55E-02	6.23E-01
**G33836**	0.68	5.76E-01	1.00E+00	-1.83	8.55E-02	6.23E-01
**NDUFAF6**	-0.06	7.90E-01	1.00E+00	-0.50	8.55E-02	6.23E-01
**FGD4**	0.16	5.17E-01	1.00E+00	-0.45	8.55E-02	6.23E-01
**XLOC_001453**	-0.60	8.31E-01	1.00E+00	-1.83	8.56E-02	6.23E-01
**KIF13B**	0.12	6.06E-01	1.00E+00	0.81	8.56E-02	6.23E-01
**PHYH**	0.29	2.59E-01	1.00E+00	-0.62	8.56E-02	6.23E-01
**NSD3**	-0.03	8.58E-01	1.00E+00	-0.31	8.57E-02	6.23E-01
**G25345**	1.68	3.05E-01	1.00E+00	1.94	8.57E-02	6.23E-01
**FES**	-0.12	7.59E-01	1.00E+00	0.76	8.57E-02	6.23E-01
**AL109936.2**	0.41	6.63E-01	1.00E+00	-1.24	8.58E-02	6.23E-01
**PCBP1**	-0.23	2.40E-01	1.00E+00	0.67	8.58E-02	6.23E-01
**ATP5MGL**	0.74	8.29E-01	1.00E+00	-1.56	8.58E-02	6.23E-01
**MEG3**	-0.49	5.10E-01	1.00E+00	1.11	8.59E-02	6.24E-01
**TTC3**	0.28	3.45E-01	1.00E+00	-0.37	8.59E-02	6.24E-01
**C2orf76**	0.19	3.78E-01	1.00E+00	-0.85	8.59E-02	6.24E-01
**TRO**	0.31	5.71E-01	1.00E+00	0.80	8.60E-02	6.24E-01
**CA1**	0.53	8.29E-01	1.00E+00	-2.88	8.61E-02	6.24E-01
**MTERF2**	1.01	5.21E-03	4.20E-01	-0.74	8.61E-02	6.24E-01
**AC002056.1**	1.02	7.67E-01	1.00E+00	-1.14	8.61E-02	6.24E-01
**FAM225B**	0.72	4.90E-01	1.00E+00	2.11	8.61E-02	6.24E-01
**SLC25A17**	0.12	5.22E-01	1.00E+00	-0.47	8.61E-02	6.24E-01
**SMCR8**	-0.01	9.76E-01	1.00E+00	0.90	8.62E-02	6.24E-01
**COQ9**	0.01	9.69E-01	1.00E+00	-0.31	8.62E-02	6.24E-01
**WASF2**	0.07	7.83E-01	1.00E+00	0.34	8.63E-02	6.24E-01
**AC114760.2**	-1.05	6.29E-01	1.00E+00	1.31	8.63E-02	6.24E-01
**PCA3**	-1.51	6.54E-01	1.00E+00	-1.87	8.63E-02	6.24E-01
**SHD**	-10.98	1.34E-03	1.97E-01	3.64	8.63E-02	6.24E-01
**RNU6-2**	-0.56	7.39E-01	1.00E+00	-1.25	8.65E-02	6.25E-01
**LRRC20**	-0.38	1.27E-01	1.00E+00	1.04	8.65E-02	6.25E-01
**AL590705.1**	-1.63	6.33E-01	1.00E+00	-2.24	8.67E-02	6.26E-01

**AC067852.3**	-1.41	4.69E-01	1.00E+00	1.91	8.67E-02	6.26E-01
**SLC37A2**	0.17	7.54E-01	1.00E+00	0.75	8.67E-02	6.26E-01
**CRISPLD2**	0.25	5.05E-01	1.00E+00	0.90	8.67E-02	6.26E-01
**CTHRC1**	0.79	1.80E-01	1.00E+00	0.99	8.68E-02	6.26E-01
**RAB24**	-0.15	7.11E-01	1.00E+00	0.70	8.68E-02	6.26E-01
**AC012358.2**	-0.18	7.68E-01	1.00E+00	0.62	8.68E-02	6.26E-01
**SPNS3**	0.16	7.99E-01	1.00E+00	0.91	8.69E-02	6.26E-01
**FASTKD1**	0.14	6.58E-01	1.00E+00	-0.37	8.69E-02	6.26E-01
**RNF215**	0.26	5.72E-01	1.00E+00	1.63	8.69E-02	6.26E-01
**LINC-PINT**	0.12	8.19E-01	1.00E+00	-0.65	8.69E-02	6.26E-01
**AL583856.2**	-0.88	5.68E-01	1.00E+00	-1.91	8.69E-02	6.26E-01
**LILRA4**	-3.54	2.26E-03	2.41E-01	2.11	8.70E-02	6.26E-01
**FRAT2**	-0.17	6.12E-01	1.00E+00	-0.94	8.70E-02	6.26E-01
**CDK11A**	-0.51	6.67E-01	1.00E+00	1.03	8.70E-02	6.26E-01
**TBC1D10A**	0.28	3.14E-01	1.00E+00	0.33	8.70E-02	6.26E-01
**VSIG4**	0.31	5.56E-01	1.00E+00	1.12	8.72E-02	6.27E-01
**CREB3L2**	0.13	7.37E-01	1.00E+00	0.61	8.73E-02	6.27E-01
**RIMBP2**	-0.76	5.10E-02	1.00E+00	1.18	8.73E-02	6.27E-01
**TSPAN12**	0.56	3.98E-01	1.00E+00	-0.84	8.73E-02	6.27E-01
**PROX2**	-2.20	1.99E-01	1.00E+00	3.28	8.73E-02	6.27E-01
**SHOX2**	0.38	3.74E-01	1.00E+00	0.93	8.74E-02	6.27E-01
**EBPL**	-0.28	2.58E-01	1.00E+00	-1.02	8.74E-02	6.27E-01
**DGCR8**	-0.31	2.27E-01	1.00E+00	0.68	8.74E-02	6.27E-01
**ANAPC10**	-0.03	9.34E-01	1.00E+00	-0.89	8.74E-02	6.27E-01
**BCAP29**	0.15	4.32E-01	1.00E+00	-0.48	8.75E-02	6.28E-01
**MICB**	-0.01	9.80E-01	1.00E+00	1.46	8.76E-02	6.28E-01
**CORT**	-1.29	5.61E-01	1.00E+00	-1.91	8.76E-02	6.28E-01
**MT1H**	-0.05	9.56E-01	1.00E+00	-1.44	8.76E-02	6.28E-01
**CDC42SE1**	-0.26	3.58E-01	1.00E+00	0.92	8.76E-02	6.28E-01
**APIP**	0.30	3.74E-01	1.00E+00	-0.48	8.76E-02	6.28E-01
**COX10-AS1**	-0.07	8.42E-01	1.00E+00	-0.56	8.77E-02	6.28E-01
**OS9**	0.01	9.61E-01	1.00E+00	0.39	8.77E-02	6.28E-01

**CHST15**	-0.01	9.75E-01	1.00E+00	0.72	8.77E-02	6.28E-01
**TCF3P1**	-2.85	2.17E-01	1.00E+00	2.81	8.78E-02	6.28E-01
**UBA6-AS1**	0.26	5.63E-01	1.00E+00	-0.47	8.79E-02	6.28E-01
**ABCB10**	0.37	2.33E-01	1.00E+00	-0.84	8.79E-02	6.28E-01
**IGLV5-37**	NA	NA	NA	5.40	8.79E-02	6.28E-01
**HLA-DRA**	-0.51	1.52E-01	1.00E+00	0.77	8.80E-02	6.28E-01
**AP003555.2**	0.05	9.81E-01	1.00E+00	1.47	8.80E-02	6.28E-01
**CCDC157**	0.01	9.89E-01	1.00E+00	0.72	8.80E-02	6.28E-01
**IL23A**	-1.26	1.75E-01	1.00E+00	1.26	8.80E-02	6.28E-01
**WDR66**	-0.36	4.60E-01	1.00E+00	1.17	8.81E-02	6.28E-01
**RBBP9**	-0.35	1.30E-01	1.00E+00	-0.46	8.81E-02	6.28E-01
**PEX5L**	-1.22	7.11E-01	1.00E+00	2.26	8.81E-02	6.28E-01
**RAB26**	-1.01	3.00E-02	9.97E-01	-1.10	8.81E-02	6.28E-01
**GAL3ST2**	-2.29	1.91E-01	1.00E+00	2.15	8.81E-02	6.28E-01
**GATD3B**	-1.32	5.06E-01	1.00E+00	1.45	8.81E-02	6.28E-01
**FPR1**	0.12	8.53E-01	1.00E+00	1.63	8.82E-02	6.29E-01
**TMEM222**	-0.04	8.84E-01	1.00E+00	0.25	8.83E-02	6.29E-01
**KMO**	0.21	8.11E-01	1.00E+00	2.17	8.83E-02	6.29E-01
**AC243964.2**	0.72	4.65E-01	1.00E+00	-1.31	8.83E-02	6.29E-01
**AP000766.1**	-0.45	3.74E-01	1.00E+00	-0.93	8.83E-02	6.29E-01
**COX14**	0.41	1.33E-01	1.00E+00	-0.71	8.84E-02	6.29E-01
**KLHL30**	1.62	4.95E-02	1.00E+00	1.25	8.84E-02	6.29E-01
**LHPP**	-0.10	7.09E-01	1.00E+00	0.28	8.84E-02	6.29E-01
**C5orf24**	0.24	4.30E-01	1.00E+00	-0.36	8.86E-02	6.30E-01
**AC064805.1**	-2.61	4.36E-01	1.00E+00	1.81	8.86E-02	6.30E-01
**SCGN**	-0.13	9.69E-01	1.00E+00	-2.70	8.86E-02	6.30E-01
**GPAT2**	-1.05	1.56E-01	1.00E+00	-1.12	8.86E-02	6.30E-01
**G34083**	-0.05	9.88E-01	1.00E+00	2.32	8.87E-02	6.30E-01
**SLCO2B1**	0.24	5.33E-01	1.00E+00	1.19	8.87E-02	6.30E-01
**WDR5B**	0.30	4.73E-01	1.00E+00	-0.58	8.88E-02	6.30E-01
**ZSCAN5A**	-0.17	5.48E-01	1.00E+00	-0.46	8.88E-02	6.30E-01
**ARIH2**	0.04	8.15E-01	1.00E+00	0.25	8.88E-02	6.30E-01

**COL4A3**	-1.84	6.72E-02	1.00E+00	1.64	8.88E-02	6.30E-01
**TRGC2**	0.54	5.92E-01	1.00E+00	1.97	8.89E-02	6.30E-01
**AL645939.5**	-0.13	8.54E-01	1.00E+00	1.03	8.89E-02	6.30E-01
**G6PD**	-0.21	4.47E-01	1.00E+00	0.80	8.90E-02	6.30E-01
**DENR**	-0.03	8.93E-01	1.00E+00	-0.33	8.90E-02	6.30E-01
**AC007342.1**	1.72	4.77E-01	1.00E+00	2.12	8.90E-02	6.30E-01
**TMEM219**	0.03	8.83E-01	1.00E+00	-0.37	8.90E-02	6.30E-01
**DHRS11**	-0.11	7.40E-01	1.00E+00	-1.14	8.91E-02	6.30E-01
**AL445490.1**	1.72	2.26E-01	1.00E+00	2.46	8.91E-02	6.30E-01
**KCNG1**	-1.10	4.89E-02	1.00E+00	1.41	8.91E-02	6.30E-01
**ARHGDIA**	-0.32	1.13E-01	1.00E+00	0.72	8.91E-02	6.30E-01
**ROPN1**	0.76	4.70E-01	1.00E+00	-1.00	8.91E-02	6.30E-01
**KBTBD6**	0.30	3.19E-01	1.00E+00	-0.50	8.92E-02	6.30E-01
**ADAMTS6**	1.07	2.67E-01	1.00E+00	1.16	8.92E-02	6.30E-01
**SNORA59B**	-0.80	8.18E-01	1.00E+00	-1.96	8.92E-02	6.30E-01
**AC006299.1**	0.20	8.99E-01	1.00E+00	-1.64	8.92E-02	6.30E-01
**YWHAG**	0.18	5.15E-01	1.00E+00	0.78	8.93E-02	6.30E-01
**CTNS**	-0.19	3.83E-01	1.00E+00	0.52	8.93E-02	6.30E-01
**AC026770.1**	NA	NA	NA	-3.45	8.93E-02	6.30E-01
**HBD**	2.36	2.27E-01	1.00E+00	2.01	8.93E-02	6.30E-01
**EGLN1**	0.05	8.39E-01	1.00E+00	-0.67	8.93E-02	6.30E-01
**USP30-AS1**	-1.04	7.63E-01	1.00E+00	1.34	8.93E-02	6.30E-01
**NQO1**	0.87	2.04E-01	1.00E+00	-0.89	8.95E-02	6.30E-01
**THRB-IT1**	1.40	4.55E-01	1.00E+00	1.17	8.95E-02	6.30E-01
**G43485**	-1.83	9.79E-02	1.00E+00	-1.90	8.95E-02	6.30E-01
**SCP2**	0.01	9.48E-01	1.00E+00	-0.54	8.97E-02	6.32E-01
**GFI1**	0.30	6.93E-01	1.00E+00	2.69	8.98E-02	6.32E-01
**CYP2S1**	0.32	2.55E-01	1.00E+00	0.91	8.98E-02	6.32E-01
**AC011477.7**	-0.18	8.79E-01	1.00E+00	2.12	8.98E-02	6.32E-01
**XLOC_003422**	-0.28	6.43E-01	1.00E+00	1.27	8.98E-02	6.32E-01
**AL357078.3**	-0.63	8.55E-01	1.00E+00	2.36	8.99E-02	6.32E-01
**LGI3**	-0.74	6.36E-02	1.00E+00	-1.34	9.00E-02	6.32E-01

**BMT2**	0.12	7.23E-01	1.00E+00	-0.31	9.00E-02	6.32E-01
**G17366**	0.04	9.82E-01	1.00E+00	3.37	9.00E-02	6.32E-01
**AC087783.2**	NA	NA	NA	-3.62	9.00E-02	6.32E-01
**OTOAP1**	1.18	6.06E-01	1.00E+00	1.70	9.00E-02	6.32E-01
**ZFAND3**	-0.19	4.39E-01	1.00E+00	0.88	9.00E-02	6.32E-01
**STXBP2**	0.01	9.85E-01	1.00E+00	0.63	9.01E-02	6.32E-01
**PLEKHM1P1**	-0.28	3.83E-01	1.00E+00	0.52	9.02E-02	6.32E-01
**AP001207.3**	0.17	7.58E-01	1.00E+00	-1.10	9.02E-02	6.32E-01
**G38114**	-2.96	4.82E-02	1.00E+00	1.46	9.02E-02	6.32E-01
**AC040160.1**	-1.05	4.50E-02	1.00E+00	0.74	9.03E-02	6.32E-01
**GDI1**	0.04	8.25E-01	1.00E+00	0.30	9.03E-02	6.32E-01
**NPM1P25**	-0.30	5.23E-01	1.00E+00	1.06	9.03E-02	6.32E-01
**CCDC102B**	-0.63	1.67E-01	1.00E+00	0.88	9.04E-02	6.32E-01
**STEAP3**	-0.78	7.06E-03	5.12E-01	0.83	9.04E-02	6.32E-01
**AC010226.1**	-0.23	7.46E-01	1.00E+00	-0.87	9.04E-02	6.32E-01
**UQCRC2**	-0.05	8.01E-01	1.00E+00	-0.19	9.04E-02	6.32E-01
**CYP8B1**	-1.18	4.21E-01	1.00E+00	1.88	9.04E-02	6.32E-01
**AC027013.1**	3.37	3.10E-01	1.00E+00	-2.95	9.05E-02	6.32E-01
**CPA6**	-2.17	5.23E-01	1.00E+00	2.53	9.05E-02	6.32E-01
**CBWD2**	-0.05	7.85E-01	1.00E+00	-0.38	9.05E-02	6.32E-01
**TCEANC**	0.61	1.22E-01	1.00E+00	-0.50	9.05E-02	6.32E-01
**DMAC1**	0.21	2.98E-01	1.00E+00	-0.46	9.05E-02	6.32E-01
**MAGOH**	-0.26	2.37E-01	1.00E+00	0.46	9.05E-02	6.32E-01
**PCP2**	2.54	1.62E-01	1.00E+00	-1.62	9.06E-02	6.32E-01
**C14orf39**	0.90	7.96E-01	1.00E+00	-1.79	9.06E-02	6.32E-01
**SH2B3**	0.07	8.49E-01	1.00E+00	1.18	9.07E-02	6.32E-01
**PCDHGA10**	-0.62	6.51E-02	1.00E+00	0.42	9.07E-02	6.32E-01
**AP004782.1**	-2.03	4.40E-01	1.00E+00	-2.82	9.07E-02	6.32E-01
**ARAP1**	-0.01	9.72E-01	1.00E+00	0.92	9.07E-02	6.32E-01
**MCHR1**	-0.70	1.49E-01	1.00E+00	1.84	9.07E-02	6.32E-01
**LGALS9C**	-1.89	2.54E-01	1.00E+00	1.48	9.08E-02	6.32E-01
**CR1**	0.37	6.78E-01	1.00E+00	1.89	9.08E-02	6.32E-01

**WDSUB1**	-0.24	4.50E-01	1.00E+00	-0.48	9.08E-02	6.32E-01
**CCK**	-2.71	2.41E-02	9.18E-01	1.39	9.09E-02	6.32E-01
**ZNF528**	0.58	1.24E-01	1.00E+00	-0.40	9.09E-02	6.32E-01
**PRR12**	-0.52	8.82E-02	1.00E+00	1.36	9.09E-02	6.32E-01
**KCNIP4**	-0.55	3.06E-01	1.00E+00	-0.85	9.09E-02	6.32E-01
**AL121772.3**	0.20	8.13E-01	1.00E+00	2.07	9.09E-02	6.32E-01
**BAG1**	-0.02	9.14E-01	1.00E+00	-0.66	9.10E-02	6.32E-01
**XLOC_004517**	4.89	4.51E-04	9.15E-02	-2.00	9.10E-02	6.32E-01
**RNU5D-1**	-0.83	6.98E-01	1.00E+00	-1.62	9.10E-02	6.32E-01
**LINC02591**	-0.53	1.02E-01	1.00E+00	0.87	9.10E-02	6.32E-01
**SMN2**	-1.02	3.57E-01	1.00E+00	-1.04	9.11E-02	6.32E-01
**LARP4P**	0.56	8.71E-01	1.00E+00	-1.99	9.11E-02	6.32E-01
**CDKN2C**	0.67	9.31E-02	1.00E+00	-0.79	9.11E-02	6.32E-01
**MMP7**	0.23	6.85E-01	1.00E+00	0.90	9.11E-02	6.32E-01
**AC092135.1**	1.13	7.36E-01	1.00E+00	2.93	9.11E-02	6.32E-01
**CABIN1**	-0.31	2.52E-01	1.00E+00	1.02	9.11E-02	6.32E-01
**ATG101**	-0.11	6.60E-01	1.00E+00	0.41	9.12E-02	6.32E-01
**G34419**	1.78	4.08E-01	1.00E+00	-2.37	9.12E-02	6.32E-01
**TIAF1**	0.14	8.12E-01	1.00E+00	1.19	9.12E-02	6.32E-01
**GOLGA6L5P**	0.45	6.55E-01	1.00E+00	1.28	9.13E-02	6.32E-01
**PDGFB**	-0.14	7.39E-01	1.00E+00	1.03	9.13E-02	6.32E-01
**AC110994.2**	-1.26	7.16E-01	1.00E+00	-1.10	9.13E-02	6.32E-01
**TCEAL3**	-0.09	7.61E-01	1.00E+00	0.45	9.14E-02	6.32E-01
**ZNF26**	0.22	4.32E-01	1.00E+00	0.37	9.14E-02	6.32E-01
**DMD**	0.01	9.61E-01	1.00E+00	-0.42	9.14E-02	6.32E-01
**TBC1D2**	-0.16	6.33E-01	1.00E+00	0.68	9.14E-02	6.32E-01
**ZNF213-AS1**	-0.44	7.46E-02	1.00E+00	-0.55	9.15E-02	6.32E-01
**ZC3HAV1**	0.21	4.35E-01	1.00E+00	0.31	9.16E-02	6.33E-01
**DDX43**	0.11	9.09E-01	1.00E+00	-1.47	9.16E-02	6.33E-01
**AL160314.2**	2.88	9.46E-02	1.00E+00	-2.38	9.17E-02	6.33E-01
**C4orf19**	0.46	5.39E-01	1.00E+00	-0.83	9.17E-02	6.33E-01
**TSPAN6**	0.41	3.48E-01	1.00E+00	-0.61	9.18E-02	6.33E-01

**NPFFR1**	-0.41	4.74E-01	1.00E+00	1.93	9.18E-02	6.33E-01
**FAM117A**	0.03	9.12E-01	1.00E+00	-0.72	9.18E-02	6.33E-01
**HLX**	0.12	7.34E-01	1.00E+00	0.85	9.19E-02	6.33E-01
**MIF4GD**	-0.14	4.53E-01	1.00E+00	-0.24	9.19E-02	6.33E-01
**AC025154.2**	-0.16	8.86E-01	1.00E+00	-1.22	9.19E-02	6.33E-01
**SMARCB1**	-0.28	1.03E-01	1.00E+00	0.30	9.20E-02	6.34E-01
**FBXL8**	-0.87	1.89E-02	8.62E-01	0.42	9.20E-02	6.34E-01
**G8412**	-0.58	3.79E-01	1.00E+00	1.07	9.22E-02	6.35E-01
**RPL28**	-0.20	3.26E-01	1.00E+00	0.80	9.22E-02	6.35E-01
**EVI5**	0.41	6.62E-02	1.00E+00	-0.50	9.23E-02	6.35E-01
**TP53TG5**	-0.14	8.72E-01	1.00E+00	1.52	9.23E-02	6.35E-01
**FDX1**	0.16	5.01E-01	1.00E+00	-0.84	9.23E-02	6.35E-01
**FAM167A-AS1**	1.01	3.64E-01	1.00E+00	-1.78	9.24E-02	6.35E-01
**TPH1**	1.78	2.54E-01	1.00E+00	-1.62	9.24E-02	6.35E-01
**INO80E**	-0.14	5.82E-01	1.00E+00	0.77	9.24E-02	6.35E-01
**TPRKB**	0.11	6.46E-01	1.00E+00	-0.61	9.25E-02	6.35E-01
**C2orf50**	-1.89	3.08E-01	1.00E+00	-1.62	9.25E-02	6.35E-01
**C11orf24**	-0.34	2.42E-01	1.00E+00	0.39	9.25E-02	6.35E-01
**LINC01011**	0.55	2.53E-01	1.00E+00	-0.99	9.26E-02	6.35E-01
**G15458**	-0.62	6.52E-01	1.00E+00	-2.35	9.26E-02	6.35E-01
**ISM2**	1.23	5.13E-01	1.00E+00	2.20	9.26E-02	6.35E-01
**AC015799.1**	-0.21	9.51E-01	1.00E+00	2.44	9.27E-02	6.35E-01
**THNSL1**	0.07	8.75E-01	1.00E+00	-0.75	9.28E-02	6.36E-01
**BOLA3**	-0.17	6.33E-01	1.00E+00	-0.61	9.28E-02	6.36E-01
**OTUD7B**	-0.03	9.14E-01	1.00E+00	0.72	9.29E-02	6.37E-01
**FDFT1**	-0.25	3.24E-01	1.00E+00	-0.48	9.30E-02	6.37E-01
**IER3**	-1.31	1.83E-02	8.50E-01	0.87	9.30E-02	6.37E-01
**SEC61A2**	-0.35	3.82E-01	1.00E+00	-0.79	9.31E-02	6.37E-01
**THCAT158**	-0.54	5.67E-01	1.00E+00	1.63	9.31E-02	6.37E-01
**SSH2**	0.20	4.07E-01	1.00E+00	0.45	9.31E-02	6.37E-01
**OR7E94P**	-0.29	7.91E-01	1.00E+00	-2.70	9.31E-02	6.37E-01
**LACRT**	-1.43	3.02E-01	1.00E+00	-1.81	9.31E-02	6.37E-01

**GAPDHP52**	-2.49	2.14E-01	1.00E+00	2.94	9.32E-02	6.37E-01
**DCAF10**	-0.01	9.71E-01	1.00E+00	-0.48	9.32E-02	6.37E-01
**DPP6**	-1.36	1.05E-02	6.35E-01	-1.17	9.32E-02	6.37E-01
**WNT7A**	-1.38	1.55E-03	2.18E-01	1.28	9.34E-02	6.38E-01
**AP001528.1**	3.15	7.48E-02	1.00E+00	-1.61	9.34E-02	6.38E-01
**G27754**	0.29	7.47E-01	1.00E+00	-1.26	9.34E-02	6.38E-01
**HDAC7**	0.18	4.61E-01	1.00E+00	0.80	9.36E-02	6.38E-01
**HSD17B14**	0.00	9.97E-01	1.00E+00	0.76	9.36E-02	6.38E-01
**COL13A1**	-0.31	4.89E-01	1.00E+00	1.04	9.36E-02	6.38E-01
**CDK4**	-0.05	7.64E-01	1.00E+00	0.24	9.37E-02	6.38E-01
**RFXANK**	-0.14	4.62E-01	1.00E+00	0.29	9.37E-02	6.38E-01
**XLOC_005458**	0.19	9.23E-01	1.00E+00	2.07	9.37E-02	6.38E-01
**HTRA3**	0.88	8.31E-02	1.00E+00	1.24	9.37E-02	6.38E-01
**PUSL1**	-0.14	6.66E-01	1.00E+00	0.42	9.37E-02	6.38E-01
**AC026801.2**	1.20	6.35E-02	1.00E+00	1.06	9.38E-02	6.38E-01
**AQP11**	0.23	7.98E-01	1.00E+00	-1.24	9.38E-02	6.38E-01
**COMMD5**	0.22	3.77E-01	1.00E+00	0.31	9.39E-02	6.39E-01
**WDR82P2**	-1.41	5.26E-01	1.00E+00	-2.33	9.39E-02	6.39E-01
**EIF2AK2**	0.32	5.05E-01	1.00E+00	0.63	9.40E-02	6.39E-01
**ADGRL1**	-0.12	6.86E-01	1.00E+00	0.83	9.40E-02	6.39E-01
**XLOC_003378**	-0.96	5.45E-01	1.00E+00	1.83	9.40E-02	6.39E-01
**TCL1A**	-0.06	9.86E-01	1.00E+00	2.67	9.41E-02	6.39E-01
**AC104819.1**	0.56	8.71E-01	1.00E+00	-3.43	9.42E-02	6.39E-01
**NLRC4**	-0.64	4.44E-01	1.00E+00	1.61	9.42E-02	6.39E-01
**CCL8**	-0.74	2.60E-01	1.00E+00	1.33	9.42E-02	6.39E-01
**GTPBP2**	-0.01	9.58E-01	1.00E+00	1.14	9.42E-02	6.39E-01
**FBXO36**	-0.09	7.17E-01	1.00E+00	-0.66	9.42E-02	6.40E-01
**ROPN1B**	0.32	6.92E-01	1.00E+00	-1.01	9.43E-02	6.40E-01
**ARL14EP**	0.23	3.30E-01	1.00E+00	-0.47	9.43E-02	6.40E-01
**PES1**	-0.12	5.97E-01	1.00E+00	0.38	9.44E-02	6.40E-01
**ZNF480**	-0.02	9.43E-01	1.00E+00	-0.43	9.45E-02	6.41E-01
**TRAF4**	-0.34	3.31E-01	1.00E+00	0.84	9.46E-02	6.41E-01

**AGBL3**	-0.24	5.91E-01	1.00E+00	-0.63	9.47E-02	6.41E-01
**LINC00603**	-0.46	8.94E-01	1.00E+00	2.17	9.47E-02	6.42E-01
**RPL10P7**	-2.75	4.09E-01	1.00E+00	1.95	9.48E-02	6.42E-01
**ATP2A2**	-0.24	2.06E-01	1.00E+00	0.90	9.48E-02	6.42E-01
**PTGES3**	0.03	8.47E-01	1.00E+00	-0.22	9.48E-02	6.42E-01
**MTCO2P12**	-0.71	6.35E-01	1.00E+00	-1.21	9.48E-02	6.42E-01
**ETFRF1**	-0.25	3.88E-01	1.00E+00	-0.64	9.49E-02	6.42E-01
**NOSIP**	-0.02	8.96E-01	1.00E+00	0.41	9.49E-02	6.42E-01
**SLCO4C1**	-0.55	5.28E-01	1.00E+00	-2.09	9.49E-02	6.42E-01
**KBTBD3**	0.14	6.44E-01	1.00E+00	-0.82	9.49E-02	6.42E-01
**FRYL**	0.35	1.98E-01	1.00E+00	0.41	9.50E-02	6.42E-01
**SAMD9L**	-0.19	7.61E-01	1.00E+00	1.03	9.50E-02	6.42E-01
**ZHX1-C8orf76**	1.96	5.66E-01	1.00E+00	-1.34	9.50E-02	6.42E-01
**BMS1P4**	-2.13	2.38E-01	1.00E+00	1.52	9.50E-02	6.42E-01
**MAST4-AS1**	0.75	2.14E-01	1.00E+00	-0.97	9.51E-02	6.42E-01
**VPS26B**	0.15	5.13E-01	1.00E+00	-0.57	9.51E-02	6.42E-01
**MRPL30**	0.05	8.12E-01	1.00E+00	-0.31	9.51E-02	6.42E-01
**CXorf58**	-0.77	5.32E-01	1.00E+00	-2.04	9.52E-02	6.42E-01
**C6orf141**	-0.23	6.08E-01	1.00E+00	-0.69	9.52E-02	6.42E-01
**ADAMTSL1**	0.88	1.09E-01	1.00E+00	1.10	9.53E-02	6.42E-01
**AP000560.1**	-0.07	9.24E-01	1.00E+00	-1.10	9.54E-02	6.42E-01
**ADIRF-AS1**	-0.68	8.95E-02	1.00E+00	-0.55	9.54E-02	6.42E-01
**XLOC_006677**	2.77	3.25E-01	1.00E+00	5.18	9.54E-02	6.42E-01
**AC016027.2**	-1.05	6.96E-01	1.00E+00	3.58	9.55E-02	6.42E-01
**UNKL**	0.04	8.96E-01	1.00E+00	0.45	9.55E-02	6.42E-01
**PRIMPOL**	0.10	7.29E-01	1.00E+00	-0.45	9.55E-02	6.42E-01
**SHROOM2**	-0.13	6.64E-01	1.00E+00	0.71	9.56E-02	6.42E-01
**UQCRFS1**	-0.04	8.51E-01	1.00E+00	-0.46	9.56E-02	6.42E-01
**MICU3**	0.23	7.70E-01	1.00E+00	-1.09	9.56E-02	6.42E-01
**ZNF608**	0.27	4.88E-01	1.00E+00	0.77	9.57E-02	6.42E-01
**Z97192.3**	-1.25	7.16E-01	1.00E+00	2.04	9.57E-02	6.42E-01
**LINC01214**	1.89	1.13E-01	1.00E+00	1.08	9.57E-02	6.42E-01

**AC138207.4**	0.32	7.56E-01	1.00E+00	1.01	9.57E-02	6.42E-01
**G35278**	-0.24	7.37E-01	1.00E+00	-1.17	9.57E-02	6.42E-01
**CYP4Z1**	1.88	4.15E-01	1.00E+00	1.95	9.58E-02	6.42E-01
**GPR1**	-0.02	9.67E-01	1.00E+00	0.72	9.58E-02	6.42E-01
**ECHDC2**	0.03	9.03E-01	1.00E+00	-0.37	9.58E-02	6.42E-01
**XLOC_000398**	0.27	7.57E-01	1.00E+00	0.68	9.58E-02	6.42E-01
**AC093909.1**	-0.52	4.36E-01	1.00E+00	-0.62	9.59E-02	6.42E-01
**G18355**	1.25	1.49E-01	1.00E+00	-1.53	9.59E-02	6.42E-01
**RCAN1**	0.20	4.48E-01	1.00E+00	-0.35	9.59E-02	6.42E-01
**CPO**	1.29	4.10E-01	1.00E+00	-2.44	9.59E-02	6.42E-01
**G18793**	-0.52	6.94E-01	1.00E+00	1.46	9.59E-02	6.42E-01
**TMCC1-AS1**	0.54	4.98E-01	1.00E+00	1.13	9.60E-02	6.42E-01
**A2ML1**	0.41	4.21E-01	1.00E+00	1.20	9.60E-02	6.42E-01
**ASAP1**	-0.31	1.51E-01	1.00E+00	0.60	9.60E-02	6.42E-01
**SLC22A12**	3.75	2.63E-01	1.00E+00	-3.10	9.60E-02	6.42E-01
**AL162377.1**	-0.22	5.61E-01	1.00E+00	-1.05	9.61E-02	6.42E-01
**AL390955.2**	-0.57	6.02E-01	1.00E+00	1.05	9.61E-02	6.42E-01
**AC040169.1**	-0.41	4.88E-01	1.00E+00	0.48	9.61E-02	6.42E-01
**OAS2**	-0.08	9.45E-01	1.00E+00	1.33	9.62E-02	6.42E-01
**ACSM3**	-0.76	8.91E-02	1.00E+00	-1.15	9.62E-02	6.42E-01
**CRYAB**	1.00	2.77E-02	9.71E-01	-0.84	9.63E-02	6.42E-01
**FAT4**	0.48	2.36E-01	1.00E+00	1.07	9.63E-02	6.42E-01
**G4024**	-0.86	8.05E-01	1.00E+00	2.22	9.63E-02	6.42E-01
**MLLT6**	-0.04	8.84E-01	1.00E+00	0.83	9.63E-02	6.42E-01
**PVR**	-0.21	3.66E-01	1.00E+00	0.63	9.63E-02	6.42E-01
**INPP5A**	-0.03	9.04E-01	1.00E+00	-1.22	9.63E-02	6.42E-01
**CETN4P**	0.50	6.39E-01	1.00E+00	-1.67	9.64E-02	6.42E-01
**AC126474.2**	0.13	6.75E-01	1.00E+00	0.69	9.64E-02	6.42E-01
**AC005162.3**	-0.01	9.95E-01	1.00E+00	-1.02	9.64E-02	6.42E-01
**HGF**	0.14	7.87E-01	1.00E+00	1.08	9.64E-02	6.42E-01
**CREBL2**	0.38	6.72E-02	1.00E+00	-0.53	9.65E-02	6.42E-01
**KRT10**	-0.03	9.41E-01	1.00E+00	-1.38	9.65E-02	6.42E-01

**BCAM**	-0.15	7.22E-01	1.00E+00	1.12	9.65E-02	6.42E-01
**FLJ20021**	-0.08	7.63E-01	1.00E+00	0.49	9.65E-02	6.42E-01
**CAMKMT**	-0.03	9.15E-01	1.00E+00	-0.47	9.65E-02	6.42E-01
**MAP4K1**	-0.40	3.60E-01	1.00E+00	1.25	9.65E-02	6.42E-01
**CKS1B**	0.01	9.25E-01	1.00E+00	-0.44	9.65E-02	6.42E-01
**SLC5A8**	-2.66	4.90E-02	1.00E+00	-1.80	9.66E-02	6.42E-01
**IGF1R**	0.32	2.78E-01	1.00E+00	0.58	9.66E-02	6.42E-01
**FAM157A**	-0.31	7.23E-01	1.00E+00	1.58	9.66E-02	6.42E-01
**IGKV2-24**	4.24	2.06E-01	1.00E+00	2.61	9.66E-02	6.42E-01
**TACC3**	-0.36	2.59E-01	1.00E+00	0.78	9.66E-02	6.42E-01
**CA9**	0.19	7.01E-01	1.00E+00	1.15	9.67E-02	6.42E-01
**PPARD**	-0.21	3.93E-01	1.00E+00	0.95	9.67E-02	6.42E-01
**HLA-L**	-0.10	9.20E-01	1.00E+00	0.89	9.68E-02	6.42E-01
**VPS37B**	0.01	9.63E-01	1.00E+00	0.51	9.68E-02	6.42E-01
**STAU2**	-0.01	9.63E-01	1.00E+00	-0.40	9.69E-02	6.42E-01
**AL138756.1**	1.50	2.06E-02	8.94E-01	1.52	9.69E-02	6.42E-01
**EGFR-AS1**	0.51	7.85E-01	1.00E+00	2.18	9.69E-02	6.42E-01
**ARHGAP17**	-0.11	5.91E-01	1.00E+00	0.46	9.70E-02	6.42E-01
**NDUFB9**	-0.03	9.02E-01	1.00E+00	-0.46	9.70E-02	6.42E-01
**KRT18**	0.07	9.29E-01	1.00E+00	-0.93	9.70E-02	6.42E-01
**SPRN**	-0.87	5.10E-02	1.00E+00	1.41	9.70E-02	6.42E-01
**AC103591.3**	-2.89	3.81E-01	1.00E+00	-1.52	9.71E-02	6.42E-01
**SPON1**	0.72	1.09E-01	1.00E+00	0.88	9.71E-02	6.42E-01
**WNT4**	-0.31	4.22E-01	1.00E+00	-1.04	9.71E-02	6.43E-01
**G40351**	0.10	9.31E-01	1.00E+00	-1.04	9.72E-02	6.43E-01
**AC021078.1**	0.82	1.27E-01	1.00E+00	0.46	9.72E-02	6.43E-01
**GASAL1**	-0.44	3.77E-01	1.00E+00	0.79	9.72E-02	6.43E-01
**AC023794.1**	1.08	4.09E-01	1.00E+00	-1.53	9.72E-02	6.43E-01
**ADGRG3**	1.59	1.34E-01	1.00E+00	1.94	9.73E-02	6.43E-01
**ITGB8**	-0.17	5.92E-01	1.00E+00	-0.74	9.73E-02	6.43E-01
**GLB1**	0.25	3.55E-01	1.00E+00	0.40	9.73E-02	6.43E-01
**RTN4**	0.13	5.90E-01	1.00E+00	-0.60	9.73E-02	6.43E-01

**SERINC3**	0.33	3.98E-02	1.00E+00	-0.32	9.74E-02	6.43E-01
**VLDLR-AS1**	3.78	2.19E-02	8.96E-01	-1.72	9.74E-02	6.43E-01
**CA4**	2.23	1.08E-02	6.40E-01	-1.31	9.75E-02	6.43E-01
**PCGF3**	0.15	5.99E-01	1.00E+00	0.35	9.75E-02	6.43E-01
**FANCC**	0.23	3.22E-01	1.00E+00	-0.44	9.75E-02	6.43E-01
**SPI1**	-0.54	1.69E-01	1.00E+00	1.60	9.75E-02	6.43E-01
**GLDC**	0.16	8.04E-01	1.00E+00	-1.66	9.76E-02	6.43E-01
**TEKT4P2**	0.80	5.27E-02	1.00E+00	0.65	9.76E-02	6.43E-01
**CD276**	-0.30	3.83E-01	1.00E+00	0.77	9.77E-02	6.43E-01
**CDKN1B**	0.16	1.94E-01	1.00E+00	0.74	9.78E-02	6.43E-01
**PSMB4**	-0.01	9.53E-01	1.00E+00	0.44	9.78E-02	6.43E-01
**FGL2**	0.37	4.00E-01	1.00E+00	0.75	9.78E-02	6.43E-01
**AC009812.4**	0.81	5.11E-01	1.00E+00	1.65	9.78E-02	6.43E-01
**AP005131.7**	-1.78	5.55E-01	1.00E+00	1.67	9.78E-02	6.43E-01
**AC005291.2**	0.76	8.26E-01	1.00E+00	2.45	9.78E-02	6.43E-01
**DNAJA2**	-0.01	9.73E-01	1.00E+00	-0.20	9.79E-02	6.43E-01
**PDIA4**	0.01	9.46E-01	1.00E+00	0.37	9.79E-02	6.43E-01
**N4BP2L2-IT2**	1.04	3.54E-01	1.00E+00	-1.83	9.79E-02	6.43E-01
**CTGF**	-0.11	8.08E-01	1.00E+00	0.76	9.80E-02	6.44E-01
**SLC6A14**	0.19	6.33E-01	1.00E+00	0.91	9.81E-02	6.44E-01
**NAA60**	-0.21	4.11E-01	1.00E+00	0.64	9.83E-02	6.45E-01
**ST3GAL5**	0.55	1.21E-01	1.00E+00	0.64	9.83E-02	6.45E-01
**1-Sep**	-0.06	9.03E-01	1.00E+00	0.82	9.83E-02	6.45E-01
**DUSP14**	-0.21	5.87E-01	1.00E+00	0.47	9.83E-02	6.45E-01
**CRTAM**	0.26	7.62E-01	1.00E+00	1.56	9.84E-02	6.45E-01
**ZSWIM4**	-0.60	9.83E-02	1.00E+00	1.44	9.84E-02	6.45E-01
**RELL2**	-1.04	3.68E-02	1.00E+00	1.05	9.84E-02	6.45E-01
**UBE2E1**	-0.01	9.69E-01	1.00E+00	0.54	9.84E-02	6.45E-01
**ZCRB1**	-0.07	6.77E-01	1.00E+00	-0.34	9.84E-02	6.45E-01
**RGS3**	-0.06	8.40E-01	1.00E+00	0.72	9.85E-02	6.45E-01
**DYRK1A**	0.03	9.03E-01	1.00E+00	0.61	9.85E-02	6.45E-01
**HPSE2**	0.40	5.19E-01	1.00E+00	-1.10	9.85E-02	6.45E-01

**SETD1B**	-0.45	1.05E-01	1.00E+00	0.92	9.86E-02	6.45E-01
**TUBA1C**	-0.26	4.83E-01	1.00E+00	0.63	9.86E-02	6.45E-01
**AC000123.3**	0.24	5.92E-01	1.00E+00	0.99	9.86E-02	6.45E-01
**ARHGEF2**	0.19	7.02E-01	1.00E+00	1.19	9.87E-02	6.45E-01
**XLOC_009668**	0.27	7.93E-01	1.00E+00	-1.07	9.88E-02	6.45E-01
**RPS12P16**	2.05	5.34E-01	1.00E+00	-2.43	9.88E-02	6.45E-01
**SOAT2**	-1.64	3.70E-01	1.00E+00	1.44	9.88E-02	6.45E-01
**XPNPEP2**	0.76	1.46E-01	1.00E+00	0.95	9.88E-02	6.45E-01
**OXR1**	0.20	4.29E-01	1.00E+00	-0.38	9.88E-02	6.45E-01
**STRBP**	-0.38	6.82E-02	1.00E+00	-0.51	9.88E-02	6.45E-01
**BCO2**	0.32	6.67E-01	1.00E+00	1.33	9.89E-02	6.46E-01
**MTG2**	0.09	7.20E-01	1.00E+00	0.42	9.90E-02	6.46E-01
**HIST1H1A**	-0.80	8.18E-01	1.00E+00	-1.42	9.90E-02	6.46E-01
**OTUD1**	0.19	5.18E-01	1.00E+00	-0.81	9.91E-02	6.46E-01
**RPL23AP7**	0.30	2.71E-01	1.00E+00	-0.45	9.91E-02	6.46E-01
**TMEM161B**	0.35	1.70E-01	1.00E+00	-0.87	9.91E-02	6.46E-01
**AC011290.1**	-0.34	9.20E-01	1.00E+00	2.33	9.91E-02	6.46E-01
**CLN6**	-0.42	1.26E-01	1.00E+00	0.81	9.92E-02	6.46E-01
**METTL5**	0.12	5.93E-01	1.00E+00	-0.55	9.93E-02	6.46E-01
**APC2**	-0.61	2.14E-01	1.00E+00	1.71	9.93E-02	6.46E-01
**SKI**	-0.21	4.50E-01	1.00E+00	1.39	9.93E-02	6.47E-01
**FRMD4A**	-0.02	9.35E-01	1.00E+00	0.57	9.95E-02	6.47E-01
**TSTD1**	0.32	2.06E-01	1.00E+00	-0.86	9.95E-02	6.47E-01
**PPIAP31**	0.04	9.76E-01	1.00E+00	-0.83	9.95E-02	6.47E-01
**AC012645.1**	-0.67	2.15E-01	1.00E+00	-0.62	9.95E-02	6.47E-01
**MPP6**	-0.60	6.97E-03	5.09E-01	-0.63	9.95E-02	6.47E-01
**AL121603.2**	-1.27	2.21E-02	8.96E-01	-1.06	9.95E-02	6.47E-01
**ADCK5**	-0.28	2.92E-01	1.00E+00	0.46	9.96E-02	6.47E-01
**SCPEP1**	0.37	5.68E-02	1.00E+00	0.31	9.97E-02	6.47E-01
**KCNH1**	-2.74	1.21E-01	1.00E+00	1.74	9.97E-02	6.47E-01
**OBSCN**	0.25	6.21E-01	1.00E+00	1.22	9.97E-02	6.47E-01
**AHCY**	-0.22	2.65E-01	1.00E+00	0.40	9.97E-02	6.47E-01

**IGLV2-18**	1.39	6.86E-01	1.00E+00	4.26	9.98E-02	6.47E-01
**RNF121**	0.00	9.83E-01	1.00E+00	0.42	1.00E-01	6.48E-01
**SETMAR**	-0.11	5.59E-01	1.00E+00	-0.34	1.00E-01	6.48E-01
**PPP1R1A**	2.86	4.20E-02	1.00E+00	-1.60	1.00E-01	6.48E-01
**TC2N**	0.18	4.67E-01	1.00E+00	-0.38	1.00E-01	6.48E-01
**AL592293.1**	-0.17	8.62E-01	1.00E+00	-1.07	1.00E-01	6.48E-01
**CDKN2A**	-0.38	4.82E-01	1.00E+00	1.05	1.00E-01	6.48E-01
**ATP2C1**	0.18	3.46E-01	1.00E+00	0.24	1.00E-01	6.48E-01
**ZNF876P**	-0.69	1.72E-01	1.00E+00	-0.91	1.00E-01	6.48E-01
**LINC00665**	0.00	9.91E-01	1.00E+00	-0.59	1.00E-01	6.48E-01
**ACAP2**	-0.04	8.72E-01	1.00E+00	-0.26	1.00E-01	6.48E-01
**PCSK1**	0.37	7.43E-01	1.00E+00	1.68	1.00E-01	6.49E-01
**XLOC_014288**	-0.11	9.39E-01	1.00E+00	1.56	1.00E-01	6.49E-01
**NENF**	-0.11	6.14E-01	1.00E+00	-1.13	1.00E-01	6.49E-01
**GAB2**	-0.10	6.26E-01	1.00E+00	0.87	1.00E-01	6.49E-01
**AP002505.2**	-2.47	3.47E-01	1.00E+00	2.59	1.00E-01	6.49E-01
**PGPEP1**	0.47	3.72E-01	1.00E+00	0.67	1.01E-01	6.49E-01
**CTDSP1**	0.00	9.90E-01	1.00E+00	0.80	1.01E-01	6.49E-01
**KCNG2**	-0.59	4.08E-01	1.00E+00	1.68	1.01E-01	6.49E-01
**RPS9**	-0.23	3.85E-01	1.00E+00	0.55	1.01E-01	6.49E-01
**PPIAP77**	-0.71	8.37E-01	1.00E+00	-2.82	1.01E-01	6.49E-01
**LENG9**	0.20	5.26E-01	1.00E+00	1.82	1.01E-01	6.49E-01
**XLOC_009279**	-0.35	7.62E-01	1.00E+00	1.29	1.01E-01	6.49E-01
**AP000525.9**	0.11	9.38E-01	1.00E+00	1.36	1.01E-01	6.49E-01
**DCN**	0.86	4.68E-02	1.00E+00	0.73	1.01E-01	6.49E-01
**AGAP6**	-0.35	6.87E-01	1.00E+00	1.17	1.01E-01	6.49E-01
**LRRC28**	-0.02	9.20E-01	1.00E+00	-0.37	1.01E-01	6.49E-01
**ENO2**	-0.27	6.29E-01	1.00E+00	0.71	1.01E-01	6.49E-01
**RPE**	0.17	5.25E-01	1.00E+00	-0.32	1.01E-01	6.49E-01
**CORO1B**	-0.03	8.92E-01	1.00E+00	0.52	1.01E-01	6.49E-01
**KLF5**	-0.19	6.37E-01	1.00E+00	-0.87	1.01E-01	6.49E-01
**SZT2**	0.06	8.23E-01	1.00E+00	0.73	1.01E-01	6.49E-01

**C20orf197**	-0.64	3.91E-01	1.00E+00	-1.60	1.01E-01	6.49E-01
**C4orf50**	-1.23	5.62E-01	1.00E+00	2.46	1.01E-01	6.49E-01
**Z92544.2**	0.16	7.16E-01	1.00E+00	1.57	1.01E-01	6.49E-01
**EEF1A1P19**	0.22	7.09E-01	1.00E+00	-0.67	1.01E-01	6.49E-01
**PLSCR1**	0.47	3.75E-01	1.00E+00	0.74	1.01E-01	6.49E-01
**GRAP2**	0.49	4.26E-01	1.00E+00	1.17	1.01E-01	6.49E-01
**EDIL3**	0.03	9.12E-01	1.00E+00	-0.65	1.01E-01	6.49E-01
**BMP2**	-0.40	3.11E-01	1.00E+00	-1.04	1.01E-01	6.49E-01
**SPATA7**	-0.11	6.77E-01	1.00E+00	-0.42	1.01E-01	6.49E-01
**LINC02285**	-0.85	8.07E-01	1.00E+00	1.95	1.01E-01	6.49E-01
**PPA1**	-0.02	9.30E-01	1.00E+00	-0.28	1.01E-01	6.49E-01
**XLOC_002552**	2.29	2.52E-01	1.00E+00	-1.60	1.01E-01	6.49E-01
**ORC6**	0.37	3.33E-01	1.00E+00	0.80	1.01E-01	6.49E-01
**ELF1**	-0.11	6.53E-01	1.00E+00	0.29	1.01E-01	6.49E-01
**PCDHGB5**	-0.24	2.91E-01	1.00E+00	-0.98	1.01E-01	6.49E-01
**AC099521.2**	0.05	9.64E-01	1.00E+00	1.83	1.01E-01	6.49E-01
**PCCB**	-0.16	4.58E-01	1.00E+00	-0.33	1.01E-01	6.49E-01
**DAPK1**	0.18	6.71E-01	1.00E+00	0.94	1.01E-01	6.49E-01
**ABRAXAS1**	0.20	4.34E-01	1.00E+00	0.36	1.01E-01	6.49E-01
**GHRL**	0.51	5.49E-01	1.00E+00	1.30	1.01E-01	6.49E-01
**AC114811.2**	-1.44	1.86E-01	1.00E+00	1.23	1.01E-01	6.49E-01
**DDX58**	0.23	7.39E-01	1.00E+00	0.88	1.01E-01	6.49E-01
**IFT122**	-0.16	4.04E-01	1.00E+00	-0.43	1.02E-01	6.50E-01
**L3MBTL3**	-0.28	2.72E-01	1.00E+00	0.70	1.02E-01	6.50E-01
**ATN1**	-0.35	1.43E-01	1.00E+00	0.93	1.02E-01	6.50E-01
**AC002543.1**	1.19	5.48E-01	1.00E+00	-1.79	1.02E-01	6.50E-01
**FFAR2**	-0.50	3.94E-01	1.00E+00	1.07	1.02E-01	6.50E-01
**ATRAID**	-0.01	9.55E-01	1.00E+00	-0.70	1.02E-01	6.51E-01
**RMND5B**	0.20	3.63E-01	1.00E+00	-0.31	1.02E-01	6.51E-01
**PALMD**	0.43	2.22E-01	1.00E+00	-0.45	1.02E-01	6.51E-01
**LAMA3**	0.17	3.66E-01	1.00E+00	0.65	1.02E-01	6.51E-01
**SIN3B**	-0.26	3.17E-01	1.00E+00	0.89	1.02E-01	6.51E-01

**ELF4**	-0.18	3.89E-01	1.00E+00	0.93	1.02E-01	6.51E-01
**SULF2**	-0.10	6.42E-01	1.00E+00	0.87	1.02E-01	6.51E-01
**TDRD9**	-0.10	9.15E-01	1.00E+00	2.11	1.02E-01	6.51E-01
**TSPAN18**	0.09	8.47E-01	1.00E+00	0.83	1.02E-01	6.51E-01
**LPAR4**	0.69	3.92E-01	1.00E+00	1.32	1.02E-01	6.51E-01
**AC024267.3**	-1.00	6.44E-01	1.00E+00	2.75	1.02E-01	6.51E-01
**AL450124.1**	-2.64	6.35E-03	4.78E-01	-0.91	1.02E-01	6.51E-01
**FAM8A1**	0.08	7.26E-01	1.00E+00	-0.54	1.02E-01	6.51E-01
**EPHA8**	-0.94	5.71E-01	1.00E+00	3.67	1.02E-01	6.51E-01
**ARHGAP33**	-1.09	1.47E-03	2.09E-01	1.74	1.02E-01	6.51E-01
**TEPSIN**	-0.31	4.81E-01	1.00E+00	0.60	1.02E-01	6.51E-01
**RBBP4P2**	NA	NA	NA	1.23	1.02E-01	6.51E-01
**XLOC_003595**	-0.39	8.67E-01	1.00E+00	2.52	1.02E-01	6.51E-01
**C12orf75**	-0.26	4.14E-01	1.00E+00	-0.64	1.03E-01	6.52E-01
**AL161421.1**	-0.74	8.09E-02	1.00E+00	-1.19	1.03E-01	6.52E-01
**SLC24A4**	-0.34	7.52E-01	1.00E+00	1.80	1.03E-01	6.52E-01
**TWIST2**	0.04	9.15E-01	1.00E+00	1.12	1.03E-01	6.52E-01
**KRBA1**	-0.21	5.09E-01	1.00E+00	1.24	1.03E-01	6.52E-01
**CHADL**	-0.61	3.46E-02	1.00E+00	-0.84	1.03E-01	6.52E-01
**RHNO1**	-0.16	4.12E-01	1.00E+00	-0.45	1.03E-01	6.52E-01
**ABCC6P2**	2.50	2.44E-01	1.00E+00	-1.63	1.03E-01	6.52E-01
**LINC00856**	-3.09	2.71E-01	1.00E+00	1.57	1.03E-01	6.52E-01
**BOK-AS1**	NA	NA	NA	-2.88	1.03E-01	6.52E-01
**WDFY4**	-0.35	5.20E-01	1.00E+00	1.60	1.03E-01	6.52E-01
**SLC16A10**	-0.12	8.38E-01	1.00E+00	-0.97	1.03E-01	6.52E-01
**RNF114**	0.06	7.27E-01	1.00E+00	0.38	1.03E-01	6.52E-01
**G8415**	-2.03	2.27E-01	1.00E+00	2.24	1.03E-01	6.52E-01
**AP3M2**	0.21	4.39E-01	1.00E+00	-0.36	1.03E-01	6.52E-01
**NADK2**	0.01	9.67E-01	1.00E+00	-0.45	1.03E-01	6.52E-01
**LTB**	-1.13	5.78E-02	1.00E+00	1.25	1.03E-01	6.52E-01
**CXCL16**	-0.23	4.95E-01	1.00E+00	0.64	1.03E-01	6.52E-01
**ERICH2**	0.20	8.52E-01	1.00E+00	1.21	1.03E-01	6.52E-01

**AL450405.1**	1.08	6.73E-02	1.00E+00	0.92	1.03E-01	6.52E-01
**KCNJ15**	-0.88	2.60E-02	9.40E-01	0.99	1.03E-01	6.52E-01
**PRDM6**	0.97	1.25E-01	1.00E+00	1.07	1.03E-01	6.52E-01
**AL512329.2**	-1.65	1.87E-02	8.59E-01	-1.19	1.03E-01	6.52E-01
**GMFG**	0.01	9.75E-01	1.00E+00	0.69	1.03E-01	6.52E-01
**LINC00992**	-0.15	7.02E-01	1.00E+00	-1.07	1.03E-01	6.52E-01
**INTS4P2**	0.00	9.98E-01	1.00E+00	-0.91	1.03E-01	6.52E-01
**AC138207.6**	-1.78	3.90E-01	1.00E+00	1.44	1.03E-01	6.52E-01
**TMED2**	0.20	2.50E-01	1.00E+00	-0.56	1.03E-01	6.52E-01
**ACTN4**	-0.30	1.20E-01	1.00E+00	0.81	1.03E-01	6.52E-01
**ZNF275**	0.08	8.41E-01	1.00E+00	0.81	1.03E-01	6.52E-01
**TEKT2**	0.01	9.87E-01	1.00E+00	1.82	1.03E-01	6.52E-01
**PCED1A**	0.03	8.97E-01	1.00E+00	0.50	1.03E-01	6.52E-01
**NCSTN**	0.07	7.29E-01	1.00E+00	0.28	1.03E-01	6.52E-01
**SFTPA1**	NA	NA	NA	3.13	1.04E-01	6.52E-01
**AL161449.2**	0.01	9.95E-01	1.00E+00	1.62	1.04E-01	6.52E-01
**NIPSNAP2**	0.09	7.55E-01	1.00E+00	-0.53	1.04E-01	6.52E-01
**ZNF628**	-1.01	1.37E-02	7.37E-01	1.39	1.04E-01	6.52E-01
**ODF2L**	-0.22	3.91E-01	1.00E+00	0.51	1.04E-01	6.52E-01
**DOCK10**	-0.33	3.00E-01	1.00E+00	0.89	1.04E-01	6.52E-01
**XLOC_012700**	-0.39	6.47E-01	1.00E+00	-1.55	1.04E-01	6.52E-01
**C1orf127**	-1.29	4.62E-01	1.00E+00	2.28	1.04E-01	6.52E-01
**ENTPD7**	0.12	6.04E-01	1.00E+00	0.79	1.04E-01	6.52E-01
**UPRT**	0.35	2.45E-01	1.00E+00	-0.42	1.04E-01	6.52E-01
**AC097376.2**	-0.28	2.81E-01	1.00E+00	-0.53	1.04E-01	6.52E-01
**ATG2A**	-0.11	7.26E-01	1.00E+00	0.91	1.04E-01	6.52E-01
**EP400**	-0.54	2.98E-02	9.97E-01	0.82	1.04E-01	6.52E-01
**HLA-DPA1**	-0.57	7.17E-02	1.00E+00	0.76	1.04E-01	6.52E-01
**XLOC_009207**	-2.24	1.16E-01	1.00E+00	-1.85	1.04E-01	6.52E-01
**TTC41P**	1.06	4.41E-01	1.00E+00	1.71	1.04E-01	6.52E-01
**ULK1**	-0.19	5.04E-01	1.00E+00	0.80	1.04E-01	6.52E-01
**DNAJB5**	-0.51	2.79E-01	1.00E+00	0.92	1.04E-01	6.52E-01

**LINGO3**	-0.98	1.69E-01	1.00E+00	2.26	1.04E-01	6.52E-01
**CDC45**	-0.24	5.44E-01	1.00E+00	0.97	1.04E-01	6.52E-01
**XLOC_011518**	-1.67	4.39E-02	1.00E+00	-1.22	1.04E-01	6.52E-01
**MT-ATP6**	0.07	8.80E-01	1.00E+00	-0.55	1.04E-01	6.52E-01
**FAM157C**	2.83	2.99E-01	1.00E+00	1.45	1.04E-01	6.52E-01
**LINC01801**	0.05	9.37E-01	1.00E+00	2.12	1.04E-01	6.52E-01
**RBM17P4**	-0.44	9.00E-01	1.00E+00	-1.68	1.04E-01	6.52E-01
**AC073073.1**	-2.60	2.90E-01	1.00E+00	1.33	1.04E-01	6.52E-01
**AC087491.1**	0.17	8.07E-01	1.00E+00	-1.20	1.04E-01	6.52E-01
**LINC01907**	-2.60	1.95E-02	8.74E-01	1.42	1.04E-01	6.52E-01
**XLOC_001787**	-0.87	7.19E-01	1.00E+00	1.97	1.04E-01	6.52E-01
**RHOXF1**	2.95	2.39E-01	1.00E+00	-1.92	1.04E-01	6.52E-01
**ABCA11P**	0.02	9.82E-01	1.00E+00	-0.75	1.04E-01	6.52E-01
**TRPV3**	0.29	5.20E-01	1.00E+00	0.99	1.04E-01	6.52E-01
**MTCH2**	0.03	8.56E-01	1.00E+00	-0.35	1.04E-01	6.52E-01
**ZNF337-AS1**	0.30	6.70E-01	1.00E+00	0.87	1.04E-01	6.52E-01
**VIP**	-3.47	3.41E-02	1.00E+00	-2.13	1.04E-01	6.52E-01
**AP2A2**	0.05	8.06E-01	1.00E+00	0.68	1.04E-01	6.52E-01
**RAVER2**	-0.37	1.71E-01	1.00E+00	-0.54	1.04E-01	6.52E-01
**COL4A2**	-0.07	9.13E-01	1.00E+00	1.36	1.05E-01	6.53E-01
**BRI3**	0.02	9.45E-01	1.00E+00	0.87	1.05E-01	6.53E-01
**G30965**	0.25	8.32E-01	1.00E+00	1.90	1.05E-01	6.53E-01
**AC006333.2**	0.05	8.62E-01	1.00E+00	-0.52	1.05E-01	6.53E-01
**ZNF528-AS1**	0.20	6.99E-01	1.00E+00	-0.60	1.05E-01	6.53E-01
**CYREN**	0.13	7.05E-01	1.00E+00	0.34	1.05E-01	6.53E-01
**CYBC1**	-0.20	3.45E-01	1.00E+00	0.58	1.05E-01	6.53E-01
**TRIM41**	-0.10	5.71E-01	1.00E+00	0.95	1.05E-01	6.53E-01
**OR5K2**	0.91	7.91E-01	1.00E+00	2.55	1.05E-01	6.54E-01
**TLDC1**	0.24	4.82E-01	1.00E+00	0.53	1.05E-01	6.54E-01
**MTHFR**	0.06	8.61E-01	1.00E+00	0.71	1.05E-01	6.54E-01
**TWF2**	0.08	7.00E-01	1.00E+00	0.37	1.05E-01	6.54E-01
**NSMCE4A**	-0.04	8.72E-01	1.00E+00	-0.49	1.05E-01	6.54E-01

**AC092279.2**	0.18	8.82E-01	1.00E+00	1.44	1.05E-01	6.54E-01
**CADM1**	-0.29	1.14E-01	1.00E+00	-0.49	1.05E-01	6.54E-01
**AARD**	1.84	1.13E-02	6.59E-01	1.19	1.05E-01	6.54E-01
**C1QTNF1-AS1**	-0.23	8.63E-01	1.00E+00	-1.75	1.05E-01	6.54E-01
**AASDH**	0.20	4.18E-01	1.00E+00	-0.39	1.05E-01	6.54E-01
**TOX3**	0.11	8.92E-01	1.00E+00	-1.29	1.05E-01	6.54E-01
**LSAMP**	-0.18	5.99E-01	1.00E+00	0.74	1.05E-01	6.54E-01
**ZNF232**	-0.02	9.46E-01	1.00E+00	-0.36	1.05E-01	6.55E-01
**AL022068.1**	-0.03	9.50E-01	1.00E+00	-1.09	1.05E-01	6.55E-01
**ADAMTS10**	0.45	3.39E-01	1.00E+00	1.68	1.05E-01	6.55E-01
**NFASC**	-0.22	4.45E-01	1.00E+00	0.65	1.06E-01	6.55E-01
**CBX1**	0.03	8.85E-01	1.00E+00	0.43	1.06E-01	6.55E-01
**KIRREL1**	0.48	3.03E-01	1.00E+00	1.36	1.06E-01	6.55E-01
**PARP11**	0.31	3.64E-01	1.00E+00	0.51	1.06E-01	6.55E-01
**TRIM46**	-1.02	2.61E-01	1.00E+00	1.79	1.06E-01	6.55E-01
**G25248**	-2.13	3.83E-01	1.00E+00	1.17	1.06E-01	6.55E-01
**AL157832.1**	1.18	3.85E-01	1.00E+00	1.82	1.06E-01	6.55E-01
**CEP170**	-0.18	5.17E-01	1.00E+00	0.53	1.06E-01	6.55E-01
**G31717**	-1.29	7.00E-02	1.00E+00	-1.70	1.06E-01	6.55E-01
**ELOVL3**	0.85	4.55E-01	1.00E+00	-1.29	1.06E-01	6.55E-01
**LINC00426**	-1.13	2.66E-01	1.00E+00	1.36	1.06E-01	6.55E-01
**AL160171.1**	-1.33	6.24E-01	1.00E+00	1.35	1.06E-01	6.55E-01
**PLOD1**	-0.03	9.25E-01	1.00E+00	0.66	1.06E-01	6.55E-01
**PTGS2**	-1.72	2.68E-02	9.55E-01	1.08	1.06E-01	6.55E-01
**LINC02137**	-0.55	8.12E-01	1.00E+00	1.22	1.06E-01	6.55E-01
**AC138932.3**	-0.41	7.25E-01	1.00E+00	-1.11	1.06E-01	6.55E-01
**ORC4**	0.03	8.86E-01	1.00E+00	-0.40	1.06E-01	6.55E-01
**TMEM97**	-0.28	4.60E-01	1.00E+00	-0.73	1.06E-01	6.55E-01
**SLC5A4**	0.36	7.65E-01	1.00E+00	2.18	1.06E-01	6.55E-01
**PFDN4**	0.10	6.94E-01	1.00E+00	-0.67	1.06E-01	6.55E-01
**NDUFAF4**	0.39	2.04E-01	1.00E+00	-0.53	1.06E-01	6.55E-01
**PLGRKT**	-0.41	2.07E-02	8.94E-01	-0.55	1.06E-01	6.55E-01

**RNF146**	0.32	7.67E-02	1.00E+00	-0.40	1.06E-01	6.55E-01
**LRRC8C-DT**	-0.27	5.55E-01	1.00E+00	-0.63	1.06E-01	6.55E-01
**CEL**	-2.22	1.55E-02	7.75E-01	-1.87	1.06E-01	6.55E-01
**SLC5A12**	-0.93	6.40E-01	1.00E+00	-2.72	1.06E-01	6.55E-01
**CNGA4**	0.51	7.12E-01	1.00E+00	3.95	1.06E-01	6.55E-01
**CMAHP**	0.62	1.38E-01	1.00E+00	0.49	1.06E-01	6.55E-01
**DOCK2**	-0.25	5.24E-01	1.00E+00	1.38	1.06E-01	6.55E-01
**AL669831.1**	-0.71	3.05E-01	1.00E+00	1.10	1.06E-01	6.55E-01
**SUMF2**	-0.02	8.97E-01	1.00E+00	0.36	1.06E-01	6.55E-01
**OTUD6B-AS1**	-0.17	5.75E-01	1.00E+00	-0.69	1.06E-01	6.55E-01
**KCNMB2**	-0.48	8.40E-01	1.00E+00	-2.60	1.06E-01	6.55E-01
**FAAP24**	0.41	2.41E-01	1.00E+00	0.43	1.06E-01	6.55E-01
**ARR3**	-1.51	1.10E-01	1.00E+00	-1.29	1.06E-01	6.55E-01
**KAZALD1**	0.06	9.01E-01	1.00E+00	0.81	1.06E-01	6.55E-01
**SPN**	-0.98	6.72E-02	1.00E+00	1.01	1.06E-01	6.55E-01
**SNORA66**	0.43	7.59E-01	1.00E+00	1.12	1.06E-01	6.55E-01
**LINC02391**	-1.67	2.55E-01	1.00E+00	-1.11	1.07E-01	6.55E-01
**RARRES3**	-0.29	5.68E-01	1.00E+00	0.77	1.07E-01	6.56E-01
**GBP1P1**	-0.49	4.80E-01	1.00E+00	1.22	1.07E-01	6.56E-01
**TMEM39B**	-0.28	1.68E-01	1.00E+00	0.69	1.07E-01	6.56E-01
**PARP8**	-0.20	4.26E-01	1.00E+00	0.68	1.07E-01	6.56E-01
**MTFP1**	0.13	9.70E-01	1.00E+00	1.04	1.07E-01	6.56E-01
**FAM241B**	-0.20	5.70E-01	1.00E+00	-0.59	1.07E-01	6.56E-01
**LMAN1**	0.15	5.36E-01	1.00E+00	0.26	1.07E-01	6.56E-01
**SRRD**	-0.04	8.26E-01	1.00E+00	-0.88	1.07E-01	6.56E-01
**LINC01979**	-0.17	9.41E-01	1.00E+00	2.08	1.07E-01	6.56E-01
**XLOC_000587**	1.22	7.25E-01	1.00E+00	1.74	1.07E-01	6.56E-01
**RPL22P3**	2.82	2.01E-01	1.00E+00	1.51	1.07E-01	6.56E-01
**GIMAP6**	0.42	4.58E-01	1.00E+00	0.81	1.07E-01	6.56E-01
**GLIPR1L2**	-0.57	4.39E-01	1.00E+00	-0.94	1.07E-01	6.57E-01
**NCKAP5L**	-0.16	6.32E-01	1.00E+00	1.20	1.07E-01	6.57E-01
**CFHR1**	-0.18	9.44E-01	1.00E+00	1.79	1.07E-01	6.57E-01

**RPA4**	-1.46	6.70E-01	1.00E+00	-1.60	1.07E-01	6.57E-01
**HSPA12A**	0.24	5.61E-01	1.00E+00	-0.73	1.07E-01	6.57E-01
**SNAPC3**	-0.02	9.19E-01	1.00E+00	-0.38	1.07E-01	6.57E-01
**ZNF496**	-0.22	3.49E-01	1.00E+00	0.89	1.07E-01	6.57E-01
**SLC6A6**	-0.32	2.45E-01	1.00E+00	0.79	1.07E-01	6.57E-01
**WDR62**	-0.16	7.08E-01	1.00E+00	0.88	1.07E-01	6.57E-01
**RAET1G**	-0.16	7.89E-01	1.00E+00	0.69	1.07E-01	6.57E-01
**ACOXL**	-2.22	6.55E-02	1.00E+00	1.95	1.07E-01	6.57E-01
**MMP23A**	-0.55	6.84E-01	1.00E+00	1.65	1.08E-01	6.57E-01
**ENTPD3**	-0.01	9.88E-01	1.00E+00	-0.70	1.08E-01	6.57E-01
**NDUFB3**	0.07	6.67E-01	1.00E+00	-0.56	1.08E-01	6.57E-01
**CRTC3-AS1**	-0.40	7.26E-01	1.00E+00	1.23	1.08E-01	6.57E-01
**G13883**	-2.05	5.50E-01	1.00E+00	2.32	1.08E-01	6.57E-01
**DOCK8**	-0.21	4.73E-01	1.00E+00	0.87	1.08E-01	6.57E-01
**XLOC_004879**	0.54	7.25E-01	1.00E+00	-2.37	1.08E-01	6.57E-01
**CCNT2-AS1**	-2.78	2.94E-02	9.91E-01	0.84	1.08E-01	6.57E-01
**SS18L1**	-0.06	8.46E-01	1.00E+00	0.51	1.08E-01	6.57E-01
**GPR65**	0.37	6.80E-01	1.00E+00	1.02	1.08E-01	6.57E-01
**DYRK3**	-0.58	9.37E-02	1.00E+00	0.86	1.08E-01	6.57E-01
**AC018797.2**	0.21	6.68E-01	1.00E+00	-0.70	1.08E-01	6.57E-01
**CIITA**	-0.73	9.63E-03	6.08E-01	1.35	1.08E-01	6.57E-01
**RMDN1**	-0.04	8.21E-01	1.00E+00	-0.40	1.08E-01	6.57E-01
**AC003986.3**	1.86	1.66E-01	1.00E+00	-1.35	1.08E-01	6.57E-01
**PP2D1**	-0.19	7.86E-01	1.00E+00	-0.88	1.08E-01	6.57E-01
**LINC01374**	1.32	7.00E-01	1.00E+00	2.27	1.08E-01	6.57E-01
**RANBP3**	-0.16	4.33E-01	1.00E+00	0.71	1.08E-01	6.57E-01
**CD38**	0.02	9.88E-01	1.00E+00	1.83	1.08E-01	6.57E-01
**GFOD2**	0.16	4.81E-01	1.00E+00	0.42	1.08E-01	6.57E-01
**ZNF827**	-0.20	5.53E-01	1.00E+00	0.62	1.08E-01	6.57E-01
**XLOC_004196**	0.63	7.58E-01	1.00E+00	-2.45	1.08E-01	6.57E-01
**GNPDA1**	0.02	9.38E-01	1.00E+00	0.25	1.08E-01	6.57E-01
**PPP2R5E**	-0.10	6.11E-01	1.00E+00	-0.46	1.08E-01	6.57E-01

**DDX60**	0.23	7.17E-01	1.00E+00	0.76	1.08E-01	6.57E-01
**PPFIA4**	-0.60	3.88E-01	1.00E+00	1.96	1.08E-01	6.57E-01
**CRYBB1**	-1.65	9.58E-02	1.00E+00	1.28	1.08E-01	6.57E-01
**ATF6B**	-0.29	3.45E-01	1.00E+00	0.86	1.08E-01	6.57E-01
**HSPB11**	0.08	7.20E-01	1.00E+00	-0.61	1.08E-01	6.57E-01
**PPAN**	-0.14	7.16E-01	1.00E+00	-0.85	1.08E-01	6.57E-01
**ERCC2**	-0.23	3.96E-01	1.00E+00	0.50	1.08E-01	6.57E-01
**CCNDBP1**	0.20	3.38E-01	1.00E+00	-0.51	1.08E-01	6.58E-01
**SCARA5**	1.29	3.00E-02	9.97E-01	0.95	1.08E-01	6.58E-01
**AC026367.2**	0.30	8.95E-01	1.00E+00	-2.37	1.08E-01	6.58E-01
**CISD1**	-0.01	9.50E-01	1.00E+00	-0.51	1.08E-01	6.58E-01
**HSD11B1**	0.18	7.48E-01	1.00E+00	-0.66	1.09E-01	6.58E-01
**HOXC6**	0.02	9.66E-01	1.00E+00	-1.07	1.09E-01	6.58E-01
**CALCOCO2**	0.12	4.93E-01	1.00E+00	-0.20	1.09E-01	6.58E-01
**GTPBP4**	-0.05	8.19E-01	1.00E+00	0.28	1.09E-01	6.58E-01
**RNVU1-18**	-1.61	4.07E-01	1.00E+00	-1.42	1.09E-01	6.58E-01
**AL138762.1**	-2.46	1.61E-01	1.00E+00	-1.27	1.09E-01	6.58E-01
**TNRC6A**	-0.03	9.20E-01	1.00E+00	0.57	1.09E-01	6.58E-01
**TMEM92**	0.16	8.76E-01	1.00E+00	1.49	1.09E-01	6.58E-01
**DENND4B**	-0.07	8.30E-01	1.00E+00	0.97	1.09E-01	6.58E-01
**PCNX2**	-0.11	8.11E-01	1.00E+00	0.88	1.09E-01	6.58E-01
**SIGLEC7**	-2.45	1.21E-01	1.00E+00	1.97	1.09E-01	6.58E-01
**MAPK11**	-0.19	6.08E-01	1.00E+00	1.27	1.09E-01	6.58E-01
**RGL4**	0.06	9.51E-01	1.00E+00	1.95	1.09E-01	6.58E-01
**TMEM220-AS1**	-0.05	9.61E-01	1.00E+00	-1.03	1.09E-01	6.58E-01
**EIF1B**	0.09	6.64E-01	1.00E+00	-0.34	1.09E-01	6.58E-01
**SAT1**	0.11	7.10E-01	1.00E+00	0.51	1.09E-01	6.58E-01
**CH17-340M24.3**	-0.09	7.78E-01	1.00E+00	-0.76	1.09E-01	6.58E-01
**EEF1A1P12**	0.68	5.35E-01	1.00E+00	-0.78	1.09E-01	6.58E-01
**TTC13**	-0.38	1.32E-01	1.00E+00	-0.56	1.09E-01	6.58E-01
**SIPA1**	-0.18	3.78E-01	1.00E+00	0.68	1.09E-01	6.58E-01
**AC007998.3**	-0.08	9.25E-01	1.00E+00	1.04	1.09E-01	6.58E-01

**G354**	-3.23	9.27E-03	6.04E-01	1.43	1.09E-01	6.58E-01
**RAB1B**	-0.08	6.83E-01	1.00E+00	0.71	1.09E-01	6.58E-01
**XLOC_013474**	-0.89	2.96E-01	1.00E+00	-1.47	1.09E-01	6.58E-01
**AL137060.1**	-0.66	6.63E-01	1.00E+00	2.09	1.09E-01	6.58E-01
**MISP**	-0.64	2.63E-01	1.00E+00	1.61	1.09E-01	6.58E-01
**CCSAP**	0.29	4.79E-01	1.00E+00	-0.58	1.09E-01	6.58E-01
**RBP5**	-0.75	2.76E-01	1.00E+00	0.96	1.09E-01	6.58E-01
**IKBKGP1**	0.00	9.97E-01	1.00E+00	-0.54	1.09E-01	6.58E-01
**NR2F2**	0.09	8.15E-01	1.00E+00	0.85	1.09E-01	6.58E-01
**CHD1**	-0.27	2.89E-01	1.00E+00	0.26	1.09E-01	6.58E-01
**CHURC1**	0.38	8.51E-02	1.00E+00	0.29	1.09E-01	6.58E-01
**CDK19**	0.32	2.20E-01	1.00E+00	0.49	1.09E-01	6.58E-01
**GMFB**	0.31	2.99E-01	1.00E+00	-0.47	1.10E-01	6.58E-01
**AP001107.3**	-0.79	6.00E-01	1.00E+00	2.25	1.10E-01	6.58E-01
**RFPL1S**	-1.88	3.00E-01	1.00E+00	1.88	1.10E-01	6.58E-01
**MRPS15**	-0.08	6.71E-01	1.00E+00	-0.33	1.10E-01	6.59E-01
**CDH4**	0.19	7.09E-01	1.00E+00	-1.28	1.10E-01	6.59E-01
**AC108134.1**	0.38	6.86E-01	1.00E+00	1.70	1.10E-01	6.60E-01
**AC006033.2**	1.58	3.88E-01	1.00E+00	2.12	1.10E-01	6.60E-01
**BBS7**	0.16	6.53E-01	1.00E+00	-0.33	1.10E-01	6.60E-01
**AP003071.4**	-0.36	6.10E-01	1.00E+00	1.36	1.10E-01	6.60E-01
**GHITM**	0.06	7.26E-01	1.00E+00	-0.47	1.10E-01	6.60E-01
**HOXA10**	0.77	8.77E-02	1.00E+00	-0.71	1.10E-01	6.60E-01
**TBC1D9**	0.46	3.76E-01	1.00E+00	0.91	1.10E-01	6.60E-01
**AC091182.2**	0.69	5.74E-01	1.00E+00	1.24	1.10E-01	6.60E-01
**AC116158.1**	1.97	4.77E-01	1.00E+00	-1.78	1.10E-01	6.60E-01
**XK**	-0.50	2.13E-01	1.00E+00	-0.89	1.10E-01	6.60E-01
**UBA52**	-0.10	6.10E-01	1.00E+00	0.36	1.10E-01	6.60E-01
**AC004825.2**	-0.61	5.65E-01	1.00E+00	-1.45	1.10E-01	6.60E-01
**NUP88**	-0.01	9.69E-01	1.00E+00	-0.33	1.10E-01	6.60E-01
**MUSK**	1.27	1.50E-01	1.00E+00	1.13	1.10E-01	6.60E-01
**LINC02562**	-0.29	5.59E-01	1.00E+00	-1.13	1.10E-01	6.60E-01

**SERPINE1**	-1.05	5.48E-02	1.00E+00	1.36	1.10E-01	6.60E-01
**LINC02158**	-1.22	6.18E-01	1.00E+00	-1.55	1.10E-01	6.60E-01
**FUT9**	-0.62	6.16E-01	1.00E+00	-1.80	1.11E-01	6.60E-01
**IER5L**	-0.20	5.04E-01	1.00E+00	1.48	1.11E-01	6.60E-01
**IGFL2-AS1**	-0.87	2.00E-01	1.00E+00	-1.10	1.11E-01	6.60E-01
**POLR2M**	0.58	7.23E-02	1.00E+00	-0.35	1.11E-01	6.60E-01
**NUP54**	0.10	5.77E-01	1.00E+00	-0.32	1.11E-01	6.60E-01
**OSBPL9**	0.10	7.11E-01	1.00E+00	-0.35	1.11E-01	6.60E-01
**AC084816.1**	0.25	9.42E-01	1.00E+00	-2.55	1.11E-01	6.60E-01
**AC004471.2**	-0.58	6.72E-01	1.00E+00	1.39	1.11E-01	6.60E-01
**CD8A**	-0.88	1.29E-01	1.00E+00	1.36	1.11E-01	6.60E-01
**FUT11**	0.36	2.98E-01	1.00E+00	0.53	1.11E-01	6.60E-01
**GIN1**	0.05	8.81E-01	1.00E+00	-0.49	1.11E-01	6.60E-01
**RAB31**	-0.07	8.34E-01	1.00E+00	0.71	1.11E-01	6.60E-01
**FMR1**	0.15	5.45E-01	1.00E+00	-0.41	1.11E-01	6.60E-01
**CYTH1**	0.04	8.40E-01	1.00E+00	0.52	1.11E-01	6.60E-01
**ST3GAL6**	0.29	4.46E-01	1.00E+00	-0.43	1.11E-01	6.60E-01
**APLP2**	0.10	7.15E-01	1.00E+00	0.40	1.11E-01	6.61E-01
**AL591069.1**	0.10	9.61E-01	1.00E+00	-1.70	1.11E-01	6.61E-01
**GLULP4**	1.13	7.44E-01	1.00E+00	1.82	1.11E-01	6.61E-01
**XLOC_009602**	-0.25	9.42E-01	1.00E+00	2.17	1.11E-01	6.61E-01
**AC131235.3**	0.94	7.50E-01	1.00E+00	-1.81	1.11E-01	6.61E-01
**RDH16**	0.41	5.38E-01	1.00E+00	1.03	1.11E-01	6.61E-01
**JADE2**	-0.03	9.12E-01	1.00E+00	0.91	1.11E-01	6.61E-01
**KCNT1**	0.14	9.08E-01	1.00E+00	1.92	1.11E-01	6.61E-01
**NOP2**	-2.71	3.01E-01	1.00E+00	0.81	1.11E-01	6.61E-01
**AL357055.3**	-0.01	9.93E-01	1.00E+00	-1.25	1.11E-01	6.61E-01
**CHST12**	-0.02	9.26E-01	1.00E+00	0.36	1.11E-01	6.61E-01
**IGLV2-14**	0.33	8.15E-01	1.00E+00	2.05	1.11E-01	6.61E-01
**HS3ST3B1**	0.01	9.86E-01	1.00E+00	0.63	1.11E-01	6.61E-01
**FFAR3**	2.56	3.14E-01	1.00E+00	1.83	1.11E-01	6.61E-01
**ATP12A**	-0.33	7.16E-01	1.00E+00	1.76	1.11E-01	6.61E-01

**ZBTB20-AS5**	-1.74	6.11E-01	1.00E+00	-2.70	1.11E-01	6.61E-01
**MGAT4EP**	-1.97	4.73E-01	1.00E+00	3.56	1.11E-01	6.61E-01
**TUBE1**	0.31	1.90E-01	1.00E+00	-0.54	1.11E-01	6.61E-01
**SNRPF**	-0.05	7.79E-01	1.00E+00	-0.49	1.11E-01	6.61E-01
**MFSD6**	0.00	9.88E-01	1.00E+00	-0.64	1.11E-01	6.61E-01
**ALG8**	0.11	6.49E-01	1.00E+00	-0.51	1.12E-01	6.61E-01
**RBM43**	0.00	9.96E-01	1.00E+00	0.50	1.12E-01	6.61E-01
**G33515**	0.12	9.45E-01	1.00E+00	-1.28	1.12E-01	6.61E-01
**RPS16**	-0.01	9.80E-01	1.00E+00	0.49	1.12E-01	6.61E-01
**GEM**	0.25	4.67E-01	1.00E+00	0.66	1.12E-01	6.61E-01
**AC079684.1**	1.50	4.44E-01	1.00E+00	2.46	1.12E-01	6.61E-01
**PRKAR1B**	-0.50	9.79E-02	1.00E+00	0.40	1.12E-01	6.61E-01
**ABCD3**	0.40	1.14E-01	1.00E+00	-0.43	1.12E-01	6.61E-01
**AC009948.1**	0.68	5.69E-02	1.00E+00	0.49	1.12E-01	6.61E-01
**USP12**	0.22	2.75E-01	1.00E+00	-0.56	1.12E-01	6.61E-01
**KDM2A**	-0.10	6.67E-01	1.00E+00	0.70	1.12E-01	6.61E-01
**AC018638.5**	-1.38	1.26E-01	1.00E+00	1.22	1.12E-01	6.61E-01
**SARDH**	0.10	8.33E-01	1.00E+00	1.28	1.12E-01	6.62E-01
**AL590822.1**	0.70	3.26E-01	1.00E+00	0.89	1.12E-01	6.62E-01
**AL391422.4**	0.22	6.33E-01	1.00E+00	1.00	1.12E-01	6.62E-01
**TRAF3**	-0.06	8.00E-01	1.00E+00	0.95	1.12E-01	6.62E-01
**APOO**	-0.11	7.33E-01	1.00E+00	-0.40	1.12E-01	6.62E-01
**AC079209.1**	-1.96	4.98E-01	1.00E+00	2.06	1.12E-01	6.62E-01
**CAV3**	-1.31	3.57E-01	1.00E+00	1.99	1.12E-01	6.62E-01
**XLOC_000389**	1.93	1.80E-01	1.00E+00	0.77	1.12E-01	6.62E-01
**EBAG9**	0.08	6.13E-01	1.00E+00	-0.43	1.12E-01	6.62E-01
**CCR4**	0.51	5.39E-01	1.00E+00	2.08	1.12E-01	6.62E-01
**GPN2**	-0.16	5.12E-01	1.00E+00	0.29	1.12E-01	6.62E-01
**LINC01578**	-0.08	7.79E-01	1.00E+00	-0.69	1.12E-01	6.62E-01
**SLC39A7**	0.02	9.48E-01	1.00E+00	0.71	1.12E-01	6.62E-01
**TMEM107**	0.00	9.96E-01	1.00E+00	-0.45	1.12E-01	6.62E-01
**TFDP1**	-0.23	3.90E-01	1.00E+00	-0.58	1.12E-01	6.62E-01

**CCR8**	-1.44	2.45E-01	1.00E+00	1.45	1.12E-01	6.62E-01
**AC004477.3**	-3.56	3.75E-03	3.33E-01	-1.23	1.12E-01	6.62E-01
**NUP62**	-0.13	4.77E-01	1.00E+00	0.50	1.13E-01	6.63E-01
**VWC2**	-0.88	1.17E-01	1.00E+00	-0.90	1.13E-01	6.63E-01
**G39927**	-1.43	3.91E-01	1.00E+00	-2.76	1.13E-01	6.63E-01
**AC243772.2**	0.73	3.82E-01	1.00E+00	2.00	1.13E-01	6.63E-01
**ATF2**	0.37	2.59E-01	1.00E+00	-0.47	1.13E-01	6.63E-01
**CBX8**	-0.16	4.73E-01	1.00E+00	0.60	1.13E-01	6.63E-01
**AL391069.3**	NA	NA	NA	-2.07	1.13E-01	6.63E-01
**MEP1B**	-1.78	6.06E-01	1.00E+00	-1.52	1.13E-01	6.63E-01
**AL357033.4**	-0.22	6.52E-01	1.00E+00	1.13	1.13E-01	6.63E-01
**AC080188.1**	-1.28	5.58E-01	1.00E+00	1.86	1.13E-01	6.63E-01
**TMEM200A**	0.17	6.80E-01	1.00E+00	0.87	1.13E-01	6.63E-01
**TNR**	-4.92	8.42E-02	1.00E+00	-0.95	1.13E-01	6.63E-01
**AL590094.1**	0.13	7.70E-01	1.00E+00	-0.95	1.13E-01	6.63E-01
**ZNF880**	0.18	5.65E-01	1.00E+00	-0.56	1.13E-01	6.63E-01
**G37087**	-2.69	1.26E-01	1.00E+00	1.48	1.13E-01	6.63E-01
**ZBTB33**	0.06	8.29E-01	1.00E+00	-0.46	1.13E-01	6.63E-01
**C12orf66**	0.04	8.87E-01	1.00E+00	0.53	1.13E-01	6.63E-01
**RCN2**	0.09	7.04E-01	1.00E+00	-0.58	1.13E-01	6.63E-01
**PRR34**	-0.02	9.70E-01	1.00E+00	1.20	1.13E-01	6.63E-01
**KLHL33**	1.02	1.85E-01	1.00E+00	-2.07	1.13E-01	6.63E-01
**FBP1**	0.26	6.87E-01	1.00E+00	0.99	1.13E-01	6.63E-01
**MIGA1**	0.31	2.62E-01	1.00E+00	-0.36	1.13E-01	6.63E-01
**AL138955.1**	1.37	5.13E-01	1.00E+00	1.99	1.13E-01	6.63E-01
**SELENON**	-0.13	6.63E-01	1.00E+00	0.87	1.13E-01	6.63E-01
**HEY1**	0.51	3.62E-01	1.00E+00	-0.72	1.13E-01	6.63E-01
**ARHGEF17**	-0.08	8.41E-01	1.00E+00	0.92	1.13E-01	6.63E-01
**ILDR1**	-0.10	7.47E-01	1.00E+00	-0.85	1.13E-01	6.63E-01
**TRAV6**	0.59	8.36E-01	1.00E+00	2.43	1.13E-01	6.63E-01
**SRPK3**	-0.24	6.83E-01	1.00E+00	-1.47	1.13E-01	6.63E-01
**TGM2**	0.85	2.91E-01	1.00E+00	1.51	1.14E-01	6.63E-01

**MT-CYB**	-0.07	8.82E-01	1.00E+00	-0.58	1.14E-01	6.63E-01
**AL513327.1**	-1.49	3.49E-01	1.00E+00	1.26	1.14E-01	6.63E-01
**CD302**	0.50	2.14E-01	1.00E+00	-0.68	1.14E-01	6.63E-01
**KERA**	1.26	4.29E-01	1.00E+00	2.06	1.14E-01	6.63E-01
**TNF**	-1.03	1.66E-01	1.00E+00	0.86	1.14E-01	6.63E-01
**AC025884.1**	-2.90	2.64E-01	1.00E+00	2.36	1.14E-01	6.63E-01
**C15orf54**	-0.68	8.44E-01	1.00E+00	3.57	1.14E-01	6.63E-01
**CKS1BP3**	2.39	4.11E-02	1.00E+00	-0.87	1.14E-01	6.63E-01
**GMIP**	-0.28	2.87E-01	1.00E+00	1.12	1.14E-01	6.63E-01
**CLEC4A**	2.25	5.07E-01	1.00E+00	0.86	1.14E-01	6.63E-01
**MIAT**	-2.66	8.20E-02	1.00E+00	1.77	1.14E-01	6.63E-01
**BMS1P10**	1.05	6.27E-02	1.00E+00	-0.86	1.14E-01	6.63E-01
**SVIL2P**	0.07	9.43E-01	1.00E+00	1.04	1.14E-01	6.63E-01
**LAMC1**	0.30	4.56E-01	1.00E+00	0.88	1.14E-01	6.63E-01
**AP002387.1**	-0.69	2.40E-01	1.00E+00	-0.87	1.14E-01	6.63E-01
**C1orf54**	0.12	8.18E-01	1.00E+00	0.51	1.14E-01	6.63E-01
**DBIL5P**	0.22	6.57E-01	1.00E+00	-1.04	1.14E-01	6.63E-01
**RBKS**	0.18	6.55E-01	1.00E+00	-0.41	1.14E-01	6.63E-01
**PIM1**	-0.23	6.56E-01	1.00E+00	0.53	1.14E-01	6.63E-01
**ZNF583**	0.54	1.53E-01	1.00E+00	-0.55	1.14E-01	6.63E-01
**AC245128.3**	-1.52	6.60E-01	1.00E+00	2.40	1.14E-01	6.63E-01
**KLF3**	-0.20	5.64E-01	1.00E+00	-0.44	1.14E-01	6.63E-01
**ZNF887P**	0.03	9.78E-01	1.00E+00	-1.43	1.14E-01	6.63E-01
**SLC25A30**	0.41	2.37E-01	1.00E+00	0.57	1.14E-01	6.63E-01
**TMEM39A**	0.23	4.09E-01	1.00E+00	0.40	1.14E-01	6.63E-01
**GOLIM4**	-0.21	5.39E-01	1.00E+00	0.50	1.14E-01	6.63E-01
**EPC2**	-0.01	9.69E-01	1.00E+00	0.65	1.14E-01	6.63E-01
**LINC02482**	-1.02	4.20E-01	1.00E+00	-1.00	1.14E-01	6.63E-01
**SIAH2**	0.15	5.53E-01	1.00E+00	-0.75	1.14E-01	6.63E-01
**RGS1**	-2.62	2.83E-02	9.75E-01	0.87	1.14E-01	6.63E-01
**ZNF276**	-0.45	1.06E-01	1.00E+00	0.78	1.14E-01	6.63E-01
**RP11-640M9.2**	0.04	9.00E-01	1.00E+00	0.66	1.14E-01	6.63E-01

**LRRC63**	-0.74	7.29E-01	1.00E+00	-1.69	1.14E-01	6.63E-01
**UTP14C**	0.49	6.37E-02	1.00E+00	-0.47	1.14E-01	6.63E-01
**MFHAS1**	-0.62	9.31E-02	1.00E+00	0.64	1.14E-01	6.63E-01
**AL031651.2**	0.41	8.93E-01	1.00E+00	2.49	1.14E-01	6.63E-01
**EEF2**	-0.28	3.29E-01	1.00E+00	0.65	1.14E-01	6.63E-01
**TMEM14C**	0.24	2.02E-01	1.00E+00	-0.38	1.14E-01	6.63E-01
**IL1R2**	0.90	1.10E-01	1.00E+00	0.71	1.15E-01	6.63E-01
**AP001000.1**	0.24	6.18E-01	1.00E+00	-0.78	1.15E-01	6.63E-01
**AL355802.1**	-2.06	3.90E-01	1.00E+00	-1.35	1.15E-01	6.63E-01
**AC144652.1**	-0.14	7.56E-01	1.00E+00	-1.03	1.15E-01	6.64E-01
**MIPOL1**	0.05	8.89E-01	1.00E+00	-0.46	1.15E-01	6.64E-01
**HMBOX1**	0.19	5.25E-01	1.00E+00	-0.47	1.15E-01	6.64E-01
**AL445253.1**	-0.51	1.71E-01	1.00E+00	-1.28	1.15E-01	6.64E-01
**CRYZL2P**	0.24	3.16E-01	1.00E+00	-0.82	1.15E-01	6.64E-01
**DM1-AS**	-0.72	6.43E-01	1.00E+00	1.34	1.15E-01	6.64E-01
**C2CD4B**	-2.80	6.94E-04	1.25E-01	-1.62	1.15E-01	6.65E-01
**DGUOK-AS1**	-0.18	7.21E-01	1.00E+00	-0.97	1.15E-01	6.65E-01
**RGL3**	0.37	5.84E-01	1.00E+00	1.26	1.15E-01	6.65E-01
**TMEM132E**	-0.59	2.76E-01	1.00E+00	1.98	1.15E-01	6.65E-01
**TMEM266**	-0.07	8.66E-01	1.00E+00	0.93	1.15E-01	6.65E-01
**CCNK**	-0.06	8.28E-01	1.00E+00	0.59	1.15E-01	6.65E-01
**NSUN5**	-0.12	5.93E-01	1.00E+00	0.32	1.15E-01	6.65E-01
**PPM1K**	0.48	2.46E-01	1.00E+00	0.67	1.15E-01	6.66E-01
**H2AFV**	0.11	6.17E-01	1.00E+00	-0.41	1.15E-01	6.66E-01
**AC087893.1**	-0.44	9.00E-01	1.00E+00	2.65	1.16E-01	6.66E-01
**CAP2**	0.49	5.21E-01	1.00E+00	-0.75	1.16E-01	6.66E-01
**U6**	2.39	4.82E-01	1.00E+00	-1.22	1.16E-01	6.66E-01
**TBC1D32**	0.12	7.61E-01	1.00E+00	-0.58	1.16E-01	6.66E-01
**ASNSD1**	0.19	4.25E-01	1.00E+00	-0.39	1.16E-01	6.66E-01
**MT-ND6**	0.07	8.61E-01	1.00E+00	-0.52	1.16E-01	6.66E-01
**SPTY2D1OS**	0.49	7.87E-01	1.00E+00	-0.98	1.16E-01	6.66E-01
**MEX3A**	-0.88	8.79E-02	1.00E+00	1.21	1.16E-01	6.66E-01

**RYR1**	0.34	3.17E-01	1.00E+00	-0.86	1.16E-01	6.66E-01
**TMEM240**	0.07	9.06E-01	1.00E+00	1.88	1.16E-01	6.66E-01
**PABPC4L**	-0.61	1.61E-01	1.00E+00	0.97	1.16E-01	6.66E-01
**NDUFA1**	0.07	7.70E-01	1.00E+00	-0.59	1.16E-01	6.66E-01
**XLOC_009680**	0.44	8.99E-01	1.00E+00	-3.03	1.16E-01	6.66E-01
**C12orf54**	-3.00	3.20E-02	1.00E+00	1.02	1.16E-01	6.66E-01
**PRDX3**	0.37	3.77E-01	1.00E+00	-0.64	1.16E-01	6.66E-01
**G33806**	0.21	8.68E-01	1.00E+00	2.02	1.16E-01	6.66E-01
**CLSPN**	-0.40	3.26E-01	1.00E+00	0.78	1.16E-01	6.66E-01
**UPF3AP3**	0.58	3.93E-01	1.00E+00	1.71	1.16E-01	6.66E-01
**AP000344.1**	1.19	1.63E-01	1.00E+00	1.41	1.16E-01	6.66E-01
**TNFSF8**	-0.04	9.46E-01	1.00E+00	1.20	1.16E-01	6.66E-01
**LINC01091**	0.46	2.83E-01	1.00E+00	-0.66	1.16E-01	6.66E-01
**TMIGD2**	-0.04	9.73E-01	1.00E+00	2.01	1.16E-01	6.66E-01
**SMOC2**	0.52	2.22E-01	1.00E+00	0.61	1.16E-01	6.66E-01
**AC079140.3**	0.24	8.92E-01	1.00E+00	-1.67	1.16E-01	6.66E-01
**RPL10P12**	0.58	8.66E-01	1.00E+00	1.44	1.16E-01	6.66E-01
**BCAT2**	-0.11	6.90E-01	1.00E+00	-0.35	1.16E-01	6.66E-01
**SLC39A14**	0.17	4.50E-01	1.00E+00	0.61	1.16E-01	6.66E-01
**CEP63**	-0.04	8.20E-01	1.00E+00	-0.32	1.16E-01	6.66E-01
**AC004980.1**	-0.24	5.46E-01	1.00E+00	-0.79	1.16E-01	6.67E-01
**XLOC_002619**	-1.00	5.65E-01	1.00E+00	-1.81	1.16E-01	6.67E-01
**CD69**	-0.16	8.52E-01	1.00E+00	0.93	1.16E-01	6.67E-01
**GLA**	0.05	7.41E-01	1.00E+00	0.41	1.17E-01	6.67E-01
**QSOX2**	0.12	5.35E-01	1.00E+00	0.95	1.17E-01	6.67E-01
**TLR6**	-0.67	1.38E-01	1.00E+00	1.11	1.17E-01	6.67E-01
**AL022328.3**	0.30	7.80E-01	1.00E+00	2.41	1.17E-01	6.67E-01
**VIPR1-AS1**	1.28	1.06E-01	1.00E+00	-1.41	1.17E-01	6.68E-01
**ZUP1**	-0.02	9.57E-01	1.00E+00	0.41	1.17E-01	6.68E-01
**F10**	0.50	1.26E-01	1.00E+00	0.84	1.17E-01	6.68E-01
**PIP4K2A**	-0.03	9.02E-01	1.00E+00	0.40	1.17E-01	6.68E-01
**AC105235.1**	-0.06	9.86E-01	1.00E+00	-3.54	1.17E-01	6.68E-01

**TAP2**	0.27	5.30E-01	1.00E+00	0.81	1.17E-01	6.68E-01
**CNNM3-DT**	-0.35	5.99E-01	1.00E+00	1.30	1.17E-01	6.68E-01
**EPB42**	6.56	4.90E-02	1.00E+00	-1.91	1.17E-01	6.68E-01
**ZBTB47**	-0.04	9.35E-01	1.00E+00	1.20	1.17E-01	6.68E-01
**DNAJC7**	-0.16	4.29E-01	1.00E+00	0.30	1.17E-01	6.68E-01
**CAMK2A**	-4.79	1.22E-02	6.87E-01	1.58	1.17E-01	6.68E-01
**ACVR1**	-0.14	5.76E-01	1.00E+00	0.60	1.17E-01	6.68E-01
**AC023024.1**	-0.30	7.07E-01	1.00E+00	1.38	1.17E-01	6.68E-01
**THRSP**	4.79	2.88E-03	2.79E-01	-1.38	1.17E-01	6.68E-01
**C8orf74**	-0.60	4.91E-01	1.00E+00	1.92	1.17E-01	6.68E-01
**PLIN2**	0.21	4.49E-01	1.00E+00	-0.65	1.17E-01	6.69E-01
**ANXA3**	0.17	7.47E-01	1.00E+00	-0.49	1.17E-01	6.69E-01
**AC090602.1**	-0.01	9.97E-01	1.00E+00	-1.07	1.17E-01	6.69E-01
**MRPS10**	0.08	5.96E-01	1.00E+00	-0.32	1.17E-01	6.69E-01
**FABP4**	3.51	1.90E-02	8.66E-01	-1.34	1.18E-01	6.70E-01
**NEK9**	0.23	2.88E-01	1.00E+00	0.52	1.18E-01	6.70E-01
**PCBD2**	0.17	4.93E-01	1.00E+00	-0.45	1.18E-01	6.70E-01
**SLPI**	0.23	5.05E-01	1.00E+00	0.73	1.18E-01	6.70E-01
**CEP170B**	-0.35	3.74E-01	1.00E+00	1.09	1.18E-01	6.70E-01
**APOOL**	-0.29	1.38E-01	1.00E+00	-0.38	1.18E-01	6.70E-01
**CRTC1**	-0.60	1.02E-01	1.00E+00	1.12	1.18E-01	6.70E-01
**TSSK3**	-0.63	8.00E-01	1.00E+00	-1.36	1.18E-01	6.70E-01
**NHP2P1**	-0.34	9.19E-01	1.00E+00	-0.86	1.18E-01	6.70E-01
**FAM156A**	-0.93	7.45E-02	1.00E+00	-0.55	1.18E-01	6.70E-01
**AP002761.4**	0.41	2.09E-01	1.00E+00	1.10	1.18E-01	6.70E-01
**PHYHIP**	0.77	1.24E-02	6.93E-01	-0.60	1.18E-01	6.70E-01
**COX11**	-0.19	4.26E-01	1.00E+00	-0.39	1.18E-01	6.70E-01
**AL139398.1**	-0.89	4.07E-01	1.00E+00	1.45	1.18E-01	6.70E-01
**TRIM24**	-0.12	4.91E-01	1.00E+00	-0.60	1.18E-01	6.70E-01
**EXOC5**	0.20	4.63E-01	1.00E+00	-0.28	1.18E-01	6.71E-01
**SPECC1**	-0.15	6.49E-01	1.00E+00	0.50	1.18E-01	6.71E-01
**CUZD1**	1.39	1.41E-01	1.00E+00	1.81	1.18E-01	6.71E-01

**DDX50P1**	2.41	3.27E-01	1.00E+00	1.79	1.18E-01	6.71E-01
**SIGMAR1**	-0.28	3.10E-01	1.00E+00	-0.31	1.18E-01	6.71E-01
**SNORA53**	0.56	8.71E-01	1.00E+00	-3.25	1.18E-01	6.71E-01
**MAPK4**	0.05	9.47E-01	1.00E+00	-1.33	1.18E-01	6.71E-01
**PKD2L1**	0.50	8.84E-01	1.00E+00	2.32	1.18E-01	6.71E-01
**MEI4**	-0.23	8.82E-01	1.00E+00	2.55	1.18E-01	6.71E-01
**AC010280.2**	0.96	4.77E-01	1.00E+00	-1.44	1.18E-01	6.71E-01
**ACYP1**	0.32	3.05E-01	1.00E+00	-0.62	1.19E-01	6.71E-01
**DACT1**	-0.16	7.41E-01	1.00E+00	1.23	1.19E-01	6.71E-01
**UBE2V2**	0.14	5.10E-01	1.00E+00	-0.62	1.19E-01	6.71E-01
**PHF21A**	-0.08	7.59E-01	1.00E+00	0.51	1.19E-01	6.71E-01
**ARHGEF10**	-0.25	3.93E-01	1.00E+00	0.69	1.19E-01	6.71E-01
**C3AR1**	0.43	2.56E-01	1.00E+00	1.10	1.19E-01	6.71E-01
**PADI1**	-0.75	2.20E-01	1.00E+00	1.36	1.19E-01	6.71E-01
**ADAP1**	-0.53	2.33E-01	1.00E+00	0.86	1.19E-01	6.71E-01
**SERPINF1**	0.68	1.39E-01	1.00E+00	0.79	1.19E-01	6.71E-01
**AC006504.3**	-0.16	9.62E-01	1.00E+00	-2.81	1.19E-01	6.71E-01
**AC124319.1**	2.57	3.89E-01	1.00E+00	2.75	1.19E-01	6.71E-01
**AC004656.1**	0.32	6.51E-01	1.00E+00	-0.70	1.19E-01	6.71E-01
**CC2D1B**	-0.04	8.25E-01	1.00E+00	0.69	1.19E-01	6.72E-01
**PRCP**	0.10	6.88E-01	1.00E+00	0.37	1.19E-01	6.72E-01
**SLC19A3**	2.47	3.05E-03	2.89E-01	-1.14	1.19E-01	6.72E-01
**RHOXF1-AS1**	1.40	2.88E-01	1.00E+00	-1.58	1.19E-01	6.72E-01
**FOLR2**	0.27	6.09E-01	1.00E+00	0.76	1.19E-01	6.72E-01
**CS**	0.03	8.68E-01	1.00E+00	-0.34	1.19E-01	6.72E-01
**XLOC_005629**	-1.85	4.39E-01	1.00E+00	-2.51	1.19E-01	6.72E-01
**KMT2D**	-0.31	2.84E-01	1.00E+00	1.21	1.19E-01	6.72E-01
**AC068580.3**	0.33	8.43E-01	1.00E+00	2.28	1.19E-01	6.72E-01
**HIST1H3H**	-1.68	5.89E-02	1.00E+00	-0.74	1.19E-01	6.72E-01
**GCAT**	-0.22	5.18E-01	1.00E+00	-0.38	1.19E-01	6.72E-01
**CCL2**	-1.02	1.05E-01	1.00E+00	0.78	1.19E-01	6.72E-01
**XLOC_002390**	1.26	6.46E-01	1.00E+00	2.57	1.19E-01	6.72E-01

**CCR2**	-0.40	4.44E-01	1.00E+00	1.44	1.19E-01	6.72E-01
**ANKRD44**	0.08	8.06E-01	1.00E+00	0.60	1.19E-01	6.72E-01
**C19orf38**	-0.56	4.18E-01	1.00E+00	1.75	1.19E-01	6.72E-01
**SENP8**	0.01	9.82E-01	1.00E+00	-0.66	1.19E-01	6.72E-01
**WASH4P**	-0.45	1.96E-01	1.00E+00	0.94	1.19E-01	6.72E-01
**CRAT**	0.50	3.73E-01	1.00E+00	-0.93	1.19E-01	6.72E-01
**SCOC**	0.16	5.92E-01	1.00E+00	-0.63	1.19E-01	6.72E-01
**RNF167**	-0.07	7.72E-01	1.00E+00	0.47	1.19E-01	6.72E-01
**CCL21**	0.10	7.80E-01	1.00E+00	0.84	1.20E-01	6.72E-01
**BAK1P1**	-0.83	3.88E-01	1.00E+00	-1.33	1.20E-01	6.72E-01
**NBR2**	-0.02	9.61E-01	1.00E+00	-0.54	1.20E-01	6.72E-01
**INAFM1**	-0.33	3.19E-01	1.00E+00	0.70	1.20E-01	6.72E-01
**SERPING1**	0.27	4.76E-01	1.00E+00	0.73	1.20E-01	6.72E-01
**DACH2**	-2.03	5.87E-02	1.00E+00	-1.83	1.20E-01	6.72E-01
**AC007182.1**	-0.36	7.47E-01	1.00E+00	1.10	1.20E-01	6.72E-01
**GMPPA**	-0.09	6.89E-01	1.00E+00	0.36	1.20E-01	6.72E-01
**USP37**	-0.29	4.36E-01	1.00E+00	-0.43	1.20E-01	6.72E-01
**ELP5**	0.05	7.94E-01	1.00E+00	-0.32	1.20E-01	6.72E-01
**AC093423.2**	0.92	4.53E-01	1.00E+00	-1.65	1.20E-01	6.72E-01
**AKAP11**	0.02	9.29E-01	1.00E+00	0.43	1.20E-01	6.73E-01
**SAMD4A**	0.34	4.15E-01	1.00E+00	0.76	1.20E-01	6.73E-01
**AC022149.1**	0.04	9.39E-01	1.00E+00	-0.56	1.20E-01	6.73E-01
**AC107021.2**	0.99	6.51E-01	1.00E+00	2.09	1.20E-01	6.73E-01
**AGPAT4-IT1**	1.77	1.80E-01	1.00E+00	1.55	1.20E-01	6.73E-01
**ELF3-AS1**	-2.55	8.32E-02	1.00E+00	1.06	1.20E-01	6.73E-01
**RNASEH2C**	-0.12	6.64E-01	1.00E+00	0.28	1.20E-01	6.73E-01
**TATDN1**	0.07	7.20E-01	1.00E+00	-0.42	1.20E-01	6.73E-01
**AC092535.4**	-0.55	6.36E-01	1.00E+00	1.67	1.20E-01	6.73E-01
**AC009271.1**	0.26	8.42E-01	1.00E+00	1.43	1.20E-01	6.73E-01
**ANKRD20A14P**	-0.95	1.35E-01	1.00E+00	-1.10	1.20E-01	6.74E-01
**AL357033.3**	-0.33	6.64E-01	1.00E+00	1.54	1.20E-01	6.74E-01
**NALT1**	0.48	6.80E-01	1.00E+00	-1.40	1.21E-01	6.74E-01

**PHF5A**	0.10	6.22E-01	1.00E+00	0.54	1.21E-01	6.74E-01
**EIF2B5**	0.16	3.42E-01	1.00E+00	-0.69	1.21E-01	6.74E-01
**LINC01564**	0.71	7.07E-01	1.00E+00	-2.39	1.21E-01	6.74E-01
**DICER1-AS1**	-0.51	2.20E-01	1.00E+00	-0.62	1.21E-01	6.74E-01
**VIL1**	-1.60	4.02E-01	1.00E+00	3.58	1.21E-01	6.74E-01
**KDELC1P1**	1.76	6.05E-01	1.00E+00	-3.70	1.21E-01	6.74E-01
**EHBP1L1**	-0.42	1.16E-01	1.00E+00	1.70	1.21E-01	6.74E-01
**SORCS3**	-0.69	4.65E-01	1.00E+00	1.86	1.21E-01	6.74E-01
**AL365203.2**	-0.22	7.22E-01	1.00E+00	1.11	1.21E-01	6.74E-01
**AC009041.2**	0.21	8.09E-01	1.00E+00	-0.95	1.21E-01	6.74E-01
**SUCO**	0.15	5.89E-01	1.00E+00	0.33	1.21E-01	6.74E-01
**AC092849.1**	-2.31	3.09E-01	1.00E+00	-1.39	1.21E-01	6.74E-01
**KLHDC2**	0.12	6.13E-01	1.00E+00	-0.65	1.21E-01	6.74E-01
**TAPT1**	0.35	1.64E-01	1.00E+00	-0.68	1.21E-01	6.74E-01
**CBWD6**	0.17	7.08E-01	1.00E+00	-0.53	1.21E-01	6.74E-01
**SCYL1**	-0.08	6.94E-01	1.00E+00	0.52	1.21E-01	6.74E-01
**ZBED8**	0.37	5.95E-01	1.00E+00	-0.93	1.21E-01	6.74E-01
**AC022413.1**	-0.44	6.67E-01	1.00E+00	-1.18	1.21E-01	6.74E-01
**NDUFA3**	0.08	7.37E-01	1.00E+00	-0.54	1.21E-01	6.74E-01
**AC099506.1**	NA	NA	NA	2.07	1.21E-01	6.74E-01
**TTC12**	0.29	4.89E-01	1.00E+00	0.56	1.21E-01	6.74E-01
**VDAC2**	-0.13	3.90E-01	1.00E+00	-0.32	1.21E-01	6.74E-01
**HIST2H2BA**	0.40	5.84E-01	1.00E+00	1.47	1.21E-01	6.74E-01
**TRAPPC11**	0.03	8.69E-01	1.00E+00	-0.34	1.21E-01	6.74E-01
**ANKRD13D**	-0.05	8.29E-01	1.00E+00	0.72	1.21E-01	6.74E-01
**ZNF826P**	0.31	6.22E-01	1.00E+00	-0.79	1.21E-01	6.74E-01
**AL451165.2**	0.17	7.13E-01	1.00E+00	-0.69	1.21E-01	6.74E-01
**AL137003.1**	0.26	6.30E-01	1.00E+00	0.57	1.21E-01	6.74E-01
**AC002524.1**	-1.18	4.82E-01	1.00E+00	-0.97	1.21E-01	6.74E-01
**GPR141**	0.67	5.94E-01	1.00E+00	1.31	1.21E-01	6.74E-01
**ELOVL6**	0.25	4.74E-01	1.00E+00	-0.46	1.21E-01	6.74E-01
**RNA5-8SP3**	0.66	8.49E-01	1.00E+00	-2.16	1.21E-01	6.74E-01

**ZNF655**	0.13	5.88E-01	1.00E+00	-0.25	1.21E-01	6.74E-01
**LINC01181**	-2.86	6.35E-02	1.00E+00	2.14	1.21E-01	6.74E-01
**KARS**	-0.25	1.09E-01	1.00E+00	-0.24	1.21E-01	6.74E-01
**CASP6**	0.06	7.74E-01	1.00E+00	-0.60	1.22E-01	6.74E-01
**GUCY2D**	-3.37	2.22E-03	2.39E-01	1.02	1.22E-01	6.74E-01
**AC215522.2**	-2.94	3.86E-01	1.00E+00	1.80	1.22E-01	6.74E-01
**AC092384.2**	0.29	5.13E-01	1.00E+00	-1.09	1.22E-01	6.75E-01
**PLXNA1**	-0.07	8.21E-01	1.00E+00	0.95	1.22E-01	6.75E-01
**CASP7**	0.23	4.68E-01	1.00E+00	0.43	1.22E-01	6.76E-01
**AL354714.2**	-0.17	9.61E-01	1.00E+00	-0.78	1.22E-01	6.76E-01
**MICAL1**	-0.34	2.78E-01	1.00E+00	0.93	1.22E-01	6.76E-01
**SIPA1L2**	-0.31	3.54E-01	1.00E+00	0.80	1.22E-01	6.76E-01
**DEFB1**	0.26	5.07E-01	1.00E+00	-0.85	1.22E-01	6.76E-01
**FAM214B**	-0.24	3.22E-01	1.00E+00	0.72	1.22E-01	6.76E-01
**AL357054.4**	-0.02	9.68E-01	1.00E+00	1.13	1.22E-01	6.76E-01
**LBH**	0.18	6.42E-01	1.00E+00	0.72	1.22E-01	6.76E-01
**ITPR1-DT**	0.03	9.87E-01	1.00E+00	-2.09	1.22E-01	6.76E-01
**TBX22**	1.71	4.45E-01	1.00E+00	-2.65	1.22E-01	6.76E-01
**MICU2**	0.03	9.22E-01	1.00E+00	-0.38	1.22E-01	6.76E-01
**BASP1**	-0.46	2.03E-01	1.00E+00	0.99	1.22E-01	6.76E-01
**RRM1**	-0.09	7.14E-01	1.00E+00	-0.35	1.22E-01	6.76E-01
**HAT1**	-0.05	7.99E-01	1.00E+00	-0.44	1.22E-01	6.76E-01
**GRID2**	-2.18	1.94E-01	1.00E+00	-3.50	1.23E-01	6.76E-01
**AC007663.1**	0.41	4.91E-01	1.00E+00	-0.87	1.23E-01	6.76E-01
**G6692**	NA	NA	NA	3.94	1.23E-01	6.76E-01
**FAM81B**	-0.74	6.61E-01	1.00E+00	1.67	1.23E-01	6.76E-01
**VAMP4**	0.40	1.87E-01	1.00E+00	-0.49	1.23E-01	6.76E-01
**TMEM200C**	-0.12	7.84E-01	1.00E+00	1.21	1.23E-01	6.76E-01
**KRAS**	-0.10	7.30E-01	1.00E+00	-0.31	1.23E-01	6.77E-01
**UBXN1**	0.01	9.76E-01	1.00E+00	0.32	1.23E-01	6.77E-01
**EIF5A2**	-0.11	7.05E-01	1.00E+00	-0.41	1.23E-01	6.77E-01
**C2orf72**	0.84	5.03E-01	1.00E+00	-1.24	1.23E-01	6.77E-01

**G39807**	-1.12	3.85E-01	1.00E+00	1.77	1.23E-01	6.78E-01
**DCAF13P3**	-0.22	9.17E-01	1.00E+00	2.17	1.23E-01	6.78E-01
**AC002091.1**	-1.78	1.96E-01	1.00E+00	2.02	1.23E-01	6.78E-01
**TSPAN32**	-0.48	5.76E-01	1.00E+00	1.22	1.23E-01	6.78E-01
**DRAM1**	0.15	6.58E-01	1.00E+00	0.84	1.23E-01	6.78E-01
**ELOF1**	0.09	5.94E-01	1.00E+00	0.41	1.23E-01	6.78E-01
**KAT7**	-0.19	3.82E-01	1.00E+00	-0.28	1.23E-01	6.78E-01
**AC016708.1**	0.17	6.66E-01	1.00E+00	-1.17	1.23E-01	6.78E-01
**AF064860.2**	-0.52	6.72E-01	1.00E+00	-2.26	1.23E-01	6.78E-01
**PIP4K2B**	0.26	3.25E-01	1.00E+00	0.67	1.23E-01	6.78E-01
**PDS5B**	0.09	6.40E-01	1.00E+00	-0.35	1.24E-01	6.78E-01
**PPP1R2P1**	-2.02	5.55E-01	1.00E+00	-1.79	1.24E-01	6.78E-01
**AC011481.3**	-1.27	1.51E-01	1.00E+00	-1.47	1.24E-01	6.78E-01
**DENND2D**	0.48	2.16E-01	1.00E+00	0.50	1.24E-01	6.78E-01
**HS6ST3**	0.52	4.80E-01	1.00E+00	-1.61	1.24E-01	6.78E-01
**SOX21**	-0.18	6.64E-01	1.00E+00	-1.08	1.24E-01	6.78E-01
**LINC00449**	-2.52	4.51E-01	1.00E+00	-1.43	1.24E-01	6.78E-01
**NUDT11**	-0.13	7.12E-01	1.00E+00	-0.57	1.24E-01	6.78E-01
**FAM69C**	-0.67	8.28E-02	1.00E+00	-1.10	1.24E-01	6.78E-01
**AC027237.3**	0.01	9.93E-01	1.00E+00	1.53	1.24E-01	6.78E-01
**ADA**	-0.03	9.61E-01	1.00E+00	0.58	1.24E-01	6.78E-01
**G12891**	1.70	1.05E-01	1.00E+00	-1.74	1.24E-01	6.78E-01
**MELK**	-0.08	8.60E-01	1.00E+00	0.73	1.24E-01	6.78E-01
**ATXN7L1**	-0.22	4.29E-01	1.00E+00	0.35	1.24E-01	6.78E-01
**G13124**	0.00	9.98E-01	1.00E+00	-1.25	1.24E-01	6.78E-01
**C3orf70**	0.15	7.55E-01	1.00E+00	-0.53	1.24E-01	6.78E-01
**SERPINB13**	-0.03	9.64E-01	1.00E+00	1.10	1.24E-01	6.78E-01
**AC138392.1**	0.83	3.66E-01	1.00E+00	0.88	1.24E-01	6.78E-01
**UFM1**	0.19	4.13E-01	1.00E+00	-0.48	1.24E-01	6.78E-01
**VPS29**	0.12	5.78E-01	1.00E+00	-0.67	1.24E-01	6.78E-01
**AL133410.1**	3.48	3.03E-01	1.00E+00	-2.40	1.24E-01	6.78E-01
**IL1B**	-0.85	3.90E-01	1.00E+00	1.43	1.24E-01	6.78E-01

**RP11-14N7.2**	3.76	5.16E-02	1.00E+00	1.34	1.24E-01	6.78E-01
**ZNF577**	0.05	8.72E-01	1.00E+00	-0.57	1.24E-01	6.78E-01
**STARD7**	0.39	5.36E-02	1.00E+00	-0.41	1.24E-01	6.78E-01
**TM2D1**	0.41	1.27E-01	1.00E+00	-0.66	1.24E-01	6.78E-01
**ADGRL3**	-0.79	1.22E-01	1.00E+00	-0.85	1.24E-01	6.78E-01
**MYH14**	-0.10	8.06E-01	1.00E+00	-1.09	1.24E-01	6.78E-01
**CHST3**	0.08	7.36E-01	1.00E+00	0.86	1.24E-01	6.78E-01
**XLOC_013424**	1.01	1.37E-01	1.00E+00	-0.83	1.24E-01	6.78E-01
**GPRASP2**	0.11	6.34E-01	1.00E+00	-0.48	1.24E-01	6.78E-01
**CASP10**	0.18	6.40E-01	1.00E+00	0.66	1.24E-01	6.78E-01
**SLC43A2**	-0.20	6.81E-01	1.00E+00	1.20	1.24E-01	6.78E-01
**USP36**	-0.04	8.54E-01	1.00E+00	0.52	1.24E-01	6.78E-01
**DMAC2**	-0.03	8.75E-01	1.00E+00	0.17	1.24E-01	6.78E-01
**PRICKLE3**	-0.33	2.84E-01	1.00E+00	0.43	1.25E-01	6.78E-01
**AC108047.1**	-0.14	7.84E-01	1.00E+00	-0.68	1.25E-01	6.78E-01
**AC017100.1**	0.44	5.98E-01	1.00E+00	-1.45	1.25E-01	6.78E-01
**KIAA1211**	-0.45	2.66E-01	1.00E+00	1.64	1.25E-01	6.78E-01
**AC019181.1**	-0.18	9.58E-01	1.00E+00	1.72	1.25E-01	6.78E-01
**DPY19L4**	0.14	6.99E-01	1.00E+00	-0.41	1.25E-01	6.78E-01
**SCN8A**	-2.41	1.32E-02	7.22E-01	1.15	1.25E-01	6.78E-01
**CCDC71**	-0.18	4.11E-01	1.00E+00	0.99	1.25E-01	6.78E-01
**CERS6**	0.12	7.13E-01	1.00E+00	-0.76	1.25E-01	6.78E-01
**TIMM10B**	0.09	6.66E-01	1.00E+00	-0.43	1.25E-01	6.78E-01
**CD52**	-0.95	1.93E-01	1.00E+00	0.78	1.25E-01	6.78E-01
**NFKBID**	-0.53	3.45E-01	1.00E+00	0.94	1.25E-01	6.78E-01
**ACSL1**	0.47	3.18E-01	1.00E+00	-0.69	1.25E-01	6.78E-01
**HEG1**	-0.06	8.90E-01	1.00E+00	0.85	1.25E-01	6.78E-01
**PGA3**	NA	NA	NA	1.62	1.25E-01	6.78E-01
**BET1**	0.35	1.31E-01	1.00E+00	-0.56	1.25E-01	6.78E-01
**LINC02568**	-0.20	8.23E-01	1.00E+00	-1.21	1.25E-01	6.78E-01
**RFC5**	-0.13	5.95E-01	1.00E+00	-0.47	1.25E-01	6.78E-01
**HHLA3**	0.24	5.02E-01	1.00E+00	-0.47	1.25E-01	6.78E-01

**HIF1A**	0.00	9.91E-01	1.00E+00	0.32	1.25E-01	6.78E-01
**SLC25A45**	-0.21	6.56E-01	1.00E+00	0.68	1.25E-01	6.78E-01
**COBLL1**	0.29	2.29E-01	1.00E+00	-0.41	1.25E-01	6.78E-01
**AL162171.3**	-0.78	5.59E-01	1.00E+00	1.63	1.25E-01	6.78E-01
**ZNF507**	-0.02	8.85E-01	1.00E+00	0.35	1.25E-01	6.78E-01
**KCNA4**	1.31	1.03E-01	1.00E+00	-1.90	1.25E-01	6.78E-01
**PGS1**	-0.40	3.34E-02	1.00E+00	0.43	1.25E-01	6.78E-01
**SLC25A39**	-0.21	4.33E-01	1.00E+00	0.24	1.25E-01	6.78E-01
**XLOC_014244**	0.13	9.71E-01	1.00E+00	2.40	1.25E-01	6.78E-01
**AC007406.3**	-1.40	4.44E-01	1.00E+00	1.69	1.25E-01	6.78E-01
**SNRNP27**	0.17	4.09E-01	1.00E+00	-0.61	1.25E-01	6.78E-01
**GTSE1**	0.00	9.96E-01	1.00E+00	0.83	1.25E-01	6.78E-01
**KLB**	2.10	5.15E-03	4.18E-01	-0.98	1.25E-01	6.78E-01
**CYP2R1**	-0.34	2.25E-01	1.00E+00	-0.41	1.25E-01	6.79E-01
**URAHP**	0.59	7.92E-01	1.00E+00	1.17	1.25E-01	6.79E-01
**HDAC11**	0.22	2.83E-01	1.00E+00	-0.35	1.26E-01	6.80E-01
**C2orf27AP1**	0.95	4.85E-01	1.00E+00	-1.89	1.26E-01	6.80E-01
**DHDDS**	0.20	4.81E-01	1.00E+00	-0.48	1.26E-01	6.80E-01
**RNH1**	-0.04	8.65E-01	1.00E+00	0.26	1.26E-01	6.80E-01
**CHPF2**	-0.35	9.01E-02	1.00E+00	0.90	1.26E-01	6.80E-01
**APOBEC3B**	-1.15	4.09E-02	1.00E+00	0.78	1.26E-01	6.80E-01
**PCNX3**	-0.38	1.82E-01	1.00E+00	0.83	1.26E-01	6.80E-01
**AL590762.3**	NA	NA	NA	-1.14	1.26E-01	6.80E-01
**MIR3188**	-0.06	9.86E-01	1.00E+00	1.67	1.26E-01	6.80E-01
**LINC00933**	-0.39	5.33E-01	1.00E+00	-0.94	1.26E-01	6.80E-01
**CIDEA**	0.83	1.58E-01	1.00E+00	-0.92	1.26E-01	6.80E-01
**MAP3K8**	0.09	8.33E-01	1.00E+00	0.47	1.26E-01	6.80E-01
**CENPA**	0.48	2.73E-01	1.00E+00	-1.17	1.26E-01	6.80E-01
**LRFN5**	1.14	5.32E-02	1.00E+00	1.07	1.26E-01	6.80E-01
**HACD4**	0.00	9.98E-01	1.00E+00	0.48	1.26E-01	6.80E-01
**PIP5K1A**	0.11	6.86E-01	1.00E+00	0.47	1.26E-01	6.80E-01
**TMTC4**	-0.39	1.83E-01	1.00E+00	-0.27	1.26E-01	6.80E-01

**TRUB2**	-0.38	4.78E-02	1.00E+00	-0.33	1.26E-01	6.80E-01
**FMC1**	1.41	3.27E-01	1.00E+00	-1.09	1.26E-01	6.80E-01
**PLCZ1**	0.20	8.65E-01	1.00E+00	1.13	1.26E-01	6.80E-01
**CLCA3P**	-0.23	9.47E-01	1.00E+00	2.08	1.26E-01	6.81E-01
**SMDT1**	-0.14	4.31E-01	1.00E+00	-0.43	1.26E-01	6.81E-01
**AC091153.1**	1.03	5.86E-01	1.00E+00	-1.03	1.26E-01	6.81E-01
**DLGAP3**	-2.06	2.44E-02	9.22E-01	1.39	1.27E-01	6.81E-01
**AC021321.1**	2.31	1.56E-01	1.00E+00	1.88	1.27E-01	6.81E-01
**MTMR7**	-0.62	2.46E-01	1.00E+00	-0.70	1.27E-01	6.81E-01
**AP005329.1**	-2.12	5.35E-01	1.00E+00	-1.24	1.27E-01	6.82E-01
**HNRNPA0**	-0.09	6.44E-01	1.00E+00	0.64	1.27E-01	6.82E-01
**AL928970.1**	-1.90	3.24E-01	1.00E+00	1.17	1.27E-01	6.82E-01
**SCAI**	-0.15	6.65E-01	1.00E+00	-0.45	1.27E-01	6.82E-01
**FKBP1A**	-0.01	9.42E-01	1.00E+00	0.43	1.27E-01	6.82E-01
**ACTR10**	0.11	5.58E-01	1.00E+00	-0.45	1.27E-01	6.82E-01
**PMEPA1**	0.70	2.37E-01	1.00E+00	0.79	1.27E-01	6.82E-01
**GGTA1P**	0.27	4.17E-01	1.00E+00	-0.54	1.27E-01	6.82E-01
**YWHAB**	-0.10	4.16E-01	1.00E+00	-0.21	1.27E-01	6.83E-01
**XLOC_014159**	1.21	3.01E-01	1.00E+00	-1.42	1.27E-01	6.83E-01
**AC074367.1**	NA	NA	NA	-2.31	1.27E-01	6.83E-01
**RCOR3**	0.09	6.59E-01	1.00E+00	-0.34	1.27E-01	6.83E-01
**MLLT10P1**	0.85	3.27E-01	1.00E+00	-1.14	1.27E-01	6.84E-01
**SEPT7P2**	0.19	5.08E-01	1.00E+00	-0.40	1.27E-01	6.84E-01
**RNF7**	0.07	7.12E-01	1.00E+00	-0.47	1.28E-01	6.84E-01
**AP000943.2**	-4.64	4.20E-03	3.64E-01	2.08	1.28E-01	6.84E-01
**SERF1B**	-0.80	8.32E-02	1.00E+00	-0.83	1.28E-01	6.84E-01
**ARHGAP45**	-0.16	5.98E-01	1.00E+00	0.99	1.28E-01	6.84E-01
**TIMP4**	3.07	3.27E-02	1.00E+00	-1.29	1.28E-01	6.84E-01
**BVES-AS1**	0.22	8.80E-01	1.00E+00	1.83	1.28E-01	6.84E-01
**ATP13A5**	-1.12	8.13E-02	1.00E+00	-1.20	1.28E-01	6.84E-01
**LINC02154**	-2.43	4.34E-01	1.00E+00	2.45	1.28E-01	6.85E-01
**AP3M1**	0.20	3.61E-01	1.00E+00	-0.28	1.28E-01	6.85E-01

**HLA-E**	0.00	9.85E-01	1.00E+00	0.40	1.28E-01	6.85E-01
**XLOC_003641**	-0.98	5.26E-01	1.00E+00	-1.49	1.28E-01	6.85E-01
**PGK1P2**	0.84	8.06E-01	1.00E+00	1.07	1.28E-01	6.85E-01
**ARMT1**	0.57	1.30E-01	1.00E+00	-0.58	1.28E-01	6.86E-01
**LAMA2**	0.49	2.66E-01	1.00E+00	0.78	1.28E-01	6.86E-01
**INTS11**	-0.21	3.99E-01	1.00E+00	0.27	1.28E-01	6.86E-01
**RPS2P46**	-0.24	3.70E-01	1.00E+00	-0.81	1.28E-01	6.86E-01
**ZNF302**	0.58	8.90E-02	1.00E+00	-0.45	1.28E-01	6.86E-01
**LRRTM1**	0.41	6.00E-01	1.00E+00	-1.68	1.28E-01	6.86E-01
**FUOM**	0.17	5.18E-01	1.00E+00	0.49	1.28E-01	6.86E-01
**LINC02273**	2.36	1.03E-01	1.00E+00	1.79	1.28E-01	6.86E-01
**FAM187B2P**	-0.18	8.80E-01	1.00E+00	-1.65	1.28E-01	6.86E-01
**PCYOX1**	0.34	2.38E-01	1.00E+00	-0.40	1.28E-01	6.86E-01
**FAM131C**	-0.38	3.48E-01	1.00E+00	-1.24	1.29E-01	6.86E-01
**PAQR7**	0.19	5.80E-01	1.00E+00	0.73	1.29E-01	6.86E-01
**AC095031.1**	2.43	1.68E-01	1.00E+00	-2.44	1.29E-01	6.86E-01
**IFT74**	-0.25	4.20E-01	1.00E+00	-0.43	1.29E-01	6.86E-01
**FABP7**	1.06	7.39E-01	1.00E+00	-1.28	1.29E-01	6.86E-01
**AHCTF1P1**	-1.36	2.62E-01	1.00E+00	-0.73	1.29E-01	6.86E-01
**HMGN2**	-0.21	1.92E-01	1.00E+00	-0.28	1.29E-01	6.86E-01
**KRT34**	-3.31	3.14E-01	1.00E+00	2.22	1.29E-01	6.86E-01
**SEC14L6**	1.16	1.72E-01	1.00E+00	-1.43	1.29E-01	6.86E-01
**HOXC5**	1.05	2.41E-01	1.00E+00	0.87	1.29E-01	6.86E-01
**NHLH2**	-0.22	6.19E-01	1.00E+00	-1.09	1.29E-01	6.86E-01
**TRHDE-AS1**	3.31	1.65E-03	2.25E-01	-1.43	1.29E-01	6.86E-01
**HORMAD2-AS1**	-2.27	4.87E-01	1.00E+00	-1.69	1.29E-01	6.86E-01
**DGCR5**	-0.46	5.10E-01	1.00E+00	-1.06	1.29E-01	6.86E-01
**ACSL4**	0.07	8.31E-01	1.00E+00	0.48	1.29E-01	6.86E-01
**AC138150.1**	-0.25	9.41E-01	1.00E+00	1.07	1.29E-01	6.86E-01
**TBKBP1**	-0.14	6.69E-01	1.00E+00	1.04	1.29E-01	6.86E-01
**RNF175**	0.08	9.11E-01	1.00E+00	1.03	1.29E-01	6.86E-01
**LINC00844**	0.59	6.94E-01	1.00E+00	-1.59	1.29E-01	6.86E-01

**SLITRK4**	0.78	8.75E-02	1.00E+00	0.82	1.29E-01	6.86E-01
**ANKRD20A5P**	-1.19	4.33E-01	1.00E+00	1.27	1.29E-01	6.86E-01
**BBS2**	0.26	2.70E-01	1.00E+00	-0.30	1.29E-01	6.86E-01
**GLI4**	-0.02	9.49E-01	1.00E+00	-0.51	1.29E-01	6.86E-01
**LINC02586**	0.61	7.33E-01	1.00E+00	-2.05	1.29E-01	6.86E-01
**ENKUR**	-3.06	1.41E-01	1.00E+00	1.91	1.29E-01	6.86E-01
**ASS1P12**	-1.02	3.10E-01	1.00E+00	1.43	1.29E-01	6.86E-01
**AC102953.2**	-0.20	7.96E-01	1.00E+00	1.64	1.29E-01	6.86E-01
**AL031055.1**	-1.16	5.14E-01	1.00E+00	-1.35	1.29E-01	6.87E-01
**CHCHD1**	0.19	3.48E-01	1.00E+00	-0.29	1.29E-01	6.87E-01
**SERPINA3**	-3.07	3.47E-01	1.00E+00	2.17	1.29E-01	6.87E-01
**CPNE7**	-0.86	1.39E-01	1.00E+00	1.64	1.29E-01	6.87E-01
**CFLAR-AS1**	-1.78	2.91E-01	1.00E+00	1.13	1.29E-01	6.87E-01
**GLP2R**	1.93	2.09E-01	1.00E+00	-1.92	1.29E-01	6.87E-01
**MAP3K2**	-0.12	6.56E-01	1.00E+00	-0.32	1.30E-01	6.87E-01
**ADH5P4**	NA	NA	NA	-0.99	1.30E-01	6.87E-01
**PXDNL**	-0.57	5.21E-01	1.00E+00	1.21	1.30E-01	6.87E-01
**NOP56P1**	0.43	9.03E-01	1.00E+00	1.99	1.30E-01	6.87E-01
**LINC01355**	1.10	5.54E-01	1.00E+00	-1.50	1.30E-01	6.87E-01
**CEBPE**	-1.68	4.39E-01	1.00E+00	2.18	1.30E-01	6.87E-01
**TCIRG1**	-0.01	9.65E-01	1.00E+00	0.91	1.30E-01	6.87E-01
**ZNF346**	-0.33	1.05E-01	1.00E+00	0.31	1.30E-01	6.88E-01
**C5orf17**	-1.75	1.61E-01	1.00E+00	-1.82	1.30E-01	6.88E-01
**PPM1F**	0.07	8.41E-01	1.00E+00	0.85	1.30E-01	6.88E-01
**G3899**	-0.18	8.38E-01	1.00E+00	1.27	1.30E-01	6.88E-01
**MIR29B2CHG**	1.31	4.10E-02	1.00E+00	0.74	1.30E-01	6.88E-01
**SERPINE2**	-0.07	8.40E-01	1.00E+00	0.69	1.30E-01	6.88E-01
**SACS**	-0.09	6.97E-01	1.00E+00	0.77	1.30E-01	6.88E-01
**HOTAIRM1**	-0.52	2.56E-01	1.00E+00	-0.69	1.30E-01	6.88E-01
**NDUFS5**	-0.03	8.47E-01	1.00E+00	-0.37	1.30E-01	6.88E-01
**RRAGA**	-0.05	7.46E-01	1.00E+00	-0.24	1.30E-01	6.89E-01
**AC113404.3**	0.18	7.74E-01	1.00E+00	-0.58	1.31E-01	6.90E-01

**AC004988.1**	-0.55	8.24E-01	1.00E+00	2.18	1.31E-01	6.90E-01
**ABCC6P1**	-1.77	4.54E-01	1.00E+00	-1.53	1.31E-01	6.90E-01
**SEC61A1**	-0.05	7.65E-01	1.00E+00	0.50	1.31E-01	6.90E-01
**RPL5P23**	-1.02	7.64E-01	1.00E+00	-0.96	1.31E-01	6.90E-01
**KIF1BP**	0.10	6.63E-01	1.00E+00	-0.39	1.31E-01	6.91E-01
**HMGA1P2**	-0.80	5.52E-01	1.00E+00	1.21	1.31E-01	6.91E-01
**ARFGAP1**	-0.04	8.49E-01	1.00E+00	0.79	1.31E-01	6.91E-01
**MGAT1**	-0.04	8.09E-01	1.00E+00	0.60	1.31E-01	6.91E-01
**BDNF**	-0.18	7.42E-01	1.00E+00	0.82	1.31E-01	6.91E-01
**CNDP2**	0.18	4.08E-01	1.00E+00	0.48	1.31E-01	6.91E-01
**RSPO2**	-15.92	3.27E-06	1.12E-03	-3.07	1.31E-01	6.91E-01
**C2orf92**	-0.37	6.38E-01	1.00E+00	-1.25	1.31E-01	6.91E-01
**HIST1H2AH**	2.14	3.70E-01	1.00E+00	-1.18	1.31E-01	6.91E-01
**ZNF282**	-0.34	1.23E-01	1.00E+00	0.98	1.31E-01	6.91E-01
**G26269**	-2.13	9.45E-02	1.00E+00	-1.67	1.31E-01	6.92E-01
**TMEM251**	0.30	3.87E-01	1.00E+00	-0.61	1.31E-01	6.92E-01
**SACM1L**	0.35	2.51E-01	1.00E+00	-0.43	1.32E-01	6.92E-01
**SPAG17**	0.25	7.27E-01	1.00E+00	1.33	1.32E-01	6.92E-01
**HIGD1A**	0.29	2.80E-01	1.00E+00	-0.73	1.32E-01	6.92E-01
**SEMA6D**	0.53	2.85E-01	1.00E+00	0.84	1.32E-01	6.92E-01
**G16199**	-0.49	6.82E-01	1.00E+00	1.43	1.32E-01	6.92E-01
**NCKAP1**	0.19	3.12E-01	1.00E+00	-0.44	1.32E-01	6.92E-01
**AC021237.1**	NA	NA	NA	4.15	1.32E-01	6.92E-01
**SYNPO**	-0.14	7.86E-01	1.00E+00	1.20	1.32E-01	6.92E-01
**PRTFDC1**	0.86	7.09E-02	1.00E+00	0.63	1.32E-01	6.92E-01
**EFCAB6-AS1**	-1.73	5.30E-01	1.00E+00	-2.37	1.32E-01	6.92E-01
**DDN-AS1**	0.64	5.89E-01	1.00E+00	1.18	1.32E-01	6.92E-01
**ZNF532**	-0.02	9.47E-01	1.00E+00	0.68	1.32E-01	6.92E-01
**ZNF816**	0.04	9.15E-01	1.00E+00	-0.67	1.32E-01	6.92E-01
**HPCAL4**	-1.98	1.27E-01	1.00E+00	2.05	1.32E-01	6.92E-01
**RP11-231C14.4**	-0.14	8.61E-01	1.00E+00	0.91	1.32E-01	6.92E-01
**LINC02542**	0.35	4.74E-01	1.00E+00	-1.07	1.32E-01	6.92E-01

**LYPD6**	-0.26	6.44E-01	1.00E+00	1.23	1.32E-01	6.92E-01
**G39805**	-0.64	7.42E-01	1.00E+00	-2.39	1.32E-01	6.92E-01
**NAF1**	-0.11	5.89E-01	1.00E+00	0.39	1.32E-01	6.92E-01
**BTNL9**	0.50	4.32E-01	1.00E+00	-1.21	1.32E-01	6.92E-01
**CBY1**	0.33	2.06E-01	1.00E+00	-0.52	1.32E-01	6.92E-01
**TAOK3**	-0.04	8.31E-01	1.00E+00	0.23	1.32E-01	6.92E-01
**TAB2**	-0.07	6.72E-01	1.00E+00	0.54	1.32E-01	6.93E-01
**ACBD3**	-0.07	7.69E-01	1.00E+00	-0.49	1.32E-01	6.93E-01
**RSPH1**	-0.29	5.72E-01	1.00E+00	-0.84	1.32E-01	6.93E-01
**ZNF888**	0.08	8.20E-01	1.00E+00	0.56	1.32E-01	6.93E-01
**SST**	-17.65	9.48E-13	6.73E-10	2.65	1.32E-01	6.93E-01
**MRPL23**	-0.17	4.14E-01	1.00E+00	-0.74	1.33E-01	6.93E-01
**ATP5F1A**	-0.03	9.03E-01	1.00E+00	-0.29	1.33E-01	6.93E-01
**DTWD2**	-0.18	4.54E-01	1.00E+00	-0.60	1.33E-01	6.94E-01
**CLEC12A**	0.37	7.49E-01	1.00E+00	1.35	1.33E-01	6.94E-01
**POU3F1**	-0.47	3.02E-01	1.00E+00	-1.09	1.33E-01	6.94E-01
**ANGPT2**	0.43	4.10E-01	1.00E+00	0.86	1.33E-01	6.95E-01
**OPA3**	-0.37	7.84E-02	1.00E+00	0.47	1.33E-01	6.95E-01
**C11orf80**	0.14	6.24E-01	1.00E+00	-0.69	1.33E-01	6.95E-01
**MRNIP**	0.28	4.61E-01	1.00E+00	0.36	1.33E-01	6.95E-01
**LINC02575**	-0.13	9.47E-01	1.00E+00	2.10	1.33E-01	6.95E-01
**ZC3HAV1L**	-0.83	5.20E-02	1.00E+00	-0.54	1.33E-01	6.95E-01
**HSPB7**	1.49	5.08E-02	1.00E+00	-1.02	1.33E-01	6.95E-01
**RCCD1**	-0.29	2.99E-01	1.00E+00	-0.54	1.33E-01	6.95E-01
**AGR3**	-2.55	1.28E-01	1.00E+00	-1.71	1.33E-01	6.95E-01
**BIN2**	-0.38	4.10E-01	1.00E+00	0.94	1.33E-01	6.95E-01
**SNED1**	0.61	1.22E-01	1.00E+00	1.13	1.33E-01	6.95E-01
**AC011374.1**	0.03	9.40E-01	1.00E+00	-0.81	1.33E-01	6.95E-01
**AC096887.1**	-0.62	6.51E-01	1.00E+00	0.93	1.33E-01	6.95E-01
**CENPT**	0.05	8.38E-01	1.00E+00	0.30	1.33E-01	6.95E-01
**RCL1**	-0.13	6.75E-01	1.00E+00	-0.32	1.33E-01	6.95E-01
**TMA7**	0.33	2.39E-01	1.00E+00	-0.95	1.33E-01	6.95E-01

**MINOS1**	0.15	7.11E-01	1.00E+00	-0.68	1.33E-01	6.95E-01
**AC116366.1**	-0.61	5.25E-01	1.00E+00	1.05	1.33E-01	6.95E-01
**TBC1D22A**	-0.03	8.80E-01	1.00E+00	0.43	1.33E-01	6.95E-01
**PPIAP39**	0.65	2.23E-01	1.00E+00	1.05	1.33E-01	6.95E-01
**AL161729.1**	-0.06	9.86E-01	1.00E+00	-1.58	1.34E-01	6.95E-01
**HERC2**	-0.07	8.12E-01	1.00E+00	0.52	1.34E-01	6.95E-01
**CCL26**	-0.57	3.91E-01	1.00E+00	0.67	1.34E-01	6.95E-01
**B9D2**	-0.07	7.86E-01	1.00E+00	0.37	1.34E-01	6.95E-01
**AC018553.1**	-0.20	5.80E-01	1.00E+00	-0.71	1.34E-01	6.95E-01
**CST7**	-0.16	8.00E-01	1.00E+00	0.78	1.34E-01	6.95E-01
**ATXN7L3**	-0.29	2.47E-01	1.00E+00	1.08	1.34E-01	6.95E-01
**NDUFB5**	0.07	7.71E-01	1.00E+00	-0.43	1.34E-01	6.95E-01
**AC009121.2**	-0.98	6.80E-01	1.00E+00	1.89	1.34E-01	6.95E-01
**CHMP2B**	0.06	8.39E-01	1.00E+00	-0.38	1.34E-01	6.95E-01
**AC006441.4**	-0.44	4.78E-01	1.00E+00	-1.11	1.34E-01	6.95E-01
**AC136475.1**	0.19	6.73E-01	1.00E+00	0.80	1.34E-01	6.95E-01
**TM7SF2**	0.09	8.21E-01	1.00E+00	-0.43	1.34E-01	6.95E-01
**AL391684.1**	2.22	1.48E-01	1.00E+00	-1.35	1.34E-01	6.95E-01
**FAM180A**	1.00	1.37E-02	7.37E-01	0.80	1.34E-01	6.95E-01
**HMGA1**	-0.42	2.01E-01	1.00E+00	0.55	1.34E-01	6.95E-01
**RAB22A**	0.15	4.40E-01	1.00E+00	-0.34	1.34E-01	6.95E-01
**INSRR**	0.56	8.71E-01	1.00E+00	2.52	1.34E-01	6.95E-01
**IGHV3-53**	4.84	1.62E-02	7.96E-01	2.66	1.34E-01	6.95E-01
**SNCB**	-4.83	5.10E-06	1.64E-03	1.67	1.34E-01	6.95E-01
**PRPF39**	0.26	4.31E-01	1.00E+00	-0.38	1.34E-01	6.95E-01
**FAM66B**	-1.32	3.04E-03	2.89E-01	-1.76	1.34E-01	6.95E-01
**RPL7P18**	2.54	1.31E-01	1.00E+00	2.05	1.34E-01	6.95E-01
**BLACAT1**	-3.06	8.04E-02	1.00E+00	2.50	1.34E-01	6.95E-01
**BMS1P9**	-1.47	3.24E-01	1.00E+00	-0.75	1.34E-01	6.95E-01
**MRPS16**	-0.07	6.90E-01	1.00E+00	-0.25	1.34E-01	6.95E-01
**SSR2**	-0.02	8.97E-01	1.00E+00	0.22	1.34E-01	6.95E-01
**AIP**	0.17	4.60E-01	1.00E+00	0.42	1.34E-01	6.95E-01

**AC009123.1**	0.56	4.33E-01	1.00E+00	-1.36	1.34E-01	6.95E-01
**PLEK**	0.14	8.20E-01	1.00E+00	1.04	1.34E-01	6.95E-01
**AL356747.1**	-2.53	4.55E-01	1.00E+00	2.06	1.34E-01	6.95E-01
**SLC25A33**	0.33	5.94E-01	1.00E+00	-1.08	1.35E-01	6.95E-01
**STX8**	-0.03	8.31E-01	1.00E+00	-0.50	1.35E-01	6.95E-01
**CES5A**	-1.52	1.02E-01	1.00E+00	-1.34	1.35E-01	6.95E-01
**LHB**	-3.26	5.93E-02	1.00E+00	1.83	1.35E-01	6.95E-01
**SCGB2A1**	-0.32	7.51E-01	1.00E+00	-1.50	1.35E-01	6.95E-01
**OBSCN-AS1**	-0.26	8.31E-01	1.00E+00	1.19	1.35E-01	6.96E-01
**AC079465.1**	0.40	6.67E-01	1.00E+00	-1.74	1.35E-01	6.96E-01
**UCHL5**	0.01	9.79E-01	1.00E+00	-0.47	1.35E-01	6.96E-01
**WWTR1-IT1**	-1.28	4.21E-01	1.00E+00	1.75	1.35E-01	6.96E-01
**HSPE1P3**	NA	NA	NA	-1.06	1.35E-01	6.96E-01
**LINC01770**	0.35	5.55E-01	1.00E+00	-0.76	1.35E-01	6.96E-01
**OGFRL1**	-0.05	8.57E-01	1.00E+00	-0.58	1.35E-01	6.96E-01
**ZNF419**	0.17	4.99E-01	1.00E+00	0.32	1.35E-01	6.96E-01
**XLOC_004803**	-0.67	8.96E-02	1.00E+00	0.91	1.35E-01	6.96E-01
**DNTTIP1**	-0.10	6.92E-01	1.00E+00	-0.75	1.35E-01	6.96E-01
**AC079610.2**	-0.01	9.91E-01	1.00E+00	-1.10	1.35E-01	6.96E-01
**PLEKHG4B**	0.10	8.88E-01	1.00E+00	1.12	1.35E-01	6.96E-01
**SESN2**	0.09	6.83E-01	1.00E+00	0.71	1.35E-01	6.96E-01
**RPL8**	0.02	9.40E-01	1.00E+00	0.39	1.35E-01	6.96E-01
**CLCN3P1**	0.95	2.29E-01	1.00E+00	-1.03	1.35E-01	6.96E-01
**NKAIN2**	-1.31	6.39E-02	1.00E+00	1.60	1.35E-01	6.96E-01
**SYDE1**	0.19	6.21E-01	1.00E+00	1.08	1.35E-01	6.96E-01
**LANCL3**	0.26	5.27E-01	1.00E+00	0.71	1.35E-01	6.96E-01
**DUX4L50**	0.44	5.51E-01	1.00E+00	0.62	1.35E-01	6.96E-01
**AC068533.2**	-0.13	8.89E-01	1.00E+00	-0.92	1.35E-01	6.96E-01
**CSTF3**	0.10	6.34E-01	1.00E+00	-0.32	1.35E-01	6.96E-01
**SEC14L2**	-0.56	1.37E-01	1.00E+00	0.66	1.35E-01	6.96E-01
**ACKR4**	0.57	1.43E-01	1.00E+00	0.52	1.35E-01	6.96E-01
**SERPINB1**	-0.12	5.19E-01	1.00E+00	0.47	1.35E-01	6.96E-01

**PSMG4**	-0.38	2.38E-01	1.00E+00	-0.36	1.35E-01	6.96E-01
**TAMM41**	-0.12	6.61E-01	1.00E+00	-0.37	1.35E-01	6.96E-01
**NF1**	0.08	7.11E-01	1.00E+00	0.56	1.36E-01	6.96E-01
**CPEB2**	-0.25	5.29E-01	1.00E+00	-0.43	1.36E-01	6.96E-01
**AC016245.1**	-1.83	5.29E-01	1.00E+00	2.52	1.36E-01	6.96E-01
**AC091965.4**	-1.78	4.86E-01	1.00E+00	-1.74	1.36E-01	6.96E-01
**AL035461.2**	3.64	1.34E-01	1.00E+00	0.99	1.36E-01	6.96E-01
**RBP4**	4.42	5.88E-03	4.57E-01	-1.38	1.36E-01	6.96E-01
**BLM**	-0.48	3.29E-01	1.00E+00	0.85	1.36E-01	6.96E-01
**G25594**	-0.12	8.26E-01	1.00E+00	-1.42	1.36E-01	6.96E-01
**IL26**	0.90	7.96E-01	1.00E+00	3.51	1.36E-01	6.96E-01
**AC010894.2**	-0.03	9.73E-01	1.00E+00	-1.11	1.36E-01	6.96E-01
**CEACAM3**	-3.24	3.38E-01	1.00E+00	1.82	1.36E-01	6.97E-01
**DNAH10OS**	-0.58	4.28E-01	1.00E+00	-0.93	1.36E-01	6.97E-01
**DDIAS**	-0.10	8.04E-01	1.00E+00	-0.57	1.36E-01	6.97E-01
**AP001189.1**	-0.99	1.72E-01	1.00E+00	1.83	1.36E-01	6.97E-01
**SFXN5**	-0.50	5.48E-02	1.00E+00	0.60	1.36E-01	6.97E-01
**ATP8B2**	0.59	1.59E-01	1.00E+00	1.28	1.36E-01	6.97E-01
**ZNF579**	-0.26	4.18E-01	1.00E+00	1.22	1.36E-01	6.97E-01
**ZNF737**	-0.03	9.41E-01	1.00E+00	-0.65	1.36E-01	6.97E-01
**PRR22**	-0.46	6.44E-01	1.00E+00	1.14	1.36E-01	6.97E-01
**ZNF664**	0.20	6.07E-01	1.00E+00	0.63	1.36E-01	6.97E-01
**PCBD1**	-0.01	9.86E-01	1.00E+00	-0.31	1.36E-01	6.97E-01
**UTP23**	0.19	3.85E-01	1.00E+00	-0.24	1.36E-01	6.97E-01
**MTCH1**	0.19	1.64E-01	1.00E+00	-0.65	1.36E-01	6.97E-01
**LYRM9**	0.44	2.19E-01	1.00E+00	-0.49	1.37E-01	6.97E-01
**AC092490.1**	3.00	1.20E-01	1.00E+00	1.33	1.37E-01	6.97E-01
**NCBP2L**	-3.14	3.87E-02	1.00E+00	-2.01	1.37E-01	6.97E-01
**SSR3**	0.06	7.51E-01	1.00E+00	-0.25	1.37E-01	6.97E-01
**DDOST**	-0.05	7.51E-01	1.00E+00	0.22	1.37E-01	6.97E-01
**AC087482.1**	2.65	9.84E-02	1.00E+00	-1.53	1.37E-01	6.97E-01
**AL353150.1**	-1.78	1.96E-01	1.00E+00	1.07	1.37E-01	6.97E-01

**ABCG8**	-0.91	6.30E-01	1.00E+00	1.92	1.37E-01	6.97E-01
**DCBLD1**	0.28	5.32E-01	1.00E+00	0.88	1.37E-01	6.97E-01
**AC099063.4**	-0.29	8.56E-01	1.00E+00	1.38	1.37E-01	6.97E-01
**RNU4ATAC**	6.34	3.90E-04	8.19E-02	-1.73	1.37E-01	6.97E-01
**TSPAN4**	0.50	1.07E-01	1.00E+00	0.48	1.37E-01	6.97E-01
**CICP14**	-0.31	6.82E-01	1.00E+00	1.82	1.37E-01	6.97E-01
**MYOT**	-0.08	9.09E-01	1.00E+00	-0.69	1.37E-01	6.97E-01
**SHISAL2A**	-1.79	5.39E-01	1.00E+00	1.96	1.37E-01	6.97E-01
**AC008764.10**	0.32	9.28E-01	1.00E+00	1.66	1.37E-01	6.97E-01
**AL358332.1**	0.03	9.75E-01	1.00E+00	-1.19	1.37E-01	6.97E-01
**BLZF1**	-0.03	8.88E-01	1.00E+00	-0.40	1.37E-01	6.97E-01
**GOLGA7B**	-0.55	4.67E-02	1.00E+00	0.88	1.37E-01	6.97E-01
**VPS8**	-0.08	6.83E-01	1.00E+00	0.30	1.37E-01	6.97E-01
**AC005726.3**	-0.16	9.15E-01	1.00E+00	1.19	1.37E-01	6.97E-01
**SH3YL1**	-0.31	2.50E-01	1.00E+00	-0.47	1.37E-01	6.97E-01
**ATP11A**	-0.24	2.16E-01	1.00E+00	0.80	1.37E-01	6.97E-01
**ZNF271P**	0.04	8.94E-01	1.00E+00	-0.49	1.37E-01	6.97E-01
**MELTF-AS1**	0.74	5.40E-01	1.00E+00	-1.14	1.37E-01	6.97E-01
**AC018521.5**	0.08	8.74E-01	1.00E+00	-0.57	1.37E-01	6.97E-01
**CLIP2**	-0.22	4.84E-01	1.00E+00	0.87	1.37E-01	6.97E-01
**PPIEL**	0.27	7.33E-01	1.00E+00	0.75	1.37E-01	6.97E-01
**AC135983.2**	-0.17	7.74E-01	1.00E+00	-0.65	1.37E-01	6.97E-01
**TENM2**	-0.95	7.05E-02	1.00E+00	0.94	1.37E-01	6.97E-01
**METTL18**	0.46	1.06E-01	1.00E+00	-0.65	1.37E-01	6.97E-01
**GOLT1B**	0.40	1.35E-01	1.00E+00	-0.46	1.37E-01	6.97E-01
**DNAJC28**	0.36	3.27E-01	1.00E+00	-0.67	1.37E-01	6.97E-01
**AC011365.1**	-0.45	8.46E-01	1.00E+00	-1.78	1.37E-01	6.97E-01
**S100PBP**	-0.05	8.72E-01	1.00E+00	0.44	1.37E-01	6.97E-01
**ZNF701**	-0.40	2.33E-01	1.00E+00	-0.60	1.37E-01	6.97E-01
**RERE**	-0.06	8.54E-01	1.00E+00	0.73	1.37E-01	6.97E-01
**CHST1**	-0.46	5.26E-01	1.00E+00	1.41	1.37E-01	6.97E-01
**AC098614.4**	-2.94	1.78E-01	1.00E+00	-1.88	1.37E-01	6.97E-01

**NLK**	-0.21	3.89E-01	1.00E+00	0.77	1.38E-01	6.97E-01
**MRTFA**	-0.38	9.95E-02	1.00E+00	0.71	1.38E-01	6.97E-01
**STK24P1**	NA	NA	NA	-1.30	1.38E-01	6.97E-01
**PPIAP40**	0.54	8.30E-01	1.00E+00	-1.17	1.38E-01	6.97E-01
**SCARB2**	0.13	5.59E-01	1.00E+00	0.45	1.38E-01	6.97E-01
**MACROD2**	-0.42	1.71E-01	1.00E+00	-0.67	1.38E-01	6.97E-01
**MYO3B**	0.60	3.41E-01	1.00E+00	1.14	1.38E-01	6.97E-01
**SGK2**	2.47	1.27E-01	1.00E+00	-1.35	1.38E-01	6.97E-01
**GDF5**	0.55	7.39E-01	1.00E+00	1.51	1.38E-01	6.97E-01
**CNP**	-0.37	2.66E-01	1.00E+00	0.48	1.38E-01	6.97E-01
**TEP1**	-0.36	2.11E-01	1.00E+00	0.91	1.38E-01	6.97E-01
**AC064807.2**	-1.05	2.03E-01	1.00E+00	-1.18	1.38E-01	6.97E-01
**ANKAR**	-0.21	6.48E-01	1.00E+00	-0.83	1.38E-01	6.97E-01
**UBE2K**	-0.01	9.80E-01	1.00E+00	0.22	1.38E-01	6.97E-01
**CAPZA2**	0.16	5.41E-01	1.00E+00	-0.51	1.38E-01	6.97E-01
**ZNF578**	0.30	7.87E-01	1.00E+00	-1.11	1.38E-01	6.97E-01
**AC097658.1**	-0.19	7.50E-01	1.00E+00	0.67	1.38E-01	6.97E-01
**AL353719.1**	-2.66	9.13E-02	1.00E+00	-1.35	1.38E-01	6.97E-01
**PTGER3**	0.63	1.10E-01	1.00E+00	-0.60	1.38E-01	6.97E-01
**AL132780.5**	0.02	9.96E-01	1.00E+00	-2.52	1.38E-01	6.97E-01
**LINC02585**	0.91	6.06E-01	1.00E+00	1.44	1.38E-01	6.97E-01
**IGLON5**	-0.93	2.04E-01	1.00E+00	1.54	1.38E-01	6.97E-01
**TDRKH**	-0.31	2.96E-01	1.00E+00	-0.42	1.38E-01	6.97E-01
**TMEM138**	0.01	9.65E-01	1.00E+00	-0.40	1.38E-01	6.97E-01
**BDKRB1**	0.37	4.62E-01	1.00E+00	0.84	1.38E-01	6.97E-01
**ADPRM**	-0.04	8.81E-01	1.00E+00	-0.29	1.38E-01	6.97E-01
**XLOC_008043**	0.37	9.12E-01	1.00E+00	-2.48	1.38E-01	6.97E-01
**AP006621.3**	-0.29	4.31E-01	1.00E+00	-0.54	1.38E-01	6.97E-01
**COX8A**	-0.15	4.59E-01	1.00E+00	-0.68	1.38E-01	6.97E-01
**AC015871.3**	-0.32	4.02E-01	1.00E+00	-0.82	1.38E-01	6.97E-01
**CAB39L**	0.27	3.24E-01	1.00E+00	-0.50	1.38E-01	6.97E-01
**CMTM8**	0.46	9.41E-02	1.00E+00	-0.93	1.38E-01	6.98E-01

**NPY1R**	0.97	1.35E-01	1.00E+00	-0.97	1.39E-01	6.98E-01
**DYSF**	-0.45	3.47E-01	1.00E+00	0.96	1.39E-01	6.98E-01
**IL20RA**	0.49	1.39E-01	1.00E+00	-0.77	1.39E-01	6.98E-01
**PCDH12**	0.55	3.97E-01	1.00E+00	0.91	1.39E-01	6.98E-01
**BICRA**	-0.61	7.60E-02	1.00E+00	1.10	1.39E-01	6.98E-01
**ALG1**	0.01	9.63E-01	1.00E+00	0.31	1.39E-01	6.98E-01
**RRH**	-1.05	6.00E-01	1.00E+00	-2.83	1.39E-01	6.98E-01
**PHKB**	0.26	4.08E-01	1.00E+00	-0.31	1.39E-01	6.98E-01
**AL158163.2**	NA	NA	NA	-1.49	1.39E-01	6.98E-01
**TMEM100**	1.26	4.38E-03	3.74E-01	0.86	1.39E-01	6.98E-01
**RTL10**	0.10	6.96E-01	1.00E+00	0.36	1.39E-01	6.98E-01
**H3F3AP4**	0.01	9.87E-01	1.00E+00	-0.59	1.39E-01	6.98E-01
**TMEM181**	0.18	5.67E-01	1.00E+00	-0.44	1.39E-01	6.98E-01
**RTL6**	0.37	2.15E-01	1.00E+00	0.82	1.39E-01	6.98E-01
**PDAP1**	-0.14	4.91E-01	1.00E+00	0.38	1.39E-01	6.98E-01
**C15orf39**	-0.46	4.48E-02	1.00E+00	0.90	1.39E-01	6.98E-01
**WNK2**	-0.67	1.39E-02	7.46E-01	-1.04	1.39E-01	6.98E-01
**RHPN1-AS1**	-0.14	7.94E-01	1.00E+00	1.15	1.39E-01	6.98E-01
**AC004846.1**	0.98	9.26E-02	1.00E+00	1.12	1.39E-01	6.98E-01
**ANKRD65**	0.13	8.35E-01	1.00E+00	-0.71	1.39E-01	6.98E-01
**ABHD2**	0.08	7.65E-01	1.00E+00	0.52	1.39E-01	6.98E-01
**GBP1**	-0.92	6.91E-02	1.00E+00	0.89	1.39E-01	6.98E-01
**HIKESHI**	-0.17	4.58E-01	1.00E+00	-0.53	1.39E-01	6.98E-01
**AC020658.6**	0.34	8.74E-01	1.00E+00	2.41	1.39E-01	6.98E-01
**AC018638.1**	-3.26	8.51E-02	1.00E+00	1.98	1.39E-01	6.98E-01
**FAM110B**	-0.03	9.40E-01	1.00E+00	0.59	1.39E-01	6.99E-01
**ITGB6**	0.24	5.35E-01	1.00E+00	0.87	1.40E-01	6.99E-01
**AL034374.1**	2.02	2.33E-01	1.00E+00	-1.68	1.40E-01	6.99E-01
**TAX1BP1**	-0.07	7.52E-01	1.00E+00	-0.36	1.40E-01	7.00E-01
**SFXN3**	-0.03	9.23E-01	1.00E+00	0.33	1.40E-01	7.00E-01
**HTR7**	-0.71	1.22E-01	1.00E+00	1.37	1.40E-01	7.00E-01
**NCAPH2**	-0.19	2.64E-01	1.00E+00	0.23	1.40E-01	7.01E-01

**AC112236.1**	-0.40	7.78E-01	1.00E+00	1.37	1.40E-01	7.01E-01
**AL162596.1**	0.81	3.86E-01	1.00E+00	1.03	1.40E-01	7.01E-01
**AC073896.2**	0.97	4.07E-01	1.00E+00	-1.20	1.40E-01	7.01E-01
**EXD3**	-0.06	7.92E-01	1.00E+00	0.38	1.40E-01	7.01E-01
**ADCY10P1**	-0.01	9.85E-01	1.00E+00	0.96	1.40E-01	7.01E-01
**C6orf106**	0.11	4.56E-01	1.00E+00	0.64	1.40E-01	7.01E-01
**PSMC4**	-0.18	4.07E-01	1.00E+00	0.25	1.40E-01	7.01E-01
**CD79B**	0.78	1.03E-01	1.00E+00	0.75	1.40E-01	7.01E-01
**C3orf49**	1.15	2.64E-01	1.00E+00	-1.16	1.40E-01	7.01E-01
**ZNF423**	0.18	7.04E-01	1.00E+00	0.65	1.40E-01	7.01E-01
**AKR1B1**	0.03	9.30E-01	1.00E+00	0.44	1.40E-01	7.01E-01
**AC073869.1**	0.15	7.48E-01	1.00E+00	0.64	1.40E-01	7.01E-01
**UXT**	-0.04	8.64E-01	1.00E+00	-0.42	1.41E-01	7.01E-01
**TYMSOS**	-1.78	4.12E-01	1.00E+00	0.75	1.41E-01	7.01E-01
**PMS2**	0.24	3.45E-01	1.00E+00	-0.29	1.41E-01	7.01E-01
**EXO5**	-0.14	6.73E-01	1.00E+00	0.44	1.41E-01	7.01E-01
**PLBD2**	-0.03	9.16E-01	1.00E+00	0.78	1.41E-01	7.01E-01
**ANGPTL6**	-1.17	3.08E-01	1.00E+00	1.20	1.41E-01	7.01E-01
**MINDY3**	0.27	2.15E-01	1.00E+00	-0.36	1.41E-01	7.01E-01
**SC5D**	-0.05	8.22E-01	1.00E+00	-0.51	1.41E-01	7.01E-01
**RASAL2**	-0.39	1.35E-01	1.00E+00	0.39	1.41E-01	7.01E-01
**ATG4C**	0.12	7.25E-01	1.00E+00	-0.46	1.41E-01	7.01E-01
**ANXA7**	0.04	7.89E-01	1.00E+00	-0.29	1.41E-01	7.01E-01
**ANO7**	-0.45	4.81E-01	1.00E+00	0.89	1.41E-01	7.01E-01
**ZNF571-AS1**	-0.31	6.69E-01	1.00E+00	-1.17	1.41E-01	7.01E-01
**AIPL1**	-3.59	2.87E-01	1.00E+00	2.56	1.41E-01	7.01E-01
**MANBA**	0.27	3.53E-01	1.00E+00	0.53	1.41E-01	7.01E-01
**ADGRV1**	-0.52	2.42E-01	1.00E+00	-0.90	1.41E-01	7.01E-01
**PIEZO1**	-0.03	8.82E-01	1.00E+00	0.87	1.41E-01	7.01E-01
**NOP14-AS1**	0.18	4.32E-01	1.00E+00	0.27	1.41E-01	7.01E-01
**APOE**	-0.05	8.98E-01	1.00E+00	1.31	1.41E-01	7.01E-01
**MKRN2OS**	-0.32	5.00E-01	1.00E+00	-0.75	1.41E-01	7.01E-01

**CA5BP1**	-0.02	9.39E-01	1.00E+00	0.32	1.41E-01	7.01E-01
**LENG8**	-0.28	3.48E-01	1.00E+00	1.01	1.41E-01	7.01E-01
**TIMM8A**	0.12	6.28E-01	1.00E+00	-0.55	1.41E-01	7.01E-01
**CRYBB2**	-0.29	8.69E-01	1.00E+00	1.91	1.41E-01	7.01E-01
**UBALD1**	-0.26	3.55E-01	1.00E+00	0.70	1.41E-01	7.01E-01
**TMEM56**	1.31	2.06E-02	8.94E-01	-1.01	1.41E-01	7.01E-01
**VPS9D1-AS1**	-1.16	1.35E-01	1.00E+00	1.16	1.41E-01	7.01E-01
**KCNK6**	-0.09	8.02E-01	1.00E+00	0.70	1.41E-01	7.01E-01
**AP001273.1**	1.10	2.24E-01	1.00E+00	-1.86	1.41E-01	7.01E-01
**ZNF542P**	0.33	3.87E-01	1.00E+00	-0.56	1.41E-01	7.01E-01
**RTN3**	0.05	7.97E-01	1.00E+00	-0.42	1.41E-01	7.01E-01
**CAPG**	0.02	9.42E-01	1.00E+00	0.45	1.41E-01	7.01E-01
**CDKN2B**	0.21	5.69E-01	1.00E+00	0.38	1.41E-01	7.01E-01
**LRRC2-AS1**	0.41	9.04E-01	1.00E+00	-3.32	1.42E-01	7.01E-01
**FAM118A**	-0.06	8.20E-01	1.00E+00	0.40	1.42E-01	7.01E-01
**AL021392.1**	-0.81	2.15E-02	8.94E-01	-1.15	1.42E-01	7.01E-01
**DNAJC19**	-0.07	7.23E-01	1.00E+00	-0.48	1.42E-01	7.01E-01
**ALK**	0.55	5.99E-01	1.00E+00	1.83	1.42E-01	7.01E-01
**PHACTR1**	-0.25	4.48E-01	1.00E+00	0.44	1.42E-01	7.01E-01
**OLR1**	-3.74	1.36E-01	1.00E+00	2.49	1.42E-01	7.01E-01
**ZNF852**	-0.11	6.93E-01	1.00E+00	-0.56	1.42E-01	7.01E-01
**CRIM1**	0.16	6.86E-01	1.00E+00	0.55	1.42E-01	7.01E-01
**PPP4R3B**	0.20	4.53E-01	1.00E+00	-0.23	1.42E-01	7.01E-01
**INSYN2**	-3.05	2.19E-02	8.96E-01	-1.05	1.42E-01	7.01E-01
**PPP1R37**	-0.18	5.51E-01	1.00E+00	1.07	1.42E-01	7.01E-01
**RNU7-40P**	2.04	1.44E-01	1.00E+00	1.57	1.42E-01	7.01E-01
**OR7E28P**	0.32	7.59E-01	1.00E+00	-1.14	1.42E-01	7.01E-01
**ETV3L**	-0.33	9.23E-01	1.00E+00	2.85	1.42E-01	7.01E-01
**PDE4B**	-0.41	3.69E-01	1.00E+00	0.52	1.42E-01	7.01E-01
**STK4**	0.19	3.46E-01	1.00E+00	0.31	1.42E-01	7.01E-01
**DMBT1**	-3.84	8.73E-03	5.76E-01	-2.33	1.42E-01	7.01E-01
**COX7C**	0.12	4.85E-01	1.00E+00	-0.48	1.42E-01	7.01E-01

**OR7E47P**	-0.47	4.26E-01	1.00E+00	-1.01	1.42E-01	7.01E-01
**SBDSP1**	-0.09	6.84E-01	1.00E+00	-0.42	1.42E-01	7.01E-01
**ERF**	-0.56	2.39E-02	9.18E-01	0.94	1.42E-01	7.01E-01
**ALG1L10P**	-1.53	2.39E-01	1.00E+00	-1.17	1.42E-01	7.01E-01
**PLEKHA1**	0.33	3.05E-01	1.00E+00	-0.49	1.42E-01	7.01E-01
**B3GNT8**	0.06	8.53E-01	1.00E+00	0.41	1.42E-01	7.01E-01
**CTIF**	-0.03	9.38E-01	1.00E+00	0.78	1.42E-01	7.01E-01
**SOX21-AS1**	-0.35	4.57E-01	1.00E+00	-0.93	1.42E-01	7.01E-01
**C6orf89**	0.15	4.31E-01	1.00E+00	0.32	1.42E-01	7.02E-01
**THAP6**	0.52	1.05E-01	1.00E+00	-0.36	1.42E-01	7.02E-01
**PHACTR3**	0.13	8.31E-01	1.00E+00	-1.03	1.42E-01	7.02E-01
**RNF139**	0.00	9.96E-01	1.00E+00	-0.39	1.42E-01	7.02E-01
**EIF2B3**	0.06	7.88E-01	1.00E+00	-0.43	1.42E-01	7.02E-01
**AL022724.1**	-0.10	9.52E-01	1.00E+00	1.73	1.42E-01	7.02E-01
**AL592429.1**	0.31	8.08E-01	1.00E+00	1.66	1.42E-01	7.02E-01
**MAPK1**	0.14	4.94E-01	1.00E+00	-0.51	1.42E-01	7.02E-01
**G41001**	-2.70	1.87E-01	1.00E+00	1.24	1.42E-01	7.02E-01
**DNASE1L3**	-0.47	1.27E-01	1.00E+00	0.61	1.43E-01	7.02E-01
**MIR497HG**	0.89	5.73E-01	1.00E+00	1.10	1.43E-01	7.02E-01
**LMO7**	0.19	4.59E-01	1.00E+00	0.53	1.43E-01	7.02E-01
**VCAM1**	0.33	4.69E-01	1.00E+00	0.90	1.43E-01	7.02E-01
**VPS4A**	-0.01	9.52E-01	1.00E+00	-0.34	1.43E-01	7.02E-01
**MAST1**	-0.35	3.06E-01	1.00E+00	-0.85	1.43E-01	7.02E-01
**PPP1R42**	-0.46	8.58E-01	1.00E+00	3.08	1.43E-01	7.02E-01
**AL161725.1**	-0.08	9.62E-01	1.00E+00	-1.39	1.43E-01	7.02E-01
**AC003102.1**	-0.06	9.01E-01	1.00E+00	0.64	1.43E-01	7.02E-01
**SPPL3**	-0.17	5.85E-01	1.00E+00	-0.59	1.43E-01	7.02E-01
**SMAD3**	0.13	5.56E-01	1.00E+00	0.56	1.43E-01	7.02E-01
**DLEU2L**	1.52	6.59E-01	1.00E+00	-2.10	1.43E-01	7.02E-01
**ASS1**	-0.16	5.10E-01	1.00E+00	-0.42	1.43E-01	7.02E-01
**AL359762.3**	-0.10	9.75E-01	1.00E+00	1.72	1.43E-01	7.02E-01
**AC026904.3**	-1.38	3.65E-01	1.00E+00	-1.59	1.43E-01	7.02E-01

**G25250**	0.07	9.44E-01	1.00E+00	1.45	1.43E-01	7.02E-01
**PABPC1L**	-1.79	6.56E-03	4.90E-01	1.33	1.43E-01	7.02E-01
**UNC13A**	-1.84	5.55E-01	1.00E+00	2.30	1.43E-01	7.02E-01
**LGI2**	0.06	9.27E-01	1.00E+00	1.24	1.43E-01	7.02E-01
**XCL1**	-4.84	1.04E-03	1.67E-01	1.34	1.43E-01	7.02E-01
**SIX4**	-0.17	8.80E-01	1.00E+00	1.49	1.43E-01	7.02E-01
**AL122125.1**	-3.47	3.03E-01	1.00E+00	2.06	1.43E-01	7.02E-01
**AC005332.1**	0.23	9.21E-01	1.00E+00	1.71	1.43E-01	7.02E-01
**MYH13**	-1.93	4.14E-01	1.00E+00	2.10	1.43E-01	7.02E-01
**INCA1**	-0.59	2.36E-01	1.00E+00	0.55	1.43E-01	7.02E-01
**GPX4**	0.30	5.38E-01	1.00E+00	-0.54	1.43E-01	7.02E-01
**LILRA1**	-1.06	2.13E-01	1.00E+00	1.84	1.43E-01	7.02E-01
**MAN2B2**	-0.06	8.31E-01	1.00E+00	0.54	1.43E-01	7.02E-01
**GOLGA2**	-0.12	6.03E-01	1.00E+00	0.58	1.43E-01	7.02E-01
**PIGK**	0.13	6.06E-01	1.00E+00	-0.49	1.43E-01	7.02E-01
**PTGER4**	0.34	2.93E-01	1.00E+00	0.69	1.43E-01	7.02E-01
**AL731577.2**	-0.23	6.61E-01	1.00E+00	1.09	1.44E-01	7.02E-01
**HIST1H2BF**	-3.23	1.57E-02	7.82E-01	-1.47	1.44E-01	7.03E-01
**IDNK**	0.18	5.16E-01	1.00E+00	-0.52	1.44E-01	7.03E-01
**REC8**	-0.61	3.03E-01	1.00E+00	1.09	1.44E-01	7.03E-01
**AC127024.8**	0.90	7.96E-01	1.00E+00	-1.72	1.44E-01	7.03E-01
**ABCA13**	-1.00	7.44E-02	1.00E+00	-1.11	1.44E-01	7.03E-01
**WDR60**	0.15	5.65E-01	1.00E+00	0.47	1.44E-01	7.03E-01
**VPS39**	0.13	4.03E-01	1.00E+00	0.38	1.44E-01	7.03E-01
**LRPAP1**	-0.13	5.86E-01	1.00E+00	0.31	1.44E-01	7.03E-01
**FAM19A2**	-0.19	7.15E-01	1.00E+00	-0.68	1.44E-01	7.03E-01
**HNRNPLL**	0.00	9.96E-01	1.00E+00	-0.53	1.44E-01	7.03E-01
**AL358813.2**	0.10	8.87E-01	1.00E+00	1.44	1.44E-01	7.03E-01
**GAPLINC**	-0.92	6.13E-01	1.00E+00	1.13	1.44E-01	7.03E-01
**AC078795.1**	0.76	8.20E-01	1.00E+00	1.89	1.44E-01	7.03E-01
**ETFA**	0.12	5.64E-01	1.00E+00	-0.36	1.44E-01	7.03E-01
**AC114763.1**	-2.02	7.45E-02	1.00E+00	-0.88	1.44E-01	7.03E-01

**CDA**	0.12	7.34E-01	1.00E+00	0.58	1.44E-01	7.03E-01
**WASF4P**	-0.86	7.46E-01	1.00E+00	-0.76	1.44E-01	7.03E-01
**UHRF1BP1**	0.16	5.79E-01	1.00E+00	0.57	1.44E-01	7.03E-01
**XLOC_004201**	-1.29	7.00E-01	1.00E+00	1.77	1.44E-01	7.03E-01
**C22orf23**	-0.24	7.88E-01	1.00E+00	1.16	1.44E-01	7.03E-01
**XLOC_006645**	-0.76	3.86E-01	1.00E+00	-0.83	1.44E-01	7.03E-01
**RNF216P1**	0.16	5.38E-01	1.00E+00	0.25	1.44E-01	7.03E-01
**AC145207.5**	0.10	8.68E-01	1.00E+00	-0.91	1.44E-01	7.03E-01
**EFNB2**	-0.32	2.30E-01	1.00E+00	-0.59	1.44E-01	7.03E-01
**MECR**	0.01	9.70E-01	1.00E+00	-0.36	1.44E-01	7.03E-01
**LINC01521**	0.23	5.93E-01	1.00E+00	1.43	1.44E-01	7.03E-01
**EPN1**	-0.19	4.66E-01	1.00E+00	0.98	1.44E-01	7.03E-01
**MANSC1**	-0.09	5.58E-01	1.00E+00	-0.26	1.44E-01	7.03E-01
**TNFRSF9**	-2.08	7.49E-02	1.00E+00	1.78	1.45E-01	7.03E-01
**ADAMTSL4**	0.67	2.12E-01	1.00E+00	1.07	1.45E-01	7.03E-01
**AL358472.2**	1.24	2.11E-01	1.00E+00	-1.21	1.45E-01	7.03E-01
**CD36**	2.39	5.06E-02	1.00E+00	-1.02	1.45E-01	7.03E-01
**MTHFD2L**	0.02	9.62E-01	1.00E+00	-0.63	1.45E-01	7.03E-01
**AMER2**	-1.07	7.49E-01	1.00E+00	-2.24	1.45E-01	7.03E-01
**CHPF**	-0.31	3.00E-01	1.00E+00	0.77	1.45E-01	7.03E-01
**LSM14B**	0.01	9.64E-01	1.00E+00	1.00	1.45E-01	7.03E-01
**SOX9**	-0.06	9.27E-01	1.00E+00	-0.80	1.45E-01	7.03E-01
**HOPX**	0.49	3.74E-01	1.00E+00	-0.91	1.45E-01	7.03E-01
**ZNF720**	-0.10	7.03E-01	1.00E+00	-0.31	1.45E-01	7.03E-01
**GJB7**	-1.17	5.13E-01	1.00E+00	2.28	1.45E-01	7.03E-01
**PPIB**	-0.12	6.24E-01	1.00E+00	0.32	1.45E-01	7.03E-01
**MYLK2**	-3.68	7.20E-02	1.00E+00	2.73	1.45E-01	7.03E-01
**ZNF517**	0.17	5.18E-01	1.00E+00	0.94	1.45E-01	7.03E-01
**XLOC_007084**	-1.64	3.97E-01	1.00E+00	-2.37	1.45E-01	7.03E-01
**LCMT1-AS1**	1.79	1.10E-01	1.00E+00	1.03	1.45E-01	7.03E-01
**NACC2**	0.18	6.67E-01	1.00E+00	0.82	1.45E-01	7.03E-01
**AC092794.1**	0.59	6.73E-01	1.00E+00	1.40	1.45E-01	7.03E-01

**G16424**	0.18	9.43E-01	1.00E+00	2.29	1.45E-01	7.03E-01
**AC025183.1**	-1.11	2.38E-01	1.00E+00	-1.07	1.45E-01	7.04E-01
**IL1RAPL1**	0.15	8.87E-01	1.00E+00	1.54	1.45E-01	7.04E-01
**GRB14**	-0.01	9.93E-01	1.00E+00	-0.84	1.45E-01	7.04E-01
**XLOC_005254**	-0.59	7.39E-01	1.00E+00	-2.11	1.45E-01	7.04E-01
**C21orf62-AS1**	0.27	5.69E-01	1.00E+00	-0.79	1.45E-01	7.04E-01
**COX18**	-0.09	7.08E-01	1.00E+00	0.39	1.45E-01	7.04E-01
**FRMD3**	0.16	7.21E-01	1.00E+00	-0.58	1.45E-01	7.04E-01
**PCAT1**	-0.04	9.45E-01	1.00E+00	-0.57	1.45E-01	7.04E-01
**LSM5**	0.16	4.54E-01	1.00E+00	-0.65	1.45E-01	7.04E-01
**AC092535.3**	0.12	9.59E-01	1.00E+00	-1.60	1.46E-01	7.04E-01
**GAB1**	-0.11	6.65E-01	1.00E+00	0.46	1.46E-01	7.04E-01
**ZMYND11**	0.04	8.50E-01	1.00E+00	-0.23	1.46E-01	7.04E-01
**SLC16A4**	1.14	5.71E-02	1.00E+00	0.77	1.46E-01	7.04E-01
**NAV1**	-0.18	5.04E-01	1.00E+00	0.61	1.46E-01	7.04E-01
**TSGA10**	-0.06	8.36E-01	1.00E+00	-0.57	1.46E-01	7.04E-01
**SP8**	0.02	9.77E-01	1.00E+00	-1.45	1.46E-01	7.04E-01
**XLOC_008855**	-0.69	5.76E-01	1.00E+00	1.53	1.46E-01	7.04E-01
**NDUFAF7**	0.22	3.95E-01	1.00E+00	-0.37	1.46E-01	7.04E-01
**SFN**	-0.40	3.94E-01	1.00E+00	0.97	1.46E-01	7.04E-01
**GPR89B**	-0.28	4.74E-01	1.00E+00	-0.30	1.46E-01	7.04E-01
**UQCR11**	-0.07	7.74E-01	1.00E+00	-0.68	1.46E-01	7.04E-01
**IPPK**	-0.27	5.10E-01	1.00E+00	0.51	1.46E-01	7.04E-01
**MIXL1**	0.44	8.39E-01	1.00E+00	0.84	1.46E-01	7.04E-01
**LY96**	0.19	7.45E-01	1.00E+00	0.60	1.46E-01	7.04E-01
**CFAP45**	-1.00	7.34E-02	1.00E+00	-0.65	1.46E-01	7.04E-01
**SLC10A7**	0.14	5.84E-01	1.00E+00	0.43	1.46E-01	7.04E-01
**GIGYF1**	-0.21	3.13E-01	1.00E+00	0.79	1.46E-01	7.04E-01
**MRPL17**	-0.12	5.66E-01	1.00E+00	0.27	1.46E-01	7.04E-01
**FBXW2**	0.30	1.24E-01	1.00E+00	0.19	1.46E-01	7.04E-01
**XLOC_012338**	2.68	4.27E-01	1.00E+00	2.10	1.46E-01	7.04E-01
**HIST1H3B**	-0.59	7.51E-01	1.00E+00	-1.63	1.46E-01	7.04E-01

**SOX5**	-0.26	3.96E-01	1.00E+00	-0.55	1.46E-01	7.04E-01
**SESN1**	0.30	3.22E-01	1.00E+00	-0.50	1.46E-01	7.04E-01
**G42257**	-1.04	3.93E-01	1.00E+00	-1.69	1.46E-01	7.04E-01
**LINC02550**	0.45	8.39E-01	1.00E+00	1.89	1.46E-01	7.04E-01
**ZNF557**	-0.12	7.39E-01	1.00E+00	-0.47	1.47E-01	7.04E-01
**U62317.2**	-0.19	8.10E-01	1.00E+00	1.00	1.47E-01	7.04E-01
**MVB12B**	-0.04	8.90E-01	1.00E+00	0.89	1.47E-01	7.04E-01
**AC006504.1**	-2.07	2.10E-01	1.00E+00	-1.78	1.47E-01	7.04E-01
**AC112484.3**	0.21	8.96E-01	1.00E+00	1.90	1.47E-01	7.04E-01
**TMEM42**	0.42	1.37E-01	1.00E+00	-0.53	1.47E-01	7.04E-01
**IPMK**	0.16	6.47E-01	1.00E+00	-0.59	1.47E-01	7.04E-01
**CADM3**	0.65	1.71E-01	1.00E+00	0.98	1.47E-01	7.04E-01
**LINC00299**	2.88	2.59E-01	1.00E+00	1.80	1.47E-01	7.04E-01
**AC026250.1**	1.07	2.78E-01	1.00E+00	1.60	1.47E-01	7.04E-01
**SMIM30**	-0.04	8.65E-01	1.00E+00	-0.51	1.47E-01	7.04E-01
**SERINC2**	-0.04	9.01E-01	1.00E+00	0.53	1.47E-01	7.04E-01
**AC093599.1**	-0.02	9.96E-01	1.00E+00	2.07	1.47E-01	7.04E-01
**GDF6**	2.09	4.12E-02	1.00E+00	2.48	1.47E-01	7.04E-01
**CD300C**	-0.91	1.87E-01	1.00E+00	1.00	1.47E-01	7.04E-01
**ZZEF1**	0.15	5.79E-01	1.00E+00	0.86	1.47E-01	7.04E-01
**MMAA**	0.09	7.35E-01	1.00E+00	-0.36	1.47E-01	7.04E-01
**AC104461.1**	0.43	9.01E-01	1.00E+00	1.95	1.47E-01	7.04E-01
**RPS6KA6**	0.31	3.64E-01	1.00E+00	-0.84	1.47E-01	7.04E-01
**LINC02210**	0.22	5.09E-01	1.00E+00	-0.41	1.47E-01	7.04E-01
**PRRG2**	-0.18	6.54E-01	1.00E+00	0.63	1.47E-01	7.04E-01
**PLOD3**	-0.13	6.15E-01	1.00E+00	0.64	1.47E-01	7.04E-01
**ABCF1**	-0.15	5.15E-01	1.00E+00	0.42	1.47E-01	7.04E-01
**BCL2A1**	-0.25	7.17E-01	1.00E+00	0.92	1.47E-01	7.04E-01
**ODR4**	0.06	8.32E-01	1.00E+00	-0.48	1.47E-01	7.04E-01
**ATL2**	0.01	9.63E-01	1.00E+00	-0.35	1.47E-01	7.04E-01
**LGI1**	1.32	1.37E-01	1.00E+00	1.39	1.47E-01	7.04E-01
**PTPN23**	-0.33	1.37E-01	1.00E+00	0.86	1.47E-01	7.04E-01

**OAS3**	0.11	8.70E-01	1.00E+00	1.05	1.47E-01	7.04E-01
**P2RY11**	-0.70	2.39E-03	2.47E-01	0.64	1.47E-01	7.04E-01
**RAB3C**	-0.23	8.10E-01	1.00E+00	1.10	1.47E-01	7.04E-01
**MGAM**	-2.15	8.82E-02	1.00E+00	2.38	1.47E-01	7.04E-01
**LINC01003**	0.28	3.80E-01	1.00E+00	-1.10	1.47E-01	7.04E-01
**G27121**	1.18	2.06E-01	1.00E+00	-1.01	1.48E-01	7.04E-01
**PPP1R36**	0.34	3.91E-01	1.00E+00	-0.86	1.48E-01	7.04E-01
**FCN3**	0.42	5.61E-01	1.00E+00	1.42	1.48E-01	7.04E-01
**ACE2**	0.13	9.69E-01	1.00E+00	1.42	1.48E-01	7.04E-01
**TRBV29-1**	1.37	4.41E-01	1.00E+00	1.88	1.48E-01	7.04E-01
**OPN1SW**	0.16	7.51E-01	1.00E+00	0.56	1.48E-01	7.04E-01
**ZNF395**	0.24	4.38E-01	1.00E+00	0.42	1.48E-01	7.04E-01
**G8455**	0.59	5.13E-01	1.00E+00	2.13	1.48E-01	7.04E-01
**NPW**	-1.45	3.27E-03	3.02E-01	-0.81	1.48E-01	7.04E-01
**OR51E2**	-0.80	3.85E-01	1.00E+00	1.42	1.48E-01	7.04E-01
**TRMT11**	0.95	2.40E-02	9.18E-01	-0.52	1.48E-01	7.04E-01
**CTSO**	0.16	6.08E-01	1.00E+00	0.37	1.48E-01	7.04E-01
**PGGHG**	0.38	4.20E-01	1.00E+00	1.35	1.48E-01	7.04E-01
**SYCP2L**	-0.66	7.70E-01	1.00E+00	2.32	1.48E-01	7.04E-01
**NDUFC1**	-0.02	9.28E-01	1.00E+00	-0.68	1.48E-01	7.04E-01
**KCNE4**	-0.07	8.42E-01	1.00E+00	0.79	1.48E-01	7.04E-01
**AC093616.1**	2.12	4.84E-05	1.30E-02	0.82	1.48E-01	7.04E-01
**OCIAD1**	-0.04	7.34E-01	1.00E+00	-0.25	1.48E-01	7.04E-01
**ZNRF3-AS1**	-2.15	5.30E-01	1.00E+00	-2.10	1.48E-01	7.04E-01
**GTF2I**	-0.27	1.97E-01	1.00E+00	-0.51	1.48E-01	7.04E-01
**SPATA41**	-0.73	5.81E-01	1.00E+00	2.82	1.48E-01	7.04E-01
**TMEM220**	0.02	9.55E-01	1.00E+00	-0.60	1.48E-01	7.04E-01
**SP140**	-0.22	7.48E-01	1.00E+00	1.17	1.48E-01	7.04E-01
**LTBP1**	0.31	4.66E-01	1.00E+00	0.71	1.48E-01	7.04E-01
**STK25P1**	1.52	3.68E-01	1.00E+00	1.99	1.48E-01	7.04E-01
**GRSF1**	0.00	9.89E-01	1.00E+00	-0.36	1.48E-01	7.04E-01
**HIST2H2BE**	-0.54	2.52E-01	1.00E+00	0.50	1.48E-01	7.04E-01

**BEX4**	-0.03	8.97E-01	1.00E+00	-0.39	1.48E-01	7.04E-01
**TMEM173**	-0.17	6.47E-01	1.00E+00	0.63	1.48E-01	7.04E-01
**ACP4**	0.83	3.54E-01	1.00E+00	-1.73	1.48E-01	7.04E-01
**AC116347.1**	-0.56	8.73E-01	1.00E+00	1.11	1.48E-01	7.04E-01
**SLC25A11**	-0.08	6.52E-01	1.00E+00	-0.32	1.48E-01	7.04E-01
**PWWP2A**	-0.20	5.01E-01	1.00E+00	-0.60	1.48E-01	7.04E-01
**NLRC5**	-0.42	2.64E-01	1.00E+00	0.84	1.48E-01	7.04E-01
**FOXF2**	-0.13	7.61E-01	1.00E+00	1.18	1.48E-01	7.04E-01
**AKNAD1**	0.00	1.00E+00	1.00E+00	0.96	1.48E-01	7.04E-01
**C5orf30**	-0.05	8.79E-01	1.00E+00	-0.51	1.48E-01	7.04E-01
**AC021086.1**	1.78	2.32E-01	1.00E+00	-1.38	1.48E-01	7.04E-01
**SULT1A1**	0.83	2.69E-01	1.00E+00	0.63	1.48E-01	7.04E-01
**FAM3B**	-0.25	7.15E-01	1.00E+00	-0.99	1.48E-01	7.04E-01
**PKD1**	-0.22	4.90E-01	1.00E+00	1.04	1.49E-01	7.05E-01
**SOX12**	-0.19	4.90E-01	1.00E+00	1.02	1.49E-01	7.05E-01
**G39379**	0.19	8.38E-01	1.00E+00	-1.68	1.49E-01	7.05E-01
**P2RY10**	-0.47	6.02E-01	1.00E+00	1.17	1.49E-01	7.05E-01
**GPANK1**	-0.07	6.82E-01	1.00E+00	0.23	1.49E-01	7.05E-01
**DSCAM**	-2.86	1.09E-01	1.00E+00	1.99	1.49E-01	7.05E-01
**XLOC_004362**	-1.44	1.39E-01	1.00E+00	1.62	1.49E-01	7.05E-01
**TMEM14A**	0.22	4.68E-01	1.00E+00	-0.63	1.49E-01	7.05E-01
**TMPRSS7**	1.36	1.21E-01	1.00E+00	-1.34	1.49E-01	7.05E-01
**RP11-435B5.5**	-1.83	3.70E-01	1.00E+00	-1.01	1.49E-01	7.05E-01
**ME1**	0.32	3.76E-01	1.00E+00	-0.56	1.49E-01	7.05E-01
**UMAD1**	0.10	7.60E-01	1.00E+00	-0.45	1.49E-01	7.05E-01
**EIF3EP1**	-0.56	2.09E-01	1.00E+00	-0.64	1.49E-01	7.05E-01
**AP000438.1**	2.44	3.93E-01	1.00E+00	-1.83	1.49E-01	7.05E-01
**AK4P3**	-1.35	4.91E-01	1.00E+00	-0.82	1.49E-01	7.06E-01
**HAGH**	0.08	6.76E-01	1.00E+00	-0.28	1.49E-01	7.06E-01
**FAM185A**	0.27	2.60E-01	1.00E+00	-0.49	1.49E-01	7.06E-01
**RPS11**	-0.20	4.53E-01	1.00E+00	0.46	1.49E-01	7.06E-01
**HNRNPMP1**	-1.25	7.14E-01	1.00E+00	3.31	1.49E-01	7.06E-01

**TMUB2**	0.06	7.51E-01	1.00E+00	0.24	1.49E-01	7.06E-01
**FNBP4**	-0.14	6.10E-01	1.00E+00	0.38	1.49E-01	7.06E-01
**HNRNPA1**	-0.03	8.48E-01	1.00E+00	0.40	1.49E-01	7.06E-01
**B4GALT6**	0.08	8.50E-01	1.00E+00	-0.78	1.50E-01	7.06E-01
**SLC48A1**	0.08	6.73E-01	1.00E+00	-0.38	1.50E-01	7.06E-01
**AC074386.1**	3.11	1.16E-02	6.66E-01	-0.89	1.50E-01	7.06E-01
**STX3**	0.09	6.95E-01	1.00E+00	0.49	1.50E-01	7.06E-01
**TMEM255B**	0.41	2.30E-01	1.00E+00	0.66	1.50E-01	7.07E-01
**AC078788.1**	-1.26	7.16E-01	1.00E+00	-2.92	1.50E-01	7.07E-01
**DHCR24**	-0.05	9.00E-01	1.00E+00	-0.45	1.50E-01	7.07E-01
**EPHA2**	-0.49	2.88E-01	1.00E+00	0.79	1.50E-01	7.07E-01
**TVP23B**	0.41	1.22E-01	1.00E+00	-0.38	1.50E-01	7.07E-01
**SIRPG**	-1.65	1.48E-01	1.00E+00	1.47	1.50E-01	7.08E-01
**CCDC7**	-0.07	9.26E-01	1.00E+00	-0.62	1.50E-01	7.08E-01
**PIBF1**	-0.12	6.43E-01	1.00E+00	-0.45	1.50E-01	7.08E-01
**GPD1**	4.35	2.49E-03	2.54E-01	-1.43	1.50E-01	7.08E-01
**SPCS1**	0.00	9.73E-01	1.00E+00	-0.44	1.50E-01	7.08E-01
**MSMO1**	0.01	9.85E-01	1.00E+00	-0.67	1.50E-01	7.08E-01
**FAM83H**	-0.40	3.30E-01	1.00E+00	1.09	1.50E-01	7.08E-01
**IRF5**	-0.48	2.00E-01	1.00E+00	0.66	1.50E-01	7.08E-01
**GOLGA8R**	0.57	4.52E-01	1.00E+00	1.51	1.50E-01	7.08E-01
**G12854**	-2.82	1.84E-01	1.00E+00	2.50	1.50E-01	7.08E-01
**NMRAL1**	-0.06	7.80E-01	1.00E+00	0.27	1.50E-01	7.08E-01
**TRAF7**	-0.26	3.32E-01	1.00E+00	0.66	1.50E-01	7.08E-01
**AC136628.3**	0.48	5.18E-01	1.00E+00	1.64	1.50E-01	7.08E-01
**CAMK4**	-0.91	1.01E-01	1.00E+00	1.18	1.50E-01	7.08E-01
**GPATCH2L**	0.16	4.73E-01	1.00E+00	0.25	1.50E-01	7.08E-01
**AC124016.1**	-2.42	2.22E-01	1.00E+00	-1.12	1.51E-01	7.08E-01
**MYOF**	-0.26	2.26E-01	1.00E+00	0.55	1.51E-01	7.08E-01
**KBTBD8**	0.63	2.77E-01	1.00E+00	1.09	1.51E-01	7.08E-01
**G5142**	1.30	1.70E-01	1.00E+00	-0.92	1.51E-01	7.08E-01
**PLEKHM3**	0.06	7.88E-01	1.00E+00	0.36	1.51E-01	7.08E-01

**CX3CR1**	0.20	7.56E-01	1.00E+00	0.82	1.51E-01	7.09E-01
**BAHCC1**	-0.67	4.83E-02	1.00E+00	1.08	1.51E-01	7.09E-01
**U3**	0.46	5.89E-01	1.00E+00	-0.54	1.51E-01	7.09E-01
**EPB41L3**	-0.02	9.24E-01	1.00E+00	0.81	1.51E-01	7.09E-01
**MRPS31P5**	-0.73	7.91E-01	1.00E+00	-1.23	1.51E-01	7.09E-01
**Z97633.1**	-0.84	2.43E-01	1.00E+00	1.15	1.51E-01	7.09E-01
**CCDC171**	-0.29	3.30E-01	1.00E+00	-0.45	1.51E-01	7.09E-01
**TCTE1**	-0.10	8.99E-01	1.00E+00	1.72	1.51E-01	7.09E-01
**DYNLRB2**	-0.38	4.30E-01	1.00E+00	-0.83	1.51E-01	7.09E-01
**VPS35**	0.07	6.88E-01	1.00E+00	-0.28	1.51E-01	7.09E-01
**NEURL4**	-0.37	1.22E-01	1.00E+00	1.04	1.51E-01	7.09E-01
**GALNT2**	0.15	5.70E-01	1.00E+00	0.66	1.51E-01	7.09E-01
**ASF1A**	0.05	8.63E-01	1.00E+00	-0.66	1.51E-01	7.09E-01
**ZNF385A**	-0.41	2.52E-01	1.00E+00	0.48	1.51E-01	7.09E-01
**SPDEF**	-0.07	9.57E-01	1.00E+00	-1.17	1.51E-01	7.09E-01
**HMGB1**	-0.01	9.80E-01	1.00E+00	-0.29	1.51E-01	7.09E-01
**NAP1L1P1**	-1.33	4.77E-01	1.00E+00	-1.54	1.52E-01	7.10E-01
**NFIA**	0.40	1.95E-01	1.00E+00	-0.63	1.52E-01	7.10E-01
**ABCC1**	-0.37	2.93E-02	9.91E-01	0.79	1.52E-01	7.10E-01
**CYP2D7**	-0.54	6.20E-01	1.00E+00	1.70	1.52E-01	7.10E-01
**EPO**	0.37	7.25E-01	1.00E+00	1.90	1.52E-01	7.10E-01
**TRMT1**	-0.20	4.55E-01	1.00E+00	0.33	1.52E-01	7.11E-01
**TRIM33**	0.18	5.05E-01	1.00E+00	-0.40	1.52E-01	7.11E-01
**RB1-DT**	0.66	4.79E-01	1.00E+00	-1.47	1.52E-01	7.11E-01
**CAVIN2**	0.95	1.38E-01	1.00E+00	-0.84	1.52E-01	7.11E-01
**GCLM**	-0.09	8.08E-01	1.00E+00	-0.67	1.52E-01	7.11E-01
**TMEM140**	0.57	2.57E-01	1.00E+00	0.73	1.52E-01	7.11E-01
**CRYZL1**	0.21	2.18E-01	1.00E+00	-0.57	1.52E-01	7.11E-01
**G2580**	1.22	4.71E-01	1.00E+00	2.01	1.52E-01	7.11E-01
**PLEKHM2**	-0.33	1.77E-01	1.00E+00	0.71	1.52E-01	7.11E-01
**GSTM2**	0.61	1.63E-01	1.00E+00	-0.63	1.52E-01	7.11E-01
**AC010615.1**	-2.12	1.65E-01	1.00E+00	1.19	1.52E-01	7.11E-01

**AL096869.2**	-1.18	7.27E-01	1.00E+00	1.60	1.52E-01	7.11E-01
**G15374**	0.29	7.44E-01	1.00E+00	-1.01	1.52E-01	7.11E-01
**SH2D2A**	-1.58	1.47E-02	7.61E-01	1.42	1.52E-01	7.11E-01
**AL359198.1**	NA	NA	NA	-1.90	1.53E-01	7.11E-01
**AC090948.1**	0.38	8.45E-01	1.00E+00	1.63	1.53E-01	7.11E-01
**AP006623.1**	-0.38	6.36E-01	1.00E+00	1.51	1.53E-01	7.11E-01
**MAML1**	-0.16	4.92E-01	1.00E+00	0.63	1.53E-01	7.11E-01
**G26897**	-0.19	7.00E-01	1.00E+00	-1.12	1.53E-01	7.11E-01
**WIF1**	1.92	3.93E-04	8.21E-02	1.32	1.53E-01	7.11E-01
**BRI3BP**	0.05	8.35E-01	1.00E+00	-0.50	1.53E-01	7.11E-01
**MGC12916**	-0.58	2.99E-01	1.00E+00	1.89	1.53E-01	7.11E-01
**ACAT1**	0.30	2.15E-01	1.00E+00	-0.60	1.53E-01	7.11E-01
**CREB3L4**	0.08	8.53E-01	1.00E+00	-0.45	1.53E-01	7.11E-01
**ILKAP**	0.17	4.74E-01	1.00E+00	-0.27	1.53E-01	7.11E-01
**CDH26**	0.87	2.76E-01	1.00E+00	1.04	1.53E-01	7.11E-01
**DPP3**	-0.31	1.25E-01	1.00E+00	0.31	1.53E-01	7.11E-01
**VWCE**	0.90	1.53E-01	1.00E+00	1.70	1.53E-01	7.11E-01
**WDR1**	-0.13	3.33E-01	1.00E+00	0.42	1.53E-01	7.12E-01
**MED8**	0.06	8.21E-01	1.00E+00	0.25	1.53E-01	7.13E-01
**AL158823.1**	-0.56	8.73E-01	1.00E+00	-0.94	1.53E-01	7.13E-01
**GCFC2**	-0.10	6.94E-01	1.00E+00	-0.39	1.53E-01	7.13E-01
**ZBED6**	0.08	9.51E-01	1.00E+00	1.19	1.53E-01	7.13E-01
**GPAT3**	0.50	3.12E-01	1.00E+00	-0.57	1.53E-01	7.13E-01
**AL035446.1**	1.00	6.74E-01	1.00E+00	-1.40	1.53E-01	7.13E-01
**TRIML2**	-0.67	8.48E-01	1.00E+00	3.21	1.53E-01	7.13E-01
**AL359183.1**	0.93	4.36E-01	1.00E+00	1.33	1.53E-01	7.13E-01
**OSBPL7**	0.30	5.74E-01	1.00E+00	1.39	1.53E-01	7.13E-01
**MAP1LC3A**	0.03	9.30E-01	1.00E+00	0.36	1.54E-01	7.13E-01
**KLHL14**	-0.32	7.34E-01	1.00E+00	-1.83	1.54E-01	7.13E-01
**XLOC_007617**	-2.19	2.54E-02	9.29E-01	2.80	1.54E-01	7.13E-01
**AC009509.4**	-0.63	7.19E-01	1.00E+00	1.91	1.54E-01	7.13E-01
**MTERF3**	0.22	4.73E-01	1.00E+00	-0.47	1.54E-01	7.13E-01

**MOB3B**	0.23	2.70E-01	1.00E+00	0.32	1.54E-01	7.13E-01
**CSMD1**	0.66	4.72E-01	1.00E+00	-1.10	1.54E-01	7.13E-01
**YAE1**	0.10	5.83E-01	1.00E+00	-0.51	1.54E-01	7.13E-01
**DUSP16**	-0.02	9.20E-01	1.00E+00	0.61	1.54E-01	7.13E-01
**AL603766.1**	NA	NA	NA	-1.17	1.54E-01	7.13E-01
**TMEM168**	0.11	7.44E-01	1.00E+00	-0.57	1.54E-01	7.13E-01
**CLDND1**	0.61	3.55E-02	1.00E+00	-0.71	1.54E-01	7.14E-01
**ERLIN2**	0.07	7.30E-01	1.00E+00	0.53	1.54E-01	7.14E-01
**XLOC_005247**	0.29	3.99E-01	1.00E+00	0.78	1.54E-01	7.14E-01
**COPS2**	0.05	8.25E-01	1.00E+00	0.25	1.54E-01	7.14E-01
**METTL25**	0.10	7.56E-01	1.00E+00	-0.49	1.54E-01	7.14E-01
**RGL1**	0.18	5.62E-01	1.00E+00	0.67	1.54E-01	7.14E-01
**ELF5**	0.31	7.04E-01	1.00E+00	-0.87	1.54E-01	7.14E-01
**LINC01915**	1.14	2.93E-01	1.00E+00	1.17	1.54E-01	7.14E-01
**AC005674.1**	-1.31	4.55E-01	1.00E+00	1.75	1.54E-01	7.14E-01
**ZNF576**	-0.09	7.40E-01	1.00E+00	-0.33	1.54E-01	7.14E-01
**CLCN3**	0.15	5.33E-01	1.00E+00	0.32	1.54E-01	7.14E-01
**UPK2**	-2.94	1.67E-02	8.12E-01	-1.49	1.55E-01	7.14E-01
**PABPC1**	-0.17	5.12E-01	1.00E+00	0.46	1.55E-01	7.14E-01
**LINC00893**	0.45	7.66E-01	1.00E+00	1.07	1.55E-01	7.14E-01
**EPHA10**	-1.26	5.09E-02	1.00E+00	1.60	1.55E-01	7.14E-01
**MATR3**	-0.04	8.13E-01	1.00E+00	-0.22	1.55E-01	7.14E-01
**AC008663.1**	-0.93	7.88E-01	1.00E+00	2.27	1.55E-01	7.14E-01
**XLOC_003077**	-1.16	6.46E-01	1.00E+00	-1.64	1.55E-01	7.14E-01
**FAM155A**	-0.64	3.97E-01	1.00E+00	1.06	1.55E-01	7.14E-01
**PHF11**	0.21	5.39E-01	1.00E+00	0.35	1.55E-01	7.14E-01
**GARS**	-0.29	2.06E-01	1.00E+00	0.23	1.55E-01	7.14E-01
**SCARF2**	-0.36	4.52E-01	1.00E+00	1.28	1.55E-01	7.14E-01
**DNAJC1**	0.05	8.01E-01	1.00E+00	0.27	1.55E-01	7.15E-01
**IQSEC1**	-0.03	9.22E-01	1.00E+00	0.72	1.55E-01	7.15E-01
**CCBE1**	0.69	5.50E-02	1.00E+00	0.68	1.55E-01	7.15E-01
**GBE1**	0.39	1.65E-01	1.00E+00	-0.57	1.55E-01	7.15E-01

**HIST1H2AE**	-2.08	1.48E-03	2.09E-01	-1.04	1.55E-01	7.15E-01
**LINC00562**	-3.69	2.90E-02	9.88E-01	1.57	1.55E-01	7.15E-01
**TRAV27**	1.08	7.51E-01	1.00E+00	2.26	1.55E-01	7.15E-01
**NUDT4B**	1.14	2.67E-01	1.00E+00	-1.04	1.55E-01	7.15E-01
**EIF3FP3**	-1.92	1.47E-03	2.09E-01	-0.59	1.55E-01	7.15E-01
**AL513497.1**	-0.19	8.42E-01	1.00E+00	1.38	1.55E-01	7.15E-01
**ZNF365**	0.09	8.97E-01	1.00E+00	1.06	1.55E-01	7.15E-01
**NR1I2**	-0.39	9.09E-01	1.00E+00	2.02	1.55E-01	7.15E-01
**HAPLN3**	-1.04	3.20E-03	2.97E-01	1.04	1.55E-01	7.15E-01
**MGAT4A**	0.10	7.22E-01	1.00E+00	0.58	1.56E-01	7.16E-01
**CDHR2**	-2.97	4.03E-03	3.54E-01	1.85	1.56E-01	7.16E-01
**AL031846.2**	1.63	2.33E-01	1.00E+00	2.21	1.56E-01	7.16E-01
**CLEC12A-AS1**	-3.24	2.22E-01	1.00E+00	2.51	1.56E-01	7.16E-01
**CLCN2**	-0.70	4.12E-02	1.00E+00	-1.70	1.56E-01	7.16E-01
**SPAG16**	0.05	8.35E-01	1.00E+00	-0.62	1.56E-01	7.16E-01
**AC016582.2**	-0.28	8.84E-01	1.00E+00	-0.76	1.56E-01	7.16E-01
**AHCYL2**	-0.07	7.80E-01	1.00E+00	-0.83	1.56E-01	7.16E-01
**FDXACB1**	0.07	9.13E-01	1.00E+00	-0.70	1.56E-01	7.16E-01
**ZNF540**	0.11	9.09E-01	1.00E+00	-0.81	1.56E-01	7.16E-01
**AL591686.1**	3.33	9.19E-02	1.00E+00	-1.73	1.56E-01	7.16E-01
**DFFB**	-0.60	2.06E-01	1.00E+00	0.60	1.56E-01	7.16E-01
**KCTD3**	0.09	6.30E-01	1.00E+00	-0.57	1.56E-01	7.16E-01
**LDLRAD3**	-0.21	4.43E-01	1.00E+00	-0.33	1.56E-01	7.16E-01
**AC006213.1**	-1.77	3.75E-01	1.00E+00	-2.02	1.56E-01	7.16E-01
**AC105074.1**	1.85	1.57E-01	1.00E+00	-2.31	1.56E-01	7.16E-01
**TBP**	-0.03	9.13E-01	1.00E+00	-0.42	1.56E-01	7.16E-01
**AC087286.2**	1.58	4.68E-01	1.00E+00	-0.80	1.56E-01	7.16E-01
**XLOC_002133**	0.22	6.79E-01	1.00E+00	0.41	1.56E-01	7.16E-01
**CHST6**	-0.43	5.82E-01	1.00E+00	1.71	1.56E-01	7.16E-01
**AL022345.4**	0.59	6.91E-01	1.00E+00	-1.14	1.56E-01	7.16E-01
**XLOC_002197**	2.32	4.97E-01	1.00E+00	-1.59	1.56E-01	7.16E-01
**WASH8P**	-0.15	7.16E-01	1.00E+00	0.65	1.56E-01	7.16E-01

**DGCR6**	0.82	9.24E-02	1.00E+00	-0.60	1.56E-01	7.16E-01
**G20595**	-0.70	7.55E-01	1.00E+00	1.97	1.56E-01	7.16E-01
**LINC01137**	0.28	2.78E-01	1.00E+00	0.51	1.56E-01	7.16E-01
**AL645608.7**	-1.32	2.01E-01	1.00E+00	1.71	1.56E-01	7.16E-01
**CNKSR2**	0.36	4.97E-01	1.00E+00	-0.82	1.56E-01	7.16E-01
**MSX2**	-0.20	6.57E-01	1.00E+00	-0.92	1.56E-01	7.16E-01
**G35671**	0.10	9.28E-01	1.00E+00	-1.56	1.56E-01	7.16E-01
**ZBTB5**	0.09	7.55E-01	1.00E+00	0.54	1.56E-01	7.16E-01
**AGAP1**	-0.12	7.64E-01	1.00E+00	0.73	1.56E-01	7.16E-01
**XLOC_005972**	1.18	6.13E-01	1.00E+00	-2.07	1.56E-01	7.16E-01
**AC016405.3**	-0.88	4.80E-01	1.00E+00	-0.97	1.56E-01	7.16E-01
**ACOT2**	0.52	3.47E-01	1.00E+00	-0.59	1.56E-01	7.16E-01
**PDE7A**	0.18	5.49E-01	1.00E+00	0.68	1.57E-01	7.16E-01
**E2F3**	-0.07	8.29E-01	1.00E+00	0.84	1.57E-01	7.16E-01
**PKLR**	-0.64	5.38E-01	1.00E+00	-1.68	1.57E-01	7.16E-01
**GPR31**	-0.88	7.19E-01	1.00E+00	2.29	1.57E-01	7.16E-01
**FOXD3-AS1**	0.31	6.55E-01	1.00E+00	-0.68	1.57E-01	7.16E-01
**GEMIN8P4**	-0.32	6.27E-01	1.00E+00	0.83	1.57E-01	7.16E-01
**HERC6**	-0.27	7.12E-01	1.00E+00	0.85	1.57E-01	7.16E-01
**MEX3D**	-0.29	2.71E-01	1.00E+00	1.02	1.57E-01	7.16E-01
**MYHAS**	2.97	1.50E-01	1.00E+00	1.73	1.57E-01	7.16E-01
**KIAA1191**	0.00	9.98E-01	1.00E+00	-0.24	1.57E-01	7.16E-01
**SUGT1**	0.13	5.96E-01	1.00E+00	-0.32	1.57E-01	7.16E-01
**SPOCK2**	-0.26	5.78E-01	1.00E+00	1.34	1.57E-01	7.16E-01
**PLEKHM1**	-0.06	7.81E-01	1.00E+00	0.76	1.57E-01	7.16E-01
**SLC37A1**	-0.10	5.67E-01	1.00E+00	0.61	1.57E-01	7.16E-01
**G24466**	0.83	6.05E-01	1.00E+00	-1.29	1.57E-01	7.16E-01
**ABCD2**	2.44	2.44E-02	9.22E-01	-1.21	1.57E-01	7.16E-01
**HLA-A**	-0.18	5.95E-01	1.00E+00	0.73	1.57E-01	7.16E-01
**SF3B2**	-0.25	1.82E-01	1.00E+00	0.34	1.57E-01	7.16E-01
**MED7**	0.10	6.51E-01	1.00E+00	-0.56	1.57E-01	7.16E-01
**THOC5**	0.22	2.47E-01	1.00E+00	0.24	1.57E-01	7.16E-01

**MRPS23**	0.05	7.14E-01	1.00E+00	0.28	1.57E-01	7.16E-01
**MEIOC**	-0.03	9.72E-01	1.00E+00	-1.37	1.57E-01	7.16E-01
**TMEM18**	0.26	2.61E-01	1.00E+00	-0.31	1.57E-01	7.16E-01
**ELL2P1**	1.24	6.28E-01	1.00E+00	0.92	1.57E-01	7.16E-01
**STKLD1**	-0.17	6.67E-01	1.00E+00	-0.79	1.57E-01	7.16E-01
**PRSS36**	-0.04	9.34E-01	1.00E+00	0.53	1.57E-01	7.16E-01
**NOTCH2**	0.25	2.90E-01	1.00E+00	0.55	1.57E-01	7.16E-01
**PROC**	-0.49	6.84E-01	1.00E+00	1.43	1.57E-01	7.16E-01
**AC032011.1**	-4.45	1.99E-01	1.00E+00	-2.42	1.57E-01	7.16E-01
**NDST2**	-1.02	6.68E-01	1.00E+00	-1.21	1.57E-01	7.16E-01
**ZNF429**	0.24	4.65E-01	1.00E+00	-0.57	1.57E-01	7.16E-01
**UPF3AP1**	-0.03	9.87E-01	1.00E+00	-0.67	1.57E-01	7.16E-01
**AC023886.2**	1.48	6.66E-01	1.00E+00	-0.92	1.57E-01	7.16E-01
**RIPK2**	-0.01	9.66E-01	1.00E+00	0.33	1.57E-01	7.16E-01
**NCR3**	-0.61	3.77E-01	1.00E+00	1.28	1.58E-01	7.16E-01
**ZC3H12D**	-0.31	6.14E-01	1.00E+00	1.51	1.58E-01	7.16E-01
**AC079921.2**	-2.57	9.04E-02	1.00E+00	1.63	1.58E-01	7.16E-01
**MIR200CHG**	0.21	4.32E-01	1.00E+00	-0.95	1.58E-01	7.16E-01
**OPRL1**	0.39	3.79E-01	1.00E+00	1.14	1.58E-01	7.16E-01
**WDTC1**	-0.14	3.17E-01	1.00E+00	0.76	1.58E-01	7.16E-01
**MT-TM**	NA	NA	NA	-1.05	1.58E-01	7.16E-01
**G39546**	0.50	8.74E-01	1.00E+00	-1.79	1.58E-01	7.16E-01
**HBEGF**	-1.30	4.81E-02	1.00E+00	0.77	1.58E-01	7.16E-01
**LRRC59**	-0.38	1.46E-02	7.60E-01	0.38	1.58E-01	7.16E-01
**AL031714.1**	0.65	3.52E-01	1.00E+00	1.59	1.58E-01	7.17E-01
**INTS10**	0.08	6.40E-01	1.00E+00	-0.22	1.58E-01	7.17E-01
**PRIM1**	-0.09	7.29E-01	1.00E+00	-0.40	1.58E-01	7.17E-01
**EEF1GP2**	-0.48	7.13E-01	1.00E+00	-1.96	1.58E-01	7.17E-01
**AP001486.2**	0.14	6.69E-01	1.00E+00	-0.63	1.58E-01	7.17E-01
**AC138356.1**	2.04	4.68E-02	1.00E+00	-0.96	1.58E-01	7.17E-01
**SAA1**	0.67	4.26E-01	1.00E+00	-1.32	1.58E-01	7.17E-01
**SLC25A3**	0.05	8.58E-01	1.00E+00	-0.17	1.58E-01	7.17E-01

**ITM2C**	0.11	7.54E-01	1.00E+00	0.45	1.58E-01	7.17E-01
**ZNF181**	0.42	1.44E-01	1.00E+00	-0.52	1.58E-01	7.17E-01
**DHFRP1**	-1.17	3.49E-01	1.00E+00	-1.04	1.58E-01	7.17E-01
**SYNE3**	0.86	7.96E-02	1.00E+00	0.74	1.58E-01	7.17E-01
**RP9**	-0.03	9.20E-01	1.00E+00	-0.38	1.58E-01	7.17E-01
**PARD6G**	-0.30	4.39E-01	1.00E+00	-0.79	1.58E-01	7.17E-01
**MAP3K7**	0.21	3.65E-01	1.00E+00	-0.33	1.58E-01	7.17E-01
**AC079949.2**	0.37	7.00E-01	1.00E+00	1.43	1.58E-01	7.17E-01
**CBWD7**	-1.11	5.54E-01	1.00E+00	-0.80	1.58E-01	7.17E-01
**MIR99AHG**	0.32	2.89E-01	1.00E+00	-0.54	1.58E-01	7.17E-01
**AC011899.2**	-1.85	4.03E-01	1.00E+00	2.03	1.58E-01	7.17E-01
**SPRR2E**	-0.66	3.45E-01	1.00E+00	1.09	1.59E-01	7.17E-01
**EIF4ENIF1**	0.03	8.59E-01	1.00E+00	-0.41	1.59E-01	7.17E-01
**B3GAT3**	-0.18	3.25E-01	1.00E+00	0.31	1.59E-01	7.17E-01
**SEC61G**	0.16	4.24E-01	1.00E+00	-0.42	1.59E-01	7.17E-01
**RTL5**	0.03	9.47E-01	1.00E+00	1.07	1.59E-01	7.17E-01
**SLC25A23**	-0.23	3.77E-01	1.00E+00	-0.71	1.59E-01	7.17E-01
**FAM120A**	-0.13	3.30E-01	1.00E+00	0.56	1.59E-01	7.17E-01
**SLC45A4**	-0.36	2.96E-01	1.00E+00	0.75	1.59E-01	7.17E-01
**ALOX5AP**	-0.32	5.03E-01	1.00E+00	0.47	1.59E-01	7.17E-01
**FTH1P12**	0.66	8.49E-01	1.00E+00	-0.95	1.59E-01	7.17E-01
**MUC20-OT1**	0.34	6.28E-01	1.00E+00	-0.58	1.59E-01	7.17E-01
**RAB33B**	0.19	4.50E-01	1.00E+00	-0.47	1.59E-01	7.17E-01
**G10181**	-0.84	4.89E-02	1.00E+00	-0.93	1.59E-01	7.17E-01
**TRDV1**	1.86	5.84E-01	1.00E+00	1.62	1.59E-01	7.17E-01
**N4BP3**	0.12	7.47E-01	1.00E+00	-0.57	1.59E-01	7.17E-01
**BORCS5**	-0.28	3.04E-01	1.00E+00	0.29	1.59E-01	7.17E-01
**ZNHIT2**	0.04	8.91E-01	1.00E+00	0.35	1.59E-01	7.17E-01
**TRAV24**	-0.29	9.21E-01	1.00E+00	2.68	1.59E-01	7.17E-01
**SLC25A43**	0.00	9.90E-01	1.00E+00	-0.36	1.59E-01	7.17E-01
**GTF2F1**	-0.22	3.06E-01	1.00E+00	0.51	1.59E-01	7.17E-01
**LARP1B**	0.00	9.87E-01	1.00E+00	-0.36	1.59E-01	7.17E-01

**ZNF823**	0.08	7.54E-01	1.00E+00	-0.40	1.59E-01	7.17E-01
**THAP1**	0.23	4.92E-01	1.00E+00	-0.43	1.59E-01	7.17E-01
**DELE1**	-0.06	8.17E-01	1.00E+00	0.37	1.59E-01	7.17E-01
**SLAMF9**	-1.43	3.79E-01	1.00E+00	1.96	1.59E-01	7.17E-01
**CDV3**	0.08	5.96E-01	1.00E+00	0.38	1.59E-01	7.17E-01
**R3HCC1L**	-0.03	9.34E-01	1.00E+00	0.38	1.59E-01	7.17E-01
**NME9**	0.44	6.71E-01	1.00E+00	-1.66	1.59E-01	7.17E-01
**POGZ**	-0.14	5.52E-01	1.00E+00	0.42	1.59E-01	7.17E-01
**AL161626.1**	0.23	5.19E-01	1.00E+00	-1.42	1.60E-01	7.17E-01
**AL136418.1**	-1.18	3.71E-01	1.00E+00	-0.93	1.60E-01	7.17E-01
**MR1**	0.01	9.60E-01	1.00E+00	0.32	1.60E-01	7.17E-01
**AC131009.2**	-1.25	7.16E-01	1.00E+00	-2.57	1.60E-01	7.17E-01
**DYNC1H1**	-0.19	4.13E-01	1.00E+00	0.47	1.60E-01	7.17E-01
**BEST1**	0.55	2.61E-01	1.00E+00	0.74	1.60E-01	7.17E-01
**NEU2**	0.66	5.70E-01	1.00E+00	1.07	1.60E-01	7.17E-01
**ZBTB8OSP2**	2.17	5.25E-01	1.00E+00	2.27	1.60E-01	7.17E-01
**AD001527.1**	0.87	5.77E-01	1.00E+00	1.74	1.60E-01	7.17E-01
**TRBV6-6**	-1.70	5.75E-01	1.00E+00	2.18	1.60E-01	7.17E-01
**LRRC8C**	-0.17	5.29E-01	1.00E+00	-0.40	1.60E-01	7.17E-01
**ZBTB32**	1.44	2.13E-01	1.00E+00	2.40	1.60E-01	7.17E-01
**C4BPA**	-1.24	7.19E-01	1.00E+00	2.54	1.60E-01	7.17E-01
**AC080162.1**	1.04	5.12E-01	1.00E+00	1.01	1.60E-01	7.17E-01
**RGS18**	0.96	2.90E-01	1.00E+00	1.15	1.60E-01	7.17E-01
**LARS2**	-0.04	8.36E-01	1.00E+00	0.50	1.60E-01	7.17E-01
**OVGP1**	-0.09	9.00E-01	1.00E+00	1.14	1.60E-01	7.17E-01
**ACTR6**	0.18	5.12E-01	1.00E+00	-0.48	1.60E-01	7.17E-01
**RNU1-1**	-0.65	8.51E-01	1.00E+00	-1.25	1.60E-01	7.17E-01
**AK6**	0.27	3.22E-01	1.00E+00	-0.64	1.60E-01	7.17E-01
**RNF44**	-0.23	4.07E-01	1.00E+00	0.83	1.60E-01	7.17E-01
**ZNF114**	0.25	7.35E-01	1.00E+00	-0.97	1.60E-01	7.17E-01
**ZDHHC12**	-0.05	8.01E-01	1.00E+00	0.41	1.60E-01	7.17E-01
**AC105399.1**	NA	NA	NA	-1.16	1.60E-01	7.17E-01

**AL138999.1**	2.55	4.53E-01	1.00E+00	2.15	1.60E-01	7.17E-01
**GRIN2A**	-0.70	1.29E-01	1.00E+00	-0.79	1.60E-01	7.17E-01
**ARSB**	0.03	9.35E-01	1.00E+00	0.68	1.60E-01	7.17E-01
**AC078846.1**	0.18	6.11E-01	1.00E+00	-0.59	1.60E-01	7.17E-01
**LINC00664**	-0.49	8.78E-01	1.00E+00	-2.54	1.60E-01	7.17E-01
**GPRIN3**	0.36	4.12E-01	1.00E+00	1.01	1.60E-01	7.18E-01
**AKR1C3**	0.89	1.14E-01	1.00E+00	-0.79	1.60E-01	7.18E-01
**AC025580.2**	-1.77	5.01E-01	1.00E+00	1.34	1.61E-01	7.18E-01
**ZAN**	-3.46	1.74E-01	1.00E+00	2.05	1.61E-01	7.18E-01
**MICALL2**	-0.57	1.85E-01	1.00E+00	0.89	1.61E-01	7.18E-01
**CYP11A1**	0.09	9.37E-01	1.00E+00	1.42	1.61E-01	7.18E-01
**PPA2**	0.04	8.35E-01	1.00E+00	-0.46	1.61E-01	7.18E-01
**HM13**	-0.18	4.54E-01	1.00E+00	0.32	1.61E-01	7.18E-01
**AC002074.1**	1.76	8.30E-02	1.00E+00	0.94	1.61E-01	7.18E-01
**AC124045.1**	0.43	4.93E-01	1.00E+00	0.83	1.61E-01	7.18E-01
**SMARCD2**	0.20	4.35E-01	1.00E+00	-0.56	1.61E-01	7.18E-01
**AC002480.2**	2.62	3.48E-01	1.00E+00	2.32	1.61E-01	7.18E-01
**KIAA1586**	0.04	9.03E-01	1.00E+00	-0.52	1.61E-01	7.18E-01
**LTBR**	-0.12	6.85E-01	1.00E+00	0.50	1.61E-01	7.18E-01
**PHF1**	0.03	9.25E-01	1.00E+00	0.70	1.61E-01	7.18E-01
**MEAF6**	0.10	5.69E-01	1.00E+00	-0.37	1.61E-01	7.18E-01
**IL36B**	0.54	5.15E-01	1.00E+00	1.01	1.61E-01	7.18E-01
**OLFML1**	0.56	2.80E-01	1.00E+00	0.60	1.61E-01	7.18E-01
**CD46**	0.52	1.50E-01	1.00E+00	-0.46	1.61E-01	7.18E-01
**AL589935.1**	-0.13	9.43E-01	1.00E+00	-1.07	1.61E-01	7.18E-01
**C1orf229**	0.83	3.70E-01	1.00E+00	-1.32	1.61E-01	7.18E-01
**BRCC3**	-0.03	8.70E-01	1.00E+00	-0.33	1.61E-01	7.18E-01
**GAR1**	-0.14	5.71E-01	1.00E+00	0.31	1.61E-01	7.18E-01
**MIR4664**	-0.97	1.85E-01	1.00E+00	2.57	1.61E-01	7.18E-01
**RMND1**	0.20	4.98E-01	1.00E+00	-0.38	1.62E-01	7.20E-01
**RASL11A**	0.28	4.47E-01	1.00E+00	1.08	1.62E-01	7.20E-01
**RPS15AP30**	0.63	8.54E-01	1.00E+00	-1.37	1.62E-01	7.20E-01

**TMEM191C**	0.24	6.17E-01	1.00E+00	0.65	1.62E-01	7.20E-01
**SPATA17**	0.12	8.29E-01	1.00E+00	0.64	1.62E-01	7.20E-01
**PRKG1**	-0.02	9.65E-01	1.00E+00	0.63	1.62E-01	7.20E-01
**GNG4**	-2.93	5.11E-03	4.18E-01	1.42	1.62E-01	7.20E-01
**Z86062.1**	0.73	8.33E-01	1.00E+00	1.26	1.62E-01	7.20E-01
**TMIE**	-0.75	4.20E-02	1.00E+00	-1.10	1.62E-01	7.20E-01
**HSD17B12**	0.10	6.05E-01	1.00E+00	-0.48	1.62E-01	7.20E-01
**XLOC_002066**	0.32	4.89E-01	1.00E+00	1.10	1.62E-01	7.20E-01
**HYDIN**	-0.19	8.68E-01	1.00E+00	1.06	1.62E-01	7.20E-01
**LGALS9B**	-3.44	4.82E-02	1.00E+00	1.63	1.62E-01	7.20E-01
**LIPK**	0.15	8.19E-01	1.00E+00	-1.05	1.62E-01	7.20E-01
**LINC00471**	0.12	8.81E-01	1.00E+00	-0.82	1.62E-01	7.20E-01
**GLG1**	0.15	5.81E-01	1.00E+00	0.31	1.62E-01	7.20E-01
**AP001922.5**	-2.91	7.91E-02	1.00E+00	1.56	1.62E-01	7.20E-01
**RBPMS-AS1**	0.69	4.37E-01	1.00E+00	-1.22	1.62E-01	7.20E-01
**AIMP1**	-0.02	8.96E-01	1.00E+00	-0.23	1.62E-01	7.20E-01
**LINC01621**	0.60	7.64E-01	1.00E+00	1.26	1.62E-01	7.20E-01
**NLRP2**	0.29	6.51E-01	1.00E+00	1.13	1.62E-01	7.20E-01
**AC018638.2**	-0.78	1.23E-01	1.00E+00	1.86	1.62E-01	7.20E-01
**CEACAM7**	0.50	4.41E-01	1.00E+00	-1.09	1.62E-01	7.20E-01
**COX15**	0.32	3.84E-01	1.00E+00	-0.35	1.62E-01	7.20E-01
**STARD9**	0.48	4.07E-01	1.00E+00	0.95	1.62E-01	7.20E-01
**RWDD4**	-0.14	5.26E-01	1.00E+00	-0.26	1.62E-01	7.20E-01
**SLC33A1**	0.03	9.36E-01	1.00E+00	-0.33	1.63E-01	7.20E-01
**RAB6C**	-0.21	9.08E-01	1.00E+00	-0.87	1.63E-01	7.20E-01
**NEU4**	-1.37	5.59E-01	1.00E+00	1.86	1.63E-01	7.20E-01
**PPP1R12C**	0.07	8.35E-01	1.00E+00	1.13	1.63E-01	7.20E-01
**RANBP3L**	0.69	5.02E-01	1.00E+00	-1.33	1.63E-01	7.20E-01
**TTC28**	-0.09	8.10E-01	1.00E+00	0.63	1.63E-01	7.20E-01
**LTA**	-0.62	5.97E-01	1.00E+00	1.45	1.63E-01	7.20E-01
**IGKV1-5**	2.52	4.23E-01	1.00E+00	4.38	1.63E-01	7.20E-01
**SMIM27**	0.74	2.39E-01	1.00E+00	-0.77	1.63E-01	7.20E-01

**DHX57**	0.10	6.31E-01	1.00E+00	0.34	1.63E-01	7.20E-01
**MEGF9**	0.08	7.94E-01	1.00E+00	-0.38	1.63E-01	7.20E-01
**ANKRD7**	0.96	5.59E-01	1.00E+00	-1.66	1.63E-01	7.20E-01
**G22284**	-2.12	2.69E-01	1.00E+00	-1.51	1.63E-01	7.20E-01
**C1GALT1C1**	0.02	9.33E-01	1.00E+00	-0.37	1.63E-01	7.20E-01
**PGM2**	0.08	8.22E-01	1.00E+00	0.36	1.63E-01	7.20E-01
**SNHG20**	0.25	6.35E-01	1.00E+00	-0.60	1.63E-01	7.20E-01
**MRPL35**	0.08	7.30E-01	1.00E+00	-0.30	1.63E-01	7.20E-01
**C15orf40**	0.11	5.74E-01	1.00E+00	-0.44	1.63E-01	7.21E-01
**AC053527.1**	0.17	7.98E-01	1.00E+00	-0.73	1.63E-01	7.21E-01
**AC016773.1**	-0.82	7.16E-01	1.00E+00	1.51	1.63E-01	7.21E-01
**SLC6A7**	-2.49	2.07E-01	1.00E+00	2.11	1.63E-01	7.21E-01
**MILR1**	0.10	8.86E-01	1.00E+00	0.86	1.63E-01	7.21E-01
**GJC3**	-0.01	9.89E-01	1.00E+00	-0.80	1.63E-01	7.21E-01
**AC118138.2**	0.42	6.68E-01	1.00E+00	-1.07	1.63E-01	7.21E-01
**AC092384.1**	-1.07	3.39E-01	1.00E+00	-1.19	1.63E-01	7.21E-01
**FAM87B**	-0.49	4.84E-01	1.00E+00	1.13	1.63E-01	7.21E-01
**TMEM156**	1.10	4.06E-01	1.00E+00	1.14	1.63E-01	7.21E-01
**VIPR1**	-0.29	2.49E-01	1.00E+00	-0.75	1.63E-01	7.21E-01
**LINC02198**	-0.44	8.67E-01	1.00E+00	1.66	1.63E-01	7.21E-01
**ARL16**	-0.08	6.62E-01	1.00E+00	-0.43	1.63E-01	7.21E-01
**AL359715.1**	0.87	6.56E-01	1.00E+00	1.44	1.63E-01	7.21E-01
**AIFM3**	-2.31	3.01E-02	9.97E-01	0.76	1.63E-01	7.21E-01
**GPR173**	0.45	2.21E-01	1.00E+00	1.57	1.63E-01	7.21E-01
**AC016629.2**	-2.08	3.32E-01	1.00E+00	1.37	1.64E-01	7.21E-01
**CFAP300**	-0.07	8.71E-01	1.00E+00	-0.56	1.64E-01	7.21E-01
**AL844908.2**	0.21	8.38E-01	1.00E+00	1.35	1.64E-01	7.21E-01
**SNRPB**	-0.15	5.38E-01	1.00E+00	0.23	1.64E-01	7.21E-01
**CASZ1**	-0.38	3.11E-01	1.00E+00	-0.76	1.64E-01	7.21E-01
**AC002310.1**	-0.42	4.87E-01	1.00E+00	-0.89	1.64E-01	7.21E-01
**TFAP2C**	-0.18	5.72E-01	1.00E+00	-0.62	1.64E-01	7.21E-01
**RASSF4**	0.34	4.57E-01	1.00E+00	0.88	1.64E-01	7.21E-01

**MPRIP-AS1**	0.26	9.00E-01	1.00E+00	1.10	1.64E-01	7.21E-01
**AC092542.1**	1.51	2.54E-02	9.29E-01	-1.20	1.64E-01	7.21E-01
**ERRFI1**	0.00	9.96E-01	1.00E+00	0.54	1.64E-01	7.21E-01
**AC131571.2**	0.63	8.54E-01	1.00E+00	-2.31	1.64E-01	7.21E-01
**PARP15**	-0.27	7.96E-01	1.00E+00	1.85	1.64E-01	7.21E-01
**LAMTOR5**	0.10	6.04E-01	1.00E+00	-0.66	1.64E-01	7.21E-01
**CRISP2**	0.42	7.10E-01	1.00E+00	-1.52	1.64E-01	7.21E-01
**PSMF1**	-0.09	5.82E-01	1.00E+00	0.15	1.64E-01	7.21E-01
**NFE2L2**	-0.05	8.80E-01	1.00E+00	-0.43	1.64E-01	7.21E-01
**AC092368.3**	-0.26	4.17E-01	1.00E+00	1.41	1.64E-01	7.21E-01
**IGHV3-13**	1.22	7.19E-01	1.00E+00	3.50	1.64E-01	7.21E-01
**ZNF268**	0.14	5.85E-01	1.00E+00	-0.26	1.64E-01	7.21E-01
**EEF1GP1**	-0.06	9.86E-01	1.00E+00	-1.38	1.64E-01	7.21E-01
**NDUFA4L2**	-0.25	2.73E-01	1.00E+00	0.53	1.64E-01	7.21E-01
**RPRD1B**	0.03	8.54E-01	1.00E+00	0.35	1.64E-01	7.21E-01
**DCUN1D1**	0.08	7.85E-01	1.00E+00	-0.38	1.64E-01	7.21E-01
**CAPN15**	-0.69	1.18E-01	1.00E+00	0.91	1.64E-01	7.21E-01
**ARMCX2**	0.19	7.13E-01	1.00E+00	0.75	1.64E-01	7.21E-01
**MCF2L-AS1**	0.19	7.65E-01	1.00E+00	-0.76	1.64E-01	7.21E-01
**FAM168A**	-0.03	9.27E-01	1.00E+00	0.87	1.65E-01	7.21E-01
**HOXB2**	-0.20	7.10E-01	1.00E+00	-0.74	1.65E-01	7.21E-01
**DPP4**	0.38	5.05E-01	1.00E+00	0.86	1.65E-01	7.21E-01
**AC007319.1**	0.98	1.93E-01	1.00E+00	-0.88	1.65E-01	7.21E-01
**PART1**	0.15	7.67E-01	1.00E+00	-0.71	1.65E-01	7.21E-01
**AL356966.1**	-0.64	5.97E-01	1.00E+00	1.20	1.65E-01	7.21E-01
**AL589986.2**	-0.56	8.73E-01	1.00E+00	-1.90	1.65E-01	7.21E-01
**KIF6**	0.23	6.58E-01	1.00E+00	1.10	1.65E-01	7.21E-01
**AC098824.1**	1.08	4.60E-01	1.00E+00	1.16	1.65E-01	7.21E-01
**AC004160.1**	5.02	3.11E-03	2.91E-01	-1.46	1.65E-01	7.21E-01
**G28010**	-0.29	7.59E-01	1.00E+00	1.76	1.65E-01	7.21E-01
**CBWD5**	-0.24	5.52E-01	1.00E+00	-0.55	1.65E-01	7.21E-01
**XLOC_007635**	0.20	9.53E-01	1.00E+00	-1.77	1.65E-01	7.21E-01

**RNF103-CHMP3**	0.31	8.10E-01	1.00E+00	1.21	1.65E-01	7.21E-01
**ARHGAP22**	-0.63	4.78E-02	1.00E+00	0.80	1.65E-01	7.21E-01
**BRIX1**	-0.07	7.73E-01	1.00E+00	-0.60	1.65E-01	7.22E-01
**SCFD1**	0.05	7.77E-01	1.00E+00	-0.38	1.65E-01	7.22E-01
**ADGRL2**	0.18	4.58E-01	1.00E+00	0.55	1.65E-01	7.22E-01
**CHRD**	0.49	2.69E-01	1.00E+00	1.27	1.65E-01	7.22E-01
**MRPL10**	0.03	8.78E-01	1.00E+00	0.30	1.65E-01	7.22E-01
**G35511**	0.34	7.56E-01	1.00E+00	-1.14	1.65E-01	7.22E-01
**FOXE3**	0.42	8.36E-01	1.00E+00	-1.95	1.65E-01	7.22E-01
**GOLPH3**	0.07	6.35E-01	1.00E+00	-0.61	1.65E-01	7.23E-01
**G23010**	-2.92	7.82E-02	1.00E+00	1.41	1.65E-01	7.23E-01
**FUBP1**	0.01	9.60E-01	1.00E+00	0.31	1.66E-01	7.23E-01
**ZNF133**	-0.03	8.65E-01	1.00E+00	0.31	1.66E-01	7.23E-01
**RAB2A**	0.24	2.20E-01	1.00E+00	-0.41	1.66E-01	7.23E-01
**ADGRG5**	-0.56	3.55E-01	1.00E+00	1.24	1.66E-01	7.23E-01
**YIPF4**	0.34	4.87E-02	1.00E+00	-0.28	1.66E-01	7.23E-01
**RNU6-137P**	-0.46	4.46E-01	1.00E+00	-1.22	1.66E-01	7.23E-01
**SLC39A9**	0.36	1.55E-01	1.00E+00	0.22	1.66E-01	7.23E-01
**PIK3C2A**	0.03	8.92E-01	1.00E+00	-0.19	1.66E-01	7.23E-01
**LINC00963**	-0.46	5.84E-02	1.00E+00	0.52	1.66E-01	7.23E-01
**AKAP2**	-2.23	3.53E-01	1.00E+00	0.87	1.66E-01	7.23E-01
**SLC27A5**	0.04	8.79E-01	1.00E+00	-0.48	1.66E-01	7.24E-01
**DSCAS**	-0.78	8.20E-01	1.00E+00	1.41	1.66E-01	7.24E-01
**AGPAT2**	0.96	9.94E-02	1.00E+00	-0.77	1.66E-01	7.24E-01
**LRRC37A7P**	-0.08	8.37E-01	1.00E+00	-0.68	1.66E-01	7.24E-01
**CENPV**	0.17	7.76E-01	1.00E+00	-1.23	1.66E-01	7.24E-01
**BCL2L12**	-0.32	2.75E-01	1.00E+00	0.46	1.66E-01	7.24E-01
**CD164**	0.48	1.84E-01	1.00E+00	-0.42	1.66E-01	7.24E-01
**NEIL2**	-0.14	5.33E-01	1.00E+00	-0.32	1.66E-01	7.24E-01
**KRT223P**	3.43	1.67E-01	1.00E+00	-1.39	1.66E-01	7.24E-01
**AC079781.4**	-0.03	9.52E-01	1.00E+00	-1.01	1.66E-01	7.24E-01
**PCDHGB3**	-0.27	5.71E-01	1.00E+00	-1.23	1.66E-01	7.24E-01

**VBP1**	0.07	7.44E-01	1.00E+00	-0.37	1.66E-01	7.24E-01
**ZNF710**	-0.58	1.04E-01	1.00E+00	0.82	1.66E-01	7.24E-01
**CYP26A1**	-0.54	8.39E-01	1.00E+00	-2.26	1.66E-01	7.24E-01
**LYN**	-0.34	1.37E-01	1.00E+00	1.00	1.66E-01	7.24E-01
**XLOC_001583**	-2.33	1.74E-01	1.00E+00	1.84	1.67E-01	7.24E-01
**HRH1**	-0.17	6.40E-01	1.00E+00	0.57	1.67E-01	7.24E-01
**NOL4**	-2.70	4.63E-02	1.00E+00	-1.91	1.67E-01	7.24E-01
**PTGES**	-0.21	5.14E-01	1.00E+00	0.49	1.67E-01	7.24E-01
**C3orf58**	-0.19	6.29E-01	1.00E+00	-0.39	1.67E-01	7.24E-01
**GPATCH3**	-0.14	4.84E-01	1.00E+00	0.29	1.67E-01	7.24E-01
**IKBKB**	-0.04	8.54E-01	1.00E+00	0.48	1.67E-01	7.24E-01
**MTMR2**	0.05	8.16E-01	1.00E+00	-0.25	1.67E-01	7.25E-01
**FCRLB**	-0.11	8.61E-01	1.00E+00	0.75	1.67E-01	7.25E-01
**AC091868.2**	0.64	3.08E-01	1.00E+00	-1.61	1.67E-01	7.25E-01
**UGT2B15**	0.41	8.42E-01	1.00E+00	-2.59	1.67E-01	7.25E-01
**ZNF592**	-0.11	6.33E-01	1.00E+00	0.65	1.67E-01	7.25E-01
**AL031729.1**	-1.57	4.35E-01	1.00E+00	-0.88	1.67E-01	7.25E-01
**G38528**	-1.14	5.10E-01	1.00E+00	1.24	1.67E-01	7.25E-01
**CNOT9**	-0.03	9.15E-01	1.00E+00	0.40	1.67E-01	7.25E-01
**ITM2B**	-0.01	9.69E-01	1.00E+00	-0.34	1.67E-01	7.25E-01
**COL9A1**	1.73	1.71E-01	1.00E+00	1.42	1.67E-01	7.25E-01
**OSGEP**	0.07	7.42E-01	1.00E+00	0.23	1.67E-01	7.25E-01
**SEL1L2**	-1.18	5.32E-01	1.00E+00	-1.28	1.67E-01	7.25E-01
**AC015971.1**	-2.07	3.41E-01	1.00E+00	-1.33	1.67E-01	7.25E-01
**DIRC1**	-2.31	1.17E-01	1.00E+00	1.45	1.67E-01	7.25E-01
**FCRL3**	-3.63	7.91E-02	1.00E+00	2.14	1.67E-01	7.25E-01
**SHKBP1**	-0.42	8.47E-02	1.00E+00	0.35	1.68E-01	7.25E-01
**ASPHD1**	-2.30	2.97E-03	2.86E-01	1.20	1.68E-01	7.25E-01
**LINC01625**	0.16	8.58E-01	1.00E+00	1.43	1.68E-01	7.26E-01
**BCLAF3**	-0.19	4.35E-01	1.00E+00	-0.47	1.68E-01	7.26E-01
**DSTN**	-0.11	5.84E-01	1.00E+00	-0.40	1.68E-01	7.26E-01
**DLX4**	-0.87	7.18E-02	1.00E+00	-0.82	1.68E-01	7.26E-01

**CCDC88C**	-0.58	2.50E-02	9.28E-01	0.80	1.68E-01	7.26E-01
**ESRRG**	0.04	9.65E-01	1.00E+00	-0.99	1.68E-01	7.26E-01
**FTH1P11**	1.79	4.99E-01	1.00E+00	-0.91	1.68E-01	7.26E-01
**NAPSB**	-0.27	5.69E-01	1.00E+00	1.02	1.68E-01	7.26E-01
**ERG28**	-0.13	6.66E-01	1.00E+00	-0.53	1.68E-01	7.26E-01
**SAMHD1**	0.45	2.71E-01	1.00E+00	0.80	1.68E-01	7.26E-01
**AC136475.7**	0.99	7.02E-01	1.00E+00	1.71	1.68E-01	7.26E-01
**AP000813.1**	-0.15	9.02E-01	1.00E+00	-1.11	1.68E-01	7.26E-01
**HIBCH**	0.05	8.34E-01	1.00E+00	-0.49	1.68E-01	7.26E-01
**UNC80**	-1.80	6.71E-02	1.00E+00	-1.38	1.68E-01	7.26E-01
**GSTP1**	-0.02	9.42E-01	1.00E+00	0.29	1.68E-01	7.26E-01
**NAGK**	-0.15	5.41E-01	1.00E+00	0.40	1.68E-01	7.26E-01
**ST6GAL2**	0.25	7.19E-01	1.00E+00	-0.98	1.68E-01	7.26E-01
**AL391056.1**	-0.93	3.23E-01	1.00E+00	1.95	1.68E-01	7.26E-01
**OR7E12P**	1.01	4.36E-01	1.00E+00	-1.81	1.68E-01	7.26E-01
**AKT2**	0.11	6.48E-01	1.00E+00	0.78	1.68E-01	7.26E-01
**LYPLA2**	-0.10	6.69E-01	1.00E+00	0.24	1.68E-01	7.26E-01
**UBXN7**	0.04	8.08E-01	1.00E+00	-0.42	1.68E-01	7.26E-01
**CNTN5**	-10.95	1.38E-03	2.02E-01	4.34	1.68E-01	7.26E-01
**AHCYL1**	0.06	7.27E-01	1.00E+00	-0.52	1.68E-01	7.26E-01
**SHANK1**	-1.11	4.11E-02	1.00E+00	1.64	1.69E-01	7.26E-01
**SGK1**	-0.45	1.91E-01	1.00E+00	0.44	1.69E-01	7.26E-01
**AL512353.1**	0.26	7.97E-01	1.00E+00	0.64	1.69E-01	7.26E-01
**PRDX2**	-0.05	8.16E-01	1.00E+00	-0.62	1.69E-01	7.26E-01
**AC011352.1**	0.39	5.54E-01	1.00E+00	-0.84	1.69E-01	7.26E-01
**TMEM211**	-1.96	1.27E-01	1.00E+00	-1.16	1.69E-01	7.26E-01
**CCL22**	-0.35	5.75E-01	1.00E+00	-0.64	1.69E-01	7.26E-01
**PLIN5**	1.42	5.66E-02	1.00E+00	-1.06	1.69E-01	7.26E-01
**WDR93**	1.05	4.70E-01	1.00E+00	-1.14	1.69E-01	7.27E-01
**NAGS**	-0.51	2.14E-01	1.00E+00	0.82	1.69E-01	7.27E-01
**CD6**	-0.29	5.95E-01	1.00E+00	1.23	1.69E-01	7.27E-01
**NDUFB4**	-0.19	2.14E-01	1.00E+00	-0.43	1.69E-01	7.27E-01

**AC080038.1**	-0.99	2.00E-01	1.00E+00	-1.14	1.69E-01	7.27E-01
**MYCBPAP**	-0.81	4.15E-01	1.00E+00	1.08	1.69E-01	7.27E-01
**HDAC1P2**	1.05	7.55E-01	1.00E+00	-2.06	1.69E-01	7.27E-01
**PJA2**	-0.05	7.66E-01	1.00E+00	-0.22	1.69E-01	7.27E-01
**MOCS2**	-0.04	8.87E-01	1.00E+00	-0.41	1.69E-01	7.27E-01
**IL1R1**	0.33	2.03E-01	1.00E+00	0.66	1.69E-01	7.27E-01
**NOB1**	-0.14	4.92E-01	1.00E+00	0.33	1.69E-01	7.27E-01
**SKAP2**	0.10	6.72E-01	1.00E+00	-0.28	1.69E-01	7.27E-01
**FLJ46284**	1.02	6.28E-01	1.00E+00	1.32	1.69E-01	7.27E-01
**AC040162.1**	1.66	1.52E-01	1.00E+00	0.98	1.69E-01	7.27E-01
**XLOC_000578**	-0.89	6.17E-01	1.00E+00	-2.06	1.69E-01	7.27E-01
**AC092017.2**	0.19	9.39E-01	1.00E+00	-1.04	1.69E-01	7.27E-01
**DARS**	0.05	8.07E-01	1.00E+00	-0.30	1.69E-01	7.27E-01
**AC107068.1**	0.29	6.18E-01	1.00E+00	0.93	1.69E-01	7.27E-01
**C12orf80**	-1.46	5.54E-01	1.00E+00	-1.51	1.69E-01	7.27E-01
**OBP2B**	-0.53	5.57E-01	1.00E+00	-1.18	1.70E-01	7.27E-01
**ZNF620**	0.37	3.93E-01	1.00E+00	0.59	1.70E-01	7.27E-01
**RASSF2**	0.34	4.11E-01	1.00E+00	0.78	1.70E-01	7.27E-01
**SDCBP2-AS1**	0.86	1.45E-01	1.00E+00	0.69	1.70E-01	7.27E-01
**MS4A4A**	1.06	4.64E-02	1.00E+00	0.87	1.70E-01	7.27E-01
**ZSWIM6**	-0.26	3.60E-01	1.00E+00	0.61	1.70E-01	7.27E-01
**RWDD1**	0.13	5.20E-01	1.00E+00	-0.26	1.70E-01	7.27E-01
**TAF9B**	0.04	8.97E-01	1.00E+00	-0.41	1.70E-01	7.27E-01
**FCGR1A**	-0.84	2.47E-01	1.00E+00	4.30	1.70E-01	7.27E-01
**MCM3AP-AS1**	0.34	4.64E-01	1.00E+00	0.63	1.70E-01	7.27E-01
**AC079880.1**	1.68	2.04E-01	1.00E+00	-0.66	1.70E-01	7.27E-01
**MGC32805**	-1.23	3.25E-01	1.00E+00	-1.28	1.70E-01	7.27E-01
**RNF103**	0.22	2.94E-01	1.00E+00	-0.42	1.70E-01	7.27E-01
**PRELID1**	-0.15	5.98E-01	1.00E+00	0.37	1.70E-01	7.27E-01
**SNX24**	0.13	6.17E-01	1.00E+00	-0.34	1.70E-01	7.27E-01
**S1PR5**	0.02	9.53E-01	1.00E+00	-0.65	1.70E-01	7.28E-01
**ATP6V0A4**	0.01	9.90E-01	1.00E+00	-0.96	1.70E-01	7.28E-01

**PSMG2**	0.02	9.35E-01	1.00E+00	-0.30	1.70E-01	7.28E-01
**AC022613.3**	-0.67	8.48E-01	1.00E+00	1.74	1.70E-01	7.28E-01
**TBC1D19**	0.06	8.78E-01	1.00E+00	-0.50	1.70E-01	7.28E-01
**FMNL3**	-0.09	8.18E-01	1.00E+00	1.02	1.70E-01	7.28E-01
**OSGIN2**	0.29	4.09E-01	1.00E+00	-0.57	1.70E-01	7.28E-01
**FADS3**	1.37	2.13E-01	1.00E+00	-0.99	1.70E-01	7.28E-01
**PRMT5**	-0.16	4.85E-01	1.00E+00	0.30	1.70E-01	7.28E-01
**ADD3-AS1**	0.95	1.48E-01	1.00E+00	1.03	1.70E-01	7.28E-01
**ARHGEF9**	-0.14	5.64E-01	1.00E+00	0.35	1.70E-01	7.28E-01
**GNAO1**	-0.98	3.27E-02	1.00E+00	1.07	1.70E-01	7.28E-01
**AC008543.1**	-0.21	8.11E-01	1.00E+00	-1.24	1.70E-01	7.28E-01
**IL18RAP**	0.08	9.60E-01	1.00E+00	2.00	1.70E-01	7.28E-01
**ERMP1**	-0.13	6.60E-01	1.00E+00	-0.58	1.71E-01	7.28E-01
**MBNL3**	0.08	7.55E-01	1.00E+00	-0.35	1.71E-01	7.28E-01
**TMEM167B**	0.17	3.59E-01	1.00E+00	-0.31	1.71E-01	7.28E-01
**ARF4-AS1**	0.63	6.80E-01	1.00E+00	-0.92	1.71E-01	7.28E-01
**AC023051.1**	-0.03	9.90E-01	1.00E+00	-0.99	1.71E-01	7.28E-01
**ENPP7P7**	-1.54	2.81E-01	1.00E+00	-1.19	1.71E-01	7.28E-01
**ADAR**	0.05	8.63E-01	1.00E+00	0.58	1.71E-01	7.28E-01
**ATP6V0C**	-0.33	6.55E-01	1.00E+00	0.94	1.71E-01	7.28E-01
**ELOC**	0.06	6.90E-01	1.00E+00	-0.37	1.71E-01	7.28E-01
**LINC01881**	0.18	8.04E-01	1.00E+00	-0.57	1.71E-01	7.28E-01
**USP22**	0.20	5.08E-01	1.00E+00	0.42	1.71E-01	7.29E-01
**PTMAP2**	-0.06	8.50E-01	1.00E+00	-0.60	1.71E-01	7.29E-01
**UIMC1**	-0.19	3.15E-01	1.00E+00	0.27	1.71E-01	7.29E-01
**AC083837.2**	0.04	9.55E-01	1.00E+00	0.76	1.71E-01	7.29E-01
**AC015819.1**	0.63	6.29E-01	1.00E+00	1.70	1.71E-01	7.29E-01
**GAS6-AS1**	-0.23	8.05E-01	1.00E+00	1.27	1.71E-01	7.29E-01
**ANKEF1**	-0.34	2.04E-01	1.00E+00	-0.43	1.71E-01	7.29E-01
**DET1**	0.74	4.93E-01	1.00E+00	-1.20	1.71E-01	7.29E-01
**EID1**	0.02	9.52E-01	1.00E+00	-0.47	1.71E-01	7.29E-01
**PRKAR2B**	1.56	3.44E-02	1.00E+00	-0.96	1.71E-01	7.29E-01

**RBM48**	-0.01	9.48E-01	1.00E+00	-0.50	1.71E-01	7.29E-01
**PIK3R6**	-0.28	7.24E-01	1.00E+00	1.09	1.72E-01	7.30E-01
**SH3BP2**	0.03	9.00E-01	1.00E+00	0.47	1.72E-01	7.30E-01
**AC131097.4**	0.20	8.91E-01	1.00E+00	1.69	1.72E-01	7.30E-01
**AL162741.1**	-1.49	2.89E-01	1.00E+00	1.61	1.72E-01	7.30E-01
**SPINK2**	-0.08	9.31E-01	1.00E+00	0.73	1.72E-01	7.30E-01
**PEAK1**	-0.13	6.78E-01	1.00E+00	0.70	1.72E-01	7.30E-01
**SLC7A11**	-1.01	1.56E-01	1.00E+00	0.77	1.72E-01	7.30E-01
**SLC24A1**	0.02	9.55E-01	1.00E+00	0.66	1.72E-01	7.30E-01
**FCF1**	0.45	1.54E-02	7.70E-01	0.28	1.72E-01	7.30E-01
**ITGB1BP2**	1.26	3.44E-01	1.00E+00	1.62	1.72E-01	7.30E-01
**BRD7P2**	0.52	6.38E-01	1.00E+00	-0.62	1.72E-01	7.30E-01
**AHSG**	-1.75	2.93E-01	1.00E+00	2.13	1.72E-01	7.30E-01
**SYT9**	0.90	9.28E-02	1.00E+00	-1.62	1.72E-01	7.30E-01
**ZNF75A**	0.19	5.45E-01	1.00E+00	-0.32	1.72E-01	7.30E-01
**COX6A1**	-0.16	5.53E-01	1.00E+00	-0.87	1.72E-01	7.30E-01
**NSMAF**	0.09	6.29E-01	1.00E+00	-0.32	1.72E-01	7.30E-01
**GMPSP1**	0.29	9.29E-01	1.00E+00	-0.79	1.72E-01	7.30E-01
**AC073130.2**	1.17	7.02E-01	1.00E+00	-1.52	1.72E-01	7.30E-01
**BOLA1**	-0.02	9.31E-01	1.00E+00	-0.30	1.72E-01	7.30E-01
**LINC01950**	-4.26	1.62E-01	1.00E+00	-1.71	1.72E-01	7.30E-01
**AL078645.2**	-1.69	5.76E-01	1.00E+00	-2.79	1.72E-01	7.30E-01
**PROB1**	-0.45	1.41E-01	1.00E+00	1.32	1.72E-01	7.30E-01
**NPAS4**	-0.94	6.64E-01	1.00E+00	1.07	1.72E-01	7.30E-01
**STX10**	-0.07	7.06E-01	1.00E+00	0.24	1.72E-01	7.30E-01
**AC079313.1**	1.74	3.72E-01	1.00E+00	-1.21	1.72E-01	7.30E-01
**AC022079.1**	0.32	9.26E-01	1.00E+00	-2.00	1.73E-01	7.30E-01
**SYT4**	-14.45	2.36E-05	6.87E-03	-2.82	1.73E-01	7.30E-01
**LINC01857**	-1.52	6.60E-01	1.00E+00	3.39	1.73E-01	7.30E-01
**SAMMSON**	-0.42	8.62E-01	1.00E+00	2.11	1.73E-01	7.30E-01
**SNX25P1**	0.16	9.39E-01	1.00E+00	1.99	1.73E-01	7.30E-01
**SPINT1-AS1**	0.44	1.44E-01	1.00E+00	-0.81	1.73E-01	7.30E-01

**KLHDC4**	-0.10	6.61E-01	1.00E+00	0.51	1.73E-01	7.30E-01
**HSPB9**	-0.02	9.83E-01	1.00E+00	0.87	1.73E-01	7.30E-01
**TENT5A**	0.16	6.79E-01	1.00E+00	0.31	1.73E-01	7.31E-01
**AL390760.1**	-2.87	1.22E-01	1.00E+00	1.56	1.73E-01	7.31E-01
**LOXL2**	-0.08	8.95E-01	1.00E+00	0.83	1.73E-01	7.31E-01
**XRN2**	-0.04	8.39E-01	1.00E+00	0.20	1.73E-01	7.31E-01
**ATP23**	0.23	2.76E-01	1.00E+00	-0.48	1.73E-01	7.31E-01
**AC068446.1**	-1.72	4.08E-01	1.00E+00	1.11	1.73E-01	7.31E-01
**GMDS-DT**	0.20	7.12E-01	1.00E+00	-0.62	1.73E-01	7.31E-01
**TCP11**	-1.78	3.06E-02	1.00E+00	1.17	1.73E-01	7.31E-01
**G6795**	-0.95	4.70E-01	1.00E+00	-1.36	1.73E-01	7.31E-01
**RPS5**	-0.19	5.74E-01	1.00E+00	0.37	1.73E-01	7.31E-01
**TEX26-AS1**	1.30	2.49E-01	1.00E+00	1.22	1.73E-01	7.31E-01
**CELF1**	0.19	3.91E-01	1.00E+00	-0.26	1.73E-01	7.31E-01
**GRAMD2A**	-0.52	8.12E-02	1.00E+00	0.80	1.73E-01	7.31E-01
**SLC50A1**	-0.26	2.51E-01	1.00E+00	-0.28	1.73E-01	7.31E-01
**YWHAH**	-0.26	3.31E-01	1.00E+00	-0.72	1.73E-01	7.31E-01
**TNFRSF10C**	0.26	7.75E-01	1.00E+00	1.06	1.73E-01	7.31E-01
**AC008035.1**	-0.67	2.06E-01	1.00E+00	-0.88	1.73E-01	7.31E-01
**SLC25A26**	0.22	3.06E-01	1.00E+00	-0.35	1.73E-01	7.31E-01
**MRPL13**	0.01	9.39E-01	1.00E+00	-0.56	1.73E-01	7.31E-01
**SPRED3**	-0.98	3.53E-02	1.00E+00	1.21	1.73E-01	7.31E-01
**AC239800.2**	1.10	2.51E-01	1.00E+00	-0.88	1.74E-01	7.31E-01
**RP11-187C18.3**	-3.44	2.75E-01	1.00E+00	-1.37	1.74E-01	7.31E-01
**RILPL2**	0.13	6.89E-01	1.00E+00	0.68	1.74E-01	7.31E-01
**SYNDIG1**	-0.57	4.98E-01	1.00E+00	1.53	1.74E-01	7.31E-01
**HAND1**	1.33	4.79E-01	1.00E+00	3.08	1.74E-01	7.31E-01
**CENPBD1**	-0.13	6.17E-01	1.00E+00	-0.24	1.74E-01	7.31E-01
**COP1**	0.14	4.15E-01	1.00E+00	-0.58	1.74E-01	7.31E-01
**KLHL7**	0.34	1.47E-01	1.00E+00	-0.35	1.74E-01	7.31E-01
**THEM6**	-0.29	1.90E-01	1.00E+00	-0.36	1.74E-01	7.31E-01
**ZBED6CL**	-0.07	8.98E-01	1.00E+00	0.95	1.74E-01	7.31E-01

**SLC1A2**	-0.76	8.12E-01	1.00E+00	0.71	1.74E-01	7.31E-01
**EPPK1**	-0.36	5.26E-01	1.00E+00	-0.94	1.74E-01	7.31E-01
**MARS**	-0.24	5.27E-01	1.00E+00	0.23	1.74E-01	7.31E-01
**S100A13**	-0.24	7.14E-01	1.00E+00	-0.84	1.74E-01	7.31E-01
**TGFB1**	-0.46	4.47E-02	1.00E+00	1.30	1.74E-01	7.31E-01
**PINLYP**	-0.31	4.96E-01	1.00E+00	0.52	1.74E-01	7.31E-01
**NEMF**	0.24	1.36E-01	1.00E+00	-0.35	1.74E-01	7.31E-01
**AC010883.1**	-0.93	6.51E-01	1.00E+00	-1.11	1.74E-01	7.31E-01
**NPIPB11**	-1.98	1.46E-01	1.00E+00	1.20	1.74E-01	7.31E-01
**MALRD1**	0.51	6.05E-01	1.00E+00	1.12	1.74E-01	7.31E-01
**RNPS1P1**	-1.31	5.30E-01	1.00E+00	-0.72	1.74E-01	7.31E-01
**ZKSCAN2**	0.12	7.20E-01	1.00E+00	0.83	1.74E-01	7.31E-01
**F11R**	0.17	3.01E-01	1.00E+00	-0.30	1.74E-01	7.31E-01
**G9021**	-0.35	7.85E-01	1.00E+00	1.07	1.74E-01	7.31E-01
**FAR2**	0.09	8.63E-01	1.00E+00	-0.87	1.74E-01	7.31E-01
**MAGI2-AS3**	0.29	5.74E-01	1.00E+00	-0.78	1.74E-01	7.31E-01
**RPTOR**	-0.18	4.63E-01	1.00E+00	0.82	1.75E-01	7.31E-01
**CEP164**	-0.10	6.87E-01	1.00E+00	0.61	1.75E-01	7.31E-01
**PPARGC1A**	0.00	1.00E+00	1.00E+00	-0.79	1.75E-01	7.31E-01
**LYPLAL1**	0.15	5.04E-01	1.00E+00	-0.64	1.75E-01	7.31E-01
**EDA**	0.31	3.64E-01	1.00E+00	-0.66	1.75E-01	7.31E-01
**LINC00271**	-0.16	8.42E-01	1.00E+00	-0.85	1.75E-01	7.31E-01
**CDYL2**	-0.45	1.46E-01	1.00E+00	0.98	1.75E-01	7.31E-01
**SIGIRR**	-0.02	9.53E-01	1.00E+00	0.45	1.75E-01	7.31E-01
**AL590723.1**	0.04	9.68E-01	1.00E+00	1.06	1.75E-01	7.31E-01
**AP001462.1**	-0.85	2.94E-01	1.00E+00	-0.71	1.75E-01	7.31E-01
**XLOC_002116**	0.76	5.37E-01	1.00E+00	-2.71	1.75E-01	7.31E-01
**ABCC2**	-0.30	6.57E-01	1.00E+00	1.06	1.75E-01	7.31E-01
**DPPA4**	NA	NA	NA	-1.83	1.75E-01	7.31E-01
**TP53I13**	-0.09	7.45E-01	1.00E+00	0.46	1.75E-01	7.31E-01
**CLDN10-AS1**	0.18	8.71E-01	1.00E+00	-0.92	1.75E-01	7.31E-01
**ZFAND2B**	0.07	7.12E-01	1.00E+00	-0.33	1.75E-01	7.31E-01

**TXNDC2**	0.52	8.50E-01	1.00E+00	2.64	1.75E-01	7.31E-01
**C5orf63**	-0.18	6.76E-01	1.00E+00	-0.55	1.75E-01	7.31E-01
**AC098934.1**	-1.01	6.25E-02	1.00E+00	0.75	1.75E-01	7.31E-01
**AC005865.1**	0.58	8.64E-01	1.00E+00	-2.63	1.75E-01	7.31E-01
**HMGCS2**	0.52	5.38E-01	1.00E+00	-1.11	1.75E-01	7.31E-01
**PIP4P2**	0.34	2.69E-01	1.00E+00	-0.62	1.75E-01	7.31E-01
**G35839**	1.15	9.37E-02	1.00E+00	-1.12	1.75E-01	7.31E-01
**AP005131.1**	-0.36	8.31E-01	1.00E+00	2.01	1.75E-01	7.31E-01
**AC109347.1**	-0.46	4.62E-01	1.00E+00	0.71	1.75E-01	7.31E-01
**AC134878.2**	0.61	5.90E-01	1.00E+00	1.01	1.75E-01	7.31E-01
**SRPX**	0.77	6.62E-02	1.00E+00	0.70	1.75E-01	7.31E-01
**PCDH18**	0.63	2.67E-01	1.00E+00	0.74	1.75E-01	7.31E-01
**G25996**	-0.54	8.76E-01	1.00E+00	-1.17	1.75E-01	7.31E-01
**ZNF12**	0.13	4.99E-01	1.00E+00	-0.45	1.75E-01	7.31E-01
**RNGTT**	0.28	1.99E-01	1.00E+00	-0.26	1.75E-01	7.31E-01
**XLOC_007654**	-0.08	9.40E-01	1.00E+00	1.81	1.75E-01	7.31E-01
**FAHD2B**	-0.07	8.14E-01	1.00E+00	-0.40	1.75E-01	7.31E-01
**PRKCQ-AS1**	-0.83	3.99E-01	1.00E+00	1.30	1.75E-01	7.31E-01
**CLEC12B**	0.29	6.80E-01	1.00E+00	1.27	1.75E-01	7.31E-01
**CDKL2**	-0.50	4.85E-01	1.00E+00	-1.09	1.75E-01	7.31E-01
**LAYN**	0.22	5.67E-01	1.00E+00	0.57	1.76E-01	7.31E-01
**PANK1**	0.04	9.35E-01	1.00E+00	-0.55	1.76E-01	7.31E-01
**HHIP**	-1.52	1.73E-01	1.00E+00	-1.39	1.76E-01	7.31E-01
**AFF2**	0.39	5.55E-01	1.00E+00	-1.08	1.76E-01	7.31E-01
**CACNA1C**	-0.44	4.77E-01	1.00E+00	1.02	1.76E-01	7.31E-01
**RASSF6**	-0.13	7.43E-01	1.00E+00	-0.94	1.76E-01	7.31E-01
**XRRA1**	-0.44	3.61E-01	1.00E+00	-0.54	1.76E-01	7.31E-01
**ATXN1L**	0.13	4.95E-01	1.00E+00	0.59	1.76E-01	7.31E-01
**SLC36A4**	-0.19	4.67E-01	1.00E+00	-0.32	1.76E-01	7.31E-01
**PRKACA**	-0.11	5.40E-01	1.00E+00	0.69	1.76E-01	7.31E-01
**AC012560.1**	-0.06	9.73E-01	1.00E+00	-1.20	1.76E-01	7.31E-01
**MED1**	-0.01	9.40E-01	1.00E+00	0.47	1.76E-01	7.31E-01

**AP000442.1**	-1.10	6.40E-01	1.00E+00	-1.38	1.76E-01	7.31E-01
**XLOC_003438**	0.08	9.14E-01	1.00E+00	1.12	1.76E-01	7.31E-01
**AQP2**	-0.11	9.38E-01	1.00E+00	-1.26	1.76E-01	7.31E-01
**MOAP1**	0.00	9.81E-01	1.00E+00	-0.23	1.76E-01	7.31E-01
**ZBTB40**	0.15	6.59E-01	1.00E+00	0.73	1.76E-01	7.31E-01
**PLPP3**	0.76	1.10E-01	1.00E+00	0.56	1.76E-01	7.31E-01
**AC018755.1**	-0.70	5.64E-01	1.00E+00	-1.58	1.76E-01	7.31E-01
**ACADSB**	-0.01	9.63E-01	1.00E+00	-0.46	1.76E-01	7.31E-01
**NR5A1**	-1.72	2.94E-01	1.00E+00	-2.26	1.76E-01	7.31E-01
**MIR616**	-2.60	1.43E-01	1.00E+00	-1.30	1.76E-01	7.31E-01
**NDUFAB1**	0.05	7.86E-01	1.00E+00	-0.51	1.76E-01	7.31E-01
**FFAR4**	1.50	3.29E-02	1.00E+00	-0.63	1.76E-01	7.31E-01
**ST6GALNAC5**	0.32	5.57E-01	1.00E+00	0.84	1.76E-01	7.31E-01
**C6orf58**	-1.36	4.09E-01	1.00E+00	-1.32	1.76E-01	7.32E-01
**TLE3**	-0.32	2.27E-01	1.00E+00	0.44	1.76E-01	7.32E-01
**GATB**	-0.02	9.43E-01	1.00E+00	0.25	1.76E-01	7.32E-01
**NGLY1**	0.08	7.54E-01	1.00E+00	-0.32	1.76E-01	7.32E-01
**TXNIP**	0.74	2.28E-02	9.09E-01	-0.45	1.76E-01	7.32E-01
**KRT42P**	-1.63	1.66E-02	8.11E-01	0.86	1.76E-01	7.32E-01
**AL356275.2**	-0.99	6.94E-01	1.00E+00	-1.80	1.76E-01	7.32E-01
**LRRC31**	-0.30	8.88E-01	1.00E+00	-1.80	1.76E-01	7.32E-01
**MAP3K12**	-0.15	6.33E-01	1.00E+00	1.25	1.76E-01	7.32E-01
**EFEMP2**	0.44	3.46E-01	1.00E+00	0.67	1.77E-01	7.32E-01
**MAEA**	-0.07	7.18E-01	1.00E+00	0.20	1.77E-01	7.32E-01
**AC092155.1**	-2.36	4.01E-01	1.00E+00	-1.85	1.77E-01	7.32E-01
**XLOC_008100**	0.08	8.61E-01	1.00E+00	0.76	1.77E-01	7.32E-01
**TNKS2-AS1**	-0.01	9.92E-01	1.00E+00	-1.18	1.77E-01	7.32E-01
**AC009303.4**	-0.05	9.30E-01	1.00E+00	0.70	1.77E-01	7.32E-01
**RAC1**	0.03	8.53E-01	1.00E+00	-0.60	1.77E-01	7.32E-01
**AC020917.4**	-2.86	6.46E-02	1.00E+00	1.21	1.77E-01	7.32E-01
**AC011603.2**	-1.03	6.58E-01	1.00E+00	-0.95	1.77E-01	7.32E-01
**FUNDC1**	0.02	9.04E-01	1.00E+00	-0.45	1.77E-01	7.32E-01

**XLOC_013984**	-0.76	4.53E-01	1.00E+00	1.87	1.77E-01	7.32E-01
**WNK3**	0.36	5.52E-01	1.00E+00	0.84	1.77E-01	7.32E-01
**AL136115.1**	-1.75	2.25E-01	1.00E+00	1.19	1.77E-01	7.32E-01
**TARS**	-0.01	9.42E-01	1.00E+00	0.26	1.77E-01	7.32E-01
**RPL37P6**	-2.24	2.44E-01	1.00E+00	-0.86	1.77E-01	7.32E-01
**PRKAR2A-AS1**	-0.28	6.01E-01	1.00E+00	-1.06	1.77E-01	7.32E-01
**TNFSF4**	0.41	5.33E-01	1.00E+00	1.15	1.77E-01	7.32E-01
**PXN**	0.20	5.31E-01	1.00E+00	0.53	1.77E-01	7.32E-01
**XPO5**	0.11	6.50E-01	1.00E+00	0.39	1.77E-01	7.32E-01
**ATP6V1B1**	-0.41	7.81E-01	1.00E+00	-1.06	1.77E-01	7.32E-01
**AMPD2**	-0.33	1.29E-01	1.00E+00	0.70	1.77E-01	7.32E-01
**ZBED2**	-0.29	5.59E-01	1.00E+00	0.85	1.77E-01	7.32E-01
**MANEAL**	-0.11	7.69E-01	1.00E+00	-0.88	1.77E-01	7.32E-01
**SMIM13**	-0.02	9.48E-01	1.00E+00	-0.56	1.77E-01	7.32E-01
**SCN1B**	-0.28	5.69E-01	1.00E+00	1.12	1.77E-01	7.32E-01
**GSTT2**	0.60	4.40E-01	1.00E+00	0.98	1.77E-01	7.32E-01
**CHSY1**	-0.06	7.55E-01	1.00E+00	0.46	1.77E-01	7.32E-01
**UBAC1**	-0.13	6.40E-01	1.00E+00	-0.31	1.77E-01	7.32E-01
**NXPE1**	-0.89	7.97E-01	1.00E+00	2.23	1.77E-01	7.32E-01
**MEST**	1.51	4.20E-02	1.00E+00	-0.91	1.77E-01	7.32E-01
**GLYAT**	3.86	5.20E-02	1.00E+00	-1.45	1.77E-01	7.32E-01
**WIZ**	-0.42	1.23E-01	1.00E+00	0.70	1.78E-01	7.32E-01
**AL807757.2**	-2.64	1.93E-01	1.00E+00	1.48	1.78E-01	7.32E-01
**AC110597.1**	2.37	2.36E-01	1.00E+00	1.82	1.78E-01	7.32E-01
**CHCHD4**	0.10	6.12E-01	1.00E+00	-0.44	1.78E-01	7.32E-01
**DAB2**	0.16	7.03E-01	1.00E+00	0.68	1.78E-01	7.32E-01
**FGF7**	0.55	3.08E-01	1.00E+00	0.61	1.78E-01	7.32E-01
**PHF10**	0.27	7.95E-02	1.00E+00	-0.57	1.78E-01	7.32E-01
**FAM167B**	0.19	7.53E-01	1.00E+00	0.93	1.78E-01	7.32E-01
**IGFN1**	-2.56	1.36E-01	1.00E+00	2.16	1.78E-01	7.32E-01
**SIGLEC9**	-0.53	5.72E-01	1.00E+00	1.44	1.78E-01	7.32E-01
**C11orf95**	0.19	6.54E-01	1.00E+00	0.92	1.78E-01	7.32E-01

**DBX2**	0.97	3.05E-01	1.00E+00	-1.34	1.78E-01	7.32E-01
**HOXA5**	-0.48	3.07E-01	1.00E+00	-0.57	1.78E-01	7.33E-01
**GPR176**	0.27	5.11E-01	1.00E+00	0.88	1.78E-01	7.33E-01
**Z95115.1**	0.04	9.10E-01	1.00E+00	-0.69	1.78E-01	7.33E-01
**ACAN**	-0.42	6.07E-01	1.00E+00	1.53	1.78E-01	7.33E-01
**TYMP**	-0.49	5.04E-01	1.00E+00	1.32	1.78E-01	7.33E-01
**SPECC1L**	0.20	4.65E-01	1.00E+00	0.65	1.78E-01	7.33E-01
**G2E3**	0.72	1.36E-01	1.00E+00	-0.45	1.78E-01	7.33E-01
**GATA3**	0.11	7.93E-01	1.00E+00	-0.75	1.78E-01	7.33E-01
**NBPF9**	-0.12	7.10E-01	1.00E+00	0.55	1.78E-01	7.33E-01
**ARL6IP1**	0.60	2.28E-03	2.42E-01	-0.41	1.78E-01	7.33E-01
**AP2A1**	-0.16	5.30E-01	1.00E+00	0.80	1.78E-01	7.33E-01
**RINL**	-0.15	6.14E-01	1.00E+00	0.32	1.78E-01	7.33E-01
**HTATSF1**	0.10	6.52E-01	1.00E+00	-0.31	1.79E-01	7.33E-01
**AP000547.3**	-0.13	7.73E-01	1.00E+00	-0.72	1.79E-01	7.33E-01
**ZNF781**	0.29	7.68E-01	1.00E+00	-1.14	1.79E-01	7.33E-01
**ID1**	-0.81	3.35E-02	1.00E+00	0.43	1.79E-01	7.33E-01
**NPIPB13**	-0.51	6.82E-01	1.00E+00	0.79	1.79E-01	7.33E-01
**AC132008.2**	-0.02	9.31E-01	1.00E+00	-0.35	1.79E-01	7.33E-01
**GRK5**	0.16	7.12E-01	1.00E+00	0.90	1.79E-01	7.33E-01
**AC060766.1**	1.14	1.75E-01	1.00E+00	1.12	1.79E-01	7.33E-01
**APOL1**	-0.90	1.42E-01	1.00E+00	1.01	1.79E-01	7.33E-01
**SLCO6A1**	-0.80	8.18E-01	1.00E+00	-3.87	1.79E-01	7.33E-01
**AC009005.1**	-2.69	2.51E-01	1.00E+00	1.11	1.79E-01	7.33E-01
**LYNX1**	-0.30	3.27E-01	1.00E+00	0.72	1.79E-01	7.33E-01
**AC048382.5**	1.29	2.98E-01	1.00E+00	-0.99	1.79E-01	7.33E-01
**AACSP1**	-0.81	4.38E-01	1.00E+00	2.00	1.79E-01	7.33E-01
**AP000640.1**	0.56	6.22E-01	1.00E+00	0.81	1.79E-01	7.33E-01
**LRRCC1**	0.23	5.70E-01	1.00E+00	-0.33	1.79E-01	7.34E-01
**XLOC_007531**	-2.21	5.19E-01	1.00E+00	2.48	1.79E-01	7.34E-01
**ABL1**	0.19	4.86E-01	1.00E+00	0.80	1.79E-01	7.34E-01
**CLUHP3**	0.15	8.13E-01	1.00E+00	0.74	1.79E-01	7.34E-01

**AC103769.1**	-0.13	9.20E-01	1.00E+00	1.08	1.79E-01	7.34E-01
**RP11-203I2.1**	-2.01	4.55E-02	1.00E+00	0.85	1.79E-01	7.34E-01
**SERTAD4-AS1**	-0.02	9.62E-01	1.00E+00	-0.58	1.79E-01	7.34E-01
**CCDC38**	-1.59	6.03E-01	1.00E+00	-1.37	1.79E-01	7.34E-01
**TSC22D2**	-0.26	2.44E-01	1.00E+00	0.43	1.80E-01	7.34E-01
**JRK**	0.33	4.90E-01	1.00E+00	0.54	1.80E-01	7.34E-01
**RRP8**	-0.20	4.32E-01	1.00E+00	-0.22	1.80E-01	7.34E-01
**SLC25A16**	0.27	5.07E-01	1.00E+00	-0.56	1.80E-01	7.34E-01
**UFSP2**	0.12	5.82E-01	1.00E+00	-0.35	1.80E-01	7.34E-01
**NCK1-DT**	-0.29	5.42E-01	1.00E+00	-0.61	1.80E-01	7.34E-01
**AC007613.1**	-0.25	8.93E-01	1.00E+00	-1.35	1.80E-01	7.34E-01
**XLOC_010031**	-0.92	7.64E-01	1.00E+00	-1.48	1.80E-01	7.34E-01
**ZMYM6**	0.41	1.62E-01	1.00E+00	-0.36	1.80E-01	7.34E-01
**C18orf25**	-0.03	9.05E-01	1.00E+00	0.32	1.80E-01	7.34E-01
**LDHA**	-0.04	8.27E-01	1.00E+00	-0.27	1.80E-01	7.34E-01
**DPYSL3**	0.36	3.34E-01	1.00E+00	0.75	1.80E-01	7.34E-01
**LINC01089**	-0.07	9.26E-01	1.00E+00	-0.54	1.80E-01	7.34E-01
**VDAC1**	-0.13	3.71E-01	1.00E+00	-0.18	1.80E-01	7.34E-01
**ELFN2**	-1.19	2.48E-01	1.00E+00	1.48	1.80E-01	7.34E-01
**TWIST1**	0.01	9.67E-01	1.00E+00	0.84	1.80E-01	7.34E-01
**MBOAT1**	0.52	3.27E-01	1.00E+00	1.08	1.80E-01	7.34E-01
**DISC1FP1**	-2.61	3.48E-01	1.00E+00	-1.93	1.80E-01	7.34E-01
**INPP1**	0.18	5.23E-01	1.00E+00	-0.42	1.80E-01	7.34E-01
**DLST**	0.17	4.07E-01	1.00E+00	0.76	1.80E-01	7.34E-01
**AC087289.5**	0.76	6.21E-01	1.00E+00	-1.57	1.80E-01	7.34E-01
**GSKIP**	0.30	4.38E-01	1.00E+00	-0.52	1.80E-01	7.35E-01
**SLC12A9-AS1**	-0.86	3.77E-01	1.00E+00	-0.71	1.80E-01	7.35E-01
**AP4B1**	0.10	7.49E-01	1.00E+00	-0.28	1.80E-01	7.35E-01
**HIST2H2AB**	-2.30	4.98E-01	1.00E+00	1.82	1.80E-01	7.35E-01
**ZCCHC14**	-0.24	3.94E-01	1.00E+00	0.63	1.80E-01	7.35E-01
**AC010616.1**	-0.37	7.71E-01	1.00E+00	2.00	1.80E-01	7.35E-01
**JUN**	-1.21	3.79E-02	1.00E+00	0.70	1.80E-01	7.35E-01

**NMD3P1**	0.36	7.29E-01	1.00E+00	1.47	1.81E-01	7.35E-01
**OXNAD1**	0.11	6.28E-01	1.00E+00	-0.27	1.81E-01	7.35E-01
**SNRPGP15**	-1.84	2.25E-01	1.00E+00	-0.54	1.81E-01	7.35E-01
**AC095057.3**	-0.83	4.72E-01	1.00E+00	-1.21	1.81E-01	7.35E-01
**XLOC_014397**	-0.80	5.03E-01	1.00E+00	1.35	1.81E-01	7.35E-01
**NBPF12**	0.41	2.98E-01	1.00E+00	0.54	1.81E-01	7.36E-01
**ATP11C**	0.13	5.62E-01	1.00E+00	0.62	1.81E-01	7.36E-01
**AL109766.1**	0.55	8.46E-01	1.00E+00	-1.05	1.81E-01	7.36E-01
**SLC29A4**	2.00	1.07E-01	1.00E+00	-1.04	1.81E-01	7.36E-01
**EPC1**	-0.05	7.87E-01	1.00E+00	0.27	1.81E-01	7.36E-01
**UBL5**	0.11	4.54E-01	1.00E+00	-0.37	1.81E-01	7.36E-01
**BTBD6**	0.08	6.86E-01	1.00E+00	-0.68	1.81E-01	7.36E-01
**EVA1C**	-0.19	5.37E-01	1.00E+00	-0.29	1.81E-01	7.36E-01
**SDHB**	-0.06	7.31E-01	1.00E+00	-0.36	1.81E-01	7.36E-01
**AURKC**	-0.05	9.28E-01	1.00E+00	0.97	1.81E-01	7.36E-01
**AC005498.2**	-1.61	3.02E-01	1.00E+00	-0.90	1.81E-01	7.36E-01
**MT2A**	-0.66	1.81E-01	1.00E+00	0.52	1.81E-01	7.36E-01
**AL121894.2**	1.33	1.41E-01	1.00E+00	1.36	1.81E-01	7.36E-01
**IYD**	-1.71	2.50E-01	1.00E+00	-1.65	1.81E-01	7.36E-01
**LRRC34**	0.84	2.89E-01	1.00E+00	-0.69	1.81E-01	7.36E-01
**ATP5F1EP2**	NA	NA	NA	-0.80	1.81E-01	7.36E-01
**MDH1B**	-0.39	5.27E-01	1.00E+00	-1.07	1.81E-01	7.36E-01
**TMEM199**	0.02	9.24E-01	1.00E+00	-0.30	1.81E-01	7.36E-01
**SPATS2**	-0.13	5.99E-01	1.00E+00	-0.35	1.81E-01	7.36E-01
**ZNF8**	0.16	7.38E-01	1.00E+00	0.49	1.81E-01	7.36E-01
**NT5C3AP1**	-0.58	8.67E-01	1.00E+00	0.97	1.81E-01	7.36E-01
**RP11-397O4.1**	-0.56	8.73E-01	1.00E+00	4.21	1.81E-01	7.36E-01
**ZBTB42**	-0.02	9.50E-01	1.00E+00	0.81	1.81E-01	7.36E-01
**LINC01562**	0.13	9.71E-01	1.00E+00	1.90	1.82E-01	7.36E-01
**AC064807.1**	-0.42	2.27E-01	1.00E+00	-0.34	1.82E-01	7.36E-01
**AC099789.1**	NA	NA	NA	-1.24	1.82E-01	7.36E-01
**OLFM2**	-0.17	5.39E-01	1.00E+00	-0.53	1.82E-01	7.36E-01

**GALNT6**	-1.49	2.20E-03	2.39E-01	0.80	1.82E-01	7.36E-01
**AC020915.1**	-1.35	2.63E-01	1.00E+00	1.19	1.82E-01	7.36E-01
**FARP1**	-0.20	6.31E-01	1.00E+00	0.64	1.82E-01	7.36E-01
**ZNF543**	-0.23	4.90E-01	1.00E+00	-0.33	1.82E-01	7.36E-01
**LETM2**	0.24	6.27E-01	1.00E+00	0.64	1.82E-01	7.36E-01
**AC058791.1**	0.16	8.80E-01	1.00E+00	0.82	1.82E-01	7.36E-01
**NLRP12**	-0.43	6.73E-01	1.00E+00	1.81	1.82E-01	7.36E-01
**AC130895.1**	1.68	6.26E-01	1.00E+00	-1.17	1.82E-01	7.36E-01
**FBN1**	0.24	6.72E-01	1.00E+00	0.81	1.82E-01	7.36E-01
**AL359764.1**	-1.48	2.88E-01	1.00E+00	-2.32	1.82E-01	7.36E-01
**NEMP2**	0.47	8.36E-02	1.00E+00	0.47	1.82E-01	7.36E-01
**AC069209.1**	-0.68	4.93E-01	1.00E+00	-1.30	1.82E-01	7.37E-01
**GPR150**	0.33	6.34E-01	1.00E+00	1.30	1.82E-01	7.37E-01
**CDC26**	0.28	2.88E-01	1.00E+00	-0.48	1.82E-01	7.37E-01
**TCF25**	-0.05	7.66E-01	1.00E+00	0.21	1.82E-01	7.37E-01
**AC083798.2**	-0.47	2.71E-01	1.00E+00	-0.66	1.82E-01	7.37E-01
**XLOC_013952**	-0.21	8.36E-01	1.00E+00	1.21	1.82E-01	7.37E-01
**RGMA**	-0.19	7.10E-01	1.00E+00	0.75	1.82E-01	7.37E-01
**PLXDC2**	-0.23	4.28E-01	1.00E+00	0.32	1.82E-01	7.37E-01
**TPT1P4**	0.65	6.49E-01	1.00E+00	-0.76	1.82E-01	7.37E-01
**BATF**	-0.33	5.39E-01	1.00E+00	1.11	1.83E-01	7.37E-01
**AF131216.4**	-2.39	3.82E-01	1.00E+00	1.70	1.83E-01	7.37E-01
**NOS2**	0.41	6.44E-01	1.00E+00	-1.48	1.83E-01	7.37E-01
**MRPL48**	-0.04	8.16E-01	1.00E+00	-0.30	1.83E-01	7.37E-01
**XLOC_013591**	-0.39	8.29E-01	1.00E+00	1.38	1.83E-01	7.37E-01
**EMC10**	-0.23	2.72E-01	1.00E+00	0.63	1.83E-01	7.37E-01
**HECW2**	-0.30	5.27E-01	1.00E+00	0.72	1.83E-01	7.37E-01
**TIMM9**	0.21	2.53E-01	1.00E+00	-0.33	1.83E-01	7.37E-01
**TMEM59**	0.17	3.26E-01	1.00E+00	-0.43	1.83E-01	7.37E-01
**LINC00517**	-1.04	2.56E-01	1.00E+00	1.23	1.83E-01	7.37E-01
**AC010148.1**	-2.45	2.30E-01	1.00E+00	1.23	1.83E-01	7.37E-01
**AP003119.2**	-0.55	4.01E-01	1.00E+00	-0.94	1.83E-01	7.37E-01

**LINC01788**	NA	NA	NA	2.41	1.83E-01	7.37E-01
**ISCU**	0.05	8.25E-01	1.00E+00	-0.39	1.83E-01	7.37E-01
**XLOC_011112**	3.97	3.98E-02	1.00E+00	-1.76	1.83E-01	7.37E-01
**AL096816.1**	-0.50	8.46E-01	1.00E+00	2.04	1.83E-01	7.37E-01
**GNG2**	0.55	2.61E-01	1.00E+00	-0.62	1.83E-01	7.37E-01
**C22orf46**	-0.22	4.57E-01	1.00E+00	0.68	1.83E-01	7.37E-01
**LINC00643**	1.07	6.29E-01	1.00E+00	-1.94	1.83E-01	7.37E-01
**TRABD**	-0.17	4.78E-01	1.00E+00	0.67	1.83E-01	7.37E-01
**FAM86HP**	0.11	8.34E-01	1.00E+00	-0.70	1.83E-01	7.37E-01
**AC118553.2**	-1.19	7.30E-01	1.00E+00	0.97	1.83E-01	7.37E-01
**RPS7P14**	NA	NA	NA	-1.17	1.83E-01	7.37E-01
**Z93930.2**	-0.05	9.10E-01	1.00E+00	-0.60	1.83E-01	7.37E-01
**STXBP4**	-0.14	6.50E-01	1.00E+00	-0.53	1.83E-01	7.37E-01
**TERF2IP**	0.13	4.04E-01	1.00E+00	-0.62	1.83E-01	7.37E-01
**AC083841.1**	0.54	6.89E-01	1.00E+00	0.93	1.83E-01	7.37E-01
**MAP4K4**	-0.07	7.85E-01	1.00E+00	0.70	1.83E-01	7.37E-01
**PIK3IP1-AS1**	-0.36	8.98E-01	1.00E+00	-1.23	1.84E-01	7.37E-01
**ING2**	-0.53	4.44E-02	1.00E+00	-0.35	1.84E-01	7.37E-01
**ITPRID1**	-0.04	9.63E-01	1.00E+00	-1.02	1.84E-01	7.37E-01
**AL162430.1**	-2.01	5.20E-01	1.00E+00	-0.96	1.84E-01	7.37E-01
**PRUNE1**	0.36	7.72E-02	1.00E+00	-0.38	1.84E-01	7.37E-01
**FSCN1**	-0.87	2.82E-04	6.27E-02	0.78	1.84E-01	7.37E-01
**NOL11**	-0.05	8.39E-01	1.00E+00	-0.21	1.84E-01	7.38E-01
**RAD51C**	0.11	6.77E-01	1.00E+00	-0.45	1.84E-01	7.38E-01
**IGFBP3**	1.02	6.39E-03	4.79E-01	0.56	1.84E-01	7.38E-01
**AWAT1**	0.37	8.33E-01	1.00E+00	-1.60	1.84E-01	7.38E-01
**DIAPH2**	-0.17	4.26E-01	1.00E+00	-0.36	1.84E-01	7.38E-01
**ALDH3A2**	0.27	3.25E-01	1.00E+00	-0.39	1.84E-01	7.38E-01
**RHOBTB2**	0.34	4.26E-01	1.00E+00	0.79	1.84E-01	7.38E-01
**C10orf143**	-0.22	4.70E-01	1.00E+00	-0.33	1.84E-01	7.38E-01
**VSTM2A**	-0.72	5.00E-01	1.00E+00	1.50	1.84E-01	7.38E-01
**EI24P2**	NA	NA	NA	-1.13	1.84E-01	7.38E-01

**ADAM33**	0.32	4.21E-01	1.00E+00	0.81	1.84E-01	7.39E-01
**CCDC80**	0.71	2.82E-01	1.00E+00	0.71	1.84E-01	7.39E-01
**SLC35F6**	-0.03	9.07E-01	1.00E+00	0.44	1.84E-01	7.39E-01
**PPP2R2A**	0.04	8.43E-01	1.00E+00	0.30	1.84E-01	7.39E-01
**AKTIP**	0.10	6.69E-01	1.00E+00	-0.33	1.84E-01	7.39E-01
**RCAN3**	0.12	7.07E-01	1.00E+00	-0.36	1.84E-01	7.39E-01
**XPOTP1**	-0.80	7.72E-01	1.00E+00	-1.43	1.84E-01	7.39E-01
**PEX2**	0.34	4.50E-02	1.00E+00	-0.39	1.84E-01	7.39E-01
**TMEM183A**	0.02	9.16E-01	1.00E+00	-0.43	1.84E-01	7.39E-01
**AC114980.1**	0.16	8.25E-01	1.00E+00	1.05	1.85E-01	7.39E-01
**CCDC26**	-1.96	3.05E-01	1.00E+00	-0.77	1.85E-01	7.39E-01
**CABLES1**	0.47	4.64E-02	1.00E+00	-0.57	1.85E-01	7.39E-01
**XLOC_000633**	1.51	6.60E-01	1.00E+00	-1.93	1.85E-01	7.39E-01
**CYR61**	-1.04	1.07E-04	2.56E-02	0.64	1.85E-01	7.39E-01
**PCDHB14**	-0.25	3.93E-01	1.00E+00	-0.64	1.85E-01	7.39E-01
**AL031282.2**	0.05	9.62E-01	1.00E+00	-0.69	1.85E-01	7.39E-01
**ZKSCAN2-DT**	0.53	7.62E-01	1.00E+00	1.70	1.85E-01	7.39E-01
**SRFBP1**	-0.25	2.36E-01	1.00E+00	-0.34	1.85E-01	7.39E-01
**FOXP2**	0.32	3.28E-01	1.00E+00	-0.49	1.85E-01	7.39E-01
**ECI1**	-0.14	6.13E-01	1.00E+00	-0.85	1.85E-01	7.39E-01
**UPF3A**	0.06	7.79E-01	1.00E+00	0.32	1.85E-01	7.39E-01
**AL136141.1**	2.44	2.98E-01	1.00E+00	-1.61	1.85E-01	7.39E-01
**NTS**	-0.48	8.57E-01	1.00E+00	1.12	1.85E-01	7.39E-01
**YEATS2**	0.23	5.38E-01	1.00E+00	0.67	1.85E-01	7.39E-01
**GPR149**	-3.86	2.30E-02	9.12E-01	1.94	1.85E-01	7.39E-01
**NUCB1**	0.01	9.77E-01	1.00E+00	0.46	1.85E-01	7.39E-01
**RAPGEF4**	-0.03	9.31E-01	1.00E+00	0.36	1.85E-01	7.39E-01
**TSC22D1-AS1**	-0.67	5.77E-01	1.00E+00	1.33	1.85E-01	7.39E-01
**BTBD10**	0.22	3.80E-01	1.00E+00	0.22	1.85E-01	7.39E-01
**OGFR-AS1**	-1.00	6.27E-01	1.00E+00	1.68	1.85E-01	7.39E-01
**XLOC_014080**	-0.24	7.87E-01	1.00E+00	1.12	1.85E-01	7.39E-01
**FCGRT**	0.28	3.15E-01	1.00E+00	0.39	1.85E-01	7.39E-01

**GOT1**	0.04	8.85E-01	1.00E+00	-0.24	1.85E-01	7.39E-01
**LINC00612**	-0.03	9.77E-01	1.00E+00	1.14	1.85E-01	7.39E-01
**AC018904.1**	0.19	8.51E-01	1.00E+00	1.13	1.86E-01	7.39E-01
**SOX30**	2.32	4.96E-02	1.00E+00	1.44	1.86E-01	7.39E-01
**G3BP2**	-0.06	7.71E-01	1.00E+00	-0.32	1.86E-01	7.39E-01
**MBD3**	-0.16	4.78E-01	1.00E+00	0.55	1.86E-01	7.39E-01
**MXRA8**	0.23	5.86E-01	1.00E+00	0.83	1.86E-01	7.39E-01
**KRT18P7**	-0.52	8.80E-01	1.00E+00	-2.03	1.86E-01	7.40E-01
**TMEM135**	0.56	3.11E-01	1.00E+00	-0.84	1.86E-01	7.40E-01
**GPI**	-0.07	7.93E-01	1.00E+00	-0.21	1.86E-01	7.40E-01
**TTN-AS1**	-0.32	6.49E-01	1.00E+00	0.49	1.86E-01	7.40E-01
**PDE1C**	0.34	7.36E-01	1.00E+00	1.18	1.86E-01	7.40E-01
**DLEU2**	0.03	9.55E-01	1.00E+00	-0.55	1.86E-01	7.40E-01
**LINC01537**	0.45	5.50E-01	1.00E+00	1.13	1.86E-01	7.40E-01
**BST1**	0.48	1.56E-01	1.00E+00	0.43	1.86E-01	7.40E-01
**TMEM105**	-0.13	8.94E-01	1.00E+00	0.98	1.86E-01	7.40E-01
**AL645608.1**	-3.22	3.41E-01	1.00E+00	1.80	1.86E-01	7.40E-01
**ABCA2**	-0.27	4.22E-01	1.00E+00	0.77	1.86E-01	7.40E-01
**KDM6B**	-0.49	5.74E-02	1.00E+00	0.84	1.86E-01	7.40E-01
**TTC34**	-0.50	6.24E-01	1.00E+00	1.88	1.86E-01	7.40E-01
**UNG**	0.10	6.66E-01	1.00E+00	-0.31	1.86E-01	7.40E-01
**OXLD1**	-0.08	7.41E-01	1.00E+00	-0.33	1.86E-01	7.40E-01
**TRGV3**	0.67	7.97E-01	1.00E+00	1.16	1.86E-01	7.40E-01
**ZBTB24**	-0.21	4.62E-01	1.00E+00	-0.45	1.86E-01	7.40E-01
**RPA2**	-0.09	7.37E-01	1.00E+00	-0.40	1.86E-01	7.40E-01
**IL18R1**	0.04	9.35E-01	1.00E+00	0.49	1.86E-01	7.40E-01
**C16orf87**	0.08	7.69E-01	1.00E+00	-0.36	1.86E-01	7.40E-01
**LINC01389**	-0.95	6.14E-01	1.00E+00	-1.58	1.86E-01	7.40E-01
**G38630**	-0.35	8.16E-01	1.00E+00	-1.81	1.86E-01	7.40E-01
**DTD1**	0.06	7.59E-01	1.00E+00	-0.35	1.87E-01	7.41E-01
**ZNF487**	-0.33	2.89E-01	1.00E+00	-0.45	1.87E-01	7.41E-01
**WNT16**	0.10	8.16E-01	1.00E+00	-0.97	1.87E-01	7.41E-01

**EPG5**	0.16	5.38E-01	1.00E+00	0.63	1.87E-01	7.41E-01
**MIR583HG**	2.12	1.95E-01	1.00E+00	-1.62	1.87E-01	7.41E-01
**PTP4A2**	0.23	3.95E-01	1.00E+00	-0.48	1.87E-01	7.41E-01
**SVEP1**	1.07	4.52E-02	1.00E+00	0.82	1.87E-01	7.41E-01
**AC079414.3**	0.35	6.11E-01	1.00E+00	-0.73	1.87E-01	7.41E-01
**CPT1A**	0.03	9.28E-01	1.00E+00	0.86	1.87E-01	7.41E-01
**G34382**	-0.22	8.68E-01	1.00E+00	-1.26	1.87E-01	7.41E-01
**PELO**	-0.25	1.32E-01	1.00E+00	0.20	1.87E-01	7.41E-01
**NKAPP1**	-0.09	8.12E-01	1.00E+00	-0.36	1.87E-01	7.41E-01
**AC012186.3**	0.56	8.71E-01	1.00E+00	2.37	1.87E-01	7.41E-01
**PSRC1**	-0.01	9.74E-01	1.00E+00	0.60	1.87E-01	7.41E-01
**TIMM50**	-0.20	2.62E-01	1.00E+00	0.17	1.87E-01	7.41E-01
**GJB1**	-1.07	9.47E-02	1.00E+00	-0.84	1.87E-01	7.41E-01
**HSF1**	-0.16	4.93E-01	1.00E+00	0.45	1.87E-01	7.41E-01
**G32232**	-3.30	1.13E-01	1.00E+00	-1.38	1.87E-01	7.41E-01
**GTF2IP4**	-0.10	7.65E-01	1.00E+00	-0.50	1.87E-01	7.41E-01
**MAN2B1**	-0.47	3.19E-02	1.00E+00	0.71	1.87E-01	7.41E-01
**HEATR6**	0.27	2.38E-01	1.00E+00	0.41	1.87E-01	7.41E-01
**TUB**	0.35	5.21E-01	1.00E+00	0.79	1.87E-01	7.41E-01
**IFIT5**	0.18	7.48E-01	1.00E+00	0.45	1.87E-01	7.41E-01
**HSD11B2**	-1.21	2.51E-02	9.28E-01	-0.76	1.87E-01	7.41E-01
**ATG13**	0.06	7.76E-01	1.00E+00	0.27	1.87E-01	7.41E-01
**POM121L9P**	-0.57	4.51E-01	1.00E+00	1.22	1.87E-01	7.41E-01
**APMAP**	0.34	4.17E-01	1.00E+00	-0.46	1.87E-01	7.41E-01
**HSPE1**	-0.01	9.74E-01	1.00E+00	-0.69	1.88E-01	7.41E-01
**XLOC_007718**	0.65	7.39E-01	1.00E+00	-1.29	1.88E-01	7.41E-01
**AC092376.1**	-0.12	9.73E-01	1.00E+00	1.60	1.88E-01	7.41E-01
**HIST1H4I**	0.21	7.21E-01	1.00E+00	-0.35	1.88E-01	7.41E-01
**ACER2**	0.34	4.65E-01	1.00E+00	0.93	1.88E-01	7.41E-01
**RPS6KB2**	-0.08	8.39E-01	1.00E+00	0.43	1.88E-01	7.41E-01
**ARF1**	0.00	9.95E-01	1.00E+00	0.15	1.88E-01	7.41E-01
**SLAIN2**	-0.14	6.24E-01	1.00E+00	-0.33	1.88E-01	7.41E-01

**AL513304.1**	-0.12	9.73E-01	1.00E+00	2.52	1.88E-01	7.41E-01
**RLIM**	0.00	9.92E-01	1.00E+00	0.28	1.88E-01	7.41E-01
**G31356**	-1.37	3.02E-01	1.00E+00	-0.89	1.88E-01	7.42E-01
**XAGE3**	NA	NA	NA	-2.57	1.88E-01	7.42E-01
**AL137784.1**	0.65	3.50E-01	1.00E+00	-0.89	1.88E-01	7.42E-01
**IGHV4-39**	7.65	4.27E-06	1.39E-03	4.14	1.88E-01	7.42E-01
**MAGEE2**	-0.54	6.31E-01	1.00E+00	1.80	1.88E-01	7.42E-01
**WTAP**	-0.04	8.34E-01	1.00E+00	0.25	1.88E-01	7.42E-01
**EFCAB7**	-0.31	3.22E-01	1.00E+00	-0.51	1.88E-01	7.42E-01
**XLOC_005571**	0.31	6.65E-01	1.00E+00	1.48	1.88E-01	7.42E-01
**AC019205.1**	-0.56	1.72E-01	1.00E+00	-0.41	1.88E-01	7.42E-01
**AC013565.1**	-3.16	3.09E-02	1.00E+00	-1.23	1.88E-01	7.42E-01
**TMEM191A**	-0.10	7.27E-01	1.00E+00	0.42	1.88E-01	7.42E-01
**PRCC**	-0.31	1.66E-01	1.00E+00	0.27	1.88E-01	7.42E-01
**NDUFA4**	0.02	9.35E-01	1.00E+00	-0.49	1.88E-01	7.42E-01
**FRAS1**	-0.45	4.30E-01	1.00E+00	1.04	1.89E-01	7.42E-01
**EP400P1**	-0.63	1.70E-01	1.00E+00	0.46	1.89E-01	7.42E-01
**G38642**	4.62	2.34E-02	9.15E-01	-1.65	1.89E-01	7.42E-01
**ARSI**	-0.40	3.65E-01	1.00E+00	0.92	1.89E-01	7.42E-01
**SYVN1**	-0.07	7.48E-01	1.00E+00	0.54	1.89E-01	7.42E-01
**KHK**	0.11	6.21E-01	1.00E+00	-0.40	1.89E-01	7.42E-01
**HS3ST2**	0.49	4.08E-01	1.00E+00	1.10	1.89E-01	7.42E-01
**RNF144A-AS1**	-3.28	1.26E-02	7.01E-01	1.32	1.89E-01	7.43E-01
**NMT2**	0.55	7.45E-02	1.00E+00	-0.51	1.89E-01	7.43E-01
**HIST2H4A**	-1.35	1.88E-01	1.00E+00	-0.78	1.89E-01	7.43E-01
**CYP7A1**	0.48	8.88E-01	1.00E+00	-2.29	1.89E-01	7.43E-01
**GFER**	0.07	8.04E-01	1.00E+00	0.63	1.89E-01	7.44E-01
**RPL29**	-0.28	3.52E-01	1.00E+00	0.38	1.89E-01	7.44E-01
**AL137026.1**	-2.29	3.84E-01	1.00E+00	-1.16	1.89E-01	7.44E-01
**AL513165.1**	-0.24	6.80E-01	1.00E+00	0.99	1.89E-01	7.44E-01
**AC234781.1**	0.70	5.28E-01	1.00E+00	0.77	1.89E-01	7.44E-01
**TNFRSF1B**	0.28	4.56E-01	1.00E+00	0.92	1.90E-01	7.44E-01

**ZHX2**	0.18	5.28E-01	1.00E+00	0.34	1.90E-01	7.44E-01
**CEP350**	-0.25	3.40E-01	1.00E+00	-0.36	1.90E-01	7.44E-01
**AL009174.1**	-0.80	4.04E-01	1.00E+00	-0.77	1.90E-01	7.45E-01
**ZNF668**	-0.23	4.07E-01	1.00E+00	0.53	1.90E-01	7.45E-01
**AREL1**	0.05	8.19E-01	1.00E+00	0.51	1.90E-01	7.45E-01
**LPCAT1**	-0.15	6.45E-01	1.00E+00	0.88	1.90E-01	7.45E-01
**AL133163.1**	1.19	5.71E-01	1.00E+00	-1.66	1.90E-01	7.45E-01
**GPAM**	3.96	5.64E-03	4.46E-01	-1.00	1.90E-01	7.45E-01
**SCML1**	0.07	8.87E-01	1.00E+00	-0.42	1.90E-01	7.45E-01
**AC016866.1**	-0.53	5.31E-01	1.00E+00	0.86	1.90E-01	7.45E-01
**NAE1**	-0.20	3.42E-01	1.00E+00	-0.41	1.90E-01	7.45E-01
**OR7E15P**	0.50	7.91E-01	1.00E+00	-2.26	1.90E-01	7.45E-01
**SOX6**	0.01	9.74E-01	1.00E+00	-0.50	1.90E-01	7.45E-01
**SDSL**	-0.18	6.47E-01	1.00E+00	0.78	1.90E-01	7.45E-01
**GPT**	0.08	8.55E-01	1.00E+00	-0.47	1.90E-01	7.45E-01
**DLG1**	0.41	5.08E-02	1.00E+00	-0.73	1.90E-01	7.45E-01
**RTN1**	-0.58	1.53E-01	1.00E+00	-1.00	1.90E-01	7.45E-01
**UNC50**	-0.01	9.53E-01	1.00E+00	-0.40	1.90E-01	7.45E-01
**FLOT1**	0.18	6.39E-01	1.00E+00	0.49	1.90E-01	7.45E-01
**GCNT7**	-0.09	9.69E-01	1.00E+00	-1.34	1.90E-01	7.45E-01
**HERC2P2**	-0.34	5.84E-01	1.00E+00	0.81	1.91E-01	7.45E-01
**SCTR**	1.19	5.66E-01	1.00E+00	-1.28	1.91E-01	7.45E-01
**HSPE1P25**	-0.80	8.18E-01	1.00E+00	-0.99	1.91E-01	7.45E-01
**GTF2IP20**	-0.05	9.35E-01	1.00E+00	0.91	1.91E-01	7.45E-01
**XLOC_000019**	-0.72	1.02E-01	1.00E+00	0.80	1.91E-01	7.45E-01
**AC034199.1**	NA	NA	NA	2.39	1.91E-01	7.45E-01
**AC062017.1**	0.14	8.77E-01	1.00E+00	-0.88	1.91E-01	7.45E-01
**BEX5**	-0.60	1.49E-01	1.00E+00	-0.43	1.91E-01	7.45E-01
**ZBTB38**	0.04	8.24E-01	1.00E+00	0.20	1.91E-01	7.45E-01
**DGAT2**	1.41	3.82E-03	3.38E-01	-0.67	1.91E-01	7.45E-01
**SRSF10**	0.12	7.05E-01	1.00E+00	-0.34	1.91E-01	7.45E-01
**TMEM230**	0.18	5.87E-01	1.00E+00	-0.47	1.91E-01	7.45E-01

**SPOPL**	0.08	8.24E-01	1.00E+00	0.31	1.91E-01	7.45E-01
**ZNF787**	-0.11	6.80E-01	1.00E+00	1.01	1.91E-01	7.45E-01
**AL158206.1**	0.95	3.55E-02	1.00E+00	-0.65	1.91E-01	7.45E-01
**AC009961.1**	-0.85	2.71E-01	1.00E+00	-0.68	1.91E-01	7.45E-01
**DMRT3**	-0.12	8.87E-01	1.00E+00	1.11	1.91E-01	7.45E-01
**CLEC2B**	0.15	7.03E-01	1.00E+00	-0.61	1.91E-01	7.45E-01
**WDR86-AS1**	-0.13	9.25E-01	1.00E+00	-1.22	1.91E-01	7.46E-01
**CIDECP**	0.20	4.49E-01	1.00E+00	-0.41	1.91E-01	7.46E-01
**EIF5AL1**	-0.67	3.70E-01	1.00E+00	-0.62	1.91E-01	7.46E-01
**ATP6AP1**	-0.08	6.91E-01	1.00E+00	0.28	1.91E-01	7.46E-01
**SH3GLB1**	0.10	6.41E-01	1.00E+00	-0.15	1.91E-01	7.46E-01
**MT-ND5**	-0.10	7.98E-01	1.00E+00	-0.44	1.91E-01	7.46E-01
**DACT2**	-0.29	4.26E-01	1.00E+00	-0.89	1.91E-01	7.46E-01
**ZNF383**	0.22	4.60E-01	1.00E+00	-0.41	1.91E-01	7.46E-01
**GMPR2**	0.05	7.70E-01	1.00E+00	0.18	1.91E-01	7.46E-01
**G22255**	-0.96	3.07E-01	1.00E+00	-1.34	1.91E-01	7.46E-01
**AC100854.1**	0.17	8.16E-01	1.00E+00	1.06	1.91E-01	7.46E-01
**PLN**	-0.06	9.36E-01	1.00E+00	-0.65	1.91E-01	7.46E-01
**ZNF615**	0.31	2.62E-01	1.00E+00	-0.41	1.91E-01	7.46E-01
**CERKL**	0.04	9.35E-01	1.00E+00	0.64	1.91E-01	7.46E-01
**AC008555.5**	0.26	6.38E-01	1.00E+00	-0.71	1.92E-01	7.46E-01
**SCRN1**	0.07	8.46E-01	1.00E+00	0.39	1.92E-01	7.46E-01
**AC079140.2**	0.41	8.14E-01	1.00E+00	-0.91	1.92E-01	7.46E-01
**CEP95**	-0.18	5.97E-01	1.00E+00	0.26	1.92E-01	7.46E-01
**AL445223.1**	1.13	7.44E-01	1.00E+00	-2.80	1.92E-01	7.46E-01
**CDH15**	-2.41	1.45E-01	1.00E+00	1.57	1.92E-01	7.46E-01
**ZNF345**	-0.01	9.68E-01	1.00E+00	-0.55	1.92E-01	7.46E-01
**LINC02235**	-1.00	5.36E-01	1.00E+00	-1.44	1.92E-01	7.46E-01
**HMGN3**	0.18	3.54E-01	1.00E+00	-0.56	1.92E-01	7.46E-01
**G29816**	-0.50	6.26E-01	1.00E+00	-1.11	1.92E-01	7.46E-01
**MGST3**	0.08	8.02E-01	1.00E+00	-0.44	1.92E-01	7.46E-01
**ILF3**	-0.09	5.82E-01	1.00E+00	0.44	1.92E-01	7.46E-01

**TAF15**	-0.15	4.96E-01	1.00E+00	0.98	1.92E-01	7.46E-01
**TFCP2L1**	0.15	8.19E-01	1.00E+00	-0.63	1.92E-01	7.46E-01
**BRD2**	-0.09	7.20E-01	1.00E+00	0.51	1.92E-01	7.46E-01
**HS3ST3A1**	-1.76	2.16E-05	6.33E-03	1.36	1.92E-01	7.46E-01
**RHOF**	-0.83	3.95E-01	1.00E+00	0.80	1.92E-01	7.46E-01
**C17orf100**	-0.06	8.55E-01	1.00E+00	-0.53	1.92E-01	7.46E-01
**HAAO**	0.10	7.42E-01	1.00E+00	0.40	1.92E-01	7.46E-01
**AC109347.2**	-1.58	3.88E-01	1.00E+00	0.85	1.92E-01	7.46E-01
**CPNE5**	-0.81	9.61E-02	1.00E+00	0.74	1.92E-01	7.46E-01
**EEF1DP4**	-2.31	8.95E-02	1.00E+00	-1.98	1.92E-01	7.46E-01
**C5orf67**	-1.42	1.59E-01	1.00E+00	-1.49	1.92E-01	7.46E-01
**AL022323.4**	0.34	7.97E-01	1.00E+00	1.08	1.92E-01	7.46E-01
**EFCAB10**	-1.18	2.11E-01	1.00E+00	0.77	1.92E-01	7.46E-01
**MAPK3**	-0.09	6.23E-01	1.00E+00	0.63	1.92E-01	7.46E-01
**COX7A1**	0.26	6.80E-01	1.00E+00	-0.65	1.92E-01	7.46E-01
**XLOC_009459**	-0.09	9.54E-01	1.00E+00	1.12	1.92E-01	7.46E-01
**TIRAP**	0.12	7.79E-01	1.00E+00	0.41	1.92E-01	7.46E-01
**INTS2**	0.24	4.05E-01	1.00E+00	-0.33	1.93E-01	7.46E-01
**QRSL1**	0.08	6.72E-01	1.00E+00	0.28	1.93E-01	7.46E-01
**TNFRSF13C**	-0.45	4.57E-01	1.00E+00	0.78	1.93E-01	7.46E-01
**AL031281.2**	-0.46	6.95E-01	1.00E+00	1.16	1.93E-01	7.46E-01
**LINC01287**	2.74	4.16E-01	1.00E+00	2.39	1.93E-01	7.46E-01
**CBR3-AS1**	0.25	5.06E-01	1.00E+00	-0.58	1.93E-01	7.46E-01
**PICSAR**	-0.46	2.04E-01	1.00E+00	0.88	1.93E-01	7.46E-01
**S100P**	-0.85	1.04E-01	1.00E+00	0.72	1.93E-01	7.46E-01
**AL590560.1**	-0.99	1.16E-01	1.00E+00	-1.25	1.93E-01	7.46E-01
**AL596244.1**	0.14	8.04E-01	1.00E+00	1.17	1.93E-01	7.46E-01
**AC087294.1**	-1.92	2.08E-01	1.00E+00	-1.53	1.93E-01	7.46E-01
**TEF**	-0.47	1.80E-01	1.00E+00	-0.49	1.93E-01	7.46E-01
**PALD1**	0.22	4.75E-01	1.00E+00	1.01	1.93E-01	7.46E-01
**AC011447.3**	0.72	5.09E-01	1.00E+00	1.38	1.93E-01	7.46E-01
**RUFY3**	-0.31	2.05E-01	1.00E+00	-0.37	1.93E-01	7.46E-01

**BRWD1-IT2**	-0.15	8.19E-01	1.00E+00	-1.07	1.93E-01	7.46E-01
**FTH1P7**	0.53	5.31E-01	1.00E+00	-0.72	1.93E-01	7.46E-01
**TMEM243**	0.30	1.67E-01	1.00E+00	-0.30	1.93E-01	7.46E-01
**ANKRD20A7P**	1.22	3.45E-01	1.00E+00	1.38	1.93E-01	7.46E-01
**TESC**	0.08	9.07E-01	1.00E+00	-0.79	1.93E-01	7.46E-01
**SMG7**	-0.04	8.49E-01	1.00E+00	0.37	1.93E-01	7.46E-01
**SPOP**	0.33	1.03E-01	1.00E+00	-0.23	1.93E-01	7.46E-01
**AC104563.1**	0.63	6.52E-01	1.00E+00	-0.84	1.93E-01	7.46E-01
**CCR10**	-0.45	6.05E-01	1.00E+00	0.75	1.93E-01	7.46E-01
**AC245060.4**	-0.07	8.09E-01	1.00E+00	-0.36	1.93E-01	7.46E-01
**FAM72C**	-0.15	9.19E-01	1.00E+00	-0.97	1.93E-01	7.46E-01
**C22orf24**	-2.57	2.37E-01	1.00E+00	1.62	1.93E-01	7.46E-01
**SMILR**	-0.69	6.76E-01	1.00E+00	1.59	1.93E-01	7.46E-01
**LMBR1**	0.05	7.56E-01	1.00E+00	-0.43	1.93E-01	7.46E-01
**TTC25**	-0.20	7.64E-01	1.00E+00	-0.72	1.93E-01	7.46E-01
**NLGN3**	-0.03	9.51E-01	1.00E+00	0.87	1.93E-01	7.46E-01
**CYLD**	0.00	1.00E+00	1.00E+00	0.34	1.93E-01	7.46E-01
**AC004975.2**	-2.79	1.72E-02	8.25E-01	1.73	1.94E-01	7.46E-01
**AL157395.1**	-2.30	1.54E-01	1.00E+00	-1.60	1.94E-01	7.46E-01
**RTN4RL1**	0.28	2.49E-01	1.00E+00	-0.99	1.94E-01	7.46E-01
**AC007671.1**	1.10	4.97E-01	1.00E+00	1.61	1.94E-01	7.46E-01
**KLRG1**	1.19	1.95E-01	1.00E+00	-0.84	1.94E-01	7.46E-01
**CFLAR**	-0.01	9.57E-01	1.00E+00	0.36	1.94E-01	7.46E-01
**COLEC11**	-0.34	6.83E-01	1.00E+00	1.15	1.94E-01	7.46E-01
**FABP5P7**	-0.03	9.35E-01	1.00E+00	0.53	1.94E-01	7.46E-01
**AC007342.3**	0.13	9.70E-01	1.00E+00	1.40	1.94E-01	7.46E-01
**EIF3LP2**	-0.50	8.26E-01	1.00E+00	-0.91	1.94E-01	7.46E-01
**KPNA6**	0.04	7.79E-01	1.00E+00	0.29	1.94E-01	7.46E-01
**BZW1P2**	-0.05	9.18E-01	1.00E+00	-0.41	1.94E-01	7.46E-01
**CD248**	0.40	4.22E-01	1.00E+00	0.78	1.94E-01	7.46E-01
**SSUH2**	-0.66	5.00E-01	1.00E+00	0.98	1.94E-01	7.46E-01
**CSTB**	-0.01	9.74E-01	1.00E+00	0.33	1.94E-01	7.46E-01

**SH3BP5-AS1**	0.97	2.70E-01	1.00E+00	0.78	1.94E-01	7.47E-01
**AL162231.2**	0.36	3.33E-01	1.00E+00	0.56	1.94E-01	7.47E-01
**ZNF408**	-0.14	6.01E-01	1.00E+00	0.34	1.94E-01	7.47E-01
**GRIK4**	-0.33	5.71E-01	1.00E+00	-1.17	1.94E-01	7.47E-01
**SEZ6**	-2.60	3.36E-02	1.00E+00	-1.39	1.94E-01	7.47E-01
**PPP1R9A**	0.72	3.75E-01	1.00E+00	-0.72	1.94E-01	7.47E-01
**AC010168.2**	0.10	9.05E-01	1.00E+00	0.86	1.94E-01	7.47E-01
**POLR2K**	0.22	3.24E-01	1.00E+00	-0.52	1.94E-01	7.47E-01
**SLIRP**	-0.04	8.55E-01	1.00E+00	-0.67	1.94E-01	7.47E-01
**LINC02473**	-2.44	3.04E-01	1.00E+00	-1.89	1.94E-01	7.47E-01
**DLGAP4**	-0.22	2.98E-01	1.00E+00	0.26	1.94E-01	7.47E-01
**NIT2**	-0.02	9.25E-01	1.00E+00	-0.33	1.94E-01	7.47E-01
**C6orf222**	0.67	5.37E-01	1.00E+00	1.60	1.94E-01	7.47E-01
**SDHDP6**	1.57	1.57E-01	1.00E+00	-0.67	1.94E-01	7.47E-01
**LINC01254**	-0.13	7.60E-01	1.00E+00	-1.04	1.94E-01	7.47E-01
**DAZAP1**	-0.33	2.92E-01	1.00E+00	0.68	1.95E-01	7.47E-01
**AL022476.1**	0.17	8.90E-01	1.00E+00	-0.99	1.95E-01	7.47E-01
**C1QTNF1**	0.58	1.74E-01	1.00E+00	0.90	1.95E-01	7.47E-01
**KREMEN2**	-0.81	1.57E-01	1.00E+00	-1.25	1.95E-01	7.47E-01
**G41779**	-0.02	9.81E-01	1.00E+00	0.93	1.95E-01	7.47E-01
**AC137932.2**	-1.83	3.51E-01	1.00E+00	-1.45	1.95E-01	7.47E-01
**RABEP2**	-0.13	6.18E-01	1.00E+00	0.52	1.95E-01	7.47E-01
**AL133371.2**	0.53	6.01E-01	1.00E+00	1.86	1.95E-01	7.47E-01
**AL161772.1**	0.85	3.59E-01	1.00E+00	-1.38	1.95E-01	7.47E-01
**RAD54B**	-1.22	1.91E-01	1.00E+00	0.64	1.95E-01	7.47E-01
**AC006946.2**	-0.28	8.26E-01	1.00E+00	-1.20	1.95E-01	7.47E-01
**AL160408.1**	-2.00	2.91E-01	1.00E+00	1.10	1.95E-01	7.47E-01
**NR2C1**	0.07	8.35E-01	1.00E+00	-0.31	1.95E-01	7.47E-01
**BZW2**	-0.06	7.87E-01	1.00E+00	-0.26	1.95E-01	7.47E-01
**S100A2**	-0.69	9.30E-02	1.00E+00	0.71	1.95E-01	7.47E-01
**PLXNB2**	-0.26	2.56E-01	1.00E+00	0.73	1.95E-01	7.47E-01
**ASH1L-AS1**	-0.26	4.98E-01	1.00E+00	0.55	1.95E-01	7.47E-01

**BEND3**	-0.10	8.09E-01	1.00E+00	0.77	1.95E-01	7.47E-01
**TAP1**	-0.11	8.05E-01	1.00E+00	0.62	1.95E-01	7.47E-01
**TNIP1**	0.30	3.04E-01	1.00E+00	0.62	1.95E-01	7.47E-01
**HIST1H2BD**	-0.65	2.77E-01	1.00E+00	0.55	1.95E-01	7.47E-01
**CREM**	0.15	6.14E-01	1.00E+00	0.30	1.95E-01	7.48E-01
**BPHL**	0.11	6.12E-01	1.00E+00	-0.37	1.95E-01	7.48E-01
**CARD6**	0.46	3.68E-01	1.00E+00	0.87	1.96E-01	7.48E-01
**HIST1H4J**	1.35	2.23E-01	1.00E+00	0.45	1.96E-01	7.48E-01
**AC046158.3**	-1.28	7.10E-01	1.00E+00	2.06	1.96E-01	7.48E-01
**AC099568.2**	-0.41	6.45E-01	1.00E+00	-1.09	1.96E-01	7.48E-01
**ESRRAP2**	1.57	5.00E-01	1.00E+00	1.42	1.96E-01	7.48E-01
**NUDCD1**	-0.05	8.74E-01	1.00E+00	-0.32	1.96E-01	7.48E-01
**ENKD1**	-0.20	4.52E-01	1.00E+00	0.63	1.96E-01	7.48E-01
**POFUT2**	0.23	5.68E-01	1.00E+00	0.79	1.96E-01	7.48E-01
**JARID2-AS1**	-0.33	8.23E-01	1.00E+00	1.26	1.96E-01	7.48E-01
**SPATA6L**	0.80	1.48E-01	1.00E+00	0.55	1.96E-01	7.49E-01
**IL17C**	-1.72	6.11E-01	1.00E+00	1.92	1.96E-01	7.49E-01
**RRAGD**	0.14	7.02E-01	1.00E+00	-0.44	1.96E-01	7.49E-01
**FAM204A**	0.00	9.89E-01	1.00E+00	-0.38	1.96E-01	7.49E-01
**ZEB2**	0.09	8.38E-01	1.00E+00	0.52	1.96E-01	7.49E-01
**DCST1**	-0.25	7.98E-01	1.00E+00	0.82	1.96E-01	7.49E-01
**GTF3C2**	0.08	7.20E-01	1.00E+00	0.27	1.96E-01	7.49E-01
**ATXN2L**	-0.27	2.23E-01	1.00E+00	0.73	1.96E-01	7.49E-01
**GSN-AS1**	1.42	1.67E-01	1.00E+00	0.94	1.96E-01	7.49E-01
**NUCKS1**	0.21	1.80E-01	1.00E+00	0.41	1.96E-01	7.49E-01
**MT1X**	-0.02	9.64E-01	1.00E+00	-0.93	1.96E-01	7.49E-01
**MAP3K15**	1.33	3.27E-01	1.00E+00	0.92	1.96E-01	7.49E-01
**NOS1**	-0.45	3.21E-01	1.00E+00	-0.85	1.96E-01	7.49E-01
**C2orf49**	0.07	7.73E-01	1.00E+00	-0.36	1.96E-01	7.49E-01
**TBC1D8-AS1**	-0.93	2.18E-01	1.00E+00	1.14	1.97E-01	7.49E-01
**ZNF660**	0.16	8.48E-01	1.00E+00	1.02	1.97E-01	7.49E-01
**LYRM2**	0.06	7.23E-01	1.00E+00	-0.30	1.97E-01	7.49E-01

**SPPL2B**	-0.04	8.60E-01	1.00E+00	0.43	1.97E-01	7.49E-01
**TOX4**	-0.16	5.08E-01	1.00E+00	0.25	1.97E-01	7.49E-01
**GPCPD1**	-0.18	5.37E-01	1.00E+00	0.28	1.97E-01	7.49E-01
**LZTS1**	-0.49	3.96E-02	1.00E+00	0.88	1.97E-01	7.49E-01
**TSPAN3**	0.16	5.42E-01	1.00E+00	-0.42	1.97E-01	7.49E-01
**CEP135**	-0.48	5.00E-02	1.00E+00	0.30	1.97E-01	7.49E-01
**DYM**	0.14	5.01E-01	1.00E+00	-0.23	1.97E-01	7.49E-01
**TAF9**	-0.06	8.19E-01	1.00E+00	-0.50	1.97E-01	7.49E-01
**PCYT1B**	-0.36	7.15E-01	1.00E+00	1.41	1.97E-01	7.49E-01
**AC119674.1**	-1.09	3.98E-01	1.00E+00	-1.28	1.97E-01	7.49E-01
**CLCN5**	0.22	4.37E-01	1.00E+00	0.56	1.97E-01	7.49E-01
**NEFH**	-2.18	1.92E-05	5.70E-03	1.36	1.97E-01	7.49E-01
**AC010247.2**	1.55	1.76E-01	1.00E+00	1.67	1.97E-01	7.49E-01
**ITLN2**	-1.17	2.68E-01	1.00E+00	-1.30	1.97E-01	7.49E-01
**NIFK-AS1**	0.16	6.82E-01	1.00E+00	-0.41	1.97E-01	7.49E-01
**MTIF3**	0.03	8.72E-01	1.00E+00	-0.39	1.97E-01	7.49E-01
**AC073896.4**	-0.59	5.66E-01	1.00E+00	-0.71	1.97E-01	7.49E-01
**SPRR5**	0.59	6.26E-01	1.00E+00	1.00	1.97E-01	7.49E-01
**AC117490.2**	-1.85	4.36E-01	1.00E+00	-1.50	1.97E-01	7.49E-01
**CD96**	-0.69	2.84E-01	1.00E+00	1.05	1.97E-01	7.49E-01
**AL445673.1**	-0.34	7.81E-01	1.00E+00	-1.46	1.97E-01	7.49E-01
**SCNN1G**	-0.63	2.10E-01	1.00E+00	-0.90	1.97E-01	7.49E-01
**CNTNAP3**	0.58	1.33E-01	1.00E+00	-0.51	1.97E-01	7.49E-01
**ELMO2**	0.06	7.97E-01	1.00E+00	0.30	1.97E-01	7.49E-01
**WASF1**	-0.23	5.69E-01	1.00E+00	0.45	1.97E-01	7.49E-01
**STAM**	0.12	6.44E-01	1.00E+00	0.22	1.97E-01	7.49E-01
**EPOR**	0.33	3.62E-01	1.00E+00	0.52	1.97E-01	7.49E-01
**AC003681.1**	0.84	2.46E-01	1.00E+00	0.55	1.98E-01	7.49E-01
**NUPR2**	1.14	8.91E-02	1.00E+00	-0.78	1.98E-01	7.49E-01
**XYLT1**	0.33	2.46E-01	1.00E+00	0.53	1.98E-01	7.49E-01
**CAMK2G**	-0.14	5.81E-01	1.00E+00	0.49	1.98E-01	7.49E-01
**ANKRD33B**	-0.27	4.17E-01	1.00E+00	-0.55	1.98E-01	7.49E-01

**SH3BP5L**	0.18	4.87E-01	1.00E+00	0.75	1.98E-01	7.49E-01
**IGHV1-46**	2.03	4.35E-01	1.00E+00	2.50	1.98E-01	7.49E-01
**NOD2**	-0.24	6.68E-01	1.00E+00	0.68	1.98E-01	7.49E-01
**SPOCD1**	-1.38	2.01E-01	1.00E+00	1.31	1.98E-01	7.49E-01
**PDIA5**	0.08	8.50E-01	1.00E+00	0.62	1.98E-01	7.49E-01
**SCRN3**	0.10	6.92E-01	1.00E+00	-0.23	1.98E-01	7.49E-01
**AC131235.4**	-2.25	5.03E-01	1.00E+00	-1.98	1.98E-01	7.49E-01
**CXorf40B**	0.31	2.46E-01	1.00E+00	0.31	1.98E-01	7.49E-01
**CDSN**	-0.67	5.03E-01	1.00E+00	1.21	1.98E-01	7.49E-01
**MACO1**	0.07	6.96E-01	1.00E+00	0.55	1.98E-01	7.49E-01
**LEF1**	-0.54	1.04E-01	1.00E+00	0.59	1.98E-01	7.49E-01
**RNF222**	-0.19	8.06E-01	1.00E+00	1.05	1.98E-01	7.49E-01
**DOCK5**	-0.14	6.85E-01	1.00E+00	0.42	1.98E-01	7.49E-01
**AC068580.1**	-3.44	3.39E-02	1.00E+00	1.94	1.98E-01	7.49E-01
**FTH1P2**	-0.01	9.88E-01	1.00E+00	-0.77	1.98E-01	7.49E-01
**TOM1**	-0.11	6.96E-01	1.00E+00	0.28	1.98E-01	7.49E-01
**KCNJ14**	-0.94	4.86E-02	1.00E+00	0.79	1.98E-01	7.50E-01
**SRSF9**	-0.06	7.66E-01	1.00E+00	-0.66	1.98E-01	7.50E-01
**GSDME**	-0.06	9.21E-01	1.00E+00	0.74	1.98E-01	7.50E-01
**AC090515.2**	-0.98	2.89E-01	1.00E+00	-0.80	1.99E-01	7.50E-01
**SNORA33**	-1.67	6.26E-01	1.00E+00	-1.60	1.99E-01	7.50E-01
**LINC01597**	1.79	5.58E-01	1.00E+00	1.37	1.99E-01	7.50E-01
**TRGV9**	-0.75	6.70E-01	1.00E+00	1.81	1.99E-01	7.50E-01
**SMARCD1**	-0.17	2.26E-01	1.00E+00	0.59	1.99E-01	7.50E-01
**MYLK-AS1**	0.89	4.97E-01	1.00E+00	-0.91	1.99E-01	7.50E-01
**FBXO44**	0.11	6.59E-01	1.00E+00	0.27	1.99E-01	7.50E-01
**SYAP1**	-0.14	2.86E-01	1.00E+00	-0.26	1.99E-01	7.50E-01
**LINC02367**	-2.83	1.79E-01	1.00E+00	-0.75	1.99E-01	7.50E-01
**SUMO1**	0.05	7.12E-01	1.00E+00	-0.41	1.99E-01	7.50E-01
**ZNF468**	0.12	6.64E-01	1.00E+00	-0.39	1.99E-01	7.50E-01
**AL450998.1**	1.14	5.65E-01	1.00E+00	1.10	1.99E-01	7.50E-01
**AC100803.1**	-1.95	5.51E-01	1.00E+00	2.84	1.99E-01	7.50E-01

**ARID1A**	-0.42	7.28E-02	1.00E+00	0.51	1.99E-01	7.50E-01
**FAHD2CP**	-0.42	4.48E-01	1.00E+00	-0.43	1.99E-01	7.50E-01
**PPCS**	0.16	3.89E-01	1.00E+00	-0.51	1.99E-01	7.50E-01
**DCAF11**	0.06	6.96E-01	1.00E+00	-0.17	1.99E-01	7.50E-01
**OLFML2A**	-0.07	8.16E-01	1.00E+00	0.45	1.99E-01	7.51E-01
**LY6K**	-0.16	7.29E-01	1.00E+00	-0.52	1.99E-01	7.51E-01
**TRMT13**	0.10	7.76E-01	1.00E+00	-0.34	1.99E-01	7.51E-01
**CCNL1**	-0.17	5.92E-01	1.00E+00	0.36	1.99E-01	7.51E-01
**GPN1**	-0.01	9.68E-01	1.00E+00	-0.42	1.99E-01	7.51E-01
**MSI1**	-0.94	1.80E-01	1.00E+00	1.12	1.99E-01	7.51E-01
**S100B**	0.74	2.98E-01	1.00E+00	-0.66	1.99E-01	7.51E-01
**XLOC_001376**	-1.12	4.47E-01	1.00E+00	1.59	1.99E-01	7.51E-01
**AC009078.2**	0.21	8.84E-01	1.00E+00	2.48	1.99E-01	7.51E-01
**GNMT**	-0.46	7.59E-01	1.00E+00	-1.24	1.99E-01	7.51E-01
**SETD3**	0.09	5.23E-01	1.00E+00	-0.27	1.99E-01	7.51E-01
**MTX3**	0.34	1.74E-01	1.00E+00	-0.29	2.00E-01	7.51E-01
**LAMB3**	-0.22	4.18E-01	1.00E+00	0.50	2.00E-01	7.51E-01
**AC138305.1**	-0.11	8.74E-01	1.00E+00	-0.79	2.00E-01	7.51E-01
**TENM4**	-0.23	6.11E-01	1.00E+00	0.61	2.00E-01	7.51E-01
**BRINP3**	1.47	3.10E-01	1.00E+00	1.39	2.00E-01	7.51E-01
**P2RY4**	0.18	8.77E-01	1.00E+00	-1.27	2.00E-01	7.51E-01
**MKRN1**	0.09	6.26E-01	1.00E+00	-0.50	2.00E-01	7.51E-01
**SYNJ2**	-0.13	6.52E-01	1.00E+00	0.70	2.00E-01	7.51E-01
**WASL**	-0.08	6.33E-01	1.00E+00	-0.27	2.00E-01	7.51E-01
**KIAA0895LP1**	-1.50	1.72E-01	1.00E+00	1.45	2.00E-01	7.51E-01
**HELQ**	-0.02	9.05E-01	1.00E+00	-0.30	2.00E-01	7.51E-01
**RBBP4**	-0.03	8.76E-01	1.00E+00	-0.21	2.00E-01	7.51E-01
**PCDHA7**	-2.84	9.77E-03	6.13E-01	1.96	2.00E-01	7.51E-01
**PCDHAC1**	0.39	7.34E-01	1.00E+00	1.48	2.00E-01	7.51E-01
**ANGPTL2**	0.24	6.33E-01	1.00E+00	0.72	2.00E-01	7.51E-01
**TMEM189**	0.00	9.92E-01	1.00E+00	-0.25	2.00E-01	7.51E-01
**AC007637.1**	1.49	8.74E-02	1.00E+00	-1.18	2.00E-01	7.51E-01

**AC019193.2**	1.19	3.69E-01	1.00E+00	-1.45	2.00E-01	7.52E-01
**GSTZ1**	-0.08	7.44E-01	1.00E+00	0.20	2.00E-01	7.52E-01
**SHPRH**	-0.07	8.01E-01	1.00E+00	-0.36	2.00E-01	7.52E-01
**GNB1L**	-0.02	9.59E-01	1.00E+00	0.31	2.00E-01	7.52E-01
**ATP6V1C1**	0.35	1.58E-01	1.00E+00	-0.28	2.01E-01	7.52E-01
**CEP57L1**	0.08	8.17E-01	1.00E+00	-0.39	2.01E-01	7.52E-01
**RAB12**	0.08	6.59E-01	1.00E+00	0.82	2.01E-01	7.52E-01
**DDX17**	-0.12	7.00E-01	1.00E+00	0.42	2.01E-01	7.52E-01
**LRRN2**	-0.15	8.01E-01	1.00E+00	1.62	2.01E-01	7.52E-01
**PPP5D1**	-0.40	4.58E-01	1.00E+00	-0.77	2.01E-01	7.52E-01
**FAM186B**	-2.28	3.14E-02	1.00E+00	1.07	2.01E-01	7.52E-01
**SYTL2**	-0.10	6.67E-01	1.00E+00	0.41	2.01E-01	7.52E-01
**MNT**	-0.77	9.36E-03	6.05E-01	0.73	2.01E-01	7.52E-01
**DDX28**	-0.18	4.52E-01	1.00E+00	0.29	2.01E-01	7.52E-01
**FILIP1L**	-0.24	5.25E-01	1.00E+00	0.35	2.01E-01	7.52E-01
**TSC22D4**	-0.29	2.55E-01	1.00E+00	0.59	2.01E-01	7.52E-01
**DLX2**	-1.13	2.28E-01	1.00E+00	1.20	2.01E-01	7.52E-01
**CAMLG**	0.09	6.29E-01	1.00E+00	-0.36	2.01E-01	7.52E-01
**RPS6KA3**	0.00	9.94E-01	1.00E+00	-0.30	2.01E-01	7.52E-01
**AC090948.3**	-2.79	4.10E-01	1.00E+00	1.99	2.01E-01	7.52E-01
**TESPA1**	-0.20	7.23E-01	1.00E+00	0.90	2.01E-01	7.52E-01
**AC135721.1**	-1.57	9.55E-02	1.00E+00	-1.49	2.01E-01	7.52E-01
**AP003469.2**	-1.99	1.91E-01	1.00E+00	-1.48	2.01E-01	7.52E-01
**MTHFD1L**	-0.05	8.74E-01	1.00E+00	0.74	2.01E-01	7.52E-01
**COX5BP6**	0.45	7.47E-01	1.00E+00	1.23	2.01E-01	7.53E-01
**PTPRG**	0.42	2.19E-01	1.00E+00	0.57	2.01E-01	7.53E-01
**DLAT**	0.00	9.91E-01	1.00E+00	-0.34	2.01E-01	7.53E-01
**AC005244.1**	-0.39	9.10E-01	1.00E+00	0.88	2.01E-01	7.53E-01
**LINC02323**	1.28	2.62E-01	1.00E+00	1.75	2.01E-01	7.53E-01
**IPCEF1**	-0.78	8.05E-02	1.00E+00	0.78	2.02E-01	7.53E-01
**HLA-K**	-0.02	9.71E-01	1.00E+00	0.73	2.02E-01	7.53E-01
**ZNF646**	-0.39	9.37E-02	1.00E+00	0.69	2.02E-01	7.53E-01

**ZNF254**	-0.18	5.55E-01	1.00E+00	-0.49	2.02E-01	7.53E-01
**CD40LG**	0.21	7.98E-01	1.00E+00	1.29	2.02E-01	7.53E-01
**DLX1**	-0.53	5.44E-01	1.00E+00	1.16	2.02E-01	7.53E-01
**AC007182.2**	0.37	9.14E-01	1.00E+00	0.91	2.02E-01	7.53E-01
**CMPK2**	-0.02	9.87E-01	1.00E+00	1.06	2.02E-01	7.53E-01
**PRKAR1A**	0.23	3.42E-01	1.00E+00	-0.32	2.02E-01	7.53E-01
**FAM21EP**	-0.39	5.98E-01	1.00E+00	0.72	2.02E-01	7.53E-01
**HPS5**	0.47	1.36E-01	1.00E+00	0.33	2.02E-01	7.53E-01
**C14orf180**	1.19	7.21E-01	1.00E+00	-1.36	2.02E-01	7.53E-01
**KIF19**	-0.27	6.51E-01	1.00E+00	1.23	2.02E-01	7.54E-01
**TGFBR3**	0.54	8.92E-02	1.00E+00	-0.42	2.02E-01	7.54E-01
**SETX**	-0.04	8.10E-01	1.00E+00	0.36	2.02E-01	7.54E-01
**MFSD5**	-0.04	9.02E-01	1.00E+00	-0.38	2.02E-01	7.54E-01
**AL391280.1**	0.66	5.67E-01	1.00E+00	2.01	2.02E-01	7.55E-01
**APOBEC3D**	-0.87	6.03E-01	1.00E+00	1.00	2.02E-01	7.55E-01
**G22374**	-2.23	1.12E-01	1.00E+00	0.98	2.03E-01	7.55E-01
**HIST1H2AL**	-2.81	3.99E-01	1.00E+00	-1.48	2.03E-01	7.55E-01
**SELP**	-0.49	3.87E-01	1.00E+00	0.75	2.03E-01	7.55E-01
**GRIA3**	0.00	9.96E-01	1.00E+00	0.92	2.03E-01	7.55E-01
**SBDS**	0.25	5.65E-02	1.00E+00	-0.40	2.03E-01	7.55E-01
**PIGV**	0.26	2.69E-01	1.00E+00	-0.26	2.03E-01	7.55E-01
**C5orf34**	0.11	7.94E-01	1.00E+00	-0.69	2.03E-01	7.55E-01
**IGKV3-15**	6.18	3.54E-06	1.17E-03	3.99	2.03E-01	7.55E-01
**MYCL**	0.31	4.38E-01	1.00E+00	-0.76	2.03E-01	7.56E-01
**ADAM17**	-0.06	7.79E-01	1.00E+00	0.49	2.03E-01	7.56E-01
**CBFA2T2**	-0.26	3.22E-01	1.00E+00	-0.47	2.03E-01	7.56E-01
**AC024610.2**	1.10	4.12E-01	1.00E+00	1.24	2.03E-01	7.56E-01
**PTOV1**	-0.21	4.72E-01	1.00E+00	-0.50	2.03E-01	7.56E-01
**LRRC6**	-0.51	2.87E-01	1.00E+00	-0.57	2.03E-01	7.56E-01
**SIRPB1**	-1.01	2.93E-01	1.00E+00	1.18	2.03E-01	7.56E-01
**ALG9**	0.64	3.93E-02	1.00E+00	0.44	2.03E-01	7.56E-01
**AL731563.3**	0.46	7.59E-01	1.00E+00	-1.29	2.03E-01	7.56E-01

**PTBP2**	0.30	5.17E-01	1.00E+00	-0.51	2.03E-01	7.56E-01
**AC021106.3**	0.25	8.32E-01	1.00E+00	1.65	2.03E-01	7.56E-01
**RCOR2**	0.17	8.07E-01	1.00E+00	1.25	2.03E-01	7.56E-01
**LINC00445**	3.73	1.49E-01	1.00E+00	-1.58	2.04E-01	7.56E-01
**TRRAP**	-0.10	7.32E-01	1.00E+00	0.67	2.04E-01	7.56E-01
**GRIN3A**	0.51	3.96E-01	1.00E+00	1.05	2.04E-01	7.56E-01
**DUSP1**	-1.23	1.23E-01	1.00E+00	-0.61	2.04E-01	7.56E-01
**GTF2H2**	0.03	9.54E-01	1.00E+00	-0.50	2.04E-01	7.56E-01
**C11orf71**	-0.09	6.93E-01	1.00E+00	-0.57	2.04E-01	7.56E-01
**XLOC_004673**	0.34	5.17E-01	1.00E+00	1.41	2.04E-01	7.56E-01
**PUS10**	-0.27	3.43E-01	1.00E+00	-0.34	2.04E-01	7.56E-01
**TAPT1-AS1**	0.26	6.00E-01	1.00E+00	-1.02	2.04E-01	7.56E-01
**AL031985.3**	-1.10	4.69E-02	1.00E+00	0.86	2.04E-01	7.56E-01
**SNORD112**	-0.39	8.96E-01	1.00E+00	1.30	2.04E-01	7.56E-01
**STX4**	-0.02	9.31E-01	1.00E+00	0.42	2.04E-01	7.56E-01
**BRF2**	0.52	6.62E-02	1.00E+00	0.28	2.04E-01	7.56E-01
**FAM122B**	-0.25	2.10E-01	1.00E+00	0.33	2.04E-01	7.56E-01
**MAPK7**	-0.31	2.36E-01	1.00E+00	0.55	2.04E-01	7.56E-01
**TRAV14DV4**	-1.75	3.38E-01	1.00E+00	2.02	2.04E-01	7.56E-01
**DCAKD**	-0.08	7.61E-01	1.00E+00	-0.41	2.04E-01	7.56E-01
**TMOD1**	-0.23	5.85E-01	1.00E+00	-0.52	2.04E-01	7.56E-01
**IGHV5-51**	1.29	3.73E-01	1.00E+00	4.01	2.04E-01	7.56E-01
**ZNF529**	0.25	4.48E-01	1.00E+00	-0.37	2.04E-01	7.56E-01
**RABGAP1L**	-0.01	9.45E-01	1.00E+00	-0.25	2.04E-01	7.56E-01
**FLT3LG**	3.28	1.10E-01	1.00E+00	1.15	2.04E-01	7.56E-01
**MRPL39**	0.12	5.71E-01	1.00E+00	-0.42	2.04E-01	7.56E-01
**CNTN1**	-0.69	1.65E-01	1.00E+00	-0.66	2.04E-01	7.56E-01
**LIMS1**	0.18	4.95E-01	1.00E+00	0.36	2.04E-01	7.56E-01
**AC046143.1**	0.54	7.97E-01	1.00E+00	1.06	2.04E-01	7.56E-01
**G4061**	-1.05	3.08E-01	1.00E+00	1.61	2.04E-01	7.56E-01
**CNOT8**	0.37	7.60E-02	1.00E+00	-0.28	2.04E-01	7.56E-01
**RPL6**	-0.16	6.29E-01	1.00E+00	0.39	2.04E-01	7.56E-01

**MRPS14**	0.01	9.79E-01	1.00E+00	-0.40	2.04E-01	7.56E-01
**POU2AF1**	0.63	4.33E-01	1.00E+00	1.61	2.04E-01	7.56E-01
**ZFP28**	0.28	2.99E-01	1.00E+00	-0.56	2.04E-01	7.56E-01
**RPL11**	-0.05	8.72E-01	1.00E+00	0.39	2.05E-01	7.56E-01
**PML**	-0.36	3.42E-01	1.00E+00	0.66	2.05E-01	7.56E-01
**SHISA2**	-0.94	9.03E-02	1.00E+00	0.66	2.05E-01	7.56E-01
**ATP5MD**	0.24	1.87E-01	1.00E+00	-0.47	2.05E-01	7.56E-01
**AC112484.1**	0.57	6.20E-01	1.00E+00	1.04	2.05E-01	7.56E-01
**AC017071.1**	-0.47	7.10E-01	1.00E+00	1.12	2.05E-01	7.56E-01
**FAM124A**	-0.21	5.33E-01	1.00E+00	0.83	2.05E-01	7.56E-01
**LASP1**	0.25	3.73E-01	1.00E+00	0.64	2.05E-01	7.56E-01
**NIPSNAP3A**	0.12	6.49E-01	1.00E+00	-0.42	2.05E-01	7.56E-01
**AMPD3**	-0.03	9.44E-01	1.00E+00	0.45	2.05E-01	7.56E-01
**AC064875.1**	-1.19	7.28E-01	1.00E+00	1.64	2.05E-01	7.57E-01
**FA2H**	-0.13	7.68E-01	1.00E+00	-1.09	2.05E-01	7.57E-01
**ARL5A**	0.08	8.53E-01	1.00E+00	-0.51	2.05E-01	7.57E-01
**KRT8P39**	-1.38	5.61E-01	1.00E+00	-1.85	2.05E-01	7.57E-01
**ADAMTS7P4**	-0.24	8.41E-01	1.00E+00	1.27	2.05E-01	7.57E-01
**PAPSS2**	0.26	5.92E-01	1.00E+00	0.68	2.05E-01	7.57E-01
**S100A1**	0.41	7.73E-01	1.00E+00	-0.88	2.05E-01	7.57E-01
**CDC16**	0.03	8.57E-01	1.00E+00	-0.37	2.05E-01	7.57E-01
**WFIKKN1**	-1.19	7.89E-03	5.46E-01	-1.32	2.05E-01	7.57E-01
**TPP1**	0.27	3.57E-01	1.00E+00	0.28	2.05E-01	7.57E-01
**PPIA**	0.05	8.01E-01	1.00E+00	-0.31	2.05E-01	7.57E-01
**EEF1AKMT2**	0.13	7.15E-01	1.00E+00	-0.65	2.05E-01	7.57E-01
**HLA-DPA3**	-0.80	8.18E-01	1.00E+00	3.00	2.05E-01	7.57E-01
**AC093827.5**	-0.70	6.98E-01	1.00E+00	-0.86	2.05E-01	7.57E-01
**ITPR1**	0.36	5.47E-01	1.00E+00	0.60	2.05E-01	7.57E-01
**HERC1**	0.18	5.32E-01	1.00E+00	0.31	2.05E-01	7.57E-01
**ZNF541**	0.24	7.79E-01	1.00E+00	-0.93	2.06E-01	7.58E-01
**AC037198.1**	0.00	9.99E-01	1.00E+00	1.38	2.06E-01	7.58E-01
**MBNL1-AS1**	-0.22	6.53E-01	1.00E+00	0.53	2.06E-01	7.58E-01

**PIGP**	0.21	5.18E-01	1.00E+00	-0.51	2.06E-01	7.58E-01
**LINC01481**	-0.62	3.91E-01	1.00E+00	-0.80	2.06E-01	7.58E-01
**AC068205.2**	-0.21	9.35E-01	1.00E+00	-2.56	2.06E-01	7.58E-01
**ORC1**	-0.58	8.79E-02	1.00E+00	0.64	2.06E-01	7.58E-01
**RRN3**	0.22	2.29E-01	1.00E+00	-0.28	2.06E-01	7.58E-01
**RFXAP**	0.04	9.07E-01	1.00E+00	-0.77	2.06E-01	7.58E-01
**ADGRE5**	0.17	6.12E-01	1.00E+00	0.73	2.06E-01	7.58E-01
**ERG**	0.29	5.38E-01	1.00E+00	0.59	2.06E-01	7.58E-01
**TEX26**	0.56	8.71E-01	1.00E+00	-1.83	2.06E-01	7.58E-01
**PGLS**	-0.08	7.34E-01	1.00E+00	0.21	2.06E-01	7.58E-01
**SLC4A9**	-0.80	8.18E-01	1.00E+00	-1.96	2.06E-01	7.58E-01
**AC021752.1**	0.44	6.56E-01	1.00E+00	-0.95	2.06E-01	7.58E-01
**FBXL5**	0.17	5.11E-01	1.00E+00	-0.41	2.06E-01	7.58E-01
**SYNE4**	0.45	4.00E-01	1.00E+00	-0.86	2.06E-01	7.58E-01
**EMC3**	0.03	8.53E-01	1.00E+00	-0.29	2.06E-01	7.58E-01
**HSPA8P1**	NA	NA	NA	1.13	2.06E-01	7.58E-01
**COX7B**	0.02	9.18E-01	1.00E+00	-0.52	2.06E-01	7.58E-01
**ARHGAP15**	0.43	3.25E-01	1.00E+00	0.41	2.06E-01	7.58E-01
**PLEC**	-0.47	1.66E-01	1.00E+00	0.88	2.06E-01	7.58E-01
**AC012442.2**	0.93	3.69E-01	1.00E+00	-0.93	2.06E-01	7.58E-01
**AC026471.4**	-0.29	7.24E-01	1.00E+00	-0.54	2.06E-01	7.58E-01
**AL353593.2**	1.42	2.89E-01	1.00E+00	-1.44	2.07E-01	7.58E-01
**GFPT2**	0.41	4.01E-01	1.00E+00	0.77	2.07E-01	7.58E-01
**EPRS**	-0.29	1.13E-01	1.00E+00	0.18	2.07E-01	7.58E-01
**AC010326.3**	-1.24	8.68E-02	1.00E+00	0.83	2.07E-01	7.58E-01
**CTU2**	-0.20	5.54E-01	1.00E+00	0.36	2.07E-01	7.58E-01
**ZNF491**	0.21	7.56E-01	1.00E+00	-0.97	2.07E-01	7.58E-01
**FBLN1**	0.78	1.61E-01	1.00E+00	0.65	2.07E-01	7.58E-01
**EXOC6**	0.00	9.90E-01	1.00E+00	-0.30	2.07E-01	7.58E-01
**C19orf73**	-0.34	5.06E-01	1.00E+00	-0.55	2.07E-01	7.58E-01
**GVQW3**	0.13	8.57E-01	1.00E+00	-0.91	2.07E-01	7.58E-01
**SH2B1**	-0.20	3.81E-01	1.00E+00	0.72	2.07E-01	7.58E-01

**GNB4**	0.18	5.67E-01	1.00E+00	-0.38	2.07E-01	7.58E-01
**SUOX**	0.26	5.61E-01	1.00E+00	-0.48	2.07E-01	7.58E-01
**GNB5**	0.09	7.31E-01	1.00E+00	0.22	2.07E-01	7.58E-01
**TNMD**	1.10	2.36E-01	1.00E+00	-1.03	2.07E-01	7.58E-01
**MT-TL1**	1.28	7.08E-01	1.00E+00	-1.04	2.07E-01	7.58E-01
**ZNF354C**	0.30	4.39E-01	1.00E+00	0.64	2.07E-01	7.58E-01
**PARD3**	0.06	8.61E-01	1.00E+00	-0.46	2.07E-01	7.58E-01
**AC104653.1**	-2.55	1.26E-01	1.00E+00	-1.07	2.07E-01	7.59E-01
**AC124804.1**	-3.06	8.46E-03	5.67E-01	-2.09	2.07E-01	7.59E-01
**TTC39A**	-0.34	5.43E-01	1.00E+00	0.62	2.07E-01	7.59E-01
**GNPAT**	0.30	1.03E-01	1.00E+00	-0.34	2.07E-01	7.59E-01
**TRAM2-AS1**	0.04	9.02E-01	1.00E+00	-0.68	2.07E-01	7.59E-01
**SBF1**	0.09	7.45E-01	1.00E+00	0.73	2.08E-01	7.59E-01
**PTH2R**	0.74	5.89E-01	1.00E+00	-1.32	2.08E-01	7.59E-01
**KCNMB4**	-0.50	1.29E-01	1.00E+00	-0.62	2.08E-01	7.59E-01
**CCDC146**	0.57	1.64E-01	1.00E+00	0.49	2.08E-01	7.59E-01
**STRIP1**	-0.01	9.38E-01	1.00E+00	0.36	2.08E-01	7.59E-01
**KPNB1**	0.10	5.48E-01	1.00E+00	0.39	2.08E-01	7.59E-01
**FAM45A**	0.00	9.99E-01	1.00E+00	-0.22	2.08E-01	7.59E-01
**AC025178.1**	0.13	9.62E-01	1.00E+00	1.34	2.08E-01	7.59E-01
**XLOC_006348**	-2.16	5.28E-01	1.00E+00	2.40	2.08E-01	7.59E-01
**GJC1**	0.06	8.63E-01	1.00E+00	0.79	2.08E-01	7.59E-01
**KCNQ3**	-0.21	7.53E-01	1.00E+00	0.91	2.08E-01	7.59E-01
**YWHAE**	0.04	8.08E-01	1.00E+00	-0.25	2.08E-01	7.59E-01
**AC105345.1**	0.63	5.17E-01	1.00E+00	1.60	2.08E-01	7.60E-01
**GNRHR2**	-0.42	2.87E-01	1.00E+00	0.79	2.08E-01	7.60E-01
**AL031429.1**	2.11	5.39E-01	1.00E+00	-1.59	2.08E-01	7.60E-01
**PLPP5**	-0.41	7.95E-02	1.00E+00	-0.33	2.08E-01	7.60E-01
**XLOC_008979**	0.31	7.52E-01	1.00E+00	1.27	2.08E-01	7.60E-01
**PLA2G7**	0.20	7.81E-01	1.00E+00	0.64	2.08E-01	7.60E-01
**ZNF90**	0.09	8.85E-01	1.00E+00	-0.72	2.08E-01	7.60E-01
**SQOR**	0.12	6.22E-01	1.00E+00	0.29	2.08E-01	7.60E-01

**GRB2**	0.04	8.16E-01	1.00E+00	0.37	2.08E-01	7.60E-01
**RPL10A**	-0.17	6.13E-01	1.00E+00	0.38	2.08E-01	7.60E-01
**SOS2**	0.25	2.04E-01	1.00E+00	-0.41	2.08E-01	7.60E-01
**HLA-J**	0.16	9.47E-01	1.00E+00	0.80	2.09E-01	7.60E-01
**GJD3**	-0.81	2.77E-01	1.00E+00	1.44	2.09E-01	7.60E-01
**MFSD14B**	0.36	1.24E-01	1.00E+00	-0.48	2.09E-01	7.60E-01
**CFAP73**	-0.72	4.07E-01	1.00E+00	-1.00	2.09E-01	7.60E-01
**USP38**	-0.07	8.24E-01	1.00E+00	0.32	2.09E-01	7.60E-01
**NDUFA12**	0.11	6.14E-01	1.00E+00	-0.53	2.09E-01	7.60E-01
**SUPT6H**	-0.13	5.05E-01	1.00E+00	0.55	2.09E-01	7.60E-01
**SORBS1**	1.30	6.88E-02	1.00E+00	-0.65	2.09E-01	7.60E-01
**TRGV5**	2.54	2.57E-01	1.00E+00	1.60	2.09E-01	7.60E-01
**AC233976.1**	-0.34	8.98E-01	1.00E+00	2.09	2.09E-01	7.60E-01
**SPTY2D1**	0.04	8.74E-01	1.00E+00	0.27	2.09E-01	7.60E-01
**AP000695.2**	-0.92	6.59E-01	1.00E+00	1.37	2.09E-01	7.60E-01
**DENND4A**	0.09	6.77E-01	1.00E+00	0.44	2.09E-01	7.61E-01
**AL357568.2**	-1.65	6.32E-01	1.00E+00	-2.14	2.09E-01	7.61E-01
**SLC12A7**	-0.42	3.00E-01	1.00E+00	0.82	2.09E-01	7.61E-01
**ZNF639**	0.04	8.35E-01	1.00E+00	-0.49	2.09E-01	7.61E-01
**AC137932.1**	0.38	5.44E-01	1.00E+00	1.00	2.09E-01	7.61E-01
**ACSBG1**	-0.15	8.38E-01	1.00E+00	-1.04	2.09E-01	7.61E-01
**TRAV16**	-2.36	3.87E-01	1.00E+00	1.66	2.09E-01	7.61E-01
**RASAL3**	-0.63	2.45E-01	1.00E+00	1.18	2.09E-01	7.61E-01
**AC004803.1**	-0.09	8.36E-01	1.00E+00	-0.57	2.10E-01	7.61E-01
**VAV2**	0.03	8.88E-01	1.00E+00	0.97	2.10E-01	7.61E-01
**G26223**	1.76	9.11E-02	1.00E+00	1.56	2.10E-01	7.61E-01
**TALDO1**	0.02	9.20E-01	1.00E+00	-0.21	2.10E-01	7.61E-01
**STX1A**	-0.70	3.62E-01	1.00E+00	0.99	2.10E-01	7.61E-01
**CUEDC2**	-0.09	6.67E-01	1.00E+00	0.21	2.10E-01	7.61E-01
**BLOC1S5**	0.42	7.84E-02	1.00E+00	-0.32	2.10E-01	7.61E-01
**XLOC_012145**	0.81	4.98E-01	1.00E+00	1.24	2.10E-01	7.61E-01
**RNF5**	0.01	9.58E-01	1.00E+00	-0.18	2.10E-01	7.61E-01

**NPIPB5**	-0.02	9.72E-01	1.00E+00	0.65	2.10E-01	7.62E-01
**HSPD1P10**	-1.66	4.66E-01	1.00E+00	-2.05	2.10E-01	7.62E-01
**GDPGP1**	0.23	7.33E-01	1.00E+00	-0.65	2.10E-01	7.62E-01
**YTHDF3-AS1**	0.25	5.74E-01	1.00E+00	-0.53	2.10E-01	7.62E-01
**TMEM87A**	0.27	7.44E-02	1.00E+00	-0.25	2.10E-01	7.62E-01
**NCBP3**	0.12	5.64E-01	1.00E+00	0.35	2.10E-01	7.62E-01
**TRAV4**	0.34	8.17E-01	1.00E+00	1.66	2.10E-01	7.62E-01
**XLOC_001195**	0.40	5.55E-01	1.00E+00	1.15	2.10E-01	7.62E-01
**STK39**	0.15	7.95E-01	1.00E+00	-0.56	2.10E-01	7.62E-01
**HVCN1**	0.06	9.04E-01	1.00E+00	0.47	2.10E-01	7.62E-01
**XLOC_007469**	-1.61	6.25E-01	1.00E+00	-1.03	2.10E-01	7.62E-01
**PSEN2**	-0.06	7.33E-01	1.00E+00	0.31	2.10E-01	7.62E-01
**PLA2G4D**	0.18	7.52E-01	1.00E+00	0.98	2.10E-01	7.62E-01
**AC093827.4**	0.00	9.90E-01	1.00E+00	0.56	2.10E-01	7.62E-01
**TMEM38B**	0.14	5.40E-01	1.00E+00	-0.47	2.10E-01	7.62E-01
**AC090970.2**	-1.18	1.34E-01	1.00E+00	-1.10	2.10E-01	7.62E-01
**AC108471.2**	-2.09	5.40E-01	1.00E+00	-1.26	2.10E-01	7.62E-01
**ACSS1**	0.02	9.10E-01	1.00E+00	-0.69	2.11E-01	7.62E-01
**HOXB9**	-1.07	1.61E-01	1.00E+00	1.45	2.11E-01	7.62E-01
**FAM189A2**	0.16	7.25E-01	1.00E+00	-0.87	2.11E-01	7.62E-01
**AL356317.1**	-0.24	8.42E-01	1.00E+00	-1.34	2.11E-01	7.62E-01
**BCL2L2**	0.39	2.91E-02	9.89E-01	0.38	2.11E-01	7.62E-01
**ARRB1**	0.27	3.18E-01	1.00E+00	0.36	2.11E-01	7.62E-01
**TFDP2**	0.08	7.94E-01	1.00E+00	-0.28	2.11E-01	7.62E-01
**IFFO1**	0.11	7.80E-01	1.00E+00	0.95	2.11E-01	7.62E-01
**FANCE**	0.12	7.04E-01	1.00E+00	-0.50	2.11E-01	7.62E-01
**MFSD10**	-0.08	7.81E-01	1.00E+00	0.27	2.11E-01	7.62E-01
**HLA-DRB9**	-0.58	8.54E-01	1.00E+00	0.87	2.11E-01	7.62E-01
**RUNX1T1**	0.49	3.72E-01	1.00E+00	0.54	2.11E-01	7.62E-01
**FHL3**	-0.28	3.53E-01	1.00E+00	0.38	2.11E-01	7.62E-01
**GABPB1**	-0.16	5.33E-01	1.00E+00	-0.31	2.11E-01	7.62E-01
**BFAR**	0.09	7.46E-01	1.00E+00	0.29	2.11E-01	7.62E-01

**AC025175.1**	1.04	5.48E-02	1.00E+00	-1.41	2.11E-01	7.62E-01
**SCAND2P**	0.73	1.79E-01	1.00E+00	0.84	2.11E-01	7.62E-01
**ALDH7A1P1**	0.43	8.27E-01	1.00E+00	-0.81	2.11E-01	7.62E-01
**NDUFS4**	0.08	7.01E-01	1.00E+00	-0.36	2.11E-01	7.62E-01
**XPO6**	-0.13	4.57E-01	1.00E+00	0.65	2.11E-01	7.62E-01
**DNM3OS**	0.45	3.65E-01	1.00E+00	0.53	2.11E-01	7.62E-01
**TIMM23**	-0.05	8.09E-01	1.00E+00	-0.34	2.11E-01	7.62E-01
**AC254562.3**	-0.15	7.84E-01	1.00E+00	0.90	2.11E-01	7.62E-01
**GPR12**	0.03	9.70E-01	1.00E+00	-0.96	2.11E-01	7.62E-01
**AC005332.6**	0.36	3.38E-01	1.00E+00	0.33	2.11E-01	7.62E-01
**XLOC_002293**	-0.03	9.77E-01	1.00E+00	0.96	2.11E-01	7.62E-01
**AC078819.1**	-0.70	3.30E-01	1.00E+00	0.69	2.11E-01	7.62E-01
**P2RX5-TAX1BP3**	-0.22	6.45E-01	1.00E+00	-0.88	2.11E-01	7.62E-01
**FAM177A1**	0.04	8.37E-01	1.00E+00	-0.34	2.11E-01	7.62E-01
**TRBV6-1**	-1.12	5.78E-01	1.00E+00	2.17	2.11E-01	7.62E-01
**P2RX1**	-0.80	3.10E-01	1.00E+00	-0.94	2.11E-01	7.62E-01
**ZNF322**	0.09	7.76E-01	1.00E+00	0.33	2.12E-01	7.62E-01
**DNAH3**	-0.37	4.94E-01	1.00E+00	-0.59	2.12E-01	7.62E-01
**BMS1P5**	-0.93	4.04E-01	1.00E+00	0.97	2.12E-01	7.62E-01
**CABP1**	0.72	5.10E-01	1.00E+00	0.91	2.12E-01	7.62E-01
**ZNF333**	0.04	8.75E-01	1.00E+00	0.33	2.12E-01	7.62E-01
**SLC22A31**	0.58	5.52E-01	1.00E+00	-1.22	2.12E-01	7.62E-01
**OSBPL1A**	0.08	7.91E-01	1.00E+00	-0.42	2.12E-01	7.62E-01
**HNRNPUL2**	0.10	6.13E-01	1.00E+00	0.58	2.12E-01	7.62E-01
**MAPRE1**	-0.01	9.64E-01	1.00E+00	0.23	2.12E-01	7.62E-01
**PANK4**	0.02	9.13E-01	1.00E+00	-0.30	2.12E-01	7.62E-01
**FAM180B**	0.56	4.30E-01	1.00E+00	0.81	2.12E-01	7.62E-01
**RP11-782C8.2**	-2.14	2.00E-01	1.00E+00	-1.08	2.12E-01	7.62E-01
**ZBTB16**	0.39	7.25E-01	1.00E+00	-0.83	2.12E-01	7.63E-01
**CNR2**	-2.47	4.70E-01	1.00E+00	2.14	2.12E-01	7.63E-01
**AKR7A2**	-0.11	6.31E-01	1.00E+00	-0.22	2.12E-01	7.63E-01
**AP001574.1**	-1.39	2.07E-01	1.00E+00	0.74	2.12E-01	7.63E-01

**AC015849.3**	0.85	3.07E-01	1.00E+00	1.19	2.12E-01	7.63E-01
**FO393419.3**	-1.68	3.87E-01	1.00E+00	1.20	2.12E-01	7.63E-01
**SLC25A25-AS1**	-0.86	5.67E-02	1.00E+00	1.26	2.12E-01	7.63E-01
**RINT1**	0.15	6.60E-01	1.00E+00	0.41	2.12E-01	7.63E-01
**DIO3OS**	0.64	4.62E-01	1.00E+00	0.92	2.12E-01	7.63E-01
**AC009053.2**	0.46	7.00E-01	1.00E+00	-1.21	2.12E-01	7.63E-01
**FAM161A**	0.14	6.96E-01	1.00E+00	-0.60	2.13E-01	7.63E-01
**G25496**	-0.58	6.59E-01	1.00E+00	-0.99	2.13E-01	7.63E-01
**GCNT4**	-0.28	5.63E-01	1.00E+00	0.51	2.13E-01	7.63E-01
**FSTL1**	0.78	1.48E-01	1.00E+00	0.65	2.13E-01	7.63E-01
**AC093827.3**	0.77	5.59E-01	1.00E+00	0.84	2.13E-01	7.63E-01
**CACFD1**	-0.35	1.80E-01	1.00E+00	0.64	2.13E-01	7.63E-01
**HRNR**	-1.78	9.61E-03	6.08E-01	3.91	2.13E-01	7.63E-01
**ANKRD18B**	0.29	5.87E-01	1.00E+00	-0.89	2.13E-01	7.63E-01
**G31132**	-2.77	1.40E-01	1.00E+00	0.85	2.13E-01	7.63E-01
**FNDC3B**	0.31	4.32E-01	1.00E+00	0.39	2.13E-01	7.63E-01
**HSD17B7**	-0.14	7.06E-01	1.00E+00	-0.44	2.13E-01	7.63E-01
**B9D1**	-0.31	4.23E-01	1.00E+00	0.35	2.13E-01	7.63E-01
**AC006978.1**	0.05	8.52E-01	1.00E+00	0.56	2.13E-01	7.63E-01
**HIST1H4K**	0.74	7.68E-01	1.00E+00	0.92	2.13E-01	7.63E-01
**U91328.1**	-0.08	8.44E-01	1.00E+00	-0.64	2.13E-01	7.63E-01
**NTAN1**	0.02	9.53E-01	1.00E+00	-0.42	2.13E-01	7.63E-01
**RNVU1-6**	-0.50	7.49E-01	1.00E+00	-1.02	2.13E-01	7.63E-01
**COPRS**	-0.16	6.01E-01	1.00E+00	-0.83	2.13E-01	7.63E-01
**COPS9**	0.02	8.76E-01	1.00E+00	-0.49	2.13E-01	7.63E-01
**AC008629.1**	-2.86	9.24E-02	1.00E+00	-1.00	2.13E-01	7.63E-01
**ITPKB**	0.09	6.59E-01	1.00E+00	0.47	2.13E-01	7.63E-01
**RHCG**	-0.64	3.19E-01	1.00E+00	0.91	2.13E-01	7.63E-01
**C3orf20**	-0.73	3.65E-01	1.00E+00	2.08	2.13E-01	7.63E-01
**INTU**	0.46	1.14E-01	1.00E+00	-0.52	2.13E-01	7.63E-01
**AC019069.1**	0.05	9.59E-01	1.00E+00	1.42	2.13E-01	7.64E-01
**C8orf37-AS1**	0.17	9.06E-01	1.00E+00	-0.96	2.13E-01	7.64E-01

**ACAT2**	-0.32	4.80E-01	1.00E+00	-0.59	2.13E-01	7.64E-01
**EEF1A1P9**	0.94	1.59E-01	1.00E+00	-0.48	2.14E-01	7.64E-01
**NPM1P27**	-0.07	8.49E-01	1.00E+00	-0.41	2.14E-01	7.64E-01
**COX6C**	0.02	9.03E-01	1.00E+00	-0.56	2.14E-01	7.64E-01
**AC246787.1**	1.01	3.30E-01	1.00E+00	-0.82	2.14E-01	7.64E-01
**HDAC1**	-0.16	4.73E-01	1.00E+00	0.23	2.14E-01	7.64E-01
**ZCWPW2**	-0.20	8.47E-01	1.00E+00	-0.89	2.14E-01	7.64E-01
**XLOC_007562**	-2.86	4.00E-01	1.00E+00	-2.27	2.14E-01	7.65E-01
**DRGX**	-0.18	8.64E-01	1.00E+00	-1.49	2.14E-01	7.65E-01
**SLC26A2**	0.33	5.99E-01	1.00E+00	-0.59	2.14E-01	7.65E-01
**KIAA0355**	-0.01	9.25E-01	1.00E+00	0.42	2.14E-01	7.65E-01
**LINC01678**	-0.38	6.53E-01	1.00E+00	1.01	2.14E-01	7.65E-01
**KLHL11**	-0.59	4.70E-02	1.00E+00	-0.87	2.14E-01	7.65E-01
**COL23A1**	0.19	6.48E-01	1.00E+00	1.13	2.14E-01	7.65E-01
**GMCL1**	0.41	4.08E-02	1.00E+00	-0.34	2.14E-01	7.65E-01
**REXO1**	-0.80	2.14E-02	8.94E-01	0.96	2.14E-01	7.65E-01
**FAIM**	0.32	2.98E-01	1.00E+00	-0.39	2.14E-01	7.65E-01
**MSRB1**	-0.06	8.27E-01	1.00E+00	-0.28	2.14E-01	7.65E-01
**DPY30**	0.00	9.94E-01	1.00E+00	-0.48	2.14E-01	7.65E-01
**LINC00205**	-0.46	1.99E-01	1.00E+00	0.61	2.14E-01	7.65E-01
**PPIP5K2**	0.17	6.02E-01	1.00E+00	-0.31	2.14E-01	7.65E-01
**BNIP3L**	0.32	1.80E-01	1.00E+00	-0.40	2.15E-01	7.65E-01
**UTP4**	0.08	6.85E-01	1.00E+00	-0.25	2.15E-01	7.65E-01
**ADRA2C**	-0.17	7.29E-01	1.00E+00	-1.06	2.15E-01	7.65E-01
**SLC22A2**	-1.32	5.57E-01	1.00E+00	1.48	2.15E-01	7.65E-01
**SNTG2**	-0.07	8.82E-01	1.00E+00	-1.18	2.15E-01	7.65E-01
**EPB41L4B**	0.22	4.01E-01	1.00E+00	-0.45	2.15E-01	7.65E-01
**ESCO2**	0.24	6.48E-01	1.00E+00	0.71	2.15E-01	7.65E-01
**NDUFA6**	-0.10	5.97E-01	1.00E+00	-0.39	2.15E-01	7.65E-01
**AC007249.2**	-0.70	3.41E-01	1.00E+00	0.96	2.15E-01	7.65E-01
**IRAK3**	0.73	8.76E-02	1.00E+00	0.71	2.15E-01	7.65E-01
**CYB5B**	0.08	6.57E-01	1.00E+00	-0.32	2.15E-01	7.65E-01

**DCTN6**	0.21	2.56E-01	1.00E+00	-0.43	2.15E-01	7.65E-01
**XLOC_013986**	0.05	9.65E-01	1.00E+00	0.93	2.15E-01	7.65E-01
**TEX101**	0.20	8.27E-01	1.00E+00	1.30	2.15E-01	7.65E-01
**HEXIM1**	-0.08	6.62E-01	1.00E+00	0.40	2.15E-01	7.65E-01
**MORC4**	-0.23	2.10E-01	1.00E+00	-0.43	2.15E-01	7.65E-01
**KLHL18**	0.08	8.45E-01	1.00E+00	0.37	2.15E-01	7.65E-01
**OIP5-AS1**	-0.03	9.57E-01	1.00E+00	-0.55	2.15E-01	7.65E-01
**HMGB1P6**	0.56	4.62E-02	1.00E+00	-0.40	2.15E-01	7.65E-01
**M6PR**	0.07	7.69E-01	1.00E+00	0.24	2.15E-01	7.66E-01
**RARA**	-0.32	2.57E-01	1.00E+00	0.82	2.15E-01	7.66E-01
**MRFAP1**	0.14	4.12E-01	1.00E+00	0.25	2.15E-01	7.66E-01
**PDCL3P4**	-0.50	7.47E-01	1.00E+00	-0.81	2.15E-01	7.66E-01
**CEP250**	0.00	9.95E-01	1.00E+00	0.63	2.15E-01	7.66E-01
**MED15**	-0.32	1.64E-01	1.00E+00	0.46	2.15E-01	7.66E-01
**DES**	-0.74	3.43E-01	1.00E+00	-0.91	2.15E-01	7.66E-01
**C20orf204**	0.07	8.80E-01	1.00E+00	1.04	2.16E-01	7.66E-01
**IGKV3-11**	4.57	1.49E-02	7.62E-01	3.89	2.16E-01	7.66E-01
**KDM2B**	-0.26	4.54E-01	1.00E+00	0.42	2.16E-01	7.66E-01
**MYO5C**	0.09	8.66E-01	1.00E+00	-0.61	2.16E-01	7.66E-01
**NDUFB4P12**	NA	NA	NA	-0.91	2.16E-01	7.66E-01
**SELENOS**	0.09	6.44E-01	1.00E+00	-0.34	2.16E-01	7.67E-01
**PRDX6**	0.06	7.20E-01	1.00E+00	-0.20	2.16E-01	7.67E-01
**WISP3**	0.15	8.18E-01	1.00E+00	0.76	2.16E-01	7.67E-01
**MYH7**	0.21	8.95E-01	1.00E+00	1.68	2.16E-01	7.67E-01
**AL592546.1**	-2.39	3.74E-01	1.00E+00	1.46	2.16E-01	7.67E-01
**KIF4A**	0.23	4.09E-01	1.00E+00	0.53	2.16E-01	7.67E-01
**ZNF195**	0.00	9.98E-01	1.00E+00	-0.34	2.16E-01	7.67E-01
**LINC02019**	0.14	8.77E-01	1.00E+00	0.81	2.16E-01	7.67E-01
**DUSP6**	0.70	9.45E-02	1.00E+00	0.46	2.16E-01	7.67E-01
**CDS2**	0.39	2.96E-01	1.00E+00	0.32	2.16E-01	7.67E-01
**CDK2AP1**	0.08	7.12E-01	1.00E+00	-0.66	2.16E-01	7.67E-01
**PDE7B**	0.26	7.16E-01	1.00E+00	0.74	2.16E-01	7.67E-01

**EMILIN2**	0.20	6.40E-01	1.00E+00	1.04	2.16E-01	7.67E-01
**COMMD8**	-0.06	8.32E-01	1.00E+00	-0.53	2.16E-01	7.67E-01
**G39823**	-0.29	7.51E-01	1.00E+00	-1.05	2.16E-01	7.67E-01
**PLEKHA3**	0.38	2.40E-01	1.00E+00	-0.43	2.17E-01	7.67E-01
**TNFAIP3**	-1.00	3.34E-01	1.00E+00	0.60	2.17E-01	7.67E-01
**Z68871.1**	-2.98	1.00E-02	6.22E-01	0.56	2.17E-01	7.67E-01
**PRSS51**	-0.52	5.78E-01	1.00E+00	1.90	2.17E-01	7.68E-01
**CNTN3**	0.95	2.37E-02	9.15E-01	0.99	2.17E-01	7.68E-01
**GNG11**	0.34	4.73E-01	1.00E+00	-0.58	2.17E-01	7.68E-01
**CYC1**	-0.26	2.48E-01	1.00E+00	-0.31	2.17E-01	7.68E-01
**ARID3C**	-1.02	1.80E-01	1.00E+00	-1.16	2.17E-01	7.68E-01
**AL022323.1**	-0.39	8.37E-01	1.00E+00	-0.92	2.17E-01	7.68E-01
**PPP2R5D**	-0.02	8.87E-01	1.00E+00	0.36	2.17E-01	7.68E-01
**ROMO1**	-0.10	5.89E-01	1.00E+00	-0.42	2.17E-01	7.68E-01
**HMGN3-AS1**	0.08	8.54E-01	1.00E+00	0.40	2.17E-01	7.68E-01
**FRG1**	-0.04	8.01E-01	1.00E+00	-0.32	2.17E-01	7.68E-01
**MEX3C**	0.13	5.70E-01	1.00E+00	0.49	2.17E-01	7.68E-01
**C1orf158**	-0.60	8.61E-01	1.00E+00	-2.20	2.17E-01	7.68E-01
**ITGB1BP1**	0.32	2.99E-01	1.00E+00	-0.48	2.17E-01	7.68E-01
**BCL2L11**	-0.13	7.56E-01	1.00E+00	0.68	2.17E-01	7.68E-01
**AC007728.2**	-2.97	7.46E-02	1.00E+00	1.44	2.18E-01	7.68E-01
**AC022730.4**	-3.06	2.17E-01	1.00E+00	2.38	2.18E-01	7.68E-01
**ARHGAP42**	-0.08	7.74E-01	1.00E+00	0.40	2.18E-01	7.68E-01
**LIMK2**	-0.02	9.51E-01	1.00E+00	0.39	2.18E-01	7.68E-01
**CDIPT**	0.17	3.78E-01	1.00E+00	-0.59	2.18E-01	7.68E-01
**CYB5R1**	0.13	6.10E-01	1.00E+00	-0.32	2.18E-01	7.68E-01
**PARP14**	0.07	8.91E-01	1.00E+00	0.65	2.18E-01	7.68E-01
**AP000962.1**	0.08	9.78E-01	1.00E+00	-1.15	2.18E-01	7.68E-01
**AZU1**	-1.54	6.54E-01	1.00E+00	2.03	2.18E-01	7.68E-01
**AL009178.2**	-0.60	3.91E-01	1.00E+00	1.43	2.18E-01	7.68E-01
**MIPEP**	-0.37	3.06E-01	1.00E+00	-0.47	2.18E-01	7.68E-01
**NAA30**	0.14	5.36E-01	1.00E+00	-0.38	2.18E-01	7.68E-01

**GNAZ**	-0.18	7.41E-01	1.00E+00	-0.83	2.18E-01	7.68E-01
**HMGN2P4**	0.94	7.83E-01	1.00E+00	-0.54	2.18E-01	7.69E-01
**MPPE1**	0.21	4.16E-01	1.00E+00	0.21	2.18E-01	7.69E-01
**STK10**	-0.18	5.94E-01	1.00E+00	0.66	2.18E-01	7.69E-01
**CSNK2A1**	0.06	7.22E-01	1.00E+00	0.28	2.18E-01	7.69E-01
**HILPDA**	0.32	4.61E-01	1.00E+00	-0.42	2.18E-01	7.69E-01
**G5109**	1.23	5.49E-01	1.00E+00	-1.01	2.18E-01	7.69E-01
**THEG**	-1.24	1.23E-01	1.00E+00	0.71	2.18E-01	7.69E-01
**JPH4**	-0.98	6.85E-02	1.00E+00	0.91	2.18E-01	7.69E-01
**XLOC_004398**	-2.13	5.36E-01	1.00E+00	-3.25	2.18E-01	7.69E-01
**CLIP3**	0.16	7.70E-01	1.00E+00	0.70	2.18E-01	7.69E-01
**NOTCH1**	-0.36	1.11E-01	1.00E+00	0.74	2.18E-01	7.69E-01
**ZNF709**	1.34	6.97E-01	1.00E+00	-1.03	2.18E-01	7.69E-01
**CPNE3**	0.14	5.17E-01	1.00E+00	-0.28	2.18E-01	7.69E-01
**AC008267.3**	0.95	2.98E-01	1.00E+00	0.95	2.18E-01	7.69E-01
**COX17**	0.18	5.26E-01	1.00E+00	-0.53	2.19E-01	7.69E-01
**PNPLA3**	0.91	6.74E-02	1.00E+00	-0.67	2.19E-01	7.69E-01
**HOXA2**	0.14	8.88E-01	1.00E+00	-0.96	2.19E-01	7.69E-01
**MAGIX**	-0.14	7.11E-01	1.00E+00	-0.53	2.19E-01	7.69E-01
**ARPC1A**	0.38	1.31E-01	1.00E+00	-0.55	2.19E-01	7.70E-01
**ASB4**	0.32	6.86E-01	1.00E+00	-1.49	2.19E-01	7.70E-01
**TTC26**	-0.28	4.20E-01	1.00E+00	0.36	2.19E-01	7.70E-01
**CTBP2P8**	-2.18	3.45E-01	1.00E+00	-1.00	2.19E-01	7.70E-01
**CISD2**	0.19	4.69E-01	1.00E+00	-0.41	2.19E-01	7.70E-01
**FRY**	0.07	7.18E-01	1.00E+00	-0.42	2.19E-01	7.70E-01
**HSPA6**	-0.42	4.10E-01	1.00E+00	0.70	2.19E-01	7.70E-01
**DSG3**	-0.32	3.22E-01	1.00E+00	0.73	2.19E-01	7.70E-01
**AC010531.6**	0.51	5.59E-01	1.00E+00	-0.91	2.19E-01	7.70E-01
**IRF2BPL**	-0.07	7.97E-01	1.00E+00	0.68	2.19E-01	7.70E-01
**CLDN15**	0.08	8.04E-01	1.00E+00	0.59	2.19E-01	7.70E-01
**LINC01778**	1.11	6.06E-01	1.00E+00	-0.99	2.19E-01	7.70E-01
**PQBP1**	-0.25	1.49E-01	1.00E+00	0.24	2.19E-01	7.70E-01

**LPCAT2**	-0.17	4.84E-01	1.00E+00	-0.18	2.19E-01	7.70E-01
**NPY5R**	0.93	2.58E-01	1.00E+00	-0.75	2.19E-01	7.70E-01
**PPM1L**	-0.20	2.59E-01	1.00E+00	-0.38	2.19E-01	7.70E-01
**TUBB2B**	-0.56	3.98E-01	1.00E+00	0.61	2.19E-01	7.70E-01
**AL356481.1**	-0.90	8.68E-02	1.00E+00	-0.69	2.19E-01	7.70E-01
**TMIGD3**	0.14	8.35E-01	1.00E+00	1.29	2.20E-01	7.70E-01
**MRPL43**	-0.15	4.39E-01	1.00E+00	-0.19	2.20E-01	7.70E-01
**SHH**	-3.40	3.42E-02	1.00E+00	-1.45	2.20E-01	7.70E-01
**KCNAB1**	0.08	9.12E-01	1.00E+00	-0.70	2.20E-01	7.70E-01
**G12163**	-1.43	7.46E-02	1.00E+00	-1.01	2.20E-01	7.71E-01
**RMRP**	0.02	9.67E-01	1.00E+00	0.88	2.20E-01	7.71E-01
**WDR13**	-0.08	7.44E-01	1.00E+00	0.22	2.20E-01	7.71E-01
**CCDC59**	-0.10	5.44E-01	1.00E+00	-0.29	2.20E-01	7.71E-01
**AC011487.2**	1.20	7.22E-01	1.00E+00	1.80	2.20E-01	7.71E-01
**AC083855.2**	0.73	5.91E-02	1.00E+00	0.46	2.20E-01	7.71E-01
**WDR31**	-0.01	9.65E-01	1.00E+00	-0.63	2.20E-01	7.71E-01
**XLOC_005587**	0.73	1.49E-01	1.00E+00	0.71	2.20E-01	7.71E-01
**FAM209B**	1.03	5.60E-01	1.00E+00	1.00	2.20E-01	7.71E-01
**AC011632.2**	-3.09	6.86E-02	1.00E+00	-1.34	2.20E-01	7.71E-01
**NR0B1**	0.76	7.85E-01	1.00E+00	-2.42	2.20E-01	7.71E-01
**GNA15**	0.11	8.01E-01	1.00E+00	0.59	2.20E-01	7.71E-01
**ANKRD52**	-0.12	6.68E-01	1.00E+00	0.83	2.20E-01	7.71E-01
**SHROOM4**	-0.03	9.29E-01	1.00E+00	0.71	2.20E-01	7.71E-01
**BX322234.1**	-0.39	3.45E-01	1.00E+00	0.77	2.20E-01	7.71E-01
**XLOC_006390**	0.50	5.58E-01	1.00E+00	1.17	2.20E-01	7.71E-01
**HOXA6**	-1.00	1.11E-01	1.00E+00	-0.59	2.20E-01	7.71E-01
**NCS1**	-0.15	6.69E-01	1.00E+00	-0.61	2.20E-01	7.71E-01
**SV2A**	-0.67	3.28E-01	1.00E+00	1.13	2.20E-01	7.71E-01
**AC092123.1**	-3.78	4.07E-02	1.00E+00	-0.93	2.21E-01	7.71E-01
**MICALL1**	-0.29	3.98E-01	1.00E+00	0.75	2.21E-01	7.71E-01
**WDR81**	-0.27	2.31E-01	1.00E+00	0.80	2.21E-01	7.71E-01
**CECR7**	-0.55	2.26E-01	1.00E+00	-0.43	2.21E-01	7.71E-01

**RPS15**	-0.10	7.02E-01	1.00E+00	0.36	2.21E-01	7.71E-01
**ARMC5**	-0.25	3.80E-01	1.00E+00	0.59	2.21E-01	7.71E-01
**SH2D1A**	-0.63	5.48E-01	1.00E+00	1.04	2.21E-01	7.71E-01
**PIP**	-0.83	6.20E-01	1.00E+00	-3.83	2.21E-01	7.71E-01
**XLOC_011981**	0.81	7.11E-01	1.00E+00	1.75	2.21E-01	7.72E-01
**IFITM10**	-0.65	1.84E-01	1.00E+00	1.06	2.21E-01	7.72E-01
**RPP14**	0.16	5.13E-01	1.00E+00	0.31	2.21E-01	7.72E-01
**LINC01352**	1.46	3.22E-02	1.00E+00	1.16	2.21E-01	7.72E-01
**MFAP3**	0.18	4.60E-01	1.00E+00	0.31	2.21E-01	7.72E-01
**TMEM63A**	0.01	9.75E-01	1.00E+00	0.56	2.21E-01	7.72E-01
**KDM4A**	0.14	4.65E-01	1.00E+00	0.47	2.21E-01	7.72E-01
**MSRB2**	0.24	5.76E-01	1.00E+00	-0.82	2.21E-01	7.72E-01
**RPS4Y1**	1.03	6.28E-01	1.00E+00	-2.52	2.21E-01	7.72E-01
**TECPR2**	-0.12	5.26E-01	1.00E+00	0.65	2.21E-01	7.72E-01
**AL354732.1**	-1.54	3.95E-01	1.00E+00	1.92	2.21E-01	7.72E-01
**AL139339.1**	-0.75	4.69E-01	1.00E+00	1.12	2.21E-01	7.72E-01
**C1QTNF12**	-1.00	4.88E-02	1.00E+00	-0.89	2.21E-01	7.72E-01
**AC096677.1**	-0.43	6.38E-01	1.00E+00	-0.65	2.21E-01	7.72E-01
**TMEM120A**	0.14	5.22E-01	1.00E+00	-0.29	2.21E-01	7.72E-01
**AL121987.1**	0.70	7.32E-01	1.00E+00	-1.50	2.21E-01	7.72E-01
**C9orf24**	-0.33	7.14E-01	1.00E+00	-0.75	2.21E-01	7.72E-01
**XLOC_007131**	NA	NA	NA	-2.23	2.21E-01	7.72E-01
**AP001189.3**	-0.26	6.00E-01	1.00E+00	1.39	2.22E-01	7.72E-01
**CAND1**	0.00	9.86E-01	1.00E+00	-0.24	2.22E-01	7.72E-01
**CDK20**	-0.28	3.13E-01	1.00E+00	0.42	2.22E-01	7.72E-01
**G28650**	0.30	7.58E-01	1.00E+00	-1.21	2.22E-01	7.72E-01
**MIR4292**	-0.83	4.06E-01	1.00E+00	2.31	2.22E-01	7.72E-01
**JCAD**	0.14	7.73E-01	1.00E+00	0.77	2.22E-01	7.72E-01
**SNHG15**	-0.33	4.10E-01	1.00E+00	0.34	2.22E-01	7.72E-01
**MTMR14**	0.05	7.77E-01	1.00E+00	0.23	2.22E-01	7.72E-01
**SMIM2-AS1**	0.55	5.64E-01	1.00E+00	-0.81	2.22E-01	7.72E-01
**ZNF433-AS1**	-0.83	2.18E-01	1.00E+00	-0.65	2.22E-01	7.72E-01

**LINC01410**	-0.50	4.49E-01	1.00E+00	0.80	2.22E-01	7.72E-01
**KCTD11**	-0.34	5.25E-01	1.00E+00	0.57	2.22E-01	7.72E-01
**TBC1D4**	0.02	9.06E-01	1.00E+00	-0.30	2.22E-01	7.72E-01
**UGP2**	0.49	2.08E-01	1.00E+00	-0.37	2.22E-01	7.72E-01
**APCDD1**	-0.10	7.44E-01	1.00E+00	-0.54	2.22E-01	7.72E-01
**ARFIP2**	-0.10	5.31E-01	1.00E+00	0.16	2.22E-01	7.72E-01
**IGBP1**	0.02	9.31E-01	1.00E+00	0.26	2.22E-01	7.72E-01
**HOXC-AS1**	-0.50	3.58E-01	1.00E+00	-0.73	2.22E-01	7.73E-01
**REXO5**	0.26	4.09E-01	1.00E+00	-0.43	2.22E-01	7.73E-01
**CNIH4**	0.26	2.46E-01	1.00E+00	-0.51	2.22E-01	7.73E-01
**RFC1**	0.11	5.28E-01	1.00E+00	-0.22	2.22E-01	7.73E-01
**CARS2**	0.04	8.79E-01	1.00E+00	-0.59	2.22E-01	7.73E-01
**BBS9**	0.25	2.71E-01	1.00E+00	-0.25	2.22E-01	7.73E-01
**GAREM2**	-0.68	1.14E-01	1.00E+00	0.59	2.22E-01	7.73E-01
**MCOLN1**	-0.26	2.42E-01	1.00E+00	0.23	2.23E-01	7.73E-01
**IRGQ**	0.28	4.77E-01	1.00E+00	-0.27	2.23E-01	7.73E-01
**HS2ST1**	0.09	6.78E-01	1.00E+00	0.33	2.23E-01	7.73E-01
**SLC18A2**	-0.09	8.87E-01	1.00E+00	-0.60	2.23E-01	7.73E-01
**MSL1**	-0.17	5.32E-01	1.00E+00	0.59	2.23E-01	7.73E-01
**AC011468.4**	0.70	7.48E-01	1.00E+00	1.57	2.23E-01	7.74E-01
**FCHO2**	0.04	8.59E-01	1.00E+00	0.23	2.23E-01	7.74E-01
**RAD21-AS1**	-0.07	8.99E-01	1.00E+00	0.94	2.23E-01	7.74E-01
**SAP30**	0.15	6.29E-01	1.00E+00	-0.81	2.23E-01	7.74E-01
**YIPF5**	0.08	6.19E-01	1.00E+00	-0.23	2.23E-01	7.74E-01
**RNASEH2B**	0.03	9.07E-01	1.00E+00	-0.29	2.23E-01	7.74E-01
**TENT5B**	-0.59	2.12E-01	1.00E+00	-0.70	2.23E-01	7.74E-01
**ABCA5**	0.14	5.79E-01	1.00E+00	-0.34	2.23E-01	7.74E-01
**INTS4**	-0.19	4.24E-01	1.00E+00	-0.21	2.23E-01	7.74E-01
**BCKDHB**	-0.33	1.90E-01	1.00E+00	-0.42	2.23E-01	7.74E-01
**SPIN4**	-0.26	5.37E-01	1.00E+00	0.46	2.23E-01	7.74E-01
**ADCY3**	0.05	8.04E-01	1.00E+00	0.79	2.23E-01	7.74E-01
**NDRG1**	0.10	6.81E-01	1.00E+00	0.61	2.23E-01	7.74E-01

**SUMO3**	0.15	3.65E-01	1.00E+00	0.20	2.23E-01	7.74E-01
**SP140L**	-0.08	8.76E-01	1.00E+00	0.76	2.24E-01	7.74E-01
**PCDHB2**	-0.01	9.80E-01	1.00E+00	-0.60	2.24E-01	7.74E-01
**ATAT1**	-0.04	9.28E-01	1.00E+00	0.57	2.24E-01	7.74E-01
**C8orf37**	-0.67	4.12E-02	1.00E+00	-0.57	2.24E-01	7.74E-01
**CNOT11**	-0.04	8.68E-01	1.00E+00	-0.58	2.24E-01	7.74E-01
**GPR61**	-1.22	2.08E-01	1.00E+00	-1.11	2.24E-01	7.74E-01
**AP001528.3**	1.44	2.52E-01	1.00E+00	-0.79	2.24E-01	7.74E-01
**POLR3GL**	0.10	7.22E-01	1.00E+00	-0.40	2.24E-01	7.74E-01
**SH3BGRL**	-0.11	5.61E-01	1.00E+00	-0.27	2.24E-01	7.74E-01
**XLOC_013570**	-1.39	4.66E-01	1.00E+00	1.77	2.24E-01	7.74E-01
**COMMD6**	0.08	7.28E-01	1.00E+00	-0.56	2.24E-01	7.74E-01
**G35951**	-0.82	4.77E-01	1.00E+00	-1.21	2.24E-01	7.74E-01
**PIN4**	-0.05	7.96E-01	1.00E+00	-0.35	2.24E-01	7.74E-01
**OR3A2**	-0.15	9.67E-01	1.00E+00	-2.99	2.24E-01	7.74E-01
**SRI**	0.17	4.29E-01	1.00E+00	-0.44	2.24E-01	7.74E-01
**ALMS1P1**	-2.14	2.49E-01	1.00E+00	1.71	2.24E-01	7.74E-01
**G32308**	-2.93	3.88E-01	1.00E+00	2.62	2.24E-01	7.74E-01
**MROH8**	0.39	4.95E-01	1.00E+00	-0.55	2.24E-01	7.74E-01
**ERN2**	-2.84	3.87E-01	1.00E+00	-1.13	2.24E-01	7.74E-01
**CMTR1**	0.13	5.94E-01	1.00E+00	0.52	2.24E-01	7.74E-01
**KRTAP5-AS1**	-1.94	1.58E-01	1.00E+00	1.25	2.24E-01	7.74E-01
**SCRIB**	-0.46	1.74E-01	1.00E+00	0.94	2.24E-01	7.74E-01
**XLOC_010043**	-1.04	7.17E-01	1.00E+00	1.33	2.24E-01	7.74E-01
**ARG1**	0.62	3.40E-01	1.00E+00	0.83	2.24E-01	7.74E-01
**AL133406.3**	0.29	6.68E-01	1.00E+00	1.27	2.24E-01	7.74E-01
**NR1D2**	-0.66	2.09E-03	2.39E-01	-0.36	2.24E-01	7.74E-01
**LAPTM4A**	0.28	9.15E-02	1.00E+00	-0.36	2.24E-01	7.74E-01
**AC020978.3**	-0.38	7.93E-01	1.00E+00	-1.55	2.24E-01	7.74E-01
**CNFN**	0.11	8.48E-01	1.00E+00	0.75	2.25E-01	7.75E-01
**GSTA4**	-0.05	8.90E-01	1.00E+00	-0.45	2.25E-01	7.75E-01
**TRAV8-3**	-2.23	5.04E-01	1.00E+00	1.72	2.25E-01	7.75E-01

**ADAM9**	0.23	3.92E-01	1.00E+00	0.39	2.25E-01	7.75E-01
**AC009152.3**	-2.47	4.69E-01	1.00E+00	1.73	2.25E-01	7.75E-01
**ARHGAP18**	0.23	5.14E-01	1.00E+00	0.33	2.25E-01	7.75E-01
**EEF2K**	0.29	3.76E-01	1.00E+00	-0.42	2.25E-01	7.75E-01
**HOXC-AS2**	-0.79	1.96E-01	1.00E+00	-0.75	2.25E-01	7.75E-01
**AQP4**	3.44	1.99E-01	1.00E+00	-1.51	2.25E-01	7.75E-01
**CDC7**	-0.42	1.94E-01	1.00E+00	0.43	2.25E-01	7.75E-01
**LINC01936**	0.47	5.02E-01	1.00E+00	1.05	2.25E-01	7.75E-01
**XLOC_005173**	-3.12	2.14E-01	1.00E+00	-3.88	2.25E-01	7.75E-01
**CD1E**	0.31	6.29E-01	1.00E+00	-0.81	2.25E-01	7.75E-01
**COMMD9**	-0.03	8.59E-01	1.00E+00	-0.36	2.25E-01	7.75E-01
**GM2A**	-0.14	6.38E-01	1.00E+00	0.41	2.25E-01	7.75E-01
**MIA2**	-0.05	8.54E-01	1.00E+00	-0.19	2.25E-01	7.75E-01
**EHD4**	-0.39	4.72E-02	1.00E+00	0.42	2.25E-01	7.75E-01
**CEP19**	0.06	9.17E-01	1.00E+00	-0.71	2.25E-01	7.75E-01
**CDIP1**	-0.04	8.63E-01	1.00E+00	0.37	2.25E-01	7.75E-01
**AC020915.2**	-1.28	3.91E-01	1.00E+00	1.13	2.25E-01	7.75E-01
**TNRC6C-AS1**	-0.36	4.91E-01	1.00E+00	1.30	2.25E-01	7.75E-01
**GCNT1**	-0.29	4.96E-01	1.00E+00	0.47	2.26E-01	7.75E-01
**NXPH4**	-0.28	5.31E-01	1.00E+00	-0.79	2.26E-01	7.75E-01
**TXNDC16**	-0.02	9.34E-01	1.00E+00	-0.26	2.26E-01	7.75E-01
**ANKRD27**	0.22	3.61E-01	1.00E+00	-0.29	2.26E-01	7.75E-01
**HNRNPA1P76**	NA	NA	NA	-2.07	2.26E-01	7.75E-01
**XLOC_010097**	0.10	8.74E-01	1.00E+00	-0.67	2.26E-01	7.75E-01
**RPS20**	-0.07	8.12E-01	1.00E+00	0.28	2.26E-01	7.75E-01
**MUC12**	-0.94	5.03E-01	1.00E+00	1.01	2.26E-01	7.75E-01
**PLK1**	0.55	1.16E-01	1.00E+00	0.43	2.26E-01	7.75E-01
**COL6A4P1**	0.18	9.09E-01	1.00E+00	1.31	2.26E-01	7.75E-01
**AC016405.2**	-2.01	4.67E-01	1.00E+00	-1.28	2.26E-01	7.75E-01
**AOX1**	1.07	5.12E-02	1.00E+00	0.79	2.26E-01	7.75E-01
**GPD1L**	0.16	5.21E-01	1.00E+00	-0.43	2.26E-01	7.75E-01
**ADAL**	-0.18	6.32E-01	1.00E+00	-0.39	2.26E-01	7.75E-01

**CCDC85A**	-0.23	6.68E-01	1.00E+00	-0.92	2.26E-01	7.75E-01
**PWWP2B**	0.03	9.16E-01	1.00E+00	0.54	2.26E-01	7.75E-01
**WDR45BP1**	-0.21	9.53E-01	1.00E+00	-1.36	2.26E-01	7.75E-01
**NRG3**	-0.44	6.21E-01	1.00E+00	-1.36	2.26E-01	7.75E-01
**LINC01119**	0.71	4.03E-01	1.00E+00	1.27	2.26E-01	7.75E-01
**SDCBP2**	0.02	9.65E-01	1.00E+00	0.49	2.26E-01	7.75E-01
**LRRIQ1**	-1.35	1.18E-01	1.00E+00	-1.12	2.26E-01	7.75E-01
**AL356599.1**	-0.27	4.40E-01	1.00E+00	-0.55	2.26E-01	7.75E-01
**RPS23P1**	-3.34	1.14E-01	1.00E+00	1.65	2.26E-01	7.75E-01
**AC004765.1**	-0.50	8.71E-01	1.00E+00	-1.97	2.26E-01	7.75E-01
**SMO**	-0.12	6.47E-01	1.00E+00	0.54	2.26E-01	7.75E-01
**RCSD1**	0.32	3.07E-01	1.00E+00	0.81	2.26E-01	7.75E-01
**ACIN1**	-0.17	4.33E-01	1.00E+00	0.52	2.26E-01	7.75E-01
**TTPA**	1.04	7.58E-01	1.00E+00	-1.27	2.26E-01	7.75E-01
**AC083873.1**	0.80	5.35E-01	1.00E+00	-0.67	2.26E-01	7.75E-01
**MMP25**	-0.76	3.14E-01	1.00E+00	1.33	2.26E-01	7.75E-01
**HOXA3**	-0.34	5.22E-01	1.00E+00	-0.55	2.26E-01	7.75E-01
**CALM2P2**	0.88	5.20E-01	1.00E+00	-0.74	2.26E-01	7.75E-01
**NUCB2**	-0.07	8.40E-01	1.00E+00	-0.42	2.26E-01	7.75E-01
**ZDHHC4**	0.25	5.81E-01	1.00E+00	-0.38	2.26E-01	7.75E-01
**DDX59**	-0.10	6.75E-01	1.00E+00	-0.25	2.26E-01	7.75E-01
**ISCA2**	0.12	6.26E-01	1.00E+00	-0.49	2.26E-01	7.75E-01
**PINX1**	-0.38	2.16E-01	1.00E+00	0.36	2.26E-01	7.75E-01
**TRAV19**	3.48	5.98E-03	4.59E-01	1.97	2.27E-01	7.75E-01
**SMAD7**	-0.12	5.04E-01	1.00E+00	0.77	2.27E-01	7.75E-01
**EPHB6**	-0.18	7.00E-01	1.00E+00	-0.57	2.27E-01	7.75E-01
**RNF144B**	-0.04	9.03E-01	1.00E+00	0.30	2.27E-01	7.75E-01
**ASXL1**	-0.07	7.82E-01	1.00E+00	0.56	2.27E-01	7.75E-01
**AC090971.3**	-2.41	1.61E-01	1.00E+00	0.94	2.27E-01	7.75E-01
**MPZL2**	-0.31	3.13E-01	1.00E+00	0.44	2.27E-01	7.75E-01
**ABHD3**	0.23	5.26E-01	1.00E+00	-0.39	2.27E-01	7.75E-01
**C9orf50**	-0.82	5.63E-01	1.00E+00	-1.45	2.27E-01	7.75E-01

**TRGV10**	2.53	2.37E-01	1.00E+00	2.23	2.27E-01	7.75E-01
**CYSTM1**	0.06	8.91E-01	1.00E+00	-0.42	2.27E-01	7.75E-01
**AC007690.1**	1.47	6.67E-01	1.00E+00	-1.60	2.27E-01	7.75E-01
**FBL**	-0.13	6.68E-01	1.00E+00	0.41	2.27E-01	7.75E-01
**KIAA0825**	-0.02	9.61E-01	1.00E+00	-0.62	2.27E-01	7.75E-01
**SYPL1**	0.09	6.57E-01	1.00E+00	-0.33	2.27E-01	7.75E-01
**AC008440.3**	0.86	2.32E-01	1.00E+00	0.95	2.27E-01	7.75E-01
**UBE2Q2L**	1.32	3.47E-01	1.00E+00	-1.22	2.27E-01	7.75E-01
**THRB**	0.12	6.24E-01	1.00E+00	-0.31	2.27E-01	7.75E-01
**TPX2**	-0.06	8.60E-01	1.00E+00	0.45	2.27E-01	7.75E-01
**TRMO**	0.21	2.78E-01	1.00E+00	0.29	2.27E-01	7.75E-01
**AL161908.1**	-1.28	2.83E-01	1.00E+00	-1.39	2.27E-01	7.75E-01
**SLC35F5**	0.42	2.52E-01	1.00E+00	-0.32	2.27E-01	7.75E-01
**SYNPO2L**	-2.33	2.53E-01	1.00E+00	1.64	2.27E-01	7.75E-01
**ZNF556**	-2.95	2.66E-01	1.00E+00	2.22	2.27E-01	7.75E-01
**HMGB1P10**	-0.09	7.81E-01	1.00E+00	-0.32	2.27E-01	7.76E-01
**CALML5**	-0.05	9.25E-01	1.00E+00	0.80	2.27E-01	7.76E-01
**CATSPERB**	0.33	6.93E-01	1.00E+00	1.22	2.27E-01	7.76E-01
**BHLHE40-AS1**	0.34	5.10E-01	1.00E+00	-0.57	2.27E-01	7.76E-01
**MIR4697HG**	-0.07	9.06E-01	1.00E+00	-1.04	2.28E-01	7.76E-01
**PACSIN1**	-1.61	3.25E-01	1.00E+00	1.36	2.28E-01	7.76E-01
**CPN2**	-3.28	9.45E-02	1.00E+00	1.48	2.28E-01	7.76E-01
**SEMA3A**	0.41	2.45E-01	1.00E+00	0.67	2.28E-01	7.76E-01
**SOX8**	0.18	7.70E-01	1.00E+00	-0.94	2.28E-01	7.76E-01
**NPM1**	-0.10	5.15E-01	1.00E+00	0.21	2.28E-01	7.76E-01
**CUBN**	0.41	2.73E-01	1.00E+00	0.76	2.28E-01	7.76E-01
**MAB21L1**	0.36	6.91E-01	1.00E+00	1.11	2.28E-01	7.76E-01
**YPEL4**	-0.11	8.46E-01	1.00E+00	0.66	2.28E-01	7.76E-01
**CLEC9A**	-0.06	9.16E-01	1.00E+00	1.01	2.28E-01	7.76E-01
**CSNK1D**	0.13	5.23E-01	1.00E+00	0.31	2.28E-01	7.76E-01
**COQ3**	-0.07	7.22E-01	1.00E+00	-0.40	2.28E-01	7.76E-01
**AC092919.2**	-2.63	9.74E-03	6.12E-01	1.16	2.28E-01	7.76E-01

**AC023830.3**	2.48	9.77E-02	1.00E+00	-3.17	2.28E-01	7.76E-01
**HMCN2**	-0.14	8.31E-01	1.00E+00	0.82	2.28E-01	7.76E-01
**PANTR1**	0.34	7.05E-01	1.00E+00	1.00	2.28E-01	7.76E-01
**DUTP6**	0.10	9.26E-01	1.00E+00	1.04	2.28E-01	7.76E-01
**PQLC3**	0.25	2.55E-01	1.00E+00	-0.48	2.28E-01	7.76E-01
**ITGA3**	-0.45	4.54E-02	1.00E+00	0.69	2.28E-01	7.76E-01
**SRD5A3**	-0.27	3.46E-01	1.00E+00	-0.36	2.28E-01	7.76E-01
**KCNQ1**	-0.03	9.48E-01	1.00E+00	1.01	2.28E-01	7.76E-01
**TRAT1**	-1.43	1.79E-01	1.00E+00	1.09	2.28E-01	7.76E-01
**SULT1B1**	1.68	2.73E-01	1.00E+00	1.52	2.28E-01	7.76E-01
**GAS2L3**	0.15	5.29E-01	1.00E+00	0.43	2.28E-01	7.76E-01
**AC097461.1**	0.16	7.19E-01	1.00E+00	-0.66	2.28E-01	7.76E-01
**MIR4482-1**	2.57	2.75E-01	1.00E+00	0.99	2.28E-01	7.76E-01
**NLRC3**	0.52	2.16E-01	1.00E+00	0.97	2.29E-01	7.76E-01
**DENND1A**	-0.35	2.08E-01	1.00E+00	0.69	2.29E-01	7.76E-01
**RBCK1**	-0.25	4.37E-01	1.00E+00	0.47	2.29E-01	7.76E-01
**LINC01549**	-2.82	4.11E-02	1.00E+00	1.20	2.29E-01	7.76E-01
**MCTS1**	-0.04	8.76E-01	1.00E+00	-0.28	2.29E-01	7.77E-01
**SPATS2L**	-0.01	9.86E-01	1.00E+00	0.34	2.29E-01	7.77E-01
**G7528**	0.29	8.50E-01	1.00E+00	1.37	2.29E-01	7.77E-01
**MED29**	-0.07	6.69E-01	1.00E+00	0.16	2.29E-01	7.77E-01
**SLC5A10**	0.00	9.99E-01	1.00E+00	0.94	2.29E-01	7.77E-01
**C2orf66**	-0.02	9.95E-01	1.00E+00	-2.18	2.29E-01	7.77E-01
**AC026369.3**	NA	NA	NA	2.40	2.29E-01	7.77E-01
**FAM3C**	-0.04	8.48E-01	1.00E+00	-0.53	2.29E-01	7.77E-01
**YPEL3**	-0.01	9.70E-01	1.00E+00	-0.73	2.29E-01	7.77E-01
**AC025594.2**	-2.68	1.18E-01	1.00E+00	1.86	2.29E-01	7.77E-01
**NETO1**	1.92	5.58E-02	1.00E+00	1.52	2.29E-01	7.77E-01
**G5134**	0.00	1.00E+00	1.00E+00	1.41	2.29E-01	7.77E-01
**CENPO**	-0.08	8.12E-01	1.00E+00	0.57	2.29E-01	7.77E-01
**RUFY1**	-0.07	6.85E-01	1.00E+00	-0.31	2.29E-01	7.77E-01
**ITPKB-IT1**	-2.98	1.32E-01	1.00E+00	-1.55	2.29E-01	7.77E-01

**RASGEF1A**	-0.09	8.52E-01	1.00E+00	0.87	2.29E-01	7.77E-01
**IGHV1-24**	1.46	6.70E-01	1.00E+00	3.80	2.29E-01	7.77E-01
**SUDS3**	0.32	2.00E-01	1.00E+00	-0.24	2.29E-01	7.77E-01
**BACE1**	-0.19	4.21E-01	1.00E+00	0.55	2.29E-01	7.77E-01
**SCGB1B2P**	-1.22	4.93E-01	1.00E+00	-3.76	2.29E-01	7.77E-01
**ALPK3**	0.23	7.68E-01	1.00E+00	1.14	2.30E-01	7.77E-01
**AC104843.1**	0.10	9.77E-01	1.00E+00	-0.82	2.30E-01	7.77E-01
**ZNF197**	0.00	9.96E-01	1.00E+00	0.43	2.30E-01	7.78E-01
**MSNP1**	0.43	9.03E-01	1.00E+00	0.78	2.30E-01	7.78E-01
**VGF**	-2.82	2.03E-04	4.65E-02	1.68	2.30E-01	7.78E-01
**HS3ST6**	-0.12	8.09E-01	1.00E+00	-0.86	2.30E-01	7.78E-01
**ZNF611**	0.05	8.31E-01	1.00E+00	-0.26	2.30E-01	7.78E-01
**SCYL3**	0.26	2.32E-01	1.00E+00	-0.23	2.30E-01	7.78E-01
**TRBV5-6**	-1.52	3.87E-01	1.00E+00	1.51	2.30E-01	7.78E-01
**OLFM4**	-0.17	8.77E-01	1.00E+00	1.64	2.30E-01	7.78E-01
**AC008622.2**	0.33	7.86E-01	1.00E+00	-0.86	2.30E-01	7.78E-01
**AC092653.2**	1.22	6.01E-01	1.00E+00	-1.03	2.30E-01	7.79E-01
**DCX**	0.09	9.38E-01	1.00E+00	1.17	2.30E-01	7.79E-01
**RIPPLY2**	-1.37	5.37E-01	1.00E+00	1.86	2.30E-01	7.79E-01
**SFT2D1**	0.49	2.44E-02	9.22E-01	-0.37	2.31E-01	7.79E-01
**UBE4A**	0.00	9.91E-01	1.00E+00	-0.20	2.31E-01	7.79E-01
**SIK2**	0.29	4.67E-01	1.00E+00	0.48	2.31E-01	7.79E-01
**ANKRD16**	-0.20	4.34E-01	1.00E+00	-0.65	2.31E-01	7.79E-01
**AC022415.1**	0.26	7.96E-01	1.00E+00	-0.76	2.31E-01	7.79E-01
**NPIPB3**	-0.30	7.37E-01	1.00E+00	0.95	2.31E-01	7.79E-01
**RNF113A**	-0.02	9.09E-01	1.00E+00	0.19	2.31E-01	7.79E-01
**ADM**	-0.63	1.39E-01	1.00E+00	0.56	2.31E-01	7.79E-01
**FAM189A1**	-0.65	5.14E-01	1.00E+00	-1.36	2.31E-01	7.79E-01
**INO80**	-0.11	5.38E-01	1.00E+00	0.48	2.31E-01	7.79E-01
**DMTN**	0.09	6.78E-01	1.00E+00	-0.46	2.31E-01	7.79E-01
**TMEM258**	-0.15	4.30E-01	1.00E+00	-0.52	2.31E-01	7.79E-01
**G39634**	-0.12	9.31E-01	1.00E+00	1.33	2.31E-01	7.79E-01

**SLC40A1**	0.53	2.37E-01	1.00E+00	-0.41	2.31E-01	7.79E-01
**PDE4A**	0.01	9.74E-01	1.00E+00	0.55	2.31E-01	7.79E-01
**PIDD1**	0.00	9.99E-01	1.00E+00	0.74	2.31E-01	7.79E-01
**XLOC_003463**	-1.92	5.78E-01	1.00E+00	-1.72	2.31E-01	7.79E-01
**ZNF445**	0.27	3.92E-01	1.00E+00	0.62	2.31E-01	7.79E-01
**RNF138**	0.02	9.38E-01	1.00E+00	-0.31	2.31E-01	7.79E-01
**RNF145**	0.10	6.69E-01	1.00E+00	0.51	2.31E-01	7.79E-01
**PHF20**	-0.06	7.41E-01	1.00E+00	0.18	2.31E-01	7.79E-01
**ZFP92**	0.31	6.82E-01	1.00E+00	1.16	2.31E-01	7.79E-01
**RNU1-59P**	-0.86	4.35E-01	1.00E+00	-0.88	2.31E-01	7.79E-01
**TSN**	0.02	8.84E-01	1.00E+00	-0.25	2.31E-01	7.79E-01
**NXPE3**	0.26	4.22E-01	1.00E+00	0.62	2.31E-01	7.79E-01
**LMF1-AS1**	0.42	7.06E-01	1.00E+00	1.19	2.31E-01	7.79E-01
**MED25**	-0.13	6.40E-01	1.00E+00	0.64	2.31E-01	7.79E-01
**9-Sep**	-0.28	1.74E-01	1.00E+00	0.68	2.32E-01	7.79E-01
**ARGFXP2**	0.67	8.44E-01	1.00E+00	2.08	2.32E-01	7.79E-01
**AC024575.1**	2.23	3.37E-01	1.00E+00	1.02	2.32E-01	7.79E-01
**PDCD5P1**	-0.58	8.67E-01	1.00E+00	-0.94	2.32E-01	7.79E-01
**BX088651.4**	-0.77	4.33E-01	1.00E+00	-0.73	2.32E-01	7.79E-01
**CLPTM1L**	0.11	5.33E-01	1.00E+00	-0.57	2.32E-01	7.79E-01
**DMGDH**	0.68	2.04E-01	1.00E+00	-0.80	2.32E-01	7.79E-01
**C16orf71**	-0.42	6.33E-01	1.00E+00	-1.21	2.32E-01	7.79E-01
**CPT1C**	-0.78	1.46E-02	7.60E-01	0.52	2.32E-01	7.79E-01
**DIAPH2-AS1**	-1.16	3.60E-01	1.00E+00	1.01	2.32E-01	7.79E-01
**AC025166.1**	-0.43	6.77E-01	1.00E+00	-0.97	2.32E-01	7.79E-01
**CASP1**	-0.17	6.04E-01	1.00E+00	0.31	2.32E-01	7.79E-01
**AC107067.1**	-0.07	9.59E-01	1.00E+00	1.17	2.32E-01	7.79E-01
**EIF4EBP1**	-0.31	3.60E-01	1.00E+00	0.39	2.32E-01	7.79E-01
**AC011416.3**	-2.54	1.76E-02	8.33E-01	-1.00	2.32E-01	7.79E-01
**AC127070.4**	0.75	7.33E-01	1.00E+00	-1.17	2.32E-01	7.79E-01
**TARS2**	-0.01	9.55E-01	1.00E+00	0.17	2.32E-01	7.79E-01
**PA2G4P6**	-1.52	6.60E-01	1.00E+00	0.96	2.32E-01	7.79E-01

**RPS6KA5**	-0.02	9.46E-01	1.00E+00	-0.34	2.32E-01	7.79E-01
**ADM2**	-0.01	9.92E-01	1.00E+00	1.22	2.32E-01	7.79E-01
**KLHL10**	-0.14	9.31E-01	1.00E+00	-1.41	2.32E-01	7.79E-01
**ARID5B**	-0.08	7.53E-01	1.00E+00	0.30	2.32E-01	7.79E-01
**ZNF568**	0.37	1.97E-01	1.00E+00	-0.37	2.32E-01	7.79E-01
**SKA1**	-0.15	7.06E-01	1.00E+00	0.69	2.32E-01	7.79E-01
**PAXX**	-0.25	4.54E-01	1.00E+00	0.28	2.32E-01	7.79E-01
**PHBP12**	-1.93	5.74E-01	1.00E+00	-1.38	2.32E-01	7.79E-01
**GLYATL1P4**	-1.47	3.14E-01	1.00E+00	-1.74	2.32E-01	7.79E-01
**BX005266.2**	0.35	7.07E-01	1.00E+00	-0.76	2.32E-01	7.79E-01
**PET100**	0.47	6.23E-01	1.00E+00	-0.70	2.32E-01	7.79E-01
**SF3B1**	0.01	9.77E-01	1.00E+00	0.14	2.32E-01	7.79E-01
**COL10A1**	0.33	7.34E-01	1.00E+00	1.04	2.32E-01	7.79E-01
**KYAT3**	-0.20	4.99E-01	1.00E+00	-0.28	2.32E-01	7.79E-01
**TUG1**	0.00	9.97E-01	1.00E+00	0.26	2.32E-01	7.79E-01
**TMEM54**	0.06	8.67E-01	1.00E+00	-0.70	2.32E-01	7.79E-01
**LRFN3**	-0.09	7.13E-01	1.00E+00	0.96	2.33E-01	7.79E-01
**TBL1X**	0.51	4.50E-02	1.00E+00	0.32	2.33E-01	7.79E-01
**TRPV6**	-0.16	6.36E-01	1.00E+00	-0.68	2.33E-01	7.79E-01
**AL590999.1**	1.18	3.85E-01	1.00E+00	1.46	2.33E-01	7.79E-01
**TRIP12**	0.06	6.81E-01	1.00E+00	0.41	2.33E-01	7.79E-01
**FSTL5**	-1.09	2.90E-01	1.00E+00	1.58	2.33E-01	7.79E-01
**RASGRP3**	0.22	7.12E-01	1.00E+00	0.56	2.33E-01	7.79E-01
**AC117402.1**	-2.88	8.48E-02	1.00E+00	-1.88	2.33E-01	7.80E-01
**LIAS**	-0.11	6.79E-01	1.00E+00	-0.33	2.33E-01	7.80E-01
**ZC3H4**	-0.09	7.22E-01	1.00E+00	0.63	2.33E-01	7.80E-01
**CTBP1**	-0.19	2.34E-01	1.00E+00	-0.26	2.33E-01	7.80E-01
**PEX26**	0.10	6.26E-01	1.00E+00	0.65	2.33E-01	7.80E-01
**C12orf4**	0.15	5.09E-01	1.00E+00	0.27	2.33E-01	7.80E-01
**GABRB3**	0.10	8.70E-01	1.00E+00	-0.78	2.33E-01	7.80E-01
**NAP1L1**	-0.04	8.37E-01	1.00E+00	0.21	2.33E-01	7.80E-01
**CUL5**	0.17	4.23E-01	1.00E+00	-0.27	2.33E-01	7.80E-01

**TSEN34**	-0.13	5.87E-01	1.00E+00	-0.55	2.33E-01	7.80E-01
**TMEM177**	0.16	6.81E-01	1.00E+00	-0.30	2.33E-01	7.80E-01
**ZDHHC15**	0.03	9.25E-01	1.00E+00	-0.47	2.33E-01	7.80E-01
**PGLYRP4**	-0.25	5.36E-01	1.00E+00	0.73	2.34E-01	7.80E-01
**EXTL3**	0.02	9.22E-01	1.00E+00	0.52	2.34E-01	7.80E-01
**STK19**	0.00	1.00E+00	1.00E+00	0.31	2.34E-01	7.81E-01
**MRPL35P2**	-3.57	2.42E-02	9.20E-01	1.21	2.34E-01	7.81E-01
**RELB**	-0.21	5.57E-01	1.00E+00	0.97	2.34E-01	7.81E-01
**AC016700.2**	0.04	9.87E-01	1.00E+00	-0.73	2.34E-01	7.81E-01
**AL355987.4**	1.08	2.98E-01	1.00E+00	-1.26	2.34E-01	7.81E-01
**AC011444.1**	0.76	5.20E-01	1.00E+00	-0.76	2.34E-01	7.81E-01
**FAM98B**	-0.21	1.64E-01	1.00E+00	0.49	2.34E-01	7.81E-01
**NEXN**	0.05	9.27E-01	1.00E+00	0.54	2.34E-01	7.81E-01
**AF127577.4**	0.99	3.37E-01	1.00E+00	-0.81	2.34E-01	7.81E-01
**PHF12**	-0.27	2.22E-01	1.00E+00	0.58	2.34E-01	7.81E-01
**FRMD6**	0.10	7.45E-01	1.00E+00	0.27	2.34E-01	7.81E-01
**AL445524.1**	0.27	4.97E-01	1.00E+00	-0.50	2.34E-01	7.81E-01
**ADCY1**	0.00	9.97E-01	1.00E+00	0.95	2.34E-01	7.81E-01
**G26051**	0.48	7.26E-01	1.00E+00	1.70	2.34E-01	7.81E-01
**ZHX3**	0.31	2.40E-01	1.00E+00	0.42	2.34E-01	7.81E-01
**UNC13D**	-0.38	3.40E-01	1.00E+00	0.93	2.34E-01	7.81E-01
**LINC00346**	0.40	4.78E-01	1.00E+00	1.27	2.34E-01	7.81E-01
**RBFOX1**	-0.41	8.55E-01	1.00E+00	1.10	2.34E-01	7.81E-01
**SLC9A9**	0.34	2.15E-01	1.00E+00	0.27	2.34E-01	7.81E-01
**RPL21P4**	NA	NA	NA	-0.89	2.34E-01	7.81E-01
**MAGED1**	-0.40	1.91E-01	1.00E+00	0.27	2.34E-01	7.81E-01
**PLB1**	-0.69	9.80E-02	1.00E+00	0.38	2.34E-01	7.81E-01
**TFAM**	0.12	6.33E-01	1.00E+00	-0.21	2.34E-01	7.81E-01
**KIF24**	-0.08	8.01E-01	1.00E+00	0.86	2.34E-01	7.81E-01
**TMEM47**	0.31	5.42E-01	1.00E+00	-0.66	2.34E-01	7.81E-01
**USP12-AS1**	-1.55	6.51E-01	1.00E+00	1.03	2.34E-01	7.81E-01
**AP003025.1**	-1.20	6.88E-01	1.00E+00	1.57	2.35E-01	7.81E-01

**ZNF555**	-0.14	7.15E-01	1.00E+00	-0.49	2.35E-01	7.81E-01
**TLN2**	0.75	2.36E-01	1.00E+00	0.62	2.35E-01	7.81E-01
**ASPRV1**	0.15	8.18E-01	1.00E+00	0.75	2.35E-01	7.81E-01
**TMEM182**	0.22	5.23E-01	1.00E+00	-0.54	2.35E-01	7.81E-01
**PRKCZ**	-0.36	2.03E-01	1.00E+00	-0.76	2.35E-01	7.81E-01
**SPRR4**	-1.48	6.43E-01	1.00E+00	1.15	2.35E-01	7.81E-01
**DAND5**	1.09	5.40E-01	1.00E+00	-1.26	2.35E-01	7.81E-01
**CHD6**	-0.03	8.73E-01	1.00E+00	0.29	2.35E-01	7.81E-01
**GPR171**	0.29	7.25E-01	1.00E+00	0.81	2.35E-01	7.81E-01
**CENPU**	-0.71	2.02E-01	1.00E+00	-0.60	2.35E-01	7.81E-01
**HOXC12**	0.34	5.32E-01	1.00E+00	-0.63	2.35E-01	7.81E-01
**MAP2K4**	0.18	4.49E-01	1.00E+00	-0.43	2.35E-01	7.81E-01
**ZNF680**	0.13	7.63E-01	1.00E+00	-0.51	2.35E-01	7.81E-01
**CHFR**	-0.14	6.57E-01	1.00E+00	0.34	2.35E-01	7.81E-01
**ADHFE1**	1.44	1.95E-01	1.00E+00	-0.92	2.35E-01	7.81E-01
**DCAF7**	0.13	5.69E-01	1.00E+00	0.33	2.35E-01	7.81E-01
**SNUPN**	0.00	9.95E-01	1.00E+00	-0.35	2.35E-01	7.81E-01
**DMRTA1**	-0.07	9.55E-01	1.00E+00	0.85	2.35E-01	7.81E-01
**CDX1**	1.37	1.33E-01	1.00E+00	1.24	2.35E-01	7.81E-01
**NBPF15**	-0.41	2.29E-01	1.00E+00	0.35	2.35E-01	7.81E-01
**N4BP2L2**	0.02	9.36E-01	1.00E+00	-0.30	2.35E-01	7.81E-01
**FURIN**	-0.29	1.62E-01	1.00E+00	0.77	2.35E-01	7.81E-01
**LRRC3-DT**	0.58	7.35E-01	1.00E+00	-1.58	2.35E-01	7.81E-01
**SORD2P**	-2.66	8.68E-02	1.00E+00	0.90	2.36E-01	7.82E-01
**TSPAN31**	0.16	4.23E-01	1.00E+00	-0.29	2.36E-01	7.82E-01
**LIN9**	0.10	7.73E-01	1.00E+00	-0.49	2.36E-01	7.82E-01
**ERCC1**	-0.29	2.39E-01	1.00E+00	0.19	2.36E-01	7.82E-01
**CDKL1**	0.70	1.01E-01	1.00E+00	0.51	2.36E-01	7.82E-01
**AL591468.1**	NA	NA	NA	2.21	2.36E-01	7.82E-01
**XPOT**	0.11	6.53E-01	1.00E+00	-0.25	2.36E-01	7.82E-01
**AL390728.4**	-0.04	9.44E-01	1.00E+00	0.40	2.36E-01	7.82E-01
**REM1**	0.18	7.55E-01	1.00E+00	0.65	2.36E-01	7.82E-01

**LINC01465**	-1.14	4.55E-02	1.00E+00	0.62	2.36E-01	7.82E-01
**TMEM88**	1.49	6.44E-02	1.00E+00	-0.81	2.36E-01	7.82E-01
**LIF**	-0.12	6.83E-01	1.00E+00	0.87	2.36E-01	7.82E-01
**BACH1-AS1**	-0.78	7.87E-01	1.00E+00	-1.39	2.36E-01	7.82E-01
**AL359541.1**	0.60	7.19E-01	1.00E+00	1.24	2.36E-01	7.82E-01
**C8orf34-AS1**	-3.35	2.92E-02	9.89E-01	1.27	2.36E-01	7.82E-01
**CALD1**	0.00	9.94E-01	1.00E+00	0.39	2.36E-01	7.82E-01
**XRCC3**	-0.06	8.74E-01	1.00E+00	0.51	2.36E-01	7.82E-01
**FOXD2-AS1**	0.02	9.83E-01	1.00E+00	1.30	2.36E-01	7.83E-01
**FAM129C**	-0.40	7.57E-01	1.00E+00	1.69	2.36E-01	7.83E-01
**TMEM95**	-0.57	6.93E-01	1.00E+00	1.67	2.36E-01	7.83E-01
**HSPD1P4**	1.33	5.65E-01	1.00E+00	-0.70	2.36E-01	7.83E-01
**NUTM2B**	0.18	7.71E-01	1.00E+00	1.61	2.37E-01	7.83E-01
**LINC01473**	-1.31	1.75E-01	1.00E+00	-0.88	2.37E-01	7.83E-01
**AL449106.1**	-0.20	9.07E-01	1.00E+00	2.37	2.37E-01	7.83E-01
**LINC00303**	0.09	9.79E-01	1.00E+00	1.87	2.37E-01	7.83E-01
**AC004771.3**	-2.49	2.50E-02	9.28E-01	0.96	2.37E-01	7.83E-01
**AC068700.1**	0.28	6.95E-01	1.00E+00	-1.20	2.37E-01	7.83E-01
**AC005725.1**	0.39	7.92E-01	1.00E+00	1.62	2.37E-01	7.83E-01
**BAG4**	0.07	7.62E-01	1.00E+00	-0.43	2.37E-01	7.83E-01
**AP001189.5**	-1.17	4.08E-01	1.00E+00	-1.20	2.37E-01	7.83E-01
**AC074011.1**	-1.51	4.58E-01	1.00E+00	-1.13	2.37E-01	7.83E-01
**XLOC_013283**	-1.14	2.71E-01	1.00E+00	1.07	2.37E-01	7.83E-01
**AC009159.3**	-1.84	5.55E-01	1.00E+00	0.93	2.37E-01	7.83E-01
**G8753**	-0.42	7.51E-01	1.00E+00	-0.92	2.37E-01	7.83E-01
**CCNC**	0.08	7.78E-01	1.00E+00	-0.44	2.37E-01	7.83E-01
**LDOC1**	-0.31	2.50E-01	1.00E+00	-0.30	2.37E-01	7.84E-01
**MYO3A**	-0.49	3.44E-01	1.00E+00	-1.09	2.37E-01	7.84E-01
**AC063952.1**	0.13	9.71E-01	1.00E+00	1.15	2.37E-01	7.84E-01
**GPR37**	-0.04	8.90E-01	1.00E+00	-0.68	2.37E-01	7.84E-01
**UCP3**	-0.26	6.71E-01	1.00E+00	1.50	2.37E-01	7.84E-01
**G10910**	0.15	8.92E-01	1.00E+00	1.28	2.38E-01	7.84E-01

**CAT**	0.28	3.91E-01	1.00E+00	-0.41	2.38E-01	7.84E-01
**AC093249.6**	-0.47	4.77E-01	1.00E+00	-0.70	2.38E-01	7.84E-01
**AL451123.1**	-0.03	9.59E-01	1.00E+00	0.73	2.38E-01	7.84E-01
**AC018638.4**	-0.87	6.64E-02	1.00E+00	1.42	2.38E-01	7.84E-01
**AC119428.2**	-2.14	2.26E-01	1.00E+00	1.09	2.38E-01	7.84E-01
**XLOC_011616**	-0.74	7.33E-01	1.00E+00	-1.66	2.38E-01	7.84E-01
**ZMYND15**	-0.25	6.06E-01	1.00E+00	1.10	2.38E-01	7.84E-01
**RGS9**	0.42	1.36E-01	1.00E+00	-0.37	2.38E-01	7.84E-01
**LZIC**	-0.16	4.53E-01	1.00E+00	-0.21	2.38E-01	7.84E-01
**AL928921.2**	0.75	1.67E-01	1.00E+00	-0.57	2.38E-01	7.84E-01
**GALNT10**	0.33	2.99E-01	1.00E+00	0.61	2.38E-01	7.84E-01
**TCTEX1D1**	0.25	7.27E-01	1.00E+00	1.04	2.38E-01	7.84E-01
**CDC42SE2**	0.09	5.89E-01	1.00E+00	0.22	2.38E-01	7.84E-01
**ADAT1**	-0.17	3.42E-01	1.00E+00	0.30	2.38E-01	7.84E-01
**UNC5C**	0.07	9.33E-01	1.00E+00	0.83	2.38E-01	7.84E-01
**PTGR1**	-0.08	7.73E-01	1.00E+00	-0.26	2.38E-01	7.84E-01
**ACTG1**	-0.28	9.18E-02	1.00E+00	0.22	2.38E-01	7.84E-01
**MYL12B**	0.00	9.80E-01	1.00E+00	-0.27	2.38E-01	7.84E-01
**ZNF260**	0.33	2.88E-01	1.00E+00	-0.33	2.38E-01	7.84E-01
**ZNF124**	0.07	8.55E-01	1.00E+00	0.45	2.38E-01	7.84E-01
**AC007229.1**	NA	NA	NA	-0.92	2.38E-01	7.84E-01
**L3MBTL2**	-0.15	4.89E-01	1.00E+00	0.34	2.38E-01	7.84E-01
**C2CD4A**	-0.39	6.81E-01	1.00E+00	-1.22	2.38E-01	7.84E-01
**PPP1R2**	0.15	4.03E-01	1.00E+00	-0.23	2.38E-01	7.84E-01
**HIST2H2AA4**	-1.40	2.70E-01	1.00E+00	-0.58	2.38E-01	7.84E-01
**SUZ12P1**	0.08	8.42E-01	1.00E+00	-0.45	2.39E-01	7.85E-01
**TMEM106A**	0.61	1.69E-01	1.00E+00	0.88	2.39E-01	7.85E-01
**CDK5RAP1**	0.26	1.45E-01	1.00E+00	0.21	2.39E-01	7.85E-01
**WIPF2**	-0.10	6.51E-01	1.00E+00	0.28	2.39E-01	7.85E-01
**TNFAIP1**	-0.17	3.42E-01	1.00E+00	0.25	2.39E-01	7.85E-01
**IGHV1-58**	1.59	6.41E-01	1.00E+00	2.46	2.39E-01	7.85E-01
**MT-CO2**	-0.11	7.68E-01	1.00E+00	-0.42	2.39E-01	7.85E-01

**C6orf52**	-1.39	1.94E-02	8.74E-01	-0.58	2.39E-01	7.85E-01
**KIF18A**	0.31	4.22E-01	1.00E+00	-0.71	2.39E-01	7.85E-01
**NOD1**	-0.17	4.44E-01	1.00E+00	0.67	2.39E-01	7.85E-01
**XLOC_003930**	-0.20	8.59E-01	1.00E+00	1.08	2.39E-01	7.85E-01
**RAC3**	-0.15	7.00E-01	1.00E+00	0.73	2.39E-01	7.85E-01
**G32202**	-0.50	5.63E-01	1.00E+00	1.16	2.39E-01	7.86E-01
**CLCA2**	-0.47	2.62E-01	1.00E+00	0.56	2.39E-01	7.86E-01
**MIR4477B**	0.72	5.15E-01	1.00E+00	1.14	2.39E-01	7.86E-01
**RAB13**	0.24	3.52E-01	1.00E+00	0.27	2.39E-01	7.86E-01
**PRRC2B**	-0.08	7.22E-01	1.00E+00	0.72	2.39E-01	7.86E-01
**AC005696.1**	-0.44	6.72E-01	1.00E+00	-0.75	2.39E-01	7.86E-01
**XLOC_005574**	-0.22	7.95E-01	1.00E+00	-0.97	2.40E-01	7.86E-01
**G15373**	-1.60	2.67E-01	1.00E+00	-0.76	2.40E-01	7.86E-01
**G38557**	-3.71	3.63E-02	1.00E+00	-1.89	2.40E-01	7.86E-01
**CDH2**	-0.99	6.35E-02	1.00E+00	0.93	2.40E-01	7.86E-01
**SLC22A16**	0.75	6.96E-01	1.00E+00	1.27	2.40E-01	7.86E-01
**CRABP2**	-0.19	4.90E-01	1.00E+00	0.47	2.40E-01	7.86E-01
**AC007036.1**	-0.60	8.59E-01	1.00E+00	1.29	2.40E-01	7.86E-01
**IGLJ3**	NA	NA	NA	2.23	2.40E-01	7.86E-01
**VPS26A**	0.02	9.30E-01	1.00E+00	-0.43	2.40E-01	7.86E-01
**FASTKD3**	-0.01	9.69E-01	1.00E+00	-0.39	2.40E-01	7.86E-01
**CAMSAP1**	-0.28	3.17E-01	1.00E+00	0.51	2.40E-01	7.86E-01
**SETD4**	0.30	2.42E-01	1.00E+00	-0.30	2.40E-01	7.86E-01
**OSCP1**	-0.13	6.71E-01	1.00E+00	-0.39	2.40E-01	7.86E-01
**DCLK1**	0.65	2.02E-01	1.00E+00	0.50	2.40E-01	7.86E-01
**TRAPPC6B**	-0.15	6.27E-01	1.00E+00	0.30	2.40E-01	7.86E-01
**CD8B**	0.56	1.12E-01	1.00E+00	0.89	2.40E-01	7.86E-01
**AL807752.5**	-1.29	3.41E-01	1.00E+00	1.76	2.40E-01	7.86E-01
**XLOC_000303**	-1.28	1.79E-01	1.00E+00	-1.29	2.40E-01	7.86E-01
**TMEM106B**	0.05	8.47E-01	1.00E+00	-0.31	2.40E-01	7.86E-01
**LCE2A**	0.86	2.07E-01	1.00E+00	0.82	2.40E-01	7.86E-01
**HTR7P1**	0.23	4.17E-01	1.00E+00	0.62	2.40E-01	7.86E-01

**AC090246.1**	1.13	5.64E-01	1.00E+00	1.48	2.40E-01	7.86E-01
**PLEKHB2**	0.10	6.50E-01	1.00E+00	0.18	2.40E-01	7.86E-01
**ERMAP**	0.13	5.95E-01	1.00E+00	0.29	2.40E-01	7.86E-01
**TLE1**	-0.03	9.14E-01	1.00E+00	-0.61	2.40E-01	7.86E-01
**CCDC9B**	0.27	4.35E-01	1.00E+00	0.49	2.41E-01	7.86E-01
**H2AFJ**	0.25	3.55E-01	1.00E+00	0.27	2.41E-01	7.86E-01
**ACTL6A**	-0.13	5.21E-01	1.00E+00	-0.30	2.41E-01	7.86E-01
**ADAMTS18**	-0.54	6.83E-01	1.00E+00	1.40	2.41E-01	7.86E-01
**TRG-AS1**	-0.22	8.44E-01	1.00E+00	1.23	2.41E-01	7.86E-01
**DNAJC21**	0.13	5.31E-01	1.00E+00	-0.33	2.41E-01	7.87E-01
**AL162151.2**	-1.04	3.94E-01	1.00E+00	-0.71	2.41E-01	7.87E-01
**GMEB1**	0.06	7.18E-01	1.00E+00	0.30	2.41E-01	7.87E-01
**TMSB4X**	0.20	4.58E-01	1.00E+00	-0.40	2.41E-01	7.87E-01
**NDUFA13**	0.20	8.69E-01	1.00E+00	-0.68	2.41E-01	7.87E-01
**FAM160B1**	0.05	8.89E-01	1.00E+00	-0.44	2.41E-01	7.87E-01
**LSM3**	0.05	8.19E-01	1.00E+00	-0.48	2.41E-01	7.87E-01
**ENTPD4**	0.06	8.55E-01	1.00E+00	0.40	2.41E-01	7.87E-01
**LRP4-AS1**	0.97	4.48E-01	1.00E+00	-1.17	2.41E-01	7.87E-01
**DPEP3**	0.90	7.91E-01	1.00E+00	1.82	2.41E-01	7.87E-01
**HMGB3**	-0.07	8.10E-01	1.00E+00	0.36	2.41E-01	7.87E-01
**STK36**	0.25	5.04E-01	1.00E+00	0.74	2.41E-01	7.87E-01
**RUNDC3A-AS1**	-0.56	5.51E-01	1.00E+00	-1.08	2.41E-01	7.87E-01
**NPTN-IT1**	1.55	3.49E-01	1.00E+00	-1.60	2.41E-01	7.87E-01
**ALG1L6P**	0.18	7.73E-01	1.00E+00	0.85	2.41E-01	7.87E-01
**TAS2R15P**	-2.72	2.50E-01	1.00E+00	-1.68	2.41E-01	7.87E-01
**NXPE2**	0.05	9.76E-01	1.00E+00	1.75	2.41E-01	7.87E-01
**XKRX**	0.32	6.02E-01	1.00E+00	0.75	2.42E-01	7.88E-01
**ATE1**	0.00	9.90E-01	1.00E+00	-0.25	2.42E-01	7.88E-01
**TRPS1**	-0.73	4.49E-02	1.00E+00	0.35	2.42E-01	7.88E-01
**ZNF331**	0.16	4.27E-01	1.00E+00	0.32	2.42E-01	7.88E-01
**TEX264**	0.12	6.56E-01	1.00E+00	0.21	2.42E-01	7.88E-01
**PDE3A**	-0.02	9.67E-01	1.00E+00	0.72	2.42E-01	7.88E-01

**FMN1**	-0.29	4.32E-01	1.00E+00	0.49	2.42E-01	7.88E-01
**ZNF587B**	0.19	6.17E-01	1.00E+00	-0.35	2.42E-01	7.88E-01
**IMPA1**	0.08	8.05E-01	1.00E+00	-0.31	2.42E-01	7.88E-01
**ZNF225**	0.21	5.86E-01	1.00E+00	-0.54	2.42E-01	7.88E-01
**TIGAR**	-0.37	5.61E-01	1.00E+00	0.42	2.42E-01	7.88E-01
**IKZF4**	0.27	4.31E-01	1.00E+00	0.55	2.42E-01	7.88E-01
**NCMAP**	-0.18	7.95E-01	1.00E+00	1.03	2.42E-01	7.88E-01
**MALL**	-0.26	5.25E-01	1.00E+00	0.28	2.42E-01	7.88E-01
**GOLGA8VP**	-0.53	8.77E-01	1.00E+00	-2.10	2.42E-01	7.88E-01
**UACA**	0.04	8.85E-01	1.00E+00	-0.25	2.42E-01	7.88E-01
**ERI3**	-0.23	2.10E-01	1.00E+00	-0.21	2.42E-01	7.88E-01
**LINC00324**	0.06	8.17E-01	1.00E+00	-0.43	2.42E-01	7.88E-01
**OR10A6**	0.90	6.37E-01	1.00E+00	-1.36	2.42E-01	7.88E-01
**LRRN3**	1.04	2.39E-01	1.00E+00	-0.82	2.42E-01	7.88E-01
**AC010186.3**	0.66	3.71E-01	1.00E+00	0.94	2.42E-01	7.88E-01
**G12578**	0.25	6.51E-01	1.00E+00	-1.05	2.42E-01	7.88E-01
**ZNF654**	-0.02	9.53E-01	1.00E+00	-0.33	2.42E-01	7.88E-01
**AC007238.1**	0.02	9.83E-01	1.00E+00	-0.55	2.42E-01	7.88E-01
**HEXB**	0.21	3.16E-01	1.00E+00	0.29	2.42E-01	7.88E-01
**MPRIPP1**	-1.41	1.84E-01	1.00E+00	1.09	2.43E-01	7.88E-01
**LDHB**	0.74	4.58E-01	1.00E+00	-0.84	2.43E-01	7.88E-01
**REPS1**	-0.01	9.61E-01	1.00E+00	-0.47	2.43E-01	7.88E-01
**G7666**	0.33	7.29E-01	1.00E+00	0.83	2.43E-01	7.88E-01
**PPP1R15B**	-0.03	9.34E-01	1.00E+00	0.26	2.43E-01	7.88E-01
**NAXE**	-0.09	5.62E-01	1.00E+00	-0.31	2.43E-01	7.88E-01
**CXCL2**	-3.18	1.37E-02	7.37E-01	1.20	2.43E-01	7.88E-01
**TXNRD3**	0.02	9.51E-01	1.00E+00	-0.73	2.43E-01	7.88E-01
**BX890604.2**	-0.33	3.54E-01	1.00E+00	-0.56	2.43E-01	7.89E-01
**FAM182B**	-0.67	3.94E-01	1.00E+00	-0.80	2.43E-01	7.89E-01
**AP001885.1**	0.05	9.88E-01	1.00E+00	-2.16	2.43E-01	7.89E-01
**ZYX**	-0.39	1.55E-01	1.00E+00	0.67	2.43E-01	7.89E-01
**RAET1L**	0.04	9.50E-01	1.00E+00	0.79	2.43E-01	7.89E-01

**TTN**	0.95	4.59E-02	1.00E+00	0.41	2.43E-01	7.89E-01
**USP32**	0.05	8.82E-01	1.00E+00	0.56	2.43E-01	7.89E-01
**ATF4**	-0.15	5.89E-01	1.00E+00	0.52	2.43E-01	7.89E-01
**SLBP**	-0.13	5.79E-01	1.00E+00	-0.44	2.43E-01	7.89E-01
**AC106827.1**	-0.67	8.47E-01	1.00E+00	-1.60	2.43E-01	7.89E-01
**PKIG**	0.05	8.70E-01	1.00E+00	0.35	2.43E-01	7.89E-01
**PDE10A**	-0.51	3.74E-01	1.00E+00	1.12	2.43E-01	7.89E-01
**COX7A2**	-0.02	8.85E-01	1.00E+00	-0.37	2.43E-01	7.89E-01
**ASAH1**	0.41	7.51E-02	1.00E+00	-0.46	2.43E-01	7.89E-01
**CCDC183**	-0.33	5.04E-01	1.00E+00	0.63	2.43E-01	7.89E-01
**GSTK1**	-0.14	4.52E-01	1.00E+00	-0.14	2.44E-01	7.89E-01
**ADGRF2**	0.29	5.17E-01	1.00E+00	0.80	2.44E-01	7.89E-01
**HS3ST4**	-0.65	4.99E-01	1.00E+00	-1.39	2.44E-01	7.89E-01
**MYO1E**	-0.27	2.84E-01	1.00E+00	0.44	2.44E-01	7.89E-01
**XLOC_012841**	-0.05	9.58E-01	1.00E+00	-1.23	2.44E-01	7.89E-01
**LINC01725**	-0.50	2.72E-01	1.00E+00	0.70	2.44E-01	7.89E-01
**OSBPL3**	-0.06	8.64E-01	1.00E+00	0.37	2.44E-01	7.90E-01
**XLOC_010811**	-2.23	2.34E-01	1.00E+00	-1.15	2.44E-01	7.90E-01
**FOXK2**	-0.18	4.94E-01	1.00E+00	0.54	2.44E-01	7.90E-01
**CFAP58**	-1.76	2.01E-02	8.84E-01	-1.17	2.44E-01	7.90E-01
**TULP2**	0.52	5.40E-01	1.00E+00	1.21	2.44E-01	7.90E-01
**EIF1P7**	0.66	8.49E-01	1.00E+00	-0.98	2.44E-01	7.90E-01
**MLLT11**	-0.86	1.05E-02	6.35E-01	0.40	2.44E-01	7.90E-01
**AL139246.3**	-1.29	5.01E-01	1.00E+00	-1.72	2.44E-01	7.90E-01
**HPS4**	0.14	5.56E-01	1.00E+00	0.22	2.44E-01	7.90E-01
**SLC9A1**	-0.22	4.79E-01	1.00E+00	0.66	2.44E-01	7.90E-01
**WDR43**	-0.23	3.70E-01	1.00E+00	0.24	2.44E-01	7.90E-01
**GIMAP1**	0.33	5.11E-01	1.00E+00	0.60	2.44E-01	7.90E-01
**C4orf33**	0.25	3.73E-01	1.00E+00	0.32	2.44E-01	7.90E-01
**YWHAZP2**	0.43	7.93E-01	1.00E+00	-0.74	2.45E-01	7.90E-01
**MME**	2.60	6.35E-02	1.00E+00	1.00	2.45E-01	7.90E-01
**CPQ**	0.54	1.36E-01	1.00E+00	0.36	2.45E-01	7.90E-01

**WWTR1**	0.14	6.33E-01	1.00E+00	-0.30	2.45E-01	7.90E-01
**AL353625.1**	0.72	2.29E-01	1.00E+00	1.17	2.45E-01	7.91E-01
**MANCR**	-0.15	9.28E-01	1.00E+00	0.93	2.45E-01	7.91E-01
**ANK1**	-1.30	5.01E-03	4.15E-01	0.94	2.45E-01	7.91E-01
**YES1P1**	-2.05	3.27E-01	1.00E+00	1.17	2.45E-01	7.91E-01
**RPLP0**	-0.20	5.69E-01	1.00E+00	0.34	2.45E-01	7.91E-01
**KIAA0895**	-0.42	3.45E-01	1.00E+00	-0.56	2.45E-01	7.91E-01
**KLF16**	-0.51	1.77E-01	1.00E+00	0.73	2.45E-01	7.91E-01
**CCT3**	-0.19	3.23E-01	1.00E+00	0.15	2.45E-01	7.91E-01
**HRAT5**	-1.54	1.56E-01	1.00E+00	-1.01	2.45E-01	7.91E-01
**TNPO2**	0.07	6.18E-01	1.00E+00	0.52	2.45E-01	7.91E-01
**AC141557.1**	-1.60	4.02E-02	1.00E+00	-0.54	2.45E-01	7.91E-01
**MED21**	0.25	3.03E-01	1.00E+00	-0.38	2.45E-01	7.91E-01
**ATXN3**	0.35	3.25E-01	1.00E+00	-0.29	2.45E-01	7.91E-01
**PPP6R2**	-0.23	1.52E-01	1.00E+00	0.35	2.46E-01	7.91E-01
**NSD1**	-0.18	4.47E-01	1.00E+00	0.49	2.46E-01	7.91E-01
**AL049840.5**	-1.03	7.15E-01	1.00E+00	-1.24	2.46E-01	7.91E-01
**ZNF525**	-0.22	5.80E-01	1.00E+00	-0.37	2.46E-01	7.91E-01
**RNF224**	0.15	9.01E-01	1.00E+00	1.20	2.46E-01	7.91E-01
**RWDD4P2**	0.02	9.61E-01	1.00E+00	-0.33	2.46E-01	7.91E-01
**SHMT2**	-0.20	1.97E-01	1.00E+00	0.26	2.46E-01	7.91E-01
**NDUFC2**	0.47	1.87E-01	1.00E+00	-0.53	2.46E-01	7.91E-01
**AC091729.3**	-0.17	7.56E-01	1.00E+00	-0.75	2.46E-01	7.91E-01
**AC127164.1**	0.33	8.86E-01	1.00E+00	1.37	2.46E-01	7.91E-01
**PRKRA**	-0.13	5.08E-01	1.00E+00	-0.46	2.46E-01	7.91E-01
**NAT8L**	2.12	7.64E-02	1.00E+00	0.99	2.46E-01	7.91E-01
**FAM3A**	0.02	9.44E-01	1.00E+00	0.30	2.46E-01	7.91E-01
**AC092162.3**	-0.08	9.38E-01	1.00E+00	-1.95	2.46E-01	7.91E-01
**RAB8A**	-0.01	9.56E-01	1.00E+00	0.32	2.46E-01	7.91E-01
**AC245140.3**	-2.40	2.83E-01	1.00E+00	-1.48	2.46E-01	7.91E-01
**LOX**	0.76	1.55E-01	1.00E+00	0.58	2.46E-01	7.91E-01
**MEIS3P2**	-0.96	1.10E-01	1.00E+00	1.19	2.46E-01	7.91E-01

**TMPRSS6**	0.35	4.42E-01	1.00E+00	-1.01	2.46E-01	7.91E-01
**AC067817.2**	0.63	6.16E-01	1.00E+00	1.16	2.46E-01	7.91E-01
**TRMT44**	-0.15	3.83E-01	1.00E+00	-0.56	2.46E-01	7.91E-01
**AL031666.1**	-0.92	4.96E-01	1.00E+00	-0.69	2.46E-01	7.92E-01
**COX20**	0.43	5.12E-01	1.00E+00	0.66	2.46E-01	7.92E-01
**HAUS6**	-0.15	5.64E-01	1.00E+00	-0.27	2.46E-01	7.92E-01
**NFAT5**	-0.19	4.12E-01	1.00E+00	-0.37	2.46E-01	7.92E-01
**AL606760.1**	0.10	9.39E-01	1.00E+00	0.79	2.46E-01	7.92E-01
**CEP112**	0.13	7.64E-01	1.00E+00	-0.45	2.46E-01	7.92E-01
**MRPL53**	1.00	2.99E-01	1.00E+00	0.81	2.46E-01	7.92E-01
**PTPN13**	0.08	8.40E-01	1.00E+00	-0.41	2.47E-01	7.92E-01
**FGF14-AS2**	-0.17	5.21E-01	1.00E+00	-0.52	2.47E-01	7.92E-01
**TRBV3-1**	-2.42	9.67E-02	1.00E+00	1.02	2.47E-01	7.92E-01
**G43247**	0.34	8.63E-01	1.00E+00	-0.78	2.47E-01	7.93E-01
**LVRN**	1.64	1.02E-02	6.27E-01	-0.92	2.47E-01	7.93E-01
**LGALS12**	4.12	3.05E-02	1.00E+00	-1.17	2.47E-01	7.93E-01
**AL590006.1**	0.56	8.71E-01	1.00E+00	1.15	2.47E-01	7.93E-01
**CMC2**	-0.06	8.16E-01	1.00E+00	-0.48	2.47E-01	7.93E-01
**AC092134.1**	2.17	6.61E-02	1.00E+00	-1.35	2.47E-01	7.93E-01
**P3H2**	0.60	4.64E-03	3.93E-01	0.52	2.47E-01	7.93E-01
**CYP2B7P**	0.84	5.20E-01	1.00E+00	-1.34	2.47E-01	7.93E-01
**SUB1**	0.16	5.66E-01	1.00E+00	-0.34	2.47E-01	7.93E-01
**MS4A4E**	-0.82	7.20E-01	1.00E+00	1.09	2.47E-01	7.93E-01
**AC092139.3**	-2.42	4.78E-01	1.00E+00	1.41	2.47E-01	7.93E-01
**USPL1**	-0.03	9.40E-01	1.00E+00	-0.38	2.47E-01	7.93E-01
**AL391244.3**	-0.39	5.74E-01	1.00E+00	1.10	2.47E-01	7.93E-01
**AC090579.1**	0.60	2.60E-01	1.00E+00	-0.63	2.47E-01	7.93E-01
**OPN4**	0.44	4.65E-01	1.00E+00	1.22	2.47E-01	7.93E-01
**RBM17**	0.06	7.29E-01	1.00E+00	-0.13	2.47E-01	7.93E-01
**TMED1**	-0.11	7.34E-01	1.00E+00	0.35	2.47E-01	7.93E-01
**VRK1**	-0.16	5.33E-01	1.00E+00	-0.39	2.47E-01	7.93E-01
**MYH11**	-0.21	7.60E-01	1.00E+00	-0.73	2.47E-01	7.93E-01

**AC084262.1**	0.64	7.51E-01	1.00E+00	-1.65	2.48E-01	7.93E-01
**MOGAT2**	-0.61	4.22E-01	1.00E+00	-0.95	2.48E-01	7.93E-01
**FCN2**	1.61	2.81E-01	1.00E+00	-2.05	2.48E-01	7.93E-01
**LYSMD1**	0.18	5.16E-01	1.00E+00	0.62	2.48E-01	7.93E-01
**MLST8**	-0.14	5.40E-01	1.00E+00	0.13	2.48E-01	7.93E-01
**ZNF438**	0.00	9.95E-01	1.00E+00	0.24	2.48E-01	7.93E-01
**MRPL47**	-0.24	2.75E-01	1.00E+00	-0.27	2.48E-01	7.93E-01
**LINC00707**	-2.55	2.20E-01	1.00E+00	1.43	2.48E-01	7.93E-01
**TRIL**	0.11	8.24E-01	1.00E+00	0.72	2.48E-01	7.93E-01
**IL18BP**	-0.54	1.93E-01	1.00E+00	0.70	2.48E-01	7.93E-01
**AC046185.3**	-1.03	6.80E-02	1.00E+00	1.08	2.48E-01	7.93E-01
**MAD2L1BP**	-0.01	9.77E-01	1.00E+00	0.20	2.48E-01	7.93E-01
**MDGA1**	-1.25	1.15E-01	1.00E+00	0.80	2.48E-01	7.93E-01
**L3MBTL4-AS1**	-1.19	3.60E-01	1.00E+00	1.02	2.48E-01	7.93E-01
**IRF2BP2**	-0.32	1.08E-01	1.00E+00	0.28	2.48E-01	7.93E-01
**PER3**	-0.79	2.62E-03	2.64E-01	-0.37	2.48E-01	7.93E-01
**SLC2A13**	-0.08	8.02E-01	1.00E+00	-0.64	2.48E-01	7.93E-01
**PDCD10**	0.11	6.60E-01	1.00E+00	-0.42	2.48E-01	7.93E-01
**IMPG1**	-0.82	6.54E-01	1.00E+00	-1.47	2.48E-01	7.93E-01
**MAL**	-0.44	2.64E-01	1.00E+00	-0.52	2.48E-01	7.93E-01
**AC007406.5**	0.43	2.71E-01	1.00E+00	-0.59	2.48E-01	7.93E-01
**HIPK3**	0.11	4.86E-01	1.00E+00	-0.26	2.48E-01	7.93E-01
**RIBC2**	-0.46	4.62E-01	1.00E+00	0.91	2.48E-01	7.93E-01
**PTMAP4**	-0.14	7.78E-01	1.00E+00	-0.43	2.48E-01	7.93E-01
**CCDC183-AS1**	-3.07	6.11E-03	4.63E-01	1.20	2.48E-01	7.93E-01
**FAM174B**	0.25	6.12E-01	1.00E+00	-0.56	2.48E-01	7.93E-01
**GCOM1**	0.32	6.01E-01	1.00E+00	0.38	2.48E-01	7.93E-01
**N4BP2L1**	0.18	3.92E-01	1.00E+00	-0.38	2.48E-01	7.93E-01
**SLC15A2**	0.53	4.91E-01	1.00E+00	0.92	2.49E-01	7.94E-01
**DHCR7**	-0.26	5.34E-01	1.00E+00	-0.38	2.49E-01	7.94E-01
**NPIPB4**	0.72	2.19E-01	1.00E+00	0.58	2.49E-01	7.94E-01
**AL122035.1**	-0.02	9.75E-01	1.00E+00	-0.58	2.49E-01	7.94E-01

**GPR146**	-0.31	4.08E-01	1.00E+00	-0.58	2.49E-01	7.94E-01
**MIRLET7BHG**	0.57	3.29E-01	1.00E+00	0.54	2.49E-01	7.94E-01
**XLOC_002951**	-1.09	4.14E-01	1.00E+00	1.40	2.49E-01	7.94E-01
**AC002467.1**	-0.42	5.39E-01	1.00E+00	-0.43	2.49E-01	7.94E-01
**ZNF37BP**	-0.13	7.18E-01	1.00E+00	-0.53	2.49E-01	7.94E-01
**AC009113.1**	0.27	3.13E-01	1.00E+00	-0.45	2.49E-01	7.94E-01
**COL18A1**	-0.66	6.16E-02	1.00E+00	1.00	2.49E-01	7.94E-01
**XLOC_003284**	-1.13	5.17E-01	1.00E+00	-1.55	2.49E-01	7.94E-01
**GTSE1-DT**	-2.32	1.13E-02	6.59E-01	-1.14	2.49E-01	7.94E-01
**ANKRD18EP**	-0.13	6.57E-01	1.00E+00	0.51	2.49E-01	7.94E-01
**MVP**	-0.21	4.49E-01	1.00E+00	0.50	2.49E-01	7.94E-01
**LINC02432**	-3.55	3.23E-02	1.00E+00	-1.43	2.49E-01	7.95E-01
**LEP**	5.30	7.44E-03	5.24E-01	-1.16	2.49E-01	7.95E-01
**MCRIP1**	0.00	9.90E-01	1.00E+00	0.42	2.49E-01	7.95E-01
**AC083862.2**	1.50	5.65E-01	1.00E+00	1.38	2.49E-01	7.95E-01
**MB**	-0.16	8.44E-01	1.00E+00	-0.70	2.50E-01	7.95E-01
**LAP3**	0.04	8.97E-01	1.00E+00	0.45	2.50E-01	7.95E-01
**ITGA7**	0.89	2.04E-01	1.00E+00	0.90	2.50E-01	7.95E-01
**HTR1B**	-2.46	4.60E-02	1.00E+00	2.03	2.50E-01	7.95E-01
**AC026470.2**	-1.59	2.50E-01	1.00E+00	0.83	2.50E-01	7.95E-01
**PDIA3P1**	-0.47	1.47E-01	1.00E+00	-0.27	2.50E-01	7.95E-01
**AC016949.1**	-2.56	1.19E-01	1.00E+00	0.81	2.50E-01	7.95E-01
**RBM18**	0.11	6.09E-01	1.00E+00	-0.23	2.50E-01	7.95E-01
**AC016705.2**	-3.49	5.54E-03	4.41E-01	1.99	2.50E-01	7.95E-01
**FBXO2**	-0.16	6.22E-01	1.00E+00	-0.68	2.50E-01	7.95E-01
**VPS37A**	0.27	1.47E-01	1.00E+00	-0.24	2.50E-01	7.95E-01
**RAD52**	0.43	2.31E-01	1.00E+00	0.35	2.50E-01	7.95E-01
**TGFBR1**	0.36	2.62E-01	1.00E+00	0.35	2.50E-01	7.95E-01
**AKAP1**	-0.16	4.43E-01	1.00E+00	-0.42	2.50E-01	7.95E-01
**RCE1**	0.01	9.82E-01	1.00E+00	0.23	2.50E-01	7.95E-01
**UCP2**	0.24	5.74E-01	1.00E+00	0.53	2.50E-01	7.95E-01
**ANKIB1**	0.01	9.59E-01	1.00E+00	0.40	2.50E-01	7.95E-01

**GABRE**	1.68	2.37E-02	9.15E-01	-0.66	2.50E-01	7.95E-01
**CUTALP**	0.40	1.27E-01	1.00E+00	0.31	2.50E-01	7.95E-01
**TRIB2**	-0.03	9.34E-01	1.00E+00	0.47	2.50E-01	7.95E-01
**PIGH**	-0.04	8.70E-01	1.00E+00	-0.46	2.50E-01	7.95E-01
**AL442125.1**	-1.39	8.21E-02	1.00E+00	-1.27	2.50E-01	7.95E-01
**MYC**	-1.18	1.26E-01	1.00E+00	0.49	2.50E-01	7.95E-01
**IGHG3**	4.08	4.02E-02	1.00E+00	3.60	2.50E-01	7.95E-01
**TRAV10**	0.52	8.74E-01	1.00E+00	2.06	2.50E-01	7.95E-01
**INCENP**	-0.11	5.50E-01	1.00E+00	0.99	2.50E-01	7.95E-01
**C15orf61**	-0.17	6.09E-01	1.00E+00	-0.49	2.50E-01	7.95E-01
**CRYBG3**	0.08	8.03E-01	1.00E+00	-0.35	2.50E-01	7.95E-01
**MGAT2**	-0.20	8.50E-01	1.00E+00	0.60	2.51E-01	7.95E-01
**GLRX3P2**	NA	NA	NA	0.97	2.51E-01	7.95E-01
**XLOC_012471**	-0.24	9.45E-01	1.00E+00	-1.62	2.51E-01	7.95E-01
**KIAA0930**	-0.20	4.79E-01	1.00E+00	0.51	2.51E-01	7.95E-01
**HOOK3**	-0.10	5.57E-01	1.00E+00	-0.26	2.51E-01	7.95E-01
**IL17RB**	-0.30	5.52E-01	1.00E+00	-0.61	2.51E-01	7.95E-01
**CMC1**	0.04	8.72E-01	1.00E+00	-0.46	2.51E-01	7.95E-01
**ZBTB8A**	-0.13	7.31E-01	1.00E+00	0.47	2.51E-01	7.95E-01
**AASDHPPT**	0.12	6.08E-01	1.00E+00	-0.36	2.51E-01	7.95E-01
**MCMDC2**	-0.02	9.73E-01	1.00E+00	0.53	2.51E-01	7.95E-01
**JKAMP**	0.14	5.10E-01	1.00E+00	-0.48	2.51E-01	7.95E-01
**DONSON**	-0.63	9.07E-02	1.00E+00	0.49	2.51E-01	7.95E-01
**GOLGA2P5**	-0.78	2.84E-01	1.00E+00	1.18	2.51E-01	7.95E-01
**G13213**	-0.17	9.61E-01	1.00E+00	1.04	2.51E-01	7.95E-01
**ZNF273**	0.33	3.41E-01	1.00E+00	-0.41	2.51E-01	7.95E-01
**SCNN1B**	-0.24	4.13E-01	1.00E+00	-0.38	2.51E-01	7.95E-01
**ADAM28**	-0.48	3.31E-01	1.00E+00	0.70	2.51E-01	7.95E-01
**FAM43B**	-0.12	8.89E-01	1.00E+00	1.22	2.51E-01	7.95E-01
**AC105020.1**	-2.22	1.17E-01	1.00E+00	1.77	2.51E-01	7.95E-01
**ASB12**	-1.22	3.84E-01	1.00E+00	-1.21	2.51E-01	7.95E-01
**PYGO1**	0.74	1.72E-02	8.26E-01	0.52	2.51E-01	7.95E-01

**ABRA**	-15.88	3.35E-06	1.14E-03	-1.74	2.51E-01	7.95E-01
**AL354836.1**	-0.27	7.56E-01	1.00E+00	1.00	2.51E-01	7.95E-01
**LAMA4**	0.54	2.49E-01	1.00E+00	0.55	2.51E-01	7.95E-01
**CAAP1**	-0.03	8.99E-01	1.00E+00	-0.23	2.52E-01	7.95E-01
**TRIM62**	-0.56	2.04E-01	1.00E+00	0.53	2.52E-01	7.95E-01
**AC005019.1**	NA	NA	NA	-0.97	2.52E-01	7.95E-01
**DZIP3**	-0.06	8.32E-01	1.00E+00	-0.34	2.52E-01	7.95E-01
**UBR7**	0.10	5.81E-01	1.00E+00	-0.23	2.52E-01	7.95E-01
**NKX2-8**	0.06	9.58E-01	1.00E+00	-1.83	2.52E-01	7.95E-01
**AC087752.4**	-1.32	4.09E-01	1.00E+00	-1.28	2.52E-01	7.95E-01
**AL163051.2**	0.54	5.93E-01	1.00E+00	-0.92	2.52E-01	7.95E-01
**RFFL**	0.00	9.97E-01	1.00E+00	0.42	2.52E-01	7.95E-01
**CRY2**	-0.39	1.87E-01	1.00E+00	-0.37	2.52E-01	7.95E-01
**FAM149B1**	0.26	1.83E-01	1.00E+00	0.30	2.52E-01	7.95E-01
**NBEAL2**	-0.02	9.68E-01	1.00E+00	1.03	2.52E-01	7.95E-01
**ELMSAN1**	-0.37	1.66E-01	1.00E+00	0.30	2.52E-01	7.95E-01
**RBL1**	-0.23	4.25E-01	1.00E+00	-0.41	2.52E-01	7.95E-01
**TOX**	-0.32	4.51E-01	1.00E+00	0.54	2.52E-01	7.95E-01
**NFKB2**	-0.26	3.45E-01	1.00E+00	0.93	2.52E-01	7.95E-01
**ZIC4**	1.07	4.88E-01	1.00E+00	1.56	2.52E-01	7.95E-01
**ING4**	0.09	6.58E-01	1.00E+00	-0.20	2.52E-01	7.95E-01
**ZCCHC10**	0.07	8.22E-01	1.00E+00	-0.44	2.52E-01	7.95E-01
**ZDHHC3**	0.05	8.34E-01	1.00E+00	-0.37	2.52E-01	7.95E-01
**EXT2**	0.05	7.69E-01	1.00E+00	0.35	2.52E-01	7.96E-01
**XLOC_006721**	-0.68	1.43E-01	1.00E+00	-0.48	2.52E-01	7.96E-01
**XLOC_009350**	-0.74	2.81E-02	9.75E-01	-0.84	2.52E-01	7.96E-01
**KIAA0319L**	-0.01	9.55E-01	1.00E+00	0.34	2.52E-01	7.96E-01
**ADNP**	-0.06	7.84E-01	1.00E+00	0.28	2.52E-01	7.96E-01
**FBXO40**	0.31	7.74E-01	1.00E+00	-1.99	2.52E-01	7.96E-01
**LINC01505**	-1.09	4.79E-01	1.00E+00	1.56	2.52E-01	7.96E-01
**G19167**	-3.90	7.66E-02	1.00E+00	1.52	2.52E-01	7.96E-01
**RXRG**	-0.35	4.38E-01	1.00E+00	0.53	2.53E-01	7.96E-01

**DLL1**	-0.27	4.47E-01	1.00E+00	0.44	2.53E-01	7.96E-01
**TAF7**	0.13	4.42E-01	1.00E+00	-0.20	2.53E-01	7.96E-01
**G4640**	-1.67	2.95E-01	1.00E+00	1.35	2.53E-01	7.96E-01
**REN**	-0.74	8.31E-01	1.00E+00	3.00	2.53E-01	7.96E-01
**CREG2**	-4.79	3.13E-06	1.09E-03	1.37	2.53E-01	7.96E-01
**ATXN10**	0.17	2.13E-01	1.00E+00	-0.31	2.53E-01	7.96E-01
**AGTR1**	1.34	3.49E-02	1.00E+00	-0.79	2.53E-01	7.96E-01
**AC103702.2**	-3.18	3.47E-01	1.00E+00	1.36	2.53E-01	7.96E-01
**RN7SK**	0.18	6.00E-01	1.00E+00	-0.69	2.53E-01	7.96E-01
**TMEM198**	-0.87	5.10E-02	1.00E+00	0.65	2.53E-01	7.97E-01
**LOXL1-AS1**	-0.03	9.59E-01	1.00E+00	-0.36	2.53E-01	7.97E-01
**RCN1P2**	0.92	2.70E-01	1.00E+00	-0.60	2.53E-01	7.97E-01
**FAM98A**	-0.13	5.21E-01	1.00E+00	0.23	2.53E-01	7.97E-01
**AC022706.1**	0.54	4.84E-01	1.00E+00	0.56	2.53E-01	7.97E-01
**CCDC124**	-0.28	2.74E-01	1.00E+00	0.29	2.53E-01	7.97E-01
**AC005005.4**	-0.40	8.24E-01	1.00E+00	1.18	2.54E-01	7.97E-01
**ZNF783**	0.05	8.72E-01	1.00E+00	0.61	2.54E-01	7.97E-01
**CDRT15P1**	1.04	3.93E-01	1.00E+00	1.73	2.54E-01	7.97E-01
**AL137002.2**	-1.26	1.06E-01	1.00E+00	0.97	2.54E-01	7.97E-01
**AL023581.2**	3.06	1.10E-01	1.00E+00	-0.79	2.54E-01	7.97E-01
**RPA3**	0.39	6.69E-02	1.00E+00	-0.42	2.54E-01	7.98E-01
**KSR2**	0.21	8.77E-01	1.00E+00	-1.03	2.54E-01	7.98E-01
**THEGL**	-0.02	9.84E-01	1.00E+00	1.03	2.54E-01	7.98E-01
**SCHIP1**	0.17	8.89E-01	1.00E+00	-1.05	2.54E-01	7.98E-01
**LYPLA1**	0.03	9.43E-01	1.00E+00	-0.42	2.54E-01	7.98E-01
**ZFP14**	0.18	7.45E-01	1.00E+00	-0.38	2.54E-01	7.98E-01
**LY6G5B**	-0.53	7.22E-01	1.00E+00	1.40	2.54E-01	7.98E-01
**XLOC_007800**	0.78	4.27E-01	1.00E+00	-0.87	2.54E-01	7.98E-01
**G38146**	-0.15	9.13E-01	1.00E+00	1.32	2.54E-01	7.98E-01
**AC015922.3**	0.83	1.32E-01	1.00E+00	0.60	2.54E-01	7.98E-01
**COX16**	0.49	2.14E-01	1.00E+00	-0.53	2.54E-01	7.98E-01
**GGPS1**	-0.03	9.05E-01	1.00E+00	-0.47	2.54E-01	7.98E-01

**PDGFRA**	0.66	5.63E-02	1.00E+00	0.51	2.54E-01	7.98E-01
**ZCCHC8**	0.17	5.56E-01	1.00E+00	0.45	2.55E-01	7.98E-01
**PLA2G15**	0.07	7.93E-01	1.00E+00	0.33	2.55E-01	7.98E-01
**DHPS**	-0.29	8.73E-01	1.00E+00	0.83	2.55E-01	7.98E-01
**TIMM8B**	0.03	8.79E-01	1.00E+00	-0.45	2.55E-01	7.98E-01
**AL133215.2**	-1.21	1.64E-01	1.00E+00	-1.14	2.55E-01	7.98E-01
**AC099343.2**	-0.28	8.02E-01	1.00E+00	1.54	2.55E-01	7.98E-01
**SNX6**	0.14	5.31E-01	1.00E+00	-0.22	2.55E-01	7.98E-01
**TUBB**	-0.17	4.53E-01	1.00E+00	0.23	2.55E-01	7.98E-01
**PPP6C**	0.27	1.59E-01	1.00E+00	-0.18	2.55E-01	7.98E-01
**CHCHD2P2**	2.04	2.25E-01	1.00E+00	0.97	2.55E-01	7.99E-01
**LINC01515**	0.10	7.91E-01	1.00E+00	-0.63	2.55E-01	7.99E-01
**RPS3AP5**	0.07	9.18E-01	1.00E+00	-0.58	2.55E-01	7.99E-01
**SOCS5**	-0.04	8.54E-01	1.00E+00	0.30	2.55E-01	7.99E-01
**XLOC_011119**	1.54	6.44E-01	1.00E+00	1.58	2.55E-01	7.99E-01
**PSPH**	-0.03	9.44E-01	1.00E+00	0.29	2.55E-01	7.99E-01
**TAOK1**	-0.01	9.49E-01	1.00E+00	0.26	2.55E-01	7.99E-01
**IREB2**	0.15	4.44E-01	1.00E+00	0.26	2.55E-01	7.99E-01
**TRAV2**	-0.73	7.05E-01	1.00E+00	1.82	2.55E-01	7.99E-01
**MST1L**	-0.57	4.14E-01	1.00E+00	1.01	2.55E-01	7.99E-01
**CALHM2**	0.15	6.90E-01	1.00E+00	0.58	2.55E-01	7.99E-01
**CNPY4**	0.09	7.93E-01	1.00E+00	0.42	2.55E-01	7.99E-01
**TTC14**	0.17	6.03E-01	1.00E+00	-0.31	2.55E-01	7.99E-01
**AC087645.2**	-2.21	4.59E-02	1.00E+00	1.03	2.55E-01	7.99E-01
**CPEB4**	-0.04	8.77E-01	1.00E+00	0.27	2.55E-01	7.99E-01
**AC019254.1**	-2.71	4.25E-01	1.00E+00	1.10	2.55E-01	7.99E-01
**G7779**	-1.17	6.94E-01	1.00E+00	2.10	2.55E-01	7.99E-01
**SEC14L1**	0.22	5.51E-01	1.00E+00	0.62	2.56E-01	7.99E-01
**TARBP2**	-0.08	7.14E-01	1.00E+00	0.28	2.56E-01	7.99E-01
**SCML2**	0.11	7.60E-01	1.00E+00	-0.67	2.56E-01	7.99E-01
**RNU6-531P**	-0.72	5.97E-01	1.00E+00	1.19	2.56E-01	7.99E-01
**NAT16**	-1.66	4.94E-01	1.00E+00	2.15	2.56E-01	7.99E-01

**LINC02005**	-0.85	7.52E-01	1.00E+00	1.94	2.56E-01	7.99E-01
**AC245140.2**	-0.10	9.49E-01	1.00E+00	0.83	2.56E-01	7.99E-01
**AC016405.1**	-2.36	1.21E-01	1.00E+00	-0.96	2.56E-01	7.99E-01
**AC002456.1**	0.44	8.99E-01	1.00E+00	-1.32	2.56E-01	7.99E-01
**G6968**	0.35	9.15E-01	1.00E+00	-1.30	2.56E-01	7.99E-01
**FLJ31104**	-0.86	5.70E-01	1.00E+00	0.85	2.56E-01	7.99E-01
**G35257**	0.57	7.22E-01	1.00E+00	-1.07	2.56E-01	7.99E-01
**VPS37C**	-0.23	3.53E-01	1.00E+00	0.42	2.56E-01	7.99E-01
**MRGPRF**	-0.15	6.44E-01	1.00E+00	0.60	2.56E-01	7.99E-01
**ANXA8**	-0.20	5.70E-01	1.00E+00	-0.75	2.56E-01	7.99E-01
**B4GALNT3**	0.06	8.77E-01	1.00E+00	0.80	2.56E-01	7.99E-01
**CLEC4C**	-3.48	7.15E-02	1.00E+00	1.85	2.56E-01	7.99E-01
**UBE3B**	0.11	5.02E-01	1.00E+00	-0.22	2.56E-01	7.99E-01
**LINC02595**	-0.85	3.71E-01	1.00E+00	0.69	2.56E-01	7.99E-01
**ETS1**	0.11	7.38E-01	1.00E+00	0.88	2.56E-01	7.99E-01
**NAT14**	-0.38	2.12E-01	1.00E+00	0.95	2.56E-01	7.99E-01
**ZNF717**	0.41	1.26E-01	1.00E+00	-0.39	2.56E-01	7.99E-01
**QPCTL**	-0.46	2.03E-01	1.00E+00	0.51	2.56E-01	7.99E-01
**BHMT2**	0.70	1.97E-01	1.00E+00	-0.79	2.56E-01	7.99E-01
**FRG2HP**	0.27	5.70E-01	1.00E+00	-0.85	2.56E-01	7.99E-01
**EIF5B**	-0.03	8.88E-01	1.00E+00	0.33	2.56E-01	7.99E-01
**ATP8A2**	-1.35	3.27E-02	1.00E+00	0.68	2.57E-01	7.99E-01
**CCRL2**	0.71	4.54E-01	1.00E+00	0.70	2.57E-01	7.99E-01
**INSIG2**	0.45	1.66E-01	1.00E+00	-0.41	2.57E-01	7.99E-01
**BOLA3-AS1**	0.38	3.48E-01	1.00E+00	-0.52	2.57E-01	7.99E-01
**RNU6-925P**	-2.01	1.55E-01	1.00E+00	-1.10	2.57E-01	8.00E-01
**MBD2**	0.15	4.86E-01	1.00E+00	-0.58	2.57E-01	8.00E-01
**AL031846.1**	-2.86	4.02E-01	1.00E+00	2.06	2.57E-01	8.00E-01
**EPS15L1**	-0.21	3.65E-01	1.00E+00	0.28	2.57E-01	8.00E-01
**ZDHHC8**	-0.20	4.39E-01	1.00E+00	0.63	2.57E-01	8.00E-01
**EXOSC3**	0.29	3.47E-01	1.00E+00	-0.36	2.57E-01	8.00E-01
**DCLRE1B**	-0.08	6.86E-01	1.00E+00	0.37	2.57E-01	8.00E-01

**G31133**	0.44	6.92E-01	1.00E+00	1.14	2.57E-01	8.00E-01
**KCNK17**	-0.76	7.81E-01	1.00E+00	1.91	2.57E-01	8.00E-01
**NEFM**	-3.62	3.72E-03	3.33E-01	1.14	2.57E-01	8.00E-01
**C8orf44**	0.13	8.17E-01	1.00E+00	-0.46	2.57E-01	8.00E-01
**ZNF605**	-0.04	9.08E-01	1.00E+00	0.35	2.57E-01	8.00E-01
**SCARNA5**	-0.67	8.47E-01	1.00E+00	-1.29	2.57E-01	8.00E-01
**SIPA1L1**	-0.37	1.01E-01	1.00E+00	0.35	2.57E-01	8.00E-01
**SPIB**	-2.78	3.35E-02	1.00E+00	1.78	2.57E-01	8.00E-01
**G18713**	-0.75	6.13E-01	1.00E+00	0.85	2.57E-01	8.00E-01
**INA**	-1.58	7.36E-03	5.21E-01	1.16	2.58E-01	8.00E-01
**PEBP4**	-0.08	9.13E-01	1.00E+00	-0.61	2.58E-01	8.00E-01
**GK**	-0.05	8.95E-01	1.00E+00	0.43	2.58E-01	8.00E-01
**ODF3L1**	0.28	5.89E-01	1.00E+00	-0.59	2.58E-01	8.00E-01
**KLRG2**	-0.51	8.36E-02	1.00E+00	-0.99	2.58E-01	8.00E-01
**OVOL2**	0.04	9.04E-01	1.00E+00	-0.76	2.58E-01	8.00E-01
**LINC02044**	0.79	6.27E-01	1.00E+00	-1.46	2.58E-01	8.00E-01
**ZC2HC1A**	0.02	9.53E-01	1.00E+00	-0.58	2.58E-01	8.00E-01
**CR381670.1**	1.16	3.79E-01	1.00E+00	-0.90	2.58E-01	8.00E-01
**POLR2C**	0.02	9.12E-01	1.00E+00	0.21	2.58E-01	8.00E-01
**CDK10**	-0.14	6.67E-01	1.00E+00	0.20	2.58E-01	8.00E-01
**AC009118.3**	-1.15	1.83E-01	1.00E+00	-0.82	2.58E-01	8.00E-01
**RRAS2**	0.40	1.84E-01	1.00E+00	-0.61	2.58E-01	8.00E-01
**P2RX6**	2.59	4.82E-02	1.00E+00	-1.29	2.58E-01	8.00E-01
**OGG1**	-0.27	2.73E-01	1.00E+00	-0.21	2.58E-01	8.00E-01
**AL121820.1**	1.10	1.22E-01	1.00E+00	1.25	2.58E-01	8.00E-01
**RNFT1**	0.36	2.14E-01	1.00E+00	-0.47	2.58E-01	8.00E-01
**AC025048.2**	0.39	6.21E-01	1.00E+00	0.99	2.58E-01	8.00E-01
**CUEDC1**	-0.07	8.51E-01	1.00E+00	0.90	2.58E-01	8.00E-01
**CHCHD3**	0.06	7.78E-01	1.00E+00	-0.23	2.58E-01	8.00E-01
**BLOC1S3**	-0.15	6.22E-01	1.00E+00	0.25	2.58E-01	8.00E-01
**AC092802.1**	-0.66	2.98E-01	1.00E+00	-0.70	2.58E-01	8.00E-01
**EOMES**	-2.42	8.13E-02	1.00E+00	1.52	2.58E-01	8.00E-01

**XLOC_010453**	0.57	7.71E-01	1.00E+00	2.45	2.58E-01	8.00E-01
**WDR74**	-0.20	4.08E-01	1.00E+00	0.29	2.58E-01	8.00E-01
**LINC01336**	-1.58	1.60E-01	1.00E+00	1.01	2.58E-01	8.00E-01
**AP001056.1**	-1.52	3.57E-01	1.00E+00	1.36	2.58E-01	8.00E-01
**CRHR2**	0.58	1.99E-01	1.00E+00	0.85	2.58E-01	8.00E-01
**LINC01835**	0.62	4.77E-01	1.00E+00	0.89	2.58E-01	8.00E-01
**SNX11**	0.20	3.43E-01	1.00E+00	0.26	2.58E-01	8.00E-01
**CD247**	0.03	9.72E-01	1.00E+00	1.01	2.58E-01	8.00E-01
**GPR25**	0.89	7.96E-01	1.00E+00	2.39	2.58E-01	8.00E-01
**ESS2**	-0.14	5.63E-01	1.00E+00	0.30	2.58E-01	8.00E-01
**KIF7**	0.18	6.10E-01	1.00E+00	1.04	2.58E-01	8.00E-01
**DSG2**	0.18	7.46E-01	1.00E+00	-0.38	2.58E-01	8.00E-01
**IFT27**	0.07	7.28E-01	1.00E+00	0.29	2.58E-01	8.00E-01
**AL450322.1**	0.65	3.81E-01	1.00E+00	-0.91	2.59E-01	8.00E-01
**AC104590.1**	-1.52	6.24E-01	1.00E+00	1.89	2.59E-01	8.00E-01
**REV3L**	0.25	3.54E-01	1.00E+00	0.31	2.59E-01	8.00E-01
**ICOSLG**	-0.37	3.19E-01	1.00E+00	0.77	2.59E-01	8.00E-01
**ABHD1**	-0.09	8.99E-01	1.00E+00	-0.99	2.59E-01	8.00E-01
**NUTM2G**	-0.95	3.12E-01	1.00E+00	1.06	2.59E-01	8.00E-01
**MTERF1**	0.11	7.17E-01	1.00E+00	-0.49	2.59E-01	8.00E-01
**TEAD2**	0.24	6.26E-01	1.00E+00	0.68	2.59E-01	8.00E-01
**AC007557.3**	-0.56	8.73E-01	1.00E+00	-0.94	2.59E-01	8.00E-01
**ADAMTS9**	-0.12	7.80E-01	1.00E+00	0.83	2.59E-01	8.00E-01
**AL354696.2**	-0.76	3.92E-01	1.00E+00	-0.52	2.59E-01	8.00E-01
**AC068790.5**	1.12	7.47E-01	1.00E+00	1.42	2.59E-01	8.00E-01
**AC116667.1**	-0.40	7.03E-01	1.00E+00	1.33	2.59E-01	8.00E-01
**AC084759.3**	-0.17	8.02E-01	1.00E+00	-1.32	2.59E-01	8.00E-01
**BRWD1**	0.07	7.60E-01	1.00E+00	-0.20	2.59E-01	8.00E-01
**RPL7AP28**	-0.30	7.64E-01	1.00E+00	-0.87	2.59E-01	8.00E-01
**ARPC4**	0.02	9.20E-01	1.00E+00	0.17	2.59E-01	8.00E-01
**ABHD14B**	0.16	6.29E-01	1.00E+00	-0.31	2.59E-01	8.00E-01
**TP73**	-0.70	1.07E-01	1.00E+00	-0.79	2.59E-01	8.00E-01

**TSEN2**	0.04	8.49E-01	1.00E+00	-0.25	2.59E-01	8.00E-01
**FUS**	-0.20	3.21E-01	1.00E+00	0.30	2.59E-01	8.00E-01
**AC093635.1**	-2.72	6.39E-02	1.00E+00	-0.94	2.59E-01	8.00E-01
**MEGF6**	-0.15	6.47E-01	1.00E+00	0.76	2.59E-01	8.00E-01
**LINC00243**	-0.13	9.16E-01	1.00E+00	-1.00	2.59E-01	8.00E-01
**SNHG7**	0.53	1.91E-01	1.00E+00	0.68	2.59E-01	8.00E-01
**MICAL2**	-0.61	1.25E-01	1.00E+00	0.50	2.59E-01	8.00E-01
**ITGA9-AS1**	-0.03	9.34E-01	1.00E+00	-0.41	2.60E-01	8.00E-01
**KANK1**	-0.01	9.83E-01	1.00E+00	-0.28	2.60E-01	8.00E-01
**GAA**	-0.49	4.26E-02	1.00E+00	0.65	2.60E-01	8.00E-01
**NFKBIL1**	0.07	7.75E-01	1.00E+00	0.48	2.60E-01	8.00E-01
**HBM**	0.56	8.71E-01	1.00E+00	1.61	2.60E-01	8.00E-01
**FAM120C**	-0.34	1.56E-01	1.00E+00	0.38	2.60E-01	8.00E-01
**AC108693.2**	-0.29	9.32E-01	1.00E+00	0.89	2.60E-01	8.00E-01
**POMT2**	0.39	2.75E-01	1.00E+00	0.66	2.60E-01	8.00E-01
**SEC11C**	-0.02	9.27E-01	1.00E+00	-0.35	2.60E-01	8.00E-01
**TUBB8P7**	0.14	8.87E-01	1.00E+00	0.97	2.60E-01	8.00E-01
**PAM**	0.82	5.94E-02	1.00E+00	0.45	2.60E-01	8.00E-01
**AC003072.1**	0.38	5.10E-01	1.00E+00	0.88	2.60E-01	8.00E-01
**ZC3H14**	-0.17	3.77E-01	1.00E+00	-0.24	2.60E-01	8.00E-01
**GPR39**	-0.04	9.53E-01	1.00E+00	0.73	2.60E-01	8.00E-01
**CSNK2B**	-0.05	9.14E-01	1.00E+00	-0.75	2.60E-01	8.00E-01
**AP2B1**	-0.02	9.37E-01	1.00E+00	0.41	2.60E-01	8.00E-01
**PRPF4B**	0.08	7.55E-01	1.00E+00	0.24	2.60E-01	8.00E-01
**FAM133A**	-0.82	5.16E-01	1.00E+00	-1.14	2.60E-01	8.00E-01
**AL160272.1**	3.19	5.14E-02	1.00E+00	1.66	2.60E-01	8.00E-01
**AC017002.1**	0.81	8.13E-01	1.00E+00	1.50	2.60E-01	8.00E-01
**CDC37**	-0.11	5.15E-01	1.00E+00	0.33	2.60E-01	8.00E-01
**PAAF1**	0.32	4.69E-02	1.00E+00	-0.32	2.60E-01	8.00E-01
**ACBD6**	-0.14	5.63E-01	1.00E+00	0.15	2.60E-01	8.00E-01
**MAP7D1**	-0.08	7.62E-01	1.00E+00	0.81	2.60E-01	8.00E-01
**CCDC9**	-0.11	7.03E-01	1.00E+00	0.74	2.61E-01	8.00E-01

**PACSIN2**	0.26	1.67E-01	1.00E+00	0.22	2.61E-01	8.00E-01
**XLOC_003741**	-2.60	1.42E-01	1.00E+00	-1.48	2.61E-01	8.00E-01
**Z82217.1**	0.01	9.96E-01	1.00E+00	1.11	2.61E-01	8.00E-01
**ARG2**	0.03	9.30E-01	1.00E+00	0.56	2.61E-01	8.00E-01
**SOD1**	0.19	3.09E-01	1.00E+00	-0.23	2.61E-01	8.00E-01
**CCNO**	-1.16	2.75E-03	2.72E-01	-0.62	2.61E-01	8.00E-01
**SF3B6**	-0.04	8.56E-01	1.00E+00	-0.37	2.61E-01	8.00E-01
**CRBN**	0.34	7.79E-02	1.00E+00	-0.42	2.61E-01	8.00E-01
**BTBD18**	0.74	7.00E-01	1.00E+00	-1.55	2.61E-01	8.00E-01
**AL078581.1**	-0.43	7.23E-01	1.00E+00	0.91	2.61E-01	8.00E-01
**THAP2**	0.06	8.91E-01	1.00E+00	0.37	2.61E-01	8.00E-01
**FLNB-AS1**	-2.95	1.32E-01	1.00E+00	1.47	2.61E-01	8.00E-01
**ZNF215**	1.23	9.22E-02	1.00E+00	0.89	2.61E-01	8.00E-01
**ATP5MPL**	0.23	2.35E-01	1.00E+00	-0.49	2.61E-01	8.00E-01
**ZNF518A**	-0.08	7.97E-01	1.00E+00	-0.25	2.61E-01	8.00E-01
**DPH3**	0.10	6.94E-01	1.00E+00	-0.32	2.61E-01	8.00E-01
**GPRC5C**	-0.29	3.84E-01	1.00E+00	-0.44	2.61E-01	8.00E-01
**PRDM8**	0.87	8.85E-02	1.00E+00	0.90	2.61E-01	8.00E-01
**XLOC_002995**	-1.64	4.04E-01	1.00E+00	-1.33	2.61E-01	8.00E-01
**AC114956.2**	-2.40	5.15E-03	4.18E-01	-1.01	2.61E-01	8.00E-01
**ITIH1**	-2.65	1.10E-01	1.00E+00	1.36	2.61E-01	8.00E-01
**ZNF358**	0.20	5.91E-01	1.00E+00	0.49	2.61E-01	8.00E-01
**SOGA1**	-0.36	1.43E-01	1.00E+00	0.67	2.61E-01	8.00E-01
**NMBR**	0.01	9.95E-01	1.00E+00	1.48	2.61E-01	8.00E-01
**XLOC_013994**	0.13	6.72E-01	1.00E+00	-0.60	2.61E-01	8.00E-01
**ZBTB11**	-0.05	8.61E-01	1.00E+00	-0.26	2.61E-01	8.00E-01
**CTDSP2**	0.20	2.81E-01	1.00E+00	0.43	2.61E-01	8.00E-01
**NDUFV3**	-0.19	5.06E-01	1.00E+00	-0.16	2.61E-01	8.00E-01
**HIGD2A**	-0.03	8.99E-01	1.00E+00	-0.47	2.61E-01	8.00E-01
**CLPTM1**	-0.18	3.93E-01	1.00E+00	0.40	2.61E-01	8.00E-01
**CABP4**	-0.06	9.43E-01	1.00E+00	0.81	2.61E-01	8.00E-01
**KCTD7**	0.41	4.98E-01	1.00E+00	0.53	2.61E-01	8.00E-01

**RP11-391M20.1**	0.25	4.56E-01	1.00E+00	-0.36	2.62E-01	8.00E-01
**TSPAN7**	0.05	9.15E-01	1.00E+00	-0.58	2.62E-01	8.00E-01
**Z99129.4**	0.30	4.03E-01	1.00E+00	-0.43	2.62E-01	8.00E-01
**H3F3A**	0.00	9.86E-01	1.00E+00	-0.27	2.62E-01	8.00E-01
**MBOAT4**	-0.19	9.30E-01	1.00E+00	0.77	2.62E-01	8.00E-01
**NT5C2**	-0.18	3.50E-01	1.00E+00	0.39	2.62E-01	8.00E-01
**IGHG1**	4.23	8.61E-11	5.11E-08	3.51	2.62E-01	8.00E-01
**AL353764.1**	0.32	8.74E-01	1.00E+00	1.34	2.62E-01	8.00E-01
**RAB36**	-0.01	9.73E-01	1.00E+00	0.58	2.62E-01	8.00E-01
**HOXA9**	-0.56	1.45E-01	1.00E+00	-0.59	2.62E-01	8.00E-01
**DENND5B-AS1**	-2.74	2.58E-01	1.00E+00	-0.64	2.62E-01	8.00E-01
**PSMC1**	-0.06	7.83E-01	1.00E+00	0.18	2.62E-01	8.00E-01
**PTPRU**	0.16	6.28E-01	1.00E+00	0.56	2.62E-01	8.00E-01
**SLC25A42**	0.10	7.71E-01	1.00E+00	-0.52	2.62E-01	8.00E-01
**UBE2Q2P2**	0.97	2.85E-01	1.00E+00	-0.81	2.62E-01	8.00E-01
**AC092053.3**	-0.94	1.24E-01	1.00E+00	-0.63	2.62E-01	8.00E-01
**DCDC2**	0.99	2.79E-01	1.00E+00	1.17	2.62E-01	8.00E-01
**TIMM21**	0.39	1.86E-01	1.00E+00	-0.38	2.62E-01	8.00E-01
**KIF26A**	-0.05	8.92E-01	1.00E+00	0.86	2.62E-01	8.00E-01
**IGKV1-12**	1.38	6.83E-01	1.00E+00	3.54	2.62E-01	8.00E-01
**CASQ2**	-0.55	3.64E-01	1.00E+00	-0.72	2.62E-01	8.00E-01
**PRR14**	-0.26	3.01E-01	1.00E+00	0.37	2.62E-01	8.00E-01
**USP16**	0.00	9.81E-01	1.00E+00	-0.15	2.62E-01	8.00E-01
**LINC02014**	-1.91	4.40E-01	1.00E+00	-1.43	2.62E-01	8.00E-01
**OLA1P1**	-1.04	5.58E-01	1.00E+00	-0.55	2.62E-01	8.00E-01
**CALR**	-0.27	2.08E-01	1.00E+00	0.66	2.62E-01	8.00E-01
**GSEC**	-0.41	4.76E-01	1.00E+00	0.67	2.62E-01	8.00E-01
**FBXO21**	0.02	9.12E-01	1.00E+00	-0.29	2.62E-01	8.00E-01
**ZSCAN20**	0.45	3.00E-01	1.00E+00	0.64	2.62E-01	8.00E-01
**ACBD5**	0.06	8.45E-01	1.00E+00	-0.19	2.62E-01	8.00E-01
**G3769**	-2.83	1.49E-01	1.00E+00	-1.42	2.62E-01	8.00E-01
**AL049840.1**	0.76	3.80E-01	1.00E+00	0.78	2.62E-01	8.00E-01

**CMSS1**	-0.33	6.03E-02	1.00E+00	-0.28	2.62E-01	8.00E-01
**VPS18**	-0.06	8.05E-01	1.00E+00	0.54	2.62E-01	8.00E-01
**AVPI1**	-0.59	1.32E-01	1.00E+00	0.30	2.62E-01	8.00E-01
**AC128688.1**	-0.79	4.94E-01	1.00E+00	-0.96	2.62E-01	8.00E-01
**DRG1**	0.08	6.35E-01	1.00E+00	-0.26	2.62E-01	8.00E-01
**HOXC-AS3**	-0.81	4.49E-01	1.00E+00	-0.53	2.63E-01	8.00E-01
**BCL2L10**	0.14	7.56E-01	1.00E+00	-0.60	2.63E-01	8.00E-01
**AGFG1**	0.19	3.82E-01	1.00E+00	-0.51	2.63E-01	8.01E-01
**OTOA**	1.27	3.42E-01	1.00E+00	1.13	2.63E-01	8.01E-01
**ATP6AP2**	0.08	6.85E-01	1.00E+00	-0.30	2.63E-01	8.01E-01
**MCM5**	-0.68	3.54E-03	3.20E-01	0.25	2.63E-01	8.01E-01
**ZNF207**	-0.24	2.17E-01	1.00E+00	0.18	2.63E-01	8.01E-01
**AL162742.1**	-1.93	3.85E-02	1.00E+00	-1.12	2.63E-01	8.01E-01
**AC010325.1**	0.24	8.31E-01	1.00E+00	-0.88	2.63E-01	8.01E-01
**NMB**	1.72	4.26E-02	1.00E+00	-0.60	2.63E-01	8.01E-01
**ECD**	0.05	7.70E-01	1.00E+00	-0.24	2.63E-01	8.01E-01
**CLEC3B**	0.90	4.67E-02	1.00E+00	-0.74	2.63E-01	8.01E-01
**AC087521.2**	1.11	6.41E-01	1.00E+00	1.22	2.63E-01	8.01E-01
**RASA4CP**	0.26	4.06E-01	1.00E+00	0.39	2.63E-01	8.01E-01
**MYBPC3**	0.75	6.70E-01	1.00E+00	2.07	2.63E-01	8.01E-01
**LINC01671**	0.62	4.58E-01	1.00E+00	1.11	2.63E-01	8.01E-01
**DUXAP9**	-1.19	6.12E-01	1.00E+00	0.99	2.63E-01	8.01E-01
**LINC01013**	-0.82	4.02E-01	1.00E+00	0.94	2.63E-01	8.01E-01
**GREB1**	-0.01	9.91E-01	1.00E+00	0.62	2.63E-01	8.01E-01
**AC092720.1**	0.26	7.66E-01	1.00E+00	1.29	2.63E-01	8.01E-01
**LETMD1**	0.12	6.99E-01	1.00E+00	-0.29	2.63E-01	8.01E-01
**STXBP5**	0.01	9.80E-01	1.00E+00	-0.41	2.63E-01	8.01E-01
**ATXN2**	-0.02	9.39E-01	1.00E+00	-0.24	2.63E-01	8.01E-01
**SPATA20P1**	NA	NA	NA	1.98	2.63E-01	8.01E-01
**PACSIN3**	-0.20	4.67E-01	1.00E+00	-0.41	2.63E-01	8.01E-01
**SUMO2**	0.12	4.43E-01	1.00E+00	-0.27	2.63E-01	8.01E-01
**GTF2H1**	0.02	9.36E-01	1.00E+00	-0.23	2.63E-01	8.01E-01

**SLC35A5**	0.20	5.97E-01	1.00E+00	-0.33	2.64E-01	8.01E-01
**TRAPPC4**	0.08	7.15E-01	1.00E+00	-0.38	2.64E-01	8.01E-01
**C6orf203**	0.27	3.49E-01	1.00E+00	-0.35	2.64E-01	8.01E-01
**SH3TC2**	-0.26	6.22E-01	1.00E+00	0.70	2.64E-01	8.01E-01
**KCNMA1**	-0.36	5.20E-01	1.00E+00	0.71	2.64E-01	8.01E-01
**AL137793.1**	-0.29	8.35E-01	1.00E+00	-1.32	2.64E-01	8.01E-01
**INPP5K**	-0.09	5.60E-01	1.00E+00	-0.17	2.64E-01	8.01E-01
**COL7A1**	-0.80	4.38E-02	1.00E+00	0.88	2.64E-01	8.01E-01
**HOXD9**	0.21	5.29E-01	1.00E+00	0.54	2.64E-01	8.01E-01
**NPTX2**	0.08	8.77E-01	1.00E+00	1.06	2.64E-01	8.01E-01
**AC091074.1**	-1.50	4.27E-01	1.00E+00	-2.06	2.64E-01	8.01E-01
**ALKBH8**	0.26	2.15E-01	1.00E+00	0.26	2.64E-01	8.01E-01
**CBLN3**	0.22	5.54E-01	1.00E+00	0.89	2.64E-01	8.01E-01
**STIM1**	0.14	3.64E-01	1.00E+00	0.38	2.64E-01	8.01E-01
**AC009302.1**	-1.86	2.69E-01	1.00E+00	-0.73	2.64E-01	8.01E-01
**VAMP5**	-0.08	8.05E-01	1.00E+00	0.44	2.64E-01	8.01E-01
**G36217**	-1.55	4.25E-01	1.00E+00	-1.55	2.64E-01	8.01E-01
**C1orf216**	-0.12	7.04E-01	1.00E+00	0.45	2.64E-01	8.01E-01
**ATP1B2**	-0.33	5.87E-01	1.00E+00	0.51	2.64E-01	8.01E-01
**XLOC_001716**	1.35	4.87E-01	1.00E+00	-1.82	2.64E-01	8.01E-01
**CAMK2N2**	-2.40	2.22E-02	8.97E-01	2.11	2.64E-01	8.01E-01
**LINC00636**	0.21	9.02E-01	1.00E+00	1.26	2.64E-01	8.01E-01
**RPL7A**	-0.22	4.93E-01	1.00E+00	0.46	2.64E-01	8.01E-01
**APOBEC3A**	-1.09	3.46E-01	1.00E+00	3.49	2.64E-01	8.01E-01
**ENPP5**	0.19	6.97E-01	1.00E+00	-0.77	2.64E-01	8.01E-01
**RALGAPA1**	-0.23	3.73E-01	1.00E+00	-0.37	2.64E-01	8.01E-01
**XLOC_009447**	-0.96	6.89E-02	1.00E+00	-0.74	2.64E-01	8.01E-01
**ZNRF1**	-0.31	3.27E-01	1.00E+00	-0.51	2.64E-01	8.01E-01
**PAICSP1**	-2.09	3.08E-01	1.00E+00	0.93	2.64E-01	8.01E-01
**PRKAA2**	0.24	7.11E-01	1.00E+00	-0.58	2.64E-01	8.01E-01
**NDUFB6**	-0.10	5.18E-01	1.00E+00	-0.47	2.64E-01	8.01E-01
**AC083899.1**	0.41	5.42E-01	1.00E+00	0.36	2.65E-01	8.01E-01

**HLA-DQB1-AS1**	1.30	4.77E-01	1.00E+00	1.18	2.65E-01	8.01E-01
**RPL18A**	-0.30	2.79E-01	1.00E+00	0.39	2.65E-01	8.01E-01
**AL691447.2**	0.64	5.15E-01	1.00E+00	1.36	2.65E-01	8.01E-01
**AGTPBP1**	-0.20	3.83E-01	1.00E+00	-0.49	2.65E-01	8.01E-01
**DGKD**	0.39	4.16E-01	1.00E+00	0.71	2.65E-01	8.01E-01
**MYEF2**	0.67	3.44E-01	1.00E+00	0.39	2.65E-01	8.01E-01
**POLR3K**	-0.06	8.62E-01	1.00E+00	-0.39	2.65E-01	8.01E-01
**TMEM8B**	0.15	7.19E-01	1.00E+00	-0.52	2.65E-01	8.01E-01
**NUDT7**	0.12	8.38E-01	1.00E+00	-0.58	2.65E-01	8.01E-01
**PPP2R3A**	-0.32	3.70E-01	1.00E+00	-0.35	2.65E-01	8.01E-01
**XLOC_001374**	-3.31	7.36E-02	1.00E+00	-0.86	2.65E-01	8.01E-01
**ATP13A3**	0.78	5.57E-02	1.00E+00	0.43	2.65E-01	8.01E-01
**NEK5**	0.86	2.97E-01	1.00E+00	0.64	2.65E-01	8.02E-01
**ANXA2R**	0.11	8.21E-01	1.00E+00	0.62	2.65E-01	8.02E-01
**URM1**	-0.26	1.41E-01	1.00E+00	0.21	2.65E-01	8.02E-01
**GABPA**	0.05	8.55E-01	1.00E+00	-0.27	2.66E-01	8.02E-01
**UPK3A**	-0.76	7.73E-01	1.00E+00	1.35	2.66E-01	8.02E-01
**ARID5A**	-0.25	4.28E-01	1.00E+00	0.66	2.66E-01	8.02E-01
**AC131206.1**	0.05	9.37E-01	1.00E+00	0.51	2.66E-01	8.02E-01
**PKP4**	0.00	9.95E-01	1.00E+00	0.37	2.66E-01	8.02E-01
**PARL**	0.24	6.30E-01	1.00E+00	-0.45	2.66E-01	8.02E-01
**LINC01762**	-2.64	2.71E-02	9.60E-01	-1.08	2.66E-01	8.02E-01
**AC104162.2**	-1.67	1.42E-01	1.00E+00	-0.65	2.66E-01	8.02E-01
**MIR133A1HG**	-2.86	1.33E-01	1.00E+00	-1.22	2.66E-01	8.03E-01
**COX19**	0.13	5.43E-01	1.00E+00	0.25	2.66E-01	8.03E-01
**CCR5**	0.16	8.16E-01	1.00E+00	3.48	2.66E-01	8.03E-01
**MYRIP**	-0.14	7.86E-01	1.00E+00	-0.87	2.66E-01	8.03E-01
**AL133520.1**	1.02	5.64E-02	1.00E+00	0.93	2.66E-01	8.03E-01
**AL445487.1**	-1.36	6.93E-01	1.00E+00	1.69	2.66E-01	8.03E-01
**SLC12A9**	-0.45	6.13E-02	1.00E+00	0.81	2.66E-01	8.03E-01
**UTP14A**	-0.10	5.98E-01	1.00E+00	0.34	2.66E-01	8.03E-01
**NPM1P37**	1.27	7.13E-01	1.00E+00	-1.40	2.66E-01	8.03E-01

**SLC4A7**	0.34	3.73E-01	1.00E+00	0.47	2.66E-01	8.03E-01
**TNFSF18**	-1.40	2.85E-01	1.00E+00	1.42	2.66E-01	8.03E-01
**G6485**	0.11	8.60E-01	1.00E+00	1.17	2.66E-01	8.03E-01
**ATP1B3-AS1**	-2.77	4.14E-01	1.00E+00	1.99	2.66E-01	8.03E-01
**PRR15L**	0.16	8.05E-01	1.00E+00	-0.78	2.66E-01	8.03E-01
**HERC2P9**	0.19	7.79E-01	1.00E+00	0.62	2.66E-01	8.03E-01
**CREG1**	-0.04	9.06E-01	1.00E+00	-0.40	2.66E-01	8.03E-01
**FST**	0.99	4.03E-02	1.00E+00	0.57	2.67E-01	8.03E-01
**LARS**	-0.28	8.99E-02	1.00E+00	-0.15	2.67E-01	8.03E-01
**LINC02182**	-2.46	2.56E-01	1.00E+00	0.99	2.67E-01	8.03E-01
**FXR1**	-0.04	7.96E-01	1.00E+00	0.21	2.67E-01	8.03E-01
**FGFR1OP2**	0.02	9.32E-01	1.00E+00	-0.23	2.67E-01	8.03E-01
**PGP**	-0.60	3.95E-02	1.00E+00	-0.78	2.67E-01	8.03E-01
**MEGF10**	0.25	7.83E-01	1.00E+00	-1.09	2.67E-01	8.03E-01
**RPS4XP14**	0.56	8.71E-01	1.00E+00	-1.07	2.67E-01	8.03E-01
**AC090409.1**	1.12	1.49E-01	1.00E+00	-0.71	2.67E-01	8.03E-01
**PUS7**	-0.53	4.67E-02	1.00E+00	-0.25	2.67E-01	8.03E-01
**TMEM45B**	0.05	8.92E-01	1.00E+00	0.55	2.67E-01	8.03E-01
**POLR3A**	0.07	7.70E-01	1.00E+00	0.33	2.67E-01	8.03E-01
**AC073072.2**	0.88	7.98E-01	1.00E+00	-0.81	2.67E-01	8.03E-01
**COG6**	0.08	7.35E-01	1.00E+00	-0.25	2.67E-01	8.03E-01
**TEX261**	0.20	4.11E-01	1.00E+00	-0.44	2.67E-01	8.03E-01
**TRMT2B**	0.20	2.78E-01	1.00E+00	-0.24	2.67E-01	8.03E-01
**HMGB1P5**	0.38	8.33E-02	1.00E+00	-0.27	2.67E-01	8.03E-01
**DNMT1**	-0.38	7.88E-02	1.00E+00	0.39	2.67E-01	8.03E-01
**MKLN1**	0.24	3.59E-01	1.00E+00	-0.23	2.67E-01	8.03E-01
**SNX18P3**	-0.07	9.26E-01	1.00E+00	-1.39	2.67E-01	8.03E-01
**PIH1D1**	0.12	4.67E-01	1.00E+00	0.19	2.67E-01	8.03E-01
**DOCK3**	-0.68	2.62E-01	1.00E+00	-0.79	2.67E-01	8.03E-01
**STPG1**	0.01	9.71E-01	1.00E+00	-0.23	2.67E-01	8.03E-01
**THYN1**	0.14	5.83E-01	1.00E+00	-0.38	2.67E-01	8.03E-01
**CUL3**	0.23	4.00E-01	1.00E+00	-0.48	2.67E-01	8.03E-01

**MRS2**	0.23	3.69E-01	1.00E+00	-0.28	2.68E-01	8.03E-01
**AP001057.1**	-0.26	9.17E-01	1.00E+00	1.48	2.68E-01	8.03E-01
**ST6GALNAC4**	-0.28	4.10E-01	1.00E+00	0.44	2.68E-01	8.03E-01
**BET1L**	-0.16	5.12E-01	1.00E+00	0.25	2.68E-01	8.03E-01
**AP001330.1**	-0.30	7.54E-01	1.00E+00	-0.93	2.68E-01	8.03E-01
**RNF216**	-0.22	2.49E-01	1.00E+00	0.30	2.68E-01	8.03E-01
**CDHR3**	0.33	5.02E-01	1.00E+00	-0.74	2.68E-01	8.03E-01
**NFIA-AS2**	-2.38	1.20E-01	1.00E+00	1.01	2.68E-01	8.03E-01
**SEZ6L**	-2.11	5.11E-01	1.00E+00	-0.98	2.68E-01	8.03E-01
**GPX8**	0.22	5.76E-01	1.00E+00	0.41	2.68E-01	8.03E-01
**G41319**	-0.06	9.49E-01	1.00E+00	0.77	2.68E-01	8.03E-01
**DNAJA3**	0.04	8.30E-01	1.00E+00	-0.25	2.68E-01	8.03E-01
**MRPL22**	0.07	6.68E-01	1.00E+00	-0.39	2.68E-01	8.03E-01
**AC103760.1**	1.12	1.41E-01	1.00E+00	-0.82	2.68E-01	8.03E-01
**RPL10P11**	-0.81	5.82E-01	1.00E+00	-1.57	2.68E-01	8.03E-01
**AC009570.1**	-2.99	8.19E-02	1.00E+00	-1.26	2.68E-01	8.03E-01
**SPIN3**	0.17	7.00E-01	1.00E+00	0.62	2.68E-01	8.03E-01
**AF131215.7**	0.04	9.57E-01	1.00E+00	-1.04	2.68E-01	8.03E-01
**PTPRD-AS1**	-2.69	7.72E-02	1.00E+00	-1.39	2.68E-01	8.03E-01
**PROKR1**	4.97	4.86E-02	1.00E+00	1.79	2.68E-01	8.03E-01
**SLC3A1**	1.05	7.57E-01	1.00E+00	-1.08	2.68E-01	8.03E-01
**PHF19**	0.26	2.75E-01	1.00E+00	0.48	2.68E-01	8.03E-01
**SUCLG2**	-0.03	8.66E-01	1.00E+00	-0.21	2.68E-01	8.03E-01
**TH**	0.41	7.63E-01	1.00E+00	1.72	2.68E-01	8.03E-01
**AC023983.1**	3.27	3.32E-01	1.00E+00	-0.82	2.68E-01	8.03E-01
**SSPO**	-0.15	8.80E-01	1.00E+00	1.27	2.68E-01	8.03E-01
**CCND2-AS1**	1.23	3.30E-01	1.00E+00	0.85	2.68E-01	8.04E-01
**KIAA1841**	-0.19	5.72E-01	1.00E+00	-0.43	2.69E-01	8.04E-01
**OXA1L**	-0.19	3.77E-01	1.00E+00	0.23	2.69E-01	8.04E-01
**ARHGAP35**	-0.20	3.78E-01	1.00E+00	0.52	2.69E-01	8.04E-01
**OSGEPL1**	0.10	7.40E-01	1.00E+00	-0.44	2.69E-01	8.04E-01
**MARK4**	-0.67	2.49E-02	9.28E-01	0.74	2.69E-01	8.04E-01

**LINC00518**	-0.30	8.13E-01	1.00E+00	1.13	2.69E-01	8.04E-01
**ST13P4**	-0.33	7.00E-01	1.00E+00	-0.46	2.69E-01	8.04E-01
**SGCG**	0.95	1.69E-03	2.28E-01	-0.83	2.69E-01	8.04E-01
**AP001324.1**	0.18	6.64E-01	1.00E+00	-0.66	2.69E-01	8.04E-01
**LCN2**	-1.00	1.76E-01	1.00E+00	0.92	2.69E-01	8.04E-01
**NELFE**	-0.14	4.52E-01	1.00E+00	-0.11	2.69E-01	8.04E-01
**ADK**	0.15	5.98E-01	1.00E+00	-0.42	2.69E-01	8.04E-01
**AL136982.5**	0.44	5.54E-01	1.00E+00	0.90	2.69E-01	8.04E-01
**AIM2**	-2.46	2.38E-02	9.16E-01	3.47	2.69E-01	8.04E-01
**SMIM6**	-2.80	1.43E-01	1.00E+00	-1.76	2.69E-01	8.04E-01
**TFR2**	-0.02	9.52E-01	1.00E+00	0.81	2.69E-01	8.04E-01
**GPR27**	-0.13	6.80E-01	1.00E+00	-0.60	2.69E-01	8.04E-01
**LYAR**	0.22	2.84E-01	1.00E+00	0.26	2.69E-01	8.04E-01
**PI4K2A**	0.13	5.10E-01	1.00E+00	0.54	2.69E-01	8.04E-01
**IQCE**	-0.15	4.37E-01	1.00E+00	0.52	2.69E-01	8.04E-01
**AL138781.2**	0.89	1.54E-01	1.00E+00	-1.19	2.69E-01	8.04E-01
**SEPHS1**	-0.07	6.58E-01	1.00E+00	-0.20	2.69E-01	8.04E-01
**CDK5RAP2**	0.00	9.88E-01	1.00E+00	0.29	2.69E-01	8.04E-01
**SNAPIN**	0.01	9.67E-01	1.00E+00	-0.34	2.69E-01	8.04E-01
**IGFALS**	-1.66	6.48E-02	1.00E+00	1.23	2.69E-01	8.04E-01
**NAP1L4P1**	0.64	5.39E-01	1.00E+00	0.84	2.69E-01	8.04E-01
**WDR91**	0.05	8.41E-01	1.00E+00	0.52	2.69E-01	8.04E-01
**GREM2**	0.07	8.55E-01	1.00E+00	0.69	2.69E-01	8.04E-01
**BCAS1**	-0.15	8.46E-01	1.00E+00	-0.65	2.70E-01	8.04E-01
**AL359220.1**	0.08	8.08E-01	1.00E+00	-0.39	2.70E-01	8.04E-01
**AC018557.2**	2.85	2.15E-01	1.00E+00	1.25	2.70E-01	8.04E-01
**LRWD1**	-0.09	6.97E-01	1.00E+00	0.38	2.70E-01	8.04E-01
**KTN1-AS1**	-0.43	2.07E-01	1.00E+00	-0.41	2.70E-01	8.04E-01
**AC242988.2**	1.12	6.05E-01	1.00E+00	1.85	2.70E-01	8.04E-01
**AC068594.1**	1.71	2.96E-01	1.00E+00	1.04	2.70E-01	8.04E-01
**CNOT7**	0.13	4.02E-01	1.00E+00	-0.32	2.70E-01	8.04E-01
**SAR1B**	-0.01	9.53E-01	1.00E+00	-0.31	2.70E-01	8.04E-01

**AL031727.1**	0.42	7.98E-01	1.00E+00	-0.79	2.70E-01	8.04E-01
**ZNF433**	-0.26	3.34E-01	1.00E+00	-0.46	2.70E-01	8.04E-01
**PRDX5**	0.02	9.28E-01	1.00E+00	0.22	2.70E-01	8.04E-01
**IL1RAP**	-0.06	8.81E-01	1.00E+00	0.29	2.70E-01	8.04E-01
**COA3**	0.03	8.70E-01	1.00E+00	-0.38	2.70E-01	8.04E-01
**FAM174A**	0.18	5.58E-01	1.00E+00	-0.29	2.70E-01	8.04E-01
**CLVS2**	-1.18	1.18E-01	1.00E+00	-1.17	2.70E-01	8.04E-01
**ST3GAL2**	-0.07	8.01E-01	1.00E+00	0.64	2.70E-01	8.04E-01
**SYT2**	-0.44	6.91E-01	1.00E+00	0.84	2.70E-01	8.04E-01
**ZNF324B**	-0.06	8.49E-01	1.00E+00	0.60	2.71E-01	8.05E-01
**PARP16**	0.08	6.82E-01	1.00E+00	-0.38	2.71E-01	8.05E-01
**ATL3**	0.42	4.99E-02	1.00E+00	-0.36	2.71E-01	8.05E-01
**RMI1**	-0.34	2.81E-01	1.00E+00	-0.33	2.71E-01	8.05E-01
**SLC36A2**	-2.46	2.91E-01	1.00E+00	-0.97	2.71E-01	8.05E-01
**PAK2**	0.02	9.01E-01	1.00E+00	0.33	2.71E-01	8.05E-01
**CACNA1A**	-0.36	5.61E-01	1.00E+00	0.91	2.71E-01	8.05E-01
**C9orf147**	-2.38	4.46E-02	1.00E+00	0.84	2.71E-01	8.05E-01
**MAFF**	-0.18	7.52E-01	1.00E+00	0.45	2.71E-01	8.06E-01
**PPIL4**	0.23	3.67E-01	1.00E+00	-0.31	2.71E-01	8.06E-01
**TMEM43**	0.21	3.35E-01	1.00E+00	-0.23	2.71E-01	8.06E-01
**UNC5B-AS1**	-0.44	5.59E-01	1.00E+00	0.77	2.71E-01	8.06E-01
**FKBP3**	-0.14	5.53E-01	1.00E+00	-0.35	2.71E-01	8.06E-01
**ACSL3**	0.31	4.50E-01	1.00E+00	-0.31	2.71E-01	8.06E-01
**COIL**	-0.10	6.21E-01	1.00E+00	-0.29	2.71E-01	8.06E-01
**ZGPAT**	-0.18	5.45E-01	1.00E+00	0.39	2.71E-01	8.06E-01
**ZC3H12A**	-1.77	8.49E-03	5.68E-01	0.69	2.71E-01	8.06E-01
**CHMP4A**	0.56	2.02E-01	1.00E+00	0.59	2.71E-01	8.06E-01
**GLMN**	0.22	3.97E-01	1.00E+00	0.37	2.71E-01	8.06E-01
**Z93241.1**	-0.45	6.94E-01	1.00E+00	-1.91	2.71E-01	8.06E-01
**PI15**	-1.76	3.55E-04	7.50E-02	1.07	2.72E-01	8.06E-01
**AC127521.1**	1.27	3.92E-01	1.00E+00	-0.99	2.72E-01	8.06E-01
**SAMD3**	0.13	8.92E-01	1.00E+00	1.27	2.72E-01	8.06E-01

**AC105760.1**	NA	NA	NA	2.41	2.72E-01	8.06E-01
**AL121987.2**	0.84	8.00E-01	1.00E+00	-1.02	2.72E-01	8.06E-01
**AL596202.1**	1.05	1.93E-01	1.00E+00	-0.76	2.72E-01	8.06E-01
**AC104339.1**	NA	NA	NA	1.09	2.72E-01	8.06E-01
**OLFM1**	-0.19	6.08E-01	1.00E+00	-0.61	2.72E-01	8.06E-01
**PSMA3**	-0.02	9.39E-01	1.00E+00	-0.23	2.72E-01	8.06E-01
**PLA2G16**	1.86	8.63E-02	1.00E+00	-0.68	2.72E-01	8.06E-01
**NUP50**	0.09	7.31E-01	1.00E+00	0.17	2.72E-01	8.06E-01
**AFF4**	-0.12	4.07E-01	1.00E+00	0.39	2.72E-01	8.06E-01
**RP11-87H9.3**	-0.58	6.35E-01	1.00E+00	-0.73	2.72E-01	8.06E-01
**G22375**	-0.04	9.75E-01	1.00E+00	0.86	2.72E-01	8.06E-01
**ITSN2**	0.07	8.12E-01	1.00E+00	-0.22	2.72E-01	8.07E-01
**DHX32**	-0.08	7.96E-01	1.00E+00	-0.39	2.72E-01	8.07E-01
**AL031722.1**	-2.95	3.16E-02	1.00E+00	0.81	2.72E-01	8.07E-01
**PENK**	0.36	5.70E-01	1.00E+00	0.75	2.72E-01	8.07E-01
**XLOC_000340**	-0.94	7.78E-01	1.00E+00	-1.28	2.72E-01	8.07E-01
**TMEM159**	0.27	1.86E-01	1.00E+00	-0.29	2.72E-01	8.07E-01
**L3MBTL1**	0.24	6.30E-01	1.00E+00	-0.74	2.72E-01	8.07E-01
**CREBRF**	0.17	4.40E-01	1.00E+00	-0.38	2.72E-01	8.07E-01
**OXSR1**	0.28	1.14E-01	1.00E+00	0.36	2.72E-01	8.07E-01
**AC003991.1**	NA	NA	NA	1.27	2.72E-01	8.07E-01
**MEP1A**	-0.87	7.40E-01	1.00E+00	1.65	2.73E-01	8.07E-01
**AL158211.1**	1.00	6.00E-01	1.00E+00	-1.93	2.73E-01	8.07E-01
**COX7CP1**	0.06	9.79E-01	1.00E+00	-0.72	2.73E-01	8.07E-01
**SCUBE2**	0.04	9.14E-01	1.00E+00	-0.46	2.73E-01	8.07E-01
**ARHGAP6**	0.41	3.08E-01	1.00E+00	0.56	2.73E-01	8.07E-01
**PSMA6**	-0.32	5.03E-01	1.00E+00	0.72	2.73E-01	8.07E-01
**S100A5**	0.63	3.72E-01	1.00E+00	0.84	2.73E-01	8.07E-01
**AC008969.1**	-0.12	7.92E-01	1.00E+00	0.60	2.73E-01	8.07E-01
**EPB41L2**	0.31	4.55E-01	1.00E+00	0.52	2.73E-01	8.07E-01
**KSR1**	-0.30	2.81E-01	1.00E+00	0.63	2.73E-01	8.07E-01
**BCKDK**	-0.07	7.72E-01	1.00E+00	0.20	2.73E-01	8.07E-01

**OBP2A**	-2.12	1.34E-01	1.00E+00	-0.86	2.73E-01	8.07E-01
**PIK3CB**	0.17	4.72E-01	1.00E+00	-0.25	2.73E-01	8.07E-01
**ULK2**	0.19	3.44E-01	1.00E+00	-0.49	2.73E-01	8.07E-01
**PHC2**	0.03	9.16E-01	1.00E+00	0.67	2.73E-01	8.07E-01
**XLOC_002882**	-0.81	8.13E-01	1.00E+00	1.85	2.73E-01	8.07E-01
**REXO2**	0.22	3.52E-01	1.00E+00	-0.42	2.73E-01	8.07E-01
**H1F0**	-0.19	3.89E-01	1.00E+00	0.35	2.73E-01	8.07E-01
**CGREF1**	-0.10	8.74E-01	1.00E+00	0.85	2.73E-01	8.07E-01
**TADA3**	-0.07	7.75E-01	1.00E+00	0.28	2.73E-01	8.07E-01
**ST3GAL4**	-0.06	8.75E-01	1.00E+00	0.32	2.73E-01	8.07E-01
**DCAF15**	-0.21	3.88E-01	1.00E+00	0.56	2.73E-01	8.07E-01
**IGKV1-9**	5.23	1.15E-01	1.00E+00	3.46	2.73E-01	8.07E-01
**TMA16**	0.03	9.15E-01	1.00E+00	-0.31	2.73E-01	8.07E-01
**MYL12BP2**	1.41	2.35E-01	1.00E+00	-1.04	2.73E-01	8.07E-01
**AC024909.2**	3.31	2.83E-01	1.00E+00	-1.18	2.73E-01	8.07E-01
**POLE4**	-0.08	6.74E-01	1.00E+00	-0.36	2.73E-01	8.07E-01
**AC084033.3**	0.74	1.72E-01	1.00E+00	-0.44	2.73E-01	8.07E-01
**PKN1**	0.20	6.76E-01	1.00E+00	0.74	2.73E-01	8.07E-01
**EPHX4**	-0.73	4.16E-01	1.00E+00	-0.78	2.73E-01	8.07E-01
**RPL13A**	-0.14	6.39E-01	1.00E+00	0.32	2.73E-01	8.07E-01
**IGHV1-2**	6.41	2.41E-04	5.49E-02	3.45	2.74E-01	8.07E-01
**CCNI2**	-2.35	8.79E-02	1.00E+00	1.43	2.74E-01	8.07E-01
**SH2D4B**	-0.24	9.02E-01	1.00E+00	1.22	2.74E-01	8.07E-01
**ZFP91**	0.12	5.26E-01	1.00E+00	0.20	2.74E-01	8.07E-01
**CWF19L1**	-0.12	6.26E-01	1.00E+00	-0.22	2.74E-01	8.07E-01
**XLOC_007955**	1.70	5.40E-01	1.00E+00	1.03	2.74E-01	8.07E-01
**CGRRF1**	0.29	2.92E-01	1.00E+00	-0.39	2.74E-01	8.07E-01
**APOL2**	-0.48	3.20E-01	1.00E+00	0.61	2.74E-01	8.07E-01
**TOP2B**	-0.02	8.95E-01	1.00E+00	-0.36	2.74E-01	8.07E-01
**BLNK**	-0.29	4.71E-01	1.00E+00	0.41	2.74E-01	8.07E-01
**PRSS21**	-2.09	8.91E-03	5.87E-01	-0.71	2.74E-01	8.07E-01
**NUPL2**	-0.17	5.18E-01	1.00E+00	-0.29	2.74E-01	8.08E-01

**OARD1**	0.23	2.36E-01	1.00E+00	-0.27	2.74E-01	8.08E-01
**APBB1IP**	1.04	1.35E-01	1.00E+00	0.78	2.74E-01	8.08E-01
**TNNI3**	-0.86	3.69E-01	1.00E+00	1.01	2.74E-01	8.08E-01
**G33879**	-0.15	9.15E-01	1.00E+00	1.34	2.74E-01	8.08E-01
**ZNF281**	0.07	8.24E-01	1.00E+00	0.28	2.74E-01	8.08E-01
**DEDD2**	-0.20	5.40E-01	1.00E+00	0.24	2.74E-01	8.08E-01
**PLXNB1**	-0.15	5.29E-01	1.00E+00	0.52	2.74E-01	8.08E-01
**G6PC3**	0.06	8.90E-01	1.00E+00	0.34	2.74E-01	8.08E-01
**TERC**	-2.83	2.07E-01	1.00E+00	-0.68	2.75E-01	8.08E-01
**CTNND2**	-0.37	9.02E-01	1.00E+00	-1.18	2.75E-01	8.08E-01
**TRIM45**	0.19	5.22E-01	1.00E+00	0.58	2.75E-01	8.08E-01
**SYT11**	-0.18	7.29E-01	1.00E+00	0.60	2.75E-01	8.08E-01
**PAOX**	0.04	8.48E-01	1.00E+00	-0.64	2.75E-01	8.08E-01
**WDPCP**	0.28	4.13E-01	1.00E+00	-0.41	2.75E-01	8.08E-01
**DCUN1D5**	-0.22	3.57E-01	1.00E+00	0.25	2.75E-01	8.08E-01
**URB1**	0.11	5.87E-01	1.00E+00	0.45	2.75E-01	8.08E-01
**AC073548.1**	-2.36	1.75E-01	1.00E+00	1.26	2.75E-01	8.08E-01
**ATP5MC1**	-0.17	3.01E-01	1.00E+00	-0.42	2.75E-01	8.08E-01
**LINC01940**	1.18	3.90E-01	1.00E+00	1.68	2.75E-01	8.08E-01
**CTNNAL1**	0.04	8.84E-01	1.00E+00	-0.35	2.75E-01	8.08E-01
**ENHO**	0.79	2.05E-01	1.00E+00	-0.63	2.75E-01	8.08E-01
**CARD9**	-0.93	2.78E-02	9.71E-01	0.63	2.75E-01	8.08E-01
**AC113410.2**	0.19	7.43E-01	1.00E+00	0.71	2.75E-01	8.08E-01
**LIPE-AS1**	2.60	8.61E-04	1.48E-01	-0.67	2.75E-01	8.08E-01
**TRIM56**	0.26	3.94E-01	1.00E+00	0.54	2.75E-01	8.08E-01
**VDAC3**	0.02	9.11E-01	1.00E+00	-0.28	2.75E-01	8.08E-01
**AC010976.1**	0.88	5.42E-01	1.00E+00	1.20	2.75E-01	8.08E-01
**TFAP2A-AS2**	-2.95	2.03E-02	8.88E-01	0.92	2.75E-01	8.08E-01
**TPST1**	0.34	2.18E-01	1.00E+00	0.34	2.75E-01	8.08E-01
**AC138393.1**	-0.43	2.22E-01	1.00E+00	-0.57	2.76E-01	8.08E-01
**AC005550.2**	-0.11	9.67E-01	1.00E+00	-1.33	2.76E-01	8.08E-01
**ZC3HC1**	0.24	4.35E-01	1.00E+00	-0.31	2.76E-01	8.08E-01

**ITIH6**	1.05	5.33E-01	1.00E+00	-1.39	2.76E-01	8.08E-01
**PCP4**	-0.18	8.07E-01	1.00E+00	-0.69	2.76E-01	8.08E-01
**PIN4P1**	-0.75	8.29E-01	1.00E+00	-1.09	2.76E-01	8.08E-01
**TCIM**	0.09	7.92E-01	1.00E+00	0.55	2.76E-01	8.08E-01
**LZTFL1**	-0.05	8.07E-01	1.00E+00	-0.25	2.76E-01	8.08E-01
**RSPO3**	0.08	8.82E-01	1.00E+00	0.66	2.76E-01	8.08E-01
**DDX21**	-0.24	4.08E-01	1.00E+00	0.40	2.76E-01	8.08E-01
**TFB1M**	0.02	9.30E-01	1.00E+00	-0.45	2.76E-01	8.08E-01
**G18534**	-0.26	7.57E-01	1.00E+00	-0.86	2.76E-01	8.08E-01
**SLC39A3**	-0.30	2.44E-01	1.00E+00	0.25	2.76E-01	8.08E-01
**RPS3**	-0.19	5.48E-01	1.00E+00	0.31	2.76E-01	8.08E-01
**AL109809.1**	-0.22	8.39E-01	1.00E+00	0.81	2.76E-01	8.08E-01
**ARL17B**	0.93	1.21E-01	1.00E+00	0.51	2.76E-01	8.08E-01
**TMEM51**	-0.06	8.48E-01	1.00E+00	0.59	2.76E-01	8.08E-01
**LINC00528**	-1.99	2.64E-01	1.00E+00	1.47	2.76E-01	8.08E-01
**WASHC3**	-0.17	3.76E-01	1.00E+00	-0.37	2.76E-01	8.08E-01
**XLOC_008614**	-2.40	3.96E-01	1.00E+00	0.87	2.76E-01	8.08E-01
**DDX24**	-0.04	8.03E-01	1.00E+00	0.17	2.76E-01	8.08E-01
**TTC23L**	-0.57	4.66E-01	1.00E+00	0.95	2.76E-01	8.08E-01
**XLOC_014129**	1.09	1.10E-01	1.00E+00	1.12	2.76E-01	8.08E-01
**ZNF101**	-0.20	4.86E-01	1.00E+00	0.32	2.76E-01	8.08E-01
**MAN1B1**	-0.09	6.41E-01	1.00E+00	0.21	2.76E-01	8.08E-01
**XLOC_010693**	-1.21	4.37E-01	1.00E+00	-0.77	2.76E-01	8.08E-01
**KIF3C**	-0.28	4.36E-01	1.00E+00	0.74	2.76E-01	8.08E-01
**LINC00667**	-0.13	6.98E-01	1.00E+00	-0.29	2.76E-01	8.08E-01
**TERF2**	-0.14	4.82E-01	1.00E+00	-0.27	2.76E-01	8.09E-01
**ZFAS1**	0.04	9.03E-01	1.00E+00	0.30	2.77E-01	8.09E-01
**G32019**	-1.00	5.58E-01	1.00E+00	-0.98	2.77E-01	8.09E-01
**Z84484.1**	0.24	8.72E-01	1.00E+00	1.02	2.77E-01	8.09E-01
**PDGFRB**	-0.19	6.61E-01	1.00E+00	0.77	2.77E-01	8.09E-01
**INTS6-AS1**	-0.57	5.03E-01	1.00E+00	-0.57	2.77E-01	8.09E-01
**TCTN2**	0.13	4.92E-01	1.00E+00	0.51	2.77E-01	8.09E-01

**GSTM1**	3.32	3.03E-01	1.00E+00	3.41	2.77E-01	8.09E-01
**CYSRT1**	-0.18	7.61E-01	1.00E+00	0.69	2.77E-01	8.09E-01
**TMEM221**	-0.03	9.72E-01	1.00E+00	-1.02	2.77E-01	8.09E-01
**ATXN7L3B**	0.15	3.75E-01	1.00E+00	-0.20	2.77E-01	8.09E-01
**XLOC_001457**	0.03	9.85E-01	1.00E+00	1.59	2.77E-01	8.09E-01
**RTTN**	0.43	2.52E-01	1.00E+00	0.53	2.77E-01	8.09E-01
**PCMTD1**	0.11	7.55E-01	1.00E+00	-0.28	2.77E-01	8.09E-01
**TMEM25**	-0.02	9.58E-01	1.00E+00	-0.28	2.77E-01	8.09E-01
**TBX2**	-0.03	9.51E-01	1.00E+00	1.02	2.77E-01	8.09E-01
**AMD1**	0.06	8.27E-01	1.00E+00	0.19	2.77E-01	8.09E-01
**AC122688.3**	0.59	5.67E-01	1.00E+00	0.72	2.77E-01	8.09E-01
**RNASE1**	0.07	8.85E-01	1.00E+00	0.49	2.77E-01	8.09E-01
**ASPN**	0.04	9.39E-01	1.00E+00	0.47	2.77E-01	8.09E-01
**FAM53B**	-0.33	2.34E-01	1.00E+00	0.58	2.77E-01	8.09E-01
**RABL6**	-0.14	5.86E-01	1.00E+00	0.66	2.77E-01	8.09E-01
**CDC6**	-0.32	3.91E-01	1.00E+00	0.46	2.77E-01	8.09E-01
**AC097662.1**	0.62	3.76E-01	1.00E+00	-1.02	2.77E-01	8.09E-01
**MT-ATP8**	-0.25	5.51E-01	1.00E+00	-0.63	2.78E-01	8.09E-01
**RPL35P5**	0.13	8.92E-01	1.00E+00	-0.66	2.78E-01	8.09E-01
**AP001469.3**	0.36	5.23E-01	1.00E+00	0.82	2.78E-01	8.09E-01
**ABCA17P**	1.07	3.17E-01	1.00E+00	-0.86	2.78E-01	8.09E-01
**SMIM26**	0.46	1.48E-01	1.00E+00	-0.62	2.78E-01	8.10E-01
**RBM20**	-0.33	5.68E-01	1.00E+00	0.87	2.78E-01	8.10E-01
**AP4S1**	-0.01	9.72E-01	1.00E+00	0.23	2.78E-01	8.10E-01
**SERPINA9**	-0.93	4.62E-01	1.00E+00	-1.22	2.78E-01	8.10E-01
**FKBP4**	-0.30	1.10E-01	1.00E+00	-0.32	2.78E-01	8.10E-01
**CTPS1**	-0.50	3.78E-02	1.00E+00	0.38	2.78E-01	8.10E-01
**GHDC**	0.15	6.03E-01	1.00E+00	0.70	2.78E-01	8.10E-01
**PJVK**	0.74	3.42E-01	1.00E+00	-0.66	2.78E-01	8.10E-01
**LACTB2**	-0.49	1.14E-01	1.00E+00	-0.45	2.78E-01	8.10E-01
**AC105749.1**	0.27	8.35E-01	1.00E+00	1.06	2.78E-01	8.10E-01
**PGAM1**	-0.12	5.06E-01	1.00E+00	0.31	2.78E-01	8.10E-01

**PIF1**	-0.30	7.49E-01	1.00E+00	1.30	2.78E-01	8.10E-01
**WDR61**	0.14	4.26E-01	1.00E+00	-0.34	2.78E-01	8.10E-01
**UGGT2**	0.11	7.31E-01	1.00E+00	-0.34	2.78E-01	8.10E-01
**VPS33B**	0.74	1.40E-01	1.00E+00	-0.38	2.78E-01	8.10E-01
**BICD1**	-0.40	1.60E-01	1.00E+00	0.51	2.78E-01	8.10E-01
**DNAAF2**	0.07	8.06E-01	1.00E+00	-0.30	2.78E-01	8.10E-01
**SNHG10**	-0.51	3.90E-01	1.00E+00	-0.53	2.78E-01	8.10E-01
**AC002511.1**	0.55	8.65E-01	1.00E+00	1.77	2.78E-01	8.10E-01
**IRF4**	0.09	8.50E-01	1.00E+00	-0.41	2.79E-01	8.10E-01
**AC008264.2**	0.10	9.77E-01	1.00E+00	2.23	2.79E-01	8.10E-01
**CUX1**	0.01	9.83E-01	1.00E+00	0.30	2.79E-01	8.10E-01
**AC110079.1**	-0.70	1.34E-01	1.00E+00	0.82	2.79E-01	8.10E-01
**YTHDC1**	0.06	7.45E-01	1.00E+00	0.30	2.79E-01	8.10E-01
**ZRSR2**	-0.06	7.89E-01	1.00E+00	-0.41	2.79E-01	8.10E-01
**SYNE2**	-0.50	3.20E-02	1.00E+00	-0.34	2.79E-01	8.10E-01
**AC007529.2**	3.87	1.74E-01	1.00E+00	-1.37	2.79E-01	8.10E-01
**ZFYVE27**	0.13	6.87E-01	1.00E+00	0.27	2.79E-01	8.10E-01
**AL357060.1**	0.28	5.34E-01	1.00E+00	0.39	2.79E-01	8.10E-01
**TINF2**	0.03	8.37E-01	1.00E+00	0.19	2.79E-01	8.10E-01
**HINT1**	-0.07	7.37E-01	1.00E+00	-0.34	2.79E-01	8.10E-01
**MGAM2**	0.41	7.51E-01	1.00E+00	1.60	2.79E-01	8.10E-01
**ATP10A**	0.26	5.42E-01	1.00E+00	0.81	2.79E-01	8.10E-01
**RCOR1**	0.03	9.06E-01	1.00E+00	0.50	2.79E-01	8.10E-01
**FAM86C1**	-0.21	6.74E-01	1.00E+00	0.32	2.79E-01	8.10E-01
**LINC01679**	-0.51	1.86E-01	1.00E+00	0.39	2.79E-01	8.10E-01
**LINC00242**	-1.96	1.08E-01	1.00E+00	0.62	2.79E-01	8.10E-01
**AMTN**	-1.70	1.89E-01	1.00E+00	-1.53	2.79E-01	8.10E-01
**RGPD8**	-0.07	8.79E-01	1.00E+00	0.59	2.79E-01	8.10E-01
**TCP11L1**	0.27	3.82E-01	1.00E+00	0.42	2.79E-01	8.10E-01
**SCRN2**	0.19	6.26E-01	1.00E+00	-0.35	2.79E-01	8.10E-01
**MRTFA-AS1**	0.91	7.54E-01	1.00E+00	1.29	2.79E-01	8.10E-01
**SMIM8**	-0.02	9.55E-01	1.00E+00	-0.52	2.79E-01	8.10E-01

**LAGE3**	-0.16	5.87E-01	1.00E+00	-0.42	2.79E-01	8.10E-01
**PCSK7**	-0.07	7.56E-01	1.00E+00	0.55	2.79E-01	8.10E-01
**SLC9A6**	0.03	8.55E-01	1.00E+00	-0.22	2.79E-01	8.10E-01
**MNAT1**	0.18	5.07E-01	1.00E+00	-0.33	2.79E-01	8.10E-01
**ZNF77**	-0.23	3.27E-01	1.00E+00	-0.33	2.79E-01	8.10E-01
**PNPLA4**	-0.16	4.59E-01	1.00E+00	-0.28	2.80E-01	8.10E-01
**CXorf57**	0.38	2.13E-01	1.00E+00	0.59	2.80E-01	8.10E-01
**TTC17**	-0.05	8.14E-01	1.00E+00	0.18	2.80E-01	8.10E-01
**MDH2**	-0.13	4.91E-01	1.00E+00	-0.21	2.80E-01	8.11E-01
**TMEM254**	0.05	8.78E-01	1.00E+00	-0.53	2.80E-01	8.11E-01
**S100A3**	-2.23	1.22E-03	1.86E-01	0.69	2.80E-01	8.11E-01
**ZCCHC9**	0.28	3.20E-01	1.00E+00	0.14	2.80E-01	8.11E-01
**WTIP**	0.15	7.35E-01	1.00E+00	0.80	2.80E-01	8.11E-01
**TBATA**	-0.30	7.54E-01	1.00E+00	-0.91	2.80E-01	8.11E-01
**EPHA1-AS1**	-0.52	4.63E-01	1.00E+00	-0.66	2.80E-01	8.11E-01
**XLOC_002897**	0.44	8.12E-01	1.00E+00	1.08	2.80E-01	8.11E-01
**C1RL-AS1**	0.13	8.72E-01	1.00E+00	0.56	2.80E-01	8.11E-01
**AC110619.1**	-0.92	3.19E-01	1.00E+00	1.11	2.80E-01	8.11E-01
**SUSD4**	-0.15	7.42E-01	1.00E+00	-0.50	2.80E-01	8.11E-01
**AP000892.3**	-1.35	1.51E-01	1.00E+00	0.65	2.80E-01	8.11E-01
**LMNB1**	-0.13	6.03E-01	1.00E+00	0.58	2.80E-01	8.11E-01
**TTI1**	0.11	6.54E-01	1.00E+00	-0.30	2.80E-01	8.11E-01
**AC004241.5**	0.48	7.41E-01	1.00E+00	-0.89	2.80E-01	8.11E-01
**SLC23A1**	0.00	1.00E+00	1.00E+00	0.70	2.80E-01	8.11E-01
**LIX1L-AS1**	1.17	4.77E-01	1.00E+00	-0.60	2.81E-01	8.11E-01
**ZNF562**	0.11	7.03E-01	1.00E+00	0.28	2.81E-01	8.11E-01
**ZNF600**	0.10	7.73E-01	1.00E+00	-0.42	2.81E-01	8.11E-01
**MEIS3**	-0.20	6.21E-01	1.00E+00	0.81	2.81E-01	8.11E-01
**SLC16A1-AS1**	0.06	9.06E-01	1.00E+00	0.55	2.81E-01	8.11E-01
**AC018607.1**	1.76	6.07E-01	1.00E+00	1.55	2.81E-01	8.11E-01
**COX5B**	-0.05	7.79E-01	1.00E+00	-0.27	2.81E-01	8.12E-01
**XLOC_011484**	3.06	9.14E-02	1.00E+00	-1.49	2.81E-01	8.12E-01

**SOX4**	-0.12	6.86E-01	1.00E+00	0.32	2.81E-01	8.12E-01
**PURA**	0.14	5.68E-01	1.00E+00	-0.28	2.81E-01	8.12E-01
**DUSP18**	-0.49	1.60E-01	1.00E+00	0.32	2.81E-01	8.12E-01
**ZSCAN2**	-0.24	7.12E-01	1.00E+00	0.51	2.81E-01	8.12E-01
**DLEU7-AS1**	0.38	9.14E-01	1.00E+00	2.19	2.81E-01	8.12E-01
**KCNK15-AS1**	-1.88	3.02E-01	1.00E+00	0.83	2.81E-01	8.12E-01
**E4F1**	-0.20	4.34E-01	1.00E+00	0.40	2.81E-01	8.12E-01
**THORLNC**	-1.70	2.66E-02	9.50E-01	-0.70	2.81E-01	8.12E-01
**AC007686.3**	0.68	4.42E-01	1.00E+00	-0.73	2.81E-01	8.12E-01
**CDC42BPB**	-0.16	4.37E-01	1.00E+00	0.75	2.81E-01	8.12E-01
**AC106028.3**	-0.36	8.74E-01	1.00E+00	-1.03	2.81E-01	8.12E-01
**AC105046.1**	-0.86	2.63E-01	1.00E+00	-0.76	2.81E-01	8.12E-01
**TMEM108**	-0.14	7.78E-01	1.00E+00	0.56	2.81E-01	8.12E-01
**RNU6-238P**	0.16	9.55E-01	1.00E+00	1.17	2.81E-01	8.12E-01
**KRT6C**	-2.79	8.45E-02	1.00E+00	3.37	2.81E-01	8.12E-01
**TRBV30**	-0.95	7.81E-01	1.00E+00	0.95	2.81E-01	8.12E-01
**ZNF454**	-0.09	8.87E-01	1.00E+00	-0.74	2.81E-01	8.12E-01
**MTAP**	0.07	7.81E-01	1.00E+00	-0.17	2.82E-01	8.12E-01
**ENO4**	-1.65	2.17E-01	1.00E+00	-0.78	2.82E-01	8.12E-01
**APOL6**	-0.11	8.57E-01	1.00E+00	0.64	2.82E-01	8.12E-01
**LINC00630**	-0.24	5.68E-01	1.00E+00	0.44	2.82E-01	8.12E-01
**P2RY6**	-0.11	8.85E-01	1.00E+00	2.58	2.82E-01	8.12E-01
**EBI3**	-0.33	6.51E-01	1.00E+00	1.14	2.82E-01	8.12E-01
**PLXNA4**	0.43	1.76E-01	1.00E+00	0.52	2.82E-01	8.12E-01
**MMP9**	-0.59	2.77E-01	1.00E+00	0.91	2.82E-01	8.12E-01
**ASIC1**	-0.33	4.97E-01	1.00E+00	0.88	2.82E-01	8.12E-01
**NCDN**	-0.58	6.03E-02	1.00E+00	0.56	2.82E-01	8.12E-01
**G38513**	-0.76	4.99E-01	1.00E+00	1.19	2.82E-01	8.12E-01
**AC090197.1**	-1.22	6.15E-01	1.00E+00	1.54	2.82E-01	8.12E-01
**PALM**	0.25	6.17E-01	1.00E+00	0.70	2.82E-01	8.12E-01
**XLOC_002196**	1.12	3.86E-01	1.00E+00	-0.77	2.82E-01	8.12E-01
**CGAS**	-0.72	7.16E-02	1.00E+00	0.61	2.82E-01	8.12E-01

**KCNAB3**	0.05	9.62E-01	1.00E+00	1.73	2.82E-01	8.12E-01
**ARRDC1-AS1**	0.07	7.77E-01	1.00E+00	-0.34	2.82E-01	8.12E-01
**BTF3L4**	0.22	3.43E-01	1.00E+00	-0.23	2.82E-01	8.12E-01
**ZYG11B**	0.03	8.92E-01	1.00E+00	-0.33	2.82E-01	8.13E-01
**ZFP36L2**	-0.51	9.84E-02	1.00E+00	-0.36	2.82E-01	8.13E-01
**TUBGCP2**	-0.40	2.17E-02	8.94E-01	0.28	2.83E-01	8.13E-01
**ARHGAP21**	-0.10	7.67E-01	1.00E+00	0.63	2.83E-01	8.13E-01
**SIK3-IT1**	0.56	8.71E-01	1.00E+00	-1.65	2.83E-01	8.13E-01
**CAMK2D**	0.24	2.69E-01	1.00E+00	0.22	2.83E-01	8.13E-01
**UGT2B7**	-3.93	2.40E-02	9.18E-01	-1.13	2.83E-01	8.13E-01
**COMMD3**	-0.03	9.21E-01	1.00E+00	-0.58	2.83E-01	8.13E-01
**ORC5**	0.34	1.11E-01	1.00E+00	-0.37	2.83E-01	8.13E-01
**AC069360.1**	-1.98	2.16E-01	1.00E+00	0.97	2.83E-01	8.13E-01
**AGBL5**	-0.07	7.16E-01	1.00E+00	-0.29	2.83E-01	8.13E-01
**RALGDS**	0.29	5.40E-01	1.00E+00	-0.33	2.83E-01	8.13E-01
**AKIRIN2**	0.09	6.73E-01	1.00E+00	0.26	2.83E-01	8.13E-01
**SH3RF3-AS1**	-0.22	5.84E-01	1.00E+00	1.11	2.83E-01	8.13E-01
**AC004921.1**	-0.85	5.79E-01	1.00E+00	1.47	2.83E-01	8.13E-01
**SGPL1**	0.23	4.76E-01	1.00E+00	0.28	2.83E-01	8.13E-01
**NKD2**	-0.04	9.21E-01	1.00E+00	0.79	2.83E-01	8.13E-01
**ELMOD1**	0.10	8.93E-01	1.00E+00	-0.71	2.83E-01	8.13E-01
**G27456**	0.66	4.00E-01	1.00E+00	1.27	2.83E-01	8.13E-01
**AC004847.1**	-0.60	5.73E-01	1.00E+00	1.60	2.83E-01	8.14E-01
**MRPS30**	-0.24	2.89E-01	1.00E+00	-0.26	2.83E-01	8.14E-01
**XLOC_001595**	2.12	2.76E-01	1.00E+00	-0.95	2.84E-01	8.14E-01
**SGPP2**	0.09	8.21E-01	1.00E+00	-0.79	2.84E-01	8.14E-01
**FAM87A**	-3.15	5.94E-03	4.59E-01	-1.06	2.84E-01	8.14E-01
**AL356515.1**	1.01	6.31E-01	1.00E+00	1.04	2.84E-01	8.14E-01
**CYGB**	0.27	4.50E-01	1.00E+00	0.75	2.84E-01	8.14E-01
**AAAS**	-0.27	1.02E-01	1.00E+00	-0.20	2.84E-01	8.14E-01
**APCDD1L**	-0.25	6.42E-01	1.00E+00	0.75	2.84E-01	8.14E-01
**TULP4**	-0.04	8.94E-01	1.00E+00	-0.38	2.84E-01	8.14E-01

**HAND2-AS1**	1.00	1.37E-01	1.00E+00	0.91	2.84E-01	8.14E-01
**AL512363.1**	-1.52	3.58E-03	3.22E-01	0.66	2.84E-01	8.14E-01
**XRCC1**	0.12	5.67E-01	1.00E+00	0.24	2.84E-01	8.14E-01
**RFESD**	0.24	7.21E-01	1.00E+00	-0.65	2.84E-01	8.14E-01
**AC125437.2**	-0.94	7.83E-01	1.00E+00	1.41	2.84E-01	8.15E-01
**SNAPC1**	0.28	3.51E-01	1.00E+00	-0.34	2.84E-01	8.15E-01
**MAPK10**	1.17	1.93E-01	1.00E+00	-0.69	2.84E-01	8.15E-01
**AC124067.4**	0.15	8.69E-01	1.00E+00	1.20	2.84E-01	8.15E-01
**PINK1**	-0.01	9.63E-01	1.00E+00	-0.69	2.84E-01	8.15E-01
**MMADHC**	0.07	7.46E-01	1.00E+00	-0.33	2.84E-01	8.15E-01
**AGMAT**	-2.50	2.19E-02	8.96E-01	0.91	2.84E-01	8.15E-01
**WNT3**	-0.11	7.75E-01	1.00E+00	-0.71	2.84E-01	8.15E-01
**SLC27A6**	-0.96	6.25E-02	1.00E+00	-1.16	2.85E-01	8.15E-01
**GNAL**	-0.37	1.65E-01	1.00E+00	-0.43	2.85E-01	8.15E-01
**ZNF132**	0.28	5.64E-01	1.00E+00	0.44	2.85E-01	8.15E-01
**CALB2**	2.99	1.98E-02	8.80E-01	-0.92	2.85E-01	8.15E-01
**TXK**	-0.71	4.14E-01	1.00E+00	0.78	2.85E-01	8.15E-01
**DCD**	-1.23	3.73E-01	1.00E+00	-3.34	2.85E-01	8.15E-01
**RRM2**	0.15	7.49E-01	1.00E+00	0.51	2.85E-01	8.15E-01
**SNAPC4**	-0.09	7.62E-01	1.00E+00	0.77	2.85E-01	8.15E-01
**AL109936.6**	0.03	9.79E-01	1.00E+00	-1.10	2.85E-01	8.15E-01
**UBE2QL1**	-0.79	2.38E-01	1.00E+00	-0.79	2.85E-01	8.15E-01
**DNM1L**	-0.04	8.30E-01	1.00E+00	-0.17	2.85E-01	8.15E-01
**FLCN**	0.33	2.23E-01	1.00E+00	0.59	2.85E-01	8.15E-01
**FKRP**	-0.10	7.60E-01	1.00E+00	0.72	2.85E-01	8.15E-01
**G4300**	-0.06	9.38E-01	1.00E+00	-0.77	2.85E-01	8.15E-01
**DCUN1D4**	0.22	3.79E-01	1.00E+00	-0.34	2.85E-01	8.15E-01
**GATM**	0.09	8.26E-01	1.00E+00	-0.56	2.85E-01	8.15E-01
**NAP1L6**	0.23	8.56E-01	1.00E+00	1.11	2.85E-01	8.15E-01
**NECTIN2**	-0.23	3.47E-01	1.00E+00	0.55	2.85E-01	8.15E-01
**OR2T10**	-3.99	8.18E-02	1.00E+00	-1.81	2.85E-01	8.15E-01
**AC010247.1**	-3.73	1.02E-01	1.00E+00	1.35	2.85E-01	8.15E-01

**AL021328.1**	0.32	6.32E-01	1.00E+00	0.44	2.85E-01	8.15E-01
**CACNA1F**	-0.94	3.36E-01	1.00E+00	0.97	2.85E-01	8.15E-01
**XLOC_010271**	1.40	4.71E-01	1.00E+00	1.61	2.85E-01	8.15E-01
**TSNAX**	0.19	5.69E-01	1.00E+00	-0.29	2.85E-01	8.15E-01
**SECISBP2L**	0.05	8.26E-01	1.00E+00	0.35	2.85E-01	8.15E-01
**PLPP1**	0.20	6.47E-01	1.00E+00	-0.42	2.86E-01	8.15E-01
**UBR1**	-0.06	8.55E-01	1.00E+00	0.31	2.86E-01	8.15E-01
**E2F5**	-0.06	9.03E-01	1.00E+00	-0.43	2.86E-01	8.15E-01
**BDP1**	-0.28	2.71E-01	1.00E+00	-0.28	2.86E-01	8.15E-01
**CSGALNACT2**	0.02	9.30E-01	1.00E+00	0.44	2.86E-01	8.15E-01
**METTL1**	-0.34	2.91E-01	1.00E+00	0.34	2.86E-01	8.15E-01
**Z95331.1**	1.42	3.85E-01	1.00E+00	0.97	2.86E-01	8.15E-01
**CASP12**	0.71	1.65E-01	1.00E+00	0.76	2.86E-01	8.15E-01
**G10695**	-0.83	1.52E-01	1.00E+00	-0.70	2.86E-01	8.16E-01
**CYB5RL**	-0.15	6.80E-01	1.00E+00	0.44	2.86E-01	8.16E-01
**CCDC189**	0.60	8.61E-01	1.00E+00	0.90	2.86E-01	8.16E-01
**XLOC_002002**	0.82	5.85E-01	1.00E+00	1.58	2.86E-01	8.16E-01
**CAMK1**	0.31	7.10E-01	1.00E+00	-0.73	2.86E-01	8.16E-01
**CELF4**	-1.14	3.14E-01	1.00E+00	0.73	2.86E-01	8.16E-01
**TUBD1**	0.32	3.42E-01	1.00E+00	-0.35	2.86E-01	8.16E-01
**CCIN**	0.64	6.99E-01	1.00E+00	1.49	2.86E-01	8.16E-01
**PTPRN**	-0.21	9.51E-01	1.00E+00	1.29	2.86E-01	8.16E-01
**PLAG1**	0.22	5.52E-01	1.00E+00	0.41	2.86E-01	8.16E-01
**DOK1**	1.20	1.20E-01	1.00E+00	0.49	2.87E-01	8.16E-01
**SEMA4D**	-0.15	5.81E-01	1.00E+00	0.46	2.87E-01	8.16E-01
**DHRS12**	0.49	1.36E-01	1.00E+00	-0.40	2.87E-01	8.16E-01
**H2AFVP1**	1.00	5.22E-01	1.00E+00	0.63	2.87E-01	8.16E-01
**MAP6**	-0.72	2.39E-01	1.00E+00	-0.70	2.87E-01	8.16E-01
**C2orf69**	-0.15	6.06E-01	1.00E+00	-0.26	2.87E-01	8.16E-01
**TBILA**	0.45	6.49E-01	1.00E+00	0.82	2.87E-01	8.16E-01
**TRIM69**	-0.04	9.22E-01	1.00E+00	0.34	2.87E-01	8.16E-01
**LDHD**	0.83	1.57E-01	1.00E+00	-0.53	2.87E-01	8.16E-01

**MRAP**	1.09	7.45E-01	1.00E+00	-1.18	2.87E-01	8.16E-01
**KIRREL3**	0.34	6.79E-01	1.00E+00	0.73	2.87E-01	8.16E-01
**RUSC2**	0.07	7.17E-01	1.00E+00	0.71	2.87E-01	8.16E-01
**PHLDB3**	0.05	9.26E-01	1.00E+00	0.51	2.87E-01	8.16E-01
**YY1**	0.12	4.45E-01	1.00E+00	0.28	2.87E-01	8.16E-01
**ST5**	-0.37	1.73E-01	1.00E+00	0.49	2.87E-01	8.16E-01
**DERL2**	0.19	3.91E-01	1.00E+00	-0.34	2.87E-01	8.16E-01
**AC023906.5**	0.89	4.63E-01	1.00E+00	0.97	2.87E-01	8.16E-01
**AC007365.1**	0.60	4.80E-01	1.00E+00	0.87	2.87E-01	8.16E-01
**TOGARAM2**	0.54	3.60E-01	1.00E+00	-0.89	2.87E-01	8.16E-01
**ZNF165**	0.09	7.97E-01	1.00E+00	0.42	2.87E-01	8.16E-01
**C19orf24**	-0.20	5.42E-01	1.00E+00	0.18	2.87E-01	8.16E-01
**STK3**	0.03	9.09E-01	1.00E+00	-0.30	2.87E-01	8.16E-01
**TERF1**	-0.04	8.51E-01	1.00E+00	-0.25	2.87E-01	8.16E-01
**ATP5MG**	0.22	4.77E-01	1.00E+00	-0.55	2.87E-01	8.16E-01
**AC004925.1**	-0.23	7.90E-01	1.00E+00	-1.22	2.87E-01	8.16E-01
**XLOC_006070**	-1.09	5.66E-01	1.00E+00	0.70	2.87E-01	8.16E-01
**AC010329.1**	0.55	1.74E-01	1.00E+00	-0.63	2.87E-01	8.16E-01
**EPOP**	-1.76	1.32E-03	1.95E-01	1.05	2.87E-01	8.16E-01
**KCTD2**	0.16	3.91E-01	1.00E+00	0.47	2.87E-01	8.16E-01
**RAD18**	-0.07	7.40E-01	1.00E+00	0.31	2.87E-01	8.16E-01
**NPDC1**	-0.05	8.54E-01	1.00E+00	0.42	2.87E-01	8.16E-01
**AL132800.1**	-1.92	2.06E-01	1.00E+00	0.96	2.87E-01	8.16E-01
**PTENP1**	0.85	3.22E-01	1.00E+00	0.67	2.87E-01	8.16E-01
**G33960**	-1.02	3.43E-01	1.00E+00	-0.98	2.87E-01	8.16E-01
**RPS3AP49**	NA	NA	NA	-0.83	2.88E-01	8.16E-01
**HNRNPA1P10**	-0.25	4.25E-01	1.00E+00	-0.25	2.88E-01	8.16E-01
**AL663070.1**	0.72	4.99E-01	1.00E+00	-0.98	2.88E-01	8.16E-01
**NDRG4**	-0.64	8.80E-02	1.00E+00	0.48	2.88E-01	8.16E-01
**FANCB**	-0.66	3.66E-01	1.00E+00	-0.61	2.88E-01	8.16E-01
**ECE1**	-0.22	4.58E-01	1.00E+00	0.61	2.88E-01	8.16E-01
**OIT3**	0.05	9.81E-01	1.00E+00	1.80	2.88E-01	8.16E-01

**AF131215.5**	-0.07	8.15E-01	1.00E+00	0.91	2.88E-01	8.16E-01
**RTN3P1**	-1.03	6.83E-01	1.00E+00	-0.63	2.88E-01	8.16E-01
**PDCD5**	-0.23	3.15E-01	1.00E+00	-0.33	2.88E-01	8.16E-01
**HNRNPA1P16**	-0.12	8.90E-01	1.00E+00	1.03	2.88E-01	8.16E-01
**KDELR2**	0.11	6.13E-01	1.00E+00	-0.22	2.88E-01	8.16E-01
**SMAD4**	0.34	8.30E-02	1.00E+00	-0.27	2.88E-01	8.16E-01
**EMP1**	0.26	2.00E-01	1.00E+00	0.21	2.88E-01	8.16E-01
**MESP2**	0.07	9.23E-01	1.00E+00	-0.79	2.88E-01	8.16E-01
**CYP20A1**	-0.12	6.34E-01	1.00E+00	-0.30	2.88E-01	8.16E-01
**AL109811.2**	2.38	4.84E-01	1.00E+00	0.91	2.88E-01	8.16E-01
**MPLKIP**	0.02	9.29E-01	1.00E+00	-0.40	2.88E-01	8.16E-01
**LINC01218**	-0.15	9.66E-01	1.00E+00	-1.35	2.88E-01	8.16E-01
**ATRN**	-0.11	6.89E-01	1.00E+00	0.33	2.88E-01	8.16E-01
**CYP4F8**	0.49	8.82E-01	1.00E+00	-1.41	2.88E-01	8.16E-01
**LRRC23**	-0.26	3.56E-01	1.00E+00	0.23	2.88E-01	8.16E-01
**PHB2**	-0.16	4.84E-01	1.00E+00	0.19	2.88E-01	8.16E-01
**SAP30BP**	-0.05	7.48E-01	1.00E+00	0.22	2.88E-01	8.16E-01
**WEE2-AS1**	-0.76	1.44E-01	1.00E+00	0.69	2.88E-01	8.16E-01
**USP14**	0.15	4.22E-01	1.00E+00	-0.23	2.88E-01	8.16E-01
**CHKA**	-0.35	5.42E-01	1.00E+00	-0.83	2.88E-01	8.16E-01
**GLCE**	-0.23	4.14E-01	1.00E+00	-0.25	2.89E-01	8.16E-01
**COA4**	-0.01	9.75E-01	1.00E+00	0.18	2.89E-01	8.16E-01
**RNF214**	-0.16	4.49E-01	1.00E+00	0.26	2.89E-01	8.16E-01
**TDRP**	0.28	4.25E-01	1.00E+00	-0.65	2.89E-01	8.16E-01
**AAMDC**	0.45	1.33E-01	1.00E+00	-0.57	2.89E-01	8.16E-01
**TAF12**	0.04	8.03E-01	1.00E+00	-0.33	2.89E-01	8.16E-01
**C19orf81**	0.65	3.20E-01	1.00E+00	-1.21	2.89E-01	8.16E-01
**VEGFD**	0.29	8.16E-01	1.00E+00	-0.94	2.89E-01	8.16E-01
**ABCB4**	-0.38	1.86E-01	1.00E+00	0.53	2.89E-01	8.16E-01
**AL139424.2**	-0.11	8.76E-01	1.00E+00	-1.00	2.89E-01	8.16E-01
**MTLN**	-0.04	8.82E-01	1.00E+00	-0.37	2.89E-01	8.16E-01
**ECH1**	0.23	4.32E-01	1.00E+00	-0.36	2.89E-01	8.16E-01

**FAM104B**	-0.23	3.66E-01	1.00E+00	-0.44	2.89E-01	8.16E-01
**SRXN1**	-1.43	6.26E-02	1.00E+00	0.98	2.89E-01	8.16E-01
**CRTAP**	0.16	5.67E-01	1.00E+00	0.29	2.89E-01	8.16E-01
**LPIN1**	0.38	3.95E-01	1.00E+00	-0.50	2.89E-01	8.16E-01
**HIC2**	0.29	4.75E-01	1.00E+00	0.70	2.89E-01	8.16E-01
**RPL23**	-0.06	8.52E-01	1.00E+00	0.27	2.89E-01	8.16E-01
**PTPN11**	0.23	2.79E-01	1.00E+00	-0.23	2.89E-01	8.16E-01
**CPSF6**	-0.12	5.38E-01	1.00E+00	-0.11	2.89E-01	8.16E-01
**TENT4B**	-0.03	9.14E-01	1.00E+00	0.29	2.89E-01	8.16E-01
**OSMR**	-0.21	3.75E-01	1.00E+00	0.59	2.89E-01	8.16E-01
**CUL7**	0.09	7.54E-01	1.00E+00	0.58	2.89E-01	8.16E-01
**AL031058.1**	-0.35	5.48E-01	1.00E+00	-0.66	2.89E-01	8.16E-01
**TRIM7**	0.06	8.96E-01	1.00E+00	-0.57	2.89E-01	8.17E-01
**LINC00526**	0.33	3.58E-01	1.00E+00	-0.44	2.90E-01	8.17E-01
**AC011468.1**	-0.27	7.92E-01	1.00E+00	0.67	2.90E-01	8.17E-01
**BAZ2B**	-0.06	7.37E-01	1.00E+00	-0.27	2.90E-01	8.17E-01
**NOL4L**	-0.13	7.74E-01	1.00E+00	0.40	2.90E-01	8.17E-01
**TWSG1**	0.14	5.98E-01	1.00E+00	0.25	2.90E-01	8.17E-01
**SPIN2B**	0.25	3.60E-01	1.00E+00	-0.30	2.90E-01	8.17E-01
**METTL24**	0.39	4.64E-01	1.00E+00	0.94	2.90E-01	8.17E-01
**RPL31P2**	-0.06	9.86E-01	1.00E+00	-0.82	2.90E-01	8.17E-01
**IL10RB**	-1.28	1.07E-01	1.00E+00	0.55	2.90E-01	8.17E-01
**AL121845.4**	-0.72	8.08E-01	1.00E+00	-1.68	2.90E-01	8.17E-01
**TAF13**	0.02	9.42E-01	1.00E+00	0.42	2.90E-01	8.17E-01
**ACTR2**	-0.02	8.98E-01	1.00E+00	0.20	2.90E-01	8.17E-01
**NKX3-2**	-3.43	3.44E-02	1.00E+00	1.50	2.90E-01	8.17E-01
**AC004917.1**	2.91	3.90E-01	1.00E+00	1.29	2.90E-01	8.17E-01
**BORCS7**	0.29	3.52E-01	1.00E+00	-0.34	2.90E-01	8.17E-01
**GYG2**	3.32	1.48E-02	7.61E-01	-0.90	2.90E-01	8.17E-01
**AL451042.2**	0.74	2.80E-01	1.00E+00	0.46	2.90E-01	8.17E-01
**AC013264.1**	-0.58	8.68E-01	1.00E+00	1.71	2.90E-01	8.17E-01
**SCN7A**	0.15	7.82E-01	1.00E+00	-0.51	2.91E-01	8.18E-01

**ELMOD2**	0.26	3.75E-01	1.00E+00	-0.32	2.91E-01	8.18E-01
**PSPHP1**	-0.48	8.67E-01	1.00E+00	3.32	2.91E-01	8.18E-01
**AC078777.1**	-0.90	4.43E-01	1.00E+00	0.98	2.91E-01	8.18E-01
**AC119396.1**	-0.83	5.68E-01	1.00E+00	1.61	2.91E-01	8.18E-01
**LINC01060**	-0.75	7.14E-01	1.00E+00	2.24	2.91E-01	8.18E-01
**PRKCA**	-0.77	1.71E-02	8.25E-01	0.44	2.91E-01	8.18E-01
**AC016907.2**	2.13	2.17E-01	1.00E+00	-1.07	2.91E-01	8.18E-01
**GEN1**	-0.19	5.69E-01	1.00E+00	0.51	2.91E-01	8.18E-01
**LINC00189**	-2.28	2.03E-01	1.00E+00	-0.71	2.91E-01	8.18E-01
**EEF1A1**	0.03	9.07E-01	1.00E+00	0.34	2.91E-01	8.18E-01
**LINC00565**	-1.60	1.76E-01	1.00E+00	1.47	2.91E-01	8.18E-01
**AC007036.2**	-0.19	9.12E-01	1.00E+00	1.20	2.91E-01	8.18E-01
**DNAAF4**	-1.39	5.40E-01	1.00E+00	0.89	2.91E-01	8.18E-01
**FAM66C**	0.13	7.76E-01	1.00E+00	-0.73	2.91E-01	8.18E-01
**AC068620.3**	0.19	9.05E-01	1.00E+00	-1.06	2.91E-01	8.18E-01
**SUPT20H**	0.18	4.91E-01	1.00E+00	0.20	2.91E-01	8.18E-01
**STPG4**	-2.21	1.08E-01	1.00E+00	1.06	2.91E-01	8.18E-01
**PARP10**	-0.20	6.48E-01	1.00E+00	0.89	2.91E-01	8.18E-01
**CA2**	-0.14	8.11E-01	1.00E+00	-0.46	2.91E-01	8.18E-01
**MRPL21**	0.01	9.41E-01	1.00E+00	-0.30	2.91E-01	8.18E-01
**CHI3L1**	-0.42	4.92E-01	1.00E+00	0.60	2.91E-01	8.18E-01
**IMMP2L**	-0.04	8.84E-01	1.00E+00	-0.39	2.91E-01	8.18E-01
**ELP6**	0.03	9.08E-01	1.00E+00	-0.23	2.91E-01	8.18E-01
**CLDN8**	0.19	5.78E-01	1.00E+00	-0.65	2.92E-01	8.18E-01
**ZFP30**	0.23	5.91E-01	1.00E+00	-0.50	2.92E-01	8.18E-01
**AC092287.1**	-1.46	2.33E-01	1.00E+00	-0.71	2.92E-01	8.18E-01
**MRM1**	0.52	1.31E-01	1.00E+00	-0.50	2.92E-01	8.18E-01
**NIM1K**	-0.66	1.04E-01	1.00E+00	0.70	2.92E-01	8.18E-01
**TRAV23DV6**	-0.19	9.56E-01	1.00E+00	1.54	2.92E-01	8.19E-01
**LINC00865**	0.39	6.97E-01	1.00E+00	0.91	2.92E-01	8.19E-01
**RAD1**	0.34	1.18E-01	1.00E+00	-0.28	2.92E-01	8.19E-01
**ATP5PB**	-0.04	8.25E-01	1.00E+00	-0.25	2.92E-01	8.19E-01

**ATE1-AS1**	-0.96	3.59E-01	1.00E+00	-1.00	2.92E-01	8.19E-01
**SFXN4**	-0.04	8.85E-01	1.00E+00	-0.26	2.92E-01	8.19E-01
**RAP2C**	0.02	9.28E-01	1.00E+00	0.21	2.92E-01	8.19E-01
**RCC1**	-0.53	4.54E-02	1.00E+00	0.37	2.92E-01	8.19E-01
**PI4KAP2**	0.40	4.20E-01	1.00E+00	0.83	2.92E-01	8.19E-01
**AC055764.2**	-1.12	6.30E-01	1.00E+00	-0.97	2.92E-01	8.19E-01
**TMEM205**	-0.31	2.54E-01	1.00E+00	-0.49	2.92E-01	8.19E-01
**ZSCAN18**	0.47	9.83E-02	1.00E+00	-0.66	2.92E-01	8.19E-01
**DTNA**	0.46	4.17E-01	1.00E+00	0.50	2.92E-01	8.19E-01
**PARD6G-AS1**	-0.57	1.39E-01	1.00E+00	-0.54	2.92E-01	8.19E-01
**TRAC**	-0.09	8.71E-01	1.00E+00	0.40	2.92E-01	8.19E-01
**RABL2A**	-0.18	5.24E-01	1.00E+00	-0.27	2.92E-01	8.19E-01
**RNF144A**	0.21	5.23E-01	1.00E+00	0.53	2.92E-01	8.19E-01
**AC092953.2**	1.16	1.36E-01	1.00E+00	0.67	2.92E-01	8.19E-01
**DHRS13**	-0.21	3.99E-01	1.00E+00	-0.48	2.92E-01	8.19E-01
**DUSP28**	0.01	9.73E-01	1.00E+00	-0.33	2.92E-01	8.19E-01
**SLIT2**	0.29	5.78E-01	1.00E+00	0.56	2.92E-01	8.19E-01
**AC108010.1**	-1.11	3.25E-02	1.00E+00	1.00	2.93E-01	8.19E-01
**CELF5**	-1.32	9.36E-02	1.00E+00	1.11	2.93E-01	8.19E-01
**XLOC_007686**	0.61	2.66E-01	1.00E+00	1.12	2.93E-01	8.19E-01
**KIAA1958**	-0.34	3.74E-01	1.00E+00	-0.41	2.93E-01	8.19E-01
**SSB**	-0.06	8.21E-01	1.00E+00	-0.23	2.93E-01	8.19E-01
**RAB9B**	-0.11	8.21E-01	1.00E+00	-0.56	2.93E-01	8.19E-01
**METAP2**	0.05	7.43E-01	1.00E+00	0.17	2.93E-01	8.19E-01
**RPS28**	-0.11	6.96E-01	1.00E+00	0.40	2.93E-01	8.19E-01
**FTLP2**	NA	NA	NA	-0.87	2.93E-01	8.19E-01
**TTR**	-3.99	6.04E-03	4.62E-01	1.08	2.93E-01	8.19E-01
**SERHL2**	0.44	4.81E-01	1.00E+00	-0.54	2.93E-01	8.19E-01
**TG**	-0.14	8.64E-01	1.00E+00	-0.82	2.93E-01	8.19E-01
**XLOC_000972**	-0.27	8.04E-01	1.00E+00	1.34	2.93E-01	8.19E-01
**UCN**	0.47	7.44E-01	1.00E+00	1.34	2.93E-01	8.19E-01
**PEG10**	0.00	9.95E-01	1.00E+00	0.57	2.93E-01	8.19E-01

**FGF12**	-0.20	8.22E-01	1.00E+00	-0.56	2.93E-01	8.19E-01
**ACE**	0.02	9.69E-01	1.00E+00	0.78	2.93E-01	8.19E-01
**MOSPD3**	0.12	6.98E-01	1.00E+00	-0.38	2.93E-01	8.19E-01
**GGT7**	0.13	5.32E-01	1.00E+00	0.42	2.93E-01	8.19E-01
**DYNLRB1**	-0.01	9.28E-01	1.00E+00	-0.17	2.93E-01	8.19E-01
**LINC01614**	0.90	7.96E-01	1.00E+00	1.96	2.93E-01	8.20E-01
**CFAP53**	-0.20	7.57E-01	1.00E+00	0.56	2.93E-01	8.20E-01
**Z82206.1**	-1.10	7.53E-01	1.00E+00	-0.86	2.94E-01	8.20E-01
**AC000124.1**	0.50	8.32E-01	1.00E+00	-2.48	2.94E-01	8.20E-01
**ICAM1**	-1.01	5.77E-02	1.00E+00	0.77	2.94E-01	8.20E-01
**MAP9**	0.41	4.44E-01	1.00E+00	0.40	2.94E-01	8.20E-01
**ALKBH2**	-0.13	4.64E-01	1.00E+00	-0.20	2.94E-01	8.21E-01
**AC132872.2**	-1.09	7.06E-01	1.00E+00	1.05	2.94E-01	8.21E-01
**AC012358.3**	-3.11	5.42E-02	1.00E+00	-0.67	2.94E-01	8.21E-01
**SYT13**	-1.94	7.25E-02	1.00E+00	0.97	2.94E-01	8.21E-01
**RPL17P50**	0.86	2.64E-01	1.00E+00	0.45	2.94E-01	8.21E-01
**SRPRB**	0.10	6.46E-01	1.00E+00	-0.18	2.94E-01	8.21E-01
**G22228**	0.55	5.47E-01	1.00E+00	1.24	2.94E-01	8.21E-01
**CSNK1A1**	-0.14	6.40E-01	1.00E+00	0.22	2.94E-01	8.21E-01
**AC090425.1**	-0.51	7.00E-01	1.00E+00	-0.64	2.94E-01	8.21E-01
**RBX1**	0.36	7.29E-02	1.00E+00	-0.35	2.94E-01	8.21E-01
**RMDN3**	0.11	6.86E-01	1.00E+00	-0.23	2.94E-01	8.21E-01
**TGFA**	0.18	6.83E-01	1.00E+00	0.49	2.95E-01	8.21E-01
**TOP1**	0.10	5.87E-01	1.00E+00	0.38	2.95E-01	8.21E-01
**SLC1A7**	1.00	7.21E-02	1.00E+00	0.77	2.95E-01	8.21E-01
**DNAAF1**	-1.14	3.34E-01	1.00E+00	-1.16	2.95E-01	8.21E-01
**C21orf62**	-1.63	4.03E-01	1.00E+00	-1.44	2.95E-01	8.21E-01
**AL353194.1**	0.84	2.92E-01	1.00E+00	-0.53	2.95E-01	8.21E-01
**C12orf65**	0.15	5.17E-01	1.00E+00	-0.21	2.95E-01	8.21E-01
**ATP8B3**	0.37	5.86E-01	1.00E+00	0.89	2.95E-01	8.21E-01
**LYPD3**	-0.32	5.47E-01	1.00E+00	0.62	2.95E-01	8.21E-01
**ZNF462**	-0.05	8.59E-01	1.00E+00	0.33	2.95E-01	8.21E-01

**GABRA4**	-1.95	1.34E-01	1.00E+00	-2.94	2.95E-01	8.21E-01
**CCDC34**	-0.04	8.51E-01	1.00E+00	-0.29	2.95E-01	8.21E-01
**TMSB4XP2**	NA	NA	NA	-0.78	2.95E-01	8.21E-01
**CCDC150**	0.73	4.43E-01	1.00E+00	0.68	2.95E-01	8.21E-01
**ARHGAP25**	0.17	6.46E-01	1.00E+00	0.63	2.95E-01	8.21E-01
**ZNF354B**	-0.32	3.37E-01	1.00E+00	-0.37	2.95E-01	8.21E-01
**DNAJC27**	0.26	4.78E-01	1.00E+00	-0.29	2.95E-01	8.21E-01
**TLE1P1**	0.83	6.25E-01	1.00E+00	-0.70	2.95E-01	8.21E-01
**ZNF252P-AS1**	2.60	1.53E-01	1.00E+00	1.26	2.95E-01	8.21E-01
**SLC43A1**	-0.02	9.60E-01	1.00E+00	0.41	2.95E-01	8.21E-01
**CKAP4**	-0.02	9.15E-01	1.00E+00	0.50	2.95E-01	8.21E-01
**AC079467.1**	0.97	2.98E-01	1.00E+00	1.55	2.95E-01	8.21E-01
**VPS33A**	0.05	8.32E-01	1.00E+00	0.21	2.95E-01	8.21E-01
**RPL41**	0.08	7.71E-01	1.00E+00	0.34	2.95E-01	8.21E-01
**DHRS7B**	-0.15	4.18E-01	1.00E+00	-0.29	2.95E-01	8.21E-01
**FNDC10**	-0.36	2.57E-01	1.00E+00	-0.66	2.95E-01	8.21E-01
**ABHD17AP6**	-1.33	4.43E-01	1.00E+00	-1.16	2.96E-01	8.21E-01
**CSRP2**	0.21	4.93E-01	1.00E+00	0.39	2.96E-01	8.21E-01
**ADAM1B**	1.42	1.29E-01	1.00E+00	-1.35	2.96E-01	8.21E-01
**AP001434.1**	0.44	8.99E-01	1.00E+00	1.60	2.96E-01	8.21E-01
**SNX25**	0.09	8.10E-01	1.00E+00	-0.53	2.96E-01	8.21E-01
**EIF4EBP2**	0.39	3.44E-02	1.00E+00	-0.25	2.96E-01	8.21E-01
**RP11-566K19.6**	-0.39	8.29E-01	1.00E+00	-1.11	2.96E-01	8.21E-01
**AFMID**	-0.30	1.60E-01	1.00E+00	0.25	2.96E-01	8.21E-01
**GYG1**	-0.25	1.90E-01	1.00E+00	0.20	2.96E-01	8.21E-01
**ARMCX4**	-0.15	6.03E-01	1.00E+00	0.50	2.96E-01	8.21E-01
**AL139128.1**	30.00	6.57E-18	5.54E-15	1.57	2.96E-01	8.21E-01
**RP6-42F4.1**	0.19	6.73E-01	1.00E+00	-0.37	2.96E-01	8.21E-01
**BBOX1-AS1**	0.93	5.19E-01	1.00E+00	0.67	2.96E-01	8.21E-01
**XLOC_003471**	-0.18	7.42E-01	1.00E+00	-0.54	2.96E-01	8.22E-01
**DNAH7**	-0.69	3.86E-01	1.00E+00	-0.97	2.96E-01	8.22E-01
**AAR2**	-0.03	9.02E-01	1.00E+00	0.16	2.96E-01	8.22E-01

**FLJ37453**	-0.07	7.86E-01	1.00E+00	-0.34	2.96E-01	8.22E-01
**TRIM16**	-0.22	4.39E-01	1.00E+00	0.25	2.96E-01	8.22E-01
**ZNF335**	-0.33	2.32E-01	1.00E+00	0.50	2.96E-01	8.22E-01
**LMX1B**	-0.39	4.11E-01	1.00E+00	0.72	2.96E-01	8.22E-01
**ZNF572**	0.05	9.49E-01	1.00E+00	-0.95	2.96E-01	8.22E-01
**AC024560.2**	-1.63	2.16E-01	1.00E+00	-1.45	2.96E-01	8.22E-01
**RELA**	-0.23	2.30E-01	1.00E+00	0.30	2.96E-01	8.22E-01
**GPX1**	0.29	5.14E-01	1.00E+00	0.41	2.96E-01	8.22E-01
**ZNF614**	-0.01	9.56E-01	1.00E+00	-0.21	2.96E-01	8.22E-01
**STMP1**	0.21	2.30E-01	1.00E+00	-0.35	2.97E-01	8.22E-01
**ZNF788**	0.54	2.16E-01	1.00E+00	-0.57	2.97E-01	8.22E-01
**RPL12**	-0.22	4.41E-01	1.00E+00	0.28	2.97E-01	8.22E-01
**SYMPK**	-0.35	1.37E-01	1.00E+00	0.68	2.97E-01	8.22E-01
**AC012313.4**	0.79	3.63E-01	1.00E+00	-0.87	2.97E-01	8.22E-01
**NRSN2-AS1**	-0.29	3.76E-01	1.00E+00	-0.35	2.97E-01	8.22E-01
**HEMGN**	0.56	8.71E-01	1.00E+00	-1.66	2.97E-01	8.22E-01
**NUDT15**	0.05	8.53E-01	1.00E+00	-0.21	2.97E-01	8.22E-01
**ADI1**	0.06	7.98E-01	1.00E+00	-0.31	2.97E-01	8.22E-01
**PCNP**	0.01	9.49E-01	1.00E+00	-0.26	2.97E-01	8.22E-01
**GAS1RR**	-0.23	6.18E-01	1.00E+00	-0.37	2.97E-01	8.22E-01
**KAT2B**	0.20	3.58E-01	1.00E+00	0.32	2.97E-01	8.22E-01
**SLC25A37**	-0.04	8.92E-01	1.00E+00	0.28	2.97E-01	8.22E-01
**GOLGA7**	0.20	3.67E-01	1.00E+00	-0.34	2.97E-01	8.22E-01
**RP6-206I17.1**	-0.18	6.80E-01	1.00E+00	0.29	2.97E-01	8.22E-01
**KIT**	0.45	3.97E-01	1.00E+00	-0.43	2.97E-01	8.22E-01
**SERBP1**	-0.09	6.86E-01	1.00E+00	0.16	2.97E-01	8.22E-01
**MAP1A**	-0.18	7.59E-01	1.00E+00	0.56	2.97E-01	8.22E-01
**S100A10**	0.10	6.54E-01	1.00E+00	-0.25	2.97E-01	8.22E-01
**NPHP1**	-0.14	6.95E-01	1.00E+00	-0.37	2.97E-01	8.22E-01
**CHCHD2P6**	0.82	1.69E-01	1.00E+00	0.60	2.97E-01	8.22E-01
**RNF149**	0.00	9.91E-01	1.00E+00	0.24	2.97E-01	8.22E-01
**AC002401.4**	NA	NA	NA	1.49	2.97E-01	8.22E-01

**CCDC12**	-0.06	8.16E-01	1.00E+00	0.26	2.97E-01	8.22E-01
**AL136979.1**	0.97	3.81E-01	1.00E+00	1.15	2.97E-01	8.22E-01
**NBAT1**	-1.38	6.12E-01	1.00E+00	1.31	2.97E-01	8.22E-01
**TNFAIP8**	0.34	2.81E-01	1.00E+00	-0.28	2.97E-01	8.22E-01
**AC026691.1**	0.35	5.49E-01	1.00E+00	0.92	2.97E-01	8.22E-01
**G27759**	-1.69	6.99E-03	5.09E-01	0.67	2.98E-01	8.22E-01
**SIGLEC22P**	0.38	7.88E-01	1.00E+00	1.42	2.98E-01	8.22E-01
**FBXL12**	-0.02	9.39E-01	1.00E+00	0.24	2.98E-01	8.22E-01
**AL590627.1**	-1.86	4.71E-01	1.00E+00	-1.04	2.98E-01	8.22E-01
**POLR2B**	0.20	3.51E-01	1.00E+00	0.17	2.98E-01	8.22E-01
**AF213884.3**	-2.87	2.65E-02	9.49E-01	0.94	2.98E-01	8.22E-01
**AL139174.1**	3.43	3.09E-01	1.00E+00	-1.14	2.98E-01	8.22E-01
**ZNF510**	0.29	4.23E-01	1.00E+00	0.38	2.98E-01	8.22E-01
**ABCC10**	0.01	9.65E-01	1.00E+00	0.61	2.98E-01	8.22E-01
**AC005237.1**	0.88	3.27E-01	1.00E+00	-1.24	2.98E-01	8.22E-01
**LINC01534**	-0.07	9.30E-01	1.00E+00	-0.71	2.98E-01	8.22E-01
**DRP2**	-0.21	8.32E-01	1.00E+00	-1.55	2.98E-01	8.22E-01
**FBLN2**	0.57	2.31E-01	1.00E+00	0.64	2.98E-01	8.22E-01
**RPL10**	-0.13	6.12E-01	1.00E+00	0.24	2.98E-01	8.22E-01
**RPL31P11**	0.43	9.03E-01	1.00E+00	-1.05	2.98E-01	8.22E-01
**RHOA**	0.07	7.19E-01	1.00E+00	-0.21	2.98E-01	8.22E-01
**KAT6B**	-0.05	8.46E-01	1.00E+00	-0.25	2.98E-01	8.22E-01
**MRPL34**	-0.13	4.39E-01	1.00E+00	-0.27	2.98E-01	8.22E-01
**ALG11**	-0.03	9.06E-01	1.00E+00	-0.27	2.98E-01	8.22E-01
**CNN2**	-0.18	3.06E-01	1.00E+00	0.33	2.98E-01	8.22E-01
**GSDMA**	-0.09	8.92E-01	1.00E+00	0.57	2.98E-01	8.22E-01
**STAM2**	-0.02	9.39E-01	1.00E+00	-0.15	2.98E-01	8.22E-01
**SELENOM**	-0.10	7.97E-01	1.00E+00	0.35	2.98E-01	8.22E-01
**AC011498.1**	0.91	4.93E-01	1.00E+00	-1.03	2.98E-01	8.22E-01
**ZNF503-AS2**	-0.24	5.67E-01	1.00E+00	0.42	2.98E-01	8.22E-01
**HSD17B1P1**	-0.45	3.78E-01	1.00E+00	1.16	2.98E-01	8.22E-01
**AXIN1**	-0.26	3.34E-01	1.00E+00	0.58	2.98E-01	8.22E-01

**RHEB**	0.27	1.54E-01	1.00E+00	-0.28	2.98E-01	8.22E-01
**MTF1**	0.15	5.17E-01	1.00E+00	0.20	2.99E-01	8.22E-01
**GABARAP**	-3.12	1.60E-01	1.00E+00	0.61	2.99E-01	8.22E-01
**NAIP**	-0.38	4.94E-01	1.00E+00	0.93	2.99E-01	8.22E-01
**AP000688.1**	0.34	7.25E-01	1.00E+00	-0.72	2.99E-01	8.22E-01
**CCNG2**	0.59	6.65E-02	1.00E+00	-0.43	2.99E-01	8.22E-01
**AC116667.2**	1.09	6.29E-01	1.00E+00	1.57	2.99E-01	8.22E-01
**HLA-DOB**	-0.66	1.42E-01	1.00E+00	0.53	2.99E-01	8.22E-01
**CCDC162P**	1.23	2.92E-01	1.00E+00	-0.84	2.99E-01	8.22E-01
**CCDC87**	-0.58	5.94E-01	1.00E+00	-1.35	2.99E-01	8.22E-01
**ZNF530**	0.39	1.42E-01	1.00E+00	-0.31	2.99E-01	8.22E-01
**SEMA5A**	-0.04	9.16E-01	1.00E+00	0.32	2.99E-01	8.22E-01
**HGSNAT**	0.09	7.50E-01	1.00E+00	-0.30	2.99E-01	8.22E-01
**AC254633.1**	0.44	4.65E-01	1.00E+00	0.81	2.99E-01	8.22E-01
**Metazoa_SRP**	-3.37	1.08E-01	1.00E+00	-1.12	2.99E-01	8.22E-01
**UBP1**	0.06	7.84E-01	1.00E+00	0.45	2.99E-01	8.22E-01
**RHEBL1**	0.46	1.28E-01	1.00E+00	0.46	2.99E-01	8.22E-01
**HCFC1**	-0.39	1.55E-01	1.00E+00	0.57	2.99E-01	8.22E-01
**FRS2**	-0.15	4.51E-01	1.00E+00	0.17	2.99E-01	8.22E-01
**LRRC40**	0.24	3.90E-01	1.00E+00	-0.26	2.99E-01	8.22E-01
**NPM1P6**	0.05	9.60E-01	1.00E+00	-0.50	2.99E-01	8.22E-01
**ARV1**	-0.13	5.55E-01	1.00E+00	-0.36	2.99E-01	8.22E-01
**AC041040.1**	0.85	4.44E-01	1.00E+00	1.50	2.99E-01	8.22E-01
**RNU7-163P**	0.12	9.71E-01	1.00E+00	1.12	2.99E-01	8.22E-01
**AC005520.2**	0.61	5.98E-02	1.00E+00	-0.60	2.99E-01	8.22E-01
**G25680**	-1.69	3.42E-01	1.00E+00	1.22	2.99E-01	8.22E-01
**AC037459.2**	0.29	5.90E-01	1.00E+00	-0.67	2.99E-01	8.22E-01
**XPO4**	-0.07	7.79E-01	1.00E+00	-0.30	2.99E-01	8.22E-01
**SLC6A11**	-0.51	2.82E-01	1.00E+00	0.65	2.99E-01	8.22E-01
**FKBP9**	0.23	4.28E-01	1.00E+00	0.31	2.99E-01	8.22E-01
**HIST4H4**	0.01	9.83E-01	1.00E+00	0.53	3.00E-01	8.22E-01
**ATP5PD**	0.13	5.07E-01	1.00E+00	-0.37	3.00E-01	8.22E-01

**AL160313.1**	-0.44	6.64E-01	1.00E+00	-0.61	3.00E-01	8.22E-01
**ELMO3**	-0.37	3.05E-01	1.00E+00	0.46	3.00E-01	8.22E-01
**FBXW4**	0.03	8.90E-01	1.00E+00	0.48	3.00E-01	8.22E-01
**GCC2-AS1**	-1.48	1.77E-01	1.00E+00	0.51	3.00E-01	8.22E-01
**VAPA**	0.06	7.54E-01	1.00E+00	-0.29	3.00E-01	8.22E-01
**CFAP58-DT**	-0.41	3.88E-01	1.00E+00	-0.51	3.00E-01	8.22E-01
**RN7SL749P**	0.36	9.17E-01	1.00E+00	1.26	3.00E-01	8.22E-01
**SLC39A13**	-0.07	7.84E-01	1.00E+00	0.32	3.00E-01	8.22E-01
**AC112236.2**	-0.02	9.84E-01	1.00E+00	0.78	3.00E-01	8.22E-01
**ZNF813**	0.32	2.06E-01	1.00E+00	0.34	3.00E-01	8.22E-01
**PSMC1P1**	-0.01	9.87E-01	1.00E+00	-0.31	3.00E-01	8.22E-01
**ARMC12**	0.80	4.80E-01	1.00E+00	1.13	3.00E-01	8.22E-01
**GDF3**	-3.47	1.73E-01	1.00E+00	1.40	3.00E-01	8.22E-01
**PDZD4**	-0.80	3.93E-02	1.00E+00	0.80	3.00E-01	8.22E-01
**CTD-3222D19.7**	-0.48	6.60E-01	1.00E+00	0.80	3.00E-01	8.22E-01
**PCDHGA6**	-0.14	6.71E-01	1.00E+00	-0.43	3.00E-01	8.22E-01
**TMEM74**	0.05	9.34E-01	1.00E+00	-0.58	3.00E-01	8.22E-01
**ZIC2**	-0.92	2.43E-01	1.00E+00	1.23	3.00E-01	8.22E-01
**MCU**	0.26	4.45E-01	1.00E+00	-0.26	3.00E-01	8.22E-01
**CLSTN2**	0.23	6.74E-01	1.00E+00	-0.69	3.00E-01	8.22E-01
**AL391834.1**	-0.14	9.33E-01	1.00E+00	0.84	3.00E-01	8.22E-01
**PAQR5**	-0.08	7.38E-01	1.00E+00	0.40	3.00E-01	8.22E-01
**ETFDH**	0.27	2.02E-01	1.00E+00	-0.29	3.00E-01	8.22E-01
**EDNRA**	0.09	8.35E-01	1.00E+00	0.40	3.00E-01	8.22E-01
**AGAP4**	0.11	9.19E-01	1.00E+00	-0.65	3.01E-01	8.22E-01
**NDUFA6-DT**	0.22	5.57E-01	1.00E+00	0.52	3.01E-01	8.22E-01
**NEDD4**	0.33	2.72E-01	1.00E+00	0.31	3.01E-01	8.22E-01
**PIAS4**	-0.03	8.93E-01	1.00E+00	0.36	3.01E-01	8.22E-01
**RGS22**	0.60	1.19E-01	1.00E+00	-0.76	3.01E-01	8.22E-01
**TTC21A**	-0.66	3.99E-01	1.00E+00	0.75	3.01E-01	8.22E-01
**TBC1D1**	-0.06	8.70E-01	1.00E+00	0.56	3.01E-01	8.23E-01
**PSMA5**	-0.03	8.47E-01	1.00E+00	0.18	3.01E-01	8.23E-01

**AC005839.1**	0.58	3.74E-01	1.00E+00	0.52	3.01E-01	8.23E-01
**SH3BGRL3**	-0.28	3.11E-01	1.00E+00	0.30	3.01E-01	8.23E-01
**ZNF131**	-0.10	5.67E-01	1.00E+00	-0.18	3.01E-01	8.23E-01
**VTI1A**	-0.08	6.02E-01	1.00E+00	-0.17	3.01E-01	8.23E-01
**AC009831.1**	0.32	7.18E-01	1.00E+00	0.67	3.01E-01	8.23E-01
**MCMBP**	-0.13	5.79E-01	1.00E+00	-0.47	3.01E-01	8.23E-01
**AC092168.2**	-2.06	4.77E-01	1.00E+00	1.99	3.01E-01	8.23E-01
**AC104438.1**	0.90	7.01E-01	1.00E+00	-1.20	3.01E-01	8.23E-01
**LMBR1L**	0.14	6.91E-01	1.00E+00	0.32	3.01E-01	8.23E-01
**AL354811.1**	0.44	8.99E-01	1.00E+00	1.10	3.01E-01	8.23E-01
**BSDC1**	0.00	9.90E-01	1.00E+00	0.22	3.01E-01	8.23E-01
**OR7E7P**	0.37	7.24E-01	1.00E+00	-1.20	3.01E-01	8.23E-01
**FAM86GP**	0.38	6.62E-01	1.00E+00	0.86	3.01E-01	8.23E-01
**PSME2P2**	0.56	8.71E-01	1.00E+00	0.72	3.01E-01	8.23E-01
**MAPRE3**	0.14	6.37E-01	1.00E+00	-0.27	3.01E-01	8.23E-01
**ZSWIM3**	0.63	1.52E-01	1.00E+00	0.55	3.01E-01	8.23E-01
**RBM25**	-0.03	8.72E-01	1.00E+00	0.23	3.01E-01	8.23E-01
**AC015912.3**	-0.13	8.94E-01	1.00E+00	0.50	3.01E-01	8.23E-01
**FAM189B**	-0.27	3.05E-01	1.00E+00	0.69	3.02E-01	8.23E-01
**ANKRD49**	0.04	8.35E-01	1.00E+00	-0.40	3.02E-01	8.23E-01
**AC009686.1**	0.56	8.71E-01	1.00E+00	1.21	3.02E-01	8.23E-01
**MRGPRX3**	-0.18	9.37E-01	1.00E+00	2.11	3.02E-01	8.23E-01
**AC135983.3**	0.63	8.23E-01	1.00E+00	1.00	3.02E-01	8.23E-01
**MED17**	0.50	2.77E-01	1.00E+00	0.36	3.02E-01	8.23E-01
**PCED1B**	-0.26	5.80E-01	1.00E+00	0.48	3.02E-01	8.23E-01
**FRG1JP**	0.03	9.47E-01	1.00E+00	-0.33	3.02E-01	8.23E-01
**CNTF**	-0.76	3.28E-01	1.00E+00	0.74	3.02E-01	8.23E-01
**XLOC_002554**	0.02	9.65E-01	1.00E+00	0.81	3.02E-01	8.23E-01
**AL135905.1**	0.38	5.85E-01	1.00E+00	-0.75	3.02E-01	8.23E-01
**AL157827.2**	-1.31	2.72E-01	1.00E+00	1.04	3.02E-01	8.23E-01
**PANO1**	0.63	5.02E-01	1.00E+00	1.14	3.02E-01	8.23E-01
**AP1B1**	-0.09	7.15E-01	1.00E+00	0.44	3.02E-01	8.23E-01

**WASH5P**	0.04	9.64E-01	1.00E+00	0.48	3.02E-01	8.23E-01
**ZFAND4**	-0.06	8.74E-01	1.00E+00	-0.45	3.02E-01	8.23E-01
**PCDHGB8P**	-1.73	2.73E-01	1.00E+00	-1.22	3.02E-01	8.23E-01
**GIMAP4**	0.46	4.17E-01	1.00E+00	0.45	3.02E-01	8.23E-01
**SLED1**	0.98	1.88E-01	1.00E+00	0.98	3.02E-01	8.23E-01
**LINC02289**	0.01	9.94E-01	1.00E+00	-0.81	3.02E-01	8.23E-01
**GRIN2C**	-0.33	6.42E-01	1.00E+00	0.83	3.02E-01	8.23E-01
**C12orf45**	0.08	7.24E-01	1.00E+00	-0.36	3.02E-01	8.23E-01
**EIF4G2**	-0.07	7.67E-01	1.00E+00	0.19	3.02E-01	8.23E-01
**AP003548.1**	2.02	1.78E-01	1.00E+00	-0.84	3.02E-01	8.23E-01
**GIMAP8**	0.59	2.12E-01	1.00E+00	0.54	3.02E-01	8.23E-01
**AL590617.1**	-1.25	7.16E-01	1.00E+00	-1.78	3.02E-01	8.23E-01
**C20orf96**	-0.27	5.57E-01	1.00E+00	0.35	3.02E-01	8.23E-01
**EGFL6**	0.89	4.65E-01	1.00E+00	0.79	3.02E-01	8.23E-01
**PRR16**	0.67	2.91E-01	1.00E+00	0.70	3.03E-01	8.23E-01
**LINC01270**	-1.32	4.75E-01	1.00E+00	-1.11	3.03E-01	8.23E-01
**RSF1**	0.06	7.20E-01	1.00E+00	0.20	3.03E-01	8.23E-01
**C12orf10**	-0.17	4.77E-01	1.00E+00	0.25	3.03E-01	8.23E-01
**PELP1**	-0.05	7.86E-01	1.00E+00	0.53	3.03E-01	8.23E-01
**MAF**	-0.19	6.23E-01	1.00E+00	0.35	3.03E-01	8.23E-01
**AC233723.1**	-1.92	2.08E-02	8.94E-01	-0.56	3.03E-01	8.23E-01
**AC009163.6**	-0.25	6.43E-01	1.00E+00	-0.75	3.03E-01	8.23E-01
**ZNF184**	0.24	4.10E-01	1.00E+00	-0.47	3.03E-01	8.23E-01
**AL445363.3**	2.30	4.29E-02	1.00E+00	-0.39	3.03E-01	8.23E-01
**LINC01697**	1.38	2.37E-01	1.00E+00	-1.01	3.03E-01	8.23E-01
**LINC00462**	-0.32	7.56E-01	1.00E+00	0.86	3.03E-01	8.23E-01
**HAUS3**	0.30	3.90E-01	1.00E+00	-0.25	3.03E-01	8.23E-01
**LINC01612**	3.14	2.11E-01	1.00E+00	-1.16	3.03E-01	8.23E-01
**NAGPA**	-0.17	4.51E-01	1.00E+00	0.38	3.03E-01	8.23E-01
**AC084824.5**	-0.43	7.86E-01	1.00E+00	-0.90	3.03E-01	8.23E-01
**ALKBH5**	0.07	6.91E-01	1.00E+00	-0.36	3.03E-01	8.23E-01
**CRHBP**	-0.16	8.75E-01	1.00E+00	-0.93	3.03E-01	8.23E-01

**ZNF385B**	0.32	4.72E-01	1.00E+00	0.65	3.03E-01	8.23E-01
**MACC1**	0.19	6.11E-01	1.00E+00	-0.59	3.03E-01	8.23E-01
**C11orf58**	0.10	5.83E-01	1.00E+00	-0.28	3.03E-01	8.23E-01
**RN7SL473P**	1.86	1.85E-01	1.00E+00	0.83	3.03E-01	8.24E-01
**SKP1**	0.19	1.69E-01	1.00E+00	-0.44	3.04E-01	8.24E-01
**G29778**	0.03	9.68E-01	1.00E+00	-0.72	3.04E-01	8.24E-01
**ZNF512B**	-0.34	2.28E-01	1.00E+00	0.88	3.04E-01	8.24E-01
**SAMD15**	-1.78	1.78E-02	8.38E-01	0.90	3.04E-01	8.24E-01
**STAB2**	-0.61	5.34E-01	1.00E+00	1.19	3.04E-01	8.24E-01
**IGFBP6**	0.77	2.74E-01	1.00E+00	0.59	3.04E-01	8.24E-01
**RPL13AP25**	-0.08	8.35E-01	1.00E+00	-0.58	3.04E-01	8.24E-01
**BRPF3**	-0.33	9.44E-02	1.00E+00	0.58	3.04E-01	8.24E-01
**G12855**	1.55	3.53E-01	1.00E+00	1.49	3.04E-01	8.24E-01
**SUCLA2-AS1**	-0.33	7.47E-01	1.00E+00	-0.49	3.04E-01	8.24E-01
**6-Sep**	0.01	9.74E-01	1.00E+00	0.38	3.04E-01	8.24E-01
**AC005076.1**	0.88	3.24E-01	1.00E+00	-0.67	3.04E-01	8.24E-01
**G1863**	-0.49	6.99E-01	1.00E+00	1.26	3.04E-01	8.24E-01
**IRF2BP1**	-0.15	5.03E-01	1.00E+00	0.64	3.04E-01	8.24E-01
**OAF**	0.29	6.12E-01	1.00E+00	0.76	3.04E-01	8.24E-01
**SMG1P2**	-0.18	7.48E-01	1.00E+00	-0.51	3.04E-01	8.24E-01
**PLCD3**	-0.02	9.41E-01	1.00E+00	0.48	3.04E-01	8.24E-01
**MAP3K7CL**	0.22	6.43E-01	1.00E+00	0.34	3.04E-01	8.24E-01
**AL137013.1**	3.52	6.75E-02	1.00E+00	-1.00	3.04E-01	8.24E-01
**UBN1**	-0.08	6.34E-01	1.00E+00	0.43	3.04E-01	8.24E-01
**C17orf64**	-1.70	5.55E-01	1.00E+00	1.36	3.04E-01	8.24E-01
**TBC1D2B**	0.29	2.62E-01	1.00E+00	0.56	3.04E-01	8.24E-01
**AC068389.3**	3.21	3.42E-01	1.00E+00	0.98	3.04E-01	8.24E-01
**CELSR3**	-1.26	1.66E-03	2.25E-01	1.16	3.04E-01	8.24E-01
**XLOC_003261**	1.02	3.01E-01	1.00E+00	-0.96	3.04E-01	8.24E-01
**SALL3**	-1.24	4.22E-01	1.00E+00	-1.97	3.05E-01	8.24E-01
**PATZ1**	0.11	7.04E-01	1.00E+00	0.53	3.05E-01	8.24E-01
**XLOC_012011**	-0.52	6.45E-01	1.00E+00	0.81	3.05E-01	8.24E-01

**SPART**	0.22	2.41E-01	1.00E+00	-0.25	3.05E-01	8.24E-01
**CCS**	-0.17	3.83E-01	1.00E+00	0.18	3.05E-01	8.25E-01
**TNFSF12**	0.11	7.77E-01	1.00E+00	0.47	3.05E-01	8.25E-01
**ALG5**	0.08	6.78E-01	1.00E+00	-0.29	3.05E-01	8.25E-01
**LINC01134**	-1.99	1.60E-01	1.00E+00	-1.09	3.05E-01	8.25E-01
**AC005064.1**	0.30	6.98E-01	1.00E+00	-1.06	3.05E-01	8.25E-01
**PJA1**	0.28	2.87E-01	1.00E+00	-0.22	3.05E-01	8.25E-01
**BTN2A2**	0.02	9.26E-01	1.00E+00	0.42	3.05E-01	8.25E-01
**PGBD2**	-0.56	1.11E-01	1.00E+00	-0.33	3.05E-01	8.25E-01
**SYNGR1**	-0.34	1.54E-01	1.00E+00	-0.29	3.05E-01	8.25E-01
**MYCBP**	0.19	3.35E-01	1.00E+00	-0.28	3.05E-01	8.25E-01
**LIG3**	-0.26	2.14E-01	1.00E+00	-0.34	3.05E-01	8.25E-01
**SERPINB9P1**	-0.32	7.84E-01	1.00E+00	0.96	3.05E-01	8.25E-01
**AC079447.1**	-1.30	7.03E-01	1.00E+00	0.73	3.05E-01	8.25E-01
**MGST2**	0.11	5.84E-01	1.00E+00	-0.35	3.05E-01	8.25E-01
**LINC00710**	2.91	3.57E-01	1.00E+00	-1.51	3.06E-01	8.25E-01
**RAB14**	0.19	2.65E-01	1.00E+00	-0.24	3.06E-01	8.25E-01
**NRSN2**	-0.20	5.67E-01	1.00E+00	0.27	3.06E-01	8.25E-01
**RBMXP4**	0.63	6.02E-01	1.00E+00	1.00	3.06E-01	8.25E-01
**FAM219A**	-0.17	4.49E-01	1.00E+00	0.53	3.06E-01	8.26E-01
**ZDHHC14**	-0.37	2.45E-01	1.00E+00	0.45	3.06E-01	8.26E-01
**TMSB4XP1**	-1.34	6.97E-01	1.00E+00	-0.64	3.06E-01	8.26E-01
**AC005180.2**	-2.16	1.24E-02	6.93E-01	0.85	3.06E-01	8.26E-01
**AC016586.1**	-0.18	8.60E-01	1.00E+00	0.93	3.06E-01	8.26E-01
**A4GALT**	0.35	2.90E-01	1.00E+00	0.71	3.06E-01	8.26E-01
**TNK2-AS1**	-1.83	7.73E-02	1.00E+00	0.84	3.06E-01	8.26E-01
**TRIP11**	-0.13	6.20E-01	1.00E+00	-0.22	3.06E-01	8.26E-01
**DAAM1**	0.21	5.11E-01	1.00E+00	-0.35	3.06E-01	8.26E-01
**EYA3**	0.08	7.58E-01	1.00E+00	0.32	3.06E-01	8.26E-01
**PRKAG1**	0.00	9.98E-01	1.00E+00	0.16	3.06E-01	8.26E-01
**AC103706.1**	1.86	3.90E-01	1.00E+00	1.42	3.06E-01	8.26E-01
**MTFR2**	0.02	9.78E-01	1.00E+00	0.50	3.07E-01	8.26E-01

**ATP2B1-AS1**	-0.42	3.87E-01	1.00E+00	0.28	3.07E-01	8.26E-01
**G9514**	-3.20	1.56E-01	1.00E+00	-1.13	3.07E-01	8.26E-01
**G7242**	0.37	6.93E-01	1.00E+00	-0.47	3.07E-01	8.26E-01
**MET**	0.08	8.43E-01	1.00E+00	-0.32	3.07E-01	8.26E-01
**MYL6B**	-0.08	7.45E-01	1.00E+00	0.23	3.07E-01	8.26E-01
**GJA1**	-0.19	6.52E-01	1.00E+00	0.42	3.07E-01	8.26E-01
**LINC02572**	0.48	6.11E-01	1.00E+00	-1.28	3.07E-01	8.26E-01
**HLA-DMA**	-0.52	1.29E-01	1.00E+00	0.35	3.07E-01	8.26E-01
**AC005229.4**	-0.52	2.81E-01	1.00E+00	-0.51	3.07E-01	8.26E-01
**DNAH12**	-2.30	3.18E-01	1.00E+00	1.13	3.07E-01	8.27E-01
**MRPS31**	0.06	7.05E-01	1.00E+00	-0.29	3.07E-01	8.27E-01
**AC011468.5**	-2.13	4.73E-01	1.00E+00	-1.15	3.07E-01	8.27E-01
**EPB41L1**	0.12	7.80E-01	1.00E+00	0.40	3.07E-01	8.27E-01
**CEP68**	0.09	7.55E-01	1.00E+00	-0.25	3.07E-01	8.27E-01
**PCDH15**	4.69	2.17E-02	8.94E-01	-1.55	3.07E-01	8.27E-01
**ERCC3**	0.00	9.88E-01	1.00E+00	-0.17	3.07E-01	8.27E-01
**ZNF136**	0.19	4.94E-01	1.00E+00	0.39	3.07E-01	8.27E-01
**LIN7C**	0.28	3.80E-01	1.00E+00	-0.45	3.07E-01	8.27E-01
**FBXO41**	-0.33	1.52E-01	1.00E+00	0.86	3.07E-01	8.27E-01
**LAMP1**	0.02	9.22E-01	1.00E+00	0.52	3.07E-01	8.27E-01
**SESN3**	0.18	7.01E-01	1.00E+00	0.37	3.07E-01	8.27E-01
**TST**	0.07	8.22E-01	1.00E+00	0.37	3.08E-01	8.27E-01
**CASS4**	0.19	7.37E-01	1.00E+00	0.83	3.08E-01	8.27E-01
**MAN1C1**	0.04	9.02E-01	1.00E+00	0.51	3.08E-01	8.27E-01
**FAM107B**	0.24	4.67E-01	1.00E+00	-0.27	3.08E-01	8.27E-01
**G22771**	-0.95	3.21E-01	1.00E+00	-0.70	3.08E-01	8.27E-01
**GGCT**	-0.19	7.11E-01	1.00E+00	0.53	3.08E-01	8.28E-01
**RASGRF2**	0.46	5.00E-01	1.00E+00	0.54	3.08E-01	8.28E-01
**LHFPL6**	0.64	7.70E-02	1.00E+00	0.43	3.08E-01	8.28E-01
**TTC5**	0.22	3.26E-01	1.00E+00	-0.31	3.08E-01	8.28E-01
**LINC01268**	0.66	6.10E-01	1.00E+00	1.50	3.08E-01	8.28E-01
**SERBP1P5**	0.26	7.09E-01	1.00E+00	-0.36	3.08E-01	8.28E-01

**FIBIN**	0.66	1.75E-01	1.00E+00	0.41	3.08E-01	8.28E-01
**FETUB**	-0.08	9.25E-01	1.00E+00	0.85	3.08E-01	8.28E-01
**PYHIN1**	-0.98	2.84E-01	1.00E+00	1.00	3.08E-01	8.28E-01
**TMBIM6**	0.28	1.35E-01	1.00E+00	-0.26	3.08E-01	8.28E-01
**DCTPP1**	0.07	7.58E-01	1.00E+00	-0.26	3.08E-01	8.28E-01
**LINC00968**	0.23	7.16E-01	1.00E+00	-0.69	3.08E-01	8.28E-01
**BATF3**	-0.42	3.03E-01	1.00E+00	0.78	3.08E-01	8.28E-01
**TMEM61**	-0.01	9.87E-01	1.00E+00	-0.55	3.08E-01	8.28E-01
**HOXB4**	0.38	3.80E-01	1.00E+00	0.61	3.08E-01	8.28E-01
**METTL13**	0.28	6.69E-02	1.00E+00	0.29	3.08E-01	8.28E-01
**SP9**	0.51	4.79E-01	1.00E+00	-0.88	3.09E-01	8.28E-01
**AC002128.1**	4.51	1.19E-01	1.00E+00	-1.02	3.09E-01	8.28E-01
**PAF1**	0.13	5.33E-01	1.00E+00	0.26	3.09E-01	8.28E-01
**CD200**	0.29	6.79E-01	1.00E+00	0.41	3.09E-01	8.28E-01
**RNF227**	0.02	9.77E-01	1.00E+00	-0.49	3.09E-01	8.28E-01
**PRKCB**	-0.39	1.00E-01	1.00E+00	-0.41	3.09E-01	8.28E-01
**EVI2A**	0.20	7.85E-01	1.00E+00	0.44	3.09E-01	8.28E-01
**LAIR1**	1.32	1.20E-01	1.00E+00	0.73	3.09E-01	8.28E-01
**CARD8-AS1**	0.64	2.17E-01	1.00E+00	0.66	3.09E-01	8.28E-01
**CIAPIN1**	0.03	8.61E-01	1.00E+00	-0.19	3.09E-01	8.28E-01
**ENTPD1**	0.09	7.71E-01	1.00E+00	0.44	3.09E-01	8.28E-01
**HCN3**	-0.55	5.46E-02	1.00E+00	-0.59	3.09E-01	8.28E-01
**RAMP2**	0.19	6.97E-01	1.00E+00	-0.44	3.09E-01	8.28E-01
**MRPL52**	-0.14	5.26E-01	1.00E+00	-0.28	3.09E-01	8.28E-01
**RGS17**	0.38	7.61E-01	1.00E+00	0.86	3.09E-01	8.28E-01
**ADGRF3**	0.00	9.97E-01	1.00E+00	1.04	3.09E-01	8.28E-01
**BAG5**	0.00	9.98E-01	1.00E+00	-0.21	3.09E-01	8.28E-01
**LAMA5**	-0.39	2.70E-01	1.00E+00	0.74	3.09E-01	8.28E-01
**FGF17**	-0.10	8.82E-01	1.00E+00	-1.63	3.09E-01	8.29E-01
**MT-CO3**	-0.11	8.12E-01	1.00E+00	-0.33	3.09E-01	8.29E-01
**CHEK1**	-0.14	5.51E-01	1.00E+00	0.27	3.09E-01	8.29E-01
**NFE2L1**	-0.05	7.64E-01	1.00E+00	0.54	3.10E-01	8.29E-01

**H3F3B**	-0.22	4.35E-01	1.00E+00	-0.40	3.10E-01	8.29E-01
**KLHDC7B**	-2.29	9.80E-02	1.00E+00	3.18	3.10E-01	8.29E-01
**FOXD2**	-0.36	6.15E-01	1.00E+00	0.77	3.10E-01	8.29E-01
**TEKT3**	0.28	6.47E-01	1.00E+00	-0.72	3.10E-01	8.29E-01
**AL031775.1**	2.57	1.04E-01	1.00E+00	-0.87	3.10E-01	8.29E-01
**RER1**	0.13	4.15E-01	1.00E+00	-0.23	3.10E-01	8.29E-01
**LINC00674**	0.09	8.02E-01	1.00E+00	-0.32	3.10E-01	8.29E-01
**NFKBIB**	0.01	9.79E-01	1.00E+00	0.41	3.10E-01	8.29E-01
**FZD6**	0.20	4.65E-01	1.00E+00	-0.24	3.10E-01	8.29E-01
**LY6E-DT**	0.16	7.88E-01	1.00E+00	0.55	3.10E-01	8.29E-01
**SNORD3B-1**	0.62	6.07E-01	1.00E+00	-0.84	3.10E-01	8.29E-01
**PPP1R1B**	0.36	6.33E-01	1.00E+00	-0.68	3.10E-01	8.29E-01
**DGAT2L6**	0.70	8.28E-01	1.00E+00	-3.19	3.10E-01	8.29E-01
**SNRNP48**	0.04	8.91E-01	1.00E+00	-0.22	3.10E-01	8.29E-01
**ZNF106**	0.40	2.26E-01	1.00E+00	-0.30	3.10E-01	8.29E-01
**TAF2**	0.02	9.25E-01	1.00E+00	-0.16	3.10E-01	8.29E-01
**ELK1**	-0.22	3.03E-01	1.00E+00	0.34	3.10E-01	8.29E-01
**PGLYRP2**	-1.80	1.31E-01	1.00E+00	0.97	3.10E-01	8.29E-01
**AC025809.2**	NA	NA	NA	-2.14	3.10E-01	8.29E-01
**SKAP1**	0.28	6.38E-01	1.00E+00	0.37	3.10E-01	8.29E-01
**XLOC_014155**	-0.31	9.09E-01	1.00E+00	-1.29	3.10E-01	8.29E-01
**APLF**	-0.02	9.66E-01	1.00E+00	-0.33	3.11E-01	8.29E-01
**AC016582.3**	0.06	8.95E-01	1.00E+00	-0.73	3.11E-01	8.29E-01
**SEMA3E**	0.12	8.39E-01	1.00E+00	-0.66	3.11E-01	8.29E-01
**XLOC_007942**	0.70	6.68E-01	1.00E+00	0.96	3.11E-01	8.29E-01
**AL031587.5**	-0.58	4.98E-01	1.00E+00	0.86	3.11E-01	8.29E-01
**HSPA7**	-1.17	1.91E-01	1.00E+00	0.69	3.11E-01	8.29E-01
**RAB32**	0.10	7.33E-01	1.00E+00	-0.52	3.11E-01	8.29E-01
**IFITM2**	-0.18	6.27E-01	1.00E+00	0.37	3.11E-01	8.29E-01
**AL158050.1**	1.52	5.47E-01	1.00E+00	-0.76	3.11E-01	8.29E-01
**ATF6**	0.00	9.91E-01	1.00E+00	-0.12	3.11E-01	8.29E-01
**LSM6**	0.28	1.85E-01	1.00E+00	-0.31	3.11E-01	8.29E-01

**CROCCP2**	0.37	4.24E-01	1.00E+00	0.57	3.11E-01	8.29E-01
**KNDC1**	-2.78	3.46E-06	1.15E-03	1.22	3.11E-01	8.30E-01
**AL049634.1**	0.25	9.42E-01	1.00E+00	2.12	3.11E-01	8.30E-01
**RXRB**	0.18	4.57E-01	1.00E+00	0.46	3.11E-01	8.30E-01
**PFN2**	-0.19	6.68E-01	1.00E+00	-0.32	3.11E-01	8.30E-01
**LAMP5**	0.15	8.21E-01	1.00E+00	0.70	3.11E-01	8.30E-01
**SLC25A25**	-0.98	1.76E-01	1.00E+00	0.58	3.11E-01	8.30E-01
**SMAD5-AS1**	-0.03	9.83E-01	1.00E+00	-0.94	3.11E-01	8.30E-01
**ARMC7**	0.01	9.59E-01	1.00E+00	0.38	3.11E-01	8.30E-01
**AC079210.1**	-2.45	2.73E-01	1.00E+00	1.24	3.11E-01	8.30E-01
**MYH16**	-2.32	2.42E-01	1.00E+00	1.12	3.11E-01	8.30E-01
**AL138831.1**	3.12	3.46E-01	1.00E+00	1.26	3.11E-01	8.30E-01
**ZBTB45**	-0.08	7.40E-01	1.00E+00	0.35	3.12E-01	8.30E-01
**TDO2**	-1.64	1.10E-01	1.00E+00	3.18	3.12E-01	8.30E-01
**RPL19**	-0.20	4.67E-01	1.00E+00	0.23	3.12E-01	8.30E-01
**USP13**	0.34	2.31E-01	1.00E+00	-0.35	3.12E-01	8.30E-01
**GNAI1**	0.22	4.91E-01	1.00E+00	-0.30	3.12E-01	8.30E-01
**MSL2**	-0.03	9.01E-01	1.00E+00	-0.36	3.12E-01	8.30E-01
**G10958**	-2.03	3.72E-02	1.00E+00	0.67	3.12E-01	8.30E-01
**AL591845.1**	-0.45	4.66E-01	1.00E+00	0.76	3.12E-01	8.30E-01
**DAB1**	-1.00	1.92E-01	1.00E+00	-1.07	3.12E-01	8.30E-01
**HNRNPA3**	-0.04	8.36E-01	1.00E+00	0.38	3.12E-01	8.30E-01
**ASB1**	0.43	3.43E-01	1.00E+00	0.41	3.12E-01	8.30E-01
**RUSC1**	-0.24	3.03E-01	1.00E+00	0.25	3.12E-01	8.30E-01
**TRDJ1**	0.67	8.43E-01	1.00E+00	-1.19	3.12E-01	8.30E-01
**EDA2R**	0.13	7.77E-01	1.00E+00	0.76	3.12E-01	8.30E-01
**PPP2CA**	0.02	9.56E-01	1.00E+00	-0.29	3.12E-01	8.30E-01
**GNG12**	0.03	9.08E-01	1.00E+00	-0.26	3.12E-01	8.30E-01
**KARSP2**	-1.41	2.79E-01	1.00E+00	-1.41	3.12E-01	8.30E-01
**STMN1**	-0.23	2.27E-01	1.00E+00	-0.30	3.12E-01	8.30E-01
**USP34**	0.00	9.94E-01	1.00E+00	0.39	3.12E-01	8.30E-01
**AC092427.1**	-1.78	6.06E-01	1.00E+00	-1.48	3.12E-01	8.30E-01

**KIF20A**	0.16	6.56E-01	1.00E+00	0.60	3.12E-01	8.31E-01
**DYNC1I2P1**	-0.26	6.32E-01	1.00E+00	0.22	3.13E-01	8.31E-01
**AC024075.3**	0.29	6.08E-01	1.00E+00	0.65	3.13E-01	8.31E-01
**XLOC_010311**	0.23	6.38E-01	1.00E+00	-1.05	3.13E-01	8.31E-01
**PRKACB**	-0.08	8.60E-01	1.00E+00	-0.30	3.13E-01	8.31E-01
**SLC9A4**	-0.06	9.06E-01	1.00E+00	-0.93	3.13E-01	8.31E-01
**BTBD19**	0.59	3.36E-01	1.00E+00	0.76	3.13E-01	8.31E-01
**C19orf12**	0.28	2.90E-01	1.00E+00	-0.40	3.13E-01	8.31E-01
**LINC00982**	-0.78	1.35E-01	1.00E+00	-1.02	3.13E-01	8.31E-01
**RPS6KA4**	-0.34	2.63E-01	1.00E+00	0.69	3.13E-01	8.31E-01
**AC022400.7**	0.22	6.42E-01	1.00E+00	-0.41	3.13E-01	8.31E-01
**ZNF548**	0.20	3.12E-01	1.00E+00	-0.23	3.13E-01	8.31E-01
**KCNN4**	0.05	9.65E-01	1.00E+00	-0.51	3.13E-01	8.31E-01
**AC116353.5**	-2.57	1.10E-01	1.00E+00	0.82	3.13E-01	8.31E-01
**AC005332.5**	0.49	4.52E-01	1.00E+00	0.67	3.13E-01	8.31E-01
**HDAC3**	0.11	5.33E-01	1.00E+00	-0.19	3.13E-01	8.31E-01
**TPTEP2**	0.25	5.65E-01	1.00E+00	0.28	3.13E-01	8.32E-01
**RRP36**	-0.22	3.22E-01	1.00E+00	-0.55	3.13E-01	8.32E-01
**KCNA2**	0.09	9.02E-01	1.00E+00	-1.12	3.14E-01	8.32E-01
**STK26**	-0.13	7.37E-01	1.00E+00	-0.32	3.14E-01	8.32E-01
**PRRX1**	-0.03	9.41E-01	1.00E+00	0.64	3.14E-01	8.32E-01
**DAD1**	-0.01	9.33E-01	1.00E+00	-0.31	3.14E-01	8.32E-01
**XLOC_009615**	-0.03	9.85E-01	1.00E+00	-1.49	3.14E-01	8.32E-01
**RPL21P120**	2.75	3.93E-01	1.00E+00	-0.64	3.14E-01	8.32E-01
**ROBO3**	0.28	5.68E-01	1.00E+00	0.53	3.14E-01	8.32E-01
**ZNF148**	0.14	5.09E-01	1.00E+00	-0.17	3.14E-01	8.32E-01
**GPR4**	-0.50	4.10E-01	1.00E+00	0.79	3.14E-01	8.32E-01
**AC026471.1**	-0.21	6.03E-01	1.00E+00	0.47	3.14E-01	8.32E-01
**IPO7**	0.16	3.38E-01	1.00E+00	0.38	3.14E-01	8.32E-01
**ZNF511**	0.57	8.14E-02	1.00E+00	-0.32	3.14E-01	8.32E-01
**AC004890.1**	-2.54	4.56E-01	1.00E+00	-0.68	3.14E-01	8.32E-01
**COL8A2**	-0.12	7.80E-01	1.00E+00	0.61	3.14E-01	8.32E-01

**TUBGCP4**	0.00	9.90E-01	1.00E+00	-0.21	3.14E-01	8.32E-01
**MINK1**	-0.19	4.90E-01	1.00E+00	0.61	3.14E-01	8.32E-01
**NAT10**	0.01	9.68E-01	1.00E+00	-0.31	3.14E-01	8.32E-01
**SOCS7**	0.35	3.51E-01	1.00E+00	0.43	3.14E-01	8.32E-01
**AC007533.1**	1.94	5.51E-01	1.00E+00	-1.26	3.14E-01	8.32E-01
**NXT2**	0.07	8.28E-01	1.00E+00	-0.41	3.14E-01	8.32E-01
**RUVBL2**	-0.24	2.18E-01	1.00E+00	0.16	3.14E-01	8.32E-01
**CA5B**	0.18	6.03E-01	1.00E+00	0.37	3.15E-01	8.33E-01
**NT5DC1**	-0.32	1.60E-01	1.00E+00	-0.19	3.15E-01	8.33E-01
**LOXL1**	-0.16	7.43E-01	1.00E+00	0.73	3.15E-01	8.33E-01
**CHMP1B2P**	-0.96	2.35E-01	1.00E+00	-0.73	3.15E-01	8.33E-01
**GPR63**	-0.99	6.30E-02	1.00E+00	-0.81	3.15E-01	8.33E-01
**CSGALNACT1**	0.30	4.60E-01	1.00E+00	0.47	3.15E-01	8.33E-01
**CXorf38**	-0.02	9.13E-01	1.00E+00	0.26	3.15E-01	8.33E-01
**LPO**	-0.09	9.44E-01	1.00E+00	1.53	3.15E-01	8.33E-01
**C9orf153**	-1.90	5.50E-01	1.00E+00	-1.28	3.15E-01	8.33E-01
**HMGXB3**	-0.15	5.29E-01	1.00E+00	0.56	3.15E-01	8.33E-01
**CCL23**	0.08	8.97E-01	1.00E+00	0.64	3.15E-01	8.33E-01
**TLL1**	0.14	7.31E-01	1.00E+00	0.42	3.15E-01	8.33E-01
**KCNF1**	-3.56	2.07E-03	2.39E-01	1.74	3.15E-01	8.33E-01
**CHMP2A**	-0.05	7.49E-01	1.00E+00	-0.15	3.15E-01	8.33E-01
**AC092809.3**	-1.07	5.40E-01	1.00E+00	-0.68	3.15E-01	8.33E-01
**HNRNPH3**	-0.07	7.16E-01	1.00E+00	-0.16	3.15E-01	8.34E-01
**ZCCHC17**	0.00	9.95E-01	1.00E+00	-0.22	3.15E-01	8.34E-01
**GDPD1**	-0.23	5.95E-01	1.00E+00	-0.58	3.15E-01	8.34E-01
**AP3D1**	-0.08	6.98E-01	1.00E+00	0.48	3.16E-01	8.34E-01
**TUBB1**	0.44	6.73E-01	1.00E+00	-1.34	3.16E-01	8.34E-01
**CDC23**	0.03	8.58E-01	1.00E+00	-0.28	3.16E-01	8.34E-01
**PGBD4**	0.13	7.61E-01	1.00E+00	0.43	3.16E-01	8.34E-01
**LMAN2**	-0.05	7.79E-01	1.00E+00	0.17	3.16E-01	8.34E-01
**ZNF217**	-0.25	2.68E-01	1.00E+00	0.38	3.16E-01	8.34E-01
**MORN2**	0.02	9.22E-01	1.00E+00	-0.37	3.16E-01	8.34E-01

**NNT**	0.17	7.08E-01	1.00E+00	-0.28	3.16E-01	8.34E-01
**IDH1-AS1**	-0.12	9.11E-01	1.00E+00	-0.76	3.16E-01	8.34E-01
**PEA15**	0.10	5.89E-01	1.00E+00	0.26	3.16E-01	8.34E-01
**SNAP91**	-0.92	7.84E-01	1.00E+00	1.47	3.16E-01	8.34E-01
**G36419**	-3.45	3.63E-02	1.00E+00	-0.86	3.16E-01	8.34E-01
**PCBP2**	-0.06	8.01E-01	1.00E+00	0.32	3.16E-01	8.34E-01
**PMS1**	-0.26	4.16E-01	1.00E+00	-0.39	3.16E-01	8.34E-01
**LRIG2**	-0.02	9.32E-01	1.00E+00	-0.25	3.16E-01	8.34E-01
**LDLRAD2**	0.77	1.36E-01	1.00E+00	0.86	3.16E-01	8.34E-01
**ZSCAN16**	-0.23	4.82E-01	1.00E+00	-0.40	3.16E-01	8.34E-01
**ZNF609**	-0.38	1.10E-01	1.00E+00	0.61	3.16E-01	8.34E-01
**ALG6**	0.09	7.64E-01	1.00E+00	-0.38	3.16E-01	8.34E-01
**HIST2H2BF**	-0.91	2.05E-01	1.00E+00	-0.68	3.16E-01	8.34E-01
**LINC01150**	-1.12	3.41E-01	1.00E+00	1.05	3.16E-01	8.34E-01
**AC114488.2**	1.64	6.29E-02	1.00E+00	0.68	3.16E-01	8.34E-01
**TTC1**	-0.01	9.42E-01	1.00E+00	-0.32	3.17E-01	8.35E-01
**AC005083.1**	0.39	2.79E-01	1.00E+00	-0.71	3.17E-01	8.35E-01
**G16361**	-4.77	1.55E-01	1.00E+00	0.69	3.17E-01	8.35E-01
**AD000090.1**	0.13	6.83E-01	1.00E+00	-0.99	3.17E-01	8.35E-01
**CD99L2**	0.59	2.12E-01	1.00E+00	0.53	3.17E-01	8.35E-01
**SMC5**	-0.24	3.01E-01	1.00E+00	-0.24	3.17E-01	8.35E-01
**MIR22HG**	-0.08	7.87E-01	1.00E+00	0.27	3.17E-01	8.35E-01
**VSIG8**	0.29	6.85E-01	1.00E+00	-0.91	3.17E-01	8.35E-01
**CEP78**	-0.53	2.38E-03	2.47E-01	-0.27	3.17E-01	8.35E-01
**SERPINC1**	0.54	7.42E-01	1.00E+00	1.28	3.17E-01	8.35E-01
**AC006042.4**	-2.16	1.93E-01	1.00E+00	0.74	3.17E-01	8.35E-01
**CYTOR**	-0.18	7.49E-01	1.00E+00	0.41	3.17E-01	8.35E-01
**EIF1P3**	1.35	6.96E-01	1.00E+00	-0.78	3.17E-01	8.35E-01
**DUSP15**	-0.30	5.34E-01	1.00E+00	0.79	3.17E-01	8.35E-01
**XLOC_006870**	-0.34	5.64E-01	1.00E+00	-0.95	3.17E-01	8.35E-01
**AP001267.3**	-3.26	3.69E-02	1.00E+00	-1.06	3.17E-01	8.35E-01
**MAOB**	0.74	1.66E-01	1.00E+00	-0.51	3.17E-01	8.35E-01

**AC125437.1**	-0.87	3.81E-01	1.00E+00	0.99	3.17E-01	8.35E-01
**CYP3A7**	-0.53	8.66E-01	1.00E+00	-1.08	3.17E-01	8.35E-01
**NRAS**	0.05	8.59E-01	1.00E+00	0.16	3.17E-01	8.35E-01
**FMO4**	0.23	5.33E-01	1.00E+00	0.40	3.17E-01	8.35E-01
**TFF3**	0.14	6.95E-01	1.00E+00	0.54	3.17E-01	8.35E-01
**LINC01285**	-0.99	6.66E-01	1.00E+00	-1.54	3.18E-01	8.35E-01
**WNT2B**	0.30	3.68E-01	1.00E+00	-0.53	3.18E-01	8.35E-01
**TTBK1**	-2.19	4.79E-01	1.00E+00	1.52	3.18E-01	8.35E-01
**AP000462.2**	NA	NA	NA	-1.73	3.18E-01	8.35E-01
**IFFO2**	-0.15	7.82E-01	1.00E+00	0.59	3.18E-01	8.35E-01
**ANGPTL7**	-0.86	2.09E-01	1.00E+00	0.88	3.18E-01	8.35E-01
**ZCCHC3**	0.25	2.81E-01	1.00E+00	0.46	3.18E-01	8.35E-01
**ZBTB34**	0.36	1.26E-01	1.00E+00	0.35	3.18E-01	8.35E-01
**LINC02256**	0.31	7.05E-01	1.00E+00	-0.44	3.18E-01	8.35E-01
**CALM1**	0.03	8.61E-01	1.00E+00	-0.24	3.18E-01	8.35E-01
**AL391807.1**	-0.65	6.97E-01	1.00E+00	-1.55	3.18E-01	8.35E-01
**EHD3**	-0.55	7.47E-02	1.00E+00	0.51	3.18E-01	8.35E-01
**TYRP1**	0.59	3.80E-01	1.00E+00	0.83	3.18E-01	8.35E-01
**FAM86JP**	-0.16	7.13E-01	1.00E+00	0.40	3.18E-01	8.35E-01
**AP002954.1**	-1.11	4.93E-01	1.00E+00	1.21	3.18E-01	8.35E-01
**AL356479.1**	-0.53	5.75E-01	1.00E+00	-0.74	3.18E-01	8.35E-01
**ENY2**	0.13	4.46E-01	1.00E+00	-0.22	3.18E-01	8.35E-01
**KATNA1**	0.02	9.37E-01	1.00E+00	-0.29	3.18E-01	8.35E-01
**LINC02001**	-0.36	2.58E-01	1.00E+00	-0.40	3.18E-01	8.35E-01
**AC092171.3**	-0.43	7.68E-02	1.00E+00	0.78	3.18E-01	8.35E-01
**TFAP2B**	-0.38	2.55E-01	1.00E+00	-0.47	3.18E-01	8.35E-01
**OR7E22P**	-2.86	1.48E-02	7.62E-01	0.96	3.18E-01	8.35E-01
**RPL27A**	-0.18	5.05E-01	1.00E+00	0.31	3.18E-01	8.35E-01
**XLOC_012077**	0.19	8.71E-01	1.00E+00	-0.99	3.18E-01	8.35E-01
**HOXD11**	1.07	5.06E-01	1.00E+00	1.30	3.18E-01	8.35E-01
**GOLGA8A**	0.21	7.84E-01	1.00E+00	0.70	3.18E-01	8.35E-01
**SEMA4G**	-0.65	1.04E-01	1.00E+00	0.57	3.18E-01	8.35E-01

**PLBD1**	-0.06	8.55E-01	1.00E+00	0.27	3.18E-01	8.35E-01
**AC084809.1**	-1.40	2.23E-01	1.00E+00	0.66	3.18E-01	8.35E-01
**COA7**	-0.06	8.13E-01	1.00E+00	0.35	3.18E-01	8.35E-01
**IL2RB**	0.12	6.98E-01	1.00E+00	0.70	3.18E-01	8.35E-01
**BMS1P16**	-3.07	3.54E-01	1.00E+00	0.81	3.18E-01	8.35E-01
**CRCT1**	0.54	3.93E-01	1.00E+00	0.76	3.18E-01	8.35E-01
**SNX1**	0.05	7.88E-01	1.00E+00	0.15	3.19E-01	8.35E-01
**NFKBIA**	-0.82	1.62E-01	1.00E+00	0.42	3.19E-01	8.35E-01
**Z73417.1**	-1.14	2.71E-01	1.00E+00	1.03	3.19E-01	8.35E-01
**SLC22A15**	0.14	7.76E-01	1.00E+00	-0.47	3.19E-01	8.35E-01
**DUSP10**	-0.01	9.68E-01	1.00E+00	0.26	3.19E-01	8.35E-01
**C10orf82**	0.39	6.50E-01	1.00E+00	-0.92	3.19E-01	8.35E-01
**DUSP2**	-1.70	4.31E-02	1.00E+00	-0.53	3.19E-01	8.35E-01
**AC106786.2**	-2.00	2.55E-01	1.00E+00	0.84	3.19E-01	8.35E-01
**RPP25**	-0.07	8.10E-01	1.00E+00	0.42	3.19E-01	8.35E-01
**CYP7B1**	-0.26	5.11E-01	1.00E+00	0.35	3.19E-01	8.35E-01
**RPS7P10**	-0.76	4.12E-01	1.00E+00	-0.48	3.19E-01	8.35E-01
**MTM1**	0.06	7.95E-01	1.00E+00	-0.28	3.19E-01	8.35E-01
**AL049776.1**	0.89	5.90E-01	1.00E+00	0.98	3.19E-01	8.35E-01
**CCDC25**	0.06	7.70E-01	1.00E+00	-0.18	3.19E-01	8.35E-01
**G3632**	1.53	9.18E-02	1.00E+00	-0.89	3.19E-01	8.35E-01
**FOXP4-AS1**	0.42	8.44E-01	1.00E+00	-1.36	3.19E-01	8.35E-01
**DCTN4**	0.06	7.15E-01	1.00E+00	0.17	3.19E-01	8.35E-01
**AC097532.2**	-0.20	8.20E-01	1.00E+00	0.72	3.19E-01	8.35E-01
**CACUL1**	0.24	2.81E-01	1.00E+00	0.25	3.19E-01	8.35E-01
**VWA7**	-0.39	3.02E-01	1.00E+00	-0.82	3.19E-01	8.35E-01
**MAGI3**	-0.18	6.18E-01	1.00E+00	-0.35	3.19E-01	8.35E-01
**LINC00327**	0.26	6.34E-01	1.00E+00	-0.69	3.20E-01	8.35E-01
**BNIP2**	0.21	3.40E-01	1.00E+00	-0.24	3.20E-01	8.35E-01
**ZNF629**	-0.41	1.38E-01	1.00E+00	0.51	3.20E-01	8.35E-01
**ZNF391**	0.81	1.03E-01	1.00E+00	-0.51	3.20E-01	8.35E-01
**AL731566.1**	0.04	9.79E-01	1.00E+00	0.69	3.20E-01	8.35E-01

**KATNB1**	-0.22	4.50E-01	1.00E+00	0.22	3.20E-01	8.35E-01
**PKD1P6**	-0.92	3.17E-02	1.00E+00	0.70	3.20E-01	8.35E-01
**LSM12P1**	-0.69	5.19E-01	1.00E+00	-0.32	3.20E-01	8.35E-01
**ARHGEF28**	-0.07	7.01E-01	1.00E+00	-0.31	3.20E-01	8.35E-01
**FAM90A1**	1.17	1.35E-01	1.00E+00	1.06	3.20E-01	8.35E-01
**RAPGEF6**	0.09	7.89E-01	1.00E+00	0.39	3.20E-01	8.35E-01
**MED24**	-0.08	7.26E-01	1.00E+00	0.39	3.20E-01	8.35E-01
**CORIN**	-0.04	9.70E-01	1.00E+00	0.65	3.20E-01	8.35E-01
**PATL2**	-1.23	1.56E-01	1.00E+00	0.82	3.20E-01	8.35E-01
**SH3BGR**	-3.06	1.23E-01	1.00E+00	-0.76	3.20E-01	8.35E-01
**TROVE2**	0.11	6.35E-01	1.00E+00	-0.41	3.20E-01	8.35E-01
**UQCRB**	0.09	7.13E-01	1.00E+00	-0.22	3.20E-01	8.35E-01
**AC007681.1**	0.45	5.80E-01	1.00E+00	-0.80	3.20E-01	8.35E-01
**FAM177B**	1.55	3.58E-01	1.00E+00	0.77	3.20E-01	8.36E-01
**TRMT112P6**	-0.20	9.53E-01	1.00E+00	-0.74	3.20E-01	8.36E-01
**AC009226.1**	0.33	7.98E-01	1.00E+00	0.59	3.20E-01	8.36E-01
**AC139491.5**	0.63	6.96E-01	1.00E+00	1.59	3.20E-01	8.36E-01
**MMP8**	3.60	2.65E-01	1.00E+00	-1.27	3.20E-01	8.36E-01
**ELK4**	0.01	9.69E-01	1.00E+00	0.22	3.20E-01	8.36E-01
**LINC02345**	-0.28	6.83E-01	1.00E+00	-0.59	3.20E-01	8.36E-01
**ICAM2**	0.14	7.70E-01	1.00E+00	0.47	3.21E-01	8.36E-01
**NDUFB2-AS1**	-0.56	7.02E-01	1.00E+00	-0.67	3.21E-01	8.36E-01
**C3**	0.62	1.49E-01	1.00E+00	0.67	3.21E-01	8.36E-01
**DNAJC19P5**	-1.44	5.34E-02	1.00E+00	1.30	3.21E-01	8.36E-01
**TNRC6C**	-0.25	4.58E-01	1.00E+00	0.46	3.21E-01	8.36E-01
**LINC00840**	-1.00	2.22E-01	1.00E+00	-0.80	3.21E-01	8.36E-01
**G42332**	-1.46	2.24E-01	1.00E+00	1.20	3.21E-01	8.36E-01
**LINC02334**	-1.30	7.06E-01	1.00E+00	-1.20	3.21E-01	8.36E-01
**SATB1**	0.29	2.08E-01	1.00E+00	0.28	3.21E-01	8.36E-01
**LRRFIP1P1**	-2.17	3.99E-02	1.00E+00	0.57	3.21E-01	8.36E-01
**AC008840.1**	0.46	8.93E-01	1.00E+00	-1.69	3.21E-01	8.36E-01
**CDC40**	0.15	5.19E-01	1.00E+00	0.18	3.21E-01	8.36E-01

**AC079921.1**	-2.39	8.56E-02	1.00E+00	-1.21	3.21E-01	8.36E-01
**MOSMO**	0.46	1.36E-01	1.00E+00	-0.30	3.21E-01	8.36E-01
**SH3BGRL2**	0.67	1.59E-01	1.00E+00	-0.36	3.21E-01	8.36E-01
**CXCL13**	-4.09	1.68E-01	1.00E+00	3.12	3.21E-01	8.36E-01
**AC114284.1**	-1.52	4.85E-01	1.00E+00	1.31	3.21E-01	8.36E-01
**IRAK4**	-0.01	9.74E-01	1.00E+00	0.24	3.21E-01	8.36E-01
**ZNF599**	0.05	8.14E-01	1.00E+00	-0.41	3.21E-01	8.36E-01
**ELK3**	0.03	9.16E-01	1.00E+00	0.39	3.21E-01	8.36E-01
**AC010320.1**	-0.03	9.85E-01	1.00E+00	1.16	3.21E-01	8.36E-01
**AC011477.3**	1.47	1.57E-01	1.00E+00	0.71	3.21E-01	8.36E-01
**IGLV1-47**	7.45	2.69E-04	6.07E-02	3.12	3.21E-01	8.36E-01
**KCNIP3**	0.39	4.06E-01	1.00E+00	0.50	3.21E-01	8.36E-01
**FAM193A**	-0.29	2.08E-01	1.00E+00	0.43	3.21E-01	8.36E-01
**TEX30**	0.38	2.61E-01	1.00E+00	-0.41	3.21E-01	8.36E-01
**LRRC8D**	-0.26	3.69E-01	1.00E+00	0.39	3.21E-01	8.36E-01
**TSKU**	-0.16	4.49E-01	1.00E+00	-0.30	3.21E-01	8.36E-01
**LRRC43**	-1.22	6.45E-02	1.00E+00	0.62	3.22E-01	8.36E-01
**MRC1L1**	0.14	8.32E-01	1.00E+00	0.68	3.22E-01	8.36E-01
**RPSAP61**	-2.28	5.05E-01	1.00E+00	-0.89	3.22E-01	8.36E-01
**MIEN1**	0.08	7.10E-01	1.00E+00	-0.38	3.22E-01	8.36E-01
**SMARCA5**	0.17	5.42E-01	1.00E+00	0.22	3.22E-01	8.36E-01
**AC005180.1**	0.05	9.60E-01	1.00E+00	0.96	3.22E-01	8.36E-01
**LMNA**	-0.32	2.41E-01	1.00E+00	0.23	3.22E-01	8.36E-01
**GCNT3**	0.23	9.18E-01	1.00E+00	1.57	3.22E-01	8.36E-01
**ASNS**	-0.24	5.13E-01	1.00E+00	-0.19	3.22E-01	8.36E-01
**UNC5A**	-0.91	3.79E-01	1.00E+00	1.57	3.22E-01	8.36E-01
**SNHG26**	-0.49	3.42E-01	1.00E+00	0.62	3.22E-01	8.36E-01
**NUDT14**	-0.11	7.05E-01	1.00E+00	0.26	3.22E-01	8.36E-01
**CHEK2**	-0.18	5.25E-01	1.00E+00	0.26	3.22E-01	8.36E-01
**ZNF17**	-0.20	5.26E-01	1.00E+00	0.28	3.22E-01	8.36E-01
**SLC2A6**	-0.08	8.89E-01	1.00E+00	0.94	3.22E-01	8.36E-01
**SPATA12**	-0.64	5.47E-01	1.00E+00	0.93	3.22E-01	8.36E-01

**AC002064.2**	3.45	8.10E-02	1.00E+00	1.56	3.22E-01	8.36E-01
**ALKBH3**	-0.11	4.97E-01	1.00E+00	-0.21	3.22E-01	8.36E-01
**PCMT1**	0.02	9.04E-01	1.00E+00	-0.33	3.22E-01	8.36E-01
**HTATIP2**	0.05	8.35E-01	1.00E+00	-0.28	3.22E-01	8.36E-01
**PARP4**	0.05	8.25E-01	1.00E+00	0.34	3.22E-01	8.36E-01
**LINC00475**	-0.72	4.36E-01	1.00E+00	-0.80	3.22E-01	8.36E-01
**POLR2A**	-0.48	3.31E-02	1.00E+00	0.45	3.22E-01	8.36E-01
**PLCL2**	0.42	2.16E-01	1.00E+00	0.73	3.22E-01	8.36E-01
**TRGV2**	-1.03	7.60E-01	1.00E+00	1.55	3.22E-01	8.36E-01
**NANP**	0.15	5.03E-01	1.00E+00	-0.29	3.23E-01	8.36E-01
**DPP9**	-0.14	5.44E-01	1.00E+00	0.47	3.23E-01	8.36E-01
**GANAB**	-0.09	6.44E-01	1.00E+00	0.39	3.23E-01	8.36E-01
**SLC25A51**	0.30	2.90E-01	1.00E+00	-0.37	3.23E-01	8.36E-01
**CLEC17A**	-2.98	3.79E-01	1.00E+00	-1.67	3.23E-01	8.36E-01
**TUBA8**	-0.55	7.21E-01	1.00E+00	1.05	3.23E-01	8.37E-01
**TP53TG1**	0.02	9.03E-01	1.00E+00	-0.35	3.23E-01	8.37E-01
**SENP6**	-0.04	8.62E-01	1.00E+00	-0.26	3.23E-01	8.37E-01
**DNTTIP2**	-0.18	3.38E-01	1.00E+00	-0.27	3.23E-01	8.37E-01
**TFAP4**	-0.05	8.44E-01	1.00E+00	0.39	3.23E-01	8.37E-01
**MRPS26**	0.03	8.87E-01	1.00E+00	-0.20	3.23E-01	8.37E-01
**CYP2T1P**	-0.19	5.92E-01	1.00E+00	0.34	3.23E-01	8.37E-01
**CNOT6**	0.18	4.92E-01	1.00E+00	0.37	3.23E-01	8.37E-01
**MAP3K14-AS1**	-0.95	4.99E-02	1.00E+00	-0.54	3.23E-01	8.37E-01
**SIN3A**	-0.10	5.97E-01	1.00E+00	0.29	3.23E-01	8.37E-01
**AL162386.2**	0.26	9.20E-01	1.00E+00	-1.29	3.23E-01	8.37E-01
**HUS1B**	-1.73	7.69E-02	1.00E+00	-0.76	3.23E-01	8.37E-01
**IDO2**	1.44	4.12E-01	1.00E+00	-1.60	3.23E-01	8.37E-01
**TSNARE1**	-0.55	9.84E-02	1.00E+00	-0.50	3.23E-01	8.37E-01
**ZNF443**	0.29	5.81E-01	1.00E+00	-0.34	3.23E-01	8.37E-01
**AC012676.3**	-3.46	1.96E-01	1.00E+00	-1.15	3.23E-01	8.37E-01
**KCTD14**	-1.93	1.04E-01	1.00E+00	-0.53	3.23E-01	8.37E-01
**NPC2**	-0.08	7.15E-01	1.00E+00	0.26	3.23E-01	8.37E-01

**OSTF1**	0.08	7.21E-01	1.00E+00	-0.16	3.23E-01	8.37E-01
**FKBP7**	0.12	7.45E-01	1.00E+00	0.34	3.23E-01	8.37E-01
**HIP1**	-0.12	5.72E-01	1.00E+00	0.35	3.23E-01	8.37E-01
**RBBP7**	0.02	9.32E-01	1.00E+00	-0.26	3.24E-01	8.37E-01
**CINP**	-0.01	9.47E-01	1.00E+00	0.16	3.24E-01	8.37E-01
**ZNRD1**	0.36	2.73E-01	1.00E+00	-0.25	3.24E-01	8.37E-01
**MAGI1-IT1**	0.24	8.00E-01	1.00E+00	-0.94	3.24E-01	8.37E-01
**RPL21P119**	-0.24	7.42E-01	1.00E+00	-0.56	3.24E-01	8.37E-01
**WNT10B**	-0.15	7.90E-01	1.00E+00	-0.42	3.24E-01	8.37E-01
**UQCRFS1P1**	-0.69	1.42E-01	1.00E+00	0.38	3.24E-01	8.37E-01
**TRPM1**	-0.26	7.08E-01	1.00E+00	0.63	3.24E-01	8.37E-01
**SWAP70**	0.33	1.06E-01	1.00E+00	-0.19	3.24E-01	8.37E-01
**EXOSC5**	0.00	9.89E-01	1.00E+00	0.22	3.24E-01	8.37E-01
**TM4SF19**	-2.37	4.86E-01	1.00E+00	1.21	3.24E-01	8.37E-01
**ZNF776**	0.07	7.32E-01	1.00E+00	-0.26	3.24E-01	8.37E-01
**TMEM132B**	0.02	9.51E-01	1.00E+00	-0.53	3.24E-01	8.38E-01
**FABP5P2**	0.24	8.98E-01	1.00E+00	0.54	3.24E-01	8.38E-01
**BLOC1S4**	-0.24	3.71E-01	1.00E+00	-0.57	3.24E-01	8.38E-01
**PHF20L1**	0.11	5.71E-01	1.00E+00	-0.25	3.24E-01	8.38E-01
**ARGLU1**	-0.19	5.08E-01	1.00E+00	0.18	3.24E-01	8.38E-01
**MEPCE**	-0.20	2.13E-01	1.00E+00	0.43	3.24E-01	8.38E-01
**DCHS2**	0.16	7.65E-01	1.00E+00	0.82	3.25E-01	8.38E-01
**PARP12**	-0.10	8.45E-01	1.00E+00	0.65	3.25E-01	8.38E-01
**BMP7**	-0.02	9.71E-01	1.00E+00	-0.48	3.25E-01	8.38E-01
**PEG13**	-0.54	3.59E-01	1.00E+00	0.97	3.25E-01	8.38E-01
**ZNF718**	0.39	6.09E-01	1.00E+00	-0.45	3.25E-01	8.38E-01
**NSDHL**	-0.28	3.31E-01	1.00E+00	-0.14	3.25E-01	8.38E-01
**MAST2**	-0.28	3.43E-01	1.00E+00	0.53	3.25E-01	8.38E-01
**MED18**	-0.21	3.56E-01	1.00E+00	0.34	3.25E-01	8.38E-01
**SDAD1**	-0.03	8.59E-01	1.00E+00	-0.20	3.25E-01	8.38E-01
**CSF2**	-0.32	8.80E-01	1.00E+00	1.07	3.25E-01	8.38E-01
**AL365436.1**	-2.12	3.51E-01	1.00E+00	-1.00	3.25E-01	8.38E-01

**EFHC2**	0.16	7.50E-01	1.00E+00	-0.60	3.25E-01	8.38E-01
**G25089**	0.39	7.17E-01	1.00E+00	-0.86	3.25E-01	8.39E-01
**G5146**	0.49	3.92E-01	1.00E+00	0.63	3.25E-01	8.39E-01
**AC007318.1**	0.37	4.83E-01	1.00E+00	-0.39	3.25E-01	8.39E-01
**IMP3**	0.03	9.03E-01	1.00E+00	-0.37	3.25E-01	8.39E-01
**POU3F3**	-0.62	3.73E-01	1.00E+00	-0.81	3.26E-01	8.39E-01
**ANKS1A**	0.05	8.06E-01	1.00E+00	0.30	3.26E-01	8.39E-01
**PDLIM1**	-0.16	4.58E-01	1.00E+00	0.15	3.26E-01	8.39E-01
**MDFIC**	0.37	1.22E-01	1.00E+00	-0.52	3.26E-01	8.39E-01
**KDM1B**	-0.06	8.54E-01	1.00E+00	0.44	3.26E-01	8.39E-01
**NME3**	-0.05	8.18E-01	1.00E+00	0.21	3.26E-01	8.39E-01
**EHBP1**	0.14	7.18E-01	1.00E+00	-0.31	3.26E-01	8.39E-01
**SLC6A20**	-0.41	5.74E-01	1.00E+00	1.20	3.26E-01	8.39E-01
**ZNF14**	0.40	3.53E-01	1.00E+00	-0.37	3.26E-01	8.39E-01
**CXXC1**	-0.16	4.83E-01	1.00E+00	0.30	3.26E-01	8.39E-01
**RPL35P1**	0.87	7.38E-01	1.00E+00	-0.85	3.26E-01	8.39E-01
**CPSF7**	-0.46	6.76E-02	1.00E+00	0.24	3.26E-01	8.39E-01
**RUNX3**	-0.62	6.53E-02	1.00E+00	0.57	3.26E-01	8.39E-01
**RASSF10**	-0.25	3.65E-01	1.00E+00	0.75	3.26E-01	8.39E-01
**PTCD3**	0.11	5.71E-01	1.00E+00	-0.21	3.26E-01	8.40E-01
**FAM27A**	-0.66	4.31E-01	1.00E+00	-0.65	3.26E-01	8.40E-01
**ADGRA3**	-0.02	9.48E-01	1.00E+00	-0.46	3.26E-01	8.40E-01
**GABRQ**	-0.53	2.89E-01	1.00E+00	0.80	3.26E-01	8.40E-01
**PSTPIP2**	0.04	9.14E-01	1.00E+00	0.36	3.26E-01	8.40E-01
**ENG**	-0.33	3.77E-01	1.00E+00	0.55	3.27E-01	8.40E-01
**SIMC1**	-0.22	4.62E-01	1.00E+00	-0.62	3.27E-01	8.40E-01
**CFAP46**	0.25	7.63E-01	1.00E+00	0.87	3.27E-01	8.40E-01
**AC099548.2**	-0.07	9.35E-01	1.00E+00	0.48	3.27E-01	8.40E-01
**G37023**	-0.46	7.83E-01	1.00E+00	1.41	3.27E-01	8.40E-01
**AQP7**	3.43	1.03E-02	6.32E-01	-0.84	3.27E-01	8.40E-01
**SYCE3**	0.84	4.32E-01	1.00E+00	-0.65	3.27E-01	8.40E-01
**HABP4**	0.01	9.80E-01	1.00E+00	-0.65	3.27E-01	8.40E-01

**EPS15**	0.05	7.81E-01	1.00E+00	-0.17	3.27E-01	8.40E-01
**LINC00506**	-0.07	9.58E-01	1.00E+00	1.09	3.27E-01	8.40E-01
**ORAI3**	0.34	2.57E-01	1.00E+00	-0.34	3.27E-01	8.40E-01
**GOLGA4**	-0.31	2.82E-01	1.00E+00	-0.29	3.27E-01	8.40E-01
**AC022898.2**	-0.54	8.75E-01	1.00E+00	-1.74	3.27E-01	8.40E-01
**JADE1**	0.16	3.79E-01	1.00E+00	-0.32	3.27E-01	8.40E-01
**GINS1**	-0.28	4.07E-01	1.00E+00	0.47	3.27E-01	8.40E-01
**RIOK3**	0.07	7.44E-01	1.00E+00	-0.17	3.27E-01	8.40E-01
**SFRP1**	0.88	2.41E-01	1.00E+00	0.55	3.27E-01	8.40E-01
**ZP1**	0.63	6.52E-01	1.00E+00	-1.13	3.27E-01	8.40E-01
**XLOC_002440**	-2.37	2.36E-01	1.00E+00	0.91	3.27E-01	8.40E-01
**FGF13**	-0.10	8.84E-01	1.00E+00	-0.62	3.27E-01	8.40E-01
**ARMC6**	-0.19	4.39E-01	1.00E+00	0.32	3.27E-01	8.40E-01
**RRP9**	-0.16	5.11E-01	1.00E+00	0.24	3.27E-01	8.40E-01
**SRPX2**	1.74	1.32E-02	7.22E-01	0.62	3.27E-01	8.40E-01
**CLEC4G**	0.61	3.19E-01	1.00E+00	0.81	3.27E-01	8.40E-01
**AC004148.2**	0.48	7.39E-01	1.00E+00	1.14	3.28E-01	8.40E-01
**MOB3C**	0.32	3.54E-01	1.00E+00	0.47	3.28E-01	8.40E-01
**AC138150.2**	0.43	7.36E-01	1.00E+00	0.74	3.28E-01	8.40E-01
**PTEN**	0.03	8.81E-01	1.00E+00	-0.47	3.28E-01	8.40E-01
**KIAA1109**	-0.10	6.32E-01	1.00E+00	0.35	3.28E-01	8.40E-01
**NKAPL**	0.37	5.02E-01	1.00E+00	-0.42	3.28E-01	8.40E-01
**CNNM1**	-0.87	9.35E-04	1.56E-01	-0.51	3.28E-01	8.40E-01
**FAM19A3**	-2.10	7.63E-02	1.00E+00	1.07	3.28E-01	8.40E-01
**CCM2**	-0.12	5.72E-01	1.00E+00	0.48	3.28E-01	8.40E-01
**LINC01278**	-0.03	9.43E-01	1.00E+00	-0.20	3.28E-01	8.40E-01
**CCDC32**	0.36	2.52E-01	1.00E+00	0.30	3.28E-01	8.40E-01
**KRT87P**	1.08	5.66E-01	1.00E+00	-1.39	3.28E-01	8.40E-01
**TAL1**	0.51	2.81E-01	1.00E+00	0.79	3.28E-01	8.40E-01
**CXXC4**	-0.23	8.42E-01	1.00E+00	-0.63	3.28E-01	8.40E-01
**ZNF343**	0.13	5.99E-01	1.00E+00	0.25	3.28E-01	8.40E-01
**AL031667.3**	-1.72	1.23E-01	1.00E+00	0.80	3.28E-01	8.40E-01

**INIP**	0.26	1.94E-01	1.00E+00	-0.30	3.28E-01	8.40E-01
**MDC1**	-0.48	1.65E-02	8.08E-01	0.38	3.28E-01	8.40E-01
**TREML1**	-0.76	6.24E-01	1.00E+00	0.88	3.28E-01	8.41E-01
**XLOC_012736**	-0.12	8.96E-01	1.00E+00	0.94	3.29E-01	8.41E-01
**AP002847.1**	1.04	5.04E-01	1.00E+00	1.20	3.29E-01	8.41E-01
**RARA-AS1**	-0.75	3.56E-01	1.00E+00	-0.88	3.29E-01	8.41E-01
**VPS36**	0.10	7.19E-01	1.00E+00	-0.25	3.29E-01	8.41E-01
**TRMT5**	0.02	9.42E-01	1.00E+00	0.19	3.29E-01	8.41E-01
**ZRANB2**	0.10	7.00E-01	1.00E+00	-0.34	3.29E-01	8.41E-01
**MRPL18**	0.04	7.87E-01	1.00E+00	-0.24	3.29E-01	8.41E-01
**MAGOHB**	-0.07	7.35E-01	1.00E+00	-0.28	3.29E-01	8.41E-01
**CCDC8**	0.02	9.76E-01	1.00E+00	0.63	3.29E-01	8.41E-01
**RAB40C**	-0.28	5.11E-01	1.00E+00	-0.39	3.29E-01	8.41E-01
**LYPD5**	-0.18	7.53E-01	1.00E+00	0.47	3.29E-01	8.41E-01
**PDCD6**	0.19	2.31E-01	1.00E+00	-0.36	3.29E-01	8.41E-01
**NCAM2**	-0.12	7.25E-01	1.00E+00	0.78	3.29E-01	8.41E-01
**NAXD**	0.16	3.16E-01	1.00E+00	-0.17	3.29E-01	8.41E-01
**AC138409.1**	1.84	2.01E-01	1.00E+00	-0.67	3.29E-01	8.41E-01
**AL356215.1**	-0.57	3.66E-01	1.00E+00	-1.00	3.29E-01	8.41E-01
**ITGA2B**	-0.07	9.25E-01	1.00E+00	-1.01	3.29E-01	8.41E-01
**G21034**	2.37	2.58E-01	1.00E+00	1.86	3.29E-01	8.42E-01
**CTAGE8**	0.84	9.84E-02	1.00E+00	0.81	3.29E-01	8.42E-01
**GAPDHP49**	0.01	9.90E-01	1.00E+00	-0.78	3.29E-01	8.42E-01
**SKOR1**	-0.99	1.37E-01	1.00E+00	1.22	3.29E-01	8.42E-01
**RALGAPA2**	0.28	1.73E-01	1.00E+00	0.33	3.29E-01	8.42E-01
**AL157935.1**	-0.20	7.79E-01	1.00E+00	0.87	3.30E-01	8.42E-01
**LAMB2**	0.23	5.29E-01	1.00E+00	0.54	3.30E-01	8.42E-01
**RPL23AP82**	0.12	6.20E-01	1.00E+00	-0.25	3.30E-01	8.42E-01
**XLOC_006333**	-2.73	2.21E-01	1.00E+00	-1.31	3.30E-01	8.42E-01
**TMEM50B**	0.26	3.42E-01	1.00E+00	-0.34	3.30E-01	8.42E-01
**XLOC_007725**	1.39	1.80E-01	1.00E+00	-0.97	3.30E-01	8.42E-01
**PPP2R1A**	-0.20	3.19E-01	1.00E+00	0.11	3.30E-01	8.42E-01

**KCNA1**	-1.11	3.70E-01	1.00E+00	1.35	3.30E-01	8.42E-01
**BLVRA**	-0.12	5.25E-01	1.00E+00	0.17	3.30E-01	8.42E-01
**FAM13A**	0.39	3.91E-01	1.00E+00	-0.31	3.30E-01	8.42E-01
**CYBA**	0.10	7.67E-01	1.00E+00	0.92	3.30E-01	8.42E-01
**PPP6R3**	-0.01	9.56E-01	1.00E+00	0.17	3.30E-01	8.42E-01
**TDG**	0.14	6.17E-01	1.00E+00	0.20	3.30E-01	8.42E-01
**MT-TT**	NA	NA	NA	-0.80	3.30E-01	8.42E-01
**LRRC73**	-0.46	5.19E-01	1.00E+00	1.09	3.30E-01	8.42E-01
**AC068888.1**	0.33	5.04E-01	1.00E+00	-0.45	3.30E-01	8.42E-01
**MYLPF**	-1.31	9.45E-02	1.00E+00	-0.54	3.30E-01	8.42E-01
**AC100861.1**	-0.23	6.83E-01	1.00E+00	0.33	3.30E-01	8.42E-01
**CACNA2D3**	-0.52	3.70E-01	1.00E+00	0.79	3.30E-01	8.42E-01
**PGRMC1**	0.09	6.46E-01	1.00E+00	-0.20	3.31E-01	8.42E-01
**AC103810.2**	-1.29	5.81E-01	1.00E+00	-0.84	3.31E-01	8.42E-01
**MZT1**	0.36	4.16E-01	1.00E+00	-0.33	3.31E-01	8.42E-01
**IFT57**	-0.10	6.89E-01	1.00E+00	-0.23	3.31E-01	8.42E-01
**NMD3**	0.07	7.41E-01	1.00E+00	-0.25	3.31E-01	8.42E-01
**ELL2**	-0.09	8.06E-01	1.00E+00	0.31	3.31E-01	8.42E-01
**HSD11B1L**	-0.87	3.74E-02	1.00E+00	-0.46	3.31E-01	8.42E-01
**EIF6**	-0.07	8.00E-01	1.00E+00	0.33	3.31E-01	8.42E-01
**LY75**	0.00	1.00E+00	1.00E+00	-0.30	3.31E-01	8.42E-01
**BX571818.1**	-0.07	9.46E-01	1.00E+00	1.01	3.31E-01	8.42E-01
**CCZ1B**	0.00	9.91E-01	1.00E+00	-0.51	3.31E-01	8.42E-01
**DHX8**	-0.22	3.05E-01	1.00E+00	0.27	3.31E-01	8.42E-01
**ATP5F1D**	-0.33	1.55E-01	1.00E+00	-0.18	3.31E-01	8.42E-01
**AWAT2**	0.27	9.32E-01	1.00E+00	-3.05	3.31E-01	8.42E-01
**XLOC_013541**	-1.53	1.42E-01	1.00E+00	1.12	3.31E-01	8.42E-01
**AC011374.2**	0.39	5.54E-01	1.00E+00	-0.52	3.31E-01	8.42E-01
**SMOC1**	0.63	3.70E-01	1.00E+00	0.75	3.31E-01	8.42E-01
**KLHL35**	-0.20	7.38E-01	1.00E+00	-0.45	3.31E-01	8.42E-01
**ASAH2**	-0.69	4.42E-01	1.00E+00	-0.61	3.31E-01	8.42E-01
**SLC16A11**	0.10	7.96E-01	1.00E+00	-0.51	3.31E-01	8.42E-01

**TOPORS**	-0.27	2.70E-01	1.00E+00	-0.24	3.31E-01	8.42E-01
**PPIAP19**	-1.12	7.18E-01	1.00E+00	0.91	3.31E-01	8.42E-01
**AC015987.1**	1.48	6.63E-01	1.00E+00	-1.11	3.31E-01	8.42E-01
**MAN2A1**	0.03	9.14E-01	1.00E+00	0.21	3.31E-01	8.42E-01
**ERCC6L**	0.08	8.53E-01	1.00E+00	0.74	3.31E-01	8.42E-01
**PPP3R1**	0.02	9.28E-01	1.00E+00	0.47	3.31E-01	8.42E-01
**ACVR2B**	0.49	2.79E-01	1.00E+00	0.42	3.31E-01	8.42E-01
**TNPO1P1**	0.90	7.96E-01	1.00E+00	-1.48	3.31E-01	8.42E-01
**LINC00987**	-3.08	2.56E-02	9.30E-01	-0.70	3.31E-01	8.42E-01
**PADI2**	0.33	7.51E-01	1.00E+00	-0.59	3.31E-01	8.42E-01
**PCDH7**	-0.34	3.47E-01	1.00E+00	0.38	3.31E-01	8.42E-01
**MARF1**	0.40	8.96E-02	1.00E+00	0.33	3.31E-01	8.42E-01
**AC008555.2**	2.97	2.26E-01	1.00E+00	-0.54	3.31E-01	8.42E-01
**PPP1R26**	0.13	7.07E-01	1.00E+00	0.69	3.32E-01	8.42E-01
**NMUR1**	1.35	1.30E-01	1.00E+00	-0.87	3.32E-01	8.42E-01
**AC010615.2**	0.66	4.65E-01	1.00E+00	-0.91	3.32E-01	8.42E-01
**TMED4**	0.11	4.22E-01	1.00E+00	-0.21	3.32E-01	8.42E-01
**ERMN**	-5.42	8.19E-09	3.93E-06	1.66	3.32E-01	8.42E-01
**MIR103A2**	0.07	9.49E-01	1.00E+00	0.39	3.32E-01	8.42E-01
**WIPI1**	-0.07	8.29E-01	1.00E+00	0.27	3.32E-01	8.42E-01
**ZNF33A**	-0.07	7.96E-01	1.00E+00	-0.26	3.32E-01	8.42E-01
**LINC01142**	-1.00	4.45E-01	1.00E+00	0.78	3.32E-01	8.42E-01
**KLF3-AS1**	-0.17	7.32E-01	1.00E+00	-0.47	3.32E-01	8.42E-01
**AC063944.2**	-3.44	8.39E-02	1.00E+00	1.39	3.32E-01	8.42E-01
**C16orf74**	-0.47	3.54E-01	1.00E+00	-0.38	3.32E-01	8.42E-01
**FXYD1**	-0.23	9.07E-01	1.00E+00	0.77	3.32E-01	8.42E-01
**AC244258.1**	-4.70	1.81E-02	8.45E-01	-0.50	3.32E-01	8.42E-01
**SH2D5**	-4.10	4.07E-02	1.00E+00	3.07	3.32E-01	8.42E-01
**TRIM52**	0.10	6.99E-01	1.00E+00	-0.24	3.32E-01	8.42E-01
**BAHD1**	-0.06	8.04E-01	1.00E+00	0.46	3.32E-01	8.42E-01
**NUP210L**	-2.71	3.31E-01	1.00E+00	-1.42	3.32E-01	8.42E-01
**Z97989.1**	-0.19	8.03E-01	1.00E+00	0.57	3.32E-01	8.42E-01

**AL122023.1**	1.81	5.80E-02	1.00E+00	-0.92	3.32E-01	8.42E-01
**AL035701.1**	-1.06	5.35E-01	1.00E+00	-1.17	3.32E-01	8.42E-01
**ANKRD26**	-0.01	9.77E-01	1.00E+00	-0.34	3.32E-01	8.43E-01
**TTC37**	0.16	5.44E-01	1.00E+00	-0.17	3.33E-01	8.43E-01
**NTPCR**	0.11	5.31E-01	1.00E+00	-0.28	3.33E-01	8.43E-01
**ATG10**	-0.14	6.22E-01	1.00E+00	-0.27	3.33E-01	8.43E-01
**ALDH4A1**	0.09	7.96E-01	1.00E+00	-0.38	3.33E-01	8.43E-01
**CARM1**	-0.45	1.82E-02	8.48E-01	0.63	3.33E-01	8.43E-01
**SLC25A29**	-0.32	5.12E-01	1.00E+00	-0.64	3.33E-01	8.43E-01
**LTB4R**	-0.05	8.85E-01	1.00E+00	0.57	3.33E-01	8.43E-01
**CCDC93**	0.41	3.73E-01	1.00E+00	0.24	3.33E-01	8.43E-01
**NSL1**	-0.03	8.73E-01	1.00E+00	-0.26	3.33E-01	8.43E-01
**SLC7A14**	-0.28	8.34E-01	1.00E+00	-1.69	3.33E-01	8.43E-01
**AP006296.1**	1.17	5.98E-01	1.00E+00	-0.84	3.33E-01	8.43E-01
**TLE2**	-0.07	8.95E-01	1.00E+00	-0.61	3.33E-01	8.43E-01
**H3F3C**	-0.43	7.70E-01	1.00E+00	-0.66	3.33E-01	8.43E-01
**SPHK2**	0.03	9.45E-01	1.00E+00	0.63	3.33E-01	8.43E-01
**SPSB3**	0.11	8.70E-01	1.00E+00	0.45	3.33E-01	8.43E-01
**TRIM36**	0.12	8.37E-01	1.00E+00	-0.68	3.33E-01	8.43E-01
**AC103702.1**	0.39	8.13E-01	1.00E+00	-1.20	3.33E-01	8.43E-01
**PARN**	-0.08	6.28E-01	1.00E+00	-0.16	3.33E-01	8.43E-01
**HSPB3**	0.47	2.24E-01	1.00E+00	0.64	3.33E-01	8.43E-01
**AC103770.1**	0.35	9.19E-01	1.00E+00	1.48	3.33E-01	8.43E-01
**RANBP10**	-0.01	9.67E-01	1.00E+00	0.49	3.33E-01	8.43E-01
**UBA2**	0.09	6.27E-01	1.00E+00	-0.36	3.33E-01	8.43E-01
**AC011815.1**	-0.74	7.36E-02	1.00E+00	-0.56	3.33E-01	8.43E-01
**OGFOD2**	-0.13	9.59E-01	1.00E+00	0.62	3.33E-01	8.43E-01
**TGFB1I1**	-0.14	7.43E-01	1.00E+00	0.36	3.34E-01	8.44E-01
**EXOC5P1**	1.26	6.84E-01	1.00E+00	-0.68	3.34E-01	8.44E-01
**VTN**	-0.30	7.67E-01	1.00E+00	1.21	3.34E-01	8.44E-01
**NEK11**	0.25	2.48E-01	1.00E+00	0.33	3.34E-01	8.44E-01
**CADM4**	-0.15	4.60E-01	1.00E+00	0.67	3.34E-01	8.44E-01

**AL445423.1**	1.91	1.52E-01	1.00E+00	0.94	3.34E-01	8.44E-01
**EEA1**	-0.14	5.51E-01	1.00E+00	-0.20	3.34E-01	8.44E-01
**G33789**	-0.26	7.03E-01	1.00E+00	1.17	3.34E-01	8.44E-01
**AC022467.1**	-0.19	9.37E-01	1.00E+00	-1.66	3.34E-01	8.44E-01
**DHX40**	0.07	7.28E-01	1.00E+00	-0.50	3.34E-01	8.44E-01
**G42509**	1.32	6.99E-01	1.00E+00	0.75	3.34E-01	8.45E-01
**TRBV19**	-3.21	4.06E-02	1.00E+00	0.93	3.34E-01	8.45E-01
**CPLANE2**	0.20	5.34E-01	1.00E+00	0.69	3.35E-01	8.45E-01
**TMC6**	-0.19	4.76E-01	1.00E+00	0.51	3.35E-01	8.45E-01
**PCLO**	-1.44	1.45E-03	2.09E-01	-0.47	3.35E-01	8.45E-01
**IRX6**	0.37	4.82E-01	1.00E+00	-0.47	3.35E-01	8.45E-01
**AL353729.2**	-1.19	7.32E-01	1.00E+00	1.23	3.35E-01	8.45E-01
**NHEJ1**	-1.41	6.33E-01	1.00E+00	0.98	3.35E-01	8.45E-01
**MGAT4C**	-0.54	6.52E-01	1.00E+00	0.82	3.35E-01	8.45E-01
**TIMM22**	-0.05	8.30E-01	1.00E+00	-0.24	3.35E-01	8.45E-01
**AC008124.1**	0.15	5.28E-01	1.00E+00	-0.34	3.35E-01	8.45E-01
**CYFIP2**	-0.75	6.01E-02	1.00E+00	0.50	3.35E-01	8.45E-01
**XLOC_009180**	1.03	5.76E-01	1.00E+00	1.08	3.35E-01	8.45E-01
**MIS18A**	0.21	3.08E-01	1.00E+00	-0.28	3.35E-01	8.45E-01
**AL357054.1**	-0.80	8.18E-01	1.00E+00	3.03	3.35E-01	8.45E-01
**RIC3**	-0.05	8.61E-01	1.00E+00	-0.42	3.35E-01	8.45E-01
**SAMD10**	-0.28	3.71E-01	1.00E+00	-0.35	3.35E-01	8.45E-01
**LYNX1-SLURP2**	-0.59	6.86E-01	1.00E+00	-0.59	3.35E-01	8.45E-01
**XLOC_000411**	1.26	5.59E-02	1.00E+00	-0.71	3.35E-01	8.45E-01
**AP001046.1**	-0.49	5.65E-01	1.00E+00	1.22	3.35E-01	8.45E-01
**CCT6P1**	-0.51	5.23E-01	1.00E+00	-0.43	3.35E-01	8.45E-01
**SRRM1P3**	NA	NA	NA	-0.89	3.35E-01	8.45E-01
**TUSC1**	-0.04	8.77E-01	1.00E+00	-0.54	3.35E-01	8.45E-01
**ZNF638**	0.01	9.76E-01	1.00E+00	-0.15	3.35E-01	8.45E-01
**ZBTB6**	0.52	5.22E-02	1.00E+00	0.31	3.35E-01	8.45E-01
**LBR**	0.42	1.13E-01	1.00E+00	-0.26	3.35E-01	8.45E-01
**FAM92A**	-0.05	8.81E-01	1.00E+00	-0.38	3.36E-01	8.45E-01

**SNAPC2**	-0.22	1.90E-01	1.00E+00	0.34	3.36E-01	8.45E-01
**GOLGA6L4**	1.36	1.65E-01	1.00E+00	0.81	3.36E-01	8.45E-01
**LINC01412**	-0.35	9.17E-01	1.00E+00	-0.79	3.36E-01	8.45E-01
**LINC02017**	-0.49	7.31E-01	1.00E+00	-1.07	3.36E-01	8.45E-01
**AC099048.1**	0.65	8.22E-01	1.00E+00	0.75	3.36E-01	8.45E-01
**PLEKHO2**	0.37	3.38E-01	1.00E+00	0.67	3.36E-01	8.45E-01
**DLGAP1**	-0.64	4.65E-01	1.00E+00	0.67	3.36E-01	8.45E-01
**KDM5C**	-0.05	8.41E-01	1.00E+00	0.58	3.36E-01	8.45E-01
**FTLP14**	-0.66	4.62E-01	1.00E+00	-0.66	3.36E-01	8.45E-01
**RIPOR2**	-0.22	6.15E-01	1.00E+00	0.44	3.36E-01	8.45E-01
**MTO1**	0.08	7.06E-01	1.00E+00	-0.21	3.36E-01	8.45E-01
**RASGRP4**	0.07	8.69E-01	1.00E+00	0.76	3.36E-01	8.45E-01
**PPP2CB**	0.27	9.01E-02	1.00E+00	0.29	3.36E-01	8.45E-01
**NLGN1**	0.07	8.65E-01	1.00E+00	-0.53	3.36E-01	8.45E-01
**CLPSL2**	1.69	6.12E-01	1.00E+00	-1.84	3.36E-01	8.45E-01
**AC002066.1**	-1.14	4.02E-01	1.00E+00	-0.86	3.36E-01	8.45E-01
**DRC3**	-0.52	2.95E-01	1.00E+00	0.26	3.36E-01	8.45E-01
**XLOC_000160**	-3.20	4.73E-02	1.00E+00	-1.18	3.36E-01	8.45E-01
**FBXO3**	0.05	8.41E-01	1.00E+00	-0.35	3.36E-01	8.45E-01
**NCOR2**	-0.52	1.30E-01	1.00E+00	0.72	3.36E-01	8.45E-01
**TRIM27**	0.01	9.50E-01	1.00E+00	0.28	3.36E-01	8.45E-01
**PRMT3**	0.22	3.26E-01	1.00E+00	-0.18	3.36E-01	8.45E-01
**SLC35D3**	0.70	4.61E-01	1.00E+00	-1.55	3.36E-01	8.45E-01
**FAM25B**	-0.30	8.67E-01	1.00E+00	-0.70	3.36E-01	8.45E-01
**POU4F3**	-0.01	9.95E-01	1.00E+00	1.16	3.36E-01	8.45E-01
**IGHG4**	0.58	8.66E-01	1.00E+00	3.02	3.36E-01	8.45E-01
**NUAK1**	0.12	6.43E-01	1.00E+00	0.32	3.36E-01	8.45E-01
**AC114488.1**	-2.03	2.88E-01	1.00E+00	0.88	3.36E-01	8.45E-01
**TCEAL8**	-0.04	8.42E-01	1.00E+00	-0.30	3.36E-01	8.45E-01
**PHOSPHO1**	-0.44	5.93E-01	1.00E+00	0.96	3.36E-01	8.45E-01
**TGOLN2**	0.32	2.36E-01	1.00E+00	0.21	3.36E-01	8.45E-01
**ANKHD1**	0.45	5.07E-01	1.00E+00	-0.42	3.37E-01	8.45E-01

**IL15**	0.18	7.60E-01	1.00E+00	0.47	3.37E-01	8.45E-01
**CSRP1**	-0.24	6.26E-01	1.00E+00	-0.32	3.37E-01	8.45E-01
**SLC28A3**	0.47	4.34E-01	1.00E+00	0.54	3.37E-01	8.45E-01
**EFR3A**	0.35	1.61E-01	1.00E+00	-0.21	3.37E-01	8.45E-01
**AC109597.2**	-3.43	1.67E-01	1.00E+00	-1.45	3.37E-01	8.45E-01
**RNU1-28P**	1.83	4.95E-01	1.00E+00	-1.34	3.37E-01	8.45E-01
**SUMO1P3**	-1.30	3.31E-01	1.00E+00	-0.56	3.37E-01	8.45E-01
**ASTE1**	0.06	8.21E-01	1.00E+00	-0.38	3.37E-01	8.45E-01
**ELN-AS1**	0.52	8.30E-01	1.00E+00	1.09	3.37E-01	8.45E-01
**ARHGEF26**	0.21	5.25E-01	1.00E+00	-0.37	3.37E-01	8.45E-01
**CCDC141**	0.70	4.51E-01	1.00E+00	0.77	3.37E-01	8.45E-01
**TDRD12**	0.17	8.58E-01	1.00E+00	0.93	3.37E-01	8.46E-01
**B3GALT2**	-1.40	3.16E-01	1.00E+00	-0.91	3.37E-01	8.46E-01
**RARRES2**	1.71	5.67E-03	4.48E-01	0.52	3.37E-01	8.46E-01
**CIAPIN1P**	0.56	8.71E-01	1.00E+00	0.92	3.37E-01	8.46E-01
**BAK1**	0.24	3.46E-01	1.00E+00	0.39	3.37E-01	8.46E-01
**C7orf50**	-0.08	8.01E-01	1.00E+00	0.20	3.37E-01	8.46E-01
**KCNMB3**	-0.63	3.83E-01	1.00E+00	-0.65	3.38E-01	8.46E-01
**AC004816.2**	1.17	7.35E-01	1.00E+00	-1.09	3.38E-01	8.46E-01
**AC092127.2**	-0.87	4.44E-01	1.00E+00	-0.93	3.38E-01	8.46E-01
**LINC02320**	-2.25	2.66E-01	1.00E+00	-1.37	3.38E-01	8.46E-01
**CCDC82**	0.28	4.08E-01	1.00E+00	-0.26	3.38E-01	8.46E-01
**AC009065.5**	-1.64	6.28E-01	1.00E+00	-1.39	3.38E-01	8.46E-01
**ZNF771**	-0.19	4.18E-01	1.00E+00	0.25	3.38E-01	8.46E-01
**FAM201A**	-0.50	2.07E-01	1.00E+00	-0.76	3.38E-01	8.46E-01
**PF4**	-2.41	2.33E-01	1.00E+00	-1.08	3.38E-01	8.46E-01
**ARL6**	0.18	6.12E-01	1.00E+00	-0.34	3.38E-01	8.46E-01
**STOX1**	-0.79	1.00E-01	1.00E+00	-0.88	3.38E-01	8.46E-01
**USP27X**	-0.31	3.02E-01	1.00E+00	0.67	3.38E-01	8.46E-01
**AC122710.2**	0.09	9.61E-01	1.00E+00	1.25	3.38E-01	8.46E-01
**ELP4**	-0.05	8.32E-01	1.00E+00	-0.31	3.38E-01	8.46E-01
**KCNS2**	-0.46	5.06E-01	1.00E+00	1.26	3.38E-01	8.46E-01

**XLOC_000555**	0.99	4.68E-01	1.00E+00	1.28	3.38E-01	8.46E-01
**SMU1**	0.00	9.97E-01	1.00E+00	-0.14	3.38E-01	8.46E-01
**OBSL1**	-0.22	4.39E-01	1.00E+00	0.52	3.38E-01	8.46E-01
**AC010969.2**	-0.66	1.60E-01	1.00E+00	0.33	3.38E-01	8.46E-01
**MCEE**	0.03	9.28E-01	1.00E+00	-0.43	3.38E-01	8.46E-01
**ZSCAN29**	-0.07	8.04E-01	1.00E+00	0.42	3.38E-01	8.46E-01
**GAST**	-1.90	5.82E-01	1.00E+00	1.17	3.38E-01	8.46E-01
**ILF3-DT**	-0.10	6.52E-01	1.00E+00	-0.24	3.39E-01	8.46E-01
**G33562**	-0.47	5.99E-01	1.00E+00	0.70	3.39E-01	8.46E-01
**EPHA3**	0.24	6.55E-01	1.00E+00	0.60	3.39E-01	8.46E-01
**NKAIN4**	-1.44	2.30E-01	1.00E+00	0.96	3.39E-01	8.46E-01
**SLC15A4**	0.01	9.78E-01	1.00E+00	-0.38	3.39E-01	8.46E-01
**AC124016.2**	-1.40	1.29E-01	1.00E+00	-0.67	3.39E-01	8.46E-01
**KLHDC7A**	0.71	6.19E-01	1.00E+00	-1.20	3.39E-01	8.46E-01
**CFL2**	0.30	5.44E-01	1.00E+00	-0.41	3.39E-01	8.46E-01
**VPS54**	0.14	5.29E-01	1.00E+00	-0.34	3.39E-01	8.46E-01
**RPL5P34**	-0.63	5.29E-01	1.00E+00	-0.55	3.39E-01	8.46E-01
**AL391650.1**	-0.08	9.44E-01	1.00E+00	-1.18	3.39E-01	8.46E-01
**AC008957.1**	-0.38	7.77E-01	1.00E+00	1.27	3.39E-01	8.46E-01
**PHF2**	0.06	7.79E-01	1.00E+00	0.48	3.39E-01	8.46E-01
**LRCH2**	0.85	2.21E-01	1.00E+00	0.88	3.39E-01	8.46E-01
**G909**	0.89	3.04E-01	1.00E+00	1.19	3.39E-01	8.46E-01
**G13548**	-2.92	3.90E-01	1.00E+00	1.62	3.39E-01	8.46E-01
**AC008063.1**	-1.83	4.11E-01	1.00E+00	1.01	3.39E-01	8.46E-01
**PZP**	-0.08	9.64E-01	1.00E+00	-1.08	3.39E-01	8.46E-01
**KRT36**	-5.54	1.06E-03	1.68E-01	-1.63	3.39E-01	8.46E-01
**AC117386.1**	-1.27	7.04E-01	1.00E+00	1.30	3.39E-01	8.46E-01
**PCDHGB4**	-0.14	7.17E-01	1.00E+00	-0.49	3.39E-01	8.46E-01
**POLRMTP1**	-0.13	9.22E-01	1.00E+00	-0.70	3.39E-01	8.46E-01
**AL355075.4**	-0.25	5.40E-01	1.00E+00	-0.63	3.39E-01	8.46E-01
**IGLV8-61**	2.10	5.34E-01	1.00E+00	3.03	3.39E-01	8.46E-01
**SCIMP**	0.82	5.68E-01	1.00E+00	2.85	3.39E-01	8.46E-01

**ACSM1**	0.91	6.18E-02	1.00E+00	0.66	3.39E-01	8.46E-01
**ZNF296**	-0.74	2.09E-02	8.94E-01	-0.36	3.39E-01	8.46E-01
**MOK**	-0.20	4.68E-01	1.00E+00	-0.37	3.39E-01	8.46E-01
**AF129075.2**	NA	NA	NA	-1.22	3.39E-01	8.46E-01
**SLC38A1**	0.17	5.24E-01	1.00E+00	-0.29	3.39E-01	8.46E-01
**C1QTNF3**	1.98	1.40E-01	1.00E+00	0.66	3.39E-01	8.46E-01
**PTRHD1**	-0.20	6.02E-01	1.00E+00	-0.46	3.39E-01	8.46E-01
**TOMM20P2**	0.49	6.72E-01	1.00E+00	1.44	3.40E-01	8.46E-01
**GPC2**	0.01	9.90E-01	1.00E+00	0.64	3.40E-01	8.46E-01
**BX284668.2**	-0.30	6.52E-01	1.00E+00	-1.39	3.40E-01	8.46E-01
**CMTM7**	-0.43	1.98E-01	1.00E+00	0.24	3.40E-01	8.46E-01
**LXN**	-0.05	7.87E-01	1.00E+00	-0.30	3.40E-01	8.46E-01
**INPP4B**	0.12	6.49E-01	1.00E+00	0.34	3.40E-01	8.46E-01
**AL731556.2**	NA	NA	NA	2.62	3.40E-01	8.46E-01
**HIST1H2BK**	-0.68	2.21E-01	1.00E+00	0.46	3.40E-01	8.46E-01
**AL591846.1**	-5.53	2.14E-03	2.39E-01	-0.98	3.40E-01	8.47E-01
**SMIM1**	0.03	9.66E-01	1.00E+00	-0.48	3.40E-01	8.47E-01
**LINC00920**	-0.47	4.22E-01	1.00E+00	0.62	3.40E-01	8.47E-01
**G16026**	4.72	1.59E-01	1.00E+00	1.01	3.40E-01	8.47E-01
**ARAP3**	0.10	8.43E-01	1.00E+00	0.67	3.40E-01	8.47E-01
**EIF3A**	-0.01	9.77E-01	1.00E+00	0.22	3.40E-01	8.47E-01
**GLB1L**	0.01	9.74E-01	1.00E+00	0.29	3.40E-01	8.47E-01
**PPIP5K1**	-0.32	3.25E-02	1.00E+00	-0.34	3.40E-01	8.47E-01
**G1580**	-2.17	4.48E-01	1.00E+00	1.48	3.40E-01	8.47E-01
**ZNF644**	-0.03	9.12E-01	1.00E+00	-0.20	3.40E-01	8.47E-01
**ARF3**	0.05	7.54E-01	1.00E+00	0.31	3.40E-01	8.47E-01
**RBM5**	0.34	3.85E-01	1.00E+00	0.21	3.40E-01	8.47E-01
**MYL6**	0.17	3.80E-01	1.00E+00	-0.28	3.40E-01	8.47E-01
**CENPE**	-0.18	6.43E-01	1.00E+00	-0.46	3.41E-01	8.47E-01
**BTBD9**	-0.23	3.34E-01	1.00E+00	0.38	3.41E-01	8.47E-01
**AC093677.2**	-0.16	8.93E-01	1.00E+00	-0.59	3.41E-01	8.47E-01
**KCTD4**	0.57	3.90E-01	1.00E+00	0.75	3.41E-01	8.47E-01

**ZNF135**	0.14	7.32E-01	1.00E+00	-0.44	3.41E-01	8.47E-01
**XLOC_000647**	-0.49	2.23E-01	1.00E+00	0.88	3.41E-01	8.47E-01
**ARHGEF19**	-0.12	7.41E-01	1.00E+00	-0.40	3.41E-01	8.47E-01
**ZGRF1**	0.24	6.60E-01	1.00E+00	-0.61	3.41E-01	8.48E-01
**ANKRD10**	0.11	6.72E-01	1.00E+00	0.24	3.41E-01	8.48E-01
**IGFLR1**	1.28	6.35E-01	1.00E+00	0.55	3.41E-01	8.48E-01
**LINC00598**	2.26	1.92E-02	8.71E-01	-0.90	3.41E-01	8.48E-01
**CAMK1G**	-4.61	1.01E-03	1.65E-01	1.02	3.41E-01	8.48E-01
**NPTN**	-0.04	8.17E-01	1.00E+00	-0.58	3.41E-01	8.48E-01
**AC084024.4**	-0.78	4.41E-01	1.00E+00	0.65	3.41E-01	8.48E-01
**COA5**	0.24	2.15E-01	1.00E+00	-0.24	3.41E-01	8.48E-01
**AMOTL1**	0.05	8.61E-01	1.00E+00	0.34	3.42E-01	8.48E-01
**AC010504.1**	-0.46	7.02E-01	1.00E+00	-0.82	3.42E-01	8.49E-01
**COX4I2**	0.52	3.59E-01	1.00E+00	0.41	3.42E-01	8.49E-01
**CD109**	0.00	9.91E-01	1.00E+00	0.22	3.42E-01	8.49E-01
**FGD5**	0.22	5.98E-01	1.00E+00	0.57	3.42E-01	8.49E-01
**USH2A**	1.33	4.83E-01	1.00E+00	1.44	3.42E-01	8.49E-01
**CDO1**	1.91	3.94E-02	1.00E+00	-0.72	3.42E-01	8.49E-01
**KLF7-IT1**	-2.08	1.78E-01	1.00E+00	0.99	3.42E-01	8.49E-01
**CNIH1**	0.08	7.09E-01	1.00E+00	-0.21	3.42E-01	8.49E-01
**PPP1R3C**	-0.14	8.20E-01	1.00E+00	-0.43	3.42E-01	8.49E-01
**AL450992.3**	0.56	8.71E-01	1.00E+00	2.55	3.42E-01	8.49E-01
**C8G**	-0.56	5.51E-01	1.00E+00	0.96	3.42E-01	8.49E-01
**TIGD5**	0.00	9.99E-01	1.00E+00	0.48	3.42E-01	8.49E-01
**ZBTB26**	0.33	3.08E-01	1.00E+00	0.48	3.42E-01	8.49E-01
**MKKS**	0.25	3.64E-01	1.00E+00	-0.28	3.42E-01	8.49E-01
**SLC5A1**	0.32	5.06E-01	1.00E+00	0.48	3.42E-01	8.49E-01
**COQ6**	0.58	2.69E-01	1.00E+00	-0.39	3.42E-01	8.49E-01
**AC009041.4**	-1.61	3.19E-01	1.00E+00	1.40	3.42E-01	8.49E-01
**DLGAP2**	-0.86	2.48E-01	1.00E+00	0.58	3.43E-01	8.49E-01
**MPRIP**	0.00	9.95E-01	1.00E+00	0.55	3.43E-01	8.49E-01
**EIPR1**	-0.14	5.64E-01	1.00E+00	0.19	3.43E-01	8.49E-01

**PKMYT1**	-0.64	1.89E-01	1.00E+00	0.58	3.43E-01	8.49E-01
**MCL1**	-0.11	7.12E-01	1.00E+00	0.45	3.43E-01	8.49E-01
**PPIH**	0.04	9.14E-01	1.00E+00	-0.16	3.43E-01	8.49E-01
**KCNA7**	0.89	4.91E-01	1.00E+00	1.58	3.43E-01	8.49E-01
**ELF2**	0.00	9.89E-01	1.00E+00	-0.26	3.43E-01	8.49E-01
**WTAPP1**	-1.75	3.31E-01	1.00E+00	1.01	3.43E-01	8.49E-01
**MAP1S**	0.04	8.90E-01	1.00E+00	0.57	3.43E-01	8.49E-01
**ASIC2**	-1.04	2.33E-01	1.00E+00	1.16	3.43E-01	8.49E-01
**LNP1**	0.29	5.86E-01	1.00E+00	0.62	3.43E-01	8.49E-01
**G36147**	-0.40	7.50E-01	1.00E+00	-3.02	3.43E-01	8.49E-01
**TRAV26-1**	2.49	2.29E-01	1.00E+00	1.52	3.43E-01	8.49E-01
**TTLL7**	-0.15	7.93E-01	1.00E+00	-0.41	3.43E-01	8.49E-01
**EXOSC3P1**	-0.39	9.10E-01	1.00E+00	-0.96	3.43E-01	8.49E-01
**UMODL1**	0.29	7.55E-01	1.00E+00	-1.04	3.43E-01	8.49E-01
**PTPN4**	0.39	1.20E-01	1.00E+00	-0.27	3.43E-01	8.49E-01
**GHR**	1.18	4.33E-02	1.00E+00	-0.59	3.43E-01	8.49E-01
**YBX1P4**	-0.38	8.87E-01	1.00E+00	1.77	3.43E-01	8.49E-01
**HES1**	-0.37	3.56E-01	1.00E+00	-0.50	3.43E-01	8.49E-01
**DCAF13**	-0.08	7.48E-01	1.00E+00	-0.31	3.43E-01	8.49E-01
**GPR161**	-0.01	9.85E-01	1.00E+00	0.31	3.44E-01	8.49E-01
**SERPINB2**	0.48	3.30E-01	1.00E+00	-0.58	3.44E-01	8.49E-01
**SHC3**	0.09	8.78E-01	1.00E+00	0.43	3.44E-01	8.49E-01
**ERGIC1**	0.18	4.69E-01	1.00E+00	0.44	3.44E-01	8.49E-01
**AC245100.8**	-0.50	1.07E-01	1.00E+00	0.48	3.44E-01	8.49E-01
**WDR17**	0.09	9.10E-01	1.00E+00	-0.86	3.44E-01	8.49E-01
**GFM1**	-0.03	9.08E-01	1.00E+00	-0.16	3.44E-01	8.49E-01
**EXOSC8**	-0.06	8.01E-01	1.00E+00	-0.40	3.44E-01	8.49E-01
**WNT10A**	-0.20	5.95E-01	1.00E+00	-0.81	3.44E-01	8.49E-01
**AC092437.1**	-0.10	9.40E-01	1.00E+00	1.08	3.44E-01	8.49E-01
**TM2D3**	0.34	6.53E-02	1.00E+00	-0.26	3.44E-01	8.49E-01
**ATP6V0D1**	-0.16	4.02E-01	1.00E+00	0.12	3.44E-01	8.49E-01
**EPN2**	0.10	7.51E-01	1.00E+00	-0.24	3.44E-01	8.49E-01

**G12287**	-1.09	4.92E-01	1.00E+00	0.90	3.44E-01	8.49E-01
**ZNF589**	-0.13	6.30E-01	1.00E+00	0.39	3.44E-01	8.49E-01
**MVK**	-0.21	5.55E-01	1.00E+00	0.27	3.44E-01	8.49E-01
**LINC00574**	-0.52	8.06E-01	1.00E+00	1.25	3.44E-01	8.49E-01
**AC090825.1**	-0.18	7.71E-01	1.00E+00	0.71	3.44E-01	8.49E-01
**RAPGEFL1**	-0.03	9.46E-01	1.00E+00	-0.65	3.44E-01	8.49E-01
**LTB4R2**	-0.14	7.36E-01	1.00E+00	-0.58	3.44E-01	8.50E-01
**XLOC_003668**	0.19	8.01E-01	1.00E+00	1.51	3.44E-01	8.50E-01
**CSNK2A2**	0.05	9.07E-01	1.00E+00	0.30	3.44E-01	8.50E-01
**AC008764.2**	-0.25	5.85E-01	1.00E+00	0.99	3.44E-01	8.50E-01
**IGHV4-59**	1.73	3.32E-01	1.00E+00	2.98	3.45E-01	8.50E-01
**LCE1F**	0.20	7.80E-01	1.00E+00	0.74	3.45E-01	8.50E-01
**CASP8AP2**	-0.05	8.45E-01	1.00E+00	-0.24	3.45E-01	8.50E-01
**HCST**	-2.04	4.72E-01	1.00E+00	0.54	3.45E-01	8.50E-01
**PORCN**	-0.36	1.07E-01	1.00E+00	0.18	3.45E-01	8.50E-01
**AC093218.1**	0.44	4.97E-01	1.00E+00	0.96	3.45E-01	8.50E-01
**SLC35A1**	0.10	7.24E-01	1.00E+00	-0.40	3.45E-01	8.50E-01
**LRRC69**	1.10	1.33E-01	1.00E+00	-0.55	3.45E-01	8.50E-01
**DPRXP4**	0.81	6.78E-01	1.00E+00	1.20	3.45E-01	8.50E-01
**SLC29A1**	0.20	5.84E-01	1.00E+00	-0.30	3.45E-01	8.50E-01
**GAS7**	0.06	8.63E-01	1.00E+00	0.34	3.45E-01	8.50E-01
**NAPSA**	0.89	4.79E-01	1.00E+00	1.23	3.45E-01	8.50E-01
**SEMA4C**	-0.26	2.82E-01	1.00E+00	0.54	3.45E-01	8.50E-01
**PPP4C**	-0.12	6.14E-01	1.00E+00	-0.38	3.45E-01	8.50E-01
**LRIF1**	0.11	6.60E-01	1.00E+00	-0.26	3.45E-01	8.50E-01
**AC009403.2**	-1.61	6.41E-01	1.00E+00	0.94	3.45E-01	8.50E-01
**TBC1D31**	-0.03	9.31E-01	1.00E+00	-0.51	3.45E-01	8.50E-01
**AL355102.1**	30.00	6.58E-18	5.54E-15	1.21	3.45E-01	8.50E-01
**B2M**	0.25	4.75E-01	1.00E+00	0.30	3.45E-01	8.50E-01
**SPRY2**	0.31	5.28E-02	1.00E+00	-0.34	3.45E-01	8.50E-01
**BARX2**	-0.35	3.51E-01	1.00E+00	0.65	3.45E-01	8.50E-01
**AL831711.1**	-0.93	5.72E-01	1.00E+00	1.01	3.45E-01	8.50E-01

**PIK3CD-AS2**	-0.55	3.75E-01	1.00E+00	0.44	3.45E-01	8.50E-01
**GMNN**	0.09	7.14E-01	1.00E+00	-0.40	3.46E-01	8.50E-01
**LCA5L**	0.53	2.83E-01	1.00E+00	-0.63	3.46E-01	8.50E-01
**MSC**	-0.30	4.72E-01	1.00E+00	0.58	3.46E-01	8.50E-01
**RRP12**	-0.34	3.11E-01	1.00E+00	0.44	3.46E-01	8.50E-01
**MYDGF**	-0.10	6.25E-01	1.00E+00	0.20	3.46E-01	8.50E-01
**TTBK2**	-0.01	9.66E-01	1.00E+00	-0.44	3.46E-01	8.50E-01
**AL022313.2**	-1.58	3.37E-01	1.00E+00	-1.00	3.46E-01	8.50E-01
**G31873**	0.33	7.10E-01	1.00E+00	0.81	3.46E-01	8.50E-01
**AMMECR1L**	-0.09	6.94E-01	1.00E+00	0.37	3.46E-01	8.50E-01
**NADK**	0.19	4.49E-01	1.00E+00	0.53	3.46E-01	8.50E-01
**3-Sep**	-0.89	1.68E-01	1.00E+00	0.87	3.46E-01	8.50E-01
**ESYT1**	0.31	3.44E-01	1.00E+00	-0.34	3.46E-01	8.50E-01
**AC092139.2**	-2.10	3.73E-01	1.00E+00	-1.38	3.46E-01	8.50E-01
**LINC02361**	-0.68	1.88E-01	1.00E+00	0.68	3.46E-01	8.50E-01
**7-Sep**	-0.04	8.44E-01	1.00E+00	-0.23	3.46E-01	8.51E-01
**ZBTB43**	-0.20	5.04E-01	1.00E+00	0.28	3.46E-01	8.51E-01
**RIC1**	0.15	5.13E-01	1.00E+00	0.35	3.46E-01	8.51E-01
**AC010210.1**	-2.14	2.57E-01	1.00E+00	0.88	3.46E-01	8.51E-01
**APOD**	0.56	2.71E-01	1.00E+00	0.42	3.46E-01	8.51E-01
**LMF1**	-0.09	7.14E-01	1.00E+00	0.18	3.46E-01	8.51E-01
**ZNF692**	-0.74	2.20E-02	8.96E-01	0.38	3.46E-01	8.51E-01
**Z98885.2**	-1.60	6.43E-01	1.00E+00	-1.38	3.46E-01	8.51E-01
**AL157895.1**	-1.05	3.40E-01	1.00E+00	0.54	3.46E-01	8.51E-01
**UBE2F**	0.11	5.87E-01	1.00E+00	-0.19	3.46E-01	8.51E-01
**CASQ1**	-0.29	7.53E-01	1.00E+00	-1.08	3.46E-01	8.51E-01
**ARID1B**	-0.29	2.27E-01	1.00E+00	-0.25	3.46E-01	8.51E-01
**LRRC39**	2.24	8.54E-02	1.00E+00	0.73	3.47E-01	8.51E-01
**MKLN1-AS**	0.23	6.08E-01	1.00E+00	-0.34	3.47E-01	8.51E-01
**CPA1**	0.15	9.22E-01	1.00E+00	-1.10	3.47E-01	8.51E-01
**ZFP3**	0.18	6.47E-01	1.00E+00	-0.39	3.47E-01	8.51E-01
**G2523**	0.58	7.19E-01	1.00E+00	-1.64	3.47E-01	8.51E-01

**RPLP1P6**	0.60	3.86E-01	1.00E+00	0.45	3.47E-01	8.51E-01
**OMD**	0.73	8.90E-02	1.00E+00	0.51	3.47E-01	8.51E-01
**LINC02603**	1.33	6.93E-01	1.00E+00	-0.78	3.47E-01	8.51E-01
**YBX1**	-0.17	3.69E-01	1.00E+00	0.49	3.47E-01	8.51E-01
**G8972**	-0.08	9.51E-01	1.00E+00	0.78	3.47E-01	8.51E-01
**PNMA3**	-4.18	6.86E-04	1.25E-01	1.28	3.47E-01	8.51E-01
**INE1**	-1.68	2.96E-01	1.00E+00	-0.76	3.47E-01	8.51E-01
**SNX5**	0.25	2.63E-01	1.00E+00	-0.24	3.47E-01	8.51E-01
**TMEM60**	0.10	6.92E-01	1.00E+00	-0.29	3.47E-01	8.51E-01
**SH3D19**	0.25	1.99E-01	1.00E+00	-0.26	3.47E-01	8.51E-01
**SNORD125**	0.69	6.05E-01	1.00E+00	1.93	3.47E-01	8.51E-01
**AC016590.3**	0.94	3.78E-01	1.00E+00	1.15	3.47E-01	8.51E-01
**BAP1**	-0.14	4.38E-01	1.00E+00	0.32	3.47E-01	8.51E-01
**CD209**	0.06	9.24E-01	1.00E+00	0.64	3.47E-01	8.51E-01
**TMEM80**	-0.15	4.48E-01	1.00E+00	-0.24	3.47E-01	8.51E-01
**PGM2L1**	-0.02	9.65E-01	1.00E+00	0.34	3.47E-01	8.51E-01
**TRIQK**	0.23	5.42E-01	1.00E+00	0.23	3.47E-01	8.51E-01
**HK2**	-0.21	6.81E-01	1.00E+00	0.52	3.47E-01	8.51E-01
**EEF1AKMT4**	-0.38	3.36E-01	1.00E+00	0.38	3.47E-01	8.51E-01
**SPPL2A**	0.40	9.95E-02	1.00E+00	-0.21	3.47E-01	8.51E-01
**CCNI**	0.11	6.44E-01	1.00E+00	-0.20	3.48E-01	8.51E-01
**ARL1**	0.21	2.44E-01	1.00E+00	-0.19	3.48E-01	8.51E-01
**MEF2C-AS1**	-0.78	1.80E-01	1.00E+00	0.62	3.48E-01	8.51E-01
**AC104695.1**	-0.67	8.48E-01	1.00E+00	1.95	3.48E-01	8.51E-01
**AL662800.1**	-1.50	1.49E-01	1.00E+00	-0.56	3.48E-01	8.51E-01
**OR7A5**	0.22	8.25E-01	1.00E+00	-1.19	3.48E-01	8.51E-01
**SPAG5**	-0.33	2.07E-01	1.00E+00	0.36	3.48E-01	8.51E-01
**CYB5R3**	0.12	6.43E-01	1.00E+00	-0.24	3.48E-01	8.51E-01
**CASTOR3**	-0.43	2.53E-01	1.00E+00	-0.61	3.48E-01	8.51E-01
**AC103853.2**	-2.19	5.21E-01	1.00E+00	1.04	3.48E-01	8.51E-01
**RNASE7**	0.24	7.50E-01	1.00E+00	0.66	3.48E-01	8.51E-01
**MFSD8**	0.38	3.22E-01	1.00E+00	-0.32	3.48E-01	8.51E-01

**RP11-592B15.4**	-0.02	9.49E-01	1.00E+00	-0.22	3.48E-01	8.51E-01
**PMAIP1**	-0.53	4.27E-01	1.00E+00	0.51	3.48E-01	8.51E-01
**ZNF561**	0.06	7.84E-01	1.00E+00	0.20	3.48E-01	8.51E-01
**EMP2**	-0.13	7.13E-01	1.00E+00	-0.35	3.48E-01	8.51E-01
**DRD1**	-0.05	9.56E-01	1.00E+00	1.32	3.48E-01	8.51E-01
**DCP1A**	0.17	4.90E-01	1.00E+00	0.22	3.48E-01	8.51E-01
**AC021851.1**	-1.30	6.82E-01	1.00E+00	1.28	3.48E-01	8.51E-01
**CEACAM4**	1.52	3.31E-01	1.00E+00	1.16	3.48E-01	8.51E-01
**EMX2**	0.31	4.58E-01	1.00E+00	0.39	3.48E-01	8.51E-01
**FIG4**	0.13	6.17E-01	1.00E+00	-0.22	3.48E-01	8.51E-01
**TMPRSS11A**	NA	NA	NA	1.76	3.48E-01	8.51E-01
**MAMDC4**	1.06	1.56E-01	1.00E+00	1.08	3.48E-01	8.51E-01
**RNF220**	0.03	8.89E-01	1.00E+00	-0.30	3.48E-01	8.51E-01
**SEM1**	0.10	6.27E-01	1.00E+00	-0.34	3.48E-01	8.51E-01
**NR4A2**	-1.38	4.28E-02	1.00E+00	0.52	3.48E-01	8.51E-01
**ELOCP2**	-0.38	9.12E-01	1.00E+00	-0.64	3.48E-01	8.51E-01
**ZNF732**	-0.67	2.72E-01	1.00E+00	-0.92	3.48E-01	8.51E-01
**LSM14A**	0.01	9.61E-01	1.00E+00	-0.13	3.48E-01	8.51E-01
**NPIPA5**	-4.34	2.13E-03	2.39E-01	-0.93	3.49E-01	8.51E-01
**APPL2**	-0.06	8.32E-01	1.00E+00	-0.18	3.49E-01	8.51E-01
**PRMT2**	-0.04	7.93E-01	1.00E+00	-0.16	3.49E-01	8.51E-01
**XLOC_000566**	1.10	6.15E-01	1.00E+00	0.92	3.49E-01	8.51E-01
**COPS4**	0.07	7.32E-01	1.00E+00	-0.28	3.49E-01	8.51E-01
**UBE3D**	0.20	5.87E-01	1.00E+00	0.46	3.49E-01	8.51E-01
**SSH3**	-0.19	3.82E-01	1.00E+00	0.31	3.49E-01	8.51E-01
**APEX1**	-0.20	2.18E-01	1.00E+00	0.15	3.49E-01	8.51E-01
**KCNIP2**	4.31	1.04E-02	6.33E-01	-0.95	3.49E-01	8.51E-01
**IMMT**	-0.18	2.43E-01	1.00E+00	-0.19	3.49E-01	8.51E-01
**LANCL1-AS1**	-0.10	9.18E-01	1.00E+00	-0.68	3.49E-01	8.51E-01
**AP001471.1**	-1.49	2.43E-01	1.00E+00	-1.14	3.49E-01	8.51E-01
**ASNSP1**	1.96	5.66E-01	1.00E+00	-1.99	3.49E-01	8.51E-01
**NFYA**	-0.12	6.92E-01	1.00E+00	0.36	3.49E-01	8.51E-01

**SLC7A6**	0.60	1.17E-01	1.00E+00	0.37	3.49E-01	8.51E-01
**ZIK1**	0.16	5.77E-01	1.00E+00	-0.30	3.49E-01	8.51E-01
**FBXL7**	-0.10	8.06E-01	1.00E+00	0.74	3.49E-01	8.51E-01
**FGF22**	0.11	8.58E-01	1.00E+00	-0.74	3.49E-01	8.51E-01
**SMCO4**	0.30	2.48E-01	1.00E+00	0.30	3.49E-01	8.51E-01
**CAV1**	0.51	2.39E-01	1.00E+00	-0.40	3.49E-01	8.51E-01
**SELENOT**	0.21	3.64E-01	1.00E+00	-0.29	3.49E-01	8.51E-01
**MAPK9**	0.18	4.16E-01	1.00E+00	-0.45	3.49E-01	8.51E-01
**GZMB**	-0.48	5.23E-01	1.00E+00	2.93	3.49E-01	8.51E-01
**BRF1**	0.05	7.88E-01	1.00E+00	0.56	3.49E-01	8.51E-01
**MAMSTR**	-0.25	5.33E-01	1.00E+00	0.34	3.49E-01	8.51E-01
**SLC6A15**	0.18	6.03E-01	1.00E+00	0.47	3.49E-01	8.51E-01
**TWNK**	0.01	9.44E-01	1.00E+00	0.49	3.49E-01	8.51E-01
**LINC01715**	-0.01	9.94E-01	1.00E+00	-0.53	3.49E-01	8.51E-01
**AC073326.1**	-1.41	5.17E-01	1.00E+00	-1.30	3.49E-01	8.51E-01
**SHC2**	-0.06	8.88E-01	1.00E+00	0.46	3.49E-01	8.51E-01
**RHPN2**	0.27	8.14E-01	1.00E+00	-0.66	3.50E-01	8.51E-01
**ZEB1-AS1**	0.60	2.60E-01	1.00E+00	-0.36	3.50E-01	8.51E-01
**CCDC160**	0.35	7.24E-01	1.00E+00	-2.00	3.50E-01	8.51E-01
**XLOC_003863**	1.12	4.71E-01	1.00E+00	-0.84	3.50E-01	8.51E-01
**AC010457.1**	-0.87	7.99E-01	1.00E+00	-1.19	3.50E-01	8.51E-01
**XKR9**	-0.73	5.59E-01	1.00E+00	-0.85	3.50E-01	8.51E-01
**MRPS12**	-0.25	2.69E-01	1.00E+00	0.27	3.50E-01	8.51E-01
**ABTB1**	0.37	1.80E-01	1.00E+00	0.28	3.50E-01	8.51E-01
**AC093510.2**	-1.49	6.18E-01	1.00E+00	-1.01	3.50E-01	8.51E-01
**PRPF19**	-0.15	4.97E-01	1.00E+00	0.56	3.50E-01	8.51E-01
**TPM1-AS**	0.81	5.27E-01	1.00E+00	0.84	3.50E-01	8.51E-01
**NCOA2**	-0.09	6.42E-01	1.00E+00	0.31	3.50E-01	8.51E-01
**AC245123.1**	2.46	1.26E-01	1.00E+00	-1.33	3.50E-01	8.51E-01
**RP11-87H9.2**	-0.61	2.80E-01	1.00E+00	-0.29	3.50E-01	8.51E-01
**GTF2IP12**	-0.46	5.38E-01	1.00E+00	-0.51	3.50E-01	8.51E-01
**G32166**	-0.73	1.77E-01	1.00E+00	-0.50	3.50E-01	8.51E-01

**DENND5A**	0.25	5.49E-01	1.00E+00	0.49	3.50E-01	8.51E-01
**GEMIN8**	-0.05	7.91E-01	1.00E+00	0.12	3.50E-01	8.51E-01
**HLA-F-AS1**	-0.59	3.53E-01	1.00E+00	-0.50	3.50E-01	8.51E-01
**LILRB5**	-0.04	9.49E-01	1.00E+00	0.58	3.50E-01	8.51E-01
**CD58**	-0.13	6.84E-01	1.00E+00	-0.43	3.50E-01	8.51E-01
**GSDMC**	0.10	8.51E-01	1.00E+00	0.63	3.50E-01	8.51E-01
**AC004492.1**	-0.67	4.28E-01	1.00E+00	0.93	3.50E-01	8.51E-01
**GS1-124K5.4**	0.19	6.51E-01	1.00E+00	-0.55	3.50E-01	8.51E-01
**AL031577.1**	-2.14	4.81E-01	1.00E+00	-0.87	3.50E-01	8.51E-01
**GPHN**	-0.20	3.86E-01	1.00E+00	-0.26	3.50E-01	8.51E-01
**CDK5RAP3**	0.20	8.84E-01	1.00E+00	0.56	3.51E-01	8.51E-01
**NUTM2B-AS1**	0.10	7.49E-01	1.00E+00	-0.30	3.51E-01	8.51E-01
**RORA-AS1**	0.94	2.85E-01	1.00E+00	0.63	3.51E-01	8.51E-01
**NECTIN3-AS1**	1.42	4.48E-01	1.00E+00	-1.08	3.51E-01	8.51E-01
**NDC1**	0.06	8.08E-01	1.00E+00	-0.21	3.51E-01	8.51E-01
**OGDH**	-0.19	3.21E-01	1.00E+00	0.35	3.51E-01	8.51E-01
**PRELP**	0.54	2.03E-01	1.00E+00	0.40	3.51E-01	8.51E-01
**GPR153**	0.11	7.42E-01	1.00E+00	0.56	3.51E-01	8.51E-01
**RNU4-1**	-0.52	7.57E-01	1.00E+00	-0.95	3.51E-01	8.51E-01
**AC104135.1**	-0.66	6.37E-01	1.00E+00	1.27	3.51E-01	8.51E-01
**LINC00535**	1.07	5.46E-01	1.00E+00	0.80	3.51E-01	8.51E-01
**GRIPAP1**	-0.13	5.67E-01	1.00E+00	0.27	3.51E-01	8.51E-01
**ACVR1B**	-0.16	4.96E-01	1.00E+00	0.46	3.51E-01	8.51E-01
**FCRLA**	-1.99	3.38E-02	1.00E+00	0.51	3.51E-01	8.51E-01
**AC025031.4**	-3.32	1.97E-01	1.00E+00	1.32	3.51E-01	8.52E-01
**AL356057.1**	-0.25	9.41E-01	1.00E+00	-1.19	3.51E-01	8.52E-01
**ZNF547**	1.44	1.42E-02	7.51E-01	-0.73	3.52E-01	8.52E-01
**CEACAM19**	-0.29	5.59E-01	1.00E+00	0.60	3.52E-01	8.52E-01
**DNAJC22**	-2.27	2.43E-03	2.50E-01	-0.69	3.52E-01	8.52E-01
**KPNA3**	-0.10	6.42E-01	1.00E+00	0.17	3.52E-01	8.52E-01
**AL121672.3**	0.90	3.61E-01	1.00E+00	-0.51	3.52E-01	8.52E-01
**AL117328.1**	-2.70	1.59E-01	1.00E+00	1.33	3.52E-01	8.52E-01

**CITED2**	-0.18	6.66E-01	1.00E+00	0.47	3.52E-01	8.52E-01
**CKS1BP1**	-3.38	7.98E-02	1.00E+00	0.74	3.52E-01	8.52E-01
**CCR3**	-0.62	3.22E-01	1.00E+00	0.73	3.52E-01	8.52E-01
**RNF208**	-0.73	2.10E-02	8.94E-01	0.58	3.52E-01	8.53E-01
**CNOT2**	-0.09	7.07E-01	1.00E+00	-0.16	3.52E-01	8.53E-01
**SUGT1P3**	-0.11	9.30E-01	1.00E+00	0.62	3.52E-01	8.53E-01
**LIPJ**	-1.68	3.15E-01	1.00E+00	-1.12	3.52E-01	8.53E-01
**RBM14**	-0.07	7.45E-01	1.00E+00	-0.24	3.52E-01	8.53E-01
**MCRS1**	-0.04	8.35E-01	1.00E+00	0.14	3.52E-01	8.53E-01
**ZNF713**	-0.84	7.13E-02	1.00E+00	-0.52	3.52E-01	8.53E-01
**RPL29P14**	-2.65	3.63E-01	1.00E+00	0.95	3.52E-01	8.53E-01
**TCFL5**	-0.19	4.16E-01	1.00E+00	-0.42	3.52E-01	8.53E-01
**TRIM17**	-0.91	1.28E-01	1.00E+00	0.97	3.52E-01	8.53E-01
**GRAP**	-0.51	4.39E-01	1.00E+00	0.79	3.53E-01	8.53E-01
**CDK2**	0.37	2.47E-01	1.00E+00	-0.22	3.53E-01	8.53E-01
**TCAIM**	0.23	3.60E-01	1.00E+00	-0.23	3.53E-01	8.53E-01
**XLOC_001984**	2.33	4.63E-01	1.00E+00	-0.84	3.53E-01	8.53E-01
**PTOV1-AS1**	-0.30	5.95E-01	1.00E+00	-0.66	3.53E-01	8.53E-01
**AC097263.1**	-0.95	7.05E-01	1.00E+00	-0.62	3.53E-01	8.53E-01
**RPS26P28**	-1.27	6.77E-01	1.00E+00	-0.50	3.53E-01	8.53E-01
**DPH7**	-0.18	4.91E-01	1.00E+00	0.17	3.53E-01	8.53E-01
**FNTB**	0.34	1.04E-01	1.00E+00	-0.22	3.53E-01	8.53E-01
**HS6ST1**	-0.33	1.35E-01	1.00E+00	0.59	3.53E-01	8.53E-01
**ZMIZ1**	-0.47	7.29E-02	1.00E+00	0.44	3.53E-01	8.53E-01
**XLOC_009683**	-2.03	4.85E-01	1.00E+00	-0.93	3.53E-01	8.53E-01
**CTNNA2**	-3.14	9.26E-04	1.56E-01	-1.15	3.53E-01	8.53E-01
**TANK**	0.13	5.50E-01	1.00E+00	-0.19	3.53E-01	8.53E-01
**PTCHD1**	0.81	3.01E-01	1.00E+00	-0.92	3.53E-01	8.53E-01
**MYL2**	-1.42	4.68E-01	1.00E+00	-0.87	3.53E-01	8.53E-01
**LINC01085**	-1.10	7.53E-01	1.00E+00	-1.72	3.53E-01	8.53E-01
**HP1BP3**	-0.34	1.23E-01	1.00E+00	-0.18	3.53E-01	8.53E-01
**SMC3**	-0.09	6.33E-01	1.00E+00	-0.15	3.53E-01	8.53E-01

**SLC25A12**	-0.24	1.62E-01	1.00E+00	-0.17	3.53E-01	8.53E-01
**SENCR**	0.01	9.91E-01	1.00E+00	0.53	3.53E-01	8.53E-01
**BCL2L15**	-2.87	1.56E-01	1.00E+00	-1.38	3.53E-01	8.53E-01
**SAAL1**	-0.05	8.34E-01	1.00E+00	0.23	3.53E-01	8.53E-01
**EWSR1**	-0.14	5.44E-01	1.00E+00	0.15	3.54E-01	8.53E-01
**MLXIP**	0.19	4.56E-01	1.00E+00	0.33	3.54E-01	8.53E-01
**TPM3**	-0.09	5.52E-01	1.00E+00	0.14	3.54E-01	8.53E-01
**AFAP1**	-0.14	7.40E-01	1.00E+00	0.47	3.54E-01	8.53E-01
**FAM72D**	0.81	1.74E-01	1.00E+00	-0.49	3.54E-01	8.53E-01
**AC102945.2**	-0.61	6.35E-01	1.00E+00	1.30	3.54E-01	8.53E-01
**RPL10P3**	-3.83	1.15E-01	1.00E+00	-2.73	3.54E-01	8.53E-01
**MALSU1**	0.16	4.29E-01	1.00E+00	-0.26	3.54E-01	8.53E-01
**XLOC_012049**	-2.03	4.08E-01	1.00E+00	1.28	3.54E-01	8.53E-01
**ACCS**	-0.95	3.63E-02	1.00E+00	-0.39	3.54E-01	8.53E-01
**AL049649.1**	-1.29	1.83E-01	1.00E+00	-0.66	3.54E-01	8.53E-01
**RBL2**	0.15	4.11E-01	1.00E+00	-0.26	3.54E-01	8.53E-01
**AP001347.1**	-0.26	7.31E-01	1.00E+00	-0.55	3.54E-01	8.53E-01
**NEBL-AS1**	-0.14	8.30E-01	1.00E+00	-0.66	3.54E-01	8.53E-01
**XLOC_014386**	0.13	8.94E-01	1.00E+00	-0.39	3.54E-01	8.53E-01
**NHSL2**	-0.12	7.64E-01	1.00E+00	0.52	3.54E-01	8.53E-01
**OMA1**	0.35	3.32E-01	1.00E+00	-0.39	3.54E-01	8.53E-01
**POP5**	-0.21	3.81E-01	1.00E+00	-0.33	3.54E-01	8.53E-01
**FAM182A**	-0.40	4.20E-01	1.00E+00	-0.56	3.54E-01	8.53E-01
**XLOC_006699**	-2.40	2.82E-01	1.00E+00	1.34	3.54E-01	8.53E-01
**ZNF461**	0.29	2.14E-01	1.00E+00	-0.36	3.54E-01	8.53E-01
**MRPL42**	0.18	4.45E-01	1.00E+00	-0.38	3.54E-01	8.54E-01
**MIR1-1HG-AS1**	-0.71	6.76E-01	1.00E+00	-1.37	3.54E-01	8.54E-01
**NINL**	-0.56	8.24E-02	1.00E+00	0.59	3.54E-01	8.54E-01
**AC108676.1**	-0.09	9.70E-01	1.00E+00	1.64	3.54E-01	8.54E-01
**PPFIBP1**	0.11	6.05E-01	1.00E+00	0.15	3.54E-01	8.54E-01
**KLF7**	0.01	9.89E-01	1.00E+00	0.36	3.54E-01	8.54E-01
**BANF1P3**	0.11	9.14E-01	1.00E+00	0.51	3.55E-01	8.54E-01

**AC011503.2**	-0.41	7.92E-01	1.00E+00	0.83	3.55E-01	8.54E-01
**AC080023.1**	-1.49	4.92E-01	1.00E+00	1.15	3.55E-01	8.54E-01
**HSPA1B**	-0.24	6.31E-01	1.00E+00	-0.55	3.55E-01	8.54E-01
**ELOVL7**	0.11	8.39E-01	1.00E+00	-0.33	3.55E-01	8.54E-01
**SDR9C7**	0.57	3.03E-01	1.00E+00	0.69	3.55E-01	8.54E-01
**AC073130.1**	-0.39	9.09E-01	1.00E+00	1.32	3.55E-01	8.54E-01
**WASHC4**	-0.01	9.77E-01	1.00E+00	0.23	3.55E-01	8.54E-01
**RPL7L1**	-0.15	4.14E-01	1.00E+00	0.21	3.55E-01	8.54E-01
**SCAMP1**	-0.01	9.67E-01	1.00E+00	-0.23	3.55E-01	8.54E-01
**EID2**	-0.16	4.47E-01	1.00E+00	-0.58	3.55E-01	8.54E-01
**AL158201.1**	0.25	9.42E-01	1.00E+00	0.61	3.55E-01	8.54E-01
**RRN3P1**	0.05	9.32E-01	1.00E+00	-0.52	3.55E-01	8.54E-01
**RUNDC3A**	-0.46	3.02E-01	1.00E+00	0.61	3.55E-01	8.54E-01
**TBC1D8**	0.36	3.96E-01	1.00E+00	0.41	3.55E-01	8.54E-01
**G19262**	0.71	8.04E-01	1.00E+00	0.70	3.55E-01	8.54E-01
**CDPF1**	-0.31	7.19E-02	1.00E+00	-0.29	3.55E-01	8.54E-01
**SLC2A1**	-0.64	9.79E-02	1.00E+00	0.44	3.55E-01	8.54E-01
**ARHGAP20**	1.71	3.31E-02	1.00E+00	0.61	3.55E-01	8.54E-01
**MON1A**	-0.05	8.44E-01	1.00E+00	-0.21	3.55E-01	8.54E-01
**NTN5**	-0.63	5.60E-01	1.00E+00	1.17	3.55E-01	8.54E-01
**SKIV2L**	-0.23	3.42E-01	1.00E+00	0.41	3.55E-01	8.54E-01
**USP51**	0.18	6.79E-01	1.00E+00	0.44	3.55E-01	8.54E-01
**PIK3C2B**	0.03	9.06E-01	1.00E+00	0.39	3.55E-01	8.54E-01
**FBXO38**	0.02	9.33E-01	1.00E+00	-0.16	3.55E-01	8.54E-01
**AC007249.1**	-0.21	9.09E-01	1.00E+00	1.19	3.55E-01	8.54E-01
**ALS2CR12**	0.13	9.27E-01	1.00E+00	-1.01	3.55E-01	8.54E-01
**BMP2K**	-0.32	3.48E-01	1.00E+00	0.54	3.56E-01	8.54E-01
**MED30**	0.06	8.20E-01	1.00E+00	-0.31	3.56E-01	8.54E-01
**LIFR**	0.43	2.46E-01	1.00E+00	-0.54	3.56E-01	8.54E-01
**AC104581.4**	0.82	2.51E-01	1.00E+00	0.54	3.56E-01	8.54E-01
**SERTAD3**	-0.44	6.68E-02	1.00E+00	0.12	3.56E-01	8.54E-01
**ZDHHC8P1**	-0.31	4.63E-01	1.00E+00	-0.68	3.56E-01	8.54E-01

**HNRNPA3P5**	0.21	8.09E-01	1.00E+00	-0.47	3.56E-01	8.54E-01
**DPH1**	-0.32	3.23E-01	1.00E+00	-0.32	3.56E-01	8.54E-01
**DNAH1**	-0.11	8.08E-01	1.00E+00	0.90	3.56E-01	8.54E-01
**LINC01535**	-2.54	6.36E-02	1.00E+00	-0.84	3.56E-01	8.54E-01
**MTA2**	-0.26	3.73E-01	1.00E+00	0.60	3.56E-01	8.55E-01
**IGLV1-40**	0.78	6.56E-01	1.00E+00	2.90	3.56E-01	8.55E-01
**TSSC2**	0.66	2.28E-01	1.00E+00	0.83	3.56E-01	8.55E-01
**LIMA1**	-0.04	8.94E-01	1.00E+00	0.22	3.56E-01	8.55E-01
**P4HA2**	-0.14	5.83E-01	1.00E+00	0.18	3.56E-01	8.55E-01
**ADPRH**	0.37	4.37E-01	1.00E+00	0.46	3.56E-01	8.55E-01
**XPA**	0.12	5.93E-01	1.00E+00	-0.33	3.56E-01	8.55E-01
**SMG5**	0.16	4.74E-01	1.00E+00	0.49	3.56E-01	8.55E-01
**CIDEC**	4.55	1.04E-02	6.33E-01	-0.91	3.56E-01	8.55E-01
**DNASE1**	0.08	8.89E-01	1.00E+00	0.49	3.57E-01	8.55E-01
**AC008533.1**	-3.30	1.94E-01	1.00E+00	0.98	3.57E-01	8.55E-01
**HCG11**	0.57	9.71E-02	1.00E+00	0.28	3.57E-01	8.55E-01
**AC000123.1**	1.46	9.75E-02	1.00E+00	0.98	3.57E-01	8.55E-01
**FOXJ2**	-0.22	2.97E-01	1.00E+00	-0.35	3.57E-01	8.55E-01
**AMMECR1**	0.05	8.92E-01	1.00E+00	0.28	3.57E-01	8.55E-01
**C11orf54**	0.65	1.03E-01	1.00E+00	-0.40	3.57E-01	8.55E-01
**AC105411.1**	0.62	5.34E-01	1.00E+00	-0.82	3.57E-01	8.55E-01
**AC010834.3**	0.16	8.39E-01	1.00E+00	0.74	3.57E-01	8.55E-01
**C5orf46**	0.15	7.93E-01	1.00E+00	-0.71	3.57E-01	8.55E-01
**TACR1**	-0.39	3.27E-01	1.00E+00	-0.56	3.57E-01	8.55E-01
**LINC00892**	-1.59	4.12E-01	1.00E+00	0.84	3.57E-01	8.55E-01
**TRMT10C**	-0.05	7.48E-01	1.00E+00	-0.25	3.57E-01	8.55E-01
**PIGF**	0.11	8.48E-01	1.00E+00	-0.42	3.57E-01	8.56E-01
**PLCE1**	0.23	6.76E-01	1.00E+00	0.28	3.57E-01	8.56E-01
**SLAIN1**	0.14	7.77E-01	1.00E+00	-0.85	3.58E-01	8.56E-01
**DNAJC9-AS1**	0.22	7.51E-01	1.00E+00	-0.56	3.58E-01	8.56E-01
**NEURL1**	-1.42	7.65E-03	5.36E-01	0.40	3.58E-01	8.56E-01
**NUDT3**	-0.51	1.58E-01	1.00E+00	0.82	3.58E-01	8.56E-01

**TSPOAP1**	0.10	8.28E-01	1.00E+00	0.60	3.58E-01	8.56E-01
**GPR174**	-0.20	8.48E-01	1.00E+00	1.18	3.58E-01	8.56E-01
**SBK3**	-1.16	4.91E-01	1.00E+00	1.44	3.58E-01	8.56E-01
**AP003059.1**	-2.02	5.55E-01	1.00E+00	-1.20	3.58E-01	8.56E-01
**ABI3BP**	0.65	2.28E-01	1.00E+00	0.43	3.58E-01	8.56E-01
**PITPNM1**	-0.81	1.08E-02	6.40E-01	0.68	3.58E-01	8.56E-01
**JUND**	-1.25	1.05E-02	6.33E-01	-0.65	3.58E-01	8.56E-01
**NOX4**	1.10	1.33E-01	1.00E+00	0.48	3.58E-01	8.56E-01
**BSPRY**	0.11	7.99E-01	1.00E+00	-0.55	3.58E-01	8.56E-01
**RBM42**	-0.26	2.99E-01	1.00E+00	0.17	3.58E-01	8.56E-01
**LSR**	-0.35	2.11E-01	1.00E+00	-0.52	3.58E-01	8.56E-01
**LINC00482**	-0.01	9.82E-01	1.00E+00	-0.82	3.58E-01	8.57E-01
**GEMIN7**	-0.04	8.97E-01	1.00E+00	-0.36	3.58E-01	8.57E-01
**LSM7**	-0.36	6.22E-02	1.00E+00	0.24	3.58E-01	8.57E-01
**IDS**	0.23	3.04E-01	1.00E+00	0.17	3.59E-01	8.57E-01
**ZNF316**	-0.20	4.47E-01	1.00E+00	0.62	3.59E-01	8.57E-01
**AC069224.1**	0.00	9.92E-01	1.00E+00	-0.81	3.59E-01	8.57E-01
**AC015911.3**	1.34	2.82E-01	1.00E+00	1.09	3.59E-01	8.57E-01
**DDX20**	-0.13	5.77E-01	1.00E+00	-0.20	3.59E-01	8.57E-01
**VAT1L**	-1.62	5.53E-01	1.00E+00	0.95	3.59E-01	8.57E-01
**CD81**	0.28	3.30E-01	1.00E+00	0.44	3.59E-01	8.57E-01
**ZKSCAN7**	-0.20	7.33E-01	1.00E+00	0.79	3.59E-01	8.57E-01
**G34277**	-1.10	4.56E-01	1.00E+00	1.15	3.59E-01	8.57E-01
**FAT3**	-0.86	4.12E-02	1.00E+00	-0.93	3.59E-01	8.57E-01
**TGFBR3L**	-0.45	4.12E-01	1.00E+00	-0.65	3.59E-01	8.57E-01
**CES1P1**	0.50	5.71E-01	1.00E+00	-0.96	3.59E-01	8.57E-01
**CMTM4**	0.27	6.45E-01	1.00E+00	0.33	3.59E-01	8.57E-01
**SLFN11**	0.16	7.35E-01	1.00E+00	0.51	3.59E-01	8.57E-01
**DDX42**	-0.04	8.14E-01	1.00E+00	0.35	3.59E-01	8.57E-01
**AC020661.1**	-1.28	3.18E-02	1.00E+00	0.94	3.59E-01	8.57E-01
**SIAE**	-0.04	8.48E-01	1.00E+00	0.54	3.59E-01	8.57E-01
**ACP1**	-0.04	7.76E-01	1.00E+00	-0.25	3.59E-01	8.57E-01

**RPL24**	-0.17	5.38E-01	1.00E+00	0.20	3.59E-01	8.57E-01
**FEZ1**	-0.08	8.46E-01	1.00E+00	0.30	3.59E-01	8.57E-01
**EFHD1**	-0.14	7.83E-01	1.00E+00	-0.61	3.59E-01	8.57E-01
**ZC3H11B**	-2.14	9.64E-02	1.00E+00	-0.51	3.59E-01	8.57E-01
**AL050341.2**	0.20	6.03E-01	1.00E+00	-0.48	3.59E-01	8.57E-01
**RRP7A**	-0.01	9.67E-01	1.00E+00	0.22	3.59E-01	8.57E-01
**SP4**	0.18	5.89E-01	1.00E+00	-0.34	3.59E-01	8.57E-01
**HLA-G**	0.11	9.02E-01	1.00E+00	0.39	3.59E-01	8.57E-01
**FOXO4**	-0.20	2.07E-01	1.00E+00	0.48	3.60E-01	8.57E-01
**ZNF20**	1.22	4.48E-01	1.00E+00	-0.67	3.60E-01	8.57E-01
**CXCL10**	-3.41	5.38E-03	4.32E-01	2.86	3.60E-01	8.57E-01
**AC027702.1**	-1.05	1.22E-01	1.00E+00	-0.54	3.60E-01	8.57E-01
**CCSER2**	-0.24	3.64E-01	1.00E+00	-0.12	3.60E-01	8.57E-01
**AP001033.2**	-0.05	9.60E-01	1.00E+00	0.74	3.60E-01	8.57E-01
**SMS**	0.27	1.83E-01	1.00E+00	-0.18	3.60E-01	8.57E-01
**IPO8**	0.10	6.38E-01	1.00E+00	0.29	3.60E-01	8.57E-01
**TM7SF3**	-0.04	8.36E-01	1.00E+00	-0.35	3.60E-01	8.57E-01
**AC006042.3**	1.77	1.77E-01	1.00E+00	0.91	3.60E-01	8.57E-01
**FLJ31356**	-0.38	6.96E-01	1.00E+00	0.88	3.60E-01	8.57E-01
**SOSTDC1**	0.22	6.11E-01	1.00E+00	-0.53	3.60E-01	8.57E-01
**GPR135**	-0.16	8.86E-01	1.00E+00	-0.65	3.60E-01	8.57E-01
**AC107072.2**	0.41	9.05E-01	1.00E+00	1.14	3.60E-01	8.57E-01
**SSBP1**	-0.05	7.75E-01	1.00E+00	-0.25	3.60E-01	8.57E-01
**G26243**	-0.84	5.05E-01	1.00E+00	1.12	3.60E-01	8.57E-01
**AC068446.2**	1.24	2.20E-01	1.00E+00	0.85	3.60E-01	8.57E-01
**AC022968.1**	-0.61	8.17E-01	1.00E+00	-0.59	3.60E-01	8.57E-01
**AC107214.1**	-1.11	1.58E-01	1.00E+00	-0.71	3.60E-01	8.57E-01
**PERP**	0.03	9.39E-01	1.00E+00	-0.46	3.60E-01	8.57E-01
**RNF223**	-0.71	3.44E-01	1.00E+00	0.72	3.60E-01	8.57E-01
**SIGLEC16**	-1.89	9.04E-02	1.00E+00	1.37	3.60E-01	8.57E-01
**CHIC1**	0.28	2.08E-01	1.00E+00	0.34	3.60E-01	8.57E-01
**ARL2BP**	0.39	4.25E-01	1.00E+00	0.48	3.60E-01	8.57E-01

**RASGRF2-AS1**	-2.56	4.55E-01	1.00E+00	1.14	3.60E-01	8.57E-01
**BROX**	0.10	5.88E-01	1.00E+00	-0.43	3.61E-01	8.57E-01
**AL021707.6**	-0.94	5.85E-01	1.00E+00	1.01	3.61E-01	8.57E-01
**LINC01460**	-0.05	9.89E-01	1.00E+00	1.59	3.61E-01	8.57E-01
**QRICH1**	-0.13	5.74E-01	1.00E+00	0.18	3.61E-01	8.57E-01
**UBE2G1**	0.19	2.37E-01	1.00E+00	-0.20	3.61E-01	8.57E-01
**RIPK3**	-0.53	5.17E-02	1.00E+00	0.32	3.61E-01	8.57E-01
**CBX3**	0.03	9.06E-01	1.00E+00	-0.45	3.61E-01	8.57E-01
**AC097637.2**	-1.55	6.52E-01	1.00E+00	-1.38	3.61E-01	8.57E-01
**SLC38A3**	1.15	3.82E-01	1.00E+00	0.97	3.61E-01	8.57E-01
**FSIP2**	-1.04	9.43E-02	1.00E+00	-0.73	3.61E-01	8.57E-01
**LINC00167**	-1.74	3.55E-01	1.00E+00	-1.33	3.61E-01	8.57E-01
**AC022868.1**	-2.49	9.57E-02	1.00E+00	-0.45	3.61E-01	8.57E-01
**CCT8**	-0.01	9.48E-01	1.00E+00	0.14	3.61E-01	8.57E-01
**KCTD21**	0.18	7.05E-01	1.00E+00	0.50	3.61E-01	8.57E-01
**YBX2**	-0.04	9.53E-01	1.00E+00	-0.66	3.61E-01	8.57E-01
**RGS12**	0.00	9.87E-01	1.00E+00	0.34	3.61E-01	8.57E-01
**RGS16**	-1.54	1.78E-02	8.38E-01	0.65	3.61E-01	8.57E-01
**ELP3**	0.20	1.97E-01	1.00E+00	-0.19	3.61E-01	8.57E-01
**ABALON**	0.96	3.74E-01	1.00E+00	0.58	3.61E-01	8.57E-01
**PSMD6**	-0.23	3.54E-01	1.00E+00	-0.23	3.61E-01	8.57E-01
**AC092718.4**	-0.45	2.59E-01	1.00E+00	0.43	3.61E-01	8.57E-01
**AC009686.2**	-0.18	7.86E-01	1.00E+00	-0.70	3.61E-01	8.57E-01
**ATP6V1C2**	-0.02	9.74E-01	1.00E+00	-0.41	3.61E-01	8.57E-01
**LINC02104**	0.98	4.17E-01	1.00E+00	-0.84	3.61E-01	8.57E-01
**AC244502.1**	-1.02	7.69E-01	1.00E+00	0.99	3.61E-01	8.57E-01
**NR1D1**	-1.14	4.02E-02	1.00E+00	0.39	3.61E-01	8.57E-01
**NAPA**	-0.13	5.57E-01	1.00E+00	0.22	3.61E-01	8.57E-01
**AC006449.6**	-0.29	2.88E-01	1.00E+00	-0.37	3.62E-01	8.57E-01
**TTLL12**	-0.16	5.88E-01	1.00E+00	-0.42	3.62E-01	8.57E-01
**PLIN1**	4.75	4.81E-03	4.02E-01	-0.95	3.62E-01	8.57E-01
**LINC02175**	-0.73	3.41E-01	1.00E+00	-0.76	3.62E-01	8.57E-01

**LMO2**	0.05	8.81E-01	1.00E+00	0.55	3.62E-01	8.57E-01
**TPCN1**	0.11	7.79E-01	1.00E+00	0.51	3.62E-01	8.57E-01
**AL365226.2**	0.99	2.69E-01	1.00E+00	-0.80	3.62E-01	8.57E-01
**SMCO3**	1.47	8.14E-02	1.00E+00	1.19	3.62E-01	8.57E-01
**COL20A1**	-0.85	5.72E-01	1.00E+00	1.46	3.62E-01	8.57E-01
**ZNF230**	0.34	3.41E-01	1.00E+00	-0.27	3.62E-01	8.57E-01
**SPCS2**	0.10	5.22E-01	1.00E+00	-0.24	3.62E-01	8.57E-01
**RRP1**	-0.41	9.98E-02	1.00E+00	0.27	3.62E-01	8.57E-01
**TYW1**	-0.06	8.13E-01	1.00E+00	0.17	3.62E-01	8.57E-01
**TRA2B**	0.05	8.17E-01	1.00E+00	-0.14	3.62E-01	8.57E-01
**CCNB3**	-0.34	5.02E-01	1.00E+00	1.07	3.62E-01	8.57E-01
**G4361**	1.54	3.32E-01	1.00E+00	0.93	3.62E-01	8.57E-01
**ZBED4**	-0.05	8.53E-01	1.00E+00	0.40	3.62E-01	8.57E-01
**G19546**	2.17	3.51E-02	1.00E+00	1.22	3.62E-01	8.57E-01
**NARF**	-0.08	6.40E-01	1.00E+00	-0.21	3.62E-01	8.57E-01
**ATL1**	-0.14	6.31E-01	1.00E+00	-0.31	3.62E-01	8.57E-01
**RGMB-AS1**	0.02	9.84E-01	1.00E+00	0.46	3.62E-01	8.58E-01
**IQUB**	1.16	3.65E-01	1.00E+00	0.93	3.62E-01	8.58E-01
**G36482**	0.06	9.15E-01	1.00E+00	0.56	3.62E-01	8.58E-01
**AL139423.1**	-0.14	8.83E-01	1.00E+00	-0.75	3.62E-01	8.58E-01
**PBK**	0.56	1.17E-01	1.00E+00	0.52	3.62E-01	8.58E-01
**AC005253.1**	1.78	3.38E-01	1.00E+00	1.24	3.62E-01	8.58E-01
**PRKAG2-AS1**	-0.36	4.29E-01	1.00E+00	-0.49	3.63E-01	8.58E-01
**AC008731.1**	0.79	7.41E-01	1.00E+00	0.89	3.63E-01	8.58E-01
**ST7-OT4**	0.81	8.13E-01	1.00E+00	1.17	3.63E-01	8.58E-01
**GCA**	0.27	4.99E-01	1.00E+00	-0.26	3.63E-01	8.58E-01
**C14orf119**	-0.07	7.39E-01	1.00E+00	-0.26	3.63E-01	8.58E-01
**AC090425.2**	-2.30	4.59E-01	1.00E+00	-1.06	3.63E-01	8.58E-01
**ADCYAP1**	-2.62	2.09E-01	1.00E+00	1.85	3.63E-01	8.58E-01
**AC023043.1**	-1.48	5.15E-01	1.00E+00	-0.70	3.63E-01	8.58E-01
**TRAF5**	0.10	8.16E-01	1.00E+00	0.32	3.63E-01	8.58E-01
**TRIM74**	-0.16	8.82E-01	1.00E+00	-0.84	3.63E-01	8.58E-01

**TOB2P1**	-1.93	1.12E-01	1.00E+00	-0.88	3.63E-01	8.58E-01
**IGLV3-1**	3.22	3.29E-01	1.00E+00	2.87	3.63E-01	8.58E-01
**AC016876.1**	-0.12	5.91E-01	1.00E+00	0.40	3.63E-01	8.58E-01
**TROAP**	-0.46	3.36E-01	1.00E+00	0.52	3.63E-01	8.58E-01
**AC036176.3**	0.16	9.28E-01	1.00E+00	0.83	3.63E-01	8.58E-01
**KITLG**	0.24	6.14E-01	1.00E+00	0.31	3.63E-01	8.58E-01
**SATB1-AS1**	0.72	5.00E-01	1.00E+00	1.00	3.63E-01	8.58E-01
**CD1D**	0.04	9.63E-01	1.00E+00	0.80	3.63E-01	8.58E-01
**IPP**	-0.20	4.63E-01	1.00E+00	-0.27	3.63E-01	8.58E-01
**AC073529.1**	-0.14	8.14E-01	1.00E+00	0.61	3.63E-01	8.58E-01
**PYCR3**	-0.45	1.84E-01	1.00E+00	0.28	3.63E-01	8.58E-01
**AC008687.4**	-0.64	3.20E-01	1.00E+00	0.99	3.63E-01	8.58E-01
**TRIM6**	0.02	9.72E-01	1.00E+00	-0.58	3.63E-01	8.58E-01
**FSTL4**	-0.61	8.53E-02	1.00E+00	0.50	3.63E-01	8.58E-01
**VTA1**	-0.02	9.32E-01	1.00E+00	-0.34	3.63E-01	8.58E-01
**AC010931.2**	-0.93	4.44E-01	1.00E+00	-1.04	3.63E-01	8.58E-01
**PPP1R7**	-0.11	5.76E-01	1.00E+00	-0.20	3.63E-01	8.58E-01
**PGBD1**	0.55	2.99E-01	1.00E+00	0.59	3.63E-01	8.58E-01
**FAM133B**	0.29	1.75E-01	1.00E+00	-0.25	3.64E-01	8.58E-01
**SNRNP40**	-0.06	7.53E-01	1.00E+00	-0.27	3.64E-01	8.58E-01
**IQANK1**	0.04	9.23E-01	1.00E+00	-0.53	3.64E-01	8.58E-01
**ZMYND19**	-0.25	3.94E-01	1.00E+00	-0.58	3.64E-01	8.58E-01
**IARS**	0.00	9.86E-01	1.00E+00	-0.19	3.64E-01	8.58E-01
**AC007601.1**	0.86	6.92E-01	1.00E+00	-1.45	3.64E-01	8.58E-01
**AC012065.2**	-0.80	7.31E-01	1.00E+00	1.08	3.64E-01	8.58E-01
**STK17B**	0.42	6.83E-02	1.00E+00	0.20	3.64E-01	8.58E-01
**PLD2**	-0.23	3.15E-01	1.00E+00	0.37	3.64E-01	8.58E-01
**RAF1**	0.16	4.37E-01	1.00E+00	0.18	3.64E-01	8.58E-01
**PRR13**	0.09	6.39E-01	1.00E+00	-0.37	3.64E-01	8.58E-01
**BTBD10P2**	0.80	6.20E-01	1.00E+00	1.05	3.64E-01	8.58E-01
**SLC35C1**	0.13	7.12E-01	1.00E+00	0.49	3.64E-01	8.58E-01
**RAB37**	0.39	5.25E-01	1.00E+00	0.63	3.64E-01	8.58E-01

**SLC22A18**	-0.48	7.18E-02	1.00E+00	0.28	3.64E-01	8.58E-01
**AL117339.4**	0.40	8.11E-01	1.00E+00	-1.58	3.64E-01	8.58E-01
**AC022098.1**	0.17	6.82E-01	1.00E+00	-0.56	3.64E-01	8.58E-01
**COG3**	0.33	8.82E-02	1.00E+00	-0.18	3.64E-01	8.58E-01
**SBSN**	0.22	6.76E-01	1.00E+00	0.65	3.64E-01	8.58E-01
**IFNAR1**	0.14	3.53E-01	1.00E+00	-0.21	3.64E-01	8.58E-01
**AP003716.1**	-0.03	9.78E-01	1.00E+00	-0.53	3.65E-01	8.58E-01
**SLC35G2**	0.62	1.81E-01	1.00E+00	-0.60	3.65E-01	8.58E-01
**IGIP**	0.49	3.36E-01	1.00E+00	0.34	3.65E-01	8.58E-01
**PDHX**	0.21	2.58E-01	1.00E+00	-0.22	3.65E-01	8.58E-01
**EFNB3**	-0.84	1.08E-02	6.42E-01	-0.35	3.65E-01	8.58E-01
**SCAPER**	-0.06	8.24E-01	1.00E+00	-0.19	3.65E-01	8.58E-01
**FOXN3-AS1**	-0.68	9.96E-02	1.00E+00	-1.98	3.65E-01	8.58E-01
**ZNF567**	0.04	8.92E-01	1.00E+00	-0.32	3.65E-01	8.58E-01
**KDM1A**	-0.11	4.89E-01	1.00E+00	-0.46	3.65E-01	8.58E-01
**CHMP1A**	-0.26	3.13E-01	1.00E+00	0.21	3.65E-01	8.58E-01
**AC005523.1**	3.78	3.98E-02	1.00E+00	-0.89	3.65E-01	8.58E-01
**ERICH5**	0.36	4.45E-01	1.00E+00	-0.61	3.65E-01	8.58E-01
**TAS2R14**	1.33	3.71E-01	1.00E+00	-0.77	3.65E-01	8.58E-01
**MLLT10**	-0.05	8.34E-01	1.00E+00	0.26	3.65E-01	8.58E-01
**UBE2Q2**	0.10	6.37E-01	1.00E+00	-0.26	3.65E-01	8.59E-01
**RNF40**	-0.17	3.31E-01	1.00E+00	0.50	3.65E-01	8.59E-01
**CLMAT3**	-0.36	8.86E-01	1.00E+00	-0.69	3.65E-01	8.59E-01
**ENO3**	-0.50	3.05E-01	1.00E+00	0.51	3.65E-01	8.59E-01
**CWC22**	-0.02	8.89E-01	1.00E+00	-0.18	3.66E-01	8.59E-01
**MFSD13A**	0.24	4.65E-01	1.00E+00	-0.30	3.66E-01	8.59E-01
**TMEM37**	1.96	1.67E-02	8.12E-01	-0.54	3.66E-01	8.59E-01
**AL132656.1**	-0.03	9.36E-01	1.00E+00	0.81	3.66E-01	8.59E-01
**TMEM185AP1**	0.09	9.43E-01	1.00E+00	-0.53	3.66E-01	8.59E-01
**ACRBP**	-0.34	4.50E-01	1.00E+00	0.90	3.66E-01	8.59E-01
**MAB21L3**	-0.22	6.71E-01	1.00E+00	-0.53	3.66E-01	8.59E-01
**AMIGO2**	0.36	5.82E-01	1.00E+00	0.57	3.66E-01	8.59E-01

**BBC3**	-0.32	2.30E-01	1.00E+00	0.50	3.66E-01	8.59E-01
**AP000769.1**	0.74	2.13E-01	1.00E+00	0.50	3.66E-01	8.59E-01
**FGFRL1**	-0.07	8.73E-01	1.00E+00	0.52	3.66E-01	8.59E-01
**ZNF442**	-0.32	5.29E-01	1.00E+00	-0.49	3.66E-01	8.59E-01
**XLOC_006485**	0.76	7.56E-01	1.00E+00	0.92	3.66E-01	8.59E-01
**TM4SF19-AS1**	-1.01	5.38E-01	1.00E+00	0.85	3.66E-01	8.59E-01
**AL441992.1**	-0.03	9.46E-01	1.00E+00	-0.31	3.66E-01	8.59E-01
**HSPA1L**	0.22	6.02E-01	1.00E+00	0.45	3.66E-01	8.59E-01
**FADD**	-0.08	7.34E-01	1.00E+00	0.32	3.66E-01	8.60E-01
**CEBPA**	-0.25	5.74E-01	1.00E+00	-1.20	3.66E-01	8.60E-01
**EIF3J-DT**	-0.02	9.28E-01	1.00E+00	-0.25	3.66E-01	8.60E-01
**FGGY**	-0.21	5.63E-01	1.00E+00	-0.31	3.66E-01	8.60E-01
**TMOD2**	-0.06	8.76E-01	1.00E+00	0.38	3.66E-01	8.60E-01
**SLC27A3**	0.06	6.89E-01	1.00E+00	-0.49	3.67E-01	8.60E-01
**MYLK**	0.10	8.53E-01	1.00E+00	-0.29	3.67E-01	8.60E-01
**AL121899.1**	-0.14	8.83E-01	1.00E+00	-0.55	3.67E-01	8.60E-01
**ESRG**	1.22	1.16E-01	1.00E+00	-0.74	3.67E-01	8.60E-01
**DENND1C**	-0.02	9.65E-01	1.00E+00	0.64	3.67E-01	8.60E-01
**EBF4**	-0.13	7.03E-01	1.00E+00	0.78	3.67E-01	8.60E-01
**G33057**	0.39	7.82E-01	1.00E+00	0.96	3.67E-01	8.60E-01
**TNFAIP8L3**	0.03	9.33E-01	1.00E+00	-0.40	3.67E-01	8.60E-01
**CCDC167**	-0.20	4.85E-01	1.00E+00	0.28	3.67E-01	8.60E-01
**SUMO2P17**	-0.54	6.30E-01	1.00E+00	-0.49	3.67E-01	8.60E-01
**EFHC1**	0.15	6.81E-01	1.00E+00	-0.26	3.67E-01	8.60E-01
**RABGGTA**	-0.44	7.38E-02	1.00E+00	0.18	3.67E-01	8.60E-01
**XLOC_000076**	-0.23	6.79E-01	1.00E+00	0.78	3.67E-01	8.60E-01
**ATAD2B**	-0.03	9.38E-01	1.00E+00	-0.26	3.67E-01	8.60E-01
**AC068722.2**	-1.96	3.00E-01	1.00E+00	0.87	3.67E-01	8.60E-01
**GGT5**	-0.41	3.46E-01	1.00E+00	0.57	3.67E-01	8.60E-01
**CCDC112**	-0.07	8.26E-01	1.00E+00	-0.24	3.67E-01	8.60E-01
**DHX37**	-0.23	3.24E-01	1.00E+00	0.64	3.67E-01	8.61E-01
**ZDHHC24**	-0.07	7.81E-01	1.00E+00	0.27	3.68E-01	8.61E-01

**SNORD3A**	0.09	9.28E-01	1.00E+00	-0.60	3.68E-01	8.61E-01
**NECTIN1**	-0.30	4.31E-01	1.00E+00	0.56	3.68E-01	8.61E-01
**SHC4**	-0.29	5.99E-01	1.00E+00	0.54	3.68E-01	8.61E-01
**PIP5K1B**	0.47	5.29E-01	1.00E+00	-0.61	3.68E-01	8.61E-01
**EMD**	-0.14	5.02E-01	1.00E+00	0.20	3.68E-01	8.61E-01
**LINC01184**	-0.11	6.36E-01	1.00E+00	-0.20	3.68E-01	8.61E-01
**CBWD1**	-0.23	3.00E-01	1.00E+00	-0.24	3.68E-01	8.61E-01
**ARFRP1**	0.13	6.14E-01	1.00E+00	0.24	3.68E-01	8.61E-01
**STAT4**	-0.67	3.91E-01	1.00E+00	0.63	3.68E-01	8.61E-01
**GUK1**	-0.06	7.94E-01	1.00E+00	-0.23	3.68E-01	8.61E-01
**CLDN12**	0.31	1.87E-01	1.00E+00	-0.25	3.68E-01	8.61E-01
**NSMF**	-0.33	2.92E-01	1.00E+00	0.50	3.68E-01	8.61E-01
**G40986**	0.75	8.23E-01	1.00E+00	-1.19	3.68E-01	8.61E-01
**AL049840.3**	0.30	6.61E-01	1.00E+00	-0.47	3.68E-01	8.61E-01
**CEP104**	-0.07	6.62E-01	1.00E+00	-0.35	3.68E-01	8.61E-01
**KIAA0319**	-0.21	7.85E-01	1.00E+00	0.72	3.68E-01	8.61E-01
**OR5BA1P**	0.24	7.97E-01	1.00E+00	1.13	3.68E-01	8.61E-01
**KIF27**	0.04	9.04E-01	1.00E+00	-0.35	3.69E-01	8.61E-01
**PRPF38B**	0.16	5.08E-01	1.00E+00	0.25	3.69E-01	8.61E-01
**AC019097.1**	-0.51	6.39E-01	1.00E+00	0.53	3.69E-01	8.62E-01
**AC002480.1**	-2.65	4.30E-01	1.00E+00	1.30	3.69E-01	8.62E-01
**AC009318.3**	0.46	7.10E-01	1.00E+00	0.67	3.69E-01	8.62E-01
**TMPRSS13**	-0.05	9.27E-01	1.00E+00	0.50	3.69E-01	8.62E-01
**AC017116.2**	0.92	4.77E-01	1.00E+00	0.57	3.69E-01	8.62E-01
**ARHGAP27**	-0.39	2.15E-01	1.00E+00	0.50	3.69E-01	8.62E-01
**SLC22A17**	-0.25	5.72E-01	1.00E+00	0.55	3.69E-01	8.62E-01
**LINC00520**	0.01	9.95E-01	1.00E+00	0.73	3.69E-01	8.62E-01
**LEPR**	0.92	5.92E-02	1.00E+00	0.45	3.69E-01	8.62E-01
**NEAT1**	-0.22	5.28E-01	1.00E+00	0.30	3.69E-01	8.62E-01
**RANBP9**	-0.10	7.67E-01	1.00E+00	0.28	3.69E-01	8.62E-01
**RNF135**	-0.08	7.38E-01	1.00E+00	-0.17	3.69E-01	8.62E-01
**MYOCD**	-0.16	7.94E-01	1.00E+00	-0.63	3.69E-01	8.62E-01

**AC132008.1**	0.47	7.87E-01	1.00E+00	-1.02	3.69E-01	8.62E-01
**PTGER1**	0.36	7.21E-01	1.00E+00	1.02	3.69E-01	8.62E-01
**AC098818.2**	1.66	3.97E-01	1.00E+00	0.96	3.69E-01	8.62E-01
**TSPAN2**	0.09	8.28E-01	1.00E+00	-0.24	3.69E-01	8.62E-01
**ADGRD2**	-2.60	2.92E-02	9.90E-01	1.09	3.69E-01	8.62E-01
**OGDHL**	-2.95	2.28E-01	1.00E+00	1.14	3.70E-01	8.62E-01
**AC021106.1**	0.87	1.88E-01	1.00E+00	0.78	3.70E-01	8.62E-01
**AC008507.1**	0.99	1.92E-01	1.00E+00	-0.60	3.70E-01	8.62E-01
**G31721**	1.35	6.95E-01	1.00E+00	2.22	3.70E-01	8.62E-01
**ACY3**	-0.96	4.62E-01	1.00E+00	1.18	3.70E-01	8.62E-01
**RPL4**	-0.27	4.42E-01	1.00E+00	0.24	3.70E-01	8.63E-01
**XLOC_003874**	-0.43	4.86E-01	1.00E+00	-0.71	3.70E-01	8.63E-01
**PPP1R11**	-0.20	2.13E-01	1.00E+00	-0.20	3.70E-01	8.63E-01
**CHMP4C**	0.19	6.21E-01	1.00E+00	-0.43	3.70E-01	8.63E-01
**SLC18B1**	0.30	3.21E-01	1.00E+00	0.19	3.70E-01	8.63E-01
**AC090061.1**	1.47	5.76E-01	1.00E+00	-1.20	3.70E-01	8.63E-01
**CFAP157**	0.25	5.34E-01	1.00E+00	0.93	3.70E-01	8.63E-01
**GSE1**	-0.38	1.68E-01	1.00E+00	0.26	3.70E-01	8.63E-01
**TAF1**	0.08	7.02E-01	1.00E+00	0.34	3.70E-01	8.63E-01
**DSTNP2**	-0.43	3.09E-01	1.00E+00	0.30	3.70E-01	8.63E-01
**AL450326.1**	-0.28	5.21E-01	1.00E+00	0.37	3.71E-01	8.63E-01
**AC068647.1**	0.95	8.31E-02	1.00E+00	-0.83	3.71E-01	8.63E-01
**CYYR1**	0.07	8.70E-01	1.00E+00	-0.41	3.71E-01	8.63E-01
**MANEA-DT**	-0.15	8.28E-01	1.00E+00	-0.46	3.71E-01	8.63E-01
**CTLA4**	-2.63	1.45E-01	1.00E+00	1.28	3.71E-01	8.63E-01
**STRA6**	0.34	7.93E-01	1.00E+00	1.06	3.71E-01	8.63E-01
**PIP5K1C**	-0.51	5.13E-02	1.00E+00	0.55	3.71E-01	8.63E-01
**LPCAT3**	-0.24	2.53E-01	1.00E+00	-0.21	3.71E-01	8.63E-01
**COA6**	0.35	1.07E-01	1.00E+00	-0.30	3.71E-01	8.63E-01
**SYNCRIP**	-0.17	4.11E-01	1.00E+00	0.15	3.71E-01	8.63E-01
**CCL19**	-0.81	2.21E-01	1.00E+00	0.60	3.71E-01	8.63E-01
**CYTH2**	0.03	8.74E-01	1.00E+00	0.14	3.71E-01	8.63E-01

**ACTC1**	-2.23	1.52E-02	7.66E-01	-0.74	3.71E-01	8.63E-01
**FCGBP**	-0.79	1.51E-01	1.00E+00	-0.53	3.71E-01	8.63E-01
**FSBP**	-0.36	6.64E-01	1.00E+00	0.50	3.71E-01	8.63E-01
**DLG5-AS1**	-0.37	3.87E-01	1.00E+00	-0.51	3.71E-01	8.63E-01
**AL031274.1**	-0.79	4.60E-01	1.00E+00	0.73	3.71E-01	8.63E-01
**SPRED1**	0.42	1.37E-01	1.00E+00	0.40	3.71E-01	8.63E-01
**INTS6**	0.00	9.99E-01	1.00E+00	-0.26	3.71E-01	8.63E-01
**G16028**	4.05	1.15E-01	1.00E+00	1.42	3.71E-01	8.63E-01
**ABHD17B**	-0.03	9.13E-01	1.00E+00	-0.33	3.71E-01	8.63E-01
**PDE12**	-0.15	5.19E-01	1.00E+00	0.17	3.71E-01	8.63E-01
**TNNI2**	-0.27	5.48E-01	1.00E+00	0.48	3.71E-01	8.63E-01
**AC097523.1**	-2.49	1.45E-01	1.00E+00	-0.42	3.71E-01	8.63E-01
**CREBZF**	-0.16	6.66E-01	1.00E+00	-0.56	3.71E-01	8.63E-01
**MXRA7**	0.25	5.87E-01	1.00E+00	-0.57	3.71E-01	8.63E-01
**ACP7**	0.45	4.99E-01	1.00E+00	0.63	3.71E-01	8.63E-01
**ZNF780A**	0.96	1.66E-01	1.00E+00	-0.43	3.71E-01	8.63E-01
**HERPUD2**	-0.12	5.56E-01	1.00E+00	-0.24	3.71E-01	8.63E-01
**SERPINB4**	-3.24	5.27E-04	1.04E-01	2.80	3.72E-01	8.63E-01
**MEIOB**	0.59	4.95E-01	1.00E+00	-0.67	3.72E-01	8.64E-01
**AC093690.1**	-2.34	2.67E-01	1.00E+00	-1.00	3.72E-01	8.64E-01
**AL139089.1**	2.59	2.26E-01	1.00E+00	-0.93	3.72E-01	8.64E-01
**G7791**	0.40	6.73E-01	1.00E+00	1.12	3.72E-01	8.64E-01
**CHSY3**	-0.81	4.71E-02	1.00E+00	0.76	3.72E-01	8.64E-01
**HSPD1P6**	-3.36	5.25E-02	1.00E+00	-0.92	3.72E-01	8.64E-01
**SDF2**	0.15	3.00E-01	1.00E+00	0.16	3.72E-01	8.64E-01
**STOML1**	-0.16	6.28E-01	1.00E+00	0.27	3.72E-01	8.64E-01
**RPL4P6**	0.59	4.75E-01	1.00E+00	-0.97	3.72E-01	8.64E-01
**OR7E102P**	-0.52	8.59E-01	1.00E+00	-1.39	3.72E-01	8.64E-01
**RPAP1**	-0.15	4.57E-01	1.00E+00	-0.37	3.72E-01	8.64E-01
**AP003472.1**	-1.55	4.54E-01	1.00E+00	-0.76	3.72E-01	8.64E-01
**CCNT1**	0.32	2.44E-01	1.00E+00	0.28	3.72E-01	8.64E-01
**C10orf67**	1.04	8.10E-02	1.00E+00	0.74	3.73E-01	8.64E-01

**CTD-2201I18.1**	0.62	5.97E-01	1.00E+00	-1.16	3.73E-01	8.64E-01
**AP003721.4**	0.49	6.63E-01	1.00E+00	-1.16	3.73E-01	8.64E-01
**AC106795.1**	0.11	7.78E-01	1.00E+00	-0.33	3.73E-01	8.64E-01
**NME1**	0.10	7.39E-01	1.00E+00	-0.39	3.73E-01	8.64E-01
**AC099343.3**	-0.33	2.99E-01	1.00E+00	0.45	3.73E-01	8.64E-01
**FOLR1**	-0.45	5.42E-01	1.00E+00	-0.65	3.73E-01	8.64E-01
**POLD4**	0.11	7.62E-01	1.00E+00	0.38	3.73E-01	8.64E-01
**PIGB**	-0.10	7.21E-01	1.00E+00	-0.33	3.73E-01	8.64E-01
**SLX4**	-0.58	6.87E-02	1.00E+00	0.75	3.73E-01	8.64E-01
**FBXL15**	-0.09	7.08E-01	1.00E+00	0.21	3.73E-01	8.64E-01
**AC107959.3**	-0.04	9.88E-01	1.00E+00	1.52	3.73E-01	8.64E-01
**DGKZ**	-0.23	3.51E-01	1.00E+00	0.53	3.73E-01	8.64E-01
**RAB3IP**	0.12	6.12E-01	1.00E+00	-0.24	3.73E-01	8.64E-01
**GZMH**	-0.62	4.30E-01	1.00E+00	0.85	3.73E-01	8.64E-01
**CHAF1A**	0.02	9.28E-01	1.00E+00	-0.37	3.73E-01	8.64E-01
**DNM3**	-0.28	4.09E-01	1.00E+00	-0.44	3.73E-01	8.64E-01
**SNX30**	0.40	7.94E-02	1.00E+00	0.32	3.73E-01	8.64E-01
**AC020910.4**	-0.62	1.84E-01	1.00E+00	-0.41	3.73E-01	8.65E-01
**AGER**	-0.16	8.33E-01	1.00E+00	0.89	3.73E-01	8.65E-01
**LINC01343**	-2.44	1.25E-01	1.00E+00	0.98	3.73E-01	8.65E-01
**LAX1**	-0.02	9.79E-01	1.00E+00	0.85	3.74E-01	8.65E-01
**DDX1**	-0.08	6.22E-01	1.00E+00	-0.20	3.74E-01	8.65E-01
**AZIN1-AS1**	-0.29	5.08E-01	1.00E+00	-0.49	3.74E-01	8.65E-01
**RMDN2**	-0.57	1.65E-01	1.00E+00	0.27	3.74E-01	8.65E-01
**RN7SL600P**	-0.20	8.69E-01	1.00E+00	0.76	3.74E-01	8.65E-01
**MRPL40**	-0.18	3.26E-01	1.00E+00	-0.26	3.74E-01	8.65E-01
**TAGLN**	-0.47	4.08E-01	1.00E+00	0.39	3.74E-01	8.65E-01
**NPPC**	-3.45	2.59E-03	2.61E-01	-0.73	3.74E-01	8.65E-01
**PTGES3P3**	-1.52	6.60E-01	1.00E+00	-0.55	3.74E-01	8.65E-01
**DLGAP1-AS1**	0.54	2.03E-01	1.00E+00	0.39	3.74E-01	8.65E-01
**ACTG1P23**	-1.52	6.60E-01	1.00E+00	0.78	3.74E-01	8.65E-01
**ZMYND8**	0.02	9.19E-01	1.00E+00	0.21	3.74E-01	8.65E-01

**SGMS2**	0.19	4.31E-01	1.00E+00	0.26	3.74E-01	8.65E-01
**IGHV3-21**	1.06	7.61E-01	1.00E+00	2.83	3.74E-01	8.65E-01
**MIF-AS1**	-0.56	6.09E-01	1.00E+00	-0.82	3.74E-01	8.65E-01
**PLPP7**	0.28	6.07E-01	1.00E+00	0.43	3.74E-01	8.65E-01
**LURAP1**	-0.27	5.72E-01	1.00E+00	0.41	3.74E-01	8.65E-01
**DDX5**	-0.26	1.82E-01	1.00E+00	0.17	3.74E-01	8.65E-01
**EAPP**	-0.11	6.05E-01	1.00E+00	-0.31	3.74E-01	8.65E-01
**B4GALNT2**	0.24	6.84E-01	1.00E+00	-0.80	3.74E-01	8.65E-01
**SYN3**	-0.10	8.66E-01	1.00E+00	-0.75	3.74E-01	8.65E-01
**AC005264.1**	-0.32	8.23E-01	1.00E+00	1.19	3.74E-01	8.65E-01
**SH3TC1**	-0.47	1.86E-01	1.00E+00	0.78	3.74E-01	8.65E-01
**AL139158.2**	0.02	9.60E-01	1.00E+00	-0.50	3.74E-01	8.65E-01
**ZNF257**	0.43	5.22E-01	1.00E+00	-0.54	3.75E-01	8.65E-01
**AC107241.1**	2.25	3.37E-01	1.00E+00	0.96	3.75E-01	8.66E-01
**AC008391.1**	0.04	9.75E-01	1.00E+00	0.81	3.75E-01	8.66E-01
**DNMT3B**	-0.41	3.15E-01	1.00E+00	0.58	3.75E-01	8.66E-01
**MAP3K13**	-0.05	7.74E-01	1.00E+00	-0.22	3.75E-01	8.66E-01
**ZNF490**	-0.33	3.93E-01	1.00E+00	0.39	3.75E-01	8.66E-01
**DIO1**	-2.41	3.77E-01	1.00E+00	1.12	3.75E-01	8.66E-01
**ZNF470**	-0.21	5.78E-01	1.00E+00	-0.26	3.75E-01	8.66E-01
**AL356056.3**	-0.80	6.90E-01	1.00E+00	-0.61	3.75E-01	8.67E-01
**GNLY**	-1.61	2.08E-02	8.94E-01	0.69	3.75E-01	8.67E-01
**CEBPB-AS1**	0.00	9.94E-01	1.00E+00	-0.74	3.75E-01	8.67E-01
**GTF2IRD2B**	-0.02	9.56E-01	1.00E+00	-0.42	3.75E-01	8.67E-01
**RAB4B**	-2.25	2.04E-01	1.00E+00	0.70	3.76E-01	8.67E-01
**ELL**	-0.21	4.29E-01	1.00E+00	0.48	3.76E-01	8.67E-01
**MAPK12**	-0.35	4.96E-01	1.00E+00	0.78	3.76E-01	8.67E-01
**TRPM6**	0.41	3.90E-01	1.00E+00	0.69	3.76E-01	8.67E-01
**AC087257.1**	-0.79	6.16E-01	1.00E+00	0.99	3.76E-01	8.67E-01
**NEK8**	-0.26	4.87E-01	1.00E+00	0.34	3.76E-01	8.67E-01
**E2F7**	-0.51	5.36E-01	1.00E+00	0.56	3.76E-01	8.67E-01
**EXOC4**	0.03	9.16E-01	1.00E+00	0.24	3.76E-01	8.67E-01

**AP001107.5**	-0.67	5.25E-01	1.00E+00	0.89	3.76E-01	8.67E-01
**TRIP10**	0.09	7.48E-01	1.00E+00	0.36	3.76E-01	8.67E-01
**AC068481.1**	1.51	4.95E-01	1.00E+00	-0.87	3.76E-01	8.67E-01
**XLOC_001309**	0.85	2.34E-01	1.00E+00	-0.67	3.76E-01	8.67E-01
**SMC1B**	-1.70	2.75E-01	1.00E+00	-0.89	3.76E-01	8.67E-01
**MS4A2**	0.61	3.71E-01	1.00E+00	0.59	3.76E-01	8.68E-01
**NDUFB10**	-0.14	4.44E-01	1.00E+00	-0.18	3.76E-01	8.68E-01
**STARD10**	-0.18	4.72E-01	1.00E+00	-0.33	3.76E-01	8.68E-01
**TRIM61**	2.37	9.73E-02	1.00E+00	1.00	3.77E-01	8.68E-01
**ZNF711**	-0.07	8.65E-01	1.00E+00	-0.48	3.77E-01	8.68E-01
**ESYT3**	0.20	7.26E-01	1.00E+00	0.46	3.77E-01	8.68E-01
**ELMOD3**	1.47	3.03E-02	1.00E+00	-0.55	3.77E-01	8.68E-01
**ZNF367**	-0.45	1.87E-01	1.00E+00	-0.53	3.77E-01	8.68E-01
**MUC1**	-0.83	1.20E-01	1.00E+00	0.70	3.77E-01	8.68E-01
**MMP16**	0.13	8.27E-01	1.00E+00	-0.48	3.77E-01	8.68E-01
**ZFP37**	0.60	3.73E-01	1.00E+00	0.59	3.77E-01	8.68E-01
**SYNRG**	-0.09	6.63E-01	1.00E+00	0.31	3.77E-01	8.68E-01
**PLEKHF1**	-0.02	9.39E-01	1.00E+00	0.43	3.77E-01	8.68E-01
**GID8**	0.12	6.51E-01	1.00E+00	0.25	3.77E-01	8.68E-01
**SNORA77**	0.88	4.28E-01	1.00E+00	0.95	3.77E-01	8.68E-01
**MT-ND2**	-0.04	9.26E-01	1.00E+00	-0.28	3.77E-01	8.68E-01
**TRDMT1**	-0.29	2.53E-01	1.00E+00	-0.32	3.77E-01	8.68E-01
**ZNF721**	-0.30	4.28E-01	1.00E+00	-0.26	3.77E-01	8.68E-01
**AC024941.2**	0.69	8.22E-01	1.00E+00	-0.95	3.77E-01	8.68E-01
**CD82**	-0.02	9.34E-01	1.00E+00	0.19	3.77E-01	8.68E-01
**XLOC_007051**	1.67	6.26E-01	1.00E+00	-1.52	3.77E-01	8.68E-01
**TIMP3**	0.71	1.35E-01	1.00E+00	-0.47	3.77E-01	8.68E-01
**GLIPR1L1**	1.49	2.12E-01	1.00E+00	-0.61	3.77E-01	8.68E-01
**PAIP2**	0.24	2.54E-01	1.00E+00	-0.35	3.77E-01	8.68E-01
**AC112777.1**	-0.19	5.37E-01	1.00E+00	0.62	3.77E-01	8.68E-01
**RPS15P5**	1.02	7.67E-01	1.00E+00	-0.72	3.77E-01	8.68E-01
**NRBP1**	-0.10	5.93E-01	1.00E+00	0.13	3.78E-01	8.68E-01

**DNAJB2**	0.24	4.34E-01	1.00E+00	0.43	3.78E-01	8.68E-01
**ANGPTL8**	5.11	2.44E-02	9.22E-01	-1.00	3.78E-01	8.68E-01
**SHB**	-0.24	4.67E-01	1.00E+00	0.50	3.78E-01	8.68E-01
**AC024075.1**	-0.05	9.39E-01	1.00E+00	0.46	3.78E-01	8.68E-01
**RPS27AP11**	-0.10	9.61E-01	1.00E+00	-0.68	3.78E-01	8.68E-01
**SLN**	0.66	4.79E-01	1.00E+00	0.89	3.78E-01	8.68E-01
**SEC23A**	0.34	2.28E-01	1.00E+00	-0.32	3.78E-01	8.68E-01
**BBOF1**	-0.10	7.68E-01	1.00E+00	-0.27	3.78E-01	8.68E-01
**FZD10-DT**	0.12	7.76E-01	1.00E+00	0.40	3.78E-01	8.68E-01
**GRIA2**	-0.24	8.82E-01	1.00E+00	-0.91	3.78E-01	8.68E-01
**PWAR6**	-0.52	4.50E-01	1.00E+00	-0.66	3.78E-01	8.68E-01
**YOD1**	0.37	5.35E-01	1.00E+00	0.41	3.78E-01	8.68E-01
**TMEM8A**	-0.15	7.11E-01	1.00E+00	0.64	3.78E-01	8.68E-01
**AL133355.1**	-0.01	9.87E-01	1.00E+00	0.48	3.78E-01	8.68E-01
**ZNF32**	0.09	7.31E-01	1.00E+00	-0.13	3.78E-01	8.68E-01
**SPDYE21P**	-0.44	6.84E-01	1.00E+00	0.43	3.78E-01	8.68E-01
**EML6**	-0.74	1.82E-01	1.00E+00	0.65	3.78E-01	8.68E-01
**PDE8B**	-0.19	6.89E-01	1.00E+00	-0.42	3.78E-01	8.68E-01
**SMC2**	-0.17	4.98E-01	1.00E+00	-0.13	3.78E-01	8.68E-01
**POMP**	0.08	6.86E-01	1.00E+00	-0.32	3.78E-01	8.68E-01
**LINC01956**	-0.45	5.21E-01	1.00E+00	-0.46	3.78E-01	8.68E-01
**MRPL4**	-0.24	3.44E-01	1.00E+00	0.19	3.78E-01	8.68E-01
**G10846**	-0.58	5.00E-01	1.00E+00	-0.59	3.78E-01	8.68E-01
**LPGAT1**	0.68	1.77E-01	1.00E+00	-0.39	3.78E-01	8.68E-01
**GFRA1**	0.92	7.42E-02	1.00E+00	0.42	3.78E-01	8.68E-01
**DIRC3**	0.65	3.41E-01	1.00E+00	-0.41	3.78E-01	8.68E-01
**AL354993.1**	0.48	7.94E-01	1.00E+00	-0.85	3.78E-01	8.68E-01
**LINC01133**	-0.35	2.97E-01	1.00E+00	-0.43	3.78E-01	8.68E-01
**LINC00511**	-0.80	5.20E-02	1.00E+00	0.44	3.78E-01	8.68E-01
**VSIG1**	-0.86	2.69E-01	1.00E+00	1.03	3.78E-01	8.68E-01
**XLOC_012301**	-0.59	6.62E-01	1.00E+00	-1.21	3.79E-01	8.68E-01
**WDR4**	-0.33	2.35E-01	1.00E+00	0.27	3.79E-01	8.68E-01

**AC008429.2**	-0.09	9.78E-01	1.00E+00	-0.50	3.79E-01	8.68E-01
**FUT2**	0.12	7.58E-01	1.00E+00	0.47	3.79E-01	8.68E-01
**DPM2**	-0.23	3.29E-01	1.00E+00	0.15	3.79E-01	8.68E-01
**NUDT2**	-0.56	2.50E-02	9.28E-01	-0.22	3.79E-01	8.68E-01
**AC074134.1**	2.53	4.56E-01	1.00E+00	1.17	3.79E-01	8.68E-01
**STRCP1**	-3.78	3.53E-03	3.20E-01	-1.06	3.79E-01	8.68E-01
**ASB11**	-0.17	8.53E-01	1.00E+00	-0.80	3.79E-01	8.68E-01
**MYH9**	-0.29	2.13E-01	1.00E+00	0.52	3.79E-01	8.68E-01
**TET1**	-0.52	2.60E-01	1.00E+00	-0.41	3.79E-01	8.68E-01
**KNTC1**	-0.12	6.75E-01	1.00E+00	0.34	3.79E-01	8.68E-01
**RPL7P1**	0.05	8.69E-01	1.00E+00	-0.48	3.79E-01	8.68E-01
**DSG4**	-1.95	6.91E-02	1.00E+00	-1.13	3.79E-01	8.68E-01
**GLTPD2**	-0.94	5.82E-02	1.00E+00	0.80	3.79E-01	8.68E-01
**PPP2R1B**	0.88	6.36E-02	1.00E+00	-0.28	3.79E-01	8.68E-01
**CCDC39**	0.44	7.13E-01	1.00E+00	0.88	3.79E-01	8.68E-01
**ALG1L9P**	-0.67	2.14E-01	1.00E+00	0.62	3.79E-01	8.68E-01
**MSTO1**	0.18	6.95E-01	1.00E+00	-0.38	3.79E-01	8.68E-01
**G31097**	-1.25	1.58E-01	1.00E+00	-0.63	3.79E-01	8.68E-01
**XLOC_008366**	1.49	5.28E-01	1.00E+00	1.03	3.79E-01	8.68E-01
**AC017104.1**	-0.51	6.02E-01	1.00E+00	1.23	3.79E-01	8.68E-01
**ZC2HC1C**	0.01	9.76E-01	1.00E+00	-0.58	3.79E-01	8.68E-01
**AUH**	0.31	2.77E-01	1.00E+00	-0.31	3.79E-01	8.68E-01
**LINC01422**	0.10	9.39E-01	1.00E+00	-1.05	3.79E-01	8.68E-01
**CCNE1**	-0.86	7.82E-02	1.00E+00	0.62	3.79E-01	8.68E-01
**MIR3150BHG**	-0.36	7.01E-01	1.00E+00	0.82	3.79E-01	8.68E-01
**NIPBL-DT**	0.41	2.36E-01	1.00E+00	-0.40	3.79E-01	8.68E-01
**STMN3**	-0.26	3.81E-01	1.00E+00	0.51	3.79E-01	8.68E-01
**MRPL2**	0.00	9.95E-01	1.00E+00	-0.18	3.79E-01	8.68E-01
**AC087741.2**	-2.93	2.81E-01	1.00E+00	-0.57	3.80E-01	8.68E-01
**ACTA1**	-1.56	1.98E-01	1.00E+00	-0.79	3.80E-01	8.68E-01
**XLOC_008703**	0.49	8.86E-01	1.00E+00	1.13	3.80E-01	8.68E-01
**MAP4K2**	-0.08	7.35E-01	1.00E+00	0.31	3.80E-01	8.68E-01

**AC115522.1**	-0.27	5.73E-01	1.00E+00	0.44	3.80E-01	8.68E-01
**PPP1R14B**	-0.13	6.71E-01	1.00E+00	-0.54	3.80E-01	8.68E-01
**C9orf72**	0.19	5.62E-01	1.00E+00	0.23	3.80E-01	8.68E-01
**FAM208B**	-0.42	2.55E-02	9.29E-01	0.36	3.80E-01	8.68E-01
**RASSF9**	-0.75	4.53E-02	1.00E+00	-0.35	3.80E-01	8.68E-01
**DUSP12**	-0.09	7.47E-01	1.00E+00	0.21	3.80E-01	8.68E-01
**AC004951.1**	-1.19	2.74E-01	1.00E+00	-0.59	3.80E-01	8.68E-01
**AKAP9**	-0.01	9.73E-01	1.00E+00	-0.28	3.80E-01	8.68E-01
**C22orf34**	-0.08	8.95E-01	1.00E+00	0.69	3.80E-01	8.68E-01
**PAQR4**	-0.54	1.98E-01	1.00E+00	-0.58	3.80E-01	8.68E-01
**AZIN2**	-0.01	9.91E-01	1.00E+00	0.55	3.80E-01	8.68E-01
**MRPS33**	-0.02	9.02E-01	1.00E+00	-0.25	3.80E-01	8.68E-01
**COQ7**	0.19	5.25E-01	1.00E+00	-0.17	3.80E-01	8.68E-01
**KCND2**	0.53	4.99E-01	1.00E+00	0.66	3.80E-01	8.68E-01
**AC124283.4**	0.04	9.81E-01	1.00E+00	1.02	3.80E-01	8.68E-01
**SWI5**	0.03	9.29E-01	1.00E+00	-0.21	3.80E-01	8.68E-01
**SLC35D1**	0.45	1.98E-01	1.00E+00	0.19	3.80E-01	8.68E-01
**ELP1**	0.27	2.25E-01	1.00E+00	0.25	3.80E-01	8.68E-01
**KCTD15**	-0.03	9.42E-01	1.00E+00	0.34	3.80E-01	8.68E-01
**CARD18**	-0.08	9.18E-01	1.00E+00	-0.58	3.80E-01	8.68E-01
**THRAP3**	-0.08	7.31E-01	1.00E+00	0.18	3.81E-01	8.68E-01
**MINOS1P3**	0.36	9.17E-01	1.00E+00	1.49	3.81E-01	8.68E-01
**TP53TG3D**	-1.62	1.85E-01	1.00E+00	-0.79	3.81E-01	8.68E-01
**AMPH**	-0.14	8.37E-01	1.00E+00	0.65	3.81E-01	8.68E-01
**PPP6R1**	-0.21	3.86E-01	1.00E+00	0.68	3.81E-01	8.68E-01
**MTFMT**	0.01	9.68E-01	1.00E+00	0.17	3.81E-01	8.68E-01
**ZBTB18**	-0.07	7.13E-01	1.00E+00	0.39	3.81E-01	8.68E-01
**GKN1**	-2.77	1.89E-01	1.00E+00	-1.58	3.81E-01	8.68E-01
**DLEU1**	0.07	8.36E-01	1.00E+00	-0.36	3.81E-01	8.68E-01
**LINC00663**	-1.38	3.32E-01	1.00E+00	-0.58	3.81E-01	8.68E-01
**AC087516.2**	0.09	9.58E-01	1.00E+00	-1.17	3.81E-01	8.68E-01
**RPF2**	0.07	7.64E-01	1.00E+00	-0.23	3.81E-01	8.68E-01

**RANBP6**	-0.06	8.06E-01	1.00E+00	-0.23	3.81E-01	8.68E-01
**C10orf88**	-0.14	6.09E-01	1.00E+00	-0.24	3.81E-01	8.68E-01
**RPL12P44**	-0.83	8.07E-01	1.00E+00	-0.81	3.81E-01	8.68E-01
**TNFRSF25**	0.59	3.32E-01	1.00E+00	0.57	3.81E-01	8.68E-01
**AL512625.3**	-2.14	4.76E-01	1.00E+00	-1.09	3.81E-01	8.68E-01
**SPINK8**	-0.40	8.85E-01	1.00E+00	-1.67	3.81E-01	8.68E-01
**IRX4**	-0.61	7.54E-02	1.00E+00	-0.69	3.81E-01	8.68E-01
**AC136621.1**	0.56	3.68E-01	1.00E+00	-0.87	3.81E-01	8.68E-01
**XLOC_003405**	0.97	3.13E-01	1.00E+00	-1.06	3.81E-01	8.68E-01
**G42447**	0.43	7.76E-01	1.00E+00	-0.80	3.81E-01	8.68E-01
**AC009063.3**	-2.54	1.95E-01	1.00E+00	0.77	3.81E-01	8.68E-01
**RGS9BP**	0.10	8.79E-01	1.00E+00	-0.87	3.81E-01	8.68E-01
**RHOC**	0.01	9.72E-01	1.00E+00	0.18	3.81E-01	8.68E-01
**IGLV1-44**	3.64	2.31E-03	2.43E-01	2.75	3.81E-01	8.68E-01
**GABRP**	-0.13	8.52E-01	1.00E+00	0.70	3.81E-01	8.68E-01
**SPRR3**	-1.24	7.17E-01	1.00E+00	2.78	3.82E-01	8.68E-01
**ABCA6**	0.47	3.53E-01	1.00E+00	0.44	3.82E-01	8.68E-01
**SLFN13**	0.01	9.78E-01	1.00E+00	0.31	3.82E-01	8.68E-01
**RAET1E**	0.08	9.03E-01	1.00E+00	0.47	3.82E-01	8.68E-01
**PSG5**	0.56	6.91E-01	1.00E+00	-0.88	3.82E-01	8.68E-01
**HELLPAR**	1.00	3.67E-01	1.00E+00	-0.60	3.82E-01	8.68E-01
**CRACR2B**	0.32	6.49E-01	1.00E+00	-0.36	3.82E-01	8.68E-01
**ABCA9**	0.31	6.13E-01	1.00E+00	0.59	3.82E-01	8.68E-01
**LINC01451**	-0.99	3.29E-01	1.00E+00	-0.66	3.82E-01	8.68E-01
**ACTG1P10**	-1.39	3.60E-01	1.00E+00	-1.13	3.82E-01	8.68E-01
**IL25**	-3.90	4.29E-02	1.00E+00	-1.08	3.82E-01	8.68E-01
**MT-TF**	0.51	5.27E-01	1.00E+00	-0.38	3.82E-01	8.68E-01
**RGS7**	2.37	4.34E-02	1.00E+00	1.31	3.82E-01	8.68E-01
**CHID1**	0.03	8.94E-01	1.00E+00	0.11	3.82E-01	8.68E-01
**RAI1**	-0.71	3.27E-02	1.00E+00	0.72	3.82E-01	8.68E-01
**ALKBH7**	-0.08	7.44E-01	1.00E+00	0.16	3.82E-01	8.68E-01
**AC016831.6**	0.96	4.35E-01	1.00E+00	0.56	3.82E-01	8.68E-01

**AL355607.1**	-1.13	6.37E-01	1.00E+00	-1.14	3.82E-01	8.68E-01
**APBB3**	-0.43	2.23E-01	1.00E+00	0.37	3.82E-01	8.68E-01
**G43312**	1.48	8.75E-02	1.00E+00	-0.82	3.82E-01	8.68E-01
**FAM131A**	-0.11	7.62E-01	1.00E+00	0.35	3.82E-01	8.68E-01
**SPATA21**	1.96	3.42E-01	1.00E+00	1.04	3.82E-01	8.68E-01
**AC079305.1**	-0.47	5.89E-01	1.00E+00	0.55	3.82E-01	8.68E-01
**TIMM17B**	0.01	9.39E-01	1.00E+00	0.17	3.82E-01	8.68E-01
**DLG3**	0.16	2.79E-01	1.00E+00	-0.41	3.82E-01	8.68E-01
**SNX14**	0.17	3.24E-01	1.00E+00	-0.17	3.82E-01	8.68E-01
**AP005264.5**	-2.36	8.04E-02	1.00E+00	-0.74	3.82E-01	8.68E-01
**G36605**	0.19	8.29E-01	1.00E+00	0.76	3.82E-01	8.68E-01
**RPGRIP1L**	-0.01	9.86E-01	1.00E+00	0.30	3.83E-01	8.68E-01
**WFIKKN2**	2.55	3.06E-03	2.89E-01	0.91	3.83E-01	8.68E-01
**EIF3H**	-0.17	5.21E-01	1.00E+00	0.15	3.83E-01	8.68E-01
**SNX27**	0.32	1.85E-01	1.00E+00	-0.24	3.83E-01	8.68E-01
**1-Mar**	2.36	5.27E-02	1.00E+00	-0.78	3.83E-01	8.68E-01
**MINCR**	0.43	3.17E-01	1.00E+00	-0.43	3.83E-01	8.68E-01
**TNIP3**	-1.31	5.49E-01	1.00E+00	1.06	3.83E-01	8.68E-01
**LINC00476**	-0.07	8.53E-01	1.00E+00	-0.26	3.83E-01	8.69E-01
**ARFGAP2**	0.13	5.79E-01	1.00E+00	0.14	3.83E-01	8.69E-01
**HNRNPA1P48**	-0.12	7.14E-01	1.00E+00	-0.25	3.83E-01	8.69E-01
**LYPD2**	0.43	4.16E-01	1.00E+00	0.55	3.83E-01	8.69E-01
**HMGN1**	0.00	9.88E-01	1.00E+00	-0.19	3.83E-01	8.69E-01
**XLOC_001642**	-0.85	5.92E-01	1.00E+00	0.94	3.83E-01	8.69E-01
**OTX1**	-0.27	4.01E-01	1.00E+00	-0.41	3.83E-01	8.69E-01
**RIC8B**	-0.04	9.16E-01	1.00E+00	-0.29	3.83E-01	8.69E-01
**RAB11FIP5**	0.06	7.76E-01	1.00E+00	0.47	3.83E-01	8.69E-01
**PIR**	0.11	6.71E-01	1.00E+00	-0.24	3.83E-01	8.69E-01
**RPL18**	-0.21	4.82E-01	1.00E+00	0.27	3.83E-01	8.69E-01
**TMEM245**	0.65	5.17E-02	1.00E+00	-0.26	3.83E-01	8.69E-01
**HOXA7**	-0.40	3.24E-01	1.00E+00	-0.43	3.83E-01	8.69E-01
**GABPB1-IT1**	-0.17	4.96E-01	1.00E+00	-0.50	3.84E-01	8.69E-01

**PFDN6**	-0.10	6.09E-01	1.00E+00	0.26	3.84E-01	8.69E-01
**MAP3K11**	-0.42	2.63E-02	9.46E-01	0.56	3.84E-01	8.69E-01
**ZNRD1ASP**	0.33	5.29E-01	1.00E+00	0.44	3.84E-01	8.69E-01
**SUCNR1**	0.56	4.32E-01	1.00E+00	-0.50	3.84E-01	8.69E-01
**HMGN1P4**	0.54	7.73E-01	1.00E+00	-0.56	3.84E-01	8.69E-01
**SOCS4**	0.09	7.41E-01	1.00E+00	-0.19	3.84E-01	8.69E-01
**VPS11**	0.09	6.14E-01	1.00E+00	0.13	3.84E-01	8.69E-01
**DECR2**	0.15	7.07E-01	1.00E+00	-0.21	3.84E-01	8.69E-01
**SCARA3**	-0.04	8.91E-01	1.00E+00	0.56	3.84E-01	8.69E-01
**CCDC130**	-0.42	1.40E-01	1.00E+00	0.24	3.84E-01	8.69E-01
**RAB6D**	1.06	7.61E-01	1.00E+00	-0.58	3.84E-01	8.69E-01
**AC011476.3**	0.07	9.25E-01	1.00E+00	0.66	3.84E-01	8.69E-01
**CLTA**	0.00	9.78E-01	1.00E+00	0.20	3.84E-01	8.69E-01
**APOBEC3F**	-0.28	4.99E-01	1.00E+00	0.41	3.84E-01	8.69E-01
**G35698**	0.15	9.04E-01	1.00E+00	-0.83	3.84E-01	8.69E-01
**LINC01484**	2.60	1.76E-01	1.00E+00	-1.00	3.84E-01	8.69E-01
**GZF1**	-0.11	6.60E-01	1.00E+00	0.34	3.84E-01	8.69E-01
**ST20-AS1**	-0.44	4.93E-01	1.00E+00	-0.53	3.84E-01	8.69E-01
**TTC27**	0.02	9.06E-01	1.00E+00	-0.18	3.84E-01	8.69E-01
**XLOC_002585**	1.62	5.35E-02	1.00E+00	0.91	3.84E-01	8.69E-01
**TUBG2**	0.03	9.45E-01	1.00E+00	0.21	3.84E-01	8.69E-01
**C9orf41-AS1**	1.88	3.76E-01	1.00E+00	-0.60	3.85E-01	8.69E-01
**USB1**	-0.25	2.38E-01	1.00E+00	0.26	3.85E-01	8.69E-01
**AC109454.2**	-0.34	8.07E-01	1.00E+00	-0.52	3.85E-01	8.69E-01
**AC093390.1**	-2.09	1.19E-01	1.00E+00	1.14	3.85E-01	8.69E-01
**TYK2**	-0.04	8.58E-01	1.00E+00	0.51	3.85E-01	8.69E-01
**AC007364.1**	0.56	8.71E-01	1.00E+00	-0.84	3.85E-01	8.69E-01
**SLC35G1**	0.36	3.98E-01	1.00E+00	-0.34	3.85E-01	8.69E-01
**DNAJB11**	-0.10	5.26E-01	1.00E+00	0.20	3.85E-01	8.69E-01
**PCTP**	0.52	1.59E-01	1.00E+00	0.31	3.85E-01	8.69E-01
**LINC00654**	0.79	2.43E-01	1.00E+00	0.84	3.85E-01	8.69E-01
**AC115618.2**	-0.24	2.07E-01	1.00E+00	0.73	3.85E-01	8.70E-01

**AP000568.1**	-0.06	9.86E-01	1.00E+00	-0.73	3.85E-01	8.70E-01
**LINC01224**	-0.16	8.88E-01	1.00E+00	-1.06	3.85E-01	8.70E-01
**NES**	0.03	9.47E-01	1.00E+00	0.70	3.85E-01	8.70E-01
**MTMR9LP**	-0.17	8.23E-01	1.00E+00	0.60	3.85E-01	8.70E-01
**ZNF30**	-0.04	9.31E-01	1.00E+00	0.35	3.85E-01	8.70E-01
**C8orf33**	0.19	3.11E-01	1.00E+00	-0.16	3.85E-01	8.70E-01
**ZNF277**	-0.11	6.68E-01	1.00E+00	0.18	3.85E-01	8.70E-01
**TSPAN5**	0.26	4.97E-01	1.00E+00	-0.34	3.86E-01	8.70E-01
**NFE2**	0.54	6.88E-01	1.00E+00	0.47	3.86E-01	8.70E-01
**DUSP22**	0.13	3.99E-01	1.00E+00	-0.10	3.86E-01	8.70E-01
**CNEP1R1**	0.23	4.99E-01	1.00E+00	-0.24	3.86E-01	8.70E-01
**LRRC8A**	-0.12	5.44E-01	1.00E+00	0.28	3.86E-01	8.70E-01
**ZNF79**	-0.43	1.58E-01	1.00E+00	0.49	3.86E-01	8.70E-01
**STUM**	-0.26	4.72E-01	1.00E+00	-0.59	3.86E-01	8.70E-01
**TIFAB**	-0.27	7.64E-01	1.00E+00	2.67	3.86E-01	8.70E-01
**DEXI**	-0.26	5.10E-01	1.00E+00	0.87	3.86E-01	8.70E-01
**ARHGEF40**	0.23	5.93E-01	1.00E+00	0.53	3.86E-01	8.70E-01
**XLOC_008667**	0.34	7.81E-01	1.00E+00	-0.90	3.86E-01	8.70E-01
**TOX4P1**	-1.60	6.43E-01	1.00E+00	-1.05	3.86E-01	8.70E-01
**GLTP**	0.14	7.31E-01	1.00E+00	-0.35	3.86E-01	8.70E-01
**MCC**	-0.31	3.50E-01	1.00E+00	-0.22	3.86E-01	8.70E-01
**FHDC1**	-0.19	7.10E-01	1.00E+00	0.54	3.86E-01	8.70E-01
**G0S2**	4.01	5.87E-03	4.57E-01	-0.69	3.86E-01	8.70E-01
**KIAA1324**	0.05	9.48E-01	1.00E+00	-0.59	3.86E-01	8.70E-01
**MFSD4A**	0.05	9.29E-01	1.00E+00	-0.42	3.86E-01	8.70E-01
**GNL3L**	-0.07	7.85E-01	1.00E+00	-0.34	3.86E-01	8.70E-01
**AJM1**	-1.01	2.24E-01	1.00E+00	0.34	3.86E-01	8.70E-01
**OGFR**	-0.11	6.98E-01	1.00E+00	0.83	3.86E-01	8.70E-01
**TMEM71**	0.67	6.29E-02	1.00E+00	0.44	3.86E-01	8.70E-01
**SLC26A1**	0.34	6.03E-01	1.00E+00	0.90	3.86E-01	8.70E-01
**BVES**	0.47	3.87E-01	1.00E+00	0.53	3.86E-01	8.70E-01
**UQCR10**	0.07	6.77E-01	1.00E+00	-0.36	3.86E-01	8.70E-01

**HSPA4**	-0.03	8.61E-01	1.00E+00	0.22	3.86E-01	8.70E-01
**BX284668.4**	-2.53	6.40E-02	1.00E+00	-0.69	3.86E-01	8.70E-01
**CCDC81**	-2.76	3.03E-02	1.00E+00	0.82	3.86E-01	8.70E-01
**SLC7A5**	-0.76	1.14E-01	1.00E+00	0.62	3.86E-01	8.70E-01
**PGAM1P8**	0.59	4.65E-01	1.00E+00	0.88	3.86E-01	8.70E-01
**DNALI1**	0.74	1.77E-01	1.00E+00	0.37	3.87E-01	8.70E-01
**AARSD1**	0.21	8.79E-01	1.00E+00	0.65	3.87E-01	8.70E-01
**DNAJB4**	0.35	2.71E-01	1.00E+00	-0.26	3.87E-01	8.70E-01
**SPRR2G**	-0.82	2.86E-01	1.00E+00	2.71	3.87E-01	8.70E-01
**ADRA2B**	-0.32	6.31E-01	1.00E+00	-0.97	3.87E-01	8.70E-01
**MSMB**	0.03	9.67E-01	1.00E+00	-0.82	3.87E-01	8.70E-01
**VPS16**	-0.19	3.70E-01	1.00E+00	0.19	3.87E-01	8.70E-01
**VPS45**	-0.05	7.91E-01	1.00E+00	-0.16	3.87E-01	8.70E-01
**LINC01909**	-0.27	9.16E-01	1.00E+00	1.00	3.87E-01	8.70E-01
**PARD3-AS1**	0.96	2.47E-01	1.00E+00	-0.70	3.87E-01	8.70E-01
**ADAMTS3**	-0.41	3.84E-01	1.00E+00	0.79	3.87E-01	8.70E-01
**AL122035.2**	-1.06	2.37E-01	1.00E+00	0.82	3.87E-01	8.70E-01
**AC108463.1**	1.67	3.71E-01	1.00E+00	1.14	3.87E-01	8.70E-01
**TMEM234**	0.53	1.50E-01	1.00E+00	0.29	3.87E-01	8.71E-01
**AC105446.1**	NA	NA	NA	-0.78	3.87E-01	8.71E-01
**C2orf73**	2.84	2.39E-02	9.18E-01	1.16	3.87E-01	8.71E-01
**CEBPA-DT**	-0.35	7.51E-01	1.00E+00	-0.52	3.87E-01	8.71E-01
**CFAP70**	-1.28	2.23E-03	2.39E-01	-0.62	3.87E-01	8.71E-01
**IZUMO4**	-0.36	4.91E-01	1.00E+00	-0.41	3.88E-01	8.71E-01
**NR2C2**	0.20	5.41E-01	1.00E+00	0.31	3.88E-01	8.71E-01
**LINC00578**	-1.99	7.75E-02	1.00E+00	-0.55	3.88E-01	8.71E-01
**ADRA2A**	0.32	4.45E-01	1.00E+00	0.68	3.88E-01	8.71E-01
**ANO7L1**	0.15	7.47E-01	1.00E+00	0.29	3.88E-01	8.71E-01
**DHRS4L2**	-0.34	2.50E-01	1.00E+00	-0.19	3.88E-01	8.71E-01
**LMO3**	0.52	6.18E-01	1.00E+00	-0.82	3.88E-01	8.71E-01
**ANO2**	0.39	3.26E-01	1.00E+00	0.89	3.88E-01	8.71E-01
**ZNF853**	0.03	9.43E-01	1.00E+00	0.66	3.88E-01	8.71E-01

**AL121761.1**	0.03	9.72E-01	1.00E+00	-0.61	3.88E-01	8.71E-01
**ERAP1**	-0.22	4.45E-01	1.00E+00	0.24	3.88E-01	8.71E-01
**TRIM68**	0.16	5.10E-01	1.00E+00	-0.37	3.88E-01	8.71E-01
**GRK6**	0.01	9.77E-01	1.00E+00	0.43	3.88E-01	8.71E-01
**URGCP**	-0.01	9.70E-01	1.00E+00	0.22	3.88E-01	8.71E-01
**IGLV3-21**	7.59	5.71E-15	4.43E-12	2.71	3.88E-01	8.71E-01
**UBQLN2**	-0.21	2.22E-01	1.00E+00	-0.18	3.88E-01	8.71E-01
**XLOC_000263**	-2.47	4.70E-01	1.00E+00	1.40	3.88E-01	8.71E-01
**LILRB1**	-0.60	3.83E-01	1.00E+00	2.70	3.88E-01	8.71E-01
**RN7SL4P**	-1.35	6.55E-01	1.00E+00	-1.00	3.88E-01	8.71E-01
**PCSK9**	-1.14	9.25E-02	1.00E+00	-0.80	3.88E-01	8.71E-01
**LILRA2**	-1.66	7.36E-02	1.00E+00	2.17	3.88E-01	8.71E-01
**RHAG**	-2.24	9.13E-02	1.00E+00	1.38	3.88E-01	8.71E-01
**MT-ND3**	0.07	8.81E-01	1.00E+00	-0.34	3.89E-01	8.71E-01
**THBS3**	0.11	8.12E-01	1.00E+00	0.46	3.89E-01	8.71E-01
**MMP1**	0.06	9.74E-01	1.00E+00	2.71	3.89E-01	8.71E-01
**TMC5**	0.55	2.36E-01	1.00E+00	-0.44	3.89E-01	8.71E-01
**AL357515.1**	-0.25	9.07E-01	1.00E+00	0.76	3.89E-01	8.71E-01
**BTBD1**	-0.11	5.91E-01	1.00E+00	-0.56	3.89E-01	8.71E-01
**ZNF83**	-0.03	9.07E-01	1.00E+00	-0.21	3.89E-01	8.71E-01
**BCL6B**	0.16	7.47E-01	1.00E+00	0.49	3.89E-01	8.71E-01
**AC079062.1**	-10.73	1.72E-03	2.29E-01	2.01	3.89E-01	8.71E-01
**ZNF160**	0.06	8.12E-01	1.00E+00	-0.19	3.89E-01	8.71E-01
**MIR3648**	-0.03	9.59E-01	1.00E+00	-1.31	3.89E-01	8.71E-01
**RBPMS2**	-0.12	8.61E-01	1.00E+00	-0.51	3.89E-01	8.72E-01
**MPPED1**	-0.89	3.87E-01	1.00E+00	-0.74	3.89E-01	8.72E-01
**SPDYE2**	-1.08	4.23E-01	1.00E+00	0.47	3.89E-01	8.72E-01
**CPA2**	7.37	1.02E-02	6.27E-01	1.10	3.89E-01	8.72E-01
**OTUD6B**	-0.01	9.72E-01	1.00E+00	-0.21	3.89E-01	8.72E-01
**ZNF619**	0.27	4.36E-01	1.00E+00	0.33	3.89E-01	8.72E-01
**TRPV1**	0.27	6.45E-01	1.00E+00	0.76	3.89E-01	8.72E-01
**FAM222B**	-0.39	4.55E-02	1.00E+00	0.25	3.89E-01	8.72E-01

**PRUNE2**	-0.25	6.85E-01	1.00E+00	0.40	3.89E-01	8.72E-01
**G16904**	0.43	6.40E-01	1.00E+00	1.16	3.89E-01	8.72E-01
**TOMM70**	0.04	7.73E-01	1.00E+00	-0.31	3.90E-01	8.72E-01
**MYOSLID**	-0.90	6.32E-01	1.00E+00	0.64	3.90E-01	8.72E-01
**C10orf105**	1.49	1.59E-01	1.00E+00	1.03	3.90E-01	8.72E-01
**TUBB6**	-0.09	6.55E-01	1.00E+00	0.25	3.90E-01	8.72E-01
**AC004024.1**	-0.70	8.10E-01	1.00E+00	-0.77	3.90E-01	8.72E-01
**BMS1P8**	3.94	3.21E-02	1.00E+00	0.51	3.90E-01	8.72E-01
**ECM2**	0.94	9.48E-03	6.06E-01	0.42	3.90E-01	8.72E-01
**TRAPPC2B**	0.49	1.29E-01	1.00E+00	-0.45	3.90E-01	8.72E-01
**CROCCP3**	-0.65	2.55E-01	1.00E+00	0.56	3.90E-01	8.72E-01
**ELOA-AS1**	-1.56	1.95E-01	1.00E+00	-0.52	3.90E-01	8.72E-01
**GNB2**	-0.12	6.42E-01	1.00E+00	0.47	3.90E-01	8.72E-01
**PREB**	0.22	4.04E-01	1.00E+00	-0.31	3.90E-01	8.72E-01
**ST8SIA5**	-5.15	6.66E-04	1.23E-01	-1.09	3.90E-01	8.72E-01
**C6orf48**	-0.10	7.72E-01	1.00E+00	0.53	3.90E-01	8.72E-01
**SLC31A2**	0.05	9.36E-01	1.00E+00	0.55	3.90E-01	8.72E-01
**FAM169A**	-1.25	6.27E-02	1.00E+00	0.91	3.90E-01	8.72E-01
**FARS2**	-0.27	3.29E-01	1.00E+00	0.13	3.90E-01	8.72E-01
**C2CD6**	-1.17	3.75E-01	1.00E+00	0.76	3.90E-01	8.72E-01
**ATPAF2**	0.02	9.44E-01	1.00E+00	0.15	3.90E-01	8.72E-01
**ZFYVE28**	-0.54	1.78E-01	1.00E+00	0.56	3.90E-01	8.72E-01
**G113**	0.22	8.54E-01	1.00E+00	-0.86	3.90E-01	8.72E-01
**DNAJC16**	0.18	5.61E-01	1.00E+00	0.24	3.90E-01	8.72E-01
**FCMR**	-0.30	4.86E-01	1.00E+00	0.38	3.91E-01	8.72E-01
**AC244153.1**	0.59	2.06E-01	1.00E+00	0.38	3.91E-01	8.73E-01
**LINC02352**	1.97	6.35E-02	1.00E+00	-0.68	3.91E-01	8.73E-01
**GP9**	1.62	3.65E-01	1.00E+00	-1.36	3.91E-01	8.73E-01
**ZSCAN25**	-0.24	2.75E-01	1.00E+00	-0.18	3.91E-01	8.73E-01
**BRINP2**	-0.25	8.56E-01	1.00E+00	1.04	3.91E-01	8.73E-01
**TTC31**	-0.04	8.18E-01	1.00E+00	0.11	3.91E-01	8.73E-01
**MYL5**	0.36	1.94E-01	1.00E+00	0.26	3.91E-01	8.73E-01

**TRAK1**	0.00	9.88E-01	1.00E+00	0.25	3.91E-01	8.73E-01
**ZNF449**	0.29	6.90E-01	1.00E+00	-0.50	3.91E-01	8.73E-01
**SMARCC2**	-0.23	2.17E-01	1.00E+00	-0.17	3.91E-01	8.73E-01
**ZNF561-AS1**	-0.17	5.38E-01	1.00E+00	-0.32	3.91E-01	8.73E-01
**KRT19**	-0.27	8.01E-01	1.00E+00	-0.58	3.91E-01	8.73E-01
**AC104123.1**	-0.35	7.25E-01	1.00E+00	-0.67	3.91E-01	8.73E-01
**SLC39A10**	-0.22	3.67E-01	1.00E+00	-0.30	3.91E-01	8.73E-01
**ZNF839**	-0.25	3.48E-01	1.00E+00	-0.55	3.91E-01	8.73E-01
**INKA1**	0.06	8.28E-01	1.00E+00	-0.33	3.91E-01	8.73E-01
**AC009237.15**	0.57	6.00E-01	1.00E+00	-1.46	3.91E-01	8.73E-01
**BATF2**	-0.59	4.68E-01	1.00E+00	0.85	3.91E-01	8.73E-01
**RYBP**	-0.02	9.11E-01	1.00E+00	0.31	3.91E-01	8.73E-01
**LBX2**	0.50	6.91E-01	1.00E+00	0.90	3.91E-01	8.73E-01
**FABP3**	-0.41	5.02E-01	1.00E+00	-0.57	3.91E-01	8.73E-01
**NPIPB2**	-0.33	7.13E-01	1.00E+00	0.55	3.91E-01	8.73E-01
**LINC01301**	0.17	8.55E-01	1.00E+00	-0.61	3.91E-01	8.73E-01
**PRDM10**	-0.17	5.17E-01	1.00E+00	-0.26	3.92E-01	8.73E-01
**SLC2A3**	-0.87	1.68E-01	1.00E+00	0.58	3.92E-01	8.73E-01
**VPS13A**	-0.23	3.75E-01	1.00E+00	-0.23	3.92E-01	8.73E-01
**RASGEF1B**	-0.10	7.71E-01	1.00E+00	0.28	3.92E-01	8.73E-01
**XLOC_006263**	-0.29	7.23E-01	1.00E+00	0.37	3.92E-01	8.73E-01
**C2orf68**	-0.10	6.77E-01	1.00E+00	0.22	3.92E-01	8.73E-01
**FAM21FP**	-0.33	6.38E-01	1.00E+00	0.50	3.92E-01	8.73E-01
**CLDN3**	0.44	5.31E-01	1.00E+00	-0.71	3.92E-01	8.73E-01
**BCAR1**	-0.31	2.91E-01	1.00E+00	0.55	3.92E-01	8.73E-01
**WDR54**	-0.25	4.04E-01	1.00E+00	0.21	3.92E-01	8.73E-01
**IFI27L1**	-0.07	8.34E-01	1.00E+00	0.18	3.92E-01	8.73E-01
**HIPK1**	0.06	7.87E-01	1.00E+00	0.25	3.92E-01	8.73E-01
**SDR42E1**	0.05	8.79E-01	1.00E+00	0.34	3.92E-01	8.73E-01
**VCPKMT**	0.08	8.40E-01	1.00E+00	-0.30	3.92E-01	8.73E-01
**TRAV17**	-1.31	4.62E-01	1.00E+00	0.96	3.92E-01	8.73E-01
**XLOC_004419**	-1.28	1.10E-01	1.00E+00	0.80	3.93E-01	8.73E-01

**GBP4**	-0.59	3.16E-01	1.00E+00	0.58	3.93E-01	8.73E-01
**AC005863.1**	-0.25	9.41E-01	1.00E+00	1.36	3.93E-01	8.73E-01
**ZNF347**	0.16	6.73E-01	1.00E+00	-0.22	3.93E-01	8.73E-01
**AC010300.1**	1.55	2.85E-01	1.00E+00	-0.75	3.93E-01	8.73E-01
**RP11-417J8.3**	1.06	6.16E-01	1.00E+00	-0.79	3.93E-01	8.73E-01
**XLOC_009548**	4.23	1.99E-01	1.00E+00	-2.71	3.93E-01	8.73E-01
**GAN**	0.66	1.11E-01	1.00E+00	-0.50	3.93E-01	8.73E-01
**CD160**	0.52	6.46E-01	1.00E+00	0.87	3.93E-01	8.73E-01
**MMP23B**	-0.12	8.83E-01	1.00E+00	1.02	3.93E-01	8.73E-01
**FUT1**	-0.17	6.43E-01	1.00E+00	0.26	3.93E-01	8.73E-01
**NPBWR1**	0.22	7.31E-01	1.00E+00	-0.66	3.93E-01	8.73E-01
**PHLPP2**	0.42	2.89E-01	1.00E+00	0.55	3.93E-01	8.73E-01
**STPG2**	-0.50	7.44E-01	1.00E+00	-0.88	3.93E-01	8.73E-01
**SULT1E1**	-1.20	1.41E-01	1.00E+00	-0.74	3.93E-01	8.73E-01
**MYH7B**	-0.43	5.11E-01	1.00E+00	0.85	3.93E-01	8.73E-01
**RREB1**	-0.49	1.25E-01	1.00E+00	0.35	3.93E-01	8.73E-01
**GPR82**	-0.73	4.92E-01	1.00E+00	0.53	3.93E-01	8.73E-01
**PFKFB1**	3.34	8.10E-04	1.42E-01	-0.87	3.93E-01	8.73E-01
**PHLDB1**	0.44	3.51E-01	1.00E+00	0.50	3.93E-01	8.73E-01
**PLEKHA5**	0.03	9.13E-01	1.00E+00	-0.23	3.93E-01	8.73E-01
**TRIM13**	0.24	2.13E-01	1.00E+00	-0.19	3.93E-01	8.73E-01
**FAM43A**	-0.48	2.34E-01	1.00E+00	0.59	3.93E-01	8.73E-01
**SP2**	-0.28	3.84E-01	1.00E+00	0.30	3.93E-01	8.73E-01
**HSPB8**	0.10	7.25E-01	1.00E+00	0.22	3.93E-01	8.73E-01
**PEX5**	0.12	4.21E-01	1.00E+00	0.19	3.93E-01	8.73E-01
**CD1A**	-0.67	2.04E-01	1.00E+00	-0.70	3.93E-01	8.73E-01
**HIVEP1**	-0.46	6.02E-02	1.00E+00	0.34	3.93E-01	8.73E-01
**LINC02166**	2.29	2.16E-01	1.00E+00	-0.90	3.93E-01	8.73E-01
**HM13-IT1**	0.77	5.98E-01	1.00E+00	1.09	3.93E-01	8.73E-01
**SENP5**	0.13	4.64E-01	1.00E+00	0.21	3.93E-01	8.73E-01
**AL031772.1**	-2.62	1.20E-01	1.00E+00	0.73	3.93E-01	8.73E-01
**NASP**	-0.05	8.14E-01	1.00E+00	0.27	3.93E-01	8.73E-01

**HIST1H2BH**	-0.62	3.79E-01	1.00E+00	0.77	3.94E-01	8.73E-01
**GOLGA3**	-0.27	2.92E-01	1.00E+00	0.29	3.94E-01	8.73E-01
**CSPG5**	-0.47	3.38E-01	1.00E+00	-0.69	3.94E-01	8.73E-01
**AC007622.2**	1.52	6.56E-01	1.00E+00	-0.92	3.94E-01	8.73E-01
**SLURP2**	1.29	3.99E-01	1.00E+00	0.79	3.94E-01	8.73E-01
**EGLN3**	-0.10	8.00E-01	1.00E+00	-0.38	3.94E-01	8.73E-01
**NEURL2**	-1.96	3.21E-01	1.00E+00	-0.61	3.94E-01	8.73E-01
**H2AFX**	-0.32	3.39E-01	1.00E+00	0.50	3.94E-01	8.73E-01
**ZNF860**	-0.01	9.77E-01	1.00E+00	0.48	3.94E-01	8.73E-01
**RCBTB1**	0.23	4.09E-01	1.00E+00	-0.32	3.94E-01	8.73E-01
**CT62**	-1.04	5.62E-01	1.00E+00	-1.32	3.94E-01	8.73E-01
**OSBPL6**	0.08	8.54E-01	1.00E+00	-0.39	3.94E-01	8.73E-01
**ZNF28**	0.23	4.86E-01	1.00E+00	-0.25	3.94E-01	8.73E-01
**AL359513.1**	-0.84	1.54E-01	1.00E+00	-0.62	3.94E-01	8.73E-01
**AC009065.4**	0.34	6.82E-01	1.00E+00	-0.47	3.94E-01	8.73E-01
**TCF19**	-0.34	6.29E-02	1.00E+00	0.26	3.94E-01	8.73E-01
**FAM135B**	-1.46	1.06E-01	1.00E+00	-1.00	3.94E-01	8.73E-01
**HOMER3**	-0.49	2.16E-01	1.00E+00	0.30	3.94E-01	8.73E-01
**SPATA1**	-1.21	3.12E-01	1.00E+00	-0.47	3.94E-01	8.73E-01
**SPRR2F**	-3.24	3.03E-01	1.00E+00	2.68	3.94E-01	8.73E-01
**TSEN15**	-0.22	3.17E-01	1.00E+00	-0.21	3.94E-01	8.73E-01
**DNAJC9**	-0.29	2.35E-01	1.00E+00	0.23	3.94E-01	8.73E-01
**ZKSCAN8**	0.04	8.72E-01	1.00E+00	0.35	3.94E-01	8.73E-01
**DCUN1D2**	0.14	5.02E-01	1.00E+00	-0.26	3.94E-01	8.73E-01
**PIPSL**	-1.16	4.23E-01	1.00E+00	-0.53	3.94E-01	8.73E-01
**PRR19**	-1.18	1.53E-01	1.00E+00	0.39	3.94E-01	8.73E-01
**LDAH**	0.00	9.97E-01	1.00E+00	-0.17	3.95E-01	8.74E-01
**C1D**	0.03	9.04E-01	1.00E+00	-0.29	3.95E-01	8.74E-01
**C18orf65**	0.15	8.84E-01	1.00E+00	0.84	3.95E-01	8.74E-01
**PYY2**	-3.04	3.67E-01	1.00E+00	-0.98	3.95E-01	8.74E-01
**GUCY1A1**	-0.39	4.43E-01	1.00E+00	0.33	3.95E-01	8.74E-01
**CLCNKA**	2.22	1.24E-01	1.00E+00	0.94	3.95E-01	8.74E-01

**RYR3**	-0.77	9.95E-02	1.00E+00	0.51	3.95E-01	8.74E-01
**SMPD3**	-0.14	8.45E-01	1.00E+00	0.58	3.95E-01	8.74E-01
**AC020637.1**	-1.27	7.14E-01	1.00E+00	-1.70	3.95E-01	8.74E-01
**SLC47A2**	-0.31	7.10E-01	1.00E+00	0.66	3.95E-01	8.74E-01
**TNFRSF10A-AS1**	-1.56	1.27E-01	1.00E+00	-0.49	3.95E-01	8.74E-01
**SH3GL1P1**	-0.41	6.53E-01	1.00E+00	0.80	3.95E-01	8.74E-01
**TMC4**	0.05	9.37E-01	1.00E+00	-0.59	3.95E-01	8.74E-01
**AC010536.1**	0.09	9.61E-01	1.00E+00	1.09	3.95E-01	8.74E-01
**SLC22A20P**	-0.78	3.62E-01	1.00E+00	1.09	3.95E-01	8.74E-01
**UTP3**	-0.17	3.55E-01	1.00E+00	0.21	3.95E-01	8.74E-01
**CCDC60**	-1.85	1.80E-01	1.00E+00	0.95	3.95E-01	8.74E-01
**KRCC1**	0.37	1.99E-01	1.00E+00	-0.29	3.95E-01	8.74E-01
**TRMT10B**	-0.08	7.68E-01	1.00E+00	-0.15	3.95E-01	8.74E-01
**DNAJC13**	0.18	4.49E-01	1.00E+00	0.33	3.95E-01	8.74E-01
**AL356740.1**	1.05	3.79E-01	1.00E+00	-1.15	3.95E-01	8.74E-01
**MRPS36P1**	NA	NA	NA	-0.62	3.95E-01	8.74E-01
**METTL9**	-0.03	8.88E-01	1.00E+00	-0.35	3.95E-01	8.74E-01
**NDUFAF2**	-0.29	1.86E-01	1.00E+00	-0.17	3.95E-01	8.74E-01
**CLPB**	-0.58	7.05E-03	5.12E-01	0.22	3.95E-01	8.74E-01
**C6orf223**	0.62	6.71E-01	1.00E+00	1.32	3.96E-01	8.74E-01
**AL445189.2**	0.79	8.21E-01	1.00E+00	0.84	3.96E-01	8.74E-01
**AC004231.1**	-0.27	6.08E-01	1.00E+00	-0.58	3.96E-01	8.74E-01
**XLOC_013960**	-0.19	7.71E-01	1.00E+00	0.94	3.96E-01	8.74E-01
**AC107375.1**	-0.18	6.59E-01	1.00E+00	0.60	3.96E-01	8.74E-01
**AL162414.1**	-1.60	6.44E-01	1.00E+00	1.49	3.96E-01	8.74E-01
**MYO5A**	-0.26	1.39E-01	1.00E+00	0.29	3.96E-01	8.74E-01
**G34929**	-0.12	8.94E-01	1.00E+00	0.94	3.96E-01	8.74E-01
**HIPK1-AS1**	-0.27	8.33E-01	1.00E+00	0.95	3.96E-01	8.74E-01
**LINC00900**	0.66	2.10E-01	1.00E+00	-0.46	3.96E-01	8.74E-01
**MAU2**	-0.08	7.31E-01	1.00E+00	0.33	3.96E-01	8.74E-01
**AFG3L1P**	-0.38	3.65E-01	1.00E+00	0.36	3.96E-01	8.74E-01
**TPT1-AS1**	0.26	4.47E-01	1.00E+00	0.26	3.96E-01	8.74E-01

**EIF3F**	-0.06	8.19E-01	1.00E+00	0.18	3.96E-01	8.74E-01
**PGM3**	-0.07	6.95E-01	1.00E+00	-0.25	3.96E-01	8.74E-01
**SECTM1**	-0.43	1.64E-01	1.00E+00	0.86	3.96E-01	8.74E-01
**MELTF**	0.02	9.83E-01	1.00E+00	-0.61	3.96E-01	8.74E-01
**DARS-AS1**	-0.20	8.25E-01	1.00E+00	-0.30	3.96E-01	8.74E-01
**MOB1B**	-0.13	6.22E-01	1.00E+00	-0.28	3.96E-01	8.74E-01
**XLOC_001781**	0.56	8.71E-01	1.00E+00	1.25	3.96E-01	8.74E-01
**LINC02031**	-0.90	4.82E-01	1.00E+00	-0.90	3.96E-01	8.74E-01
**MMP27**	0.68	1.68E-01	1.00E+00	0.50	3.96E-01	8.74E-01
**HSD17B2**	-0.20	8.43E-01	1.00E+00	0.64	3.96E-01	8.74E-01
**ARC**	-2.83	2.24E-02	9.00E-01	-0.86	3.96E-01	8.74E-01
**ATAD3B**	-0.47	3.20E-01	1.00E+00	0.53	3.96E-01	8.74E-01
**ADRB3**	5.22	2.81E-02	9.75E-01	1.36	3.96E-01	8.74E-01
**RPL21P75**	0.28	7.23E-01	1.00E+00	-0.58	3.96E-01	8.74E-01
**DNAJB6**	0.09	7.65E-01	1.00E+00	-0.19	3.96E-01	8.74E-01
**GSTT2B**	0.64	5.01E-01	1.00E+00	0.63	3.96E-01	8.74E-01
**DCAF6**	0.05	7.94E-01	1.00E+00	-0.19	3.97E-01	8.74E-01
**ALG2**	-0.06	7.02E-01	1.00E+00	0.13	3.97E-01	8.74E-01
**G30323**	-1.21	1.24E-01	1.00E+00	0.76	3.97E-01	8.74E-01
**THADA**	0.01	9.61E-01	1.00E+00	-0.20	3.97E-01	8.74E-01
**MSX1**	0.19	7.08E-01	1.00E+00	0.63	3.97E-01	8.74E-01
**ERBIN**	0.11	6.77E-01	1.00E+00	-0.20	3.97E-01	8.74E-01
**ZNF761**	0.05	8.88E-01	1.00E+00	-0.28	3.97E-01	8.74E-01
**AC072062.1**	0.23	8.28E-01	1.00E+00	0.63	3.97E-01	8.74E-01
**APAF1**	0.44	3.68E-01	1.00E+00	0.52	3.97E-01	8.74E-01
**ARF4**	-0.01	9.56E-01	1.00E+00	-0.24	3.97E-01	8.75E-01
**INHBA-AS1**	-1.74	4.21E-01	1.00E+00	-1.07	3.97E-01	8.75E-01
**TRIM2**	0.05	9.08E-01	1.00E+00	-0.34	3.97E-01	8.75E-01
**DDX49**	-0.21	4.50E-01	1.00E+00	0.20	3.97E-01	8.75E-01
**SLIT3**	0.40	3.57E-01	1.00E+00	0.53	3.98E-01	8.75E-01
**SLC39A4**	-0.60	1.46E-01	1.00E+00	-0.34	3.98E-01	8.75E-01
**TPGS2**	0.05	8.12E-01	1.00E+00	-0.19	3.98E-01	8.75E-01

**CWH43**	-0.21	6.28E-01	1.00E+00	-0.63	3.98E-01	8.75E-01
**NRN1**	0.25	4.88E-01	1.00E+00	-0.37	3.98E-01	8.75E-01
**AJ003147.3**	2.01	3.92E-01	1.00E+00	1.27	3.98E-01	8.75E-01
**AC011389.1**	-1.30	6.21E-01	1.00E+00	0.92	3.98E-01	8.75E-01
**WDYHV1**	0.02	9.17E-01	1.00E+00	0.20	3.98E-01	8.75E-01
**ISM1**	0.19	7.05E-01	1.00E+00	0.50	3.98E-01	8.75E-01
**ZNF627**	0.22	3.56E-01	1.00E+00	0.21	3.98E-01	8.76E-01
**RP11-312O7.2**	-0.54	2.11E-01	1.00E+00	0.53	3.98E-01	8.76E-01
**AMHR2**	1.75	1.81E-01	1.00E+00	-0.99	3.98E-01	8.76E-01
**DCTN5**	-0.07	6.85E-01	1.00E+00	0.14	3.98E-01	8.76E-01
**G39812**	0.66	4.17E-01	1.00E+00	0.66	3.98E-01	8.76E-01
**DEPDC4**	0.13	8.79E-01	1.00E+00	-0.61	3.98E-01	8.76E-01
**LRRC3**	0.09	7.49E-01	1.00E+00	0.74	3.98E-01	8.76E-01
**AC084125.2**	0.95	5.85E-01	1.00E+00	0.93	3.99E-01	8.76E-01
**FAM111B**	-0.11	7.24E-01	1.00E+00	0.52	3.99E-01	8.76E-01
**HLA-U**	-2.59	2.02E-01	1.00E+00	-1.38	3.99E-01	8.76E-01
**SEC24D**	-0.12	6.29E-01	1.00E+00	0.41	3.99E-01	8.76E-01
**TBC1D22B**	0.15	6.07E-01	1.00E+00	0.27	3.99E-01	8.76E-01
**AL353743.1**	-0.02	9.69E-01	1.00E+00	-0.61	3.99E-01	8.76E-01
**HOXC13**	-0.19	5.69E-01	1.00E+00	-0.53	3.99E-01	8.76E-01
**HIST1H3C**	-1.05	6.58E-01	1.00E+00	-1.15	3.99E-01	8.76E-01
**ACBD7**	-0.13	9.27E-01	1.00E+00	0.94	3.99E-01	8.76E-01
**PGAM5**	-0.32	1.36E-01	1.00E+00	-0.42	3.99E-01	8.76E-01
**ZAP70**	0.18	7.42E-01	1.00E+00	2.09	3.99E-01	8.76E-01
**GFPT1**	0.30	4.74E-01	1.00E+00	-0.34	3.99E-01	8.76E-01
**AC090589.1**	-0.25	9.41E-01	1.00E+00	0.54	3.99E-01	8.76E-01
**PMM2**	-0.29	3.39E-01	1.00E+00	0.20	3.99E-01	8.76E-01
**ATG7**	-0.02	9.34E-01	1.00E+00	0.12	3.99E-01	8.76E-01
**RASSF8**	0.04	9.20E-01	1.00E+00	0.43	3.99E-01	8.76E-01
**AL513477.2**	-0.92	7.64E-01	1.00E+00	-0.67	3.99E-01	8.76E-01
**KCNJ11**	0.49	2.55E-01	1.00E+00	-0.76	3.99E-01	8.76E-01
**ZNF708**	0.26	5.99E-01	1.00E+00	-0.30	3.99E-01	8.76E-01

**TTC30A**	0.18	5.98E-01	1.00E+00	-0.36	3.99E-01	8.76E-01
**ZRANB3**	-0.79	6.88E-03	5.06E-01	-0.35	3.99E-01	8.76E-01
**HGC6.3**	-1.86	4.68E-01	1.00E+00	1.62	3.99E-01	8.76E-01
**REV1**	-0.23	3.94E-01	1.00E+00	-0.30	3.99E-01	8.76E-01
**CRK**	0.29	1.41E-01	1.00E+00	-0.26	3.99E-01	8.76E-01
**C7**	0.60	4.08E-01	1.00E+00	0.59	3.99E-01	8.76E-01
**AC097460.1**	0.55	6.77E-01	1.00E+00	-0.97	4.00E-01	8.76E-01
**DNAJC17**	0.35	1.59E-01	1.00E+00	0.17	4.00E-01	8.76E-01
**TBX19**	0.00	9.96E-01	1.00E+00	0.39	4.00E-01	8.76E-01
**NEDD8**	0.17	4.56E-01	1.00E+00	-0.45	4.00E-01	8.76E-01
**XLOC_001726**	-1.96	3.48E-02	1.00E+00	0.76	4.00E-01	8.76E-01
**PIK3CD-AS1**	1.10	7.48E-01	1.00E+00	1.16	4.00E-01	8.76E-01
**TANGO2**	0.00	9.95E-01	1.00E+00	0.22	4.00E-01	8.76E-01
**CYP51A1**	-0.16	6.25E-01	1.00E+00	0.38	4.00E-01	8.76E-01
**RUNDC3B**	-0.69	3.74E-02	1.00E+00	-0.32	4.00E-01	8.76E-01
**CALML3**	-0.77	8.24E-02	1.00E+00	0.61	4.00E-01	8.76E-01
**AC009404.1**	-0.05	9.21E-01	1.00E+00	0.65	4.00E-01	8.76E-01
**ZNF564**	0.95	6.42E-01	1.00E+00	0.58	4.00E-01	8.76E-01
**RNF141**	0.18	4.90E-01	1.00E+00	-0.28	4.00E-01	8.76E-01
**XLOC_010336**	0.26	5.83E-01	1.00E+00	0.60	4.00E-01	8.76E-01
**C19orf53**	-0.03	8.74E-01	1.00E+00	0.17	4.00E-01	8.76E-01
**GUSBP1**	0.38	3.28E-01	1.00E+00	-0.35	4.00E-01	8.76E-01
**IQCJ-SCHIP1**	0.08	8.79E-01	1.00E+00	0.84	4.00E-01	8.76E-01
**AL590787.1**	-1.67	3.90E-01	1.00E+00	1.56	4.00E-01	8.76E-01
**CCPG1**	0.49	2.87E-01	1.00E+00	-0.24	4.00E-01	8.76E-01
**PLK3**	-0.42	4.47E-01	1.00E+00	0.33	4.00E-01	8.76E-01
**NMT1**	0.21	2.62E-01	1.00E+00	0.35	4.00E-01	8.76E-01
**B3GNT5**	-0.47	4.27E-01	1.00E+00	0.25	4.00E-01	8.76E-01
**BACH1-IT1**	-0.70	5.77E-01	1.00E+00	0.76	4.01E-01	8.76E-01
**CACNB4**	-0.18	7.99E-01	1.00E+00	0.40	4.01E-01	8.76E-01
**ZNF382**	0.79	3.50E-01	1.00E+00	0.49	4.01E-01	8.76E-01
**G1047**	-1.25	4.67E-02	1.00E+00	0.78	4.01E-01	8.76E-01

**AC092807.3**	-1.20	1.55E-01	1.00E+00	-0.48	4.01E-01	8.76E-01
**MST1**	-0.94	2.96E-02	9.96E-01	0.59	4.01E-01	8.76E-01
**CCDC106**	0.11	7.54E-01	1.00E+00	0.36	4.01E-01	8.76E-01
**FTH1**	0.24	5.49E-01	1.00E+00	0.48	4.01E-01	8.76E-01
**RAP1GAP2**	0.00	9.96E-01	1.00E+00	0.49	4.01E-01	8.76E-01
**ATG3**	0.06	7.78E-01	1.00E+00	-0.20	4.01E-01	8.76E-01
**AL359711.2**	0.12	8.29E-01	1.00E+00	-0.50	4.01E-01	8.76E-01
**NAALAD2**	0.84	2.06E-01	1.00E+00	-0.68	4.01E-01	8.76E-01
**SRP14-AS1**	-0.83	1.48E-01	1.00E+00	-0.48	4.01E-01	8.76E-01
**GSTA3**	0.40	3.89E-01	1.00E+00	-0.68	4.01E-01	8.76E-01
**ICA1**	0.06	8.81E-01	1.00E+00	-0.24	4.01E-01	8.76E-01
**TOE1**	0.07	8.23E-01	1.00E+00	0.22	4.01E-01	8.76E-01
**MPND**	-0.34	2.34E-01	1.00E+00	-0.44	4.01E-01	8.76E-01
**WDR27**	-0.26	5.44E-01	1.00E+00	0.27	4.01E-01	8.76E-01
**AL022341.1**	1.39	4.66E-01	1.00E+00	-0.86	4.01E-01	8.76E-01
**LRRC7**	-0.06	9.20E-01	1.00E+00	-0.62	4.01E-01	8.77E-01
**HADHA**	-0.04	8.61E-01	1.00E+00	-0.11	4.01E-01	8.77E-01
**TRMT9B**	0.23	4.76E-01	1.00E+00	-0.35	4.01E-01	8.77E-01
**FYCO1**	-0.16	5.42E-01	1.00E+00	0.31	4.01E-01	8.77E-01
**MAGEE1**	-0.39	1.53E-01	1.00E+00	0.36	4.01E-01	8.77E-01
**AC099494.2**	0.16	8.35E-01	1.00E+00	-0.81	4.01E-01	8.77E-01
**ALCAM**	-0.31	3.29E-02	1.00E+00	-0.22	4.01E-01	8.77E-01
**FAM3D**	-0.49	4.11E-01	1.00E+00	-0.64	4.01E-01	8.77E-01
**HHEX**	0.14	8.02E-01	1.00E+00	0.35	4.02E-01	8.77E-01
**RRS1**	-0.43	9.47E-02	1.00E+00	0.32	4.02E-01	8.77E-01
**AP002761.3**	0.72	3.52E-01	1.00E+00	0.95	4.02E-01	8.77E-01
**PTPRN2**	-0.25	6.87E-01	1.00E+00	0.41	4.02E-01	8.77E-01
**OR7E62P**	0.59	2.68E-01	1.00E+00	-0.55	4.02E-01	8.77E-01
**TRMT2A**	-0.03	9.11E-01	1.00E+00	0.28	4.02E-01	8.77E-01
**TTF2**	-0.11	7.06E-01	1.00E+00	0.24	4.02E-01	8.77E-01
**ADGRG4**	-1.87	3.71E-01	1.00E+00	1.65	4.02E-01	8.77E-01
**AC132872.1**	-0.31	5.06E-01	1.00E+00	0.54	4.02E-01	8.77E-01

**HMGN2P46**	-0.18	7.68E-01	1.00E+00	-0.46	4.02E-01	8.77E-01
**HSD3BP4**	-1.28	5.05E-01	1.00E+00	-0.84	4.02E-01	8.77E-01
**RNA5-8SP6**	-0.39	4.19E-01	1.00E+00	-0.57	4.02E-01	8.77E-01
**SPX**	4.69	1.34E-02	7.26E-01	-2.63	4.02E-01	8.77E-01
**TM9SF4**	-0.05	8.21E-01	1.00E+00	0.21	4.02E-01	8.77E-01
**RAB3B**	0.11	7.29E-01	1.00E+00	-0.45	4.02E-01	8.77E-01
**AL139099.3**	-1.37	6.93E-01	1.00E+00	-0.73	4.02E-01	8.77E-01
**RNPEP**	-0.38	2.33E-01	1.00E+00	-0.32	4.02E-01	8.77E-01
**TPRXL**	-0.64	2.66E-02	9.50E-01	0.47	4.02E-01	8.77E-01
**POM121C**	-0.26	1.42E-01	1.00E+00	0.48	4.02E-01	8.77E-01
**TRIM67**	1.09	3.28E-01	1.00E+00	1.09	4.02E-01	8.77E-01
**PHKA2-AS1**	-0.29	8.05E-01	1.00E+00	0.85	4.02E-01	8.77E-01
**SLC2A10**	0.41	4.30E-01	1.00E+00	0.34	4.02E-01	8.77E-01
**SH3GLB2**	0.02	9.53E-01	1.00E+00	-0.40	4.02E-01	8.77E-01
**PCDHGC4**	-1.18	8.35E-02	1.00E+00	1.12	4.02E-01	8.77E-01
**TMEM213**	1.19	1.57E-01	1.00E+00	-0.71	4.02E-01	8.77E-01
**MOB1A**	0.04	8.99E-01	1.00E+00	-0.18	4.02E-01	8.77E-01
**XLOC_012763**	-0.07	8.92E-01	1.00E+00	-0.76	4.03E-01	8.77E-01
**SFSWAP**	-0.21	3.85E-01	1.00E+00	0.30	4.03E-01	8.77E-01
**PSMD13**	0.03	8.47E-01	1.00E+00	0.11	4.03E-01	8.77E-01
**SLC7A5P1**	-2.79	3.04E-01	1.00E+00	1.16	4.03E-01	8.77E-01
**LARGE1**	-0.17	5.49E-01	1.00E+00	0.40	4.03E-01	8.77E-01
**ASIC3**	-0.93	4.03E-01	1.00E+00	1.05	4.03E-01	8.77E-01
**CNTD1**	1.10	2.52E-01	1.00E+00	-0.55	4.03E-01	8.77E-01
**C9orf106**	1.82	2.40E-01	1.00E+00	1.05	4.03E-01	8.77E-01
**SMARCE1P2**	0.51	6.51E-01	1.00E+00	0.96	4.03E-01	8.77E-01
**MINOS1P2**	NA	NA	NA	0.58	4.03E-01	8.77E-01
**CATSPERD**	-0.90	7.82E-01	1.00E+00	1.02	4.03E-01	8.77E-01
**DRG2**	-0.22	3.04E-01	1.00E+00	-0.12	4.03E-01	8.77E-01
**NAAA**	0.19	4.56E-01	1.00E+00	-0.15	4.03E-01	8.77E-01
**RPL36AP21**	NA	NA	NA	-0.66	4.03E-01	8.77E-01
**NIFK**	0.25	2.79E-01	1.00E+00	-0.28	4.03E-01	8.77E-01

**ST8SIA1**	-0.10	6.72E-01	1.00E+00	0.34	4.03E-01	8.77E-01
**IQCG**	-0.06	8.09E-01	1.00E+00	-0.19	4.03E-01	8.77E-01
**AL359962.2**	-2.05	7.16E-02	1.00E+00	0.58	4.03E-01	8.77E-01
**IL21**	-1.26	7.16E-01	1.00E+00	-2.20	4.03E-01	8.77E-01
**KIRREL1-IT1**	-1.10	5.55E-01	1.00E+00	0.82	4.03E-01	8.77E-01
**KCNV2**	-0.06	9.86E-01	1.00E+00	-1.60	4.03E-01	8.77E-01
**KCNQ5**	-1.33	4.26E-02	1.00E+00	-0.69	4.03E-01	8.77E-01
**AC105036.3**	-2.73	3.24E-02	1.00E+00	-0.63	4.03E-01	8.77E-01
**CRTC3**	-0.01	9.61E-01	1.00E+00	0.34	4.03E-01	8.77E-01
**HMGB2**	0.25	2.82E-01	1.00E+00	-0.16	4.03E-01	8.77E-01
**OTULINL**	-0.14	6.63E-01	1.00E+00	0.24	4.03E-01	8.77E-01
**NR1H3**	0.53	2.78E-01	1.00E+00	0.40	4.04E-01	8.77E-01
**AC093772.1**	-2.45	2.70E-01	1.00E+00	1.41	4.04E-01	8.77E-01
**ICA1L**	-0.15	7.26E-01	1.00E+00	-0.44	4.04E-01	8.77E-01
**DUOXA1**	-0.07	8.80E-01	1.00E+00	0.48	4.04E-01	8.77E-01
**AC245884.12**	3.82	9.57E-02	1.00E+00	2.46	4.04E-01	8.77E-01
**CHM**	0.22	3.22E-01	1.00E+00	-0.16	4.04E-01	8.77E-01
**SGSM2**	0.20	6.33E-01	1.00E+00	0.39	4.04E-01	8.77E-01
**MSH2**	-0.13	6.08E-01	1.00E+00	-0.19	4.04E-01	8.77E-01
**LPP-AS2**	0.39	3.04E-01	1.00E+00	-0.37	4.04E-01	8.77E-01
**LAMTOR4**	0.34	2.43E-01	1.00E+00	-0.38	4.04E-01	8.77E-01
**MTA1**	-0.15	5.30E-01	1.00E+00	0.53	4.04E-01	8.77E-01
**TCEANC2**	0.13	5.36E-01	1.00E+00	-0.20	4.04E-01	8.77E-01
**AP002884.1**	0.55	2.61E-01	1.00E+00	-0.47	4.04E-01	8.77E-01
**RPL7AP50**	-1.70	6.79E-02	1.00E+00	-0.48	4.04E-01	8.77E-01
**CEBPG**	0.14	6.05E-01	1.00E+00	-0.15	4.04E-01	8.77E-01
**GAS8**	-0.19	4.97E-01	1.00E+00	0.26	4.04E-01	8.77E-01
**ZBTB2**	-0.05	8.16E-01	1.00E+00	0.17	4.04E-01	8.78E-01
**RDH5**	2.87	4.09E-02	1.00E+00	-0.54	4.04E-01	8.78E-01
**SPATA33**	-0.21	6.20E-01	1.00E+00	-0.20	4.05E-01	8.78E-01
**EDDM13**	-0.04	9.54E-01	1.00E+00	-0.42	4.05E-01	8.78E-01
**POLH**	-0.35	2.27E-01	1.00E+00	0.24	4.05E-01	8.78E-01

**PLEKHJ1**	0.01	9.60E-01	1.00E+00	0.15	4.05E-01	8.78E-01
**NEU3**	0.37	3.58E-01	1.00E+00	0.34	4.05E-01	8.78E-01
**GDPD2**	0.02	9.59E-01	1.00E+00	-0.73	4.05E-01	8.78E-01
**JMY**	-0.23	4.55E-01	1.00E+00	0.30	4.05E-01	8.78E-01
**PDPR**	0.21	5.62E-01	1.00E+00	0.33	4.05E-01	8.78E-01
**AC018529.2**	1.28	3.67E-01	1.00E+00	0.88	4.05E-01	8.78E-01
**SLC30A1**	-0.14	6.97E-01	1.00E+00	-0.32	4.05E-01	8.78E-01
**BIRC7**	-0.32	5.93E-01	1.00E+00	-0.54	4.05E-01	8.78E-01
**MTPN**	0.03	8.58E-01	1.00E+00	0.20	4.05E-01	8.78E-01
**FAM104A**	0.01	9.74E-01	1.00E+00	-0.24	4.05E-01	8.78E-01
**ZNF212**	-0.09	7.01E-01	1.00E+00	0.17	4.06E-01	8.78E-01
**EDEM1**	0.60	6.07E-03	4.63E-01	0.17	4.06E-01	8.78E-01
**AC092894.1**	-0.40	7.19E-01	1.00E+00	1.03	4.06E-01	8.78E-01
**EXT1**	-0.32	2.87E-01	1.00E+00	0.33	4.06E-01	8.78E-01
**MAP1LC3B**	0.10	6.79E-01	1.00E+00	-0.24	4.06E-01	8.78E-01
**AL844908.1**	0.08	9.34E-01	1.00E+00	-0.85	4.06E-01	8.78E-01
**CLEC2A**	0.44	3.80E-01	1.00E+00	-0.75	4.06E-01	8.78E-01
**TLK1**	-0.17	4.30E-01	1.00E+00	-0.29	4.06E-01	8.78E-01
**XLOC_001664**	-0.53	5.72E-01	1.00E+00	-1.22	4.06E-01	8.78E-01
**AC036108.1**	0.56	8.71E-01	1.00E+00	1.29	4.06E-01	8.78E-01
**NOP10**	0.04	8.52E-01	1.00E+00	-0.29	4.06E-01	8.78E-01
**NOVA2**	-0.04	9.35E-01	1.00E+00	0.55	4.06E-01	8.78E-01
**AC068987.5**	1.68	5.11E-02	1.00E+00	0.92	4.06E-01	8.78E-01
**AC010468.1**	0.24	7.55E-01	1.00E+00	-0.48	4.06E-01	8.78E-01
**XLOC_012692**	-0.59	1.26E-01	1.00E+00	0.61	4.06E-01	8.78E-01
**G26344**	0.30	5.94E-01	1.00E+00	-0.76	4.06E-01	8.78E-01
**COL15A1**	0.04	9.41E-01	1.00E+00	0.57	4.06E-01	8.78E-01
**ZDHHC2**	0.25	6.12E-01	1.00E+00	-0.28	4.06E-01	8.78E-01
**PLA2G2A**	0.39	5.48E-01	1.00E+00	-0.57	4.06E-01	8.78E-01
**U52111.1**	1.61	2.47E-01	1.00E+00	0.69	4.06E-01	8.79E-01
**ADSL**	-0.67	2.02E-01	1.00E+00	0.30	4.06E-01	8.79E-01
**P2RX7**	0.21	4.67E-01	1.00E+00	0.28	4.06E-01	8.79E-01

**AL139317.5**	-1.84	5.92E-01	1.00E+00	-0.90	4.06E-01	8.79E-01
**YEATS2-AS1**	-3.51	1.46E-01	1.00E+00	1.14	4.06E-01	8.79E-01
**SARS**	-0.10	6.10E-01	1.00E+00	0.15	4.06E-01	8.79E-01
**AP001646.1**	1.44	6.75E-01	1.00E+00	1.29	4.06E-01	8.79E-01
**KLF8**	-0.15	6.01E-01	1.00E+00	-0.18	4.07E-01	8.79E-01
**CTTNBP2NL**	-0.31	2.94E-01	1.00E+00	-0.16	4.07E-01	8.79E-01
**XLOC_004195**	1.26	2.68E-01	1.00E+00	-0.95	4.07E-01	8.79E-01
**CNKSR1**	-0.15	5.93E-01	1.00E+00	0.45	4.07E-01	8.79E-01
**ATMIN**	-0.04	8.44E-01	1.00E+00	-0.32	4.07E-01	8.79E-01
**ECT2**	-0.04	8.90E-01	1.00E+00	0.25	4.07E-01	8.79E-01
**PLK5**	-0.31	8.60E-01	1.00E+00	1.24	4.07E-01	8.79E-01
**XLOC_005542**	0.68	1.76E-01	1.00E+00	0.51	4.07E-01	8.79E-01
**G36324**	-2.98	5.91E-02	1.00E+00	1.19	4.07E-01	8.79E-01
**SPINK1**	0.68	5.22E-01	1.00E+00	0.85	4.07E-01	8.79E-01
**SRP19**	-0.06	8.20E-01	1.00E+00	-0.32	4.07E-01	8.79E-01
**PLCD1**	-0.08	6.94E-01	1.00E+00	0.26	4.07E-01	8.79E-01
**DEFB4A**	-2.99	2.98E-01	1.00E+00	2.61	4.07E-01	8.79E-01
**C1orf115**	0.50	4.34E-01	1.00E+00	-0.55	4.07E-01	8.79E-01
**EPHX2**	-0.18	6.29E-01	1.00E+00	-0.28	4.07E-01	8.79E-01
**DCXR**	0.03	8.97E-01	1.00E+00	-0.19	4.07E-01	8.79E-01
**LINC01806**	-0.99	1.79E-02	8.39E-01	0.39	4.07E-01	8.79E-01
**ACOT9**	0.25	4.39E-01	1.00E+00	0.17	4.07E-01	8.79E-01
**AC092747.4**	0.14	6.31E-01	1.00E+00	0.29	4.07E-01	8.79E-01
**FRMD5**	-0.80	2.14E-01	1.00E+00	0.79	4.07E-01	8.79E-01
**CACNB3**	-0.58	3.65E-02	1.00E+00	0.25	4.07E-01	8.79E-01
**ATRIP**	-0.07	8.09E-01	1.00E+00	-0.32	4.07E-01	8.79E-01
**SLFN12L**	0.50	5.17E-01	1.00E+00	0.75	4.07E-01	8.79E-01
**FUT10**	0.08	7.17E-01	1.00E+00	-0.27	4.07E-01	8.79E-01
**DGAT1**	0.10	8.12E-01	1.00E+00	-0.49	4.07E-01	8.79E-01
**SNRPEP2**	-0.34	9.22E-01	1.00E+00	-0.57	4.07E-01	8.79E-01
**AL163541.1**	-2.45	3.52E-01	1.00E+00	-1.87	4.07E-01	8.79E-01
**AATF**	-0.14	5.33E-01	1.00E+00	0.12	4.07E-01	8.79E-01

**KCNN3**	-0.10	7.84E-01	1.00E+00	0.43	4.08E-01	8.79E-01
**RGS5**	0.13	8.25E-01	1.00E+00	-0.40	4.08E-01	8.79E-01
**ANKRD36**	0.68	7.39E-02	1.00E+00	0.27	4.08E-01	8.79E-01
**TFIP11**	-0.20	3.29E-01	1.00E+00	0.19	4.08E-01	8.79E-01
**XLOC_010040**	0.14	9.02E-01	1.00E+00	-1.06	4.08E-01	8.79E-01
**TMCO6**	-0.39	1.63E-01	1.00E+00	-0.43	4.08E-01	8.79E-01
**PRRC2C**	-0.32	2.18E-01	1.00E+00	0.29	4.08E-01	8.79E-01
**ERBB4**	0.58	3.79E-01	1.00E+00	-0.67	4.08E-01	8.79E-01
**HFM1**	0.18	8.10E-01	1.00E+00	-0.80	4.08E-01	8.79E-01
**AC063948.1**	-0.51	5.42E-01	1.00E+00	-0.55	4.08E-01	8.79E-01
**ZNF586**	0.08	8.14E-01	1.00E+00	0.25	4.08E-01	8.79E-01
**RNF170**	0.13	6.56E-01	1.00E+00	-0.21	4.08E-01	8.79E-01
**API5**	0.06	7.43E-01	1.00E+00	-0.18	4.08E-01	8.79E-01
**SLC38A6**	0.09	8.30E-01	1.00E+00	-0.39	4.08E-01	8.79E-01
**LGI4**	-0.03	9.42E-01	1.00E+00	0.57	4.08E-01	8.79E-01
**GTDC1**	0.01	9.66E-01	1.00E+00	-0.22	4.08E-01	8.79E-01
**SPRR2B**	-0.83	3.50E-01	1.00E+00	2.59	4.08E-01	8.79E-01
**AC104819.3**	1.14	9.67E-02	1.00E+00	0.69	4.08E-01	8.79E-01
**POU5F2**	-0.50	7.17E-01	1.00E+00	-0.79	4.08E-01	8.79E-01
**CNIH2**	-1.02	7.30E-03	5.19E-01	-0.63	4.08E-01	8.79E-01
**HSPD1P1**	0.09	9.57E-01	1.00E+00	-0.40	4.08E-01	8.79E-01
**AL662907.2**	-0.25	7.94E-01	1.00E+00	0.67	4.08E-01	8.79E-01
**AC239799.2**	0.88	2.36E-01	1.00E+00	-0.72	4.08E-01	8.79E-01
**JMJD8**	-0.16	5.22E-01	1.00E+00	-0.44	4.08E-01	8.79E-01
**AP000351.10**	-1.51	4.38E-01	1.00E+00	0.87	4.08E-01	8.79E-01
**TBC1D14**	0.05	8.22E-01	1.00E+00	0.27	4.08E-01	8.79E-01
**ANKRD11**	-0.28	2.68E-01	1.00E+00	0.38	4.08E-01	8.79E-01
**DOP1B**	-0.50	1.36E-02	7.32E-01	-0.30	4.08E-01	8.79E-01
**LPL**	3.70	8.41E-03	5.67E-01	-0.74	4.08E-01	8.79E-01
**TJP3**	-0.30	2.23E-01	1.00E+00	-0.43	4.09E-01	8.79E-01
**FGFR1**	0.52	2.85E-01	1.00E+00	0.48	4.09E-01	8.79E-01
**COQ2**	-0.03	9.44E-01	1.00E+00	-0.27	4.09E-01	8.79E-01

**C20orf144**	-2.06	3.43E-01	1.00E+00	-0.96	4.09E-01	8.79E-01
**PRPF31**	-0.16	4.85E-01	1.00E+00	0.14	4.09E-01	8.79E-01
**ILK**	5.13	3.79E-02	1.00E+00	0.72	4.09E-01	8.79E-01
**HEBP1**	0.22	2.66E-01	1.00E+00	-0.17	4.09E-01	8.79E-01
**AC068587.2**	-0.47	5.95E-01	1.00E+00	-0.54	4.09E-01	8.79E-01
**NDUFB8**	-0.38	5.29E-01	1.00E+00	-0.61	4.09E-01	8.79E-01
**NIPA1**	0.01	9.56E-01	1.00E+00	-0.43	4.09E-01	8.79E-01
**RPL5P1**	0.44	8.99E-01	1.00E+00	-0.64	4.09E-01	8.79E-01
**SSRP1**	-0.15	4.56E-01	1.00E+00	0.27	4.09E-01	8.79E-01
**CATSPERE**	-0.32	7.58E-01	1.00E+00	-0.62	4.09E-01	8.79E-01
**RHOBTB3**	0.39	1.53E-01	1.00E+00	-0.35	4.09E-01	8.79E-01
**G26250**	0.76	5.26E-01	1.00E+00	-0.78	4.09E-01	8.79E-01
**LINC01588**	0.30	5.21E-01	1.00E+00	-0.29	4.09E-01	8.79E-01
**SLTM**	0.05	8.06E-01	1.00E+00	0.20	4.09E-01	8.79E-01
**TUBA3FP**	-2.69	4.26E-01	1.00E+00	-1.12	4.09E-01	8.79E-01
**ALDH3A1**	0.02	9.66E-01	1.00E+00	-0.50	4.09E-01	8.79E-01
**ICOS**	-0.38	7.28E-01	1.00E+00	2.60	4.09E-01	8.79E-01
**XLOC_006415**	-1.16	7.37E-01	1.00E+00	1.41	4.09E-01	8.79E-01
**PCDHGB7**	-0.04	9.02E-01	1.00E+00	0.41	4.09E-01	8.79E-01
**TRIP4**	-0.02	9.35E-01	1.00E+00	0.12	4.09E-01	8.79E-01
**AP000759.1**	-0.62	1.70E-01	1.00E+00	0.49	4.09E-01	8.79E-01
**ENPP6**	1.55	1.81E-01	1.00E+00	0.78	4.09E-01	8.79E-01
**HCP5**	0.12	7.47E-01	1.00E+00	0.30	4.09E-01	8.79E-01
**AC078785.1**	-1.82	2.11E-01	1.00E+00	0.61	4.09E-01	8.79E-01
**TSPO**	-0.06	8.40E-01	1.00E+00	0.20	4.09E-01	8.79E-01
**MAN1A1**	0.76	7.16E-03	5.13E-01	0.45	4.10E-01	8.79E-01
**IFNGR1**	0.22	2.80E-01	1.00E+00	-0.22	4.10E-01	8.79E-01
**PSMD12**	-0.12	5.16E-01	1.00E+00	-0.21	4.10E-01	8.79E-01
**RPL37A**	-0.09	7.66E-01	1.00E+00	0.24	4.10E-01	8.79E-01
**DEPDC5**	-0.10	7.52E-01	1.00E+00	0.36	4.10E-01	8.79E-01
**AC026401.2**	-0.88	7.20E-01	1.00E+00	0.77	4.10E-01	8.79E-01
**F2RL2**	-0.42	5.01E-01	1.00E+00	0.48	4.10E-01	8.79E-01

**AC015813.5**	-3.16	1.72E-02	8.25E-01	0.58	4.10E-01	8.79E-01
**TAF1B**	-0.26	1.94E-01	1.00E+00	-0.19	4.10E-01	8.79E-01
**AC073389.1**	-0.33	6.32E-01	1.00E+00	-0.47	4.10E-01	8.79E-01
**MTND4P12**	0.44	3.57E-01	1.00E+00	0.63	4.10E-01	8.79E-01
**AKIP1**	-0.12	6.13E-01	1.00E+00	0.19	4.10E-01	8.79E-01
**AL662907.1**	-0.35	5.66E-01	1.00E+00	0.73	4.10E-01	8.79E-01
**AP001330.5**	-0.68	3.63E-01	1.00E+00	0.55	4.10E-01	8.79E-01
**CMTM3**	0.05	8.76E-01	1.00E+00	0.51	4.10E-01	8.79E-01
**NECAP1**	0.25	2.71E-01	1.00E+00	-0.19	4.10E-01	8.79E-01
**CEACAM5**	0.17	8.05E-01	1.00E+00	-0.56	4.10E-01	8.79E-01
**DALRD3**	0.12	5.25E-01	1.00E+00	0.16	4.10E-01	8.79E-01
**AL513122.2**	-2.81	1.55E-01	1.00E+00	-0.95	4.10E-01	8.79E-01
**GATAD2A**	-0.31	3.16E-01	1.00E+00	0.32	4.10E-01	8.79E-01
**AC080013.5**	-2.09	2.12E-01	1.00E+00	-0.74	4.10E-01	8.79E-01
**PSMG3-AS1**	0.02	9.74E-01	1.00E+00	0.35	4.10E-01	8.79E-01
**TAF1A**	0.28	6.28E-01	1.00E+00	0.49	4.10E-01	8.79E-01
**TEX35**	-0.59	5.57E-01	1.00E+00	-0.93	4.10E-01	8.79E-01
**ZNF790-AS1**	-0.19	6.48E-01	1.00E+00	-0.35	4.10E-01	8.79E-01
**GPC5**	0.63	5.29E-01	1.00E+00	-0.74	4.10E-01	8.79E-01
**CCDC136**	-0.72	2.63E-01	1.00E+00	0.54	4.10E-01	8.79E-01
**TMX2**	0.10	6.52E-01	1.00E+00	-0.16	4.10E-01	8.79E-01
**GRIP1**	-0.62	5.85E-02	1.00E+00	-0.47	4.10E-01	8.79E-01
**G23403**	1.20	5.22E-01	1.00E+00	-0.75	4.11E-01	8.79E-01
**ABCB1**	-0.51	1.14E-01	1.00E+00	0.44	4.11E-01	8.79E-01
**AC007448.3**	-2.45	4.74E-01	1.00E+00	-1.06	4.11E-01	8.79E-01
**XLOC_005452**	0.55	4.69E-01	1.00E+00	0.57	4.11E-01	8.79E-01
**SMG6**	-0.19	4.60E-01	1.00E+00	0.43	4.11E-01	8.79E-01
**RAB11B-AS1**	0.20	7.90E-01	1.00E+00	-0.33	4.11E-01	8.79E-01
**CERS5**	0.07	6.11E-01	1.00E+00	0.10	4.11E-01	8.79E-01
**FAM221A**	0.05	8.95E-01	1.00E+00	-0.37	4.11E-01	8.79E-01
**MT1E**	0.01	9.74E-01	1.00E+00	-0.28	4.11E-01	8.79E-01
**SAMD12**	0.11	7.54E-01	1.00E+00	-0.31	4.11E-01	8.79E-01

**AC010618.3**	0.00	1.00E+00	1.00E+00	0.54	4.11E-01	8.79E-01
**MRPL45**	0.04	8.31E-01	1.00E+00	-0.17	4.11E-01	8.79E-01
**GLT1D1**	-1.08	7.29E-03	5.19E-01	0.54	4.11E-01	8.79E-01
**FAM86EP**	-0.57	1.11E-01	1.00E+00	-0.42	4.11E-01	8.79E-01
**SIL1**	0.12	7.04E-01	1.00E+00	0.11	4.11E-01	8.79E-01
**CTNNB1**	-0.11	5.70E-01	1.00E+00	0.26	4.11E-01	8.79E-01
**CELSR1**	-0.28	3.56E-01	1.00E+00	0.52	4.11E-01	8.79E-01
**AC034236.1**	-0.18	7.41E-01	1.00E+00	-0.42	4.11E-01	8.79E-01
**SEL1L3**	0.08	7.93E-01	1.00E+00	0.47	4.11E-01	8.79E-01
**LSP1P4**	0.54	4.51E-01	1.00E+00	0.35	4.11E-01	8.79E-01
**ARHGEF7**	0.21	3.78E-01	1.00E+00	0.21	4.11E-01	8.79E-01
**RCAN2**	0.17	6.68E-01	1.00E+00	-0.36	4.11E-01	8.79E-01
**PIANP**	1.14	2.85E-01	1.00E+00	-0.89	4.11E-01	8.79E-01
**AP003351.1**	-2.43	1.93E-01	1.00E+00	1.09	4.11E-01	8.79E-01
**HNRNPM**	-0.17	4.18E-01	1.00E+00	0.16	4.12E-01	8.79E-01
**MARVELD1**	-0.13	6.85E-01	1.00E+00	0.42	4.12E-01	8.79E-01
**AADACL3**	0.97	7.63E-01	1.00E+00	-2.57	4.12E-01	8.79E-01
**TSHZ1**	-0.16	4.84E-01	1.00E+00	0.28	4.12E-01	8.79E-01
**PCAT6**	-0.56	3.12E-01	1.00E+00	-0.39	4.12E-01	8.79E-01
**SLC23A2**	0.28	5.73E-01	1.00E+00	0.34	4.12E-01	8.79E-01
**PRELID2**	-0.12	6.90E-01	1.00E+00	-0.27	4.12E-01	8.79E-01
**PGR**	0.54	3.20E-01	1.00E+00	0.43	4.12E-01	8.79E-01
**CIR1**	0.07	6.96E-01	1.00E+00	0.18	4.12E-01	8.79E-01
**TMEM116**	0.35	7.15E-02	1.00E+00	-0.36	4.12E-01	8.79E-01
**TAS2R5**	-2.08	5.05E-01	1.00E+00	1.30	4.12E-01	8.80E-01
**TUBB4B**	-0.64	8.21E-02	1.00E+00	-0.22	4.12E-01	8.80E-01
**SUPV3L1**	0.26	2.39E-01	1.00E+00	-0.14	4.12E-01	8.80E-01
**PTPRS**	-0.02	9.45E-01	1.00E+00	0.61	4.12E-01	8.80E-01
**SLC22A18AS**	0.12	8.28E-01	1.00E+00	0.29	4.12E-01	8.80E-01
**AL354696.1**	-0.44	6.95E-01	1.00E+00	0.84	4.13E-01	8.80E-01
**ZNF432**	0.09	8.03E-01	1.00E+00	0.38	4.13E-01	8.80E-01
**KHSRP**	-0.28	2.27E-01	1.00E+00	0.48	4.13E-01	8.80E-01

**HBP1**	0.15	5.05E-01	1.00E+00	-0.27	4.13E-01	8.80E-01
**AC138696.2**	-0.01	9.90E-01	1.00E+00	-0.44	4.13E-01	8.80E-01
**G26573**	-1.18	3.12E-01	1.00E+00	-0.60	4.13E-01	8.80E-01
**ANKRD37**	-0.04	8.58E-01	1.00E+00	0.24	4.13E-01	8.80E-01
**PLD6**	-0.55	1.87E-01	1.00E+00	-0.35	4.13E-01	8.80E-01
**PGM5P2**	0.72	3.92E-01	1.00E+00	-0.57	4.13E-01	8.80E-01
**AIG1**	0.13	6.32E-01	1.00E+00	-0.21	4.13E-01	8.80E-01
**SMG1P5**	0.28	5.80E-01	1.00E+00	-0.39	4.13E-01	8.80E-01
**ZNF430**	-0.03	9.09E-01	1.00E+00	-0.29	4.13E-01	8.80E-01
**AC116351.1**	0.53	7.31E-01	1.00E+00	-1.15	4.13E-01	8.80E-01
**BAZ1B**	0.08	7.19E-01	1.00E+00	0.34	4.13E-01	8.80E-01
**AC073896.3**	0.83	5.55E-01	1.00E+00	-0.48	4.13E-01	8.80E-01
**AC012414.5**	1.13	3.06E-01	1.00E+00	0.76	4.13E-01	8.80E-01
**STK32A-AS1**	0.28	7.88E-01	1.00E+00	-0.67	4.13E-01	8.80E-01
**AC005586.1**	-0.80	3.63E-01	1.00E+00	-0.68	4.13E-01	8.80E-01
**RPL21P1**	-0.56	8.73E-01	1.00E+00	-0.60	4.13E-01	8.80E-01
**CCNF**	-0.02	9.36E-01	1.00E+00	-0.44	4.13E-01	8.80E-01
**FAT2**	-0.43	2.15E-01	1.00E+00	0.45	4.13E-01	8.80E-01
**GALNTL6**	0.04	9.60E-01	1.00E+00	0.81	4.13E-01	8.80E-01
**TCF20**	-0.47	6.30E-02	1.00E+00	0.54	4.14E-01	8.80E-01
**EBP**	-0.10	7.53E-01	1.00E+00	-0.27	4.14E-01	8.80E-01
**IL17RA**	0.01	9.64E-01	1.00E+00	0.48	4.14E-01	8.80E-01
**ACOX2**	0.98	1.19E-01	1.00E+00	-0.44	4.14E-01	8.80E-01
**CLK4**	0.18	7.03E-01	1.00E+00	-0.25	4.14E-01	8.81E-01
**LYST**	0.11	6.82E-01	1.00E+00	-0.18	4.14E-01	8.81E-01
**AC004637.1**	0.33	8.61E-01	1.00E+00	1.35	4.14E-01	8.81E-01
**SPESP1**	-1.12	1.03E-01	1.00E+00	-0.43	4.14E-01	8.81E-01
**ZP3**	0.22	4.42E-01	1.00E+00	-0.32	4.14E-01	8.81E-01
**GPAT4**	0.10	6.16E-01	1.00E+00	0.23	4.14E-01	8.81E-01
**APELA**	-0.71	4.28E-01	1.00E+00	0.79	4.14E-01	8.81E-01
**METTL6**	0.19	4.08E-01	1.00E+00	-0.20	4.14E-01	8.81E-01
**XLOC_006898**	0.01	9.92E-01	1.00E+00	-0.69	4.14E-01	8.81E-01

**FTSJ3**	-0.13	4.74E-01	1.00E+00	0.20	4.14E-01	8.81E-01
**DDX56**	-0.12	5.50E-01	1.00E+00	0.14	4.14E-01	8.81E-01
**ATAD2**	0.38	2.13E-01	1.00E+00	-0.27	4.14E-01	8.81E-01
**AC025470.2**	2.18	3.29E-01	1.00E+00	-0.83	4.14E-01	8.81E-01
**ARMCX3**	0.54	2.61E-01	1.00E+00	-0.25	4.14E-01	8.81E-01
**AOC1**	-1.36	4.08E-03	3.55E-01	-0.62	4.14E-01	8.81E-01
**BAIAP2-DT**	-0.27	4.87E-01	1.00E+00	0.46	4.15E-01	8.81E-01
**CD7**	-0.76	2.56E-01	1.00E+00	2.55	4.15E-01	8.81E-01
**NOP58**	-0.13	6.11E-01	1.00E+00	-0.27	4.15E-01	8.81E-01
**LARP1**	-0.27	2.41E-01	1.00E+00	0.30	4.15E-01	8.81E-01
**EFCAB5**	-2.68	3.29E-02	1.00E+00	-0.64	4.15E-01	8.81E-01
**XLOC_014403**	-0.41	8.00E-01	1.00E+00	-0.70	4.15E-01	8.81E-01
**G19572**	0.26	8.62E-01	1.00E+00	0.84	4.15E-01	8.81E-01
**TSC22D3**	0.63	2.06E-01	1.00E+00	-0.33	4.15E-01	8.81E-01
**VGLL3**	0.29	3.77E-01	1.00E+00	0.38	4.15E-01	8.81E-01
**XLOC_000294**	1.39	5.22E-02	1.00E+00	0.39	4.15E-01	8.81E-01
**BNIP3P11**	-0.42	6.38E-01	1.00E+00	0.56	4.15E-01	8.81E-01
**HDX**	-0.25	5.34E-01	1.00E+00	0.30	4.15E-01	8.81E-01
**CCNYL1**	-0.29	3.01E-01	1.00E+00	0.42	4.15E-01	8.81E-01
**ARHGAP26**	0.01	9.58E-01	1.00E+00	0.39	4.15E-01	8.81E-01
**PIPOX**	-0.29	5.41E-01	1.00E+00	0.47	4.15E-01	8.81E-01
**KRT71**	-6.02	6.82E-02	1.00E+00	-2.56	4.15E-01	8.81E-01
**UBLCP1**	-0.01	9.75E-01	1.00E+00	-0.11	4.15E-01	8.81E-01
**IL11RA**	0.25	5.57E-01	1.00E+00	0.27	4.15E-01	8.81E-01
**LINC01572**	2.72	1.11E-01	1.00E+00	0.73	4.15E-01	8.81E-01
**CNNM2**	-0.20	4.74E-01	1.00E+00	0.34	4.15E-01	8.81E-01
**SMURF1**	0.12	5.70E-01	1.00E+00	0.31	4.15E-01	8.81E-01
**AC007996.1**	-0.39	5.72E-01	1.00E+00	0.52	4.15E-01	8.81E-01
**TCAF2P1**	1.06	2.10E-01	1.00E+00	0.82	4.15E-01	8.81E-01
**DHX38**	-0.05	7.66E-01	1.00E+00	0.32	4.16E-01	8.81E-01
**ZRANB1**	0.06	8.31E-01	1.00E+00	-0.24	4.16E-01	8.81E-01
**ZNF397**	0.19	5.38E-01	1.00E+00	-0.19	4.16E-01	8.81E-01

**MRPL57**	-0.24	1.90E-01	1.00E+00	-0.28	4.16E-01	8.81E-01
**SNX13**	0.32	1.56E-01	1.00E+00	-0.15	4.16E-01	8.81E-01
**FOXI1**	0.59	6.37E-01	1.00E+00	-2.46	4.16E-01	8.81E-01
**SEC13**	-0.17	3.93E-01	1.00E+00	0.11	4.16E-01	8.81E-01
**XLOC_010047**	-0.49	4.65E-01	1.00E+00	-0.51	4.16E-01	8.81E-01
**SLC2A11**	0.44	3.03E-01	1.00E+00	0.35	4.16E-01	8.81E-01
**LINC01356**	-2.25	3.54E-01	1.00E+00	1.13	4.16E-01	8.81E-01
**CCDC127**	0.32	2.09E-01	1.00E+00	0.14	4.16E-01	8.81E-01
**U2SURP**	-0.34	1.20E-01	1.00E+00	-0.19	4.16E-01	8.81E-01
**THAP11**	0.16	4.98E-01	1.00E+00	-0.23	4.16E-01	8.81E-01
**RBPMS**	0.32	5.30E-01	1.00E+00	-0.45	4.16E-01	8.81E-01
**GPM6A**	-0.65	8.11E-01	1.00E+00	0.69	4.16E-01	8.81E-01
**FAM84A**	-0.63	5.40E-02	1.00E+00	0.30	4.16E-01	8.82E-01
**TRIM15**	-3.84	2.01E-02	8.84E-01	2.56	4.16E-01	8.82E-01
**TMEM40**	0.08	8.65E-01	1.00E+00	0.45	4.16E-01	8.82E-01
**RAG1**	-0.33	6.98E-01	1.00E+00	0.71	4.16E-01	8.82E-01
**ARHGEF5**	-0.25	5.29E-01	1.00E+00	-0.38	4.16E-01	8.82E-01
**MRPL50**	0.16	4.43E-01	1.00E+00	-0.26	4.16E-01	8.82E-01
**ADAMTS5**	0.80	7.12E-02	1.00E+00	0.51	4.16E-01	8.82E-01
**DNM1P35**	-1.06	3.20E-01	1.00E+00	1.17	4.16E-01	8.82E-01
**THTPA**	0.53	2.52E-01	1.00E+00	0.28	4.16E-01	8.82E-01
**MFSD9**	-0.05	7.94E-01	1.00E+00	0.25	4.16E-01	8.82E-01
**RPL34P27**	-1.19	7.32E-01	1.00E+00	-0.62	4.17E-01	8.82E-01
**PGAP3**	0.04	8.04E-01	1.00E+00	-0.26	4.17E-01	8.82E-01
**CENPP**	0.02	9.52E-01	1.00E+00	0.29	4.17E-01	8.82E-01
**XLOC_009313**	0.75	6.38E-01	1.00E+00	-0.91	4.17E-01	8.82E-01
**LINC00854**	-1.11	2.13E-01	1.00E+00	0.46	4.17E-01	8.82E-01
**XLOC_007043**	0.49	2.75E-01	1.00E+00	0.48	4.17E-01	8.82E-01
**XLOC_006349**	-0.74	2.81E-01	1.00E+00	-0.54	4.17E-01	8.82E-01
**RNU6-529P**	0.29	6.94E-01	1.00E+00	0.49	4.17E-01	8.82E-01
**AC067930.3**	0.63	5.32E-01	1.00E+00	-1.26	4.17E-01	8.82E-01
**KU-MEL-3**	-2.58	1.19E-02	6.78E-01	0.93	4.17E-01	8.82E-01

**SLC13A2**	-0.05	9.68E-01	1.00E+00	-0.61	4.17E-01	8.82E-01
**SLC46A3**	-0.16	6.88E-01	1.00E+00	-0.28	4.17E-01	8.82E-01
**FBXL18**	-0.17	6.53E-01	1.00E+00	0.43	4.17E-01	8.82E-01
**POPDC3**	1.42	3.35E-01	1.00E+00	1.05	4.17E-01	8.82E-01
**ST3GAL5-AS1**	0.18	8.45E-01	1.00E+00	0.65	4.17E-01	8.82E-01
**EEF1D**	-0.07	7.06E-01	1.00E+00	0.35	4.17E-01	8.82E-01
**DTX4**	0.48	2.46E-01	1.00E+00	0.55	4.17E-01	8.82E-01
**SMCO2**	0.95	4.09E-01	1.00E+00	0.98	4.17E-01	8.82E-01
**WDFY1**	0.09	5.78E-01	1.00E+00	0.21	4.17E-01	8.82E-01
**SOX17**	-1.13	2.99E-02	9.97E-01	-0.81	4.17E-01	8.82E-01
**AL136368.1**	0.89	4.13E-01	1.00E+00	-0.54	4.17E-01	8.82E-01
**G38764**	0.08	9.53E-01	1.00E+00	0.85	4.17E-01	8.82E-01
**JPX**	-0.38	4.93E-01	1.00E+00	-0.38	4.17E-01	8.82E-01
**USP11**	-0.01	9.75E-01	1.00E+00	0.14	4.17E-01	8.82E-01
**TFE3**	-0.01	9.65E-01	1.00E+00	0.38	4.17E-01	8.82E-01
**FBXO33**	-0.01	9.75E-01	1.00E+00	-0.27	4.17E-01	8.82E-01
**CSPG4**	-0.29	5.90E-01	1.00E+00	0.50	4.17E-01	8.82E-01
**DUSP19**	0.64	3.58E-01	1.00E+00	-0.44	4.17E-01	8.82E-01
**TMEM147-AS1**	-0.32	5.45E-01	1.00E+00	-0.34	4.17E-01	8.82E-01
**LRRN4CL**	0.31	4.45E-01	1.00E+00	0.40	4.18E-01	8.82E-01
**BFSP2**	0.58	5.28E-01	1.00E+00	-0.68	4.18E-01	8.82E-01
**KRT16P3**	0.15	9.30E-01	1.00E+00	0.57	4.18E-01	8.82E-01
**LRRC18**	1.74	9.78E-02	1.00E+00	-0.96	4.18E-01	8.82E-01
**DENND6A-AS1**	0.49	8.88E-01	1.00E+00	-0.72	4.18E-01	8.82E-01
**KHDC4**	0.27	6.52E-01	1.00E+00	0.32	4.18E-01	8.82E-01
**RNU6-118P**	-0.11	9.70E-01	1.00E+00	1.07	4.18E-01	8.82E-01
**AQP7P2**	4.17	2.90E-02	9.89E-01	-0.91	4.18E-01	8.82E-01
**ABHD18**	0.37	2.63E-01	1.00E+00	-0.23	4.18E-01	8.82E-01
**KIAA1143**	-0.11	4.74E-01	1.00E+00	-0.12	4.18E-01	8.82E-01
**PRDM1**	0.01	9.91E-01	1.00E+00	0.31	4.18E-01	8.82E-01
**AC005034.3**	-0.11	6.94E-01	1.00E+00	-0.30	4.18E-01	8.82E-01
**SRPK1**	0.03	9.45E-01	1.00E+00	0.26	4.18E-01	8.82E-01

**C6orf136**	-0.03	9.21E-01	1.00E+00	-0.24	4.18E-01	8.82E-01
**STK40**	0.07	6.91E-01	1.00E+00	-0.42	4.18E-01	8.82E-01
**SPATC1L**	-0.24	3.93E-01	1.00E+00	-0.51	4.18E-01	8.82E-01
**PPP1R15A**	-1.30	5.12E-02	1.00E+00	0.35	4.18E-01	8.82E-01
**CFI**	0.69	4.68E-01	1.00E+00	-0.47	4.18E-01	8.82E-01
**AC099524.1**	0.25	9.42E-01	1.00E+00	1.30	4.18E-01	8.82E-01
**SLC27A4**	-0.29	4.44E-01	1.00E+00	0.31	4.18E-01	8.82E-01
**DDIT4**	-0.03	9.59E-01	1.00E+00	0.35	4.18E-01	8.82E-01
**CYP4F26P**	-0.07	9.37E-01	1.00E+00	0.62	4.18E-01	8.82E-01
**ZNF70**	0.50	1.40E-01	1.00E+00	0.40	4.18E-01	8.82E-01
**PRR15**	-0.76	4.75E-01	1.00E+00	0.59	4.18E-01	8.82E-01
**CCDC170**	0.51	3.02E-01	1.00E+00	0.50	4.19E-01	8.82E-01
**AMBRA1**	-0.27	2.18E-01	1.00E+00	0.40	4.19E-01	8.82E-01
**G37757**	-2.87	2.82E-01	1.00E+00	0.88	4.19E-01	8.82E-01
**RNU6-6P**	-2.07	5.48E-01	1.00E+00	0.90	4.19E-01	8.82E-01
**NFATC3**	-0.06	7.64E-01	1.00E+00	-0.18	4.19E-01	8.82E-01
**MIR4754**	2.97	3.82E-01	1.00E+00	0.79	4.19E-01	8.82E-01
**AL121839.2**	0.79	1.24E-01	1.00E+00	-0.50	4.19E-01	8.82E-01
**ZNF521**	0.27	5.25E-01	1.00E+00	0.37	4.19E-01	8.83E-01
**DBF4B**	-0.62	9.79E-02	1.00E+00	0.43	4.19E-01	8.83E-01
**TINCR**	0.04	9.17E-01	1.00E+00	-0.49	4.19E-01	8.83E-01
**PLAC8L1**	0.14	9.10E-01	1.00E+00	0.91	4.19E-01	8.83E-01
**TCEAL1**	-0.04	8.26E-01	1.00E+00	-0.26	4.19E-01	8.83E-01
**AC087752.3**	-0.60	5.50E-01	1.00E+00	0.91	4.20E-01	8.83E-01
**CAP2P1**	0.87	3.31E-01	1.00E+00	-1.29	4.20E-01	8.83E-01
**USO1**	-0.02	9.44E-01	1.00E+00	-0.12	4.20E-01	8.83E-01
**ZCCHC12**	-1.52	6.33E-01	1.00E+00	1.70	4.20E-01	8.83E-01
**TP53INP2**	-0.53	2.17E-01	1.00E+00	0.51	4.20E-01	8.83E-01
**AL158829.1**	-1.23	7.24E-01	1.00E+00	1.37	4.20E-01	8.83E-01
**YAF2**	0.18	3.93E-01	1.00E+00	0.19	4.20E-01	8.83E-01
**TPD52**	0.12	6.04E-01	1.00E+00	-0.42	4.20E-01	8.83E-01
**AC097478.1**	0.50	3.04E-01	1.00E+00	0.49	4.20E-01	8.83E-01

**ZDHHC13**	0.17	6.42E-01	1.00E+00	-0.33	4.20E-01	8.84E-01
**ZNF431**	0.09	7.80E-01	1.00E+00	-0.23	4.20E-01	8.84E-01
**ZNF513**	-0.45	7.10E-02	1.00E+00	0.48	4.20E-01	8.84E-01
**CD19**	-0.38	8.30E-01	1.00E+00	0.87	4.20E-01	8.84E-01
**KRT17P2**	-0.05	9.87E-01	1.00E+00	0.76	4.20E-01	8.84E-01
**G28649**	0.50	4.38E-01	1.00E+00	-0.53	4.20E-01	8.84E-01
**XLOC_004611**	0.88	1.33E-01	1.00E+00	1.09	4.20E-01	8.84E-01
**AC104051.2**	0.50	6.11E-01	1.00E+00	1.06	4.21E-01	8.84E-01
**SGSH**	-0.34	1.20E-01	1.00E+00	0.29	4.21E-01	8.84E-01
**DTYMK**	-0.05	8.18E-01	1.00E+00	-0.20	4.21E-01	8.84E-01
**HSD17B4**	0.01	9.65E-01	1.00E+00	-0.14	4.21E-01	8.84E-01
**AC012291.2**	0.16	9.14E-01	1.00E+00	0.60	4.21E-01	8.84E-01
**RPS24**	-0.09	7.76E-01	1.00E+00	0.21	4.21E-01	8.84E-01
**EXOSC2**	0.09	6.74E-01	1.00E+00	-0.13	4.21E-01	8.84E-01
**NUDT16P1**	-0.51	2.53E-01	1.00E+00	-0.51	4.21E-01	8.84E-01
**AC025280.1**	2.11	1.44E-01	1.00E+00	0.76	4.21E-01	8.84E-01
**UQCC2**	-0.26	3.85E-01	1.00E+00	-0.30	4.21E-01	8.84E-01
**AC004585.1**	-3.16	1.48E-02	7.61E-01	0.97	4.21E-01	8.84E-01
**AC087203.2**	-1.53	4.05E-01	1.00E+00	-1.81	4.21E-01	8.84E-01
**NBDY**	0.00	9.95E-01	1.00E+00	-0.32	4.21E-01	8.84E-01
**MEI1**	0.57	5.03E-01	1.00E+00	0.50	4.21E-01	8.84E-01
**HDAC2**	0.15	4.83E-01	1.00E+00	-0.13	4.21E-01	8.84E-01
**TEX12**	-1.83	5.85E-01	1.00E+00	0.78	4.21E-01	8.84E-01
**SEC22C**	0.44	1.48E-01	1.00E+00	-0.19	4.21E-01	8.84E-01
**AC006547.1**	-0.15	7.44E-01	1.00E+00	0.68	4.21E-01	8.84E-01
**GRHPR**	-0.09	5.23E-01	1.00E+00	-0.10	4.21E-01	8.84E-01
**WDR86**	0.49	3.38E-01	1.00E+00	1.03	4.21E-01	8.84E-01
**CAMTA1-DT**	0.23	7.46E-01	1.00E+00	-0.81	4.21E-01	8.84E-01
**ACBD4**	0.22	5.07E-01	1.00E+00	-0.34	4.21E-01	8.84E-01
**PMS2P4**	0.10	7.18E-01	1.00E+00	0.20	4.21E-01	8.84E-01
**AC005703.6**	0.81	4.98E-01	1.00E+00	0.59	4.21E-01	8.84E-01
**SBSPON**	-0.29	5.83E-01	1.00E+00	-0.39	4.21E-01	8.84E-01

**ZNF385D**	0.06	8.79E-01	1.00E+00	0.31	4.21E-01	8.84E-01
**G5553**	-0.02	9.90E-01	1.00E+00	0.85	4.21E-01	8.84E-01
**GATA5**	-5.16	1.09E-05	3.44E-03	-1.59	4.21E-01	8.84E-01
**TMC3-AS1**	0.84	5.57E-01	1.00E+00	0.68	4.21E-01	8.84E-01
**ZMYM1**	0.17	6.22E-01	1.00E+00	-0.23	4.21E-01	8.84E-01
**EDC3**	0.00	9.97E-01	1.00E+00	-0.18	4.22E-01	8.84E-01
**BIRC3**	0.16	6.58E-01	1.00E+00	0.34	4.22E-01	8.84E-01
**MPHOSPH9**	-0.03	9.21E-01	1.00E+00	-0.32	4.22E-01	8.84E-01
**SPIN2A**	0.22	7.50E-01	1.00E+00	0.52	4.22E-01	8.84E-01
**NRXN1**	0.06	9.05E-01	1.00E+00	-0.50	4.22E-01	8.84E-01
**NUDT1**	-0.44	2.20E-02	8.96E-01	0.18	4.22E-01	8.84E-01
**FOCAD**	0.05	8.18E-01	1.00E+00	-0.17	4.22E-01	8.84E-01
**COMMD1**	-0.25	2.31E-01	1.00E+00	-0.28	4.22E-01	8.84E-01
**TCOF1**	-0.35	8.82E-02	1.00E+00	0.45	4.22E-01	8.84E-01
**SV2C**	-1.03	1.19E-01	1.00E+00	-0.66	4.22E-01	8.84E-01
**HES6**	-0.70	1.95E-01	1.00E+00	-0.60	4.22E-01	8.84E-01
**ZNF585A**	0.15	6.87E-01	1.00E+00	0.27	4.22E-01	8.84E-01
**RORA**	0.14	7.93E-01	1.00E+00	-0.41	4.22E-01	8.84E-01
**CRIPT**	0.30	2.47E-01	1.00E+00	-0.28	4.22E-01	8.84E-01
**HNRNPCP2**	-0.14	6.90E-01	1.00E+00	-0.19	4.22E-01	8.84E-01
**BCL7B**	0.08	7.46E-01	1.00E+00	-0.35	4.22E-01	8.84E-01
**RND3**	-0.23	4.18E-01	1.00E+00	0.22	4.22E-01	8.84E-01
**AFF3**	0.50	2.76E-01	1.00E+00	-0.47	4.22E-01	8.84E-01
**NIPAL4**	-0.07	9.00E-01	1.00E+00	0.49	4.22E-01	8.84E-01
**PAM16**	0.02	9.78E-01	1.00E+00	0.39	4.22E-01	8.84E-01
**AC130466.1**	0.57	5.01E-01	1.00E+00	-0.85	4.22E-01	8.84E-01
**MCM10**	-0.45	2.32E-01	1.00E+00	0.43	4.22E-01	8.84E-01
**TRIM31-AS1**	0.23	8.66E-01	1.00E+00	0.64	4.22E-01	8.84E-01
**ANXA8L1**	-0.38	4.52E-01	1.00E+00	0.50	4.22E-01	8.84E-01
**AC092718.3**	-0.90	4.00E-01	1.00E+00	0.58	4.22E-01	8.84E-01
**MYRFL**	-0.27	6.91E-01	1.00E+00	-0.52	4.22E-01	8.84E-01
**TEX10**	-0.09	6.70E-01	1.00E+00	0.18	4.22E-01	8.84E-01

**ARL4A**	0.17	5.68E-01	1.00E+00	-0.31	4.23E-01	8.84E-01
**PSCA**	-0.52	1.86E-01	1.00E+00	0.50	4.23E-01	8.84E-01
**ZNF460**	0.07	8.50E-01	1.00E+00	0.42	4.23E-01	8.84E-01
**ANXA4**	0.02	9.09E-01	1.00E+00	-0.11	4.23E-01	8.84E-01
**XLOC_009167**	-2.82	2.79E-01	1.00E+00	-1.04	4.23E-01	8.84E-01
**WDR18**	-0.32	3.20E-01	1.00E+00	0.21	4.23E-01	8.84E-01
**SLC7A1**	-0.04	9.02E-01	1.00E+00	0.33	4.23E-01	8.84E-01
**SOX2-OT**	-0.06	9.48E-01	1.00E+00	-0.50	4.23E-01	8.84E-01
**S100A7**	-1.32	1.04E-01	1.00E+00	2.51	4.23E-01	8.84E-01
**AC243964.3**	-0.16	6.56E-01	1.00E+00	0.43	4.23E-01	8.84E-01
**ZNF71**	-0.02	9.13E-01	1.00E+00	-0.31	4.23E-01	8.84E-01
**IL2RA**	0.61	4.98E-01	1.00E+00	2.51	4.23E-01	8.84E-01
**AC034231.1**	-0.12	8.71E-01	1.00E+00	0.56	4.23E-01	8.84E-01
**ANKDD1A**	0.55	2.03E-01	1.00E+00	-0.41	4.23E-01	8.84E-01
**RBM47**	0.11	7.38E-01	1.00E+00	0.21	4.23E-01	8.84E-01
**NUP85**	0.01	9.79E-01	1.00E+00	-0.15	4.23E-01	8.84E-01
**AC087672.2**	-0.69	7.32E-01	1.00E+00	0.69	4.23E-01	8.84E-01
**LRRC37A5P**	-0.10	9.23E-01	1.00E+00	0.46	4.23E-01	8.84E-01
**AL390719.1**	-0.74	3.88E-01	1.00E+00	0.46	4.23E-01	8.84E-01
**CLIC6**	0.08	8.78E-01	1.00E+00	0.53	4.23E-01	8.84E-01
**RACGAP1**	-0.02	9.45E-01	1.00E+00	-0.21	4.23E-01	8.84E-01
**HNRNPF**	-0.06	7.98E-01	1.00E+00	0.14	4.23E-01	8.84E-01
**C9orf40**	0.38	3.20E-01	1.00E+00	-0.47	4.24E-01	8.84E-01
**ZNF117**	0.05	9.06E-01	1.00E+00	-0.40	4.24E-01	8.84E-01
**GAS2L1**	-0.43	2.23E-01	1.00E+00	-0.49	4.24E-01	8.84E-01
**ELF3**	-0.69	1.86E-01	1.00E+00	0.46	4.24E-01	8.84E-01
**BCOR**	-0.49	1.08E-01	1.00E+00	0.30	4.24E-01	8.84E-01
**RNVU1-20**	-1.51	5.84E-01	1.00E+00	-0.65	4.24E-01	8.84E-01
**SIRT5**	-0.20	4.97E-01	1.00E+00	0.19	4.24E-01	8.84E-01
**AC009403.1**	0.17	8.52E-01	1.00E+00	-0.49	4.24E-01	8.84E-01
**SMIM10L2B**	0.05	8.76E-01	1.00E+00	-0.58	4.24E-01	8.84E-01
**NARS**	-0.11	5.33E-01	1.00E+00	-0.13	4.24E-01	8.84E-01

**TMEM128**	0.28	2.01E-01	1.00E+00	-0.26	4.24E-01	8.85E-01
**FOXJ3**	0.07	6.16E-01	1.00E+00	-0.26	4.24E-01	8.85E-01
**NACA**	-0.11	6.78E-01	1.00E+00	-0.19	4.24E-01	8.85E-01
**ZDHHC20**	-0.04	9.04E-01	1.00E+00	0.21	4.24E-01	8.85E-01
**AL035071.2**	-2.07	1.62E-01	1.00E+00	-0.68	4.24E-01	8.85E-01
**SLC9A8**	-0.18	5.72E-01	1.00E+00	0.41	4.24E-01	8.85E-01
**CLINT1**	0.18	4.81E-01	1.00E+00	-0.11	4.24E-01	8.85E-01
**NONO**	-0.10	5.19E-01	1.00E+00	0.14	4.24E-01	8.85E-01
**MCIDAS**	-2.54	3.63E-01	1.00E+00	-1.71	4.25E-01	8.85E-01
**IFT172**	-0.15	6.01E-01	1.00E+00	-0.27	4.25E-01	8.85E-01
**XRCC6**	-0.11	4.66E-01	1.00E+00	-0.06	4.25E-01	8.85E-01
**EIF4B**	0.03	9.15E-01	1.00E+00	0.20	4.25E-01	8.85E-01
**LINC01115**	0.65	8.12E-01	1.00E+00	0.72	4.25E-01	8.85E-01
**RICTOR**	0.03	9.03E-01	1.00E+00	0.23	4.25E-01	8.85E-01
**TBC1D12**	0.42	1.07E-01	1.00E+00	-0.27	4.25E-01	8.85E-01
**RBM15-AS1**	-2.66	8.06E-02	1.00E+00	-0.83	4.25E-01	8.85E-01
**CES2**	-0.21	2.48E-01	1.00E+00	0.16	4.25E-01	8.85E-01
**AC110048.2**	-0.93	5.92E-01	1.00E+00	-1.12	4.25E-01	8.85E-01
**CACNG4**	0.72	4.09E-01	1.00E+00	0.99	4.25E-01	8.85E-01
**AC025048.4**	0.47	5.95E-01	1.00E+00	0.55	4.25E-01	8.85E-01
**XLOC_005221**	-0.14	9.58E-01	1.00E+00	0.84	4.25E-01	8.85E-01
**SHLD1**	-0.02	9.51E-01	1.00E+00	-0.15	4.25E-01	8.85E-01
**XLOC_009415**	-1.08	4.34E-01	1.00E+00	0.91	4.25E-01	8.85E-01
**RPL7P46**	1.49	6.65E-01	1.00E+00	-0.87	4.25E-01	8.85E-01
**LRRC75A-AS1**	-0.02	9.46E-01	1.00E+00	0.20	4.25E-01	8.85E-01
**RPS15A**	-0.14	6.45E-01	1.00E+00	-0.34	4.25E-01	8.85E-01
**MAP1LC3C**	1.50	4.34E-02	1.00E+00	0.72	4.25E-01	8.85E-01
**EEF1A1P5**	-0.02	9.47E-01	1.00E+00	-0.17	4.25E-01	8.85E-01
**B4GALNT1**	-0.23	7.31E-01	1.00E+00	0.64	4.25E-01	8.85E-01
**FGFBP3**	-0.04	9.24E-01	1.00E+00	-0.59	4.25E-01	8.85E-01
**PDE4DIP**	0.09	6.91E-01	1.00E+00	0.20	4.25E-01	8.85E-01
**ITSN1**	0.47	2.42E-01	1.00E+00	-0.29	4.25E-01	8.85E-01

**IQCB1**	-0.02	9.25E-01	1.00E+00	-0.20	4.25E-01	8.85E-01
**MYH10**	0.06	8.56E-01	1.00E+00	0.31	4.25E-01	8.85E-01
**CYP39A1**	0.38	3.76E-01	1.00E+00	-0.49	4.25E-01	8.85E-01
**RPH3A**	-2.79	4.15E-01	1.00E+00	1.25	4.25E-01	8.85E-01
**KIF1B**	-0.03	8.96E-01	1.00E+00	0.25	4.25E-01	8.85E-01
**NSMCE3**	0.00	9.99E-01	1.00E+00	-0.46	4.25E-01	8.85E-01
**FMO2**	1.07	1.29E-01	1.00E+00	-0.58	4.25E-01	8.85E-01
**TMEM267**	0.16	6.35E-01	1.00E+00	-0.33	4.26E-01	8.85E-01
**DDI2**	0.28	2.84E-01	1.00E+00	0.19	4.26E-01	8.85E-01
**LIMD1**	0.14	6.19E-01	1.00E+00	0.46	4.26E-01	8.85E-01
**AC080013.6**	1.63	4.49E-02	1.00E+00	0.45	4.26E-01	8.85E-01
**AC016716.1**	-0.23	9.47E-01	1.00E+00	-0.62	4.26E-01	8.85E-01
**AC016831.4**	-1.10	7.50E-01	1.00E+00	0.65	4.26E-01	8.85E-01
**NRIP2**	0.12	8.34E-01	1.00E+00	0.43	4.26E-01	8.85E-01
**PLCB3**	-0.39	8.91E-02	1.00E+00	0.47	4.26E-01	8.85E-01
**ZNF622**	-0.19	4.24E-01	1.00E+00	0.16	4.26E-01	8.85E-01
**PAK4**	-0.26	2.41E-01	1.00E+00	0.46	4.26E-01	8.85E-01
**ABCF3**	0.01	9.44E-01	1.00E+00	0.12	4.26E-01	8.85E-01
**TGFB2**	-0.28	4.97E-01	1.00E+00	0.33	4.26E-01	8.85E-01
**XLOC_008224**	-1.46	6.72E-01	1.00E+00	-2.11	4.26E-01	8.85E-01
**HOXA13**	0.60	1.81E-01	1.00E+00	0.67	4.26E-01	8.85E-01
**MIRLET7A1**	0.86	8.04E-01	1.00E+00	-0.82	4.26E-01	8.85E-01
**LINC02062**	0.68	4.69E-01	1.00E+00	-0.54	4.26E-01	8.85E-01
**TMEM248**	0.14	3.98E-01	1.00E+00	-0.33	4.26E-01	8.85E-01
**PEX11B**	0.02	9.22E-01	1.00E+00	-0.22	4.26E-01	8.85E-01
**LINC01805**	0.24	6.68E-01	1.00E+00	-0.58	4.27E-01	8.85E-01
**XLOC_004198**	-1.33	2.20E-01	1.00E+00	1.25	4.27E-01	8.85E-01
**GABRD**	-1.37	4.35E-02	1.00E+00	-0.47	4.27E-01	8.85E-01
**AC090515.4**	0.78	6.47E-01	1.00E+00	0.73	4.27E-01	8.85E-01
**MIR4530**	-0.64	8.32E-01	1.00E+00	0.76	4.27E-01	8.85E-01
**RNU6-722P**	-2.80	3.95E-02	1.00E+00	0.45	4.27E-01	8.85E-01
**NEK2**	0.45	2.85E-01	1.00E+00	-0.46	4.27E-01	8.85E-01

**AC011450.1**	-1.11	3.50E-01	1.00E+00	0.95	4.27E-01	8.85E-01
**GAREM1**	0.12	6.53E-01	1.00E+00	-0.27	4.27E-01	8.85E-01
**FAM86KP**	-2.26	3.51E-01	1.00E+00	0.88	4.27E-01	8.85E-01
**SHQ1**	0.01	9.51E-01	1.00E+00	-0.17	4.27E-01	8.85E-01
**B3GALNT1**	0.55	2.53E-01	1.00E+00	0.20	4.27E-01	8.85E-01
**PHETA2**	-0.04	8.95E-01	1.00E+00	0.32	4.27E-01	8.85E-01
**CHD8**	-0.29	2.71E-01	1.00E+00	0.34	4.27E-01	8.85E-01
**AP000753.1**	NA	NA	NA	-0.81	4.27E-01	8.85E-01
**TNKS**	0.31	2.40E-01	1.00E+00	0.26	4.27E-01	8.85E-01
**ASPHD2**	-0.50	4.86E-01	1.00E+00	-0.44	4.27E-01	8.85E-01
**ANKRD46**	0.58	8.27E-02	1.00E+00	-0.23	4.27E-01	8.85E-01
**NBEAP1**	1.53	4.70E-01	1.00E+00	0.97	4.27E-01	8.85E-01
**GALNT18**	-0.26	3.16E-01	1.00E+00	0.22	4.27E-01	8.85E-01
**RAB43P1**	-0.61	5.04E-01	1.00E+00	0.86	4.27E-01	8.85E-01
**ARAF**	-0.08	7.05E-01	1.00E+00	0.13	4.27E-01	8.85E-01
**CNOT6L**	0.01	9.75E-01	1.00E+00	0.18	4.27E-01	8.85E-01
**COX6B1**	-0.17	2.41E-01	1.00E+00	-0.17	4.27E-01	8.85E-01
**AP3S2**	0.33	2.70E-01	1.00E+00	-0.24	4.27E-01	8.85E-01
**TRPC4**	-0.73	6.72E-01	1.00E+00	0.78	4.28E-01	8.85E-01
**NOTCH4**	0.53	3.93E-01	1.00E+00	0.46	4.28E-01	8.85E-01
**CPEB2-DT**	-0.08	9.51E-01	1.00E+00	0.52	4.28E-01	8.85E-01
**AC092155.2**	-1.26	7.16E-01	1.00E+00	-0.54	4.28E-01	8.85E-01
**AC022182.1**	-1.13	5.51E-01	1.00E+00	0.85	4.28E-01	8.85E-01
**DPY19L3**	0.29	4.94E-01	1.00E+00	0.36	4.28E-01	8.85E-01
**ZNF33B**	0.05	8.70E-01	1.00E+00	-0.22	4.28E-01	8.85E-01
**RGPD6**	1.29	3.81E-01	1.00E+00	0.43	4.28E-01	8.85E-01
**LINC00519**	-0.50	7.34E-01	1.00E+00	0.48	4.28E-01	8.85E-01
**ARMH1**	-0.45	5.85E-01	1.00E+00	0.67	4.28E-01	8.85E-01
**FDXR**	-0.24	4.83E-01	1.00E+00	0.29	4.28E-01	8.85E-01
**KLHL23**	0.30	4.76E-01	1.00E+00	-0.34	4.28E-01	8.85E-01
**LINC00847**	0.37	4.72E-01	1.00E+00	0.26	4.28E-01	8.85E-01
**CXorf40A**	-0.07	7.57E-01	1.00E+00	0.21	4.28E-01	8.85E-01

**LRG1**	-0.98	3.59E-02	1.00E+00	-0.42	4.28E-01	8.85E-01
**FKBP8**	-0.02	9.23E-01	1.00E+00	0.48	4.28E-01	8.85E-01
**ZW10**	0.19	5.52E-01	1.00E+00	-0.20	4.28E-01	8.85E-01
**ZNF580**	0.00	9.89E-01	1.00E+00	-0.39	4.28E-01	8.85E-01
**CFL1**	-0.15	4.15E-01	1.00E+00	-0.11	4.28E-01	8.85E-01
**BANP**	0.12	5.44E-01	1.00E+00	0.25	4.28E-01	8.85E-01
**SMIM15**	0.13	5.09E-01	1.00E+00	-0.20	4.28E-01	8.85E-01
**AC069366.1**	2.60	4.45E-01	1.00E+00	-1.39	4.28E-01	8.85E-01
**AP004609.1**	-1.16	7.39E-01	1.00E+00	1.45	4.28E-01	8.85E-01
**TRIM16L**	0.03	9.21E-01	1.00E+00	-0.31	4.28E-01	8.85E-01
**CHRNB4**	-0.72	5.42E-01	1.00E+00	0.87	4.28E-01	8.85E-01
**WDFY2**	0.09	8.13E-01	1.00E+00	0.25	4.28E-01	8.85E-01
**FAM78B**	-0.85	1.06E-01	1.00E+00	0.70	4.28E-01	8.85E-01
**RPP25L**	0.14	5.94E-01	1.00E+00	0.17	4.28E-01	8.85E-01
**SLC8A1**	-0.19	6.88E-01	1.00E+00	0.34	4.28E-01	8.85E-01
**RTL8A**	-0.13	4.90E-01	1.00E+00	-0.16	4.28E-01	8.85E-01
**NCAPG**	0.39	2.32E-01	1.00E+00	0.42	4.28E-01	8.85E-01
**XLOC_007437**	0.80	1.12E-01	1.00E+00	-0.67	4.29E-01	8.85E-01
**BX470102.2**	-0.18	6.64E-01	1.00E+00	-0.34	4.29E-01	8.85E-01
**TNNI1**	-0.59	2.81E-01	1.00E+00	-0.59	4.29E-01	8.85E-01
**AP000688.2**	0.72	5.69E-01	1.00E+00	-0.78	4.29E-01	8.85E-01
**TMEM160**	-0.30	1.79E-01	1.00E+00	-0.49	4.29E-01	8.85E-01
**WNT9B**	-0.58	6.25E-01	1.00E+00	0.82	4.29E-01	8.85E-01
**WARS2-IT1**	1.91	4.63E-02	1.00E+00	0.99	4.29E-01	8.85E-01
**OSBPL5**	-0.04	8.61E-01	1.00E+00	0.39	4.29E-01	8.85E-01
**PRDX1P1**	0.87	5.39E-01	1.00E+00	-0.57	4.29E-01	8.85E-01
**ATM**	0.15	6.82E-01	1.00E+00	0.31	4.29E-01	8.85E-01
**XLOC_007801**	0.91	1.53E-01	1.00E+00	-0.57	4.29E-01	8.85E-01
**RPS4X**	-0.22	5.06E-01	1.00E+00	0.22	4.29E-01	8.85E-01
**NAA38**	0.04	8.63E-01	1.00E+00	-0.24	4.29E-01	8.85E-01
**HSPB1P1**	-0.62	5.40E-01	1.00E+00	-0.55	4.29E-01	8.85E-01
**AC010997.3**	1.46	2.78E-01	1.00E+00	-0.78	4.29E-01	8.85E-01

**LLPH-DT**	-0.39	5.81E-01	1.00E+00	-0.82	4.29E-01	8.85E-01
**USP40**	0.40	4.57E-02	1.00E+00	0.31	4.29E-01	8.85E-01
**AC009244.1**	1.77	8.51E-03	5.68E-01	-0.37	4.29E-01	8.85E-01
**AC000403.1**	-0.30	5.01E-01	1.00E+00	0.59	4.29E-01	8.85E-01
**CTDNEP1**	0.01	9.78E-01	1.00E+00	0.23	4.29E-01	8.86E-01
**AC004967.1**	-0.43	3.44E-01	1.00E+00	-0.36	4.29E-01	8.86E-01
**DDO**	0.64	4.03E-01	1.00E+00	0.70	4.29E-01	8.86E-01
**EFCAB2**	0.49	2.46E-01	1.00E+00	-0.31	4.29E-01	8.86E-01
**RCVRN**	-0.05	9.54E-01	1.00E+00	0.91	4.29E-01	8.86E-01
**XLOC_004842**	-0.51	8.61E-01	1.00E+00	1.58	4.29E-01	8.86E-01
**AC024597.1**	-2.94	3.83E-01	1.00E+00	1.02	4.29E-01	8.86E-01
**MIR573**	1.72	1.95E-01	1.00E+00	0.75	4.30E-01	8.86E-01
**LUC7L2**	0.13	5.47E-01	1.00E+00	-0.21	4.30E-01	8.86E-01
**AC012615.1**	-2.30	5.27E-02	1.00E+00	-0.50	4.30E-01	8.86E-01
**CASP5**	3.55	1.81E-01	1.00E+00	2.49	4.30E-01	8.86E-01
**DRAXIN**	-1.05	2.41E-01	1.00E+00	0.69	4.30E-01	8.86E-01
**TK2**	0.86	5.02E-02	1.00E+00	0.35	4.30E-01	8.86E-01
**DPM1**	0.20	4.64E-01	1.00E+00	-0.17	4.30E-01	8.86E-01
**RASGEF1C**	0.33	6.68E-01	1.00E+00	-0.81	4.30E-01	8.86E-01
**COL22A1**	-1.41	1.24E-01	1.00E+00	2.48	4.30E-01	8.86E-01
**GABPB1-AS1**	0.14	7.50E-01	1.00E+00	-0.22	4.30E-01	8.86E-01
**GATD1**	-0.32	2.14E-01	1.00E+00	0.38	4.30E-01	8.86E-01
**AC008915.2**	-0.37	8.59E-01	1.00E+00	-0.56	4.30E-01	8.86E-01
**AC008763.1**	-1.10	3.83E-01	1.00E+00	-0.61	4.30E-01	8.86E-01
**COQ8A**	0.21	2.82E-01	1.00E+00	-0.32	4.30E-01	8.86E-01
**CCL28**	0.20	6.30E-01	1.00E+00	0.34	4.30E-01	8.86E-01
**GTF3A**	0.03	9.10E-01	1.00E+00	-0.20	4.30E-01	8.86E-01
**RPS15AP12**	1.32	4.06E-01	1.00E+00	-0.45	4.30E-01	8.86E-01
**AC044860.1**	0.15	8.64E-01	1.00E+00	1.04	4.30E-01	8.86E-01
**STXBP3**	0.08	7.77E-01	1.00E+00	-0.26	4.30E-01	8.86E-01
**MAN1A2**	-0.12	4.74E-01	1.00E+00	-0.17	4.30E-01	8.86E-01
**PRDM11**	0.08	7.00E-01	1.00E+00	0.53	4.30E-01	8.86E-01

**PPP1R35**	0.31	2.88E-01	1.00E+00	-0.27	4.30E-01	8.86E-01
**OSBP**	0.01	9.53E-01	1.00E+00	-0.30	4.30E-01	8.86E-01
**MRM2**	0.00	9.96E-01	1.00E+00	-0.19	4.30E-01	8.86E-01
**AC117503.3**	-3.23	6.91E-02	1.00E+00	-1.21	4.30E-01	8.86E-01
**YTHDF1**	-0.11	5.74E-01	1.00E+00	-0.32	4.30E-01	8.86E-01
**SYTL3**	0.18	7.43E-01	1.00E+00	0.22	4.30E-01	8.86E-01
**NMU**	0.01	9.83E-01	1.00E+00	-0.63	4.31E-01	8.86E-01
**HDC**	0.60	3.95E-01	1.00E+00	0.70	4.31E-01	8.86E-01
**SEC16A**	-0.12	5.01E-01	1.00E+00	0.35	4.31E-01	8.86E-01
**TEAD3**	-0.14	6.77E-01	1.00E+00	0.39	4.31E-01	8.86E-01
**TSR2**	0.25	1.88E-01	1.00E+00	-0.17	4.31E-01	8.86E-01
**PCYOX1L**	-0.10	6.93E-01	1.00E+00	0.34	4.31E-01	8.86E-01
**CD300H**	0.49	7.55E-01	1.00E+00	1.29	4.31E-01	8.86E-01
**ITGA6-AS1**	-3.31	1.29E-01	1.00E+00	-0.94	4.31E-01	8.86E-01
**ZSCAN22**	-0.04	9.19E-01	1.00E+00	-0.37	4.31E-01	8.86E-01
**UBE2MP1**	-0.18	9.35E-01	1.00E+00	-0.43	4.31E-01	8.86E-01
**ZBP1**	-0.51	6.91E-01	1.00E+00	2.47	4.31E-01	8.86E-01
**ACAA2**	1.37	1.40E-02	7.46E-01	-0.33	4.31E-01	8.86E-01
**COMT**	-0.04	8.47E-01	1.00E+00	0.14	4.31E-01	8.86E-01
**SSX2IP**	-0.30	2.83E-01	1.00E+00	-0.23	4.31E-01	8.86E-01
**SNRPN**	1.14	3.18E-01	1.00E+00	-0.54	4.31E-01	8.86E-01
**CCDC169**	-0.59	7.92E-01	1.00E+00	-0.81	4.31E-01	8.86E-01
**RPIA**	0.10	6.99E-01	1.00E+00	-0.21	4.31E-01	8.86E-01
**CD9**	0.30	9.87E-02	1.00E+00	-0.23	4.31E-01	8.86E-01
**HEPACAM2**	-2.56	1.89E-01	1.00E+00	-1.23	4.31E-01	8.86E-01
**AC073857.1**	-0.94	2.59E-01	1.00E+00	0.41	4.31E-01	8.86E-01
**RBM7**	0.28	2.85E-01	1.00E+00	-0.26	4.31E-01	8.86E-01
**NRK**	0.85	4.67E-01	1.00E+00	0.98	4.32E-01	8.86E-01
**CCNQ**	-0.13	5.51E-01	1.00E+00	-0.45	4.32E-01	8.86E-01
**LUZP2**	0.37	7.20E-01	1.00E+00	-0.58	4.32E-01	8.86E-01
**TREML2**	0.49	7.44E-01	1.00E+00	1.44	4.32E-01	8.86E-01
**CCDC181**	-0.66	5.06E-01	1.00E+00	-0.54	4.32E-01	8.86E-01

**SFMBT1**	-0.23	3.12E-01	1.00E+00	-0.19	4.32E-01	8.86E-01
**AC019197.1**	1.20	3.60E-01	1.00E+00	-0.74	4.32E-01	8.86E-01
**SPATA24**	-0.04	8.91E-01	1.00E+00	0.29	4.32E-01	8.86E-01
**NUDT5**	-0.11	5.32E-01	1.00E+00	-0.21	4.32E-01	8.86E-01
**HOXB8**	0.01	9.90E-01	1.00E+00	0.66	4.32E-01	8.86E-01
**C3orf35**	0.20	8.65E-01	1.00E+00	-0.75	4.32E-01	8.86E-01
**MATN2**	-0.04	8.08E-01	1.00E+00	-0.23	4.32E-01	8.86E-01
**LINC02511**	3.40	2.31E-02	9.12E-01	-0.81	4.32E-01	8.86E-01
**SEC24B**	0.05	8.13E-01	1.00E+00	-0.21	4.32E-01	8.86E-01
**LINC02593**	0.21	8.41E-01	1.00E+00	1.04	4.32E-01	8.86E-01
**GLIS1**	-0.34	5.11E-01	1.00E+00	0.41	4.32E-01	8.86E-01
**NOX1**	0.10	9.14E-01	1.00E+00	-0.48	4.32E-01	8.86E-01
**ADH1C**	1.79	1.78E-01	1.00E+00	-0.86	4.32E-01	8.86E-01
**NXN**	-0.15	5.35E-01	1.00E+00	0.42	4.32E-01	8.86E-01
**MANBAL**	-0.11	5.36E-01	1.00E+00	-0.14	4.32E-01	8.86E-01
**XLOC_005182**	-0.23	8.46E-01	1.00E+00	0.86	4.32E-01	8.86E-01
**ADGRD1**	0.81	8.58E-02	1.00E+00	0.41	4.32E-01	8.86E-01
**WASHC5**	0.05	7.66E-01	1.00E+00	-0.15	4.32E-01	8.86E-01
**AP002812.3**	-1.89	5.83E-01	1.00E+00	0.82	4.32E-01	8.86E-01
**SLC30A7**	0.17	5.75E-01	1.00E+00	0.21	4.32E-01	8.86E-01
**ZSCAN23**	0.46	7.28E-01	1.00E+00	0.68	4.33E-01	8.86E-01
**ZNF142**	-0.38	4.05E-02	1.00E+00	0.49	4.33E-01	8.86E-01
**BCAS2**	-0.09	6.30E-01	1.00E+00	-0.27	4.33E-01	8.86E-01
**GLIPR2**	0.44	2.86E-01	1.00E+00	0.34	4.33E-01	8.86E-01
**HAUS8**	0.30	2.10E-01	1.00E+00	0.20	4.33E-01	8.86E-01
**RAI2**	0.55	1.90E-01	1.00E+00	-0.38	4.33E-01	8.86E-01
**AC012640.2**	-0.42	2.31E-01	1.00E+00	-0.20	4.33E-01	8.86E-01
**LINC01397**	-2.96	5.39E-02	1.00E+00	1.67	4.33E-01	8.86E-01
**CDK17**	-0.11	6.31E-01	1.00E+00	-0.16	4.33E-01	8.86E-01
**CCL17**	0.42	5.32E-01	1.00E+00	0.59	4.33E-01	8.86E-01
**AL445231.1**	-3.11	1.67E-02	8.13E-01	0.59	4.33E-01	8.86E-01
**TRBV10-2**	0.56	8.71E-01	1.00E+00	1.18	4.33E-01	8.86E-01

**DNAJB1**	-0.44	1.88E-01	1.00E+00	0.18	4.33E-01	8.86E-01
**TMEM186**	0.05	8.37E-01	1.00E+00	-0.13	4.33E-01	8.86E-01
**VDAC1P1**	-0.69	7.91E-01	1.00E+00	-0.42	4.33E-01	8.86E-01
**AC092691.1**	0.79	4.57E-01	1.00E+00	0.65	4.33E-01	8.86E-01
**AL355512.1**	-1.97	1.87E-01	1.00E+00	-0.74	4.33E-01	8.86E-01
**EXTL1**	0.06	9.31E-01	1.00E+00	-0.66	4.33E-01	8.86E-01
**C3orf62**	0.36	1.21E-01	1.00E+00	0.23	4.33E-01	8.86E-01
**LY86-AS1**	-1.47	4.51E-01	1.00E+00	-0.86	4.33E-01	8.86E-01
**MRPS7**	-0.18	2.07E-01	1.00E+00	-0.16	4.33E-01	8.86E-01
**MIR181A2HG**	0.09	8.92E-01	1.00E+00	-0.56	4.33E-01	8.86E-01
**MYO7A**	-0.22	7.41E-01	1.00E+00	0.74	4.33E-01	8.87E-01
**MOB2**	-0.22	3.09E-01	1.00E+00	-0.33	4.34E-01	8.87E-01
**ATP5PO**	0.17	5.55E-01	1.00E+00	-0.47	4.34E-01	8.87E-01
**RNF217-AS1**	-2.71	2.36E-02	9.15E-01	-0.50	4.34E-01	8.87E-01
**LYL1**	0.13	7.94E-01	1.00E+00	0.88	4.34E-01	8.87E-01
**PRO1804**	1.35	6.84E-01	1.00E+00	-1.04	4.34E-01	8.87E-01
**STK32C**	-0.34	1.30E-01	1.00E+00	0.28	4.34E-01	8.87E-01
**NGDN**	-0.06	7.42E-01	1.00E+00	-0.22	4.34E-01	8.87E-01
**AC244093.2**	NA	NA	NA	1.22	4.34E-01	8.87E-01
**KLF9**	-0.24	6.71E-01	1.00E+00	0.39	4.34E-01	8.87E-01
**UGT3A2**	0.68	3.19E-01	1.00E+00	-0.75	4.34E-01	8.87E-01
**AKAP12**	0.40	3.92E-01	1.00E+00	0.37	4.34E-01	8.87E-01
**BBS5**	0.23	3.84E-01	1.00E+00	0.64	4.34E-01	8.87E-01
**NMNAT2**	0.05	9.33E-01	1.00E+00	0.37	4.34E-01	8.87E-01
**HPF1**	0.06	7.65E-01	1.00E+00	-0.27	4.34E-01	8.87E-01
**TBXA2R**	-0.35	5.44E-01	1.00E+00	0.54	4.34E-01	8.87E-01
**FASTKD5**	-0.29	1.45E-01	1.00E+00	0.11	4.34E-01	8.87E-01
**CDCA8**	-0.01	9.83E-01	1.00E+00	0.30	4.34E-01	8.87E-01
**AC245014.3**	-0.47	8.12E-01	1.00E+00	-0.81	4.34E-01	8.87E-01
**CROCC2**	-0.64	5.58E-01	1.00E+00	1.32	4.34E-01	8.87E-01
**SPATC1**	-2.65	2.56E-01	1.00E+00	1.67	4.34E-01	8.87E-01
**ZRANB2-AS2**	0.21	7.71E-01	1.00E+00	0.47	4.35E-01	8.87E-01

**CCDC47**	-0.13	5.16E-01	1.00E+00	-0.17	4.35E-01	8.87E-01
**AC034236.2**	0.33	5.26E-01	1.00E+00	-0.38	4.35E-01	8.87E-01
**FAM200A**	0.26	3.82E-01	1.00E+00	0.29	4.35E-01	8.87E-01
**STRADA**	0.72	4.23E-01	1.00E+00	0.32	4.35E-01	8.87E-01
**XLOC_010829**	-0.02	9.86E-01	1.00E+00	0.80	4.35E-01	8.87E-01
**IAH1**	0.05	8.17E-01	1.00E+00	-0.17	4.35E-01	8.87E-01
**UTP6**	0.09	6.71E-01	1.00E+00	0.15	4.35E-01	8.87E-01
**AP005131.6**	-0.67	6.42E-01	1.00E+00	-0.91	4.35E-01	8.87E-01
**AL162578.1**	-0.25	9.42E-01	1.00E+00	0.74	4.35E-01	8.88E-01
**AC097493.4**	1.08	2.20E-01	1.00E+00	0.68	4.35E-01	8.88E-01
**FANCF**	-0.09	7.37E-01	1.00E+00	-0.23	4.35E-01	8.88E-01
**SELENOK**	0.10	5.67E-01	1.00E+00	-0.26	4.35E-01	8.88E-01
**ZNF236-DT**	-0.49	3.52E-01	1.00E+00	0.70	4.35E-01	8.88E-01
**POLR2G**	-0.18	2.81E-01	1.00E+00	-0.20	4.35E-01	8.88E-01
**FLJ45513**	-0.42	7.70E-01	1.00E+00	-1.05	4.35E-01	8.88E-01
**ASH2L**	0.02	8.93E-01	1.00E+00	0.16	4.35E-01	8.88E-01
**AL442663.4**	-1.50	4.04E-01	1.00E+00	-0.75	4.35E-01	8.88E-01
**POMZP3**	-0.06	9.02E-01	1.00E+00	-0.41	4.35E-01	8.88E-01
**ATP6V1FNB**	-0.06	8.93E-01	1.00E+00	0.54	4.36E-01	8.88E-01
**AC006330.1**	4.27	7.40E-02	1.00E+00	-1.10	4.36E-01	8.88E-01
**SGMS1-AS1**	0.13	7.46E-01	1.00E+00	0.36	4.36E-01	8.88E-01
**SNAI1**	0.31	2.85E-01	1.00E+00	1.73	4.36E-01	8.88E-01
**AC096751.1**	-0.29	8.37E-01	1.00E+00	-0.69	4.36E-01	8.88E-01
**BRK1**	0.07	6.31E-01	1.00E+00	-0.15	4.36E-01	8.88E-01
**STOX2**	-0.52	3.21E-02	1.00E+00	-0.23	4.36E-01	8.88E-01
**AC092809.4**	0.48	3.75E-01	1.00E+00	-0.67	4.36E-01	8.88E-01
**FKBP9P1**	-0.16	7.72E-01	1.00E+00	0.29	4.36E-01	8.88E-01
**P2RY12**	0.96	1.78E-01	1.00E+00	-0.63	4.36E-01	8.88E-01
**ST6GALNAC2**	-0.09	7.48E-01	1.00E+00	0.29	4.36E-01	8.88E-01
**GNA12**	0.11	7.56E-01	1.00E+00	0.44	4.36E-01	8.88E-01
**MAT2B**	0.23	2.98E-01	1.00E+00	-0.18	4.36E-01	8.88E-01
**LRR1**	0.10	7.38E-01	1.00E+00	-0.30	4.36E-01	8.89E-01

**AC139149.1**	-1.90	4.14E-02	1.00E+00	0.96	4.36E-01	8.89E-01
**THOC2**	0.14	4.94E-01	1.00E+00	0.09	4.36E-01	8.89E-01
**AC007541.1**	0.88	1.34E-01	1.00E+00	-0.58	4.36E-01	8.89E-01
**BECN1**	-0.03	8.32E-01	1.00E+00	-0.15	4.36E-01	8.89E-01
**PRB3**	-1.07	2.32E-01	1.00E+00	-0.66	4.36E-01	8.89E-01
**CYP2W1**	-0.23	6.71E-01	1.00E+00	-0.71	4.37E-01	8.89E-01
**ANXA5**	0.26	5.31E-01	1.00E+00	-0.28	4.37E-01	8.89E-01
**MAP3K1**	-0.13	6.73E-01	1.00E+00	-0.27	4.37E-01	8.89E-01
**ZNF326**	-0.39	9.27E-02	1.00E+00	-0.16	4.37E-01	8.89E-01
**BAZ2A**	-0.31	1.92E-01	1.00E+00	0.41	4.37E-01	8.89E-01
**AC068580.4**	-0.53	7.40E-01	1.00E+00	1.35	4.37E-01	8.89E-01
**G39837**	0.77	4.77E-01	1.00E+00	1.07	4.37E-01	8.89E-01
**LEFTY2**	-0.52	6.56E-01	1.00E+00	-0.80	4.37E-01	8.89E-01
**NORAD**	0.10	5.42E-01	1.00E+00	0.30	4.37E-01	8.89E-01
**MLH1**	-0.21	1.17E-01	1.00E+00	0.13	4.37E-01	8.89E-01
**ZBED5-AS1**	-0.12	6.72E-01	1.00E+00	-0.40	4.37E-01	8.89E-01
**LGALS7**	2.86	3.68E-01	1.00E+00	2.43	4.37E-01	8.89E-01
**PPP1R2B**	-1.71	1.89E-01	1.00E+00	-0.34	4.37E-01	8.89E-01
**XLOC_005050**	0.95	1.39E-01	1.00E+00	-0.71	4.37E-01	8.89E-01
**NRG2**	-0.32	3.61E-01	1.00E+00	-0.52	4.37E-01	8.89E-01
**LRFN4**	-0.27	3.96E-01	1.00E+00	0.59	4.37E-01	8.89E-01
**LRRK1**	-0.25	2.68E-01	1.00E+00	0.35	4.37E-01	8.89E-01
**ANKRD20A1**	0.29	8.44E-01	1.00E+00	0.63	4.37E-01	8.89E-01
**AL021707.2**	0.17	7.65E-01	1.00E+00	0.39	4.37E-01	8.89E-01
**ZNF846**	-0.42	1.26E-01	1.00E+00	-0.30	4.37E-01	8.89E-01
**MIB1**	0.17	4.38E-01	1.00E+00	0.24	4.37E-01	8.89E-01
**HLCS**	0.05	8.81E-01	1.00E+00	-0.22	4.38E-01	8.89E-01
**CARD19**	-0.21	2.77E-01	1.00E+00	-0.18	4.38E-01	8.89E-01
**PPP3CB**	-0.05	8.10E-01	1.00E+00	0.26	4.38E-01	8.89E-01
**CLASRP**	-0.30	3.13E-01	1.00E+00	0.41	4.38E-01	8.89E-01
**ZNF641**	0.11	7.23E-01	1.00E+00	0.34	4.38E-01	8.89E-01
**YWHAEP7**	-0.50	8.83E-01	1.00E+00	-0.69	4.38E-01	8.89E-01

**PAK1**	-0.18	4.06E-01	1.00E+00	0.18	4.38E-01	8.89E-01
**EGFL7**	-0.32	3.86E-01	1.00E+00	0.38	4.38E-01	8.89E-01
**KBTBD13**	0.30	7.44E-01	1.00E+00	-1.06	4.38E-01	8.89E-01
**PLK4**	-0.21	3.57E-01	1.00E+00	-0.28	4.38E-01	8.89E-01
**AC091849.1**	-0.74	3.72E-01	1.00E+00	0.70	4.38E-01	8.89E-01
**SMARCAL1**	-0.04	8.42E-01	1.00E+00	0.17	4.38E-01	8.89E-01
**CACNA2D1**	0.44	2.34E-01	1.00E+00	-0.38	4.38E-01	8.89E-01
**HMGN2P41**	0.25	7.27E-01	1.00E+00	-0.37	4.38E-01	8.89E-01
**ATP13A2**	-0.08	7.00E-01	1.00E+00	0.43	4.38E-01	8.89E-01
**AC010632.2**	0.00	9.99E-01	1.00E+00	1.01	4.38E-01	8.89E-01
**PKHD1L1**	0.62	3.41E-01	1.00E+00	0.68	4.38E-01	8.89E-01
**SLC30A3**	-1.82	1.33E-01	1.00E+00	-0.84	4.38E-01	8.89E-01
**AC006942.1**	NA	NA	NA	-0.90	4.38E-01	8.89E-01
**SMG8**	0.15	4.33E-01	1.00E+00	0.16	4.38E-01	8.89E-01
**XLOC_009994**	0.16	9.07E-01	1.00E+00	0.84	4.39E-01	8.89E-01
**AC138627.1**	2.10	4.02E-01	1.00E+00	1.09	4.39E-01	8.89E-01
**DKK1**	0.94	1.64E-01	1.00E+00	1.41	4.39E-01	8.89E-01
**APOC1P1**	-0.08	9.82E-01	1.00E+00	0.83	4.39E-01	8.89E-01
**NUP50-DT**	-0.28	4.57E-01	1.00E+00	0.19	4.39E-01	8.89E-01
**ARL8A**	0.03	8.95E-01	1.00E+00	0.40	4.39E-01	8.89E-01
**AC010754.1**	1.16	4.37E-01	1.00E+00	1.10	4.39E-01	8.89E-01
**TVP23C**	-0.03	9.37E-01	1.00E+00	-0.21	4.39E-01	8.89E-01
**NXT1**	-0.19	4.65E-01	1.00E+00	0.21	4.39E-01	8.89E-01
**DNAJC18**	0.24	6.12E-01	1.00E+00	0.47	4.39E-01	8.89E-01
**AL008635.1**	-1.19	7.29E-01	1.00E+00	0.74	4.39E-01	8.89E-01
**OPLAH**	-0.05	9.01E-01	1.00E+00	-0.58	4.39E-01	8.89E-01
**CDC27**	-0.03	8.29E-01	1.00E+00	0.14	4.39E-01	8.89E-01
**ENTPD5**	0.05	8.49E-01	1.00E+00	0.15	4.39E-01	8.89E-01
**FAM205C**	-0.85	5.27E-01	1.00E+00	0.81	4.39E-01	8.89E-01
**AC124248.1**	-0.53	6.63E-01	1.00E+00	0.43	4.39E-01	8.89E-01
**MARK2P9**	3.27	6.68E-02	1.00E+00	-0.78	4.39E-01	8.89E-01
**ADAMTSL3**	0.64	6.99E-02	1.00E+00	-0.28	4.39E-01	8.89E-01

**GSPT2**	0.17	6.81E-01	1.00E+00	-0.27	4.39E-01	8.89E-01
**RPL36**	-0.18	5.00E-01	1.00E+00	0.26	4.39E-01	8.89E-01
**SYS1**	0.32	1.32E-01	1.00E+00	0.15	4.39E-01	8.90E-01
**AL109918.1**	-0.05	9.31E-01	1.00E+00	0.58	4.39E-01	8.90E-01
**SLCO4A1**	0.02	9.54E-01	1.00E+00	0.24	4.39E-01	8.90E-01
**PODXL2**	-0.69	3.07E-02	1.00E+00	-0.59	4.39E-01	8.90E-01
**NPC1L1**	0.90	5.77E-01	1.00E+00	1.27	4.40E-01	8.90E-01
**DYNLT3**	-0.09	7.09E-01	1.00E+00	-0.25	4.40E-01	8.90E-01
**FDCSP**	-1.70	6.21E-01	1.00E+00	1.24	4.40E-01	8.90E-01
**IGSF9**	-0.42	1.91E-01	1.00E+00	-0.50	4.40E-01	8.90E-01
**AC079228.1**	0.47	8.34E-01	1.00E+00	-0.47	4.40E-01	8.90E-01
**XLOC_013347**	-0.64	3.56E-01	1.00E+00	0.58	4.40E-01	8.90E-01
**SYCP1**	-0.19	8.94E-01	1.00E+00	-0.99	4.40E-01	8.90E-01
**KLK4**	-1.05	3.30E-01	1.00E+00	0.88	4.40E-01	8.90E-01
**AC120036.4**	1.23	1.48E-01	1.00E+00	-0.78	4.40E-01	8.90E-01
**AC003965.1**	-0.86	8.02E-01	1.00E+00	-1.31	4.40E-01	8.90E-01
**G39803**	0.67	8.05E-01	1.00E+00	-0.58	4.40E-01	8.90E-01
**AFG3L2**	-0.11	6.07E-01	1.00E+00	-0.21	4.40E-01	8.90E-01
**BCL9**	-0.29	2.20E-01	1.00E+00	0.38	4.40E-01	8.90E-01
**N4BP1**	-0.07	8.01E-01	1.00E+00	0.33	4.40E-01	8.90E-01
**BPNT1**	-0.17	3.88E-01	1.00E+00	0.17	4.40E-01	8.90E-01
**SGSM1**	0.16	6.47E-01	1.00E+00	-0.39	4.40E-01	8.90E-01
**ZNF341**	0.15	6.70E-01	1.00E+00	0.33	4.40E-01	8.90E-01
**EPCAM**	0.38	4.93E-01	1.00E+00	-0.39	4.40E-01	8.90E-01
**CCT7P1**	0.55	6.57E-01	1.00E+00	1.15	4.40E-01	8.90E-01
**KLHDC1**	-0.23	6.04E-01	1.00E+00	-0.28	4.40E-01	8.90E-01
**LRP5**	0.05	8.71E-01	1.00E+00	0.43	4.40E-01	8.90E-01
**AL391825.1**	0.47	8.93E-01	1.00E+00	-1.32	4.40E-01	8.90E-01
**PDCD6IP**	-0.21	3.17E-01	1.00E+00	-0.15	4.40E-01	8.90E-01
**AC016769.1**	-1.05	3.21E-01	1.00E+00	1.03	4.40E-01	8.90E-01
**AC107075.1**	0.30	7.99E-01	1.00E+00	-0.47	4.40E-01	8.90E-01
**STK35**	0.07	7.51E-01	1.00E+00	0.28	4.40E-01	8.90E-01

**TEX41**	-0.17	6.73E-01	1.00E+00	0.31	4.40E-01	8.90E-01
**AC026748.1**	-1.19	2.55E-01	1.00E+00	1.06	4.40E-01	8.90E-01
**COPB1**	0.24	1.05E-01	1.00E+00	-0.13	4.40E-01	8.90E-01
**G39721**	-2.31	4.62E-02	1.00E+00	0.87	4.40E-01	8.90E-01
**EIF1AD**	-0.09	7.02E-01	1.00E+00	0.16	4.40E-01	8.90E-01
**AC130650.2**	-0.47	7.06E-01	1.00E+00	-0.64	4.41E-01	8.90E-01
**ST13**	0.06	7.72E-01	1.00E+00	-0.10	4.41E-01	8.90E-01
**XLOC_002900**	-0.71	7.14E-01	1.00E+00	-0.90	4.41E-01	8.90E-01
**VMP1**	0.23	1.49E-01	1.00E+00	-0.21	4.41E-01	8.90E-01
**G14419**	-0.74	4.63E-01	1.00E+00	-0.60	4.41E-01	8.90E-01
**POPDC2**	-0.49	4.33E-01	1.00E+00	0.43	4.41E-01	8.90E-01
**KRT14**	-0.30	3.26E-01	1.00E+00	0.41	4.41E-01	8.90E-01
**CTC-338M12.4**	0.05	9.44E-01	1.00E+00	-0.30	4.41E-01	8.90E-01
**NEK6**	0.21	6.10E-01	1.00E+00	0.38	4.41E-01	8.90E-01
**DBH**	-2.89	3.82E-02	1.00E+00	1.23	4.41E-01	8.90E-01
**TMEM141**	0.26	2.76E-01	1.00E+00	-0.23	4.41E-01	8.90E-01
**FAM151B**	0.06	9.28E-01	1.00E+00	-0.40	4.41E-01	8.90E-01
**PPP1R12A-AS1**	0.47	3.11E-01	1.00E+00	-0.43	4.41E-01	8.90E-01
**DPYD**	0.18	4.78E-01	1.00E+00	0.31	4.41E-01	8.90E-01
**SERF2**	0.12	5.43E-01	1.00E+00	-0.40	4.41E-01	8.90E-01
**AC139256.1**	-1.24	5.67E-01	1.00E+00	0.93	4.41E-01	8.90E-01
**DLG3-AS1**	-1.71	5.44E-01	1.00E+00	-0.65	4.41E-01	8.90E-01
**G3BP1**	0.25	2.13E-01	1.00E+00	-0.13	4.41E-01	8.90E-01
**CDK15**	0.61	3.00E-01	1.00E+00	0.53	4.41E-01	8.90E-01
**LRRC58**	0.07	7.06E-01	1.00E+00	-0.13	4.41E-01	8.90E-01
**NOL3**	-0.03	9.42E-01	1.00E+00	-0.25	4.41E-01	8.90E-01
**EHMT1**	-0.14	5.19E-01	1.00E+00	0.28	4.41E-01	8.90E-01
**IL9**	NA	NA	NA	2.07	4.41E-01	8.90E-01
**XRCC4**	0.11	7.53E-01	1.00E+00	-0.25	4.41E-01	8.90E-01
**ZNF765**	0.10	7.57E-01	1.00E+00	-0.20	4.42E-01	8.90E-01
**XLOC_006495**	0.20	9.20E-01	1.00E+00	0.95	4.42E-01	8.90E-01
**SLAMF1**	0.45	5.57E-01	1.00E+00	2.41	4.42E-01	8.90E-01

**XLOC_013263**	-2.90	8.34E-03	5.64E-01	-0.63	4.42E-01	8.90E-01
**LGALS8-AS1**	-2.97	2.92E-01	1.00E+00	-1.05	4.42E-01	8.90E-01
**IBTK**	0.20	4.83E-01	1.00E+00	0.23	4.42E-01	8.90E-01
**FLJ37035**	-0.40	6.57E-01	1.00E+00	-0.57	4.42E-01	8.90E-01
**FOXP4**	-0.34	2.98E-01	1.00E+00	0.53	4.42E-01	8.90E-01
**PCDHB15**	-0.27	5.60E-01	1.00E+00	0.49	4.42E-01	8.90E-01
**TBX2-AS1**	0.31	7.66E-01	1.00E+00	-0.56	4.42E-01	8.90E-01
**PLCG1**	0.00	9.75E-01	1.00E+00	0.43	4.42E-01	8.90E-01
**ARHGEF10L**	-0.09	7.58E-01	1.00E+00	0.33	4.42E-01	8.90E-01
**LMCD1**	0.10	7.96E-01	1.00E+00	0.34	4.42E-01	8.90E-01
**TKFC**	-0.09	6.53E-01	1.00E+00	-0.14	4.42E-01	8.90E-01
**ENDOD1**	0.02	9.39E-01	1.00E+00	0.19	4.42E-01	8.90E-01
**FTX**	0.27	3.84E-01	1.00E+00	-0.14	4.42E-01	8.90E-01
**CAGE1**	-2.89	3.93E-01	1.00E+00	0.93	4.42E-01	8.90E-01
**SERINC1**	0.28	2.36E-01	1.00E+00	-0.22	4.42E-01	8.90E-01
**FAM198B-AS1**	-0.57	3.74E-01	1.00E+00	-0.45	4.42E-01	8.90E-01
**ACTR5**	-0.09	6.57E-01	1.00E+00	-0.21	4.42E-01	8.90E-01
**TUT7**	0.15	7.23E-01	1.00E+00	0.21	4.42E-01	8.90E-01
**HSP90AA1**	0.07	6.95E-01	1.00E+00	0.20	4.42E-01	8.90E-01
**BLOC1S2**	-0.11	6.66E-01	1.00E+00	-0.27	4.42E-01	8.90E-01
**CHRNB1**	-0.13	5.71E-01	1.00E+00	-0.21	4.42E-01	8.90E-01
**PBXIP1**	0.02	9.56E-01	1.00E+00	0.27	4.43E-01	8.90E-01
**HAGLROS**	-1.46	2.75E-01	1.00E+00	-0.61	4.43E-01	8.90E-01
**OTUB2**	-0.76	2.13E-01	1.00E+00	0.42	4.43E-01	8.90E-01
**SPAG4**	0.06	9.26E-01	1.00E+00	0.84	4.43E-01	8.90E-01
**RPH3AL**	-0.42	2.52E-01	1.00E+00	-0.42	4.43E-01	8.91E-01
**TSG101**	0.08	7.02E-01	1.00E+00	-0.23	4.43E-01	8.91E-01
**UBTD2**	0.21	4.58E-01	1.00E+00	0.34	4.43E-01	8.91E-01
**GRIA4**	-0.11	8.42E-01	1.00E+00	-0.63	4.43E-01	8.91E-01
**ARL3**	-0.16	5.66E-01	1.00E+00	-0.20	4.43E-01	8.91E-01
**USP46**	0.40	7.56E-02	1.00E+00	0.18	4.43E-01	8.91E-01
**RPL21P16**	-0.10	7.77E-01	1.00E+00	-0.43	4.43E-01	8.91E-01

**PDCD11**	-0.32	2.47E-01	1.00E+00	0.28	4.43E-01	8.91E-01
**QRFP**	-0.28	7.94E-01	1.00E+00	1.03	4.43E-01	8.91E-01
**ZFR**	-0.15	3.93E-01	1.00E+00	-0.12	4.43E-01	8.91E-01
**EFR3B**	-0.19	7.77E-01	1.00E+00	0.40	4.43E-01	8.91E-01
**SLC35A4**	-0.27	9.83E-02	1.00E+00	0.10	4.43E-01	8.91E-01
**ROBO2**	-0.27	5.63E-01	1.00E+00	-0.40	4.43E-01	8.91E-01
**ZNF451-AS1**	-0.53	6.74E-01	1.00E+00	-0.50	4.44E-01	8.91E-01
**UBE2M**	-0.12	5.78E-01	1.00E+00	-0.41	4.44E-01	8.91E-01
**AGA**	-0.26	3.10E-01	1.00E+00	-0.24	4.44E-01	8.91E-01
**TRAPPC6A**	-0.12	5.55E-01	1.00E+00	-0.28	4.44E-01	8.91E-01
**AL133383.1**	-2.59	3.06E-01	1.00E+00	1.21	4.44E-01	8.91E-01
**BMPR1AP1**	-0.99	7.76E-01	1.00E+00	-0.54	4.44E-01	8.91E-01
**PKN2-AS1**	-0.51	5.23E-01	1.00E+00	-0.63	4.44E-01	8.91E-01
**AL034379.1**	-1.61	6.41E-01	1.00E+00	-0.56	4.44E-01	8.91E-01
**GNA13**	0.10	6.46E-01	1.00E+00	0.17	4.44E-01	8.91E-01
**PRSS30P**	-1.58	1.64E-01	1.00E+00	-0.91	4.44E-01	8.91E-01
**BAG3**	-0.27	2.37E-01	1.00E+00	-0.35	4.44E-01	8.91E-01
**AL355482.2**	-2.29	2.22E-01	1.00E+00	-1.07	4.44E-01	8.91E-01
**NF2**	0.04	8.70E-01	1.00E+00	0.13	4.44E-01	8.91E-01
**PPP2R2D**	0.03	8.31E-01	1.00E+00	-0.36	4.44E-01	8.91E-01
**RPL27**	-0.11	7.38E-01	1.00E+00	0.20	4.44E-01	8.91E-01
**ATP5MF**	-0.06	8.05E-01	1.00E+00	-0.39	4.44E-01	8.91E-01
**AL359532.1**	1.13	1.34E-01	1.00E+00	0.77	4.44E-01	8.91E-01
**PVALB**	-0.54	8.28E-01	1.00E+00	1.04	4.44E-01	8.91E-01
**HPGD**	0.21	6.61E-01	1.00E+00	-0.32	4.44E-01	8.91E-01
**ZFAND1**	0.14	5.48E-01	1.00E+00	-0.28	4.44E-01	8.91E-01
**AC092999.1**	1.58	2.89E-01	1.00E+00	0.75	4.44E-01	8.91E-01
**G12029**	1.37	3.90E-01	1.00E+00	1.04	4.44E-01	8.91E-01
**PRR7**	-1.16	1.76E-02	8.33E-01	-0.43	4.44E-01	8.91E-01
**AQP9**	0.61	3.09E-01	1.00E+00	0.36	4.44E-01	8.91E-01
**AC135506.1**	0.01	9.83E-01	1.00E+00	-0.53	4.44E-01	8.91E-01
**LRRC4C**	0.03	9.64E-01	1.00E+00	0.53	4.44E-01	8.91E-01

**RNF111**	0.00	9.88E-01	1.00E+00	0.18	4.44E-01	8.91E-01
**AC007298.2**	0.35	8.08E-01	1.00E+00	0.64	4.45E-01	8.91E-01
**CHST13**	1.15	4.11E-01	1.00E+00	0.96	4.45E-01	8.91E-01
**DDX39A**	-0.23	3.85E-01	1.00E+00	0.18	4.45E-01	8.91E-01
**SAP18**	0.04	7.94E-01	1.00E+00	-0.19	4.45E-01	8.91E-01
**TARSL2**	-0.14	6.39E-01	1.00E+00	-0.24	4.45E-01	8.91E-01
**TRAV25**	-1.45	5.31E-01	1.00E+00	1.24	4.45E-01	8.91E-01
**SNAP25**	-3.49	8.57E-02	1.00E+00	1.00	4.45E-01	8.91E-01
**AF201337.1**	-1.84	2.69E-01	1.00E+00	-0.32	4.45E-01	8.91E-01
**FBRSL1**	-0.37	2.55E-01	1.00E+00	0.56	4.45E-01	8.91E-01
**AL390879.1**	-0.51	4.45E-01	1.00E+00	-0.39	4.45E-01	8.91E-01
**AC107983.1**	0.66	5.48E-01	1.00E+00	-0.37	4.45E-01	8.91E-01
**ALG1L8P**	-0.31	6.99E-01	1.00E+00	-0.58	4.45E-01	8.91E-01
**DYNC1I1**	-0.48	2.83E-01	1.00E+00	-0.35	4.45E-01	8.91E-01
**AC005899.8**	-0.36	7.68E-01	1.00E+00	0.57	4.45E-01	8.91E-01
**SRSF9P1**	-2.46	4.68E-03	3.94E-01	0.60	4.45E-01	8.91E-01
**HNRNPL**	-0.19	3.97E-01	1.00E+00	0.40	4.45E-01	8.91E-01
**XLOC_014268**	-1.53	1.79E-01	1.00E+00	1.07	4.45E-01	8.91E-01
**SORL1**	0.26	6.75E-01	1.00E+00	0.28	4.45E-01	8.91E-01
**PHYHD1**	0.14	6.03E-01	1.00E+00	-0.29	4.46E-01	8.92E-01
**SSR1**	0.10	4.62E-01	1.00E+00	-0.15	4.46E-01	8.92E-01
**KRT72**	-0.46	8.59E-01	1.00E+00	-0.92	4.46E-01	8.92E-01
**LSG1**	-0.17	3.29E-01	1.00E+00	-0.12	4.46E-01	8.92E-01
**ZMPSTE24**	0.03	8.74E-01	1.00E+00	-0.17	4.46E-01	8.92E-01
**WDR41**	0.22	2.15E-01	1.00E+00	-0.17	4.46E-01	8.92E-01
**PEX12**	0.18	6.11E-01	1.00E+00	-0.19	4.46E-01	8.92E-01
**AC090844.2**	1.89	1.57E-01	1.00E+00	-0.76	4.46E-01	8.92E-01
**LUC7L**	-0.26	5.09E-01	1.00E+00	0.26	4.46E-01	8.92E-01
**SPOCK3**	-2.75	2.99E-02	9.97E-01	-0.94	4.46E-01	8.92E-01
**ADGRB1**	-0.75	1.45E-01	1.00E+00	0.82	4.46E-01	8.92E-01
**LINC02232**	0.86	4.65E-01	1.00E+00	0.85	4.46E-01	8.92E-01
**SMYD4**	-0.07	7.51E-01	1.00E+00	-0.24	4.46E-01	8.92E-01

**XLOC_004989**	0.75	4.40E-01	1.00E+00	-0.80	4.46E-01	8.92E-01
**AL445933.1**	-0.88	5.94E-02	1.00E+00	-0.77	4.46E-01	8.92E-01
**HIST1H2BC**	-1.25	5.43E-02	1.00E+00	-0.31	4.46E-01	8.92E-01
**MT2P1**	-0.56	8.73E-01	1.00E+00	0.68	4.46E-01	8.92E-01
**DANT2**	0.96	5.39E-01	1.00E+00	-0.62	4.46E-01	8.92E-01
**TNFSF14**	-0.43	5.76E-01	1.00E+00	0.75	4.46E-01	8.92E-01
**MGME1**	0.04	8.89E-01	1.00E+00	0.19	4.46E-01	8.92E-01
**TK1**	-0.55	7.31E-02	1.00E+00	0.25	4.46E-01	8.92E-01
**UNC93B1**	0.01	9.79E-01	1.00E+00	0.75	4.46E-01	8.92E-01
**SV2B**	-0.30	5.24E-01	1.00E+00	-0.51	4.46E-01	8.92E-01
**VPS4B**	0.09	6.56E-01	1.00E+00	-0.17	4.47E-01	8.92E-01
**AP000787.1**	-0.19	7.14E-01	1.00E+00	-0.42	4.47E-01	8.92E-01
**TBC1D23**	0.18	4.52E-01	1.00E+00	-0.17	4.47E-01	8.92E-01
**CCDC85C**	-0.06	8.49E-01	1.00E+00	0.55	4.47E-01	8.92E-01
**ZBTB46**	-0.13	6.70E-01	1.00E+00	0.49	4.47E-01	8.92E-01
**TIGIT**	-0.35	6.90E-01	1.00E+00	2.38	4.47E-01	8.92E-01
**EXOC3L4**	-1.17	9.97E-02	1.00E+00	0.63	4.47E-01	8.92E-01
**KLK5**	-0.05	9.32E-01	1.00E+00	0.49	4.47E-01	8.92E-01
**ZFYVE21**	0.17	3.95E-01	1.00E+00	-0.24	4.47E-01	8.92E-01
**SYT1**	0.01	9.97E-01	1.00E+00	0.44	4.47E-01	8.92E-01
**AC138783.12**	-3.16	7.47E-02	1.00E+00	-0.63	4.47E-01	8.92E-01
**CA8**	1.11	7.49E-02	1.00E+00	-0.54	4.47E-01	8.92E-01
**SPDYE5**	0.10	9.22E-01	1.00E+00	0.49	4.47E-01	8.92E-01
**AL132857.2**	-0.07	9.73E-01	1.00E+00	-0.66	4.47E-01	8.92E-01
**AC107884.1**	-0.73	2.44E-01	1.00E+00	0.53	4.47E-01	8.92E-01
**EIF4EP2**	0.04	9.73E-01	1.00E+00	-0.32	4.47E-01	8.92E-01
**IL27**	-2.81	1.31E-01	1.00E+00	2.40	4.47E-01	8.92E-01
**POLR2L**	-0.11	5.31E-01	1.00E+00	-0.23	4.47E-01	8.92E-01
**C6orf62**	0.33	2.49E-01	1.00E+00	0.27	4.47E-01	8.92E-01
**SMIM10L1**	0.04	8.93E-01	1.00E+00	-0.23	4.47E-01	8.92E-01
**ARHGAP12**	0.08	7.44E-01	1.00E+00	0.16	4.47E-01	8.92E-01
**LINC00884**	-2.43	2.94E-02	9.92E-01	-0.56	4.47E-01	8.92E-01

**AC013400.1**	-1.78	6.06E-01	1.00E+00	1.12	4.48E-01	8.92E-01
**XLOC_006242**	-0.02	9.75E-01	1.00E+00	0.74	4.48E-01	8.92E-01
**AC069282.1**	1.37	6.91E-02	1.00E+00	0.43	4.48E-01	8.92E-01
**BCAP31**	0.16	4.88E-01	1.00E+00	-0.16	4.48E-01	8.92E-01
**HMGB1P1**	-2.51	1.02E-01	1.00E+00	-0.43	4.48E-01	8.92E-01
**AL136531.1**	-2.21	3.45E-01	1.00E+00	0.51	4.48E-01	8.92E-01
**AC026979.3**	0.87	4.83E-01	1.00E+00	0.88	4.48E-01	8.93E-01
**AL928654.4**	0.21	6.17E-01	1.00E+00	0.58	4.48E-01	8.93E-01
**ARMCX5**	0.06	8.05E-01	1.00E+00	-0.20	4.48E-01	8.93E-01
**AL353622.1**	2.46	3.18E-02	1.00E+00	0.82	4.48E-01	8.93E-01
**PI16**	1.96	1.86E-03	2.39E-01	0.50	4.48E-01	8.93E-01
**AC073063.1**	-0.59	7.11E-01	1.00E+00	-0.53	4.48E-01	8.93E-01
**AC017074.1**	-0.38	5.90E-01	1.00E+00	0.80	4.48E-01	8.93E-01
**SRCIN1**	-0.45	2.40E-01	1.00E+00	0.43	4.48E-01	8.93E-01
**G8456**	-0.13	8.90E-01	1.00E+00	0.83	4.48E-01	8.93E-01
**NPL**	-0.33	2.61E-01	1.00E+00	0.31	4.48E-01	8.93E-01
**CHMP1B**	-0.14	6.47E-01	1.00E+00	0.13	4.48E-01	8.93E-01
**STAG3**	-0.94	2.16E-02	8.94E-01	0.31	4.48E-01	8.93E-01
**MTERF4**	0.04	7.77E-01	1.00E+00	0.15	4.48E-01	8.93E-01
**AC074032.1**	0.26	6.27E-01	1.00E+00	-0.31	4.48E-01	8.93E-01
**AL360219.1**	0.50	8.32E-01	1.00E+00	0.66	4.48E-01	8.93E-01
**AF111167.2**	-0.38	4.70E-01	1.00E+00	0.67	4.49E-01	8.93E-01
**PTX3**	0.36	4.45E-01	1.00E+00	0.79	4.49E-01	8.93E-01
**MAN2A2**	0.25	5.32E-01	1.00E+00	0.46	4.49E-01	8.93E-01
**CCNB1IP1**	-0.25	2.93E-01	1.00E+00	-0.19	4.49E-01	8.93E-01
**SYNDIG1L**	0.80	3.71E-01	1.00E+00	-1.03	4.49E-01	8.93E-01
**MRPS36**	-0.01	9.78E-01	1.00E+00	-0.25	4.49E-01	8.93E-01
**ID2-AS1**	-0.23	6.04E-01	1.00E+00	-0.40	4.49E-01	8.93E-01
**AC084757.3**	-1.75	2.35E-01	1.00E+00	1.00	4.49E-01	8.93E-01
**CASC2**	-0.21	7.23E-01	1.00E+00	0.35	4.49E-01	8.93E-01
**RP11-439A17.7**	1.42	4.60E-01	1.00E+00	0.58	4.49E-01	8.93E-01
**CFHR3**	0.90	7.96E-01	1.00E+00	0.53	4.49E-01	8.93E-01

**ADCY6**	0.48	3.37E-01	1.00E+00	0.56	4.49E-01	8.93E-01
**PNMA2**	-0.91	2.27E-01	1.00E+00	0.75	4.49E-01	8.93E-01
**ARRDC5**	-2.47	2.39E-01	1.00E+00	1.38	4.49E-01	8.93E-01
**PHTF1**	0.44	3.38E-01	1.00E+00	0.49	4.49E-01	8.93E-01
**SERGEF**	-0.09	7.70E-01	1.00E+00	-0.18	4.49E-01	8.93E-01
**GPM6B**	-0.29	4.25E-01	1.00E+00	0.18	4.49E-01	8.93E-01
**TRAV38-2DV8**	0.37	8.75E-01	1.00E+00	1.00	4.49E-01	8.93E-01
**RAB39B**	-1.30	1.48E-01	1.00E+00	0.84	4.49E-01	8.93E-01
**POLE3**	0.04	8.57E-01	1.00E+00	-0.14	4.49E-01	8.93E-01
**AC012184.3**	0.56	5.31E-01	1.00E+00	-0.72	4.49E-01	8.93E-01
**HARS**	-0.03	8.71E-01	1.00E+00	0.13	4.49E-01	8.93E-01
**SNRPGP10**	0.03	9.59E-01	1.00E+00	-0.36	4.50E-01	8.93E-01
**ZNF226**	0.14	4.32E-01	1.00E+00	-0.21	4.50E-01	8.93E-01
**AL023806.1**	0.30	8.76E-01	1.00E+00	-1.07	4.50E-01	8.93E-01
**RNF11**	0.13	5.49E-01	1.00E+00	-0.23	4.50E-01	8.93E-01
**RPS6KB1**	-0.04	8.41E-01	1.00E+00	0.13	4.50E-01	8.94E-01
**CD3E**	-1.06	5.93E-02	1.00E+00	2.03	4.50E-01	8.94E-01
**POM121**	-0.34	1.42E-01	1.00E+00	0.50	4.50E-01	8.94E-01
**FAM86FP**	0.23	6.88E-01	1.00E+00	0.39	4.50E-01	8.94E-01
**CACNA1D**	-0.01	9.79E-01	1.00E+00	-0.53	4.50E-01	8.94E-01
**TPM1**	-0.02	9.68E-01	1.00E+00	-0.24	4.50E-01	8.94E-01
**ZNF10**	0.28	4.71E-01	1.00E+00	-0.41	4.50E-01	8.94E-01
**DHX30**	-0.17	3.61E-01	1.00E+00	0.17	4.50E-01	8.94E-01
**SF3B4**	-0.33	1.44E-01	1.00E+00	0.15	4.50E-01	8.94E-01
**XLOC_001162**	-0.17	9.28E-01	1.00E+00	0.94	4.50E-01	8.94E-01
**SNX2**	-0.04	8.45E-01	1.00E+00	-0.21	4.50E-01	8.94E-01
**TMEM131L**	0.21	5.65E-01	1.00E+00	0.20	4.50E-01	8.94E-01
**IGHGP**	2.35	4.24E-02	1.00E+00	-0.73	4.50E-01	8.94E-01
**GJA4**	0.14	7.84E-01	1.00E+00	0.42	4.51E-01	8.94E-01
**LANCL1**	-0.05	8.52E-01	1.00E+00	-0.22	4.51E-01	8.94E-01
**SCARNA9**	0.44	7.12E-01	1.00E+00	0.41	4.51E-01	8.94E-01
**TIMM17A**	0.05	8.27E-01	1.00E+00	-0.20	4.51E-01	8.94E-01

**AC023043.4**	-0.31	7.84E-01	1.00E+00	0.68	4.51E-01	8.94E-01
**SMARCAD1**	-0.02	9.45E-01	1.00E+00	0.21	4.51E-01	8.94E-01
**TRERF1**	-0.47	1.87E-01	1.00E+00	0.36	4.51E-01	8.94E-01
**SNRPCP3**	-2.23	4.84E-01	1.00E+00	-0.83	4.51E-01	8.94E-01
**ZNF287**	-0.22	2.98E-01	1.00E+00	-0.26	4.51E-01	8.94E-01
**AL358115.1**	0.38	8.75E-01	1.00E+00	-1.06	4.51E-01	8.94E-01
**POGLUT1**	0.22	4.25E-01	1.00E+00	-0.17	4.51E-01	8.94E-01
**G16245**	-1.32	6.50E-01	1.00E+00	1.37	4.51E-01	8.94E-01
**OXCT2P1**	-0.64	5.59E-01	1.00E+00	0.73	4.51E-01	8.94E-01
**HNRNPUL1**	-0.20	3.97E-01	1.00E+00	0.28	4.51E-01	8.94E-01
**MT-ND4**	0.03	9.49E-01	1.00E+00	-0.28	4.51E-01	8.94E-01
**SKIL**	-0.01	9.80E-01	1.00E+00	0.22	4.51E-01	8.94E-01
**UHRF1**	-0.93	1.41E-02	7.46E-01	0.37	4.51E-01	8.94E-01
**CDC20P1**	0.26	7.38E-01	1.00E+00	-0.41	4.51E-01	8.94E-01
**AC009120.1**	-0.24	8.71E-01	1.00E+00	0.86	4.51E-01	8.94E-01
**AMZ2P1**	0.24	4.53E-01	1.00E+00	-0.35	4.51E-01	8.94E-01
**ZNF552**	0.48	1.03E-01	1.00E+00	0.23	4.51E-01	8.94E-01
**ARPC5L**	-0.23	4.98E-01	1.00E+00	-0.25	4.51E-01	8.94E-01
**AC026356.1**	-0.37	7.13E-01	1.00E+00	0.77	4.51E-01	8.94E-01
**TPT1P6**	-1.52	6.60E-01	1.00E+00	-0.72	4.52E-01	8.94E-01
**AL157400.4**	-1.19	4.74E-01	1.00E+00	-1.01	4.52E-01	8.94E-01
**XLOC_008530**	-1.49	1.08E-01	1.00E+00	-0.89	4.52E-01	8.94E-01
**TXNRD2**	0.02	9.45E-01	1.00E+00	0.31	4.52E-01	8.94E-01
**ZWILCH**	-0.42	1.25E-01	1.00E+00	0.18	4.52E-01	8.94E-01
**TBL2**	-0.03	8.89E-01	1.00E+00	-0.18	4.52E-01	8.94E-01
**XLOC_009526**	-0.59	6.40E-01	1.00E+00	-1.07	4.52E-01	8.95E-01
**KIF23**	0.22	5.17E-01	1.00E+00	0.33	4.52E-01	8.95E-01
**SMG1**	-0.08	7.65E-01	1.00E+00	0.32	4.52E-01	8.95E-01
**HLA-DRB1**	-0.01	9.87E-01	1.00E+00	0.33	4.52E-01	8.95E-01
**FAP**	0.15	8.34E-01	1.00E+00	1.81	4.52E-01	8.95E-01
**MIR600HG**	0.01	9.89E-01	1.00E+00	-0.64	4.52E-01	8.95E-01
**NEB**	-0.57	2.77E-01	1.00E+00	0.49	4.52E-01	8.95E-01

**AC138207.1**	1.12	5.64E-01	1.00E+00	-0.94	4.52E-01	8.95E-01
**G37230**	-1.88	6.20E-02	1.00E+00	0.59	4.52E-01	8.95E-01
**ZC3H8**	0.03	9.34E-01	1.00E+00	-0.22	4.52E-01	8.95E-01
**MIER3**	0.17	6.55E-01	1.00E+00	0.22	4.52E-01	8.95E-01
**KLHDC9**	0.25	6.94E-01	1.00E+00	-0.41	4.52E-01	8.95E-01
**ZNF649**	0.06	8.20E-01	1.00E+00	-0.17	4.53E-01	8.95E-01
**TSPAN8**	0.73	2.98E-01	1.00E+00	-0.53	4.53E-01	8.95E-01
**SRSF11**	0.18	3.97E-01	1.00E+00	-0.12	4.53E-01	8.95E-01
**PIGBOS1**	0.03	9.04E-01	1.00E+00	-0.23	4.53E-01	8.95E-01
**AP001636.2**	-2.96	7.12E-02	1.00E+00	-0.72	4.53E-01	8.95E-01
**FUT7**	0.03	9.76E-01	1.00E+00	0.74	4.53E-01	8.95E-01
**AC104109.4**	-1.08	5.13E-01	1.00E+00	0.65	4.53E-01	8.95E-01
**SNCG**	0.60	3.01E-01	1.00E+00	-0.35	4.53E-01	8.95E-01
**KLHL42**	-0.13	4.39E-01	1.00E+00	-0.22	4.53E-01	8.95E-01
**AP000704.1**	-0.61	8.55E-01	1.00E+00	-0.62	4.53E-01	8.95E-01
**AC140479.5**	-0.57	6.53E-01	1.00E+00	-0.34	4.53E-01	8.95E-01
**XKR4**	0.23	6.18E-01	1.00E+00	-0.62	4.53E-01	8.95E-01
**SLC22A23**	-0.43	2.42E-01	1.00E+00	0.36	4.53E-01	8.95E-01
**DNAH2**	-1.21	2.59E-01	1.00E+00	-0.72	4.53E-01	8.95E-01
**AP003501.3**	0.36	7.62E-01	1.00E+00	-0.90	4.53E-01	8.95E-01
**MYZAP**	1.14	5.77E-02	1.00E+00	-0.34	4.53E-01	8.95E-01
**SLC10A6**	-0.21	6.74E-01	1.00E+00	0.31	4.53E-01	8.95E-01
**PCMTD2**	0.09	6.99E-01	1.00E+00	-0.20	4.53E-01	8.95E-01
**MMP15**	-0.22	4.37E-01	1.00E+00	0.51	4.53E-01	8.95E-01
**CCT6B**	0.51	1.51E-01	1.00E+00	-0.35	4.53E-01	8.95E-01
**AC016597.1**	1.52	4.97E-01	1.00E+00	0.93	4.53E-01	8.95E-01
**FAM217B**	-0.01	9.70E-01	1.00E+00	0.15	4.54E-01	8.95E-01
**LIN54**	0.01	9.69E-01	1.00E+00	-0.27	4.54E-01	8.95E-01
**RNF6**	0.00	9.94E-01	1.00E+00	-0.18	4.54E-01	8.95E-01
**RPS19P1**	-0.58	8.67E-01	1.00E+00	-0.55	4.54E-01	8.95E-01
**AC096733.2**	0.12	8.26E-01	1.00E+00	0.55	4.54E-01	8.95E-01
**SOHLH1**	-2.79	5.39E-02	1.00E+00	-1.11	4.54E-01	8.95E-01

**RASGRP2**	-0.30	5.48E-01	1.00E+00	0.44	4.54E-01	8.95E-01
**IGSF10**	0.49	2.91E-01	1.00E+00	0.52	4.54E-01	8.95E-01
**NUMB**	-0.06	7.54E-01	1.00E+00	0.14	4.54E-01	8.95E-01
**KLRF2**	-0.28	7.81E-01	1.00E+00	-0.56	4.54E-01	8.95E-01
**AP001363.1**	0.55	5.44E-01	1.00E+00	0.61	4.54E-01	8.95E-01
**PPP4R2**	0.19	4.98E-01	1.00E+00	-0.33	4.54E-01	8.95E-01
**DEGS1**	-0.02	9.62E-01	1.00E+00	-0.37	4.54E-01	8.95E-01
**CD28**	1.27	1.26E-01	1.00E+00	0.72	4.54E-01	8.95E-01
**TRPC6**	-0.15	6.76E-01	1.00E+00	0.34	4.54E-01	8.95E-01
**AP003774.1**	-1.14	2.99E-01	1.00E+00	1.16	4.54E-01	8.95E-01
**ADCY8**	0.73	5.94E-01	1.00E+00	-0.65	4.54E-01	8.95E-01
**SLC9A5**	-0.80	5.96E-02	1.00E+00	0.47	4.54E-01	8.95E-01
**AC009032.1**	-1.09	6.50E-01	1.00E+00	-0.79	4.54E-01	8.95E-01
**AGAP3**	-0.10	7.06E-01	1.00E+00	0.40	4.54E-01	8.95E-01
**CHORDC1**	-0.20	7.12E-01	1.00E+00	-0.24	4.54E-01	8.95E-01
**SFR1**	0.34	2.89E-01	1.00E+00	-0.29	4.54E-01	8.95E-01
**AC091152.4**	-0.87	4.97E-01	1.00E+00	0.96	4.54E-01	8.95E-01
**MYO1B**	-0.19	3.98E-01	1.00E+00	0.41	4.54E-01	8.95E-01
**G31765**	1.48	3.51E-02	1.00E+00	-0.72	4.54E-01	8.95E-01
**AC095055.1**	-0.74	1.91E-01	1.00E+00	-0.47	4.54E-01	8.95E-01
**DNAJC15**	0.10	6.71E-01	1.00E+00	-0.20	4.54E-01	8.95E-01
**EDN1**	-0.04	9.06E-01	1.00E+00	-0.36	4.54E-01	8.95E-01
**ARSF**	-0.86	1.60E-01	1.00E+00	0.56	4.54E-01	8.95E-01
**PADI4**	-1.17	7.28E-01	1.00E+00	0.76	4.54E-01	8.95E-01
**AL121871.1**	-0.67	8.47E-01	1.00E+00	-0.61	4.54E-01	8.95E-01
**KY**	0.15	7.21E-01	1.00E+00	-0.47	4.54E-01	8.95E-01
**AC007000.1**	-2.52	4.93E-02	1.00E+00	0.61	4.54E-01	8.95E-01
**S100A16**	-0.17	6.25E-01	1.00E+00	0.21	4.55E-01	8.95E-01
**CFH**	0.67	1.21E-01	1.00E+00	0.41	4.55E-01	8.95E-01
**PSMA2**	0.17	6.69E-01	1.00E+00	-0.34	4.55E-01	8.95E-01
**MLF1**	0.03	9.29E-01	1.00E+00	-0.35	4.55E-01	8.95E-01
**FTSJ1**	-0.25	3.36E-01	1.00E+00	0.12	4.55E-01	8.95E-01

**EIF4G1**	-0.31	2.33E-01	1.00E+00	0.36	4.55E-01	8.95E-01
**ARCN1**	-0.01	9.55E-01	1.00E+00	-0.22	4.55E-01	8.95E-01
**SEC22A**	0.09	6.94E-01	1.00E+00	-0.19	4.55E-01	8.95E-01
**TPSAB1**	0.80	2.00E-01	1.00E+00	0.40	4.55E-01	8.95E-01
**AC106897.1**	0.72	7.87E-01	1.00E+00	-0.54	4.55E-01	8.96E-01
**ADAMTS7P3**	0.43	7.55E-01	1.00E+00	-0.38	4.55E-01	8.96E-01
**RNF4**	-0.01	9.52E-01	1.00E+00	0.11	4.55E-01	8.96E-01
**RPL22L1**	-0.20	4.34E-01	1.00E+00	0.24	4.55E-01	8.96E-01
**WDR7**	-0.05	7.73E-01	1.00E+00	0.25	4.55E-01	8.96E-01
**G43235**	0.80	3.36E-01	1.00E+00	0.71	4.56E-01	8.96E-01
**MCM8**	-0.28	2.74E-01	1.00E+00	0.36	4.56E-01	8.96E-01
**AL356740.3**	0.02	9.86E-01	1.00E+00	-0.61	4.56E-01	8.96E-01
**AC104211.1**	-0.90	7.87E-01	1.00E+00	-1.01	4.56E-01	8.96E-01
**KIR2DL4**	-3.59	2.77E-01	1.00E+00	1.06	4.56E-01	8.96E-01
**AL161911.1**	-1.92	1.06E-01	1.00E+00	-0.93	4.56E-01	8.96E-01
**HOXA-AS3**	-0.88	3.55E-01	1.00E+00	-0.45	4.56E-01	8.96E-01
**NRDE2**	0.04	8.77E-01	1.00E+00	0.28	4.56E-01	8.96E-01
**SEPT7P6**	-0.06	9.86E-01	1.00E+00	-0.48	4.56E-01	8.96E-01
**XLOC_001640**	0.45	7.71E-01	1.00E+00	-0.54	4.56E-01	8.96E-01
**ZNHIT3**	0.46	7.39E-02	1.00E+00	0.15	4.56E-01	8.96E-01
**AL590452.1**	-0.60	6.99E-01	1.00E+00	0.79	4.56E-01	8.96E-01
**INTS6L**	0.36	2.45E-01	1.00E+00	0.31	4.56E-01	8.96E-01
**KRT18P59**	1.52	3.14E-01	1.00E+00	0.99	4.56E-01	8.96E-01
**PPP2R2C**	-0.06	8.58E-01	1.00E+00	0.51	4.56E-01	8.96E-01
**RN7SL336P**	2.83	3.71E-02	1.00E+00	0.62	4.56E-01	8.96E-01
**PHETA1**	-0.07	8.23E-01	1.00E+00	0.47	4.56E-01	8.96E-01
**NODAL**	-2.84	1.14E-01	1.00E+00	1.02	4.56E-01	8.96E-01
**ATXN7L2**	-0.01	9.66E-01	1.00E+00	0.34	4.56E-01	8.96E-01
**KMT5A**	-0.04	8.93E-01	1.00E+00	0.20	4.56E-01	8.96E-01
**AL031432.3**	0.66	4.49E-01	1.00E+00	0.40	4.56E-01	8.96E-01
**WDR83**	0.08	8.84E-01	1.00E+00	0.32	4.57E-01	8.96E-01
**ZNF598**	-0.44	2.02E-01	1.00E+00	0.53	4.57E-01	8.96E-01

**ZNF784**	-0.14	6.58E-01	1.00E+00	0.59	4.57E-01	8.96E-01
**AC008393.1**	-0.95	5.55E-02	1.00E+00	-0.51	4.57E-01	8.96E-01
**AC006262.1**	-0.09	8.94E-01	1.00E+00	0.63	4.57E-01	8.96E-01
**AC022211.2**	-0.76	1.10E-01	1.00E+00	0.54	4.57E-01	8.96E-01
**AL606970.3**	-2.95	3.06E-01	1.00E+00	1.28	4.57E-01	8.96E-01
**ZNF224**	0.41	4.47E-01	1.00E+00	-0.26	4.57E-01	8.96E-01
**G26828**	0.81	3.79E-01	1.00E+00	0.70	4.57E-01	8.96E-01
**CHCHD2P9**	-1.77	2.03E-01	1.00E+00	0.54	4.57E-01	8.96E-01
**PLPPR1**	0.48	6.65E-01	1.00E+00	-0.70	4.57E-01	8.96E-01
**TMEM38A**	-0.02	9.75E-01	1.00E+00	-0.38	4.57E-01	8.96E-01
**RNF207**	-0.03	9.62E-01	1.00E+00	0.58	4.57E-01	8.96E-01
**RHBDL2**	0.26	5.35E-01	1.00E+00	-0.36	4.57E-01	8.96E-01
**AC105206.2**	-2.36	7.86E-02	1.00E+00	1.03	4.57E-01	8.96E-01
**SCO2**	-0.17	7.19E-01	1.00E+00	0.23	4.57E-01	8.96E-01
**ABCB7**	0.10	6.21E-01	1.00E+00	0.17	4.57E-01	8.96E-01
**G37568**	-1.34	3.52E-01	1.00E+00	0.90	4.57E-01	8.96E-01
**CALHM6**	-0.86	2.07E-01	1.00E+00	2.33	4.57E-01	8.96E-01
**SLC6A1**	0.66	4.06E-01	1.00E+00	0.89	4.57E-01	8.96E-01
**XLOC_010885**	-0.06	9.33E-01	1.00E+00	-0.37	4.57E-01	8.96E-01
**AC007485.2**	-0.83	2.51E-01	1.00E+00	0.39	4.57E-01	8.96E-01
**LINC01320**	0.10	9.23E-01	1.00E+00	-0.68	4.57E-01	8.96E-01
**CAMKK2**	0.04	8.54E-01	1.00E+00	0.23	4.57E-01	8.96E-01
**AC010913.1**	0.03	9.63E-01	1.00E+00	-0.50	4.57E-01	8.96E-01
**BCL7A**	-0.11	5.11E-01	1.00E+00	0.22	4.57E-01	8.96E-01
**NCLN**	-0.22	3.45E-01	1.00E+00	0.40	4.57E-01	8.96E-01
**LRAT**	0.46	5.67E-01	1.00E+00	2.25	4.57E-01	8.96E-01
**PAFAH2**	0.24	2.09E-01	1.00E+00	-0.17	4.57E-01	8.96E-01
**LINC01852**	0.88	1.98E-01	1.00E+00	-0.55	4.57E-01	8.96E-01
**THAP7**	-0.10	6.40E-01	1.00E+00	0.17	4.57E-01	8.96E-01
**TXNP6**	2.12	5.34E-01	1.00E+00	-0.51	4.57E-01	8.96E-01
**AL136366.1**	-1.12	2.11E-01	1.00E+00	-0.76	4.58E-01	8.96E-01
**RPS10P7**	-0.28	7.43E-01	1.00E+00	0.42	4.58E-01	8.96E-01

**POLD1**	-0.19	4.00E-01	1.00E+00	0.42	4.58E-01	8.96E-01
**AL355472.1**	0.32	5.87E-01	1.00E+00	-0.29	4.58E-01	8.96E-01
**GBA2**	0.20	4.82E-01	1.00E+00	0.28	4.58E-01	8.96E-01
**GNPTAB**	0.12	6.35E-01	1.00E+00	-0.21	4.58E-01	8.96E-01
**GRM1**	-0.78	5.80E-01	1.00E+00	1.11	4.58E-01	8.96E-01
**MS4A6E**	-1.71	6.18E-01	1.00E+00	-1.39	4.58E-01	8.96E-01
**LY6G6F-LY6G6D**	0.08	9.57E-01	1.00E+00	-0.96	4.58E-01	8.96E-01
**SPC25**	-0.07	8.66E-01	1.00E+00	0.40	4.58E-01	8.96E-01
**BCCIP**	0.10	5.67E-01	1.00E+00	-0.20	4.58E-01	8.96E-01
**AC115618.3**	-0.37	3.06E-01	1.00E+00	0.33	4.58E-01	8.96E-01
**MTOR**	-0.14	4.86E-01	1.00E+00	0.25	4.58E-01	8.96E-01
**KCND1**	-0.15	7.67E-01	1.00E+00	0.73	4.58E-01	8.96E-01
**FRMPD3**	-2.73	1.20E-02	6.78E-01	1.04	4.58E-01	8.96E-01
**AC097103.2**	-0.83	1.19E-01	1.00E+00	-0.26	4.58E-01	8.96E-01
**MGARP**	0.00	9.99E-01	1.00E+00	0.60	4.58E-01	8.96E-01
**THOC6**	-0.24	3.78E-01	1.00E+00	0.15	4.58E-01	8.96E-01
**AL645728.1**	-0.32	6.02E-01	1.00E+00	0.39	4.58E-01	8.96E-01
**MRPL15**	-0.02	9.19E-01	1.00E+00	-0.22	4.58E-01	8.96E-01
**STS**	0.61	4.84E-02	1.00E+00	0.28	4.58E-01	8.96E-01
**CASP9**	-0.01	9.78E-01	1.00E+00	0.29	4.58E-01	8.96E-01
**POP4**	-0.09	6.26E-01	1.00E+00	-0.19	4.58E-01	8.96E-01
**GNE**	0.14	7.82E-01	1.00E+00	0.42	4.58E-01	8.96E-01
**TTI2**	-0.10	7.21E-01	1.00E+00	-0.19	4.58E-01	8.96E-01
**AP003117.1**	-0.50	6.89E-01	1.00E+00	0.71	4.58E-01	8.96E-01
**RAB11A**	0.06	8.51E-01	1.00E+00	-0.19	4.58E-01	8.96E-01
**GANC**	0.04	8.63E-01	1.00E+00	0.18	4.58E-01	8.96E-01
**WAPL**	-0.11	6.92E-01	1.00E+00	-0.22	4.59E-01	8.96E-01
**LINC00607**	-1.60	1.40E-01	1.00E+00	0.89	4.59E-01	8.96E-01
**AL512844.1**	-0.67	8.48E-01	1.00E+00	-0.53	4.59E-01	8.96E-01
**AC109322.1**	-0.49	4.48E-01	1.00E+00	-0.35	4.59E-01	8.96E-01
**G12085**	-2.28	2.10E-01	1.00E+00	0.70	4.59E-01	8.96E-01
**FREM2**	-1.00	2.18E-01	1.00E+00	-0.66	4.59E-01	8.96E-01

**AC009812.3**	-0.69	6.37E-01	1.00E+00	-0.82	4.59E-01	8.96E-01
**FAM169B**	1.09	5.71E-01	1.00E+00	0.76	4.59E-01	8.96E-01
**MCFD2**	0.22	3.42E-01	1.00E+00	0.17	4.59E-01	8.96E-01
**SLC16A6**	-0.65	3.69E-01	1.00E+00	0.49	4.59E-01	8.96E-01
**AC005972.3**	-1.03	1.18E-01	1.00E+00	1.01	4.59E-01	8.96E-01
**RNF20**	0.17	4.00E-01	1.00E+00	-0.10	4.59E-01	8.96E-01
**AL356488.3**	-0.51	2.48E-01	1.00E+00	-0.32	4.59E-01	8.96E-01
**XLOC_000166**	3.41	3.13E-01	1.00E+00	0.97	4.59E-01	8.96E-01
**ERP44**	-0.19	1.69E-01	1.00E+00	-0.15	4.59E-01	8.96E-01
**WHAMMP3**	0.37	5.77E-01	1.00E+00	0.56	4.59E-01	8.96E-01
**KRT8P26**	0.13	8.26E-01	1.00E+00	0.73	4.59E-01	8.96E-01
**RING1**	-0.35	9.90E-02	1.00E+00	0.45	4.59E-01	8.96E-01
**G36840**	0.27	7.87E-01	1.00E+00	-0.94	4.59E-01	8.96E-01
**Y_RNA**	0.26	5.34E-01	1.00E+00	0.16	4.59E-01	8.96E-01
**XLOC_005181**	0.24	8.06E-01	1.00E+00	0.64	4.59E-01	8.96E-01
**LRRC49**	0.12	7.11E-01	1.00E+00	-0.24	4.59E-01	8.96E-01
**BCL2L13**	0.11	5.34E-01	1.00E+00	0.11	4.59E-01	8.96E-01
**TTC4**	0.37	6.38E-01	1.00E+00	0.43	4.59E-01	8.96E-01
**XLOC_013072**	-0.37	9.16E-01	1.00E+00	0.68	4.60E-01	8.96E-01
**LARP4**	-0.16	6.28E-01	1.00E+00	-0.16	4.60E-01	8.96E-01
**RAMP3**	-0.49	3.63E-01	1.00E+00	0.34	4.60E-01	8.96E-01
**AC079760.2**	-2.63	1.11E-01	1.00E+00	-0.77	4.60E-01	8.96E-01
**KCMF1**	0.05	7.62E-01	1.00E+00	-0.32	4.60E-01	8.96E-01
**PCDHB3**	0.38	3.93E-01	1.00E+00	-0.40	4.60E-01	8.96E-01
**CDHR1**	0.06	8.72E-01	1.00E+00	-0.54	4.60E-01	8.96E-01
**HSPA9**	-0.15	2.90E-01	1.00E+00	-0.10	4.60E-01	8.96E-01
**CDKAL1**	-0.14	5.11E-01	1.00E+00	-0.19	4.60E-01	8.96E-01
**FARSB**	-0.26	2.20E-01	1.00E+00	-0.18	4.60E-01	8.97E-01
**SRGAP3**	0.02	9.47E-01	1.00E+00	0.33	4.60E-01	8.97E-01
**MIR589**	1.30	3.02E-01	1.00E+00	0.83	4.60E-01	8.97E-01
**AC105233.5**	-0.62	2.06E-01	1.00E+00	-0.48	4.60E-01	8.97E-01
**WFS1**	-0.10	6.40E-01	1.00E+00	-0.29	4.60E-01	8.97E-01

**INTS14**	0.22	3.19E-01	1.00E+00	-0.13	4.60E-01	8.97E-01
**MFNG**	0.38	4.49E-01	1.00E+00	0.42	4.60E-01	8.97E-01
**RIOK1**	-0.07	7.00E-01	1.00E+00	-0.17	4.60E-01	8.97E-01
**CHRNA10**	-0.96	4.68E-01	1.00E+00	1.30	4.61E-01	8.97E-01
**AMER1**	-0.38	1.73E-01	1.00E+00	0.57	4.61E-01	8.97E-01
**KIF9**	0.03	9.24E-01	1.00E+00	-0.23	4.61E-01	8.97E-01
**SCN2B**	0.25	7.30E-01	1.00E+00	0.51	4.61E-01	8.97E-01
**NPM1P39**	0.12	9.12E-01	1.00E+00	-0.36	4.61E-01	8.97E-01
**SECISBP2**	0.22	2.76E-01	1.00E+00	0.14	4.61E-01	8.97E-01
**NOMO3**	-0.46	2.42E-01	1.00E+00	-1.91	4.61E-01	8.97E-01
**MRM3**	-0.11	6.10E-01	1.00E+00	-0.18	4.61E-01	8.97E-01
**PAX3**	-0.03	9.48E-01	1.00E+00	-0.40	4.61E-01	8.97E-01
**MEF2D**	-0.19	4.46E-01	1.00E+00	0.44	4.61E-01	8.97E-01
**NUDT8**	0.03	9.29E-01	1.00E+00	-0.23	4.61E-01	8.97E-01
**RNU1-120P**	-0.74	7.77E-01	1.00E+00	-0.85	4.61E-01	8.97E-01
**MBNL1**	0.06	7.72E-01	1.00E+00	-0.10	4.61E-01	8.97E-01
**PITPNM3**	0.15	6.62E-01	1.00E+00	-0.43	4.61E-01	8.97E-01
**BCAN**	-1.04	4.77E-02	1.00E+00	-0.50	4.61E-01	8.97E-01
**AL139260.1**	-0.18	8.16E-01	1.00E+00	-0.48	4.61E-01	8.97E-01
**XLOC_007879**	1.03	4.91E-01	1.00E+00	-0.85	4.61E-01	8.97E-01
**PRODH**	-0.01	9.78E-01	1.00E+00	-0.38	4.61E-01	8.97E-01
**RANBP2**	0.04	8.34E-01	1.00E+00	0.24	4.61E-01	8.97E-01
**C1orf131**	0.00	9.99E-01	1.00E+00	-0.20	4.61E-01	8.97E-01
**ZNF300**	0.19	5.04E-01	1.00E+00	-0.41	4.61E-01	8.97E-01
**AC026333.4**	1.35	6.94E-01	1.00E+00	-1.58	4.61E-01	8.97E-01
**RPL10P15**	0.30	8.82E-01	1.00E+00	0.47	4.61E-01	8.97E-01
**BAALC**	0.01	9.80E-01	1.00E+00	-0.41	4.61E-01	8.97E-01
**PITPNB**	0.10	5.57E-01	1.00E+00	-0.14	4.61E-01	8.97E-01
**C22orf31**	-0.35	6.79E-01	1.00E+00	0.53	4.62E-01	8.97E-01
**CYP51A1-AS1**	-1.22	2.55E-01	1.00E+00	0.62	4.62E-01	8.97E-01
**SRM**	-0.34	1.47E-01	1.00E+00	-0.44	4.62E-01	8.97E-01
**ATP5PBP1**	-0.06	9.86E-01	1.00E+00	-1.06	4.62E-01	8.97E-01

**RNVU1-13**	-3.75	6.86E-02	1.00E+00	-0.71	4.62E-01	8.97E-01
**AC007240.1**	-1.96	1.58E-01	1.00E+00	0.80	4.62E-01	8.97E-01
**ACVR2A**	0.20	4.20E-01	1.00E+00	-0.29	4.62E-01	8.97E-01
**SEMA5B**	-0.39	4.02E-01	1.00E+00	0.56	4.62E-01	8.97E-01
**BX322562.1**	0.03	9.62E-01	1.00E+00	-0.57	4.62E-01	8.97E-01
**AL356019.2**	0.12	8.89E-01	1.00E+00	0.68	4.62E-01	8.97E-01
**MOB4**	0.02	9.49E-01	1.00E+00	-0.22	4.62E-01	8.97E-01
**TRPM4**	-0.30	2.66E-01	1.00E+00	-0.36	4.62E-01	8.97E-01
**KATNBL1P6**	-0.39	9.10E-01	1.00E+00	-0.47	4.62E-01	8.97E-01
**CDC42**	0.26	7.21E-01	1.00E+00	-0.31	4.62E-01	8.97E-01
**TMED6**	0.51	5.76E-01	1.00E+00	-0.68	4.62E-01	8.97E-01
**LNPK**	0.36	8.88E-02	1.00E+00	-0.21	4.62E-01	8.97E-01
**AL136531.2**	-3.18	1.16E-01	1.00E+00	1.31	4.62E-01	8.97E-01
**AC026979.2**	-0.03	9.51E-01	1.00E+00	-0.29	4.62E-01	8.97E-01
**G3694**	-1.83	5.01E-01	1.00E+00	0.83	4.62E-01	8.97E-01
**AC139099.2**	-1.54	1.39E-01	1.00E+00	0.73	4.62E-01	8.97E-01
**AP003068.2**	-0.52	2.99E-01	1.00E+00	-0.46	4.62E-01	8.97E-01
**CA3-AS1**	-0.37	5.10E-01	1.00E+00	-0.49	4.62E-01	8.97E-01
**KIAA0408**	-1.95	5.71E-01	1.00E+00	-0.76	4.62E-01	8.97E-01
**PRR7-AS1**	-2.05	1.40E-01	1.00E+00	0.91	4.62E-01	8.97E-01
**PABPN1**	-0.07	7.58E-01	1.00E+00	0.24	4.62E-01	8.97E-01
**RPL26**	-0.04	8.93E-01	1.00E+00	-0.44	4.62E-01	8.97E-01
**SMYD3**	0.01	9.63E-01	1.00E+00	-0.21	4.63E-01	8.97E-01
**XLOC_000917**	0.17	6.88E-01	1.00E+00	-0.70	4.63E-01	8.97E-01
**USP8**	0.08	6.17E-01	1.00E+00	0.12	4.63E-01	8.97E-01
**RETSAT**	0.83	4.15E-02	1.00E+00	-0.34	4.63E-01	8.97E-01
**NDUFA8**	-0.01	9.68E-01	1.00E+00	-0.16	4.63E-01	8.97E-01
**MIR155HG**	-0.25	7.57E-01	1.00E+00	0.60	4.63E-01	8.97E-01
**FHL1**	1.15	4.50E-02	1.00E+00	-0.42	4.63E-01	8.98E-01
**XLOC_012064**	-0.46	7.81E-01	1.00E+00	-0.92	4.63E-01	8.98E-01
**TMEM126A**	-0.04	8.34E-01	1.00E+00	-0.21	4.63E-01	8.98E-01
**S100A14**	-0.05	8.90E-01	1.00E+00	0.47	4.63E-01	8.98E-01

**APOL3**	-0.02	9.74E-01	1.00E+00	0.52	4.63E-01	8.98E-01
**CRYM-AS1**	-1.29	2.84E-01	1.00E+00	-0.56	4.63E-01	8.98E-01
**AP002807.1**	-0.15	9.17E-01	1.00E+00	1.32	4.63E-01	8.98E-01
**ZMIZ2**	-0.28	2.50E-01	1.00E+00	0.41	4.63E-01	8.98E-01
**MC1R**	1.81	9.88E-02	1.00E+00	0.77	4.63E-01	8.98E-01
**LRRK2**	-0.07	8.80E-01	1.00E+00	0.41	4.64E-01	8.98E-01
**LYSMD4**	-0.22	4.60E-01	1.00E+00	-0.21	4.64E-01	8.98E-01
**SCMH1**	0.32	4.13E-01	1.00E+00	0.27	4.64E-01	8.98E-01
**USP33**	0.36	1.66E-01	1.00E+00	-0.20	4.64E-01	8.98E-01
**FLJ21408**	-0.50	7.16E-01	1.00E+00	0.57	4.64E-01	8.98E-01
**G5758**	-1.17	3.93E-01	1.00E+00	-1.03	4.64E-01	8.98E-01
**GPKOW**	-0.10	6.53E-01	1.00E+00	0.09	4.64E-01	8.98E-01
**GRB10**	0.17	6.86E-01	1.00E+00	0.51	4.64E-01	8.98E-01
**CIRBP-AS1**	-0.77	4.48E-01	1.00E+00	0.70	4.64E-01	8.98E-01
**CTBS**	0.05	8.64E-01	1.00E+00	-0.24	4.64E-01	8.98E-01
**HTRA1**	0.44	2.40E-01	1.00E+00	0.44	4.64E-01	8.98E-01
**CDKL5**	-0.30	3.38E-01	1.00E+00	0.30	4.64E-01	8.98E-01
**AC092045.1**	-1.00	7.70E-01	1.00E+00	0.52	4.64E-01	8.98E-01
**DMAP1**	0.03	8.97E-01	1.00E+00	0.12	4.64E-01	8.98E-01
**DDX19B**	0.03	8.87E-01	1.00E+00	-0.10	4.64E-01	8.98E-01
**CAPN3**	0.52	7.45E-01	1.00E+00	0.52	4.64E-01	8.98E-01
**RPN1**	-0.03	8.14E-01	1.00E+00	0.13	4.64E-01	8.98E-01
**HCG15**	-1.71	3.22E-01	1.00E+00	-0.81	4.64E-01	8.98E-01
**RRAS**	0.25	5.42E-01	1.00E+00	-0.41	4.64E-01	8.98E-01
**CPS1**	0.44	1.70E-01	1.00E+00	0.40	4.64E-01	8.98E-01
**ERBB3**	0.09	7.89E-01	1.00E+00	-0.36	4.64E-01	8.98E-01
**DNAJC3-DT**	-0.06	8.78E-01	1.00E+00	-0.25	4.64E-01	8.98E-01
**TMEM79**	-0.27	5.73E-01	1.00E+00	0.36	4.64E-01	8.98E-01
**SNX21**	-0.07	8.50E-01	1.00E+00	-0.22	4.64E-01	8.98E-01
**GNG10**	0.24	8.41E-01	1.00E+00	0.52	4.65E-01	8.98E-01
**NPR2**	0.65	2.25E-01	1.00E+00	0.47	4.65E-01	8.98E-01
**SNRPE**	-0.07	6.36E-01	1.00E+00	-0.24	4.65E-01	8.98E-01

**TFEC**	0.40	6.02E-01	1.00E+00	2.29	4.65E-01	8.98E-01
**RWDD2B**	-0.03	9.10E-01	1.00E+00	0.17	4.65E-01	8.98E-01
**MCAM**	0.44	5.23E-01	1.00E+00	0.54	4.65E-01	8.98E-01
**ABCC9**	0.52	2.98E-01	1.00E+00	0.36	4.65E-01	8.98E-01
**NANS**	-0.01	9.78E-01	1.00E+00	-0.21	4.65E-01	8.98E-01
**HJURP**	0.08	8.83E-01	1.00E+00	0.43	4.65E-01	8.98E-01
**TET2**	-0.48	1.64E-01	1.00E+00	-0.22	4.65E-01	8.98E-01
**GSS**	-0.27	1.85E-01	1.00E+00	-0.08	4.65E-01	8.98E-01
**TRIM50**	0.80	4.81E-01	1.00E+00	-1.13	4.65E-01	8.98E-01
**ELANE**	0.53	5.44E-01	1.00E+00	0.48	4.65E-01	8.98E-01
**LMF2**	-0.10	7.32E-01	1.00E+00	0.31	4.65E-01	8.98E-01
**PAIP2B**	0.55	7.72E-02	1.00E+00	-0.33	4.65E-01	8.98E-01
**FLRT1**	-0.29	6.91E-01	1.00E+00	0.68	4.65E-01	8.98E-01
**FGF11**	-0.59	1.93E-01	1.00E+00	0.57	4.65E-01	8.98E-01
**ARPC2**	0.02	9.11E-01	1.00E+00	-0.19	4.65E-01	8.98E-01
**ADIPOQ**	4.57	1.16E-02	6.66E-01	-0.71	4.65E-01	8.98E-01
**CCDC158**	1.23	2.34E-01	1.00E+00	-0.61	4.65E-01	8.98E-01
**SLC25A22**	-0.64	4.19E-02	1.00E+00	-0.48	4.65E-01	8.99E-01
**NGRN**	-0.17	4.04E-01	1.00E+00	0.21	4.65E-01	8.99E-01
**GRM8**	0.28	8.59E-01	1.00E+00	1.02	4.65E-01	8.99E-01
**RACK1**	-0.26	4.26E-01	1.00E+00	0.16	4.66E-01	8.99E-01
**ZNF502**	0.46	8.39E-02	1.00E+00	-0.22	4.66E-01	8.99E-01
**NUP98**	-0.04	8.09E-01	1.00E+00	0.24	4.66E-01	8.99E-01
**OTOP2**	-1.80	1.36E-01	1.00E+00	0.78	4.66E-01	8.99E-01
**KMT2E**	0.05	8.16E-01	1.00E+00	-0.18	4.66E-01	8.99E-01
**SRP14P3**	NA	NA	NA	-1.01	4.66E-01	8.99E-01
**LINC00957**	0.07	9.02E-01	1.00E+00	0.52	4.66E-01	8.99E-01
**CPNE1**	-0.04	8.94E-01	1.00E+00	0.19	4.66E-01	8.99E-01
**RNU6-574P**	0.00	9.99E-01	1.00E+00	0.60	4.66E-01	8.99E-01
**AC139795.2**	-0.14	7.68E-01	1.00E+00	0.41	4.66E-01	8.99E-01
**COL11A2**	-0.52	5.49E-01	1.00E+00	0.78	4.66E-01	8.99E-01
**AC139769.1**	0.07	8.40E-01	1.00E+00	-0.37	4.66E-01	8.99E-01

**PDHB**	0.09	6.49E-01	1.00E+00	-0.23	4.66E-01	8.99E-01
**ATP1B3P1**	NA	NA	NA	-0.57	4.66E-01	8.99E-01
**WASF3**	0.52	2.75E-01	1.00E+00	-0.33	4.66E-01	8.99E-01
**ALX1**	-0.02	9.79E-01	1.00E+00	-0.54	4.66E-01	8.99E-01
**PITPNM2**	-0.15	4.97E-01	1.00E+00	0.52	4.66E-01	8.99E-01
**TCAF2**	-0.27	4.12E-01	1.00E+00	0.33	4.67E-01	8.99E-01
**AC034198.2**	0.92	2.32E-01	1.00E+00	-1.01	4.67E-01	8.99E-01
**FGFBP1**	0.13	8.26E-01	1.00E+00	0.49	4.67E-01	8.99E-01
**ZNF253**	0.29	4.61E-01	1.00E+00	-0.22	4.67E-01	8.99E-01
**TMEM106C**	0.24	2.23E-01	1.00E+00	-0.22	4.67E-01	8.99E-01
**MESD**	0.15	4.85E-01	1.00E+00	0.14	4.67E-01	8.99E-01
**PCDH11X**	-1.70	4.84E-02	1.00E+00	0.54	4.67E-01	8.99E-01
**XLOC_008618**	0.72	2.85E-01	1.00E+00	0.67	4.67E-01	8.99E-01
**LINC01118**	0.81	5.45E-01	1.00E+00	0.83	4.67E-01	8.99E-01
**G30960**	-0.19	8.98E-01	1.00E+00	-0.57	4.67E-01	8.99E-01
**AC018690.1**	0.04	9.55E-01	1.00E+00	-0.78	4.67E-01	8.99E-01
**G4403**	0.05	9.08E-01	1.00E+00	-0.72	4.67E-01	8.99E-01
**AC124916.1**	NA	NA	NA	0.71	4.67E-01	8.99E-01
**PIGCP1**	-0.32	4.29E-01	1.00E+00	0.42	4.67E-01	8.99E-01
**XLOC_002461**	-1.11	2.34E-01	1.00E+00	-0.87	4.67E-01	8.99E-01
**SEC63P1**	-0.16	9.50E-01	1.00E+00	0.45	4.67E-01	8.99E-01
**AGGF1P2**	1.53	4.28E-01	1.00E+00	-1.98	4.67E-01	8.99E-01
**C19orf71**	-0.38	4.95E-01	1.00E+00	0.80	4.67E-01	8.99E-01
**TPD52L2**	0.03	8.48E-01	1.00E+00	0.14	4.67E-01	8.99E-01
**SPRR2A**	-1.94	5.99E-02	1.00E+00	2.27	4.67E-01	8.99E-01
**AL024508.2**	-0.78	5.61E-01	1.00E+00	0.52	4.67E-01	8.99E-01
**FAM153C**	-0.38	8.08E-01	1.00E+00	-0.63	4.67E-01	8.99E-01
**AC009054.2**	-0.62	8.29E-01	1.00E+00	-0.46	4.67E-01	8.99E-01
**AC010422.6**	-0.06	9.14E-01	1.00E+00	0.50	4.67E-01	8.99E-01
**AL353586.1**	-1.01	5.04E-01	1.00E+00	-0.76	4.67E-01	8.99E-01
**CFAP36**	-0.09	7.36E-01	1.00E+00	-0.14	4.67E-01	8.99E-01
**GADD45GIP1**	-0.11	6.26E-01	1.00E+00	-0.32	4.68E-01	8.99E-01

**G34070**	0.02	9.89E-01	1.00E+00	-1.02	4.68E-01	8.99E-01
**ADPRHL2**	-0.24	3.25E-01	1.00E+00	0.14	4.68E-01	8.99E-01
**MPL**	1.02	2.62E-01	1.00E+00	0.76	4.68E-01	8.99E-01
**C3orf80**	1.38	1.31E-01	1.00E+00	0.72	4.68E-01	8.99E-01
**HOXB-AS1**	0.11	9.24E-01	1.00E+00	-0.46	4.68E-01	8.99E-01
**C20orf27**	-0.32	1.71E-01	1.00E+00	-0.18	4.68E-01	8.99E-01
**TECR**	-0.19	4.75E-01	1.00E+00	-0.23	4.68E-01	8.99E-01
**HRCT1**	0.69	3.41E-01	1.00E+00	-0.45	4.68E-01	8.99E-01
**MED26**	-0.28	1.38E-01	1.00E+00	-0.37	4.68E-01	8.99E-01
**DPY19L1P1**	-0.22	6.17E-01	1.00E+00	0.37	4.68E-01	8.99E-01
**SGO1-AS1**	0.16	9.09E-01	1.00E+00	0.75	4.68E-01	8.99E-01
**HRAT92**	-2.17	2.55E-01	1.00E+00	-1.17	4.68E-01	8.99E-01
**TMEM163**	1.51	2.23E-01	1.00E+00	0.80	4.68E-01	8.99E-01
**PRB4**	-0.14	9.16E-01	1.00E+00	-0.96	4.68E-01	9.00E-01
**RPL21P93**	-0.22	9.02E-01	1.00E+00	-0.44	4.68E-01	9.00E-01
**CLN8**	0.23	5.56E-01	1.00E+00	0.18	4.68E-01	9.00E-01
**PLCG2**	-0.15	6.54E-01	1.00E+00	-0.32	4.68E-01	9.00E-01
**RPP30**	0.13	4.15E-01	1.00E+00	-0.19	4.68E-01	9.00E-01
**G32150**	-0.17	9.07E-01	1.00E+00	0.98	4.68E-01	9.00E-01
**TRPA1**	-0.41	8.20E-01	1.00E+00	0.89	4.68E-01	9.00E-01
**EEF1A1P13**	0.19	6.36E-01	1.00E+00	-0.30	4.68E-01	9.00E-01
**GATAD2B**	-0.10	6.85E-01	1.00E+00	0.20	4.68E-01	9.00E-01
**CBWD4P**	-0.44	8.26E-01	1.00E+00	-0.71	4.68E-01	9.00E-01
**GNPTG**	-0.01	9.77E-01	1.00E+00	0.13	4.69E-01	9.00E-01
**PLEKHA7**	-0.02	9.40E-01	1.00E+00	0.36	4.69E-01	9.00E-01
**G11194**	0.14	8.57E-01	1.00E+00	-0.83	4.69E-01	9.00E-01
**CD300E**	-0.12	8.46E-01	1.00E+00	2.27	4.69E-01	9.00E-01
**ZNF862**	-0.09	7.03E-01	1.00E+00	0.21	4.69E-01	9.00E-01
**LINC02587**	1.60	2.27E-01	1.00E+00	-0.58	4.69E-01	9.00E-01
**TNFRSF18**	-0.68	6.62E-02	1.00E+00	-0.37	4.69E-01	9.00E-01
**AC010342.1**	2.05	4.12E-01	1.00E+00	-0.45	4.69E-01	9.00E-01
**CYP2U1**	0.48	2.54E-01	1.00E+00	-0.38	4.69E-01	9.00E-01

**PITRM1-AS1**	-1.72	2.18E-01	1.00E+00	-0.79	4.69E-01	9.00E-01
**DRD2**	0.03	9.66E-01	1.00E+00	-0.97	4.69E-01	9.00E-01
**AL513320.1**	-2.47	1.98E-01	1.00E+00	1.13	4.69E-01	9.00E-01
**OR2W3**	1.17	2.82E-01	1.00E+00	-0.76	4.69E-01	9.00E-01
**TTYH2**	-0.26	3.73E-01	1.00E+00	0.41	4.69E-01	9.00E-01
**AC012498.2**	-4.37	4.53E-02	1.00E+00	0.77	4.69E-01	9.00E-01
**AC022167.2**	0.26	8.18E-01	1.00E+00	0.64	4.69E-01	9.00E-01
**SLC22A1**	0.70	5.40E-01	1.00E+00	1.01	4.69E-01	9.00E-01
**LINC02328**	-1.01	3.40E-01	1.00E+00	0.83	4.69E-01	9.00E-01
**MED20**	0.10	7.20E-01	1.00E+00	-0.21	4.69E-01	9.00E-01
**FGF16**	2.35	1.82E-01	1.00E+00	1.00	4.69E-01	9.00E-01
**DDRGK1**	0.00	9.96E-01	1.00E+00	0.29	4.69E-01	9.00E-01
**VIRMA**	0.18	2.34E-01	1.00E+00	0.12	4.70E-01	9.00E-01
**WDR47**	0.01	9.74E-01	1.00E+00	-0.25	4.70E-01	9.00E-01
**C5orf15**	0.18	3.61E-01	1.00E+00	-0.17	4.70E-01	9.00E-01
**FTH1P23**	-0.05	9.89E-01	1.00E+00	-0.43	4.70E-01	9.00E-01
**AC018645.2**	-0.05	8.97E-01	1.00E+00	0.37	4.70E-01	9.00E-01
**ZWINT**	-0.31	2.95E-01	1.00E+00	0.26	4.70E-01	9.00E-01
**ANKS3**	0.07	8.57E-01	1.00E+00	0.40	4.70E-01	9.00E-01
**AC026124.1**	-1.95	4.67E-01	1.00E+00	-1.20	4.70E-01	9.00E-01
**AC010967.1**	0.78	8.19E-01	1.00E+00	0.98	4.70E-01	9.00E-01
**LINC00211**	-0.06	9.63E-01	1.00E+00	0.87	4.70E-01	9.00E-01
**ADAM19**	0.11	8.53E-01	1.00E+00	2.25	4.70E-01	9.00E-01
**LHFPL4**	-4.14	3.63E-05	9.90E-03	-0.82	4.70E-01	9.00E-01
**SERHL**	0.78	2.80E-01	1.00E+00	0.68	4.70E-01	9.00E-01
**TRIM26**	-0.33	1.78E-01	1.00E+00	0.31	4.70E-01	9.00E-01
**AC091946.1**	-4.71	6.17E-04	1.17E-01	0.64	4.70E-01	9.00E-01
**BTRC**	0.09	6.44E-01	1.00E+00	0.16	4.70E-01	9.00E-01
**ENSA**	0.03	9.12E-01	1.00E+00	0.12	4.70E-01	9.00E-01
**MRTO4**	-0.26	3.13E-01	1.00E+00	0.15	4.70E-01	9.00E-01
**FAM49A**	-0.01	9.86E-01	1.00E+00	0.37	4.70E-01	9.00E-01
**COPS3**	0.14	3.68E-01	1.00E+00	-0.13	4.70E-01	9.00E-01

**KCTD1**	-0.29	2.79E-01	1.00E+00	-0.31	4.70E-01	9.00E-01
**LIG4**	-0.23	4.92E-01	1.00E+00	-0.18	4.70E-01	9.00E-01
**SNU13**	0.04	8.17E-01	1.00E+00	-0.15	4.70E-01	9.00E-01
**BTD**	0.46	2.36E-01	1.00E+00	-0.26	4.70E-01	9.00E-01
**SCLY**	-0.13	9.19E-01	1.00E+00	-0.37	4.71E-01	9.00E-01
**AMY2B**	-0.28	6.31E-01	1.00E+00	-0.56	4.71E-01	9.00E-01
**SMC4**	0.26	3.79E-01	1.00E+00	-0.16	4.71E-01	9.00E-01
**AL121672.1**	-3.08	1.88E-02	8.62E-01	-0.59	4.71E-01	9.00E-01
**AL163051.1**	0.40	5.80E-01	1.00E+00	-0.41	4.71E-01	9.00E-01
**ANGEL2**	0.38	2.49E-01	1.00E+00	-0.16	4.71E-01	9.00E-01
**TDRKH-AS1**	-1.92	5.74E-01	1.00E+00	-0.68	4.71E-01	9.00E-01
**FBH1**	-0.11	5.59E-01	1.00E+00	-0.37	4.71E-01	9.00E-01
**DND1P1**	-1.64	5.84E-02	1.00E+00	0.40	4.71E-01	9.00E-01
**SMIM31**	0.89	6.18E-01	1.00E+00	-1.38	4.71E-01	9.00E-01
**NHLRC3**	0.64	1.11E-01	1.00E+00	0.26	4.71E-01	9.00E-01
**LONRF2**	-0.11	8.50E-01	1.00E+00	-0.55	4.71E-01	9.00E-01
**ZNF594**	-0.17	7.02E-01	1.00E+00	0.48	4.71E-01	9.00E-01
**ZNF706**	-0.01	9.63E-01	1.00E+00	-0.31	4.71E-01	9.00E-01
**AL031320.2**	0.38	7.65E-01	1.00E+00	0.41	4.71E-01	9.00E-01
**AC020898.1**	0.71	6.93E-01	1.00E+00	-0.41	4.71E-01	9.00E-01
**INTS8**	0.16	4.63E-01	1.00E+00	-0.26	4.71E-01	9.00E-01
**AF250324.1**	-0.74	6.92E-01	1.00E+00	1.17	4.71E-01	9.00E-01
**AL359091.4**	-0.99	3.35E-01	1.00E+00	0.80	4.71E-01	9.00E-01
**6-Mar**	0.20	2.97E-01	1.00E+00	-0.24	4.71E-01	9.00E-01
**NUBPL**	-0.10	6.82E-01	1.00E+00	-0.27	4.71E-01	9.00E-01
**TENT4A**	0.11	7.07E-01	1.00E+00	0.34	4.71E-01	9.00E-01
**SOX10**	-0.18	7.27E-01	1.00E+00	0.45	4.71E-01	9.00E-01
**CYTL1**	1.32	4.34E-02	1.00E+00	0.44	4.71E-01	9.00E-01
**CNTN2**	-0.30	5.44E-01	1.00E+00	-1.62	4.71E-01	9.00E-01
**AC060780.1**	-0.21	5.55E-01	1.00E+00	-0.24	4.72E-01	9.00E-01
**SLC10A5**	1.25	1.44E-01	1.00E+00	-0.85	4.72E-01	9.00E-01
**AC010978.1**	-3.61	8.51E-02	1.00E+00	0.77	4.72E-01	9.00E-01

**UGGT1**	0.08	7.15E-01	1.00E+00	0.25	4.72E-01	9.00E-01
**AC115837.1**	0.38	7.66E-01	1.00E+00	0.38	4.72E-01	9.00E-01
**SCARF1**	0.21	6.00E-01	1.00E+00	0.56	4.72E-01	9.00E-01
**ATP6V1A**	0.03	8.94E-01	1.00E+00	-0.15	4.72E-01	9.00E-01
**TEAD4**	0.02	9.50E-01	1.00E+00	0.25	4.72E-01	9.00E-01
**ERP29**	0.00	9.86E-01	1.00E+00	0.12	4.72E-01	9.00E-01
**PROCR**	0.39	3.26E-01	1.00E+00	0.25	4.72E-01	9.00E-01
**MAGEA11**	-0.12	9.73E-01	1.00E+00	-1.77	4.72E-01	9.00E-01
**CACNA2D4**	0.09	7.85E-01	1.00E+00	0.42	4.72E-01	9.00E-01
**CAPNS1**	0.03	9.46E-01	1.00E+00	0.39	4.72E-01	9.00E-01
**AL109976.1**	-0.79	1.24E-01	1.00E+00	0.46	4.72E-01	9.00E-01
**LINC01480**	NA	NA	NA	0.87	4.72E-01	9.00E-01
**STX18**	0.14	3.74E-01	1.00E+00	0.13	4.72E-01	9.00E-01
**PCSK5**	0.59	1.25E-01	1.00E+00	0.39	4.72E-01	9.00E-01
**IFT22**	-0.20	4.63E-01	1.00E+00	-0.16	4.72E-01	9.00E-01
**LINC01550**	-0.12	7.17E-01	1.00E+00	-0.38	4.72E-01	9.00E-01
**TMTC3**	-0.28	4.15E-01	1.00E+00	-0.30	4.72E-01	9.00E-01
**LIN7A**	0.26	5.58E-01	1.00E+00	0.53	4.72E-01	9.00E-01
**IGHD**	2.48	4.89E-02	1.00E+00	2.27	4.72E-01	9.00E-01
**MED13L**	-0.15	4.81E-01	1.00E+00	0.25	4.72E-01	9.00E-01
**AP005230.1**	-0.56	8.73E-01	1.00E+00	1.34	4.72E-01	9.00E-01
**AK9**	0.04	8.67E-01	1.00E+00	-0.25	4.72E-01	9.00E-01
**G21027**	0.15	8.91E-01	1.00E+00	0.61	4.73E-01	9.00E-01
**MTX2**	0.11	6.03E-01	1.00E+00	-0.18	4.73E-01	9.00E-01
**MYMX**	2.04	4.19E-02	1.00E+00	-0.63	4.73E-01	9.00E-01
**TUBGCP3**	0.21	3.50E-01	1.00E+00	0.24	4.73E-01	9.00E-01
**AL513343.1**	0.44	7.59E-01	1.00E+00	0.95	4.73E-01	9.00E-01
**GBF1**	-0.29	8.66E-02	1.00E+00	0.29	4.73E-01	9.00E-01
**LSM4**	-0.12	4.86E-01	1.00E+00	-0.16	4.73E-01	9.00E-01
**ENTPD6**	0.11	8.12E-01	1.00E+00	0.33	4.73E-01	9.00E-01
**C5orf66**	-0.57	7.80E-01	1.00E+00	0.56	4.73E-01	9.00E-01
**UCHL3**	-0.64	4.83E-01	1.00E+00	-0.54	4.73E-01	9.00E-01

**LGMNP1**	NA	NA	NA	0.65	4.73E-01	9.00E-01
**CHAC1**	-0.71	3.37E-01	1.00E+00	0.50	4.73E-01	9.00E-01
**HGS**	-0.32	1.60E-01	1.00E+00	0.21	4.73E-01	9.00E-01
**YWHAZP3**	-0.46	6.37E-01	1.00E+00	-0.33	4.73E-01	9.00E-01
**TMEM53**	0.28	3.97E-01	1.00E+00	-0.19	4.73E-01	9.00E-01
**SETDB1**	-0.24	4.09E-01	1.00E+00	0.23	4.73E-01	9.00E-01
**PLCH2**	-0.70	1.22E-01	1.00E+00	-0.58	4.73E-01	9.00E-01
**AC012640.4**	-0.51	8.61E-01	1.00E+00	1.25	4.73E-01	9.00E-01
**PXN-AS1**	-0.53	3.42E-01	1.00E+00	-0.42	4.73E-01	9.00E-01
**TUBGCP5**	-0.02	9.32E-01	1.00E+00	-0.15	4.73E-01	9.00E-01
**AC005005.3**	1.90	5.75E-01	1.00E+00	-0.86	4.73E-01	9.00E-01
**ROPN1L**	-1.21	3.33E-01	1.00E+00	-0.62	4.73E-01	9.00E-01
**DPH5**	-0.15	6.42E-01	1.00E+00	-0.19	4.73E-01	9.00E-01
**TMEM184A**	-0.36	4.65E-01	1.00E+00	0.59	4.73E-01	9.00E-01
**XLOC_013940**	-1.75	6.03E-01	1.00E+00	-0.88	4.73E-01	9.00E-01
**UST**	0.43	6.38E-02	1.00E+00	-0.23	4.73E-01	9.00E-01
**LMNTD2**	-0.35	4.51E-01	1.00E+00	-0.79	4.73E-01	9.00E-01
**ERVK13-1**	-0.23	5.41E-01	1.00E+00	0.27	4.74E-01	9.00E-01
**TMEM63B**	-0.11	7.37E-01	1.00E+00	0.36	4.74E-01	9.00E-01
**VEZT**	0.09	6.96E-01	1.00E+00	0.12	4.74E-01	9.00E-01
**TUBA4A**	-0.07	8.64E-01	1.00E+00	0.30	4.74E-01	9.00E-01
**HNRNPC**	-0.06	7.37E-01	1.00E+00	0.06	4.74E-01	9.00E-01
**SERP1**	0.28	1.38E-01	1.00E+00	-0.16	4.74E-01	9.00E-01
**LRRC77P**	2.45	6.93E-02	1.00E+00	-1.14	4.74E-01	9.00E-01
**PGD**	0.02	9.37E-01	1.00E+00	0.26	4.74E-01	9.00E-01
**PDCL**	0.16	4.20E-01	1.00E+00	-0.13	4.74E-01	9.00E-01
**ERGIC2**	0.02	9.52E-01	1.00E+00	-0.26	4.74E-01	9.00E-01
**SLC35A3**	0.53	1.69E-01	1.00E+00	-0.20	4.74E-01	9.00E-01
**BRCA2**	-0.29	5.40E-01	1.00E+00	0.27	4.74E-01	9.00E-01
**AC048341.1**	0.27	5.67E-01	1.00E+00	0.51	4.74E-01	9.00E-01
**AC096667.1**	-1.58	5.99E-01	1.00E+00	1.20	4.74E-01	9.01E-01
**SPARC**	0.16	8.07E-01	1.00E+00	0.37	4.74E-01	9.01E-01

**EPHA6**	0.60	4.28E-01	1.00E+00	0.81	4.74E-01	9.01E-01
**FAM209A**	0.24	9.45E-01	1.00E+00	1.16	4.74E-01	9.01E-01
**HRAS**	-0.37	2.46E-01	1.00E+00	-0.28	4.74E-01	9.01E-01
**SLC6A13**	-0.76	6.12E-01	1.00E+00	0.78	4.74E-01	9.01E-01
**IL36RN**	0.16	8.18E-01	1.00E+00	0.57	4.74E-01	9.01E-01
**FBXL13**	-0.20	7.75E-01	1.00E+00	0.41	4.74E-01	9.01E-01
**AC121761.1**	-0.42	3.37E-01	1.00E+00	0.47	4.74E-01	9.01E-01
**FUT8**	0.13	7.11E-01	1.00E+00	0.19	4.74E-01	9.01E-01
**SLC2A8**	-0.04	8.95E-01	1.00E+00	-0.24	4.74E-01	9.01E-01
**TMPRSS12**	0.49	7.08E-01	1.00E+00	0.62	4.74E-01	9.01E-01
**SELE**	-2.35	6.35E-02	1.00E+00	2.24	4.74E-01	9.01E-01
**HNRNPA1P7**	0.94	7.83E-01	1.00E+00	-0.50	4.74E-01	9.01E-01
**C11orf21**	0.02	9.85E-01	1.00E+00	0.76	4.74E-01	9.01E-01
**ABCC12**	-4.09	1.15E-01	1.00E+00	-1.25	4.75E-01	9.01E-01
**ZFAND6**	0.34	1.06E-01	1.00E+00	-0.22	4.75E-01	9.01E-01
**SPRR2D**	-0.79	3.43E-01	1.00E+00	2.24	4.75E-01	9.01E-01
**USP9X**	0.15	3.92E-01	1.00E+00	0.21	4.75E-01	9.01E-01
**LINC01634**	-3.07	1.15E-01	1.00E+00	1.04	4.75E-01	9.01E-01
**ARHGAP5-AS1**	0.38	3.16E-01	1.00E+00	-0.33	4.75E-01	9.01E-01
**TCHH**	-7.27	1.31E-06	4.82E-04	-0.79	4.75E-01	9.01E-01
**AL590764.1**	0.79	5.43E-01	1.00E+00	0.82	4.75E-01	9.01E-01
**KRT12**	0.61	5.91E-01	1.00E+00	0.81	4.75E-01	9.01E-01
**SETD5**	0.00	9.84E-01	1.00E+00	0.27	4.75E-01	9.01E-01
**STARD13**	0.34	3.87E-01	1.00E+00	0.38	4.75E-01	9.01E-01
**AC009120.2**	-2.67	8.98E-02	1.00E+00	-0.42	4.75E-01	9.01E-01
**AC018629.1**	-0.76	9.07E-02	1.00E+00	0.47	4.75E-01	9.01E-01
**ST6GAL1**	0.42	3.91E-01	1.00E+00	0.31	4.75E-01	9.01E-01
**ITPKC**	-0.52	2.30E-01	1.00E+00	-0.38	4.75E-01	9.01E-01
**FGFBP2**	1.02	1.45E-01	1.00E+00	-0.32	4.75E-01	9.01E-01
**ZBTB41**	0.09	8.20E-01	1.00E+00	-0.24	4.75E-01	9.01E-01
**TECTA**	1.14	1.15E-01	1.00E+00	0.88	4.75E-01	9.01E-01
**CLEC4E**	-1.38	3.51E-02	1.00E+00	2.24	4.75E-01	9.01E-01

**ARID3B**	-0.73	5.66E-02	1.00E+00	0.54	4.75E-01	9.01E-01
**GSN**	0.53	1.60E-01	1.00E+00	-0.30	4.75E-01	9.01E-01
**IGHV3-30**	3.60	1.52E-02	7.65E-01	2.25	4.75E-01	9.01E-01
**DEFB132**	4.35	4.53E-02	1.00E+00	-2.26	4.75E-01	9.01E-01
**NIP7**	-0.03	8.95E-01	1.00E+00	0.12	4.75E-01	9.01E-01
**CEP85**	-0.31	3.81E-01	1.00E+00	-0.16	4.75E-01	9.01E-01
**G14802**	0.32	8.26E-01	1.00E+00	0.95	4.76E-01	9.01E-01
**SERAC1**	0.24	4.77E-01	1.00E+00	-0.16	4.76E-01	9.01E-01
**GOLPH3L**	0.42	1.10E-01	1.00E+00	-0.18	4.76E-01	9.01E-01
**NPIPB15**	-0.29	6.69E-01	1.00E+00	0.52	4.76E-01	9.01E-01
**AC093752.1**	-0.50	1.68E-01	1.00E+00	-0.24	4.76E-01	9.01E-01
**AC139530.1**	-0.51	2.68E-01	1.00E+00	-0.36	4.76E-01	9.01E-01
**RBM28**	0.11	6.91E-01	1.00E+00	0.22	4.76E-01	9.01E-01
**RBM45**	-0.21	4.12E-01	1.00E+00	0.18	4.76E-01	9.01E-01
**SNRPG**	-0.09	6.37E-01	1.00E+00	-0.28	4.76E-01	9.01E-01
**C21orf58**	-0.14	7.55E-01	1.00E+00	0.37	4.76E-01	9.01E-01
**AC010894.3**	0.18	9.56E-01	1.00E+00	0.98	4.76E-01	9.01E-01
**CADPS**	-0.72	3.52E-01	1.00E+00	0.62	4.76E-01	9.01E-01
**LINC01010**	-1.01	3.38E-01	1.00E+00	0.73	4.76E-01	9.01E-01
**SMC1A**	-0.03	8.70E-01	1.00E+00	0.22	4.76E-01	9.01E-01
**CARD11**	-0.52	4.37E-01	1.00E+00	2.24	4.76E-01	9.01E-01
**AL353796.1**	0.43	5.46E-01	1.00E+00	-0.62	4.76E-01	9.01E-01
**CALHM5**	0.80	2.05E-01	1.00E+00	-0.44	4.76E-01	9.01E-01
**RSRC2**	0.08	7.39E-01	1.00E+00	-0.13	4.76E-01	9.01E-01
**AL133338.1**	-0.24	5.50E-01	1.00E+00	-0.40	4.76E-01	9.01E-01
**NBN**	0.14	5.74E-01	1.00E+00	-0.13	4.76E-01	9.01E-01
**RAD21**	0.11	6.21E-01	1.00E+00	0.12	4.76E-01	9.01E-01
**RPGRIP1**	-1.28	1.28E-01	1.00E+00	0.41	4.76E-01	9.01E-01
**KRT33A**	-2.51	4.52E-01	1.00E+00	1.14	4.76E-01	9.01E-01
**EIF1**	-0.11	5.45E-01	1.00E+00	-0.15	4.76E-01	9.01E-01
**ENTPD3-AS1**	0.59	1.47E-01	1.00E+00	-0.36	4.76E-01	9.01E-01
**ZNF630**	0.10	8.37E-01	1.00E+00	-0.32	4.76E-01	9.01E-01

**SNIP1**	0.09	6.03E-01	1.00E+00	0.28	4.76E-01	9.01E-01
**AC068587.4**	-1.04	4.26E-02	1.00E+00	-0.33	4.76E-01	9.01E-01
**TPBG**	-0.29	3.26E-01	1.00E+00	0.37	4.77E-01	9.01E-01
**AL445305.1**	0.88	7.98E-01	1.00E+00	-0.47	4.77E-01	9.01E-01
**BTN2A1**	0.08	8.14E-01	1.00E+00	0.31	4.77E-01	9.01E-01
**YKT6**	0.01	9.58E-01	1.00E+00	0.27	4.77E-01	9.01E-01
**CPVL**	-0.74	6.45E-02	1.00E+00	0.21	4.77E-01	9.01E-01
**AP000676.5**	-2.55	7.64E-02	1.00E+00	0.61	4.77E-01	9.01E-01
**FAM149A**	0.46	4.16E-01	1.00E+00	-0.54	4.77E-01	9.01E-01
**ELAVL1**	-0.02	8.84E-01	1.00E+00	-0.31	4.77E-01	9.01E-01
**HCG23**	-0.33	5.85E-01	1.00E+00	0.39	4.77E-01	9.01E-01
**SEMA3D**	0.61	1.41E-01	1.00E+00	-0.36	4.77E-01	9.01E-01
**TFCP2**	0.07	6.52E-01	1.00E+00	0.25	4.77E-01	9.01E-01
**ARRDC2**	0.15	6.35E-01	1.00E+00	0.48	4.77E-01	9.01E-01
**MTUS2**	-0.52	5.73E-01	1.00E+00	-0.65	4.77E-01	9.01E-01
**AC092111.1**	-0.75	3.67E-01	1.00E+00	0.77	4.77E-01	9.01E-01
**MBD5**	-0.39	6.74E-02	1.00E+00	0.12	4.77E-01	9.01E-01
**KCNJ13**	0.42	6.57E-01	1.00E+00	-0.55	4.77E-01	9.01E-01
**LINC01273**	-0.07	9.66E-01	1.00E+00	-0.92	4.77E-01	9.01E-01
**MAP3K3**	0.09	6.65E-01	1.00E+00	0.37	4.77E-01	9.01E-01
**ATF1**	0.34	1.77E-01	1.00E+00	-0.16	4.77E-01	9.01E-01
**TMEM99**	-0.04	9.42E-01	1.00E+00	-0.37	4.77E-01	9.01E-01
**ZFP69B**	-0.98	1.99E-02	8.80E-01	0.38	4.77E-01	9.01E-01
**SIT1**	-0.19	7.29E-01	1.00E+00	0.58	4.77E-01	9.01E-01
**AL354702.1**	-1.48	2.32E-01	1.00E+00	-0.34	4.78E-01	9.01E-01
**AC145138.1**	0.59	6.09E-01	1.00E+00	0.58	4.78E-01	9.01E-01
**ZC3H13**	-0.02	9.25E-01	1.00E+00	0.13	4.78E-01	9.01E-01
**U2AF1L4**	-0.33	7.47E-01	1.00E+00	0.30	4.78E-01	9.01E-01
**MIR503HG**	-2.01	5.50E-01	1.00E+00	1.15	4.78E-01	9.01E-01
**AL691432.1**	0.93	5.05E-01	1.00E+00	0.88	4.78E-01	9.01E-01
**AC104825.1**	0.37	3.56E-01	1.00E+00	-0.31	4.78E-01	9.01E-01
**SHCBP1**	0.05	8.94E-01	1.00E+00	0.35	4.78E-01	9.01E-01

**NTF3**	0.06	8.91E-01	1.00E+00	-0.24	4.78E-01	9.01E-01
**PPP1R12A**	0.14	5.44E-01	1.00E+00	-0.15	4.78E-01	9.01E-01
**G26815**	-0.99	4.65E-01	1.00E+00	1.01	4.78E-01	9.01E-01
**NDRG3**	-0.14	5.56E-01	1.00E+00	-0.22	4.78E-01	9.01E-01
**AL136298.3**	-0.31	9.01E-01	1.00E+00	-0.40	4.78E-01	9.01E-01
**MCM7**	-0.09	5.80E-01	1.00E+00	0.20	4.78E-01	9.01E-01
**ETFB**	0.26	5.23E-01	1.00E+00	-0.32	4.78E-01	9.01E-01
**STX11**	-0.11	8.12E-01	1.00E+00	0.32	4.78E-01	9.01E-01
**ROCK1**	-0.01	9.70E-01	1.00E+00	0.17	4.78E-01	9.01E-01
**CCNY**	0.21	1.92E-01	1.00E+00	-0.26	4.78E-01	9.01E-01
**ELN**	-0.06	9.25E-01	1.00E+00	0.48	4.78E-01	9.01E-01
**AC119396.2**	-1.94	4.01E-01	1.00E+00	0.67	4.78E-01	9.01E-01
**RNU5F-1**	2.23	5.07E-01	1.00E+00	-0.75	4.78E-01	9.01E-01
**DGCR9**	-1.58	1.98E-01	1.00E+00	0.96	4.78E-01	9.01E-01
**SLC35E1**	0.08	6.53E-01	1.00E+00	0.18	4.78E-01	9.01E-01
**G11854**	2.01	2.67E-01	1.00E+00	-0.75	4.79E-01	9.01E-01
**RIMKLA**	-0.14	8.56E-01	1.00E+00	-0.68	4.79E-01	9.01E-01
**KIF21A**	0.32	3.17E-01	1.00E+00	-0.23	4.79E-01	9.01E-01
**OXCT1**	0.02	9.41E-01	1.00E+00	-0.13	4.79E-01	9.01E-01
**EMBP1**	-0.02	9.85E-01	1.00E+00	0.31	4.79E-01	9.01E-01
**AC025287.2**	1.84	2.14E-01	1.00E+00	0.62	4.79E-01	9.01E-01
**MYH3**	-1.37	1.99E-02	8.80E-01	-0.44	4.79E-01	9.01E-01
**C12orf43**	-0.02	9.28E-01	1.00E+00	0.24	4.79E-01	9.01E-01
**MIR222HG**	-0.48	7.59E-01	1.00E+00	0.57	4.79E-01	9.01E-01
**XLOC_004197**	-3.39	1.23E-01	1.00E+00	1.28	4.79E-01	9.01E-01
**SUSD5**	-0.03	9.45E-01	1.00E+00	-0.49	4.79E-01	9.01E-01
**PSMA7**	0.20	2.59E-01	1.00E+00	-0.15	4.79E-01	9.01E-01
**SDHA**	0.06	8.19E-01	1.00E+00	-0.13	4.79E-01	9.01E-01
**G38115**	-0.01	9.91E-01	1.00E+00	0.46	4.79E-01	9.01E-01
**OLMALINC**	0.15	7.76E-01	1.00E+00	-0.28	4.79E-01	9.02E-01
**SLC6A8**	-0.13	6.36E-01	1.00E+00	-0.34	4.79E-01	9.02E-01
**G30345**	0.27	7.27E-01	1.00E+00	0.65	4.79E-01	9.02E-01

**SRSF3**	0.01	9.81E-01	1.00E+00	-0.09	4.79E-01	9.02E-01
**ZNF444**	-0.06	8.38E-01	1.00E+00	0.47	4.79E-01	9.02E-01
**RAB3IL1**	-0.12	7.99E-01	1.00E+00	0.47	4.79E-01	9.02E-01
**AGAP8**	-3.26	3.17E-02	1.00E+00	0.69	4.79E-01	9.02E-01
**PBDC1**	0.06	8.01E-01	1.00E+00	-0.19	4.79E-01	9.02E-01
**MEGF11**	0.07	9.28E-01	1.00E+00	-0.63	4.79E-01	9.02E-01
**GBP5**	-0.77	2.22E-01	1.00E+00	2.21	4.80E-01	9.02E-01
**MLH3**	0.19	5.41E-01	1.00E+00	0.30	4.80E-01	9.02E-01
**AC105429.1**	-0.10	9.37E-01	1.00E+00	0.89	4.80E-01	9.02E-01
**ANKHD1-EIF4EBP3**	-2.78	3.60E-02	1.00E+00	0.44	4.80E-01	9.02E-01
**XLOC_007524**	0.56	5.14E-01	1.00E+00	0.65	4.80E-01	9.02E-01
**CYSLTR2**	0.32	4.85E-01	1.00E+00	0.57	4.80E-01	9.02E-01
**ZNF350**	0.28	1.81E-01	1.00E+00	-0.16	4.80E-01	9.02E-01
**RPS18P12**	-0.08	9.65E-01	1.00E+00	0.77	4.80E-01	9.02E-01
**AC124944.3**	-1.09	3.37E-01	1.00E+00	0.50	4.80E-01	9.02E-01
**SHROOM1**	0.08	8.62E-01	1.00E+00	0.57	4.80E-01	9.02E-01
**ANO10**	-0.06	8.09E-01	1.00E+00	-0.13	4.80E-01	9.02E-01
**MAD2L1**	0.09	8.25E-01	1.00E+00	0.25	4.80E-01	9.02E-01
**AL590648.3**	-2.74	2.09E-01	1.00E+00	0.92	4.80E-01	9.02E-01
**AC145422.1**	-2.22	2.80E-01	1.00E+00	-0.60	4.80E-01	9.02E-01
**AL161668.3**	-1.15	3.52E-01	1.00E+00	-0.64	4.80E-01	9.02E-01
**AL391001.1**	0.30	8.17E-01	1.00E+00	-0.39	4.80E-01	9.02E-01
**AC008736.1**	-0.53	2.58E-01	1.00E+00	-0.53	4.80E-01	9.02E-01
**PRR36**	-1.18	3.38E-02	1.00E+00	-0.61	4.80E-01	9.02E-01
**SETBP1**	-0.34	2.73E-01	1.00E+00	0.28	4.80E-01	9.02E-01
**G28332**	0.42	5.86E-01	1.00E+00	0.55	4.80E-01	9.02E-01
**MORC3**	-0.13	5.88E-01	1.00E+00	-0.13	4.80E-01	9.02E-01
**TP53INP1**	0.32	9.66E-02	1.00E+00	-0.21	4.80E-01	9.02E-01
**MRVI1**	-0.26	6.10E-01	1.00E+00	0.43	4.80E-01	9.02E-01
**JMJD1C**	-0.22	1.86E-01	1.00E+00	0.14	4.81E-01	9.02E-01
**AL358933.1**	-2.02	1.94E-01	1.00E+00	-1.24	4.81E-01	9.02E-01
**TMTC2**	-0.23	4.93E-01	1.00E+00	0.38	4.81E-01	9.02E-01

**PKD2**	0.35	1.83E-01	1.00E+00	0.36	4.81E-01	9.02E-01
**TBX15**	0.66	1.95E-01	1.00E+00	0.37	4.81E-01	9.02E-01
**TNKS1BP1**	-0.05	8.48E-01	1.00E+00	0.41	4.81E-01	9.02E-01
**LINC01315**	0.10	7.15E-01	1.00E+00	-0.23	4.81E-01	9.02E-01
**SAA2**	-1.18	6.10E-01	1.00E+00	-0.77	4.81E-01	9.02E-01
**AGAP10P**	-2.80	1.31E-01	1.00E+00	0.54	4.81E-01	9.02E-01
**KAZN**	0.00	9.97E-01	1.00E+00	-0.30	4.81E-01	9.02E-01
**SLC2A4**	0.15	8.47E-01	1.00E+00	-0.37	4.81E-01	9.02E-01
**SLC1A5**	-0.03	9.53E-01	1.00E+00	-0.33	4.81E-01	9.02E-01
**CARMIL3**	-0.26	6.33E-01	1.00E+00	0.59	4.81E-01	9.02E-01
**MPI**	0.05	8.43E-01	1.00E+00	0.14	4.81E-01	9.02E-01
**AC092944.1**	-0.42	6.86E-01	1.00E+00	-0.40	4.81E-01	9.02E-01
**ZNF19**	0.73	2.55E-01	1.00E+00	-0.44	4.81E-01	9.02E-01
**AL049796.1**	-0.32	5.71E-01	1.00E+00	-0.58	4.81E-01	9.02E-01
**AC078899.1**	0.60	5.40E-01	1.00E+00	-0.56	4.81E-01	9.02E-01
**FLJ22447**	-0.51	4.35E-01	1.00E+00	-0.56	4.81E-01	9.02E-01
**CDH20**	0.45	4.96E-01	1.00E+00	0.65	4.81E-01	9.02E-01
**HSD3B1**	0.99	7.52E-01	1.00E+00	-2.21	4.81E-01	9.02E-01
**ADGRF1**	2.12	4.21E-01	1.00E+00	1.17	4.81E-01	9.02E-01
**CRAMP1**	0.09	7.71E-01	1.00E+00	0.41	4.81E-01	9.02E-01
**TSHR**	-0.07	9.59E-01	1.00E+00	0.60	4.81E-01	9.02E-01
**MATN4**	0.72	2.67E-01	1.00E+00	0.49	4.82E-01	9.02E-01
**AL358777.1**	0.00	9.98E-01	1.00E+00	0.86	4.82E-01	9.02E-01
**PLA2G2F**	-0.22	7.75E-01	1.00E+00	0.49	4.82E-01	9.02E-01
**MAGEF1**	-0.01	9.70E-01	1.00E+00	-0.31	4.82E-01	9.02E-01
**HBS1L**	0.18	4.25E-01	1.00E+00	-0.17	4.82E-01	9.02E-01
**HAUS1**	-0.15	5.70E-01	1.00E+00	-0.18	4.82E-01	9.02E-01
**POLM**	-0.04	8.87E-01	1.00E+00	0.25	4.82E-01	9.02E-01
**IKZF3**	0.03	9.72E-01	1.00E+00	1.95	4.82E-01	9.02E-01
**PPP1CB**	0.17	4.86E-01	1.00E+00	-0.27	4.82E-01	9.02E-01
**ADNP-AS1**	-0.12	8.53E-01	1.00E+00	0.49	4.82E-01	9.02E-01
**G43233**	0.43	6.88E-01	1.00E+00	0.68	4.82E-01	9.02E-01

**NUDT18**	-0.21	4.99E-01	1.00E+00	0.41	4.82E-01	9.02E-01
**TRAV8-4**	-0.32	8.91E-01	1.00E+00	0.91	4.82E-01	9.03E-01
**AC026877.1**	-0.41	9.05E-01	1.00E+00	0.50	4.82E-01	9.03E-01
**IGHV3-7**	2.10	2.97E-01	1.00E+00	2.22	4.82E-01	9.03E-01
**C1QTNF9**	0.54	8.06E-01	1.00E+00	-1.60	4.82E-01	9.03E-01
**MIA3**	-0.16	4.93E-01	1.00E+00	0.11	4.82E-01	9.03E-01
**NACAD**	0.15	7.43E-01	1.00E+00	0.41	4.83E-01	9.03E-01
**AC008074.2**	0.24	7.74E-01	1.00E+00	0.34	4.83E-01	9.03E-01
**FERMT1**	0.13	7.07E-01	1.00E+00	0.41	4.83E-01	9.03E-01
**AC126773.2**	1.34	2.08E-01	1.00E+00	-0.35	4.83E-01	9.03E-01
**UBXN8**	0.18	6.00E-01	1.00E+00	0.19	4.83E-01	9.03E-01
**ART3**	-1.03	1.00E-01	1.00E+00	-0.34	4.83E-01	9.03E-01
**MT-TV**	-0.56	8.73E-01	1.00E+00	-0.70	4.83E-01	9.03E-01
**AC022497.1**	0.72	8.09E-02	1.00E+00	0.68	4.83E-01	9.03E-01
**LINC00842**	0.04	9.21E-01	1.00E+00	-0.39	4.83E-01	9.03E-01
**HUWE1**	-0.20	3.91E-01	1.00E+00	0.31	4.83E-01	9.03E-01
**ABHD16A**	0.01	9.90E-01	1.00E+00	0.50	4.83E-01	9.03E-01
**ZNF3**	-0.01	9.75E-01	1.00E+00	-0.14	4.83E-01	9.03E-01
**NKG7**	-0.20	7.73E-01	1.00E+00	1.91	4.83E-01	9.03E-01
**FAM178B**	-0.59	3.15E-01	1.00E+00	-0.31	4.83E-01	9.03E-01
**NR4A3**	-1.85	1.80E-02	8.42E-01	2.19	4.83E-01	9.03E-01
**TIPARP-AS1**	-0.17	8.21E-01	1.00E+00	-0.36	4.83E-01	9.03E-01
**PLET1**	-2.06	5.41E-01	1.00E+00	-1.41	4.83E-01	9.03E-01
**PTCHD4**	2.17	8.83E-02	1.00E+00	0.88	4.83E-01	9.03E-01
**DDX27**	-0.21	3.06E-01	1.00E+00	-0.12	4.83E-01	9.03E-01
**AL445426.1**	2.28	7.26E-02	1.00E+00	0.56	4.83E-01	9.03E-01
**CLPP**	-0.09	7.26E-01	1.00E+00	0.09	4.83E-01	9.03E-01
**PHF6**	0.40	1.77E-01	1.00E+00	-0.18	4.84E-01	9.03E-01
**HNRNPA3P6**	0.06	9.09E-01	1.00E+00	0.20	4.84E-01	9.03E-01
**TMEM9B**	0.21	3.51E-01	1.00E+00	-0.26	4.84E-01	9.03E-01
**XIAP**	0.15	4.72E-01	1.00E+00	0.21	4.84E-01	9.03E-01
**PM20D1**	-0.08	9.79E-01	1.00E+00	-2.19	4.84E-01	9.03E-01

**MAS1L**	1.32	1.70E-01	1.00E+00	0.57	4.84E-01	9.03E-01
**RPL11P3**	0.37	7.70E-01	1.00E+00	0.37	4.84E-01	9.03E-01
**NEK4**	0.23	3.01E-01	1.00E+00	0.22	4.84E-01	9.04E-01
**EIF1AY**	0.33	5.78E-01	1.00E+00	1.66	4.84E-01	9.04E-01
**ETNK2**	-0.55	1.77E-01	1.00E+00	-0.31	4.84E-01	9.04E-01
**ZBTB14**	0.15	4.68E-01	1.00E+00	-0.14	4.84E-01	9.04E-01
**CLEC4F**	-0.92	6.05E-01	1.00E+00	1.12	4.84E-01	9.04E-01
**PHEX**	0.40	6.09E-01	1.00E+00	0.63	4.84E-01	9.04E-01
**IGKV2D-29**	1.50	6.62E-01	1.00E+00	2.23	4.84E-01	9.04E-01
**APOBEC3H**	-1.90	3.57E-01	1.00E+00	0.79	4.84E-01	9.04E-01
**CEP83**	-0.32	4.37E-01	1.00E+00	-0.19	4.84E-01	9.04E-01
**WDR89**	0.09	8.28E-01	1.00E+00	-0.23	4.84E-01	9.04E-01
**LCE1E**	-1.71	1.33E-01	1.00E+00	0.58	4.84E-01	9.04E-01
**QTRT2**	-0.16	5.22E-01	1.00E+00	-0.15	4.84E-01	9.04E-01
**AC098829.1**	-1.30	1.94E-01	1.00E+00	-0.44	4.84E-01	9.04E-01
**SESTD1**	0.77	1.09E-01	1.00E+00	-0.33	4.84E-01	9.04E-01
**C5orf58**	1.77	2.33E-01	1.00E+00	0.77	4.85E-01	9.04E-01
**NOSTRIN**	0.53	3.98E-01	1.00E+00	-0.30	4.85E-01	9.04E-01
**ZNF75D**	0.16	5.22E-01	1.00E+00	0.32	4.85E-01	9.04E-01
**SELENBP1**	0.53	3.29E-01	1.00E+00	-0.26	4.85E-01	9.04E-01
**LINC00896**	2.01	5.54E-01	1.00E+00	-0.95	4.85E-01	9.04E-01
**RTKN2**	-0.21	6.15E-01	1.00E+00	0.34	4.85E-01	9.04E-01
**TBC1D9B**	-0.14	5.27E-01	1.00E+00	0.31	4.85E-01	9.04E-01
**AC005702.1**	0.39	5.79E-01	1.00E+00	0.51	4.85E-01	9.04E-01
**RNF187**	-0.03	9.04E-01	1.00E+00	-0.39	4.85E-01	9.04E-01
**PRRX2**	-0.30	2.70E-01	1.00E+00	0.43	4.85E-01	9.04E-01
**DIXDC1**	0.32	4.61E-01	1.00E+00	-0.28	4.85E-01	9.04E-01
**XLOC_003854**	NA	NA	NA	-1.14	4.85E-01	9.04E-01
**IL20RB-AS1**	-0.86	4.60E-01	1.00E+00	0.55	4.85E-01	9.04E-01
**TMEM170A**	0.50	1.19E-01	1.00E+00	-0.16	4.85E-01	9.04E-01
**AC109326.1**	0.13	9.14E-01	1.00E+00	-0.52	4.85E-01	9.04E-01
**SLC24A5**	0.19	8.17E-01	1.00E+00	0.56	4.86E-01	9.04E-01

**AL118506.1**	-0.10	7.40E-01	1.00E+00	0.44	4.86E-01	9.04E-01
**BANF1P2**	-1.38	1.56E-01	1.00E+00	0.48	4.86E-01	9.04E-01
**NIPAL3**	0.17	5.87E-01	1.00E+00	-0.17	4.86E-01	9.04E-01
**LINC02453**	-1.79	2.70E-01	1.00E+00	-0.47	4.86E-01	9.04E-01
**MFSD11**	0.21	3.34E-01	1.00E+00	-0.17	4.86E-01	9.04E-01
**G3616**	0.43	6.04E-01	1.00E+00	0.58	4.86E-01	9.04E-01
**G32994**	-1.94	2.72E-01	1.00E+00	0.69	4.86E-01	9.04E-01
**AC137767.1**	-0.23	8.43E-01	1.00E+00	0.48	4.86E-01	9.04E-01
**AL079303.1**	3.63	2.27E-01	1.00E+00	-0.98	4.86E-01	9.04E-01
**GPR22**	2.41	4.65E-01	1.00E+00	-0.70	4.86E-01	9.04E-01
**LRCOL1**	-1.28	2.94E-01	1.00E+00	0.73	4.86E-01	9.04E-01
**AL353662.2**	0.36	9.17E-01	1.00E+00	0.55	4.86E-01	9.04E-01
**PTPN22**	0.38	5.09E-01	1.00E+00	0.50	4.86E-01	9.04E-01
**BLOC1S1**	0.62	2.04E-01	1.00E+00	-0.42	4.86E-01	9.04E-01
**FAHD1**	-0.11	7.22E-01	1.00E+00	-0.20	4.86E-01	9.04E-01
**WDR37**	0.55	8.35E-02	1.00E+00	0.16	4.86E-01	9.04E-01
**FOXP1**	0.01	9.83E-01	1.00E+00	0.28	4.86E-01	9.04E-01
**ZSWIM9**	-0.25	4.00E-01	1.00E+00	0.49	4.86E-01	9.04E-01
**AC103923.1**	-0.96	4.47E-01	1.00E+00	-0.66	4.86E-01	9.04E-01
**ZSWIM8**	-0.20	4.24E-01	1.00E+00	0.39	4.86E-01	9.04E-01
**AC005261.3**	0.39	3.68E-01	1.00E+00	0.25	4.86E-01	9.04E-01
**PRAF2**	0.23	7.11E-01	1.00E+00	0.34	4.86E-01	9.04E-01
**S100A9**	-1.29	4.31E-02	1.00E+00	2.18	4.86E-01	9.04E-01
**BX276092.7**	-0.32	8.26E-01	1.00E+00	0.54	4.86E-01	9.04E-01
**SLC41A2**	0.16	7.49E-01	1.00E+00	0.34	4.86E-01	9.04E-01
**MRPL53P1**	-1.33	6.81E-01	1.00E+00	0.68	4.86E-01	9.04E-01
**HDGFL3**	0.05	8.53E-01	1.00E+00	0.29	4.87E-01	9.05E-01
**LINC01136**	-0.02	9.87E-01	1.00E+00	-0.52	4.87E-01	9.05E-01
**DSEL**	0.48	2.35E-01	1.00E+00	0.33	4.87E-01	9.05E-01
**SCAMP2**	0.09	6.56E-01	1.00E+00	0.10	4.87E-01	9.05E-01
**MCF2L2**	-2.62	2.59E-05	7.38E-03	0.53	4.87E-01	9.05E-01
**PTPRG-AS1**	-0.87	4.63E-01	1.00E+00	0.49	4.87E-01	9.05E-01

**ITCH**	0.07	7.62E-01	1.00E+00	0.19	4.87E-01	9.05E-01
**CCDC110**	-0.27	6.07E-01	1.00E+00	-0.49	4.87E-01	9.05E-01
**CEACAM21**	0.66	4.85E-01	1.00E+00	0.72	4.87E-01	9.05E-01
**MRPL11**	-0.08	6.52E-01	1.00E+00	-0.12	4.87E-01	9.05E-01
**ITGA1**	0.30	5.85E-01	1.00E+00	0.39	4.87E-01	9.05E-01
**AC022217.3**	-1.42	4.24E-01	1.00E+00	0.72	4.87E-01	9.05E-01
**AC087392.2**	-0.93	5.39E-02	1.00E+00	-0.57	4.87E-01	9.05E-01
**FAM69A**	0.45	2.12E-01	1.00E+00	-0.27	4.87E-01	9.05E-01
**CNNM3**	0.01	9.48E-01	1.00E+00	-0.40	4.87E-01	9.05E-01
**AC084880.1**	-0.58	2.92E-01	1.00E+00	0.37	4.87E-01	9.05E-01
**MGAT3-AS1**	-1.84	2.84E-01	1.00E+00	-0.81	4.87E-01	9.05E-01
**FAM69B**	-0.22	5.23E-01	1.00E+00	-0.41	4.87E-01	9.05E-01
**NCEH1**	0.05	9.06E-01	1.00E+00	0.28	4.87E-01	9.05E-01
**UBE2N**	-0.05	8.37E-01	1.00E+00	-0.20	4.87E-01	9.05E-01
**MBP**	-0.19	6.43E-01	1.00E+00	-0.17	4.87E-01	9.05E-01
**ULBP3**	-0.28	7.59E-01	1.00E+00	0.53	4.87E-01	9.05E-01
**RPL10P16**	-0.01	9.84E-01	1.00E+00	-0.28	4.87E-01	9.05E-01
**RAB18**	0.15	5.84E-01	1.00E+00	-0.22	4.87E-01	9.05E-01
**CYP4F3**	-0.50	3.43E-01	1.00E+00	-0.41	4.87E-01	9.05E-01
**ABHD13**	-0.01	9.58E-01	1.00E+00	-0.15	4.88E-01	9.05E-01
**KIRREL2**	0.25	9.29E-01	1.00E+00	-1.17	4.88E-01	9.05E-01
**FTH1P22**	2.17	4.06E-01	1.00E+00	1.34	4.88E-01	9.05E-01
**LRRC46**	-0.86	4.38E-01	1.00E+00	-0.69	4.88E-01	9.05E-01
**XLOC_003782**	1.92	2.57E-03	2.61E-01	0.44	4.88E-01	9.05E-01
**AC106872.2**	-1.71	5.35E-01	1.00E+00	-0.42	4.88E-01	9.05E-01
**ADORA2A-AS1**	-2.86	4.41E-03	3.75E-01	-0.82	4.88E-01	9.05E-01
**KCND3**	-0.24	3.71E-01	1.00E+00	0.28	4.88E-01	9.05E-01
**JMJD7-PLA2G4B**	0.39	7.57E-01	1.00E+00	0.66	4.88E-01	9.05E-01
**HOXC9**	-0.06	8.99E-01	1.00E+00	-0.22	4.88E-01	9.05E-01
**PFKP**	-0.53	9.86E-03	6.15E-01	0.26	4.88E-01	9.05E-01
**SEMA3B**	0.53	3.61E-01	1.00E+00	0.46	4.88E-01	9.05E-01
**PMEL**	0.05	9.21E-01	1.00E+00	0.45	4.88E-01	9.05E-01

**GRM7-AS1**	0.92	4.35E-01	1.00E+00	-0.87	4.88E-01	9.05E-01
**AC114810.1**	-0.47	6.68E-01	1.00E+00	-0.61	4.88E-01	9.05E-01
**RABGAP1L-IT1**	2.69	4.27E-01	1.00E+00	0.91	4.88E-01	9.05E-01
**EIF4BP3**	-0.07	9.20E-01	1.00E+00	0.27	4.88E-01	9.05E-01
**AC093297.2**	0.55	1.53E-01	1.00E+00	-0.28	4.88E-01	9.05E-01
**ACAD8**	0.03	8.90E-01	1.00E+00	-0.19	4.88E-01	9.05E-01
**TUBBP1**	0.23	8.73E-01	1.00E+00	-0.34	4.89E-01	9.05E-01
**DHCR24-DT**	1.67	2.11E-01	1.00E+00	0.56	4.89E-01	9.05E-01
**ASCC2**	-0.20	3.64E-01	1.00E+00	0.12	4.89E-01	9.05E-01
**C6orf120**	0.14	5.73E-01	1.00E+00	0.30	4.89E-01	9.05E-01
**AL356275.1**	0.16	7.45E-01	1.00E+00	-0.51	4.89E-01	9.05E-01
**FKBP14**	0.18	5.02E-01	1.00E+00	-0.20	4.89E-01	9.05E-01
**NAPEPLD**	0.11	7.36E-01	1.00E+00	-0.18	4.89E-01	9.05E-01
**AL121757.1**	0.84	7.86E-01	1.00E+00	1.39	4.89E-01	9.05E-01
**AC012313.3**	0.98	3.88E-01	1.00E+00	0.67	4.89E-01	9.05E-01
**PLEKHA4**	-0.16	7.05E-01	1.00E+00	0.42	4.89E-01	9.05E-01
**XLOC_011822**	-0.74	7.47E-01	1.00E+00	-0.65	4.89E-01	9.05E-01
**SNORA59A**	-0.51	8.81E-01	1.00E+00	0.51	4.89E-01	9.05E-01
**SYT15**	1.04	2.76E-01	1.00E+00	0.28	4.89E-01	9.05E-01
**HAND2**	1.66	1.51E-02	7.65E-01	0.67	4.89E-01	9.05E-01
**TMEM94**	0.05	8.55E-01	1.00E+00	0.33	4.89E-01	9.05E-01
**INO80C**	0.31	3.24E-01	1.00E+00	-0.24	4.89E-01	9.05E-01
**FAM216A**	0.14	6.97E-01	1.00E+00	-0.25	4.89E-01	9.05E-01
**AC069200.1**	-0.87	4.99E-01	1.00E+00	-0.47	4.89E-01	9.05E-01
**CDC42EP5**	-0.19	5.64E-01	1.00E+00	0.71	4.89E-01	9.05E-01
**XLOC_004881**	-0.08	8.68E-01	1.00E+00	-0.34	4.89E-01	9.05E-01
**AC124944.2**	-2.27	4.43E-01	1.00E+00	1.04	4.89E-01	9.05E-01
**NEU1**	0.11	4.84E-01	1.00E+00	0.12	4.89E-01	9.05E-01
**GCH1**	-0.26	3.50E-01	1.00E+00	0.38	4.89E-01	9.05E-01
**CYP4Z2P**	0.78	3.76E-01	1.00E+00	0.53	4.89E-01	9.05E-01
**CLEC5A**	-0.63	2.36E-01	1.00E+00	0.51	4.90E-01	9.05E-01
**ZNF233**	0.51	5.49E-01	1.00E+00	0.58	4.90E-01	9.05E-01

**SEMA3C**	0.27	5.88E-01	1.00E+00	-0.22	4.90E-01	9.05E-01
**LINC00174**	-0.47	3.94E-01	1.00E+00	0.38	4.90E-01	9.05E-01
**MEDAG**	0.56	2.49E-01	1.00E+00	0.51	4.90E-01	9.05E-01
**ZNF613**	0.09	7.02E-01	1.00E+00	0.19	4.90E-01	9.06E-01
**MRPL20**	0.02	8.85E-01	1.00E+00	-0.16	4.90E-01	9.06E-01
**GALT**	-0.08	8.24E-01	1.00E+00	-0.23	4.90E-01	9.06E-01
**ERO1B**	0.48	2.42E-01	1.00E+00	0.25	4.90E-01	9.06E-01
**AL604028.1**	0.25	5.26E-01	1.00E+00	0.41	4.90E-01	9.06E-01
**MRPL58**	-0.18	5.11E-01	1.00E+00	-0.17	4.90E-01	9.06E-01
**AP002840.2**	0.35	5.63E-01	1.00E+00	0.47	4.90E-01	9.06E-01
**KIAA1324L**	0.05	8.96E-01	1.00E+00	-0.37	4.90E-01	9.06E-01
**HNRNPA1P33**	0.08	8.96E-01	1.00E+00	0.43	4.90E-01	9.06E-01
**UPK1B**	-0.34	5.14E-01	1.00E+00	-0.63	4.90E-01	9.06E-01
**AC046168.1**	0.23	8.77E-01	1.00E+00	-0.97	4.90E-01	9.06E-01
**TXNRD1**	0.41	1.42E-01	1.00E+00	0.30	4.90E-01	9.06E-01
**BRD8**	0.28	1.07E-01	1.00E+00	0.15	4.90E-01	9.06E-01
**AMBP**	-3.33	2.09E-02	8.94E-01	-0.64	4.90E-01	9.06E-01
**CDC123**	-0.07	6.81E-01	1.00E+00	-0.26	4.90E-01	9.06E-01
**AC016747.1**	0.42	3.34E-01	1.00E+00	0.34	4.90E-01	9.06E-01
**UNC13C**	-0.16	8.64E-01	1.00E+00	-0.59	4.90E-01	9.06E-01
**SGTA**	-0.12	5.75E-01	1.00E+00	0.16	4.91E-01	9.06E-01
**AL732292.2**	0.63	7.30E-01	1.00E+00	0.61	4.91E-01	9.06E-01
**LRRC42**	-0.17	4.92E-01	1.00E+00	-0.11	4.91E-01	9.06E-01
**PSMB6**	-0.04	8.40E-01	1.00E+00	-0.23	4.91E-01	9.06E-01
**HERC2P8**	NA	NA	NA	0.82	4.91E-01	9.06E-01
**AC109462.1**	1.14	6.37E-01	1.00E+00	-0.80	4.91E-01	9.06E-01
**B4GALT3**	0.10	7.06E-01	1.00E+00	0.12	4.91E-01	9.07E-01
**MYL3**	0.15	7.48E-01	1.00E+00	0.48	4.91E-01	9.07E-01
**AL031985.2**	NA	NA	NA	-0.45	4.91E-01	9.07E-01
**AC073316.1**	0.10	9.43E-01	1.00E+00	0.88	4.91E-01	9.07E-01
**LINC02240**	3.27	2.96E-02	9.96E-01	-2.17	4.91E-01	9.07E-01
**DKKL1**	1.52	1.43E-01	1.00E+00	0.68	4.91E-01	9.07E-01

**PRRG3**	0.67	2.28E-01	1.00E+00	0.34	4.91E-01	9.07E-01
**AC005062.1**	-3.02	3.72E-01	1.00E+00	0.62	4.91E-01	9.07E-01
**CD207**	-0.88	1.39E-01	1.00E+00	-0.51	4.92E-01	9.07E-01
**ANG**	0.29	3.43E-01	1.00E+00	-0.34	4.92E-01	9.07E-01
**PDPK2P**	-0.74	1.94E-01	1.00E+00	0.49	4.92E-01	9.07E-01
**RPS14**	-0.22	5.33E-01	1.00E+00	0.22	4.92E-01	9.07E-01
**ZNF610**	0.33	6.21E-01	1.00E+00	-0.55	4.92E-01	9.07E-01
**SLC25A28**	-0.27	3.73E-01	1.00E+00	0.41	4.92E-01	9.07E-01
**ADAMTS13**	0.13	8.44E-01	1.00E+00	0.67	4.92E-01	9.07E-01
**FAM229B**	-0.31	4.63E-01	1.00E+00	-0.21	4.92E-01	9.07E-01
**MIF**	-1.17	1.33E-01	1.00E+00	0.50	4.92E-01	9.07E-01
**ALDH5A1**	0.06	7.31E-01	1.00E+00	-0.26	4.92E-01	9.07E-01
**RABEPK**	-0.04	7.92E-01	1.00E+00	-0.11	4.92E-01	9.07E-01
**XLOC_013181**	-1.20	3.49E-01	1.00E+00	-0.45	4.92E-01	9.07E-01
**DNAH14**	0.11	8.84E-01	1.00E+00	0.59	4.92E-01	9.07E-01
**AL603839.3**	1.97	3.44E-02	1.00E+00	0.36	4.92E-01	9.07E-01
**SAV1**	0.04	8.75E-01	1.00E+00	-0.29	4.92E-01	9.07E-01
**AC091982.3**	-0.07	8.18E-01	1.00E+00	-0.24	4.92E-01	9.07E-01
**AP001318.2**	0.97	1.12E-01	1.00E+00	0.30	4.92E-01	9.07E-01
**CTSG**	0.80	1.98E-01	1.00E+00	0.42	4.92E-01	9.07E-01
**SGTB**	-0.01	9.72E-01	1.00E+00	0.22	4.92E-01	9.07E-01
**G35570**	-2.48	4.68E-01	1.00E+00	-1.10	4.92E-01	9.07E-01
**TCEAL5**	0.79	5.09E-01	1.00E+00	-0.63	4.92E-01	9.07E-01
**MAL2-AS1**	-2.12	4.32E-01	1.00E+00	-0.95	4.92E-01	9.07E-01
**HS3ST5**	0.35	7.55E-01	1.00E+00	-0.92	4.92E-01	9.07E-01
**AP001351.1**	-1.78	1.06E-01	1.00E+00	0.50	4.92E-01	9.07E-01
**OSR1**	0.69	6.44E-02	1.00E+00	0.25	4.92E-01	9.07E-01
**POLR2I**	0.47	4.04E-01	1.00E+00	-0.44	4.92E-01	9.07E-01
**TMEM98**	0.17	6.38E-01	1.00E+00	0.18	4.92E-01	9.07E-01
**G15642**	0.73	1.32E-01	1.00E+00	-0.43	4.93E-01	9.07E-01
**LINC01122**	-2.13	3.54E-01	1.00E+00	-0.96	4.93E-01	9.07E-01
**RPS6KC1**	-0.14	5.52E-01	1.00E+00	0.15	4.93E-01	9.07E-01

**CERK**	0.36	3.51E-01	1.00E+00	0.27	4.93E-01	9.07E-01
**STAT2**	0.06	9.01E-01	1.00E+00	0.30	4.93E-01	9.07E-01
**UFL1**	0.04	8.77E-01	1.00E+00	-0.15	4.93E-01	9.07E-01
**ANGPTL5**	1.17	1.76E-02	8.33E-01	0.45	4.93E-01	9.07E-01
**PYGO2**	0.00	9.83E-01	1.00E+00	-0.30	4.93E-01	9.07E-01
**AC067852.2**	NA	NA	NA	0.58	4.93E-01	9.07E-01
**BRD9**	0.15	3.79E-01	1.00E+00	0.26	4.93E-01	9.07E-01
**CELF2-AS2**	1.96	5.66E-01	1.00E+00	0.81	4.93E-01	9.07E-01
**C2orf15**	0.05	8.92E-01	1.00E+00	-0.33	4.93E-01	9.07E-01
**TMEM121B**	-0.67	8.97E-02	1.00E+00	0.64	4.93E-01	9.07E-01
**ZNF581**	-0.04	8.56E-01	1.00E+00	0.18	4.93E-01	9.07E-01
**ACVR1C**	3.55	1.71E-02	8.25E-01	-0.62	4.93E-01	9.07E-01
**TRIM40**	-0.20	9.53E-01	1.00E+00	-2.19	4.93E-01	9.07E-01
**HSP90AB3P**	-0.37	8.19E-01	1.00E+00	-0.43	4.93E-01	9.07E-01
**ANGPTL1**	0.82	8.44E-02	1.00E+00	0.32	4.93E-01	9.07E-01
**MAFG-DT**	-0.22	4.45E-01	1.00E+00	-0.55	4.93E-01	9.07E-01
**C11orf96**	-1.76	2.82E-06	9.97E-04	0.56	4.93E-01	9.07E-01
**AC006001.3**	-0.11	6.30E-01	1.00E+00	0.15	4.93E-01	9.07E-01
**AC104046.1**	-2.44	1.72E-01	1.00E+00	-0.60	4.93E-01	9.07E-01
**TOP3A**	0.01	9.71E-01	1.00E+00	0.29	4.94E-01	9.07E-01
**CLK1**	-0.63	3.11E-01	1.00E+00	-0.27	4.94E-01	9.07E-01
**FBXL16**	-0.43	3.04E-01	1.00E+00	-0.45	4.94E-01	9.07E-01
**SPRYD7**	-0.07	7.80E-01	1.00E+00	-0.19	4.94E-01	9.07E-01
**SGCZ**	1.72	2.43E-01	1.00E+00	1.67	4.94E-01	9.07E-01
**FOXL1**	-0.74	3.50E-01	1.00E+00	0.85	4.94E-01	9.07E-01
**RPL9P8**	-1.09	6.07E-01	1.00E+00	-0.47	4.94E-01	9.07E-01
**ME2**	-0.07	8.06E-01	1.00E+00	-0.23	4.94E-01	9.07E-01
**IL20**	-2.56	2.34E-01	1.00E+00	2.15	4.94E-01	9.07E-01
**GFM2**	0.07	7.96E-01	1.00E+00	-0.14	4.94E-01	9.07E-01
**G30952**	-0.40	7.81E-01	1.00E+00	0.80	4.94E-01	9.07E-01
**METRNL**	-0.06	8.74E-01	1.00E+00	0.36	4.94E-01	9.07E-01
**TRMT10A**	0.86	7.84E-02	1.00E+00	-0.26	4.94E-01	9.07E-01

**NTAN1P2**	1.38	3.76E-01	1.00E+00	0.51	4.94E-01	9.07E-01
**AC018845.3**	-1.15	4.85E-01	1.00E+00	0.85	4.94E-01	9.07E-01
**PACS1**	-0.28	3.29E-01	1.00E+00	0.39	4.94E-01	9.07E-01
**IGSF3**	-0.53	5.06E-02	1.00E+00	0.35	4.94E-01	9.07E-01
**TMEM126B**	0.21	3.65E-01	1.00E+00	-0.19	4.94E-01	9.07E-01
**ABHD12B**	0.20	7.22E-01	1.00E+00	-0.40	4.94E-01	9.07E-01
**PILRB**	1.65	1.71E-01	1.00E+00	0.75	4.94E-01	9.07E-01
**NCR1**	-2.69	3.05E-01	1.00E+00	1.06	4.94E-01	9.07E-01
**ATF7IP2**	0.35	3.41E-01	1.00E+00	0.26	4.94E-01	9.07E-01
**AC087620.1**	1.90	2.50E-01	1.00E+00	0.63	4.94E-01	9.07E-01
**COTL1**	0.09	8.32E-01	1.00E+00	0.45	4.94E-01	9.07E-01
**DIP2B**	0.07	7.89E-01	1.00E+00	0.28	4.94E-01	9.07E-01
**B3GAT2**	0.95	5.18E-01	1.00E+00	0.64	4.94E-01	9.07E-01
**TADA1**	0.05	8.37E-01	1.00E+00	-0.24	4.94E-01	9.07E-01
**LINC01558**	-0.66	5.00E-01	1.00E+00	0.58	4.94E-01	9.07E-01
**LINC00113**	2.50	3.74E-01	1.00E+00	-0.80	4.94E-01	9.07E-01
**AP005329.2**	-0.98	4.12E-01	1.00E+00	-0.45	4.94E-01	9.07E-01
**AGBL2**	-0.69	5.63E-01	1.00E+00	-0.64	4.94E-01	9.07E-01
**AC055720.2**	-0.14	8.27E-01	1.00E+00	0.51	4.95E-01	9.07E-01
**GBP6**	-1.81	1.16E-04	2.76E-02	0.59	4.95E-01	9.07E-01
**GAPDHP1**	-0.10	9.20E-01	1.00E+00	-0.36	4.95E-01	9.07E-01
**PRG4**	-1.03	4.66E-01	1.00E+00	0.56	4.95E-01	9.07E-01
**SNORD89**	-2.65	3.40E-01	1.00E+00	0.48	4.95E-01	9.07E-01
**XPNPEP3**	0.07	8.04E-01	1.00E+00	-0.19	4.95E-01	9.07E-01
**ZMAT1**	0.41	5.08E-01	1.00E+00	-0.42	4.95E-01	9.07E-01
**Z97200.1**	0.60	6.19E-01	1.00E+00	0.62	4.95E-01	9.07E-01
**ST7**	0.03	8.88E-01	1.00E+00	-0.22	4.95E-01	9.07E-01
**C9orf3**	-0.15	3.12E-01	1.00E+00	-0.16	4.95E-01	9.07E-01
**CCNA2**	0.14	7.29E-01	1.00E+00	0.34	4.95E-01	9.07E-01
**NYAP1**	-0.56	2.14E-01	1.00E+00	0.88	4.95E-01	9.07E-01
**BRICD5**	0.11	9.38E-01	1.00E+00	0.80	4.95E-01	9.07E-01
**SOWAHA**	-0.24	7.87E-01	1.00E+00	-0.60	4.95E-01	9.07E-01

**NEMP1**	0.28	5.80E-01	1.00E+00	-0.26	4.95E-01	9.07E-01
**SCO1**	-0.18	3.56E-01	1.00E+00	0.11	4.95E-01	9.07E-01
**GEMIN4**	-0.27	3.50E-01	1.00E+00	-0.17	4.95E-01	9.07E-01
**TMEM11**	-0.09	7.11E-01	1.00E+00	-0.19	4.95E-01	9.07E-01
**AC008610.1**	2.01	2.81E-01	1.00E+00	-0.63	4.95E-01	9.07E-01
**AP001783.1**	0.37	8.64E-01	1.00E+00	-0.79	4.95E-01	9.07E-01
**DTHD1**	-2.79	6.52E-02	1.00E+00	1.04	4.95E-01	9.07E-01
**CLN3**	2.03	8.65E-02	1.00E+00	0.30	4.95E-01	9.07E-01
**LINC01985**	1.32	2.10E-01	1.00E+00	0.69	4.95E-01	9.07E-01
**LUC7L3**	0.35	3.09E-01	1.00E+00	0.21	4.96E-01	9.07E-01
**GPBAR1**	0.78	3.57E-01	1.00E+00	0.49	4.96E-01	9.07E-01
**KRT16P4**	-1.16	2.68E-01	1.00E+00	-0.50	4.96E-01	9.07E-01
**ZNF35**	0.03	9.04E-01	1.00E+00	-0.18	4.96E-01	9.07E-01
**FSIP1**	0.18	8.88E-01	1.00E+00	-0.51	4.96E-01	9.07E-01
**LINC01929**	0.44	6.94E-01	1.00E+00	-1.08	4.96E-01	9.07E-01
**KIAA1522**	-0.29	2.83E-01	1.00E+00	0.34	4.96E-01	9.07E-01
**NLRP10**	0.16	8.49E-01	1.00E+00	0.52	4.96E-01	9.07E-01
**LINC01918**	-0.01	9.84E-01	1.00E+00	-0.66	4.96E-01	9.07E-01
**KIAA1217**	-0.46	6.60E-02	1.00E+00	0.23	4.96E-01	9.07E-01
**AC130324.1**	0.68	7.52E-01	1.00E+00	-0.56	4.96E-01	9.07E-01
**AF129075.1**	-0.62	7.88E-01	1.00E+00	0.70	4.96E-01	9.07E-01
**DNAJA4**	0.01	9.63E-01	1.00E+00	-0.22	4.96E-01	9.07E-01
**LINC00640**	-0.52	1.45E-01	1.00E+00	-0.47	4.96E-01	9.07E-01
**SERTAD1**	-0.75	1.06E-01	1.00E+00	0.23	4.96E-01	9.07E-01
**IWS1**	-0.04	8.03E-01	1.00E+00	0.16	4.96E-01	9.07E-01
**UNC5CL**	0.63	4.78E-01	1.00E+00	0.77	4.96E-01	9.07E-01
**C12orf73**	0.31	5.39E-01	1.00E+00	0.18	4.96E-01	9.07E-01
**MT-ND4L**	-0.16	7.29E-01	1.00E+00	-0.46	4.96E-01	9.07E-01
**ANAPC5**	-0.12	5.94E-01	1.00E+00	-0.14	4.96E-01	9.07E-01
**AP005264.1**	-0.24	8.66E-01	1.00E+00	-0.76	4.96E-01	9.07E-01
**FLT4**	0.13	7.51E-01	1.00E+00	0.47	4.97E-01	9.07E-01
**CDC5L**	-0.07	7.27E-01	1.00E+00	0.08	4.97E-01	9.07E-01

**WWP1**	0.26	2.14E-01	1.00E+00	-0.13	4.97E-01	9.07E-01
**NR2C2AP**	0.21	2.96E-01	1.00E+00	-0.19	4.97E-01	9.07E-01
**IQCD**	0.07	8.63E-01	1.00E+00	0.37	4.97E-01	9.07E-01
**AC007614.1**	0.46	7.13E-01	1.00E+00	0.81	4.97E-01	9.07E-01
**B4GAT1**	0.02	9.29E-01	1.00E+00	-0.12	4.97E-01	9.07E-01
**AL035411.1**	0.98	3.21E-01	1.00E+00	-0.32	4.97E-01	9.07E-01
**AC093799.1**	0.30	5.92E-01	1.00E+00	-0.65	4.97E-01	9.07E-01
**ITPR2**	-0.02	9.40E-01	1.00E+00	0.23	4.97E-01	9.07E-01
**RN7SKP23**	1.07	4.73E-01	1.00E+00	0.75	4.97E-01	9.07E-01
**XLOC_009294**	1.20	4.16E-01	1.00E+00	-1.05	4.97E-01	9.07E-01
**ETFBKMT**	0.19	4.99E-01	1.00E+00	-0.28	4.97E-01	9.07E-01
**PSMC3**	0.02	9.26E-01	1.00E+00	0.12	4.97E-01	9.07E-01
**GTF3C6**	-0.01	9.60E-01	1.00E+00	-0.21	4.97E-01	9.07E-01
**AC097375.1**	-0.02	9.77E-01	1.00E+00	-0.65	4.97E-01	9.07E-01
**GPR137C**	-0.62	3.21E-01	1.00E+00	0.71	4.97E-01	9.07E-01
**AP5B1**	-0.51	1.26E-01	1.00E+00	0.40	4.97E-01	9.07E-01
**EPHB3**	-0.16	4.03E-01	1.00E+00	-0.35	4.97E-01	9.07E-01
**SPSB4**	-0.36	7.10E-01	1.00E+00	0.65	4.97E-01	9.07E-01
**SLC44A3**	0.18	6.16E-01	1.00E+00	-0.24	4.97E-01	9.07E-01
**AC105450.1**	-1.14	6.66E-01	1.00E+00	-0.87	4.97E-01	9.07E-01
**MFSD14C**	-0.14	6.14E-01	1.00E+00	-0.26	4.97E-01	9.07E-01
**AL132989.1**	0.56	6.22E-01	1.00E+00	0.42	4.97E-01	9.07E-01
**MSLN**	0.16	7.78E-01	1.00E+00	-0.67	4.97E-01	9.07E-01
**CD2AP**	-0.06	8.25E-01	1.00E+00	-0.12	4.97E-01	9.07E-01
**ZNHIT6**	0.03	8.84E-01	1.00E+00	-0.11	4.97E-01	9.07E-01
**TMEM216**	0.01	9.84E-01	1.00E+00	-0.20	4.97E-01	9.07E-01
**IGHV4-61**	-0.80	8.18E-01	1.00E+00	2.16	4.98E-01	9.07E-01
**PROK2**	-0.51	8.06E-01	1.00E+00	0.94	4.98E-01	9.07E-01
**XLOC_012574**	0.03	9.93E-01	1.00E+00	0.47	4.98E-01	9.07E-01
**BTBD8**	-0.13	8.26E-01	1.00E+00	0.34	4.98E-01	9.07E-01
**LINC00926**	0.59	5.11E-01	1.00E+00	0.52	4.98E-01	9.07E-01
**TTC29**	-1.84	5.15E-01	1.00E+00	-1.09	4.98E-01	9.07E-01

**ATP7A**	0.14	7.43E-01	1.00E+00	-0.25	4.98E-01	9.07E-01
**SLC8B1**	0.00	9.98E-01	1.00E+00	0.26	4.98E-01	9.07E-01
**ZER1**	-0.16	4.40E-01	1.00E+00	-0.39	4.98E-01	9.07E-01
**AC005899.5**	1.82	3.04E-01	1.00E+00	-0.88	4.98E-01	9.07E-01
**AL512625.2**	0.72	2.39E-01	1.00E+00	0.47	4.98E-01	9.07E-01
**FANCL**	-0.46	1.28E-01	1.00E+00	-0.19	4.98E-01	9.07E-01
**BHMT**	-1.28	3.08E-01	1.00E+00	-0.75	4.98E-01	9.07E-01
**XXYLT1-AS2**	-2.06	5.14E-01	1.00E+00	-0.83	4.98E-01	9.07E-01
**KCNA3**	0.44	7.28E-01	1.00E+00	2.13	4.98E-01	9.07E-01
**SLC25A48**	0.08	8.24E-01	1.00E+00	0.33	4.98E-01	9.07E-01
**EFEMP1**	0.41	9.91E-02	1.00E+00	0.24	4.98E-01	9.07E-01
**WDR11**	0.01	9.34E-01	1.00E+00	0.11	4.98E-01	9.07E-01
**TRAJ39**	NA	NA	NA	-0.69	4.98E-01	9.07E-01
**LINC00342**	0.27	7.81E-01	1.00E+00	0.68	4.98E-01	9.07E-01
**CLIC1**	-0.01	9.81E-01	1.00E+00	0.13	4.98E-01	9.07E-01
**FZD7**	-0.05	9.23E-01	1.00E+00	0.31	4.98E-01	9.07E-01
**HRASLS5**	2.30	5.41E-02	1.00E+00	-2.12	4.98E-01	9.07E-01
**VNN1**	0.02	9.74E-01	1.00E+00	0.62	4.98E-01	9.07E-01
**MIATNB**	-0.16	7.36E-01	1.00E+00	0.18	4.98E-01	9.07E-01
**LAMC3**	0.79	3.84E-02	1.00E+00	0.48	4.98E-01	9.07E-01
**ANKRD9**	-0.21	3.30E-01	1.00E+00	0.47	4.98E-01	9.07E-01
**VDR**	-0.20	5.81E-01	1.00E+00	0.32	4.98E-01	9.07E-01
**NUDT4**	0.20	4.08E-01	1.00E+00	-0.36	4.98E-01	9.07E-01
**RPL21P32**	NA	NA	NA	0.86	4.99E-01	9.07E-01
**HDLBP**	-0.07	7.43E-01	1.00E+00	0.23	4.99E-01	9.07E-01
**AC008537.3**	-0.07	9.55E-01	1.00E+00	-0.91	4.99E-01	9.07E-01
**PDCL3**	0.03	9.09E-01	1.00E+00	-0.23	4.99E-01	9.07E-01
**AC091563.1**	0.31	3.92E-01	1.00E+00	-0.38	4.99E-01	9.07E-01
**MOGAT1**	1.47	3.23E-01	1.00E+00	-2.14	4.99E-01	9.07E-01
**AC128709.1**	-0.73	5.52E-01	1.00E+00	-0.83	4.99E-01	9.07E-01
**G22923**	-0.30	8.24E-01	1.00E+00	-0.86	4.99E-01	9.07E-01
**MTG1**	-1.29	4.86E-01	1.00E+00	0.46	4.99E-01	9.07E-01

**PTK2B**	0.26	3.69E-01	1.00E+00	0.28	4.99E-01	9.07E-01
**CPA5**	-2.70	2.89E-02	9.88E-01	-0.57	4.99E-01	9.07E-01
**AC005183.1**	3.28	3.32E-01	1.00E+00	1.09	4.99E-01	9.07E-01
**PRED62**	0.54	8.35E-01	1.00E+00	1.53	4.99E-01	9.07E-01
**FABP5P1**	0.38	9.13E-01	1.00E+00	0.37	4.99E-01	9.07E-01
**CHCHD10**	0.02	9.75E-01	1.00E+00	0.45	4.99E-01	9.07E-01
**TJAP1**	0.05	8.36E-01	1.00E+00	0.31	4.99E-01	9.07E-01
**RPL4P5**	-0.43	6.60E-01	1.00E+00	-0.39	4.99E-01	9.07E-01
**KRBOX4**	0.17	5.99E-01	1.00E+00	-0.18	4.99E-01	9.07E-01
**TMEM81**	0.43	4.35E-01	1.00E+00	0.42	4.99E-01	9.07E-01
**DLK2**	0.30	4.34E-01	1.00E+00	-0.40	4.99E-01	9.07E-01
**AC007906.2**	-4.19	8.46E-03	5.67E-01	-0.42	4.99E-01	9.07E-01
**ARPC1B**	-0.95	2.09E-01	1.00E+00	0.33	4.99E-01	9.07E-01
**CCDC22**	0.01	9.44E-01	1.00E+00	0.22	4.99E-01	9.07E-01
**CIRBP**	0.23	3.68E-01	1.00E+00	0.17	4.99E-01	9.07E-01
**UQCRC1**	-0.25	1.60E-01	1.00E+00	-0.14	4.99E-01	9.07E-01
**ITPRID2**	0.11	7.61E-01	1.00E+00	0.18	4.99E-01	9.07E-01
**PSMB1**	0.00	9.87E-01	1.00E+00	-0.18	4.99E-01	9.07E-01
**XLOC_012382**	0.83	4.07E-01	1.00E+00	-0.65	5.00E-01	9.07E-01
**AC024896.1**	-0.20	6.18E-01	1.00E+00	0.22	5.00E-01	9.07E-01
**F8**	0.41	4.51E-01	1.00E+00	-0.35	5.00E-01	9.07E-01
**XKR6**	-0.27	4.95E-01	1.00E+00	-0.31	5.00E-01	9.08E-01
**SPATA9**	-1.03	6.76E-01	1.00E+00	-0.52	5.00E-01	9.08E-01
**TRIM11**	-0.21	3.72E-01	1.00E+00	0.35	5.00E-01	9.08E-01
**CCDC90B**	0.16	4.86E-01	1.00E+00	-0.20	5.00E-01	9.08E-01
**FARP2**	0.09	7.02E-01	1.00E+00	0.12	5.00E-01	9.08E-01
**MEFV**	-0.75	4.29E-01	1.00E+00	2.12	5.00E-01	9.08E-01
**MICE**	2.73	1.34E-01	1.00E+00	0.77	5.00E-01	9.08E-01
**ERICD**	0.70	4.81E-01	1.00E+00	0.85	5.00E-01	9.08E-01
**TICAM1**	-0.43	2.31E-01	1.00E+00	0.34	5.00E-01	9.08E-01
**PSPN**	0.17	6.51E-01	1.00E+00	0.28	5.00E-01	9.08E-01
**AL365273.1**	-0.02	9.68E-01	1.00E+00	-0.25	5.00E-01	9.08E-01

**AP001527.2**	-0.47	5.26E-01	1.00E+00	-0.44	5.00E-01	9.08E-01
**RSPH4A**	-0.36	6.96E-01	1.00E+00	0.64	5.00E-01	9.08E-01
**RRAD**	-0.24	6.17E-01	1.00E+00	0.60	5.00E-01	9.08E-01
**BCL3**	-0.65	2.67E-02	9.52E-01	0.42	5.01E-01	9.08E-01
**KNOP1**	-0.18	4.15E-01	1.00E+00	0.17	5.01E-01	9.08E-01
**AC005229.1**	-0.19	6.83E-01	1.00E+00	0.22	5.01E-01	9.08E-01
**FLVCR1-DT**	-0.30	6.92E-01	1.00E+00	0.43	5.01E-01	9.08E-01
**IPO4**	NA	NA	NA	0.41	5.01E-01	9.08E-01
**LINC01132**	-1.36	1.74E-01	1.00E+00	0.68	5.01E-01	9.08E-01
**AC021028.1**	-0.30	8.81E-01	1.00E+00	-0.73	5.01E-01	9.08E-01
**AC008429.1**	-2.30	1.68E-01	1.00E+00	-0.53	5.01E-01	9.08E-01
**XLOC_008613**	2.09	3.70E-01	1.00E+00	-0.68	5.01E-01	9.08E-01
**SIRT4**	-0.81	1.17E-01	1.00E+00	-0.43	5.01E-01	9.09E-01
**EGFLAM**	0.04	9.10E-01	1.00E+00	0.40	5.01E-01	9.09E-01
**AL139005.1**	-2.20	1.66E-01	1.00E+00	-0.86	5.01E-01	9.09E-01
**AC015818.3**	-1.19	6.39E-01	1.00E+00	-0.74	5.01E-01	9.09E-01
**SLC11A2**	0.27	1.81E-01	1.00E+00	0.14	5.02E-01	9.09E-01
**AC008079.1**	1.13	2.24E-01	1.00E+00	-0.56	5.02E-01	9.09E-01
**RP11-435B5.4**	0.38	4.27E-01	1.00E+00	-0.31	5.02E-01	9.09E-01
**MYCBP2**	-0.22	4.01E-01	1.00E+00	0.17	5.02E-01	9.09E-01
**RPS27**	-0.12	7.08E-01	1.00E+00	0.25	5.02E-01	9.09E-01
**PBX1**	0.09	7.99E-01	1.00E+00	0.25	5.02E-01	9.09E-01
**SUPT5H**	-0.24	2.49E-01	1.00E+00	0.23	5.02E-01	9.09E-01
**STN1**	-0.06	7.50E-01	1.00E+00	0.15	5.02E-01	9.09E-01
**SLC34A2**	0.88	4.40E-01	1.00E+00	-2.10	5.02E-01	9.09E-01
**MIR193BHG**	-0.27	4.41E-01	1.00E+00	-0.17	5.02E-01	9.09E-01
**KIF5A**	-0.24	9.39E-01	1.00E+00	0.91	5.02E-01	9.09E-01
**BANCR**	-0.87	8.00E-01	1.00E+00	-1.09	5.02E-01	9.09E-01
**BMPR1A**	0.16	4.25E-01	1.00E+00	-0.16	5.02E-01	9.09E-01
**IGKV1D-13**	2.23	3.38E-01	1.00E+00	2.13	5.02E-01	9.09E-01
**SNURF**	-0.81	8.14E-01	1.00E+00	-0.63	5.02E-01	9.09E-01
**ZEB2-AS1**	-0.49	8.87E-01	1.00E+00	0.61	5.02E-01	9.09E-01

**XLOC_004800**	3.67	8.10E-02	1.00E+00	-2.11	5.02E-01	9.09E-01
**PICALM**	0.02	8.96E-01	1.00E+00	0.26	5.02E-01	9.09E-01
**SEC1P**	-1.74	7.48E-03	5.25E-01	0.59	5.02E-01	9.09E-01
**COMMD2**	0.06	7.50E-01	1.00E+00	-0.20	5.02E-01	9.09E-01
**G32125**	0.41	6.50E-01	1.00E+00	0.66	5.02E-01	9.09E-01
**LINC01772**	-0.30	6.84E-01	1.00E+00	0.47	5.02E-01	9.09E-01
**DNAJC11**	-0.26	2.40E-01	1.00E+00	0.18	5.02E-01	9.09E-01
**FZD2**	-0.05	9.10E-01	1.00E+00	1.92	5.03E-01	9.09E-01
**IGSF1**	1.22	2.32E-02	9.14E-01	0.63	5.03E-01	9.09E-01
**LILRA5**	0.51	7.13E-01	1.00E+00	2.10	5.03E-01	9.10E-01
**AC132942.1**	-2.64	3.29E-01	1.00E+00	0.55	5.03E-01	9.10E-01
**RPSAP36**	1.48	3.96E-01	1.00E+00	0.67	5.03E-01	9.10E-01
**LCP1**	-0.46	2.05E-01	1.00E+00	0.34	5.03E-01	9.10E-01
**TMEM147**	0.00	9.93E-01	1.00E+00	-0.23	5.03E-01	9.10E-01
**RBM44**	-0.38	8.62E-01	1.00E+00	0.83	5.03E-01	9.10E-01
**RAD51B**	-0.10	6.36E-01	1.00E+00	-0.23	5.03E-01	9.10E-01
**SNORA2**	-0.57	8.15E-01	1.00E+00	0.46	5.03E-01	9.10E-01
**AC020907.1**	-0.72	3.68E-01	1.00E+00	-0.57	5.03E-01	9.10E-01
**RECQL5**	0.05	8.46E-01	1.00E+00	0.27	5.03E-01	9.10E-01
**KIAA1210**	1.00	6.64E-01	1.00E+00	-1.09	5.03E-01	9.10E-01
**DMBX1**	0.57	7.19E-01	1.00E+00	-1.22	5.03E-01	9.10E-01
**CTSW**	-0.81	1.69E-01	1.00E+00	1.78	5.03E-01	9.10E-01
**MB21D2**	-0.05	8.37E-01	1.00E+00	0.15	5.04E-01	9.10E-01
**AC096586.2**	-0.45	8.72E-01	1.00E+00	0.75	5.04E-01	9.10E-01
**TM4SF1-AS1**	0.52	7.41E-01	1.00E+00	-0.52	5.04E-01	9.10E-01
**ZNF267**	-0.41	2.43E-01	1.00E+00	0.13	5.04E-01	9.10E-01
**PARP6**	-0.07	7.99E-01	1.00E+00	0.25	5.04E-01	9.10E-01
**PELI2**	0.09	7.86E-01	1.00E+00	-0.24	5.04E-01	9.10E-01
**C10orf71**	-2.80	7.69E-02	1.00E+00	-0.80	5.04E-01	9.10E-01
**ERCC5**	-0.47	2.66E-01	1.00E+00	0.21	5.04E-01	9.10E-01
**SNRPCP2**	0.56	8.71E-01	1.00E+00	0.75	5.04E-01	9.10E-01
**G39663**	0.87	3.55E-01	1.00E+00	-0.48	5.04E-01	9.10E-01

**XLOC_009475**	0.62	7.04E-01	1.00E+00	-0.70	5.04E-01	9.10E-01
**VGLL2**	1.16	3.74E-01	1.00E+00	-1.71	5.04E-01	9.10E-01
**MAMDC2**	0.78	4.06E-02	1.00E+00	-0.33	5.04E-01	9.10E-01
**AC022007.1**	0.65	4.06E-01	1.00E+00	-0.47	5.04E-01	9.10E-01
**FTH1P16**	0.61	7.16E-01	1.00E+00	-0.39	5.04E-01	9.10E-01
**HRC**	0.23	6.65E-01	1.00E+00	1.37	5.04E-01	9.10E-01
**LINC02302**	-0.46	8.05E-01	1.00E+00	0.93	5.04E-01	9.10E-01
**ZNF844**	0.06	8.09E-01	1.00E+00	0.23	5.04E-01	9.10E-01
**INKA2**	0.97	4.20E-01	1.00E+00	0.36	5.04E-01	9.10E-01
**AL049873.1**	-1.54	2.91E-01	1.00E+00	-0.44	5.04E-01	9.10E-01
**FRMD1**	0.67	4.71E-01	1.00E+00	0.63	5.04E-01	9.10E-01
**LRRC14**	-0.22	3.15E-01	1.00E+00	0.17	5.04E-01	9.10E-01
**MAPK1IP1L**	-0.17	2.57E-01	1.00E+00	-0.17	5.04E-01	9.10E-01
**AC073957.3**	1.54	1.03E-01	1.00E+00	-0.75	5.04E-01	9.10E-01
**PPM1J**	-0.17	6.64E-01	1.00E+00	-0.26	5.05E-01	9.10E-01
**XLOC_000133**	-0.38	3.99E-01	1.00E+00	0.46	5.05E-01	9.10E-01
**ANKRD20A11P**	0.42	6.07E-01	1.00E+00	-0.59	5.05E-01	9.11E-01
**HEYL**	-0.48	1.30E-01	1.00E+00	0.41	5.05E-01	9.11E-01
**AC006014.1**	0.13	8.89E-01	1.00E+00	0.43	5.05E-01	9.11E-01
**KLRA1P**	-0.48	7.11E-01	1.00E+00	0.48	5.05E-01	9.11E-01
**ZBTB49**	-0.43	1.87E-01	1.00E+00	0.25	5.05E-01	9.11E-01
**SFPQ**	-0.20	3.84E-01	1.00E+00	-0.33	5.05E-01	9.11E-01
**SEMA6A**	0.16	7.36E-01	1.00E+00	0.20	5.05E-01	9.11E-01
**TDRD1**	1.73	1.39E-01	1.00E+00	-0.63	5.05E-01	9.11E-01
**HAS1**	-0.59	4.42E-01	1.00E+00	0.42	5.05E-01	9.11E-01
**AL357093.2**	-2.03	2.93E-01	1.00E+00	0.56	5.05E-01	9.11E-01
**G33807**	-1.68	3.34E-01	1.00E+00	0.82	5.05E-01	9.11E-01
**MALT1**	-0.17	5.93E-01	1.00E+00	-0.28	5.05E-01	9.11E-01
**CFL1P1**	0.38	4.16E-01	1.00E+00	-0.37	5.05E-01	9.11E-01
**ZNF566**	0.21	4.45E-01	1.00E+00	-0.19	5.06E-01	9.11E-01
**CAPN8**	-1.74	8.42E-02	1.00E+00	0.80	5.06E-01	9.11E-01
**MMP17**	0.20	6.61E-01	1.00E+00	0.57	5.06E-01	9.11E-01

**KIAA1147**	0.30	4.48E-01	1.00E+00	0.32	5.06E-01	9.11E-01
**CNOT4**	0.02	9.28E-01	1.00E+00	-0.17	5.06E-01	9.11E-01
**AC114296.1**	-0.38	2.76E-01	1.00E+00	0.57	5.06E-01	9.11E-01
**ARID4A**	0.09	6.37E-01	1.00E+00	-0.12	5.06E-01	9.11E-01
**AL122020.1**	-0.59	8.47E-01	1.00E+00	-2.09	5.06E-01	9.11E-01
**CTB-178M22.2**	-0.37	7.12E-01	1.00E+00	-0.54	5.06E-01	9.11E-01
**UBL3**	0.42	1.13E-01	1.00E+00	-0.15	5.06E-01	9.11E-01
**PCDHB8**	-1.95	7.85E-03	5.45E-01	-1.40	5.06E-01	9.11E-01
**GOSR2**	0.25	3.76E-01	1.00E+00	-0.14	5.06E-01	9.11E-01
**AP005435.1**	-1.28	7.75E-02	1.00E+00	-0.62	5.06E-01	9.11E-01
**HMGN5**	-0.09	8.00E-01	1.00E+00	-0.23	5.06E-01	9.11E-01
**ALPK1**	-0.14	4.44E-01	1.00E+00	0.14	5.06E-01	9.11E-01
**HTR4**	1.57	1.30E-01	1.00E+00	-0.94	5.06E-01	9.11E-01
**C3orf14**	-0.55	2.19E-02	8.96E-01	-0.28	5.06E-01	9.11E-01
**STAP2**	-0.31	4.49E-01	1.00E+00	0.28	5.06E-01	9.11E-01
**SF3B5**	-0.17	3.89E-01	1.00E+00	-0.11	5.06E-01	9.11E-01
**TRA2A**	0.03	8.97E-01	1.00E+00	-0.12	5.06E-01	9.11E-01
**TRMT61A**	-0.23	3.47E-01	1.00E+00	0.31	5.06E-01	9.11E-01
**C1DP5**	-2.07	4.80E-01	1.00E+00	1.11	5.06E-01	9.11E-01
**DNAAF3**	-3.69	6.03E-05	1.57E-02	0.34	5.06E-01	9.11E-01
**JPH2**	-0.30	6.64E-01	1.00E+00	-0.52	5.06E-01	9.11E-01
**PIAS1**	-0.05	8.08E-01	1.00E+00	-0.22	5.07E-01	9.11E-01
**PSAT1**	-0.07	8.60E-01	1.00E+00	-0.30	5.07E-01	9.11E-01
**AL118516.1**	-0.48	3.40E-01	1.00E+00	-0.30	5.07E-01	9.11E-01
**ROBO1**	-0.23	4.58E-01	1.00E+00	0.28	5.07E-01	9.11E-01
**RIPK1**	-0.07	7.50E-01	1.00E+00	0.26	5.07E-01	9.11E-01
**NPM2**	-0.50	3.68E-01	1.00E+00	-0.39	5.07E-01	9.11E-01
**G32314**	-1.32	3.45E-03	3.14E-01	0.48	5.07E-01	9.11E-01
**CLLU1**	-0.70	8.41E-01	1.00E+00	1.11	5.07E-01	9.11E-01
**TAOK2**	-0.18	3.59E-01	1.00E+00	0.40	5.07E-01	9.11E-01
**AL359955.1**	2.90	1.67E-01	1.00E+00	-1.01	5.07E-01	9.11E-01
**ELAC1**	-0.05	8.28E-01	1.00E+00	-0.22	5.07E-01	9.11E-01

**DNAJB12**	-0.17	3.43E-01	1.00E+00	0.10	5.07E-01	9.11E-01
**YJEFN3**	-2.25	1.13E-01	1.00E+00	-0.64	5.07E-01	9.11E-01
**NCK1**	0.12	5.47E-01	1.00E+00	-0.18	5.07E-01	9.11E-01
**CORO6**	-0.80	6.65E-02	1.00E+00	0.50	5.07E-01	9.11E-01
**KAT5**	-0.28	1.67E-01	1.00E+00	0.13	5.07E-01	9.11E-01
**TEAD1**	0.12	5.93E-01	1.00E+00	0.22	5.07E-01	9.11E-01
**GSTO2**	-0.09	7.20E-01	1.00E+00	-0.34	5.07E-01	9.11E-01
**RBM10**	-0.06	7.89E-01	1.00E+00	0.21	5.07E-01	9.11E-01
**UBXN2A**	0.02	9.27E-01	1.00E+00	-0.13	5.07E-01	9.11E-01
**INAFM2**	0.06	8.22E-01	1.00E+00	0.42	5.07E-01	9.11E-01
**HSP90B2P**	-2.73	7.46E-02	1.00E+00	0.28	5.07E-01	9.11E-01
**PSKH1**	-0.05	8.16E-01	1.00E+00	0.36	5.07E-01	9.11E-01
**C6**	1.73	1.01E-01	1.00E+00	0.72	5.07E-01	9.11E-01
**DIABLO**	-0.81	3.33E-01	1.00E+00	0.36	5.07E-01	9.11E-01
**IGSF11**	0.12	8.16E-01	1.00E+00	0.50	5.07E-01	9.11E-01
**CLEC10A**	-0.52	3.32E-01	1.00E+00	0.43	5.07E-01	9.11E-01
**SNHG1**	0.28	4.13E-01	1.00E+00	0.31	5.07E-01	9.11E-01
**TBC1D7**	-0.16	4.82E-01	1.00E+00	-0.25	5.07E-01	9.11E-01
**XLOC_011308**	-0.41	7.68E-01	1.00E+00	1.11	5.08E-01	9.11E-01
**RPS7**	-0.16	6.27E-01	1.00E+00	0.15	5.08E-01	9.11E-01
**ITFG1**	0.00	9.79E-01	1.00E+00	-0.18	5.08E-01	9.11E-01
**MSH5**	2.79	2.85E-01	1.00E+00	0.58	5.08E-01	9.11E-01
**RHOBTB1**	0.42	2.02E-01	1.00E+00	0.41	5.08E-01	9.11E-01
**GPX2**	-0.40	1.14E-01	1.00E+00	0.30	5.08E-01	9.11E-01
**MIR646HG**	-0.35	7.25E-01	1.00E+00	-0.49	5.08E-01	9.11E-01
**CASKIN2**	-0.46	2.61E-02	9.44E-01	0.39	5.08E-01	9.11E-01
**GTF2B**	0.07	7.07E-01	1.00E+00	-0.14	5.08E-01	9.11E-01
**YME1L1**	0.01	9.75E-01	1.00E+00	-0.12	5.08E-01	9.11E-01
**UBAP2L**	-0.18	3.10E-01	1.00E+00	0.15	5.08E-01	9.11E-01
**AC099850.1**	0.10	8.47E-01	1.00E+00	-0.31	5.08E-01	9.11E-01
**RPS26P3**	2.09	1.15E-01	1.00E+00	0.49	5.08E-01	9.11E-01
**BIRC6-AS2**	-0.18	9.59E-01	1.00E+00	-0.89	5.08E-01	9.11E-01

**AC023090.2**	-1.73	5.28E-01	1.00E+00	0.81	5.08E-01	9.11E-01
**PITPNC1**	0.15	7.77E-01	1.00E+00	0.54	5.08E-01	9.11E-01
**MOSPD1**	0.16	6.42E-01	1.00E+00	-0.27	5.08E-01	9.11E-01
**LEPROTL1**	0.19	4.31E-01	1.00E+00	-0.16	5.08E-01	9.11E-01
**VASN**	0.00	9.90E-01	1.00E+00	0.45	5.08E-01	9.11E-01
**FAM133DP**	-0.79	1.61E-01	1.00E+00	-0.24	5.08E-01	9.11E-01
**ITGAL**	-0.66	3.08E-01	1.00E+00	2.07	5.08E-01	9.11E-01
**LINC01894**	0.76	7.69E-01	1.00E+00	-1.14	5.09E-01	9.11E-01
**SLCO1C1**	0.34	6.15E-01	1.00E+00	-0.53	5.09E-01	9.11E-01
**LBX2-AS1**	0.61	2.17E-01	1.00E+00	0.35	5.09E-01	9.11E-01
**MAG**	-3.81	1.25E-03	1.89E-01	-0.88	5.09E-01	9.11E-01
**CNPY2**	-1.01	1.29E-01	1.00E+00	-0.30	5.09E-01	9.11E-01
**AC002454.1**	2.65	7.27E-02	1.00E+00	-0.62	5.09E-01	9.11E-01
**AC092070.2**	-0.75	5.59E-02	1.00E+00	0.27	5.09E-01	9.11E-01
**ZNF778**	0.08	7.65E-01	1.00E+00	0.17	5.09E-01	9.11E-01
**KCNRG**	-2.36	1.64E-01	1.00E+00	-0.41	5.09E-01	9.11E-01
**TNFSF9**	-1.02	1.17E-01	1.00E+00	-0.63	5.09E-01	9.11E-01
**MOXD1**	0.03	9.53E-01	1.00E+00	0.33	5.09E-01	9.11E-01
**FNDC11**	-1.88	1.26E-01	1.00E+00	-0.76	5.09E-01	9.11E-01
**YIPF1**	0.16	4.40E-01	1.00E+00	-0.07	5.09E-01	9.11E-01
**XLOC_001415**	0.58	5.08E-01	1.00E+00	0.74	5.09E-01	9.12E-01
**AC006042.2**	0.48	8.76E-01	1.00E+00	0.39	5.09E-01	9.12E-01
**ANP32A**	0.10	7.36E-01	1.00E+00	0.17	5.09E-01	9.12E-01
**PSMG1**	0.15	5.51E-01	1.00E+00	-0.17	5.09E-01	9.12E-01
**LMBRD1**	0.28	1.92E-01	1.00E+00	-0.24	5.09E-01	9.12E-01
**MUC7**	-0.47	7.16E-01	1.00E+00	-0.57	5.09E-01	9.12E-01
**CENPJ**	-0.40	2.24E-01	1.00E+00	0.27	5.09E-01	9.12E-01
**MYBL1**	-0.22	6.21E-01	1.00E+00	-0.46	5.10E-01	9.12E-01
**AL138724.1**	-0.77	3.53E-01	1.00E+00	0.38	5.10E-01	9.12E-01
**GRIK2**	-0.20	6.16E-01	1.00E+00	-0.47	5.10E-01	9.12E-01
**FAR1**	0.10	7.59E-01	1.00E+00	-0.16	5.10E-01	9.12E-01
**AC006252.1**	-1.57	8.41E-02	1.00E+00	-0.68	5.10E-01	9.12E-01

**ZNF248**	0.43	2.47E-01	1.00E+00	-0.29	5.10E-01	9.12E-01
**ACTN1-AS1**	-1.00	7.69E-01	1.00E+00	-0.91	5.10E-01	9.12E-01
**HDHD5-AS1**	-0.18	8.79E-01	1.00E+00	-0.52	5.10E-01	9.12E-01
**AL590428.1**	-0.56	2.42E-01	1.00E+00	0.42	5.10E-01	9.12E-01
**POMGNT1**	-0.07	7.46E-01	1.00E+00	-0.30	5.10E-01	9.12E-01
**CHRM5**	-2.50	4.61E-02	1.00E+00	-0.82	5.10E-01	9.12E-01
**ABCC6**	1.96	5.48E-02	1.00E+00	-0.51	5.10E-01	9.12E-01
**AC139795.3**	0.57	5.70E-01	1.00E+00	-0.59	5.10E-01	9.12E-01
**LDLRAD4**	0.54	2.95E-01	1.00E+00	0.27	5.10E-01	9.12E-01
**MAGED2**	-0.16	5.23E-01	1.00E+00	0.12	5.10E-01	9.12E-01
**DDX47**	1.27	6.79E-01	1.00E+00	-0.86	5.10E-01	9.12E-01
**HNRNPA1P35**	0.40	7.67E-01	1.00E+00	-0.33	5.10E-01	9.12E-01
**G39214**	0.41	6.92E-01	1.00E+00	0.36	5.10E-01	9.12E-01
**LINC00638**	-0.79	2.64E-01	1.00E+00	-0.83	5.10E-01	9.12E-01
**PRDM2**	-0.23	2.54E-01	1.00E+00	0.21	5.10E-01	9.12E-01
**DAG1**	-0.12	5.65E-01	1.00E+00	0.21	5.10E-01	9.12E-01
**SOD3**	0.69	7.07E-02	1.00E+00	0.52	5.10E-01	9.12E-01
**USHBP1**	0.37	4.68E-01	1.00E+00	-0.48	5.10E-01	9.12E-01
**UFC1**	-0.08	7.72E-01	1.00E+00	-0.19	5.10E-01	9.12E-01
**ARHGEF15**	0.06	9.01E-01	1.00E+00	0.44	5.11E-01	9.12E-01
**MICU1**	0.02	9.27E-01	1.00E+00	0.13	5.11E-01	9.12E-01
**SF3A2**	-0.44	1.39E-01	1.00E+00	0.34	5.11E-01	9.12E-01
**AC024293.1**	-0.24	5.36E-01	1.00E+00	0.30	5.11E-01	9.12E-01
**PRDM4**	-0.11	5.80E-01	1.00E+00	0.28	5.11E-01	9.12E-01
**BIRC6**	-0.15	4.12E-01	1.00E+00	0.18	5.11E-01	9.12E-01
**ATG5**	0.24	2.40E-01	1.00E+00	-0.16	5.11E-01	9.12E-01
**AC131009.1**	-0.75	7.58E-01	1.00E+00	0.63	5.11E-01	9.12E-01
**PTAR1**	0.11	7.24E-01	1.00E+00	-0.12	5.11E-01	9.12E-01
**CD200R1**	-0.38	4.31E-01	1.00E+00	0.31	5.11E-01	9.12E-01
**AL590399.1**	0.71	5.68E-01	1.00E+00	-0.51	5.11E-01	9.12E-01
**PDCD6IPP1**	-2.12	3.06E-01	1.00E+00	0.40	5.11E-01	9.12E-01
**ZNF789**	0.05	8.62E-01	1.00E+00	-0.25	5.11E-01	9.12E-01

**CTD-2145A24.3**	0.32	8.33E-01	1.00E+00	-0.61	5.11E-01	9.12E-01
**COX17P1**	NA	NA	NA	-0.51	5.11E-01	9.12E-01
**AL157400.3**	0.63	6.98E-01	1.00E+00	-0.82	5.11E-01	9.12E-01
**AC019171.1**	3.16	3.44E-01	1.00E+00	0.67	5.11E-01	9.12E-01
**AMH**	-1.52	4.47E-01	1.00E+00	0.80	5.11E-01	9.12E-01
**AL645504.1**	-0.02	9.94E-01	1.00E+00	-0.97	5.11E-01	9.12E-01
**BASP1-AS1**	0.16	8.85E-01	1.00E+00	-0.93	5.11E-01	9.12E-01
**AL109811.3**	2.72	1.47E-01	1.00E+00	-0.42	5.11E-01	9.12E-01
**ARHGAP5**	-0.13	5.88E-01	1.00E+00	-0.15	5.11E-01	9.12E-01
**PIH1D2**	-0.33	5.12E-01	1.00E+00	-0.32	5.11E-01	9.12E-01
**AL606763.1**	-3.07	1.34E-01	1.00E+00	0.89	5.11E-01	9.12E-01
**G26658**	-0.26	8.60E-01	1.00E+00	-0.70	5.11E-01	9.12E-01
**PTCD2**	0.12	5.56E-01	1.00E+00	0.19	5.11E-01	9.12E-01
**SIGLEC11**	-2.36	6.43E-02	1.00E+00	1.03	5.12E-01	9.12E-01
**TNFAIP2**	-0.14	7.59E-01	1.00E+00	0.48	5.12E-01	9.12E-01
**MSRA**	-0.30	2.82E-01	1.00E+00	0.20	5.12E-01	9.12E-01
**SMAGP**	-0.34	2.75E-01	1.00E+00	-0.19	5.12E-01	9.12E-01
**ERAP2**	0.23	8.45E-01	1.00E+00	0.38	5.12E-01	9.12E-01
**IGKV2D-28**	4.11	1.01E-02	6.26E-01	2.07	5.12E-01	9.12E-01
**PODXL**	0.06	9.04E-01	1.00E+00	0.35	5.12E-01	9.12E-01
**AFDN-DT**	1.16	1.24E-01	1.00E+00	-0.25	5.12E-01	9.12E-01
**RDH8**	2.43	4.71E-01	1.00E+00	-1.26	5.12E-01	9.12E-01
**C9orf16**	-0.33	1.90E-01	1.00E+00	0.15	5.12E-01	9.12E-01
**PLD5**	0.36	6.98E-01	1.00E+00	-0.72	5.12E-01	9.12E-01
**H1FX-AS1**	-0.15	8.23E-01	1.00E+00	-0.34	5.12E-01	9.12E-01
**XLOC_006941**	-2.04	2.77E-01	1.00E+00	0.94	5.12E-01	9.12E-01
**ASB14**	0.67	5.00E-01	1.00E+00	0.29	5.12E-01	9.12E-01
**EIF4A2P1**	0.41	7.98E-01	1.00E+00	0.68	5.12E-01	9.12E-01
**TUBB8P12**	1.68	3.44E-01	1.00E+00	-0.61	5.12E-01	9.12E-01
**FZD8**	-0.40	3.84E-01	1.00E+00	0.56	5.12E-01	9.12E-01
**HSPA14**	-0.10	6.79E-01	1.00E+00	0.11	5.12E-01	9.12E-01
**RSL24D1P6**	-1.26	3.22E-01	1.00E+00	0.71	5.12E-01	9.12E-01

**FAM83D**	0.30	5.55E-01	1.00E+00	-0.38	5.12E-01	9.12E-01
**TCTEX1D2**	-1.22	4.63E-01	1.00E+00	-0.52	5.12E-01	9.12E-01
**ACTA2-AS1**	-1.81	5.86E-01	1.00E+00	0.56	5.12E-01	9.12E-01
**GTPBP1**	-0.26	2.85E-01	1.00E+00	0.38	5.13E-01	9.12E-01
**ZNF770**	0.04	9.02E-01	1.00E+00	-0.24	5.13E-01	9.12E-01
**AC008440.1**	0.39	8.39E-01	1.00E+00	0.73	5.13E-01	9.13E-01
**MAGI2**	0.07	8.55E-01	1.00E+00	-0.32	5.13E-01	9.13E-01
**TDP2**	0.02	8.98E-01	1.00E+00	0.11	5.13E-01	9.13E-01
**PNKP**	-0.22	4.17E-01	1.00E+00	0.18	5.13E-01	9.13E-01
**DERA**	-0.15	5.08E-01	1.00E+00	-0.21	5.13E-01	9.13E-01
**LINC01208**	-2.09	5.41E-01	1.00E+00	1.18	5.13E-01	9.13E-01
**PDIA3**	-0.06	7.23E-01	1.00E+00	0.08	5.13E-01	9.13E-01
**AL133445.2**	0.30	9.13E-01	1.00E+00	0.76	5.13E-01	9.13E-01
**ZNF41**	-0.21	2.33E-01	1.00E+00	-0.18	5.13E-01	9.13E-01
**AC008494.1**	-2.47	2.25E-01	1.00E+00	-0.77	5.13E-01	9.13E-01
**AC004922.1**	0.42	8.41E-01	1.00E+00	-0.87	5.13E-01	9.13E-01
**XLOC_013770**	-0.28	7.60E-01	1.00E+00	0.47	5.13E-01	9.13E-01
**FLI1**	0.25	5.70E-01	1.00E+00	0.40	5.13E-01	9.13E-01
**AC092162.2**	-1.34	2.60E-01	1.00E+00	0.73	5.13E-01	9.13E-01
**ZNF24**	0.07	7.09E-01	1.00E+00	0.09	5.13E-01	9.13E-01
**ALOX12B**	0.14	8.31E-01	1.00E+00	0.46	5.14E-01	9.13E-01
**BUD13P1**	-2.36	1.35E-01	1.00E+00	-0.98	5.14E-01	9.13E-01
**PSMA3-AS1**	0.11	6.28E-01	1.00E+00	-0.13	5.14E-01	9.13E-01
**GABPB2**	-0.05	8.42E-01	1.00E+00	-0.22	5.14E-01	9.13E-01
**SLC38A4**	-1.37	3.75E-03	3.33E-01	0.39	5.14E-01	9.13E-01
**AC004908.3**	-1.32	6.96E-01	1.00E+00	0.90	5.14E-01	9.13E-01
**ZFP62**	-0.26	2.53E-01	1.00E+00	-0.15	5.14E-01	9.13E-01
**AKAP6**	-0.67	7.66E-02	1.00E+00	-0.39	5.14E-01	9.14E-01
**RPS20P14**	0.22	7.98E-01	1.00E+00	-0.46	5.14E-01	9.14E-01
**SPNS2**	-0.04	9.23E-01	1.00E+00	0.35	5.14E-01	9.14E-01
**PHF7**	-0.04	8.86E-01	1.00E+00	-0.21	5.14E-01	9.14E-01
**SLC9A2**	-0.80	2.49E-01	1.00E+00	-0.56	5.14E-01	9.14E-01

**RNASEL**	0.05	8.77E-01	1.00E+00	0.19	5.14E-01	9.14E-01
**STAT6**	-0.16	5.09E-01	1.00E+00	0.26	5.14E-01	9.14E-01
**MYO18A**	-0.40	1.90E-01	1.00E+00	0.41	5.14E-01	9.14E-01
**ARRDC3**	0.41	4.99E-02	1.00E+00	0.21	5.14E-01	9.14E-01
**CAB39**	-0.13	6.19E-01	1.00E+00	-0.25	5.14E-01	9.14E-01
**ANTXR2**	0.71	7.76E-02	1.00E+00	0.38	5.14E-01	9.14E-01
**DDX11**	0.41	2.10E-01	1.00E+00	0.30	5.15E-01	9.14E-01
**VSTM2L**	-0.75	4.68E-02	1.00E+00	0.55	5.15E-01	9.14E-01
**AL591438.2**	-0.72	8.35E-01	1.00E+00	0.81	5.15E-01	9.14E-01
**CLDN19**	-0.36	6.60E-01	1.00E+00	-0.87	5.15E-01	9.14E-01
**ADGRG6**	0.30	3.63E-01	1.00E+00	-0.28	5.15E-01	9.14E-01
**MCOLN2**	-0.37	6.55E-01	1.00E+00	0.61	5.15E-01	9.14E-01
**HIGD1AP1**	-0.81	8.14E-01	1.00E+00	0.53	5.15E-01	9.14E-01
**LCMT1-AS2**	0.16	8.98E-01	1.00E+00	0.93	5.15E-01	9.14E-01
**MAGT1**	0.02	9.10E-01	1.00E+00	0.17	5.15E-01	9.14E-01
**CRIP1**	0.56	3.95E-01	1.00E+00	0.33	5.15E-01	9.14E-01
**ERICH1**	-0.22	3.85E-01	1.00E+00	0.13	5.15E-01	9.14E-01
**G34413**	-0.29	8.05E-01	1.00E+00	-0.49	5.15E-01	9.14E-01
**AP001877.1**	-0.81	2.81E-01	1.00E+00	0.30	5.15E-01	9.14E-01
**ZNF821**	-0.52	4.15E-01	1.00E+00	-0.21	5.15E-01	9.14E-01
**AL731533.2**	-1.97	5.09E-01	1.00E+00	-0.98	5.16E-01	9.14E-01
**G29539**	1.16	2.43E-01	1.00E+00	0.73	5.16E-01	9.14E-01
**EIF4E**	0.09	7.08E-01	1.00E+00	-0.18	5.16E-01	9.14E-01
**FLRT2**	0.24	5.65E-01	1.00E+00	0.24	5.16E-01	9.14E-01
**INAVA**	-0.26	5.49E-01	1.00E+00	-0.42	5.16E-01	9.14E-01
**LAMTOR3**	0.29	8.66E-02	1.00E+00	-0.21	5.16E-01	9.14E-01
**OR2A20P**	0.51	3.84E-01	1.00E+00	-0.32	5.16E-01	9.14E-01
**LINC01088**	-0.99	3.01E-01	1.00E+00	-0.52	5.16E-01	9.14E-01
**AC010359.1**	-1.53	3.16E-01	1.00E+00	-0.84	5.16E-01	9.14E-01
**THRA**	-0.09	7.71E-01	1.00E+00	0.34	5.16E-01	9.14E-01
**PIGL**	-0.36	1.70E-01	1.00E+00	-0.17	5.16E-01	9.14E-01
**DYNLT1**	-0.15	4.44E-01	1.00E+00	0.15	5.16E-01	9.14E-01

**PRKCH**	0.36	6.85E-02	1.00E+00	0.11	5.16E-01	9.15E-01
**RBM41**	-0.18	4.44E-01	1.00E+00	-0.16	5.16E-01	9.15E-01
**AL359710.1**	1.68	1.52E-01	1.00E+00	-0.44	5.16E-01	9.15E-01
**TMEM238**	-0.19	5.74E-01	1.00E+00	-0.67	5.16E-01	9.15E-01
**AL133523.1**	-1.21	4.66E-01	1.00E+00	-0.51	5.16E-01	9.15E-01
**KLF6**	0.23	4.05E-01	1.00E+00	0.15	5.16E-01	9.15E-01
**MDM1**	0.22	4.73E-01	1.00E+00	-0.27	5.16E-01	9.15E-01
**RTN2**	-0.47	4.36E-02	1.00E+00	0.15	5.17E-01	9.15E-01
**ARHGEF33**	0.09	9.30E-01	1.00E+00	-0.56	5.17E-01	9.15E-01
**WNK1**	-0.22	3.68E-01	1.00E+00	0.24	5.17E-01	9.15E-01
**XCR1**	0.10	8.76E-01	1.00E+00	0.43	5.17E-01	9.15E-01
**AC114341.1**	-2.07	1.32E-01	1.00E+00	0.70	5.17E-01	9.15E-01
**RIMKLB**	0.19	5.67E-01	1.00E+00	0.18	5.17E-01	9.15E-01
**SDHAP1**	0.09	8.46E-01	1.00E+00	0.22	5.17E-01	9.15E-01
**FMO3**	-0.55	3.27E-01	1.00E+00	0.54	5.17E-01	9.15E-01
**AC244034.3**	-0.11	9.74E-01	1.00E+00	1.04	5.17E-01	9.15E-01
**BIK**	-0.02	9.74E-01	1.00E+00	0.33	5.17E-01	9.15E-01
**AC021016.2**	-0.04	8.82E-01	1.00E+00	0.32	5.17E-01	9.15E-01
**HHATL**	-1.88	3.13E-02	1.00E+00	-0.74	5.17E-01	9.15E-01
**HMGN4**	0.03	8.81E-01	1.00E+00	-0.09	5.17E-01	9.15E-01
**G39184**	-0.17	9.61E-01	1.00E+00	-0.87	5.17E-01	9.15E-01
**C19orf47**	-0.49	2.66E-02	9.50E-01	0.26	5.17E-01	9.15E-01
**LINC01569**	-0.11	8.18E-01	1.00E+00	-0.44	5.17E-01	9.15E-01
**XLOC_003985**	0.23	9.33E-01	1.00E+00	-0.79	5.17E-01	9.15E-01
**GLRX2**	0.24	2.09E-01	1.00E+00	-0.20	5.17E-01	9.15E-01
**ATP5MC2**	-0.16	3.67E-01	1.00E+00	-0.19	5.17E-01	9.15E-01
**EGFR**	-0.19	4.33E-01	1.00E+00	-0.21	5.17E-01	9.15E-01
**CRLF3**	0.39	2.17E-01	1.00E+00	0.19	5.18E-01	9.15E-01
**HCCS**	0.09	6.56E-01	1.00E+00	-0.16	5.18E-01	9.16E-01
**SNTA1**	-0.11	7.38E-01	1.00E+00	-0.40	5.18E-01	9.16E-01
**CHIT1**	0.27	8.35E-01	1.00E+00	2.03	5.18E-01	9.16E-01
**G31837**	0.04	9.78E-01	1.00E+00	-0.53	5.18E-01	9.16E-01

**AC126175.1**	-0.41	7.92E-01	1.00E+00	0.79	5.18E-01	9.16E-01
**AC006077.2**	-0.17	9.01E-01	1.00E+00	-0.49	5.18E-01	9.16E-01
**MYEOV**	0.34	5.85E-01	1.00E+00	-0.40	5.18E-01	9.16E-01
**SLAMF6**	-1.39	1.23E-01	1.00E+00	2.03	5.18E-01	9.16E-01
**OMG**	-0.37	7.26E-01	1.00E+00	0.69	5.18E-01	9.16E-01
**AC011330.2**	-0.07	9.24E-01	1.00E+00	-0.84	5.18E-01	9.16E-01
**RIBC1**	-0.36	3.04E-01	1.00E+00	-0.29	5.18E-01	9.16E-01
**ANKRD29**	0.07	8.74E-01	1.00E+00	-0.34	5.18E-01	9.16E-01
**HNRNPA1P27**	0.27	7.20E-01	1.00E+00	0.41	5.18E-01	9.16E-01
**NHLRC4**	0.34	6.43E-01	1.00E+00	0.73	5.18E-01	9.16E-01
**AC117395.1**	0.29	7.78E-01	1.00E+00	0.77	5.18E-01	9.16E-01
**METTL2A**	-0.22	3.82E-01	1.00E+00	0.18	5.18E-01	9.16E-01
**XLOC_000152**	-1.60	3.33E-01	1.00E+00	0.68	5.18E-01	9.16E-01
**TIMM8BP2**	0.56	8.71E-01	1.00E+00	-0.62	5.19E-01	9.16E-01
**BNIP3P1**	-0.36	8.31E-01	1.00E+00	-0.45	5.19E-01	9.16E-01
**RABGGTB**	0.37	2.18E-01	1.00E+00	0.14	5.19E-01	9.16E-01
**PNPLA2**	0.86	1.49E-01	1.00E+00	-0.37	5.19E-01	9.16E-01
**MYH2**	3.15	5.19E-02	1.00E+00	-1.08	5.19E-01	9.16E-01
**RAB15**	-0.62	2.61E-02	9.44E-01	0.43	5.19E-01	9.16E-01
**AC009962.1**	-0.52	3.47E-01	1.00E+00	0.47	5.19E-01	9.16E-01
**AL078459.1**	-1.45	3.65E-01	1.00E+00	0.41	5.19E-01	9.16E-01
**XLOC_013235**	0.35	6.78E-01	1.00E+00	0.58	5.19E-01	9.16E-01
**PI4KB**	-0.15	3.98E-01	1.00E+00	0.16	5.19E-01	9.16E-01
**SLC38A10**	-0.16	6.53E-01	1.00E+00	0.41	5.19E-01	9.16E-01
**DPF2**	0.07	7.30E-01	1.00E+00	0.13	5.19E-01	9.16E-01
**CACNB1**	-0.26	5.20E-01	1.00E+00	-0.44	5.19E-01	9.16E-01
**IL1F10**	-0.02	9.77E-01	1.00E+00	0.44	5.19E-01	9.16E-01
**EXOSC10**	-0.13	4.85E-01	1.00E+00	0.12	5.19E-01	9.16E-01
**PMS2P2**	1.26	3.41E-01	1.00E+00	-0.37	5.19E-01	9.16E-01
**LONP1**	-0.13	5.52E-01	1.00E+00	0.35	5.19E-01	9.16E-01
**POP1**	-0.07	8.30E-01	1.00E+00	0.26	5.19E-01	9.16E-01
**AL049775.1**	-1.82	1.45E-01	1.00E+00	0.45	5.19E-01	9.16E-01

**MCF2L**	0.18	2.98E-01	1.00E+00	-0.34	5.19E-01	9.16E-01
**SPTBN5**	-0.48	3.91E-01	1.00E+00	0.42	5.19E-01	9.16E-01
**AP002800.1**	-0.56	4.54E-01	1.00E+00	0.45	5.19E-01	9.16E-01
**RABGEF1**	-0.35	2.73E-01	1.00E+00	0.24	5.19E-01	9.16E-01
**AC007389.1**	0.01	9.97E-01	1.00E+00	0.78	5.19E-01	9.16E-01
**TCF21**	-3.50	4.56E-02	1.00E+00	-0.87	5.19E-01	9.16E-01
**PFKM**	0.07	8.07E-01	1.00E+00	0.23	5.19E-01	9.16E-01
**DAPK2**	-0.12	7.37E-01	1.00E+00	-0.26	5.20E-01	9.16E-01
**MIR1245A**	0.03	9.93E-01	1.00E+00	0.84	5.20E-01	9.16E-01
**VAPB**	0.33	3.71E-01	1.00E+00	-0.11	5.20E-01	9.16E-01
**ASB16-AS1**	-0.13	7.63E-01	1.00E+00	-0.23	5.20E-01	9.16E-01
**PER1**	-1.02	9.46E-02	1.00E+00	-0.36	5.20E-01	9.16E-01
**AC012617.1**	1.38	4.76E-01	1.00E+00	-0.79	5.20E-01	9.16E-01
**SLC46A1**	0.56	4.41E-01	1.00E+00	0.30	5.20E-01	9.16E-01
**RPL41P2**	-0.59	4.07E-01	1.00E+00	-0.39	5.20E-01	9.16E-01
**ANXA6**	0.51	3.27E-01	1.00E+00	0.30	5.20E-01	9.16E-01
**METTL21A**	-0.09	7.76E-01	1.00E+00	-0.17	5.20E-01	9.16E-01
**CNN2P9**	-2.16	9.06E-02	1.00E+00	-1.06	5.20E-01	9.16E-01
**C1orf105**	-3.18	1.56E-02	7.78E-01	-0.70	5.20E-01	9.16E-01
**FAM153A**	0.83	2.40E-01	1.00E+00	-0.44	5.20E-01	9.16E-01
**KDR**	0.41	4.50E-01	1.00E+00	0.40	5.20E-01	9.16E-01
**ZDHHC16**	0.09	6.63E-01	1.00E+00	-0.26	5.20E-01	9.17E-01
**FOXC1**	-0.46	2.98E-01	1.00E+00	0.32	5.20E-01	9.17E-01
**WDR53**	0.16	6.34E-01	1.00E+00	-0.14	5.20E-01	9.17E-01
**EID3**	-0.32	3.72E-01	1.00E+00	0.25	5.20E-01	9.17E-01
**MPHOSPH10**	-0.30	4.25E-02	1.00E+00	-0.11	5.21E-01	9.17E-01
**TPM3P6**	-0.32	7.54E-01	1.00E+00	-0.28	5.21E-01	9.17E-01
**WDR44**	0.28	2.79E-01	1.00E+00	-0.20	5.21E-01	9.17E-01
**STEAP4**	0.64	9.67E-02	1.00E+00	-0.32	5.21E-01	9.17E-01
**PHKA1**	-0.06	7.77E-01	1.00E+00	-0.50	5.21E-01	9.17E-01
**ERV3-1**	-0.59	3.02E-01	1.00E+00	-0.38	5.21E-01	9.17E-01
**ZNF174**	-0.19	4.74E-01	1.00E+00	-0.25	5.21E-01	9.17E-01

**NFKBIE**	-0.23	4.02E-01	1.00E+00	0.40	5.21E-01	9.17E-01
**NETO2**	-2.18	1.26E-04	2.99E-02	0.59	5.21E-01	9.17E-01
**DHRS1**	-0.53	8.76E-02	1.00E+00	-0.18	5.21E-01	9.17E-01
**PACRGL**	-0.06	7.94E-01	1.00E+00	-0.21	5.21E-01	9.17E-01
**ERAL1**	-0.11	5.50E-01	1.00E+00	-0.14	5.21E-01	9.17E-01
**RPL23AP32**	1.68	3.65E-01	1.00E+00	-0.82	5.21E-01	9.17E-01
**HAUS7**	-2.67	1.10E-01	1.00E+00	0.55	5.21E-01	9.17E-01
**NUDT10**	0.02	9.74E-01	1.00E+00	-0.47	5.21E-01	9.17E-01
**KIF22**	-0.25	4.20E-01	1.00E+00	-0.21	5.21E-01	9.17E-01
**EDF1**	-0.10	6.44E-01	1.00E+00	0.11	5.21E-01	9.17E-01
**INHBB**	0.51	3.03E-01	1.00E+00	0.35	5.21E-01	9.17E-01
**ABCA8**	0.75	1.81E-01	1.00E+00	0.35	5.22E-01	9.17E-01
**RASSF5**	-0.36	3.92E-01	1.00E+00	0.28	5.22E-01	9.17E-01
**RBMS3-AS3**	-0.53	5.54E-01	1.00E+00	-0.70	5.22E-01	9.17E-01
**AL591848.4**	0.04	9.63E-01	1.00E+00	0.79	5.22E-01	9.17E-01
**JAG2**	-0.29	2.18E-01	1.00E+00	-0.34	5.22E-01	9.17E-01
**UGT1A7**	-1.29	2.63E-01	1.00E+00	0.60	5.22E-01	9.17E-01
**ZNF738**	0.37	4.67E-01	1.00E+00	-0.29	5.22E-01	9.17E-01
**LSS**	0.22	2.40E-01	1.00E+00	-0.21	5.22E-01	9.17E-01
**DYRK2**	-0.18	4.02E-01	1.00E+00	0.30	5.22E-01	9.17E-01
**RP11-343N15.5**	0.39	2.74E-01	1.00E+00	-0.21	5.22E-01	9.17E-01
**C3orf38**	-0.13	6.31E-01	1.00E+00	-0.10	5.22E-01	9.17E-01
**GDAP2**	0.44	3.16E-02	1.00E+00	-0.15	5.22E-01	9.17E-01
**URB2**	-0.24	4.57E-01	1.00E+00	0.29	5.22E-01	9.17E-01
**AL669831.3**	-3.21	3.78E-02	1.00E+00	-0.41	5.22E-01	9.17E-01
**KRT79**	-0.06	9.57E-01	1.00E+00	-0.57	5.22E-01	9.17E-01
**BCDIN3D-AS1**	-0.32	7.98E-01	1.00E+00	-0.79	5.22E-01	9.17E-01
**ISCA1**	0.15	5.25E-01	1.00E+00	-0.18	5.22E-01	9.17E-01
**KRT15**	0.27	4.72E-01	1.00E+00	-0.36	5.22E-01	9.17E-01
**TPRG1L**	-0.01	9.50E-01	1.00E+00	-0.27	5.22E-01	9.17E-01
**GLYATL2**	1.49	1.09E-01	1.00E+00	0.44	5.23E-01	9.17E-01
**PDIK1L**	0.05	8.86E-01	1.00E+00	-0.20	5.23E-01	9.17E-01

**NKRF**	-0.18	4.42E-01	1.00E+00	0.19	5.23E-01	9.17E-01
**HCAR3**	-0.26	7.34E-01	1.00E+00	0.37	5.23E-01	9.17E-01
**EPHA1**	-0.15	7.56E-01	1.00E+00	0.38	5.23E-01	9.17E-01
**PKD1P6-NPIPP1**	-3.46	3.19E-03	2.97E-01	0.73	5.23E-01	9.17E-01
**AC009299.3**	2.14	3.77E-01	1.00E+00	-0.58	5.23E-01	9.17E-01
**COG2**	-0.02	9.18E-01	1.00E+00	0.10	5.23E-01	9.17E-01
**PLCB2**	-0.36	4.74E-01	1.00E+00	2.00	5.23E-01	9.17E-01
**KEL**	-1.33	1.96E-01	1.00E+00	0.65	5.23E-01	9.17E-01
**MAD2L2**	-0.34	3.07E-02	1.00E+00	0.10	5.23E-01	9.17E-01
**PMVK**	-0.09	7.33E-01	1.00E+00	-0.15	5.23E-01	9.17E-01
**MTX1**	-0.03	8.87E-01	1.00E+00	0.11	5.23E-01	9.17E-01
**AK3P3**	0.82	3.51E-01	1.00E+00	0.51	5.23E-01	9.17E-01
**DIP2C**	0.49	3.22E-01	1.00E+00	0.26	5.23E-01	9.17E-01
**TPM3P9**	0.29	3.24E-01	1.00E+00	-0.23	5.23E-01	9.17E-01
**HNRNPLP2**	0.49	6.22E-01	1.00E+00	0.34	5.23E-01	9.17E-01
**C2orf48**	0.68	6.62E-01	1.00E+00	0.77	5.23E-01	9.17E-01
**CCHCR1**	-0.04	8.57E-01	1.00E+00	0.14	5.23E-01	9.17E-01
**HECW1**	-0.56	5.68E-01	1.00E+00	0.69	5.23E-01	9.17E-01
**PRIMA1**	0.13	7.50E-01	1.00E+00	0.42	5.23E-01	9.17E-01
**EIF4A1P10**	0.54	4.85E-01	1.00E+00	0.37	5.23E-01	9.17E-01
**TYMS**	-0.38	5.14E-01	1.00E+00	0.20	5.23E-01	9.17E-01
**LINC00339**	0.92	4.59E-01	1.00E+00	0.63	5.23E-01	9.17E-01
**PI3**	-0.55	2.99E-01	1.00E+00	1.99	5.23E-01	9.17E-01
**AC012158.1**	0.30	8.13E-01	1.00E+00	-0.44	5.23E-01	9.17E-01
**RNF212B**	-2.21	1.46E-01	1.00E+00	0.78	5.23E-01	9.17E-01
**AC092115.1**	-0.26	8.55E-01	1.00E+00	-0.30	5.23E-01	9.17E-01
**ZNF154**	-0.28	7.64E-01	1.00E+00	0.45	5.24E-01	9.17E-01
**AL353708.1**	-2.19	8.76E-02	1.00E+00	-0.60	5.24E-01	9.17E-01
**WDR49**	-2.26	3.12E-01	1.00E+00	-0.80	5.24E-01	9.17E-01
**TOMM34**	-0.16	4.71E-01	1.00E+00	-0.12	5.24E-01	9.17E-01
**KRT75**	-0.59	8.23E-01	1.00E+00	1.99	5.24E-01	9.17E-01
**AL031282.1**	2.15	2.91E-01	1.00E+00	0.66	5.24E-01	9.17E-01

**GUCY1A2**	-0.55	3.43E-01	1.00E+00	0.33	5.24E-01	9.17E-01
**BTF3**	-0.19	4.65E-01	1.00E+00	0.11	5.24E-01	9.17E-01
**ZFYVE16**	0.00	9.94E-01	1.00E+00	-0.13	5.24E-01	9.17E-01
**QPCT**	0.14	6.34E-01	1.00E+00	0.24	5.24E-01	9.17E-01
**DAXX**	0.03	8.80E-01	1.00E+00	0.10	5.24E-01	9.17E-01
**ZNF23**	0.24	7.60E-01	1.00E+00	-0.45	5.24E-01	9.17E-01
**Z97192.2**	-0.14	9.23E-01	1.00E+00	1.23	5.24E-01	9.17E-01
**ZKSCAN4**	0.14	6.32E-01	1.00E+00	0.28	5.24E-01	9.17E-01
**ATP6V1G1**	0.05	7.84E-01	1.00E+00	-0.20	5.24E-01	9.17E-01
**XLOC_004501**	0.90	2.75E-01	1.00E+00	-0.50	5.24E-01	9.17E-01
**HERC2P4**	1.08	5.51E-01	1.00E+00	0.77	5.24E-01	9.17E-01
**RABEP1**	0.07	8.31E-01	1.00E+00	0.14	5.24E-01	9.17E-01
**ZNF845**	-0.16	5.96E-01	1.00E+00	0.21	5.24E-01	9.17E-01
**PDE5A**	0.25	6.48E-01	1.00E+00	0.27	5.24E-01	9.17E-01
**NAPA-AS1**	0.83	4.94E-01	1.00E+00	0.45	5.24E-01	9.17E-01
**RPSA**	-0.15	5.07E-01	1.00E+00	0.22	5.24E-01	9.17E-01
**XLOC_011623**	-0.75	4.03E-01	1.00E+00	0.48	5.24E-01	9.18E-01
**CDC34**	0.04	8.97E-01	1.00E+00	-0.47	5.24E-01	9.18E-01
**STXBP1**	-0.23	5.67E-01	1.00E+00	-0.25	5.25E-01	9.18E-01
**TMEM67**	0.28	3.75E-01	1.00E+00	0.21	5.25E-01	9.18E-01
**EVI5L**	-0.55	5.53E-02	1.00E+00	0.42	5.25E-01	9.18E-01
**AL133467.1**	0.17	8.23E-01	1.00E+00	0.43	5.25E-01	9.18E-01
**TRIM29**	-0.33	4.50E-01	1.00E+00	-0.35	5.25E-01	9.18E-01
**KIF20B**	-0.16	5.75E-01	1.00E+00	-0.22	5.25E-01	9.18E-01
**ATP6V1E1**	0.08	6.03E-01	1.00E+00	0.08	5.25E-01	9.18E-01
**H2AFZ**	-0.01	9.78E-01	1.00E+00	-0.27	5.25E-01	9.18E-01
**TAF5L**	0.13	4.94E-01	1.00E+00	0.42	5.25E-01	9.18E-01
**CCDC33**	-0.29	9.33E-01	1.00E+00	-0.72	5.25E-01	9.18E-01
**POLR3H**	-0.31	1.77E-01	1.00E+00	0.16	5.25E-01	9.18E-01
**UBB**	-0.01	9.64E-01	1.00E+00	0.20	5.25E-01	9.18E-01
**RBBP8**	-0.10	6.49E-01	1.00E+00	0.15	5.25E-01	9.18E-01
**BACE1-AS**	0.40	6.66E-01	1.00E+00	0.38	5.25E-01	9.18E-01

**TTC28-AS1**	0.01	9.68E-01	1.00E+00	0.16	5.25E-01	9.18E-01
**Z83843.1**	0.00	9.98E-01	1.00E+00	-0.48	5.25E-01	9.18E-01
**JUNB**	-1.21	1.24E-01	1.00E+00	-0.34	5.25E-01	9.18E-01
**AL359752.1**	1.04	2.10E-03	2.39E-01	0.38	5.25E-01	9.18E-01
**MROH6**	-0.03	9.49E-01	1.00E+00	0.39	5.25E-01	9.18E-01
**AL662795.2**	0.31	2.96E-01	1.00E+00	0.36	5.26E-01	9.18E-01
**XLOC_006188**	0.02	9.83E-01	1.00E+00	-0.52	5.26E-01	9.18E-01
**EEF1DP5**	2.62	3.24E-01	1.00E+00	-1.03	5.26E-01	9.18E-01
**AC073257.2**	-0.85	5.82E-01	1.00E+00	-0.84	5.26E-01	9.18E-01
**TBX18**	0.49	2.51E-01	1.00E+00	0.31	5.26E-01	9.18E-01
**POLR1A**	-0.26	2.38E-01	1.00E+00	0.36	5.26E-01	9.18E-01
**AC007387.1**	1.38	6.90E-01	1.00E+00	-0.66	5.26E-01	9.18E-01
**SLITRK6**	0.12	6.53E-01	1.00E+00	-0.36	5.26E-01	9.18E-01
**KPNA4**	0.01	9.44E-01	1.00E+00	-0.12	5.26E-01	9.18E-01
**ZFP36**	-2.00	2.51E-02	9.28E-01	-0.39	5.26E-01	9.19E-01
**AC010761.3**	-0.44	8.30E-01	1.00E+00	-0.85	5.26E-01	9.19E-01
**ELP2**	0.17	3.45E-01	1.00E+00	-0.10	5.26E-01	9.19E-01
**LRRFIP1**	-0.27	1.38E-01	1.00E+00	0.14	5.26E-01	9.19E-01
**SLC26A5**	-1.05	3.60E-01	1.00E+00	0.55	5.26E-01	9.19E-01
**LINC00652**	-0.23	8.71E-01	1.00E+00	0.82	5.26E-01	9.19E-01
**AC139491.7**	0.98	3.65E-01	1.00E+00	0.60	5.26E-01	9.19E-01
**AC133552.2**	-0.22	6.93E-01	1.00E+00	-0.33	5.26E-01	9.19E-01
**PLPPR2**	-0.39	1.26E-01	1.00E+00	0.28	5.26E-01	9.19E-01
**MT-RNR1**	0.03	9.33E-01	1.00E+00	-0.22	5.26E-01	9.19E-01
**TESMIN**	-0.23	5.70E-01	1.00E+00	0.38	5.26E-01	9.19E-01
**AP001065.1**	0.13	8.60E-01	1.00E+00	0.65	5.26E-01	9.19E-01
**NLRP1**	-0.22	3.46E-01	1.00E+00	0.22	5.27E-01	9.19E-01
**ABCB11**	-1.75	9.39E-02	1.00E+00	0.71	5.27E-01	9.19E-01
**EDEM3**	0.25	3.72E-01	1.00E+00	0.18	5.27E-01	9.19E-01
**SAPCD2**	-0.69	2.46E-02	9.27E-01	-0.40	5.27E-01	9.19E-01
**PLEKHG1**	-0.33	2.57E-01	1.00E+00	0.22	5.27E-01	9.19E-01
**SNHG19**	0.09	8.39E-01	1.00E+00	-0.27	5.27E-01	9.19E-01

**SPTSSA**	-0.02	9.03E-01	1.00E+00	-0.22	5.27E-01	9.19E-01
**IGKV1-16**	1.60	6.38E-01	1.00E+00	1.44	5.27E-01	9.19E-01
**GCSAML**	0.98	1.89E-01	1.00E+00	-0.49	5.27E-01	9.19E-01
**HSPA12B**	-0.13	7.77E-01	1.00E+00	0.34	5.27E-01	9.19E-01
**MADD**	0.06	7.32E-01	1.00E+00	0.24	5.27E-01	9.19E-01
**POLDIP2**	-0.05	7.79E-01	1.00E+00	-0.16	5.27E-01	9.19E-01
**AL136295.7**	0.24	6.70E-01	1.00E+00	-0.34	5.27E-01	9.19E-01
**PRKAB2**	0.54	1.19E-01	1.00E+00	-0.18	5.27E-01	9.19E-01
**MRPS31P4**	-0.03	9.79E-01	1.00E+00	0.33	5.27E-01	9.19E-01
**PKDCC**	0.72	2.61E-01	1.00E+00	0.49	5.27E-01	9.19E-01
**RDH13**	-0.20	6.01E-01	1.00E+00	0.22	5.27E-01	9.19E-01
**PLS3**	0.05	8.44E-01	1.00E+00	-0.14	5.27E-01	9.19E-01
**G25699**	-0.67	2.97E-01	1.00E+00	-0.38	5.27E-01	9.19E-01
**DCAF4**	0.12	6.58E-01	1.00E+00	-0.17	5.27E-01	9.19E-01
**DNPH1**	0.26	3.46E-01	1.00E+00	0.18	5.27E-01	9.19E-01
**RP11-337C18.9**	-0.28	7.96E-01	1.00E+00	-0.53	5.27E-01	9.19E-01
**RFC4**	0.04	8.90E-01	1.00E+00	0.17	5.27E-01	9.19E-01
**SLC16A9**	-1.32	1.87E-02	8.59E-01	1.51	5.27E-01	9.19E-01
**CENPS**	-1.06	3.88E-01	1.00E+00	-0.40	5.27E-01	9.19E-01
**ZNF554**	-0.13	6.82E-01	1.00E+00	-0.33	5.27E-01	9.19E-01
**AC117409.1**	NA	NA	NA	-0.52	5.27E-01	9.19E-01
**MPDU1**	-0.11	6.38E-01	1.00E+00	0.14	5.27E-01	9.19E-01
**GUSB**	0.13	6.01E-01	1.00E+00	0.16	5.28E-01	9.19E-01
**AGPAT5**	-0.08	7.69E-01	1.00E+00	0.13	5.28E-01	9.19E-01
**THNSL2**	-0.43	4.29E-01	1.00E+00	0.28	5.28E-01	9.19E-01
**CELF2**	0.62	1.39E-01	1.00E+00	0.22	5.28E-01	9.19E-01
**CD320**	0.22	6.59E-01	1.00E+00	-0.27	5.28E-01	9.19E-01
**BORCS8**	-0.51	1.75E-01	1.00E+00	-0.17	5.28E-01	9.19E-01
**HSD3BP5**	-0.11	8.89E-01	1.00E+00	0.42	5.28E-01	9.19E-01
**TESK2**	-0.04	8.58E-01	1.00E+00	0.19	5.28E-01	9.19E-01
**SLC25A21**	-0.86	4.65E-01	1.00E+00	-0.43	5.28E-01	9.19E-01
**ZNF774**	-0.26	4.94E-01	1.00E+00	0.29	5.28E-01	9.19E-01

**PRPF38A**	0.21	3.03E-01	1.00E+00	-0.09	5.28E-01	9.19E-01
**VSTM4**	0.33	4.14E-01	1.00E+00	0.31	5.28E-01	9.19E-01
**FAM160B2**	-0.40	1.52E-01	1.00E+00	0.37	5.28E-01	9.19E-01
**MYBPH**	3.58	2.87E-01	1.00E+00	1.22	5.28E-01	9.19E-01
**SYT8**	-1.29	1.95E-02	8.74E-01	-0.50	5.28E-01	9.19E-01
**HMGB1P31**	1.16	1.23E-01	1.00E+00	-0.51	5.28E-01	9.19E-01
**RP11-436G20.1**	0.19	8.84E-01	1.00E+00	-0.44	5.28E-01	9.19E-01
**CD164L2**	-0.27	5.41E-01	1.00E+00	0.31	5.28E-01	9.19E-01
**FXYD7**	-1.34	2.52E-01	1.00E+00	0.46	5.28E-01	9.19E-01
**AC084824.1**	-0.21	8.39E-01	1.00E+00	0.33	5.28E-01	9.19E-01
**CHODL-AS1**	0.49	7.58E-01	1.00E+00	1.13	5.28E-01	9.19E-01
**PAPOLA**	-0.05	8.04E-01	1.00E+00	0.13	5.28E-01	9.19E-01
**EGR3**	-0.25	6.59E-01	1.00E+00	-0.30	5.28E-01	9.19E-01
**IGKV1D-12**	1.97	4.75E-01	1.00E+00	2.01	5.28E-01	9.19E-01
**SMARCC1**	-0.09	7.01E-01	1.00E+00	-0.21	5.28E-01	9.19E-01
**EZR**	-0.19	6.42E-01	1.00E+00	0.21	5.29E-01	9.19E-01
**SNW1**	-0.11	5.17E-01	1.00E+00	-0.16	5.29E-01	9.19E-01
**PIGHP1**	-1.25	4.35E-01	1.00E+00	-0.48	5.29E-01	9.19E-01
**SNX33**	-0.13	5.69E-01	1.00E+00	0.33	5.29E-01	9.19E-01
**RNF19B**	-0.35	1.14E-01	1.00E+00	0.40	5.29E-01	9.19E-01
**ATP6V1G2**	0.06	9.86E-01	1.00E+00	-0.59	5.29E-01	9.19E-01
**SLC30A10**	-1.02	3.97E-01	1.00E+00	0.74	5.29E-01	9.19E-01
**C2CD4C**	-0.31	7.07E-01	1.00E+00	0.79	5.29E-01	9.19E-01
**PLEKHD1**	0.23	7.74E-01	1.00E+00	-0.48	5.29E-01	9.19E-01
**PSD4**	-0.35	1.84E-01	1.00E+00	0.28	5.29E-01	9.19E-01
**AC138028.6**	-0.89	5.43E-01	1.00E+00	1.10	5.29E-01	9.19E-01
**AC040160.2**	1.43	2.17E-01	1.00E+00	0.76	5.29E-01	9.19E-01
**MRPL46**	0.62	5.16E-01	1.00E+00	0.43	5.29E-01	9.19E-01
**ZFAT**	-0.03	9.36E-01	1.00E+00	0.19	5.29E-01	9.19E-01
**AP001350.2**	0.02	9.79E-01	1.00E+00	0.51	5.29E-01	9.19E-01
**MMP12**	-1.95	2.32E-01	1.00E+00	1.97	5.29E-01	9.19E-01
**AL359644.1**	-2.33	1.77E-01	1.00E+00	-0.50	5.29E-01	9.19E-01

**AC123777.1**	-1.80	1.21E-01	1.00E+00	0.70	5.29E-01	9.19E-01
**GRIK3**	-0.31	6.00E-01	1.00E+00	0.65	5.29E-01	9.19E-01
**PSAT1P3**	-0.06	9.69E-01	1.00E+00	-0.64	5.29E-01	9.19E-01
**HKR1**	-0.01	9.71E-01	1.00E+00	-0.14	5.30E-01	9.19E-01
**TPPP3**	0.08	8.81E-01	1.00E+00	-0.24	5.30E-01	9.19E-01
**APH1B**	0.22	5.72E-01	1.00E+00	0.25	5.30E-01	9.19E-01
**AC131097.2**	1.01	3.36E-02	1.00E+00	0.35	5.30E-01	9.19E-01
**AC098650.1**	2.12	5.34E-01	1.00E+00	0.87	5.30E-01	9.19E-01
**SEMA3F-AS1**	-0.94	6.15E-01	1.00E+00	-0.61	5.30E-01	9.19E-01
**EIF4A1P4**	-0.56	8.73E-01	1.00E+00	0.81	5.30E-01	9.19E-01
**AL023807.1**	0.73	6.30E-01	1.00E+00	0.59	5.30E-01	9.19E-01
**SART3**	-0.10	5.82E-01	1.00E+00	0.11	5.30E-01	9.19E-01
**TRIM8**	-0.16	5.57E-01	1.00E+00	0.35	5.30E-01	9.19E-01
**XLOC_012879**	0.49	8.44E-01	1.00E+00	0.86	5.30E-01	9.19E-01
**AP3B1**	-0.03	8.96E-01	1.00E+00	-0.12	5.30E-01	9.19E-01
**XLOC_007614**	-1.58	5.56E-02	1.00E+00	-0.61	5.30E-01	9.19E-01
**G5143**	0.96	1.68E-01	1.00E+00	0.66	5.30E-01	9.19E-01
**RPAP2**	-0.28	2.47E-01	1.00E+00	-0.14	5.30E-01	9.19E-01
**SIGLEC15**	-0.88	4.08E-01	1.00E+00	0.45	5.30E-01	9.19E-01
**GNPDA2**	0.12	5.58E-01	1.00E+00	-0.19	5.30E-01	9.19E-01
**ALDH1L1-AS2**	1.13	6.07E-01	1.00E+00	0.95	5.30E-01	9.19E-01
**AL035458.1**	-0.67	8.48E-01	1.00E+00	-0.39	5.30E-01	9.19E-01
**B3GLCT**	-0.08	8.13E-01	1.00E+00	-0.15	5.30E-01	9.19E-01
**PPP3CB-AS1**	-0.21	5.95E-01	1.00E+00	-0.24	5.30E-01	9.19E-01
**ZNF485**	0.10	8.39E-01	1.00E+00	-0.25	5.30E-01	9.19E-01
**AL135936.1**	-0.30	8.27E-01	1.00E+00	0.62	5.30E-01	9.19E-01
**ERI2**	-0.07	8.25E-01	1.00E+00	0.17	5.30E-01	9.19E-01
**HYAL2**	-0.30	1.35E-01	1.00E+00	0.24	5.30E-01	9.19E-01
**AC026462.1**	-1.49	5.00E-01	1.00E+00	-0.32	5.30E-01	9.19E-01
**NOLC1**	-0.25	1.90E-01	1.00E+00	0.16	5.31E-01	9.19E-01
**PPT2**	0.10	7.36E-01	1.00E+00	-0.14	5.31E-01	9.19E-01
**ATP1A1-AS1**	0.02	9.49E-01	1.00E+00	-0.19	5.31E-01	9.19E-01

**GALK2**	0.27	2.72E-01	1.00E+00	-0.15	5.31E-01	9.19E-01
**AVEN**	0.05	8.18E-01	1.00E+00	-0.30	5.31E-01	9.19E-01
**HOXA10-AS**	-1.53	2.23E-01	1.00E+00	0.35	5.31E-01	9.19E-01
**SLC25A19**	0.68	1.94E-01	1.00E+00	0.26	5.31E-01	9.19E-01
**STIP1**	-0.04	8.45E-01	1.00E+00	0.11	5.31E-01	9.19E-01
**ANKRD10-IT1**	0.57	6.46E-01	1.00E+00	0.36	5.31E-01	9.19E-01
**UBR5-AS1**	0.30	6.69E-01	1.00E+00	-0.22	5.31E-01	9.19E-01
**AL133384.2**	-1.14	7.43E-01	1.00E+00	0.83	5.31E-01	9.19E-01
**AC108134.4**	-0.70	5.81E-01	1.00E+00	-0.59	5.31E-01	9.19E-01
**TPM4**	-0.15	4.04E-01	1.00E+00	0.18	5.31E-01	9.19E-01
**LINC00265**	-0.32	6.64E-01	1.00E+00	0.54	5.31E-01	9.19E-01
**TOB2**	-0.19	5.69E-01	1.00E+00	-0.17	5.31E-01	9.19E-01
**PDCD4-AS1**	-0.65	2.18E-01	1.00E+00	0.38	5.31E-01	9.19E-01
**PRAL**	-1.59	4.60E-01	1.00E+00	0.79	5.31E-01	9.19E-01
**MCUB**	-0.01	9.79E-01	1.00E+00	-0.21	5.31E-01	9.19E-01
**AC100803.2**	1.11	1.19E-01	1.00E+00	0.92	5.31E-01	9.19E-01
**IFT43**	-0.18	3.61E-01	1.00E+00	-0.18	5.31E-01	9.19E-01
**SIAH3**	-0.86	5.47E-01	1.00E+00	0.75	5.31E-01	9.19E-01
**EDEM2**	0.10	6.55E-01	1.00E+00	0.16	5.31E-01	9.19E-01
**PELI3**	-0.02	9.30E-01	1.00E+00	-0.39	5.31E-01	9.19E-01
**AC061992.1**	-0.46	7.39E-01	1.00E+00	0.78	5.31E-01	9.19E-01
**TATDN2**	0.11	5.65E-01	1.00E+00	0.25	5.31E-01	9.19E-01
**AC027020.2**	0.33	7.16E-01	1.00E+00	0.75	5.31E-01	9.19E-01
**XLOC_009592**	-2.96	1.09E-01	1.00E+00	0.92	5.31E-01	9.19E-01
**CCT8P1**	0.62	1.07E-01	1.00E+00	0.25	5.31E-01	9.19E-01
**SPACA6**	0.56	2.08E-01	1.00E+00	-0.24	5.31E-01	9.19E-01
**SNRNP35**	-0.02	9.06E-01	1.00E+00	-0.14	5.32E-01	9.19E-01
**DEFB103A**	-1.93	5.74E-01	1.00E+00	0.53	5.32E-01	9.19E-01
**CKMT1A**	-0.57	4.97E-02	1.00E+00	-0.31	5.32E-01	9.19E-01
**DDX6**	0.17	3.23E-01	1.00E+00	0.19	5.32E-01	9.19E-01
**PDS5A**	0.00	9.90E-01	1.00E+00	-0.21	5.32E-01	9.19E-01
**KLRC1**	-3.36	1.19E-01	1.00E+00	0.76	5.32E-01	9.19E-01

**OPRD1**	-0.08	9.66E-01	1.00E+00	-0.89	5.32E-01	9.19E-01
**PRRT4**	3.04	3.30E-02	1.00E+00	0.66	5.32E-01	9.19E-01
**SEMA4B**	-0.54	1.32E-01	1.00E+00	0.32	5.32E-01	9.19E-01
**HOXC11**	-0.41	3.23E-01	1.00E+00	0.19	5.32E-01	9.19E-01
**PYDC1**	-0.40	5.12E-01	1.00E+00	0.42	5.32E-01	9.19E-01
**ARPP21**	-2.47	1.23E-01	1.00E+00	0.91	5.32E-01	9.19E-01
**PDP1**	0.27	3.79E-01	1.00E+00	0.12	5.32E-01	9.19E-01
**AC011632.1**	-2.33	1.95E-01	1.00E+00	-0.67	5.32E-01	9.19E-01
**ALDOA**	-0.09	8.60E-01	1.00E+00	0.44	5.32E-01	9.19E-01
**MKS1**	-0.07	6.64E-01	1.00E+00	0.16	5.32E-01	9.19E-01
**CLSTN1**	-0.26	2.94E-01	1.00E+00	0.32	5.32E-01	9.19E-01
**CASC9**	1.98	3.38E-01	1.00E+00	-1.35	5.32E-01	9.19E-01
**XLOC_013164**	-0.22	9.28E-01	1.00E+00	-1.14	5.32E-01	9.19E-01
**GPT2**	-0.19	5.48E-01	1.00E+00	-0.27	5.33E-01	9.19E-01
**RPL5P5**	-0.58	8.67E-01	1.00E+00	0.64	5.33E-01	9.19E-01
**ID3**	0.34	1.82E-01	1.00E+00	0.23	5.33E-01	9.19E-01
**C9orf170**	-1.29	2.73E-01	1.00E+00	0.62	5.33E-01	9.19E-01
**KAT14**	0.06	7.19E-01	1.00E+00	0.11	5.33E-01	9.19E-01
**PUS1**	-0.55	6.41E-02	1.00E+00	0.37	5.33E-01	9.20E-01
**SH2D3C**	-0.06	8.94E-01	1.00E+00	0.35	5.33E-01	9.20E-01
**TRAV12-1**	-1.49	3.90E-01	1.00E+00	0.81	5.33E-01	9.20E-01
**KRR1**	-0.19	3.68E-01	1.00E+00	-0.13	5.33E-01	9.20E-01
**LRP10**	-0.07	7.52E-01	1.00E+00	0.30	5.33E-01	9.20E-01
**ID2**	0.00	9.91E-01	1.00E+00	-0.20	5.33E-01	9.20E-01
**INSM1**	-0.64	5.15E-01	1.00E+00	-0.66	5.33E-01	9.20E-01
**SAMD4B**	-0.30	8.67E-02	1.00E+00	0.27	5.33E-01	9.20E-01
**GLOD4**	0.16	2.93E-01	1.00E+00	-0.10	5.33E-01	9.20E-01
**TREML3P**	NA	NA	NA	1.64	5.33E-01	9.20E-01
**H2AFZP3**	-0.37	9.15E-01	1.00E+00	-0.47	5.33E-01	9.20E-01
**PTOV1-AS2**	-1.72	2.77E-01	1.00E+00	0.84	5.33E-01	9.20E-01
**HSP90AA2P**	-0.81	4.54E-01	1.00E+00	-0.37	5.33E-01	9.20E-01
**TIE1**	0.15	7.69E-01	1.00E+00	0.44	5.33E-01	9.20E-01

**AC011379.2**	0.90	2.86E-01	1.00E+00	0.42	5.33E-01	9.20E-01
**BUD13**	-0.07	6.75E-01	1.00E+00	0.17	5.33E-01	9.20E-01
**HYOU1**	-0.16	4.38E-01	1.00E+00	0.38	5.34E-01	9.20E-01
**IARS2**	0.19	2.24E-01	1.00E+00	-0.08	5.34E-01	9.20E-01
**XLOC_012740**	-1.72	8.89E-02	1.00E+00	-0.30	5.34E-01	9.20E-01
**XLOC_004203**	-0.95	5.28E-01	1.00E+00	-0.87	5.34E-01	9.20E-01
**AL031963.3**	0.81	4.25E-01	1.00E+00	0.50	5.34E-01	9.20E-01
**XLOC_005510**	-0.21	7.51E-01	1.00E+00	-0.57	5.34E-01	9.20E-01
**MYL9**	-0.20	7.39E-01	1.00E+00	0.28	5.34E-01	9.20E-01
**BNC1**	-0.68	9.68E-02	1.00E+00	0.40	5.34E-01	9.20E-01
**ETS2**	-0.35	3.83E-01	1.00E+00	0.20	5.34E-01	9.20E-01
**CCDC15**	-0.31	5.53E-01	1.00E+00	-0.27	5.34E-01	9.20E-01
**IGLV2-11**	4.14	2.54E-02	9.29E-01	1.97	5.34E-01	9.20E-01
**AL358216.1**	-0.74	3.44E-01	1.00E+00	0.61	5.34E-01	9.20E-01
**OGFOD3**	-0.04	8.56E-01	1.00E+00	0.23	5.34E-01	9.20E-01
**RNU6-415P**	-1.90	9.91E-02	1.00E+00	0.57	5.34E-01	9.20E-01
**ADCK2**	0.01	9.70E-01	1.00E+00	-0.22	5.34E-01	9.20E-01
**AC103740.2**	0.62	8.57E-01	1.00E+00	-1.29	5.34E-01	9.21E-01
**HPN**	-0.78	3.70E-01	1.00E+00	0.77	5.35E-01	9.21E-01
**ITGA8**	-0.02	9.74E-01	1.00E+00	-0.34	5.35E-01	9.21E-01
**AC093157.2**	-2.94	3.86E-01	1.00E+00	0.58	5.35E-01	9.21E-01
**TSC22D1**	0.34	1.91E-01	1.00E+00	-0.20	5.35E-01	9.21E-01
**ZNRF2**	-0.12	6.80E-01	1.00E+00	-0.30	5.35E-01	9.21E-01
**IBSP**	-3.68	2.75E-01	1.00E+00	1.11	5.35E-01	9.21E-01
**AC245884.11**	-0.70	8.41E-01	1.00E+00	1.04	5.35E-01	9.21E-01
**LETM1**	-0.23	3.25E-01	1.00E+00	0.16	5.35E-01	9.21E-01
**SMIM12**	0.00	9.81E-01	1.00E+00	0.10	5.35E-01	9.21E-01
**LINC02454**	-0.22	8.41E-01	1.00E+00	-0.52	5.35E-01	9.21E-01
**AL445471.2**	-2.03	3.98E-01	1.00E+00	-0.97	5.35E-01	9.21E-01
**CBX3P2**	-0.56	2.61E-01	1.00E+00	-0.35	5.35E-01	9.21E-01
**AC073316.3**	-0.33	8.31E-01	1.00E+00	0.71	5.35E-01	9.21E-01
**G19165**	-3.11	9.81E-02	1.00E+00	0.82	5.35E-01	9.21E-01

**AL121760.1**	-0.52	7.40E-01	1.00E+00	0.37	5.35E-01	9.21E-01
**AC063960.2**	-0.01	9.87E-01	1.00E+00	0.60	5.35E-01	9.21E-01
**NAPG**	0.15	5.76E-01	1.00E+00	-0.17	5.35E-01	9.21E-01
**TMPRSS11E**	-0.81	2.94E-01	1.00E+00	-0.46	5.35E-01	9.21E-01
**SGK494**	-0.98	4.48E-01	1.00E+00	0.63	5.35E-01	9.21E-01
**TAGLN3**	-2.85	1.65E-01	1.00E+00	0.75	5.36E-01	9.21E-01
**GOLGA2P8**	-1.37	6.93E-01	1.00E+00	-0.76	5.36E-01	9.21E-01
**CEP83-DT**	-0.01	9.90E-01	1.00E+00	0.67	5.36E-01	9.21E-01
**AC025171.2**	-0.46	4.96E-01	1.00E+00	0.46	5.36E-01	9.21E-01
**JMJD6**	-0.24	3.50E-01	1.00E+00	0.17	5.36E-01	9.21E-01
**AC142086.6**	-0.13	8.75E-01	1.00E+00	-0.71	5.36E-01	9.21E-01
**CCND3**	0.28	2.31E-01	1.00E+00	0.20	5.36E-01	9.21E-01
**G20736**	0.35	5.62E-01	1.00E+00	0.43	5.36E-01	9.21E-01
**AC026367.3**	1.93	3.83E-02	1.00E+00	-0.58	5.36E-01	9.21E-01
**CIART**	-1.51	5.40E-03	4.32E-01	-0.29	5.36E-01	9.21E-01
**HSPD1**	0.19	4.37E-01	1.00E+00	-0.14	5.36E-01	9.21E-01
**FAM207A**	-0.20	4.37E-01	1.00E+00	0.33	5.36E-01	9.21E-01
**GPR158**	-0.49	7.68E-01	1.00E+00	0.81	5.36E-01	9.21E-01
**TYW3**	0.16	4.70E-01	1.00E+00	-0.11	5.36E-01	9.21E-01
**AC010735.2**	2.52	5.89E-02	1.00E+00	0.60	5.36E-01	9.21E-01
**HEXDC**	-0.11	7.27E-01	1.00E+00	0.33	5.36E-01	9.21E-01
**EFL1**	-0.08	6.25E-01	1.00E+00	0.13	5.36E-01	9.21E-01
**CCNG1**	0.20	4.34E-01	1.00E+00	-0.21	5.36E-01	9.21E-01
**VASP**	-0.27	1.68E-01	1.00E+00	0.40	5.36E-01	9.21E-01
**CCDC14**	-0.35	1.94E-01	1.00E+00	0.11	5.36E-01	9.21E-01
**EXOC3L2**	-0.05	9.19E-01	1.00E+00	0.44	5.36E-01	9.21E-01
**KLHL13**	0.10	8.55E-01	1.00E+00	-0.33	5.36E-01	9.21E-01
**AC116345.1**	0.11	8.40E-01	1.00E+00	0.35	5.36E-01	9.21E-01
**ROBO4**	0.16	7.63E-01	1.00E+00	0.39	5.36E-01	9.21E-01
**AC027682.6**	0.45	6.38E-01	1.00E+00	-0.43	5.36E-01	9.21E-01
**ARPIN**	0.14	5.99E-01	1.00E+00	0.21	5.37E-01	9.21E-01
**TNFRSF10D**	0.03	9.60E-01	1.00E+00	0.50	5.37E-01	9.21E-01

**GTF2IRD2**	0.46	1.44E-01	1.00E+00	-0.32	5.37E-01	9.21E-01
**L1TD1**	-1.30	2.99E-01	1.00E+00	0.66	5.37E-01	9.21E-01
**RAB3A**	-1.17	4.81E-03	4.02E-01	0.32	5.37E-01	9.21E-01
**SRR**	-0.36	1.48E-01	1.00E+00	-0.15	5.37E-01	9.21E-01
**WDR97**	-1.27	4.11E-01	1.00E+00	1.05	5.37E-01	9.21E-01
**RPS10P5**	-0.21	8.81E-01	1.00E+00	0.39	5.37E-01	9.21E-01
**AC092017.1**	0.56	8.71E-01	1.00E+00	-0.50	5.37E-01	9.21E-01
**CPNE8-AS1**	-2.39	1.34E-01	1.00E+00	-0.59	5.37E-01	9.21E-01
**CIP2A**	0.21	6.30E-01	1.00E+00	0.23	5.37E-01	9.21E-01
**NUF2**	0.23	6.32E-01	1.00E+00	0.33	5.37E-01	9.21E-01
**TMEM44**	0.20	6.99E-01	1.00E+00	0.37	5.37E-01	9.21E-01
**AC068152.1**	-0.12	7.86E-01	1.00E+00	0.24	5.37E-01	9.21E-01
**DUSP8**	-0.93	4.67E-02	1.00E+00	0.42	5.37E-01	9.21E-01
**NRXN3**	-0.77	3.00E-02	9.97E-01	-0.37	5.37E-01	9.21E-01
**KANSL1**	-0.04	8.51E-01	1.00E+00	0.19	5.37E-01	9.21E-01
**L3MBTL4**	0.36	3.32E-01	1.00E+00	-0.21	5.37E-01	9.21E-01
**S100A7A**	-3.08	9.81E-03	6.13E-01	1.93	5.37E-01	9.21E-01
**RASA1**	0.22	2.96E-01	1.00E+00	0.23	5.37E-01	9.21E-01
**C14orf132**	0.16	5.56E-01	1.00E+00	-0.21	5.37E-01	9.21E-01
**CD101**	-1.04	9.44E-02	1.00E+00	0.54	5.37E-01	9.21E-01
**PCDHB9**	-0.22	6.78E-01	1.00E+00	-0.39	5.37E-01	9.21E-01
**STIMATE**	0.38	8.71E-01	1.00E+00	-0.35	5.38E-01	9.21E-01
**AL157871.3**	0.66	8.49E-01	1.00E+00	0.56	5.38E-01	9.21E-01
**DCAF5**	-0.01	9.62E-01	1.00E+00	0.13	5.38E-01	9.21E-01
**CXCR3**	-1.03	2.56E-01	1.00E+00	1.76	5.38E-01	9.21E-01
**STT3B**	0.03	8.70E-01	1.00E+00	-0.25	5.38E-01	9.21E-01
**LRGUK**	-1.99	1.80E-01	1.00E+00	0.61	5.38E-01	9.21E-01
**TMEM35A**	-0.11	8.85E-01	1.00E+00	-0.41	5.38E-01	9.21E-01
**HLX-AS1**	2.02	2.79E-01	1.00E+00	0.81	5.38E-01	9.21E-01
**LINC01684**	0.41	6.55E-01	1.00E+00	0.69	5.38E-01	9.21E-01
**AGT**	-0.16	8.30E-01	1.00E+00	1.68	5.38E-01	9.21E-01
**ERN1**	0.17	4.09E-01	1.00E+00	0.25	5.38E-01	9.21E-01

**RPS8**	-0.19	5.90E-01	1.00E+00	0.17	5.38E-01	9.21E-01
**CRELD1**	-0.17	6.45E-01	1.00E+00	-0.17	5.38E-01	9.21E-01
**ZNF93**	-0.40	1.95E-01	1.00E+00	-0.30	5.38E-01	9.21E-01
**CMBL**	0.43	4.02E-01	1.00E+00	-0.25	5.38E-01	9.21E-01
**GXYLT1**	0.34	2.17E-01	1.00E+00	0.21	5.38E-01	9.21E-01
**ZIC1**	-0.36	7.32E-01	1.00E+00	1.93	5.38E-01	9.21E-01
**FHL5**	-0.61	3.24E-01	1.00E+00	0.32	5.38E-01	9.21E-01
**TBC1D10B**	-0.15	5.25E-01	1.00E+00	0.36	5.38E-01	9.21E-01
**LINC01888**	30.00	6.58E-18	5.54E-15	-1.03	5.38E-01	9.21E-01
**XLOC_008024**	0.13	7.68E-01	1.00E+00	0.29	5.38E-01	9.21E-01
**XLOC_013866**	0.47	6.20E-01	1.00E+00	-0.62	5.38E-01	9.21E-01
**XLOC_000523**	-0.15	8.48E-01	1.00E+00	0.66	5.38E-01	9.21E-01
**G10272**	-0.01	9.96E-01	1.00E+00	0.41	5.39E-01	9.21E-01
**NAB2**	-0.28	3.94E-01	1.00E+00	0.35	5.39E-01	9.21E-01
**BNIP1**	0.28	2.66E-01	1.00E+00	0.13	5.39E-01	9.21E-01
**XLOC_002032**	-1.63	1.58E-01	1.00E+00	-0.76	5.39E-01	9.21E-01
**PHF2P2**	-0.88	4.36E-01	1.00E+00	-0.64	5.39E-01	9.21E-01
**AHDC1**	-0.75	4.42E-02	1.00E+00	0.36	5.39E-01	9.21E-01
**TMEM158**	-0.09	8.71E-01	1.00E+00	-0.40	5.39E-01	9.21E-01
**ZFP36L1**	-0.92	4.05E-02	1.00E+00	0.28	5.39E-01	9.21E-01
**LRP12**	0.01	9.59E-01	1.00E+00	0.17	5.39E-01	9.21E-01
**PLEKHG3**	-0.47	6.96E-02	1.00E+00	0.43	5.39E-01	9.21E-01
**ENDOG**	-0.53	4.73E-02	1.00E+00	-0.43	5.39E-01	9.22E-01
**AC084117.1**	-0.25	7.83E-01	1.00E+00	0.53	5.39E-01	9.22E-01
**RPL30**	-0.29	3.56E-01	1.00E+00	0.25	5.39E-01	9.22E-01
**TAF3**	-0.06	7.53E-01	1.00E+00	0.11	5.39E-01	9.22E-01
**ECEL1**	-1.67	4.83E-02	1.00E+00	1.93	5.39E-01	9.22E-01
**CALML3-AS1**	-0.10	8.03E-01	1.00E+00	-0.45	5.39E-01	9.22E-01
**CDC42EP4**	-0.39	4.86E-02	1.00E+00	-0.23	5.39E-01	9.22E-01
**EMC1**	0.03	8.80E-01	1.00E+00	0.14	5.39E-01	9.22E-01
**DGKI**	0.23	8.16E-01	1.00E+00	0.43	5.39E-01	9.22E-01
**NEURL3**	-0.20	8.63E-01	1.00E+00	0.64	5.39E-01	9.22E-01

**TCTE3**	-0.29	6.19E-01	1.00E+00	-0.38	5.39E-01	9.22E-01
**AL355355.2**	0.59	3.12E-01	1.00E+00	0.44	5.39E-01	9.22E-01
**FKBP1C**	0.27	7.25E-01	1.00E+00	0.33	5.40E-01	9.22E-01
**FAM135A**	0.19	6.49E-01	1.00E+00	-0.20	5.40E-01	9.22E-01
**RAI14**	-0.16	6.37E-01	1.00E+00	0.18	5.40E-01	9.22E-01
**AADAT**	-0.03	9.46E-01	1.00E+00	-0.33	5.40E-01	9.22E-01
**PAN3**	0.16	3.86E-01	1.00E+00	0.24	5.40E-01	9.22E-01
**RASAL1**	-0.31	3.19E-01	1.00E+00	0.41	5.40E-01	9.22E-01
**THEM4**	-0.29	3.22E-01	1.00E+00	-0.16	5.40E-01	9.22E-01
**ADAD2**	-0.26	7.03E-01	1.00E+00	0.33	5.40E-01	9.22E-01
**AC009549.1**	-1.06	2.96E-01	1.00E+00	-0.70	5.40E-01	9.22E-01
**TSR3**	-0.01	9.63E-01	1.00E+00	-0.41	5.40E-01	9.22E-01
**IGHM**	-0.40	8.23E-01	1.00E+00	1.92	5.40E-01	9.22E-01
**AKIRIN1**	0.24	3.66E-01	1.00E+00	0.19	5.40E-01	9.22E-01
**AC010319.1**	-4.79	3.27E-03	3.02E-01	1.00	5.40E-01	9.22E-01
**DBT**	0.05	8.17E-01	1.00E+00	-0.13	5.40E-01	9.22E-01
**AL121929.2**	-0.79	4.13E-01	1.00E+00	-0.88	5.40E-01	9.22E-01
**PPDPF**	-0.08	7.83E-01	1.00E+00	0.16	5.40E-01	9.22E-01
**SQSTM1**	0.01	9.63E-01	1.00E+00	0.16	5.40E-01	9.22E-01
**PPME1**	0.05	8.17E-01	1.00E+00	-0.13	5.40E-01	9.22E-01
**OGFRP1**	-1.15	4.36E-02	1.00E+00	0.67	5.40E-01	9.22E-01
**FAXDC2**	0.55	6.95E-02	1.00E+00	-0.23	5.40E-01	9.22E-01
**PSMD10**	0.23	1.99E-01	1.00E+00	-0.16	5.40E-01	9.22E-01
**ZNF266**	-0.12	6.45E-01	1.00E+00	0.22	5.41E-01	9.22E-01
**ZNF192P1**	-0.84	6.12E-01	1.00E+00	0.66	5.41E-01	9.23E-01
**INSL6**	2.13	3.24E-01	1.00E+00	-0.53	5.41E-01	9.23E-01
**WNT7B**	-0.06	8.85E-01	1.00E+00	-0.46	5.41E-01	9.23E-01
**SMG9**	0.13	7.53E-01	1.00E+00	0.31	5.41E-01	9.23E-01
**ARSH**	-2.36	7.07E-02	1.00E+00	0.76	5.41E-01	9.23E-01
**TLN1**	-0.04	9.03E-01	1.00E+00	0.36	5.41E-01	9.23E-01
**LINC02246**	0.03	9.87E-01	1.00E+00	-0.69	5.41E-01	9.23E-01
**KLHL28**	0.15	6.22E-01	1.00E+00	-0.24	5.41E-01	9.23E-01

**GABBR1**	-0.36	5.17E-01	1.00E+00	0.34	5.41E-01	9.23E-01
**COG4**	-0.20	4.56E-01	1.00E+00	-0.09	5.41E-01	9.23E-01
**MRPL24**	-0.05	7.72E-01	1.00E+00	-0.17	5.41E-01	9.23E-01
**LINC01485**	3.89	1.07E-02	6.40E-01	-0.68	5.41E-01	9.23E-01
**POLR3F**	-0.01	9.81E-01	1.00E+00	-0.14	5.41E-01	9.23E-01
**HIST1H3G**	0.11	9.54E-01	1.00E+00	0.75	5.41E-01	9.23E-01
**CCDC89**	0.10	7.67E-01	1.00E+00	0.42	5.41E-01	9.23E-01
**PMS2P5**	-0.27	4.98E-01	1.00E+00	-0.19	5.41E-01	9.23E-01
**DDN**	-5.61	3.35E-12	2.25E-09	1.09	5.41E-01	9.23E-01
**AC104031.1**	-1.88	2.31E-01	1.00E+00	-0.42	5.42E-01	9.23E-01
**UBE2V1P2**	1.20	5.88E-01	1.00E+00	0.51	5.42E-01	9.23E-01
**AL022311.1**	-0.23	7.91E-01	1.00E+00	0.37	5.42E-01	9.23E-01
**APTX**	0.06	7.51E-01	1.00E+00	-0.13	5.42E-01	9.23E-01
**ANKFY1**	0.25	2.10E-01	1.00E+00	0.21	5.42E-01	9.23E-01
**RNF2**	-0.07	7.26E-01	1.00E+00	-0.11	5.42E-01	9.23E-01
**AC008443.5**	-0.18	9.22E-01	1.00E+00	0.66	5.42E-01	9.23E-01
**NOVA1**	1.23	3.33E-02	1.00E+00	-0.37	5.42E-01	9.23E-01
**INO80D**	0.16	5.92E-01	1.00E+00	0.20	5.42E-01	9.23E-01
**PLIN4**	4.88	2.86E-03	2.79E-01	-1.91	5.42E-01	9.23E-01
**ARHGEF1**	-0.34	1.29E-01	1.00E+00	0.32	5.42E-01	9.23E-01
**MYSM1**	0.02	9.49E-01	1.00E+00	-0.18	5.42E-01	9.23E-01
**DDX50**	0.21	2.53E-01	1.00E+00	-0.12	5.42E-01	9.23E-01
**AC009362.1**	NA	NA	NA	0.55	5.42E-01	9.23E-01
**RFTN1**	0.33	3.86E-01	1.00E+00	0.31	5.42E-01	9.23E-01
**NME2**	-0.86	4.10E-01	1.00E+00	0.47	5.42E-01	9.23E-01
**GAS5**	-0.02	9.49E-01	1.00E+00	0.22	5.42E-01	9.23E-01
**CCNJL**	-0.33	5.65E-01	1.00E+00	0.54	5.42E-01	9.23E-01
**CIB2**	-0.45	1.66E-01	1.00E+00	0.42	5.42E-01	9.23E-01
**DTNB**	0.05	9.09E-01	1.00E+00	-0.25	5.42E-01	9.23E-01
**ATG16L2**	-0.40	3.34E-01	1.00E+00	0.27	5.42E-01	9.23E-01
**IST1**	0.27	9.84E-02	1.00E+00	0.09	5.42E-01	9.23E-01
**AC008771.1**	0.18	7.33E-01	1.00E+00	-0.28	5.43E-01	9.23E-01

**XLOC_008955**	1.06	4.61E-02	1.00E+00	0.46	5.43E-01	9.23E-01
**CKMT1B**	-0.68	2.87E-04	6.33E-02	-0.28	5.43E-01	9.23E-01
**ZNF829**	0.10	7.40E-01	1.00E+00	-0.18	5.43E-01	9.23E-01
**KIF13A**	-0.05	7.83E-01	1.00E+00	0.28	5.43E-01	9.23E-01
**B3GALT1**	-0.09	9.28E-01	1.00E+00	-0.57	5.43E-01	9.23E-01
**MRPS18B**	0.05	7.62E-01	1.00E+00	-0.14	5.43E-01	9.23E-01
**FBXO48**	0.02	9.73E-01	1.00E+00	-0.26	5.43E-01	9.23E-01
**AC024270.4**	-2.82	1.80E-03	2.38E-01	-0.68	5.43E-01	9.23E-01
**AC006059.1**	-0.83	2.31E-01	1.00E+00	0.42	5.43E-01	9.23E-01
**RPS18**	-0.03	9.30E-01	1.00E+00	0.18	5.43E-01	9.23E-01
**PRMT1**	-0.32	8.59E-02	1.00E+00	0.12	5.43E-01	9.23E-01
**NEDD4L**	0.11	8.21E-01	1.00E+00	-0.22	5.43E-01	9.23E-01
**COX7A2L**	0.23	2.84E-01	1.00E+00	-0.20	5.43E-01	9.23E-01
**ZMAT2**	0.11	5.85E-01	1.00E+00	0.13	5.43E-01	9.23E-01
**SLC27A2**	-0.47	3.29E-01	1.00E+00	-0.45	5.43E-01	9.23E-01
**PDCD2L**	0.42	4.16E-01	1.00E+00	0.22	5.43E-01	9.23E-01
**AC010768.2**	2.49	3.69E-01	1.00E+00	0.66	5.43E-01	9.23E-01
**ZNF815P**	-0.08	8.46E-01	1.00E+00	0.24	5.43E-01	9.23E-01
**IFT46**	0.15	5.82E-01	1.00E+00	0.12	5.44E-01	9.23E-01
**G30615**	-1.79	1.77E-01	1.00E+00	-0.89	5.44E-01	9.23E-01
**C5orf49**	0.47	3.49E-01	1.00E+00	-0.45	5.44E-01	9.23E-01
**MTND1P23**	0.75	4.65E-01	1.00E+00	0.61	5.44E-01	9.23E-01
**CREB1**	0.08	7.09E-01	1.00E+00	-0.10	5.44E-01	9.23E-01
**MAN1B1-DT**	0.16	6.20E-01	1.00E+00	-0.39	5.44E-01	9.23E-01
**TTC39C-AS1**	1.52	3.38E-01	1.00E+00	0.53	5.44E-01	9.23E-01
**TACSTD2**	0.09	8.37E-01	1.00E+00	0.32	5.44E-01	9.23E-01
**AL031123.2**	-1.01	2.30E-01	1.00E+00	-0.70	5.44E-01	9.23E-01
**RNASEH1-AS1**	-0.47	1.97E-01	1.00E+00	-0.21	5.44E-01	9.23E-01
**STK38**	0.18	5.28E-01	1.00E+00	-0.13	5.44E-01	9.23E-01
**SCGB3A2**	0.76	3.19E-01	1.00E+00	-0.51	5.44E-01	9.23E-01
**GLUD2**	0.19	7.88E-01	1.00E+00	-0.33	5.44E-01	9.23E-01
**CROCC**	-0.31	3.39E-01	1.00E+00	0.41	5.44E-01	9.23E-01

**KCNK13**	-1.91	1.56E-02	7.80E-01	0.89	5.44E-01	9.23E-01
**AP000442.2**	-0.76	1.18E-01	1.00E+00	-0.39	5.44E-01	9.23E-01
**MTMR12**	0.22	2.93E-01	1.00E+00	-0.21	5.44E-01	9.23E-01
**XLOC_004632**	-0.98	4.65E-01	1.00E+00	0.42	5.44E-01	9.23E-01
**NCBP2**	0.07	7.20E-01	1.00E+00	-0.12	5.44E-01	9.23E-01
**ADIPOR1**	0.03	8.99E-01	1.00E+00	-0.16	5.44E-01	9.23E-01
**TASP1**	0.03	9.17E-01	1.00E+00	-0.19	5.44E-01	9.23E-01
**CSNK1G3**	0.20	3.93E-01	1.00E+00	-0.16	5.44E-01	9.23E-01
**SERTM1**	-0.04	9.85E-01	1.00E+00	0.79	5.44E-01	9.23E-01
**NEIL3**	-0.10	8.70E-01	1.00E+00	-0.41	5.44E-01	9.23E-01
**AL445472.1**	-0.05	9.27E-01	1.00E+00	0.22	5.44E-01	9.23E-01
**AL137127.1**	-0.01	9.92E-01	1.00E+00	0.41	5.44E-01	9.23E-01
**MAJIN**	-0.88	4.30E-01	1.00E+00	-0.62	5.44E-01	9.23E-01
**NDUFA10**	-0.08	6.77E-01	1.00E+00	-0.12	5.44E-01	9.23E-01
**MAML2**	0.02	9.44E-01	1.00E+00	0.18	5.44E-01	9.23E-01
**PPP4R4**	-0.17	7.37E-01	1.00E+00	0.53	5.44E-01	9.23E-01
**FJX1**	-0.59	1.04E-01	1.00E+00	0.25	5.45E-01	9.23E-01
**KIR2DS4**	0.67	8.06E-01	1.00E+00	0.93	5.45E-01	9.23E-01
**AC244230.1**	-1.50	4.48E-01	1.00E+00	-0.45	5.45E-01	9.23E-01
**DIO3**	0.73	2.66E-01	1.00E+00	1.42	5.45E-01	9.23E-01
**UNC93A**	0.19	7.45E-01	1.00E+00	0.45	5.45E-01	9.23E-01
**PTPMT1**	0.10	8.87E-01	1.00E+00	-0.33	5.45E-01	9.23E-01
**MFAP4**	0.76	1.47E-01	1.00E+00	0.34	5.45E-01	9.23E-01
**DSCC1**	0.10	7.97E-01	1.00E+00	-0.30	5.45E-01	9.23E-01
**AC105277.1**	-0.36	6.02E-01	1.00E+00	0.47	5.45E-01	9.23E-01
**SFT2D2**	0.54	2.31E-02	9.12E-01	-0.19	5.45E-01	9.23E-01
**TMEM91**	-0.01	9.77E-01	1.00E+00	-0.35	5.45E-01	9.23E-01
**AC023157.1**	3.53	2.96E-01	1.00E+00	1.91	5.45E-01	9.23E-01
**H2AFY**	-0.22	4.64E-01	1.00E+00	-0.17	5.45E-01	9.23E-01
**AL512791.2**	0.88	5.68E-01	1.00E+00	-0.62	5.45E-01	9.23E-01
**PEX1**	-0.02	9.21E-01	1.00E+00	-0.12	5.45E-01	9.23E-01
**HDAC5**	0.19	4.26E-01	1.00E+00	-0.23	5.45E-01	9.23E-01

**SUB1P3**	0.66	8.49E-01	1.00E+00	-0.41	5.45E-01	9.23E-01
**DCP1B**	-0.07	6.95E-01	1.00E+00	0.11	5.45E-01	9.23E-01
**RN7SKP80**	0.87	5.62E-01	1.00E+00	-0.69	5.45E-01	9.23E-01
**RNU7-45P**	-0.10	9.75E-01	1.00E+00	0.60	5.45E-01	9.23E-01
**G31689**	-1.87	4.62E-01	1.00E+00	0.57	5.45E-01	9.23E-01
**RDH10**	0.12	6.88E-01	1.00E+00	-0.21	5.45E-01	9.23E-01
**AL360267.2**	-0.99	3.29E-01	1.00E+00	0.56	5.45E-01	9.23E-01
**PCDHB13**	-0.49	1.01E-01	1.00E+00	-0.31	5.46E-01	9.23E-01
**ICE2**	0.03	8.85E-01	1.00E+00	-0.11	5.46E-01	9.23E-01
**GNS**	0.37	3.30E-01	1.00E+00	0.24	5.46E-01	9.23E-01
**KIAA1614**	0.21	5.62E-01	1.00E+00	0.47	5.46E-01	9.23E-01
**UXS1**	0.01	9.69E-01	1.00E+00	-0.08	5.46E-01	9.23E-01
**PRRT1**	-1.07	3.48E-02	1.00E+00	0.46	5.46E-01	9.23E-01
**ABCA7**	-0.21	5.77E-01	1.00E+00	0.35	5.46E-01	9.23E-01
**TXN**	-0.04	8.69E-01	1.00E+00	-0.24	5.46E-01	9.23E-01
**WRAP53**	-0.12	6.32E-01	1.00E+00	0.09	5.46E-01	9.23E-01
**XLOC_007263**	-0.80	7.97E-01	1.00E+00	-0.87	5.46E-01	9.23E-01
**PLAC4**	-0.11	9.21E-01	1.00E+00	-0.63	5.46E-01	9.23E-01
**KATNAL1**	0.22	5.52E-01	1.00E+00	-0.23	5.46E-01	9.23E-01
**SRARP**	1.05	5.58E-01	1.00E+00	0.92	5.46E-01	9.24E-01
**KDM7A-DT**	-0.16	4.42E-01	1.00E+00	0.26	5.46E-01	9.24E-01
**GPR89P**	-1.00	6.89E-01	1.00E+00	-1.43	5.46E-01	9.24E-01
**AC093525.6**	-0.27	8.49E-01	1.00E+00	-0.71	5.46E-01	9.24E-01
**PPBP**	-0.20	9.29E-01	1.00E+00	-1.92	5.46E-01	9.24E-01
**MED11**	-0.08	7.99E-01	1.00E+00	-0.20	5.46E-01	9.24E-01
**AC131009.4**	1.77	2.62E-01	1.00E+00	-0.56	5.46E-01	9.24E-01
**ZBED3-AS1**	1.66	7.94E-02	1.00E+00	-0.50	5.46E-01	9.24E-01
**ZKSCAN1**	0.07	7.82E-01	1.00E+00	-0.16	5.46E-01	9.24E-01
**SLC25A38**	0.37	2.47E-02	9.28E-01	-0.09	5.46E-01	9.24E-01
**STAT3**	-0.18	3.65E-01	1.00E+00	0.20	5.46E-01	9.24E-01
**MYLK4**	0.60	2.55E-01	1.00E+00	0.54	5.46E-01	9.24E-01
**TMEM117**	-0.19	5.18E-01	1.00E+00	-0.25	5.46E-01	9.24E-01

**MPP7**	0.19	6.78E-01	1.00E+00	-0.24	5.47E-01	9.24E-01
**PPM1N**	0.09	8.05E-01	1.00E+00	-0.20	5.47E-01	9.24E-01
**SHLD3**	-0.34	4.61E-01	1.00E+00	0.28	5.47E-01	9.24E-01
**CDCA4**	-0.24	5.33E-01	1.00E+00	0.20	5.47E-01	9.24E-01
**ILF2**	-0.19	3.31E-01	1.00E+00	-0.14	5.47E-01	9.24E-01
**MAP3K14**	-0.90	4.27E-02	1.00E+00	0.45	5.47E-01	9.24E-01
**MAP3K6**	-0.03	9.28E-01	1.00E+00	-0.29	5.47E-01	9.24E-01
**AC105384.1**	1.25	2.92E-01	1.00E+00	0.75	5.47E-01	9.24E-01
**BMP5**	0.34	7.76E-01	1.00E+00	-1.79	5.47E-01	9.24E-01
**AC025280.2**	0.37	7.85E-01	1.00E+00	-0.67	5.47E-01	9.24E-01
**ENO1-AS1**	3.05	3.65E-01	1.00E+00	-0.49	5.47E-01	9.24E-01
**AC132938.3**	-0.22	5.82E-01	1.00E+00	-0.32	5.47E-01	9.24E-01
**CBR1**	0.04	8.84E-01	1.00E+00	-0.24	5.47E-01	9.24E-01
**XLOC_002194**	0.26	8.27E-01	1.00E+00	0.75	5.47E-01	9.24E-01
**G24237**	0.46	6.84E-01	1.00E+00	-0.38	5.47E-01	9.24E-01
**AL356270.1**	-2.17	1.57E-01	1.00E+00	-0.49	5.47E-01	9.24E-01
**DGKZP1**	0.41	5.05E-01	1.00E+00	-0.25	5.48E-01	9.24E-01
**TRIM28**	-0.13	5.62E-01	1.00E+00	0.28	5.48E-01	9.24E-01
**AC105114.2**	-1.24	7.19E-01	1.00E+00	0.87	5.48E-01	9.24E-01
**SLC6A12**	-0.47	4.55E-01	1.00E+00	0.70	5.48E-01	9.24E-01
**PLEKHA3P1**	-1.85	5.10E-01	1.00E+00	0.75	5.48E-01	9.24E-01
**AC004923.1**	-0.80	3.35E-01	1.00E+00	-0.50	5.48E-01	9.24E-01
**NUFIP2**	-0.14	5.00E-01	1.00E+00	0.23	5.48E-01	9.24E-01
**IGKV1D-8**	NA	NA	NA	1.92	5.48E-01	9.24E-01
**G38700**	-1.39	2.10E-01	1.00E+00	0.39	5.48E-01	9.24E-01
**MOBP**	-5.87	2.83E-06	9.97E-04	1.34	5.48E-01	9.24E-01
**TTC39C**	0.12	6.92E-01	1.00E+00	-0.20	5.48E-01	9.24E-01
**SLC31A1**	0.02	9.51E-01	1.00E+00	0.16	5.48E-01	9.24E-01
**BCL6**	-0.22	4.56E-01	1.00E+00	0.18	5.48E-01	9.24E-01
**DHX29**	-0.10	6.34E-01	1.00E+00	-0.11	5.48E-01	9.24E-01
**SETD2**	-0.13	5.63E-01	1.00E+00	0.18	5.48E-01	9.24E-01
**AC004156.1**	-0.61	5.53E-01	1.00E+00	-0.51	5.48E-01	9.24E-01

**SASS6**	-0.05	8.81E-01	1.00E+00	-0.20	5.48E-01	9.24E-01
**MYB**	0.43	3.93E-01	1.00E+00	-0.48	5.48E-01	9.24E-01
**LNCOC1**	1.02	3.11E-01	1.00E+00	-0.74	5.48E-01	9.24E-01
**AC090114.3**	-1.59	1.66E-01	1.00E+00	0.55	5.48E-01	9.24E-01
**NPHP3**	0.65	2.02E-01	1.00E+00	0.27	5.48E-01	9.24E-01
**PPP1R1C**	-0.88	1.81E-01	1.00E+00	-0.33	5.48E-01	9.24E-01
**ZNF404**	-0.21	7.09E-01	1.00E+00	-0.59	5.48E-01	9.24E-01
**GAK**	-0.25	2.21E-01	1.00E+00	0.31	5.48E-01	9.24E-01
**PLA2G4F**	-0.27	5.26E-01	1.00E+00	-0.44	5.48E-01	9.24E-01
**FOXC2**	-0.15	7.88E-01	1.00E+00	0.55	5.49E-01	9.24E-01
**NUP133**	0.09	6.54E-01	1.00E+00	-0.08	5.49E-01	9.24E-01
**NOM1**	-0.05	7.86E-01	1.00E+00	0.31	5.49E-01	9.24E-01
**TENT2**	0.33	1.32E-01	1.00E+00	0.13	5.49E-01	9.24E-01
**LINC01126**	-1.10	3.54E-01	1.00E+00	0.75	5.49E-01	9.24E-01
**NME8**	0.91	6.00E-01	1.00E+00	0.57	5.49E-01	9.24E-01
**ADD3**	0.44	1.23E-01	1.00E+00	-0.15	5.49E-01	9.24E-01
**RAP2C-AS1**	-0.19	5.51E-01	1.00E+00	-0.24	5.49E-01	9.24E-01
**Z95152.1**	-0.39	8.71E-01	1.00E+00	-0.61	5.49E-01	9.24E-01
**LINC01303**	-1.42	4.36E-01	1.00E+00	-0.64	5.49E-01	9.24E-01
**ZNF318**	-0.38	8.30E-02	1.00E+00	0.28	5.49E-01	9.24E-01
**AL359962.1**	0.10	9.06E-01	1.00E+00	-0.47	5.49E-01	9.24E-01
**FOS**	-0.77	4.95E-01	1.00E+00	-0.33	5.49E-01	9.24E-01
**RNF166**	0.23	3.22E-01	1.00E+00	0.36	5.49E-01	9.24E-01
**METTL17**	-0.10	6.72E-01	1.00E+00	-0.13	5.49E-01	9.24E-01
**ABHD11-AS1**	-0.53	5.95E-01	1.00E+00	-0.37	5.49E-01	9.24E-01
**CSF3**	-2.23	5.02E-01	1.00E+00	1.88	5.49E-01	9.24E-01
**INTS13**	0.19	5.80E-01	1.00E+00	0.19	5.49E-01	9.24E-01
**MOCOS**	-0.15	6.52E-01	1.00E+00	0.23	5.49E-01	9.24E-01
**SEPT7-AS1**	-0.34	5.49E-01	1.00E+00	-0.31	5.49E-01	9.24E-01
**ARHGEF11**	-0.24	3.29E-01	1.00E+00	0.50	5.49E-01	9.24E-01
**PNPLA7**	-0.10	7.96E-01	1.00E+00	-0.26	5.49E-01	9.24E-01
**ZKSCAN3**	0.00	9.98E-01	1.00E+00	0.22	5.49E-01	9.24E-01

**LRP2BP**	-0.09	8.92E-01	1.00E+00	-0.28	5.49E-01	9.24E-01
**AL133268.1**	-1.11	4.72E-01	1.00E+00	-0.51	5.49E-01	9.24E-01
**AL596325.2**	0.50	7.12E-01	1.00E+00	0.62	5.49E-01	9.24E-01
**AC006001.2**	-0.35	5.74E-01	1.00E+00	0.20	5.49E-01	9.24E-01
**EMC7**	0.05	7.54E-01	1.00E+00	-0.16	5.49E-01	9.24E-01
**NOL8**	-0.13	5.69E-01	1.00E+00	-0.11	5.50E-01	9.25E-01
**ZNF831**	-1.00	2.54E-01	1.00E+00	1.88	5.50E-01	9.25E-01
**ASB5**	3.24	1.76E-01	1.00E+00	-0.85	5.50E-01	9.25E-01
**GPR137**	-0.26	1.67E-01	1.00E+00	0.25	5.50E-01	9.25E-01
**PRPF6**	-0.12	5.81E-01	1.00E+00	0.12	5.50E-01	9.25E-01
**KRT32**	-4.14	8.42E-03	5.67E-01	-1.88	5.50E-01	9.25E-01
**AL353593.1**	-0.49	4.75E-01	1.00E+00	-0.60	5.50E-01	9.25E-01
**NSFP1**	-1.29	7.04E-01	1.00E+00	0.66	5.50E-01	9.25E-01
**SLC12A3**	-2.63	3.01E-01	1.00E+00	0.87	5.50E-01	9.25E-01
**NAA15**	-0.28	3.09E-01	1.00E+00	0.14	5.50E-01	9.25E-01
**AC011912.1**	0.57	3.58E-01	1.00E+00	0.57	5.50E-01	9.25E-01
**EXPH5**	-0.01	9.69E-01	1.00E+00	-0.40	5.50E-01	9.25E-01
**AL162424.1**	-0.81	1.78E-01	1.00E+00	-0.31	5.50E-01	9.25E-01
**CPEB3**	-0.24	4.66E-01	1.00E+00	-0.28	5.50E-01	9.25E-01
**PITPNA**	0.09	7.31E-01	1.00E+00	-0.28	5.50E-01	9.25E-01
**PTPN14**	0.37	2.96E-01	1.00E+00	-0.17	5.50E-01	9.25E-01
**AC011586.2**	-0.08	9.81E-01	1.00E+00	0.75	5.50E-01	9.25E-01
**HECTD4**	-0.14	4.40E-01	1.00E+00	0.31	5.50E-01	9.25E-01
**AC008764.9**	-4.03	5.42E-02	1.00E+00	-0.86	5.50E-01	9.25E-01
**TMEM242**	0.30	7.91E-02	1.00E+00	-0.20	5.50E-01	9.25E-01
**GDI2P2**	-2.01	5.57E-01	1.00E+00	0.35	5.50E-01	9.25E-01
**LRRC32**	-0.03	9.46E-01	1.00E+00	0.40	5.50E-01	9.25E-01
**ADARB2**	0.23	7.52E-01	1.00E+00	-0.62	5.50E-01	9.25E-01
**CSN1S1**	3.77	2.49E-01	1.00E+00	-1.89	5.51E-01	9.25E-01
**LINC00924**	0.85	2.33E-01	1.00E+00	0.55	5.51E-01	9.25E-01
**SFI1**	-0.12	6.58E-01	1.00E+00	0.21	5.51E-01	9.25E-01
**LINC00964**	0.12	8.76E-01	1.00E+00	-0.50	5.51E-01	9.25E-01

**MYT1L**	-3.55	1.23E-02	6.92E-01	0.53	5.51E-01	9.25E-01
**BBS1**	-0.87	5.72E-01	1.00E+00	-0.34	5.51E-01	9.25E-01
**SAMD11**	-0.58	4.36E-01	1.00E+00	0.72	5.51E-01	9.25E-01
**XLOC_002071**	-0.43	6.60E-01	1.00E+00	-0.60	5.51E-01	9.25E-01
**ILDR2**	-2.50	1.10E-01	1.00E+00	0.83	5.51E-01	9.25E-01
**AES**	-0.17	4.53E-01	1.00E+00	0.29	5.51E-01	9.25E-01
**G36170**	0.62	5.23E-01	1.00E+00	-0.47	5.51E-01	9.25E-01
**LCE1D**	-3.61	1.66E-02	8.10E-01	0.57	5.51E-01	9.25E-01
**DVL1**	-0.07	7.53E-01	1.00E+00	0.41	5.51E-01	9.25E-01
**UBAC2-AS1**	-0.43	4.50E-01	1.00E+00	0.28	5.51E-01	9.25E-01
**AL035446.2**	-1.71	6.18E-01	1.00E+00	-0.67	5.52E-01	9.25E-01
**XLOC_001489**	-0.23	6.97E-01	1.00E+00	-0.38	5.52E-01	9.25E-01
**FSCN2**	-0.72	2.83E-01	1.00E+00	0.57	5.52E-01	9.25E-01
**UPF2**	0.08	6.56E-01	1.00E+00	0.16	5.52E-01	9.25E-01
**CD1C**	-0.71	1.81E-01	1.00E+00	0.28	5.52E-01	9.25E-01
**LYPD6B**	0.32	4.49E-01	1.00E+00	-0.44	5.52E-01	9.25E-01
**AC092171.5**	0.07	8.62E-01	1.00E+00	0.33	5.52E-01	9.25E-01
**TOR1AIP2**	0.22	3.57E-01	1.00E+00	0.16	5.52E-01	9.25E-01
**LYSMD2**	0.34	3.30E-01	1.00E+00	0.22	5.52E-01	9.25E-01
**MAP3K9**	0.22	6.37E-01	1.00E+00	0.35	5.52E-01	9.25E-01
**DEPDC7**	-0.01	9.85E-01	1.00E+00	0.27	5.52E-01	9.25E-01
**TCEAL7**	0.58	4.36E-01	1.00E+00	-0.34	5.52E-01	9.25E-01
**CMTM6**	0.33	3.45E-01	1.00E+00	-0.17	5.52E-01	9.25E-01
**SMG1P1**	-0.17	8.52E-01	1.00E+00	0.33	5.52E-01	9.25E-01
**AC020916.1**	-0.75	1.44E-01	1.00E+00	-0.19	5.52E-01	9.25E-01
**AC107398.2**	-1.34	4.68E-01	1.00E+00	-0.64	5.52E-01	9.25E-01
**AC103957.2**	0.79	3.43E-01	1.00E+00	0.46	5.52E-01	9.25E-01
**DIS3L2**	-0.30	1.40E-01	1.00E+00	0.21	5.52E-01	9.25E-01
**MMS22L**	0.03	9.03E-01	1.00E+00	-0.21	5.52E-01	9.25E-01
**ZNF440**	-0.18	4.58E-01	1.00E+00	-0.18	5.52E-01	9.25E-01
**G35488**	-0.95	3.87E-01	1.00E+00	-0.44	5.52E-01	9.25E-01
**MAK16**	0.22	4.89E-01	1.00E+00	-0.16	5.52E-01	9.25E-01

**KRTAP3-1**	-20.24	1.45E-09	7.84E-07	-1.90	5.52E-01	9.25E-01
**SRP54-AS1**	0.87	1.77E-01	1.00E+00	-0.30	5.52E-01	9.25E-01
**DUS3L**	-0.54	9.67E-02	1.00E+00	0.17	5.52E-01	9.25E-01
**TSC1**	0.47	1.23E-01	1.00E+00	0.12	5.52E-01	9.25E-01
**AL356801.1**	-1.71	5.60E-01	1.00E+00	-0.75	5.53E-01	9.25E-01
**RAP2A**	0.05	8.67E-01	1.00E+00	0.22	5.53E-01	9.25E-01
**UNC119**	-0.17	4.48E-01	1.00E+00	-0.21	5.53E-01	9.25E-01
**AC036214.2**	-0.42	2.95E-01	1.00E+00	0.33	5.53E-01	9.25E-01
**A2M-AS1**	0.45	4.51E-01	1.00E+00	0.39	5.53E-01	9.25E-01
**CR1L**	-1.73	6.12E-01	1.00E+00	0.87	5.53E-01	9.25E-01
**TMEM184B**	0.21	5.89E-01	1.00E+00	0.30	5.53E-01	9.25E-01
**NEXN-AS1**	-1.09	4.94E-01	1.00E+00	-0.51	5.53E-01	9.25E-01
**TCF7L1-IT1**	-1.24	7.19E-01	1.00E+00	1.14	5.53E-01	9.25E-01
**ZNF317**	-0.04	8.06E-01	1.00E+00	0.14	5.53E-01	9.25E-01
**C11orf45**	0.05	8.67E-01	1.00E+00	-0.29	5.53E-01	9.25E-01
**SUSD1**	0.69	1.10E-01	1.00E+00	0.50	5.53E-01	9.25E-01
**GGA1**	-0.22	2.29E-01	1.00E+00	0.27	5.53E-01	9.25E-01
**FAM98C**	-0.12	6.57E-01	1.00E+00	-0.28	5.53E-01	9.25E-01
**UGT2A1**	-1.61	4.03E-01	1.00E+00	-1.89	5.53E-01	9.25E-01
**NR4A1**	-2.49	4.35E-04	8.92E-02	0.44	5.53E-01	9.25E-01
**UGT1A6**	-0.83	3.51E-01	1.00E+00	-0.45	5.53E-01	9.25E-01
**SOWAHC**	-0.11	7.24E-01	1.00E+00	-0.23	5.53E-01	9.25E-01
**SPATA5**	-0.01	9.58E-01	1.00E+00	-0.13	5.53E-01	9.25E-01
**AC118344.2**	-0.72	5.65E-01	1.00E+00	0.55	5.53E-01	9.25E-01
**HAS3**	-0.25	6.66E-01	1.00E+00	0.53	5.54E-01	9.25E-01
**AP2M1**	-0.05	7.75E-01	1.00E+00	0.10	5.54E-01	9.25E-01
**GGT6**	-0.02	9.54E-01	1.00E+00	-0.34	5.54E-01	9.25E-01
**CNN3**	0.19	5.85E-01	1.00E+00	-0.20	5.54E-01	9.25E-01
**AC005842.1**	0.98	7.03E-01	1.00E+00	0.72	5.54E-01	9.25E-01
**LINC01293**	0.51	8.05E-01	1.00E+00	-1.12	5.54E-01	9.25E-01
**BIRC5**	1.06	6.75E-03	4.99E-01	0.32	5.54E-01	9.25E-01
**WDR34**	-0.26	2.88E-01	1.00E+00	0.13	5.54E-01	9.25E-01

**AC092809.2**	-0.89	2.73E-01	1.00E+00	-0.55	5.54E-01	9.25E-01
**SYNE1**	0.09	7.23E-01	1.00E+00	0.19	5.54E-01	9.25E-01
**MIOS**	0.28	2.83E-01	1.00E+00	-0.12	5.54E-01	9.25E-01
**LINC00515**	-2.80	3.52E-02	1.00E+00	-0.48	5.54E-01	9.25E-01
**METTL16**	0.06	7.14E-01	1.00E+00	0.21	5.54E-01	9.25E-01
**ZBTB8OS**	0.22	2.48E-01	1.00E+00	-0.18	5.54E-01	9.25E-01
**BMPR2**	-0.12	4.75E-01	1.00E+00	0.13	5.54E-01	9.25E-01
**GMPS**	-0.12	5.00E-01	1.00E+00	-0.07	5.54E-01	9.25E-01
**AC008537.2**	-0.44	7.03E-01	1.00E+00	0.71	5.54E-01	9.25E-01
**FBXL20**	0.23	2.84E-01	1.00E+00	0.15	5.54E-01	9.25E-01
**FEZ2**	0.25	4.29E-01	1.00E+00	0.22	5.54E-01	9.25E-01
**TRAK2**	0.11	7.87E-01	1.00E+00	-0.21	5.54E-01	9.25E-01
**TPMT**	0.02	9.42E-01	1.00E+00	0.12	5.54E-01	9.25E-01
**AC114490.3**	-0.50	6.10E-01	1.00E+00	-0.46	5.54E-01	9.25E-01
**ANKRA2**	0.63	2.12E-02	8.94E-01	-0.16	5.54E-01	9.26E-01
**AC138409.2**	1.25	9.57E-02	1.00E+00	-0.31	5.54E-01	9.26E-01
**BRMS1L**	0.17	5.27E-01	1.00E+00	0.18	5.54E-01	9.26E-01
**RPSAP70**	0.33	6.67E-01	1.00E+00	-0.50	5.54E-01	9.26E-01
**SNRPD3**	0.05	7.39E-01	1.00E+00	-0.13	5.54E-01	9.26E-01
**TRPM3**	1.33	2.51E-01	1.00E+00	0.53	5.54E-01	9.26E-01
**AC016739.1**	0.01	9.82E-01	1.00E+00	-0.36	5.55E-01	9.26E-01
**ZNF768**	-0.06	7.63E-01	1.00E+00	0.31	5.55E-01	9.26E-01
**TMSB15A**	-0.72	5.90E-01	1.00E+00	0.44	5.55E-01	9.26E-01
**SSC4D**	0.65	3.43E-01	1.00E+00	0.32	5.55E-01	9.26E-01
**CHUK**	0.33	2.28E-01	1.00E+00	-0.30	5.55E-01	9.26E-01
**CARD14**	-0.26	5.48E-01	1.00E+00	0.34	5.55E-01	9.26E-01
**EIF2B4**	0.17	4.25E-01	1.00E+00	0.15	5.55E-01	9.26E-01
**SEC31B**	0.65	3.72E-01	1.00E+00	0.58	5.55E-01	9.26E-01
**TSPYL1**	0.11	4.60E-01	1.00E+00	0.22	5.55E-01	9.26E-01
**SYTL4**	1.15	3.04E-02	1.00E+00	0.35	5.55E-01	9.26E-01
**GBP2**	-0.36	1.06E-01	1.00E+00	0.24	5.55E-01	9.26E-01
**AL135925.1**	-0.82	5.68E-03	4.48E-01	-0.17	5.55E-01	9.26E-01

**PPP1R16B**	0.83	1.53E-01	1.00E+00	0.39	5.55E-01	9.26E-01
**CELSR2**	-0.34	3.28E-01	1.00E+00	0.34	5.55E-01	9.26E-01
**TRIP6**	-0.11	6.71E-01	1.00E+00	0.22	5.55E-01	9.26E-01
**SLC4A2**	-0.29	9.48E-02	1.00E+00	0.38	5.55E-01	9.26E-01
**MRPS11**	-0.13	5.76E-01	1.00E+00	0.14	5.55E-01	9.26E-01
**BRAP**	0.04	8.24E-01	1.00E+00	-0.12	5.55E-01	9.26E-01
**TMEFF2**	-0.94	3.34E-01	1.00E+00	0.61	5.55E-01	9.26E-01
**C1orf50**	0.14	6.75E-01	1.00E+00	0.36	5.55E-01	9.26E-01
**GPIHBP1**	1.14	4.17E-02	1.00E+00	-0.45	5.55E-01	9.26E-01
**B3GNT9**	-0.46	1.22E-01	1.00E+00	0.43	5.55E-01	9.26E-01
**PTPN9**	-0.01	9.74E-01	1.00E+00	0.30	5.55E-01	9.26E-01
**ALDH1L1**	1.44	2.25E-01	1.00E+00	-1.84	5.55E-01	9.26E-01
**CLDN5**	0.48	3.46E-01	1.00E+00	-0.36	5.55E-01	9.26E-01
**EVA1B**	0.12	7.04E-01	1.00E+00	0.40	5.55E-01	9.26E-01
**PPP1R13B**	-0.14	5.46E-01	1.00E+00	0.19	5.55E-01	9.26E-01
**SLA2**	-0.15	8.21E-01	1.00E+00	1.85	5.55E-01	9.26E-01
**HSD17B10**	0.02	9.12E-01	1.00E+00	-0.10	5.55E-01	9.26E-01
**SH3KBP1**	0.16	4.90E-01	1.00E+00	0.14	5.55E-01	9.26E-01
**IL27RA**	-0.12	7.32E-01	1.00E+00	0.37	5.55E-01	9.26E-01
**ITK**	0.48	4.92E-01	1.00E+00	1.70	5.56E-01	9.26E-01
**RN7SL689P**	-0.14	8.61E-01	1.00E+00	-0.31	5.56E-01	9.26E-01
**SYNM**	0.20	7.41E-01	1.00E+00	-0.33	5.56E-01	9.26E-01
**RAB3GAP1**	0.18	3.28E-01	1.00E+00	0.14	5.56E-01	9.26E-01
**G34931**	-0.59	4.22E-01	1.00E+00	0.67	5.56E-01	9.26E-01
**EMC6**	-2.56	3.23E-01	1.00E+00	0.54	5.56E-01	9.26E-01
**KRT74**	-10.41	1.52E-07	6.03E-05	-1.87	5.56E-01	9.26E-01
**GLUD1P2**	-1.46	1.82E-01	1.00E+00	-0.60	5.56E-01	9.26E-01
**LINC02354**	-3.24	8.28E-03	5.61E-01	-0.68	5.56E-01	9.26E-01
**MALINC1**	0.59	3.25E-01	1.00E+00	-0.30	5.56E-01	9.26E-01
**LINC02463**	-0.76	5.99E-01	1.00E+00	-0.67	5.56E-01	9.26E-01
**LINC01554**	-0.20	6.23E-01	1.00E+00	-0.24	5.56E-01	9.26E-01
**SLC52A1**	-0.21	6.58E-01	1.00E+00	-0.43	5.56E-01	9.26E-01

**MMP24**	-0.46	2.43E-01	1.00E+00	-0.58	5.56E-01	9.26E-01
**RBM27**	0.05	8.27E-01	1.00E+00	0.18	5.56E-01	9.26E-01
**LINC00466**	0.17	9.59E-01	1.00E+00	-1.18	5.57E-01	9.26E-01
**G31022**	0.64	3.24E-01	1.00E+00	-0.31	5.57E-01	9.26E-01
**AC018755.3**	-0.19	7.85E-01	1.00E+00	0.67	5.57E-01	9.26E-01
**WI2-3658N16.1**	-0.85	1.26E-01	1.00E+00	0.25	5.57E-01	9.26E-01
**NDUFS6**	-0.07	6.70E-01	1.00E+00	-0.16	5.57E-01	9.26E-01
**GET4**	-0.84	4.18E-01	1.00E+00	0.39	5.57E-01	9.26E-01
**TYRO3**	0.21	4.67E-01	1.00E+00	-0.21	5.57E-01	9.26E-01
**DNAH6**	0.95	3.12E-01	1.00E+00	-0.51	5.57E-01	9.26E-01
**PURPL**	-1.37	6.00E-01	1.00E+00	0.80	5.57E-01	9.26E-01
**PPP1R16A**	-0.10	7.64E-01	1.00E+00	-0.20	5.57E-01	9.27E-01
**DLX6**	0.72	2.50E-01	1.00E+00	0.39	5.57E-01	9.27E-01
**MYO1D**	-0.20	4.02E-01	1.00E+00	0.27	5.57E-01	9.27E-01
**PRNP**	-0.02	9.06E-01	1.00E+00	0.08	5.57E-01	9.27E-01
**AC092611.1**	-0.97	7.77E-01	1.00E+00	-1.05	5.57E-01	9.27E-01
**LPAR6**	0.05	8.69E-01	1.00E+00	-0.20	5.58E-01	9.27E-01
**ZNF140**	-0.05	7.91E-01	1.00E+00	-0.18	5.58E-01	9.27E-01
**AL049844.2**	0.84	6.39E-01	1.00E+00	0.74	5.58E-01	9.27E-01
**RP11-782C8.5**	-1.81	5.87E-01	1.00E+00	-0.50	5.58E-01	9.27E-01
**TVP23A**	-0.09	9.08E-01	1.00E+00	0.52	5.58E-01	9.27E-01
**SAR1A**	0.43	1.21E-01	1.00E+00	-0.16	5.58E-01	9.27E-01
**CTBP2**	0.21	4.70E-01	1.00E+00	0.18	5.58E-01	9.27E-01
**AC016730.1**	0.12	8.90E-01	1.00E+00	-0.66	5.58E-01	9.27E-01
**DOLK**	0.02	9.28E-01	1.00E+00	0.13	5.58E-01	9.27E-01
**RPL22P24**	-0.38	5.68E-01	1.00E+00	0.52	5.58E-01	9.27E-01
**NTMT1**	-0.12	5.97E-01	1.00E+00	0.15	5.58E-01	9.27E-01
**DNAJC19P9**	1.78	6.05E-01	1.00E+00	-0.39	5.58E-01	9.27E-01
**CPD**	0.25	3.25E-01	1.00E+00	0.18	5.58E-01	9.27E-01
**GPR3**	-1.16	2.14E-01	1.00E+00	0.62	5.59E-01	9.27E-01
**HDAC6**	-0.26	1.27E-01	1.00E+00	0.25	5.59E-01	9.27E-01
**PDZPH1P**	-2.63	1.81E-01	1.00E+00	-0.88	5.59E-01	9.27E-01

**PARD6A**	-0.32	4.56E-01	1.00E+00	0.43	5.59E-01	9.27E-01
**ZC3H18**	-0.12	5.97E-01	1.00E+00	0.35	5.59E-01	9.27E-01
**AC137936.2**	1.74	1.66E-01	1.00E+00	0.83	5.59E-01	9.27E-01
**RNFT1-DT**	1.73	6.11E-01	1.00E+00	0.50	5.59E-01	9.27E-01
**ADRA1D**	-0.08	9.13E-01	1.00E+00	0.66	5.59E-01	9.27E-01
**ST13P19**	-0.37	8.30E-01	1.00E+00	-0.35	5.59E-01	9.27E-01
**ZNF749**	-0.51	3.10E-01	1.00E+00	0.21	5.59E-01	9.27E-01
**XLOC_014103**	-1.37	1.79E-02	8.38E-01	-0.36	5.59E-01	9.27E-01
**WDR82**	0.08	5.28E-01	1.00E+00	0.24	5.59E-01	9.27E-01
**ROR1-AS1**	2.71	2.25E-01	1.00E+00	-0.51	5.59E-01	9.27E-01
**AP001107.1**	1.15	5.56E-01	1.00E+00	0.66	5.59E-01	9.27E-01
**ZNF274**	0.08	6.95E-01	1.00E+00	-0.14	5.59E-01	9.27E-01
**SSR4**	-0.28	9.13E-02	1.00E+00	0.15	5.59E-01	9.27E-01
**XLOC_011766**	0.60	7.55E-01	1.00E+00	0.70	5.59E-01	9.27E-01
**PKIB**	0.02	9.66E-01	1.00E+00	-0.35	5.59E-01	9.27E-01
**MRPS9**	-0.21	2.98E-01	1.00E+00	-0.21	5.59E-01	9.27E-01
**FBLN5**	0.57	2.39E-01	1.00E+00	0.29	5.59E-01	9.27E-01
**EFCAB1**	0.18	6.03E-01	1.00E+00	-0.38	5.59E-01	9.27E-01
**TSHZ2**	0.33	3.25E-01	1.00E+00	0.17	5.59E-01	9.27E-01
**LINC00184**	2.42	3.59E-01	1.00E+00	-0.87	5.59E-01	9.27E-01
**LRP11**	0.46	3.10E-01	1.00E+00	-0.37	5.59E-01	9.27E-01
**AL691403.2**	-3.61	3.44E-02	1.00E+00	0.55	5.59E-01	9.27E-01
**RPL7AP66**	0.39	6.32E-01	1.00E+00	0.52	5.59E-01	9.27E-01
**ATP1A1**	-0.04	9.09E-01	1.00E+00	0.26	5.59E-01	9.27E-01
**AC099329.2**	-0.70	6.73E-01	1.00E+00	0.73	5.59E-01	9.27E-01
**CENPX**	-0.21	3.27E-01	1.00E+00	0.17	5.59E-01	9.27E-01
**DAGLB**	0.23	3.55E-01	1.00E+00	0.20	5.59E-01	9.27E-01
**DIMT1**	0.00	9.99E-01	1.00E+00	0.13	5.59E-01	9.27E-01
**KIF1C**	0.06	8.09E-01	1.00E+00	0.30	5.60E-01	9.27E-01
**G3900**	0.21	8.28E-01	1.00E+00	-0.40	5.60E-01	9.27E-01
**DNER**	0.00	9.95E-01	1.00E+00	-0.44	5.60E-01	9.27E-01
**AL049840.2**	0.07	9.60E-01	1.00E+00	-0.52	5.60E-01	9.27E-01

**USP7**	0.07	7.73E-01	1.00E+00	-0.19	5.60E-01	9.27E-01
**AL139383.1**	-0.49	4.94E-01	1.00E+00	-0.31	5.60E-01	9.27E-01
**TPST2**	0.20	4.37E-01	1.00E+00	0.14	5.60E-01	9.27E-01
**SLC52A3**	0.70	7.65E-02	1.00E+00	-0.36	5.60E-01	9.27E-01
**AL604028.2**	0.89	4.31E-01	1.00E+00	0.58	5.60E-01	9.27E-01
**BCAS2P2**	-1.12	7.40E-01	1.00E+00	-0.35	5.60E-01	9.27E-01
**MRPL37P1**	-0.34	8.14E-01	1.00E+00	-0.66	5.60E-01	9.27E-01
**ROGDI**	-0.46	3.88E-01	1.00E+00	-0.15	5.60E-01	9.27E-01
**AC022915.2**	0.76	4.41E-01	1.00E+00	-0.81	5.60E-01	9.27E-01
**MIR4432HG**	-0.76	1.13E-01	1.00E+00	0.46	5.60E-01	9.27E-01
**FOPNL**	0.40	9.73E-02	1.00E+00	-0.19	5.60E-01	9.27E-01
**LINC01070**	3.28	2.05E-01	1.00E+00	-0.78	5.60E-01	9.27E-01
**COX6B2**	-1.54	7.01E-02	1.00E+00	-0.73	5.60E-01	9.27E-01
**STAG3L1**	0.14	8.19E-01	1.00E+00	0.22	5.60E-01	9.27E-01
**SSTR1**	0.26	6.42E-01	1.00E+00	0.59	5.60E-01	9.28E-01
**AC022148.1**	-2.58	1.71E-01	1.00E+00	0.94	5.60E-01	9.28E-01
**VLDLR**	1.06	1.61E-01	1.00E+00	-0.31	5.60E-01	9.28E-01
**AC002350.1**	0.08	9.29E-01	1.00E+00	0.29	5.61E-01	9.28E-01
**CYP4F12**	-0.01	9.83E-01	1.00E+00	-0.28	5.61E-01	9.28E-01
**AC040970.1**	0.38	6.77E-01	1.00E+00	0.40	5.61E-01	9.28E-01
**COG1**	-0.18	3.49E-01	1.00E+00	-0.10	5.61E-01	9.28E-01
**PLCXD3**	0.55	3.32E-01	1.00E+00	0.50	5.61E-01	9.28E-01
**MORF4L2-AS1**	0.02	9.93E-01	1.00E+00	-0.51	5.61E-01	9.28E-01
**CEACAM6**	-0.51	4.10E-01	1.00E+00	-0.37	5.61E-01	9.28E-01
**PHF23**	0.01	9.76E-01	1.00E+00	0.28	5.61E-01	9.28E-01
**VNN2**	0.57	5.33E-01	1.00E+00	0.63	5.61E-01	9.28E-01
**CLDN7**	0.64	3.73E-01	1.00E+00	-0.23	5.61E-01	9.28E-01
**AC007389.3**	-0.70	4.92E-01	1.00E+00	-0.68	5.61E-01	9.28E-01
**METTL15**	0.01	9.66E-01	1.00E+00	-0.14	5.61E-01	9.28E-01
**TAS1R1**	0.55	5.47E-01	1.00E+00	-0.75	5.61E-01	9.28E-01
**FPR2**	0.27	8.77E-01	1.00E+00	1.83	5.61E-01	9.28E-01
**SRP14P2**	0.56	8.71E-01	1.00E+00	-0.38	5.61E-01	9.28E-01

**XLOC_004829**	-2.53	3.13E-02	1.00E+00	-0.36	5.61E-01	9.28E-01
**AL080317.3**	0.44	7.38E-01	1.00E+00	-0.38	5.61E-01	9.28E-01
**HIST3H2A**	-0.31	5.19E-01	1.00E+00	0.29	5.61E-01	9.28E-01
**NKAPD1**	0.19	2.99E-01	1.00E+00	0.11	5.62E-01	9.28E-01
**ARF5**	-0.15	5.61E-01	1.00E+00	0.12	5.62E-01	9.28E-01
**AC008105.3**	-1.32	4.63E-01	1.00E+00	0.63	5.62E-01	9.28E-01
**IGFBP5**	0.74	1.49E-01	1.00E+00	0.29	5.62E-01	9.28E-01
**CNTROB**	-0.11	6.13E-01	1.00E+00	0.28	5.62E-01	9.28E-01
**MGLL**	0.69	1.89E-01	1.00E+00	-0.25	5.62E-01	9.28E-01
**RP11-417J8.6**	0.74	4.69E-01	1.00E+00	0.47	5.62E-01	9.28E-01
**SP3**	0.04	8.62E-01	1.00E+00	-0.16	5.62E-01	9.28E-01
**MUC3A**	-0.40	7.82E-01	1.00E+00	0.68	5.62E-01	9.28E-01
**PANK2**	0.18	3.82E-01	1.00E+00	-0.30	5.62E-01	9.28E-01
**DDX3X**	0.05	8.53E-01	1.00E+00	0.11	5.62E-01	9.28E-01
**PYGB**	-0.16	6.74E-01	1.00E+00	-0.26	5.62E-01	9.28E-01
**AC124798.1**	0.19	7.14E-01	1.00E+00	-0.43	5.62E-01	9.28E-01
**PKIA-AS1**	-2.49	2.47E-01	1.00E+00	0.58	5.62E-01	9.28E-01
**PHC1**	0.10	8.04E-01	1.00E+00	0.39	5.62E-01	9.28E-01
**KIAA0040**	-0.15	6.29E-01	1.00E+00	-0.32	5.62E-01	9.28E-01
**LINC00622**	1.37	2.20E-01	1.00E+00	1.46	5.62E-01	9.28E-01
**AC068522.1**	-0.23	9.23E-01	1.00E+00	0.31	5.62E-01	9.28E-01
**PERM1**	0.21	6.52E-01	1.00E+00	0.33	5.62E-01	9.28E-01
**WASH2P**	0.52	7.37E-01	1.00E+00	0.31	5.62E-01	9.28E-01
**AC083973.1**	-1.41	4.71E-02	1.00E+00	0.38	5.62E-01	9.28E-01
**ZNF549**	0.18	6.50E-01	1.00E+00	-0.27	5.62E-01	9.28E-01
**AC022107.1**	-0.29	4.51E-01	1.00E+00	0.35	5.62E-01	9.28E-01
**SART1**	-0.17	4.23E-01	1.00E+00	0.32	5.62E-01	9.28E-01
**NSFL1C**	-0.12	5.53E-01	1.00E+00	0.10	5.62E-01	9.28E-01
**UTY**	0.34	5.56E-01	1.00E+00	-0.50	5.63E-01	9.28E-01
**AC138356.2**	0.16	8.38E-01	1.00E+00	-0.28	5.63E-01	9.28E-01
**G35086**	0.13	8.75E-01	1.00E+00	-0.46	5.63E-01	9.28E-01
**CYP51A1P2**	1.32	6.04E-01	1.00E+00	-0.30	5.63E-01	9.28E-01

**IBA57-DT**	-0.71	6.37E-01	1.00E+00	-0.66	5.63E-01	9.28E-01
**AF001548.2**	0.50	3.63E-01	1.00E+00	-0.35	5.63E-01	9.28E-01
**SYNPO2**	-0.28	5.84E-01	1.00E+00	0.24	5.63E-01	9.28E-01
**EPHB1**	-0.04	9.11E-01	1.00E+00	-0.26	5.63E-01	9.28E-01
**INSYN1**	0.20	6.20E-01	1.00E+00	-0.54	5.63E-01	9.28E-01
**ZNF516**	-0.19	4.42E-01	1.00E+00	0.21	5.63E-01	9.28E-01
**NSMCE2**	-0.10	6.69E-01	1.00E+00	-0.12	5.63E-01	9.28E-01
**HSBP1**	0.02	9.14E-01	1.00E+00	-0.17	5.63E-01	9.28E-01
**MAGEH1**	0.16	6.32E-01	1.00E+00	0.18	5.63E-01	9.28E-01
**PPP1R21**	-0.07	6.95E-01	1.00E+00	-0.10	5.63E-01	9.28E-01
**MEIS2**	-0.03	9.41E-01	1.00E+00	0.27	5.63E-01	9.28E-01
**DOCK1**	0.29	2.69E-01	1.00E+00	0.14	5.63E-01	9.28E-01
**SLU7**	0.11	5.02E-01	1.00E+00	-0.11	5.63E-01	9.28E-01
**LINS1**	-0.08	8.41E-01	1.00E+00	-0.19	5.63E-01	9.28E-01
**ASCL2**	-0.09	8.13E-01	1.00E+00	-0.51	5.63E-01	9.28E-01
**CNBD2**	0.04	9.69E-01	1.00E+00	-0.53	5.63E-01	9.28E-01
**SPAG9**	-0.01	9.77E-01	1.00E+00	0.21	5.63E-01	9.28E-01
**SREBF2**	0.07	8.26E-01	1.00E+00	-0.32	5.63E-01	9.28E-01
**LINC00235**	-0.12	7.84E-01	1.00E+00	0.49	5.63E-01	9.28E-01
**PFN1P1**	0.83	7.11E-01	1.00E+00	-0.39	5.63E-01	9.28E-01
**RAB11FIP3**	-0.24	4.07E-01	1.00E+00	0.49	5.64E-01	9.28E-01
**RAP1GDS1**	0.04	8.30E-01	1.00E+00	0.07	5.64E-01	9.28E-01
**RRAGB**	0.49	2.60E-02	9.40E-01	-0.16	5.64E-01	9.28E-01
**CXCL6**	2.30	3.58E-01	1.00E+00	1.84	5.64E-01	9.28E-01
**ZNF418**	0.12	7.75E-01	1.00E+00	-0.33	5.64E-01	9.28E-01
**MTSS1**	-0.14	6.12E-01	1.00E+00	0.14	5.64E-01	9.28E-01
**ARHGEF26-AS1**	-0.40	6.70E-01	1.00E+00	0.44	5.64E-01	9.28E-01
**ADARB1**	0.00	9.95E-01	1.00E+00	0.31	5.64E-01	9.28E-01
**AL157786.1**	1.93	2.42E-01	1.00E+00	0.56	5.64E-01	9.28E-01
**TRAPPC2**	0.39	2.34E-01	1.00E+00	-0.17	5.64E-01	9.28E-01
**EMC9**	-0.09	7.86E-01	1.00E+00	-0.15	5.64E-01	9.28E-01
**DGLUCY**	-0.17	4.25E-01	1.00E+00	0.10	5.64E-01	9.28E-01

**XLOC_007999**	0.52	7.16E-01	1.00E+00	-0.53	5.64E-01	9.28E-01
**SCAMP3**	-0.10	5.59E-01	1.00E+00	-0.13	5.64E-01	9.28E-01
**MATN1-AS1**	0.68	5.03E-01	1.00E+00	-0.53	5.64E-01	9.28E-01
**AC012442.1**	0.02	9.86E-01	1.00E+00	-0.49	5.64E-01	9.28E-01
**DCC**	0.46	5.44E-01	1.00E+00	-0.77	5.64E-01	9.28E-01
**EBF1**	0.58	2.84E-01	1.00E+00	-0.28	5.64E-01	9.28E-01
**MROH3P**	-2.40	3.02E-01	1.00E+00	0.69	5.64E-01	9.28E-01
**SYBU**	0.61	1.24E-02	6.93E-01	-0.21	5.64E-01	9.28E-01
**AC136604.3**	-1.13	4.83E-01	1.00E+00	-0.40	5.64E-01	9.28E-01
**DHX16**	-0.14	5.33E-01	1.00E+00	0.18	5.64E-01	9.28E-01
**AC006970.1**	NA	NA	NA	-0.91	5.64E-01	9.28E-01
**RANGRF**	-0.23	9.43E-01	1.00E+00	-0.39	5.64E-01	9.28E-01
**G7822**	0.12	8.94E-01	1.00E+00	-0.44	5.64E-01	9.29E-01
**C1QTNF7**	1.08	2.55E-02	9.29E-01	0.42	5.64E-01	9.29E-01
**RAB9A**	0.10	6.44E-01	1.00E+00	-0.15	5.65E-01	9.29E-01
**MED27**	-0.39	1.19E-01	1.00E+00	0.10	5.65E-01	9.29E-01
**AP000254.1**	1.10	1.43E-01	1.00E+00	-0.26	5.65E-01	9.29E-01
**TMEM44-AS1**	0.08	8.42E-01	1.00E+00	-0.22	5.65E-01	9.29E-01
**BCL2**	0.35	1.20E-01	1.00E+00	0.27	5.65E-01	9.29E-01
**AC241585.1**	-1.76	8.72E-02	1.00E+00	0.42	5.65E-01	9.29E-01
**PCNA**	-0.16	5.20E-01	1.00E+00	0.12	5.65E-01	9.29E-01
**PPEF1**	-1.68	5.56E-02	1.00E+00	0.70	5.65E-01	9.29E-01
**AC005840.3**	1.56	4.48E-01	1.00E+00	0.73	5.65E-01	9.29E-01
**RHBDD2**	-0.27	2.00E-01	1.00E+00	0.08	5.65E-01	9.29E-01
**VSIG2**	0.10	8.78E-01	1.00E+00	-0.38	5.65E-01	9.29E-01
**MUC16**	-0.17	8.90E-01	1.00E+00	-0.58	5.65E-01	9.29E-01
**SNX4**	0.33	1.77E-01	1.00E+00	-0.15	5.65E-01	9.29E-01
**ZNF69**	-0.61	3.96E-01	1.00E+00	0.28	5.65E-01	9.29E-01
**WWTR1-AS1**	-0.10	9.05E-01	1.00E+00	0.54	5.65E-01	9.29E-01
**XLOC_007993**	-1.24	2.70E-02	9.59E-01	0.41	5.65E-01	9.29E-01
**CENPC**	-0.13	5.16E-01	1.00E+00	-0.14	5.65E-01	9.29E-01
**GRHL1**	-0.06	8.82E-01	1.00E+00	-0.25	5.65E-01	9.29E-01

**ZG16B**	0.09	8.84E-01	1.00E+00	-0.22	5.65E-01	9.29E-01
**XLOC_006630**	-0.24	7.84E-01	1.00E+00	-0.39	5.65E-01	9.29E-01
**TMEM192**	0.27	1.96E-01	1.00E+00	-0.11	5.66E-01	9.29E-01
**AC084871.1**	2.74	1.93E-01	1.00E+00	0.39	5.66E-01	9.29E-01
**AC023794.3**	-0.42	5.02E-01	1.00E+00	0.43	5.66E-01	9.29E-01
**TFB2M**	0.29	3.43E-01	1.00E+00	-0.15	5.66E-01	9.29E-01
**ACTG1P3**	0.64	6.88E-01	1.00E+00	-0.89	5.66E-01	9.29E-01
**KRTAP13-2**	-6.43	2.82E-02	9.75E-01	1.48	5.66E-01	9.29E-01
**AC009414.2**	0.08	8.75E-01	1.00E+00	-0.34	5.66E-01	9.29E-01
**KRTAP10-6**	-1.60	4.78E-01	1.00E+00	1.81	5.66E-01	9.29E-01
**G23773**	-2.50	1.30E-01	1.00E+00	-0.66	5.66E-01	9.29E-01
**AC006378.1**	-0.50	5.20E-01	1.00E+00	0.34	5.66E-01	9.29E-01
**DDX54**	-0.25	2.72E-01	1.00E+00	0.27	5.66E-01	9.29E-01
**ALYREF**	-0.14	5.73E-01	1.00E+00	-0.37	5.66E-01	9.29E-01
**ABHD14A**	-1.16	7.66E-02	1.00E+00	-0.33	5.66E-01	9.29E-01
**NBPF24**	0.59	3.77E-01	1.00E+00	0.42	5.66E-01	9.29E-01
**HSD17B11**	0.32	4.36E-01	1.00E+00	-0.19	5.66E-01	9.29E-01
**TAF4**	-0.09	7.44E-01	1.00E+00	0.26	5.66E-01	9.29E-01
**AC015712.6**	0.75	5.41E-01	1.00E+00	-0.65	5.66E-01	9.29E-01
**RFX2**	-0.27	5.86E-01	1.00E+00	0.17	5.66E-01	9.29E-01
**FLT3**	-0.18	7.51E-01	1.00E+00	-0.47	5.66E-01	9.29E-01
**SREBF1**	-0.33	3.33E-01	1.00E+00	-0.42	5.66E-01	9.29E-01
**CHODL**	-0.60	4.23E-02	1.00E+00	-0.25	5.66E-01	9.29E-01
**G19261**	-1.16	1.73E-01	1.00E+00	-0.54	5.66E-01	9.29E-01
**USP24**	0.34	1.91E-01	1.00E+00	0.23	5.66E-01	9.29E-01
**CSF1**	0.06	8.46E-01	1.00E+00	0.38	5.66E-01	9.29E-01
**ZNF674-AS1**	0.22	6.24E-01	1.00E+00	0.25	5.66E-01	9.29E-01
**SIKE1**	0.25	3.19E-01	1.00E+00	-0.14	5.66E-01	9.29E-01
**EIF4BP7**	0.19	7.07E-01	1.00E+00	0.25	5.66E-01	9.29E-01
**AP006219.1**	-0.18	9.23E-01	1.00E+00	0.84	5.67E-01	9.29E-01
**MGC4771**	3.39	1.63E-01	1.00E+00	-0.45	5.67E-01	9.29E-01
**FAM166B**	-0.62	2.19E-01	1.00E+00	-0.46	5.67E-01	9.29E-01

**GPATCH4**	-0.18	4.49E-01	1.00E+00	0.16	5.67E-01	9.29E-01
**AC079834.2**	-0.82	1.86E-01	1.00E+00	-0.34	5.67E-01	9.29E-01
**BNC2**	-0.09	8.52E-01	1.00E+00	0.21	5.67E-01	9.29E-01
**FAM161B**	0.29	3.81E-01	1.00E+00	-0.23	5.67E-01	9.29E-01
**B4GALT7**	0.02	9.23E-01	1.00E+00	-0.14	5.67E-01	9.29E-01
**AL162718.1**	-1.91	4.97E-01	1.00E+00	-0.51	5.67E-01	9.29E-01
**UVSSA**	-0.08	8.17E-01	1.00E+00	0.16	5.67E-01	9.29E-01
**CA3**	0.39	6.40E-01	1.00E+00	-1.45	5.67E-01	9.29E-01
**ACTBP2**	0.39	9.10E-01	1.00E+00	0.47	5.67E-01	9.29E-01
**AC010273.2**	-0.12	9.73E-01	1.00E+00	0.63	5.67E-01	9.29E-01
**IGLV9-49**	2.25	5.10E-01	1.00E+00	1.60	5.67E-01	9.29E-01
**DNASE2**	0.07	7.09E-01	1.00E+00	0.13	5.67E-01	9.29E-01
**TBC1D15**	0.08	7.39E-01	1.00E+00	-0.09	5.67E-01	9.29E-01
**ITGAV**	0.00	9.87E-01	1.00E+00	0.15	5.67E-01	9.29E-01
**KLHL2P1**	-3.23	1.61E-01	1.00E+00	0.52	5.67E-01	9.29E-01
**CSTF1**	0.14	4.32E-01	1.00E+00	-0.14	5.67E-01	9.29E-01
**HECTD1**	0.13	5.70E-01	1.00E+00	0.15	5.67E-01	9.29E-01
**XLOC_008072**	-0.69	5.84E-02	1.00E+00	-0.32	5.68E-01	9.29E-01
**G4330**	-1.26	4.83E-02	1.00E+00	0.46	5.68E-01	9.29E-01
**BCS1L**	-0.13	5.59E-01	1.00E+00	-0.15	5.68E-01	9.29E-01
**CHL1**	-0.10	7.72E-01	1.00E+00	-0.23	5.68E-01	9.29E-01
**AC087071.1**	-0.82	7.31E-01	1.00E+00	-0.70	5.68E-01	9.29E-01
**AL133342.1**	-0.93	7.87E-01	1.00E+00	-0.98	5.68E-01	9.29E-01
**ZNF428**	-0.12	7.68E-01	1.00E+00	0.29	5.68E-01	9.29E-01
**TMEM14DP**	1.93	5.74E-01	1.00E+00	-0.31	5.68E-01	9.29E-01
**RPSAP9**	-1.04	2.14E-01	1.00E+00	-0.28	5.68E-01	9.29E-01
**ZNF728**	-2.24	1.56E-01	1.00E+00	0.84	5.68E-01	9.30E-01
**TCTEX1D4**	-0.87	5.30E-01	1.00E+00	0.64	5.68E-01	9.30E-01
**VIM**	0.12	7.40E-01	1.00E+00	0.19	5.68E-01	9.30E-01
**ADORA3**	0.77	4.00E-01	1.00E+00	0.45	5.68E-01	9.30E-01
**XLOC_003364**	-3.06	1.10E-01	1.00E+00	-0.67	5.68E-01	9.30E-01
**SSNA1**	-0.12	6.25E-01	1.00E+00	0.11	5.68E-01	9.30E-01

**CDK8**	0.03	9.04E-01	1.00E+00	0.30	5.68E-01	9.30E-01
**LINC00702**	0.34	5.11E-01	1.00E+00	-0.32	5.68E-01	9.30E-01
**SLC1A4**	-0.14	6.14E-01	1.00E+00	-0.22	5.68E-01	9.30E-01
**LINC00877**	0.76	5.18E-01	1.00E+00	0.43	5.68E-01	9.30E-01
**RABIF**	0.13	5.04E-01	1.00E+00	0.16	5.68E-01	9.30E-01
**XLOC_013461**	-0.22	7.16E-01	1.00E+00	-0.45	5.69E-01	9.30E-01
**SCNM1**	-0.17	3.76E-01	1.00E+00	0.14	5.69E-01	9.30E-01
**S100A12**	-1.10	6.79E-01	1.00E+00	1.79	5.69E-01	9.30E-01
**CHD4**	-0.06	8.46E-01	1.00E+00	-0.18	5.69E-01	9.30E-01
**HOXC8**	0.09	7.93E-01	1.00E+00	0.22	5.69E-01	9.30E-01
**SGCB**	0.48	1.50E-01	1.00E+00	-0.18	5.69E-01	9.30E-01
**PARPBP**	0.62	2.92E-01	1.00E+00	-0.18	5.69E-01	9.30E-01
**FUK**	-0.12	5.35E-01	1.00E+00	0.28	5.69E-01	9.30E-01
**AC246817.2**	0.57	3.98E-01	1.00E+00	-0.38	5.69E-01	9.30E-01
**ZNF624**	-0.13	7.12E-01	1.00E+00	0.17	5.69E-01	9.30E-01
**AC091982.1**	0.54	6.11E-01	1.00E+00	0.60	5.69E-01	9.30E-01
**IPO11**	0.03	8.97E-01	1.00E+00	0.16	5.69E-01	9.30E-01
**IRAK1**	-0.11	5.50E-01	1.00E+00	0.33	5.69E-01	9.30E-01
**AP001528.2**	0.92	3.67E-01	1.00E+00	-0.46	5.69E-01	9.30E-01
**AC006566.1**	-0.64	8.49E-01	1.00E+00	-0.62	5.69E-01	9.30E-01
**TRBV28**	-0.03	9.80E-01	1.00E+00	-0.75	5.69E-01	9.30E-01
**MIR570**	-1.50	6.52E-01	1.00E+00	-0.49	5.69E-01	9.30E-01
**DNAJC30**	0.02	9.55E-01	1.00E+00	0.12	5.69E-01	9.30E-01
**HCAR1**	1.03	3.96E-01	1.00E+00	0.51	5.69E-01	9.30E-01
**ANKRD23**	-0.26	8.36E-01	1.00E+00	0.52	5.69E-01	9.30E-01
**ZNHIT1**	-0.08	5.92E-01	1.00E+00	0.11	5.69E-01	9.30E-01
**IRF8**	-0.01	9.88E-01	1.00E+00	0.37	5.69E-01	9.30E-01
**RAPH1**	-0.23	4.67E-01	1.00E+00	0.30	5.69E-01	9.30E-01
**PTCH2**	0.01	9.92E-01	1.00E+00	0.43	5.70E-01	9.30E-01
**IGKV1-8**	1.23	6.58E-01	1.00E+00	1.82	5.70E-01	9.30E-01
**ASB3**	-1.28	2.45E-01	1.00E+00	-0.33	5.70E-01	9.30E-01
**PRAME**	1.60	6.32E-01	1.00E+00	0.95	5.70E-01	9.30E-01

**SLC25A44**	0.08	6.61E-01	1.00E+00	0.13	5.70E-01	9.30E-01
**AC244033.2**	-0.02	9.75E-01	1.00E+00	0.42	5.70E-01	9.30E-01
**AC073410.1**	-1.63	3.42E-01	1.00E+00	0.73	5.70E-01	9.30E-01
**HEATR5A**	0.44	1.96E-01	1.00E+00	-0.18	5.70E-01	9.30E-01
**AC015911.7**	0.28	7.89E-01	1.00E+00	1.80	5.70E-01	9.30E-01
**G33445**	0.23	5.27E-01	1.00E+00	0.29	5.70E-01	9.30E-01
**SERPINA11**	-2.84	7.81E-03	5.43E-01	-0.64	5.70E-01	9.30E-01
**TNPO1**	0.33	1.31E-01	1.00E+00	0.13	5.70E-01	9.30E-01
**TMED8**	0.15	5.32E-01	1.00E+00	0.19	5.70E-01	9.30E-01
**ZNF222**	-0.28	5.34E-01	1.00E+00	0.32	5.70E-01	9.30E-01
**BIVM**	0.41	2.85E-01	1.00E+00	-0.17	5.70E-01	9.30E-01
**LHFPL1**	0.38	7.75E-01	1.00E+00	0.72	5.70E-01	9.30E-01
**LATS1**	-0.06	7.76E-01	1.00E+00	-0.18	5.70E-01	9.30E-01
**FRMD7**	-1.17	2.36E-01	1.00E+00	-1.49	5.70E-01	9.30E-01
**PHC1P1**	-0.79	7.98E-02	1.00E+00	0.55	5.70E-01	9.30E-01
**AKAP10**	0.16	3.45E-01	1.00E+00	-0.21	5.70E-01	9.30E-01
**MORF4L1**	0.24	2.85E-01	1.00E+00	-0.12	5.70E-01	9.30E-01
**ICMT**	-0.16	2.88E-01	1.00E+00	0.16	5.71E-01	9.30E-01
**IQGAP2**	0.20	7.36E-01	1.00E+00	0.32	5.71E-01	9.30E-01
**AC007342.5**	-1.04	4.29E-01	1.00E+00	0.54	5.71E-01	9.30E-01
**CD81-AS1**	-0.18	8.47E-01	1.00E+00	-0.73	5.71E-01	9.30E-01
**AC004112.1**	-1.13	4.95E-01	1.00E+00	-0.57	5.71E-01	9.30E-01
**CDK7**	0.16	5.51E-01	1.00E+00	-0.20	5.71E-01	9.30E-01
**AC022034.1**	0.15	7.66E-01	1.00E+00	-0.37	5.71E-01	9.30E-01
**NBEAL1**	0.19	5.01E-01	1.00E+00	-0.17	5.71E-01	9.30E-01
**AC122713.2**	1.06	5.82E-01	1.00E+00	0.83	5.71E-01	9.30E-01
**PRKXP1**	-0.99	3.12E-01	1.00E+00	-0.66	5.71E-01	9.30E-01
**AC023794.2**	0.50	7.97E-01	1.00E+00	-0.58	5.71E-01	9.30E-01
**RNF126**	-0.31	3.78E-01	1.00E+00	-0.18	5.71E-01	9.30E-01
**UBE2D1**	0.13	7.00E-01	1.00E+00	-0.19	5.71E-01	9.30E-01
**OR7E39P**	0.29	7.59E-01	1.00E+00	-0.49	5.71E-01	9.30E-01
**PCDHGA9**	-0.19	6.22E-01	1.00E+00	-0.39	5.71E-01	9.30E-01

**PLEKHH1**	-0.48	2.43E-01	1.00E+00	-0.28	5.71E-01	9.30E-01
**NUP155**	-0.10	6.43E-01	1.00E+00	-0.11	5.71E-01	9.30E-01
**PIGA**	-0.08	8.20E-01	1.00E+00	0.19	5.71E-01	9.30E-01
**MESP1**	2.31	1.52E-01	1.00E+00	-0.45	5.71E-01	9.30E-01
**ACVR2B-AS1**	-0.35	5.36E-01	1.00E+00	0.44	5.71E-01	9.30E-01
**LTBP4**	0.14	6.90E-01	1.00E+00	-0.31	5.71E-01	9.30E-01
**AL356273.3**	0.59	1.84E-01	1.00E+00	0.48	5.71E-01	9.30E-01
**AC005736.1**	0.66	3.17E-01	1.00E+00	-0.40	5.71E-01	9.30E-01
**RNF10**	-0.04	7.65E-01	1.00E+00	-0.19	5.71E-01	9.30E-01
**AC124242.1**	-0.53	4.07E-01	1.00E+00	-0.34	5.71E-01	9.30E-01
**FUCA2**	0.01	9.78E-01	1.00E+00	0.20	5.71E-01	9.30E-01
**EID2B**	0.12	8.34E-01	1.00E+00	0.29	5.72E-01	9.30E-01
**ZNF366**	0.18	7.49E-01	1.00E+00	-0.36	5.72E-01	9.30E-01
**ADM5**	-0.77	1.95E-02	8.75E-01	0.30	5.72E-01	9.30E-01
**NR5A2**	0.46	3.20E-01	1.00E+00	0.29	5.72E-01	9.30E-01
**SAXO2**	0.32	5.67E-01	1.00E+00	0.30	5.72E-01	9.30E-01
**OR7E91P**	0.57	2.37E-01	1.00E+00	-0.37	5.72E-01	9.30E-01
**LINC01388**	-2.44	2.72E-01	1.00E+00	-1.04	5.72E-01	9.30E-01
**ANGPT4**	0.40	4.87E-01	1.00E+00	-0.49	5.72E-01	9.30E-01
**AC012074.1**	-1.34	1.85E-01	1.00E+00	-0.71	5.72E-01	9.30E-01
**7-Mar**	-0.04	8.77E-01	1.00E+00	-0.12	5.72E-01	9.30E-01
**AL583785.1**	-0.71	4.23E-01	1.00E+00	0.32	5.72E-01	9.30E-01
**PTPN18**	-0.08	7.99E-01	1.00E+00	0.33	5.72E-01	9.30E-01
**MIR4442**	-0.63	3.57E-01	1.00E+00	0.94	5.72E-01	9.30E-01
**PPP2R5A**	0.30	2.18E-01	1.00E+00	-0.24	5.72E-01	9.30E-01
**RPP40**	-0.43	2.44E-01	1.00E+00	-0.20	5.72E-01	9.30E-01
**SIX2**	0.56	4.60E-01	1.00E+00	-0.66	5.72E-01	9.30E-01
**KLC1**	-0.13	6.37E-01	1.00E+00	0.19	5.72E-01	9.30E-01
**LINC01914**	-0.37	9.12E-01	1.00E+00	0.50	5.72E-01	9.31E-01
**BACE2**	-0.05	8.40E-01	1.00E+00	0.28	5.72E-01	9.31E-01
**LINC01290**	0.50	7.36E-01	1.00E+00	0.52	5.72E-01	9.31E-01
**CDCA7**	-0.37	2.72E-01	1.00E+00	-0.22	5.73E-01	9.31E-01

**AC125494.2**	-0.80	3.79E-01	1.00E+00	-0.65	5.73E-01	9.31E-01
**NUMA1**	-0.11	6.49E-01	1.00E+00	0.31	5.73E-01	9.31E-01
**SELENOH**	-0.16	4.85E-01	1.00E+00	0.23	5.73E-01	9.31E-01
**GNG5**	-0.33	4.45E-01	1.00E+00	0.39	5.73E-01	9.31E-01
**FAM199X**	0.19	4.05E-01	1.00E+00	-0.18	5.73E-01	9.31E-01
**VTI1B**	0.39	7.48E-02	1.00E+00	-0.18	5.73E-01	9.31E-01
**AC012368.1**	0.07	8.95E-01	1.00E+00	0.44	5.73E-01	9.31E-01
**HYLS1**	0.00	9.95E-01	1.00E+00	0.14	5.73E-01	9.31E-01
**AC016027.1**	0.26	8.16E-01	1.00E+00	-0.28	5.73E-01	9.31E-01
**G40865**	-0.53	5.03E-01	1.00E+00	0.31	5.73E-01	9.31E-01
**SPDYE3**	-0.45	1.61E-01	1.00E+00	-0.20	5.73E-01	9.31E-01
**GADD45A**	-0.02	9.57E-01	1.00E+00	-0.26	5.73E-01	9.31E-01
**HAUS4**	0.19	4.86E-01	1.00E+00	0.20	5.73E-01	9.31E-01
**IRF1**	-1.01	8.77E-04	1.50E-01	0.33	5.73E-01	9.31E-01
**MAVS**	0.08	7.94E-01	1.00E+00	0.21	5.73E-01	9.31E-01
**MYO18B**	-0.71	5.17E-01	1.00E+00	-0.68	5.73E-01	9.31E-01
**GNAI2**	0.02	9.42E-01	1.00E+00	0.33	5.73E-01	9.31E-01
**MIPEPP3**	-1.07	2.30E-02	9.12E-01	0.16	5.73E-01	9.31E-01
**CA14**	0.20	6.70E-01	1.00E+00	0.28	5.73E-01	9.31E-01
**ASCC1**	0.00	9.82E-01	1.00E+00	-0.12	5.73E-01	9.31E-01
**AL139011.1**	-2.90	7.77E-02	1.00E+00	-0.38	5.73E-01	9.31E-01
**HTR1F**	0.23	7.62E-01	1.00E+00	0.52	5.74E-01	9.31E-01
**AC004908.1**	1.32	3.73E-01	1.00E+00	-0.67	5.74E-01	9.31E-01
**PLP1**	-0.87	5.99E-02	1.00E+00	-0.25	5.74E-01	9.31E-01
**ART4**	0.27	6.93E-01	1.00E+00	0.47	5.74E-01	9.31E-01
**TIGD4**	-0.05	9.63E-01	1.00E+00	0.55	5.74E-01	9.31E-01
**PEX16**	-0.26	2.87E-01	1.00E+00	0.11	5.74E-01	9.31E-01
**TRIP13**	-0.06	8.74E-01	1.00E+00	0.24	5.74E-01	9.31E-01
**QSER1**	0.00	9.98E-01	1.00E+00	0.22	5.74E-01	9.31E-01
**ARL8B**	0.13	5.45E-01	1.00E+00	-0.10	5.74E-01	9.31E-01
**AC022364.1**	0.00	9.96E-01	1.00E+00	-0.27	5.74E-01	9.31E-01
**NPPA**	0.90	7.96E-01	1.00E+00	1.08	5.74E-01	9.31E-01

**TIMM29**	0.03	8.92E-01	1.00E+00	-0.20	5.74E-01	9.31E-01
**IL17D**	0.64	2.12E-01	1.00E+00	-0.29	5.74E-01	9.31E-01
**AC008894.3**	0.67	6.43E-01	1.00E+00	0.69	5.74E-01	9.31E-01
**AL158212.3**	0.54	3.51E-01	1.00E+00	-0.38	5.74E-01	9.31E-01
**AC009090.4**	-1.20	6.11E-01	1.00E+00	-0.60	5.74E-01	9.31E-01
**SCEL**	0.08	8.95E-01	1.00E+00	-0.39	5.74E-01	9.31E-01
**AC104237.3**	-0.81	4.72E-01	1.00E+00	-0.57	5.74E-01	9.31E-01
**HDAC4**	0.34	2.89E-01	1.00E+00	0.27	5.74E-01	9.31E-01
**ETAA1**	-0.32	1.49E-01	1.00E+00	-0.17	5.74E-01	9.31E-01
**BOLA2B**	-0.50	4.00E-01	1.00E+00	0.16	5.74E-01	9.31E-01
**AP000866.6**	0.63	8.26E-01	1.00E+00	0.53	5.74E-01	9.31E-01
**GRB7**	0.25	3.43E-01	1.00E+00	0.24	5.75E-01	9.31E-01
**C16orf86**	0.88	4.03E-02	1.00E+00	0.37	5.75E-01	9.31E-01
**METTL3**	0.30	2.30E-01	1.00E+00	-0.14	5.75E-01	9.31E-01
**MINPP1**	0.33	3.15E-01	1.00E+00	-0.13	5.75E-01	9.31E-01
**CD63**	0.25	4.99E-01	1.00E+00	0.18	5.75E-01	9.31E-01
**NR6A1**	-0.51	2.40E-01	1.00E+00	-0.27	5.75E-01	9.31E-01
**HOXB6**	0.02	9.79E-01	1.00E+00	0.29	5.75E-01	9.31E-01
**ZNF252P**	0.29	3.19E-01	1.00E+00	0.11	5.75E-01	9.31E-01
**G18962**	-2.99	2.15E-01	1.00E+00	0.91	5.75E-01	9.31E-01
**LNCTAM34A**	-0.38	4.05E-01	1.00E+00	0.30	5.75E-01	9.31E-01
**CATSPER3**	0.42	6.82E-01	1.00E+00	0.65	5.75E-01	9.31E-01
**AC011405.1**	-1.24	3.54E-01	1.00E+00	-0.55	5.75E-01	9.31E-01
**AC097639.1**	-0.26	5.58E-01	1.00E+00	0.26	5.75E-01	9.31E-01
**AC100812.1**	1.04	7.65E-01	1.00E+00	-0.86	5.75E-01	9.31E-01
**AC048344.4**	-1.99	5.63E-01	1.00E+00	0.75	5.75E-01	9.31E-01
**LRRC47**	-0.11	6.08E-01	1.00E+00	-0.20	5.75E-01	9.31E-01
**C19orf57**	-0.34	3.04E-01	1.00E+00	-0.30	5.75E-01	9.31E-01
**PFDN1**	-0.07	7.25E-01	1.00E+00	-0.14	5.75E-01	9.31E-01
**AP003119.3**	0.88	1.74E-02	8.33E-01	0.37	5.75E-01	9.31E-01
**AC007342.8**	-0.19	9.25E-01	1.00E+00	-0.83	5.75E-01	9.31E-01
**PLLP**	0.07	8.62E-01	1.00E+00	-0.27	5.75E-01	9.31E-01

**MAPK15**	0.40	3.32E-01	1.00E+00	0.44	5.75E-01	9.31E-01
**SPDYE6**	-0.49	6.34E-01	1.00E+00	0.37	5.75E-01	9.31E-01
**TMEM253**	4.23	5.86E-02	1.00E+00	-0.49	5.75E-01	9.31E-01
**AL035071.1**	0.22	5.96E-01	1.00E+00	-0.32	5.75E-01	9.31E-01
**MFSD4B**	-0.20	4.66E-01	1.00E+00	0.21	5.75E-01	9.31E-01
**GPHA2**	-2.35	4.34E-01	1.00E+00	-0.89	5.75E-01	9.31E-01
**CFAP298**	0.00	9.92E-01	1.00E+00	-0.28	5.75E-01	9.31E-01
**TRARG1**	4.97	2.67E-03	2.68E-01	0.56	5.75E-01	9.31E-01
**C15orf65**	-0.01	9.82E-01	1.00E+00	-0.23	5.76E-01	9.31E-01
**COPG2**	-0.44	1.21E-01	1.00E+00	0.21	5.76E-01	9.31E-01
**ANGEL1**	0.14	7.21E-01	1.00E+00	0.25	5.76E-01	9.31E-01
**YPEL1**	-0.52	2.72E-01	1.00E+00	-0.48	5.76E-01	9.31E-01
**XLOC_007992**	-0.34	9.03E-01	1.00E+00	-0.33	5.76E-01	9.31E-01
**C12orf57**	0.07	7.80E-01	1.00E+00	-0.25	5.76E-01	9.31E-01
**LINC02533**	NA	NA	NA	-1.80	5.76E-01	9.31E-01
**EMC2**	0.24	2.18E-01	1.00E+00	-0.12	5.76E-01	9.31E-01
**AP003068.4**	-0.57	5.23E-01	1.00E+00	-0.42	5.76E-01	9.31E-01
**ANXA9**	-0.16	7.68E-01	1.00E+00	0.32	5.76E-01	9.31E-01
**AC012557.1**	1.84	8.58E-02	1.00E+00	0.42	5.76E-01	9.31E-01
**INTS4P1**	1.26	1.20E-01	1.00E+00	-0.47	5.76E-01	9.31E-01
**AC004982.1**	1.04	3.60E-01	1.00E+00	0.49	5.76E-01	9.31E-01
**CD177**	-2.23	8.49E-02	1.00E+00	1.76	5.76E-01	9.31E-01
**OTUB1**	-0.24	2.93E-01	1.00E+00	0.12	5.76E-01	9.31E-01
**TMEM150B**	0.43	7.37E-01	1.00E+00	1.77	5.76E-01	9.31E-01
**MT-TP**	0.05	9.30E-01	1.00E+00	0.22	5.76E-01	9.32E-01
**ZFAND2A**	0.04	8.95E-01	1.00E+00	0.17	5.76E-01	9.32E-01
**CAVIN4**	-0.10	9.33E-01	1.00E+00	-0.67	5.76E-01	9.32E-01
**ZNF341-AS1**	1.63	2.10E-01	1.00E+00	0.39	5.76E-01	9.32E-01
**AC048341.2**	-0.36	9.16E-01	1.00E+00	-0.77	5.77E-01	9.32E-01
**AC027307.3**	-1.19	2.10E-03	2.39E-01	0.31	5.77E-01	9.32E-01
**MATN3**	0.12	8.50E-01	1.00E+00	-1.24	5.77E-01	9.32E-01
**RN7SL68P**	-1.32	6.20E-01	1.00E+00	0.70	5.77E-01	9.32E-01

**RAPGEF5**	-0.04	8.95E-01	1.00E+00	0.25	5.77E-01	9.32E-01
**GFAP**	-2.36	1.57E-01	1.00E+00	-0.54	5.77E-01	9.32E-01
**C1orf140**	-0.01	9.93E-01	1.00E+00	-0.57	5.77E-01	9.32E-01
**XLOC_008957**	-2.61	1.63E-01	1.00E+00	-0.65	5.77E-01	9.32E-01
**AC004834.1**	-1.80	2.78E-01	1.00E+00	0.59	5.77E-01	9.32E-01
**AANAT**	-0.13	9.26E-01	1.00E+00	0.80	5.77E-01	9.32E-01
**KCTD16**	0.62	7.02E-01	1.00E+00	1.42	5.77E-01	9.32E-01
**NRG4**	-0.74	1.02E-01	1.00E+00	-0.43	5.77E-01	9.32E-01
**STK24**	0.00	9.88E-01	1.00E+00	-0.29	5.77E-01	9.32E-01
**PGM5**	0.18	7.27E-01	1.00E+00	-0.37	5.77E-01	9.32E-01
**LCE4A**	0.20	7.90E-01	1.00E+00	0.38	5.77E-01	9.32E-01
**AC008065.1**	NA	NA	NA	0.52	5.77E-01	9.32E-01
**SLC45A2**	-0.10	8.99E-01	1.00E+00	0.47	5.77E-01	9.32E-01
**MYL12BP1**	-2.80	5.63E-02	1.00E+00	-0.30	5.78E-01	9.32E-01
**AC002044.1**	-1.71	6.18E-01	1.00E+00	0.43	5.78E-01	9.32E-01
**CA12**	-0.17	5.57E-01	1.00E+00	0.21	5.78E-01	9.33E-01
**MRTFB**	-0.10	6.61E-01	1.00E+00	0.18	5.78E-01	9.33E-01
**TNFRSF4**	-0.17	7.99E-01	1.00E+00	0.55	5.78E-01	9.33E-01
**LGALS1**	0.38	4.18E-01	1.00E+00	0.23	5.78E-01	9.33E-01
**AC108058.1**	-2.69	1.44E-01	1.00E+00	0.60	5.78E-01	9.33E-01
**FBXO6**	-0.13	7.37E-01	1.00E+00	0.17	5.78E-01	9.33E-01
**STBD1**	0.45	4.58E-01	1.00E+00	-0.38	5.78E-01	9.33E-01
**SLC35B2**	-0.15	5.42E-01	1.00E+00	0.13	5.78E-01	9.33E-01
**NATD1**	0.33	3.52E-01	1.00E+00	0.36	5.78E-01	9.33E-01
**AC091182.1**	-1.01	1.64E-01	1.00E+00	1.18	5.78E-01	9.33E-01
**NECTIN3**	0.13	6.70E-01	1.00E+00	0.33	5.79E-01	9.33E-01
**HIST1H3A**	1.31	4.37E-01	1.00E+00	0.57	5.79E-01	9.33E-01
**ARHGAP29**	0.41	2.19E-01	1.00E+00	-0.18	5.79E-01	9.33E-01
**MRPL38**	-0.15	8.64E-01	1.00E+00	0.37	5.79E-01	9.33E-01
**PSG7**	0.18	8.72E-01	1.00E+00	-0.65	5.79E-01	9.33E-01
**RPL23AP87**	1.20	2.39E-01	1.00E+00	-0.38	5.79E-01	9.33E-01
**TXNL1**	0.11	4.88E-01	1.00E+00	-0.13	5.79E-01	9.33E-01

**G28648**	0.52	3.85E-01	1.00E+00	-0.33	5.79E-01	9.33E-01
**TTLL5**	-0.23	2.73E-01	1.00E+00	-0.19	5.79E-01	9.33E-01
**PPP1R10**	-0.22	4.32E-01	1.00E+00	-0.16	5.79E-01	9.33E-01
**GPR137B**	0.22	4.17E-01	1.00E+00	-0.27	5.79E-01	9.33E-01
**TDGP1**	-0.70	8.41E-01	1.00E+00	-0.40	5.79E-01	9.33E-01
**PRKN**	-0.09	7.56E-01	1.00E+00	-0.25	5.79E-01	9.33E-01
**CNOT3**	-0.26	3.15E-01	1.00E+00	0.33	5.79E-01	9.33E-01
**AC007228.1**	1.92	3.73E-01	1.00E+00	0.33	5.79E-01	9.34E-01
**ABHD11**	-0.03	8.94E-01	1.00E+00	-0.15	5.79E-01	9.34E-01
**RBMS2P1**	-1.07	2.46E-01	1.00E+00	-0.27	5.79E-01	9.34E-01
**LLGL2**	-0.31	3.03E-01	1.00E+00	-0.29	5.80E-01	9.34E-01
**HMGB1P20**	NA	NA	NA	-0.36	5.80E-01	9.34E-01
**GNA11**	-0.03	9.00E-01	1.00E+00	0.29	5.80E-01	9.34E-01
**SYNGR2**	-0.25	3.32E-01	1.00E+00	0.13	5.80E-01	9.34E-01
**SPATA22**	-1.20	7.05E-01	1.00E+00	-0.50	5.80E-01	9.34E-01
**ACBD3-AS1**	1.21	6.41E-02	1.00E+00	-0.44	5.80E-01	9.34E-01
**UBR5**	0.05	8.21E-01	1.00E+00	0.25	5.80E-01	9.34E-01
**XLOC_004363**	-0.81	6.17E-01	1.00E+00	-0.55	5.80E-01	9.34E-01
**FLNB**	-0.23	4.00E-01	1.00E+00	0.26	5.80E-01	9.34E-01
**KIAA1328**	-0.29	1.96E-01	1.00E+00	-0.18	5.80E-01	9.34E-01
**SMPDL3A**	0.36	3.01E-01	1.00E+00	-0.26	5.80E-01	9.34E-01
**SEMA3G**	0.89	1.51E-01	1.00E+00	0.42	5.80E-01	9.34E-01
**ASTL**	-0.68	2.95E-01	1.00E+00	-0.60	5.80E-01	9.34E-01
**FBXO34**	-0.04	8.47E-01	1.00E+00	-0.10	5.80E-01	9.34E-01
**RLF**	-0.04	8.55E-01	1.00E+00	-0.17	5.80E-01	9.34E-01
**ZMYM5**	0.16	4.94E-01	1.00E+00	-0.17	5.80E-01	9.34E-01
**AC083801.2**	-0.77	5.55E-01	1.00E+00	0.67	5.80E-01	9.34E-01
**PPP5C**	-0.11	5.69E-01	1.00E+00	0.18	5.80E-01	9.34E-01
**SLIT1**	0.51	5.93E-01	1.00E+00	0.75	5.80E-01	9.34E-01
**LGALS7B**	-0.82	9.42E-02	1.00E+00	-0.37	5.81E-01	9.34E-01
**AC025165.5**	0.58	3.94E-01	1.00E+00	0.25	5.81E-01	9.34E-01
**TRAV22**	2.93	1.01E-01	1.00E+00	0.81	5.81E-01	9.34E-01

**LINGO4**	-1.29	6.53E-02	1.00E+00	-0.52	5.81E-01	9.34E-01
**SNHG11**	0.06	8.73E-01	1.00E+00	0.12	5.81E-01	9.34E-01
**AL138720.1**	0.12	9.18E-01	1.00E+00	0.57	5.81E-01	9.34E-01
**NAT9**	-0.10	7.40E-01	1.00E+00	0.15	5.81E-01	9.34E-01
**XLOC_001333**	-0.19	6.26E-01	1.00E+00	-0.44	5.81E-01	9.34E-01
**G31549**	0.18	8.74E-01	1.00E+00	0.47	5.81E-01	9.34E-01
**FBXO32**	0.29	5.04E-01	1.00E+00	-0.15	5.81E-01	9.34E-01
**PPIL2**	-0.07	7.65E-01	1.00E+00	0.23	5.81E-01	9.34E-01
**RPL7P16**	-2.93	2.36E-01	1.00E+00	0.51	5.81E-01	9.34E-01
**TBK1**	0.17	5.53E-01	1.00E+00	0.09	5.81E-01	9.34E-01
**NAGLU**	0.19	6.15E-01	1.00E+00	0.36	5.81E-01	9.34E-01
**FAM111A-DT**	0.46	9.49E-02	1.00E+00	0.14	5.81E-01	9.34E-01
**SUV39H2**	-0.02	9.64E-01	1.00E+00	0.16	5.81E-01	9.34E-01
**EIF3L**	-0.19	5.94E-01	1.00E+00	0.18	5.82E-01	9.35E-01
**SLC39A6**	-0.14	6.57E-01	1.00E+00	0.12	5.82E-01	9.35E-01
**AC097374.1**	1.09	7.54E-01	1.00E+00	-0.43	5.82E-01	9.35E-01
**BCORL1**	0.12	7.27E-01	1.00E+00	0.36	5.82E-01	9.35E-01
**PSORS1C1**	0.04	9.29E-01	1.00E+00	-0.23	5.82E-01	9.35E-01
**AC008105.1**	-0.57	7.43E-01	1.00E+00	-0.72	5.82E-01	9.35E-01
**TLK2P1**	-0.32	7.46E-01	1.00E+00	-0.25	5.82E-01	9.35E-01
**RNF185**	0.09	6.47E-01	1.00E+00	0.10	5.82E-01	9.35E-01
**DKC1**	-0.06	7.81E-01	1.00E+00	0.13	5.82E-01	9.35E-01
**BEND3P1**	0.75	3.46E-01	1.00E+00	0.60	5.82E-01	9.35E-01
**FAM72A**	-0.32	6.10E-01	1.00E+00	-0.16	5.82E-01	9.35E-01
**YTHDF2**	-0.06	7.61E-01	1.00E+00	-0.11	5.82E-01	9.35E-01
**AP3S1**	0.16	3.72E-01	1.00E+00	-0.13	5.82E-01	9.35E-01
**KRTAP5-10**	-1.05	6.68E-01	1.00E+00	0.91	5.82E-01	9.35E-01
**PITX3**	-1.24	7.13E-01	1.00E+00	1.06	5.82E-01	9.35E-01
**PCDHGC3**	-0.24	3.36E-01	1.00E+00	0.33	5.82E-01	9.35E-01
**AP003469.4**	-0.07	8.82E-01	1.00E+00	0.32	5.82E-01	9.35E-01
**AC005288.1**	-0.09	7.59E-01	1.00E+00	0.19	5.82E-01	9.35E-01
**CDYL**	-0.10	6.56E-01	1.00E+00	0.13	5.82E-01	9.35E-01

**AC060766.7**	-2.25	2.20E-01	1.00E+00	0.39	5.82E-01	9.35E-01
**AL590491.2**	-0.62	5.65E-01	1.00E+00	-0.55	5.82E-01	9.35E-01
**AL355297.4**	-3.10	3.60E-01	1.00E+00	-0.79	5.82E-01	9.35E-01
**SPIRE1**	-0.05	8.84E-01	1.00E+00	0.27	5.82E-01	9.35E-01
**GPRC5B**	0.02	9.78E-01	1.00E+00	-0.24	5.82E-01	9.35E-01
**AC006449.2**	-0.11	8.55E-01	1.00E+00	0.33	5.82E-01	9.35E-01
**LAMP3**	-0.07	9.30E-01	1.00E+00	0.43	5.83E-01	9.35E-01
**GALNT15**	1.55	3.09E-02	1.00E+00	0.37	5.83E-01	9.35E-01
**IGLV5-52**	0.38	5.44E-01	1.00E+00	0.35	5.83E-01	9.35E-01
**HOXD10**	0.16	7.06E-01	1.00E+00	0.51	5.83E-01	9.35E-01
**DCBLD2**	0.12	5.88E-01	1.00E+00	-0.14	5.83E-01	9.35E-01
**DZIP1L**	-0.27	4.85E-01	1.00E+00	0.29	5.83E-01	9.35E-01
**G32319**	-0.37	6.05E-01	1.00E+00	0.37	5.83E-01	9.35E-01
**PTP4A2P2**	0.92	7.40E-01	1.00E+00	0.54	5.83E-01	9.35E-01
**LRRIQ3**	0.78	3.70E-01	1.00E+00	-0.30	5.83E-01	9.35E-01
**TCEA2**	-0.04	8.88E-01	1.00E+00	-0.32	5.83E-01	9.35E-01
**AL158166.1**	-2.67	1.48E-01	1.00E+00	0.95	5.83E-01	9.35E-01
**CHRM3**	0.08	9.23E-01	1.00E+00	-1.26	5.83E-01	9.35E-01
**MED28**	0.35	8.82E-02	1.00E+00	0.12	5.83E-01	9.35E-01
**AC092574.2**	-2.63	3.21E-01	1.00E+00	-0.40	5.83E-01	9.35E-01
**ZDHHC23**	-0.20	6.98E-01	1.00E+00	0.35	5.83E-01	9.35E-01
**REEP4**	-0.05	9.06E-01	1.00E+00	0.19	5.83E-01	9.35E-01
**PRR29**	0.50	3.59E-01	1.00E+00	0.47	5.83E-01	9.35E-01
**GSTT1**	0.48	6.93E-01	1.00E+00	1.71	5.83E-01	9.35E-01
**SLC25A15**	-0.30	3.41E-01	1.00E+00	-0.31	5.84E-01	9.35E-01
**ABCC3**	-0.38	3.00E-01	1.00E+00	-0.21	5.84E-01	9.35E-01
**NRG1**	-0.26	4.83E-01	1.00E+00	0.28	5.84E-01	9.35E-01
**RNA5-8SP4**	NA	NA	NA	-0.75	5.84E-01	9.35E-01
**NNAT**	3.10	1.50E-02	7.62E-01	-0.36	5.84E-01	9.35E-01
**RECQL4**	-0.37	2.24E-01	1.00E+00	0.35	5.84E-01	9.35E-01
**AL353795.3**	0.56	8.71E-01	1.00E+00	0.69	5.84E-01	9.35E-01
**CYCSP55**	-1.03	6.94E-01	1.00E+00	-0.33	5.84E-01	9.35E-01

**POLK**	0.22	5.38E-01	1.00E+00	-0.14	5.84E-01	9.35E-01
**IGKV1D-16**	1.94	4.96E-01	1.00E+00	1.75	5.84E-01	9.35E-01
**SLC15A5**	0.56	8.71E-01	1.00E+00	1.26	5.84E-01	9.35E-01
**RNASE2**	-1.75	4.42E-01	1.00E+00	1.73	5.84E-01	9.35E-01
**GRWD1**	-0.04	8.87E-01	1.00E+00	0.11	5.84E-01	9.35E-01
**XLOC_001406**	-3.40	4.18E-02	1.00E+00	0.65	5.84E-01	9.35E-01
**AC113383.1**	-0.38	4.72E-01	1.00E+00	-0.33	5.84E-01	9.35E-01
**TBC1D24**	0.39	3.27E-01	1.00E+00	-0.17	5.84E-01	9.35E-01
**EML3**	0.18	3.10E-01	1.00E+00	0.17	5.84E-01	9.35E-01
**ZBTB4**	-0.17	3.18E-01	1.00E+00	0.26	5.84E-01	9.35E-01
**AK2**	0.00	9.88E-01	1.00E+00	-0.08	5.84E-01	9.35E-01
**PRH1**	1.30	2.62E-01	1.00E+00	0.29	5.84E-01	9.35E-01
**ZNF460-AS1**	1.23	1.83E-01	1.00E+00	-0.39	5.84E-01	9.35E-01
**VPS72**	0.05	7.34E-01	1.00E+00	-0.09	5.84E-01	9.35E-01
**EIF2S3**	0.13	3.66E-01	1.00E+00	0.13	5.84E-01	9.35E-01
**LINC00310**	-0.30	7.40E-01	1.00E+00	0.45	5.84E-01	9.35E-01
**KRT26**	-6.44	5.02E-02	1.00E+00	-1.75	5.84E-01	9.35E-01
**SF3B3**	0.02	9.28E-01	1.00E+00	0.10	5.85E-01	9.35E-01
**Z98049.1**	1.29	7.10E-01	1.00E+00	-0.86	5.85E-01	9.35E-01
**PTPN7**	-0.13	8.22E-01	1.00E+00	1.45	5.85E-01	9.35E-01
**PRF1**	0.14	8.24E-01	1.00E+00	1.66	5.85E-01	9.36E-01
**ASXL3**	1.05	1.49E-01	1.00E+00	0.54	5.85E-01	9.36E-01
**ZNF467**	-0.87	2.02E-02	8.85E-01	-0.26	5.85E-01	9.36E-01
**HMGN2P8**	NA	NA	NA	-0.45	5.85E-01	9.36E-01
**G36510**	-0.88	2.09E-01	1.00E+00	-0.53	5.85E-01	9.36E-01
**ZFPM2**	-0.17	6.78E-01	1.00E+00	-0.28	5.85E-01	9.36E-01
**NWD1**	0.23	8.00E-01	1.00E+00	-0.60	5.85E-01	9.36E-01
**AC134349.1**	NA	NA	NA	0.72	5.86E-01	9.36E-01
**RBMS3-AS2**	0.39	8.42E-01	1.00E+00	0.41	5.86E-01	9.36E-01
**CYP4B1**	-0.08	8.88E-01	1.00E+00	-0.27	5.86E-01	9.36E-01
**IQGAP3**	0.15	6.38E-01	1.00E+00	0.35	5.86E-01	9.36E-01
**FOXE1**	-1.50	5.02E-03	4.15E-01	0.59	5.86E-01	9.36E-01

**ZNF202**	-0.25	3.30E-01	1.00E+00	-0.22	5.86E-01	9.36E-01
**AC009554.2**	1.61	2.79E-01	1.00E+00	-0.57	5.86E-01	9.36E-01
**BTNL8**	1.76	3.88E-01	1.00E+00	0.74	5.86E-01	9.36E-01
**SUCLG2P2**	1.51	3.88E-01	1.00E+00	-0.24	5.86E-01	9.36E-01
**XXYLT1**	-0.28	2.90E-01	1.00E+00	0.25	5.86E-01	9.36E-01
**IL23R**	-1.73	6.13E-01	1.00E+00	0.86	5.86E-01	9.36E-01
**TAC1**	-0.70	4.15E-01	1.00E+00	-0.42	5.86E-01	9.36E-01
**PIK3CA**	0.34	2.08E-01	1.00E+00	-0.15	5.86E-01	9.36E-01
**AC134312.1**	0.53	3.10E-01	1.00E+00	0.52	5.86E-01	9.36E-01
**SPRR2C**	3.53	2.96E-01	1.00E+00	1.74	5.86E-01	9.36E-01
**AC138470.1**	1.43	2.37E-01	1.00E+00	0.60	5.86E-01	9.36E-01
**CARD16**	-0.03	9.47E-01	1.00E+00	0.16	5.86E-01	9.36E-01
**ZC3H12C**	-0.35	3.97E-01	1.00E+00	0.23	5.86E-01	9.36E-01
**FAM234B**	0.34	3.05E-01	1.00E+00	-0.22	5.86E-01	9.36E-01
**CEPT1**	0.20	4.73E-01	1.00E+00	0.20	5.86E-01	9.36E-01
**PCOLCE2**	1.87	2.35E-03	2.45E-01	-0.36	5.86E-01	9.36E-01
**LINC00092**	-0.16	8.31E-01	1.00E+00	-0.66	5.86E-01	9.36E-01
**PCM1**	-0.08	5.82E-01	1.00E+00	-0.09	5.86E-01	9.36E-01
**DMC1**	-0.39	4.11E-01	1.00E+00	0.28	5.86E-01	9.36E-01
**WDR35**	0.00	9.92E-01	1.00E+00	0.17	5.86E-01	9.36E-01
**P4HA1**	0.18	5.30E-01	1.00E+00	0.13	5.86E-01	9.36E-01
**AC226118.1**	-0.84	4.69E-01	1.00E+00	0.70	5.86E-01	9.36E-01
**AC021087.2**	-1.46	2.25E-01	1.00E+00	-0.49	5.86E-01	9.36E-01
**ENPP3**	0.90	8.74E-02	1.00E+00	0.42	5.86E-01	9.36E-01
**GNL1**	0.02	8.56E-01	1.00E+00	0.22	5.87E-01	9.36E-01
**AC010503.4**	0.23	5.91E-01	1.00E+00	-0.28	5.87E-01	9.36E-01
**ZNF488**	-0.28	5.66E-01	1.00E+00	0.44	5.87E-01	9.36E-01
**RAB35**	-0.15	4.44E-01	1.00E+00	0.25	5.87E-01	9.36E-01
**RP11-61J19.4**	0.22	6.01E-01	1.00E+00	-0.33	5.87E-01	9.36E-01
**BCR**	-0.36	8.97E-02	1.00E+00	0.28	5.87E-01	9.36E-01
**PDE11A**	1.75	2.78E-01	1.00E+00	0.49	5.87E-01	9.36E-01
**HDDC3**	0.18	4.74E-01	1.00E+00	-0.21	5.87E-01	9.36E-01

**LINC00989**	-0.94	7.99E-02	1.00E+00	-0.40	5.87E-01	9.36E-01
**ZNF329**	-0.08	7.06E-01	1.00E+00	0.26	5.87E-01	9.36E-01
**AC009309.1**	-1.82	1.69E-01	1.00E+00	-0.32	5.87E-01	9.36E-01
**OTUD5**	-0.11	4.83E-01	1.00E+00	0.22	5.87E-01	9.36E-01
**SOCS1**	-1.53	1.13E-03	1.76E-01	-0.44	5.87E-01	9.36E-01
**NKAIN1**	-0.26	7.39E-01	1.00E+00	0.25	5.87E-01	9.36E-01
**TIFA**	0.40	2.01E-01	1.00E+00	-0.18	5.87E-01	9.36E-01
**KALRN**	-0.20	6.90E-01	1.00E+00	0.25	5.87E-01	9.36E-01
**SLC6A17**	-0.32	5.16E-01	1.00E+00	0.48	5.87E-01	9.36E-01
**STAMBPL1**	-0.24	5.28E-01	1.00E+00	0.18	5.87E-01	9.36E-01
**RPS7P11**	-0.35	4.44E-01	1.00E+00	-0.25	5.87E-01	9.36E-01
**MRPS25**	-0.01	9.58E-01	1.00E+00	-0.14	5.87E-01	9.36E-01
**DYRK4**	0.16	4.96E-01	1.00E+00	0.13	5.87E-01	9.36E-01
**HELZ**	0.18	4.71E-01	1.00E+00	0.09	5.87E-01	9.36E-01
**PCDHB10**	0.14	6.92E-01	1.00E+00	0.35	5.87E-01	9.36E-01
**AC126614.1**	-1.85	3.29E-01	1.00E+00	0.74	5.87E-01	9.36E-01
**SAE1**	0.03	8.48E-01	1.00E+00	0.07	5.87E-01	9.36E-01
**ARHGAP40**	0.38	5.34E-01	1.00E+00	0.31	5.87E-01	9.36E-01
**CASP2**	-0.01	9.64E-01	1.00E+00	0.14	5.87E-01	9.36E-01
**IL6ST**	0.41	2.63E-01	1.00E+00	0.19	5.87E-01	9.36E-01
**MIGA2**	-0.01	9.73E-01	1.00E+00	0.31	5.87E-01	9.36E-01
**AC046168.2**	-0.91	1.65E-01	1.00E+00	0.40	5.87E-01	9.36E-01
**TKTL1**	-1.97	2.29E-01	1.00E+00	-0.56	5.88E-01	9.36E-01
**TUBAL3**	-0.18	8.26E-01	1.00E+00	-0.53	5.88E-01	9.36E-01
**ARHGDIB**	0.00	9.93E-01	1.00E+00	0.16	5.88E-01	9.36E-01
**FN3KRP**	0.55	9.53E-03	6.06E-01	-0.10	5.88E-01	9.36E-01
**SLC9B1**	0.46	4.72E-01	1.00E+00	0.30	5.88E-01	9.36E-01
**LAMA1**	-0.38	5.42E-01	1.00E+00	1.51	5.88E-01	9.36E-01
**RPSAP18**	0.97	4.11E-01	1.00E+00	0.50	5.88E-01	9.36E-01
**ST7-AS1**	-0.75	6.35E-02	1.00E+00	-0.44	5.88E-01	9.36E-01
**RTCA**	0.28	2.53E-01	1.00E+00	0.11	5.88E-01	9.36E-01
**G32932**	0.22	9.17E-01	1.00E+00	0.70	5.88E-01	9.36E-01

**CDCA3**	0.24	5.43E-01	1.00E+00	0.31	5.88E-01	9.36E-01
**STAR**	-0.15	6.60E-01	1.00E+00	-0.33	5.88E-01	9.36E-01
**THAP4**	-0.23	2.32E-01	1.00E+00	0.11	5.88E-01	9.36E-01
**LEXM**	0.35	6.59E-01	1.00E+00	0.43	5.88E-01	9.36E-01
**CHCHD6**	-0.13	4.63E-01	1.00E+00	-0.11	5.88E-01	9.36E-01
**PISD**	0.24	4.23E-01	1.00E+00	-0.16	5.88E-01	9.36E-01
**NET1**	-0.11	5.99E-01	1.00E+00	-0.12	5.88E-01	9.36E-01
**NME5**	0.17	7.99E-01	1.00E+00	-0.34	5.88E-01	9.36E-01
**ODF3L2**	-1.19	3.02E-01	1.00E+00	0.70	5.88E-01	9.36E-01
**KCNH8**	-1.70	1.59E-01	1.00E+00	-0.57	5.88E-01	9.36E-01
**PROS2P**	-2.28	2.65E-01	1.00E+00	0.48	5.88E-01	9.36E-01
**F12**	-0.02	9.62E-01	1.00E+00	0.32	5.88E-01	9.36E-01
**DDX52**	-0.06	7.74E-01	1.00E+00	-0.10	5.88E-01	9.36E-01
**AL356535.1**	1.06	7.61E-01	1.00E+00	0.47	5.88E-01	9.36E-01
**EEF1B2P3**	-0.04	9.29E-01	1.00E+00	-0.22	5.88E-01	9.36E-01
**SYCE2**	0.23	7.63E-01	1.00E+00	-0.34	5.88E-01	9.36E-01
**XLOC_001435**	0.25	7.69E-01	1.00E+00	0.49	5.88E-01	9.36E-01
**SPINK5**	0.15	7.33E-01	1.00E+00	0.39	5.88E-01	9.36E-01
**CASC1**	-1.25	7.62E-02	1.00E+00	-0.42	5.89E-01	9.36E-01
**ZNF691**	-0.12	6.83E-01	1.00E+00	-0.17	5.89E-01	9.36E-01
**AC244517.1**	0.53	8.13E-01	1.00E+00	0.57	5.89E-01	9.36E-01
**B3GNT10**	-0.01	9.68E-01	1.00E+00	-0.21	5.89E-01	9.36E-01
**ZNF606**	0.05	8.65E-01	1.00E+00	-0.15	5.89E-01	9.36E-01
**NEK3**	-0.23	5.41E-01	1.00E+00	-0.22	5.89E-01	9.36E-01
**XLOC_001373**	-3.17	7.84E-02	1.00E+00	0.62	5.89E-01	9.36E-01
**SNX18P7**	0.23	6.64E-01	1.00E+00	0.84	5.89E-01	9.36E-01
**BICD2**	0.09	8.11E-01	1.00E+00	-0.26	5.89E-01	9.36E-01
**PIK3R1**	0.61	1.02E-01	1.00E+00	0.21	5.89E-01	9.36E-01
**UAP1L1**	-0.31	2.74E-01	1.00E+00	0.37	5.89E-01	9.36E-01
**HOXC10**	-0.21	6.21E-01	1.00E+00	-0.20	5.89E-01	9.36E-01
**TAF6**	-0.07	7.43E-01	1.00E+00	0.14	5.89E-01	9.36E-01
**GPER1**	0.85	1.18E-01	1.00E+00	-0.31	5.89E-01	9.36E-01

**TCF12**	0.11	6.40E-01	1.00E+00	0.10	5.89E-01	9.36E-01
**ZC3H10**	0.17	6.52E-01	1.00E+00	-0.21	5.89E-01	9.36E-01
**G2927**	-3.12	7.10E-02	1.00E+00	0.76	5.89E-01	9.36E-01
**KRT25**	-5.06	1.23E-01	1.00E+00	-1.70	5.89E-01	9.36E-01
**DNAH5**	-0.21	8.27E-01	1.00E+00	0.51	5.89E-01	9.36E-01
**C7orf57**	2.80	1.89E-01	1.00E+00	0.77	5.89E-01	9.36E-01
**CCDC57**	-0.20	5.16E-01	1.00E+00	0.17	5.89E-01	9.36E-01
**YPEL5**	0.08	7.04E-01	1.00E+00	-0.15	5.89E-01	9.36E-01
**AC145285.6**	-3.11	8.05E-03	5.53E-01	0.40	5.90E-01	9.36E-01
**AC002525.1**	0.34	6.38E-01	1.00E+00	0.34	5.90E-01	9.36E-01
**PTP4A1**	-1.09	1.81E-01	1.00E+00	0.44	5.90E-01	9.36E-01
**SNHG16**	0.07	7.45E-01	1.00E+00	0.12	5.90E-01	9.36E-01
**ZNF16**	0.04	8.61E-01	1.00E+00	0.12	5.90E-01	9.36E-01
**ZNF891**	0.04	9.01E-01	1.00E+00	-0.24	5.90E-01	9.36E-01
**FAM114A2**	0.11	5.91E-01	1.00E+00	-0.12	5.90E-01	9.36E-01
**EPB41L5**	0.15	5.36E-01	1.00E+00	-0.14	5.90E-01	9.36E-01
**TSTA3**	-0.33	3.67E-01	1.00E+00	0.16	5.90E-01	9.36E-01
**DNAJC8**	-0.03	8.51E-01	1.00E+00	-0.13	5.90E-01	9.36E-01
**SLK**	-0.22	3.39E-01	1.00E+00	-0.18	5.90E-01	9.36E-01
**CC2D2B**	-0.17	9.01E-01	1.00E+00	0.40	5.90E-01	9.36E-01
**IGHV3-43**	2.13	3.93E-01	1.00E+00	1.72	5.90E-01	9.36E-01
**TDRD6**	-0.43	5.99E-01	1.00E+00	0.49	5.90E-01	9.36E-01
**AL022067.1**	-1.92	5.74E-01	1.00E+00	-0.74	5.90E-01	9.36E-01
**AC004552.1**	1.03	2.12E-01	1.00E+00	-0.34	5.90E-01	9.36E-01
**DDX51**	-0.24	3.18E-01	1.00E+00	-0.26	5.90E-01	9.36E-01
**UQCC3**	-0.33	4.40E-01	1.00E+00	-0.28	5.91E-01	9.36E-01
**NPAS3**	-0.08	8.83E-01	1.00E+00	0.33	5.91E-01	9.36E-01
**MPIG6B**	-0.07	9.02E-01	1.00E+00	-0.28	5.91E-01	9.36E-01
**NR3C2**	-0.14	7.14E-01	1.00E+00	-0.21	5.91E-01	9.36E-01
**NPTXR**	-0.13	8.17E-01	1.00E+00	0.35	5.91E-01	9.36E-01
**GALNT4**	0.53	4.62E-01	1.00E+00	0.29	5.91E-01	9.36E-01
**ZNF584**	-0.32	3.82E-01	1.00E+00	0.12	5.91E-01	9.36E-01

**RNMT**	0.17	3.74E-01	1.00E+00	0.11	5.91E-01	9.36E-01
**RHEBP2**	0.21	7.78E-01	1.00E+00	-0.22	5.91E-01	9.36E-01
**LRRC38**	1.00	3.40E-01	1.00E+00	-0.63	5.91E-01	9.36E-01
**ACSM6**	1.10	3.67E-01	1.00E+00	-1.69	5.91E-01	9.36E-01
**SRBD1**	-0.04	8.92E-01	1.00E+00	0.14	5.91E-01	9.36E-01
**TPI1P1**	-0.90	1.82E-01	1.00E+00	0.31	5.91E-01	9.36E-01
**ZNF782**	-0.10	8.06E-01	1.00E+00	-0.24	5.91E-01	9.36E-01
**LAS1L**	-0.21	2.24E-01	1.00E+00	0.08	5.91E-01	9.36E-01
**SLC25A46**	-0.05	8.67E-01	1.00E+00	-0.08	5.91E-01	9.36E-01
**MCM6**	-0.09	6.11E-01	1.00E+00	0.10	5.91E-01	9.36E-01
**DPCD**	0.08	7.90E-01	1.00E+00	0.14	5.91E-01	9.36E-01
**DDB1**	0.06	7.36E-01	1.00E+00	-0.14	5.91E-01	9.36E-01
**GSR**	0.28	2.26E-01	1.00E+00	-0.36	5.91E-01	9.36E-01
**ENPP1**	0.76	1.57E-01	1.00E+00	-0.28	5.91E-01	9.36E-01
**UBTF**	-0.25	2.49E-01	1.00E+00	0.18	5.91E-01	9.36E-01
**AC027290.2**	0.18	7.81E-01	1.00E+00	-0.24	5.91E-01	9.36E-01
**LIN37**	-1.94	1.34E-01	1.00E+00	0.35	5.91E-01	9.36E-01
**ANKRD42**	0.16	3.95E-01	1.00E+00	0.17	5.91E-01	9.36E-01
**AC092645.1**	0.56	1.40E-01	1.00E+00	0.43	5.91E-01	9.36E-01
**AC108215.1**	0.30	5.99E-01	1.00E+00	0.37	5.91E-01	9.36E-01
**RAD17**	0.34	8.51E-02	1.00E+00	-0.12	5.92E-01	9.36E-01
**N6AMT1**	0.51	3.01E-01	1.00E+00	0.16	5.92E-01	9.36E-01
**HCG9**	-0.54	8.41E-01	1.00E+00	0.55	5.92E-01	9.36E-01
**LINC00539**	-0.11	9.31E-01	1.00E+00	0.33	5.92E-01	9.36E-01
**SCAMP4**	-0.30	2.01E-01	1.00E+00	0.22	5.92E-01	9.36E-01
**AL022322.2**	-1.41	4.82E-01	1.00E+00	0.46	5.92E-01	9.36E-01
**AL031963.2**	0.76	7.00E-01	1.00E+00	-0.79	5.92E-01	9.36E-01
**ABCE1**	0.15	5.04E-01	1.00E+00	0.11	5.92E-01	9.36E-01
**KCNS1**	-0.10	8.03E-01	1.00E+00	0.46	5.92E-01	9.36E-01
**C8orf82**	-0.33	1.94E-01	1.00E+00	-0.42	5.92E-01	9.36E-01
**AL353801.3**	-1.10	7.51E-01	1.00E+00	0.84	5.92E-01	9.36E-01
**RAB30**	0.44	1.98E-01	1.00E+00	0.18	5.92E-01	9.36E-01

**BX640514.2**	-1.52	6.54E-02	1.00E+00	-0.36	5.92E-01	9.36E-01
**FSTL3**	0.24	6.34E-01	1.00E+00	0.36	5.92E-01	9.36E-01
**THUMPD3**	-0.02	9.13E-01	1.00E+00	-0.09	5.92E-01	9.36E-01
**DGCR6L**	-0.20	3.71E-01	1.00E+00	0.12	5.92E-01	9.36E-01
**DNHD1**	-0.37	3.52E-01	1.00E+00	0.31	5.92E-01	9.36E-01
**MRPS17**	0.44	1.01E-01	1.00E+00	-0.20	5.92E-01	9.36E-01
**LDB3**	-0.33	6.30E-01	1.00E+00	-0.35	5.92E-01	9.36E-01
**MAP7D2**	-1.01	2.32E-02	9.14E-01	-0.34	5.92E-01	9.36E-01
**RGS17P1**	-2.76	2.14E-01	1.00E+00	-0.56	5.92E-01	9.36E-01
**UBE2A**	0.02	9.25E-01	1.00E+00	-0.11	5.92E-01	9.36E-01
**MIB2**	-0.25	4.43E-01	1.00E+00	-0.33	5.92E-01	9.36E-01
**LEKR1**	-0.28	7.39E-01	1.00E+00	0.32	5.92E-01	9.36E-01
**DUS2**	-0.33	2.25E-01	1.00E+00	-0.12	5.92E-01	9.36E-01
**RAB7A**	0.04	8.19E-01	1.00E+00	0.06	5.92E-01	9.36E-01
**COCH**	-1.62	6.12E-01	1.00E+00	-0.39	5.93E-01	9.37E-01
**GCC2**	-0.09	7.76E-01	1.00E+00	0.11	5.93E-01	9.37E-01
**ERCC6L2**	-0.17	5.51E-01	1.00E+00	-0.12	5.93E-01	9.37E-01
**ACAD9**	-0.08	8.44E-01	1.00E+00	-0.16	5.93E-01	9.37E-01
**G15152**	-0.48	6.32E-01	1.00E+00	0.60	5.93E-01	9.37E-01
**INVS**	0.20	4.53E-01	1.00E+00	0.16	5.93E-01	9.37E-01
**LINC01366**	2.90	7.01E-02	1.00E+00	-0.82	5.93E-01	9.37E-01
**C4orf36**	-2.57	6.36E-03	4.78E-01	0.33	5.93E-01	9.37E-01
**PACS2**	-0.10	7.15E-01	1.00E+00	0.21	5.93E-01	9.37E-01
**PRRT2**	1.24	6.19E-01	1.00E+00	-0.36	5.93E-01	9.37E-01
**ARID2**	0.13	5.75E-01	1.00E+00	-0.17	5.93E-01	9.37E-01
**AL132656.2**	-0.48	5.50E-01	1.00E+00	0.33	5.93E-01	9.37E-01
**AC011287.1**	-0.61	6.68E-01	1.00E+00	-0.61	5.93E-01	9.37E-01
**ANKRD13C**	0.12	5.60E-01	1.00E+00	-0.11	5.93E-01	9.37E-01
**SPSB1**	-0.32	2.71E-01	1.00E+00	-0.24	5.93E-01	9.37E-01
**AC012378.2**	-2.97	6.43E-03	4.81E-01	-0.61	5.93E-01	9.37E-01
**MMP24OS**	0.04	8.80E-01	1.00E+00	0.34	5.93E-01	9.37E-01
**NOX5**	-0.27	6.54E-01	1.00E+00	0.51	5.93E-01	9.37E-01

**C18orf54**	0.21	5.94E-01	1.00E+00	-0.32	5.93E-01	9.37E-01
**ACOX3**	-0.31	1.76E-01	1.00E+00	0.11	5.93E-01	9.37E-01
**Z82243.1**	0.05	9.89E-01	1.00E+00	-0.69	5.93E-01	9.37E-01
**MRPL51**	0.09	5.92E-01	1.00E+00	-0.15	5.93E-01	9.37E-01
**KRTAP5-8**	-1.89	5.82E-01	1.00E+00	-1.71	5.93E-01	9.37E-01
**SLC35D2**	-0.05	8.86E-01	1.00E+00	-0.24	5.94E-01	9.37E-01
**COL21A1**	0.02	9.39E-01	1.00E+00	0.18	5.94E-01	9.37E-01
**MRPS5**	0.09	6.72E-01	1.00E+00	0.09	5.94E-01	9.37E-01
**ATP5IF1**	-0.08	7.02E-01	1.00E+00	-0.19	5.94E-01	9.37E-01
**SMYD2**	-0.03	8.74E-01	1.00E+00	-0.14	5.94E-01	9.37E-01
**SELENOO**	-0.02	9.36E-01	1.00E+00	0.30	5.94E-01	9.37E-01
**SNORD12C**	-1.71	6.18E-01	1.00E+00	-0.46	5.94E-01	9.37E-01
**FCRL6**	-0.19	8.55E-01	1.00E+00	1.68	5.94E-01	9.37E-01
**G10656**	-0.29	7.00E-01	1.00E+00	0.41	5.94E-01	9.37E-01
**STX7**	0.47	2.88E-02	9.88E-01	-0.08	5.94E-01	9.37E-01
**ZXDC**	-0.30	3.00E-01	1.00E+00	-0.28	5.94E-01	9.37E-01
**DSN1**	0.10	6.55E-01	1.00E+00	-0.13	5.94E-01	9.37E-01
**CNNM4**	-0.18	6.71E-01	1.00E+00	0.30	5.94E-01	9.37E-01
**SLC5A3**	0.52	1.98E-01	1.00E+00	0.17	5.94E-01	9.37E-01
**AC103591.4**	-0.40	6.71E-01	1.00E+00	-0.46	5.94E-01	9.37E-01
**DCAF12**	0.19	4.20E-01	1.00E+00	0.11	5.94E-01	9.37E-01
**TXNDC5**	-1.10	7.44E-02	1.00E+00	0.57	5.94E-01	9.37E-01
**HELB**	0.46	4.54E-01	1.00E+00	0.33	5.95E-01	9.37E-01
**CPA4**	-0.10	8.43E-01	1.00E+00	0.30	5.95E-01	9.37E-01
**ZNF596**	0.32	2.12E-01	1.00E+00	-0.24	5.95E-01	9.37E-01
**UXT-AS1**	-1.38	4.48E-01	1.00E+00	-0.53	5.95E-01	9.37E-01
**KIF17**	-0.30	4.55E-01	1.00E+00	0.37	5.95E-01	9.37E-01
**CARD8**	0.41	3.18E-01	1.00E+00	0.18	5.95E-01	9.37E-01
**OSGIN1**	-0.33	4.98E-01	1.00E+00	0.24	5.95E-01	9.37E-01
**ABCF2**	-0.34	1.56E-01	1.00E+00	0.12	5.95E-01	9.37E-01
**AC129492.1**	-3.02	4.40E-02	1.00E+00	0.32	5.95E-01	9.37E-01
**SYTL5**	1.55	2.50E-01	1.00E+00	0.66	5.95E-01	9.38E-01

**UTS2B**	0.28	8.41E-01	1.00E+00	0.70	5.95E-01	9.38E-01
**HTRA2**	0.16	4.82E-01	1.00E+00	-0.11	5.95E-01	9.38E-01
**CD80**	-1.76	4.94E-01	1.00E+00	1.68	5.95E-01	9.38E-01
**SERBP1P1**	-0.27	7.70E-01	1.00E+00	0.23	5.95E-01	9.38E-01
**OTULIN**	0.21	5.10E-01	1.00E+00	-0.19	5.95E-01	9.38E-01
**TMEM231P1**	-0.83	5.07E-01	1.00E+00	0.48	5.95E-01	9.38E-01
**AC021739.2**	-0.37	7.94E-01	1.00E+00	0.68	5.95E-01	9.38E-01
**ACSM2B**	-1.71	5.49E-01	1.00E+00	-1.04	5.95E-01	9.38E-01
**CHKB-DT**	1.00	2.12E-01	1.00E+00	-0.22	5.95E-01	9.38E-01
**SPP1**	1.26	3.78E-01	1.00E+00	1.66	5.96E-01	9.38E-01
**C16orf45**	0.39	3.45E-01	1.00E+00	0.26	5.96E-01	9.38E-01
**DANCR**	-0.37	4.30E-01	1.00E+00	-0.25	5.96E-01	9.38E-01
**LINC01694**	-2.36	1.61E-01	1.00E+00	0.86	5.96E-01	9.38E-01
**AC004908.2**	-2.78	3.43E-02	1.00E+00	-0.37	5.96E-01	9.38E-01
**ZNF221**	0.65	1.74E-01	1.00E+00	-0.36	5.96E-01	9.38E-01
**WIPI2**	0.03	8.60E-01	1.00E+00	-0.21	5.96E-01	9.38E-01
**NFXL1**	-0.14	6.90E-01	1.00E+00	0.16	5.96E-01	9.38E-01
**G7635**	-1.11	4.12E-01	1.00E+00	-0.35	5.96E-01	9.38E-01
**APEH**	-0.19	3.65E-01	1.00E+00	0.12	5.96E-01	9.38E-01
**ZNF808**	-0.01	9.77E-01	1.00E+00	-0.15	5.96E-01	9.38E-01
**THAP9**	0.22	3.86E-01	1.00E+00	-0.19	5.96E-01	9.38E-01
**DRC1**	-0.64	7.33E-01	1.00E+00	-0.73	5.96E-01	9.38E-01
**XLOC_006441**	-0.27	5.97E-01	1.00E+00	-0.32	5.96E-01	9.38E-01
**KRT28**	-10.48	1.66E-03	2.25E-01	-1.68	5.96E-01	9.38E-01
**MED6**	0.06	7.51E-01	1.00E+00	-0.11	5.96E-01	9.38E-01
**AC073861.1**	-0.10	7.83E-01	1.00E+00	-0.29	5.96E-01	9.38E-01
**IL37**	0.80	2.52E-01	1.00E+00	-0.52	5.96E-01	9.38E-01
**HCFC1R1**	-0.21	4.78E-01	1.00E+00	0.12	5.96E-01	9.38E-01
**SLC35F1**	-0.07	8.73E-01	1.00E+00	0.31	5.96E-01	9.38E-01
**CTCF**	-0.10	5.84E-01	1.00E+00	-0.19	5.96E-01	9.38E-01
**ZDHHC18**	-0.14	6.15E-01	1.00E+00	0.27	5.96E-01	9.38E-01
**AC009237.3**	-0.01	9.89E-01	1.00E+00	-0.28	5.96E-01	9.38E-01

**KIAA2013**	-0.21	4.26E-01	1.00E+00	-0.29	5.96E-01	9.38E-01
**XLOC_002080**	0.92	6.01E-01	1.00E+00	-0.72	5.97E-01	9.38E-01
**AC005779.1**	0.53	7.78E-01	1.00E+00	0.51	5.97E-01	9.38E-01
**PYURF**	0.17	3.54E-01	1.00E+00	-0.19	5.97E-01	9.38E-01
**SRP72**	-0.12	5.86E-01	1.00E+00	0.06	5.97E-01	9.38E-01
**RAB5IF**	0.02	9.49E-01	1.00E+00	0.24	5.97E-01	9.38E-01
**AL136984.1**	1.44	6.75E-01	1.00E+00	-1.13	5.97E-01	9.38E-01
**SNAI3**	-0.26	6.22E-01	1.00E+00	0.47	5.97E-01	9.38E-01
**TNNC1**	0.09	7.74E-01	1.00E+00	-0.29	5.97E-01	9.38E-01
**PDZD3**	1.73	1.95E-01	1.00E+00	-0.88	5.97E-01	9.38E-01
**SPICE1**	0.40	5.65E-01	1.00E+00	0.31	5.97E-01	9.38E-01
**TMEM167A**	0.33	1.44E-01	1.00E+00	-0.12	5.97E-01	9.38E-01
**LYPLAL1-DT**	-2.70	1.00E-01	1.00E+00	-0.52	5.97E-01	9.38E-01
**CCDC152**	0.72	1.87E-01	1.00E+00	0.24	5.97E-01	9.38E-01
**KRTAP26-1**	-5.08	1.21E-01	1.00E+00	-1.69	5.97E-01	9.38E-01
**CASP8**	-0.06	8.81E-01	1.00E+00	0.17	5.97E-01	9.38E-01
**BEND4**	-1.04	5.07E-01	1.00E+00	0.68	5.97E-01	9.38E-01
**GPATCH8**	-0.24	2.08E-01	1.00E+00	0.14	5.97E-01	9.38E-01
**G37438**	-0.54	5.67E-01	1.00E+00	-0.42	5.97E-01	9.38E-01
**AQP5**	-0.14	9.04E-01	1.00E+00	-0.44	5.97E-01	9.38E-01
**G35531**	1.97	4.07E-01	1.00E+00	-0.57	5.98E-01	9.38E-01
**LCE6A**	0.02	9.75E-01	1.00E+00	0.38	5.98E-01	9.38E-01
**TXNL4B**	0.09	7.29E-01	1.00E+00	0.11	5.98E-01	9.38E-01
**LINC02303**	0.04	9.91E-01	1.00E+00	1.31	5.98E-01	9.38E-01
**NPM1P30**	-1.52	6.60E-01	1.00E+00	-0.64	5.98E-01	9.38E-01
**GABRA3**	-1.76	6.93E-03	5.08E-01	-0.56	5.98E-01	9.38E-01
**AL021068.1**	0.53	4.07E-01	1.00E+00	0.36	5.98E-01	9.38E-01
**G480**	-0.82	6.13E-01	1.00E+00	-0.54	5.98E-01	9.38E-01
**AC022809.1**	0.60	8.40E-01	1.00E+00	1.06	5.98E-01	9.38E-01
**AQP3**	-0.79	7.06E-02	1.00E+00	-0.29	5.98E-01	9.38E-01
**TFEB**	-0.31	1.86E-01	1.00E+00	0.24	5.98E-01	9.38E-01
**HNRNPD**	-0.25	2.06E-01	1.00E+00	-0.25	5.98E-01	9.38E-01

**MAPK8IP1**	-0.48	3.38E-01	1.00E+00	0.41	5.98E-01	9.38E-01
**WDR55**	0.10	6.58E-01	1.00E+00	-0.10	5.98E-01	9.38E-01
**ZNF2**	0.39	1.93E-01	1.00E+00	-0.15	5.98E-01	9.38E-01
**SLC38A2**	0.04	8.90E-01	1.00E+00	-0.13	5.98E-01	9.38E-01
**LRRFIP2**	-0.02	9.45E-01	1.00E+00	0.10	5.98E-01	9.38E-01
**AL159169.2**	-0.06	9.86E-01	1.00E+00	0.60	5.98E-01	9.38E-01
**TRAM1L1**	-0.59	7.89E-02	1.00E+00	0.22	5.98E-01	9.38E-01
**DLL4**	0.29	5.61E-01	1.00E+00	0.29	5.98E-01	9.38E-01
**RPS3AP25**	-1.21	4.79E-01	1.00E+00	-0.36	5.98E-01	9.38E-01
**NLE1**	0.14	6.12E-01	1.00E+00	0.22	5.98E-01	9.38E-01
**SFRP4**	0.42	7.09E-01	1.00E+00	1.51	5.98E-01	9.38E-01
**AC012676.5**	-1.13	5.45E-01	1.00E+00	0.72	5.98E-01	9.38E-01
**UCK1**	0.16	4.82E-01	1.00E+00	0.15	5.98E-01	9.38E-01
**ADCY9**	-0.04	8.75E-01	1.00E+00	0.36	5.98E-01	9.38E-01
**CAMKK1**	-0.48	1.30E-01	1.00E+00	-0.21	5.98E-01	9.38E-01
**AL139022.2**	-0.46	6.33E-01	1.00E+00	0.62	5.98E-01	9.38E-01
**HIF1AN**	0.49	2.93E-02	9.91E-01	0.10	5.98E-01	9.38E-01
**SMARCE1**	0.40	2.36E-01	1.00E+00	0.20	5.99E-01	9.38E-01
**TPTE2P1**	0.91	3.96E-01	1.00E+00	0.40	5.99E-01	9.38E-01
**ILVBL**	-0.03	8.88E-01	1.00E+00	0.10	5.99E-01	9.38E-01
**GK5**	0.19	4.13E-01	1.00E+00	-0.21	5.99E-01	9.38E-01
**AC115989.1**	-0.22	9.37E-01	1.00E+00	-1.09	5.99E-01	9.38E-01
**AC022075.2**	0.83	5.93E-01	1.00E+00	-0.67	5.99E-01	9.38E-01
**UEVLD**	0.04	8.96E-01	1.00E+00	-0.09	5.99E-01	9.38E-01
**G31307**	-1.18	4.35E-01	1.00E+00	-0.38	5.99E-01	9.38E-01
**SCN2A**	-0.36	7.60E-01	1.00E+00	0.46	5.99E-01	9.38E-01
**PRR11**	-0.44	2.89E-01	1.00E+00	0.27	5.99E-01	9.38E-01
**ITIH5**	1.15	9.73E-02	1.00E+00	0.33	5.99E-01	9.38E-01
**AC108749.1**	-1.53	4.85E-01	1.00E+00	-0.66	5.99E-01	9.38E-01
**GORAB**	0.03	9.38E-01	1.00E+00	-0.18	5.99E-01	9.38E-01
**CREBBP**	-0.36	1.46E-01	1.00E+00	0.25	5.99E-01	9.38E-01
**MDN1**	0.12	6.45E-01	1.00E+00	0.21	5.99E-01	9.38E-01

**SLC35A2**	-0.02	9.30E-01	1.00E+00	0.12	5.99E-01	9.38E-01
**CCT5**	-0.16	2.94E-01	1.00E+00	0.09	5.99E-01	9.38E-01
**FAM222A-AS1**	-0.20	8.58E-01	1.00E+00	-0.69	5.99E-01	9.38E-01
**CLP1**	-0.15	3.58E-01	1.00E+00	0.08	5.99E-01	9.38E-01
**MAP6D1**	-0.41	6.07E-01	1.00E+00	0.66	6.00E-01	9.39E-01
**AC006453.2**	-0.38	7.24E-01	1.00E+00	0.38	6.00E-01	9.39E-01
**TDGF1**	-0.70	7.15E-01	1.00E+00	-0.67	6.00E-01	9.39E-01
**INSYN2B**	-0.19	7.93E-01	1.00E+00	0.51	6.00E-01	9.39E-01
**TIMELESS**	-0.64	4.09E-03	3.55E-01	0.25	6.00E-01	9.39E-01
**AC012603.1**	-3.35	3.92E-02	1.00E+00	0.50	6.00E-01	9.39E-01
**MYOZ3**	-0.79	3.22E-01	1.00E+00	0.35	6.00E-01	9.39E-01
**SRMS**	-2.69	9.72E-04	1.61E-01	0.62	6.00E-01	9.39E-01
**RPSAP47**	-0.16	8.84E-01	1.00E+00	0.39	6.00E-01	9.39E-01
**CENPK**	-0.22	6.20E-01	1.00E+00	-0.23	6.00E-01	9.39E-01
**BX284668.6**	0.23	9.46E-01	1.00E+00	0.71	6.00E-01	9.39E-01
**DAZAP2P1**	-2.02	4.59E-01	1.00E+00	-0.42	6.00E-01	9.39E-01
**CYP27A1**	-0.11	7.85E-01	1.00E+00	0.23	6.00E-01	9.39E-01
**ARRDC3-AS1**	0.57	3.98E-01	1.00E+00	0.24	6.00E-01	9.39E-01
**AL078604.1**	0.13	9.70E-01	1.00E+00	0.45	6.00E-01	9.39E-01
**SLC4A3**	-0.53	4.51E-01	1.00E+00	0.40	6.00E-01	9.39E-01
**USP10**	0.02	8.92E-01	1.00E+00	-0.08	6.00E-01	9.39E-01
**TRIB3**	-0.09	7.98E-01	1.00E+00	-0.15	6.00E-01	9.39E-01
**TNNC2**	0.66	6.73E-02	1.00E+00	-0.27	6.00E-01	9.39E-01
**GON4L**	-0.24	1.99E-01	1.00E+00	0.13	6.00E-01	9.39E-01
**NTF4**	-0.26	4.37E-01	1.00E+00	-0.28	6.00E-01	9.39E-01
**RGP1**	0.25	3.61E-01	1.00E+00	0.30	6.01E-01	9.39E-01
**G36500**	-2.52	1.39E-01	1.00E+00	0.41	6.01E-01	9.39E-01
**TMEM165**	0.31	2.33E-01	1.00E+00	-0.22	6.01E-01	9.39E-01
**AC005840.4**	-2.68	2.33E-01	1.00E+00	0.53	6.01E-01	9.39E-01
**AGO4**	0.32	2.49E-01	1.00E+00	-0.16	6.01E-01	9.39E-01
**PAXIP1-AS1**	0.13	7.21E-01	1.00E+00	0.21	6.01E-01	9.39E-01
**PIP4P1**	-0.22	4.07E-01	1.00E+00	-0.29	6.01E-01	9.39E-01

**CXCL11**	-2.11	3.07E-02	1.00E+00	1.64	6.01E-01	9.39E-01
**SMIM14**	0.27	1.85E-01	1.00E+00	-0.12	6.01E-01	9.39E-01
**SLC35E3**	-0.23	3.39E-01	1.00E+00	0.11	6.01E-01	9.39E-01
**XPO7**	0.05	7.54E-01	1.00E+00	0.16	6.01E-01	9.39E-01
**LINC02254**	-0.68	8.44E-01	1.00E+00	-0.95	6.01E-01	9.39E-01
**PRPH2**	0.25	8.02E-01	1.00E+00	-1.50	6.01E-01	9.39E-01
**G17634**	NA	NA	NA	-0.95	6.01E-01	9.39E-01
**PUM1**	-0.06	6.97E-01	1.00E+00	-0.10	6.01E-01	9.39E-01
**TACC2**	-0.15	5.95E-01	1.00E+00	-0.15	6.01E-01	9.39E-01
**LINC01277**	-0.41	7.62E-01	1.00E+00	-0.56	6.01E-01	9.39E-01
**AL139120.1**	0.32	9.28E-01	1.00E+00	0.47	6.01E-01	9.39E-01
**CAPS**	-0.07	8.88E-01	1.00E+00	0.26	6.01E-01	9.39E-01
**AL590652.1**	-0.17	7.66E-01	1.00E+00	-0.35	6.01E-01	9.39E-01
**LRRC37BP1**	0.04	9.08E-01	1.00E+00	0.20	6.01E-01	9.39E-01
**DDB2**	-0.17	4.77E-01	1.00E+00	0.10	6.01E-01	9.39E-01
**AC108062.1**	0.09	8.85E-01	1.00E+00	-0.32	6.02E-01	9.39E-01
**FBXO15**	-0.39	6.76E-01	1.00E+00	-0.57	6.02E-01	9.39E-01
**KXD1**	-0.14	4.05E-01	1.00E+00	0.16	6.02E-01	9.39E-01
**ST13P20**	-0.99	2.66E-01	1.00E+00	0.51	6.02E-01	9.39E-01
**MRC1**	-1.08	3.44E-01	1.00E+00	0.48	6.02E-01	9.39E-01
**HMGN1P38**	-0.11	9.51E-01	1.00E+00	0.31	6.02E-01	9.39E-01
**TNIP2**	-0.14	5.40E-01	1.00E+00	0.29	6.02E-01	9.39E-01
**ATG12**	0.25	1.86E-01	1.00E+00	-0.16	6.02E-01	9.39E-01
**AL592429.2**	2.03	2.65E-02	9.49E-01	-0.48	6.02E-01	9.39E-01
**EMX2OS**	0.31	5.03E-01	1.00E+00	0.21	6.02E-01	9.39E-01
**SNX22**	-0.49	4.57E-01	1.00E+00	0.34	6.02E-01	9.39E-01
**PCDHA12**	1.49	2.29E-01	1.00E+00	-0.78	6.02E-01	9.39E-01
**WFDC2**	0.27	5.13E-01	1.00E+00	0.28	6.02E-01	9.39E-01
**GPD2**	0.09	7.35E-01	1.00E+00	-0.13	6.02E-01	9.39E-01
**ETV3**	0.17	5.76E-01	1.00E+00	0.22	6.02E-01	9.39E-01
**RHOV**	-0.21	5.05E-01	1.00E+00	-0.28	6.02E-01	9.39E-01
**BRIP1**	-0.34	3.42E-01	1.00E+00	0.31	6.02E-01	9.39E-01

**ERICH6-AS1**	-0.28	7.19E-01	1.00E+00	-0.28	6.02E-01	9.39E-01
**NRM**	-0.33	1.84E-01	1.00E+00	-0.16	6.02E-01	9.39E-01
**LINC01358**	-0.46	6.98E-01	1.00E+00	0.31	6.02E-01	9.39E-01
**EME1**	0.14	8.57E-01	1.00E+00	0.57	6.02E-01	9.39E-01
**AC232271.1**	1.52	4.16E-01	1.00E+00	0.64	6.02E-01	9.39E-01
**PDGFC**	0.36	1.83E-01	1.00E+00	0.12	6.02E-01	9.39E-01
**AP001055.1**	-1.38	1.77E-01	1.00E+00	0.48	6.02E-01	9.39E-01
**XLOC_008164**	-2.02	6.40E-02	1.00E+00	0.58	6.03E-01	9.39E-01
**PDE1A**	-0.10	8.45E-01	1.00E+00	0.33	6.03E-01	9.39E-01
**ZMYM2**	0.04	8.72E-01	1.00E+00	0.11	6.03E-01	9.39E-01
**EIF4E2**	-0.01	9.37E-01	1.00E+00	0.08	6.03E-01	9.39E-01
**G7721**	-0.57	8.28E-01	1.00E+00	-1.53	6.03E-01	9.39E-01
**NFIC**	-0.31	3.04E-01	1.00E+00	0.18	6.03E-01	9.39E-01
**OCIAD2**	-0.32	1.16E-01	1.00E+00	-0.20	6.03E-01	9.39E-01
**HEXA**	0.37	3.77E-01	1.00E+00	0.19	6.03E-01	9.39E-01
**LINC00158**	-2.79	4.12E-01	1.00E+00	0.90	6.03E-01	9.39E-01
**EIF2B1**	-0.17	3.86E-01	1.00E+00	-0.11	6.03E-01	9.39E-01
**RERG**	0.13	7.12E-01	1.00E+00	-0.18	6.03E-01	9.39E-01
**AC109460.2**	2.46	2.60E-01	1.00E+00	-0.93	6.03E-01	9.39E-01
**MKX**	0.12	8.78E-01	1.00E+00	0.95	6.03E-01	9.39E-01
**SMAP2**	-0.01	9.84E-01	1.00E+00	0.30	6.03E-01	9.39E-01
**CBX7**	0.32	2.87E-01	1.00E+00	-0.22	6.03E-01	9.39E-01
**GOLGA1**	-0.26	2.75E-01	1.00E+00	0.14	6.03E-01	9.39E-01
**RAB3GAP2**	0.15	5.09E-01	1.00E+00	0.11	6.03E-01	9.40E-01
**SSTR2**	-0.98	7.60E-02	1.00E+00	0.37	6.03E-01	9.40E-01
**XLOC_009060**	0.52	8.79E-01	1.00E+00	0.64	6.03E-01	9.40E-01
**ACTR8**	-0.18	4.43E-01	1.00E+00	0.08	6.04E-01	9.40E-01
**TMEM209**	0.21	2.57E-01	1.00E+00	-0.17	6.04E-01	9.40E-01
**AP006222.2**	-0.12	8.01E-01	1.00E+00	-0.22	6.04E-01	9.40E-01
**ANK2**	0.33	3.99E-01	1.00E+00	0.25	6.04E-01	9.40E-01
**IDO1**	-0.31	7.18E-01	1.00E+00	1.63	6.04E-01	9.40E-01
**G32333**	0.32	6.21E-01	1.00E+00	0.50	6.04E-01	9.40E-01

**G37465**	-0.67	4.02E-01	1.00E+00	0.55	6.04E-01	9.40E-01
**AL512646.1**	NA	NA	NA	-1.63	6.04E-01	9.40E-01
**PLXNA3**	-0.26	3.02E-01	1.00E+00	0.27	6.04E-01	9.40E-01
**ASB16**	-1.41	6.75E-02	1.00E+00	-0.62	6.04E-01	9.40E-01
**BX470102.1**	1.07	1.73E-01	1.00E+00	0.37	6.04E-01	9.40E-01
**PYGM**	-0.55	3.68E-01	1.00E+00	-1.04	6.04E-01	9.40E-01
**RNF152**	-0.08	8.53E-01	1.00E+00	-0.25	6.04E-01	9.40E-01
**P2RY1**	-0.11	8.12E-01	1.00E+00	-0.24	6.04E-01	9.40E-01
**CTNNA1**	-0.18	2.63E-01	1.00E+00	0.11	6.04E-01	9.40E-01
**FARSA**	-0.03	8.76E-01	1.00E+00	0.11	6.04E-01	9.40E-01
**MUS81**	-0.22	4.22E-01	1.00E+00	0.15	6.04E-01	9.40E-01
**CCDC113**	-0.11	6.30E-01	1.00E+00	0.13	6.04E-01	9.40E-01
**WBP4**	0.28	1.33E-01	1.00E+00	-0.11	6.04E-01	9.40E-01
**RHOT2**	-0.20	2.71E-01	1.00E+00	0.12	6.04E-01	9.40E-01
**FBXO11**	-0.23	1.10E-01	1.00E+00	0.20	6.04E-01	9.40E-01
**CCDC134**	0.22	5.71E-01	1.00E+00	-0.21	6.04E-01	9.40E-01
**AC012313.2**	0.78	4.68E-01	1.00E+00	-0.60	6.04E-01	9.40E-01
**FAM66A**	-1.78	4.67E-02	1.00E+00	-0.74	6.05E-01	9.40E-01
**TWISTNB**	-0.03	8.58E-01	1.00E+00	-0.12	6.05E-01	9.40E-01
**BX571846.1**	3.14	8.52E-02	1.00E+00	0.61	6.05E-01	9.40E-01
**AL512274.1**	0.39	2.37E-01	1.00E+00	0.26	6.05E-01	9.40E-01
**NAALADL1**	0.06	8.78E-01	1.00E+00	0.15	6.05E-01	9.40E-01
**GPR143**	0.07	8.69E-01	1.00E+00	0.24	6.05E-01	9.40E-01
**AC024451.2**	-2.02	3.11E-01	1.00E+00	0.64	6.05E-01	9.40E-01
**AC016734.1**	-2.33	1.27E-01	1.00E+00	-0.26	6.05E-01	9.40E-01
**DLG1-AS1**	-0.36	8.33E-01	1.00E+00	-0.58	6.05E-01	9.40E-01
**AL133330.1**	-1.55	4.06E-01	1.00E+00	-0.51	6.05E-01	9.40E-01
**FAM71F2**	-0.02	9.88E-01	1.00E+00	0.63	6.05E-01	9.40E-01
**DIAPH3**	-0.17	6.91E-01	1.00E+00	0.23	6.05E-01	9.40E-01
**RBM19**	-0.25	2.72E-01	1.00E+00	0.17	6.05E-01	9.40E-01
**OSR2**	1.11	1.84E-02	8.51E-01	0.28	6.05E-01	9.40E-01
**MKRN2**	0.04	8.69E-01	1.00E+00	-0.10	6.05E-01	9.40E-01

**MRE11**	0.03	9.14E-01	1.00E+00	-0.10	6.05E-01	9.40E-01
**TMCO1**	0.13	4.17E-01	1.00E+00	-0.12	6.05E-01	9.40E-01
**NOS1AP**	-0.05	9.21E-01	1.00E+00	-0.30	6.05E-01	9.40E-01
**FAM86B2**	-2.47	3.07E-02	1.00E+00	-0.39	6.05E-01	9.40E-01
**ARMC2**	-0.20	6.06E-01	1.00E+00	-0.19	6.05E-01	9.40E-01
**NOL9**	0.09	7.83E-01	1.00E+00	-0.16	6.05E-01	9.40E-01
**C17orf67**	-0.80	1.45E-01	1.00E+00	0.37	6.05E-01	9.40E-01
**PIAS3**	-0.47	1.57E-01	1.00E+00	0.16	6.05E-01	9.40E-01
**CHP2**	0.34	4.04E-01	1.00E+00	-0.47	6.06E-01	9.40E-01
**ADRB1**	-0.08	8.96E-01	1.00E+00	0.32	6.06E-01	9.40E-01
**XLOC_001097**	0.25	9.42E-01	1.00E+00	-0.62	6.06E-01	9.40E-01
**DCTD**	0.07	6.77E-01	1.00E+00	0.08	6.06E-01	9.40E-01
**2-Mar**	-0.17	5.26E-01	1.00E+00	-0.32	6.06E-01	9.40E-01
**STMND1**	1.11	3.76E-01	1.00E+00	-0.76	6.06E-01	9.40E-01
**RAB27B**	0.04	9.17E-01	1.00E+00	0.25	6.06E-01	9.40E-01
**METTL23**	0.11	6.16E-01	1.00E+00	-0.15	6.06E-01	9.40E-01
**RP11-353N4.4**	0.02	9.88E-01	1.00E+00	0.35	6.06E-01	9.40E-01
**AC083906.5**	-4.49	5.63E-03	4.46E-01	-0.52	6.06E-01	9.40E-01
**MORC2**	0.02	9.32E-01	1.00E+00	0.32	6.06E-01	9.40E-01
**AC073107.1**	-0.47	2.88E-01	1.00E+00	0.19	6.06E-01	9.40E-01
**MORC2-AS1**	-2.10	4.84E-01	1.00E+00	0.53	6.06E-01	9.40E-01
**C12orf49**	0.39	8.31E-02	1.00E+00	0.19	6.06E-01	9.40E-01
**H3F3BP1**	-0.30	9.31E-01	1.00E+00	-0.35	6.06E-01	9.40E-01
**TFAP2A-AS1**	-0.55	5.67E-01	1.00E+00	0.33	6.06E-01	9.40E-01
**AC093110.1**	0.33	7.88E-01	1.00E+00	0.52	6.06E-01	9.40E-01
**AC090607.2**	0.61	8.57E-01	1.00E+00	0.60	6.07E-01	9.40E-01
**C11orf74**	0.50	1.31E-01	1.00E+00	-0.16	6.07E-01	9.40E-01
**MARK2**	-0.08	7.78E-01	1.00E+00	-0.31	6.07E-01	9.40E-01
**MTR**	0.31	3.70E-01	1.00E+00	0.16	6.07E-01	9.40E-01
**BICRAL**	-0.07	7.22E-01	1.00E+00	0.18	6.07E-01	9.40E-01
**CAPN6**	-0.03	9.62E-01	1.00E+00	0.39	6.07E-01	9.40E-01
**AL451164.1**	0.03	9.89E-01	1.00E+00	0.69	6.07E-01	9.40E-01

**DUOX1**	-0.16	7.02E-01	1.00E+00	0.34	6.07E-01	9.40E-01
**AP001208.2**	-0.01	9.96E-01	1.00E+00	-0.47	6.07E-01	9.40E-01
**G39661**	1.27	2.82E-01	1.00E+00	0.44	6.07E-01	9.40E-01
**AL031291.1**	-0.32	7.57E-01	1.00E+00	0.78	6.07E-01	9.40E-01
**PGAP1**	0.24	3.79E-01	1.00E+00	-0.20	6.07E-01	9.40E-01
**SVOPL**	-0.82	4.78E-01	1.00E+00	-0.47	6.07E-01	9.40E-01
**AC023157.3**	0.18	6.45E-01	1.00E+00	0.16	6.07E-01	9.40E-01
**CDKL3**	-1.77	1.79E-01	1.00E+00	0.31	6.07E-01	9.40E-01
**TP53BP2**	-0.08	7.25E-01	1.00E+00	0.22	6.07E-01	9.40E-01
**UNC119B**	0.73	1.55E-01	1.00E+00	0.23	6.07E-01	9.40E-01
**AC138866.1**	-3.32	2.05E-02	8.94E-01	0.28	6.07E-01	9.40E-01
**CCL24**	0.09	9.36E-01	1.00E+00	-0.37	6.07E-01	9.40E-01
**KIAA0556**	-0.29	2.45E-01	1.00E+00	0.28	6.07E-01	9.40E-01
**GRK2**	0.03	8.90E-01	1.00E+00	0.31	6.07E-01	9.40E-01
**PAFAH1B3**	-0.30	1.96E-01	1.00E+00	-0.13	6.07E-01	9.40E-01
**IFT81**	0.26	3.83E-01	1.00E+00	0.13	6.07E-01	9.41E-01
**FAM222A**	-0.21	5.64E-01	1.00E+00	-0.34	6.07E-01	9.41E-01
**MIR9-3HG**	-0.88	1.33E-01	1.00E+00	0.59	6.08E-01	9.41E-01
**PEBP1P2**	1.17	5.30E-01	1.00E+00	-0.30	6.08E-01	9.41E-01
**AC022150.4**	0.65	1.50E-01	1.00E+00	-0.30	6.08E-01	9.41E-01
**YY1AP1**	-0.34	3.27E-02	1.00E+00	0.10	6.08E-01	9.41E-01
**TOMM22**	-0.02	9.10E-01	1.00E+00	-0.08	6.08E-01	9.41E-01
**KCNU1**	-1.61	6.32E-01	1.00E+00	0.73	6.08E-01	9.41E-01
**CSTA**	-0.02	9.62E-01	1.00E+00	0.26	6.08E-01	9.41E-01
**PAXIP1**	-0.21	4.24E-01	1.00E+00	0.18	6.08E-01	9.41E-01
**AC018553.2**	0.60	2.99E-01	1.00E+00	0.36	6.08E-01	9.41E-01
**UBQLN1**	0.13	5.36E-01	1.00E+00	-0.12	6.08E-01	9.41E-01
**FCGR3B**	-0.29	7.21E-01	1.00E+00	-0.27	6.08E-01	9.41E-01
**TRAV5**	0.78	7.32E-01	1.00E+00	0.78	6.08E-01	9.41E-01
**AC025164.1**	-0.31	4.60E-01	1.00E+00	-0.29	6.08E-01	9.41E-01
**EMG1**	0.25	2.48E-01	1.00E+00	-0.15	6.08E-01	9.41E-01
**AC022898.1**	2.62	4.41E-01	1.00E+00	-0.87	6.08E-01	9.41E-01

**ADORA1**	0.66	5.70E-01	1.00E+00	-1.61	6.08E-01	9.41E-01
**XLOC_009221**	-0.29	9.32E-01	1.00E+00	0.83	6.08E-01	9.41E-01
**OGA**	0.00	9.98E-01	1.00E+00	-0.15	6.08E-01	9.41E-01
**FABP5P3**	1.08	7.53E-01	1.00E+00	-0.30	6.08E-01	9.41E-01
**G28383**	3.75	1.18E-01	1.00E+00	-0.54	6.08E-01	9.41E-01
**AC005920.1**	0.70	6.23E-01	1.00E+00	0.51	6.08E-01	9.41E-01
**AP000692.2**	0.95	3.51E-01	1.00E+00	-0.36	6.08E-01	9.41E-01
**AL137918.1**	1.19	6.74E-01	1.00E+00	-0.88	6.08E-01	9.41E-01
**PREP**	0.08	7.80E-01	1.00E+00	-0.15	6.08E-01	9.41E-01
**MCM9**	-0.37	2.21E-01	1.00E+00	0.16	6.09E-01	9.41E-01
**MTHFSD**	0.07	7.72E-01	1.00E+00	-0.12	6.09E-01	9.41E-01
**AC011997.1**	-0.29	9.01E-01	1.00E+00	0.63	6.09E-01	9.41E-01
**XLOC_010292**	-0.72	3.49E-01	1.00E+00	0.42	6.09E-01	9.41E-01
**TIMM8AP1**	-1.86	2.12E-01	1.00E+00	0.39	6.09E-01	9.41E-01
**AL138921.2**	0.59	5.67E-01	1.00E+00	-0.47	6.09E-01	9.41E-01
**CAD**	-0.17	5.50E-01	1.00E+00	0.27	6.09E-01	9.41E-01
**RFNG**	-0.10	7.05E-01	1.00E+00	0.22	6.09E-01	9.41E-01
**TNFRSF11B**	-0.32	5.04E-01	1.00E+00	-0.39	6.09E-01	9.41E-01
**TMED3**	-0.20	4.05E-01	1.00E+00	0.14	6.09E-01	9.41E-01
**BUB1B**	-0.26	4.82E-01	1.00E+00	-0.36	6.09E-01	9.41E-01
**PAQR6**	-0.49	4.53E-01	1.00E+00	0.41	6.09E-01	9.41E-01
**OPA1**	0.13	3.93E-01	1.00E+00	-0.07	6.09E-01	9.41E-01
**KIR2DL3**	-2.11	5.36E-01	1.00E+00	-0.83	6.09E-01	9.41E-01
**KDM7A**	0.42	2.60E-01	1.00E+00	0.21	6.09E-01	9.41E-01
**DBNDD2**	-0.15	7.60E-01	1.00E+00	0.27	6.09E-01	9.41E-01
**FASTKD2**	-0.05	7.91E-01	1.00E+00	-0.14	6.09E-01	9.41E-01
**HNRNPA1P21**	0.19	8.75E-01	1.00E+00	0.71	6.09E-01	9.41E-01
**TBC1D8B**	0.26	6.39E-01	1.00E+00	-0.15	6.09E-01	9.41E-01
**CDIPTOSP**	-2.16	3.44E-01	1.00E+00	-0.73	6.09E-01	9.41E-01
**AL132656.4**	0.21	5.27E-01	1.00E+00	0.27	6.09E-01	9.41E-01
**AC110373.1**	-0.29	7.44E-01	1.00E+00	-0.64	6.10E-01	9.41E-01
**SELENOW**	0.19	2.27E-01	1.00E+00	-0.14	6.10E-01	9.42E-01

**CHDH**	0.03	9.67E-01	1.00E+00	-0.25	6.10E-01	9.42E-01
**AC008760.2**	-0.05	9.74E-01	1.00E+00	1.47	6.10E-01	9.42E-01
**SNAP23**	0.28	2.77E-01	1.00E+00	0.15	6.10E-01	9.42E-01
**TMEM246**	0.15	6.19E-01	1.00E+00	-0.16	6.10E-01	9.42E-01
**TMEM201**	-0.23	2.80E-01	1.00E+00	0.24	6.10E-01	9.42E-01
**AC083949.1**	-0.38	9.11E-01	1.00E+00	0.79	6.10E-01	9.42E-01
**AC026124.2**	-3.06	4.83E-02	1.00E+00	0.46	6.10E-01	9.42E-01
**KRTAP3-3**	-23.49	6.16E-12	4.05E-09	-1.63	6.10E-01	9.42E-01
**XLOC_002455**	-1.03	3.42E-01	1.00E+00	0.58	6.11E-01	9.42E-01
**NISCH**	-0.24	3.29E-01	1.00E+00	0.18	6.11E-01	9.42E-01
**AC022075.1**	0.27	6.88E-01	1.00E+00	0.30	6.11E-01	9.42E-01
**KBTBD7**	0.31	2.07E-01	1.00E+00	-0.12	6.11E-01	9.42E-01
**SOCS6**	-0.12	6.26E-01	1.00E+00	0.11	6.11E-01	9.42E-01
**SF1**	-0.29	1.64E-01	1.00E+00	0.21	6.11E-01	9.42E-01
**PDCD7**	0.03	8.22E-01	1.00E+00	0.17	6.11E-01	9.42E-01
**XLOC_008357**	-1.29	1.75E-01	1.00E+00	0.34	6.11E-01	9.42E-01
**GUSBP2**	-0.35	7.15E-01	1.00E+00	-0.39	6.11E-01	9.42E-01
**EPB41L4A-AS1**	-0.21	5.12E-01	1.00E+00	0.17	6.11E-01	9.42E-01
**FAM219B**	0.15	5.85E-01	1.00E+00	-0.20	6.11E-01	9.42E-01
**COPB2**	0.05	7.95E-01	1.00E+00	0.10	6.11E-01	9.42E-01
**CCT6A**	-0.10	6.08E-01	1.00E+00	0.07	6.11E-01	9.42E-01
**MPDZ**	0.52	1.92E-01	1.00E+00	0.27	6.11E-01	9.42E-01
**G35852**	-1.94	5.71E-01	1.00E+00	-1.16	6.11E-01	9.42E-01
**VEGFC**	0.56	1.82E-01	1.00E+00	0.34	6.11E-01	9.42E-01
**AC027031.2**	0.25	6.93E-01	1.00E+00	-0.39	6.11E-01	9.42E-01
**RFLNB**	-0.20	6.91E-01	1.00E+00	-0.27	6.11E-01	9.42E-01
**CAPN12**	0.61	3.54E-01	1.00E+00	-0.42	6.11E-01	9.42E-01
**AP001922.6**	2.38	2.33E-02	9.15E-01	0.57	6.11E-01	9.42E-01
**VNN3**	NA	NA	NA	1.61	6.11E-01	9.42E-01
**RBBP4P1**	0.33	8.76E-01	1.00E+00	0.30	6.11E-01	9.42E-01
**ROCK1P1**	-0.19	8.70E-01	1.00E+00	-0.39	6.11E-01	9.42E-01
**FBXL17**	-0.03	8.87E-01	1.00E+00	0.23	6.11E-01	9.42E-01

**AC004918.3**	0.36	6.92E-01	1.00E+00	-0.53	6.11E-01	9.42E-01
**HINT2**	-0.71	8.37E-01	1.00E+00	-0.31	6.11E-01	9.42E-01
**PLCL1**	-0.15	7.55E-01	1.00E+00	0.31	6.11E-01	9.42E-01
**INPP5B**	0.06	7.57E-01	1.00E+00	0.17	6.12E-01	9.42E-01
**AK8**	-0.45	3.01E-01	1.00E+00	-0.23	6.12E-01	9.42E-01
**CRTC2**	-0.20	3.66E-01	1.00E+00	0.24	6.12E-01	9.42E-01
**C17orf75**	0.12	6.39E-01	1.00E+00	0.11	6.12E-01	9.42E-01
**IL10RB-DT**	0.26	7.75E-01	1.00E+00	0.43	6.12E-01	9.42E-01
**PIGC**	0.12	6.36E-01	1.00E+00	-0.12	6.12E-01	9.42E-01
**KRT18P34**	1.26	4.43E-01	1.00E+00	0.47	6.12E-01	9.42E-01
**NBPF14**	-0.89	2.61E-01	1.00E+00	-0.24	6.12E-01	9.42E-01
**FAM131B**	0.09	9.00E-01	1.00E+00	0.50	6.12E-01	9.42E-01
**AC005593.1**	-2.21	2.63E-01	1.00E+00	-0.60	6.12E-01	9.42E-01
**CTTNBP2**	0.26	4.89E-01	1.00E+00	0.22	6.12E-01	9.42E-01
**AC004540.1**	-0.25	7.15E-01	1.00E+00	-0.31	6.12E-01	9.43E-01
**ZBTB7C**	-0.32	1.47E-01	1.00E+00	-0.26	6.12E-01	9.43E-01
**DMTF1**	-0.03	9.05E-01	1.00E+00	-0.17	6.12E-01	9.43E-01
**GRP**	-2.81	5.97E-02	1.00E+00	0.63	6.12E-01	9.43E-01
**KMT2E-AS1**	-0.14	7.12E-01	1.00E+00	0.13	6.12E-01	9.43E-01
**LINC01012**	-0.67	6.97E-01	1.00E+00	0.56	6.13E-01	9.43E-01
**PCDH1**	-0.25	2.83E-01	1.00E+00	0.20	6.13E-01	9.43E-01
**EOGT**	0.32	2.66E-01	1.00E+00	0.18	6.13E-01	9.43E-01
**AF131216.1**	2.18	4.02E-01	1.00E+00	-0.52	6.13E-01	9.43E-01
**AC046185.2**	-0.16	8.06E-01	1.00E+00	-0.26	6.13E-01	9.43E-01
**GRPEL1**	-0.07	7.42E-01	1.00E+00	0.13	6.13E-01	9.43E-01
**CHRNA5**	-1.36	2.00E-01	1.00E+00	-0.39	6.13E-01	9.43E-01
**PIGT**	-0.04	8.68E-01	1.00E+00	0.11	6.13E-01	9.43E-01
**FAM122C**	-0.27	4.42E-01	1.00E+00	-0.21	6.13E-01	9.43E-01
**ERCC6**	-0.11	7.16E-01	1.00E+00	0.13	6.13E-01	9.43E-01
**HSF2BP**	-0.32	7.30E-01	1.00E+00	-0.32	6.13E-01	9.43E-01
**DNASE2B**	-2.04	5.54E-01	1.00E+00	-1.10	6.13E-01	9.43E-01
**DGCR11**	0.12	7.65E-01	1.00E+00	0.33	6.13E-01	9.43E-01

**PCDH8**	-2.76	3.42E-03	3.13E-01	0.68	6.13E-01	9.43E-01
**RIOX2**	-0.09	7.22E-01	1.00E+00	0.10	6.13E-01	9.43E-01
**PABPC5**	0.28	6.10E-01	1.00E+00	-0.43	6.13E-01	9.43E-01
**WWC1**	-0.10	6.40E-01	1.00E+00	0.25	6.13E-01	9.43E-01
**MYPN**	-3.64	8.75E-02	1.00E+00	0.78	6.13E-01	9.43E-01
**VIM-AS1**	0.00	9.96E-01	1.00E+00	-0.28	6.13E-01	9.43E-01
**AC092821.3**	-1.93	5.07E-01	1.00E+00	-0.66	6.14E-01	9.43E-01
**LRPPRC**	0.21	4.79E-01	1.00E+00	0.08	6.14E-01	9.43E-01
**AL033519.2**	0.36	7.46E-01	1.00E+00	-0.36	6.14E-01	9.43E-01
**RHBDF2**	-0.20	6.33E-01	1.00E+00	0.36	6.14E-01	9.43E-01
**HIRIP3**	0.04	8.28E-01	1.00E+00	0.09	6.14E-01	9.43E-01
**SYT6**	-1.29	2.00E-01	1.00E+00	-0.52	6.14E-01	9.43E-01
**ITLN1**	-3.18	3.22E-01	1.00E+00	0.89	6.14E-01	9.43E-01
**NUP107**	0.10	7.59E-01	1.00E+00	-0.14	6.14E-01	9.43E-01
**ZNF205**	-0.17	6.01E-01	1.00E+00	0.45	6.14E-01	9.43E-01
**NDUFV2**	0.81	8.05E-01	1.00E+00	0.27	6.14E-01	9.43E-01
**NUDT9P1**	-3.44	3.54E-02	1.00E+00	0.65	6.14E-01	9.43E-01
**IFT52**	0.19	3.47E-01	1.00E+00	-0.11	6.14E-01	9.43E-01
**AC006504.5**	0.43	2.35E-01	1.00E+00	-0.16	6.14E-01	9.43E-01
**WASHC2A**	-0.62	7.31E-02	1.00E+00	0.19	6.14E-01	9.43E-01
**AC073111.5**	-0.15	6.52E-01	1.00E+00	0.13	6.14E-01	9.43E-01
**ADGRF4**	0.63	2.36E-01	1.00E+00	0.38	6.14E-01	9.43E-01
**AC048346.1**	NA	NA	NA	1.59	6.14E-01	9.43E-01
**GPNMB**	0.02	9.35E-01	1.00E+00	0.13	6.14E-01	9.43E-01
**AC007639.1**	-2.19	5.23E-01	1.00E+00	-0.69	6.14E-01	9.43E-01
**PPP1R8**	-0.09	5.92E-01	1.00E+00	0.11	6.15E-01	9.43E-01
**RPL12P17**	1.15	7.38E-01	1.00E+00	-0.36	6.15E-01	9.43E-01
**LINC02437**	-0.80	8.27E-02	1.00E+00	-0.28	6.15E-01	9.43E-01
**PKD1L1**	0.21	8.17E-01	1.00E+00	0.54	6.15E-01	9.43E-01
**LINC02195**	-1.78	5.46E-01	1.00E+00	0.79	6.15E-01	9.43E-01
**XLOC_009153**	NA	NA	NA	0.64	6.15E-01	9.43E-01
**KRTAP9-3**	-18.04	1.08E-07	4.40E-05	-1.61	6.15E-01	9.43E-01

**AL365295.1**	0.02	9.74E-01	1.00E+00	-0.28	6.15E-01	9.43E-01
**LNCSRLR**	-2.84	2.97E-01	1.00E+00	-0.76	6.15E-01	9.43E-01
**RAB28**	0.28	2.23E-01	1.00E+00	-0.18	6.15E-01	9.43E-01
**CRB1**	-2.08	9.08E-02	1.00E+00	-0.51	6.15E-01	9.43E-01
**R3HDM2**	-0.24	3.71E-01	1.00E+00	0.14	6.15E-01	9.43E-01
**TMC7**	-0.53	3.55E-01	1.00E+00	0.43	6.15E-01	9.43E-01
**XLOC_011753**	-0.51	7.84E-01	1.00E+00	0.69	6.15E-01	9.43E-01
**ARIH1**	0.13	5.33E-01	1.00E+00	-0.13	6.15E-01	9.43E-01
**SGO2**	0.19	6.52E-01	1.00E+00	0.14	6.15E-01	9.43E-01
**CDK5**	-0.25	1.37E-01	1.00E+00	0.09	6.15E-01	9.43E-01
**C1orf226**	-0.36	2.68E-01	1.00E+00	-0.28	6.15E-01	9.43E-01
**AP4E1**	-0.17	6.36E-01	1.00E+00	0.15	6.15E-01	9.43E-01
**FAAHP1**	-0.54	8.69E-01	1.00E+00	0.76	6.15E-01	9.43E-01
**LNX1**	0.12	7.15E-01	1.00E+00	0.13	6.15E-01	9.43E-01
**RAB5B**	-0.07	8.15E-01	1.00E+00	0.17	6.15E-01	9.43E-01
**CRYL1**	0.10	8.01E-01	1.00E+00	-0.16	6.15E-01	9.43E-01
**CARNS1**	-1.20	1.40E-02	7.46E-01	-0.38	6.15E-01	9.43E-01
**MPZL1**	0.19	4.83E-01	1.00E+00	0.13	6.15E-01	9.43E-01
**OTOP1**	-0.01	9.90E-01	1.00E+00	-0.42	6.16E-01	9.43E-01
**MIER2**	-0.46	1.12E-01	1.00E+00	0.29	6.16E-01	9.43E-01
**XRCC6P2**	-0.03	9.85E-01	1.00E+00	-0.30	6.16E-01	9.43E-01
**ZNF682**	-0.34	4.30E-01	1.00E+00	-0.22	6.16E-01	9.43E-01
**ORM2**	NA	NA	NA	1.00	6.16E-01	9.44E-01
**EBNA1BP2**	-0.24	2.02E-01	1.00E+00	-0.12	6.16E-01	9.44E-01
**KDELC2**	0.24	2.39E-01	1.00E+00	-0.15	6.16E-01	9.44E-01
**CALCB**	-2.46	8.36E-02	1.00E+00	0.69	6.16E-01	9.44E-01
**SERPINB11**	1.76	6.07E-01	1.00E+00	1.61	6.16E-01	9.44E-01
**SSBP4**	-0.50	1.32E-02	7.22E-01	0.34	6.16E-01	9.44E-01
**AC007285.1**	0.25	6.88E-01	1.00E+00	0.47	6.16E-01	9.44E-01
**EIF3B**	-0.10	6.78E-01	1.00E+00	-0.25	6.16E-01	9.44E-01
**AC044787.1**	0.10	9.30E-01	1.00E+00	-0.32	6.16E-01	9.44E-01
**AC007494.2**	NA	NA	NA	0.60	6.16E-01	9.44E-01

**PROK1**	-0.07	9.65E-01	1.00E+00	0.72	6.16E-01	9.44E-01
**AL080317.1**	1.76	2.23E-02	8.98E-01	-0.33	6.16E-01	9.44E-01
**SIGLEC6**	0.53	6.48E-01	1.00E+00	-0.40	6.16E-01	9.44E-01
**TEFM**	0.12	7.45E-01	1.00E+00	-0.16	6.16E-01	9.44E-01
**LIPH**	0.46	6.26E-02	1.00E+00	-0.25	6.16E-01	9.44E-01
**SLC12A6**	0.39	1.75E-01	1.00E+00	0.23	6.16E-01	9.44E-01
**SLC4A8**	-1.59	6.75E-02	1.00E+00	0.59	6.16E-01	9.44E-01
**AL138885.3**	-0.50	8.56E-01	1.00E+00	0.57	6.17E-01	9.44E-01
**SYN1**	0.08	9.25E-01	1.00E+00	0.44	6.17E-01	9.44E-01
**AATK**	-0.72	1.35E-01	1.00E+00	-1.51	6.17E-01	9.44E-01
**EEF1B2**	-0.23	5.06E-01	1.00E+00	0.11	6.17E-01	9.44E-01
**G28443**	-0.84	3.28E-01	1.00E+00	0.41	6.17E-01	9.44E-01
**MAP2K1**	0.33	3.99E-01	1.00E+00	-0.25	6.17E-01	9.44E-01
**PDE9A**	-0.04	9.49E-01	1.00E+00	-0.28	6.17E-01	9.44E-01
**XLOC_000302**	0.42	3.74E-01	1.00E+00	-0.38	6.17E-01	9.44E-01
**MRPL1**	0.09	6.79E-01	1.00E+00	0.10	6.17E-01	9.44E-01
**IGHV2-5**	3.93	3.81E-02	1.00E+00	1.59	6.17E-01	9.44E-01
**PLCH1**	-0.83	2.13E-01	1.00E+00	-0.36	6.17E-01	9.44E-01
**LINC01560**	-0.15	6.66E-01	1.00E+00	-0.17	6.17E-01	9.44E-01
**CGNL1**	0.00	9.93E-01	1.00E+00	-0.23	6.17E-01	9.44E-01
**AC018742.1**	1.21	1.28E-01	1.00E+00	-0.37	6.17E-01	9.44E-01
**KTI12**	-0.32	4.39E-01	1.00E+00	-0.16	6.17E-01	9.44E-01
**SMIM5**	-0.15	7.67E-01	1.00E+00	0.26	6.17E-01	9.44E-01
**TSIX**	-1.93	5.74E-01	1.00E+00	-0.73	6.17E-01	9.44E-01
**FAM49B**	0.00	9.97E-01	1.00E+00	-0.15	6.17E-01	9.44E-01
**GTPBP10**	0.18	4.43E-01	1.00E+00	0.09	6.17E-01	9.44E-01
**NOP56**	-0.15	5.32E-01	1.00E+00	0.11	6.17E-01	9.44E-01
**LINC02599**	-2.40	2.83E-02	9.75E-01	0.71	6.17E-01	9.44E-01
**PSMD14**	-0.01	9.76E-01	1.00E+00	-0.14	6.17E-01	9.44E-01
**ECSIT**	-0.23	3.31E-01	1.00E+00	0.12	6.17E-01	9.44E-01
**CDCA7L**	0.15	6.52E-01	1.00E+00	-0.19	6.17E-01	9.44E-01
**GRIN2B**	-0.83	5.65E-01	1.00E+00	-0.47	6.17E-01	9.44E-01

**RALGAPA1P1**	0.34	6.66E-01	1.00E+00	-0.19	6.18E-01	9.44E-01
**POMGNT2**	0.00	9.96E-01	1.00E+00	-0.20	6.18E-01	9.44E-01
**AC011933.1**	-0.92	6.91E-01	1.00E+00	-0.25	6.18E-01	9.44E-01
**ZNF197-AS1**	-1.62	3.87E-01	1.00E+00	-0.59	6.18E-01	9.44E-01
**AC005790.1**	-0.98	5.48E-01	1.00E+00	0.57	6.18E-01	9.44E-01
**TRAV3**	-2.09	3.59E-01	1.00E+00	0.62	6.18E-01	9.44E-01
**RPS3AP26**	-0.20	6.13E-01	1.00E+00	-0.22	6.18E-01	9.44E-01
**KIF9-AS1**	0.46	6.09E-01	1.00E+00	0.32	6.18E-01	9.44E-01
**PTPN21**	0.15	6.47E-01	1.00E+00	-0.16	6.18E-01	9.44E-01
**LRP6**	0.16	6.13E-01	1.00E+00	-0.19	6.18E-01	9.44E-01
**TMOD3**	0.08	7.29E-01	1.00E+00	0.09	6.18E-01	9.44E-01
**FGF2**	0.56	3.96E-01	1.00E+00	-0.31	6.18E-01	9.44E-01
**KRT35**	-3.45	2.99E-01	1.00E+00	-1.58	6.18E-01	9.44E-01
**MAP7D3**	0.16	6.62E-01	1.00E+00	0.17	6.18E-01	9.44E-01
**MID2**	-0.04	9.22E-01	1.00E+00	-0.25	6.18E-01	9.44E-01
**AC092376.3**	-0.35	6.54E-01	1.00E+00	-0.63	6.18E-01	9.44E-01
**LINC02447**	0.31	6.96E-01	1.00E+00	-0.32	6.18E-01	9.44E-01
**AC068790.9**	-1.29	7.04E-01	1.00E+00	-0.68	6.18E-01	9.44E-01
**AIFM2**	1.73	1.68E-02	8.16E-01	-0.29	6.18E-01	9.44E-01
**TUBAP2**	1.02	4.13E-01	1.00E+00	-0.25	6.18E-01	9.44E-01
**AL592148.3**	0.63	3.09E-01	1.00E+00	-0.23	6.18E-01	9.44E-01
**BBS4**	-0.18	4.94E-01	1.00E+00	-0.14	6.18E-01	9.44E-01
**RBM24**	0.14	8.25E-01	1.00E+00	-0.45	6.18E-01	9.44E-01
**AL136295.5**	0.34	7.91E-01	1.00E+00	0.40	6.18E-01	9.44E-01
**AC125807.1**	0.76	6.87E-01	1.00E+00	-0.27	6.18E-01	9.44E-01
**NDUFS2**	-0.01	9.73E-01	1.00E+00	-0.10	6.19E-01	9.44E-01
**SIX5**	0.02	9.43E-01	1.00E+00	0.38	6.19E-01	9.44E-01
**KRTAP12-3**	-3.31	1.06E-01	1.00E+00	-1.15	6.19E-01	9.44E-01
**ZBTB39**	-0.04	8.52E-01	1.00E+00	0.26	6.19E-01	9.44E-01
**AC092903.2**	0.45	6.69E-01	1.00E+00	-0.45	6.19E-01	9.44E-01
**VHL**	-0.29	2.58E-01	1.00E+00	0.28	6.19E-01	9.44E-01
**AK4P1**	0.29	6.44E-01	1.00E+00	0.29	6.19E-01	9.44E-01

**GAS1**	0.53	1.13E-01	1.00E+00	0.28	6.19E-01	9.44E-01
**TM6SF1**	-0.05	9.25E-01	1.00E+00	0.23	6.19E-01	9.44E-01
**RPARP-AS1**	-0.14	7.16E-01	1.00E+00	-0.18	6.19E-01	9.44E-01
**ZNF623**	0.33	1.20E-01	1.00E+00	-0.12	6.19E-01	9.44E-01
**SMAP1**	-0.16	5.62E-01	1.00E+00	-0.11	6.19E-01	9.44E-01
**AC007292.2**	-1.06	2.66E-01	1.00E+00	-0.40	6.19E-01	9.44E-01
**DGKA**	0.07	8.45E-01	1.00E+00	-0.16	6.19E-01	9.44E-01
**GPX3**	0.80	1.60E-01	1.00E+00	-0.29	6.19E-01	9.44E-01
**MYO9A**	0.08	7.23E-01	1.00E+00	-0.10	6.19E-01	9.44E-01
**C10orf90**	-0.37	4.32E-01	1.00E+00	0.32	6.19E-01	9.44E-01
**AC023906.2**	0.52	4.29E-01	1.00E+00	0.36	6.19E-01	9.44E-01
**ZNF767P**	-0.71	1.25E-01	1.00E+00	-0.32	6.19E-01	9.44E-01
**FRRS1L**	-3.58	1.25E-02	6.96E-01	-0.60	6.19E-01	9.44E-01
**TNS1**	0.37	4.90E-01	1.00E+00	0.37	6.19E-01	9.44E-01
**SFRP5**	-0.43	5.78E-01	1.00E+00	-1.57	6.19E-01	9.44E-01
**AP005271.1**	3.23	1.43E-01	1.00E+00	0.58	6.19E-01	9.44E-01
**TMSB4Y**	-0.15	8.72E-01	1.00E+00	0.30	6.19E-01	9.44E-01
**CACHD1**	-0.03	9.38E-01	1.00E+00	-0.23	6.20E-01	9.44E-01
**ROR1**	-0.11	7.62E-01	1.00E+00	-0.22	6.20E-01	9.44E-01
**APOPT1**	0.39	1.48E-01	1.00E+00	-0.17	6.20E-01	9.44E-01
**STX16**	0.13	5.26E-01	1.00E+00	0.13	6.20E-01	9.44E-01
**TMEM131**	-0.28	1.76E-01	1.00E+00	-0.18	6.20E-01	9.44E-01
**ALG13**	-0.13	5.31E-01	1.00E+00	-0.12	6.20E-01	9.44E-01
**LRRC70**	1.74	2.06E-01	1.00E+00	-0.45	6.20E-01	9.44E-01
**KRTCAP2**	-0.65	8.31E-01	1.00E+00	0.41	6.20E-01	9.44E-01
**AL161756.1**	-0.71	8.32E-01	1.00E+00	0.67	6.20E-01	9.44E-01
**NXF1**	-0.63	2.63E-02	9.46E-01	0.12	6.20E-01	9.44E-01
**AC013275.1**	2.53	1.95E-01	1.00E+00	-0.87	6.20E-01	9.44E-01
**SUGP1**	-0.18	4.24E-01	1.00E+00	0.09	6.20E-01	9.44E-01
**XLOC_002108**	-3.10	6.28E-02	1.00E+00	0.67	6.20E-01	9.44E-01
**DYRK1B**	-0.22	4.51E-01	1.00E+00	0.22	6.20E-01	9.44E-01
**PHB**	-0.01	9.48E-01	1.00E+00	0.10	6.20E-01	9.44E-01

**ABT1**	-0.19	3.58E-01	1.00E+00	0.11	6.20E-01	9.44E-01
**ZNF793**	0.07	8.08E-01	1.00E+00	-0.22	6.20E-01	9.44E-01
**AC007878.1**	-0.35	7.51E-01	1.00E+00	0.44	6.20E-01	9.44E-01
**OSM**	-2.94	3.07E-02	1.00E+00	1.56	6.20E-01	9.44E-01
**ARFIP1**	0.11	6.69E-01	1.00E+00	-0.10	6.20E-01	9.44E-01
**RN7SL832P**	-0.48	3.83E-01	1.00E+00	0.28	6.20E-01	9.44E-01
**AP001029.2**	-1.95	5.69E-01	1.00E+00	0.66	6.20E-01	9.44E-01
**TRIM9**	-0.74	2.18E-01	1.00E+00	0.33	6.20E-01	9.44E-01
**SPEF1**	-0.22	8.27E-01	1.00E+00	-0.60	6.20E-01	9.44E-01
**MICAL3**	-0.49	2.98E-02	9.97E-01	0.37	6.20E-01	9.44E-01
**MT1A**	-1.67	1.42E-01	1.00E+00	-0.28	6.20E-01	9.44E-01
**SRSF7**	-0.10	7.07E-01	1.00E+00	0.10	6.20E-01	9.44E-01
**EFHD2**	-0.31	1.47E-01	1.00E+00	0.29	6.20E-01	9.44E-01
**JAK1**	-0.13	5.56E-01	1.00E+00	-0.13	6.21E-01	9.44E-01
**AC068768.1**	-0.33	5.23E-01	1.00E+00	0.27	6.21E-01	9.44E-01
**PA2G4P4**	-1.54	4.47E-01	1.00E+00	0.35	6.21E-01	9.44E-01
**IFRD2**	-0.23	4.47E-01	1.00E+00	0.13	6.21E-01	9.44E-01
**POF1B**	0.10	8.83E-01	1.00E+00	-0.34	6.21E-01	9.44E-01
**AC006206.2**	-2.28	4.05E-02	1.00E+00	0.63	6.21E-01	9.44E-01
**ARPC5**	0.22	1.57E-01	1.00E+00	-0.14	6.21E-01	9.44E-01
**AC098487.1**	0.65	6.32E-01	1.00E+00	-0.33	6.21E-01	9.44E-01
**BTBD2**	-0.19	4.34E-01	1.00E+00	0.21	6.21E-01	9.44E-01
**G230**	0.70	8.09E-01	1.00E+00	0.75	6.21E-01	9.44E-01
**WAC**	0.02	9.16E-01	1.00E+00	0.11	6.21E-01	9.44E-01
**RIN1**	-0.12	7.32E-01	1.00E+00	0.21	6.21E-01	9.44E-01
**UGDH**	0.34	2.51E-01	1.00E+00	-0.16	6.21E-01	9.44E-01
**GCN1**	0.21	3.96E-01	1.00E+00	0.24	6.21E-01	9.44E-01
**B3GALNT2**	0.28	4.39E-01	1.00E+00	-0.20	6.21E-01	9.44E-01
**NDUFV2-AS1**	-0.06	8.90E-01	1.00E+00	0.19	6.21E-01	9.44E-01
**AC069281.2**	-2.22	2.46E-01	1.00E+00	-0.48	6.21E-01	9.44E-01
**CD274**	-0.71	2.68E-01	1.00E+00	1.55	6.21E-01	9.44E-01
**SH3RF1**	-0.14	6.39E-01	1.00E+00	0.12	6.21E-01	9.44E-01

**AC016722.2**	3.98	2.17E-02	8.94E-01	-0.48	6.21E-01	9.44E-01
**AF117829.1**	0.66	1.80E-01	1.00E+00	0.20	6.21E-01	9.44E-01
**AL162171.2**	0.37	9.14E-01	1.00E+00	0.73	6.21E-01	9.44E-01
**CLDN1**	0.38	3.95E-01	1.00E+00	-0.28	6.21E-01	9.44E-01
**CXCR2P1**	-1.02	5.28E-01	1.00E+00	1.55	6.22E-01	9.44E-01
**G8154**	-0.38	6.85E-01	1.00E+00	-0.36	6.22E-01	9.44E-01
**ENDOU**	-0.16	7.62E-01	1.00E+00	0.31	6.22E-01	9.44E-01
**AC099550.1**	-1.58	5.52E-01	1.00E+00	-0.54	6.22E-01	9.44E-01
**AC091948.1**	1.40	2.72E-01	1.00E+00	-0.50	6.22E-01	9.44E-01
**RPL26P6**	-1.49	4.50E-01	1.00E+00	-0.36	6.22E-01	9.44E-01
**MYO1C**	0.24	4.88E-01	1.00E+00	-0.24	6.22E-01	9.44E-01
**PIK3C2G**	-0.15	6.68E-01	1.00E+00	-0.31	6.22E-01	9.44E-01
**AFF1**	-0.06	7.74E-01	1.00E+00	-0.17	6.22E-01	9.44E-01
**ZMAT3**	0.28	2.61E-01	1.00E+00	0.14	6.22E-01	9.44E-01
**ZNF726**	0.09	9.18E-01	1.00E+00	-0.30	6.22E-01	9.44E-01
**G5664**	0.75	5.75E-01	1.00E+00	0.46	6.22E-01	9.44E-01
**LINP1**	-1.48	5.25E-01	1.00E+00	0.70	6.22E-01	9.44E-01
**SARM1**	-0.24	4.11E-01	1.00E+00	0.27	6.22E-01	9.44E-01
**MED16**	-0.18	4.67E-01	1.00E+00	0.18	6.22E-01	9.44E-01
**CD70**	0.10	9.08E-01	1.00E+00	0.88	6.22E-01	9.44E-01
**CYP3A4**	0.89	1.39E-01	1.00E+00	-0.43	6.22E-01	9.44E-01
**OVCH1-AS1**	0.45	5.99E-01	1.00E+00	0.35	6.22E-01	9.44E-01
**CRYGS**	-0.24	9.21E-01	1.00E+00	0.79	6.22E-01	9.44E-01
**LDB1**	-0.37	1.97E-01	1.00E+00	0.29	6.22E-01	9.44E-01
**MAP3K4**	0.26	2.35E-01	1.00E+00	0.12	6.22E-01	9.44E-01
**CHTOP**	0.09	6.33E-01	1.00E+00	0.07	6.22E-01	9.44E-01
**LRRC27**	-0.03	9.27E-01	1.00E+00	0.19	6.22E-01	9.44E-01
**CCDC18**	-0.27	6.09E-01	1.00E+00	-0.17	6.23E-01	9.44E-01
**ADPGK-AS1**	-0.57	6.66E-01	1.00E+00	-0.39	6.23E-01	9.44E-01
**MAPK8IP3**	-0.62	6.52E-02	1.00E+00	0.40	6.23E-01	9.44E-01
**NECAP2**	-0.07	7.09E-01	1.00E+00	0.08	6.23E-01	9.44E-01
**UROS**	-0.27	1.50E-01	1.00E+00	-0.13	6.23E-01	9.44E-01

**ARHGEF39**	0.42	3.67E-01	1.00E+00	0.22	6.23E-01	9.44E-01
**CENPQ**	0.39	2.85E-01	1.00E+00	-0.13	6.23E-01	9.44E-01
**SOBP**	0.02	9.67E-01	1.00E+00	0.34	6.23E-01	9.44E-01
**AC099518.1**	-1.69	3.79E-01	1.00E+00	-0.54	6.23E-01	9.44E-01
**C7orf31**	-0.07	8.21E-01	1.00E+00	-0.20	6.23E-01	9.44E-01
**AC009119.1**	-0.43	8.25E-01	1.00E+00	0.47	6.23E-01	9.44E-01
**KDM3B**	-0.10	6.11E-01	1.00E+00	0.17	6.23E-01	9.44E-01
**SKA3**	-0.23	5.21E-01	1.00E+00	0.26	6.23E-01	9.44E-01
**ARHGAP19**	0.61	1.93E-02	8.74E-01	0.13	6.23E-01	9.45E-01
**NFKBIZ**	-2.50	1.94E-02	8.74E-01	0.28	6.23E-01	9.45E-01
**RASL10B**	1.05	1.61E-01	1.00E+00	0.56	6.23E-01	9.45E-01
**snoU109**	-0.05	9.62E-01	1.00E+00	-0.26	6.23E-01	9.45E-01
**SDF4**	-0.05	8.09E-01	1.00E+00	0.33	6.24E-01	9.45E-01
**PSG4**	1.17	3.41E-01	1.00E+00	0.63	6.24E-01	9.45E-01
**TMEM101**	0.30	2.64E-02	9.49E-01	0.10	6.24E-01	9.45E-01
**PIGN**	-0.04	8.88E-01	1.00E+00	-0.16	6.24E-01	9.45E-01
**LRRC37A2**	0.25	4.47E-01	1.00E+00	-0.15	6.24E-01	9.45E-01
**ERICH3**	0.25	8.53E-01	1.00E+00	1.56	6.24E-01	9.45E-01
**TMEM51-AS1**	0.88	9.07E-02	1.00E+00	0.41	6.24E-01	9.45E-01
**CNBP**	-0.02	9.22E-01	1.00E+00	-0.12	6.24E-01	9.45E-01
**AC090186.1**	-1.65	3.43E-01	1.00E+00	-0.49	6.24E-01	9.45E-01
**PDSS2**	-0.04	8.51E-01	1.00E+00	0.12	6.24E-01	9.45E-01
**RNASE4**	-0.13	8.29E-01	1.00E+00	-0.25	6.24E-01	9.45E-01
**FOSL2**	-0.43	2.80E-01	1.00E+00	0.23	6.24E-01	9.45E-01
**AC007683.1**	0.43	9.03E-01	1.00E+00	-0.31	6.24E-01	9.45E-01
**MFSD1**	0.41	8.62E-02	1.00E+00	-0.10	6.24E-01	9.45E-01
**CEP152**	0.19	4.97E-01	1.00E+00	-0.27	6.24E-01	9.45E-01
**AL122010.1**	-0.39	4.62E-01	1.00E+00	0.28	6.24E-01	9.45E-01
**XLOC_012288**	-0.53	6.53E-01	1.00E+00	-0.48	6.24E-01	9.45E-01
**SLC9A3R2**	0.10	8.19E-01	1.00E+00	0.27	6.24E-01	9.45E-01
**RNF8**	0.11	6.22E-01	1.00E+00	0.10	6.24E-01	9.45E-01
**SEPT7P7**	NA	NA	NA	-0.35	6.24E-01	9.45E-01

**AC004477.1**	-0.96	3.43E-01	1.00E+00	0.50	6.24E-01	9.45E-01
**CEP162**	-0.41	1.76E-01	1.00E+00	-0.21	6.25E-01	9.45E-01
**MYOM3**	-0.44	4.28E-01	1.00E+00	0.30	6.25E-01	9.45E-01
**AL050343.1**	1.44	1.05E-01	1.00E+00	-0.56	6.25E-01	9.45E-01
**PCNX4**	0.01	9.46E-01	1.00E+00	-0.10	6.25E-01	9.45E-01
**MSANTD2**	0.03	9.43E-01	1.00E+00	-0.25	6.25E-01	9.45E-01
**TMEM52B**	-2.13	4.31E-01	1.00E+00	0.59	6.25E-01	9.45E-01
**FAM32A**	0.08	7.04E-01	1.00E+00	0.08	6.25E-01	9.45E-01
**ZBTB44**	0.14	4.08E-01	1.00E+00	0.13	6.25E-01	9.45E-01
**COX10**	-0.10	6.37E-01	1.00E+00	-0.11	6.25E-01	9.45E-01
**LINC00899**	-0.51	2.11E-01	1.00E+00	0.24	6.25E-01	9.45E-01
**AC233266.2**	-1.15	7.39E-01	1.00E+00	-0.46	6.25E-01	9.45E-01
**AC096564.1**	-0.74	8.32E-01	1.00E+00	0.39	6.25E-01	9.45E-01
**DRAP1**	-0.28	3.30E-01	1.00E+00	0.13	6.25E-01	9.45E-01
**PTPRF**	0.01	9.74E-01	1.00E+00	-0.17	6.25E-01	9.45E-01
**MTMR8**	-0.62	1.69E-01	1.00E+00	-0.21	6.25E-01	9.45E-01
**ST7L**	-0.32	1.18E-01	1.00E+00	-0.14	6.25E-01	9.45E-01
**ABAT**	-0.85	1.29E-02	7.15E-01	-0.19	6.25E-01	9.45E-01
**BOD1L1**	-0.12	5.00E-01	1.00E+00	0.19	6.25E-01	9.45E-01
**AC087257.2**	-0.81	7.43E-01	1.00E+00	-0.47	6.25E-01	9.45E-01
**AC005046.1**	1.10	4.43E-01	1.00E+00	0.37	6.25E-01	9.45E-01
**NUS1**	0.05	8.25E-01	1.00E+00	0.17	6.25E-01	9.45E-01
**AASS**	-0.42	2.02E-01	1.00E+00	-0.21	6.25E-01	9.45E-01
**GATD3A**	-0.75	1.76E-01	1.00E+00	-0.43	6.26E-01	9.45E-01
**GAPDHP14**	-1.93	4.21E-01	1.00E+00	0.65	6.26E-01	9.45E-01
**FRG2DP**	-1.48	3.02E-01	1.00E+00	0.55	6.26E-01	9.45E-01
**LINC01090**	-0.23	9.24E-01	1.00E+00	0.38	6.26E-01	9.45E-01
**ALMS1-IT1**	0.85	1.72E-01	1.00E+00	0.44	6.26E-01	9.45E-01
**TXN2**	-0.11	5.78E-01	1.00E+00	-0.11	6.26E-01	9.45E-01
**MPZL3**	0.54	2.70E-01	1.00E+00	0.29	6.26E-01	9.45E-01
**PPFIA3**	-0.12	7.90E-01	1.00E+00	-0.22	6.26E-01	9.45E-01
**MFSD14A**	-0.24	7.20E-01	1.00E+00	0.27	6.26E-01	9.45E-01

**MIR4740**	0.10	9.18E-01	1.00E+00	0.71	6.26E-01	9.45E-01
**PGM5P4-AS1**	-0.05	9.35E-01	1.00E+00	-0.25	6.26E-01	9.45E-01
**ATP6V0D2**	1.40	6.82E-01	1.00E+00	1.55	6.26E-01	9.45E-01
**AC090013.1**	0.25	9.42E-01	1.00E+00	-0.38	6.26E-01	9.45E-01
**AL035078.1**	NA	NA	NA	0.36	6.26E-01	9.45E-01
**AL137058.1**	-0.37	8.38E-01	1.00E+00	-0.51	6.26E-01	9.45E-01
**ZNF211**	-0.27	4.48E-01	1.00E+00	0.15	6.26E-01	9.45E-01
**CCDC13**	-1.16	7.37E-01	1.00E+00	0.55	6.26E-01	9.45E-01
**AL591895.1**	1.15	3.15E-02	1.00E+00	0.28	6.26E-01	9.45E-01
**POC5**	0.35	1.55E-01	1.00E+00	-0.12	6.26E-01	9.45E-01
**SERTAD2**	-0.20	3.74E-01	1.00E+00	0.14	6.26E-01	9.45E-01
**AC012513.3**	0.79	8.27E-02	1.00E+00	0.37	6.26E-01	9.45E-01
**ERVK3-1**	0.21	7.20E-01	1.00E+00	0.18	6.26E-01	9.45E-01
**TMEM143**	-0.06	8.05E-01	1.00E+00	-0.15	6.26E-01	9.45E-01
**CEBPZ**	0.08	7.11E-01	1.00E+00	-0.10	6.26E-01	9.45E-01
**AC090498.1**	-0.29	2.63E-01	1.00E+00	0.37	6.26E-01	9.45E-01
**USP5**	-0.10	6.99E-01	1.00E+00	0.13	6.27E-01	9.45E-01
**PCDHGA5**	0.53	3.74E-01	1.00E+00	-0.37	6.27E-01	9.45E-01
**AC092119.3**	-1.83	3.76E-01	1.00E+00	0.35	6.27E-01	9.45E-01
**C9orf152**	-0.08	9.03E-01	1.00E+00	-0.36	6.27E-01	9.45E-01
**DLG5**	-0.10	7.13E-01	1.00E+00	0.32	6.27E-01	9.45E-01
**VSNL1**	-0.67	4.05E-02	1.00E+00	0.26	6.27E-01	9.45E-01
**G6708**	0.60	4.84E-01	1.00E+00	0.26	6.27E-01	9.45E-01
**FGF14**	0.28	6.96E-01	1.00E+00	0.28	6.27E-01	9.45E-01
**CLVS1**	0.46	3.72E-01	1.00E+00	0.28	6.27E-01	9.45E-01
**FLG-AS1**	0.21	6.59E-01	1.00E+00	-0.28	6.27E-01	9.45E-01
**PAX8-AS1**	-0.08	9.07E-01	1.00E+00	0.24	6.27E-01	9.45E-01
**PLA2G6**	-0.09	7.62E-01	1.00E+00	0.19	6.27E-01	9.45E-01
**CFAP20**	0.05	7.82E-01	1.00E+00	-0.14	6.27E-01	9.45E-01
**AC107294.3**	-2.87	1.22E-01	1.00E+00	-0.53	6.27E-01	9.45E-01
**FAM171A2**	-0.69	1.21E-01	1.00E+00	-0.58	6.27E-01	9.45E-01
**NAP1L3**	0.18	7.19E-01	1.00E+00	0.21	6.27E-01	9.45E-01

**YDJC**	-0.26	4.09E-01	1.00E+00	0.13	6.27E-01	9.45E-01
**FNIP2**	0.39	5.15E-02	1.00E+00	0.15	6.27E-01	9.45E-01
**CCDC61**	-0.24	3.57E-01	1.00E+00	-0.35	6.27E-01	9.45E-01
**RARS2**	0.09	6.72E-01	1.00E+00	-0.14	6.28E-01	9.45E-01
**XLOC_008405**	-1.76	3.38E-01	1.00E+00	0.52	6.28E-01	9.45E-01
**OSTC**	0.23	1.89E-01	1.00E+00	-0.17	6.28E-01	9.45E-01
**HYKK**	-0.07	8.64E-01	1.00E+00	-0.21	6.28E-01	9.45E-01
**COL9A3**	-0.09	7.89E-01	1.00E+00	0.22	6.28E-01	9.45E-01
**PATJ**	0.15	5.66E-01	1.00E+00	-0.16	6.28E-01	9.45E-01
**RGS11**	0.21	7.78E-01	1.00E+00	0.57	6.28E-01	9.45E-01
**DDX46**	0.06	7.87E-01	1.00E+00	0.10	6.28E-01	9.45E-01
**CDC20**	0.30	3.76E-01	1.00E+00	0.23	6.28E-01	9.45E-01
**AL353771.1**	0.56	8.73E-01	1.00E+00	0.50	6.28E-01	9.45E-01
**ZNF446**	0.05	8.55E-01	1.00E+00	0.18	6.28E-01	9.45E-01
**KRTCAP3**	-0.21	4.81E-01	1.00E+00	-0.23	6.28E-01	9.45E-01
**ZNF571**	0.03	9.47E-01	1.00E+00	-0.21	6.28E-01	9.45E-01
**AC026202.2**	-0.96	7.78E-01	1.00E+00	-0.57	6.28E-01	9.45E-01
**TCEAL6**	0.71	6.28E-01	1.00E+00	0.42	6.28E-01	9.45E-01
**YTHDC2**	0.03	9.14E-01	1.00E+00	-0.14	6.28E-01	9.45E-01
**AL031595.3**	1.49	2.72E-01	1.00E+00	-0.39	6.28E-01	9.45E-01
**AL590399.4**	-0.22	7.98E-01	1.00E+00	0.47	6.28E-01	9.45E-01
**WDR46**	-0.28	1.67E-01	1.00E+00	0.09	6.28E-01	9.45E-01
**LINC00592**	-0.30	5.64E-01	1.00E+00	-0.35	6.28E-01	9.45E-01
**LINC01546**	-0.69	3.37E-01	1.00E+00	0.38	6.28E-01	9.45E-01
**AC103736.1**	2.28	5.03E-01	1.00E+00	-0.65	6.28E-01	9.45E-01
**DNAL1**	0.18	6.23E-01	1.00E+00	-0.16	6.28E-01	9.45E-01
**MON1B**	-0.12	4.99E-01	1.00E+00	0.16	6.28E-01	9.45E-01
**CDH8**	-0.16	8.85E-01	1.00E+00	0.55	6.28E-01	9.45E-01
**MID1IP1-AS1**	0.88	3.18E-01	1.00E+00	0.60	6.28E-01	9.45E-01
**GAMT**	-0.05	8.42E-01	1.00E+00	-0.19	6.29E-01	9.45E-01
**ZNF250**	0.23	4.19E-01	1.00E+00	-0.14	6.29E-01	9.45E-01
**MSRB3**	0.28	5.72E-01	1.00E+00	-0.19	6.29E-01	9.45E-01

**AC134312.5**	-2.51	2.16E-02	8.94E-01	0.46	6.29E-01	9.45E-01
**ZNF763**	0.56	5.00E-01	1.00E+00	0.24	6.29E-01	9.45E-01
**AC023421.1**	1.20	5.15E-01	1.00E+00	-0.71	6.29E-01	9.45E-01
**ZDHHC6**	0.52	1.63E-01	1.00E+00	0.12	6.29E-01	9.45E-01
**RPS17**	-0.56	8.73E-01	1.00E+00	0.54	6.29E-01	9.45E-01
**CWC15**	-0.02	8.63E-01	1.00E+00	-0.14	6.29E-01	9.45E-01
**HBA1**	1.65	8.18E-03	5.56E-01	-1.51	6.29E-01	9.45E-01
**XLOC_009614**	0.85	4.18E-01	1.00E+00	-0.37	6.29E-01	9.45E-01
**XLOC_008473**	-0.75	2.87E-01	1.00E+00	0.44	6.29E-01	9.45E-01
**CLDN23**	0.03	9.44E-01	1.00E+00	-0.25	6.29E-01	9.45E-01
**SNAI2**	-0.41	3.09E-02	1.00E+00	0.16	6.29E-01	9.45E-01
**TCTN1**	-0.14	5.65E-01	1.00E+00	-0.18	6.29E-01	9.45E-01
**ZDHHC7**	0.12	5.64E-01	1.00E+00	0.22	6.29E-01	9.45E-01
**EMC3-AS1**	-0.47	2.15E-01	1.00E+00	0.32	6.29E-01	9.45E-01
**MAT2A**	-0.04	9.18E-01	1.00E+00	-0.22	6.29E-01	9.45E-01
**SGMS1**	-0.03	9.32E-01	1.00E+00	-0.10	6.29E-01	9.45E-01
**ATP9A**	0.57	2.35E-01	1.00E+00	0.16	6.29E-01	9.45E-01
**AC016355.1**	0.47	3.41E-01	1.00E+00	0.25	6.29E-01	9.45E-01
**GPRC5D**	0.75	3.88E-01	1.00E+00	0.53	6.29E-01	9.45E-01
**AC099689.1**	-0.86	7.99E-01	1.00E+00	0.77	6.29E-01	9.45E-01
**GTF2A1**	0.15	5.64E-01	1.00E+00	0.15	6.29E-01	9.45E-01
**ZNF850**	-0.12	7.99E-01	1.00E+00	-0.32	6.29E-01	9.45E-01
**NUDT13**	-0.21	7.15E-01	1.00E+00	-0.30	6.29E-01	9.45E-01
**MMP25-AS1**	-0.43	3.30E-01	1.00E+00	-0.27	6.29E-01	9.45E-01
**HSDL2**	0.37	1.76E-01	1.00E+00	-0.13	6.29E-01	9.45E-01
**XLOC_012057**	-1.02	3.17E-01	1.00E+00	0.62	6.29E-01	9.45E-01
**AC106782.2**	0.07	8.97E-01	1.00E+00	0.20	6.30E-01	9.45E-01
**FAM13B**	0.18	5.44E-01	1.00E+00	0.16	6.30E-01	9.45E-01
**PI4K2B**	0.16	6.18E-01	1.00E+00	-0.17	6.30E-01	9.45E-01
**ZFHX2**	-0.05	9.25E-01	1.00E+00	0.37	6.30E-01	9.45E-01
**TMEM204**	0.46	2.48E-01	1.00E+00	0.21	6.30E-01	9.45E-01
**XLOC_014050**	0.08	9.43E-01	1.00E+00	-0.52	6.30E-01	9.45E-01

**LINC00662**	0.57	9.00E-02	1.00E+00	-0.14	6.30E-01	9.45E-01
**AP000662.1**	0.80	2.34E-01	1.00E+00	0.45	6.30E-01	9.45E-01
**MAFG**	0.29	1.63E-01	1.00E+00	0.19	6.30E-01	9.45E-01
**PLEK2**	-0.41	3.23E-01	1.00E+00	-0.25	6.30E-01	9.45E-01
**MRPL54**	-0.16	5.06E-01	1.00E+00	0.09	6.30E-01	9.46E-01
**APLP1**	-1.64	2.10E-02	8.94E-01	-0.44	6.30E-01	9.46E-01
**FBXW8**	-0.16	5.27E-01	1.00E+00	0.20	6.30E-01	9.46E-01
**BAIAP2**	-0.33	3.90E-01	1.00E+00	0.16	6.30E-01	9.46E-01
**LRSAM1**	-0.26	3.74E-01	1.00E+00	0.13	6.30E-01	9.46E-01
**SIRT1**	0.30	2.27E-01	1.00E+00	-0.17	6.30E-01	9.46E-01
**RPL7AP64**	0.18	7.94E-01	1.00E+00	0.33	6.30E-01	9.46E-01
**G12735**	0.72	3.33E-01	1.00E+00	0.53	6.30E-01	9.46E-01
**PKDREJ**	-0.32	2.67E-01	1.00E+00	-0.40	6.30E-01	9.46E-01
**EIF5**	0.35	2.19E-01	1.00E+00	-0.12	6.30E-01	9.46E-01
**DCDC1**	-0.28	8.65E-01	1.00E+00	-0.52	6.30E-01	9.46E-01
**C19orf25**	-0.14	5.21E-01	1.00E+00	0.11	6.31E-01	9.46E-01
**TXNDC9**	0.08	7.26E-01	1.00E+00	-0.21	6.31E-01	9.46E-01
**B3GALT5-AS1**	-0.51	4.52E-01	1.00E+00	-1.30	6.31E-01	9.46E-01
**AC011451.1**	0.81	2.96E-01	1.00E+00	0.30	6.31E-01	9.46E-01
**MYOM1**	0.60	3.66E-01	1.00E+00	-0.38	6.31E-01	9.46E-01
**UBL7**	-0.08	7.08E-01	1.00E+00	-0.10	6.31E-01	9.46E-01
**AQR**	-0.07	7.18E-01	1.00E+00	-0.13	6.31E-01	9.46E-01
**RPL13AP6**	-3.06	1.20E-01	1.00E+00	-0.32	6.31E-01	9.46E-01
**XLOC_005482**	0.88	6.32E-01	1.00E+00	0.35	6.31E-01	9.46E-01
**RASIP1**	-0.01	9.84E-01	1.00E+00	-0.25	6.31E-01	9.46E-01
**THOP1**	-0.31	2.34E-01	1.00E+00	0.20	6.31E-01	9.46E-01
**SOX15**	-0.38	3.36E-01	1.00E+00	0.23	6.31E-01	9.46E-01
**AL354893.1**	2.56	7.72E-02	1.00E+00	-0.55	6.31E-01	9.46E-01
**XLOC_003153**	-0.16	9.17E-01	1.00E+00	-0.50	6.31E-01	9.46E-01
**MAPK8**	-0.02	9.27E-01	1.00E+00	0.11	6.31E-01	9.46E-01
**PAN2**	-0.39	8.06E-02	1.00E+00	-0.18	6.31E-01	9.46E-01
**SEC22B**	0.21	4.44E-01	1.00E+00	-0.18	6.31E-01	9.46E-01

**RAB8B**	0.34	3.17E-01	1.00E+00	0.15	6.31E-01	9.46E-01
**CWC27**	-0.02	9.30E-01	1.00E+00	-0.06	6.31E-01	9.46E-01
**LINC01816**	0.43	6.64E-01	1.00E+00	-0.33	6.31E-01	9.46E-01
**YARS**	-0.35	1.95E-01	1.00E+00	0.11	6.31E-01	9.46E-01
**RNF41**	0.00	9.91E-01	1.00E+00	0.10	6.31E-01	9.46E-01
**XLOC_014038**	-0.76	7.12E-01	1.00E+00	0.53	6.31E-01	9.46E-01
**WSB1**	0.17	5.54E-01	1.00E+00	0.11	6.31E-01	9.46E-01
**ANKRD55**	-1.94	7.43E-02	1.00E+00	0.22	6.31E-01	9.46E-01
**CENPH**	-0.35	1.29E-01	1.00E+00	-0.14	6.31E-01	9.46E-01
**GPX1P1**	1.27	1.24E-01	1.00E+00	0.29	6.32E-01	9.46E-01
**SMG7-AS1**	0.19	8.14E-01	1.00E+00	0.68	6.32E-01	9.46E-01
**KIF1A**	-1.06	1.34E-01	1.00E+00	-0.40	6.32E-01	9.46E-01
**DCST1-AS1**	-0.21	6.38E-01	1.00E+00	-0.21	6.32E-01	9.46E-01
**FAM83C-AS1**	-0.08	9.06E-01	1.00E+00	0.41	6.32E-01	9.46E-01
**KDELR3**	-0.22	6.91E-01	1.00E+00	0.21	6.32E-01	9.46E-01
**AC089984.2**	1.61	1.01E-01	1.00E+00	0.41	6.32E-01	9.46E-01
**LEAP2**	-2.53	3.34E-01	1.00E+00	-0.45	6.32E-01	9.46E-01
**KDELR1**	-0.02	9.13E-01	1.00E+00	0.11	6.32E-01	9.46E-01
**DPYS**	0.83	6.12E-01	1.00E+00	0.66	6.32E-01	9.46E-01
**SLC5A6**	0.34	4.18E-01	1.00E+00	-0.20	6.32E-01	9.46E-01
**ZFPM1**	-0.64	1.49E-01	1.00E+00	0.49	6.32E-01	9.46E-01
**PAGE5**	0.36	9.17E-01	1.00E+00	-0.65	6.32E-01	9.46E-01
**MOV10**	-0.15	6.50E-01	1.00E+00	0.19	6.32E-01	9.46E-01
**CHGB**	-3.80	1.62E-03	2.24E-01	-0.65	6.32E-01	9.46E-01
**SEC23B**	-0.13	5.24E-01	1.00E+00	-0.09	6.32E-01	9.46E-01
**LINC02015**	1.07	7.55E-01	1.00E+00	0.70	6.32E-01	9.46E-01
**ABCG2**	0.69	1.10E-01	1.00E+00	0.31	6.32E-01	9.46E-01
**IGLV3-9**	2.01	3.24E-01	1.00E+00	1.53	6.32E-01	9.46E-01
**EXOC3**	-0.08	6.41E-01	1.00E+00	0.14	6.32E-01	9.46E-01
**ACAP3**	-0.33	3.21E-01	1.00E+00	-0.33	6.32E-01	9.46E-01
**AL355490.1**	-0.41	7.48E-01	1.00E+00	0.52	6.32E-01	9.46E-01
**FTOP1**	0.84	4.79E-01	1.00E+00	-0.36	6.33E-01	9.46E-01

**HSF4**	0.32	4.15E-01	1.00E+00	0.40	6.33E-01	9.46E-01
**KIAA0232**	0.23	3.71E-01	1.00E+00	0.19	6.33E-01	9.46E-01
**MTX1P1**	0.00	1.00E+00	1.00E+00	0.35	6.33E-01	9.46E-01
**FOXCUT**	0.28	5.16E-01	1.00E+00	-0.42	6.33E-01	9.46E-01
**ZNF582**	0.31	4.49E-01	1.00E+00	-0.19	6.33E-01	9.46E-01
**WASHC2C**	-0.19	2.71E-01	1.00E+00	0.17	6.33E-01	9.46E-01
**GPR34**	1.05	1.20E-01	1.00E+00	-0.31	6.33E-01	9.46E-01
**GNAS**	-0.10	6.88E-01	1.00E+00	-0.21	6.33E-01	9.46E-01
**AC015813.1**	1.42	1.60E-01	1.00E+00	-0.46	6.33E-01	9.46E-01
**AC105760.2**	-0.38	4.37E-01	1.00E+00	-0.30	6.33E-01	9.46E-01
**LINC01170**	0.69	3.62E-01	1.00E+00	0.40	6.33E-01	9.46E-01
**AL162724.2**	-2.24	1.48E-01	1.00E+00	-0.44	6.33E-01	9.46E-01
**MTA3**	0.06	7.65E-01	1.00E+00	-0.11	6.33E-01	9.46E-01
**PCK1**	1.62	6.13E-01	1.00E+00	-1.49	6.33E-01	9.46E-01
**FAM136A**	-0.02	9.38E-01	1.00E+00	-0.11	6.33E-01	9.46E-01
**CHMP5**	0.41	1.24E-01	1.00E+00	0.10	6.33E-01	9.46E-01
**SCAF4**	-0.27	3.01E-01	1.00E+00	-0.13	6.33E-01	9.46E-01
**TBCE**	0.09	5.92E-01	1.00E+00	-0.08	6.33E-01	9.46E-01
**MFN2**	-0.07	7.14E-01	1.00E+00	-0.16	6.33E-01	9.46E-01
**PPIL1**	-0.21	3.72E-01	1.00E+00	0.10	6.34E-01	9.46E-01
**LINC00393**	-0.13	8.53E-01	1.00E+00	-0.35	6.34E-01	9.46E-01
**SDE2**	0.08	7.49E-01	1.00E+00	0.09	6.34E-01	9.46E-01
**AURKA**	0.29	4.04E-01	1.00E+00	0.25	6.34E-01	9.46E-01
**CLN5**	-0.15	4.97E-01	1.00E+00	-0.12	6.34E-01	9.46E-01
**APOC1**	1.01	9.84E-02	1.00E+00	0.33	6.34E-01	9.46E-01
**WT1**	NA	NA	NA	1.53	6.34E-01	9.46E-01
**COLEC12**	0.05	8.86E-01	1.00E+00	0.31	6.34E-01	9.46E-01
**CLCNKB**	0.32	7.34E-01	1.00E+00	-0.49	6.34E-01	9.46E-01
**XLOC_005560**	-2.54	2.15E-02	8.94E-01	-0.74	6.34E-01	9.46E-01
**C6orf226**	-0.12	7.09E-01	1.00E+00	0.13	6.34E-01	9.46E-01
**BRD4**	-0.43	1.70E-01	1.00E+00	0.16	6.34E-01	9.46E-01
**3-Mar**	0.26	5.81E-01	1.00E+00	0.17	6.34E-01	9.46E-01

**LINC00670**	-1.96	5.26E-01	1.00E+00	-0.62	6.34E-01	9.46E-01
**PROX1-AS1**	-0.53	7.42E-01	1.00E+00	0.64	6.34E-01	9.46E-01
**G39215**	0.21	7.80E-01	1.00E+00	-0.33	6.34E-01	9.46E-01
**AL353804.1**	-0.59	4.19E-01	1.00E+00	0.33	6.34E-01	9.46E-01
**LPP**	0.07	8.59E-01	1.00E+00	-0.15	6.34E-01	9.46E-01
**AL139247.1**	0.28	7.88E-01	1.00E+00	-0.35	6.34E-01	9.46E-01
**FAM162A**	-0.28	1.92E-01	1.00E+00	-0.15	6.34E-01	9.46E-01
**ESCO1**	-0.05	8.62E-01	1.00E+00	-0.15	6.34E-01	9.46E-01
**IGLV10-54**	4.15	3.81E-02	1.00E+00	1.52	6.34E-01	9.46E-01
**SPOCK1**	0.29	6.80E-01	1.00E+00	0.25	6.34E-01	9.46E-01
**C10orf111**	-0.89	7.97E-01	1.00E+00	0.38	6.34E-01	9.46E-01
**ZNF667**	0.08	8.50E-01	1.00E+00	-0.19	6.34E-01	9.46E-01
**RNU4-47P**	0.01	9.89E-01	1.00E+00	0.35	6.35E-01	9.46E-01
**OR2I1P**	-1.55	5.89E-02	1.00E+00	1.49	6.35E-01	9.46E-01
**PTPRR**	-0.88	3.59E-01	1.00E+00	0.36	6.35E-01	9.46E-01
**FOXO6**	-0.69	6.75E-02	1.00E+00	-0.40	6.35E-01	9.46E-01
**EPHX3**	-0.11	8.32E-01	1.00E+00	0.26	6.35E-01	9.46E-01
**ATIC**	-0.13	6.39E-01	1.00E+00	-0.10	6.35E-01	9.46E-01
**AC145098.2**	-2.33	6.40E-02	1.00E+00	-0.57	6.35E-01	9.46E-01
**G38688**	-1.08	5.40E-01	1.00E+00	0.46	6.35E-01	9.46E-01
**TBCA**	0.21	1.75E-01	1.00E+00	-0.07	6.35E-01	9.46E-01
**RPL5P4**	1.10	4.85E-01	1.00E+00	-0.33	6.35E-01	9.46E-01
**ACSBG2**	-1.83	2.59E-01	1.00E+00	-0.59	6.35E-01	9.46E-01
**AC087071.2**	-0.29	9.01E-01	1.00E+00	-0.45	6.35E-01	9.46E-01
**ACTG1P9**	-1.37	6.93E-01	1.00E+00	0.59	6.35E-01	9.46E-01
**MORN4**	-0.04	8.92E-01	1.00E+00	0.15	6.35E-01	9.46E-01
**NBL1**	0.11	6.68E-01	1.00E+00	0.25	6.35E-01	9.46E-01
**EPHB4**	-0.24	2.42E-01	1.00E+00	0.23	6.35E-01	9.46E-01
**ANXA2P1**	-1.10	7.18E-01	1.00E+00	-0.28	6.35E-01	9.46E-01
**GTPBP8**	-0.14	5.94E-01	1.00E+00	-0.11	6.35E-01	9.46E-01
**NUTF2**	-0.02	8.85E-01	1.00E+00	0.06	6.35E-01	9.46E-01
**AGR2**	0.62	1.92E-01	1.00E+00	-0.31	6.35E-01	9.46E-01

**MXD3**	0.21	6.54E-01	1.00E+00	0.15	6.35E-01	9.46E-01
**AL035258.1**	-3.19	1.00E-02	6.22E-01	0.52	6.36E-01	9.46E-01
**AATBC**	-1.25	8.71E-03	5.76E-01	-0.33	6.36E-01	9.46E-01
**GGCX**	0.05	8.06E-01	1.00E+00	-0.10	6.36E-01	9.46E-01
**RBM12B-AS1**	1.08	4.02E-01	1.00E+00	0.57	6.36E-01	9.46E-01
**LINC01232**	-0.17	7.54E-01	1.00E+00	0.39	6.36E-01	9.46E-01
**XLOC_003514**	-1.11	3.60E-01	1.00E+00	1.50	6.36E-01	9.46E-01
**KIF2A**	-0.25	2.48E-01	1.00E+00	-0.08	6.36E-01	9.46E-01
**AC093627.5**	-0.44	3.31E-01	1.00E+00	-0.28	6.36E-01	9.46E-01
**CDKN1A**	-1.18	4.31E-02	1.00E+00	0.17	6.36E-01	9.46E-01
**YEATS4**	-0.09	7.23E-01	1.00E+00	-0.15	6.36E-01	9.46E-01
**ISG20L2**	-0.11	5.87E-01	1.00E+00	0.13	6.36E-01	9.46E-01
**HDAC8**	0.21	5.28E-01	1.00E+00	-0.15	6.36E-01	9.46E-01
**BBS12**	0.37	2.27E-01	1.00E+00	0.22	6.36E-01	9.46E-01
**WARS2**	0.15	6.16E-01	1.00E+00	0.11	6.36E-01	9.46E-01
**E2F2**	0.11	6.41E-01	1.00E+00	0.23	6.36E-01	9.46E-01
**SIAH2-AS1**	2.34	7.92E-02	1.00E+00	0.46	6.36E-01	9.46E-01
**EGR4**	-3.43	1.97E-02	8.79E-01	0.86	6.36E-01	9.46E-01
**LYPLA2P1**	-0.05	9.88E-01	1.00E+00	0.27	6.36E-01	9.46E-01
**WDR63**	-0.08	8.46E-01	1.00E+00	0.28	6.36E-01	9.46E-01
**AC107027.1**	1.50	6.62E-01	1.00E+00	0.95	6.36E-01	9.46E-01
**CCDC92**	0.03	9.53E-01	1.00E+00	-0.27	6.36E-01	9.46E-01
**C2orf88**	0.34	5.55E-01	1.00E+00	-0.15	6.36E-01	9.46E-01
**KRTAP17-1**	-5.57	8.91E-02	1.00E+00	-1.51	6.36E-01	9.46E-01
**CLIC2**	-0.24	4.28E-01	1.00E+00	0.15	6.36E-01	9.46E-01
**TMEM236**	-3.00	9.18E-02	1.00E+00	-0.64	6.36E-01	9.46E-01
**SDCBP**	0.37	2.35E-01	1.00E+00	-0.17	6.36E-01	9.46E-01
**PLEKHF2**	0.15	5.69E-01	1.00E+00	-0.17	6.36E-01	9.46E-01
**PDK1**	-0.20	5.69E-01	1.00E+00	-0.16	6.36E-01	9.46E-01
**OR14L1P**	-1.01	7.18E-01	1.00E+00	-1.10	6.36E-01	9.46E-01
**CD300LB**	0.84	3.60E-01	1.00E+00	0.45	6.36E-01	9.46E-01
**L1CAM**	-0.31	2.51E-01	1.00E+00	0.33	6.36E-01	9.46E-01

**C2orf42**	0.00	9.88E-01	1.00E+00	-0.13	6.37E-01	9.46E-01
**RDH11**	0.04	8.59E-01	1.00E+00	-0.13	6.37E-01	9.46E-01
**ZNF559**	0.20	5.24E-01	1.00E+00	-0.16	6.37E-01	9.46E-01
**ESAM**	-0.28	5.27E-01	1.00E+00	-0.24	6.37E-01	9.46E-01
**EXD2**	0.09	7.13E-01	1.00E+00	0.12	6.37E-01	9.46E-01
**SLC25A53**	0.12	7.47E-01	1.00E+00	0.14	6.37E-01	9.46E-01
**PTMS**	-0.26	2.59E-01	1.00E+00	-0.29	6.37E-01	9.46E-01
**ZSWIM1**	-0.36	2.11E-01	1.00E+00	-0.20	6.37E-01	9.46E-01
**MRPL36**	0.11	6.59E-01	1.00E+00	-0.17	6.37E-01	9.46E-01
**DEPDC1**	0.10	8.34E-01	1.00E+00	-0.27	6.37E-01	9.46E-01
**G1920**	2.68	1.92E-02	8.71E-01	-0.62	6.37E-01	9.46E-01
**QRSL1P3**	NA	NA	NA	-0.44	6.37E-01	9.46E-01
**NPRL3**	0.05	8.65E-01	1.00E+00	0.12	6.37E-01	9.46E-01
**LINC02147**	-1.80	2.82E-01	1.00E+00	0.57	6.37E-01	9.46E-01
**MBOAT2**	0.09	8.27E-01	1.00E+00	-0.20	6.37E-01	9.46E-01
**IL7**	0.11	8.14E-01	1.00E+00	0.18	6.37E-01	9.46E-01
**PHGDH**	-0.31	3.56E-01	1.00E+00	-0.14	6.37E-01	9.46E-01
**SPINK6**	-2.82	1.17E-01	1.00E+00	1.48	6.37E-01	9.46E-01
**AC104695.3**	-4.33	1.86E-02	8.56E-01	0.30	6.37E-01	9.46E-01
**SPINT1**	-0.19	5.12E-01	1.00E+00	-0.20	6.37E-01	9.46E-01
**LINC01238**	-0.77	4.98E-01	1.00E+00	-0.51	6.37E-01	9.46E-01
**ZEB1**	0.34	4.90E-01	1.00E+00	-0.20	6.37E-01	9.46E-01
**ADAMTSL5**	0.37	5.39E-01	1.00E+00	0.28	6.37E-01	9.46E-01
**RBM4B**	0.27	4.64E-01	1.00E+00	-0.13	6.37E-01	9.46E-01
**AC121761.2**	-0.57	5.83E-01	1.00E+00	-0.40	6.38E-01	9.47E-01
**ELAC2**	-0.11	5.71E-01	1.00E+00	-0.07	6.38E-01	9.47E-01
**AC123595.1**	-0.64	6.40E-01	1.00E+00	0.48	6.38E-01	9.47E-01
**LPAR2**	0.15	6.66E-01	1.00E+00	0.18	6.38E-01	9.47E-01
**CCDC28A**	0.25	3.95E-01	1.00E+00	-0.14	6.38E-01	9.47E-01
**STON1**	0.20	5.95E-01	1.00E+00	0.27	6.38E-01	9.47E-01
**PPIL3**	-0.14	6.78E-01	1.00E+00	-0.13	6.38E-01	9.47E-01
**G9812**	-1.70	2.76E-01	1.00E+00	0.64	6.38E-01	9.47E-01

**XLOC_013825**	2.22	4.96E-01	1.00E+00	0.76	6.38E-01	9.47E-01
**BCL11A**	-0.18	5.61E-01	1.00E+00	-0.28	6.38E-01	9.47E-01
**POLRMT**	-0.50	7.19E-02	1.00E+00	-0.29	6.38E-01	9.47E-01
**AC007611.1**	0.30	6.80E-01	1.00E+00	-0.38	6.38E-01	9.47E-01
**RAB11FIP2**	0.37	2.13E-01	1.00E+00	0.09	6.38E-01	9.47E-01
**FABP9**	-3.65	2.76E-01	1.00E+00	-1.49	6.38E-01	9.47E-01
**AL161719.1**	-1.05	4.32E-01	1.00E+00	0.53	6.38E-01	9.47E-01
**ZNF684**	-0.01	9.75E-01	1.00E+00	0.12	6.38E-01	9.47E-01
**AC112907.2**	-0.41	6.78E-01	1.00E+00	0.44	6.38E-01	9.47E-01
**ZNF175**	0.29	4.50E-01	1.00E+00	0.21	6.38E-01	9.47E-01
**SMCHD1**	0.24	3.76E-01	1.00E+00	0.22	6.38E-01	9.47E-01
**GVQW2**	-3.00	1.01E-01	1.00E+00	0.48	6.38E-01	9.47E-01
**GLIDR**	0.59	1.47E-01	1.00E+00	0.22	6.38E-01	9.47E-01
**ZNF544**	0.12	6.95E-01	1.00E+00	-0.12	6.39E-01	9.47E-01
**GABRR1**	-1.44	2.05E-01	1.00E+00	-1.19	6.39E-01	9.47E-01
**INPP4A**	-0.01	9.72E-01	1.00E+00	0.24	6.39E-01	9.47E-01
**AC127537.1**	2.35	1.90E-02	8.67E-01	0.63	6.39E-01	9.47E-01
**AARS2**	-0.12	5.34E-01	1.00E+00	0.25	6.39E-01	9.47E-01
**LYPLA1P3**	-0.93	7.86E-01	1.00E+00	-0.26	6.39E-01	9.47E-01
**ORC3**	0.11	5.34E-01	1.00E+00	-0.13	6.39E-01	9.47E-01
**AC092745.2**	0.65	3.75E-01	1.00E+00	-0.42	6.39E-01	9.47E-01
**KRTAP4-7**	-30.00	6.80E-18	5.56E-15	-1.50	6.39E-01	9.47E-01
**C2orf16**	0.47	4.57E-01	1.00E+00	0.39	6.39E-01	9.47E-01
**PPARA**	0.43	1.90E-01	1.00E+00	0.15	6.39E-01	9.47E-01
**AMOT**	0.23	5.53E-01	1.00E+00	-0.25	6.39E-01	9.47E-01
**AC092117.2**	0.39	7.72E-01	1.00E+00	-0.61	6.39E-01	9.47E-01
**PNN**	-0.31	1.50E-01	1.00E+00	0.11	6.39E-01	9.47E-01
**IL12RB2**	-0.87	7.39E-02	1.00E+00	0.32	6.39E-01	9.47E-01
**HAX1**	-0.01	9.51E-01	1.00E+00	-0.08	6.39E-01	9.47E-01
**HENMT1**	0.21	4.95E-01	1.00E+00	-0.16	6.39E-01	9.47E-01
**FBXO8**	0.42	6.44E-02	1.00E+00	-0.11	6.40E-01	9.47E-01
**WIPF3**	-0.23	5.71E-01	1.00E+00	0.20	6.40E-01	9.47E-01

**AHSP**	-1.45	6.70E-01	1.00E+00	-0.52	6.40E-01	9.47E-01
**TRDN**	1.94	1.18E-01	1.00E+00	-1.48	6.40E-01	9.47E-01
**KRTAP16-1**	-5.34	1.03E-01	1.00E+00	-1.50	6.40E-01	9.47E-01
**AC079035.1**	0.80	8.15E-01	1.00E+00	-0.48	6.40E-01	9.47E-01
**TTC30B**	0.35	1.57E-01	1.00E+00	-0.16	6.40E-01	9.47E-01
**MPP3**	-0.24	6.44E-01	1.00E+00	-0.30	6.40E-01	9.47E-01
**THOC1**	0.18	5.29E-01	1.00E+00	-0.12	6.40E-01	9.47E-01
**ASCC3**	-0.14	5.86E-01	1.00E+00	-0.15	6.40E-01	9.47E-01
**HMGCL**	0.31	2.13E-01	1.00E+00	-0.10	6.40E-01	9.47E-01
**AL139300.2**	-1.13	4.42E-01	1.00E+00	0.48	6.40E-01	9.47E-01
**SLC39A1**	0.02	9.92E-01	1.00E+00	0.26	6.40E-01	9.47E-01
**SLCO3A1**	0.17	3.39E-01	1.00E+00	-0.13	6.40E-01	9.47E-01
**EXTL2**	0.31	2.75E-01	1.00E+00	0.12	6.40E-01	9.47E-01
**IGLV4-69**	2.11	5.31E-01	1.00E+00	1.03	6.40E-01	9.47E-01
**PSMA1**	0.49	2.36E-01	1.00E+00	-0.23	6.40E-01	9.47E-01
**HIGD1B**	-0.28	6.76E-01	1.00E+00	0.23	6.40E-01	9.47E-01
**AP005209.1**	-3.07	5.90E-02	1.00E+00	-0.57	6.40E-01	9.47E-01
**IL1RL1**	0.61	3.27E-01	1.00E+00	0.42	6.40E-01	9.47E-01
**USP42**	-0.27	3.33E-01	1.00E+00	-0.21	6.40E-01	9.47E-01
**COLCA2**	0.28	7.60E-01	1.00E+00	0.31	6.40E-01	9.47E-01
**UHRF2**	0.47	2.54E-01	1.00E+00	0.29	6.40E-01	9.47E-01
**NFATC2**	-0.41	7.68E-02	1.00E+00	0.18	6.41E-01	9.47E-01
**RPL15P2**	-0.46	7.70E-01	1.00E+00	-0.25	6.41E-01	9.47E-01
**LRRC56**	-0.25	5.49E-01	1.00E+00	0.43	6.41E-01	9.47E-01
**SPTA1**	-0.88	6.34E-01	1.00E+00	-0.80	6.41E-01	9.47E-01
**CUL4A**	0.17	5.13E-01	1.00E+00	-0.08	6.41E-01	9.47E-01
**FAM198A**	0.75	1.69E-01	1.00E+00	0.32	6.41E-01	9.47E-01
**AC099522.2**	-0.12	8.45E-01	1.00E+00	-0.28	6.41E-01	9.47E-01
**AL807742.1**	1.03	6.23E-01	1.00E+00	-0.76	6.41E-01	9.47E-01
**NOMO1**	0.05	8.57E-01	1.00E+00	0.25	6.41E-01	9.47E-01
**ZNF436-AS1**	0.72	1.81E-01	1.00E+00	-0.22	6.41E-01	9.47E-01
**CASP14**	0.01	9.81E-01	1.00E+00	0.35	6.41E-01	9.47E-01

**ITGB4**	-0.37	1.26E-01	1.00E+00	0.27	6.41E-01	9.47E-01
**ATP2C2**	0.18	7.33E-01	1.00E+00	0.28	6.41E-01	9.47E-01
**OAZ2**	-0.08	6.44E-01	1.00E+00	-0.08	6.41E-01	9.47E-01
**G5264**	-0.77	1.84E-01	1.00E+00	0.30	6.41E-01	9.47E-01
**XLOC_009577**	-2.08	2.28E-01	1.00E+00	1.07	6.41E-01	9.47E-01
**CAPRIN2**	-0.07	7.67E-01	1.00E+00	-0.17	6.41E-01	9.47E-01
**ATP2B4**	-0.02	9.10E-01	1.00E+00	-0.10	6.41E-01	9.47E-01
**AL132639.2**	-0.01	9.91E-01	1.00E+00	-0.27	6.41E-01	9.47E-01
**AC007785.1**	0.44	6.37E-01	1.00E+00	-0.38	6.41E-01	9.47E-01
**SELENOP**	0.41	1.04E-01	1.00E+00	0.17	6.41E-01	9.47E-01
**ANXA2**	0.13	5.40E-01	1.00E+00	-0.11	6.41E-01	9.47E-01
**MAP3K20**	0.31	2.89E-01	1.00E+00	-0.13	6.41E-01	9.47E-01
**JADE3**	0.21	3.98E-01	1.00E+00	0.21	6.41E-01	9.47E-01
**LRRC75B**	-0.55	1.59E-01	1.00E+00	-0.24	6.41E-01	9.47E-01
**AL353759.1**	-0.27	8.40E-01	1.00E+00	-0.42	6.41E-01	9.47E-01
**XLOC_005449**	1.18	3.21E-01	1.00E+00	-0.48	6.42E-01	9.47E-01
**AL136441.1**	-0.85	4.84E-01	1.00E+00	-0.50	6.42E-01	9.47E-01
**APOLD1**	-0.45	5.32E-01	1.00E+00	-0.33	6.42E-01	9.47E-01
**AC018804.1**	0.15	9.11E-01	1.00E+00	-0.36	6.42E-01	9.47E-01
**RPL3**	-0.31	3.74E-01	1.00E+00	0.15	6.42E-01	9.47E-01
**HAL**	-1.40	9.08E-02	1.00E+00	0.33	6.42E-01	9.47E-01
**BAZ1A**	-0.06	8.35E-01	1.00E+00	0.17	6.42E-01	9.47E-01
**PUS3**	0.20	2.85E-01	1.00E+00	0.09	6.42E-01	9.47E-01
**GRHL3**	0.17	7.66E-01	1.00E+00	0.25	6.42E-01	9.47E-01
**TANC1**	0.16	6.35E-01	1.00E+00	0.14	6.42E-01	9.47E-01
**REX1BD**	-0.22	4.04E-01	1.00E+00	0.12	6.42E-01	9.47E-01
**AC083900.1**	-2.75	4.16E-01	1.00E+00	-0.44	6.42E-01	9.47E-01
**AC091729.1**	-0.38	4.37E-01	1.00E+00	-0.36	6.42E-01	9.47E-01
**AL035425.3**	0.08	9.36E-01	1.00E+00	1.25	6.42E-01	9.47E-01
**SAFB**	-0.05	8.06E-01	1.00E+00	0.13	6.42E-01	9.47E-01
**NUP210**	-0.38	1.74E-01	1.00E+00	0.37	6.42E-01	9.47E-01
**SNORA73B**	0.17	9.28E-01	1.00E+00	-0.55	6.42E-01	9.47E-01

**FAM66D**	-0.78	3.51E-01	1.00E+00	-0.44	6.42E-01	9.47E-01
**SPATA18**	0.04	8.83E-01	1.00E+00	0.18	6.42E-01	9.47E-01
**UNC13B**	0.15	4.03E-01	1.00E+00	0.17	6.42E-01	9.47E-01
**SLC35B3**	0.31	2.06E-01	1.00E+00	-0.12	6.42E-01	9.47E-01
**XYLB**	-0.31	3.78E-01	1.00E+00	-0.17	6.42E-01	9.47E-01
**TRMT61B**	-0.14	5.16E-01	1.00E+00	0.12	6.42E-01	9.47E-01
**DUSP5**	-0.35	5.03E-01	1.00E+00	0.27	6.43E-01	9.47E-01
**PIGO**	-0.02	8.73E-01	1.00E+00	-0.18	6.43E-01	9.47E-01
**SH3PXD2A-AS1**	-0.52	2.99E-01	1.00E+00	0.27	6.43E-01	9.47E-01
**LRRC37A6P**	-0.14	8.50E-01	1.00E+00	-0.51	6.43E-01	9.47E-01
**GLRX3**	-0.10	6.51E-01	1.00E+00	-0.12	6.43E-01	9.47E-01
**RPL21P39**	1.57	6.33E-01	1.00E+00	-0.36	6.43E-01	9.47E-01
**AC080112.1**	-0.50	9.57E-02	1.00E+00	0.31	6.43E-01	9.47E-01
**CSRNP3**	-0.15	6.33E-01	1.00E+00	-0.19	6.43E-01	9.47E-01
**BFSP1**	-1.11	2.48E-03	2.54E-01	0.32	6.43E-01	9.47E-01
**NUP58**	0.23	4.99E-01	1.00E+00	0.14	6.43E-01	9.47E-01
**GLT8D1**	-0.07	7.75E-01	1.00E+00	-0.10	6.43E-01	9.47E-01
**MLLT3**	-0.05	8.72E-01	1.00E+00	-0.08	6.43E-01	9.47E-01
**KRT27**	-8.64	1.39E-07	5.62E-05	-1.45	6.43E-01	9.47E-01
**XLOC_005194**	4.12	1.35E-01	1.00E+00	0.91	6.43E-01	9.47E-01
**CPEB1**	0.54	5.96E-01	1.00E+00	0.39	6.43E-01	9.47E-01
**MAPK6-DT**	-1.76	7.66E-02	1.00E+00	-0.46	6.43E-01	9.47E-01
**RAVER1**	-0.45	3.26E-02	1.00E+00	0.41	6.43E-01	9.47E-01
**MED31**	0.41	3.18E-01	1.00E+00	-0.21	6.43E-01	9.47E-01
**RP11-292F9.1**	-0.14	9.20E-01	1.00E+00	0.43	6.43E-01	9.47E-01
**AC021054.1**	-0.20	5.45E-01	1.00E+00	-0.12	6.43E-01	9.47E-01
**PGLYRP3**	-0.12	7.63E-01	1.00E+00	0.30	6.43E-01	9.47E-01
**H1FX**	-0.94	1.63E-05	4.93E-03	-0.35	6.43E-01	9.47E-01
**TRIT1**	0.26	3.77E-01	1.00E+00	0.11	6.43E-01	9.47E-01
**FYTTD1**	0.04	8.73E-01	1.00E+00	-0.10	6.43E-01	9.47E-01
**AC131571.1**	-1.96	3.34E-01	1.00E+00	-0.44	6.43E-01	9.47E-01
**RPL9P7**	-0.47	4.84E-01	1.00E+00	0.42	6.43E-01	9.47E-01

**PSMD6-AS2**	0.28	7.59E-01	1.00E+00	-0.30	6.43E-01	9.47E-01
**AL121832.2**	-1.22	2.03E-02	8.88E-01	0.25	6.44E-01	9.47E-01
**SSTR3**	-0.68	7.19E-01	1.00E+00	1.48	6.44E-01	9.47E-01
**SNX32**	-1.06	3.56E-02	1.00E+00	0.24	6.44E-01	9.47E-01
**SCN5A**	-0.50	2.84E-01	1.00E+00	-0.48	6.44E-01	9.47E-01
**TSFM**	-0.05	8.21E-01	1.00E+00	0.12	6.44E-01	9.47E-01
**AL139082.1**	-1.23	4.44E-01	1.00E+00	-0.70	6.44E-01	9.47E-01
**SBNO1**	0.26	3.29E-01	1.00E+00	-0.14	6.44E-01	9.47E-01
**SBF2-AS1**	-0.49	7.62E-02	1.00E+00	-0.20	6.44E-01	9.47E-01
**DGKQ**	0.13	6.36E-01	1.00E+00	0.26	6.44E-01	9.47E-01
**DGCR2**	0.03	9.16E-01	1.00E+00	-0.24	6.44E-01	9.47E-01
**CIB1**	-0.07	7.57E-01	1.00E+00	-0.11	6.44E-01	9.47E-01
**LINC00028**	-1.65	6.30E-01	1.00E+00	0.52	6.44E-01	9.47E-01
**VAT1**	0.05	7.94E-01	1.00E+00	0.14	6.44E-01	9.47E-01
**MEF2C**	0.05	9.19E-01	1.00E+00	0.22	6.44E-01	9.47E-01
**MIS12**	0.34	1.55E-01	1.00E+00	-0.12	6.44E-01	9.47E-01
**APPBP2**	0.11	5.46E-01	1.00E+00	-0.10	6.44E-01	9.47E-01
**MBOAT7**	-0.37	8.25E-02	1.00E+00	0.20	6.44E-01	9.47E-01
**AL031666.2**	1.02	6.72E-01	1.00E+00	0.73	6.44E-01	9.47E-01
**LINC00960**	-0.51	8.74E-01	1.00E+00	-0.39	6.44E-01	9.47E-01
**RANBP17**	0.64	5.87E-01	1.00E+00	0.36	6.44E-01	9.47E-01
**CPA3**	0.77	1.76E-01	1.00E+00	0.31	6.44E-01	9.47E-01
**SPAG5-AS1**	1.80	3.14E-01	1.00E+00	0.47	6.44E-01	9.47E-01
**AP003498.2**	0.02	9.63E-01	1.00E+00	-0.28	6.44E-01	9.47E-01
**KTN1**	-0.21	5.35E-01	1.00E+00	-0.13	6.44E-01	9.47E-01
**AL359265.3**	0.62	4.33E-01	1.00E+00	0.36	6.44E-01	9.47E-01
**AC027329.1**	1.33	3.03E-01	1.00E+00	-0.52	6.44E-01	9.47E-01
**TCAP**	-1.13	3.44E-01	1.00E+00	0.41	6.44E-01	9.47E-01
**LINGO1**	-0.32	5.74E-01	1.00E+00	0.38	6.44E-01	9.47E-01
**EIF4A1P2**	0.70	7.54E-01	1.00E+00	0.34	6.44E-01	9.47E-01
**TRAPPC9**	-0.18	5.27E-01	1.00E+00	0.20	6.44E-01	9.47E-01
**ZNF407**	-0.43	4.16E-02	1.00E+00	0.17	6.44E-01	9.47E-01

**FER**	-0.10	5.39E-01	1.00E+00	-0.10	6.44E-01	9.47E-01
**SARS2**	-1.00	5.22E-01	1.00E+00	-0.23	6.44E-01	9.47E-01
**G16268**	-0.14	8.23E-01	1.00E+00	-0.39	6.44E-01	9.47E-01
**AC064799.2**	-1.24	7.19E-01	1.00E+00	0.54	6.45E-01	9.47E-01
**APBA3**	-0.17	6.48E-01	1.00E+00	-0.18	6.45E-01	9.47E-01
**CD55**	0.39	2.02E-01	1.00E+00	-0.13	6.45E-01	9.47E-01
**RPL41P1**	-0.04	8.69E-01	1.00E+00	0.23	6.45E-01	9.47E-01
**FBXO16**	0.44	6.75E-01	1.00E+00	0.35	6.45E-01	9.47E-01
**WDR48**	0.24	2.00E-01	1.00E+00	-0.06	6.45E-01	9.47E-01
**C1orf210**	-0.13	7.81E-01	1.00E+00	-0.27	6.45E-01	9.47E-01
**MED4**	0.08	6.89E-01	1.00E+00	-0.18	6.45E-01	9.47E-01
**HKDC1**	-1.37	2.17E-01	1.00E+00	0.65	6.45E-01	9.47E-01
**ARMCX6**	0.18	4.29E-01	1.00E+00	-0.10	6.45E-01	9.47E-01
**SENP1**	0.06	7.76E-01	1.00E+00	-0.22	6.45E-01	9.47E-01
**AC093512.2**	0.50	5.19E-01	1.00E+00	-0.28	6.45E-01	9.47E-01
**PRRG1**	-0.10	7.77E-01	1.00E+00	0.18	6.45E-01	9.47E-01
**DOCK7**	0.21	3.85E-01	1.00E+00	-0.13	6.45E-01	9.47E-01
**AC055839.1**	-2.65	1.82E-01	1.00E+00	0.58	6.45E-01	9.47E-01
**FITM1**	0.79	2.39E-01	1.00E+00	-0.31	6.45E-01	9.47E-01
**TSPYL2**	-0.16	4.63E-01	1.00E+00	0.10	6.45E-01	9.47E-01
**EBLN2**	1.39	1.59E-01	1.00E+00	-0.50	6.45E-01	9.47E-01
**G3030**	-1.64	2.94E-01	1.00E+00	0.50	6.45E-01	9.47E-01
**KLHDC8A**	0.20	7.95E-01	1.00E+00	0.44	6.45E-01	9.47E-01
**AC005070.3**	1.47	7.43E-02	1.00E+00	-0.29	6.45E-01	9.47E-01
**AC073046.1**	-0.66	1.84E-01	1.00E+00	-0.47	6.45E-01	9.47E-01
**OSTM1**	0.25	4.54E-01	1.00E+00	-0.13	6.45E-01	9.47E-01
**CLTC**	0.09	6.25E-01	1.00E+00	0.10	6.45E-01	9.47E-01
**SYF2**	0.08	6.28E-01	1.00E+00	-0.13	6.45E-01	9.47E-01
**AC068189.1**	0.12	9.39E-01	1.00E+00	0.44	6.45E-01	9.47E-01
**FAM171B**	-0.45	1.70E-01	1.00E+00	0.20	6.45E-01	9.47E-01
**AC090114.2**	-0.75	4.01E-02	1.00E+00	0.27	6.45E-01	9.47E-01
**CALCRL**	0.28	5.43E-01	1.00E+00	-0.18	6.45E-01	9.47E-01

**LINC01006**	-0.33	6.01E-01	1.00E+00	-0.23	6.46E-01	9.47E-01
**XLOC_001447**	-0.87	5.65E-01	1.00E+00	0.35	6.46E-01	9.47E-01
**LINC01160**	-0.82	5.80E-01	1.00E+00	0.44	6.46E-01	9.47E-01
**C16orf91**	-0.10	6.49E-01	1.00E+00	-0.13	6.46E-01	9.47E-01
**AC138207.2**	-0.69	6.50E-01	1.00E+00	0.39	6.46E-01	9.47E-01
**IMPDH1P5**	-1.14	2.66E-01	1.00E+00	0.58	6.46E-01	9.47E-01
**PYGL**	0.38	3.54E-01	1.00E+00	-0.17	6.46E-01	9.47E-01
**PAPSS1**	0.17	3.70E-01	1.00E+00	0.06	6.46E-01	9.47E-01
**AC055822.1**	-2.39	1.04E-01	1.00E+00	-0.43	6.46E-01	9.47E-01
**GCNT2**	0.41	3.43E-01	1.00E+00	-0.21	6.46E-01	9.47E-01
**AC121247.1**	-0.79	4.70E-01	1.00E+00	0.48	6.46E-01	9.47E-01
**PLAC9P1**	0.17	8.98E-01	1.00E+00	0.76	6.46E-01	9.47E-01
**CHRNA6**	1.34	6.98E-01	1.00E+00	0.74	6.46E-01	9.47E-01
**FSD2**	-2.94	1.98E-01	1.00E+00	0.46	6.46E-01	9.47E-01
**RPL13P12**	-1.05	7.42E-01	1.00E+00	0.31	6.46E-01	9.47E-01
**AC147067.1**	-0.20	6.74E-01	1.00E+00	0.19	6.46E-01	9.47E-01
**AL138976.2**	0.11	8.35E-01	1.00E+00	0.30	6.46E-01	9.47E-01
**AL591479.1**	0.13	7.11E-01	1.00E+00	0.29	6.46E-01	9.47E-01
**LEO1**	-0.07	8.29E-01	1.00E+00	-0.15	6.46E-01	9.47E-01
**XLOC_013420**	0.38	3.91E-01	1.00E+00	-0.20	6.46E-01	9.47E-01
**RWDD4P1**	-1.07	6.52E-01	1.00E+00	-0.25	6.46E-01	9.47E-01
**ZFP42**	4.06	7.92E-03	5.47E-01	1.47	6.46E-01	9.47E-01
**PIGZ**	-0.15	6.55E-01	1.00E+00	-0.26	6.46E-01	9.47E-01
**AL592114.1**	0.00	9.99E-01	1.00E+00	-0.35	6.46E-01	9.47E-01
**RPS19**	-0.25	3.76E-01	1.00E+00	0.17	6.46E-01	9.47E-01
**LSM11**	0.51	1.04E-01	1.00E+00	0.28	6.46E-01	9.47E-01
**HAPLN1**	0.41	7.34E-01	1.00E+00	0.44	6.46E-01	9.47E-01
**TBC1D10C**	0.03	9.62E-01	1.00E+00	1.19	6.46E-01	9.47E-01
**HAUS5**	-0.46	7.16E-02	1.00E+00	0.09	6.47E-01	9.47E-01
**G36779**	0.32	6.49E-01	1.00E+00	0.24	6.47E-01	9.47E-01
**AC073648.3**	-0.51	8.17E-01	1.00E+00	-0.64	6.47E-01	9.47E-01
**RPL17**	0.04	9.48E-01	1.00E+00	0.29	6.47E-01	9.47E-01

**STUB1**	-0.01	9.75E-01	1.00E+00	-0.28	6.47E-01	9.47E-01
**SPCS3**	0.08	7.56E-01	1.00E+00	0.11	6.47E-01	9.47E-01
**ADH5**	0.30	3.43E-01	1.00E+00	-0.14	6.47E-01	9.47E-01
**XLOC_000610**	-0.08	8.46E-01	1.00E+00	-0.26	6.47E-01	9.47E-01
**NFKB1**	-0.05	7.86E-01	1.00E+00	-0.20	6.47E-01	9.47E-01
**AGGF1**	-0.07	7.63E-01	1.00E+00	-0.11	6.47E-01	9.47E-01
**SUGT1P4-STRA6LP**	-0.41	7.78E-01	1.00E+00	0.59	6.47E-01	9.47E-01
**AC114489.1**	-0.58	8.67E-01	1.00E+00	-0.60	6.47E-01	9.47E-01
**DXO**	0.10	7.48E-01	1.00E+00	-0.12	6.47E-01	9.47E-01
**LAMTOR2**	-0.03	8.81E-01	1.00E+00	-0.13	6.47E-01	9.47E-01
**AL358472.4**	-0.70	3.94E-01	1.00E+00	-0.18	6.47E-01	9.47E-01
**UBASH3A**	-1.06	4.52E-01	1.00E+00	1.45	6.47E-01	9.47E-01
**DCLK2**	-0.25	6.06E-01	1.00E+00	0.37	6.47E-01	9.47E-01
**CH25H**	-0.72	3.26E-01	1.00E+00	0.32	6.47E-01	9.47E-01
**AVPR2**	0.29	6.30E-01	1.00E+00	-0.31	6.47E-01	9.47E-01
**HMGA1P5**	-3.79	2.45E-01	1.00E+00	-0.68	6.47E-01	9.47E-01
**MTMR1**	0.07	8.22E-01	1.00E+00	-0.19	6.47E-01	9.47E-01
**TSPAN15**	0.77	1.72E-01	1.00E+00	-0.28	6.47E-01	9.47E-01
**FAM120B**	-0.12	5.04E-01	1.00E+00	-0.08	6.47E-01	9.47E-01
**MID1**	-0.06	8.33E-01	1.00E+00	0.13	6.47E-01	9.47E-01
**NABP1**	-0.07	8.66E-01	1.00E+00	-0.26	6.48E-01	9.47E-01
**MRPS21**	0.11	5.48E-01	1.00E+00	0.10	6.48E-01	9.47E-01
**SLC30A4**	0.03	9.37E-01	1.00E+00	-0.16	6.48E-01	9.47E-01
**TTL**	-0.10	5.70E-01	1.00E+00	-0.18	6.48E-01	9.47E-01
**AC009533.1**	-0.96	8.17E-02	1.00E+00	0.32	6.48E-01	9.47E-01
**TGM3**	0.10	8.55E-01	1.00E+00	-0.29	6.48E-01	9.47E-01
**RPL21P11**	0.01	9.94E-01	1.00E+00	-0.36	6.48E-01	9.47E-01
**GNRH1**	1.49	4.03E-01	1.00E+00	0.63	6.48E-01	9.47E-01
**STX18-AS1**	-0.15	7.97E-01	1.00E+00	0.24	6.48E-01	9.47E-01
**AL355877.1**	1.49	2.43E-01	1.00E+00	0.48	6.48E-01	9.47E-01
**PCDH10**	-1.78	3.10E-03	2.91E-01	-0.63	6.48E-01	9.47E-01
**DUSP3**	0.18	4.44E-01	1.00E+00	0.12	6.48E-01	9.47E-01

**G34192**	-0.78	2.25E-01	1.00E+00	-0.41	6.48E-01	9.47E-01
**AZIN1**	0.26	2.51E-01	1.00E+00	-0.13	6.48E-01	9.47E-01
**THAP8**	0.15	6.09E-01	1.00E+00	0.12	6.49E-01	9.47E-01
**AC011487.1**	0.11	9.73E-01	1.00E+00	0.54	6.49E-01	9.47E-01
**IL36A**	-1.68	6.27E-01	1.00E+00	1.46	6.49E-01	9.47E-01
**MREG**	-0.07	8.56E-01	1.00E+00	0.22	6.49E-01	9.47E-01
**FANCI**	-0.04	8.86E-01	1.00E+00	0.12	6.49E-01	9.47E-01
**FCHO1**	-0.41	4.47E-01	1.00E+00	0.18	6.49E-01	9.47E-01
**RAB11FIP1**	-0.14	7.78E-01	1.00E+00	-0.16	6.49E-01	9.47E-01
**XLOC_003234**	0.63	6.60E-01	1.00E+00	-0.48	6.49E-01	9.47E-01
**N4BP2**	-0.43	2.03E-01	1.00E+00	-0.17	6.49E-01	9.47E-01
**SPDYE18**	0.26	7.20E-01	1.00E+00	-0.29	6.49E-01	9.47E-01
**XLOC_008975**	-2.48	2.55E-01	1.00E+00	-0.53	6.49E-01	9.47E-01
**CRNKL1**	0.09	6.81E-01	1.00E+00	-0.07	6.49E-01	9.47E-01
**RNU5B-1**	-1.52	6.60E-01	1.00E+00	-0.56	6.49E-01	9.47E-01
**PAXBP1**	-0.02	9.48E-01	1.00E+00	-0.21	6.49E-01	9.47E-01
**FOXJ1**	0.07	9.02E-01	1.00E+00	0.34	6.49E-01	9.47E-01
**RP11-187C18.4**	0.41	6.23E-01	1.00E+00	0.31	6.49E-01	9.47E-01
**NSD2**	-0.19	3.38E-01	1.00E+00	0.21	6.49E-01	9.47E-01
**AC018647.1**	-0.74	3.19E-01	1.00E+00	-0.32	6.49E-01	9.47E-01
**B4GALT4-AS1**	-1.68	6.27E-01	1.00E+00	-0.73	6.49E-01	9.47E-01
**AC127496.5**	-1.16	5.83E-01	1.00E+00	-0.63	6.49E-01	9.47E-01
**BAG2**	0.22	6.76E-01	1.00E+00	-0.20	6.49E-01	9.47E-01
**AC100849.1**	-3.41	3.11E-01	1.00E+00	0.51	6.49E-01	9.47E-01
**LINC02193**	-0.26	8.19E-01	1.00E+00	0.33	6.49E-01	9.47E-01
**FAM228B**	-0.48	3.85E-01	1.00E+00	-0.17	6.49E-01	9.47E-01
**AC010487.1**	0.54	3.35E-01	1.00E+00	0.47	6.49E-01	9.47E-01
**BABAM1**	-0.01	9.76E-01	1.00E+00	-0.18	6.49E-01	9.47E-01
**ZNF182**	0.12	7.21E-01	1.00E+00	-0.28	6.49E-01	9.47E-01
**FOXN1**	-0.28	5.94E-01	1.00E+00	0.32	6.49E-01	9.47E-01
**ZSCAN31**	0.16	6.63E-01	1.00E+00	-0.18	6.49E-01	9.47E-01
**CXCL5**	-0.97	7.31E-01	1.00E+00	1.44	6.49E-01	9.47E-01

**Z74021.1**	0.15	9.22E-01	1.00E+00	0.38	6.49E-01	9.47E-01
**KATNBL1**	0.18	4.81E-01	1.00E+00	-0.15	6.50E-01	9.47E-01
**ATP6V0B**	-0.03	8.89E-01	1.00E+00	0.13	6.50E-01	9.47E-01
**FAM129A**	0.12	7.65E-01	1.00E+00	0.19	6.50E-01	9.47E-01
**NCL**	-0.27	1.50E-01	1.00E+00	0.12	6.50E-01	9.47E-01
**LINC01563**	-1.63	2.89E-01	1.00E+00	-0.47	6.50E-01	9.47E-01
**C17orf58**	0.16	6.30E-01	1.00E+00	-0.14	6.50E-01	9.47E-01
**AXIN2**	0.37	2.29E-01	1.00E+00	0.19	6.50E-01	9.47E-01
**NKILA**	-0.17	8.55E-01	1.00E+00	-0.37	6.50E-01	9.47E-01
**HSP90AB1**	-0.10	5.32E-01	1.00E+00	0.08	6.50E-01	9.47E-01
**FLVCR1**	0.04	9.17E-01	1.00E+00	-0.17	6.50E-01	9.47E-01
**XLOC_006144**	-0.75	3.30E-01	1.00E+00	-0.30	6.50E-01	9.47E-01
**AC019257.1**	0.34	8.08E-01	1.00E+00	-0.57	6.50E-01	9.47E-01
**RPL13P5**	-0.61	4.46E-01	1.00E+00	0.21	6.50E-01	9.48E-01
**IL6**	-6.66	3.39E-03	3.11E-01	1.42	6.50E-01	9.48E-01
**ING3**	-0.08	7.31E-01	1.00E+00	-0.15	6.50E-01	9.48E-01
**AC104066.1**	0.08	9.48E-01	1.00E+00	-0.39	6.51E-01	9.48E-01
**MAP4**	-0.15	5.03E-01	1.00E+00	0.16	6.51E-01	9.48E-01
**SCG2**	-0.22	8.67E-01	1.00E+00	0.60	6.51E-01	9.48E-01
**AP003774.4**	0.16	9.63E-01	1.00E+00	0.58	6.51E-01	9.48E-01
**PDCD6IPP2**	0.38	6.92E-01	1.00E+00	-0.31	6.51E-01	9.48E-01
**SELENOTP1**	1.52	6.43E-01	1.00E+00	-0.32	6.51E-01	9.48E-01
**RNF34**	0.20	3.88E-01	1.00E+00	-0.12	6.51E-01	9.48E-01
**HMGB3P6**	NA	NA	NA	0.58	6.51E-01	9.48E-01
**SRGAP2**	0.15	6.71E-01	1.00E+00	0.14	6.51E-01	9.48E-01
**TRAV1-1**	1.06	7.54E-01	1.00E+00	0.83	6.51E-01	9.48E-01
**TRPC1**	0.41	2.43E-01	1.00E+00	-0.26	6.51E-01	9.48E-01
**FADS6**	-0.06	9.17E-01	1.00E+00	-0.31	6.51E-01	9.48E-01
**AC097468.3**	-0.38	6.51E-01	1.00E+00	0.33	6.51E-01	9.48E-01
**EPM2AIP1**	-0.27	1.67E-01	1.00E+00	-0.08	6.51E-01	9.48E-01
**EIF3I**	-0.13	4.65E-01	1.00E+00	0.05	6.51E-01	9.48E-01
**CHRM3-AS2**	-1.10	7.53E-01	1.00E+00	0.57	6.51E-01	9.48E-01

**HSD17B6**	0.40	8.03E-01	1.00E+00	-0.34	6.51E-01	9.48E-01
**AC127502.1**	0.08	8.91E-01	1.00E+00	-0.26	6.51E-01	9.48E-01
**SRD5A1**	0.11	6.91E-01	1.00E+00	-0.15	6.51E-01	9.48E-01
**AL596330.1**	-0.85	5.97E-01	1.00E+00	0.63	6.51E-01	9.48E-01
**UPK1A**	2.10	3.53E-02	1.00E+00	0.44	6.51E-01	9.48E-01
**AC055811.4**	0.03	9.85E-01	1.00E+00	0.63	6.51E-01	9.48E-01
**KIR3DX1**	-0.16	9.62E-01	1.00E+00	0.70	6.51E-01	9.48E-01
**AC007495.1**	-1.08	1.64E-01	1.00E+00	-0.53	6.51E-01	9.48E-01
**SNRPD2**	-0.17	4.17E-01	1.00E+00	0.12	6.51E-01	9.48E-01
**CFAP99**	-3.71	4.92E-02	1.00E+00	0.66	6.51E-01	9.48E-01
**BPIFC**	0.12	8.12E-01	1.00E+00	0.36	6.51E-01	9.48E-01
**G9978**	-0.28	6.76E-01	1.00E+00	0.33	6.51E-01	9.48E-01
**AC244517.3**	-0.67	8.48E-01	1.00E+00	0.84	6.51E-01	9.48E-01
**TARBP1**	0.25	3.92E-01	1.00E+00	0.23	6.52E-01	9.48E-01
**XLOC_013703**	1.17	3.59E-01	1.00E+00	-0.41	6.52E-01	9.48E-01
**TLE6**	-0.74	4.40E-01	1.00E+00	0.37	6.52E-01	9.48E-01
**PDZK1IP1**	0.15	7.44E-01	1.00E+00	0.25	6.52E-01	9.48E-01
**FLNA**	-0.46	2.37E-01	1.00E+00	0.29	6.52E-01	9.48E-01
**PHLDB2**	0.25	6.15E-01	1.00E+00	0.18	6.52E-01	9.48E-01
**AL445187.1**	1.45	4.52E-01	1.00E+00	-0.60	6.52E-01	9.48E-01
**SLC13A3**	-0.14	6.60E-01	1.00E+00	-0.27	6.52E-01	9.48E-01
**AC079336.5**	0.04	9.80E-01	1.00E+00	-0.58	6.52E-01	9.48E-01
**XLOC_010023**	-1.48	3.00E-01	1.00E+00	-0.35	6.52E-01	9.48E-01
**PFDN5**	-0.05	8.42E-01	1.00E+00	0.14	6.52E-01	9.48E-01
**CCNJ**	-0.32	5.26E-01	1.00E+00	0.18	6.52E-01	9.48E-01
**PCDHB11**	0.67	2.56E-01	1.00E+00	0.20	6.52E-01	9.48E-01
**ANKRD13A**	-0.14	6.05E-01	1.00E+00	-0.16	6.52E-01	9.48E-01
**NINJ1**	-0.61	3.89E-02	1.00E+00	-0.11	6.52E-01	9.48E-01
**KLC3**	-0.51	2.28E-01	1.00E+00	-0.30	6.52E-01	9.48E-01
**MFSD13B**	0.03	9.82E-01	1.00E+00	-0.32	6.52E-01	9.48E-01
**UBXN10-AS1**	0.63	5.10E-01	1.00E+00	0.50	6.52E-01	9.48E-01
**AC084783.1**	0.97	7.33E-01	1.00E+00	-0.53	6.52E-01	9.48E-01

**CHMP3**	0.15	3.33E-01	1.00E+00	-0.09	6.52E-01	9.48E-01
**XLOC_013434**	0.24	8.48E-01	1.00E+00	0.62	6.52E-01	9.48E-01
**SFTPD**	-0.04	9.24E-01	1.00E+00	0.23	6.53E-01	9.48E-01
**ZNF595**	0.41	3.98E-01	1.00E+00	0.21	6.53E-01	9.48E-01
**AC114956.3**	2.40	2.69E-03	2.68E-01	-0.26	6.53E-01	9.48E-01
**CXCL9**	-4.11	9.12E-07	3.41E-04	1.41	6.53E-01	9.48E-01
**AP006621.5**	-1.71	8.31E-02	1.00E+00	-0.53	6.53E-01	9.48E-01
**PTS**	0.20	5.03E-01	1.00E+00	-0.16	6.53E-01	9.48E-01
**HSPB1P2**	-1.33	6.98E-01	1.00E+00	-0.36	6.53E-01	9.48E-01
**ZNF398**	-0.18	3.84E-01	1.00E+00	-0.16	6.53E-01	9.48E-01
**CDADC1**	0.23	4.01E-01	1.00E+00	-0.13	6.53E-01	9.48E-01
**AC114728.1**	0.80	8.17E-01	1.00E+00	-0.36	6.53E-01	9.48E-01
**MLIP-IT1**	0.32	8.41E-01	1.00E+00	-0.26	6.53E-01	9.48E-01
**ZNF486**	0.35	3.49E-01	1.00E+00	0.16	6.53E-01	9.48E-01
**NRCAM**	0.09	8.69E-01	1.00E+00	0.27	6.53E-01	9.48E-01
**GPC1**	-0.35	3.04E-01	1.00E+00	-0.25	6.53E-01	9.48E-01
**DTX2**	-0.17	6.75E-01	1.00E+00	0.17	6.53E-01	9.48E-01
**CCNE2**	-0.27	6.64E-01	1.00E+00	-0.24	6.53E-01	9.48E-01
**MED12L**	-0.54	6.07E-01	1.00E+00	-0.28	6.53E-01	9.48E-01
**ANKRD30B**	-0.98	7.31E-01	1.00E+00	-1.06	6.53E-01	9.48E-01
**PEX13**	-0.25	3.33E-01	1.00E+00	-0.13	6.53E-01	9.48E-01
**UBAP1**	0.02	8.98E-01	1.00E+00	0.07	6.53E-01	9.48E-01
**DTX1**	0.87	1.54E-01	1.00E+00	0.35	6.53E-01	9.48E-01
**MYRF**	-1.50	4.62E-04	9.31E-02	-0.44	6.53E-01	9.48E-01
**CYP51A1P3**	0.69	6.14E-01	1.00E+00	-0.46	6.53E-01	9.48E-01
**FAM160A1**	-0.20	5.49E-01	1.00E+00	0.27	6.53E-01	9.48E-01
**TET2-AS1**	NA	NA	NA	0.47	6.53E-01	9.48E-01
**SYT3**	-2.55	3.01E-02	9.97E-01	0.60	6.53E-01	9.48E-01
**LINC00641**	-0.97	1.61E-01	1.00E+00	-0.34	6.54E-01	9.48E-01
**G42780**	0.41	8.81E-01	1.00E+00	0.42	6.54E-01	9.48E-01
**SORBS2**	-0.02	9.71E-01	1.00E+00	-0.19	6.54E-01	9.48E-01
**ACOT1**	0.78	3.02E-01	1.00E+00	-0.32	6.54E-01	9.48E-01

**MTCP1**	0.06	9.62E-01	1.00E+00	-0.36	6.54E-01	9.48E-01
**LMO1**	-0.99	8.65E-03	5.74E-01	-0.28	6.54E-01	9.48E-01
**LCE1C**	0.45	5.02E-01	1.00E+00	1.40	6.54E-01	9.48E-01
**RAMP1**	-0.56	1.37E-01	1.00E+00	0.14	6.54E-01	9.48E-01
**RPS26**	0.50	2.79E-02	9.73E-01	0.11	6.54E-01	9.48E-01
**AC018695.1**	NA	NA	NA	0.49	6.54E-01	9.48E-01
**MAZ**	-0.47	1.38E-01	1.00E+00	0.20	6.54E-01	9.48E-01
**AC012065.3**	-0.27	9.34E-01	1.00E+00	-0.72	6.54E-01	9.48E-01
**MROH1**	-0.26	4.25E-01	1.00E+00	0.28	6.54E-01	9.48E-01
**G39254**	-0.69	4.39E-01	1.00E+00	0.63	6.54E-01	9.48E-01
**PPP1CC**	0.13	4.74E-01	1.00E+00	-0.14	6.54E-01	9.48E-01
**SLF1**	0.43	2.43E-01	1.00E+00	-0.14	6.54E-01	9.48E-01
**KRTAP13-1**	-1.16	6.65E-01	1.00E+00	1.42	6.54E-01	9.48E-01
**STK31**	0.78	6.01E-01	1.00E+00	0.56	6.54E-01	9.48E-01
**MYBPC2**	-1.63	6.24E-02	1.00E+00	0.67	6.54E-01	9.48E-01
**KCNB2**	1.01	5.80E-01	1.00E+00	0.56	6.54E-01	9.48E-01
**IGSF21**	-1.07	7.04E-02	1.00E+00	0.48	6.54E-01	9.48E-01
**AC017083.1**	2.60	3.47E-01	1.00E+00	0.49	6.54E-01	9.48E-01
**NAP1L2**	-0.13	6.52E-01	1.00E+00	0.24	6.54E-01	9.48E-01
**LIG1**	-0.51	1.20E-01	1.00E+00	0.15	6.54E-01	9.48E-01
**CCDC188**	-0.41	6.93E-01	1.00E+00	0.66	6.54E-01	9.48E-01
**C2orf40**	0.46	3.94E-01	1.00E+00	-0.22	6.54E-01	9.48E-01
**AL356489.2**	0.72	3.27E-01	1.00E+00	0.49	6.54E-01	9.48E-01
**LRRC2**	0.64	2.32E-01	1.00E+00	0.25	6.54E-01	9.48E-01
**GCDH**	0.01	9.83E-01	1.00E+00	0.07	6.55E-01	9.48E-01
**TMPPE**	-0.19	6.06E-01	1.00E+00	0.27	6.55E-01	9.48E-01
**AC106791.1**	3.45	1.53E-01	1.00E+00	-0.41	6.55E-01	9.48E-01
**AL022157.1**	1.32	3.43E-01	1.00E+00	0.54	6.55E-01	9.48E-01
**AL157938.2**	-1.87	5.55E-01	1.00E+00	-0.50	6.55E-01	9.48E-01
**AC024060.1**	0.81	5.05E-01	1.00E+00	-0.28	6.55E-01	9.48E-01
**UBC**	-0.31	1.83E-01	1.00E+00	0.11	6.55E-01	9.48E-01
**RPS23P8**	0.06	9.23E-01	1.00E+00	-0.30	6.55E-01	9.48E-01

**SSBP3**	-0.38	1.33E-01	1.00E+00	0.14	6.55E-01	9.48E-01
**MYO10**	-0.13	5.65E-01	1.00E+00	0.18	6.55E-01	9.48E-01
**SRRT**	-0.50	1.24E-02	6.93E-01	0.16	6.55E-01	9.48E-01
**SEC31A**	-0.10	6.00E-01	1.00E+00	0.09	6.55E-01	9.48E-01
**ZFP41**	0.25	5.29E-01	1.00E+00	0.29	6.55E-01	9.48E-01
**ACSS3**	0.89	7.71E-02	1.00E+00	-0.24	6.55E-01	9.48E-01
**PDZD11**	0.06	7.10E-01	1.00E+00	-0.12	6.55E-01	9.48E-01
**XLOC_008504**	-1.58	4.67E-01	1.00E+00	0.54	6.55E-01	9.48E-01
**OPRK1**	2.84	1.07E-01	1.00E+00	-0.65	6.56E-01	9.48E-01
**GNG12-AS1**	-0.43	3.01E-01	1.00E+00	-0.24	6.56E-01	9.48E-01
**ENSAP2**	-1.22	3.91E-01	1.00E+00	-0.29	6.56E-01	9.48E-01
**BTBD7**	0.36	8.27E-02	1.00E+00	0.09	6.56E-01	9.48E-01
**XLOC_010367**	1.38	6.90E-01	1.00E+00	-0.57	6.56E-01	9.48E-01
**GALR2**	0.14	8.84E-01	1.00E+00	0.51	6.56E-01	9.48E-01
**PCCA-AS1**	-0.70	8.41E-01	1.00E+00	0.64	6.56E-01	9.48E-01
**THSD7B**	0.20	8.03E-01	1.00E+00	-0.53	6.56E-01	9.48E-01
**ARPC3P1**	0.26	9.40E-01	1.00E+00	-0.31	6.56E-01	9.48E-01
**REM2**	-0.22	8.09E-01	1.00E+00	-0.26	6.56E-01	9.48E-01
**POLE2**	0.02	9.62E-01	1.00E+00	0.20	6.56E-01	9.48E-01
**TMEM178B**	-0.77	2.47E-01	1.00E+00	-0.38	6.56E-01	9.48E-01
**OXCT2**	-1.92	4.22E-01	1.00E+00	-0.41	6.56E-01	9.48E-01
**AC068473.3**	-1.96	6.52E-02	1.00E+00	-0.45	6.56E-01	9.48E-01
**CBX2**	-0.43	1.24E-01	1.00E+00	0.20	6.56E-01	9.48E-01
**TRHDE**	1.37	6.52E-02	1.00E+00	-0.41	6.56E-01	9.49E-01
**AC006213.2**	-3.01	6.26E-02	1.00E+00	0.24	6.56E-01	9.49E-01
**AIFM1**	0.12	6.93E-01	1.00E+00	-0.15	6.56E-01	9.49E-01
**RPS27AP16**	-0.57	1.21E-01	1.00E+00	-0.25	6.56E-01	9.49E-01
**PDLIM4**	-0.39	2.16E-01	1.00E+00	-0.12	6.56E-01	9.49E-01
**CCL1**	-0.66	7.72E-01	1.00E+00	-0.64	6.56E-01	9.49E-01
**BAMBI**	0.04	9.61E-01	1.00E+00	-0.33	6.56E-01	9.49E-01
**XLOC_012210**	-0.57	8.42E-01	1.00E+00	0.64	6.56E-01	9.49E-01
**C16orf95**	-0.50	7.19E-01	1.00E+00	-0.27	6.57E-01	9.49E-01

**BUB3**	-0.11	6.01E-01	1.00E+00	-0.14	6.57E-01	9.49E-01
**FLJ16779**	-0.96	3.12E-01	1.00E+00	-0.48	6.57E-01	9.49E-01
**DLG2**	0.04	9.29E-01	1.00E+00	-0.24	6.57E-01	9.49E-01
**TRIM63**	0.31	4.94E-01	1.00E+00	0.30	6.57E-01	9.49E-01
**STARD8**	-0.01	9.89E-01	1.00E+00	0.36	6.57E-01	9.49E-01
**SLC16A12**	1.07	6.15E-02	1.00E+00	-0.42	6.57E-01	9.49E-01
**LHX6**	-0.51	2.32E-01	1.00E+00	0.27	6.57E-01	9.49E-01
**CCDC186**	-0.09	7.83E-01	1.00E+00	-0.12	6.57E-01	9.49E-01
**FAF2**	-0.01	9.52E-01	1.00E+00	-0.06	6.57E-01	9.49E-01
**GID4**	0.24	3.66E-01	1.00E+00	-0.18	6.57E-01	9.49E-01
**Z82195.2**	-3.01	3.64E-01	1.00E+00	-0.80	6.57E-01	9.49E-01
**AP003392.1**	-1.10	3.93E-01	1.00E+00	0.40	6.57E-01	9.49E-01
**SMPDL3B**	-1.08	3.44E-02	1.00E+00	0.21	6.57E-01	9.49E-01
**VEGFB**	0.39	2.89E-01	1.00E+00	-0.23	6.57E-01	9.49E-01
**RGS2**	0.22	4.95E-01	1.00E+00	0.13	6.57E-01	9.49E-01
**CHRNA1**	0.45	8.24E-01	1.00E+00	-0.58	6.57E-01	9.49E-01
**G35510**	-0.09	8.35E-01	1.00E+00	0.30	6.57E-01	9.49E-01
**TCF3**	-0.28	2.89E-01	1.00E+00	0.24	6.57E-01	9.49E-01
**NDUFS3**	-0.06	6.61E-01	1.00E+00	-0.09	6.57E-01	9.49E-01
**LINC01018**	-0.19	8.13E-01	1.00E+00	-0.40	6.57E-01	9.49E-01
**FASTK**	-0.10	6.08E-01	1.00E+00	-0.10	6.58E-01	9.49E-01
**AC079630.1**	0.54	3.07E-01	1.00E+00	0.33	6.58E-01	9.49E-01
**CEP44**	0.06	8.42E-01	1.00E+00	-0.14	6.58E-01	9.49E-01
**LINC00887**	0.68	7.02E-01	1.00E+00	-0.40	6.58E-01	9.49E-01
**AC023906.1**	1.37	6.91E-01	1.00E+00	-0.31	6.58E-01	9.49E-01
**PCDHGA1**	0.27	6.11E-01	1.00E+00	0.45	6.58E-01	9.49E-01
**MSTO2P**	0.04	9.61E-01	1.00E+00	0.34	6.58E-01	9.49E-01
**GJA1P1**	-0.64	6.25E-01	1.00E+00	-0.30	6.58E-01	9.49E-01
**XLOC_001401**	0.68	2.66E-01	1.00E+00	-0.45	6.58E-01	9.49E-01
**TMEM179B**	0.01	9.68E-01	1.00E+00	-0.21	6.58E-01	9.49E-01
**AL713852.1**	-1.89	3.25E-01	1.00E+00	-0.48	6.58E-01	9.49E-01
**snoU13**	0.22	6.14E-01	1.00E+00	0.12	6.58E-01	9.49E-01

**URB1-AS1**	-0.24	4.43E-01	1.00E+00	-0.14	6.58E-01	9.49E-01
**NPM1P24**	1.22	6.21E-01	1.00E+00	-0.32	6.58E-01	9.49E-01
**AC119044.1**	0.36	8.30E-01	1.00E+00	0.46	6.58E-01	9.49E-01
**AC116407.4**	-1.45	4.10E-01	1.00E+00	0.50	6.58E-01	9.49E-01
**AC072039.1**	-1.47	6.70E-01	1.00E+00	-1.19	6.58E-01	9.49E-01
**ADCK1**	-0.03	8.72E-01	1.00E+00	-0.11	6.58E-01	9.49E-01
**C2orf27A**	-0.07	9.08E-01	1.00E+00	0.19	6.58E-01	9.49E-01
**PARP1**	-0.14	4.02E-01	1.00E+00	0.11	6.58E-01	9.49E-01
**AC145285.2**	0.17	8.29E-01	1.00E+00	0.32	6.58E-01	9.49E-01
**CASC3**	-0.22	2.85E-01	1.00E+00	-0.11	6.59E-01	9.49E-01
**SNHG28**	-0.42	5.04E-01	1.00E+00	-0.14	6.59E-01	9.49E-01
**AC005480.1**	1.31	7.03E-01	1.00E+00	0.74	6.59E-01	9.49E-01
**HOXA11**	0.97	1.15E-01	1.00E+00	0.42	6.59E-01	9.49E-01
**4-Sep**	-0.43	3.68E-01	1.00E+00	-0.16	6.59E-01	9.49E-01
**SULT1C4**	0.77	2.95E-01	1.00E+00	0.32	6.59E-01	9.49E-01
**BUB1**	-0.05	8.70E-01	1.00E+00	0.24	6.59E-01	9.49E-01
**FAM83F**	0.15	6.43E-01	1.00E+00	-0.22	6.59E-01	9.49E-01
**FEM1C**	0.01	9.62E-01	1.00E+00	0.12	6.59E-01	9.49E-01
**MCM3AP**	0.07	7.67E-01	1.00E+00	0.19	6.59E-01	9.49E-01
**NSUN2**	-0.25	3.22E-01	1.00E+00	-0.10	6.59E-01	9.49E-01
**ZNF621**	0.29	4.42E-01	1.00E+00	0.20	6.59E-01	9.49E-01
**EIF2S3B**	-2.70	2.38E-02	9.16E-01	-0.23	6.59E-01	9.49E-01
**KIFC2**	-0.46	2.13E-01	1.00E+00	0.41	6.59E-01	9.49E-01
**PEG3**	-0.20	7.20E-01	1.00E+00	0.21	6.59E-01	9.49E-01
**MCPH1**	-0.15	4.36E-01	1.00E+00	0.09	6.59E-01	9.49E-01
**XLOC_009868**	-0.03	9.66E-01	1.00E+00	0.49	6.59E-01	9.49E-01
**NPEPPS**	0.20	3.61E-01	1.00E+00	0.07	6.59E-01	9.49E-01
**BX255925.3**	0.01	9.64E-01	1.00E+00	0.16	6.59E-01	9.49E-01
**FOXI2**	0.30	4.99E-01	1.00E+00	-0.42	6.59E-01	9.49E-01
**AC007598.3**	1.64	5.50E-01	1.00E+00	-0.68	6.59E-01	9.49E-01
**TIPARP**	-0.33	5.59E-01	1.00E+00	0.11	6.59E-01	9.49E-01
**MFN1**	0.02	9.33E-01	1.00E+00	0.08	6.59E-01	9.49E-01

**MAP2**	-0.26	4.58E-01	1.00E+00	0.16	6.59E-01	9.49E-01
**ZBED9**	0.41	6.23E-01	1.00E+00	0.45	6.59E-01	9.49E-01
**CTH**	0.52	2.34E-01	1.00E+00	-0.25	6.59E-01	9.49E-01
**ZNF830**	0.00	9.86E-01	1.00E+00	0.09	6.59E-01	9.49E-01
**PBLD**	0.39	4.23E-01	1.00E+00	0.19	6.59E-01	9.49E-01
**BLOC1S6**	0.21	4.30E-01	1.00E+00	0.11	6.59E-01	9.49E-01
**NVL**	0.14	5.25E-01	1.00E+00	-0.09	6.59E-01	9.49E-01
**F7**	-0.65	1.75E-01	1.00E+00	0.47	6.60E-01	9.49E-01
**UBR2**	0.28	1.86E-01	1.00E+00	-0.11	6.60E-01	9.49E-01
**AC103740.1**	-1.86	9.84E-02	1.00E+00	0.42	6.60E-01	9.49E-01
**KRTAP12-1**	-30.00	2.73E-18	5.54E-15	-1.41	6.60E-01	9.49E-01
**HMMR**	0.30	4.25E-01	1.00E+00	-0.23	6.60E-01	9.49E-01
**SDHAF1**	-0.14	5.48E-01	1.00E+00	0.07	6.60E-01	9.49E-01
**ABCB9**	-0.18	6.65E-01	1.00E+00	-0.25	6.60E-01	9.49E-01
**TMEM241**	0.10	7.64E-01	1.00E+00	0.18	6.60E-01	9.49E-01
**TNFAIP8L1**	-0.05	8.24E-01	1.00E+00	0.22	6.60E-01	9.49E-01
**AC027097.1**	-0.28	7.39E-01	1.00E+00	-0.18	6.60E-01	9.49E-01
**SNCAIP**	-0.17	6.71E-01	1.00E+00	-0.20	6.60E-01	9.49E-01
**AC005332.3**	0.04	9.64E-01	1.00E+00	-0.24	6.60E-01	9.49E-01
**PCSK6**	-0.26	6.34E-01	1.00E+00	0.27	6.60E-01	9.49E-01
**TMEM41B**	0.35	3.00E-01	1.00E+00	-0.16	6.60E-01	9.49E-01
**SDR16C5**	0.41	4.07E-01	1.00E+00	-0.26	6.60E-01	9.49E-01
**AC118549.1**	0.14	5.42E-01	1.00E+00	0.09	6.60E-01	9.49E-01
**DGKH**	-0.19	5.14E-01	1.00E+00	0.21	6.60E-01	9.49E-01
**NHSL1**	-0.30	4.69E-01	1.00E+00	0.15	6.61E-01	9.49E-01
**TBCAP1**	-1.50	5.52E-01	1.00E+00	-0.26	6.61E-01	9.49E-01
**ARL13A**	1.57	1.80E-01	1.00E+00	-0.48	6.61E-01	9.49E-01
**AP1G1**	0.27	2.12E-01	1.00E+00	0.12	6.61E-01	9.49E-01
**G22214**	0.83	5.42E-01	1.00E+00	-0.30	6.61E-01	9.49E-01
**IMPDH1**	0.18	3.92E-01	1.00E+00	0.13	6.61E-01	9.49E-01
**ARHGEF18**	-0.35	5.54E-01	1.00E+00	0.22	6.61E-01	9.49E-01
**WWC2-AS2**	-0.47	2.47E-01	1.00E+00	0.36	6.61E-01	9.49E-01

**KLHL22**	-0.36	2.50E-01	1.00E+00	0.15	6.61E-01	9.49E-01
**SPRYD3**	0.06	7.49E-01	1.00E+00	0.12	6.61E-01	9.49E-01
**FAM85B**	-0.39	7.68E-01	1.00E+00	-0.31	6.61E-01	9.49E-01
**RPL7AP6**	-0.49	2.45E-01	1.00E+00	0.16	6.61E-01	9.49E-01
**KRT13**	0.91	4.50E-01	1.00E+00	0.63	6.61E-01	9.49E-01
**KLHL9**	0.14	6.26E-01	1.00E+00	-0.07	6.61E-01	9.49E-01
**LAG3**	-0.02	9.71E-01	1.00E+00	1.37	6.61E-01	9.49E-01
**TAF4B**	0.02	9.41E-01	1.00E+00	-0.16	6.61E-01	9.49E-01
**KLHL32**	-1.22	3.95E-01	1.00E+00	-0.53	6.61E-01	9.50E-01
**TOR1AIP1**	0.39	7.80E-02	1.00E+00	-0.09	6.61E-01	9.50E-01
**TMEM74B**	0.15	7.05E-01	1.00E+00	0.33	6.61E-01	9.50E-01
**OR51E1**	0.73	4.78E-01	1.00E+00	1.14	6.61E-01	9.50E-01
**SIVA1**	0.00	9.87E-01	1.00E+00	0.10	6.61E-01	9.50E-01
**ELAVL4**	-2.31	1.04E-03	1.67E-01	0.36	6.61E-01	9.50E-01
**KRT8P12**	-0.38	2.96E-01	1.00E+00	0.25	6.62E-01	9.50E-01
**C6orf163**	-0.53	6.20E-01	1.00E+00	-0.36	6.62E-01	9.50E-01
**ZNF98**	-1.62	3.81E-01	1.00E+00	0.50	6.62E-01	9.50E-01
**IL17RD**	0.27	5.21E-01	1.00E+00	0.21	6.62E-01	9.50E-01
**TOPBP1**	0.09	6.39E-01	1.00E+00	-0.17	6.62E-01	9.50E-01
**CLBA1**	-0.23	3.35E-01	1.00E+00	-0.24	6.62E-01	9.50E-01
**C1GALT1**	-0.13	6.87E-01	1.00E+00	-0.09	6.62E-01	9.50E-01
**AC093627.6**	-0.21	8.49E-01	1.00E+00	0.47	6.62E-01	9.50E-01
**CCDC102A**	-0.10	7.06E-01	1.00E+00	0.23	6.62E-01	9.50E-01
**MS4A3**	0.44	8.98E-01	1.00E+00	0.80	6.62E-01	9.50E-01
**IFNA20P**	0.87	6.12E-01	1.00E+00	-0.50	6.62E-01	9.50E-01
**RIMBP3B**	0.43	7.18E-01	1.00E+00	0.67	6.62E-01	9.50E-01
**XLOC_002384**	-0.06	9.59E-01	1.00E+00	-0.35	6.62E-01	9.50E-01
**AC073611.1**	-1.02	7.49E-01	1.00E+00	0.29	6.62E-01	9.50E-01
**RAD50**	-0.55	2.39E-01	1.00E+00	-0.16	6.62E-01	9.50E-01
**DICER1**	0.12	5.73E-01	1.00E+00	0.12	6.62E-01	9.50E-01
**CSNK1G2**	-0.27	2.86E-01	1.00E+00	0.25	6.62E-01	9.50E-01
**VPS37D**	0.13	7.50E-01	1.00E+00	-1.38	6.62E-01	9.50E-01

**ZNF785**	0.06	8.77E-01	1.00E+00	-0.23	6.62E-01	9.50E-01
**2-Sep**	0.24	3.13E-01	1.00E+00	-0.12	6.62E-01	9.50E-01
**ZBTB10**	0.15	6.26E-01	1.00E+00	0.20	6.63E-01	9.50E-01
**PSD2**	-0.18	8.88E-01	1.00E+00	-0.57	6.63E-01	9.50E-01
**VPS51**	-0.08	7.94E-01	1.00E+00	0.15	6.63E-01	9.50E-01
**DENND2A**	0.51	2.95E-01	1.00E+00	0.37	6.63E-01	9.51E-01
**NDUFA11**	0.05	9.19E-01	1.00E+00	-0.26	6.63E-01	9.51E-01
**AC093484.4**	-1.82	4.45E-01	1.00E+00	-0.36	6.63E-01	9.51E-01
**WAC-AS1**	-0.01	9.76E-01	1.00E+00	0.16	6.63E-01	9.51E-01
**PER2**	-0.98	5.09E-03	4.18E-01	-0.14	6.63E-01	9.51E-01
**FAM227A**	0.10	8.21E-01	1.00E+00	-0.38	6.63E-01	9.51E-01
**ST18**	-4.31	5.40E-04	1.05E-01	0.65	6.64E-01	9.51E-01
**PPP3CA**	-0.22	2.06E-01	1.00E+00	0.14	6.64E-01	9.51E-01
**AC009093.2**	-0.13	8.95E-01	1.00E+00	0.29	6.64E-01	9.51E-01
**PTTG1IP**	-0.08	7.42E-01	1.00E+00	-0.09	6.64E-01	9.51E-01
**DDIT4L**	-0.57	3.67E-01	1.00E+00	-0.31	6.64E-01	9.51E-01
**TMEM268**	-0.16	6.83E-01	1.00E+00	0.26	6.64E-01	9.51E-01
**SLC35B1**	-0.19	3.17E-01	1.00E+00	-0.06	6.64E-01	9.51E-01
**AC008747.1**	-3.28	2.65E-01	1.00E+00	-0.42	6.64E-01	9.51E-01
**AC090519.1**	0.23	8.64E-01	1.00E+00	-0.53	6.64E-01	9.51E-01
**AC018809.2**	-0.15	6.07E-01	1.00E+00	0.25	6.64E-01	9.51E-01
**ITFG2**	0.03	9.15E-01	1.00E+00	0.07	6.64E-01	9.51E-01
**AC025171.4**	1.10	5.19E-01	1.00E+00	0.48	6.64E-01	9.51E-01
**AC005224.3**	1.50	6.61E-01	1.00E+00	-0.56	6.64E-01	9.51E-01
**AC106786.1**	-0.11	9.13E-01	1.00E+00	0.42	6.64E-01	9.51E-01
**AC103746.1**	0.07	9.43E-01	1.00E+00	0.57	6.64E-01	9.51E-01
**MYO15A**	-0.52	5.99E-01	1.00E+00	0.42	6.64E-01	9.51E-01
**DUSP11**	0.00	9.99E-01	1.00E+00	-0.16	6.64E-01	9.51E-01
**G39463**	0.64	5.28E-01	1.00E+00	-0.39	6.64E-01	9.51E-01
**AC243836.1**	3.10	2.02E-01	1.00E+00	0.72	6.64E-01	9.51E-01
**INPP5J**	-0.58	1.41E-01	1.00E+00	0.23	6.64E-01	9.51E-01
**AC068473.4**	-1.92	1.52E-02	7.65E-01	-0.39	6.64E-01	9.51E-01

**MPHOSPH8**	0.22	3.30E-01	1.00E+00	0.12	6.64E-01	9.51E-01
**CALHM4**	-3.78	2.28E-01	1.00E+00	-1.39	6.64E-01	9.51E-01
**AC023590.1**	-1.68	6.27E-01	1.00E+00	0.47	6.65E-01	9.51E-01
**AARS**	-0.13	6.56E-01	1.00E+00	0.14	6.65E-01	9.51E-01
**RPSAP19**	0.24	7.43E-01	1.00E+00	-0.21	6.65E-01	9.51E-01
**RPL10P6**	0.04	9.74E-01	1.00E+00	0.37	6.65E-01	9.51E-01
**AC079949.1**	0.10	7.86E-01	1.00E+00	-0.46	6.65E-01	9.51E-01
**AC113143.1**	-0.44	9.00E-01	1.00E+00	0.61	6.65E-01	9.51E-01
**PMS2CL**	-0.45	1.71E-01	1.00E+00	0.20	6.65E-01	9.51E-01
**MMS19**	0.01	9.49E-01	1.00E+00	0.12	6.65E-01	9.51E-01
**EIF4H**	0.00	9.86E-01	1.00E+00	0.07	6.65E-01	9.51E-01
**WNT2**	0.02	9.68E-01	1.00E+00	0.26	6.65E-01	9.51E-01
**AL121753.2**	-0.15	7.16E-01	1.00E+00	-0.18	6.65E-01	9.51E-01
**FAM50A**	-0.01	9.58E-01	1.00E+00	0.16	6.65E-01	9.51E-01
**AC092306.1**	-0.35	4.62E-01	1.00E+00	0.30	6.65E-01	9.51E-01
**ATP13A4**	0.16	6.77E-01	1.00E+00	-0.21	6.65E-01	9.51E-01
**EXOSC7**	0.40	1.25E-01	1.00E+00	-0.17	6.65E-01	9.51E-01
**LCLAT1**	0.04	8.92E-01	1.00E+00	0.15	6.65E-01	9.51E-01
**AL592295.4**	-0.74	1.46E-01	1.00E+00	0.37	6.66E-01	9.51E-01
**PLD1**	-0.07	8.34E-01	1.00E+00	-0.16	6.66E-01	9.51E-01
**ESRP1**	-0.03	9.38E-01	1.00E+00	0.24	6.66E-01	9.51E-01
**MAFB**	-0.70	1.75E-01	1.00E+00	0.20	6.66E-01	9.51E-01
**SERPINB8**	-0.08	8.68E-01	1.00E+00	-0.14	6.66E-01	9.51E-01
**CCNA1**	-1.27	2.90E-01	1.00E+00	0.52	6.66E-01	9.51E-01
**SLC25A36**	0.24	4.07E-01	1.00E+00	-0.13	6.66E-01	9.51E-01
**KLLN**	0.09	8.28E-01	1.00E+00	-0.23	6.66E-01	9.51E-01
**AC008268.1**	1.44	2.48E-01	1.00E+00	-1.38	6.66E-01	9.51E-01
**LINC01752**	0.66	6.17E-01	1.00E+00	-0.42	6.66E-01	9.51E-01
**PPP2R5C**	0.07	6.25E-01	1.00E+00	0.09	6.66E-01	9.51E-01
**LONRF3**	0.09	8.89E-01	1.00E+00	-0.31	6.66E-01	9.51E-01
**RPS26P6**	-0.33	7.01E-01	1.00E+00	0.28	6.66E-01	9.51E-01
**LINC01886**	-0.59	6.93E-01	1.00E+00	-0.54	6.66E-01	9.51E-01

**FBF1**	-0.20	6.41E-01	1.00E+00	-0.23	6.66E-01	9.51E-01
**AC009061.2**	0.15	8.31E-01	1.00E+00	-0.25	6.66E-01	9.51E-01
**SS18**	0.04	8.79E-01	1.00E+00	0.08	6.66E-01	9.51E-01
**AL356653.1**	-0.08	9.82E-01	1.00E+00	-0.30	6.66E-01	9.51E-01
**RHOQ**	0.38	3.15E-01	1.00E+00	-0.21	6.66E-01	9.51E-01
**IL12A-AS1**	-0.22	8.89E-01	1.00E+00	0.58	6.66E-01	9.51E-01
**AC106881.1**	-0.21	8.35E-01	1.00E+00	-0.44	6.66E-01	9.51E-01
**FLRT3**	-0.03	9.35E-01	1.00E+00	0.23	6.66E-01	9.51E-01
**CCDC17**	0.80	1.88E-01	1.00E+00	-0.37	6.66E-01	9.51E-01
**MPHOSPH6**	0.10	7.18E-01	1.00E+00	-0.15	6.66E-01	9.51E-01
**AC145343.1**	-0.52	7.86E-01	1.00E+00	0.62	6.66E-01	9.51E-01
**HSDL1**	0.23	4.26E-01	1.00E+00	0.15	6.66E-01	9.51E-01
**DOLPP1**	-0.03	9.12E-01	1.00E+00	-0.10	6.66E-01	9.51E-01
**AC093591.1**	0.88	7.04E-01	1.00E+00	-0.31	6.67E-01	9.51E-01
**PPM1D**	-0.14	6.58E-01	1.00E+00	0.14	6.67E-01	9.51E-01
**MAB21L2**	0.08	9.79E-01	1.00E+00	0.65	6.67E-01	9.51E-01
**VANGL1**	0.04	8.62E-01	1.00E+00	0.10	6.67E-01	9.51E-01
**C15orf48**	0.98	1.11E-01	1.00E+00	-0.31	6.67E-01	9.51E-01
**AC068305.2**	-0.11	8.96E-01	1.00E+00	0.28	6.67E-01	9.51E-01
**CDH5**	0.24	6.32E-01	1.00E+00	0.27	6.67E-01	9.51E-01
**AC073415.1**	-1.01	3.51E-01	1.00E+00	0.42	6.67E-01	9.51E-01
**AC068533.3**	4.47	7.17E-02	1.00E+00	-0.65	6.67E-01	9.51E-01
**XIRP1**	3.00	1.11E-01	1.00E+00	1.37	6.67E-01	9.51E-01
**PEX6**	-0.25	3.59E-01	1.00E+00	-0.24	6.67E-01	9.51E-01
**MYH15**	-0.97	2.05E-01	1.00E+00	-0.31	6.67E-01	9.51E-01
**AGO2**	0.08	8.31E-01	1.00E+00	0.14	6.67E-01	9.51E-01
**ATP2B1**	-0.05	8.22E-01	1.00E+00	0.12	6.67E-01	9.51E-01
**FAAP100**	-0.48	5.64E-02	1.00E+00	0.22	6.67E-01	9.51E-01
**NSMCE1**	-0.02	8.99E-01	1.00E+00	-0.09	6.67E-01	9.51E-01
**GINS3**	-0.13	6.76E-01	1.00E+00	0.15	6.67E-01	9.51E-01
**AL118522.1**	-0.41	6.36E-01	1.00E+00	-0.31	6.67E-01	9.51E-01
**AL158827.2**	-1.37	4.55E-01	1.00E+00	-0.66	6.67E-01	9.51E-01

**BMP6**	0.29	5.84E-01	1.00E+00	0.31	6.67E-01	9.51E-01
**LAMTOR1**	0.03	8.59E-01	1.00E+00	0.07	6.67E-01	9.51E-01
**ANKS1B**	-0.64	3.02E-01	1.00E+00	-0.26	6.67E-01	9.51E-01
**ATP1A4**	-2.39	2.25E-01	1.00E+00	-0.48	6.67E-01	9.51E-01
**KRTAP4-9**	-19.72	5.40E-09	2.68E-06	-1.38	6.67E-01	9.51E-01
**LINC02247**	0.47	3.85E-01	1.00E+00	-0.28	6.67E-01	9.51E-01
**FRMD6-AS2**	0.06	9.71E-01	1.00E+00	0.37	6.67E-01	9.51E-01
**NTNG1**	-1.85	6.64E-03	4.94E-01	0.34	6.67E-01	9.51E-01
**ACSL5**	0.12	7.67E-01	1.00E+00	0.19	6.67E-01	9.51E-01
**AC139099.1**	-2.36	3.16E-01	1.00E+00	0.42	6.67E-01	9.51E-01
**SCN3A**	1.01	1.76E-01	1.00E+00	0.33	6.68E-01	9.51E-01
**AK6P1**	NA	NA	NA	0.64	6.68E-01	9.51E-01
**SLC25A14**	0.18	5.59E-01	1.00E+00	-0.13	6.68E-01	9.51E-01
**NEIL1**	0.59	7.75E-02	1.00E+00	-0.18	6.68E-01	9.51E-01
**ACKR3**	0.23	5.31E-01	1.00E+00	-0.33	6.68E-01	9.51E-01
**ERVFRD-1**	1.55	2.66E-01	1.00E+00	0.46	6.68E-01	9.51E-01
**RAP1A**	0.08	7.36E-01	1.00E+00	0.11	6.68E-01	9.51E-01
**FXYD6**	0.06	8.89E-01	1.00E+00	-0.16	6.68E-01	9.51E-01
**USP47**	0.04	7.96E-01	1.00E+00	-0.08	6.68E-01	9.51E-01
**POMC**	-0.50	2.72E-01	1.00E+00	0.13	6.68E-01	9.51E-01
**8-Mar**	0.13	5.59E-01	1.00E+00	0.15	6.68E-01	9.51E-01
**CDH17**	-0.89	6.27E-01	1.00E+00	0.70	6.68E-01	9.51E-01
**TMEM231**	0.35	1.15E-01	1.00E+00	0.15	6.68E-01	9.51E-01
**VCP**	-0.03	8.65E-01	1.00E+00	0.07	6.68E-01	9.51E-01
**DLX3**	-0.08	8.54E-01	1.00E+00	-0.26	6.68E-01	9.51E-01
**GPC4**	-0.13	7.62E-01	1.00E+00	0.23	6.68E-01	9.51E-01
**G38113**	0.04	9.74E-01	1.00E+00	-0.43	6.68E-01	9.51E-01
**AL391244.2**	-0.74	2.42E-01	1.00E+00	-0.50	6.68E-01	9.51E-01
**WDR36**	0.17	5.29E-01	1.00E+00	-0.10	6.68E-01	9.51E-01
**CCT6P3**	0.46	2.98E-01	1.00E+00	-0.22	6.68E-01	9.51E-01
**MRPS18C**	0.42	1.79E-01	1.00E+00	0.22	6.68E-01	9.51E-01
**AC023078.1**	0.50	6.63E-01	1.00E+00	0.36	6.68E-01	9.51E-01

**AC019080.3**	1.09	4.55E-01	1.00E+00	-0.52	6.68E-01	9.51E-01
**RTL3**	-0.55	5.05E-01	1.00E+00	-0.45	6.68E-01	9.51E-01
**HSPA2**	-0.31	3.00E-01	1.00E+00	-0.12	6.68E-01	9.51E-01
**ZNF320**	0.82	3.10E-01	1.00E+00	0.26	6.68E-01	9.51E-01
**AC005911.1**	-1.26	1.83E-01	1.00E+00	0.24	6.68E-01	9.51E-01
**XLOC_011384**	0.81	4.25E-01	1.00E+00	0.37	6.69E-01	9.51E-01
**ARHGDIG**	-1.51	6.51E-01	1.00E+00	-0.51	6.69E-01	9.51E-01
**ANKRD39**	-0.10	7.97E-01	1.00E+00	-0.21	6.69E-01	9.52E-01
**PDIA6**	0.09	6.06E-01	1.00E+00	0.08	6.69E-01	9.52E-01
**PTK2**	0.15	4.81E-01	1.00E+00	0.11	6.69E-01	9.52E-01
**HSCB**	0.07	7.94E-01	1.00E+00	-0.10	6.69E-01	9.52E-01
**RUFY4**	-2.87	2.87E-01	1.00E+00	1.36	6.69E-01	9.52E-01
**RTF2**	-0.04	7.91E-01	1.00E+00	-0.08	6.69E-01	9.52E-01
**ZNF37A**	-0.01	9.67E-01	1.00E+00	0.11	6.69E-01	9.52E-01
**RPL7**	-0.08	8.22E-01	1.00E+00	0.17	6.69E-01	9.52E-01
**FAM151A**	-4.17	1.62E-03	2.24E-01	0.48	6.69E-01	9.52E-01
**EEF1E1**	0.10	8.42E-01	1.00E+00	-0.25	6.69E-01	9.52E-01
**MASP2**	0.19	8.54E-01	1.00E+00	-0.55	6.69E-01	9.52E-01
**INTS1**	-0.25	3.08E-01	1.00E+00	0.26	6.69E-01	9.52E-01
**GSAP**	0.57	1.31E-01	1.00E+00	0.17	6.69E-01	9.52E-01
**NBPF3**	-0.34	3.92E-01	1.00E+00	0.18	6.70E-01	9.52E-01
**RPL5P30**	0.59	6.08E-01	1.00E+00	0.36	6.70E-01	9.52E-01
**C4orf47**	-1.78	2.99E-03	2.87E-01	-0.25	6.70E-01	9.52E-01
**PCDHGC5**	-1.36	2.17E-01	1.00E+00	1.36	6.70E-01	9.52E-01
**SMARCA2**	-0.03	8.43E-01	1.00E+00	-0.13	6.70E-01	9.52E-01
**AC096887.2**	0.18	8.70E-01	1.00E+00	0.42	6.70E-01	9.52E-01
**AP001636.1**	-0.21	9.53E-01	1.00E+00	-0.39	6.70E-01	9.52E-01
**GTF2IP7**	-2.24	4.20E-01	1.00E+00	0.45	6.70E-01	9.52E-01
**ZDHHC19**	0.22	9.47E-01	1.00E+00	0.81	6.70E-01	9.52E-01
**CNGB1**	-4.49	2.72E-03	2.69E-01	1.35	6.70E-01	9.52E-01
**PROM1**	0.46	5.90E-01	1.00E+00	-1.28	6.70E-01	9.52E-01
**NBPF17P**	-0.56	8.73E-01	1.00E+00	-0.40	6.70E-01	9.52E-01

**PPTC7**	0.20	3.78E-01	1.00E+00	0.16	6.70E-01	9.52E-01
**BRINP1**	-0.77	5.43E-01	1.00E+00	0.50	6.70E-01	9.52E-01
**ASRGL1**	-0.08	9.24E-01	1.00E+00	-0.26	6.70E-01	9.52E-01
**RPS29P5**	-0.80	8.18E-01	1.00E+00	0.36	6.71E-01	9.52E-01
**ZSCAN12**	0.04	9.07E-01	1.00E+00	0.23	6.71E-01	9.52E-01
**KLHDC10**	0.36	2.02E-01	1.00E+00	0.13	6.71E-01	9.52E-01
**MRPS2**	0.17	5.10E-01	1.00E+00	0.09	6.71E-01	9.52E-01
**SCARB1**	0.22	4.34E-01	1.00E+00	-0.14	6.71E-01	9.52E-01
**COPS7A**	-0.04	7.96E-01	1.00E+00	-0.05	6.71E-01	9.52E-01
**RMRPP2**	0.10	8.57E-01	1.00E+00	0.39	6.71E-01	9.52E-01
**IFNLR1**	-0.12	7.67E-01	1.00E+00	-0.22	6.71E-01	9.52E-01
**RPL36AL**	-0.06	7.46E-01	1.00E+00	0.13	6.71E-01	9.52E-01
**C2orf70**	-0.56	6.37E-01	1.00E+00	0.54	6.71E-01	9.52E-01
**GRASP**	-0.15	6.80E-01	1.00E+00	0.37	6.71E-01	9.52E-01
**VPS28**	-0.15	4.85E-01	1.00E+00	0.07	6.71E-01	9.52E-01
**C12orf76**	0.03	9.13E-01	1.00E+00	-0.19	6.71E-01	9.52E-01
**IMPAD1**	0.15	5.65E-01	1.00E+00	-0.17	6.71E-01	9.52E-01
**AC022417.1**	-0.37	6.74E-01	1.00E+00	-0.38	6.71E-01	9.52E-01
**GOSR1**	-0.02	8.96E-01	1.00E+00	0.05	6.71E-01	9.52E-01
**GREB1L**	0.76	2.98E-01	1.00E+00	0.31	6.71E-01	9.52E-01
**TAF6L**	-0.31	1.84E-01	1.00E+00	-0.34	6.71E-01	9.52E-01
**NRDC**	0.12	7.00E-01	1.00E+00	0.06	6.71E-01	9.52E-01
**SRRM2-AS1**	0.22	7.87E-01	1.00E+00	-0.25	6.71E-01	9.52E-01
**MYNN**	0.03	9.17E-01	1.00E+00	-0.14	6.71E-01	9.52E-01
**POLA2**	0.16	7.24E-01	1.00E+00	0.13	6.71E-01	9.52E-01
**AL603750.1**	0.98	3.43E-01	1.00E+00	0.52	6.71E-01	9.52E-01
**MEMO1**	0.18	6.81E-01	1.00E+00	-0.17	6.71E-01	9.52E-01
**ACTL10**	-0.79	1.13E-01	1.00E+00	-0.28	6.71E-01	9.52E-01
**NAA50**	0.10	5.76E-01	1.00E+00	0.10	6.71E-01	9.52E-01
**HIST1H2BN**	0.67	3.06E-01	1.00E+00	0.20	6.72E-01	9.52E-01
**NRP1**	0.27	4.75E-01	1.00E+00	0.16	6.72E-01	9.52E-01
**PMP2**	-0.85	1.10E-01	1.00E+00	0.32	6.72E-01	9.52E-01

**RFT1**	0.03	8.70E-01	1.00E+00	0.09	6.72E-01	9.53E-01
**AP001453.2**	NA	NA	NA	0.41	6.72E-01	9.53E-01
**LINC02363**	1.34	4.10E-01	1.00E+00	-0.40	6.72E-01	9.53E-01
**AC026271.3**	0.38	3.46E-01	1.00E+00	0.28	6.72E-01	9.53E-01
**GAPDHP35**	NA	NA	NA	0.56	6.72E-01	9.53E-01
**PTK6**	0.06	8.96E-01	1.00E+00	-0.22	6.72E-01	9.53E-01
**EHD1**	-0.23	3.46E-01	1.00E+00	0.19	6.72E-01	9.53E-01
**XLOC_013243**	0.92	7.83E-01	1.00E+00	-1.35	6.72E-01	9.53E-01
**LIN52**	0.10	6.42E-01	1.00E+00	0.12	6.72E-01	9.53E-01
**VARS2**	-0.06	8.06E-01	1.00E+00	0.25	6.72E-01	9.53E-01
**RBM3**	-0.19	4.67E-01	1.00E+00	-0.08	6.72E-01	9.53E-01
**PPP4R3A**	-0.04	8.79E-01	1.00E+00	-0.16	6.72E-01	9.53E-01
**NQO2**	0.16	5.77E-01	1.00E+00	-0.13	6.72E-01	9.53E-01
**GPR37L1**	-3.41	8.17E-02	1.00E+00	-0.47	6.72E-01	9.53E-01
**ACSL6**	0.50	6.22E-01	1.00E+00	0.39	6.72E-01	9.53E-01
**ZNF793-AS1**	-0.49	4.66E-01	1.00E+00	-0.23	6.72E-01	9.53E-01
**PEAR1**	0.29	5.40E-01	1.00E+00	0.30	6.72E-01	9.53E-01
**KLHL36**	0.37	2.98E-01	1.00E+00	0.12	6.73E-01	9.53E-01
**SNHG18**	-0.14	7.19E-01	1.00E+00	-0.14	6.73E-01	9.53E-01
**DAZAP2**	-0.01	9.55E-01	1.00E+00	-0.13	6.73E-01	9.53E-01
**PLEKHH2**	-0.06	8.71E-01	1.00E+00	-0.19	6.73E-01	9.53E-01
**MACF1**	-0.37	1.10E-01	1.00E+00	0.18	6.73E-01	9.53E-01
**AKR1C1**	1.29	8.17E-02	1.00E+00	-0.24	6.73E-01	9.53E-01
**VCPIP1**	-0.09	6.25E-01	1.00E+00	0.07	6.73E-01	9.53E-01
**RPL14**	-0.10	7.22E-01	1.00E+00	0.12	6.73E-01	9.53E-01
**KIF2C**	0.22	4.49E-01	1.00E+00	-0.23	6.73E-01	9.53E-01
**CPOX**	0.17	4.72E-01	1.00E+00	0.23	6.73E-01	9.53E-01
**CLK3**	-0.05	7.44E-01	1.00E+00	-0.09	6.73E-01	9.53E-01
**RHBDD1**	-0.19	2.96E-01	1.00E+00	-0.08	6.73E-01	9.53E-01
**XLOC_009135**	0.71	4.95E-01	1.00E+00	0.36	6.73E-01	9.53E-01
**PDXP**	-0.13	7.94E-01	1.00E+00	0.49	6.73E-01	9.53E-01
**SREK1**	0.16	5.31E-01	1.00E+00	-0.09	6.73E-01	9.53E-01

**AC087163.2**	-2.73	6.41E-02	1.00E+00	0.63	6.73E-01	9.53E-01
**THUMPD1**	0.16	3.87E-01	1.00E+00	-0.09	6.73E-01	9.53E-01
**GPR88**	1.30	3.11E-01	1.00E+00	0.49	6.73E-01	9.53E-01
**ALOX15**	-0.57	1.25E-01	1.00E+00	-0.34	6.73E-01	9.53E-01
**POLE**	-0.16	5.08E-01	1.00E+00	0.22	6.73E-01	9.53E-01
**QARS**	-0.27	3.80E-01	1.00E+00	0.08	6.73E-01	9.53E-01
**BUD31**	-0.09	6.52E-01	1.00E+00	0.14	6.74E-01	9.53E-01
**BSCL2**	0.11	8.75E-01	1.00E+00	0.23	6.74E-01	9.53E-01
**BID**	-0.14	5.95E-01	1.00E+00	0.12	6.74E-01	9.53E-01
**CORO2A**	0.35	3.37E-01	1.00E+00	0.13	6.74E-01	9.53E-01
**Z97055.2**	-0.46	6.79E-01	1.00E+00	-0.45	6.74E-01	9.53E-01
**SLC25A27**	0.31	6.10E-01	1.00E+00	-0.35	6.74E-01	9.53E-01
**MID1IP1**	0.23	2.86E-01	1.00E+00	0.18	6.74E-01	9.53E-01
**RBSN**	-0.18	4.62E-01	1.00E+00	-0.09	6.74E-01	9.53E-01
**METAP1**	0.07	7.07E-01	1.00E+00	0.08	6.74E-01	9.53E-01
**SLC35E2B**	0.10	7.35E-01	1.00E+00	0.20	6.74E-01	9.53E-01
**CBFA2T3**	-0.13	7.16E-01	1.00E+00	-0.18	6.74E-01	9.53E-01
**CCDC115**	0.09	5.83E-01	1.00E+00	-0.11	6.74E-01	9.53E-01
**AP000785.2**	0.48	6.52E-01	1.00E+00	0.40	6.74E-01	9.53E-01
**MON2**	0.08	7.17E-01	1.00E+00	-0.11	6.74E-01	9.53E-01
**SH3BP4**	-0.12	7.36E-01	1.00E+00	-0.18	6.74E-01	9.53E-01
**G15375**	-0.48	3.20E-01	1.00E+00	0.37	6.74E-01	9.53E-01
**PRKD1**	0.93	2.71E-02	9.60E-01	-0.28	6.74E-01	9.53E-01
**ENGASE**	0.14	6.19E-01	1.00E+00	0.29	6.74E-01	9.53E-01
**ENO1**	-0.24	2.80E-01	1.00E+00	0.10	6.75E-01	9.53E-01
**LDHC**	-1.66	1.67E-02	8.13E-01	0.42	6.75E-01	9.53E-01
**HACD1**	-0.51	2.82E-01	1.00E+00	-0.18	6.75E-01	9.53E-01
**RPL21P131**	NA	NA	NA	0.43	6.75E-01	9.53E-01
**ZNRF3**	-0.17	6.30E-01	1.00E+00	-0.14	6.75E-01	9.53E-01
**DCLRE1A**	0.26	3.52E-01	1.00E+00	0.14	6.75E-01	9.53E-01
**AC117382.1**	1.05	3.89E-01	1.00E+00	-0.19	6.75E-01	9.53E-01
**LAMB4**	0.21	5.25E-01	1.00E+00	-0.28	6.75E-01	9.53E-01

**ZFY**	0.53	4.17E-01	1.00E+00	-0.38	6.75E-01	9.53E-01
**UBE2Z**	-0.02	9.20E-01	1.00E+00	0.20	6.75E-01	9.53E-01
**AC139769.2**	-0.33	7.93E-01	1.00E+00	-0.29	6.75E-01	9.53E-01
**IGKV1-13**	1.78	5.77E-01	1.00E+00	1.35	6.75E-01	9.53E-01
**RPL18AP3**	-0.25	4.47E-01	1.00E+00	0.28	6.75E-01	9.53E-01
**ANKRD35**	0.11	8.14E-01	1.00E+00	0.13	6.75E-01	9.53E-01
**PRPF4**	-0.11	5.66E-01	1.00E+00	0.07	6.75E-01	9.53E-01
**XLOC_003787**	-2.05	3.04E-01	1.00E+00	0.47	6.75E-01	9.53E-01
**ISYNA1**	-0.47	2.52E-01	1.00E+00	-0.20	6.75E-01	9.53E-01
**CYP1B1-AS1**	-1.01	2.32E-01	1.00E+00	-0.23	6.75E-01	9.53E-01
**ACSM5**	1.59	1.43E-02	7.51E-01	0.25	6.75E-01	9.53E-01
**HPRT1**	-0.19	3.71E-01	1.00E+00	-0.10	6.75E-01	9.54E-01
**AL162390.1**	1.61	4.79E-01	1.00E+00	0.48	6.75E-01	9.54E-01
**IGHEP1**	-1.57	4.68E-01	1.00E+00	-1.34	6.75E-01	9.54E-01
**ZBTB48**	-0.16	5.36E-01	1.00E+00	0.10	6.76E-01	9.54E-01
**BACH1-IT2**	-0.09	9.43E-01	1.00E+00	-0.49	6.76E-01	9.54E-01
**FABP5P11**	-1.52	6.60E-01	1.00E+00	0.28	6.76E-01	9.54E-01
**LINC01978**	-1.00	5.86E-01	1.00E+00	0.79	6.76E-01	9.54E-01
**RPL14P3**	-0.47	8.70E-01	1.00E+00	-0.37	6.76E-01	9.54E-01
**AC108693.1**	-0.09	9.68E-01	1.00E+00	0.41	6.76E-01	9.54E-01
**LINC02169**	-1.06	3.31E-01	1.00E+00	-1.33	6.76E-01	9.54E-01
**GABBR2**	-1.26	6.67E-01	1.00E+00	-0.39	6.76E-01	9.54E-01
**AC092611.3**	0.75	2.01E-02	8.84E-01	0.22	6.76E-01	9.54E-01
**AC068025.1**	-2.08	2.53E-01	1.00E+00	0.40	6.76E-01	9.54E-01
**SNX31**	-0.19	8.28E-01	1.00E+00	-0.42	6.76E-01	9.54E-01
**ATP5PFP1**	NA	NA	NA	0.40	6.76E-01	9.54E-01
**CASP17P**	-0.34	5.13E-01	1.00E+00	-0.16	6.76E-01	9.54E-01
**G14979**	-1.22	5.87E-01	1.00E+00	-0.49	6.76E-01	9.54E-01
**NOXA1**	-0.08	7.76E-01	1.00E+00	0.38	6.76E-01	9.54E-01
**LIX1**	-2.59	5.83E-02	1.00E+00	0.49	6.76E-01	9.54E-01
**ALG1L12P**	-2.23	7.92E-02	1.00E+00	0.28	6.76E-01	9.54E-01
**AKR1A1**	-0.20	3.59E-01	1.00E+00	0.08	6.77E-01	9.54E-01

**AC005906.2**	-2.30	2.35E-01	1.00E+00	0.57	6.77E-01	9.54E-01
**ZNF251**	-0.02	9.39E-01	1.00E+00	0.12	6.77E-01	9.54E-01
**MT1F**	-0.38	2.25E-01	1.00E+00	-0.12	6.77E-01	9.54E-01
**AC145676.1**	-1.01	5.07E-01	1.00E+00	0.81	6.77E-01	9.54E-01
**UTP25**	0.00	9.85E-01	1.00E+00	-0.07	6.77E-01	9.54E-01
**PLA2G2D**	-1.30	3.28E-01	1.00E+00	1.32	6.77E-01	9.54E-01
**COG7**	0.13	5.48E-01	1.00E+00	-0.07	6.77E-01	9.54E-01
**CENPF**	-0.36	2.02E-01	1.00E+00	0.18	6.77E-01	9.54E-01
**SVIL-AS1**	0.39	5.83E-02	1.00E+00	-0.10	6.77E-01	9.54E-01
**FMN2**	-0.51	5.07E-01	1.00E+00	-0.41	6.77E-01	9.54E-01
**EYS**	1.31	1.70E-01	1.00E+00	-0.61	6.77E-01	9.54E-01
**AC067838.1**	0.14	8.30E-01	1.00E+00	0.22	6.77E-01	9.54E-01
**DUSP13**	-0.28	5.79E-01	1.00E+00	-0.31	6.77E-01	9.54E-01
**CLC**	1.49	5.13E-01	1.00E+00	0.75	6.77E-01	9.54E-01
**BORA**	-0.35	4.06E-01	1.00E+00	0.15	6.77E-01	9.54E-01
**A1BG-AS1**	-1.49	9.63E-02	1.00E+00	0.38	6.77E-01	9.54E-01
**RP11-680G24.5**	0.82	5.08E-01	1.00E+00	-0.30	6.77E-01	9.54E-01
**ANKMY2**	0.00	9.91E-01	1.00E+00	-0.11	6.77E-01	9.54E-01
**AC023632.6**	0.43	4.01E-01	1.00E+00	0.27	6.77E-01	9.54E-01
**TOMM7**	0.06	8.51E-01	1.00E+00	-0.09	6.77E-01	9.54E-01
**SOX2**	-0.66	3.52E-01	1.00E+00	0.35	6.78E-01	9.54E-01
**G33207**	-0.16	7.89E-01	1.00E+00	-0.33	6.78E-01	9.54E-01
**PRCD**	-0.11	8.13E-01	1.00E+00	0.22	6.78E-01	9.55E-01
**ACACB**	1.78	1.75E-02	8.33E-01	0.29	6.78E-01	9.55E-01
**HNRNPU**	-0.23	6.53E-02	1.00E+00	-0.17	6.78E-01	9.55E-01
**SLC16A5**	0.04	9.22E-01	1.00E+00	-0.12	6.78E-01	9.55E-01
**AC009090.1**	2.72	1.85E-02	8.53E-01	0.44	6.78E-01	9.55E-01
**AC109635.4**	2.74	4.20E-01	1.00E+00	0.62	6.78E-01	9.55E-01
**RP11-640M9.1**	0.31	3.97E-01	1.00E+00	0.10	6.78E-01	9.55E-01
**CLGN**	0.12	9.05E-01	1.00E+00	-0.29	6.78E-01	9.55E-01
**SERINC5**	0.10	7.31E-01	1.00E+00	0.15	6.78E-01	9.55E-01
**NT5C**	-0.05	8.63E-01	1.00E+00	-0.10	6.78E-01	9.55E-01

**RPL7AP14**	NA	NA	NA	0.55	6.78E-01	9.55E-01
**C9orf43**	0.04	9.53E-01	1.00E+00	-0.29	6.78E-01	9.55E-01
**SLC12A4**	0.18	5.68E-01	1.00E+00	0.20	6.79E-01	9.55E-01
**DBR1**	0.06	7.21E-01	1.00E+00	0.07	6.79E-01	9.55E-01
**SGK3**	0.33	3.23E-01	1.00E+00	-0.13	6.79E-01	9.55E-01
**AC110285.7**	-0.22	7.22E-01	1.00E+00	0.35	6.79E-01	9.55E-01
**RPS27L**	0.20	3.73E-01	1.00E+00	-0.14	6.79E-01	9.55E-01
**CSKMT**	0.06	8.99E-01	1.00E+00	-0.23	6.79E-01	9.55E-01
**TMED10**	0.24	8.83E-02	1.00E+00	-0.09	6.79E-01	9.55E-01
**UBN2**	0.16	6.14E-01	1.00E+00	-0.16	6.79E-01	9.55E-01
**AC078923.1**	-1.40	4.49E-01	1.00E+00	0.44	6.79E-01	9.55E-01
**CNTN4**	0.15	8.14E-01	1.00E+00	0.25	6.79E-01	9.55E-01
**IGF1**	0.46	5.32E-01	1.00E+00	0.23	6.79E-01	9.55E-01
**FAM124B**	0.80	2.07E-02	8.94E-01	-0.19	6.79E-01	9.55E-01
**PANK3**	0.14	5.66E-01	1.00E+00	0.08	6.79E-01	9.55E-01
**ANKRD17**	-0.17	3.72E-01	1.00E+00	0.11	6.79E-01	9.55E-01
**NEO1**	0.24	3.28E-01	1.00E+00	0.17	6.79E-01	9.55E-01
**AL139220.2**	0.14	8.24E-01	1.00E+00	-0.21	6.79E-01	9.55E-01
**IGKV1D-43**	NA	NA	NA	1.33	6.79E-01	9.55E-01
**ATF7IP**	-0.08	7.62E-01	1.00E+00	-0.09	6.79E-01	9.55E-01
**LRRC10B**	-0.35	4.02E-01	1.00E+00	-0.42	6.79E-01	9.55E-01
**ARSG**	-0.58	9.68E-02	1.00E+00	-0.09	6.80E-01	9.55E-01
**DNAJA1**	-0.08	7.94E-01	1.00E+00	0.12	6.80E-01	9.55E-01
**AC027801.1**	-0.35	8.16E-01	1.00E+00	0.38	6.80E-01	9.55E-01
**GRAMD1C**	0.29	5.40E-01	1.00E+00	0.15	6.80E-01	9.55E-01
**AC010980.1**	0.38	5.71E-01	1.00E+00	0.27	6.80E-01	9.55E-01
**XLOC_005479**	3.27	1.84E-01	1.00E+00	-0.30	6.80E-01	9.55E-01
**PRND**	0.03	9.76E-01	1.00E+00	0.45	6.80E-01	9.55E-01
**AL591866.1**	-0.65	8.31E-01	1.00E+00	0.25	6.80E-01	9.55E-01
**EIF1B-AS1**	0.37	4.13E-01	1.00E+00	-0.18	6.80E-01	9.55E-01
**ZNF678**	0.08	8.28E-01	1.00E+00	0.14	6.80E-01	9.55E-01
**TSPYL4**	-0.40	8.20E-02	1.00E+00	0.12	6.80E-01	9.55E-01

**AC090181.2**	-1.70	3.01E-01	1.00E+00	-0.67	6.80E-01	9.55E-01
**PKNOX2**	-0.70	3.13E-02	1.00E+00	0.23	6.80E-01	9.55E-01
**RPP38**	0.04	8.87E-01	1.00E+00	-0.15	6.80E-01	9.55E-01
**C19orf48**	-0.31	2.40E-01	1.00E+00	0.15	6.80E-01	9.55E-01
**RLIMP1**	-1.70	6.01E-01	1.00E+00	0.48	6.80E-01	9.55E-01
**AC069155.1**	-1.77	2.53E-01	1.00E+00	0.43	6.80E-01	9.55E-01
**RPUSD2**	-0.07	7.59E-01	1.00E+00	0.09	6.80E-01	9.55E-01
**AP1M2**	-0.05	8.63E-01	1.00E+00	-0.17	6.80E-01	9.55E-01
**NUS1P1**	-0.09	9.32E-01	1.00E+00	0.24	6.80E-01	9.55E-01
**SLC2A5**	0.14	8.11E-01	1.00E+00	0.26	6.80E-01	9.55E-01
**TTC23**	0.38	1.96E-01	1.00E+00	0.10	6.80E-01	9.55E-01
**PTRH2**	0.06	7.79E-01	1.00E+00	0.10	6.81E-01	9.56E-01
**AL135744.1**	-2.99	2.53E-01	1.00E+00	0.35	6.81E-01	9.56E-01
**POC1B**	0.21	3.34E-01	1.00E+00	-0.11	6.81E-01	9.56E-01
**G29144**	-0.78	6.49E-01	1.00E+00	-0.47	6.81E-01	9.56E-01
**SPATA2L**	-0.16	5.41E-01	1.00E+00	0.21	6.81E-01	9.56E-01
**AFG1L**	0.07	8.54E-01	1.00E+00	0.11	6.81E-01	9.56E-01
**ITGA9**	0.24	5.36E-01	1.00E+00	0.20	6.81E-01	9.56E-01
**FRG1-DT**	1.04	2.72E-01	1.00E+00	-0.32	6.81E-01	9.56E-01
**AL132712.1**	-0.65	3.77E-01	1.00E+00	-0.40	6.81E-01	9.56E-01
**HIGD1AP14**	NA	NA	NA	-0.31	6.81E-01	9.56E-01
**PSMD5**	0.16	3.86E-01	1.00E+00	-0.08	6.81E-01	9.56E-01
**AL392046.2**	-0.14	7.56E-01	1.00E+00	0.24	6.81E-01	9.56E-01
**C5**	-0.01	9.75E-01	1.00E+00	-0.26	6.81E-01	9.56E-01
**GDF10**	0.58	4.49E-01	1.00E+00	-0.31	6.81E-01	9.56E-01
**NDUFA9**	-0.08	8.05E-01	1.00E+00	-0.20	6.81E-01	9.56E-01
**ARL13B**	0.24	4.38E-01	1.00E+00	-0.10	6.81E-01	9.56E-01
**TSPAN17**	-0.25	2.80E-01	1.00E+00	0.21	6.81E-01	9.56E-01
**AC004067.1**	-0.89	2.58E-01	1.00E+00	0.25	6.82E-01	9.56E-01
**PPT1**	-0.02	9.13E-01	1.00E+00	0.09	6.82E-01	9.56E-01
**COL6A5**	0.39	5.61E-01	1.00E+00	0.38	6.82E-01	9.56E-01
**AF274858.1**	0.08	9.05E-01	1.00E+00	-0.16	6.82E-01	9.56E-01

**RBMX**	-0.09	5.47E-01	1.00E+00	-0.07	6.82E-01	9.56E-01
**XRN1**	0.23	4.70E-01	1.00E+00	0.11	6.82E-01	9.56E-01
**SCART1**	-0.73	3.88E-01	1.00E+00	-0.31	6.82E-01	9.56E-01
**PITX2**	-0.04	9.65E-01	1.00E+00	0.31	6.82E-01	9.56E-01
**VMAC**	-0.40	2.07E-01	1.00E+00	-0.11	6.82E-01	9.56E-01
**AC034102.1**	0.52	6.92E-01	1.00E+00	0.18	6.82E-01	9.56E-01
**Z97353.1**	-2.23	5.12E-01	1.00E+00	0.32	6.82E-01	9.56E-01
**LYRM4**	0.01	9.72E-01	1.00E+00	0.08	6.82E-01	9.56E-01
**ADAMTS14**	-0.61	3.29E-01	1.00E+00	1.29	6.82E-01	9.56E-01
**MTMR6**	0.36	8.54E-02	1.00E+00	-0.12	6.82E-01	9.56E-01
**GPS2P1**	1.50	6.62E-01	1.00E+00	-0.45	6.82E-01	9.56E-01
**XLOC_014219**	-0.34	7.26E-01	1.00E+00	-0.66	6.82E-01	9.56E-01
**CLUH**	-0.28	3.01E-01	1.00E+00	-0.25	6.82E-01	9.56E-01
**IGHEP2**	-1.57	4.23E-01	1.00E+00	-0.52	6.82E-01	9.56E-01
**AC079145.1**	-0.44	8.90E-01	1.00E+00	0.52	6.83E-01	9.56E-01
**IL31RA**	-0.04	9.65E-01	1.00E+00	-0.42	6.83E-01	9.56E-01
**SYNGR3**	-1.59	8.23E-02	1.00E+00	-0.33	6.83E-01	9.56E-01
**CREB3**	0.05	8.80E-01	1.00E+00	0.09	6.83E-01	9.56E-01
**AC026803.2**	-0.28	5.89E-01	1.00E+00	-0.46	6.83E-01	9.56E-01
**XLOC_006088**	-2.72	3.34E-01	1.00E+00	0.55	6.83E-01	9.56E-01
**AC135048.3**	-2.00	1.85E-01	1.00E+00	0.39	6.83E-01	9.56E-01
**GJC2**	-0.07	8.41E-01	1.00E+00	0.32	6.83E-01	9.56E-01
**EEF1AKMT1**	0.04	8.89E-01	1.00E+00	-0.15	6.83E-01	9.56E-01
**AC004490.1**	0.83	4.44E-01	1.00E+00	0.36	6.83E-01	9.56E-01
**G16011**	-0.72	6.17E-01	1.00E+00	0.44	6.83E-01	9.56E-01
**CPNE6**	-1.57	1.71E-01	1.00E+00	-0.35	6.83E-01	9.56E-01
**DSC1**	0.09	8.69E-01	1.00E+00	0.28	6.83E-01	9.56E-01
**DDX31**	-0.02	9.43E-01	1.00E+00	0.17	6.83E-01	9.56E-01
**SEMA6A-AS1**	1.26	3.91E-01	1.00E+00	0.43	6.83E-01	9.56E-01
**LRRC45**	-0.26	3.63E-01	1.00E+00	0.23	6.83E-01	9.56E-01
**MNS1**	-0.17	6.63E-01	1.00E+00	-0.14	6.83E-01	9.56E-01
**ADAMTS4**	-2.06	9.13E-02	1.00E+00	0.38	6.83E-01	9.56E-01

**VIPR2**	0.02	9.80E-01	1.00E+00	0.43	6.83E-01	9.56E-01
**CPLX1**	-1.15	1.20E-01	1.00E+00	0.46	6.83E-01	9.56E-01
**TRIB1**	-0.27	4.37E-01	1.00E+00	-0.23	6.83E-01	9.56E-01
**CLIP1**	-0.28	3.39E-01	1.00E+00	-0.09	6.83E-01	9.56E-01
**FAM156B**	0.52	2.74E-01	1.00E+00	0.13	6.83E-01	9.56E-01
**USP2-AS1**	-0.16	9.09E-01	1.00E+00	-0.35	6.83E-01	9.56E-01
**DAP3**	0.05	7.91E-01	1.00E+00	0.06	6.83E-01	9.56E-01
**ZFR2**	-2.88	3.04E-02	1.00E+00	-0.40	6.83E-01	9.56E-01
**AL139287.1**	-0.75	4.53E-01	1.00E+00	0.36	6.83E-01	9.56E-01
**VWA1**	-0.31	3.72E-01	1.00E+00	0.33	6.84E-01	9.56E-01
**LINC01135**	-0.64	4.88E-01	1.00E+00	-0.33	6.84E-01	9.56E-01
**TMEM30A**	0.20	4.60E-01	1.00E+00	0.11	6.84E-01	9.56E-01
**SCAF8**	-0.35	1.47E-01	1.00E+00	0.12	6.84E-01	9.56E-01
**STYK1**	-1.34	1.17E-02	6.70E-01	-0.32	6.84E-01	9.56E-01
**BTC**	0.18	7.79E-01	1.00E+00	-0.27	6.84E-01	9.56E-01
**C9orf38**	1.04	5.21E-01	1.00E+00	0.35	6.84E-01	9.56E-01
**CCND2**	0.45	2.38E-01	1.00E+00	-0.13	6.84E-01	9.56E-01
**SOX7**	-0.54	1.13E-02	6.59E-01	-0.16	6.84E-01	9.56E-01
**ABCC11**	-0.03	9.78E-01	1.00E+00	-0.39	6.84E-01	9.56E-01
**XLOC_002997**	1.14	6.01E-01	1.00E+00	0.61	6.84E-01	9.56E-01
**ALG3**	-0.51	3.90E-02	1.00E+00	0.08	6.84E-01	9.56E-01
**PHYHIPL**	-0.83	4.31E-01	1.00E+00	-0.32	6.84E-01	9.56E-01
**AC005776.2**	0.04	9.68E-01	1.00E+00	0.47	6.84E-01	9.56E-01
**MCTP2**	0.06	8.54E-01	1.00E+00	0.21	6.84E-01	9.56E-01
**SPDYA**	-1.94	2.89E-01	1.00E+00	0.26	6.84E-01	9.56E-01
**CCKBR**	-1.34	1.47E-02	7.60E-01	-0.35	6.84E-01	9.56E-01
**ZNF724**	0.48	5.09E-01	1.00E+00	-0.23	6.84E-01	9.56E-01
**AC090970.1**	1.55	4.69E-01	1.00E+00	0.35	6.84E-01	9.56E-01
**ICK**	0.03	9.01E-01	1.00E+00	0.09	6.84E-01	9.56E-01
**RPL3P9**	NA	NA	NA	-0.34	6.84E-01	9.56E-01
**AL391244.1**	-0.14	5.85E-01	1.00E+00	0.11	6.84E-01	9.56E-01
**MTREX**	0.13	5.62E-01	1.00E+00	-0.08	6.85E-01	9.56E-01

**AP000811.1**	0.25	9.10E-01	1.00E+00	0.47	6.85E-01	9.56E-01
**NOL6**	-0.26	3.17E-01	1.00E+00	0.22	6.85E-01	9.56E-01
**RASA3**	0.48	1.38E-01	1.00E+00	0.23	6.85E-01	9.57E-01
**AC123912.1**	1.47	1.76E-01	1.00E+00	-0.30	6.85E-01	9.57E-01
**PDE1B**	0.48	4.14E-01	1.00E+00	0.34	6.85E-01	9.57E-01
**MYO1A**	-3.09	2.29E-02	9.12E-01	-0.39	6.85E-01	9.57E-01
**DYNC2LI1**	0.31	2.18E-01	1.00E+00	-0.14	6.85E-01	9.57E-01
**GRPR**	0.80	6.50E-01	1.00E+00	-0.61	6.85E-01	9.57E-01
**TREM2**	-0.60	3.64E-01	1.00E+00	0.44	6.85E-01	9.57E-01
**UBFD1**	0.24	2.82E-01	1.00E+00	0.21	6.85E-01	9.57E-01
**PTPDC1**	0.13	7.20E-01	1.00E+00	-0.24	6.85E-01	9.57E-01
**ANP32BP1**	-0.55	8.75E-01	1.00E+00	-0.29	6.85E-01	9.57E-01
**TPO**	0.48	5.03E-01	1.00E+00	-0.31	6.85E-01	9.57E-01
**C4orf3**	0.13	3.68E-01	1.00E+00	-0.12	6.85E-01	9.57E-01
**FBXW11**	0.23	2.34E-01	1.00E+00	0.10	6.85E-01	9.57E-01
**POLB**	-0.01	9.64E-01	1.00E+00	0.12	6.85E-01	9.57E-01
**KCNE1**	-2.59	5.32E-02	1.00E+00	-0.52	6.85E-01	9.57E-01
**TMEM108-AS1**	0.43	9.03E-01	1.00E+00	-0.75	6.85E-01	9.57E-01
**MYOM2**	0.29	2.76E-01	1.00E+00	-0.12	6.85E-01	9.57E-01
**AC092573.2**	0.05	9.76E-01	1.00E+00	0.51	6.85E-01	9.57E-01
**AC109587.1**	0.48	4.47E-01	1.00E+00	-0.30	6.85E-01	9.57E-01
**PDLIM1P4**	-0.81	6.00E-01	1.00E+00	0.25	6.85E-01	9.57E-01
**ST6GALNAC1**	-0.39	5.32E-01	1.00E+00	0.25	6.85E-01	9.57E-01
**AC091057.1**	0.46	4.06E-01	1.00E+00	0.39	6.85E-01	9.57E-01
**CLIP4**	0.22	3.57E-01	1.00E+00	-0.09	6.86E-01	9.57E-01
**NPNT**	-0.15	6.70E-01	1.00E+00	-0.20	6.86E-01	9.57E-01
**MED10**	0.14	5.00E-01	1.00E+00	-0.10	6.86E-01	9.57E-01
**RNPS1**	-0.04	8.27E-01	1.00E+00	-0.07	6.86E-01	9.57E-01
**GLYR1**	-0.06	7.36E-01	1.00E+00	0.18	6.86E-01	9.57E-01
**G40907**	-0.04	9.76E-01	1.00E+00	-0.37	6.86E-01	9.57E-01
**DDX11-AS1**	-1.69	1.54E-01	1.00E+00	0.43	6.86E-01	9.57E-01
**PNPLA8**	0.15	5.23E-01	1.00E+00	-0.09	6.86E-01	9.57E-01

**C15orf62**	0.34	5.02E-01	1.00E+00	-0.26	6.86E-01	9.57E-01
**KCNJ2**	0.02	9.54E-01	1.00E+00	-0.16	6.86E-01	9.57E-01
**PRR4**	-1.64	6.28E-01	1.00E+00	-1.27	6.86E-01	9.57E-01
**RPL13**	-0.17	5.55E-01	1.00E+00	0.12	6.86E-01	9.57E-01
**MFSD6L**	0.13	8.11E-01	1.00E+00	0.98	6.86E-01	9.57E-01
**GTF2H2C**	-0.17	6.29E-01	1.00E+00	-0.12	6.86E-01	9.57E-01
**AL117339.5**	0.56	8.57E-01	1.00E+00	-1.27	6.86E-01	9.57E-01
**TMEM19**	0.29	2.50E-01	1.00E+00	-0.14	6.86E-01	9.57E-01
**AC021188.1**	-0.08	9.00E-01	1.00E+00	-0.33	6.86E-01	9.57E-01
**AC018752.1**	-2.07	1.01E-01	1.00E+00	-0.34	6.86E-01	9.57E-01
**SHANK2**	-0.07	9.29E-01	1.00E+00	0.30	6.86E-01	9.57E-01
**ZNF587**	-0.11	7.52E-01	1.00E+00	0.13	6.86E-01	9.57E-01
**AC096540.1**	1.65	2.74E-01	1.00E+00	-0.37	6.86E-01	9.57E-01
**AC087276.1**	0.58	4.23E-01	1.00E+00	0.27	6.86E-01	9.57E-01
**CCDC116**	-2.64	1.62E-01	1.00E+00	0.56	6.87E-01	9.57E-01
**PRRG4**	0.25	5.28E-01	1.00E+00	-0.17	6.87E-01	9.57E-01
**ZNF141**	-0.35	3.74E-01	1.00E+00	-0.17	6.87E-01	9.57E-01
**CNOT1**	0.22	3.10E-01	1.00E+00	0.14	6.87E-01	9.57E-01
**EPS8L2**	-0.32	3.88E-01	1.00E+00	0.18	6.87E-01	9.57E-01
**FAM86DP**	0.10	7.17E-01	1.00E+00	0.11	6.87E-01	9.57E-01
**LINC01311**	-2.26	5.00E-02	1.00E+00	-0.39	6.87E-01	9.57E-01
**AL135818.2**	-0.35	7.81E-01	1.00E+00	0.64	6.87E-01	9.57E-01
**C1orf21**	0.02	9.46E-01	1.00E+00	-0.11	6.87E-01	9.57E-01
**CTNNBL1**	0.00	9.90E-01	1.00E+00	0.05	6.87E-01	9.57E-01
**AL121944.1**	0.34	5.43E-01	1.00E+00	-0.17	6.87E-01	9.57E-01
**ZDHHC11B**	-0.23	7.42E-01	1.00E+00	0.31	6.87E-01	9.57E-01
**AP001148.1**	-1.02	4.38E-01	1.00E+00	0.45	6.87E-01	9.57E-01
**AC011477.2**	-0.43	5.21E-01	1.00E+00	-0.18	6.87E-01	9.57E-01
**SLC7A3**	0.54	7.31E-01	1.00E+00	-0.84	6.87E-01	9.57E-01
**ARHGAP39**	-0.52	1.07E-01	1.00E+00	0.31	6.87E-01	9.57E-01
**UFD1**	-0.12	4.91E-01	1.00E+00	-0.09	6.87E-01	9.57E-01
**FN3K**	0.03	9.55E-01	1.00E+00	0.29	6.87E-01	9.57E-01

**KIF21B**	-0.14	8.22E-01	1.00E+00	1.27	6.87E-01	9.57E-01
**DNAH11**	0.38	4.49E-01	1.00E+00	-0.33	6.88E-01	9.57E-01
**MED22**	0.14	5.91E-01	1.00E+00	0.27	6.88E-01	9.57E-01
**EIF2AK3**	0.33	1.72E-01	1.00E+00	-0.20	6.88E-01	9.57E-01
**AC125232.2**	-0.34	4.99E-01	1.00E+00	0.36	6.88E-01	9.57E-01
**AC096745.2**	0.07	9.49E-01	1.00E+00	-0.38	6.88E-01	9.57E-01
**MYLK3**	-0.57	6.71E-01	1.00E+00	0.64	6.88E-01	9.57E-01
**DMPK**	-0.40	3.91E-01	1.00E+00	-0.33	6.88E-01	9.57E-01
**AC090912.2**	-2.93	1.87E-01	1.00E+00	-0.44	6.88E-01	9.57E-01
**WI2-1896O14.1**	0.02	9.74E-01	1.00E+00	-0.21	6.88E-01	9.57E-01
**AOC3**	0.92	2.18E-01	1.00E+00	-0.29	6.88E-01	9.57E-01
**G32346**	-2.19	2.32E-01	1.00E+00	-1.28	6.88E-01	9.57E-01
**PCDHB5**	-0.21	7.69E-01	1.00E+00	-0.27	6.88E-01	9.57E-01
**AL021578.1**	-1.86	6.40E-02	1.00E+00	-0.66	6.88E-01	9.57E-01
**ASF1B**	-0.28	3.53E-01	1.00E+00	0.16	6.88E-01	9.57E-01
**PWP1**	-0.14	4.63E-01	1.00E+00	-0.06	6.88E-01	9.57E-01
**IL21-AS1**	NA	NA	NA	0.98	6.88E-01	9.57E-01
**PTP4A2P1**	1.09	5.64E-01	1.00E+00	-0.31	6.88E-01	9.57E-01
**ASB2**	-0.07	8.99E-01	1.00E+00	0.25	6.88E-01	9.57E-01
**AC113191.1**	0.09	9.20E-01	1.00E+00	0.25	6.88E-01	9.57E-01
**KC6**	-3.12	1.58E-01	1.00E+00	-1.29	6.88E-01	9.57E-01
**KRTAP4-3**	-1.62	5.15E-01	1.00E+00	-1.29	6.89E-01	9.57E-01
**AC008966.1**	0.08	8.99E-01	1.00E+00	0.22	6.89E-01	9.57E-01
**ZADH2**	-0.04	8.31E-01	1.00E+00	0.11	6.89E-01	9.57E-01
**DHX35**	0.16	4.37E-01	1.00E+00	0.11	6.89E-01	9.57E-01
**TLE4**	0.09	7.66E-01	1.00E+00	0.12	6.89E-01	9.57E-01
**MPP4**	-1.96	1.47E-01	1.00E+00	0.42	6.89E-01	9.57E-01
**CD300LF**	0.17	9.06E-01	1.00E+00	1.26	6.89E-01	9.57E-01
**AC006504.8**	0.11	9.27E-01	1.00E+00	-0.41	6.89E-01	9.57E-01
**TSPY26P**	0.55	2.34E-01	1.00E+00	0.30	6.89E-01	9.57E-01
**SUN2**	0.16	4.33E-01	1.00E+00	-0.22	6.89E-01	9.57E-01
**TOMM5**	-0.10	8.94E-01	1.00E+00	0.30	6.89E-01	9.57E-01

**SLF2**	0.00	9.96E-01	1.00E+00	0.10	6.89E-01	9.57E-01
**LRRC26**	0.48	6.87E-01	1.00E+00	-0.41	6.89E-01	9.57E-01
**TEKT4**	-1.70	6.18E-01	1.00E+00	-0.38	6.89E-01	9.57E-01
**TEC**	0.20	7.50E-01	1.00E+00	0.20	6.89E-01	9.57E-01
**TMEM187**	-0.08	7.66E-01	1.00E+00	-0.10	6.89E-01	9.57E-01
**HERPUD1**	-0.20	3.06E-01	1.00E+00	-0.14	6.89E-01	9.57E-01
**KLHL29**	-0.39	2.40E-01	1.00E+00	0.20	6.89E-01	9.57E-01
**C15orf53**	-2.32	2.97E-01	1.00E+00	0.31	6.89E-01	9.57E-01
**GPA33**	0.41	7.14E-01	1.00E+00	-0.48	6.89E-01	9.57E-01
**SMIM17**	-0.25	9.10E-01	1.00E+00	-0.50	6.89E-01	9.57E-01
**MT1XP1**	-0.41	7.96E-01	1.00E+00	-0.24	6.89E-01	9.58E-01
**AC000068.1**	-0.56	4.34E-01	1.00E+00	-0.32	6.90E-01	9.58E-01
**MAPKAPK3**	-0.13	6.89E-01	1.00E+00	0.14	6.90E-01	9.58E-01
**FEM1A**	-0.13	7.66E-01	1.00E+00	0.19	6.90E-01	9.58E-01
**SLC25A5**	-0.38	4.44E-02	1.00E+00	0.11	6.90E-01	9.58E-01
**TAGLN2P1**	-2.32	2.41E-01	1.00E+00	0.34	6.90E-01	9.58E-01
**STAU2-AS1**	1.18	1.88E-01	1.00E+00	-0.39	6.90E-01	9.58E-01
**ELMO1**	-0.16	6.82E-01	1.00E+00	0.15	6.90E-01	9.58E-01
**FBXO31**	0.06	7.18E-01	1.00E+00	-0.22	6.90E-01	9.58E-01
**YLPM1**	-0.18	3.89E-01	1.00E+00	0.15	6.90E-01	9.58E-01
**LINC01237**	0.81	3.85E-01	1.00E+00	-0.42	6.90E-01	9.58E-01
**PTPRT**	-0.12	8.17E-01	1.00E+00	0.28	6.90E-01	9.58E-01
**GJA3**	-1.93	1.12E-02	6.54E-01	-0.48	6.90E-01	9.58E-01
**APLNR**	0.62	4.16E-01	1.00E+00	0.23	6.90E-01	9.58E-01
**SURF6**	0.04	8.73E-01	1.00E+00	0.12	6.90E-01	9.58E-01
**C10orf55**	-1.25	2.68E-01	1.00E+00	-0.32	6.90E-01	9.58E-01
**TSPAN10**	-0.13	7.15E-01	1.00E+00	0.17	6.90E-01	9.58E-01
**DNPEP**	-0.07	7.27E-01	1.00E+00	-0.16	6.90E-01	9.58E-01
**AL359762.1**	-0.54	4.95E-01	1.00E+00	0.23	6.90E-01	9.58E-01
**WBP2**	0.00	9.79E-01	1.00E+00	0.06	6.90E-01	9.58E-01
**AC092354.2**	-0.38	6.17E-01	1.00E+00	-0.24	6.90E-01	9.58E-01
**MRO**	0.79	3.62E-01	1.00E+00	0.49	6.90E-01	9.58E-01

**AC100810.1**	-0.13	6.96E-01	1.00E+00	-0.14	6.91E-01	9.58E-01
**CNIH3**	-0.16	6.95E-01	1.00E+00	0.15	6.91E-01	9.58E-01
**IGKV1-6**	1.34	4.83E-01	1.00E+00	1.27	6.91E-01	9.58E-01
**CPNE9**	-4.39	1.61E-05	4.93E-03	-0.36	6.91E-01	9.58E-01
**TIMM44**	-0.17	3.72E-01	1.00E+00	0.09	6.91E-01	9.58E-01
**MINDY4**	-2.02	1.61E-01	1.00E+00	-0.24	6.91E-01	9.58E-01
**LINC00888**	0.20	7.22E-01	1.00E+00	-0.16	6.91E-01	9.58E-01
**RPL39L**	0.08	7.89E-01	1.00E+00	0.10	6.91E-01	9.58E-01
**LINC02188**	-0.29	7.50E-01	1.00E+00	0.39	6.91E-01	9.58E-01
**RYR2**	-1.37	1.40E-02	7.46E-01	0.31	6.91E-01	9.58E-01
**ADRB2**	-1.09	3.09E-01	1.00E+00	0.23	6.91E-01	9.58E-01
**STRN4**	-0.26	3.20E-01	1.00E+00	0.20	6.91E-01	9.58E-01
**GSTM4**	-0.04	8.93E-01	1.00E+00	-0.09	6.91E-01	9.58E-01
**AL049629.1**	0.50	6.54E-01	1.00E+00	0.30	6.91E-01	9.58E-01
**DST**	-0.08	6.81E-01	1.00E+00	-0.13	6.91E-01	9.58E-01
**CCAR1**	-0.02	9.21E-01	1.00E+00	0.06	6.91E-01	9.58E-01
**AMDHD1**	-0.56	3.61E-01	1.00E+00	0.25	6.91E-01	9.58E-01
**CNN1**	-0.15	8.30E-01	1.00E+00	-0.18	6.91E-01	9.58E-01
**AKAP8L**	-0.25	3.07E-01	1.00E+00	-0.30	6.91E-01	9.58E-01
**SFTA1P**	2.32	2.78E-01	1.00E+00	-0.58	6.91E-01	9.58E-01
**DHFR2**	0.14	5.35E-01	1.00E+00	-0.15	6.91E-01	9.58E-01
**AC018738.1**	0.67	6.08E-01	1.00E+00	0.30	6.91E-01	9.58E-01
**KRTAP4-6**	-18.10	1.06E-07	4.38E-05	-1.27	6.91E-01	9.58E-01
**ACCSL**	-2.45	2.18E-01	1.00E+00	0.75	6.92E-01	9.58E-01
**PHF13**	-0.07	7.76E-01	1.00E+00	0.17	6.92E-01	9.58E-01
**TRAPPC3**	0.14	4.13E-01	1.00E+00	-0.11	6.92E-01	9.58E-01
**LINC02570**	-0.25	6.09E-01	1.00E+00	-0.30	6.92E-01	9.58E-01
**SEPHS2**	-0.17	2.68E-01	1.00E+00	-0.18	6.92E-01	9.58E-01
**GLRA4**	-2.26	1.99E-02	8.80E-01	0.40	6.92E-01	9.58E-01
**AL353147.1**	-0.50	5.51E-01	1.00E+00	-0.25	6.92E-01	9.58E-01
**FASLG**	-2.56	1.70E-01	1.00E+00	0.54	6.92E-01	9.58E-01
**PRKAG2**	0.16	5.28E-01	1.00E+00	-0.13	6.92E-01	9.58E-01

**KRTAP10-3**	-1.59	1.44E-01	1.00E+00	-1.27	6.92E-01	9.58E-01
**PSMD4**	0.04	8.03E-01	1.00E+00	-0.10	6.92E-01	9.58E-01
**AC018413.1**	-0.63	2.81E-01	1.00E+00	0.26	6.92E-01	9.58E-01
**LSM10**	-0.04	8.18E-01	1.00E+00	0.09	6.92E-01	9.58E-01
**GRM7**	-2.26	3.11E-01	1.00E+00	-0.48	6.92E-01	9.58E-01
**C1orf53**	-1.35	1.95E-02	8.74E-01	0.12	6.92E-01	9.58E-01
**YAP1**	0.12	5.46E-01	1.00E+00	0.18	6.92E-01	9.58E-01
**IDUA**	0.12	7.69E-01	1.00E+00	0.34	6.93E-01	9.58E-01
**CASD1**	0.31	3.03E-01	1.00E+00	-0.19	6.93E-01	9.58E-01
**XLOC_010249**	-3.89	1.58E-01	1.00E+00	0.61	6.93E-01	9.58E-01
**NT5C3B**	-0.32	2.44E-01	1.00E+00	-0.13	6.93E-01	9.58E-01
**AP005205.2**	NA	NA	NA	-0.44	6.93E-01	9.58E-01
**THSD8**	-3.28	7.52E-02	1.00E+00	-0.51	6.93E-01	9.58E-01
**EDAR**	-0.84	1.27E-01	1.00E+00	0.32	6.93E-01	9.58E-01
**AP000873.2**	-0.13	9.00E-01	1.00E+00	0.20	6.93E-01	9.58E-01
**AC006262.2**	0.31	5.44E-01	1.00E+00	-0.31	6.93E-01	9.58E-01
**KCNK5**	-0.17	8.05E-01	1.00E+00	-1.06	6.93E-01	9.58E-01
**HARS2**	0.13	4.92E-01	1.00E+00	-0.08	6.93E-01	9.58E-01
**SAP30L**	0.39	1.61E-01	1.00E+00	0.16	6.93E-01	9.58E-01
**PRDX1**	0.08	5.49E-01	1.00E+00	0.06	6.93E-01	9.58E-01
**AADACP1**	2.10	2.80E-02	9.75E-01	0.46	6.93E-01	9.58E-01
**PPP2R2B**	-0.32	5.27E-01	1.00E+00	0.14	6.93E-01	9.58E-01
**AC005077.4**	-1.06	4.59E-02	1.00E+00	0.57	6.93E-01	9.58E-01
**UTP11**	-0.01	9.76E-01	1.00E+00	-0.11	6.93E-01	9.58E-01
**WDR88**	-2.36	1.04E-03	1.67E-01	-0.27	6.93E-01	9.58E-01
**PTGES3P2**	3.08	8.13E-02	1.00E+00	0.54	6.93E-01	9.58E-01
**LINC00853**	1.08	2.60E-01	1.00E+00	0.28	6.93E-01	9.58E-01
**G27536**	4.40	6.77E-03	4.99E-01	-0.40	6.93E-01	9.58E-01
**GPR55**	-2.13	1.03E-01	1.00E+00	0.56	6.93E-01	9.58E-01
**USP46-AS1**	-0.54	1.08E-01	1.00E+00	-0.30	6.94E-01	9.58E-01
**LINC02004**	-1.44	4.73E-01	1.00E+00	-0.34	6.94E-01	9.58E-01
**CACTIN**	-0.32	1.60E-01	1.00E+00	0.28	6.94E-01	9.58E-01

**RDH12**	0.24	6.66E-01	1.00E+00	0.24	6.94E-01	9.58E-01
**THUMPD3-AS1**	-0.16	5.20E-01	1.00E+00	-0.13	6.94E-01	9.58E-01
**CABYR**	-0.47	3.66E-01	1.00E+00	-0.15	6.94E-01	9.58E-01
**CX3CL1**	-0.57	2.08E-01	1.00E+00	0.27	6.94E-01	9.58E-01
**PBX4**	-0.21	7.07E-01	1.00E+00	-0.26	6.94E-01	9.58E-01
**NUDT9**	0.08	6.78E-01	1.00E+00	0.10	6.94E-01	9.58E-01
**TMSB4XP4**	-0.48	3.11E-01	1.00E+00	0.29	6.94E-01	9.58E-01
**CYP4V2**	0.09	8.30E-01	1.00E+00	-0.17	6.94E-01	9.58E-01
**AP004609.3**	0.20	6.93E-01	1.00E+00	-0.13	6.94E-01	9.58E-01
**AC027117.2**	-1.87	1.59E-01	1.00E+00	0.31	6.94E-01	9.58E-01
**KCTD9**	0.26	3.00E-01	1.00E+00	-0.09	6.94E-01	9.58E-01
**CXCL14**	0.21	4.94E-01	1.00E+00	-0.18	6.94E-01	9.58E-01
**RPL23AP25**	-0.89	5.03E-01	1.00E+00	-0.29	6.94E-01	9.58E-01
**RBP2**	1.03	4.94E-01	1.00E+00	0.57	6.94E-01	9.58E-01
**AC008982.2**	1.00	5.15E-01	1.00E+00	-0.25	6.94E-01	9.58E-01
**XLOC_005592**	0.20	7.39E-01	1.00E+00	0.31	6.94E-01	9.58E-01
**AC010343.1**	-1.15	2.14E-01	1.00E+00	0.39	6.94E-01	9.58E-01
**BAALC-AS2**	-0.52	7.83E-01	1.00E+00	-0.31	6.94E-01	9.58E-01
**ITPK1**	0.07	8.52E-01	1.00E+00	0.26	6.94E-01	9.58E-01
**HERC2P3**	-0.70	4.01E-01	1.00E+00	-0.28	6.94E-01	9.58E-01
**C1QTNF4**	0.12	8.10E-01	1.00E+00	0.33	6.94E-01	9.58E-01
**ENTR1**	-0.21	2.95E-01	1.00E+00	0.20	6.94E-01	9.58E-01
**COQ4**	-0.21	4.87E-01	1.00E+00	-0.08	6.94E-01	9.58E-01
**RPS18P9**	-0.72	4.99E-03	4.14E-01	0.13	6.95E-01	9.58E-01
**ZNF473**	0.23	5.20E-01	1.00E+00	-0.13	6.95E-01	9.58E-01
**ZMIZ1-AS1**	-0.31	4.95E-01	1.00E+00	0.19	6.95E-01	9.58E-01
**SWSAP1**	-0.60	7.86E-02	1.00E+00	-0.13	6.95E-01	9.58E-01
**GPR108**	-0.19	2.26E-01	1.00E+00	-0.07	6.95E-01	9.58E-01
**LINC00240**	-0.32	6.98E-01	1.00E+00	-0.28	6.95E-01	9.58E-01
**AC090543.2**	1.08	4.48E-01	1.00E+00	-0.25	6.95E-01	9.58E-01
**POLR1C**	0.07	7.50E-01	1.00E+00	-0.14	6.95E-01	9.58E-01
**DAGLA**	0.12	8.32E-01	1.00E+00	-1.23	6.95E-01	9.58E-01

**AL356512.1**	-3.21	6.08E-02	1.00E+00	-0.32	6.95E-01	9.58E-01
**AC007216.3**	-1.48	3.24E-01	1.00E+00	0.36	6.95E-01	9.58E-01
**AC022509.3**	-0.73	2.99E-01	1.00E+00	-0.20	6.95E-01	9.58E-01
**ULK4**	0.39	3.69E-01	1.00E+00	-0.16	6.95E-01	9.58E-01
**B3GALT5**	-0.76	2.39E-01	1.00E+00	-0.33	6.95E-01	9.58E-01
**LINC01197**	-0.35	4.84E-01	1.00E+00	0.23	6.95E-01	9.58E-01
**LRP5L**	0.09	9.23E-01	1.00E+00	0.40	6.95E-01	9.58E-01
**VPREB3**	2.74	2.27E-02	9.05E-01	-0.43	6.95E-01	9.58E-01
**RPL7P50**	-1.10	7.53E-01	1.00E+00	0.47	6.95E-01	9.58E-01
**PCNPP1**	-1.60	6.41E-01	1.00E+00	-0.29	6.95E-01	9.58E-01
**G41367**	2.07	1.42E-03	2.07E-01	0.41	6.95E-01	9.58E-01
**USP20**	0.01	9.70E-01	1.00E+00	0.18	6.95E-01	9.58E-01
**SH2D1B**	0.42	7.85E-01	1.00E+00	0.46	6.95E-01	9.58E-01
**AL080243.2**	-0.32	7.92E-01	1.00E+00	0.20	6.95E-01	9.58E-01
**AC135584.1**	0.35	7.59E-01	1.00E+00	-0.41	6.95E-01	9.58E-01
**AFDN**	0.11	6.51E-01	1.00E+00	0.15	6.95E-01	9.58E-01
**SOAT1**	0.29	4.65E-01	1.00E+00	-0.22	6.95E-01	9.58E-01
**PPP1R26P1**	-1.41	2.12E-01	1.00E+00	-0.43	6.95E-01	9.58E-01
**GNAI3**	0.04	9.14E-01	1.00E+00	-0.11	6.95E-01	9.58E-01
**MTRF1**	-0.07	8.39E-01	1.00E+00	0.09	6.95E-01	9.58E-01
**MTDH**	0.08	6.41E-01	1.00E+00	-0.09	6.95E-01	9.58E-01
**LRRC36**	-0.24	8.29E-01	1.00E+00	-0.41	6.96E-01	9.58E-01
**XLOC_002192**	0.88	5.45E-01	1.00E+00	-0.29	6.96E-01	9.58E-01
**IL13RA2**	0.15	8.73E-01	1.00E+00	-0.41	6.96E-01	9.58E-01
**C9**	-0.95	6.36E-01	1.00E+00	0.44	6.96E-01	9.58E-01
**AC091153.3**	0.15	9.62E-01	1.00E+00	-0.40	6.96E-01	9.58E-01
**AC106900.2**	-0.08	9.58E-01	1.00E+00	0.36	6.96E-01	9.58E-01
**FILIP1**	0.21	7.40E-01	1.00E+00	-0.20	6.96E-01	9.58E-01
**DHX9**	-0.06	7.09E-01	1.00E+00	0.11	6.96E-01	9.58E-01
**AC084082.1**	3.06	2.73E-01	1.00E+00	0.69	6.96E-01	9.58E-01
**AC006064.5**	0.31	8.66E-01	1.00E+00	0.39	6.96E-01	9.58E-01
**AC092376.2**	0.16	7.74E-01	1.00E+00	0.27	6.96E-01	9.58E-01

**C12orf29**	0.07	8.61E-01	1.00E+00	-0.18	6.96E-01	9.58E-01
**FDPS**	-0.14	7.04E-01	1.00E+00	-0.15	6.96E-01	9.58E-01
**MEN1**	-0.26	1.55E-01	1.00E+00	-0.26	6.96E-01	9.58E-01
**GALNT11**	0.09	6.88E-01	1.00E+00	-0.09	6.96E-01	9.58E-01
**CDK13**	-0.20	2.71E-01	1.00E+00	0.15	6.96E-01	9.58E-01
**AC079807.1**	-0.09	7.82E-01	1.00E+00	0.26	6.96E-01	9.58E-01
**PSMC5**	-0.08	7.00E-01	1.00E+00	-0.08	6.96E-01	9.58E-01
**NTRK3**	-0.69	2.22E-01	1.00E+00	-0.24	6.96E-01	9.58E-01
**LINC01990**	-0.52	7.03E-01	1.00E+00	0.36	6.96E-01	9.58E-01
**TBCB**	-0.15	3.62E-01	1.00E+00	0.08	6.96E-01	9.58E-01
**AL513303.1**	-0.40	3.95E-01	1.00E+00	-0.30	6.96E-01	9.58E-01
**AC007423.1**	0.48	8.87E-01	1.00E+00	0.55	6.97E-01	9.58E-01
**RPS11P5**	-0.67	4.17E-01	1.00E+00	0.36	6.97E-01	9.58E-01
**ICE1**	-0.09	6.01E-01	1.00E+00	0.15	6.97E-01	9.58E-01
**GINS2**	-0.81	5.75E-03	4.50E-01	-0.15	6.97E-01	9.58E-01
**AC245297.2**	0.25	6.58E-01	1.00E+00	0.29	6.97E-01	9.58E-01
**AL445483.1**	-0.66	5.28E-01	1.00E+00	0.39	6.97E-01	9.58E-01
**AL513523.1**	-1.11	7.45E-01	1.00E+00	0.52	6.97E-01	9.58E-01
**SLC38A11**	0.62	2.93E-01	1.00E+00	0.43	6.97E-01	9.58E-01
**CPSF1**	-0.24	2.16E-01	1.00E+00	0.17	6.97E-01	9.58E-01
**ARL4AP2**	1.46	6.71E-01	1.00E+00	-0.25	6.97E-01	9.58E-01
**HSBP1L1**	0.03	9.38E-01	1.00E+00	-0.16	6.97E-01	9.58E-01
**HIST2H2AA3**	-2.94	8.14E-03	5.56E-01	0.21	6.97E-01	9.58E-01
**TGFBR2**	0.27	4.90E-01	1.00E+00	0.24	6.97E-01	9.58E-01
**TNRC6B**	0.01	9.68E-01	1.00E+00	0.14	6.97E-01	9.58E-01
**AQP7P1**	1.73	6.05E-01	1.00E+00	-0.95	6.97E-01	9.58E-01
**AC087500.1**	0.60	7.24E-01	1.00E+00	-0.19	6.97E-01	9.58E-01
**RPS2P32**	1.19	1.15E-01	1.00E+00	-0.29	6.97E-01	9.58E-01
**CTR9**	0.11	4.83E-01	1.00E+00	0.08	6.97E-01	9.58E-01
**PTCD1**	-0.54	3.09E-01	1.00E+00	0.15	6.97E-01	9.58E-01
**KPTN**	-0.31	3.51E-01	1.00E+00	0.16	6.97E-01	9.58E-01
**FAM86B1**	0.40	5.17E-01	1.00E+00	-0.18	6.97E-01	9.58E-01

**ANKRD30A**	2.52	2.74E-01	1.00E+00	-1.24	6.97E-01	9.58E-01
**MT4**	0.42	8.97E-01	1.00E+00	-0.44	6.97E-01	9.58E-01
**LLPH**	-0.14	5.56E-01	1.00E+00	-0.12	6.97E-01	9.58E-01
**AP1G2**	-0.10	7.80E-01	1.00E+00	0.12	6.97E-01	9.58E-01
**TPSG1**	-0.61	4.65E-01	1.00E+00	0.36	6.97E-01	9.58E-01
**CACNB2**	-0.78	1.24E-01	1.00E+00	-0.21	6.97E-01	9.58E-01
**FBXL2**	0.28	5.98E-01	1.00E+00	0.25	6.97E-01	9.58E-01
**FAM218A**	0.71	3.10E-01	1.00E+00	0.28	6.97E-01	9.58E-01
**XLOC_012849**	-0.42	7.11E-01	1.00E+00	-0.40	6.98E-01	9.58E-01
**TACC1**	0.44	2.17E-01	1.00E+00	0.15	6.98E-01	9.58E-01
**TIGD6**	-0.08	8.17E-01	1.00E+00	0.07	6.98E-01	9.58E-01
**ZC3H3**	-0.39	1.40E-01	1.00E+00	0.18	6.98E-01	9.58E-01
**SCAP**	-0.09	5.71E-01	1.00E+00	-0.19	6.98E-01	9.58E-01
**IFNGR2**	-0.03	8.86E-01	1.00E+00	-0.09	6.98E-01	9.58E-01
**VEZF1**	0.10	6.23E-01	1.00E+00	0.08	6.98E-01	9.58E-01
**AL358334.2**	0.34	9.14E-01	1.00E+00	0.70	6.98E-01	9.58E-01
**DAZL**	-3.36	3.21E-01	1.00E+00	1.24	6.98E-01	9.58E-01
**PCDHAC2**	-0.59	2.85E-01	1.00E+00	-0.54	6.98E-01	9.58E-01
**KLHL30-AS1**	-1.72	1.65E-01	1.00E+00	0.51	6.98E-01	9.58E-01
**SRRM1**	-0.13	6.03E-01	1.00E+00	-0.11	6.98E-01	9.58E-01
**AL928921.1**	1.67	5.91E-01	1.00E+00	-1.24	6.98E-01	9.58E-01
**TFAP2E**	-0.31	4.91E-01	1.00E+00	-0.32	6.98E-01	9.58E-01
**ANKRD40**	0.31	3.32E-01	1.00E+00	0.15	6.98E-01	9.58E-01
**AC015802.4**	-1.14	7.33E-01	1.00E+00	-0.40	6.98E-01	9.58E-01
**G10801**	-0.54	7.63E-01	1.00E+00	1.22	6.98E-01	9.58E-01
**FIS1**	0.06	6.84E-01	1.00E+00	0.04	6.98E-01	9.58E-01
**TOX2**	-0.12	7.77E-01	1.00E+00	0.27	6.98E-01	9.58E-01
**SCAMP1-AS1**	0.00	9.99E-01	1.00E+00	0.11	6.98E-01	9.58E-01
**FAAH2**	-0.24	4.53E-01	1.00E+00	-0.22	6.98E-01	9.58E-01
**AL355312.1**	-0.17	8.87E-01	1.00E+00	-0.45	6.98E-01	9.58E-01
**MED12**	0.00	9.86E-01	1.00E+00	0.20	6.98E-01	9.58E-01
**MXD4**	0.02	9.53E-01	1.00E+00	0.18	6.98E-01	9.58E-01

**AL031284.1**	-0.08	9.82E-01	1.00E+00	-0.27	6.98E-01	9.58E-01
**RPL23AP49**	0.29	8.69E-01	1.00E+00	-0.34	6.98E-01	9.58E-01
**AC134312.4**	-0.06	9.45E-01	1.00E+00	0.41	6.99E-01	9.58E-01
**EFTUD2**	-0.02	9.14E-01	1.00E+00	0.10	6.99E-01	9.59E-01
**FO393411.1**	-0.88	7.97E-01	1.00E+00	-0.27	6.99E-01	9.59E-01
**LIPG**	-0.46	5.90E-01	1.00E+00	0.38	6.99E-01	9.59E-01
**AC114939.1**	0.42	9.02E-01	1.00E+00	-0.59	6.99E-01	9.59E-01
**AC008608.2**	-0.23	7.94E-01	1.00E+00	-0.21	6.99E-01	9.59E-01
**RPS29**	-0.19	5.42E-01	1.00E+00	-0.15	6.99E-01	9.59E-01
**LTV1**	0.09	7.22E-01	1.00E+00	-0.11	6.99E-01	9.59E-01
**AC109454.4**	0.31	8.24E-01	1.00E+00	-0.44	6.99E-01	9.59E-01
**TSSK4**	-0.08	9.21E-01	1.00E+00	0.40	6.99E-01	9.59E-01
**PTCHD3**	-0.19	8.76E-01	1.00E+00	-0.45	6.99E-01	9.59E-01
**RGPD2**	-1.31	5.21E-01	1.00E+00	-0.36	6.99E-01	9.59E-01
**AC010422.3**	NA	NA	NA	0.47	6.99E-01	9.59E-01
**NXNL2**	-0.04	9.72E-01	1.00E+00	0.25	6.99E-01	9.59E-01
**FAM208A**	-0.06	7.72E-01	1.00E+00	0.11	6.99E-01	9.59E-01
**PAK3**	0.46	2.94E-01	1.00E+00	-0.17	6.99E-01	9.59E-01
**KLHL7-DT**	-0.55	6.71E-01	1.00E+00	-0.38	6.99E-01	9.59E-01
**G32279**	0.08	9.52E-01	1.00E+00	0.43	7.00E-01	9.59E-01
**NAPRT**	0.02	9.66E-01	1.00E+00	0.12	7.00E-01	9.59E-01
**BOD1**	0.04	8.11E-01	1.00E+00	-0.22	7.00E-01	9.59E-01
**TEX14**	-0.25	7.86E-01	1.00E+00	0.31	7.00E-01	9.59E-01
**C1QL3**	-0.97	7.15E-02	1.00E+00	-0.21	7.00E-01	9.59E-01
**TPR**	-0.27	8.70E-02	1.00E+00	0.09	7.00E-01	9.59E-01
**G31554**	-0.98	3.49E-01	1.00E+00	-0.27	7.00E-01	9.59E-01
**AL137025.1**	0.34	7.45E-01	1.00E+00	0.41	7.00E-01	9.59E-01
**XLOC_000585**	1.73	2.36E-01	1.00E+00	0.60	7.00E-01	9.59E-01
**LINC01124**	0.62	4.48E-01	1.00E+00	-0.35	7.00E-01	9.59E-01
**RFX7**	-0.15	6.19E-01	1.00E+00	0.17	7.00E-01	9.59E-01
**RPL23A**	-0.23	4.05E-01	1.00E+00	0.21	7.00E-01	9.59E-01
**RPL31**	-0.06	8.58E-01	1.00E+00	0.10	7.00E-01	9.59E-01

**TOMM20P4**	0.17	9.60E-01	1.00E+00	-0.25	7.00E-01	9.59E-01
**HACE1**	0.07	7.54E-01	1.00E+00	-0.14	7.00E-01	9.59E-01
**IK**	-0.01	9.60E-01	1.00E+00	-0.08	7.00E-01	9.59E-01
**ADSSL1**	-0.10	7.32E-01	1.00E+00	-0.27	7.00E-01	9.59E-01
**FAM167A**	-0.04	9.27E-01	1.00E+00	-0.22	7.00E-01	9.59E-01
**PPP2R5B**	0.02	9.45E-01	1.00E+00	-0.20	7.00E-01	9.59E-01
**CHRNA2**	0.69	7.29E-01	1.00E+00	-0.55	7.00E-01	9.59E-01
**ARRDC4**	0.33	1.57E-01	1.00E+00	0.14	7.00E-01	9.59E-01
**KRTAP9-4**	-18.47	4.07E-08	1.76E-05	-1.23	7.01E-01	9.59E-01
**PROCA1**	-0.35	6.18E-01	1.00E+00	0.23	7.01E-01	9.59E-01
**LINC00839**	-0.74	1.51E-01	1.00E+00	0.32	7.01E-01	9.59E-01
**UCK2**	0.27	3.99E-01	1.00E+00	0.09	7.01E-01	9.59E-01
**CREB5**	0.25	5.67E-01	1.00E+00	0.18	7.01E-01	9.59E-01
**AC116036.2**	1.20	3.59E-01	1.00E+00	-0.34	7.01E-01	9.59E-01
**RPL14P1**	-0.38	2.48E-01	1.00E+00	-0.23	7.01E-01	9.59E-01
**GCHFR**	-0.89	3.63E-01	1.00E+00	-0.30	7.01E-01	9.59E-01
**AC116407.3**	-4.65	1.99E-03	2.39E-01	-0.37	7.01E-01	9.59E-01
**BDNF-AS**	-0.06	9.05E-01	1.00E+00	-0.21	7.01E-01	9.59E-01
**SHARPIN**	-0.09	7.03E-01	1.00E+00	-0.08	7.01E-01	9.59E-01
**AC074194.2**	-3.19	4.05E-02	1.00E+00	-0.37	7.01E-01	9.59E-01
**AC004057.1**	0.59	5.88E-02	1.00E+00	0.22	7.01E-01	9.59E-01
**ZNF219**	-0.14	5.92E-01	1.00E+00	0.27	7.01E-01	9.60E-01
**ACTN1**	-0.28	4.79E-01	1.00E+00	0.17	7.01E-01	9.60E-01
**AL449212.1**	-0.12	8.86E-01	1.00E+00	-0.30	7.01E-01	9.60E-01
**AC074135.1**	0.76	7.93E-01	1.00E+00	-0.42	7.01E-01	9.60E-01
**SKP2**	0.26	2.67E-01	1.00E+00	-0.10	7.02E-01	9.60E-01
**UMPS**	0.04	8.60E-01	1.00E+00	-0.10	7.02E-01	9.60E-01
**AIDA**	0.22	3.34E-01	1.00E+00	0.18	7.02E-01	9.60E-01
**MIR2117HG**	5.14	5.95E-02	1.00E+00	0.53	7.02E-01	9.60E-01
**RPL9**	-0.01	9.73E-01	1.00E+00	-0.10	7.02E-01	9.60E-01
**AC026740.1**	-0.34	5.11E-01	1.00E+00	0.36	7.02E-01	9.60E-01
**TRPC2**	0.03	9.79E-01	1.00E+00	0.30	7.02E-01	9.60E-01

**AC135279.2**	0.56	8.71E-01	1.00E+00	0.34	7.02E-01	9.60E-01
**PSEN1**	0.35	8.75E-02	1.00E+00	-0.05	7.02E-01	9.60E-01
**PCDHGA3**	-0.35	6.13E-01	1.00E+00	-0.31	7.02E-01	9.60E-01
**LRP1B**	0.88	3.45E-01	1.00E+00	-0.38	7.02E-01	9.60E-01
**AL391988.1**	0.91	1.83E-01	1.00E+00	-0.30	7.02E-01	9.60E-01
**OPCML**	-1.61	1.00E-01	1.00E+00	-0.41	7.02E-01	9.60E-01
**SULT2B1**	0.17	7.26E-01	1.00E+00	0.21	7.02E-01	9.60E-01
**PLAGL2**	-0.27	4.36E-01	1.00E+00	0.21	7.02E-01	9.60E-01
**ECSCR**	0.04	9.31E-01	1.00E+00	0.16	7.02E-01	9.60E-01
**KRTAP3-2**	-19.52	8.03E-09	3.92E-06	-1.23	7.02E-01	9.60E-01
**EEF1A1P10**	-1.46	6.72E-01	1.00E+00	-0.29	7.02E-01	9.60E-01
**HPGDS**	0.48	2.38E-01	1.00E+00	-0.20	7.02E-01	9.60E-01
**AC127526.2**	0.01	9.97E-01	1.00E+00	0.46	7.02E-01	9.60E-01
**LINC00330**	0.02	9.87E-01	1.00E+00	0.68	7.02E-01	9.60E-01
**DNAL4**	0.15	2.91E-01	1.00E+00	0.07	7.02E-01	9.60E-01
**FZD3**	-0.17	5.18E-01	1.00E+00	-0.16	7.02E-01	9.60E-01
**ITGA6**	-0.09	7.14E-01	1.00E+00	0.12	7.02E-01	9.60E-01
**AC084262.2**	1.57	4.26E-01	1.00E+00	-0.63	7.02E-01	9.60E-01
**VPS52**	-0.27	2.14E-01	1.00E+00	0.08	7.03E-01	9.60E-01
**MTTP**	-2.22	1.57E-01	1.00E+00	-0.31	7.03E-01	9.60E-01
**GTF2H2B**	-0.10	8.89E-01	1.00E+00	0.24	7.03E-01	9.60E-01
**KRTAP24-1**	-5.21	1.13E-01	1.00E+00	-1.22	7.03E-01	9.60E-01
**UTP15**	-0.04	8.78E-01	1.00E+00	0.08	7.03E-01	9.60E-01
**CCDC43**	0.19	4.11E-01	1.00E+00	-0.10	7.03E-01	9.60E-01
**RNF182**	-0.19	7.56E-01	1.00E+00	0.33	7.03E-01	9.60E-01
**AC010538.1**	-0.11	9.02E-01	1.00E+00	-0.29	7.03E-01	9.60E-01
**AC104113.1**	0.24	8.43E-01	1.00E+00	-0.26	7.03E-01	9.60E-01
**KIF25**	0.63	5.75E-01	1.00E+00	-0.40	7.03E-01	9.60E-01
**NANOS1**	1.90	7.18E-03	5.13E-01	-0.28	7.03E-01	9.60E-01
**TBCK**	-0.10	8.26E-01	1.00E+00	-0.14	7.03E-01	9.60E-01
**NCAPG2**	-0.10	6.96E-01	1.00E+00	-0.11	7.03E-01	9.60E-01
**XLOC_000403**	-0.94	3.10E-01	1.00E+00	0.28	7.03E-01	9.60E-01

**G25631**	0.28	7.01E-01	1.00E+00	-0.38	7.03E-01	9.60E-01
**WWC3**	0.12	4.24E-01	1.00E+00	0.13	7.03E-01	9.60E-01
**XLOC_005361**	-2.92	5.28E-02	1.00E+00	-0.24	7.03E-01	9.60E-01
**MORF4L2**	0.08	6.67E-01	1.00E+00	-0.06	7.03E-01	9.60E-01
**TESK1**	-0.22	3.21E-01	1.00E+00	0.24	7.03E-01	9.60E-01
**LINC02237**	2.16	4.72E-01	1.00E+00	-0.50	7.03E-01	9.60E-01
**AC087623.2**	-1.27	8.51E-02	1.00E+00	-0.23	7.03E-01	9.60E-01
**PRMT9**	0.03	8.75E-01	1.00E+00	0.10	7.04E-01	9.60E-01
**JPH3**	-1.55	1.02E-01	1.00E+00	0.57	7.04E-01	9.60E-01
**ALDH1A3**	0.49	4.32E-01	1.00E+00	0.19	7.04E-01	9.60E-01
**SOST**	3.62	3.21E-02	1.00E+00	0.42	7.04E-01	9.60E-01
**TMTC1**	0.34	4.88E-01	1.00E+00	0.24	7.04E-01	9.60E-01
**ZNF585B**	0.30	4.61E-01	1.00E+00	0.16	7.04E-01	9.60E-01
**SYTL1**	-0.10	8.00E-01	1.00E+00	-0.20	7.04E-01	9.60E-01
**AIMP2**	-0.34	2.11E-01	1.00E+00	0.11	7.04E-01	9.60E-01
**RSBN1L**	0.09	6.61E-01	1.00E+00	0.09	7.04E-01	9.60E-01
**FCER1A**	-0.44	3.70E-01	1.00E+00	0.23	7.04E-01	9.60E-01
**HMGB3P24**	0.59	7.40E-01	1.00E+00	0.50	7.04E-01	9.60E-01
**ARF6**	-0.06	8.56E-01	1.00E+00	-0.09	7.04E-01	9.60E-01
**SBK1**	-0.83	3.19E-02	1.00E+00	-0.30	7.04E-01	9.60E-01
**AC093752.2**	2.55	4.51E-01	1.00E+00	-0.79	7.04E-01	9.60E-01
**PCDHGB6**	-0.26	4.17E-01	1.00E+00	-0.14	7.04E-01	9.60E-01
**UBE2V1**	0.10	9.13E-01	1.00E+00	0.18	7.04E-01	9.60E-01
**BX539320.1**	0.01	9.91E-01	1.00E+00	0.47	7.04E-01	9.60E-01
**OSBP2**	-0.80	2.51E-02	9.28E-01	0.23	7.04E-01	9.60E-01
**EMSY**	0.04	8.95E-01	1.00E+00	0.16	7.05E-01	9.60E-01
**LINC02242**	1.67	4.21E-01	1.00E+00	0.38	7.05E-01	9.60E-01
**NT5C1B**	-3.21	3.57E-02	1.00E+00	-0.49	7.05E-01	9.60E-01
**SSSCA1**	-0.24	3.10E-01	1.00E+00	0.10	7.05E-01	9.60E-01
**AC009107.2**	-2.09	2.62E-01	1.00E+00	0.37	7.05E-01	9.60E-01
**DPP10**	-1.57	6.65E-02	1.00E+00	-0.49	7.05E-01	9.60E-01
**LRRTM2**	-1.32	8.50E-02	1.00E+00	-0.25	7.05E-01	9.60E-01

**AL355472.2**	NA	NA	NA	-0.34	7.05E-01	9.60E-01
**RELL1**	-0.24	2.56E-01	1.00E+00	-0.16	7.05E-01	9.60E-01
**AL137779.2**	0.05	9.06E-01	1.00E+00	-0.16	7.05E-01	9.60E-01
**FZR1**	-0.23	3.57E-01	1.00E+00	0.20	7.05E-01	9.60E-01
**AP000251.1**	-1.54	1.98E-01	1.00E+00	0.54	7.05E-01	9.60E-01
**MEIS1**	0.38	2.91E-01	1.00E+00	0.14	7.05E-01	9.60E-01
**SNRPB2**	0.12	5.10E-01	1.00E+00	0.05	7.05E-01	9.60E-01
**CHST10**	0.30	4.90E-01	1.00E+00	0.28	7.05E-01	9.60E-01
**SLC15A1**	-0.13	8.22E-01	1.00E+00	0.20	7.05E-01	9.60E-01
**SRA1**	-0.27	2.79E-01	1.00E+00	0.10	7.05E-01	9.61E-01
**SELENOKP1**	0.68	8.42E-01	1.00E+00	-0.27	7.05E-01	9.61E-01
**PMM1**	0.26	2.60E-01	1.00E+00	-0.08	7.05E-01	9.61E-01
**SNHG21**	-2.50	8.39E-02	1.00E+00	-0.27	7.06E-01	9.61E-01
**AC084064.1**	-0.71	6.99E-01	1.00E+00	-0.44	7.06E-01	9.61E-01
**RNF217**	-0.26	3.42E-01	1.00E+00	0.10	7.06E-01	9.61E-01
**RPL21**	-0.11	7.25E-01	1.00E+00	-0.12	7.06E-01	9.61E-01
**AC016957.2**	-1.30	8.63E-02	1.00E+00	-0.23	7.06E-01	9.61E-01
**UBE2W**	0.24	4.59E-01	1.00E+00	-0.11	7.06E-01	9.61E-01
**CGA**	-5.23	1.18E-01	1.00E+00	-1.21	7.06E-01	9.61E-01
**FKBP1AP1**	-0.68	8.44E-01	1.00E+00	-0.45	7.06E-01	9.61E-01
**SEMA4A**	-0.33	3.07E-01	1.00E+00	0.20	7.06E-01	9.61E-01
**CHST9**	0.38	7.04E-01	1.00E+00	0.35	7.06E-01	9.61E-01
**AC005912.1**	0.10	7.90E-01	1.00E+00	0.27	7.06E-01	9.61E-01
**HYMAI**	0.71	6.66E-01	1.00E+00	-0.41	7.06E-01	9.61E-01
**IFI30**	1.63	3.59E-01	1.00E+00	0.31	7.06E-01	9.61E-01
**GLRB**	0.53	4.46E-01	1.00E+00	-0.24	7.06E-01	9.61E-01
**SMURF2**	0.40	5.85E-02	1.00E+00	0.08	7.06E-01	9.61E-01
**CA11**	-0.32	2.88E-01	1.00E+00	0.12	7.06E-01	9.61E-01
**CCDC24**	-0.01	9.85E-01	1.00E+00	0.12	7.06E-01	9.61E-01
**ARSK**	0.10	7.38E-01	1.00E+00	0.12	7.06E-01	9.61E-01
**RPL7P13**	0.90	7.96E-01	1.00E+00	0.41	7.06E-01	9.61E-01
**SAXO1**	-0.52	6.17E-01	1.00E+00	-0.30	7.06E-01	9.61E-01

**AC107294.2**	0.93	4.60E-01	1.00E+00	-0.30	7.06E-01	9.61E-01
**TTC16**	0.21	9.32E-01	1.00E+00	0.54	7.07E-01	9.61E-01
**CAPN9**	-1.03	2.61E-01	1.00E+00	0.35	7.07E-01	9.61E-01
**AC122129.1**	0.72	3.39E-01	1.00E+00	-0.27	7.07E-01	9.61E-01
**RGPD5**	-0.72	2.59E-01	1.00E+00	-0.17	7.07E-01	9.61E-01
**EXOG**	0.36	4.84E-01	1.00E+00	-0.11	7.07E-01	9.61E-01
**PDK4**	1.20	6.06E-02	1.00E+00	-0.25	7.07E-01	9.61E-01
**AL358472.3**	-0.34	7.62E-01	1.00E+00	-0.53	7.07E-01	9.61E-01
**KRTAP10-4**	-11.50	8.60E-04	1.48E-01	-1.21	7.07E-01	9.61E-01
**AC002550.2**	1.15	3.49E-01	1.00E+00	-0.42	7.07E-01	9.61E-01
**AL161909.1**	-1.05	6.77E-01	1.00E+00	0.30	7.07E-01	9.61E-01
**RGS6**	0.20	7.23E-01	1.00E+00	-0.32	7.07E-01	9.61E-01
**KCNJ2-AS1**	-0.14	8.03E-01	1.00E+00	-0.22	7.07E-01	9.61E-01
**IP6K1**	-0.09	5.34E-01	1.00E+00	0.16	7.07E-01	9.61E-01
**SHPK**	0.04	8.86E-01	1.00E+00	0.08	7.07E-01	9.61E-01
**RNF138P1**	0.85	8.06E-01	1.00E+00	0.49	7.07E-01	9.61E-01
**MBD4**	0.01	9.48E-01	1.00E+00	-0.09	7.07E-01	9.61E-01
**TTC7A**	-0.22	4.58E-01	1.00E+00	0.20	7.07E-01	9.61E-01
**AL136295.6**	1.11	1.98E-01	1.00E+00	0.26	7.07E-01	9.61E-01
**FOXN2**	0.05	8.62E-01	1.00E+00	0.07	7.07E-01	9.61E-01
**ZNF7**	0.13	5.94E-01	1.00E+00	-0.08	7.07E-01	9.61E-01
**RPL37**	-0.14	6.53E-01	1.00E+00	0.10	7.07E-01	9.61E-01
**S100A6**	-0.01	9.82E-01	1.00E+00	0.11	7.08E-01	9.61E-01
**RFK**	0.13	7.38E-01	1.00E+00	-0.10	7.08E-01	9.61E-01
**ENTPD2**	-0.69	1.33E-02	7.22E-01	0.24	7.08E-01	9.61E-01
**HS6ST1P1**	-0.27	7.29E-01	1.00E+00	-0.38	7.08E-01	9.61E-01
**AC093462.1**	-0.05	9.72E-01	1.00E+00	0.43	7.08E-01	9.61E-01
**SIGLEC12**	-2.25	2.91E-01	1.00E+00	1.19	7.08E-01	9.61E-01
**TP53RK**	0.14	5.93E-01	1.00E+00	0.10	7.08E-01	9.61E-01
**TRAF3IP1**	-0.13	5.84E-01	1.00E+00	-0.13	7.08E-01	9.61E-01
**F5**	0.70	3.39E-01	1.00E+00	-0.28	7.08E-01	9.61E-01
**LRMP**	-0.10	8.43E-01	1.00E+00	-0.14	7.08E-01	9.61E-01

**EN2**	0.16	8.50E-01	1.00E+00	-0.48	7.08E-01	9.61E-01
**WDR20**	-0.04	8.38E-01	1.00E+00	0.08	7.08E-01	9.61E-01
**AF131215.6**	0.51	4.66E-01	1.00E+00	-0.26	7.08E-01	9.61E-01
**XLOC_007925**	-2.26	7.30E-02	1.00E+00	-0.33	7.08E-01	9.61E-01
**TRAIP**	-0.20	3.60E-01	1.00E+00	-0.12	7.08E-01	9.61E-01
**GYPE**	0.75	1.83E-01	1.00E+00	0.41	7.08E-01	9.61E-01
**COMTD1**	-0.59	8.72E-02	1.00E+00	-0.12	7.08E-01	9.61E-01
**XLOC_005724**	-0.06	9.38E-01	1.00E+00	-0.24	7.08E-01	9.61E-01
**AC243562.2**	0.48	7.15E-01	1.00E+00	0.48	7.08E-01	9.61E-01
**MATN1**	0.43	8.10E-01	1.00E+00	-0.53	7.08E-01	9.61E-01
**ODC1**	0.01	9.70E-01	1.00E+00	0.22	7.08E-01	9.61E-01
**PHPT1**	-0.13	5.71E-01	1.00E+00	0.12	7.08E-01	9.61E-01
**PLOD2**	0.93	1.83E-01	1.00E+00	-0.20	7.08E-01	9.61E-01
**XLOC_005026**	-1.71	6.18E-01	1.00E+00	-0.37	7.08E-01	9.61E-01
**ULBP1**	-2.32	4.15E-02	1.00E+00	0.45	7.08E-01	9.61E-01
**MGAT5B**	-1.25	3.61E-02	1.00E+00	0.42	7.08E-01	9.61E-01
**S1PR4**	0.00	9.97E-01	1.00E+00	1.17	7.09E-01	9.61E-01
**DHDH**	-0.58	5.03E-01	1.00E+00	-0.29	7.09E-01	9.61E-01
**AL008721.2**	0.10	9.24E-01	1.00E+00	-0.37	7.09E-01	9.61E-01
**C10orf95**	0.25	8.34E-01	1.00E+00	-0.30	7.09E-01	9.61E-01
**AL139099.4**	0.27	4.86E-01	1.00E+00	-0.28	7.09E-01	9.61E-01
**LINC01123**	-0.50	2.87E-01	1.00E+00	0.23	7.09E-01	9.61E-01
**MAMLD1**	0.14	7.24E-01	1.00E+00	0.29	7.09E-01	9.61E-01
**BEX3**	0.12	6.74E-01	1.00E+00	0.11	7.09E-01	9.61E-01
**MTND6P4**	-0.31	7.11E-01	1.00E+00	-0.25	7.09E-01	9.61E-01
**BMS1**	-0.19	3.60E-01	1.00E+00	-0.07	7.09E-01	9.61E-01
**CYB5R4**	-0.46	7.84E-02	1.00E+00	0.09	7.09E-01	9.61E-01
**ZNF45**	0.37	2.13E-01	1.00E+00	0.13	7.09E-01	9.61E-01
**KLF1**	-1.07	6.76E-01	1.00E+00	0.67	7.09E-01	9.61E-01
**WBP11**	-0.10	5.79E-01	1.00E+00	-0.05	7.09E-01	9.61E-01
**ATP6V1F**	0.07	7.03E-01	1.00E+00	-0.12	7.09E-01	9.61E-01
**AC084337.1**	-0.53	4.69E-01	1.00E+00	0.29	7.09E-01	9.61E-01

**LINC00365**	2.47	1.20E-01	1.00E+00	0.46	7.09E-01	9.61E-01
**HECTD3**	-0.06	7.56E-01	1.00E+00	-0.14	7.10E-01	9.61E-01
**ATP6V0E2**	0.09	8.60E-01	1.00E+00	-0.11	7.10E-01	9.61E-01
**GAPDH**	-0.51	5.59E-02	1.00E+00	0.06	7.10E-01	9.61E-01
**LONP2**	-0.04	8.07E-01	1.00E+00	-0.06	7.10E-01	9.61E-01
**AC133435.1**	1.01	4.14E-01	1.00E+00	0.39	7.10E-01	9.61E-01
**AC019117.1**	-0.21	8.95E-01	1.00E+00	0.54	7.10E-01	9.61E-01
**C1orf122**	-0.09	8.37E-01	1.00E+00	-0.13	7.10E-01	9.61E-01
**METRN**	-0.37	3.39E-01	1.00E+00	0.28	7.10E-01	9.61E-01
**AL138781.1**	-0.62	2.40E-01	1.00E+00	-0.39	7.10E-01	9.61E-01
**CCDC117**	0.18	4.51E-01	1.00E+00	-0.08	7.10E-01	9.61E-01
**LHCGR**	1.17	2.73E-01	1.00E+00	-0.39	7.10E-01	9.61E-01
**PTMA**	-0.06	7.60E-01	1.00E+00	0.07	7.10E-01	9.61E-01
**SWT1**	-0.01	9.70E-01	1.00E+00	0.13	7.10E-01	9.61E-01
**PPP1R14A**	0.31	6.68E-01	1.00E+00	-0.15	7.10E-01	9.61E-01
**PPHLN1**	-0.23	2.38E-01	1.00E+00	-0.05	7.10E-01	9.61E-01
**AC011825.4**	-0.61	7.70E-01	1.00E+00	0.33	7.10E-01	9.61E-01
**KAT2A**	-0.09	7.64E-01	1.00E+00	0.16	7.10E-01	9.61E-01
**UPK1A-AS1**	-0.10	9.77E-01	1.00E+00	-0.45	7.10E-01	9.61E-01
**MTPAP**	-0.11	6.67E-01	1.00E+00	-0.13	7.10E-01	9.61E-01
**AC006538.1**	-1.03	2.22E-01	1.00E+00	0.48	7.10E-01	9.61E-01
**HDHD3**	-0.17	5.59E-01	1.00E+00	0.10	7.10E-01	9.61E-01
**TPRA1**	0.21	4.22E-01	1.00E+00	-0.14	7.10E-01	9.61E-01
**FAM210A**	0.10	7.37E-01	1.00E+00	-0.10	7.10E-01	9.61E-01
**HSP90AA5P**	0.36	9.17E-01	1.00E+00	-1.20	7.10E-01	9.61E-01
**ZBTB7B**	-0.26	5.01E-01	1.00E+00	0.24	7.10E-01	9.61E-01
**B3GALT4**	-0.25	3.56E-01	1.00E+00	0.07	7.10E-01	9.61E-01
**RASA4**	0.02	9.45E-01	1.00E+00	0.19	7.10E-01	9.61E-01
**AC121757.1**	-1.50	9.06E-02	1.00E+00	0.37	7.10E-01	9.61E-01
**YIF1A**	-0.11	5.86E-01	1.00E+00	0.08	7.10E-01	9.61E-01
**AC099489.1**	-0.25	6.73E-01	1.00E+00	-0.22	7.10E-01	9.61E-01
**G38775**	-0.62	5.66E-01	1.00E+00	-0.30	7.10E-01	9.61E-01

**CST2**	-3.43	4.98E-02	1.00E+00	0.50	7.10E-01	9.61E-01
**AC010186.2**	-0.25	6.61E-01	1.00E+00	0.18	7.10E-01	9.61E-01
**AC010980.2**	-0.11	9.09E-01	1.00E+00	0.43	7.11E-01	9.61E-01
**FUNDC2P1**	-0.54	8.37E-01	1.00E+00	0.25	7.11E-01	9.61E-01
**UBE2B**	0.21	3.62E-01	1.00E+00	-0.11	7.11E-01	9.61E-01
**NUP214**	-0.17	3.88E-01	1.00E+00	0.12	7.11E-01	9.61E-01
**PTPN3**	0.31	3.77E-01	1.00E+00	-0.10	7.11E-01	9.61E-01
**MSC-AS1**	1.29	8.11E-02	1.00E+00	0.20	7.11E-01	9.61E-01
**ITGA10**	-0.51	4.30E-01	1.00E+00	0.35	7.11E-01	9.61E-01
**FOXD3**	-0.62	2.62E-01	1.00E+00	-0.41	7.11E-01	9.61E-01
**TCF7L2**	0.15	7.37E-01	1.00E+00	0.13	7.11E-01	9.61E-01
**WDR90**	-0.39	1.96E-01	1.00E+00	0.25	7.11E-01	9.61E-01
**CAPRIN1**	-0.18	2.58E-01	1.00E+00	0.08	7.11E-01	9.61E-01
**AC020978.4**	-0.27	7.50E-01	1.00E+00	0.46	7.11E-01	9.61E-01
**G42642**	0.55	2.37E-01	1.00E+00	-0.40	7.11E-01	9.61E-01
**AC091429.1**	0.84	5.53E-01	1.00E+00	-0.26	7.11E-01	9.61E-01
**UBL4B**	0.55	5.84E-01	1.00E+00	1.17	7.11E-01	9.61E-01
**AC092653.1**	-0.78	5.82E-01	1.00E+00	0.57	7.11E-01	9.61E-01
**F3**	0.09	8.54E-01	1.00E+00	0.13	7.11E-01	9.61E-01
**GHET1**	0.00	9.99E-01	1.00E+00	-0.41	7.11E-01	9.61E-01
**APCDD1L-DT**	-0.14	7.24E-01	1.00E+00	-0.19	7.11E-01	9.61E-01
**XLOC_005715**	-0.36	7.32E-01	1.00E+00	-0.46	7.11E-01	9.61E-01
**CUTA**	-0.22	2.81E-01	1.00E+00	-0.09	7.11E-01	9.61E-01
**FAM83B**	-0.44	3.91E-01	1.00E+00	-0.25	7.11E-01	9.61E-01
**AL606491.1**	-3.46	5.43E-02	1.00E+00	-0.24	7.11E-01	9.61E-01
**BTBD11**	-0.45	1.63E-01	1.00E+00	-0.19	7.11E-01	9.61E-01
**ATF4P3**	0.03	9.84E-01	1.00E+00	0.20	7.11E-01	9.61E-01
**TRAPPC2L**	-0.12	5.60E-01	1.00E+00	-0.09	7.12E-01	9.61E-01
**USP15**	-0.12	7.16E-01	1.00E+00	-0.10	7.12E-01	9.61E-01
**FBXO45**	0.27	4.90E-01	1.00E+00	0.12	7.12E-01	9.61E-01
**KRTAP9-8**	-12.32	3.28E-04	7.05E-02	-1.19	7.12E-01	9.61E-01
**PDSS1**	-0.51	2.09E-01	1.00E+00	0.23	7.12E-01	9.61E-01

**ZNF229**	0.28	5.38E-01	1.00E+00	-0.22	7.12E-01	9.61E-01
**ZFP90**	-0.02	9.50E-01	1.00E+00	0.12	7.12E-01	9.61E-01
**CABCOCO1**	0.48	6.68E-01	1.00E+00	0.33	7.12E-01	9.61E-01
**FAM114A1**	0.06	8.52E-01	1.00E+00	0.09	7.12E-01	9.61E-01
**SDC2**	0.49	1.89E-01	1.00E+00	-0.15	7.12E-01	9.61E-01
**RBM39**	-0.12	6.80E-01	1.00E+00	0.06	7.12E-01	9.61E-01
**PRPSAP1**	-0.17	5.00E-01	1.00E+00	-0.07	7.12E-01	9.61E-01
**KIAA1671**	-0.18	2.81E-01	1.00E+00	0.14	7.12E-01	9.61E-01
**AC090651.1**	0.90	7.96E-01	1.00E+00	-0.66	7.12E-01	9.61E-01
**AC012186.2**	-2.87	3.96E-01	1.00E+00	-0.36	7.12E-01	9.61E-01
**MAK**	-1.45	1.43E-01	1.00E+00	0.23	7.12E-01	9.61E-01
**TUSC3**	-0.12	7.68E-01	1.00E+00	0.20	7.12E-01	9.61E-01
**EIF5AP4**	-1.17	3.90E-01	1.00E+00	-0.21	7.12E-01	9.61E-01
**ZKSCAN5**	-0.01	9.63E-01	1.00E+00	0.18	7.12E-01	9.61E-01
**RUVBL1**	-0.21	3.33E-01	1.00E+00	0.07	7.12E-01	9.61E-01
**RAB33A**	-0.80	2.63E-01	1.00E+00	0.24	7.12E-01	9.61E-01
**G11279**	-2.35	2.81E-01	1.00E+00	0.48	7.12E-01	9.61E-01
**TBC1D20**	0.05	7.67E-01	1.00E+00	-0.15	7.12E-01	9.61E-01
**RBM34**	-0.05	9.66E-01	1.00E+00	-0.16	7.12E-01	9.61E-01
**NDST3**	-2.08	1.87E-01	1.00E+00	0.38	7.12E-01	9.61E-01
**AC016735.1**	-2.18	1.82E-01	1.00E+00	-0.35	7.13E-01	9.61E-01
**GAPDHP40**	-1.19	7.32E-01	1.00E+00	0.22	7.13E-01	9.61E-01
**FTH1P8**	-0.02	9.88E-01	1.00E+00	-0.24	7.13E-01	9.61E-01
**IRX2**	-0.34	1.49E-01	1.00E+00	-0.22	7.13E-01	9.61E-01
**AP001363.2**	-0.91	4.09E-01	1.00E+00	0.24	7.13E-01	9.61E-01
**UBIAD1**	-0.01	9.84E-01	1.00E+00	-0.10	7.13E-01	9.61E-01
**PRKCD**	0.23	2.74E-01	1.00E+00	-0.19	7.13E-01	9.61E-01
**RSAD1**	0.03	8.73E-01	1.00E+00	-0.12	7.13E-01	9.61E-01
**CD8B2**	1.44	3.73E-03	3.33E-01	-0.27	7.13E-01	9.61E-01
**AL096711.2**	2.26	3.89E-01	1.00E+00	-0.61	7.13E-01	9.61E-01
**ARHGAP1**	-0.18	4.23E-01	1.00E+00	0.10	7.13E-01	9.61E-01
**AC011352.3**	0.24	5.98E-01	1.00E+00	-0.20	7.13E-01	9.61E-01

**AC011472.2**	-0.15	8.60E-01	1.00E+00	-0.19	7.13E-01	9.61E-01
**LINC01230**	2.23	4.92E-01	1.00E+00	-1.17	7.13E-01	9.61E-01
**PDRG1**	0.15	5.01E-01	1.00E+00	0.09	7.13E-01	9.61E-01
**AC009086.3**	-0.85	1.83E-01	1.00E+00	-0.22	7.13E-01	9.61E-01
**SERF1A**	1.27	5.89E-01	1.00E+00	-0.31	7.13E-01	9.61E-01
**TOLLIP-AS1**	-0.95	4.30E-02	1.00E+00	-0.17	7.13E-01	9.61E-01
**TUT4**	0.09	6.63E-01	1.00E+00	-0.10	7.13E-01	9.61E-01
**ZFPL1**	-0.21	6.16E-01	1.00E+00	0.17	7.13E-01	9.61E-01
**DPY19L2**	-0.13	8.33E-01	1.00E+00	0.28	7.13E-01	9.61E-01
**ODF2**	-0.32	1.15E-01	1.00E+00	-0.08	7.13E-01	9.61E-01
**NMRAL2P**	0.55	3.72E-01	1.00E+00	-0.20	7.13E-01	9.61E-01
**USP31**	-0.09	6.43E-01	1.00E+00	0.12	7.13E-01	9.61E-01
**LEPROT**	0.44	7.86E-02	1.00E+00	-0.08	7.13E-01	9.61E-01
**AC139887.4**	-0.62	6.72E-01	1.00E+00	0.30	7.13E-01	9.61E-01
**CHAC2**	-0.54	2.17E-01	1.00E+00	-0.13	7.13E-01	9.61E-01
**MTHFS**	2.75	7.15E-02	1.00E+00	-0.25	7.14E-01	9.61E-01
**AC004982.2**	0.62	5.02E-01	1.00E+00	-0.20	7.14E-01	9.61E-01
**AL139095.2**	0.59	6.80E-01	1.00E+00	0.21	7.14E-01	9.61E-01
**AL357054.3**	-2.70	4.27E-01	1.00E+00	-0.68	7.14E-01	9.61E-01
**C7orf25**	0.18	8.90E-01	1.00E+00	-0.19	7.14E-01	9.61E-01
**TRIM59**	0.55	1.15E-01	1.00E+00	0.21	7.14E-01	9.61E-01
**FBXW5**	-0.23	2.57E-01	1.00E+00	0.14	7.14E-01	9.61E-01
**TTC39B**	0.28	4.79E-01	1.00E+00	0.14	7.14E-01	9.61E-01
**STIL**	-0.14	6.90E-01	1.00E+00	0.20	7.14E-01	9.61E-01
**RPS12**	-0.06	8.19E-01	1.00E+00	0.10	7.14E-01	9.61E-01
**PKP1**	-0.05	9.23E-01	1.00E+00	0.22	7.14E-01	9.61E-01
**ST3GAL6-AS1**	-0.04	9.68E-01	1.00E+00	0.28	7.14E-01	9.61E-01
**ANP32B**	0.10	5.56E-01	1.00E+00	0.09	7.14E-01	9.61E-01
**CXorf56**	0.09	5.91E-01	1.00E+00	-0.07	7.14E-01	9.61E-01
**ABHD17C**	0.13	5.46E-01	1.00E+00	-0.22	7.14E-01	9.61E-01
**DNASE1L2**	-0.05	9.42E-01	1.00E+00	0.25	7.14E-01	9.61E-01
**ASTN2**	-0.18	7.32E-01	1.00E+00	0.20	7.14E-01	9.61E-01

**PDE2A**	0.31	4.83E-01	1.00E+00	0.27	7.14E-01	9.61E-01
**AC015726.1**	0.01	9.93E-01	1.00E+00	-0.29	7.14E-01	9.61E-01
**LPAR3**	0.20	5.73E-01	1.00E+00	-0.24	7.14E-01	9.61E-01
**GATA6**	-0.36	4.24E-01	1.00E+00	0.22	7.14E-01	9.61E-01
**SUFU**	-0.17	4.85E-01	1.00E+00	0.13	7.14E-01	9.61E-01
**LINC01176**	0.93	2.94E-01	1.00E+00	0.24	7.14E-01	9.61E-01
**ZXDB**	0.03	9.21E-01	1.00E+00	-0.14	7.14E-01	9.61E-01
**ANAPC11**	-0.06	7.32E-01	1.00E+00	-0.09	7.14E-01	9.61E-01
**PRPS2**	-0.06	8.18E-01	1.00E+00	0.07	7.14E-01	9.61E-01
**EPB41**	0.10	7.52E-01	1.00E+00	-0.11	7.14E-01	9.61E-01
**RPS13**	-0.05	8.59E-01	1.00E+00	0.11	7.15E-01	9.61E-01
**RSPH9**	0.11	8.70E-01	1.00E+00	0.17	7.15E-01	9.61E-01
**AC079203.1**	1.77	4.24E-01	1.00E+00	-0.41	7.15E-01	9.61E-01
**UNC45A**	-0.08	7.05E-01	1.00E+00	0.10	7.15E-01	9.61E-01
**HPCAL1**	-0.29	2.50E-01	1.00E+00	0.17	7.15E-01	9.61E-01
**DGKE**	0.14	7.48E-01	1.00E+00	0.18	7.15E-01	9.61E-01
**GADD45G**	-0.54	3.25E-01	1.00E+00	0.35	7.15E-01	9.61E-01
**GLYATL1B**	-1.09	7.51E-01	1.00E+00	0.66	7.15E-01	9.61E-01
**LARP4B**	-0.01	9.83E-01	1.00E+00	0.09	7.15E-01	9.61E-01
**LCOR**	0.04	8.46E-01	1.00E+00	0.08	7.15E-01	9.61E-01
**AGAP7P**	1.57	1.92E-01	1.00E+00	0.35	7.15E-01	9.61E-01
**NHS**	-0.34	2.49E-01	1.00E+00	0.21	7.15E-01	9.61E-01
**NDUFB11**	0.08	6.71E-01	1.00E+00	-0.09	7.15E-01	9.61E-01
**G5482**	0.52	4.82E-01	1.00E+00	-0.32	7.15E-01	9.61E-01
**PSMC6**	0.21	4.06E-01	1.00E+00	-0.08	7.15E-01	9.61E-01
**RPSAP54**	-1.46	2.43E-01	1.00E+00	-0.24	7.15E-01	9.61E-01
**ZNF286A**	0.44	1.21E-01	1.00E+00	-0.10	7.15E-01	9.61E-01
**C9orf64**	0.39	2.19E-01	1.00E+00	0.13	7.15E-01	9.61E-01
**CCDC125**	-0.51	4.54E-02	1.00E+00	0.09	7.15E-01	9.61E-01
**ACTRT3**	0.07	8.76E-01	1.00E+00	0.22	7.15E-01	9.61E-01
**BX679664.1**	-0.39	9.10E-01	1.00E+00	0.27	7.15E-01	9.61E-01
**CLPX**	-0.05	8.80E-01	1.00E+00	0.08	7.16E-01	9.61E-01

**KRT16P6**	-0.52	5.40E-01	1.00E+00	-0.25	7.16E-01	9.61E-01
**RNF130**	0.18	6.58E-01	1.00E+00	0.12	7.16E-01	9.61E-01
**TRAFD1**	-0.19	4.52E-01	1.00E+00	0.07	7.16E-01	9.61E-01
**ATP5F1AP2**	-0.56	8.73E-01	1.00E+00	0.26	7.16E-01	9.61E-01
**MIDN**	-1.09	7.81E-02	1.00E+00	0.21	7.16E-01	9.61E-01
**FAM53A**	-0.05	9.15E-01	1.00E+00	-0.23	7.16E-01	9.61E-01
**AL136295.2**	-2.27	4.38E-01	1.00E+00	-0.30	7.16E-01	9.61E-01
**ANKRD36C**	0.69	5.14E-02	1.00E+00	-0.14	7.16E-01	9.61E-01
**AC022893.1**	0.15	8.03E-01	1.00E+00	0.23	7.16E-01	9.61E-01
**IGHJ3**	NA	NA	NA	1.17	7.16E-01	9.61E-01
**ASB13**	-0.07	7.78E-01	1.00E+00	0.11	7.16E-01	9.61E-01
**PPIC**	0.16	7.06E-01	1.00E+00	0.11	7.16E-01	9.61E-01
**G38643**	-0.17	9.61E-01	1.00E+00	-0.43	7.16E-01	9.61E-01
**HDAC10**	0.28	8.40E-01	1.00E+00	-0.16	7.16E-01	9.61E-01
**SAT2**	0.21	4.00E-01	1.00E+00	-0.08	7.16E-01	9.61E-01
**KRTAP2-1**	-30.00	4.49E-18	5.54E-15	-1.17	7.16E-01	9.61E-01
**FBXO17**	0.16	5.63E-01	1.00E+00	-0.10	7.16E-01	9.61E-01
**ZNF777**	-0.53	7.02E-02	1.00E+00	-1.14	7.16E-01	9.61E-01
**AC093627.7**	-1.88	2.50E-01	1.00E+00	0.60	7.16E-01	9.61E-01
**SIPA1L3**	-0.33	2.79E-01	1.00E+00	0.22	7.16E-01	9.61E-01
**TCAF1**	0.18	3.83E-01	1.00E+00	0.12	7.16E-01	9.61E-01
**INHBE**	-0.32	7.04E-01	1.00E+00	0.42	7.16E-01	9.61E-01
**SOCS3**	-3.02	2.12E-02	8.94E-01	-0.31	7.16E-01	9.61E-01
**AL354718.1**	-0.30	6.76E-01	1.00E+00	0.23	7.16E-01	9.61E-01
**TMEM120B**	0.34	4.40E-01	1.00E+00	-0.20	7.16E-01	9.61E-01
**AC009237.8**	-0.97	6.15E-01	1.00E+00	0.39	7.17E-01	9.61E-01
**SCG3**	-0.19	9.38E-01	1.00E+00	0.53	7.17E-01	9.61E-01
**KRT78**	-0.15	8.12E-01	1.00E+00	0.25	7.17E-01	9.61E-01
**ABRACL**	-0.09	7.25E-01	1.00E+00	-0.12	7.17E-01	9.61E-01
**TP53I11**	-0.15	5.66E-01	1.00E+00	0.17	7.17E-01	9.61E-01
**AL161935.3**	1.26	1.78E-01	1.00E+00	-0.31	7.17E-01	9.61E-01
**RPRM**	-1.70	2.18E-03	2.39E-01	0.24	7.17E-01	9.61E-01

**TMX1**	0.11	7.13E-01	1.00E+00	-0.11	7.17E-01	9.61E-01
**ZNF44**	0.17	5.54E-01	1.00E+00	0.08	7.17E-01	9.61E-01
**CDC42BPA**	0.19	5.61E-01	1.00E+00	0.12	7.17E-01	9.61E-01
**WDR73**	-0.19	7.39E-01	1.00E+00	-0.12	7.17E-01	9.61E-01
**LINC02569**	1.63	2.06E-01	1.00E+00	0.33	7.17E-01	9.61E-01
**POLR1B**	0.04	8.52E-01	1.00E+00	-0.08	7.17E-01	9.61E-01
**SOWAHB**	0.10	8.39E-01	1.00E+00	-0.89	7.17E-01	9.61E-01
**DENND5B**	-0.11	7.88E-01	1.00E+00	0.20	7.17E-01	9.61E-01
**LINC00680**	-0.13	7.74E-01	1.00E+00	0.11	7.17E-01	9.61E-01
**TSEN54**	-0.25	3.84E-01	1.00E+00	0.08	7.17E-01	9.61E-01
**GACAT2**	-1.98	4.38E-01	1.00E+00	-0.38	7.17E-01	9.61E-01
**AC009171.2**	0.87	3.95E-01	1.00E+00	-0.17	7.17E-01	9.61E-01
**AL512625.1**	-0.22	7.26E-01	1.00E+00	-0.16	7.17E-01	9.61E-01
**PRKDC**	-0.29	1.33E-01	1.00E+00	0.11	7.17E-01	9.61E-01
**ZNF527**	0.07	8.21E-01	1.00E+00	-0.11	7.17E-01	9.61E-01
**MRPS18A**	-0.02	9.40E-01	1.00E+00	0.12	7.17E-01	9.61E-01
**MRPL44**	-0.13	4.32E-01	1.00E+00	0.05	7.17E-01	9.61E-01
**MPV17**	-0.10	6.42E-01	1.00E+00	-0.07	7.18E-01	9.61E-01
**AC092828.1**	0.27	8.83E-01	1.00E+00	-0.42	7.18E-01	9.61E-01
**UCA1**	-2.56	6.28E-02	1.00E+00	0.37	7.18E-01	9.61E-01
**HCG18**	-0.26	3.15E-01	1.00E+00	0.06	7.18E-01	9.61E-01
**MRPL16**	0.03	8.72E-01	1.00E+00	-0.08	7.18E-01	9.61E-01
**CLEC1B**	NA	NA	NA	0.50	7.18E-01	9.61E-01
**IER5**	-0.53	1.92E-01	1.00E+00	0.23	7.18E-01	9.61E-01
**PIWIL4**	0.75	1.19E-01	1.00E+00	-0.35	7.18E-01	9.62E-01
**TMED5**	0.15	5.97E-01	1.00E+00	-0.10	7.18E-01	9.62E-01
**AC009630.1**	2.41	3.29E-01	1.00E+00	-0.36	7.18E-01	9.62E-01
**PPP3CC**	-0.59	2.52E-02	9.28E-01	-0.16	7.18E-01	9.62E-01
**BPGM**	0.09	6.69E-01	1.00E+00	0.09	7.18E-01	9.62E-01
**ZNF519**	-0.43	4.86E-01	1.00E+00	0.16	7.18E-01	9.62E-01
**VWA8**	0.05	8.94E-01	1.00E+00	0.16	7.18E-01	9.62E-01
**MTRF1L**	-0.08	7.61E-01	1.00E+00	-0.05	7.18E-01	9.62E-01

**HOMER2**	0.02	9.66E-01	1.00E+00	-0.19	7.19E-01	9.62E-01
**RPL32P3**	0.70	2.74E-02	9.66E-01	0.10	7.19E-01	9.62E-01
**AGO3**	0.19	5.14E-01	1.00E+00	-0.09	7.19E-01	9.62E-01
**TRAPPC1**	-0.22	3.37E-01	1.00E+00	0.11	7.19E-01	9.62E-01
**GPR17**	-0.56	3.68E-01	1.00E+00	0.38	7.19E-01	9.62E-01
**HIST1H2AC**	0.12	8.14E-01	1.00E+00	0.11	7.19E-01	9.62E-01
**C14orf93**	-0.15	4.76E-01	1.00E+00	-0.11	7.19E-01	9.62E-01
**ELOVL1**	0.30	4.80E-01	1.00E+00	0.09	7.19E-01	9.62E-01
**JPT2**	0.08	6.89E-01	1.00E+00	0.10	7.19E-01	9.62E-01
**RPL7P19**	0.65	8.50E-01	1.00E+00	0.33	7.19E-01	9.62E-01
**PARM1**	0.19	5.40E-01	1.00E+00	-0.16	7.19E-01	9.62E-01
**LCE1A**	0.53	4.88E-01	1.00E+00	0.30	7.19E-01	9.62E-01
**CYHR1**	-0.26	2.79E-01	1.00E+00	-0.09	7.19E-01	9.62E-01
**PLAT**	0.05	9.33E-01	1.00E+00	0.17	7.19E-01	9.62E-01
**RPL17P34**	-0.54	7.99E-01	1.00E+00	-0.28	7.19E-01	9.62E-01
**AKT1S1**	-0.19	4.84E-01	1.00E+00	0.19	7.19E-01	9.62E-01
**AL137784.3**	0.91	3.92E-01	1.00E+00	0.41	7.19E-01	9.62E-01
**OAZ1**	-0.02	8.58E-01	1.00E+00	0.08	7.19E-01	9.62E-01
**TTF1**	0.11	5.59E-01	1.00E+00	0.09	7.19E-01	9.62E-01
**RAB11FIP4**	-0.04	9.17E-01	1.00E+00	0.19	7.19E-01	9.62E-01
**RRM2B**	0.29	2.78E-01	1.00E+00	0.06	7.19E-01	9.62E-01
**NOC4L**	-0.25	3.85E-01	1.00E+00	0.09	7.20E-01	9.62E-01
**AC005921.3**	0.54	8.76E-01	1.00E+00	-0.44	7.20E-01	9.62E-01
**EPHA7**	0.65	3.22E-01	1.00E+00	-0.33	7.20E-01	9.62E-01
**AL450344.3**	-0.67	8.47E-01	1.00E+00	-0.51	7.20E-01	9.62E-01
**ADAMTSL4-AS1**	0.32	7.40E-01	1.00E+00	0.26	7.20E-01	9.62E-01
**AL139274.1**	NA	NA	NA	0.74	7.20E-01	9.62E-01
**FGD1**	-0.14	4.25E-01	1.00E+00	0.15	7.20E-01	9.62E-01
**RUNDC1**	-0.16	3.94E-01	1.00E+00	-0.16	7.20E-01	9.62E-01
**ESPNL**	0.90	3.69E-02	1.00E+00	0.50	7.20E-01	9.62E-01
**PAQR8**	0.04	8.90E-01	1.00E+00	0.16	7.20E-01	9.62E-01
**AC026403.1**	0.48	2.78E-01	1.00E+00	0.27	7.20E-01	9.62E-01

**C8orf89**	-1.69	3.26E-01	1.00E+00	0.29	7.20E-01	9.62E-01
**NDFIP2**	0.14	7.37E-01	1.00E+00	-0.16	7.20E-01	9.62E-01
**NINJ2**	0.22	5.77E-01	1.00E+00	-0.14	7.20E-01	9.62E-01
**NBPF1**	-0.40	3.65E-01	1.00E+00	-0.13	7.20E-01	9.62E-01
**DGUOK**	0.12	5.53E-01	1.00E+00	-0.13	7.20E-01	9.62E-01
**BLVRB**	-0.04	8.08E-01	1.00E+00	0.06	7.20E-01	9.62E-01
**GDF7**	-0.36	6.14E-01	1.00E+00	0.22	7.20E-01	9.62E-01
**AC114491.1**	-0.60	8.18E-01	1.00E+00	-0.29	7.20E-01	9.62E-01
**SLC7A6OS**	-0.06	8.10E-01	1.00E+00	0.13	7.20E-01	9.62E-01
**STT3A**	0.20	4.73E-01	1.00E+00	0.09	7.20E-01	9.62E-01
**EDN3**	-0.86	3.19E-01	1.00E+00	0.92	7.20E-01	9.62E-01
**MYO6**	0.13	5.83E-01	1.00E+00	-0.06	7.20E-01	9.62E-01
**AL008727.1**	1.07	6.53E-01	1.00E+00	0.47	7.20E-01	9.62E-01
**AC068050.1**	-0.58	8.67E-01	1.00E+00	0.53	7.20E-01	9.62E-01
**LINC01948**	-1.23	2.88E-01	1.00E+00	-0.22	7.20E-01	9.62E-01
**LINC01619**	-0.04	9.34E-01	1.00E+00	-0.23	7.21E-01	9.62E-01
**AC090114.1**	0.00	1.00E+00	1.00E+00	0.32	7.21E-01	9.62E-01
**AC133041.1**	-2.35	1.64E-01	1.00E+00	0.37	7.21E-01	9.62E-01
**AC020765.2**	NA	NA	NA	-0.39	7.21E-01	9.62E-01
**TIMM13**	0.01	9.58E-01	1.00E+00	-0.09	7.21E-01	9.62E-01
**GUCA1A**	-3.43	1.65E-01	1.00E+00	0.51	7.21E-01	9.62E-01
**FRMPD4**	-1.89	1.01E-01	1.00E+00	0.61	7.21E-01	9.62E-01
**AC073389.3**	-0.75	4.71E-01	1.00E+00	0.27	7.21E-01	9.62E-01
**CDH10**	2.75	4.39E-02	1.00E+00	1.13	7.21E-01	9.62E-01
**C6orf132**	-0.31	4.25E-01	1.00E+00	0.17	7.21E-01	9.62E-01
**AL109741.2**	0.36	9.17E-01	1.00E+00	0.19	7.21E-01	9.62E-01
**ACOT13**	-0.14	6.22E-01	1.00E+00	0.12	7.21E-01	9.62E-01
**LINC01503**	0.22	6.99E-01	1.00E+00	-0.13	7.21E-01	9.62E-01
**KRTAP4-2**	-19.13	2.43E-08	1.12E-05	-1.04	7.21E-01	9.62E-01
**HES2**	-0.48	2.12E-01	1.00E+00	0.21	7.21E-01	9.62E-01
**NPR3**	-0.14	6.55E-01	1.00E+00	-0.16	7.21E-01	9.62E-01
**HIF3A**	1.32	1.76E-02	8.33E-01	-0.26	7.21E-01	9.62E-01

**MND1**	-0.25	6.08E-01	1.00E+00	-0.17	7.21E-01	9.62E-01
**SYNC**	-0.60	1.98E-01	1.00E+00	-0.26	7.22E-01	9.62E-01
**AL731557.1**	1.48	1.90E-01	1.00E+00	0.39	7.22E-01	9.62E-01
**RXFP1**	-0.33	8.23E-01	1.00E+00	1.13	7.22E-01	9.62E-01
**UBE2L3**	-0.04	7.75E-01	1.00E+00	-0.05	7.22E-01	9.62E-01
**PRPF8**	-0.13	5.49E-01	1.00E+00	-0.12	7.22E-01	9.62E-01
**WFDC1**	-0.95	1.27E-01	1.00E+00	-0.29	7.22E-01	9.62E-01
**ZNF264**	0.26	2.11E-01	1.00E+00	0.12	7.22E-01	9.62E-01
**SLC37A3**	0.46	1.46E-01	1.00E+00	0.09	7.22E-01	9.62E-01
**G38011**	-0.04	9.47E-01	1.00E+00	0.37	7.22E-01	9.62E-01
**AP000944.2**	-1.67	2.87E-01	1.00E+00	0.38	7.22E-01	9.62E-01
**XLOC_001855**	-1.68	6.27E-01	1.00E+00	1.09	7.22E-01	9.62E-01
**SLC26A9**	-0.35	6.36E-01	1.00E+00	0.29	7.22E-01	9.62E-01
**SOX18**	0.22	6.58E-01	1.00E+00	0.30	7.22E-01	9.62E-01
**HMGB3P22**	-0.17	9.22E-01	1.00E+00	-0.39	7.22E-01	9.62E-01
**CHAD**	0.22	7.27E-01	1.00E+00	-0.24	7.22E-01	9.62E-01
**SSR4P1**	-0.90	4.93E-02	1.00E+00	0.31	7.22E-01	9.62E-01
**UBL7-AS1**	-0.41	3.10E-01	1.00E+00	-0.10	7.22E-01	9.62E-01
**RPL21P89**	0.10	9.41E-01	1.00E+00	0.27	7.22E-01	9.62E-01
**AL139805.1**	1.23	5.09E-01	1.00E+00	-0.44	7.22E-01	9.62E-01
**IL12RB1**	-0.34	5.83E-01	1.00E+00	1.11	7.22E-01	9.62E-01
**ALAS2**	0.29	7.84E-01	1.00E+00	-0.43	7.22E-01	9.62E-01
**CPE**	0.44	3.73E-01	1.00E+00	0.23	7.22E-01	9.62E-01
**RN7SL181P**	-1.14	4.91E-02	1.00E+00	0.38	7.23E-01	9.62E-01
**TRBV5-4**	-0.76	6.81E-01	1.00E+00	0.46	7.23E-01	9.62E-01
**PNMT**	-0.24	7.47E-01	1.00E+00	-0.36	7.23E-01	9.62E-01
**KRTDAP**	0.29	5.64E-01	1.00E+00	-0.28	7.23E-01	9.62E-01
**TSPAN14**	0.07	7.23E-01	1.00E+00	0.11	7.23E-01	9.62E-01
**SELENOF**	0.19	3.01E-01	1.00E+00	-0.05	7.23E-01	9.62E-01
**ANAPC1**	-0.06	7.53E-01	1.00E+00	-0.09	7.23E-01	9.62E-01
**GLE1**	0.21	3.48E-01	1.00E+00	0.05	7.23E-01	9.62E-01
**HOXD3**	-0.17	8.40E-01	1.00E+00	0.44	7.23E-01	9.63E-01

**AC007384.1**	-0.85	3.49E-01	1.00E+00	-0.36	7.23E-01	9.63E-01
**DUSP4**	0.51	4.68E-01	1.00E+00	0.18	7.23E-01	9.63E-01
**AL161785.1**	-0.39	4.89E-01	1.00E+00	0.20	7.23E-01	9.63E-01
**KRTAP5-4**	5.18	2.36E-02	9.15E-01	0.68	7.23E-01	9.63E-01
**UBTD1**	-0.12	7.74E-01	1.00E+00	-0.17	7.23E-01	9.63E-01
**BCKDHA**	-0.84	7.19E-01	1.00E+00	0.34	7.23E-01	9.63E-01
**PARP3**	-0.03	9.48E-01	1.00E+00	0.14	7.23E-01	9.63E-01
**WRAP73**	0.06	8.25E-01	1.00E+00	-0.10	7.23E-01	9.63E-01
**BHLHA15**	-0.91	4.23E-01	1.00E+00	-0.44	7.23E-01	9.63E-01
**C2CD5**	0.08	7.52E-01	1.00E+00	-0.12	7.23E-01	9.63E-01
**XLOC_000266**	0.03	9.93E-01	1.00E+00	-0.87	7.23E-01	9.63E-01
**RALY-AS1**	-0.54	2.50E-01	1.00E+00	0.12	7.23E-01	9.63E-01
**POLQ**	0.02	9.69E-01	1.00E+00	0.30	7.24E-01	9.63E-01
**SDCCAG8**	0.06	7.67E-01	1.00E+00	-0.08	7.24E-01	9.63E-01
**PELI1**	0.10	7.19E-01	1.00E+00	0.07	7.24E-01	9.63E-01
**PINK1-AS**	0.22	5.90E-01	1.00E+00	-0.16	7.24E-01	9.63E-01
**AC005722.2**	-0.47	5.60E-01	1.00E+00	0.37	7.24E-01	9.63E-01
**NPAT**	-0.11	6.17E-01	1.00E+00	0.09	7.24E-01	9.63E-01
**RNF148**	-2.91	3.91E-01	1.00E+00	0.59	7.24E-01	9.63E-01
**PLA2G4E**	-0.57	3.22E-01	1.00E+00	0.28	7.24E-01	9.63E-01
**ARFGEF3**	0.28	6.41E-01	1.00E+00	-0.16	7.24E-01	9.63E-01
**XLOC_002099**	4.66	4.33E-02	1.00E+00	0.56	7.24E-01	9.63E-01
**ITPRIP**	-0.61	2.94E-01	1.00E+00	0.15	7.24E-01	9.63E-01
**PITRM1**	-0.32	2.18E-01	1.00E+00	-0.11	7.24E-01	9.63E-01
**ERVMER34-1**	-0.30	6.50E-01	1.00E+00	0.24	7.24E-01	9.63E-01
**ALOXE3**	0.11	8.63E-01	1.00E+00	0.21	7.24E-01	9.63E-01
**PTPRQ**	2.06	2.55E-01	1.00E+00	-0.38	7.24E-01	9.63E-01
**CPLANE1**	-0.29	2.38E-01	1.00E+00	0.13	7.24E-01	9.63E-01
**KLK8**	-0.10	8.69E-01	1.00E+00	0.26	7.24E-01	9.63E-01
**CYCS**	-0.15	4.87E-01	1.00E+00	-0.07	7.24E-01	9.63E-01
**AC074117.1**	-0.71	2.21E-02	8.96E-01	-0.27	7.24E-01	9.63E-01
**NRIP3**	0.13	8.15E-01	1.00E+00	-0.21	7.24E-01	9.63E-01

**CARD17**	0.39	3.82E-01	1.00E+00	-0.22	7.24E-01	9.63E-01
**FRMPD1**	0.00	9.94E-01	1.00E+00	0.22	7.24E-01	9.63E-01
**LINC02600**	-0.03	9.66E-01	1.00E+00	-1.12	7.24E-01	9.63E-01
**AL031432.2**	-0.33	7.21E-01	1.00E+00	0.47	7.25E-01	9.63E-01
**AC005042.1**	NA	NA	NA	0.60	7.25E-01	9.63E-01
**MPZ**	-0.11	8.61E-01	1.00E+00	0.98	7.25E-01	9.63E-01
**HSPA13**	0.03	9.20E-01	1.00E+00	0.11	7.25E-01	9.63E-01
**AKR7A3**	-1.46	8.36E-02	1.00E+00	-0.25	7.25E-01	9.63E-01
**EXOSC1**	-0.10	6.95E-01	1.00E+00	0.08	7.25E-01	9.63E-01
**KHDRBS1**	-0.01	9.61E-01	1.00E+00	-0.12	7.25E-01	9.63E-01
**TUFM**	-0.10	6.39E-01	1.00E+00	0.04	7.25E-01	9.63E-01
**C19orf33**	0.91	5.00E-01	1.00E+00	-0.26	7.25E-01	9.63E-01
**FAM234A**	-0.01	9.54E-01	1.00E+00	0.08	7.25E-01	9.63E-01
**KANSL1L**	0.01	9.60E-01	1.00E+00	-0.12	7.25E-01	9.63E-01
**NFATC2IP**	-0.04	8.61E-01	1.00E+00	-0.16	7.25E-01	9.63E-01
**AC078850.1**	0.77	6.63E-01	1.00E+00	0.49	7.25E-01	9.63E-01
**AL133260.1**	-1.49	5.00E-01	1.00E+00	-0.21	7.25E-01	9.63E-01
**AL357568.1**	0.23	9.47E-01	1.00E+00	0.82	7.25E-01	9.63E-01
**UBL4A**	0.12	5.03E-01	1.00E+00	-0.07	7.25E-01	9.63E-01
**OR10A3**	-1.62	3.04E-01	1.00E+00	0.59	7.25E-01	9.63E-01
**CEP295NL**	0.11	9.19E-01	1.00E+00	-0.57	7.25E-01	9.63E-01
**USP39**	-0.02	8.89E-01	1.00E+00	0.06	7.25E-01	9.63E-01
**AC115223.1**	-0.03	9.26E-01	1.00E+00	-0.12	7.25E-01	9.63E-01
**RNA5SP283**	-0.95	4.09E-01	1.00E+00	0.32	7.25E-01	9.63E-01
**AC007256.1**	-2.51	3.44E-01	1.00E+00	0.35	7.25E-01	9.63E-01
**LINC01876**	-0.71	5.49E-01	1.00E+00	0.27	7.25E-01	9.63E-01
**FGF9**	1.46	1.08E-01	1.00E+00	0.44	7.26E-01	9.63E-01
**NCOA1**	-0.22	1.78E-01	1.00E+00	-0.11	7.26E-01	9.63E-01
**G41823**	0.10	9.23E-01	1.00E+00	-0.22	7.26E-01	9.63E-01
**GTF3C3**	0.07	7.72E-01	1.00E+00	-0.08	7.26E-01	9.63E-01
**ZNF841**	0.13	7.49E-01	1.00E+00	0.14	7.26E-01	9.63E-01
**XLOC_005110**	-1.02	6.91E-01	1.00E+00	-0.36	7.26E-01	9.63E-01

**FAM53C**	0.11	4.55E-01	1.00E+00	-0.14	7.26E-01	9.63E-01
**KRTAP4-12**	-19.06	2.73E-08	1.21E-05	-1.12	7.26E-01	9.63E-01
**OVOL1-AS1**	0.22	7.36E-01	1.00E+00	-0.25	7.26E-01	9.63E-01
**KRT5**	-0.13	7.02E-01	1.00E+00	0.21	7.26E-01	9.63E-01
**SH2D3A**	0.07	8.32E-01	1.00E+00	-0.22	7.26E-01	9.63E-01
**STAMBP**	0.20	2.81E-01	1.00E+00	-0.06	7.26E-01	9.63E-01
**RPF1**	0.01	9.70E-01	1.00E+00	-0.08	7.26E-01	9.63E-01
**TMEM200B**	0.50	1.10E-01	1.00E+00	0.25	7.26E-01	9.63E-01
**EYA4**	2.21	6.24E-02	1.00E+00	0.38	7.26E-01	9.63E-01
**G12890**	-0.23	7.15E-01	1.00E+00	-0.33	7.27E-01	9.63E-01
**AC118754.1**	-0.92	4.20E-01	1.00E+00	0.28	7.27E-01	9.63E-01
**SLC7A10**	4.66	1.31E-03	1.95E-01	0.43	7.27E-01	9.63E-01
**AL450992.2**	0.53	9.29E-02	1.00E+00	-0.21	7.27E-01	9.63E-01
**SNX16**	0.34	3.35E-01	1.00E+00	-0.10	7.27E-01	9.63E-01
**CACNA2D2**	-0.23	5.92E-01	1.00E+00	0.21	7.27E-01	9.63E-01
**KIAA0895L**	-0.37	2.51E-01	1.00E+00	0.15	7.27E-01	9.63E-01
**UBE2D2**	0.01	9.65E-01	1.00E+00	-0.07	7.27E-01	9.63E-01
**G22291**	0.50	5.92E-01	1.00E+00	-0.35	7.27E-01	9.63E-01
**AC010332.1**	1.34	3.28E-01	1.00E+00	-0.28	7.27E-01	9.63E-01
**CHD9**	0.08	6.74E-01	1.00E+00	-0.07	7.27E-01	9.63E-01
**CARF**	0.17	5.94E-01	1.00E+00	0.14	7.27E-01	9.63E-01
**CYB5D2**	-0.03	9.08E-01	1.00E+00	-0.08	7.27E-01	9.63E-01
**AL136531.3**	-0.76	5.13E-01	1.00E+00	-0.32	7.27E-01	9.63E-01
**RPS7P3**	-0.09	9.66E-01	1.00E+00	0.28	7.27E-01	9.63E-01
**LRP3**	0.09	8.07E-01	1.00E+00	0.23	7.27E-01	9.63E-01
**USP49**	0.08	8.19E-01	1.00E+00	-0.13	7.27E-01	9.63E-01
**C12orf60**	0.91	1.26E-01	1.00E+00	0.18	7.27E-01	9.63E-01
**ITGB1-DT**	-2.12	1.97E-01	1.00E+00	0.51	7.27E-01	9.63E-01
**XLOC_001338**	-0.13	9.07E-01	1.00E+00	0.34	7.27E-01	9.63E-01
**LINC01698**	0.53	4.04E-01	1.00E+00	0.33	7.27E-01	9.63E-01
**CNPPD1**	-0.07	6.92E-01	1.00E+00	0.05	7.27E-01	9.63E-01
**CLHC1**	-1.23	5.50E-02	1.00E+00	-0.16	7.27E-01	9.63E-01

**SHROOM3**	0.36	4.60E-01	1.00E+00	0.16	7.27E-01	9.63E-01
**CERS4**	-0.38	2.39E-01	1.00E+00	0.11	7.28E-01	9.63E-01
**PDE3B**	3.69	5.14E-03	4.18E-01	-0.33	7.28E-01	9.63E-01
**EIF4G3**	-0.55	3.40E-03	3.11E-01	0.14	7.28E-01	9.63E-01
**XLOC_005409**	-0.47	8.06E-01	1.00E+00	-0.39	7.28E-01	9.63E-01
**ANKRD36B**	0.37	4.84E-01	1.00E+00	-0.12	7.28E-01	9.63E-01
**TOMM40L**	0.14	6.48E-01	1.00E+00	0.11	7.28E-01	9.63E-01
**PAX9**	-1.01	1.70E-01	1.00E+00	-0.18	7.28E-01	9.63E-01
**GIMAP7**	0.33	5.47E-01	1.00E+00	0.15	7.28E-01	9.63E-01
**SFTPC**	-0.96	8.23E-02	1.00E+00	0.32	7.28E-01	9.63E-01
**RAET1E-AS1**	-0.62	3.42E-01	1.00E+00	0.25	7.28E-01	9.63E-01
**HDAC9**	0.13	7.68E-01	1.00E+00	0.13	7.28E-01	9.63E-01
**LINC00681**	1.29	1.63E-01	1.00E+00	-0.39	7.28E-01	9.63E-01
**C4A**	-0.09	9.39E-01	1.00E+00	1.09	7.28E-01	9.63E-01
**FAM126B**	-0.06	8.58E-01	1.00E+00	0.11	7.28E-01	9.63E-01
**KIAA2026**	-0.03	8.37E-01	1.00E+00	-0.10	7.28E-01	9.63E-01
**AC016205.1**	-0.53	3.27E-01	1.00E+00	-0.26	7.28E-01	9.63E-01
**PIK3IP1**	-0.17	4.54E-01	1.00E+00	-0.09	7.28E-01	9.63E-01
**CCDC50**	0.06	8.40E-01	1.00E+00	0.11	7.28E-01	9.63E-01
**ZSCAN30**	0.27	4.53E-01	1.00E+00	0.17	7.28E-01	9.63E-01
**ZNF805**	-0.02	9.34E-01	1.00E+00	0.22	7.28E-01	9.63E-01
**ANGPT1**	0.85	2.44E-01	1.00E+00	-0.22	7.28E-01	9.63E-01
**IP6K2**	-0.06	8.78E-01	1.00E+00	0.11	7.28E-01	9.63E-01
**SHLD2**	0.23	4.16E-01	1.00E+00	-0.10	7.28E-01	9.63E-01
**G39784**	0.53	6.72E-01	1.00E+00	0.37	7.29E-01	9.63E-01
**NACA3P**	-0.61	7.72E-01	1.00E+00	-0.29	7.29E-01	9.63E-01
**AC010463.3**	0.88	7.47E-01	1.00E+00	0.36	7.29E-01	9.63E-01
**YBX1P10**	-0.40	6.00E-01	1.00E+00	-0.11	7.29E-01	9.63E-01
**ESF1**	0.11	5.57E-01	1.00E+00	-0.09	7.29E-01	9.63E-01
**AL117336.3**	-3.23	9.85E-05	2.44E-02	0.19	7.29E-01	9.63E-01
**AL445437.1**	0.80	5.28E-01	1.00E+00	0.40	7.29E-01	9.63E-01
**BANK1**	0.15	7.76E-01	1.00E+00	0.19	7.29E-01	9.63E-01

**SPTAN1**	-0.28	2.05E-01	1.00E+00	0.12	7.29E-01	9.63E-01
**TMF1**	-0.12	6.89E-01	1.00E+00	-0.07	7.29E-01	9.63E-01
**AC115618.1**	-0.66	5.29E-01	1.00E+00	0.22	7.29E-01	9.63E-01
**RPL7P47**	0.88	7.98E-01	1.00E+00	0.30	7.29E-01	9.63E-01
**RND2**	-0.11	6.99E-01	1.00E+00	-0.16	7.29E-01	9.63E-01
**ALKBH6**	-0.41	8.02E-01	1.00E+00	-0.17	7.29E-01	9.63E-01
**AP000842.2**	0.06	9.38E-01	1.00E+00	0.32	7.29E-01	9.63E-01
**DCAF17**	0.02	9.17E-01	1.00E+00	-0.08	7.29E-01	9.63E-01
**AL357552.2**	-0.46	8.15E-01	1.00E+00	0.40	7.29E-01	9.63E-01
**AL161804.1**	-1.43	6.77E-01	1.00E+00	-0.85	7.29E-01	9.63E-01
**ST13P18**	0.47	8.15E-01	1.00E+00	-0.17	7.29E-01	9.63E-01
**PNLDC1**	2.62	8.00E-02	1.00E+00	-0.39	7.29E-01	9.63E-01
**CHKB**	1.55	3.94E-01	1.00E+00	0.26	7.29E-01	9.63E-01
**SP6**	0.02	9.70E-01	1.00E+00	0.26	7.29E-01	9.63E-01
**ADNP2**	-0.22	2.90E-01	1.00E+00	0.12	7.29E-01	9.63E-01
**AC092687.3**	-0.28	7.88E-01	1.00E+00	0.18	7.29E-01	9.63E-01
**FGF13-AS1**	0.28	8.54E-01	1.00E+00	-0.41	7.29E-01	9.63E-01
**DDR1**	-0.24	1.71E-01	1.00E+00	-0.16	7.29E-01	9.63E-01
**DHX15**	0.19	4.67E-01	1.00E+00	0.06	7.29E-01	9.63E-01
**C1orf43**	0.19	2.55E-01	1.00E+00	0.05	7.29E-01	9.63E-01
**SIDT1**	0.45	2.97E-01	1.00E+00	0.28	7.29E-01	9.64E-01
**AP003108.2**	2.33	2.69E-01	1.00E+00	-0.48	7.30E-01	9.64E-01
**CLIC3**	0.01	9.85E-01	1.00E+00	0.19	7.30E-01	9.64E-01
**SRSF12**	0.31	5.57E-01	1.00E+00	-0.25	7.30E-01	9.64E-01
**DAAM2**	0.29	4.82E-01	1.00E+00	0.22	7.30E-01	9.64E-01
**NIPSNAP1**	-0.05	8.06E-01	1.00E+00	0.06	7.30E-01	9.64E-01
**AC003986.2**	-0.02	9.88E-01	1.00E+00	0.27	7.30E-01	9.64E-01
**TSACC**	0.38	7.58E-01	1.00E+00	0.32	7.30E-01	9.64E-01
**RAB39A**	-2.59	1.48E-01	1.00E+00	1.09	7.30E-01	9.64E-01
**EPS8L1**	-0.04	9.39E-01	1.00E+00	0.22	7.30E-01	9.64E-01
**AL353626.2**	-2.36	4.90E-01	1.00E+00	0.30	7.30E-01	9.64E-01
**STX12**	0.30	8.33E-02	1.00E+00	-0.06	7.30E-01	9.64E-01

**SORBS3**	-0.32	2.05E-01	1.00E+00	0.14	7.30E-01	9.64E-01
**KCNE2**	0.43	9.01E-01	1.00E+00	-0.34	7.30E-01	9.64E-01
**FAM110D**	-0.38	2.70E-01	1.00E+00	-0.30	7.30E-01	9.64E-01
**GOLGA5**	-0.13	5.09E-01	1.00E+00	0.05	7.30E-01	9.64E-01
**AC004854.2**	0.41	5.01E-01	1.00E+00	0.34	7.30E-01	9.64E-01
**BRSK2**	-0.73	6.18E-01	1.00E+00	-0.39	7.30E-01	9.64E-01
**AL391069.2**	-0.12	9.73E-01	1.00E+00	0.22	7.30E-01	9.64E-01
**ACACA**	0.43	1.53E-01	1.00E+00	-0.12	7.30E-01	9.64E-01
**C17orf80**	-0.06	8.53E-01	1.00E+00	-0.12	7.31E-01	9.64E-01
**G12586**	-0.61	7.55E-01	1.00E+00	-0.37	7.31E-01	9.64E-01
**BEST4**	-2.32	7.57E-02	1.00E+00	0.38	7.31E-01	9.64E-01
**DHRS2**	2.51	1.50E-02	7.62E-01	-0.41	7.31E-01	9.64E-01
**AC105219.3**	-1.10	2.11E-01	1.00E+00	-0.42	7.31E-01	9.64E-01
**PNO1**	-0.13	5.82E-01	1.00E+00	0.09	7.31E-01	9.64E-01
**HOMER3-AS1**	-0.53	8.11E-01	1.00E+00	-0.39	7.31E-01	9.64E-01
**WDR78**	0.05	9.21E-01	1.00E+00	-0.13	7.31E-01	9.64E-01
**AC120036.3**	0.57	6.09E-01	1.00E+00	0.36	7.31E-01	9.64E-01
**HORMAD1**	-2.67	3.83E-01	1.00E+00	-0.40	7.31E-01	9.64E-01
**MZT2A**	-0.08	7.28E-01	1.00E+00	-0.17	7.31E-01	9.64E-01
**AC008147.2**	1.85	4.14E-01	1.00E+00	0.36	7.31E-01	9.64E-01
**SEMA3F**	-0.16	6.53E-01	1.00E+00	0.23	7.31E-01	9.64E-01
**TXNP5**	-0.58	8.67E-01	1.00E+00	0.38	7.31E-01	9.64E-01
**NLN**	-0.14	5.21E-01	1.00E+00	0.08	7.31E-01	9.64E-01
**NNT-AS1**	0.17	6.14E-01	1.00E+00	-0.10	7.31E-01	9.64E-01
**DARS2**	-0.03	9.16E-01	1.00E+00	0.12	7.31E-01	9.64E-01
**GABRA2**	-0.09	9.15E-01	1.00E+00	0.31	7.31E-01	9.64E-01
**LIPM**	0.15	7.94E-01	1.00E+00	0.22	7.31E-01	9.64E-01
**EPDR1**	0.31	6.45E-01	1.00E+00	-0.24	7.32E-01	9.64E-01
**UBE2I**	0.15	4.49E-01	1.00E+00	0.12	7.32E-01	9.64E-01
**IGFBP7**	0.08	8.61E-01	1.00E+00	-0.20	7.32E-01	9.64E-01
**MED14**	-0.03	9.23E-01	1.00E+00	0.10	7.32E-01	9.64E-01
**SNAP47**	-0.09	6.32E-01	1.00E+00	0.06	7.32E-01	9.64E-01

**EEF1DP7**	-3.42	1.60E-01	1.00E+00	-0.42	7.32E-01	9.64E-01
**KCNH4**	-0.53	5.75E-01	1.00E+00	0.49	7.32E-01	9.64E-01
**LMTK2**	0.02	9.49E-01	1.00E+00	0.20	7.32E-01	9.64E-01
**CES3**	0.33	6.47E-01	1.00E+00	-0.34	7.32E-01	9.64E-01
**PDXDC2P**	-2.83	1.85E-01	1.00E+00	-0.38	7.32E-01	9.64E-01
**MRPL3**	-0.19	3.17E-01	1.00E+00	-0.06	7.32E-01	9.64E-01
**NOMO2**	0.02	9.66E-01	1.00E+00	0.24	7.32E-01	9.64E-01
**FAM118B**	0.10	7.40E-01	1.00E+00	0.11	7.32E-01	9.64E-01
**PCDHB12**	0.12	8.24E-01	1.00E+00	-0.26	7.32E-01	9.64E-01
**ZNF425**	0.07	8.36E-01	1.00E+00	-0.15	7.33E-01	9.64E-01
**OXER1**	-0.10	8.52E-01	1.00E+00	0.23	7.33E-01	9.64E-01
**SPIN1**	0.12	4.91E-01	1.00E+00	-0.07	7.33E-01	9.64E-01
**EXOSC9**	-0.11	6.18E-01	1.00E+00	-0.08	7.33E-01	9.64E-01
**METTL7A**	0.83	1.23E-02	6.93E-01	0.12	7.33E-01	9.64E-01
**NUFIP1**	-0.29	1.66E-01	1.00E+00	0.07	7.33E-01	9.64E-01
**CHTF8**	-0.18	2.06E-01	1.00E+00	0.07	7.33E-01	9.64E-01
**AP000845.1**	4.70	1.21E-01	1.00E+00	0.45	7.33E-01	9.64E-01
**ZNF727**	1.79	2.03E-02	8.88E-01	-0.17	7.33E-01	9.64E-01
**CLDN14**	0.02	9.89E-01	1.00E+00	0.33	7.33E-01	9.64E-01
**DBF4**	-0.27	4.19E-01	1.00E+00	0.07	7.33E-01	9.64E-01
**ZNF436**	0.19	3.83E-01	1.00E+00	0.11	7.33E-01	9.64E-01
**AC026471.2**	-2.29	2.77E-01	1.00E+00	-0.29	7.33E-01	9.64E-01
**CPED1**	0.65	2.88E-01	1.00E+00	-0.16	7.33E-01	9.64E-01
**FAM25G**	-0.56	8.73E-01	1.00E+00	-0.28	7.33E-01	9.64E-01
**UBXN10**	-0.27	4.82E-01	1.00E+00	0.17	7.33E-01	9.64E-01
**TLK2**	-0.17	3.31E-01	1.00E+00	0.13	7.33E-01	9.64E-01
**PHLPP1**	-0.30	5.81E-02	1.00E+00	0.17	7.33E-01	9.64E-01
**AC027277.2**	-0.58	5.52E-01	1.00E+00	-0.34	7.33E-01	9.64E-01
**EP300**	-0.43	6.56E-02	1.00E+00	0.14	7.33E-01	9.64E-01
**RGS14**	-0.52	2.29E-01	1.00E+00	0.13	7.33E-01	9.64E-01
**TPP2**	0.19	5.17E-01	1.00E+00	0.08	7.33E-01	9.64E-01
**MED23**	0.42	2.15E-01	1.00E+00	-0.11	7.33E-01	9.64E-01

**XLOC_003544**	2.14	4.67E-01	1.00E+00	-0.32	7.33E-01	9.64E-01
**AMN1**	0.12	7.61E-01	1.00E+00	-0.14	7.33E-01	9.64E-01
**ZNF213**	-0.33	1.87E-01	1.00E+00	0.19	7.33E-01	9.64E-01
**HIVEP2**	-0.34	9.50E-02	1.00E+00	0.18	7.33E-01	9.64E-01
**STK25**	-0.07	7.52E-01	1.00E+00	-0.11	7.33E-01	9.64E-01
**AL049840.4**	0.27	5.07E-01	1.00E+00	0.13	7.34E-01	9.64E-01
**IGHMBP2**	-0.23	3.13E-01	1.00E+00	0.08	7.34E-01	9.64E-01
**TRIM10**	-2.19	1.90E-01	1.00E+00	1.08	7.34E-01	9.64E-01
**KLKP1**	-0.27	8.03E-01	1.00E+00	0.26	7.34E-01	9.64E-01
**KIAA1551**	-0.35	1.37E-01	1.00E+00	0.10	7.34E-01	9.64E-01
**XDH**	-0.42	4.47E-01	1.00E+00	-0.28	7.34E-01	9.64E-01
**PRMT7**	0.16	4.81E-01	1.00E+00	0.05	7.34E-01	9.64E-01
**PTGES3P1**	-0.07	8.56E-01	1.00E+00	0.18	7.34E-01	9.64E-01
**CDKN2D**	0.03	9.16E-01	1.00E+00	-0.10	7.34E-01	9.64E-01
**KCNC4**	-0.19	6.34E-01	1.00E+00	0.15	7.34E-01	9.64E-01
**ELOVL4**	0.16	7.81E-01	1.00E+00	0.19	7.34E-01	9.64E-01
**TPTEP1**	-0.76	4.87E-02	1.00E+00	-0.14	7.34E-01	9.64E-01
**AC002470.1**	-0.35	6.69E-01	1.00E+00	-0.16	7.34E-01	9.64E-01
**SLC13A4**	-0.37	6.68E-01	1.00E+00	-0.22	7.34E-01	9.64E-01
**POLR3D**	-0.01	9.69E-01	1.00E+00	0.14	7.34E-01	9.64E-01
**C18orf21**	0.03	9.16E-01	1.00E+00	0.06	7.34E-01	9.64E-01
**RPL24P4**	-0.53	5.48E-01	1.00E+00	0.30	7.34E-01	9.64E-01
**PAX8**	-0.15	7.94E-01	1.00E+00	0.19	7.34E-01	9.64E-01
**KRT7**	1.01	2.24E-01	1.00E+00	-0.24	7.34E-01	9.64E-01
**AC064799.1**	0.05	9.29E-01	1.00E+00	-0.22	7.34E-01	9.64E-01
**AL136084.2**	-10.68	1.81E-03	2.38E-01	-0.88	7.34E-01	9.64E-01
**CMTM2**	-2.20	2.44E-01	1.00E+00	0.39	7.34E-01	9.64E-01
**AL928654.1**	0.85	3.33E-01	1.00E+00	-0.26	7.34E-01	9.64E-01
**XLOC_007462**	-0.09	7.73E-01	1.00E+00	-0.17	7.34E-01	9.64E-01
**MAPKAP1**	0.07	7.10E-01	1.00E+00	-0.17	7.34E-01	9.64E-01
**EIF4A3**	-0.25	3.72E-01	1.00E+00	0.08	7.35E-01	9.64E-01
**KLF4**	-0.28	5.82E-01	1.00E+00	0.13	7.35E-01	9.64E-01

**PSMD3**	-0.14	5.93E-01	1.00E+00	0.12	7.35E-01	9.64E-01
**GSC**	0.61	2.65E-01	1.00E+00	0.33	7.35E-01	9.64E-01
**LINC01004**	-0.26	8.03E-01	1.00E+00	0.18	7.35E-01	9.64E-01
**MIR621**	-0.22	6.17E-01	1.00E+00	0.45	7.35E-01	9.64E-01
**SEC61B**	-0.02	8.82E-01	1.00E+00	-0.08	7.35E-01	9.64E-01
**AC104109.2**	-0.93	1.93E-01	1.00E+00	0.17	7.35E-01	9.64E-01
**ACAA1**	-0.21	3.88E-01	1.00E+00	0.09	7.35E-01	9.64E-01
**AC245100.4**	0.49	7.86E-01	1.00E+00	0.29	7.35E-01	9.64E-01
**MAPRE2**	0.04	8.50E-01	1.00E+00	-0.10	7.35E-01	9.64E-01
**TEX11**	-2.08	2.53E-01	1.00E+00	0.42	7.35E-01	9.64E-01
**RPL23AP57**	NA	NA	NA	0.37	7.35E-01	9.64E-01
**PRSS8**	-0.15	7.52E-01	1.00E+00	0.19	7.35E-01	9.64E-01
**PTGES3L**	-4.61	1.75E-02	8.33E-01	0.32	7.35E-01	9.64E-01
**USP25**	-0.02	9.03E-01	1.00E+00	-0.17	7.35E-01	9.64E-01
**KRBA2**	-0.38	4.98E-01	1.00E+00	-0.17	7.35E-01	9.64E-01
**CUL9**	-0.11	5.82E-01	1.00E+00	0.16	7.35E-01	9.64E-01
**TCP11L2**	0.10	6.74E-01	1.00E+00	-0.13	7.35E-01	9.64E-01
**SLC26A3**	0.92	6.94E-01	1.00E+00	-0.42	7.35E-01	9.64E-01
**WDR6**	-0.38	1.72E-01	1.00E+00	0.14	7.35E-01	9.64E-01
**EEF1A1P3**	0.79	5.40E-01	1.00E+00	-0.28	7.35E-01	9.64E-01
**TSNAXIP1**	-1.14	1.58E-01	1.00E+00	-0.32	7.35E-01	9.64E-01
**KRTAP4-1**	-2.42	4.64E-01	1.00E+00	-1.08	7.35E-01	9.64E-01
**ZNF451**	0.08	7.26E-01	1.00E+00	0.10	7.35E-01	9.64E-01
**G25291**	0.58	6.56E-01	1.00E+00	0.42	7.35E-01	9.64E-01
**AC023424.2**	-0.11	9.20E-01	1.00E+00	-0.38	7.35E-01	9.64E-01
**AC010970.1**	-0.38	3.84E-01	1.00E+00	-0.27	7.35E-01	9.64E-01
**EFHB**	-1.00	2.88E-01	1.00E+00	-0.25	7.35E-01	9.64E-01
**AC093904.2**	0.42	6.62E-01	1.00E+00	0.34	7.35E-01	9.64E-01
**AC087473.1**	-1.96	5.91E-02	1.00E+00	0.38	7.36E-01	9.64E-01
**ZSCAN32**	0.13	6.30E-01	1.00E+00	-0.07	7.36E-01	9.64E-01
**C8orf76**	0.31	7.39E-01	1.00E+00	-0.16	7.36E-01	9.64E-01
**DAPP1**	0.41	4.20E-01	1.00E+00	0.14	7.36E-01	9.64E-01

**RP1L1**	-0.24	6.37E-01	1.00E+00	-0.70	7.36E-01	9.64E-01
**CLASP2**	0.01	9.66E-01	1.00E+00	-0.09	7.36E-01	9.64E-01
**OCLN**	0.40	1.94E-01	1.00E+00	-0.16	7.36E-01	9.64E-01
**AC007376.2**	2.85	2.51E-01	1.00E+00	0.39	7.36E-01	9.64E-01
**AC019330.1**	-0.22	9.33E-01	1.00E+00	-0.58	7.36E-01	9.64E-01
**AL137796.1**	-1.55	5.69E-01	1.00E+00	-0.32	7.36E-01	9.64E-01
**PKHD1**	-0.86	1.00E-01	1.00E+00	-0.34	7.36E-01	9.64E-01
**MAPKAPK5-AS1**	0.40	2.19E-01	1.00E+00	-0.13	7.36E-01	9.64E-01
**AC105052.4**	-0.42	5.86E-01	1.00E+00	0.19	7.36E-01	9.64E-01
**AL162274.2**	-0.26	7.78E-01	1.00E+00	0.19	7.36E-01	9.64E-01
**SDF2L1**	-0.38	5.73E-02	1.00E+00	0.11	7.36E-01	9.64E-01
**JAZF1**	0.24	3.67E-01	1.00E+00	-0.10	7.36E-01	9.64E-01
**GPR89A**	-0.12	6.08E-01	1.00E+00	-0.08	7.36E-01	9.64E-01
**LINC01359**	-2.03	3.89E-01	1.00E+00	-0.38	7.36E-01	9.64E-01
**EEF1DP1**	-0.06	9.23E-01	1.00E+00	-0.23	7.36E-01	9.65E-01
**LINC00513**	2.21	1.00E-01	1.00E+00	0.28	7.36E-01	9.65E-01
**IGKV1-17**	4.05	1.27E-01	1.00E+00	1.08	7.36E-01	9.65E-01
**AC007842.1**	-2.74	4.15E-02	1.00E+00	0.46	7.36E-01	9.65E-01
**IGFL4**	-1.07	1.28E-01	1.00E+00	0.23	7.36E-01	9.65E-01
**KRTAP10-5**	-18.79	2.66E-08	1.20E-05	-1.08	7.36E-01	9.65E-01
**C20orf202**	0.49	3.54E-01	1.00E+00	0.25	7.37E-01	9.65E-01
**ADH4**	1.24	4.25E-01	1.00E+00	-0.38	7.37E-01	9.65E-01
**CCDC148**	0.34	6.18E-01	1.00E+00	0.25	7.37E-01	9.65E-01
**MCPH1-AS1**	0.11	7.97E-01	1.00E+00	-0.14	7.37E-01	9.65E-01
**FSD1L**	0.29	7.82E-01	1.00E+00	-0.24	7.37E-01	9.65E-01
**CNST**	0.10	7.29E-01	1.00E+00	-0.07	7.37E-01	9.65E-01
**RFX3-AS1**	0.79	2.98E-01	1.00E+00	0.17	7.37E-01	9.65E-01
**AC136475.2**	-0.27	5.99E-01	1.00E+00	0.23	7.37E-01	9.65E-01
**AMACR**	-0.53	5.33E-01	1.00E+00	0.17	7.37E-01	9.65E-01
**AC006141.1**	3.12	3.08E-02	1.00E+00	0.27	7.37E-01	9.65E-01
**SEH1L**	0.06	8.08E-01	1.00E+00	-0.09	7.37E-01	9.65E-01
**PCDHA13**	-1.93	1.95E-01	1.00E+00	-0.58	7.37E-01	9.65E-01

**SCLT1**	-0.22	4.58E-01	1.00E+00	-0.07	7.37E-01	9.65E-01
**G25515**	-1.50	5.47E-01	1.00E+00	-0.40	7.37E-01	9.65E-01
**LINC00115**	-1.37	2.19E-01	1.00E+00	0.23	7.37E-01	9.65E-01
**TFAP2A**	-0.45	2.13E-01	1.00E+00	0.18	7.37E-01	9.65E-01
**SINHCAF**	0.01	9.81E-01	1.00E+00	-0.09	7.37E-01	9.65E-01
**TMEM170B**	1.14	1.12E-01	1.00E+00	-0.20	7.37E-01	9.65E-01
**LRRTM4**	0.08	9.66E-01	1.00E+00	-0.43	7.37E-01	9.65E-01
**SNORC**	1.01	2.59E-01	1.00E+00	0.26	7.37E-01	9.65E-01
**EWSAT1**	2.55	3.21E-02	1.00E+00	0.34	7.37E-01	9.65E-01
**KCNJ3**	-1.69	5.54E-01	1.00E+00	-0.20	7.38E-01	9.65E-01
**AC092910.3**	0.16	7.86E-01	1.00E+00	-0.27	7.38E-01	9.65E-01
**SUMF1**	0.09	6.75E-01	1.00E+00	0.08	7.38E-01	9.65E-01
**UBE2J2**	-0.18	4.37E-01	1.00E+00	0.05	7.38E-01	9.65E-01
**GIPR**	-1.66	2.13E-02	8.94E-01	0.17	7.38E-01	9.65E-01
**STK38L**	0.17	4.95E-01	1.00E+00	-0.08	7.38E-01	9.65E-01
**MYL12A**	0.09	5.60E-01	1.00E+00	-0.10	7.38E-01	9.65E-01
**XLOC_009810**	-0.80	8.18E-01	1.00E+00	-0.49	7.38E-01	9.65E-01
**AL136162.1**	-2.20	8.98E-03	5.88E-01	-0.32	7.38E-01	9.65E-01
**DEAF1**	-0.39	8.79E-02	1.00E+00	-0.14	7.38E-01	9.65E-01
**HOMEZ**	0.18	5.72E-01	1.00E+00	-0.13	7.38E-01	9.65E-01
**HCG27**	1.24	2.55E-01	1.00E+00	0.32	7.38E-01	9.65E-01
**LAMTOR5-AS1**	-0.33	6.95E-01	1.00E+00	-0.20	7.38E-01	9.65E-01
**STARD6**	1.95	2.87E-01	1.00E+00	0.49	7.38E-01	9.65E-01
**LINC01637**	-3.18	2.10E-02	8.94E-01	-0.25	7.38E-01	9.65E-01
**KLHL20**	-0.08	6.64E-01	1.00E+00	0.06	7.38E-01	9.65E-01
**TES**	-0.15	4.30E-01	1.00E+00	-0.05	7.38E-01	9.65E-01
**RPL21P134**	2.33	4.95E-01	1.00E+00	0.26	7.38E-01	9.65E-01
**AC087481.3**	0.16	6.97E-01	1.00E+00	0.25	7.38E-01	9.65E-01
**RIMS4**	1.24	3.98E-01	1.00E+00	0.41	7.38E-01	9.65E-01
**AC010969.1**	4.37	9.55E-02	1.00E+00	-0.40	7.38E-01	9.65E-01
**XLOC_006335**	-0.26	7.49E-01	1.00E+00	0.31	7.39E-01	9.65E-01
**XLOC_011031**	-0.80	2.73E-01	1.00E+00	-0.22	7.39E-01	9.65E-01

**GNG7**	-0.03	9.63E-01	1.00E+00	-0.14	7.39E-01	9.65E-01
**MDGA2**	-0.28	6.91E-01	1.00E+00	-0.35	7.39E-01	9.65E-01
**RPGR**	0.41	7.54E-02	1.00E+00	0.11	7.39E-01	9.65E-01
**G35484**	-1.23	7.24E-01	1.00E+00	1.06	7.39E-01	9.65E-01
**AC087741.3**	1.00	4.81E-02	1.00E+00	0.26	7.39E-01	9.65E-01
**XLOC_005180**	-1.52	1.85E-01	1.00E+00	0.35	7.39E-01	9.65E-01
**COPS6**	-0.06	7.51E-01	1.00E+00	-0.08	7.39E-01	9.65E-01
**G11188**	0.49	6.70E-02	1.00E+00	-0.18	7.39E-01	9.65E-01
**XLOC_011677**	-2.12	2.29E-01	1.00E+00	0.49	7.39E-01	9.65E-01
**SLC26A11**	-0.21	4.29E-01	1.00E+00	0.18	7.39E-01	9.65E-01
**AL031775.2**	2.30	3.94E-02	1.00E+00	0.34	7.39E-01	9.65E-01
**SNRPGP2**	-0.55	7.91E-01	1.00E+00	-0.19	7.39E-01	9.65E-01
**CBX4**	-0.84	3.02E-02	1.00E+00	-0.23	7.39E-01	9.65E-01
**TMBIM1**	0.21	1.62E-01	1.00E+00	-0.06	7.39E-01	9.65E-01
**XLOC_003406**	0.57	4.68E-01	1.00E+00	-0.39	7.39E-01	9.65E-01
**NCOA4**	0.06	7.68E-01	1.00E+00	-0.06	7.39E-01	9.65E-01
**TRAPPC12**	-0.21	4.80E-01	1.00E+00	-0.16	7.39E-01	9.65E-01
**GATA2**	0.55	1.62E-01	1.00E+00	0.22	7.39E-01	9.65E-01
**FZD9**	0.36	2.57E-01	1.00E+00	-0.29	7.39E-01	9.65E-01
**FAM215B**	1.39	3.81E-01	1.00E+00	0.36	7.40E-01	9.65E-01
**SNORA7**	-1.45	5.00E-01	1.00E+00	0.24	7.40E-01	9.65E-01
**ARSE**	-0.38	5.46E-01	1.00E+00	0.24	7.40E-01	9.66E-01
**CFAP77**	-0.90	7.47E-01	1.00E+00	0.39	7.40E-01	9.66E-01
**MRPS27**	-0.09	6.53E-01	1.00E+00	0.05	7.40E-01	9.66E-01
**CMTM5**	0.47	5.00E-01	1.00E+00	-0.21	7.40E-01	9.66E-01
**AC110285.1**	-0.05	9.20E-01	1.00E+00	0.26	7.40E-01	9.66E-01
**LINC01198**	0.91	5.51E-01	1.00E+00	-0.31	7.40E-01	9.66E-01
**CCND1**	0.37	6.38E-02	1.00E+00	-0.11	7.40E-01	9.66E-01
**LINC01869**	-2.54	2.19E-01	1.00E+00	-0.40	7.40E-01	9.66E-01
**LRRC37B**	-0.04	9.30E-01	1.00E+00	-0.14	7.40E-01	9.66E-01
**PEX10**	-0.29	2.75E-01	1.00E+00	-0.11	7.40E-01	9.66E-01
**KLK11**	-0.07	8.62E-01	1.00E+00	-0.19	7.40E-01	9.66E-01

**NLRP6**	-1.69	2.31E-02	9.12E-01	0.99	7.40E-01	9.66E-01
**PCSK1N**	-0.85	1.10E-01	1.00E+00	0.21	7.40E-01	9.66E-01
**RB1CC1**	0.09	7.36E-01	1.00E+00	-0.07	7.40E-01	9.66E-01
**ATP5F1AP3**	0.36	8.97E-01	1.00E+00	-0.25	7.40E-01	9.66E-01
**CFAP57**	0.49	5.84E-01	1.00E+00	0.21	7.41E-01	9.66E-01
**TM9SF1**	0.11	7.47E-01	1.00E+00	-0.09	7.41E-01	9.66E-01
**G10019**	0.82	6.82E-01	1.00E+00	0.46	7.41E-01	9.66E-01
**SRP54**	0.01	9.45E-01	1.00E+00	-0.05	7.41E-01	9.66E-01
**G23966**	0.57	5.38E-01	1.00E+00	0.21	7.41E-01	9.66E-01
**LMNB2**	-0.07	7.30E-01	1.00E+00	0.20	7.41E-01	9.66E-01
**CMTM1**	0.22	6.80E-01	1.00E+00	0.19	7.41E-01	9.66E-01
**XLOC_001286**	-0.87	6.50E-01	1.00E+00	0.70	7.41E-01	9.66E-01
**CCDC149**	-0.05	8.68E-01	1.00E+00	0.16	7.41E-01	9.66E-01
**KRTAP10-12**	-30.00	3.46E-18	5.54E-15	-1.06	7.41E-01	9.66E-01
**AQP1**	0.01	9.81E-01	1.00E+00	-0.15	7.41E-01	9.66E-01
**DIRAS2**	-1.60	1.32E-02	7.22E-01	0.28	7.41E-01	9.66E-01
**Z83851.2**	-2.14	2.36E-02	9.15E-01	0.31	7.42E-01	9.66E-01
**MRPL37**	-0.19	3.22E-01	1.00E+00	0.08	7.42E-01	9.66E-01
**SPA17**	-0.05	9.23E-01	1.00E+00	-0.11	7.42E-01	9.66E-01
**RSPH14**	-0.39	3.46E-01	1.00E+00	0.25	7.42E-01	9.66E-01
**PUF60**	-0.39	1.29E-01	1.00E+00	0.06	7.42E-01	9.66E-01
**DEK**	-0.03	8.85E-01	1.00E+00	-0.06	7.42E-01	9.66E-01
**TRPV2**	0.09	8.20E-01	1.00E+00	0.18	7.42E-01	9.66E-01
**RSRC1**	-0.12	5.53E-01	1.00E+00	-0.07	7.42E-01	9.66E-01
**CIPC**	-0.21	4.54E-01	1.00E+00	-0.09	7.42E-01	9.66E-01
**ZSCAN9**	-0.05	8.94E-01	1.00E+00	0.14	7.42E-01	9.66E-01
**G9222**	-0.02	9.81E-01	1.00E+00	0.33	7.42E-01	9.66E-01
**TDP1**	-0.07	7.75E-01	1.00E+00	0.08	7.42E-01	9.66E-01
**NOXRED1**	0.30	8.23E-01	1.00E+00	0.41	7.42E-01	9.66E-01
**AC011379.1**	-0.06	9.86E-01	1.00E+00	-0.39	7.42E-01	9.66E-01
**TAF1D**	-0.15	3.14E-01	1.00E+00	0.08	7.43E-01	9.66E-01
**RPS2**	-0.44	1.83E-01	1.00E+00	-0.19	7.43E-01	9.66E-01

**AHNAK2**	-0.25	5.34E-01	1.00E+00	0.15	7.43E-01	9.66E-01
**MRRF**	-0.43	6.63E-02	1.00E+00	-0.06	7.43E-01	9.66E-01
**NPIPP1**	0.20	8.23E-01	1.00E+00	-0.19	7.43E-01	9.66E-01
**FAM192BP**	-1.52	5.23E-01	1.00E+00	-0.19	7.43E-01	9.66E-01
**M1AP**	-0.70	4.24E-01	1.00E+00	0.28	7.43E-01	9.66E-01
**GDF9**	-0.31	5.61E-01	1.00E+00	-0.31	7.43E-01	9.66E-01
**AC027117.1**	1.27	3.66E-01	1.00E+00	-0.27	7.43E-01	9.66E-01
**GTF3C5**	-0.23	2.94E-01	1.00E+00	0.06	7.43E-01	9.66E-01
**RBM23**	0.03	8.45E-01	1.00E+00	0.09	7.43E-01	9.66E-01
**AC116913.1**	-0.88	3.07E-01	1.00E+00	0.18	7.43E-01	9.66E-01
**AC025035.1**	-0.24	8.53E-01	1.00E+00	0.43	7.43E-01	9.66E-01
**API5P1**	0.91	7.69E-01	1.00E+00	0.18	7.43E-01	9.66E-01
**WWC2**	0.33	4.45E-01	1.00E+00	0.15	7.43E-01	9.66E-01
**GBP7**	4.18	1.67E-01	1.00E+00	0.30	7.43E-01	9.66E-01
**UNC79**	-0.53	5.16E-01	1.00E+00	-0.27	7.43E-01	9.66E-01
**PROS1**	0.15	4.33E-01	1.00E+00	-0.09	7.43E-01	9.66E-01
**GLYATL1**	0.53	4.47E-01	1.00E+00	-0.21	7.43E-01	9.66E-01
**ASXL2**	-0.10	6.22E-01	1.00E+00	0.11	7.43E-01	9.66E-01
**STX2**	0.33	4.38E-01	1.00E+00	-0.16	7.43E-01	9.66E-01
**AL109614.1**	2.79	2.01E-01	1.00E+00	-0.38	7.43E-01	9.66E-01
**ACTR1A**	-0.02	8.57E-01	1.00E+00	0.05	7.43E-01	9.66E-01
**CASP3**	-0.02	9.32E-01	1.00E+00	0.08	7.43E-01	9.66E-01
**MAIP1**	-0.03	8.92E-01	1.00E+00	-0.09	7.43E-01	9.66E-01
**AP005136.2**	-1.44	6.75E-01	1.00E+00	0.45	7.43E-01	9.66E-01
**PKD1L2**	0.56	4.08E-01	1.00E+00	0.75	7.43E-01	9.66E-01
**TNFRSF10A**	-0.50	4.80E-02	1.00E+00	0.12	7.43E-01	9.66E-01
**DSC3**	-0.10	7.84E-01	1.00E+00	-0.19	7.44E-01	9.66E-01
**CDT1**	-0.77	1.78E-02	8.38E-01	0.18	7.44E-01	9.66E-01
**JMJD7**	-1.57	4.05E-01	1.00E+00	-0.27	7.44E-01	9.66E-01
**AL512598.1**	-2.45	3.77E-01	1.00E+00	0.27	7.44E-01	9.66E-01
**NSUN5P1**	-0.21	4.99E-01	1.00E+00	-0.10	7.44E-01	9.66E-01
**FANK1**	0.64	5.62E-02	1.00E+00	0.12	7.44E-01	9.66E-01

**GAD1**	-0.04	9.72E-01	1.00E+00	0.40	7.44E-01	9.66E-01
**BOP1**	-0.41	2.34E-01	1.00E+00	-0.28	7.44E-01	9.66E-01
**PDE6B**	0.67	3.28E-01	1.00E+00	0.19	7.44E-01	9.66E-01
**AL136084.3**	1.63	6.32E-01	1.00E+00	-0.35	7.44E-01	9.66E-01
**CKMT2-AS1**	0.03	9.38E-01	1.00E+00	-0.12	7.44E-01	9.66E-01
**LINC02073**	-1.65	1.44E-01	1.00E+00	-0.39	7.44E-01	9.66E-01
**AL365475.1**	-1.60	6.34E-01	1.00E+00	-0.33	7.44E-01	9.66E-01
**EFNA4**	-0.11	7.05E-01	1.00E+00	-0.15	7.44E-01	9.66E-01
**TNS4**	-0.72	8.52E-02	1.00E+00	0.21	7.44E-01	9.66E-01
**AP002495.1**	0.25	5.45E-01	1.00E+00	0.15	7.44E-01	9.66E-01
**H2AFY2**	-0.32	2.21E-01	1.00E+00	-0.09	7.44E-01	9.66E-01
**MALAT1**	-0.01	9.87E-01	1.00E+00	0.10	7.44E-01	9.66E-01
**MFSD3**	-0.32	3.53E-01	1.00E+00	0.15	7.44E-01	9.66E-01
**KCNMB2-AS1**	NA	NA	NA	-0.42	7.44E-01	9.66E-01
**ZNF780B**	0.44	3.69E-01	1.00E+00	-0.13	7.44E-01	9.66E-01
**CD99**	0.71	6.59E-01	1.00E+00	-0.23	7.44E-01	9.66E-01
**AL356488.2**	-0.14	7.40E-01	1.00E+00	-0.14	7.44E-01	9.66E-01
**FAM25A**	-2.34	3.00E-01	1.00E+00	0.30	7.44E-01	9.66E-01
**KCNT2**	0.11	8.29E-01	1.00E+00	0.31	7.44E-01	9.66E-01
**C6orf99**	-0.02	9.76E-01	1.00E+00	0.24	7.44E-01	9.66E-01
**DNAH10**	0.07	9.19E-01	1.00E+00	-0.18	7.44E-01	9.66E-01
**OR2A1**	0.28	7.99E-01	1.00E+00	-0.39	7.44E-01	9.66E-01
**UTRN**	-0.21	3.21E-01	1.00E+00	0.09	7.44E-01	9.66E-01
**FRRS1**	0.38	3.63E-01	1.00E+00	0.24	7.44E-01	9.66E-01
**AC008014.1**	-0.01	9.85E-01	1.00E+00	-0.16	7.44E-01	9.66E-01
**TMEM171**	-1.08	7.86E-02	1.00E+00	-0.20	7.44E-01	9.66E-01
**LINC02515**	-0.33	7.50E-01	1.00E+00	0.34	7.44E-01	9.66E-01
**RPL7P6**	-0.83	6.36E-01	1.00E+00	0.31	7.44E-01	9.66E-01
**RORC**	-0.18	5.15E-01	1.00E+00	-0.14	7.44E-01	9.66E-01
**RMND5A**	0.28	3.03E-01	1.00E+00	-0.11	7.44E-01	9.66E-01
**PNRC2**	-0.01	9.53E-01	1.00E+00	0.06	7.44E-01	9.66E-01
**AL683813.1**	0.87	3.44E-01	1.00E+00	-0.29	7.45E-01	9.66E-01

**RN7SKP230**	-3.77	4.82E-02	1.00E+00	-0.28	7.45E-01	9.66E-01
**RPL26P19**	-0.49	3.19E-01	1.00E+00	-0.16	7.45E-01	9.66E-01
**PSMD8**	0.02	9.02E-01	1.00E+00	-0.05	7.45E-01	9.66E-01
**HGD**	3.47	2.13E-01	1.00E+00	-1.04	7.45E-01	9.66E-01
**ATP13A1**	-0.29	1.76E-01	1.00E+00	0.20	7.45E-01	9.66E-01
**AEBP2**	-0.12	6.64E-01	1.00E+00	-0.13	7.45E-01	9.66E-01
**AL357140.5**	0.09	9.50E-01	1.00E+00	0.39	7.45E-01	9.66E-01
**IGFL3**	0.12	7.87E-01	1.00E+00	-0.24	7.45E-01	9.66E-01
**APOA1**	-0.96	6.21E-01	1.00E+00	-0.22	7.45E-01	9.66E-01
**TDRD10**	0.66	1.42E-01	1.00E+00	-0.38	7.45E-01	9.66E-01
**RPL13AP7**	-0.46	6.37E-01	1.00E+00	0.26	7.45E-01	9.66E-01
**MORF4L1P1**	0.10	7.08E-01	1.00E+00	0.11	7.45E-01	9.66E-01
**ARHGEF3**	-0.46	1.96E-02	8.75E-01	0.09	7.45E-01	9.66E-01
**ATXN2-AS**	2.07	5.39E-01	1.00E+00	-0.34	7.45E-01	9.66E-01
**G25045**	-2.69	2.37E-01	1.00E+00	-0.43	7.45E-01	9.66E-01
**ZNF43**	0.07	8.41E-01	1.00E+00	-0.10	7.45E-01	9.66E-01
**DPF3**	-0.35	3.57E-01	1.00E+00	-0.20	7.45E-01	9.66E-01
**CPM**	0.21	3.26E-01	1.00E+00	-0.09	7.45E-01	9.66E-01
**NIPAL2**	0.01	9.88E-01	1.00E+00	-0.10	7.45E-01	9.66E-01
**PXMP4**	-0.39	2.53E-01	1.00E+00	-0.12	7.45E-01	9.66E-01
**PARAL1**	5.55	7.44E-03	5.24E-01	-0.35	7.45E-01	9.66E-01
**THAP12**	0.31	1.58E-01	1.00E+00	0.16	7.45E-01	9.66E-01
**LHX4**	-1.42	3.27E-01	1.00E+00	0.42	7.45E-01	9.66E-01
**ZSCAN12P1**	0.33	6.83E-01	1.00E+00	0.21	7.45E-01	9.66E-01
**AC027228.2**	1.17	3.46E-01	1.00E+00	-0.28	7.45E-01	9.66E-01
**GCC1**	-0.01	9.30E-01	1.00E+00	0.10	7.45E-01	9.66E-01
**ARPC3P5**	-0.80	8.18E-01	1.00E+00	0.26	7.45E-01	9.66E-01
**TEX22**	0.24	8.50E-01	1.00E+00	0.40	7.45E-01	9.66E-01
**CASC11**	-2.82	2.25E-01	1.00E+00	-0.33	7.45E-01	9.66E-01
**PHAX**	0.02	9.27E-01	1.00E+00	-0.07	7.45E-01	9.66E-01
**PSME1**	-0.09	7.10E-01	1.00E+00	0.06	7.45E-01	9.66E-01
**LINC00648**	-2.83	1.90E-01	1.00E+00	0.43	7.45E-01	9.66E-01

**MKI67**	-0.42	1.76E-01	1.00E+00	0.21	7.45E-01	9.66E-01
**BRAF**	0.01	9.46E-01	1.00E+00	0.09	7.45E-01	9.66E-01
**RRNAD1**	-0.10	5.46E-01	1.00E+00	0.09	7.45E-01	9.66E-01
**C5orf51**	0.04	8.40E-01	1.00E+00	0.11	7.45E-01	9.66E-01
**G6277**	0.72	1.44E-01	1.00E+00	0.20	7.46E-01	9.66E-01
**SLC6A16**	0.51	1.31E-01	1.00E+00	0.20	7.46E-01	9.66E-01
**AC009902.2**	-2.91	5.03E-02	1.00E+00	0.26	7.46E-01	9.66E-01
**AC015802.6**	0.23	8.04E-01	1.00E+00	-0.32	7.46E-01	9.66E-01
**B3GNT2**	-0.01	9.74E-01	1.00E+00	0.11	7.46E-01	9.66E-01
**ARL4AP1**	-2.93	1.17E-01	1.00E+00	-0.83	7.46E-01	9.66E-01
**TTC38**	0.62	6.09E-03	4.63E-01	-0.10	7.46E-01	9.66E-01
**STOM**	0.20	4.37E-01	1.00E+00	-0.10	7.46E-01	9.66E-01
**RAMP2-AS1**	0.21	7.16E-01	1.00E+00	0.63	7.46E-01	9.66E-01
**AL157813.1**	-1.19	3.82E-01	1.00E+00	0.21	7.46E-01	9.66E-01
**PHBP19**	0.36	9.16E-01	1.00E+00	0.43	7.46E-01	9.66E-01
**AC104134.1**	-2.73	2.39E-01	1.00E+00	-0.47	7.46E-01	9.66E-01
**MTCO3P12**	-0.42	3.34E-01	1.00E+00	-0.21	7.46E-01	9.66E-01
**LINC02055**	-2.84	9.86E-02	1.00E+00	-1.03	7.46E-01	9.66E-01
**DNM1**	0.11	8.04E-01	1.00E+00	0.19	7.46E-01	9.66E-01
**ZDHHC11**	0.26	7.38E-01	1.00E+00	0.32	7.46E-01	9.66E-01
**AC012073.1**	0.52	6.78E-01	1.00E+00	0.43	7.46E-01	9.66E-01
**AURKB**	0.09	7.87E-01	1.00E+00	0.18	7.46E-01	9.66E-01
**CEP76**	0.11	7.88E-01	1.00E+00	-0.08	7.47E-01	9.66E-01
**AP001636.3**	0.12	8.29E-01	1.00E+00	-0.17	7.47E-01	9.66E-01
**HOXA4**	-0.62	4.08E-01	1.00E+00	-0.23	7.47E-01	9.66E-01
**ARHGEF35**	0.14	7.50E-01	1.00E+00	-0.14	7.47E-01	9.66E-01
**CARMN**	0.29	6.81E-01	1.00E+00	-0.18	7.47E-01	9.66E-01
**P4HTM**	-0.08	7.27E-01	1.00E+00	-0.14	7.47E-01	9.66E-01
**PTPRB**	0.29	6.09E-01	1.00E+00	0.19	7.47E-01	9.66E-01
**RPL22P2**	0.56	6.95E-01	1.00E+00	-0.18	7.47E-01	9.66E-01
**C2CD3**	0.07	7.26E-01	1.00E+00	0.18	7.47E-01	9.66E-01
**AL031777.1**	-3.04	2.50E-02	9.28E-01	-0.47	7.47E-01	9.66E-01

**ACOD1**	NA	NA	NA	0.70	7.47E-01	9.66E-01
**FREM1**	0.77	7.26E-02	1.00E+00	-0.23	7.47E-01	9.66E-01
**RAB38**	0.04	9.33E-01	1.00E+00	0.20	7.47E-01	9.66E-01
**ARL6IP6**	0.11	7.70E-01	1.00E+00	-0.12	7.47E-01	9.66E-01
**SUPT3H**	0.06	8.71E-01	1.00E+00	0.11	7.47E-01	9.66E-01
**HEIH**	0.08	7.64E-01	1.00E+00	-0.11	7.47E-01	9.66E-01
**RBBP6**	-0.27	3.70E-01	1.00E+00	0.12	7.47E-01	9.66E-01
**HSP90AB2P**	-1.33	7.02E-01	1.00E+00	-0.20	7.47E-01	9.66E-01
**FANCM**	-0.59	2.15E-01	1.00E+00	-0.09	7.47E-01	9.66E-01
**CST1**	-5.85	7.91E-02	1.00E+00	-0.66	7.47E-01	9.66E-01
**RALB**	0.05	8.13E-01	1.00E+00	-0.08	7.47E-01	9.66E-01
**XLOC_006923**	-2.57	4.51E-01	1.00E+00	0.38	7.47E-01	9.66E-01
**RAB41**	1.26	1.47E-02	7.61E-01	-0.21	7.47E-01	9.66E-01
**HERC4**	0.16	6.05E-01	1.00E+00	0.06	7.48E-01	9.66E-01
**CTNND1**	-0.18	4.33E-01	1.00E+00	-0.07	7.48E-01	9.66E-01
**AJ009632.2**	-2.85	1.94E-01	1.00E+00	-0.44	7.48E-01	9.66E-01
**FAM173A**	-0.45	1.41E-01	1.00E+00	0.19	7.48E-01	9.66E-01
**LMOD3**	-0.06	9.64E-01	1.00E+00	-0.28	7.48E-01	9.66E-01
**CEP57**	0.27	2.99E-01	1.00E+00	-0.06	7.48E-01	9.66E-01
**AL359091.5**	0.29	8.29E-01	1.00E+00	0.33	7.48E-01	9.66E-01
**TMPRSS11GP**	4.69	6.76E-02	1.00E+00	-1.00	7.48E-01	9.66E-01
**RNF165**	-0.62	1.48E-01	1.00E+00	-0.18	7.48E-01	9.66E-01
**PNKD**	0.11	6.19E-01	1.00E+00	0.09	7.48E-01	9.66E-01
**GSK3A**	-0.07	7.55E-01	1.00E+00	0.16	7.48E-01	9.66E-01
**AL358942.1**	-2.66	4.18E-01	1.00E+00	-0.20	7.48E-01	9.66E-01
**FER1L4**	0.98	3.35E-01	1.00E+00	-0.37	7.48E-01	9.66E-01
**LINC00624**	0.45	6.60E-01	1.00E+00	-0.30	7.48E-01	9.66E-01
**CARS**	0.16	5.56E-01	1.00E+00	0.05	7.48E-01	9.66E-01
**SMG1P4**	-3.50	2.13E-01	1.00E+00	-0.33	7.48E-01	9.66E-01
**KIAA2012**	-3.70	4.08E-02	1.00E+00	-0.22	7.48E-01	9.66E-01
**CDK14**	0.21	6.74E-01	1.00E+00	0.13	7.48E-01	9.66E-01
**GNL3**	0.00	9.86E-01	1.00E+00	0.05	7.48E-01	9.66E-01

**DHODH**	0.17	3.95E-01	1.00E+00	0.09	7.48E-01	9.66E-01
**FPGS**	-0.09	7.59E-01	1.00E+00	0.12	7.49E-01	9.66E-01
**DNAJC10**	-0.10	7.03E-01	1.00E+00	-0.06	7.49E-01	9.67E-01
**PPM1M**	0.33	3.28E-01	1.00E+00	0.17	7.49E-01	9.67E-01
**ALDH8A1**	0.24	7.88E-01	1.00E+00	0.38	7.49E-01	9.67E-01
**AL359922.2**	-1.62	5.96E-01	1.00E+00	-0.54	7.49E-01	9.67E-01
**INSR**	0.33	3.27E-01	1.00E+00	-0.08	7.49E-01	9.67E-01
**IGSF9B**	-0.74	2.55E-01	1.00E+00	0.43	7.49E-01	9.67E-01
**TIGD3**	-1.93	1.44E-01	1.00E+00	0.35	7.49E-01	9.67E-01
**AL161645.1**	-3.01	3.19E-02	1.00E+00	0.42	7.49E-01	9.67E-01
**ZNF671**	0.65	1.26E-01	1.00E+00	0.19	7.49E-01	9.67E-01
**TRH**	-0.89	5.88E-01	1.00E+00	0.45	7.49E-01	9.67E-01
**GDI2**	0.00	9.83E-01	1.00E+00	-0.04	7.49E-01	9.67E-01
**CEP295**	0.15	5.85E-01	1.00E+00	-0.09	7.49E-01	9.67E-01
**AC092756.1**	-3.52	3.60E-02	1.00E+00	-0.29	7.49E-01	9.67E-01
**AC100858.3**	-3.43	1.19E-02	6.78E-01	-0.23	7.49E-01	9.67E-01
**AC011495.1**	0.03	9.80E-01	1.00E+00	0.20	7.49E-01	9.67E-01
**AC133134.1**	-0.67	8.48E-01	1.00E+00	-0.25	7.49E-01	9.67E-01
**SPDL1**	-0.11	7.26E-01	1.00E+00	-0.09	7.49E-01	9.67E-01
**ZNF414**	-0.51	1.77E-01	1.00E+00	-0.19	7.49E-01	9.67E-01
**SMARCA4**	-0.31	1.65E-01	1.00E+00	0.12	7.49E-01	9.67E-01
**LYG2**	-4.53	7.65E-02	1.00E+00	-1.01	7.50E-01	9.67E-01
**PTBP3**	0.07	8.27E-01	1.00E+00	-0.11	7.50E-01	9.67E-01
**LINC01106**	-1.04	1.99E-01	1.00E+00	-0.22	7.50E-01	9.67E-01
**AL353898.2**	1.59	4.07E-01	1.00E+00	0.39	7.50E-01	9.67E-01
**MOGS**	-0.30	3.09E-01	1.00E+00	0.16	7.50E-01	9.67E-01
**ATR**	0.09	6.03E-01	1.00E+00	-0.12	7.50E-01	9.67E-01
**AC008663.2**	-0.28	7.96E-01	1.00E+00	-0.40	7.50E-01	9.67E-01
**HECA**	0.16	6.23E-01	1.00E+00	-0.08	7.50E-01	9.67E-01
**BZW1**	0.00	9.89E-01	1.00E+00	-0.07	7.50E-01	9.67E-01
**IGLV5-45**	0.74	8.28E-01	1.00E+00	1.02	7.50E-01	9.67E-01
**RAD9B**	0.11	8.87E-01	1.00E+00	0.26	7.50E-01	9.67E-01

**KIZ**	-0.13	5.96E-01	1.00E+00	-0.09	7.50E-01	9.67E-01
**FGD6**	-0.04	9.10E-01	1.00E+00	-0.13	7.50E-01	9.67E-01
**LINC00910**	-0.51	2.67E-01	1.00E+00	0.09	7.50E-01	9.67E-01
**MARS2**	-0.25	3.57E-01	1.00E+00	-0.13	7.50E-01	9.67E-01
**CCDC151**	-0.48	4.25E-01	1.00E+00	-0.30	7.50E-01	9.67E-01
**AC090912.1**	-0.56	7.00E-01	1.00E+00	0.21	7.50E-01	9.67E-01
**HSD3B7**	0.05	8.65E-01	1.00E+00	-0.10	7.50E-01	9.67E-01
**AL365357.1**	-3.10	7.64E-02	1.00E+00	-1.00	7.50E-01	9.67E-01
**CYP2C18**	-1.09	1.12E-01	1.00E+00	-0.23	7.50E-01	9.67E-01
**LINC02381**	0.36	3.10E-01	1.00E+00	0.10	7.50E-01	9.67E-01
**DEDD**	0.08	6.60E-01	1.00E+00	-0.07	7.50E-01	9.67E-01
**KL**	0.20	7.60E-01	1.00E+00	-0.25	7.50E-01	9.67E-01
**APBA1**	0.00	9.99E-01	1.00E+00	0.22	7.50E-01	9.67E-01
**CTD-2173L22.4**	-0.41	4.54E-01	1.00E+00	-0.20	7.50E-01	9.67E-01
**MED14OS**	-1.85	3.20E-01	1.00E+00	-0.24	7.51E-01	9.67E-01
**LINC02086**	-2.23	5.15E-01	1.00E+00	0.61	7.51E-01	9.67E-01
**PLA2G4E-AS1**	1.01	2.25E-01	1.00E+00	0.28	7.51E-01	9.67E-01
**WDFY3**	-0.01	9.76E-01	1.00E+00	0.15	7.51E-01	9.67E-01
**NEDD9**	0.07	9.10E-01	1.00E+00	-0.17	7.51E-01	9.67E-01
**HDHD5**	0.12	5.18E-01	1.00E+00	-0.04	7.51E-01	9.67E-01
**XLOC_004049**	-2.29	5.04E-01	1.00E+00	0.37	7.51E-01	9.67E-01
**RPL32**	-0.16	5.80E-01	1.00E+00	0.07	7.51E-01	9.67E-01
**G19170**	1.27	4.21E-01	1.00E+00	0.32	7.51E-01	9.67E-01
**AC025887.2**	0.59	6.37E-01	1.00E+00	-0.38	7.51E-01	9.67E-01
**KCTD18**	0.02	9.36E-01	1.00E+00	-0.08	7.51E-01	9.67E-01
**EAF2**	-0.21	5.33E-01	1.00E+00	0.12	7.51E-01	9.67E-01
**ASAP3**	0.03	9.06E-01	1.00E+00	0.09	7.51E-01	9.67E-01
**ARNTL2**	-0.03	9.64E-01	1.00E+00	-0.14	7.51E-01	9.67E-01
**KRIT1**	0.10	7.92E-01	1.00E+00	-0.11	7.51E-01	9.67E-01
**CFAP410**	-0.26	4.64E-01	1.00E+00	0.08	7.51E-01	9.67E-01
**LINC00885**	-0.65	1.76E-01	1.00E+00	0.21	7.51E-01	9.67E-01
**ASB7**	-0.03	9.10E-01	1.00E+00	0.08	7.51E-01	9.67E-01

**RPL13AP5**	-0.17	5.23E-01	1.00E+00	0.18	7.51E-01	9.67E-01
**LOXHD1**	-0.50	6.49E-01	1.00E+00	0.38	7.51E-01	9.67E-01
**COQ10B**	-0.15	5.85E-01	1.00E+00	-0.09	7.51E-01	9.67E-01
**ZNF514**	-0.12	6.84E-01	1.00E+00	0.09	7.51E-01	9.67E-01
**RPRD2**	-0.11	6.24E-01	1.00E+00	0.08	7.51E-01	9.67E-01
**PPP2R3C**	0.14	7.11E-01	1.00E+00	0.11	7.52E-01	9.67E-01
**ANP32AP1**	-1.91	3.22E-01	1.00E+00	0.25	7.52E-01	9.67E-01
**UBE2D3**	0.05	7.74E-01	1.00E+00	-0.06	7.52E-01	9.67E-01
**STRN3**	0.05	8.38E-01	1.00E+00	0.08	7.52E-01	9.67E-01
**SRP68**	-0.02	9.21E-01	1.00E+00	-0.07	7.52E-01	9.67E-01
**AC139887.2**	-0.10	9.08E-01	1.00E+00	0.14	7.52E-01	9.67E-01
**IKBIP**	0.09	8.20E-01	1.00E+00	0.18	7.52E-01	9.67E-01
**MYL4**	-2.01	1.65E-01	1.00E+00	0.30	7.52E-01	9.67E-01
**RP1-12G14.6**	-1.24	7.19E-01	1.00E+00	0.59	7.52E-01	9.67E-01
**NHP2**	-0.10	6.61E-01	1.00E+00	0.11	7.52E-01	9.67E-01
**CDCA4P4**	1.26	8.64E-02	1.00E+00	0.26	7.52E-01	9.67E-01
**MGA**	-0.05	8.84E-01	1.00E+00	-0.12	7.52E-01	9.67E-01
**KIAA1549L**	-1.10	8.70E-05	2.21E-02	-0.21	7.52E-01	9.67E-01
**G34066**	0.85	4.69E-01	1.00E+00	0.41	7.52E-01	9.67E-01
**FAM228A**	-0.45	6.31E-01	1.00E+00	-0.27	7.52E-01	9.67E-01
**CHN2**	0.16	6.47E-01	1.00E+00	0.17	7.52E-01	9.67E-01
**KRTAP2-4**	-30.00	4.60E-18	5.54E-15	-1.01	7.52E-01	9.67E-01
**FKTN**	0.25	4.02E-01	1.00E+00	0.09	7.52E-01	9.67E-01
**AADACL2**	0.35	5.30E-01	1.00E+00	-0.20	7.52E-01	9.67E-01
**AL157756.1**	1.14	1.98E-01	1.00E+00	0.37	7.52E-01	9.67E-01
**RPL34P34**	0.56	8.71E-01	1.00E+00	-0.24	7.52E-01	9.67E-01
**STARD4**	-0.91	1.14E-01	1.00E+00	0.08	7.52E-01	9.67E-01
**HCG20**	1.75	1.17E-01	1.00E+00	0.34	7.52E-01	9.67E-01
**AC010864.1**	-0.01	9.94E-01	1.00E+00	-0.32	7.52E-01	9.67E-01
**ZNF169**	-0.43	2.42E-01	1.00E+00	-0.16	7.52E-01	9.67E-01
**KRT24**	1.64	6.31E-01	1.00E+00	1.01	7.52E-01	9.67E-01
**RRP1B**	-0.12	5.81E-01	1.00E+00	-0.11	7.53E-01	9.67E-01

**CTDP1**	-0.26	4.55E-01	1.00E+00	0.23	7.53E-01	9.67E-01
**ABLIM2**	-0.31	3.22E-01	1.00E+00	0.14	7.53E-01	9.67E-01
**AC018647.2**	0.00	9.80E-01	1.00E+00	-0.09	7.53E-01	9.67E-01
**LINC00882**	0.39	6.73E-01	1.00E+00	-0.21	7.53E-01	9.67E-01
**PSMC1P12**	NA	NA	NA	0.39	7.53E-01	9.67E-01
**TRGV4**	1.95	5.65E-01	1.00E+00	0.42	7.53E-01	9.67E-01
**MAPKBP1**	-0.01	9.74E-01	1.00E+00	0.16	7.53E-01	9.67E-01
**AL359715.3**	-0.69	2.51E-01	1.00E+00	-0.21	7.53E-01	9.67E-01
**SLC39A2**	0.38	4.38E-01	1.00E+00	0.23	7.53E-01	9.67E-01
**AC021224.1**	-0.37	9.16E-01	1.00E+00	0.99	7.53E-01	9.67E-01
**AC016065.1**	-0.56	1.09E-01	1.00E+00	0.11	7.53E-01	9.67E-01
**AL445645.1**	-1.21	3.00E-01	1.00E+00	0.27	7.53E-01	9.67E-01
**RNF5P1**	-1.97	6.54E-02	1.00E+00	0.20	7.53E-01	9.67E-01
**PRL**	-1.74	6.11E-01	1.00E+00	1.01	7.53E-01	9.67E-01
**RPS6**	-0.17	6.43E-01	1.00E+00	-0.09	7.53E-01	9.67E-01
**CWC25**	0.43	8.68E-02	1.00E+00	0.08	7.53E-01	9.67E-01
**ORMDL1**	0.13	7.52E-01	1.00E+00	-0.14	7.53E-01	9.67E-01
**KRT38**	-19.07	9.57E-13	6.73E-10	-1.01	7.53E-01	9.67E-01
**ABHD8**	-0.03	8.85E-01	1.00E+00	0.22	7.53E-01	9.67E-01
**COL28A1**	-0.06	9.01E-01	1.00E+00	-0.21	7.53E-01	9.67E-01
**ZGLP1**	-1.30	4.61E-01	1.00E+00	0.33	7.53E-01	9.67E-01
**XLOC_002282**	0.53	5.00E-02	1.00E+00	-0.12	7.53E-01	9.67E-01
**CARMIL2**	-1.88	1.10E-02	6.48E-01	0.99	7.53E-01	9.67E-01
**FGF10-AS1**	1.27	7.13E-01	1.00E+00	0.36	7.53E-01	9.67E-01
**XLOC_002616**	-1.70	2.60E-01	1.00E+00	-0.28	7.53E-01	9.67E-01
**TOM1L2**	-0.09	8.06E-01	1.00E+00	0.12	7.53E-01	9.67E-01
**AC087284.1**	-0.73	6.72E-01	1.00E+00	-0.21	7.53E-01	9.67E-01
**MEMO1P1**	0.53	5.03E-01	1.00E+00	-0.11	7.53E-01	9.67E-01
**KRTAP2-2**	-11.69	6.66E-04	1.23E-01	-1.01	7.53E-01	9.67E-01
**RBBP8NL**	-0.04	9.14E-01	1.00E+00	-0.24	7.54E-01	9.67E-01
**CSE1L**	0.01	9.59E-01	1.00E+00	-0.08	7.54E-01	9.67E-01
**XLOC_005681**	0.55	5.07E-01	1.00E+00	0.30	7.54E-01	9.67E-01

**PHF3**	-0.17	2.92E-01	1.00E+00	0.08	7.54E-01	9.67E-01
**AHSA1**	-0.22	2.61E-01	1.00E+00	0.04	7.54E-01	9.67E-01
**CBR4**	0.01	9.79E-01	1.00E+00	-0.09	7.54E-01	9.67E-01
**BRCA1**	0.00	9.90E-01	1.00E+00	0.09	7.54E-01	9.67E-01
**RNF19A**	-0.12	4.79E-01	1.00E+00	0.06	7.54E-01	9.67E-01
**CCT7**	-0.11	5.21E-01	1.00E+00	0.05	7.54E-01	9.67E-01
**CENPW**	0.11	6.81E-01	1.00E+00	-0.10	7.54E-01	9.67E-01
**SNORA34**	-0.39	9.07E-01	1.00E+00	0.38	7.54E-01	9.67E-01
**UBE2H**	0.13	5.02E-01	1.00E+00	0.12	7.54E-01	9.67E-01
**GSK3B**	-0.02	9.14E-01	1.00E+00	-0.15	7.54E-01	9.67E-01
**C10orf91**	-1.16	1.10E-01	1.00E+00	0.28	7.54E-01	9.67E-01
**G37296**	-0.29	5.90E-01	1.00E+00	0.19	7.54E-01	9.67E-01
**AC108734.4**	0.79	6.25E-01	1.00E+00	-0.33	7.54E-01	9.67E-01
**SGCD**	0.20	5.78E-01	1.00E+00	0.15	7.54E-01	9.67E-01
**RNF157**	0.39	6.04E-01	1.00E+00	-0.24	7.54E-01	9.67E-01
**AP000866.2**	-0.13	8.86E-01	1.00E+00	-0.29	7.54E-01	9.67E-01
**HIST2H2BC**	0.36	3.72E-01	1.00E+00	-0.20	7.54E-01	9.67E-01
**HYAL3**	-0.27	6.42E-01	1.00E+00	0.15	7.54E-01	9.67E-01
**XLOC_014082**	0.92	1.37E-01	1.00E+00	-0.28	7.55E-01	9.67E-01
**YARS2**	-0.20	3.26E-01	1.00E+00	0.06	7.55E-01	9.67E-01
**TNFRSF19**	0.03	9.37E-01	1.00E+00	-0.20	7.55E-01	9.67E-01
**CRADD**	-0.10	6.07E-01	1.00E+00	-0.10	7.55E-01	9.67E-01
**QRICH2**	0.27	6.82E-01	1.00E+00	0.99	7.55E-01	9.67E-01
**NKAIN3-IT1**	-0.89	1.18E-01	1.00E+00	0.38	7.55E-01	9.67E-01
**HOXD1**	0.53	6.04E-01	1.00E+00	0.24	7.55E-01	9.67E-01
**HSD3BP2**	-2.24	2.31E-01	1.00E+00	-0.88	7.55E-01	9.67E-01
**RNASEH2B-AS1**	1.02	5.40E-01	1.00E+00	0.30	7.55E-01	9.67E-01
**AL606834.2**	-0.21	7.44E-01	1.00E+00	-0.19	7.55E-01	9.67E-01
**RPS15AP38**	0.57	8.70E-01	1.00E+00	0.19	7.55E-01	9.67E-01
**VARS**	-0.41	1.14E-01	1.00E+00	-0.17	7.55E-01	9.67E-01
**ZNRF2P1**	-0.30	3.52E-01	1.00E+00	-0.31	7.55E-01	9.67E-01
**AC073648.6**	-1.07	2.70E-01	1.00E+00	0.22	7.55E-01	9.67E-01

**AC017002.3**	0.10	9.48E-01	1.00E+00	-0.29	7.55E-01	9.67E-01
**MGP**	0.55	3.11E-01	1.00E+00	-0.15	7.55E-01	9.67E-01
**ATP6V1E1P1**	NA	NA	NA	-0.24	7.55E-01	9.67E-01
**LINC01843**	0.93	6.79E-01	1.00E+00	-0.63	7.55E-01	9.67E-01
**AC011477.1**	0.43	6.55E-01	1.00E+00	0.13	7.55E-01	9.67E-01
**C7orf43**	-0.40	3.06E-01	1.00E+00	0.20	7.55E-01	9.67E-01
**HOXD8**	0.24	5.32E-01	1.00E+00	0.20	7.55E-01	9.67E-01
**HUS1**	0.01	9.79E-01	1.00E+00	-0.07	7.55E-01	9.67E-01
**IRAK2**	0.47	2.56E-01	1.00E+00	0.18	7.55E-01	9.67E-01
**AC026979.4**	-0.04	9.36E-01	1.00E+00	0.19	7.55E-01	9.67E-01
**NAV3**	-0.34	2.28E-01	1.00E+00	0.11	7.55E-01	9.67E-01
**SUV39H1**	-0.03	9.04E-01	1.00E+00	0.11	7.55E-01	9.67E-01
**Z97634.1**	-0.07	9.09E-01	1.00E+00	-0.24	7.55E-01	9.67E-01
**AC023389.2**	-2.89	3.92E-01	1.00E+00	-0.57	7.56E-01	9.67E-01
**AL137779.1**	3.48	2.10E-02	8.94E-01	-0.33	7.56E-01	9.67E-01
**ZFX**	0.15	5.29E-01	1.00E+00	-0.06	7.56E-01	9.67E-01
**LTO1**	0.28	3.25E-01	1.00E+00	-0.08	7.56E-01	9.67E-01
**PCDHGA2**	-0.52	2.15E-01	1.00E+00	-0.18	7.56E-01	9.67E-01
**TCEAL9**	0.04	8.68E-01	1.00E+00	-0.07	7.56E-01	9.67E-01
**YBX1P6**	-1.18	1.26E-01	1.00E+00	-0.20	7.56E-01	9.67E-01
**TUSC2**	0.17	4.25E-01	1.00E+00	0.06	7.56E-01	9.67E-01
**AC093904.4**	-2.45	1.22E-01	1.00E+00	0.43	7.56E-01	9.67E-01
**SPEF2**	0.05	8.77E-01	1.00E+00	-0.15	7.56E-01	9.67E-01
**G11189**	-1.94	3.44E-01	1.00E+00	0.30	7.56E-01	9.67E-01
**EED**	0.30	3.11E-01	1.00E+00	0.09	7.56E-01	9.67E-01
**AF228730.4**	-2.91	2.01E-01	1.00E+00	0.32	7.56E-01	9.67E-01
**C3orf67**	0.17	7.33E-01	1.00E+00	0.22	7.56E-01	9.67E-01
**ING5**	0.12	5.64E-01	1.00E+00	-0.09	7.57E-01	9.67E-01
**PRSS35**	-0.38	5.26E-01	1.00E+00	0.32	7.57E-01	9.67E-01
**ASB9**	0.17	6.61E-01	1.00E+00	0.11	7.57E-01	9.68E-01
**LDLRAP1**	-0.06	7.67E-01	1.00E+00	-0.08	7.57E-01	9.68E-01
**CNKSR3**	-0.19	2.81E-01	1.00E+00	0.15	7.57E-01	9.68E-01

**ARL5B**	-0.21	6.40E-01	1.00E+00	0.12	7.57E-01	9.68E-01
**USP1**	0.08	7.49E-01	1.00E+00	-0.06	7.57E-01	9.68E-01
**SDR39U1**	-1.26	1.77E-01	1.00E+00	-0.16	7.57E-01	9.68E-01
**AL132780.1**	-1.26	4.74E-01	1.00E+00	0.26	7.57E-01	9.68E-01
**GFOD1**	-0.22	5.05E-01	1.00E+00	0.09	7.57E-01	9.68E-01
**AC082651.3**	-0.52	3.40E-01	1.00E+00	-0.23	7.57E-01	9.68E-01
**ZNF843**	-0.65	9.15E-02	1.00E+00	0.24	7.57E-01	9.68E-01
**AP005482.3**	0.34	6.25E-01	1.00E+00	-0.13	7.57E-01	9.68E-01
**G4338**	-0.31	9.27E-01	1.00E+00	-0.35	7.58E-01	9.68E-01
**TMEM132C**	1.47	2.79E-03	2.73E-01	-0.93	7.58E-01	9.68E-01
**ADAM22**	0.36	3.86E-01	1.00E+00	-0.20	7.58E-01	9.68E-01
**GPATCH2**	0.07	8.02E-01	1.00E+00	0.06	7.58E-01	9.68E-01
**RPL13AP20**	1.69	1.83E-01	1.00E+00	0.19	7.58E-01	9.68E-01
**SERPINA12**	0.42	4.38E-01	1.00E+00	-0.26	7.58E-01	9.68E-01
**KRR1P1**	2.77	4.15E-01	1.00E+00	-0.42	7.58E-01	9.68E-01
**SOS1**	-0.15	4.20E-01	1.00E+00	-0.12	7.58E-01	9.68E-01
**ETV1**	0.43	5.14E-01	1.00E+00	-0.13	7.58E-01	9.68E-01
**AC004870.1**	-2.69	2.54E-01	1.00E+00	-0.49	7.58E-01	9.68E-01
**RHEX**	1.00	2.36E-01	1.00E+00	0.23	7.58E-01	9.68E-01
**RC3H1**	0.04	8.57E-01	1.00E+00	0.07	7.58E-01	9.68E-01
**PRSS3**	0.07	8.71E-01	1.00E+00	0.24	7.58E-01	9.68E-01
**C1orf159**	-0.12	6.96E-01	1.00E+00	0.16	7.58E-01	9.68E-01
**UNGP3**	-0.88	6.27E-01	1.00E+00	-0.29	7.58E-01	9.68E-01
**EIF2S2P4**	0.15	7.94E-01	1.00E+00	-0.12	7.58E-01	9.68E-01
**PNPLA1**	0.10	8.50E-01	1.00E+00	0.18	7.58E-01	9.68E-01
**SLC5A9**	-1.36	3.54E-02	1.00E+00	-0.26	7.58E-01	9.68E-01
**IL13RA1**	0.06	8.03E-01	1.00E+00	0.15	7.58E-01	9.68E-01
**TPT1**	-0.08	7.79E-01	1.00E+00	-0.08	7.58E-01	9.68E-01
**DHRS3**	0.11	7.01E-01	1.00E+00	-0.11	7.58E-01	9.68E-01
**GRTP1**	-0.11	7.57E-01	1.00E+00	-0.14	7.58E-01	9.68E-01
**JMJD4**	-0.05	8.70E-01	1.00E+00	0.09	7.58E-01	9.68E-01
**AC134682.1**	0.31	6.83E-01	1.00E+00	-0.38	7.58E-01	9.68E-01

**RPL34P18**	-1.53	4.08E-01	1.00E+00	-0.25	7.58E-01	9.68E-01
**G33346**	-0.36	8.94E-01	1.00E+00	-0.42	7.59E-01	9.68E-01
**S100A4**	-0.01	9.86E-01	1.00E+00	0.13	7.59E-01	9.68E-01
**ESPL1**	0.05	9.08E-01	1.00E+00	0.18	7.59E-01	9.68E-01
**SDHAF2**	-0.14	5.61E-01	1.00E+00	-0.12	7.59E-01	9.68E-01
**ST3GAL3**	-0.15	8.21E-01	1.00E+00	0.15	7.59E-01	9.68E-01
**AL138785.1**	NA	NA	NA	-0.96	7.59E-01	9.68E-01
**EEF1A1P38**	1.50	6.62E-01	1.00E+00	0.28	7.59E-01	9.68E-01
**IDH2**	-0.38	6.57E-02	1.00E+00	0.06	7.59E-01	9.68E-01
**FAM76A**	-0.31	1.60E-01	1.00E+00	-0.11	7.59E-01	9.68E-01
**RDM1P5**	1.29	2.99E-01	1.00E+00	-0.24	7.59E-01	9.68E-01
**ZFYVE9**	0.10	6.25E-01	1.00E+00	0.14	7.59E-01	9.68E-01
**COPS8**	0.21	1.91E-01	1.00E+00	0.10	7.59E-01	9.68E-01
**ZNF736**	0.54	1.24E-01	1.00E+00	0.13	7.59E-01	9.68E-01
**AC233279.1**	NA	NA	NA	0.22	7.59E-01	9.68E-01
**ACPP**	-0.17	7.75E-01	1.00E+00	-0.18	7.59E-01	9.68E-01
**SLC29A2**	-0.18	5.90E-01	1.00E+00	-0.19	7.59E-01	9.68E-01
**PRICKLE1**	-0.44	4.45E-01	1.00E+00	0.14	7.59E-01	9.68E-01
**MOSPD2**	0.24	5.01E-01	1.00E+00	-0.10	7.59E-01	9.68E-01
**AKAP5**	-0.47	3.70E-01	1.00E+00	-0.25	7.59E-01	9.68E-01
**NUPR1**	0.38	1.87E-01	1.00E+00	0.12	7.59E-01	9.68E-01
**DGKB**	-0.36	7.44E-01	1.00E+00	-0.42	7.59E-01	9.68E-01
**FAM168B**	0.21	4.20E-01	1.00E+00	0.08	7.59E-01	9.68E-01
**NUBP2**	-0.20	2.64E-01	1.00E+00	0.06	7.59E-01	9.68E-01
**TRAPPC3L**	3.28	1.38E-01	1.00E+00	-0.32	7.60E-01	9.68E-01
**HNRNPAB**	-0.30	3.13E-01	1.00E+00	-0.17	7.60E-01	9.68E-01
**IL5RA**	-0.32	8.03E-01	1.00E+00	0.33	7.60E-01	9.68E-01
**LINC00504**	-0.32	5.59E-01	1.00E+00	0.13	7.60E-01	9.68E-01
**FUCA1**	0.17	5.87E-01	1.00E+00	0.08	7.60E-01	9.68E-01
**TFPI2**	1.69	6.23E-02	1.00E+00	-0.24	7.60E-01	9.68E-01
**SRPRA**	-0.14	4.05E-01	1.00E+00	0.05	7.60E-01	9.68E-01
**SLITRK5**	-0.35	5.43E-01	1.00E+00	-0.22	7.60E-01	9.68E-01

**STAG3L2**	-0.15	5.89E-01	1.00E+00	-0.08	7.60E-01	9.68E-01
**AL117329.1**	-0.83	2.94E-01	1.00E+00	0.22	7.60E-01	9.68E-01
**ZNF730**	0.11	9.55E-01	1.00E+00	-0.46	7.60E-01	9.68E-01
**AC107952.2**	0.33	8.22E-01	1.00E+00	0.36	7.60E-01	9.68E-01
**LSM8**	0.35	2.16E-01	1.00E+00	-0.08	7.60E-01	9.68E-01
**RAMACL**	0.48	5.83E-01	1.00E+00	-0.17	7.60E-01	9.68E-01
**LBHD1**	-1.22	2.82E-01	1.00E+00	0.21	7.60E-01	9.68E-01
**AL035448.1**	-1.29	5.95E-01	1.00E+00	0.35	7.60E-01	9.68E-01
**FAM160A2**	-0.38	2.60E-01	1.00E+00	-0.16	7.60E-01	9.68E-01
**AL355312.2**	-0.72	6.03E-01	1.00E+00	0.23	7.60E-01	9.68E-01
**SGF29**	-0.10	7.57E-01	1.00E+00	0.08	7.60E-01	9.68E-01
**XLOC_002005**	0.91	3.80E-01	1.00E+00	0.42	7.60E-01	9.68E-01
**FGF18**	-0.92	3.09E-01	1.00E+00	0.27	7.60E-01	9.68E-01
**NT5DC3**	0.14	7.64E-01	1.00E+00	-0.15	7.60E-01	9.68E-01
**MYOC**	1.06	1.77E-01	1.00E+00	0.24	7.60E-01	9.68E-01
**AL512408.1**	-0.63	2.37E-01	1.00E+00	-0.24	7.60E-01	9.68E-01
**AC022144.1**	-0.60	5.72E-01	1.00E+00	0.35	7.61E-01	9.68E-01
**ABCA3**	0.12	7.74E-01	1.00E+00	-0.14	7.61E-01	9.68E-01
**SPTB**	-0.17	7.12E-01	1.00E+00	0.30	7.61E-01	9.68E-01
**THAP3**	0.26	3.52E-01	1.00E+00	0.08	7.61E-01	9.68E-01
**ZNF837**	-0.10	7.10E-01	1.00E+00	-0.27	7.61E-01	9.68E-01
**IRX5**	-0.25	4.47E-01	1.00E+00	-0.17	7.61E-01	9.68E-01
**PPP1R12B**	-0.07	8.86E-01	1.00E+00	0.14	7.61E-01	9.68E-01
**SLC26A8**	-1.13	7.40E-01	1.00E+00	0.42	7.61E-01	9.69E-01
**SPHKAP**	-4.14	1.00E-04	2.46E-02	-0.97	7.61E-01	9.69E-01
**AL080250.1**	0.01	9.85E-01	1.00E+00	0.19	7.61E-01	9.69E-01
**F2RL1**	0.26	6.45E-01	1.00E+00	0.16	7.61E-01	9.69E-01
**XLOC_010588**	-0.37	7.78E-01	1.00E+00	-0.30	7.61E-01	9.69E-01
**AC092620.2**	0.25	8.75E-01	1.00E+00	-0.36	7.61E-01	9.69E-01
**GOLGA8H**	0.62	5.84E-01	1.00E+00	0.18	7.61E-01	9.69E-01
**LSM1**	0.07	8.23E-01	1.00E+00	0.09	7.62E-01	9.69E-01
**PYCARD**	-0.27	4.25E-01	1.00E+00	0.12	7.62E-01	9.69E-01

**G17182**	-0.09	9.39E-01	1.00E+00	0.95	7.62E-01	9.69E-01
**PPP1R26-AS1**	-0.26	6.58E-01	1.00E+00	-0.25	7.62E-01	9.69E-01
**AC091057.2**	0.40	8.67E-01	1.00E+00	-0.19	7.62E-01	9.69E-01
**AC010478.1**	0.25	8.01E-01	1.00E+00	-0.39	7.62E-01	9.69E-01
**MYL6P3**	NA	NA	NA	0.25	7.62E-01	9.69E-01
**MUC15**	0.13	7.37E-01	1.00E+00	-0.19	7.62E-01	9.69E-01
**CFAP161**	-1.35	4.08E-01	1.00E+00	0.24	7.62E-01	9.69E-01
**AC004884.2**	0.32	9.28E-01	1.00E+00	-0.24	7.62E-01	9.69E-01
**PIGW**	-0.24	3.40E-01	1.00E+00	0.07	7.62E-01	9.69E-01
**PPFIBP2**	-0.10	6.14E-01	1.00E+00	0.10	7.62E-01	9.69E-01
**AL133243.2**	1.01	3.08E-01	1.00E+00	0.22	7.62E-01	9.69E-01
**TWF1**	-0.17	5.48E-01	1.00E+00	0.06	7.62E-01	9.69E-01
**PIP4K2C**	-0.15	4.73E-01	1.00E+00	0.06	7.62E-01	9.69E-01
**RPL15P3**	0.06	8.62E-01	1.00E+00	0.21	7.62E-01	9.69E-01
**G2582**	0.24	8.44E-01	1.00E+00	-0.24	7.62E-01	9.69E-01
**RPL7AP30**	-0.50	5.01E-01	1.00E+00	-0.13	7.63E-01	9.69E-01
**PDE4D**	-0.30	2.22E-01	1.00E+00	0.08	7.63E-01	9.69E-01
**VEPH1**	-0.40	2.30E-01	1.00E+00	0.15	7.63E-01	9.69E-01
**AC010733.1**	-0.02	9.86E-01	1.00E+00	-0.22	7.63E-01	9.69E-01
**GAPVD1**	0.00	9.94E-01	1.00E+00	-0.06	7.63E-01	9.69E-01
**C12orf56**	0.15	8.77E-01	1.00E+00	0.20	7.63E-01	9.69E-01
**SS18L2**	-0.06	8.53E-01	1.00E+00	-0.12	7.63E-01	9.69E-01
**ARHGEF34P**	0.59	2.52E-01	1.00E+00	0.18	7.63E-01	9.69E-01
**AL121985.1**	0.36	9.17E-01	1.00E+00	-0.42	7.63E-01	9.69E-01
**FNIP1**	0.17	4.71E-01	1.00E+00	0.06	7.63E-01	9.69E-01
**ANKH**	0.11	6.53E-01	1.00E+00	-0.11	7.63E-01	9.69E-01
**NUDT21**	0.04	8.49E-01	1.00E+00	-0.04	7.63E-01	9.69E-01
**TRBV11-2**	0.09	9.64E-01	1.00E+00	0.43	7.63E-01	9.69E-01
**TXLNGY**	0.44	5.25E-01	1.00E+00	-0.21	7.63E-01	9.69E-01
**ZMAT5**	0.18	4.69E-01	1.00E+00	0.08	7.63E-01	9.69E-01
**KIAA1755**	-0.29	5.51E-01	1.00E+00	0.94	7.63E-01	9.69E-01
**SERBP1P6**	-2.14	4.14E-01	1.00E+00	-0.18	7.63E-01	9.69E-01

**CCDC174**	0.12	5.30E-01	1.00E+00	-0.07	7.63E-01	9.69E-01
**ATG16L1**	0.25	2.16E-01	1.00E+00	0.06	7.63E-01	9.69E-01
**AC025171.5**	-0.62	7.66E-01	1.00E+00	-0.43	7.63E-01	9.69E-01
**NDUFV1**	-0.12	5.85E-01	1.00E+00	-0.04	7.63E-01	9.69E-01
**C1orf68**	0.57	3.51E-01	1.00E+00	0.94	7.64E-01	9.69E-01
**GALNT8**	1.18	8.32E-02	1.00E+00	-0.26	7.64E-01	9.69E-01
**FAM20B**	0.04	8.17E-01	1.00E+00	-0.12	7.64E-01	9.69E-01
**AL035681.1**	0.65	3.53E-01	1.00E+00	-0.27	7.64E-01	9.69E-01
**GLRX**	0.14	5.71E-01	1.00E+00	0.08	7.64E-01	9.69E-01
**TLR5**	0.37	4.41E-01	1.00E+00	0.09	7.64E-01	9.69E-01
**JAK2**	0.11	6.74E-01	1.00E+00	0.13	7.64E-01	9.69E-01
**SPTBN1**	0.07	8.33E-01	1.00E+00	0.12	7.64E-01	9.69E-01
**2-Mar**	0.89	1.42E-01	1.00E+00	-0.22	7.64E-01	9.69E-01
**MORN1**	-0.26	3.55E-01	1.00E+00	-0.12	7.64E-01	9.69E-01
**AL513534.1**	0.52	5.86E-01	1.00E+00	-0.21	7.64E-01	9.69E-01
**AC011294.1**	1.36	3.29E-01	1.00E+00	-0.96	7.64E-01	9.69E-01
**AC131212.3**	-0.57	4.67E-01	1.00E+00	0.95	7.64E-01	9.69E-01
**STYXL1**	-0.18	4.28E-01	1.00E+00	0.08	7.64E-01	9.69E-01
**AC004453.1**	-0.66	4.08E-01	1.00E+00	0.25	7.64E-01	9.69E-01
**AC018628.1**	0.15	8.79E-01	1.00E+00	-0.18	7.64E-01	9.69E-01
**G40536**	-0.85	2.71E-01	1.00E+00	-0.35	7.64E-01	9.69E-01
**KLC4**	-0.21	4.65E-01	1.00E+00	-0.08	7.64E-01	9.69E-01
**CNTFR**	1.56	6.96E-04	1.25E-01	0.22	7.64E-01	9.69E-01
**AC005224.1**	-1.82	2.12E-01	1.00E+00	0.30	7.64E-01	9.69E-01
**TIGD1**	-0.65	1.88E-01	1.00E+00	0.16	7.65E-01	9.69E-01
**DOCK11**	0.72	1.81E-01	1.00E+00	0.18	7.65E-01	9.69E-01
**C1orf56**	0.04	8.85E-01	1.00E+00	0.08	7.65E-01	9.69E-01
**DYNLL1**	0.09	7.82E-01	1.00E+00	0.09	7.65E-01	9.69E-01
**CLDN20**	1.15	4.25E-01	1.00E+00	0.37	7.65E-01	9.69E-01
**TRAPPC5**	-0.66	3.28E-01	1.00E+00	0.23	7.65E-01	9.69E-01
**AC007390.2**	-1.98	8.44E-02	1.00E+00	0.18	7.65E-01	9.69E-01
**CCDC74A**	0.18	5.73E-01	1.00E+00	0.09	7.65E-01	9.69E-01

**RRN3P3**	0.17	6.90E-01	1.00E+00	0.12	7.65E-01	9.69E-01
**CCDC144NL-AS1**	0.80	2.35E-01	1.00E+00	0.20	7.65E-01	9.69E-01
**SLC51B**	-0.25	6.54E-01	1.00E+00	0.28	7.65E-01	9.69E-01
**TBCC**	-0.09	6.73E-01	1.00E+00	0.10	7.65E-01	9.69E-01
**AC015922.2**	0.40	5.23E-01	1.00E+00	0.22	7.65E-01	9.69E-01
**OST4**	0.03	8.78E-01	1.00E+00	-0.14	7.65E-01	9.69E-01
**VPS13B**	-0.03	8.65E-01	1.00E+00	0.12	7.65E-01	9.69E-01
**LINC02574**	-1.64	3.63E-01	1.00E+00	0.37	7.65E-01	9.69E-01
**EEF1A2**	-0.55	1.42E-01	1.00E+00	-0.28	7.65E-01	9.69E-01
**KIF18B**	-0.02	9.48E-01	1.00E+00	0.22	7.65E-01	9.69E-01
**ZBTB20**	0.36	3.71E-01	1.00E+00	-0.13	7.65E-01	9.69E-01
**MSI2**	-0.19	4.82E-01	1.00E+00	0.13	7.65E-01	9.69E-01
**DQX1**	-0.37	3.95E-01	1.00E+00	-0.28	7.65E-01	9.69E-01
**SLX4IP**	0.26	4.26E-01	1.00E+00	-0.11	7.65E-01	9.69E-01
**SLC9A7P1**	0.84	4.18E-01	1.00E+00	-0.26	7.65E-01	9.69E-01
**RGS13**	0.61	3.95E-01	1.00E+00	-0.17	7.65E-01	9.69E-01
**RBM8A**	-0.05	7.96E-01	1.00E+00	-0.09	7.65E-01	9.69E-01
**KRTAP19-1**	-2.55	2.52E-01	1.00E+00	-0.96	7.65E-01	9.69E-01
**PARVA**	0.15	6.93E-01	1.00E+00	-0.11	7.66E-01	9.69E-01
**AC012313.1**	-0.26	3.26E-01	1.00E+00	0.27	7.66E-01	9.69E-01
**UBE2Q2P1**	0.20	6.98E-01	1.00E+00	-0.10	7.66E-01	9.69E-01
**GEMIN5**	0.21	3.43E-01	1.00E+00	0.09	7.66E-01	9.69E-01
**ZSCAN21**	-0.09	6.85E-01	1.00E+00	-0.07	7.66E-01	9.70E-01
**RPS3AP34**	0.03	9.77E-01	1.00E+00	0.25	7.66E-01	9.70E-01
**XLOC_001023**	-0.44	2.98E-01	1.00E+00	-0.12	7.66E-01	9.70E-01
**SLC5A2**	-1.22	4.58E-01	1.00E+00	0.28	7.66E-01	9.70E-01
**TEX9**	-0.32	4.70E-01	1.00E+00	-0.11	7.66E-01	9.70E-01
**TUBA4B**	1.09	3.58E-01	1.00E+00	0.28	7.66E-01	9.70E-01
**GSX2**	-0.32	7.65E-01	1.00E+00	-0.50	7.66E-01	9.70E-01
**PRDX4**	0.01	9.77E-01	1.00E+00	-0.09	7.66E-01	9.70E-01
**CEP131**	-0.27	3.34E-01	1.00E+00	-0.16	7.66E-01	9.70E-01
**FBXL6**	-0.56	5.45E-02	1.00E+00	-0.18	7.66E-01	9.70E-01

**ATAD3C**	-0.64	2.60E-01	1.00E+00	0.14	7.66E-01	9.70E-01
**SLC2A14**	-2.96	3.68E-02	1.00E+00	-0.94	7.66E-01	9.70E-01
**KIFC1**	-0.08	7.97E-01	1.00E+00	0.17	7.66E-01	9.70E-01
**SNRPEP4**	1.09	4.73E-01	1.00E+00	-0.17	7.66E-01	9.70E-01
**AL626787.1**	-2.64	1.04E-01	1.00E+00	0.33	7.66E-01	9.70E-01
**TMEM65**	-0.10	6.68E-01	1.00E+00	-0.12	7.66E-01	9.70E-01
**CCL14**	0.58	5.83E-01	1.00E+00	0.28	7.66E-01	9.70E-01
**C17orf53**	-0.09	8.03E-01	1.00E+00	0.13	7.66E-01	9.70E-01
**RTCB**	-0.10	7.25E-01	1.00E+00	0.06	7.67E-01	9.70E-01
**RPAIN**	0.27	3.32E-01	1.00E+00	-0.08	7.67E-01	9.70E-01
**LINC01117**	-0.89	4.81E-01	1.00E+00	0.18	7.67E-01	9.70E-01
**STARD5**	0.34	5.54E-01	1.00E+00	0.13	7.67E-01	9.70E-01
**KHNYN**	0.05	8.69E-01	1.00E+00	0.15	7.67E-01	9.70E-01
**EIF3E**	0.00	9.98E-01	1.00E+00	0.07	7.67E-01	9.70E-01
**PCBP4**	-0.11	5.86E-01	1.00E+00	-0.12	7.67E-01	9.70E-01
**PNCK**	-0.85	1.92E-01	1.00E+00	-0.26	7.67E-01	9.70E-01
**PNPO**	0.09	7.64E-01	1.00E+00	-0.08	7.67E-01	9.70E-01
**ABLIM1**	0.20	4.35E-01	1.00E+00	0.08	7.67E-01	9.70E-01
**FBXO22**	0.11	7.45E-01	1.00E+00	-0.09	7.67E-01	9.70E-01
**DLGAP4-AS1**	-1.00	2.63E-01	1.00E+00	0.31	7.67E-01	9.70E-01
**AC005840.1**	NA	NA	NA	0.39	7.67E-01	9.70E-01
**MFSD12**	-0.64	4.46E-02	1.00E+00	0.18	7.67E-01	9.70E-01
**UBA6**	0.01	9.70E-01	1.00E+00	0.07	7.67E-01	9.70E-01
**CSDC2**	-0.94	1.17E-01	1.00E+00	0.71	7.67E-01	9.70E-01
**DOK6**	-0.18	7.46E-01	1.00E+00	0.22	7.67E-01	9.70E-01
**AC087521.1**	-4.34	8.46E-05	2.17E-02	0.34	7.67E-01	9.70E-01
**SMTNL2**	0.72	3.11E-01	1.00E+00	0.83	7.67E-01	9.70E-01
**11-Sep**	0.52	2.66E-01	1.00E+00	-0.13	7.67E-01	9.70E-01
**MEOX1**	-0.07	8.95E-01	1.00E+00	-0.16	7.67E-01	9.70E-01
**ORM1**	NA	NA	NA	0.95	7.67E-01	9.70E-01
**GORASP2**	-0.13	4.90E-01	1.00E+00	-0.05	7.67E-01	9.70E-01
**AL157394.1**	0.82	2.23E-01	1.00E+00	-0.23	7.67E-01	9.70E-01

**ADD1**	0.07	7.82E-01	1.00E+00	0.12	7.67E-01	9.70E-01
**RIOK2**	0.05	8.26E-01	1.00E+00	-0.08	7.68E-01	9.70E-01
**SLC30A2**	-1.14	1.22E-01	1.00E+00	0.29	7.68E-01	9.70E-01
**APBB2**	0.31	3.29E-01	1.00E+00	0.10	7.68E-01	9.70E-01
**RPL21P28**	-0.27	5.70E-01	1.00E+00	-0.20	7.68E-01	9.70E-01
**SLC37A4**	-0.05	8.67E-01	1.00E+00	-0.07	7.68E-01	9.70E-01
**DBNDD1**	-0.04	8.96E-01	1.00E+00	-0.19	7.68E-01	9.70E-01
**TRIM4**	0.43	1.42E-02	7.51E-01	0.05	7.68E-01	9.70E-01
**RILPL1**	-0.32	1.20E-01	1.00E+00	0.18	7.68E-01	9.70E-01
**ERCC4**	0.20	4.18E-01	1.00E+00	0.07	7.68E-01	9.70E-01
**GALNT16**	0.48	3.12E-01	1.00E+00	0.18	7.68E-01	9.70E-01
**ORMDL3**	0.49	1.37E-01	1.00E+00	-0.09	7.68E-01	9.70E-01
**FAM117B**	-0.22	4.64E-01	1.00E+00	-0.11	7.68E-01	9.70E-01
**RALA**	0.24	2.01E-01	1.00E+00	0.05	7.68E-01	9.70E-01
**VEGFA**	-0.25	4.37E-01	1.00E+00	-0.23	7.68E-01	9.70E-01
**CCDC97**	-0.11	5.70E-01	1.00E+00	0.10	7.68E-01	9.70E-01
**ADAT2**	0.05	9.18E-01	1.00E+00	0.09	7.68E-01	9.70E-01
**RPL23AP65**	-1.60	3.93E-01	1.00E+00	0.30	7.68E-01	9.70E-01
**TFRC**	0.19	5.32E-01	1.00E+00	0.11	7.68E-01	9.70E-01
**TEDC2**	-0.99	1.82E-02	8.48E-01	-0.21	7.68E-01	9.70E-01
**AC007750.1**	-0.80	8.18E-01	1.00E+00	-0.74	7.68E-01	9.70E-01
**THSD4**	-0.03	9.40E-01	1.00E+00	-0.12	7.68E-01	9.70E-01
**DEF8**	-0.08	6.95E-01	1.00E+00	0.05	7.68E-01	9.70E-01
**FTCDNL1**	-0.07	8.60E-01	1.00E+00	0.12	7.69E-01	9.70E-01
**PROM2**	-0.30	3.96E-01	1.00E+00	0.18	7.69E-01	9.70E-01
**LINC00659**	-0.49	8.87E-01	1.00E+00	0.47	7.69E-01	9.70E-01
**DEPDC1B**	0.29	5.09E-01	1.00E+00	0.13	7.69E-01	9.70E-01
**PLXNB3**	-0.29	4.68E-01	1.00E+00	0.28	7.69E-01	9.70E-01
**ZNF550**	0.20	6.31E-01	1.00E+00	0.11	7.69E-01	9.70E-01
**RBM22**	-0.16	2.71E-01	1.00E+00	-0.04	7.69E-01	9.70E-01
**EHF**	0.16	6.35E-01	1.00E+00	0.13	7.69E-01	9.70E-01
**C16orf96**	-0.87	3.16E-01	1.00E+00	0.38	7.69E-01	9.70E-01

**TGS1**	-0.16	3.26E-01	1.00E+00	0.05	7.69E-01	9.70E-01
**UAP1**	0.48	1.93E-01	1.00E+00	0.13	7.69E-01	9.70E-01
**C17orf50**	-0.26	7.88E-01	1.00E+00	0.32	7.69E-01	9.70E-01
**SUMO2P1**	0.82	4.43E-01	1.00E+00	-0.12	7.69E-01	9.70E-01
**TMEM255A**	0.53	2.88E-01	1.00E+00	-0.13	7.69E-01	9.70E-01
**RHBDF1**	-0.05	8.36E-01	1.00E+00	0.14	7.69E-01	9.70E-01
**AL035252.3**	1.25	3.78E-01	1.00E+00	0.40	7.69E-01	9.70E-01
**XLOC_002198**	-0.98	2.87E-01	1.00E+00	0.20	7.69E-01	9.70E-01
**AC002553.1**	-0.42	7.09E-01	1.00E+00	-0.21	7.69E-01	9.70E-01
**MBNL2**	0.02	9.43E-01	1.00E+00	-0.08	7.69E-01	9.70E-01
**HBQ1**	-1.31	3.57E-01	1.00E+00	0.42	7.69E-01	9.70E-01
**PHC3**	-0.16	4.97E-01	1.00E+00	0.08	7.69E-01	9.70E-01
**LRRTM3**	-0.56	7.56E-01	1.00E+00	-0.46	7.69E-01	9.70E-01
**AL022328.4**	-0.04	9.47E-01	1.00E+00	0.34	7.69E-01	9.70E-01
**HIST3H2BB**	-2.33	1.53E-02	7.66E-01	-0.18	7.69E-01	9.70E-01
**CYP4F11**	0.14	7.49E-01	1.00E+00	0.24	7.70E-01	9.70E-01
**SRGAP2B**	-0.02	9.58E-01	1.00E+00	0.13	7.70E-01	9.70E-01
**AC005726.1**	-0.35	5.46E-01	1.00E+00	0.26	7.70E-01	9.70E-01
**HIST1H2AG**	-0.97	1.25E-01	1.00E+00	0.24	7.70E-01	9.70E-01
**RAB11FIP1P1**	0.18	9.06E-01	1.00E+00	-0.39	7.70E-01	9.70E-01
**DSE**	0.06	8.05E-01	1.00E+00	0.09	7.70E-01	9.70E-01
**G16985**	-3.68	2.30E-02	9.12E-01	-0.22	7.70E-01	9.70E-01
**MTHFD1**	0.17	4.14E-01	1.00E+00	-0.08	7.70E-01	9.70E-01
**RBFA**	0.09	7.46E-01	1.00E+00	-0.08	7.70E-01	9.70E-01
**E2F6**	0.17	4.29E-01	1.00E+00	-0.13	7.70E-01	9.70E-01
**BTLA**	-1.09	2.46E-01	1.00E+00	-0.29	7.70E-01	9.70E-01
**TET3**	-0.14	7.59E-01	1.00E+00	0.16	7.70E-01	9.70E-01
**AL589843.1**	-0.12	8.56E-01	1.00E+00	0.17	7.70E-01	9.70E-01
**PTGS1**	0.25	5.83E-01	1.00E+00	-0.12	7.70E-01	9.70E-01
**IGSF8**	-0.37	2.52E-01	1.00E+00	0.10	7.70E-01	9.70E-01
**OGN**	1.49	4.60E-02	1.00E+00	0.19	7.70E-01	9.70E-01
**LINC01426**	1.57	2.54E-01	1.00E+00	0.27	7.70E-01	9.70E-01

**CYP26B1**	0.41	1.81E-01	1.00E+00	0.16	7.70E-01	9.70E-01
**ZNF672**	-0.29	2.12E-01	1.00E+00	0.14	7.70E-01	9.70E-01
**RBM11**	-0.73	3.49E-01	1.00E+00	-0.21	7.70E-01	9.70E-01
**CBL**	0.17	5.01E-01	1.00E+00	0.09	7.70E-01	9.70E-01
**OR1L8**	-2.13	1.63E-01	1.00E+00	-0.33	7.70E-01	9.70E-01
**BTBD16**	0.11	8.33E-01	1.00E+00	-0.22	7.70E-01	9.70E-01
**PDF**	-0.89	3.44E-02	1.00E+00	0.20	7.70E-01	9.70E-01
**AC130686.1**	-0.75	5.77E-01	1.00E+00	-0.45	7.70E-01	9.70E-01
**PTGIS**	0.47	3.37E-01	1.00E+00	0.18	7.70E-01	9.70E-01
**LINC02009**	1.92	4.39E-01	1.00E+00	0.35	7.71E-01	9.70E-01
**ALG1L2**	-0.76	3.42E-01	1.00E+00	-0.19	7.71E-01	9.70E-01
**TM4SF4**	-3.45	1.98E-02	8.80E-01	-0.43	7.71E-01	9.70E-01
**CACNA1I**	-1.85	3.55E-02	1.00E+00	0.92	7.71E-01	9.70E-01
**AC104837.1**	NA	NA	NA	0.21	7.71E-01	9.70E-01
**XLOC_001935**	-3.75	1.08E-02	6.42E-01	-0.32	7.71E-01	9.70E-01
**CILP**	1.22	1.37E-01	1.00E+00	-0.20	7.71E-01	9.70E-01
**AC092614.1**	1.31	1.16E-01	1.00E+00	0.27	7.71E-01	9.70E-01
**TMEM41A**	0.23	3.04E-01	1.00E+00	-0.06	7.71E-01	9.70E-01
**AC006273.1**	-0.75	6.20E-01	1.00E+00	-0.27	7.71E-01	9.70E-01
**GPRASP1**	0.27	5.22E-01	1.00E+00	0.19	7.71E-01	9.70E-01
**EXO1**	-0.32	3.33E-01	1.00E+00	-0.15	7.71E-01	9.70E-01
**DPAGT1**	-0.02	9.41E-01	1.00E+00	-0.07	7.71E-01	9.70E-01
**MPPED2**	0.39	5.41E-01	1.00E+00	-0.21	7.71E-01	9.70E-01
**NOL10**	-0.06	7.39E-01	1.00E+00	-0.07	7.71E-01	9.70E-01
**AC013476.1**	-1.58	6.01E-01	1.00E+00	0.24	7.71E-01	9.70E-01
**C19orf18**	0.30	7.76E-01	1.00E+00	0.18	7.71E-01	9.70E-01
**UBE2G2**	-0.18	2.72E-01	1.00E+00	0.05	7.71E-01	9.70E-01
**U47924.2**	0.56	8.71E-01	1.00E+00	-0.27	7.71E-01	9.70E-01
**ACA64**	-2.28	1.78E-01	1.00E+00	0.34	7.71E-01	9.70E-01
**SLC30A9**	0.06	6.86E-01	1.00E+00	-0.05	7.71E-01	9.70E-01
**TLR10**	-0.37	7.46E-01	1.00E+00	0.25	7.72E-01	9.70E-01
**G25251**	0.25	8.65E-01	1.00E+00	0.45	7.72E-01	9.70E-01

**THAP7-AS1**	-0.20	6.40E-01	1.00E+00	0.13	7.72E-01	9.70E-01
**ARHGAP24**	-0.34	1.88E-01	1.00E+00	-0.09	7.72E-01	9.70E-01
**CSTF2**	0.21	4.87E-01	1.00E+00	0.08	7.72E-01	9.70E-01
**EMB**	-0.12	8.34E-01	1.00E+00	-0.11	7.72E-01	9.70E-01
**NOC2L**	-0.27	3.11E-01	1.00E+00	0.08	7.72E-01	9.70E-01
**AC138207.3**	-0.06	9.54E-01	1.00E+00	-0.20	7.72E-01	9.70E-01
**ADGRF5**	0.55	2.91E-01	1.00E+00	0.17	7.72E-01	9.70E-01
**G33852**	0.40	5.51E-01	1.00E+00	0.21	7.72E-01	9.70E-01
**GALNT14**	-0.60	1.46E-01	1.00E+00	0.18	7.72E-01	9.70E-01
**FZD5**	0.05	9.23E-01	1.00E+00	0.17	7.72E-01	9.70E-01
**NOTCH3**	-0.12	5.99E-01	1.00E+00	0.18	7.72E-01	9.70E-01
**SPAG6**	0.73	5.57E-01	1.00E+00	-0.23	7.72E-01	9.70E-01
**AL161787.1**	-0.54	8.75E-01	1.00E+00	-0.22	7.72E-01	9.70E-01
**DNAJC4**	-0.11	6.97E-01	1.00E+00	0.06	7.72E-01	9.70E-01
**KRT2**	0.18	7.96E-01	1.00E+00	0.90	7.72E-01	9.70E-01
**YPEL2**	0.50	2.82E-02	9.75E-01	0.07	7.72E-01	9.70E-01
**SGO1**	-0.83	1.90E-01	1.00E+00	-0.18	7.72E-01	9.70E-01
**AF186192.2**	0.48	4.00E-01	1.00E+00	0.21	7.72E-01	9.70E-01
**XRCC5**	-0.10	5.83E-01	1.00E+00	-0.04	7.73E-01	9.70E-01
**PPRC1**	-0.47	1.14E-01	1.00E+00	-0.13	7.73E-01	9.70E-01
**AC142472.1**	0.13	8.41E-01	1.00E+00	-0.15	7.73E-01	9.70E-01
**PRR13P5**	-0.12	8.52E-01	1.00E+00	-0.13	7.73E-01	9.70E-01
**C6orf15**	-0.86	1.93E-01	1.00E+00	0.18	7.73E-01	9.70E-01
**AL590867.2**	0.02	9.57E-01	1.00E+00	0.23	7.73E-01	9.70E-01
**FXYD3**	-0.04	9.50E-01	1.00E+00	0.15	7.73E-01	9.70E-01
**AC007780.1**	-0.86	7.04E-01	1.00E+00	0.25	7.73E-01	9.70E-01
**ARFGEF1**	0.27	2.58E-01	1.00E+00	0.12	7.73E-01	9.70E-01
**FHOD3**	-0.19	6.82E-01	1.00E+00	0.14	7.73E-01	9.70E-01
**ZNF74**	-0.17	5.27E-01	1.00E+00	-0.15	7.73E-01	9.70E-01
**CLK2**	-0.08	7.27E-01	1.00E+00	-0.16	7.73E-01	9.70E-01
**HNRNPCP7**	1.10	1.79E-01	1.00E+00	-0.27	7.73E-01	9.70E-01
**AC139713.2**	-0.85	4.29E-01	1.00E+00	-0.18	7.73E-01	9.70E-01

**ZNF790**	0.13	6.80E-01	1.00E+00	-0.09	7.73E-01	9.70E-01
**CEBPB**	-0.17	6.27E-01	1.00E+00	0.24	7.73E-01	9.70E-01
**DDHD1**	-0.04	8.90E-01	1.00E+00	0.09	7.73E-01	9.70E-01
**RALGAPB**	0.24	2.29E-01	1.00E+00	0.10	7.73E-01	9.70E-01
**PDZD2**	0.18	5.01E-01	1.00E+00	-0.07	7.73E-01	9.70E-01
**ANKFN1**	-0.06	8.88E-01	1.00E+00	-0.20	7.73E-01	9.70E-01
**SUGP2**	-0.25	3.25E-01	1.00E+00	0.15	7.73E-01	9.70E-01
**GCNA**	-1.71	3.48E-01	1.00E+00	0.41	7.73E-01	9.70E-01
**LIFR-AS1**	-0.09	8.31E-01	1.00E+00	-0.12	7.73E-01	9.70E-01
**CYP2J2**	-0.14	7.69E-01	1.00E+00	-0.20	7.73E-01	9.70E-01
**AC009283.1**	-0.59	5.11E-01	1.00E+00	-0.32	7.74E-01	9.70E-01
**AC064801.1**	-2.76	1.22E-01	1.00E+00	0.25	7.74E-01	9.70E-01
**PLEKHA8**	0.43	2.35E-01	1.00E+00	-0.15	7.74E-01	9.70E-01
**AC098614.1**	0.61	2.50E-01	1.00E+00	-0.10	7.74E-01	9.70E-01
**HSPA8**	-0.35	1.37E-01	1.00E+00	0.10	7.74E-01	9.70E-01
**ADCYAP1R1**	0.53	3.72E-01	1.00E+00	0.22	7.74E-01	9.70E-01
**LMOD1**	0.00	9.94E-01	1.00E+00	-0.15	7.74E-01	9.70E-01
**AC093311.1**	-2.01	3.08E-01	1.00E+00	-0.92	7.74E-01	9.70E-01
**FAM220A**	-0.03	8.95E-01	1.00E+00	0.07	7.74E-01	9.70E-01
**RPS27A**	-0.08	7.92E-01	1.00E+00	-0.07	7.74E-01	9.71E-01
**RARG**	-0.08	8.19E-01	1.00E+00	-0.12	7.74E-01	9.71E-01
**HMG20B**	-0.34	7.59E-02	1.00E+00	-0.08	7.74E-01	9.71E-01
**BRD3**	-0.18	4.13E-01	1.00E+00	0.06	7.74E-01	9.71E-01
**PRKCI**	0.23	4.40E-01	1.00E+00	-0.09	7.74E-01	9.71E-01
**APOB**	1.58	5.47E-02	1.00E+00	-0.21	7.74E-01	9.71E-01
**WDCP**	-0.01	9.67E-01	1.00E+00	-0.16	7.74E-01	9.71E-01
**MLPH**	0.02	9.77E-01	1.00E+00	0.10	7.74E-01	9.71E-01
**RFX4**	-1.82	5.12E-01	1.00E+00	0.57	7.74E-01	9.71E-01
**NTRK1**	0.10	9.38E-01	1.00E+00	0.91	7.74E-01	9.71E-01
**LINC01128**	0.02	9.45E-01	1.00E+00	-0.09	7.74E-01	9.71E-01
**G10252**	-0.86	2.52E-01	1.00E+00	-0.20	7.74E-01	9.71E-01
**TENM1**	-0.69	2.52E-01	1.00E+00	-0.16	7.75E-01	9.71E-01

**FRS3**	-0.38	2.22E-01	1.00E+00	0.24	7.75E-01	9.71E-01
**RPL12P38**	1.78	2.78E-01	1.00E+00	-0.14	7.75E-01	9.71E-01
**DMWD**	-0.44	1.34E-01	1.00E+00	0.17	7.75E-01	9.71E-01
**ABCC8**	-3.90	2.69E-03	2.68E-01	-0.37	7.75E-01	9.71E-01
**MTFR1**	-0.04	8.66E-01	1.00E+00	0.05	7.75E-01	9.71E-01
**CPT2**	-0.12	6.57E-01	1.00E+00	0.07	7.75E-01	9.71E-01
**SMC2-AS1**	0.24	8.93E-01	1.00E+00	0.44	7.75E-01	9.71E-01
**TAF10**	0.00	9.90E-01	1.00E+00	-0.22	7.75E-01	9.71E-01
**RPS2P5**	-0.67	5.21E-02	1.00E+00	-0.20	7.75E-01	9.71E-01
**HEATR3**	0.01	9.81E-01	1.00E+00	0.16	7.75E-01	9.71E-01
**AL359182.2**	-0.57	7.05E-01	1.00E+00	0.34	7.75E-01	9.71E-01
**SLC44A2**	0.07	6.91E-01	1.00E+00	-0.09	7.75E-01	9.71E-01
**AL603839.2**	0.41	6.90E-01	1.00E+00	0.27	7.75E-01	9.71E-01
**RN7SL382P**	-2.57	4.51E-01	1.00E+00	0.36	7.75E-01	9.71E-01
**AC092611.2**	0.48	2.14E-01	1.00E+00	0.19	7.75E-01	9.71E-01
**LINC02028**	2.62	2.18E-02	8.95E-01	0.24	7.75E-01	9.71E-01
**GCLC**	-0.24	3.37E-01	1.00E+00	-0.09	7.75E-01	9.71E-01
**EEF1B2P1**	-1.19	7.32E-01	1.00E+00	-0.23	7.75E-01	9.71E-01
**EEF1B2P2**	-0.80	8.18E-01	1.00E+00	0.19	7.75E-01	9.71E-01
**DDX23**	-0.06	6.98E-01	1.00E+00	0.10	7.75E-01	9.71E-01
**NEPRO**	0.06	7.47E-01	1.00E+00	0.05	7.75E-01	9.71E-01
**PHIP**	0.11	5.73E-01	1.00E+00	-0.05	7.75E-01	9.71E-01
**G10667**	0.48	7.73E-01	1.00E+00	0.42	7.75E-01	9.71E-01
**AC092835.1**	0.85	1.53E-01	1.00E+00	0.25	7.75E-01	9.71E-01
**AC092279.1**	0.00	9.95E-01	1.00E+00	-0.22	7.75E-01	9.71E-01
**BRWD3**	0.34	4.53E-01	1.00E+00	-0.12	7.75E-01	9.71E-01
**ANXA13**	-0.20	8.58E-01	1.00E+00	0.33	7.75E-01	9.71E-01
**POLR3B**	0.02	9.55E-01	1.00E+00	-0.06	7.76E-01	9.71E-01
**LINC01703**	-0.44	2.94E-01	1.00E+00	-0.16	7.76E-01	9.71E-01
**DISP2**	-2.66	1.55E-03	2.18E-01	-0.29	7.76E-01	9.71E-01
**XLOC_010305**	2.05	1.36E-01	1.00E+00	0.31	7.76E-01	9.71E-01
**SPTLC3**	0.26	6.11E-01	1.00E+00	0.10	7.76E-01	9.71E-01

**RTEL1**	1.08	4.48E-01	1.00E+00	-0.22	7.76E-01	9.71E-01
**RPS19BP1**	0.13	5.04E-01	1.00E+00	-0.05	7.76E-01	9.71E-01
**POU5F1**	-0.32	4.77E-01	1.00E+00	-0.22	7.76E-01	9.71E-01
**GUCY1B1**	0.17	7.28E-01	1.00E+00	-0.10	7.76E-01	9.71E-01
**MLX**	0.03	8.73E-01	1.00E+00	0.05	7.76E-01	9.71E-01
**AL031665.2**	-2.68	5.46E-02	1.00E+00	0.16	7.76E-01	9.71E-01
**LDLR**	-0.85	3.32E-02	1.00E+00	0.13	7.76E-01	9.71E-01
**EMCN**	0.31	5.17E-01	1.00E+00	-0.15	7.76E-01	9.71E-01
**MRFAP1L1**	0.19	2.31E-01	1.00E+00	-0.03	7.76E-01	9.71E-01
**MXRA5Y**	0.16	9.07E-01	1.00E+00	-0.21	7.76E-01	9.71E-01
**IL21R**	-0.18	8.22E-01	1.00E+00	0.89	7.76E-01	9.71E-01
**C4B**	0.22	8.08E-01	1.00E+00	0.89	7.76E-01	9.71E-01
**POLR2H**	0.04	8.50E-01	1.00E+00	0.06	7.77E-01	9.71E-01
**AL023284.4**	0.36	4.73E-01	1.00E+00	-0.15	7.77E-01	9.71E-01
**SNORA25**	-1.65	4.96E-01	1.00E+00	-0.31	7.77E-01	9.71E-01
**ST8SIA6**	-0.96	4.93E-02	1.00E+00	0.20	7.77E-01	9.71E-01
**NR1I3**	0.22	6.57E-01	1.00E+00	0.22	7.77E-01	9.71E-01
**MUM1**	-0.05	8.13E-01	1.00E+00	-0.09	7.77E-01	9.71E-01
**AC084782.3**	-3.19	2.31E-01	1.00E+00	-0.29	7.77E-01	9.71E-01
**CC2D1A**	-0.28	2.43E-01	1.00E+00	-0.15	7.77E-01	9.71E-01
**DUSP7**	-0.27	4.59E-01	1.00E+00	0.12	7.77E-01	9.71E-01
**FAM3C2**	-0.02	9.57E-01	1.00E+00	-0.16	7.77E-01	9.71E-01
**ZNF814**	-0.64	1.40E-01	1.00E+00	-0.11	7.77E-01	9.71E-01
**PKN3**	0.11	7.93E-01	1.00E+00	0.19	7.77E-01	9.71E-01
**XLOC_004478**	0.58	6.27E-01	1.00E+00	-0.33	7.77E-01	9.71E-01
**HHAT**	-0.23	5.36E-01	1.00E+00	0.08	7.77E-01	9.71E-01
**HOXC4**	-0.39	2.33E-01	1.00E+00	-0.14	7.77E-01	9.71E-01
**DBN1**	-0.03	9.62E-01	1.00E+00	0.16	7.77E-01	9.71E-01
**AP002990.1**	-0.69	4.53E-02	1.00E+00	0.09	7.77E-01	9.71E-01
**GSTCD**	0.31	2.36E-01	1.00E+00	0.07	7.77E-01	9.71E-01
**AC009090.3**	-1.82	4.97E-01	1.00E+00	0.25	7.77E-01	9.71E-01
**ABHD15**	0.36	3.35E-01	1.00E+00	-0.15	7.77E-01	9.71E-01

**LCE2C**	0.92	1.97E-01	1.00E+00	0.22	7.77E-01	9.71E-01
**ANKRD24**	-1.04	6.92E-02	1.00E+00	0.33	7.77E-01	9.71E-01
**GFRA2**	-0.20	6.36E-01	1.00E+00	0.16	7.77E-01	9.71E-01
**STK24-AS1**	-0.20	8.21E-01	1.00E+00	0.33	7.77E-01	9.71E-01
**XLOC_008811**	7.46	7.73E-03	5.39E-01	-0.25	7.77E-01	9.71E-01
**BEST2**	1.10	3.59E-02	1.00E+00	-0.26	7.77E-01	9.71E-01
**CCDC77**	0.26	3.22E-01	1.00E+00	-0.14	7.77E-01	9.71E-01
**SAMD8**	0.10	6.76E-01	1.00E+00	-0.08	7.77E-01	9.71E-01
**KCNN2**	-2.49	5.98E-03	4.59E-01	0.15	7.77E-01	9.71E-01
**CDC42EP3**	0.00	9.92E-01	1.00E+00	-0.11	7.77E-01	9.71E-01
**PHRF1**	-0.31	2.26E-01	1.00E+00	0.22	7.77E-01	9.71E-01
**RNF169**	-0.20	4.65E-01	1.00E+00	0.16	7.78E-01	9.71E-01
**LCA5**	0.30	4.81E-01	1.00E+00	-0.11	7.78E-01	9.71E-01
**KRTAP8-1**	-1.59	6.38E-01	1.00E+00	-0.91	7.78E-01	9.71E-01
**PAMR1**	0.15	7.12E-01	1.00E+00	-0.10	7.78E-01	9.71E-01
**AL161636.1**	0.20	9.01E-01	1.00E+00	0.37	7.78E-01	9.71E-01
**SPARCL1**	0.18	7.04E-01	1.00E+00	0.14	7.78E-01	9.71E-01
**RPRD1A**	0.16	4.74E-01	1.00E+00	-0.05	7.78E-01	9.71E-01
**NGFR**	-0.32	4.38E-01	1.00E+00	0.19	7.78E-01	9.71E-01
**HIRA**	-0.21	5.41E-01	1.00E+00	0.14	7.78E-01	9.71E-01
**AL136309.2**	1.05	3.99E-01	1.00E+00	0.45	7.78E-01	9.71E-01
**AL355304.1**	0.37	9.14E-01	1.00E+00	-0.28	7.78E-01	9.71E-01
**PHF24**	-1.08	4.15E-02	1.00E+00	-0.21	7.78E-01	9.71E-01
**RPS27P29**	-1.60	6.41E-01	1.00E+00	0.28	7.78E-01	9.71E-01
**G18874**	1.50	1.24E-01	1.00E+00	-0.22	7.78E-01	9.71E-01
**G28331**	0.35	7.04E-01	1.00E+00	-0.17	7.78E-01	9.71E-01
**NAA80**	-0.26	3.60E-01	1.00E+00	-0.07	7.78E-01	9.71E-01
**FAU**	-0.10	6.86E-01	1.00E+00	0.07	7.78E-01	9.71E-01
**HCN1**	-2.00	1.60E-02	7.92E-01	-0.34	7.78E-01	9.71E-01
**NT5DC2**	-0.17	6.52E-01	1.00E+00	0.09	7.78E-01	9.71E-01
**CISH**	-0.89	4.05E-03	3.54E-01	-0.13	7.78E-01	9.71E-01
**FAM84B**	0.18	6.25E-01	1.00E+00	-0.09	7.78E-01	9.71E-01

**SERPINF2**	-0.41	2.09E-01	1.00E+00	0.12	7.79E-01	9.71E-01
**RPS4XP16**	-0.63	5.31E-01	1.00E+00	-0.23	7.79E-01	9.71E-01
**RN7SL751P**	-1.97	5.67E-01	1.00E+00	-0.30	7.79E-01	9.71E-01
**RABGAP1**	0.32	3.32E-02	1.00E+00	-0.12	7.79E-01	9.71E-01
**SPC24**	-0.34	4.38E-01	1.00E+00	0.12	7.79E-01	9.71E-01
**NUSAP1**	-2.27	3.94E-02	1.00E+00	0.19	7.79E-01	9.71E-01
**SENP3**	-0.02	9.54E-01	1.00E+00	0.23	7.79E-01	9.71E-01
**G24400**	-0.48	6.19E-01	1.00E+00	0.21	7.79E-01	9.71E-01
**KRT39**	-4.67	4.75E-02	1.00E+00	0.88	7.79E-01	9.71E-01
**GALNT3**	0.23	5.38E-01	1.00E+00	0.11	7.79E-01	9.71E-01
**ADD2**	-1.09	4.24E-02	1.00E+00	-0.19	7.79E-01	9.71E-01
**CYB561A3**	-0.12	6.40E-01	1.00E+00	0.07	7.79E-01	9.71E-01
**ZNF704**	0.20	5.39E-01	1.00E+00	0.11	7.79E-01	9.71E-01
**BMX**	0.29	6.65E-01	1.00E+00	-0.17	7.79E-01	9.71E-01
**KRTAP19-5**	-1.75	5.98E-01	1.00E+00	-0.90	7.79E-01	9.71E-01
**PDXK**	0.75	1.19E-01	1.00E+00	-0.15	7.80E-01	9.71E-01
**ATP5PBP5**	-0.06	9.23E-01	1.00E+00	0.35	7.80E-01	9.71E-01
**SLC6A2**	0.53	3.40E-01	1.00E+00	0.13	7.80E-01	9.71E-01
**SPAAR**	1.85	1.77E-02	8.36E-01	0.63	7.80E-01	9.71E-01
**NCAPH**	0.03	9.49E-01	1.00E+00	0.18	7.80E-01	9.71E-01
**CYP4F22**	-0.05	9.23E-01	1.00E+00	0.16	7.80E-01	9.71E-01
**AC009779.2**	-0.32	5.21E-01	1.00E+00	0.18	7.80E-01	9.71E-01
**PSMD7**	-0.04	8.62E-01	1.00E+00	-0.08	7.80E-01	9.71E-01
**ANP32E**	0.07	7.66E-01	1.00E+00	-0.09	7.80E-01	9.71E-01
**C3orf33**	-1.44	1.99E-01	1.00E+00	-0.17	7.80E-01	9.71E-01
**ANXA8L2**	-0.59	1.72E-01	1.00E+00	0.16	7.80E-01	9.71E-01
**SMYD5**	-0.26	3.62E-01	1.00E+00	0.07	7.80E-01	9.71E-01
**UBXN4**	0.00	9.95E-01	1.00E+00	-0.04	7.80E-01	9.71E-01
**CHMP6**	-0.35	2.26E-01	1.00E+00	0.05	7.80E-01	9.71E-01
**UBOX5**	-0.31	2.20E-01	1.00E+00	-0.07	7.80E-01	9.71E-01
**IL34**	0.10	8.10E-01	1.00E+00	0.12	7.80E-01	9.71E-01
**AC114947.2**	-0.14	8.90E-01	1.00E+00	-0.23	7.80E-01	9.71E-01

**XLOC_013697**	0.84	1.56E-01	1.00E+00	-0.27	7.80E-01	9.71E-01
**SLC4A4**	-0.19	8.26E-01	1.00E+00	-0.25	7.80E-01	9.71E-01
**AC022973.4**	-0.42	8.31E-01	1.00E+00	0.36	7.80E-01	9.71E-01
**AL590139.1**	-0.20	8.73E-01	1.00E+00	-0.17	7.80E-01	9.71E-01
**AJ011932.1**	0.80	4.74E-01	1.00E+00	0.23	7.80E-01	9.71E-01
**G26757**	-0.13	8.90E-01	1.00E+00	-0.20	7.81E-01	9.71E-01
**AC022558.1**	1.33	6.09E-01	1.00E+00	-0.35	7.81E-01	9.71E-01
**XLOC_001934**	-1.02	6.57E-01	1.00E+00	0.31	7.81E-01	9.71E-01
**AL662844.4**	0.53	3.70E-01	1.00E+00	-0.10	7.81E-01	9.71E-01
**NFE2L3**	-0.05	9.14E-01	1.00E+00	0.15	7.81E-01	9.71E-01
**ACADVL**	0.35	1.09E-01	1.00E+00	-0.09	7.81E-01	9.71E-01
**CYP4F2**	0.03	9.86E-01	1.00E+00	-0.88	7.81E-01	9.71E-01
**ETHE1**	-0.26	4.71E-01	1.00E+00	-0.06	7.81E-01	9.71E-01
**CCDC178**	-0.97	4.62E-01	1.00E+00	-0.66	7.81E-01	9.71E-01
**ACLY**	-0.06	8.44E-01	1.00E+00	-0.11	7.81E-01	9.71E-01
**ADSS**	0.03	8.87E-01	1.00E+00	-0.05	7.81E-01	9.71E-01
**PRORSD1P**	-0.32	6.33E-01	1.00E+00	-0.21	7.81E-01	9.71E-01
**UBAC2**	0.36	4.14E-02	1.00E+00	0.03	7.81E-01	9.71E-01
**IGKJ3**	-0.48	8.90E-01	1.00E+00	0.74	7.81E-01	9.71E-01
**RUSC1-AS1**	-0.92	3.38E-01	1.00E+00	0.22	7.81E-01	9.71E-01
**XLOC_008141**	0.23	8.77E-01	1.00E+00	0.39	7.81E-01	9.71E-01
**CBR3**	-0.51	2.99E-01	1.00E+00	-0.15	7.81E-01	9.71E-01
**CERS2**	0.02	9.56E-01	1.00E+00	0.08	7.81E-01	9.71E-01
**SYT14**	-4.92	1.09E-03	1.72E-01	0.28	7.81E-01	9.71E-01
**WDR33**	-0.33	1.87E-01	1.00E+00	-0.05	7.81E-01	9.71E-01
**KNL1**	-0.56	1.47E-01	1.00E+00	-0.18	7.82E-01	9.71E-01
**ZNF321P**	-0.99	1.79E-01	1.00E+00	0.15	7.82E-01	9.71E-01
**ITPR3**	-0.15	7.31E-01	1.00E+00	-0.16	7.82E-01	9.71E-01
**LARGE2**	-0.50	3.11E-02	1.00E+00	-0.17	7.82E-01	9.71E-01
**G5265**	-2.18	5.60E-02	1.00E+00	-0.33	7.82E-01	9.71E-01
**ERLEC1**	0.16	4.09E-01	1.00E+00	0.05	7.82E-01	9.71E-01
**AC092868.2**	0.32	9.26E-01	1.00E+00	-0.32	7.82E-01	9.71E-01

**Z99289.1**	1.54	5.30E-01	1.00E+00	-0.37	7.82E-01	9.71E-01
**CDK2AP2**	-0.40	1.93E-01	1.00E+00	-0.07	7.82E-01	9.71E-01
**ARNTL**	1.01	8.16E-03	5.56E-01	0.09	7.82E-01	9.71E-01
**RPS7P1**	-0.46	2.32E-01	1.00E+00	-0.15	7.82E-01	9.71E-01
**PSPC1P1**	-2.11	3.56E-01	1.00E+00	-0.22	7.82E-01	9.71E-01
**AP003119.1**	-1.28	7.10E-01	1.00E+00	0.28	7.82E-01	9.71E-01
**MAATS1**	0.06	9.55E-01	1.00E+00	0.22	7.82E-01	9.71E-01
**C9orf78**	0.09	6.37E-01	1.00E+00	-0.06	7.82E-01	9.71E-01
**SENP7**	0.33	3.35E-01	1.00E+00	-0.06	7.82E-01	9.72E-01
**C11orf68**	0.01	9.69E-01	1.00E+00	0.15	7.82E-01	9.72E-01
**FAM83C**	-0.32	5.53E-01	1.00E+00	-0.19	7.82E-01	9.72E-01
**GATA3-AS1**	-0.94	3.10E-01	1.00E+00	0.25	7.82E-01	9.72E-01
**AC007881.3**	1.92	4.92E-01	1.00E+00	-0.85	7.82E-01	9.72E-01
**NUDCD3**	0.02	8.55E-01	1.00E+00	0.06	7.82E-01	9.72E-01
**CMYA5**	-0.22	6.10E-01	1.00E+00	-0.14	7.82E-01	9.72E-01
**XLOC_013022**	4.66	3.87E-03	3.41E-01	0.87	7.82E-01	9.72E-01
**AL022322.1**	-1.32	4.51E-01	1.00E+00	0.72	7.82E-01	9.72E-01
**SYPL2**	1.61	6.41E-02	1.00E+00	0.23	7.82E-01	9.72E-01
**SMKR1**	0.15	8.01E-01	1.00E+00	-0.23	7.82E-01	9.72E-01
**EXOC1**	0.05	8.29E-01	1.00E+00	-0.05	7.82E-01	9.72E-01
**NUP93**	-0.33	4.98E-02	1.00E+00	-0.04	7.82E-01	9.72E-01
**GPR84**	-1.63	2.05E-01	1.00E+00	0.87	7.82E-01	9.72E-01
**XLOC_008114**	-1.27	5.47E-01	1.00E+00	0.34	7.82E-01	9.72E-01
**AC022150.2**	0.80	1.33E-01	1.00E+00	0.29	7.83E-01	9.72E-01
**GPAA1**	0.01	9.82E-01	1.00E+00	0.06	7.83E-01	9.72E-01
**AGPAT1**	0.22	4.87E-01	1.00E+00	0.09	7.83E-01	9.72E-01
**AC006213.4**	-0.10	9.16E-01	1.00E+00	-0.30	7.83E-01	9.72E-01
**AL445250.1**	-0.56	8.26E-01	1.00E+00	-0.27	7.83E-01	9.72E-01
**C18orf32**	0.42	3.26E-01	1.00E+00	0.14	7.83E-01	9.72E-01
**HSPB2**	-1.32	6.38E-01	1.00E+00	0.19	7.83E-01	9.72E-01
**CHRNA3**	-2.74	4.43E-02	1.00E+00	0.30	7.83E-01	9.72E-01
**KRTAP10-11**	-13.54	7.77E-05	2.01E-02	-0.88	7.83E-01	9.72E-01

**ELOVL2**	0.14	7.84E-01	1.00E+00	-0.26	7.83E-01	9.72E-01
**G36295**	-3.38	5.17E-02	1.00E+00	0.27	7.83E-01	9.72E-01
**INPP5E**	-0.21	3.60E-01	1.00E+00	0.17	7.83E-01	9.72E-01
**AC111152.3**	1.16	7.39E-01	1.00E+00	0.26	7.83E-01	9.72E-01
**DBH-AS1**	-2.86	6.74E-02	1.00E+00	0.49	7.83E-01	9.72E-01
**GLIS2-AS1**	-2.27	1.09E-01	1.00E+00	-0.19	7.83E-01	9.72E-01
**HPDL**	-0.08	8.72E-01	1.00E+00	0.19	7.83E-01	9.72E-01
**RASAL2-AS1**	-0.72	2.39E-01	1.00E+00	-0.21	7.83E-01	9.72E-01
**HMSD**	0.69	4.01E-01	1.00E+00	-0.18	7.83E-01	9.72E-01
**AHR**	-0.08	7.36E-01	1.00E+00	0.09	7.83E-01	9.72E-01
**TMEM109**	0.10	6.03E-01	1.00E+00	0.05	7.83E-01	9.72E-01
**GTF2H3**	0.10	6.36E-01	1.00E+00	-0.04	7.83E-01	9.72E-01
**RNPC3**	0.11	6.79E-01	1.00E+00	-0.08	7.84E-01	9.72E-01
**PXK**	0.17	4.99E-01	1.00E+00	-0.08	7.84E-01	9.72E-01
**SLITRK2**	0.02	9.73E-01	1.00E+00	0.12	7.84E-01	9.72E-01
**C1orf174**	-0.28	4.26E-01	1.00E+00	-0.06	7.84E-01	9.72E-01
**TTC22**	0.01	9.86E-01	1.00E+00	-0.19	7.84E-01	9.72E-01
**NUB1**	-0.15	3.60E-01	1.00E+00	0.06	7.84E-01	9.72E-01
**RPL3P4**	0.24	7.59E-01	1.00E+00	0.18	7.84E-01	9.72E-01
**FANCG**	0.07	7.59E-01	1.00E+00	-0.09	7.84E-01	9.72E-01
**ATG2B**	0.19	5.39E-01	1.00E+00	-0.08	7.84E-01	9.72E-01
**NDUFAF5**	0.20	3.51E-01	1.00E+00	-0.07	7.84E-01	9.72E-01
**PTMAP5**	-0.05	8.78E-01	1.00E+00	-0.14	7.84E-01	9.72E-01
**AC105020.6**	0.08	9.32E-01	1.00E+00	0.23	7.84E-01	9.72E-01
**TMEM155**	-15.09	3.64E-09	1.90E-06	-0.87	7.84E-01	9.72E-01
**GADD45B**	-1.52	7.07E-03	5.12E-01	-0.17	7.84E-01	9.72E-01
**MAP3K5**	0.26	1.93E-01	1.00E+00	0.12	7.84E-01	9.72E-01
**AC004889.1**	0.37	3.59E-01	1.00E+00	-0.14	7.84E-01	9.72E-01
**AP001442.1**	0.76	7.95E-01	1.00E+00	-0.21	7.84E-01	9.72E-01
**AL135818.1**	-0.12	9.07E-01	1.00E+00	0.25	7.84E-01	9.72E-01
**ATP8A1**	-0.20	6.06E-01	1.00E+00	0.15	7.84E-01	9.72E-01
**AC007684.1**	2.49	3.02E-01	1.00E+00	-0.33	7.84E-01	9.72E-01

**LINGO2**	0.05	9.42E-01	1.00E+00	-0.24	7.85E-01	9.72E-01
**AC025171.1**	-0.10	8.19E-01	1.00E+00	0.12	7.85E-01	9.72E-01
**CDK16**	-0.06	7.94E-01	1.00E+00	0.10	7.85E-01	9.72E-01
**CCT4**	0.16	4.12E-01	1.00E+00	-0.05	7.85E-01	9.72E-01
**BMPR1B**	-0.95	4.69E-04	9.32E-02	-0.11	7.85E-01	9.72E-01
**DPY19L2P3**	-0.25	7.12E-01	1.00E+00	-0.17	7.85E-01	9.72E-01
**RPS4XP11**	-1.26	7.16E-01	1.00E+00	-0.24	7.85E-01	9.72E-01
**AL451085.1**	-2.22	2.41E-01	1.00E+00	0.36	7.85E-01	9.72E-01
**KRTAP4-8**	-20.07	5.11E-09	2.58E-06	-0.88	7.85E-01	9.72E-01
**FLG2**	0.20	7.74E-01	1.00E+00	0.20	7.85E-01	9.72E-01
**RPL15**	-0.04	8.87E-01	1.00E+00	-0.06	7.85E-01	9.72E-01
**TSSK6**	-1.33	3.62E-02	1.00E+00	0.18	7.85E-01	9.72E-01
**TTPAL**	0.31	4.16E-01	1.00E+00	0.07	7.85E-01	9.72E-01
**CHERP**	-0.32	2.93E-01	1.00E+00	0.11	7.85E-01	9.72E-01
**IPO9**	0.00	1.00E+00	1.00E+00	-0.05	7.85E-01	9.72E-01
**AL355488.1**	-0.67	6.56E-01	1.00E+00	-0.28	7.85E-01	9.72E-01
**CGN**	0.01	9.82E-01	1.00E+00	0.15	7.85E-01	9.72E-01
**AC067750.1**	0.86	2.13E-01	1.00E+00	0.16	7.85E-01	9.72E-01
**ZCCHC18**	-0.31	6.25E-01	1.00E+00	0.30	7.85E-01	9.72E-01
**SNPH**	-0.35	4.62E-01	1.00E+00	0.68	7.85E-01	9.72E-01
**ASPM**	0.12	7.59E-01	1.00E+00	-0.16	7.85E-01	9.72E-01
**ZNF474**	0.88	3.69E-01	1.00E+00	-0.28	7.85E-01	9.72E-01
**WNK4**	1.17	3.25E-01	1.00E+00	0.31	7.85E-01	9.72E-01
**PSMB5**	0.16	3.78E-01	1.00E+00	0.09	7.85E-01	9.72E-01
**AZGP1**	0.18	6.50E-01	1.00E+00	-0.10	7.85E-01	9.72E-01
**KRTAP5-3**	-19.62	1.01E-08	4.69E-06	-0.87	7.85E-01	9.72E-01
**AC005410.2**	-2.05	1.66E-01	1.00E+00	-0.25	7.85E-01	9.72E-01
**AC108134.2**	-0.64	3.34E-01	1.00E+00	0.21	7.86E-01	9.72E-01
**KRTAP10-1**	-29.97	9.47E-19	5.54E-15	-0.87	7.86E-01	9.72E-01
**CYB561D2**	-0.33	2.38E-01	1.00E+00	0.08	7.86E-01	9.72E-01
**RALGPS2**	-0.19	6.60E-01	1.00E+00	0.10	7.86E-01	9.72E-01
**THAP10**	-0.11	7.25E-01	1.00E+00	-0.11	7.86E-01	9.72E-01

**LINC01704**	-0.46	8.94E-01	1.00E+00	0.71	7.86E-01	9.72E-01
**AC069547.2**	-10.48	2.23E-03	2.39E-01	0.88	7.86E-01	9.72E-01
**CNGA1**	0.69	3.51E-02	1.00E+00	-0.11	7.86E-01	9.72E-01
**PCBP3**	-0.44	3.52E-01	1.00E+00	-0.17	7.86E-01	9.72E-01
**PPDPFL**	0.57	5.14E-01	1.00E+00	-0.21	7.86E-01	9.72E-01
**SCN4B**	0.17	7.68E-01	1.00E+00	-0.21	7.86E-01	9.72E-01
**MAP3K21**	-0.09	7.70E-01	1.00E+00	-0.15	7.86E-01	9.72E-01
**PCGF5**	0.13	4.78E-01	1.00E+00	-0.05	7.86E-01	9.72E-01
**USF3**	-0.23	2.40E-01	1.00E+00	0.10	7.86E-01	9.72E-01
**EIF5P1**	NA	NA	NA	0.22	7.86E-01	9.72E-01
**GLI3**	-0.32	3.69E-01	1.00E+00	0.09	7.86E-01	9.72E-01
**GATSL2**	0.46	6.25E-01	1.00E+00	-0.31	7.86E-01	9.72E-01
**LINC02185**	-0.23	7.15E-01	1.00E+00	0.20	7.86E-01	9.72E-01
**ADAM21**	-0.12	9.47E-01	1.00E+00	0.34	7.86E-01	9.72E-01
**CCDC184**	-0.18	6.81E-01	1.00E+00	0.29	7.86E-01	9.72E-01
**FENDRR**	-2.24	2.17E-01	1.00E+00	0.87	7.86E-01	9.72E-01
**GATA2-AS1**	-0.12	7.70E-01	1.00E+00	0.21	7.86E-01	9.72E-01
**FAN1**	0.33	1.34E-01	1.00E+00	-0.07	7.86E-01	9.72E-01
**NDUFA2**	-0.15	5.56E-01	1.00E+00	-0.13	7.86E-01	9.72E-01
**TRNAU1AP**	-0.12	5.58E-01	1.00E+00	-0.05	7.86E-01	9.72E-01
**AC016745.2**	-0.45	8.95E-01	1.00E+00	0.55	7.86E-01	9.72E-01
**KIFAP3**	0.14	4.29E-01	1.00E+00	0.05	7.87E-01	9.72E-01
**TBRG1**	-0.01	9.80E-01	1.00E+00	0.07	7.87E-01	9.72E-01
**ATP1B1**	-0.02	9.76E-01	1.00E+00	-0.11	7.87E-01	9.72E-01
**THBD**	-0.47	4.70E-01	1.00E+00	0.10	7.87E-01	9.72E-01
**NCAPD2**	-0.06	7.70E-01	1.00E+00	0.10	7.87E-01	9.72E-01
**PTGDR**	0.32	6.55E-01	1.00E+00	0.20	7.87E-01	9.72E-01
**ZNF227**	-0.03	9.00E-01	1.00E+00	0.11	7.87E-01	9.72E-01
**HPS6**	0.00	1.00E+00	1.00E+00	0.15	7.87E-01	9.72E-01
**AC093525.8**	1.27	3.80E-01	1.00E+00	0.45	7.87E-01	9.72E-01
**AC018475.1**	-0.36	7.71E-01	1.00E+00	-0.17	7.87E-01	9.72E-01
**ASGR1**	-0.14	7.57E-01	1.00E+00	-0.10	7.87E-01	9.72E-01

**AC004816.1**	-0.05	9.49E-01	1.00E+00	0.14	7.87E-01	9.72E-01
**MAP3K20-AS1**	-2.67	1.01E-01	1.00E+00	0.16	7.87E-01	9.72E-01
**CRTAC1**	-0.88	6.94E-02	1.00E+00	0.21	7.87E-01	9.72E-01
**HOXB3**	0.18	7.78E-01	1.00E+00	-0.14	7.87E-01	9.72E-01
**CSAD**	0.24	5.27E-01	1.00E+00	-0.10	7.87E-01	9.72E-01
**MAP3K19**	-1.21	5.74E-01	1.00E+00	-0.29	7.87E-01	9.72E-01
**SIM2**	0.99	2.30E-01	1.00E+00	-0.22	7.87E-01	9.72E-01
**SPRY1**	0.26	2.81E-01	1.00E+00	-0.09	7.87E-01	9.72E-01
**SYNJ2BP**	0.20	5.96E-01	1.00E+00	0.07	7.87E-01	9.72E-01
**LZTS2**	-0.15	4.65E-01	1.00E+00	0.15	7.88E-01	9.72E-01
**EIF4EBP3**	-0.20	8.95E-01	1.00E+00	0.24	7.88E-01	9.72E-01
**FAM155B**	0.34	6.62E-01	1.00E+00	0.85	7.88E-01	9.72E-01
**AL603965.1**	-0.42	7.20E-01	1.00E+00	0.20	7.88E-01	9.72E-01
**AL034397.3**	1.07	6.85E-01	1.00E+00	0.31	7.88E-01	9.72E-01
**AP000679.1**	-2.32	1.95E-01	1.00E+00	0.19	7.88E-01	9.72E-01
**PAPOLG**	0.19	5.13E-01	1.00E+00	-0.07	7.88E-01	9.72E-01
**AL591721.1**	0.37	8.91E-01	1.00E+00	-0.31	7.88E-01	9.72E-01
**KRT9**	-0.53	2.71E-01	1.00E+00	0.84	7.88E-01	9.72E-01
**PDE6D**	-0.47	2.15E-02	8.94E-01	-0.07	7.88E-01	9.72E-01
**EFNA5**	-0.08	7.75E-01	1.00E+00	-0.11	7.88E-01	9.72E-01
**ZNF234**	0.27	4.08E-01	1.00E+00	0.14	7.88E-01	9.72E-01
**AC136424.2**	1.23	4.69E-01	1.00E+00	0.35	7.88E-01	9.72E-01
**AC046143.2**	-0.44	7.32E-01	1.00E+00	0.27	7.88E-01	9.72E-01
**REV3L-IT1**	-1.91	5.75E-01	1.00E+00	-0.33	7.88E-01	9.72E-01
**AL109955.1**	-0.30	8.80E-01	1.00E+00	0.29	7.88E-01	9.72E-01
**XLOC_012467**	-0.68	4.11E-01	1.00E+00	-0.28	7.88E-01	9.72E-01
**L3HYPDH**	0.20	5.62E-01	1.00E+00	-0.09	7.88E-01	9.72E-01
**PCDHA4**	-0.46	5.89E-01	1.00E+00	0.32	7.88E-01	9.72E-01
**TRIM37**	-0.05	7.73E-01	1.00E+00	0.07	7.88E-01	9.72E-01
**CDKN2B-AS1**	-0.49	7.67E-01	1.00E+00	-0.29	7.88E-01	9.72E-01
**MOV10L1**	-1.97	2.26E-02	9.05E-01	0.24	7.88E-01	9.72E-01
**SRRM3**	-0.45	1.15E-01	1.00E+00	0.17	7.88E-01	9.72E-01

**SPEN**	-0.31	2.76E-01	1.00E+00	0.15	7.89E-01	9.72E-01
**MCM2**	-0.33	2.10E-01	1.00E+00	0.10	7.89E-01	9.72E-01
**LSMEM1**	-0.24	8.27E-01	1.00E+00	0.27	7.89E-01	9.72E-01
**SMARCD3**	-0.19	6.41E-01	1.00E+00	-0.18	7.89E-01	9.72E-01
**AP1S2**	-0.30	3.65E-01	1.00E+00	0.08	7.89E-01	9.72E-01
**GRM6**	-1.50	4.72E-01	1.00E+00	-0.39	7.89E-01	9.72E-01
**LINC01783**	-0.57	4.70E-01	1.00E+00	-0.40	7.89E-01	9.72E-01
**AC005261.5**	-0.52	5.76E-01	1.00E+00	-0.17	7.89E-01	9.72E-01
**CHAMP1**	0.13	5.08E-01	1.00E+00	-0.09	7.89E-01	9.72E-01
**AC010307.2**	-2.03	6.44E-02	1.00E+00	-0.22	7.89E-01	9.72E-01
**SLC4A10**	-3.96	1.40E-02	7.46E-01	0.26	7.89E-01	9.72E-01
**AC006277.1**	-2.99	1.22E-01	1.00E+00	-0.36	7.89E-01	9.72E-01
**RPL24P8**	0.27	7.42E-01	1.00E+00	-0.16	7.89E-01	9.72E-01
**PLA2G12A**	0.01	9.38E-01	1.00E+00	-0.07	7.89E-01	9.72E-01
**AF196972.1**	-1.34	3.39E-01	1.00E+00	-0.60	7.89E-01	9.72E-01
**KRT16P5**	-0.97	4.02E-01	1.00E+00	0.17	7.89E-01	9.72E-01
**NELFCD**	0.07	6.78E-01	1.00E+00	0.04	7.89E-01	9.72E-01
**G8925**	0.38	8.16E-01	1.00E+00	-0.86	7.89E-01	9.72E-01
**LTC4S**	0.11	7.55E-01	1.00E+00	0.09	7.89E-01	9.72E-01
**FLYWCH2**	-0.28	2.47E-01	1.00E+00	-0.07	7.89E-01	9.72E-01
**XLOC_005834**	0.92	7.31E-01	1.00E+00	-0.71	7.89E-01	9.72E-01
**PPAT**	0.00	9.96E-01	1.00E+00	0.09	7.89E-01	9.72E-01
**DPRXP3**	-1.03	6.42E-01	1.00E+00	-0.32	7.89E-01	9.72E-01
**NUTM2A**	0.08	9.33E-01	1.00E+00	0.84	7.89E-01	9.72E-01
**FANCA**	-0.74	3.68E-02	1.00E+00	-0.18	7.89E-01	9.72E-01
**TANGO6**	-0.23	3.09E-01	1.00E+00	0.09	7.89E-01	9.72E-01
**NSUN4**	0.00	9.83E-01	1.00E+00	0.05	7.89E-01	9.72E-01
**TMPRSS2**	0.08	9.25E-01	1.00E+00	-0.21	7.90E-01	9.72E-01
**LINC01514**	-1.17	3.70E-01	1.00E+00	0.20	7.90E-01	9.72E-01
**CEP126**	0.60	1.56E-01	1.00E+00	-0.10	7.90E-01	9.72E-01
**AR**	0.17	6.57E-01	1.00E+00	-0.10	7.90E-01	9.72E-01
**AL080317.2**	-1.30	5.05E-01	1.00E+00	-0.21	7.90E-01	9.72E-01

**G17702**	-0.99	3.32E-01	1.00E+00	-0.27	7.90E-01	9.72E-01
**PLEKHG5**	-0.39	2.64E-01	1.00E+00	-0.13	7.90E-01	9.73E-01
**OR2A9P**	-0.30	7.84E-01	1.00E+00	-0.14	7.90E-01	9.73E-01
**AC022872.1**	-1.95	2.44E-01	1.00E+00	-0.32	7.90E-01	9.73E-01
**PDLIM3**	0.31	5.41E-01	1.00E+00	0.08	7.90E-01	9.73E-01
**TMPO**	-0.08	7.52E-01	1.00E+00	0.06	7.90E-01	9.73E-01
**AP000487.1**	0.08	9.31E-01	1.00E+00	-0.15	7.90E-01	9.73E-01
**PSMC1P9**	-1.67	2.73E-01	1.00E+00	-0.19	7.90E-01	9.73E-01
**SELENOI**	-0.01	9.73E-01	1.00E+00	0.11	7.91E-01	9.73E-01
**CKS1BP7**	0.98	7.78E-01	1.00E+00	0.25	7.91E-01	9.73E-01
**STK4-AS1**	-0.27	7.94E-01	1.00E+00	-0.29	7.91E-01	9.73E-01
**CDC14A**	-0.14	5.93E-01	1.00E+00	0.10	7.91E-01	9.73E-01
**RTL8B**	-0.09	5.90E-01	1.00E+00	-0.05	7.91E-01	9.73E-01
**TAZ**	-0.03	9.20E-01	1.00E+00	-0.12	7.91E-01	9.73E-01
**AL137847.1**	-1.96	1.84E-01	1.00E+00	-0.24	7.91E-01	9.73E-01
**PXYLP1**	0.21	2.70E-01	1.00E+00	-0.10	7.91E-01	9.73E-01
**AC124312.2**	-2.47	4.79E-02	1.00E+00	0.24	7.91E-01	9.73E-01
**DLGAP5**	0.09	8.08E-01	1.00E+00	-0.14	7.91E-01	9.73E-01
**AC022336.3**	-2.30	1.74E-01	1.00E+00	0.46	7.91E-01	9.73E-01
**RAB11B**	-0.17	5.31E-01	1.00E+00	-0.10	7.91E-01	9.73E-01
**RAD54L**	0.68	1.45E-01	1.00E+00	-0.21	7.91E-01	9.73E-01
**AL078621.3**	0.58	2.11E-01	1.00E+00	-0.16	7.91E-01	9.73E-01
**TOM1L1**	-0.32	2.31E-01	1.00E+00	-0.07	7.92E-01	9.73E-01
**RTN4RL2**	-1.24	1.11E-03	1.75E-01	0.20	7.92E-01	9.73E-01
**ASPG**	-0.01	9.90E-01	1.00E+00	0.16	7.92E-01	9.74E-01
**G28942**	0.33	6.36E-01	1.00E+00	-0.17	7.92E-01	9.74E-01
**TPI1P2**	-0.82	1.42E-01	1.00E+00	-0.18	7.92E-01	9.74E-01
**KRTAP9-2**	-9.36	7.16E-03	5.13E-01	-0.85	7.92E-01	9.74E-01
**RPLP0P6**	-0.19	6.32E-01	1.00E+00	0.14	7.92E-01	9.74E-01
**KLRF1**	-0.33	7.14E-01	1.00E+00	0.15	7.92E-01	9.74E-01
**PNLIPRP3**	0.02	9.66E-01	1.00E+00	-0.17	7.92E-01	9.74E-01
**EIF2S1**	0.08	7.34E-01	1.00E+00	-0.07	7.92E-01	9.74E-01

**CBLC**	-0.25	6.11E-01	1.00E+00	0.16	7.92E-01	9.74E-01
**G31829**	3.57	1.44E-02	7.57E-01	0.24	7.92E-01	9.74E-01
**ANKMY1**	-0.38	1.90E-01	1.00E+00	0.09	7.92E-01	9.74E-01
**SERPINI1**	0.15	8.14E-01	1.00E+00	-0.11	7.92E-01	9.74E-01
**HIST1H2BB**	-1.87	5.81E-01	1.00E+00	-0.46	7.92E-01	9.74E-01
**CCDC85B**	-0.62	1.76E-02	8.33E-01	0.17	7.92E-01	9.74E-01
**SEC11A**	0.20	3.43E-01	1.00E+00	0.04	7.92E-01	9.74E-01
**AL589765.4**	0.15	7.58E-01	1.00E+00	0.30	7.92E-01	9.74E-01
**PDCL3P5**	-1.44	1.89E-01	1.00E+00	0.13	7.92E-01	9.74E-01
**G25097**	-1.09	3.83E-01	1.00E+00	0.84	7.92E-01	9.74E-01
**PDLIM2**	-0.07	8.65E-01	1.00E+00	-0.07	7.93E-01	9.74E-01
**PPIG**	0.07	7.22E-01	1.00E+00	0.05	7.93E-01	9.74E-01
**TMSB15B**	-0.21	6.52E-01	1.00E+00	0.11	7.93E-01	9.74E-01
**LIMCH1**	0.54	2.09E-01	1.00E+00	-0.08	7.93E-01	9.74E-01
**AC097376.1**	-2.56	6.72E-02	1.00E+00	0.32	7.93E-01	9.74E-01
**RPL26L1**	-0.30	9.97E-02	1.00E+00	0.06	7.93E-01	9.74E-01
**AC034213.1**	0.62	8.53E-01	1.00E+00	0.31	7.93E-01	9.74E-01
**AC024257.3**	-0.16	9.43E-01	1.00E+00	-0.31	7.93E-01	9.74E-01
**ANKRD19P**	0.73	4.13E-01	1.00E+00	0.11	7.93E-01	9.74E-01
**ADGRE1**	-0.15	9.36E-01	1.00E+00	0.83	7.93E-01	9.74E-01
**TTLL11**	0.01	9.71E-01	1.00E+00	-0.26	7.93E-01	9.74E-01
**RPL35**	-0.14	6.33E-01	1.00E+00	0.07	7.93E-01	9.74E-01
**SNRPA1**	0.13	5.84E-01	1.00E+00	-0.06	7.93E-01	9.74E-01
**TIMM10**	-0.01	9.59E-01	1.00E+00	-0.05	7.93E-01	9.74E-01
**UCKL1-AS1**	-1.74	3.27E-01	1.00E+00	0.31	7.93E-01	9.74E-01
**AP002851.1**	-0.09	8.95E-01	1.00E+00	0.11	7.93E-01	9.74E-01
**AC051619.5**	-2.38	9.17E-02	1.00E+00	-0.28	7.93E-01	9.74E-01
**PHTF2**	0.07	8.43E-01	1.00E+00	0.05	7.93E-01	9.74E-01
**AC020663.3**	-0.35	9.18E-01	1.00E+00	0.25	7.93E-01	9.74E-01
**BEND6**	-0.12	8.82E-01	1.00E+00	-0.13	7.93E-01	9.74E-01
**AMFR**	0.18	4.19E-01	1.00E+00	0.12	7.93E-01	9.74E-01
**SPRY4-AS1**	-0.73	7.10E-01	1.00E+00	-0.22	7.93E-01	9.74E-01

**LINC01686**	-0.55	4.95E-01	1.00E+00	0.23	7.93E-01	9.74E-01
**MGAT4B**	-0.01	9.57E-01	1.00E+00	0.24	7.93E-01	9.74E-01
**ZYG11A**	-1.89	1.73E-01	1.00E+00	0.29	7.94E-01	9.74E-01
**SSSCA1-AS1**	-0.35	3.59E-01	1.00E+00	0.09	7.94E-01	9.74E-01
**G32993**	-1.16	2.91E-01	1.00E+00	0.21	7.94E-01	9.74E-01
**CDH6**	-0.38	4.16E-01	1.00E+00	0.13	7.94E-01	9.74E-01
**AC012085.1**	-0.66	6.93E-02	1.00E+00	-0.10	7.94E-01	9.74E-01
**LZTR1**	0.39	3.76E-01	1.00E+00	0.11	7.94E-01	9.74E-01
**ZNF792**	-0.11	7.25E-01	1.00E+00	0.18	7.94E-01	9.74E-01
**GAS2L2**	0.75	4.40E-01	1.00E+00	0.31	7.94E-01	9.74E-01
**FBLL1**	-0.68	4.22E-01	1.00E+00	0.31	7.94E-01	9.74E-01
**CLIC1P1**	0.52	8.81E-01	1.00E+00	-0.21	7.94E-01	9.74E-01
**SORCS1**	-0.29	5.50E-01	1.00E+00	-0.21	7.94E-01	9.74E-01
**KRTAP4-5**	-12.19	4.13E-04	8.56E-02	-0.84	7.94E-01	9.74E-01
**EPX**	0.39	7.15E-01	1.00E+00	0.20	7.94E-01	9.74E-01
**HSPA4L**	-0.11	7.30E-01	1.00E+00	0.10	7.94E-01	9.74E-01
**ZNF416**	0.15	6.14E-01	1.00E+00	0.07	7.94E-01	9.74E-01
**AC093702.1**	-2.87	5.71E-02	1.00E+00	-0.40	7.94E-01	9.74E-01
**KREMEN1**	-0.06	8.21E-01	1.00E+00	-0.13	7.94E-01	9.74E-01
**AL390039.1**	NA	NA	NA	-0.21	7.94E-01	9.74E-01
**CNRIP1**	0.39	3.71E-01	1.00E+00	0.12	7.95E-01	9.74E-01
**NFIB**	0.15	6.41E-01	1.00E+00	-0.09	7.95E-01	9.74E-01
**CD5**	-0.53	2.69E-01	1.00E+00	0.82	7.95E-01	9.74E-01
**TCTN3**	0.00	9.89E-01	1.00E+00	-0.05	7.95E-01	9.74E-01
**ATP6V1E2**	-0.14	5.83E-01	1.00E+00	-0.09	7.95E-01	9.74E-01
**LGR5**	0.88	5.23E-01	1.00E+00	-0.75	7.95E-01	9.74E-01
**FBXW7**	-0.14	6.74E-01	1.00E+00	0.11	7.95E-01	9.74E-01
**PRPF3**	-0.08	7.20E-01	1.00E+00	0.06	7.95E-01	9.74E-01
**SLC22A4**	0.56	4.63E-01	1.00E+00	0.13	7.95E-01	9.74E-01
**MIEF2**	0.13	5.60E-01	1.00E+00	0.09	7.95E-01	9.74E-01
**AC121338.2**	0.26	6.56E-01	1.00E+00	-0.20	7.95E-01	9.74E-01
**ZNF501**	0.19	5.64E-01	1.00E+00	0.11	7.95E-01	9.74E-01

**AC068473.5**	0.24	2.84E-01	1.00E+00	-0.09	7.95E-01	9.74E-01
**SORD**	0.16	7.58E-01	1.00E+00	-0.10	7.95E-01	9.74E-01
**AL592295.3**	-2.92	1.10E-01	1.00E+00	-0.30	7.95E-01	9.74E-01
**RPL18AP7**	-0.10	9.59E-01	1.00E+00	0.24	7.95E-01	9.74E-01
**AC007041.1**	-2.96	3.82E-01	1.00E+00	-0.17	7.95E-01	9.74E-01
**IFNAR2**	0.57	6.10E-01	1.00E+00	0.14	7.95E-01	9.74E-01
**AC112715.1**	0.05	9.88E-01	1.00E+00	0.30	7.95E-01	9.74E-01
**IPO13**	-0.16	3.74E-01	1.00E+00	0.14	7.95E-01	9.74E-01
**AC093726.2**	-2.09	3.26E-01	1.00E+00	0.37	7.95E-01	9.74E-01
**XLOC_011448**	-0.84	5.90E-01	1.00E+00	-0.16	7.95E-01	9.74E-01
**ZNF500**	-0.30	2.30E-01	1.00E+00	0.12	7.96E-01	9.75E-01
**ZNF223**	1.20	2.05E-01	1.00E+00	-0.16	7.96E-01	9.75E-01
**EXTL3-AS1**	3.02	1.68E-01	1.00E+00	0.35	7.96E-01	9.75E-01
**AC024995.1**	NA	NA	NA	-0.23	7.96E-01	9.75E-01
**ACTG2**	-0.45	5.06E-01	1.00E+00	0.12	7.96E-01	9.75E-01
**GOLGA8N**	0.52	6.16E-01	1.00E+00	0.25	7.96E-01	9.75E-01
**RNF212**	0.07	8.52E-01	1.00E+00	-0.12	7.96E-01	9.75E-01
**SPAG8**	-0.83	4.12E-01	1.00E+00	-0.22	7.96E-01	9.75E-01
**ANKRD22**	0.29	4.84E-01	1.00E+00	-0.16	7.96E-01	9.75E-01
**AC073343.1**	-0.79	4.92E-01	1.00E+00	0.35	7.96E-01	9.75E-01
**AC244154.1**	-0.24	6.91E-01	1.00E+00	0.16	7.96E-01	9.75E-01
**GLP1R**	-0.63	4.89E-01	1.00E+00	0.33	7.96E-01	9.75E-01
**CPXM2**	-0.09	8.36E-01	1.00E+00	0.10	7.96E-01	9.75E-01
**HEXDC-IT1**	0.02	9.90E-01	1.00E+00	0.32	7.96E-01	9.75E-01
**TMEM9B-AS1**	-0.21	6.91E-01	1.00E+00	-0.18	7.96E-01	9.75E-01
**CD1B**	-0.47	5.64E-01	1.00E+00	-0.22	7.97E-01	9.75E-01
**TMPRSS9**	-0.26	7.28E-01	1.00E+00	0.30	7.97E-01	9.75E-01
**TCF7L1**	0.16	7.24E-01	1.00E+00	0.12	7.97E-01	9.75E-01
**HNRNPKP1**	-1.10	7.53E-01	1.00E+00	0.23	7.97E-01	9.75E-01
**SF3A1**	-0.22	2.88E-01	1.00E+00	0.07	7.97E-01	9.75E-01
**G4402**	-0.01	9.92E-01	1.00E+00	-0.27	7.97E-01	9.75E-01
**SCYL2**	0.00	9.96E-01	1.00E+00	0.04	7.97E-01	9.75E-01

**AC079322.1**	0.08	9.69E-01	1.00E+00	-0.37	7.97E-01	9.75E-01
**AC013652.1**	-0.53	4.47E-01	1.00E+00	-0.16	7.97E-01	9.75E-01
**CDS1**	0.37	2.74E-01	1.00E+00	-0.10	7.97E-01	9.75E-01
**TRIM32**	0.14	4.58E-01	1.00E+00	-0.06	7.97E-01	9.75E-01
**LTA4H**	0.17	5.36E-01	1.00E+00	0.06	7.97E-01	9.75E-01
**AC011773.3**	1.34	6.98E-01	1.00E+00	-0.31	7.97E-01	9.75E-01
**MUM1L1**	-2.22	8.29E-02	1.00E+00	0.31	7.97E-01	9.75E-01
**AC245452.1**	-0.60	2.41E-01	1.00E+00	0.14	7.97E-01	9.75E-01
**AL031587.3**	-0.40	7.90E-01	1.00E+00	-0.28	7.98E-01	9.75E-01
**LPAR5**	-0.38	2.27E-01	1.00E+00	0.10	7.98E-01	9.75E-01
**GRAMD2B**	0.14	6.55E-01	1.00E+00	-0.07	7.98E-01	9.75E-01
**AC093909.6**	0.26	6.00E-01	1.00E+00	-0.11	7.98E-01	9.75E-01
**AC021483.2**	1.31	5.67E-01	1.00E+00	0.40	7.98E-01	9.75E-01
**IER2**	-1.13	5.44E-02	1.00E+00	-0.11	7.98E-01	9.75E-01
**ZNF696**	-0.03	8.99E-01	1.00E+00	-0.11	7.98E-01	9.75E-01
**AJUBA**	-0.04	8.93E-01	1.00E+00	-0.10	7.98E-01	9.75E-01
**CACNG8**	-4.29	3.18E-05	8.82E-03	-0.29	7.98E-01	9.75E-01
**NEGR1**	0.34	4.35E-01	1.00E+00	0.14	7.98E-01	9.75E-01
**CNOT6LP1**	-0.04	9.57E-01	1.00E+00	0.16	7.98E-01	9.75E-01
**GALC**	0.23	3.53E-01	1.00E+00	-0.06	7.98E-01	9.75E-01
**ARL4AP5**	-0.08	9.82E-01	1.00E+00	-0.19	7.98E-01	9.75E-01
**AURKAIP1**	-0.20	3.59E-01	1.00E+00	-0.05	7.98E-01	9.75E-01
**SNHG17**	0.44	6.12E-02	1.00E+00	-0.09	7.98E-01	9.75E-01
**XLOC_012664**	NA	NA	NA	-0.49	7.98E-01	9.75E-01
**BANF1**	-0.11	5.62E-01	1.00E+00	0.04	7.98E-01	9.75E-01
**PLS1**	-0.37	3.21E-01	1.00E+00	-0.13	7.98E-01	9.75E-01
**ADPRHL1**	-0.41	2.27E-01	1.00E+00	-0.08	7.98E-01	9.75E-01
**TOR3A**	-0.06	7.15E-01	1.00E+00	0.07	7.98E-01	9.75E-01
**FLJ13224**	-0.13	9.48E-01	1.00E+00	-0.28	7.98E-01	9.75E-01
**USP53**	0.51	4.47E-02	1.00E+00	-0.06	7.98E-01	9.75E-01
**AQP6**	-0.30	8.57E-01	1.00E+00	-0.42	7.98E-01	9.75E-01
**FTLP3**	-0.64	5.84E-01	1.00E+00	-0.19	7.98E-01	9.75E-01

**NFIX**	-0.34	1.56E-01	1.00E+00	-0.15	7.98E-01	9.75E-01
**HMGCLL1**	0.41	5.95E-01	1.00E+00	0.26	7.98E-01	9.75E-01
**EIF4A2**	0.57	3.70E-02	1.00E+00	0.07	7.99E-01	9.75E-01
**RIPPLY3**	-1.01	3.86E-01	1.00E+00	-0.29	7.99E-01	9.75E-01
**CCDC68**	0.71	2.22E-01	1.00E+00	-0.50	7.99E-01	9.75E-01
**EPB41L4A**	-0.15	4.69E-01	1.00E+00	-0.06	7.99E-01	9.75E-01
**FAM25C**	0.66	4.23E-01	1.00E+00	-0.20	7.99E-01	9.75E-01
**RPS4XP3**	-1.71	6.18E-01	1.00E+00	-0.20	7.99E-01	9.75E-01
**MLYCD**	0.31	2.28E-01	1.00E+00	-0.17	7.99E-01	9.75E-01
**CD40**	-0.20	4.53E-01	1.00E+00	-0.08	7.99E-01	9.75E-01
**APC**	-0.23	2.44E-01	1.00E+00	0.07	7.99E-01	9.75E-01
**HDGFL2**	-0.12	5.32E-01	1.00E+00	0.23	7.99E-01	9.75E-01
**OR7E108P**	0.28	7.43E-01	1.00E+00	-0.26	7.99E-01	9.75E-01
**AL096828.3**	0.40	8.34E-01	1.00E+00	0.30	7.99E-01	9.75E-01
**EN1**	-0.24	5.60E-01	1.00E+00	-0.12	7.99E-01	9.75E-01
**CTSV**	0.28	6.74E-01	1.00E+00	-0.17	7.99E-01	9.75E-01
**CKS2**	0.32	3.38E-01	1.00E+00	-0.11	7.99E-01	9.75E-01
**LINC00863**	-0.26	5.27E-01	1.00E+00	-0.07	7.99E-01	9.75E-01
**VTCN1**	0.42	6.38E-01	1.00E+00	-0.22	7.99E-01	9.75E-01
**DLX5**	-0.07	8.77E-01	1.00E+00	0.12	7.99E-01	9.75E-01
**STAG3L3**	-0.04	9.08E-01	1.00E+00	0.08	7.99E-01	9.75E-01
**TLL2**	-1.93	1.07E-02	6.39E-01	0.23	7.99E-01	9.75E-01
**KLHDC3**	0.14	5.46E-01	1.00E+00	0.04	7.99E-01	9.75E-01
**RPS23**	-0.91	5.48E-02	1.00E+00	0.10	7.99E-01	9.75E-01
**DNM1P46**	1.58	3.20E-01	1.00E+00	-0.25	7.99E-01	9.75E-01
**DACT3**	-0.40	3.75E-01	1.00E+00	0.23	7.99E-01	9.75E-01
**AC136424.1**	1.51	6.62E-01	1.00E+00	-0.82	8.00E-01	9.75E-01
**CIAO1**	0.16	3.89E-01	1.00E+00	0.04	8.00E-01	9.75E-01
**SPACA4**	-1.14	1.60E-01	1.00E+00	0.29	8.00E-01	9.75E-01
**WDR25**	-0.13	6.10E-01	1.00E+00	-0.05	8.00E-01	9.75E-01
**AC093388.1**	0.59	4.38E-01	1.00E+00	-0.16	8.00E-01	9.75E-01
**PTCSC2**	-0.72	8.34E-01	1.00E+00	0.41	8.00E-01	9.75E-01

**HRASLS2**	-3.28	1.10E-01	1.00E+00	-0.76	8.00E-01	9.75E-01
**BAIAP3**	-0.38	3.27E-01	1.00E+00	-0.15	8.00E-01	9.75E-01
**AL109741.1**	0.00	9.97E-01	1.00E+00	-0.21	8.00E-01	9.75E-01
**AC245041.1**	-0.19	7.81E-01	1.00E+00	0.15	8.00E-01	9.75E-01
**PSMA6P2**	NA	NA	NA	0.17	8.00E-01	9.75E-01
**WBP1**	0.06	8.49E-01	1.00E+00	-0.09	8.00E-01	9.75E-01
**AC022568.1**	-1.40	6.04E-01	1.00E+00	-0.41	8.00E-01	9.75E-01
**AC069528.2**	-0.44	7.99E-01	1.00E+00	-0.22	8.00E-01	9.75E-01
**GLI2**	-0.88	6.29E-02	1.00E+00	-0.16	8.00E-01	9.75E-01
**MIR17HG**	0.27	8.86E-01	1.00E+00	0.33	8.00E-01	9.75E-01
**UFSP1**	0.14	7.58E-01	1.00E+00	0.07	8.00E-01	9.75E-01
**NCBP1**	0.01	9.58E-01	1.00E+00	-0.05	8.00E-01	9.75E-01
**KLHL15**	0.09	7.79E-01	1.00E+00	-0.11	8.00E-01	9.75E-01
**PTGFR**	0.77	1.64E-01	1.00E+00	0.11	8.00E-01	9.75E-01
**SLC25A5-AS1**	0.97	8.46E-02	1.00E+00	0.14	8.00E-01	9.75E-01
**KLF10**	-0.49	2.90E-01	1.00E+00	0.07	8.01E-01	9.75E-01
**ABCD4**	0.06	8.34E-01	1.00E+00	0.08	8.01E-01	9.75E-01
**LINC01659**	1.10	1.39E-01	1.00E+00	-0.26	8.01E-01	9.75E-01
**PPP4R1L**	-0.04	9.34E-01	1.00E+00	-0.10	8.01E-01	9.75E-01
**RTKN**	-0.26	4.24E-01	1.00E+00	0.09	8.01E-01	9.75E-01
**SPATA32**	-1.61	5.83E-01	1.00E+00	-0.28	8.01E-01	9.75E-01
**TRPM2-AS**	0.59	8.39E-01	1.00E+00	0.28	8.01E-01	9.75E-01
**AL390957.1**	-0.26	8.02E-01	1.00E+00	0.20	8.01E-01	9.75E-01
**TRAPPC8**	0.31	2.36E-01	1.00E+00	-0.06	8.01E-01	9.75E-01
**AC114737.1**	1.13	7.44E-01	1.00E+00	0.35	8.01E-01	9.75E-01
**SEC23IP**	0.31	1.35E-01	1.00E+00	0.03	8.01E-01	9.75E-01
**CANX**	0.06	7.82E-01	1.00E+00	-0.05	8.01E-01	9.75E-01
**EFCAB6**	-0.42	4.44E-01	1.00E+00	-0.14	8.01E-01	9.75E-01
**RPSAP12**	0.22	7.01E-01	1.00E+00	-0.10	8.01E-01	9.75E-01
**GMEB2**	-0.04	8.69E-01	1.00E+00	0.13	8.01E-01	9.75E-01
**VSIG10**	0.00	9.86E-01	1.00E+00	0.07	8.01E-01	9.75E-01
**MAP7**	-0.30	3.76E-01	1.00E+00	0.09	8.01E-01	9.75E-01

**AC090181.3**	-0.56	8.73E-01	1.00E+00	0.34	8.01E-01	9.75E-01
**AFM**	-1.73	3.68E-01	1.00E+00	-0.32	8.01E-01	9.75E-01
**EML2**	-0.01	9.51E-01	1.00E+00	-0.11	8.01E-01	9.75E-01
**RNASEH1**	0.15	4.57E-01	1.00E+00	0.07	8.01E-01	9.75E-01
**SAC3D1**	0.00	9.98E-01	1.00E+00	-0.19	8.01E-01	9.75E-01
**IQSEC3**	1.73	1.05E-01	1.00E+00	-0.24	8.01E-01	9.75E-01
**GPR155**	-0.07	7.98E-01	1.00E+00	-0.07	8.02E-01	9.75E-01
**MARK1**	0.05	8.99E-01	1.00E+00	0.12	8.02E-01	9.75E-01
**OXT**	0.21	8.38E-01	1.00E+00	-0.21	8.02E-01	9.75E-01
**RIC8A**	-0.10	5.15E-01	1.00E+00	0.04	8.02E-01	9.75E-01
**SERPIND1**	-1.63	3.41E-01	1.00E+00	0.71	8.02E-01	9.75E-01
**RAB6C-AS1**	-0.22	9.36E-01	1.00E+00	0.29	8.02E-01	9.75E-01
**CTNNA3**	0.15	8.01E-01	1.00E+00	-0.20	8.02E-01	9.75E-01
**C1GALT1C1L**	0.37	5.72E-01	1.00E+00	-0.14	8.02E-01	9.75E-01
**JAG1**	-0.29	9.79E-02	1.00E+00	0.09	8.02E-01	9.75E-01
**AC106872.5**	3.02	8.84E-02	1.00E+00	0.27	8.02E-01	9.75E-01
**AC124067.2**	-1.58	6.63E-02	1.00E+00	-0.14	8.02E-01	9.75E-01
**MPST**	0.09	7.72E-01	1.00E+00	-0.05	8.02E-01	9.75E-01
**AIF1L**	0.19	6.39E-01	1.00E+00	-0.09	8.02E-01	9.75E-01
**ADRM1**	-0.23	3.45E-01	1.00E+00	0.09	8.02E-01	9.75E-01
**CDH7**	-0.88	5.00E-01	1.00E+00	0.36	8.02E-01	9.75E-01
**ZFYVE19**	0.13	4.97E-01	1.00E+00	0.05	8.02E-01	9.75E-01
**LRRC8E**	-0.74	3.33E-01	1.00E+00	0.15	8.02E-01	9.75E-01
**XLOC_004631**	0.10	8.57E-01	1.00E+00	-0.15	8.02E-01	9.75E-01
**RPL10AP6**	-0.64	2.68E-01	1.00E+00	0.19	8.02E-01	9.75E-01
**GIPC3**	-0.33	5.30E-01	1.00E+00	0.21	8.02E-01	9.75E-01
**KLHL4**	0.02	9.82E-01	1.00E+00	-0.50	8.02E-01	9.75E-01
**DFFA**	-0.22	2.81E-01	1.00E+00	0.04	8.02E-01	9.75E-01
**CAMK1D**	0.01	9.84E-01	1.00E+00	0.08	8.02E-01	9.75E-01
**RBM4**	-0.61	9.51E-03	6.06E-01	0.09	8.02E-01	9.75E-01
**C16orf70**	-0.15	6.43E-01	1.00E+00	0.11	8.02E-01	9.75E-01
**AC020904.2**	-0.45	6.94E-01	1.00E+00	0.08	8.02E-01	9.75E-01

**RPL19P21**	NA	NA	NA	0.44	8.02E-01	9.75E-01
**CSE1L-AS1**	-0.05	9.88E-01	1.00E+00	0.39	8.02E-01	9.75E-01
**ANKRD28**	0.10	5.63E-01	1.00E+00	0.06	8.02E-01	9.75E-01
**COASY**	-0.15	4.41E-01	1.00E+00	0.06	8.03E-01	9.75E-01
**TM4SF1**	0.24	6.25E-01	1.00E+00	0.08	8.03E-01	9.75E-01
**RPL39P3**	-0.28	5.42E-01	1.00E+00	0.13	8.03E-01	9.75E-01
**C8orf48**	-0.12	7.02E-01	1.00E+00	-0.12	8.03E-01	9.75E-01
**LINC02541**	0.03	9.38E-01	1.00E+00	0.13	8.03E-01	9.75E-01
**LMX1A**	-0.05	9.54E-01	1.00E+00	-0.28	8.03E-01	9.75E-01
**TUSC8**	-0.66	5.90E-01	1.00E+00	-0.24	8.03E-01	9.75E-01
**STK11IP**	-0.04	8.88E-01	1.00E+00	0.14	8.03E-01	9.75E-01
**ATP7B**	0.27	5.75E-01	1.00E+00	0.18	8.03E-01	9.75E-01
**SLC24A3**	0.00	9.95E-01	1.00E+00	0.12	8.03E-01	9.75E-01
**ABTB2**	-0.14	5.97E-01	1.00E+00	0.13	8.03E-01	9.75E-01
**WDR19**	0.06	8.31E-01	1.00E+00	0.07	8.03E-01	9.75E-01
**PA2G4**	-0.14	5.36E-01	1.00E+00	-0.04	8.03E-01	9.75E-01
**RHBDL1**	-0.50	2.68E-01	1.00E+00	0.14	8.03E-01	9.75E-01
**FAM27E3**	-0.61	7.71E-01	1.00E+00	0.30	8.03E-01	9.75E-01
**AL024507.2**	-0.05	9.53E-01	1.00E+00	0.23	8.03E-01	9.75E-01
**CLEC18B**	-0.65	6.29E-01	1.00E+00	0.39	8.03E-01	9.75E-01
**LINC01794**	-0.19	9.04E-01	1.00E+00	0.31	8.03E-01	9.75E-01
**HAP1**	-1.11	1.15E-01	1.00E+00	0.29	8.03E-01	9.75E-01
**MPV17L2**	-0.30	1.80E-01	1.00E+00	-0.05	8.03E-01	9.75E-01
**AC104447.1**	-0.61	3.27E-01	1.00E+00	0.23	8.03E-01	9.75E-01
**LSP1**	-0.37	2.11E-01	1.00E+00	0.08	8.03E-01	9.75E-01
**NUAK2**	-1.20	1.42E-01	1.00E+00	-0.13	8.03E-01	9.75E-01
**PSAPL1**	0.01	9.90E-01	1.00E+00	-0.19	8.03E-01	9.75E-01
**CEP128**	-0.40	1.49E-01	1.00E+00	-0.11	8.03E-01	9.75E-01
**BX323845.3**	NA	NA	NA	-0.21	8.03E-01	9.75E-01
**FBXO24**	1.14	5.45E-01	1.00E+00	0.25	8.03E-01	9.75E-01
**SPDYE16**	0.23	8.90E-01	1.00E+00	0.22	8.03E-01	9.75E-01
**FAM76B**	0.01	9.73E-01	1.00E+00	-0.06	8.03E-01	9.75E-01

**MAGOH3P**	NA	NA	NA	-0.19	8.03E-01	9.75E-01
**LINC00857**	0.23	8.08E-01	1.00E+00	-0.26	8.03E-01	9.75E-01
**MLANA**	0.27	5.34E-01	1.00E+00	0.20	8.03E-01	9.75E-01
**AC009948.4**	-3.15	2.81E-01	1.00E+00	0.18	8.03E-01	9.75E-01
**CDCA2**	-0.19	6.08E-01	1.00E+00	0.16	8.03E-01	9.75E-01
**TPRG1**	0.30	4.38E-01	1.00E+00	-0.11	8.04E-01	9.75E-01
**PCAT19**	0.13	8.10E-01	1.00E+00	-0.13	8.04E-01	9.75E-01
**TUT1**	0.36	2.85E-01	1.00E+00	0.09	8.04E-01	9.75E-01
**G1997**	-2.00	7.78E-02	1.00E+00	0.29	8.04E-01	9.75E-01
**PFKFB2**	0.04	9.07E-01	1.00E+00	0.07	8.04E-01	9.75E-01
**JAGN1**	0.10	5.25E-01	1.00E+00	-0.06	8.04E-01	9.75E-01
**SLC45A1**	-0.52	2.48E-01	1.00E+00	0.18	8.04E-01	9.75E-01
**AL731571.1**	-0.09	8.86E-01	1.00E+00	0.15	8.04E-01	9.75E-01
**HCN2**	-1.05	5.90E-03	4.57E-01	0.74	8.04E-01	9.75E-01
**AC000089.1**	0.16	8.85E-01	1.00E+00	0.21	8.04E-01	9.75E-01
**DCSTAMP**	-0.86	8.05E-01	1.00E+00	0.79	8.04E-01	9.75E-01
**UBE2S**	-0.20	4.66E-01	1.00E+00	0.23	8.04E-01	9.75E-01
**TMC2**	1.40	2.34E-01	1.00E+00	0.31	8.04E-01	9.75E-01
**AL353791.1**	1.49	7.33E-02	1.00E+00	-0.18	8.04E-01	9.75E-01
**ITPRIPL2**	0.13	6.25E-01	1.00E+00	-0.11	8.04E-01	9.75E-01
**AC007066.2**	0.24	6.79E-01	1.00E+00	0.28	8.04E-01	9.75E-01
**HELLS**	0.15	5.74E-01	1.00E+00	-0.13	8.04E-01	9.75E-01
**SPERT**	-2.84	2.63E-02	9.46E-01	0.31	8.04E-01	9.75E-01
**PROSER2**	-0.09	7.53E-01	1.00E+00	-0.18	8.04E-01	9.75E-01
**TOLLIP**	-0.12	6.63E-01	1.00E+00	0.04	8.04E-01	9.75E-01
**SH2D7**	-1.23	4.35E-01	1.00E+00	0.33	8.04E-01	9.75E-01
**TBCD**	-0.12	5.49E-01	1.00E+00	0.09	8.04E-01	9.75E-01
**SHBG**	-0.65	6.42E-01	1.00E+00	-0.27	8.04E-01	9.75E-01
**AL022718.1**	NA	NA	NA	0.19	8.04E-01	9.75E-01
**UPF1**	0.01	9.46E-01	1.00E+00	0.17	8.04E-01	9.75E-01
**SLC25A35**	-0.26	5.69E-01	1.00E+00	0.12	8.04E-01	9.75E-01
**MFSD2A**	-0.23	6.86E-01	1.00E+00	-0.16	8.04E-01	9.75E-01

**AL445228.2**	-2.36	1.33E-01	1.00E+00	-0.24	8.04E-01	9.75E-01
**AC005632.2**	-0.80	7.51E-01	1.00E+00	0.26	8.05E-01	9.75E-01
**CIAO2A**	0.24	3.58E-01	1.00E+00	-0.08	8.05E-01	9.75E-01
**EMILIN1**	0.01	9.77E-01	1.00E+00	0.77	8.05E-01	9.75E-01
**SPTSSB**	0.23	5.73E-01	1.00E+00	-0.16	8.05E-01	9.75E-01
**SLC4A1APP1**	-0.16	9.39E-01	1.00E+00	-0.16	8.05E-01	9.75E-01
**HMG20A**	0.04	8.13E-01	1.00E+00	0.05	8.05E-01	9.75E-01
**ARHGEF38**	-0.69	4.79E-01	1.00E+00	0.17	8.05E-01	9.75E-01
**DHX36**	0.18	2.59E-01	1.00E+00	-0.08	8.05E-01	9.75E-01
**KIF12**	-0.58	4.19E-01	1.00E+00	-0.18	8.05E-01	9.75E-01
**POC1A**	-0.06	8.00E-01	1.00E+00	0.09	8.05E-01	9.75E-01
**HOOK1**	0.17	6.64E-01	1.00E+00	-0.12	8.05E-01	9.75E-01
**EIF4A1P7**	0.25	9.42E-01	1.00E+00	0.22	8.05E-01	9.75E-01
**AC233723.2**	-0.44	2.30E-01	1.00E+00	-0.14	8.05E-01	9.75E-01
**RPL34**	-0.01	9.62E-01	1.00E+00	0.11	8.05E-01	9.75E-01
**UBAP1L**	0.28	7.20E-01	1.00E+00	-0.26	8.05E-01	9.75E-01
**GNA14**	0.13	7.28E-01	1.00E+00	-0.13	8.05E-01	9.75E-01
**BX322639.1**	-1.00	2.11E-01	1.00E+00	0.18	8.05E-01	9.75E-01
**MAST4**	-0.02	9.39E-01	1.00E+00	0.08	8.05E-01	9.75E-01
**AC008897.2**	-3.53	8.92E-02	1.00E+00	0.27	8.05E-01	9.75E-01
**AC093159.1**	-2.44	8.84E-02	1.00E+00	0.28	8.05E-01	9.75E-01
**ABCA12**	0.05	9.27E-01	1.00E+00	0.18	8.06E-01	9.75E-01
**LARP6**	0.47	3.22E-01	1.00E+00	-0.16	8.06E-01	9.75E-01
**CD3EAP**	-0.22	5.29E-01	1.00E+00	-0.08	8.06E-01	9.75E-01
**HNRNPA1P4**	1.22	2.30E-01	1.00E+00	-0.10	8.06E-01	9.75E-01
**CHRDL1**	1.57	3.24E-02	1.00E+00	0.17	8.06E-01	9.75E-01
**AL645568.1**	-0.16	7.99E-01	1.00E+00	0.14	8.06E-01	9.75E-01
**KRTAP9-9**	-6.43	6.45E-02	1.00E+00	-0.79	8.06E-01	9.75E-01
**MYCN**	-0.59	4.23E-01	1.00E+00	-0.21	8.06E-01	9.75E-01
**G3878**	0.28	8.43E-01	1.00E+00	0.48	8.06E-01	9.75E-01
**ABI1**	-0.04	8.81E-01	1.00E+00	-0.06	8.06E-01	9.75E-01
**LINC02068**	-0.62	6.94E-01	1.00E+00	0.25	8.06E-01	9.75E-01

**AC138028.2**	-0.52	5.77E-01	1.00E+00	-0.36	8.06E-01	9.75E-01
**KIR3DL2**	-0.29	8.97E-01	1.00E+00	0.38	8.06E-01	9.75E-01
**AL356423.1**	NA	NA	NA	0.37	8.06E-01	9.75E-01
**PGK1**	0.00	9.80E-01	1.00E+00	0.04	8.06E-01	9.75E-01
**AC099522.1**	0.56	8.71E-01	1.00E+00	-0.20	8.06E-01	9.75E-01
**MYO19**	-0.06	7.28E-01	1.00E+00	0.08	8.06E-01	9.75E-01
**AC010491.1**	-0.21	7.78E-01	1.00E+00	-0.21	8.06E-01	9.75E-01
**PNMA1**	0.12	7.40E-01	1.00E+00	0.11	8.06E-01	9.75E-01
**ZMYM4-AS1**	NA	NA	NA	-0.32	8.06E-01	9.75E-01
**CCDC163**	-0.10	8.71E-01	1.00E+00	-0.14	8.06E-01	9.75E-01
**AC022509.2**	0.61	4.28E-01	1.00E+00	-0.14	8.06E-01	9.75E-01
**ZC3H12B**	-0.28	6.79E-01	1.00E+00	0.16	8.06E-01	9.75E-01
**YES1**	-0.04	8.58E-01	1.00E+00	-0.05	8.06E-01	9.75E-01
**GPR180**	0.12	7.42E-01	1.00E+00	-0.09	8.06E-01	9.75E-01
**RPLP2**	-0.10	6.66E-01	1.00E+00	0.05	8.06E-01	9.75E-01
**LINC01638**	4.65	1.37E-01	1.00E+00	-0.30	8.07E-01	9.75E-01
**CLUAP1**	0.14	5.34E-01	1.00E+00	-0.06	8.07E-01	9.75E-01
**GGN**	0.03	9.64E-01	1.00E+00	0.26	8.07E-01	9.75E-01
**FBXL19-AS1**	0.63	1.66E-01	1.00E+00	0.19	8.07E-01	9.75E-01
**ALKBH1**	-0.02	9.21E-01	1.00E+00	0.06	8.07E-01	9.75E-01
**ATP6V0A2**	0.26	4.72E-01	1.00E+00	-0.10	8.07E-01	9.75E-01
**WDR3**	-0.05	8.48E-01	1.00E+00	0.08	8.07E-01	9.75E-01
**XLOC_001527**	-1.06	1.97E-01	1.00E+00	-0.29	8.07E-01	9.75E-01
**FKBP5**	0.22	6.98E-01	1.00E+00	-0.11	8.07E-01	9.75E-01
**DCPS**	-0.23	2.53E-01	1.00E+00	-0.05	8.07E-01	9.75E-01
**SMAD6**	0.78	6.08E-02	1.00E+00	-0.77	8.07E-01	9.75E-01
**XLOC_009920**	0.14	9.67E-01	1.00E+00	0.35	8.07E-01	9.75E-01
**JPH1**	-0.13	7.66E-01	1.00E+00	-0.17	8.07E-01	9.75E-01
**AP4M1**	0.16	5.20E-01	1.00E+00	-0.05	8.07E-01	9.75E-01
**GORASP1**	0.12	5.47E-01	1.00E+00	-0.04	8.07E-01	9.75E-01
**IFI27L2**	-0.16	6.45E-01	1.00E+00	0.07	8.07E-01	9.75E-01
**PIK3R4**	-0.07	7.40E-01	1.00E+00	-0.06	8.07E-01	9.75E-01

**SLC16A2**	0.11	8.01E-01	1.00E+00	0.14	8.07E-01	9.75E-01
**AP000692.1**	1.98	1.80E-01	1.00E+00	0.25	8.07E-01	9.75E-01
**KCNK9**	-1.95	2.38E-01	1.00E+00	0.40	8.07E-01	9.75E-01
**NDE1**	-0.31	2.40E-01	1.00E+00	-0.09	8.07E-01	9.75E-01
**AC093890.1**	1.05	1.02E-01	1.00E+00	-0.23	8.07E-01	9.75E-01
**AC138305.3**	-0.49	8.39E-01	1.00E+00	-0.18	8.07E-01	9.75E-01
**MYORG**	0.15	6.41E-01	1.00E+00	0.12	8.08E-01	9.75E-01
**DROSHA**	0.24	2.30E-01	1.00E+00	0.06	8.08E-01	9.75E-01
**FAM126A**	0.03	9.19E-01	1.00E+00	-0.08	8.08E-01	9.75E-01
**OSER1**	0.12	5.45E-01	1.00E+00	-0.07	8.08E-01	9.75E-01
**KRT33B**	-17.78	6.49E-08	2.73E-05	0.77	8.08E-01	9.75E-01
**MYCBP2-AS1**	-2.15	4.21E-01	1.00E+00	-0.18	8.08E-01	9.75E-01
**PRSS16**	-0.12	7.19E-01	1.00E+00	-0.14	8.08E-01	9.75E-01
**TSPAN1**	0.08	8.58E-01	1.00E+00	0.10	8.08E-01	9.75E-01
**PHACTR2**	0.11	7.29E-01	1.00E+00	0.06	8.08E-01	9.75E-01
**CPB2-AS1**	0.47	6.24E-01	1.00E+00	0.20	8.08E-01	9.75E-01
**AC005865.2**	-1.02	4.83E-01	1.00E+00	0.21	8.08E-01	9.75E-01
**PRXL2C**	0.01	9.81E-01	1.00E+00	0.07	8.08E-01	9.75E-01
**KIAA0753**	0.00	9.88E-01	1.00E+00	-0.15	8.08E-01	9.75E-01
**RAP1GAP**	-0.55	3.10E-01	1.00E+00	-0.17	8.08E-01	9.75E-01
**AC048380.2**	3.26	1.85E-01	1.00E+00	-0.35	8.08E-01	9.75E-01
**TMEM9**	0.15	5.13E-01	1.00E+00	0.05	8.09E-01	9.76E-01
**AC106865.1**	0.04	9.77E-01	1.00E+00	0.77	8.09E-01	9.76E-01
**OGT**	0.06	9.02E-01	1.00E+00	-0.07	8.09E-01	9.76E-01
**NBPF2P**	-0.28	7.30E-01	1.00E+00	-0.12	8.09E-01	9.76E-01
**WDR75**	0.14	6.13E-01	1.00E+00	0.06	8.09E-01	9.76E-01
**AP000880.1**	-2.33	2.13E-01	1.00E+00	-0.25	8.09E-01	9.76E-01
**WWOX**	0.16	4.60E-01	1.00E+00	0.05	8.09E-01	9.76E-01
**PHKG1**	-0.18	6.99E-01	1.00E+00	0.13	8.09E-01	9.76E-01
**EXOC6B**	-0.06	8.44E-01	1.00E+00	-0.07	8.09E-01	9.76E-01
**RMC1**	0.13	5.38E-01	1.00E+00	-0.05	8.09E-01	9.76E-01
**TMEM208**	0.14	4.49E-01	1.00E+00	0.05	8.09E-01	9.76E-01

**ALS2**	-0.01	9.75E-01	1.00E+00	0.05	8.09E-01	9.76E-01
**ZNF18**	-0.15	5.28E-01	1.00E+00	-0.05	8.09E-01	9.76E-01
**RPS3AP6**	-0.22	5.73E-01	1.00E+00	-0.13	8.09E-01	9.76E-01
**AP1M1**	0.05	8.40E-01	1.00E+00	-0.05	8.09E-01	9.76E-01
**LRP8**	-0.99	2.15E-03	2.39E-01	-0.19	8.09E-01	9.76E-01
**AC146944.2**	-1.63	3.96E-01	1.00E+00	0.30	8.09E-01	9.76E-01
**TMEM223**	0.04	8.61E-01	1.00E+00	-0.07	8.09E-01	9.76E-01
**AP002383.3**	-0.74	7.30E-01	1.00E+00	-0.26	8.09E-01	9.76E-01
**XLOC_011210**	-1.06	7.00E-01	1.00E+00	-0.41	8.09E-01	9.76E-01
**LINC00323**	0.49	6.60E-01	1.00E+00	0.23	8.09E-01	9.76E-01
**KIN**	0.28	4.21E-01	1.00E+00	0.05	8.10E-01	9.76E-01
**C2CD2**	0.61	2.17E-02	8.94E-01	0.08	8.10E-01	9.76E-01
**LPCAT4**	0.18	5.03E-01	1.00E+00	0.09	8.10E-01	9.76E-01
**ZNF362**	-0.40	1.47E-01	1.00E+00	-0.18	8.10E-01	9.76E-01
**DSTYK**	0.28	2.66E-01	1.00E+00	0.07	8.10E-01	9.76E-01
**SUSD2**	-0.16	7.17E-01	1.00E+00	0.10	8.10E-01	9.76E-01
**TBC1D30**	0.18	7.84E-01	1.00E+00	0.20	8.10E-01	9.76E-01
**PCDHGB1**	-0.67	3.30E-01	1.00E+00	0.28	8.10E-01	9.76E-01
**SNORA40**	0.54	6.35E-01	1.00E+00	-0.20	8.10E-01	9.76E-01
**AC011978.2**	0.74	4.01E-01	1.00E+00	-0.15	8.10E-01	9.76E-01
**AC008669.1**	-0.25	5.90E-01	1.00E+00	-0.14	8.10E-01	9.76E-01
**LGALS17A**	-2.45	2.90E-01	1.00E+00	0.76	8.10E-01	9.76E-01
**XLOC_013174**	0.29	6.82E-01	1.00E+00	-0.17	8.10E-01	9.76E-01
**ZNF121**	0.10	8.02E-01	1.00E+00	-0.06	8.10E-01	9.76E-01
**SPRED2**	0.15	6.02E-01	1.00E+00	0.09	8.10E-01	9.76E-01
**AL355102.4**	0.56	8.71E-01	1.00E+00	0.34	8.10E-01	9.76E-01
**SPIRE2**	-0.04	9.45E-01	1.00E+00	-0.19	8.10E-01	9.76E-01
**HAO2**	0.96	6.15E-01	1.00E+00	-0.75	8.10E-01	9.76E-01
**AC040174.2**	-2.18	4.21E-01	1.00E+00	-0.29	8.11E-01	9.76E-01
**AC044810.2**	0.26	7.85E-01	1.00E+00	-0.17	8.11E-01	9.76E-01
**SBF2**	0.22	2.85E-01	1.00E+00	-0.10	8.11E-01	9.76E-01
**DOCK6**	0.05	8.60E-01	1.00E+00	0.12	8.11E-01	9.76E-01

**AC090204.1**	-0.15	8.88E-01	1.00E+00	0.18	8.11E-01	9.76E-01
**POLR3E**	-0.22	4.15E-01	1.00E+00	0.06	8.11E-01	9.76E-01
**VPS13D**	0.02	9.53E-01	1.00E+00	0.08	8.11E-01	9.76E-01
**AL512770.1**	-1.71	4.97E-01	1.00E+00	-0.25	8.11E-01	9.76E-01
**POLR2E**	-0.11	6.50E-01	1.00E+00	-0.05	8.11E-01	9.76E-01
**AC104118.1**	-0.71	6.76E-01	1.00E+00	0.76	8.11E-01	9.76E-01
**SPATA2**	-0.35	3.78E-02	1.00E+00	-0.08	8.11E-01	9.76E-01
**AC079922.1**	-1.28	4.96E-01	1.00E+00	0.24	8.11E-01	9.76E-01
**NIPBL**	-0.11	5.88E-01	1.00E+00	0.06	8.11E-01	9.76E-01
**SFXN1**	-0.21	3.79E-01	1.00E+00	0.05	8.11E-01	9.76E-01
**ZBTB12**	-0.61	8.84E-02	1.00E+00	0.76	8.11E-01	9.76E-01
**CAPN2**	0.00	9.94E-01	1.00E+00	0.10	8.11E-01	9.76E-01
**EEF1B2P6**	-0.81	3.08E-01	1.00E+00	0.15	8.11E-01	9.76E-01
**RAE1**	0.23	3.45E-01	1.00E+00	-0.07	8.11E-01	9.76E-01
**AC093278.2**	0.06	9.23E-01	1.00E+00	0.19	8.11E-01	9.76E-01
**MCOLN3**	0.53	1.90E-01	1.00E+00	0.12	8.11E-01	9.76E-01
**AC069549.1**	-0.97	7.73E-01	1.00E+00	0.22	8.11E-01	9.76E-01
**KLF2**	-0.76	2.90E-02	9.88E-01	0.18	8.11E-01	9.76E-01
**ATOX1**	-0.01	9.84E-01	1.00E+00	0.05	8.11E-01	9.76E-01
**Z83851.1**	0.22	7.39E-01	1.00E+00	-0.20	8.11E-01	9.76E-01
**STAC2**	0.23	8.36E-01	1.00E+00	-0.17	8.11E-01	9.76E-01
**XLOC_006072**	-0.27	8.25E-01	1.00E+00	0.31	8.11E-01	9.76E-01
**TM9SF2**	0.15	3.19E-01	1.00E+00	-0.05	8.11E-01	9.76E-01
**LIPA**	0.23	4.27E-01	1.00E+00	-0.12	8.11E-01	9.76E-01
**PACRG**	0.04	9.07E-01	1.00E+00	-0.12	8.12E-01	9.76E-01
**TCF15**	0.13	8.98E-01	1.00E+00	-0.27	8.12E-01	9.76E-01
**ANOS1**	-0.67	6.09E-02	1.00E+00	-0.11	8.12E-01	9.76E-01
**FIRRE**	-1.19	6.76E-01	1.00E+00	-0.31	8.12E-01	9.76E-01
**AC004706.1**	-0.87	3.09E-01	1.00E+00	-0.19	8.12E-01	9.76E-01
**CA13**	0.26	4.16E-01	1.00E+00	-0.10	8.12E-01	9.76E-01
**AC008592.5**	-0.45	4.20E-01	1.00E+00	-0.15	8.12E-01	9.76E-01
**IDH3B**	-0.15	4.33E-01	1.00E+00	-0.03	8.12E-01	9.76E-01

**CDC25A**	-0.31	4.04E-01	1.00E+00	0.21	8.12E-01	9.76E-01
**HES4**	-0.69	9.11E-03	5.95E-01	-0.74	8.12E-01	9.76E-01
**CRSP8P**	0.48	8.88E-01	1.00E+00	-0.14	8.12E-01	9.76E-01
**XLOC_009026**	-1.28	5.08E-01	1.00E+00	-0.74	8.12E-01	9.76E-01
**HNMT**	0.47	2.67E-01	1.00E+00	-0.10	8.12E-01	9.76E-01
**AC096992.2**	-0.20	7.64E-01	1.00E+00	0.18	8.12E-01	9.76E-01
**LTK**	-0.43	4.76E-01	1.00E+00	0.27	8.12E-01	9.76E-01
**TULP1**	0.11	9.04E-01	1.00E+00	0.15	8.12E-01	9.76E-01
**RASL11B**	0.32	5.11E-01	1.00E+00	-0.15	8.12E-01	9.76E-01
**CCNT2**	0.07	7.98E-01	1.00E+00	-0.06	8.12E-01	9.76E-01
**BDH1**	-0.49	1.82E-01	1.00E+00	0.08	8.13E-01	9.76E-01
**AC005537.1**	-0.74	3.48E-01	1.00E+00	0.18	8.13E-01	9.76E-01
**TMEM179**	-4.66	9.22E-05	2.32E-02	0.42	8.13E-01	9.76E-01
**DNAJC24**	0.02	9.58E-01	1.00E+00	-0.04	8.13E-01	9.76E-01
**DCTN1**	-0.15	5.03E-01	1.00E+00	0.10	8.13E-01	9.76E-01
**AL359643.2**	0.74	2.81E-01	1.00E+00	-0.22	8.13E-01	9.76E-01
**B3GNT4**	0.14	7.37E-01	1.00E+00	-0.14	8.13E-01	9.76E-01
**ENPP2**	0.49	1.59E-01	1.00E+00	-0.12	8.13E-01	9.76E-01
**CXCL17**	-0.04	9.68E-01	1.00E+00	-0.64	8.13E-01	9.76E-01
**SURF2**	-0.10	6.98E-01	1.00E+00	-0.07	8.13E-01	9.76E-01
**AC005829.1**	-0.62	4.90E-01	1.00E+00	0.16	8.13E-01	9.76E-01
**BORCS6**	-0.14	5.61E-01	1.00E+00	-0.05	8.13E-01	9.76E-01
**AC116533.1**	-0.22	5.69E-01	1.00E+00	-0.11	8.13E-01	9.76E-01
**SNHG3**	-0.32	3.44E-01	1.00E+00	-0.09	8.13E-01	9.76E-01
**XLOC_011052**	-2.22	5.19E-01	1.00E+00	-0.35	8.13E-01	9.76E-01
**PRICKLE2**	-0.14	7.76E-01	1.00E+00	-0.06	8.13E-01	9.76E-01
**LRIG1**	0.11	7.65E-01	1.00E+00	-0.12	8.13E-01	9.76E-01
**OTUD3**	0.12	7.38E-01	1.00E+00	-0.10	8.13E-01	9.76E-01
**UQCRH**	0.04	8.56E-01	1.00E+00	-0.04	8.13E-01	9.76E-01
**VASH2**	-0.13	8.44E-01	1.00E+00	0.15	8.13E-01	9.76E-01
**MIR100HG**	0.68	1.12E-01	1.00E+00	0.09	8.13E-01	9.76E-01
**AL049872.1**	-0.96	7.80E-01	1.00E+00	-0.16	8.14E-01	9.76E-01

**AL121655.1**	-1.03	6.75E-01	1.00E+00	0.19	8.14E-01	9.76E-01
**RNF150**	0.70	2.65E-01	1.00E+00	0.14	8.14E-01	9.76E-01
**NAP1L5**	0.04	9.24E-01	1.00E+00	-0.10	8.14E-01	9.76E-01
**CLTCL1**	0.27	4.01E-01	1.00E+00	0.13	8.14E-01	9.76E-01
**PRKG2**	0.81	2.35E-01	1.00E+00	0.14	8.14E-01	9.76E-01
**COL4A2-AS2**	NA	NA	NA	0.76	8.14E-01	9.76E-01
**RARB**	-0.22	7.10E-01	1.00E+00	-0.14	8.14E-01	9.76E-01
**EBF2**	0.71	2.80E-01	1.00E+00	0.14	8.14E-01	9.76E-01
**OCA2**	0.05	9.11E-01	1.00E+00	0.13	8.14E-01	9.76E-01
**ZFC3H1**	-0.28	3.03E-01	1.00E+00	0.07	8.14E-01	9.76E-01
**ATF7**	-0.08	6.74E-01	1.00E+00	-0.04	8.14E-01	9.76E-01
**TRIM55**	2.27	2.68E-01	1.00E+00	-0.26	8.14E-01	9.76E-01
**GGACT**	-0.21	5.23E-01	1.00E+00	0.08	8.14E-01	9.76E-01
**EHD2**	0.17	6.93E-01	1.00E+00	-0.12	8.14E-01	9.76E-01
**S1PR1**	0.29	5.76E-01	1.00E+00	-0.12	8.14E-01	9.76E-01
**PPOX**	-0.07	7.76E-01	1.00E+00	-0.06	8.14E-01	9.76E-01
**CFDP1**	0.38	1.05E-01	1.00E+00	-0.04	8.14E-01	9.76E-01
**ZNF285**	1.15	4.36E-02	1.00E+00	0.25	8.14E-01	9.76E-01
**NME4**	0.13	7.72E-01	1.00E+00	-0.08	8.14E-01	9.76E-01
**TRMT1L**	0.58	4.96E-02	1.00E+00	0.06	8.14E-01	9.76E-01
**LY6G6D**	-2.36	2.14E-01	1.00E+00	0.42	8.14E-01	9.76E-01
**TRIM31**	0.19	7.33E-01	1.00E+00	-0.14	8.15E-01	9.76E-01
**SPEG**	-0.43	5.18E-01	1.00E+00	-0.19	8.15E-01	9.76E-01
**AL157392.3**	0.34	4.40E-01	1.00E+00	-0.10	8.15E-01	9.76E-01
**ASTN1**	-1.38	5.22E-02	1.00E+00	-0.28	8.15E-01	9.76E-01
**AL359922.3**	3.04	2.19E-02	8.96E-01	-0.19	8.15E-01	9.76E-01
**G36957**	-0.45	8.13E-01	1.00E+00	0.50	8.15E-01	9.76E-01
**KRT23**	0.18	7.32E-01	1.00E+00	0.13	8.15E-01	9.76E-01
**PXDC1**	0.44	2.18E-01	1.00E+00	-0.12	8.15E-01	9.76E-01
**AL139246.5**	0.06	8.64E-01	1.00E+00	0.24	8.15E-01	9.76E-01
**SEPSECS**	0.39	1.08E-01	1.00E+00	-0.08	8.15E-01	9.76E-01
**MTMR4**	-0.04	8.61E-01	1.00E+00	-0.07	8.15E-01	9.76E-01

**RNF168**	0.09	7.19E-01	1.00E+00	-0.07	8.15E-01	9.76E-01
**XLOC_008190**	-1.07	4.79E-01	1.00E+00	-0.27	8.15E-01	9.76E-01
**LRP4**	0.20	5.38E-01	1.00E+00	-0.09	8.15E-01	9.76E-01
**FAM13C**	0.21	5.49E-01	1.00E+00	-0.09	8.15E-01	9.76E-01
**AC022182.3**	-2.19	5.21E-01	1.00E+00	0.19	8.15E-01	9.76E-01
**CLCA4**	0.19	7.52E-01	1.00E+00	-0.17	8.15E-01	9.76E-01
**RBM15B**	-0.27	2.56E-01	1.00E+00	0.10	8.15E-01	9.76E-01
**KRTAP12-2**	-30.00	1.37E-18	5.54E-15	-0.75	8.15E-01	9.76E-01
**ZNF214**	0.87	2.19E-01	1.00E+00	0.15	8.15E-01	9.76E-01
**PIGM**	0.28	3.05E-01	1.00E+00	0.06	8.15E-01	9.76E-01
**TNFSF11**	-3.81	1.46E-02	7.60E-01	-0.74	8.15E-01	9.76E-01
**IFT80**	-0.29	4.91E-01	1.00E+00	0.07	8.15E-01	9.76E-01
**BOC**	-0.25	4.89E-01	1.00E+00	-0.09	8.15E-01	9.76E-01
**SHISA8**	-1.64	2.89E-02	9.88E-01	0.33	8.15E-01	9.76E-01
**LINC02301**	-0.37	8.20E-01	1.00E+00	-0.28	8.15E-01	9.76E-01
**THBS4**	1.33	2.54E-01	1.00E+00	-0.18	8.15E-01	9.76E-01
**SIRT2**	-0.16	4.71E-01	1.00E+00	0.05	8.16E-01	9.76E-01
**SNRNP200**	-0.15	4.98E-01	1.00E+00	0.09	8.16E-01	9.76E-01
**SETDB2**	0.03	8.82E-01	1.00E+00	0.06	8.16E-01	9.77E-01
**KMT5C**	-0.43	1.40E-01	1.00E+00	0.18	8.16E-01	9.77E-01
**SOS1-IT1**	1.38	2.84E-01	1.00E+00	-0.58	8.16E-01	9.77E-01
**CXorf36**	0.14	7.98E-01	1.00E+00	0.16	8.16E-01	9.77E-01
**SETD7**	0.44	9.14E-02	1.00E+00	0.06	8.16E-01	9.77E-01
**BICDL2**	-0.37	4.16E-01	1.00E+00	0.14	8.16E-01	9.77E-01
**USH1G**	-0.26	7.00E-01	1.00E+00	-0.18	8.16E-01	9.77E-01
**SMAD9**	-0.14	6.74E-01	1.00E+00	0.11	8.16E-01	9.77E-01
**MIR205HG**	-0.34	2.54E-01	1.00E+00	-0.16	8.16E-01	9.77E-01
**GAPDHS**	-0.27	9.03E-01	1.00E+00	-0.20	8.16E-01	9.77E-01
**ATP6V1H**	0.20	2.83E-01	1.00E+00	0.03	8.16E-01	9.77E-01
**EZH1**	0.49	1.64E-01	1.00E+00	-0.08	8.16E-01	9.77E-01
**AC135050.1**	-0.76	2.59E-01	1.00E+00	0.15	8.16E-01	9.77E-01
**PIGU**	-0.07	7.17E-01	1.00E+00	0.04	8.16E-01	9.77E-01

**LINC00165**	4.20	1.31E-03	1.95E-01	-0.35	8.16E-01	9.77E-01
**AC022400.5**	2.55	6.99E-02	1.00E+00	0.25	8.16E-01	9.77E-01
**FTH1P10**	0.09	9.64E-01	1.00E+00	-0.19	8.16E-01	9.77E-01
**LINC02172**	1.62	5.51E-01	1.00E+00	-0.26	8.16E-01	9.77E-01
**CRKL**	0.14	5.00E-01	1.00E+00	-0.05	8.17E-01	9.77E-01
**AP001381.1**	1.08	5.09E-01	1.00E+00	0.31	8.17E-01	9.77E-01
**PWAR5**	0.18	6.68E-01	1.00E+00	-0.17	8.17E-01	9.77E-01
**RPL10AP2**	0.03	9.85E-01	1.00E+00	0.18	8.17E-01	9.77E-01
**CNGA3**	-2.85	1.54E-01	1.00E+00	0.74	8.17E-01	9.77E-01
**PSMB8-AS1**	0.19	6.05E-01	1.00E+00	-0.08	8.17E-01	9.77E-01
**RPS25**	-0.11	7.05E-01	1.00E+00	-0.06	8.17E-01	9.77E-01
**KRTAP5-11**	-6.46	6.36E-02	1.00E+00	-0.74	8.17E-01	9.77E-01
**G10021**	1.06	4.03E-01	1.00E+00	0.18	8.17E-01	9.77E-01
**RBP1**	-0.41	2.14E-01	1.00E+00	0.09	8.17E-01	9.77E-01
**PRX**	-0.14	7.39E-01	1.00E+00	0.72	8.17E-01	9.77E-01
**KDM4D**	0.19	5.70E-01	1.00E+00	-0.13	8.17E-01	9.77E-01
**LINC00886**	0.31	4.96E-01	1.00E+00	0.12	8.17E-01	9.77E-01
**RRP15**	0.19	3.95E-01	1.00E+00	-0.05	8.17E-01	9.77E-01
**DISP1**	0.42	1.65E-01	1.00E+00	-0.13	8.17E-01	9.77E-01
**AC027288.3**	1.18	2.11E-01	1.00E+00	-0.20	8.17E-01	9.77E-01
**RHOB**	-0.65	7.91E-02	1.00E+00	0.13	8.17E-01	9.77E-01
**TRNT1**	0.56	8.08E-02	1.00E+00	0.07	8.18E-01	9.77E-01
**ZNF700**	0.01	9.73E-01	1.00E+00	0.07	8.18E-01	9.77E-01
**AC039056.2**	-2.67	7.83E-02	1.00E+00	-0.26	8.18E-01	9.77E-01
**UBE2D4**	0.17	6.34E-01	1.00E+00	-0.09	8.18E-01	9.77E-01
**PTTG1**	-0.02	9.63E-01	1.00E+00	0.11	8.18E-01	9.77E-01
**POU5F1B**	0.01	9.92E-01	1.00E+00	-0.23	8.18E-01	9.77E-01
**SMUG1**	0.15	5.80E-01	1.00E+00	-0.06	8.18E-01	9.77E-01
**G35913**	-1.42	2.34E-01	1.00E+00	-0.28	8.18E-01	9.77E-01
**GCKR**	0.30	9.27E-01	1.00E+00	-0.38	8.18E-01	9.77E-01
**TRPM7**	-0.14	5.97E-01	1.00E+00	-0.06	8.18E-01	9.77E-01
**PCDHGB2**	-0.35	3.40E-01	1.00E+00	0.18	8.18E-01	9.77E-01

**G35786**	-0.73	6.36E-01	1.00E+00	0.22	8.18E-01	9.77E-01
**LIMD1-AS1**	-2.08	5.41E-01	1.00E+00	-0.23	8.18E-01	9.77E-01
**GALNT13**	0.68	4.73E-01	1.00E+00	0.50	8.18E-01	9.77E-01
**LINC01393**	-0.67	8.48E-01	1.00E+00	0.32	8.18E-01	9.77E-01
**AL109615.3**	0.89	6.85E-01	1.00E+00	-0.22	8.18E-01	9.77E-01
**AC105285.1**	0.56	6.26E-01	1.00E+00	-0.21	8.18E-01	9.77E-01
**AC120114.3**	-1.07	5.71E-01	1.00E+00	0.24	8.18E-01	9.77E-01
**SASH1**	0.02	8.92E-01	1.00E+00	-0.06	8.18E-01	9.77E-01
**FAM24B**	-1.10	5.79E-01	1.00E+00	0.22	8.18E-01	9.77E-01
**AC009133.1**	-0.52	1.92E-01	1.00E+00	0.15	8.19E-01	9.77E-01
**AC087385.1**	-1.39	6.23E-01	1.00E+00	0.21	8.19E-01	9.77E-01
**SLC41A3**	0.05	8.44E-01	1.00E+00	0.08	8.19E-01	9.77E-01
**CR392039.4**	-1.26	1.84E-01	1.00E+00	-0.25	8.19E-01	9.77E-01
**KDM5A**	0.10	6.64E-01	1.00E+00	-0.05	8.19E-01	9.77E-01
**AC083843.2**	0.73	5.06E-01	1.00E+00	-0.28	8.19E-01	9.77E-01
**ZXDA**	-0.16	4.53E-01	1.00E+00	-0.15	8.19E-01	9.77E-01
**NDUFA9P1**	0.54	5.80E-01	1.00E+00	0.16	8.19E-01	9.77E-01
**GSTM3**	0.55	7.18E-02	1.00E+00	-0.12	8.19E-01	9.77E-01
**CCDC191**	-0.09	7.46E-01	1.00E+00	-0.08	8.19E-01	9.77E-01
**VWA3A**	-0.90	3.11E-01	1.00E+00	0.16	8.19E-01	9.77E-01
**ARIH2OS**	0.07	8.87E-01	1.00E+00	0.10	8.19E-01	9.77E-01
**SHISA6**	0.84	1.12E-01	1.00E+00	0.72	8.19E-01	9.77E-01
**AC022390.1**	1.80	3.52E-01	1.00E+00	0.27	8.19E-01	9.77E-01
**PLA2G4A**	0.35	1.95E-01	1.00E+00	-0.06	8.19E-01	9.77E-01
**LAMP2**	0.02	9.12E-01	1.00E+00	-0.05	8.19E-01	9.77E-01
**ZMAT4**	-0.57	7.30E-01	1.00E+00	0.35	8.19E-01	9.77E-01
**AC027097.2**	-0.08	8.50E-01	1.00E+00	-0.15	8.19E-01	9.77E-01
**CNTNAP3B**	1.06	2.06E-01	1.00E+00	0.12	8.19E-01	9.77E-01
**TPSD1**	1.90	1.27E-01	1.00E+00	-0.30	8.19E-01	9.77E-01
**KANTR**	0.07	8.59E-01	1.00E+00	0.12	8.19E-01	9.77E-01
**BTBD3**	0.22	3.44E-01	1.00E+00	0.05	8.19E-01	9.77E-01
**NKAP**	0.03	8.73E-01	1.00E+00	-0.06	8.19E-01	9.77E-01

**TTK**	0.08	8.17E-01	1.00E+00	0.11	8.19E-01	9.77E-01
**NAA40**	0.21	5.92E-01	1.00E+00	0.10	8.19E-01	9.77E-01
**GDPD5**	0.23	7.00E-01	1.00E+00	0.16	8.19E-01	9.77E-01
**ZSCAN1**	0.63	6.43E-01	1.00E+00	0.28	8.19E-01	9.77E-01
**LINC01559**	NA	NA	NA	0.73	8.19E-01	9.77E-01
**XLOC_002943**	0.02	9.79E-01	1.00E+00	-0.17	8.19E-01	9.77E-01
**ADAMTS8**	-0.12	7.87E-01	1.00E+00	-0.72	8.20E-01	9.77E-01
**POGK**	-0.28	2.49E-01	1.00E+00	-0.06	8.20E-01	9.77E-01
**CHCHD2**	-0.10	5.47E-01	1.00E+00	-0.03	8.20E-01	9.77E-01
**SHISA4**	-0.24	4.60E-01	1.00E+00	-0.08	8.20E-01	9.77E-01
**AL390719.2**	0.68	4.36E-01	1.00E+00	0.26	8.20E-01	9.77E-01
**ACY1**	-1.26	5.28E-01	1.00E+00	0.20	8.20E-01	9.77E-01
**ZSWIM5**	-0.37	2.71E-01	1.00E+00	0.16	8.20E-01	9.77E-01
**AC073321.1**	NA	NA	NA	0.26	8.20E-01	9.77E-01
**MRPS28**	0.28	5.61E-01	1.00E+00	0.12	8.20E-01	9.77E-01
**AC092652.3**	-2.53	3.48E-01	1.00E+00	0.28	8.20E-01	9.77E-01
**FASN**	0.57	3.97E-01	1.00E+00	-0.15	8.20E-01	9.77E-01
**UPF3B**	0.09	7.38E-01	1.00E+00	-0.05	8.20E-01	9.77E-01
**LRIT2**	0.70	4.44E-01	1.00E+00	0.21	8.20E-01	9.77E-01
**POLA1**	-0.22	2.98E-01	1.00E+00	0.07	8.20E-01	9.77E-01
**MSN**	-0.17	4.26E-01	1.00E+00	0.09	8.20E-01	9.77E-01
**GSTM5**	1.40	2.10E-03	2.39E-01	0.12	8.20E-01	9.77E-01
**PMF1**	0.16	6.69E-01	1.00E+00	-0.09	8.20E-01	9.77E-01
**MIR4453HG**	-0.30	2.94E-01	1.00E+00	-0.11	8.20E-01	9.77E-01
**SATB2-AS1**	-0.76	2.99E-01	1.00E+00	0.11	8.21E-01	9.77E-01
**CLEC16A**	-0.27	3.36E-01	1.00E+00	0.10	8.21E-01	9.77E-01
**MITD1**	-0.03	8.99E-01	1.00E+00	-0.04	8.21E-01	9.77E-01
**MIS18BP1**	-0.08	7.63E-01	1.00E+00	-0.05	8.21E-01	9.77E-01
**AL133406.2**	-1.68	4.99E-01	1.00E+00	-0.39	8.21E-01	9.77E-01
**XLOC_000895**	-1.10	2.38E-01	1.00E+00	0.25	8.21E-01	9.77E-01
**SF3A3**	-0.05	7.42E-01	1.00E+00	-0.03	8.21E-01	9.77E-01
**ISLR2**	-0.33	6.27E-01	1.00E+00	0.22	8.21E-01	9.77E-01

**DENND6A**	-0.19	4.86E-01	1.00E+00	0.12	8.21E-01	9.77E-01
**RBAK**	-0.03	9.20E-01	1.00E+00	-0.09	8.21E-01	9.77E-01
**GYG2P1**	-0.91	6.59E-01	1.00E+00	0.51	8.21E-01	9.77E-01
**MRVI1-AS1**	-0.41	8.01E-01	1.00E+00	-0.26	8.21E-01	9.78E-01
**C17orf82**	-1.05	3.62E-01	1.00E+00	-0.26	8.21E-01	9.78E-01
**FLYWCH1**	-0.24	1.81E-01	1.00E+00	0.14	8.21E-01	9.78E-01
**BMS1P11**	-0.23	9.02E-01	1.00E+00	-0.15	8.21E-01	9.78E-01
**PTPN20A**	-1.90	1.25E-01	1.00E+00	-0.14	8.21E-01	9.78E-01
**GDE1**	0.03	8.98E-01	1.00E+00	0.05	8.21E-01	9.78E-01
**GJA5**	0.81	2.87E-01	1.00E+00	0.20	8.22E-01	9.78E-01
**XLOC_007512**	-0.17	8.85E-01	1.00E+00	0.69	8.22E-01	9.78E-01
**LYPLA2P2**	NA	NA	NA	0.15	8.22E-01	9.78E-01
**USP21**	0.14	5.49E-01	1.00E+00	-0.04	8.22E-01	9.78E-01
**FBXL14**	-0.13	5.68E-01	1.00E+00	0.20	8.22E-01	9.78E-01
**GJB5**	0.12	7.84E-01	1.00E+00	0.14	8.22E-01	9.78E-01
**TUFT1**	0.03	9.38E-01	1.00E+00	-0.08	8.22E-01	9.78E-01
**RBM6**	-0.24	2.61E-01	1.00E+00	0.05	8.22E-01	9.78E-01
**MAN2C1**	-0.15	5.33E-01	1.00E+00	0.09	8.22E-01	9.78E-01
**OPTN**	0.16	4.94E-01	1.00E+00	-0.03	8.22E-01	9.78E-01
**MEG8**	-0.11	8.79E-01	1.00E+00	-0.19	8.22E-01	9.78E-01
**RAD51D**	0.20	5.78E-01	1.00E+00	-0.08	8.22E-01	9.78E-01
**SQLE**	-0.38	4.13E-01	1.00E+00	0.08	8.22E-01	9.78E-01
**AL136419.1**	1.58	5.55E-01	1.00E+00	0.25	8.22E-01	9.78E-01
**CCDC42**	-1.04	7.61E-01	1.00E+00	-0.37	8.22E-01	9.78E-01
**KRTAP10-8**	-30.00	3.42E-18	5.54E-15	-0.72	8.22E-01	9.78E-01
**C1QTNF2**	0.43	1.80E-01	1.00E+00	-0.09	8.22E-01	9.78E-01
**HNRNPA2B1**	-0.12	5.15E-01	1.00E+00	-0.06	8.22E-01	9.78E-01
**ERC2**	-4.71	3.49E-05	9.61E-03	-0.32	8.23E-01	9.78E-01
**AC106820.4**	-0.34	7.67E-01	1.00E+00	0.34	8.23E-01	9.78E-01
**SMTN**	-0.25	5.79E-01	1.00E+00	0.11	8.23E-01	9.78E-01
**NPAS2**	0.02	9.49E-01	1.00E+00	-0.11	8.23E-01	9.78E-01
**TRUB1**	0.14	5.83E-01	1.00E+00	-0.05	8.23E-01	9.78E-01

**RAB2B**	0.18	4.89E-01	1.00E+00	0.07	8.23E-01	9.78E-01
**PAICS**	-0.12	6.15E-01	1.00E+00	0.05	8.23E-01	9.78E-01
**MAPK14**	0.42	7.11E-02	1.00E+00	-0.09	8.23E-01	9.78E-01
**G41381**	0.05	9.62E-01	1.00E+00	-0.27	8.23E-01	9.78E-01
**TP53BP1**	-0.24	3.03E-01	1.00E+00	0.10	8.23E-01	9.78E-01
**FGF7P8**	-1.44	2.49E-01	1.00E+00	0.11	8.23E-01	9.78E-01
**GLO1**	0.13	5.77E-01	1.00E+00	-0.07	8.23E-01	9.78E-01
**MEF2A**	-0.08	7.26E-01	1.00E+00	-0.04	8.23E-01	9.78E-01
**AL161910.1**	1.54	1.34E-01	1.00E+00	-0.31	8.23E-01	9.78E-01
**LINC01220**	1.91	1.00E-01	1.00E+00	-0.24	8.23E-01	9.78E-01
**ABHD5**	0.21	5.70E-01	1.00E+00	-0.06	8.23E-01	9.78E-01
**AC008280.3**	-0.42	6.36E-01	1.00E+00	-0.14	8.23E-01	9.78E-01
**SMIM20**	0.06	8.63E-01	1.00E+00	-0.06	8.23E-01	9.78E-01
**HARBI1**	-0.01	9.66E-01	1.00E+00	0.07	8.23E-01	9.78E-01
**CHL1-AS1**	0.19	9.06E-01	1.00E+00	-0.18	8.23E-01	9.78E-01
**STRN**	0.22	4.50E-01	1.00E+00	-0.11	8.23E-01	9.78E-01
**KLHL8**	0.36	9.38E-02	1.00E+00	0.04	8.23E-01	9.78E-01
**FRG1BP**	-0.02	9.38E-01	1.00E+00	0.06	8.24E-01	9.78E-01
**ROCK2**	-0.05	8.46E-01	1.00E+00	-0.09	8.24E-01	9.78E-01
**KLHL3**	0.17	7.55E-01	1.00E+00	-0.13	8.24E-01	9.78E-01
**AL078644.1**	2.13	2.43E-01	1.00E+00	-0.25	8.24E-01	9.78E-01
**E2F8**	-0.48	3.14E-01	1.00E+00	0.18	8.24E-01	9.78E-01
**AC024940.2**	1.86	2.95E-01	1.00E+00	-0.15	8.24E-01	9.78E-01
**PTRH1**	-1.43	3.85E-01	1.00E+00	0.18	8.24E-01	9.78E-01
**PDZK1**	0.66	3.48E-01	1.00E+00	-0.20	8.24E-01	9.78E-01
**LRRC4B**	-0.41	4.24E-01	1.00E+00	0.70	8.24E-01	9.78E-01
**CDC25C**	-0.02	9.76E-01	1.00E+00	0.17	8.24E-01	9.78E-01
**NME6**	0.16	4.15E-01	1.00E+00	0.05	8.24E-01	9.78E-01
**BDH2**	0.20	4.86E-01	1.00E+00	0.07	8.24E-01	9.78E-01
**ALDOB**	3.20	1.66E-01	1.00E+00	-0.28	8.24E-01	9.78E-01
**AC007490.1**	0.07	9.72E-01	1.00E+00	0.27	8.24E-01	9.78E-01
**AC092484.1**	-2.71	1.02E-01	1.00E+00	0.19	8.24E-01	9.78E-01

**ENPEP**	0.08	8.80E-01	1.00E+00	0.15	8.24E-01	9.78E-01
**ALG10B**	0.39	3.41E-01	1.00E+00	-0.06	8.24E-01	9.78E-01
**SNX9**	0.12	5.78E-01	1.00E+00	-0.09	8.24E-01	9.78E-01
**EFNA1**	-0.26	4.34E-01	1.00E+00	-0.08	8.24E-01	9.78E-01
**UBE2J1**	-0.05	8.03E-01	1.00E+00	0.06	8.24E-01	9.78E-01
**FAM193B**	-0.41	1.85E-01	1.00E+00	0.12	8.25E-01	9.78E-01
**PCARE**	0.44	3.79E-01	1.00E+00	-0.10	8.25E-01	9.78E-01
**TMEM263**	0.50	1.27E-01	1.00E+00	0.08	8.25E-01	9.78E-01
**MEA1**	-0.02	9.21E-01	1.00E+00	0.05	8.25E-01	9.78E-01
**FAM107A**	0.43	2.68E-01	1.00E+00	0.11	8.25E-01	9.78E-01
**NIF3L1**	0.10	5.55E-01	1.00E+00	-0.07	8.25E-01	9.78E-01
**MFAP5**	0.50	4.96E-01	1.00E+00	-0.14	8.25E-01	9.78E-01
**VAMP8**	0.14	6.12E-01	1.00E+00	-0.08	8.25E-01	9.78E-01
**AL450332.1**	-2.69	1.37E-01	1.00E+00	0.23	8.25E-01	9.78E-01
**PIGR**	2.03	2.49E-01	1.00E+00	-0.70	8.25E-01	9.78E-01
**SVIL**	-0.07	8.15E-01	1.00E+00	-0.07	8.25E-01	9.78E-01
**HNRNPA1P1**	-0.06	9.86E-01	1.00E+00	-0.22	8.25E-01	9.78E-01
**ATP6V0E1**	0.08	6.13E-01	1.00E+00	0.05	8.25E-01	9.78E-01
**RWDD2A**	-0.16	5.77E-01	1.00E+00	-0.08	8.25E-01	9.78E-01
**AC104024.2**	-0.70	5.24E-01	1.00E+00	-0.30	8.25E-01	9.78E-01
**RSPH3**	-0.23	2.36E-01	1.00E+00	0.04	8.25E-01	9.78E-01
**MCAT**	-0.08	7.38E-01	1.00E+00	0.09	8.25E-01	9.78E-01
**FAM86B3P**	0.00	9.95E-01	1.00E+00	0.15	8.25E-01	9.78E-01
**Z95704.5**	-1.03	5.57E-01	1.00E+00	-0.22	8.25E-01	9.78E-01
**AC012676.1**	0.83	4.10E-01	1.00E+00	0.29	8.25E-01	9.78E-01
**XLOC_005818**	2.75	1.33E-01	1.00E+00	-0.25	8.25E-01	9.78E-01
**RPL9P9**	-0.27	6.65E-01	1.00E+00	-0.19	8.25E-01	9.78E-01
**STX6**	0.01	9.51E-01	1.00E+00	-0.09	8.25E-01	9.78E-01
**MSS51**	1.09	2.92E-01	1.00E+00	0.27	8.25E-01	9.78E-01
**ZNF799**	0.29	5.07E-01	1.00E+00	-0.07	8.25E-01	9.78E-01
**ZFP82**	0.46	4.70E-01	1.00E+00	0.09	8.25E-01	9.78E-01
**KIAA1549**	-0.02	9.66E-01	1.00E+00	0.15	8.25E-01	9.78E-01

**VPS25**	-0.18	3.75E-01	1.00E+00	0.04	8.25E-01	9.78E-01
**AL020997.1**	-2.85	1.43E-01	1.00E+00	-0.20	8.26E-01	9.78E-01
**XLOC_014066**	0.92	7.33E-01	1.00E+00	-0.23	8.26E-01	9.78E-01
**AC008957.3**	0.01	9.96E-01	1.00E+00	0.38	8.26E-01	9.78E-01
**CALU**	0.01	9.70E-01	1.00E+00	0.07	8.26E-01	9.78E-01
**UBE2R2**	-0.06	7.54E-01	1.00E+00	0.07	8.26E-01	9.78E-01
**RBFADN**	0.69	5.11E-01	1.00E+00	-0.18	8.26E-01	9.78E-01
**SPSB2**	-0.23	3.47E-01	1.00E+00	-0.07	8.26E-01	9.78E-01
**PRDM16**	-0.16	8.06E-01	1.00E+00	0.19	8.26E-01	9.78E-01
**AL137003.2**	0.66	2.13E-01	1.00E+00	0.12	8.26E-01	9.78E-01
**DMKN**	0.07	8.94E-01	1.00E+00	0.14	8.26E-01	9.78E-01
**AXDND1**	-1.35	5.75E-01	1.00E+00	0.29	8.26E-01	9.78E-01
**AC012531.1**	-0.30	8.66E-01	1.00E+00	0.30	8.26E-01	9.78E-01
**PGPEP1L**	-1.27	6.74E-01	1.00E+00	0.26	8.26E-01	9.78E-01
**BCL2L14**	-1.54	1.44E-01	1.00E+00	0.69	8.26E-01	9.78E-01
**KCNK1**	-0.25	6.44E-01	1.00E+00	0.12	8.26E-01	9.78E-01
**AL136126.1**	0.90	7.96E-01	1.00E+00	-0.19	8.26E-01	9.78E-01
**DYNC1LI2**	0.04	8.69E-01	1.00E+00	-0.05	8.26E-01	9.78E-01
**XLOC_001343**	-0.06	9.03E-01	1.00E+00	-0.11	8.26E-01	9.78E-01
**BMP8B**	-0.37	1.37E-01	1.00E+00	-0.17	8.26E-01	9.78E-01
**IVNS1ABP**	0.04	8.48E-01	1.00E+00	-0.05	8.26E-01	9.78E-01
**FPGT**	0.27	5.18E-01	1.00E+00	-0.09	8.26E-01	9.78E-01
**G31075**	-2.45	4.63E-02	1.00E+00	0.28	8.26E-01	9.78E-01
**LMO4**	-0.08	7.52E-01	1.00E+00	-0.04	8.26E-01	9.78E-01
**AC069213.1**	-0.85	5.05E-01	1.00E+00	0.21	8.26E-01	9.78E-01
**CADM3-AS1**	0.12	8.98E-01	1.00E+00	-0.23	8.27E-01	9.78E-01
**MAT1A**	-0.78	1.29E-01	1.00E+00	-0.17	8.27E-01	9.78E-01
**NIPA2**	0.07	6.68E-01	1.00E+00	0.04	8.27E-01	9.78E-01
**G39662**	1.21	3.68E-01	1.00E+00	-0.17	8.27E-01	9.78E-01
**MRPL45P2**	-1.24	6.10E-02	1.00E+00	-0.08	8.27E-01	9.78E-01
**AC007405.3**	0.74	3.85E-01	1.00E+00	-0.16	8.27E-01	9.78E-01
**AC118553.1**	0.07	9.48E-01	1.00E+00	-0.19	8.27E-01	9.78E-01

**G23907**	1.58	1.22E-01	1.00E+00	-0.23	8.27E-01	9.78E-01
**CSPP1**	0.19	3.62E-01	1.00E+00	-0.05	8.27E-01	9.78E-01
**RAB10**	0.05	8.84E-01	1.00E+00	0.05	8.27E-01	9.78E-01
**PICART1**	-1.23	5.60E-02	1.00E+00	0.22	8.27E-01	9.78E-01
**DNLZ**	-1.48	1.40E-01	1.00E+00	0.32	8.27E-01	9.78E-01
**ASCL4**	-1.07	3.49E-01	1.00E+00	0.24	8.27E-01	9.78E-01
**RASGRF1**	0.25	7.87E-01	1.00E+00	0.27	8.27E-01	9.78E-01
**APOL4**	-0.34	5.23E-01	1.00E+00	0.11	8.27E-01	9.78E-01
**TACR2**	-0.09	8.93E-01	1.00E+00	-0.13	8.27E-01	9.78E-01
**ZDHHC21**	-0.21	4.79E-01	1.00E+00	0.07	8.27E-01	9.78E-01
**FAUP1**	-1.87	3.77E-01	1.00E+00	-0.14	8.27E-01	9.78E-01
**AL139353.3**	-2.97	3.05E-01	1.00E+00	0.28	8.27E-01	9.78E-01
**TJP2**	0.05	7.92E-01	1.00E+00	-0.11	8.27E-01	9.78E-01
**TUBB4A**	-0.88	2.97E-01	1.00E+00	0.11	8.27E-01	9.78E-01
**SLC4A1**	-1.27	3.70E-01	1.00E+00	-0.29	8.27E-01	9.78E-01
**IGHV6-1**	0.56	8.71E-01	1.00E+00	0.70	8.27E-01	9.78E-01
**POMT1**	0.21	5.12E-01	1.00E+00	-0.05	8.27E-01	9.78E-01
**XLOC_003775**	-1.27	7.14E-01	1.00E+00	-0.31	8.27E-01	9.78E-01
**UTS2**	-3.43	3.11E-01	1.00E+00	-0.33	8.27E-01	9.78E-01
**PTDSS2**	-0.11	6.70E-01	1.00E+00	0.10	8.27E-01	9.78E-01
**AC016588.2**	0.38	6.03E-01	1.00E+00	-0.12	8.28E-01	9.78E-01
**UBA3**	0.17	4.08E-01	1.00E+00	-0.08	8.28E-01	9.78E-01
**CIT**	-0.23	4.86E-01	1.00E+00	0.08	8.28E-01	9.78E-01
**AL645929.1**	0.18	8.65E-01	1.00E+00	0.14	8.28E-01	9.78E-01
**PCIF1**	-0.19	3.92E-01	1.00E+00	0.03	8.28E-01	9.78E-01
**TRPC3**	-1.20	3.42E-01	1.00E+00	0.31	8.28E-01	9.78E-01
**RPL7AP60**	-3.01	9.96E-02	1.00E+00	0.19	8.28E-01	9.78E-01
**VPS35L**	-0.12	4.89E-01	1.00E+00	-0.04	8.28E-01	9.78E-01
**AC098591.2**	0.91	7.93E-01	1.00E+00	-0.17	8.28E-01	9.78E-01
**PLBD1-AS1**	0.08	8.88E-01	1.00E+00	0.10	8.28E-01	9.78E-01
**SLC25A21-AS1**	0.48	5.30E-01	1.00E+00	-0.15	8.28E-01	9.78E-01
**GAPDHP65**	1.56	6.54E-01	1.00E+00	0.17	8.28E-01	9.78E-01

**SEL1L**	0.18	3.43E-01	1.00E+00	0.04	8.28E-01	9.78E-01
**ASL**	-0.06	7.83E-01	1.00E+00	0.05	8.28E-01	9.78E-01
**NECAB3**	-0.40	2.62E-01	1.00E+00	-0.12	8.28E-01	9.78E-01
**PRKAR2A**	0.19	3.42E-01	1.00E+00	0.04	8.28E-01	9.78E-01
**EFS**	-0.34	2.15E-01	1.00E+00	-0.11	8.28E-01	9.78E-01
**G23048**	-0.24	8.99E-01	1.00E+00	-0.26	8.28E-01	9.78E-01
**FUBP3**	0.05	7.67E-01	1.00E+00	-0.07	8.28E-01	9.78E-01
**NUMBL**	-0.07	8.22E-01	1.00E+00	0.13	8.28E-01	9.78E-01
**ACAD10**	0.24	3.15E-01	1.00E+00	0.05	8.28E-01	9.78E-01
**AC090517.2**	-0.63	5.20E-01	1.00E+00	-0.14	8.28E-01	9.78E-01
**LINC01139**	1.09	5.35E-03	4.31E-01	0.20	8.28E-01	9.78E-01
**IL13**	3.51	2.97E-01	1.00E+00	-0.50	8.28E-01	9.78E-01
**CYYR1-AS1**	-4.11	3.97E-02	1.00E+00	-0.28	8.29E-01	9.78E-01
**ARHGEF37**	0.24	2.49E-01	1.00E+00	-0.08	8.29E-01	9.78E-01
**CD34**	0.27	5.61E-01	1.00E+00	0.10	8.29E-01	9.78E-01
**ECT2L**	-0.96	1.23E-01	1.00E+00	-0.15	8.29E-01	9.78E-01
**AC092295.2**	-0.29	4.15E-01	1.00E+00	0.18	8.29E-01	9.78E-01
**ECPAS**	0.00	9.81E-01	1.00E+00	-0.07	8.29E-01	9.78E-01
**IL12A**	0.02	9.91E-01	1.00E+00	0.28	8.29E-01	9.78E-01
**TEPP**	-3.01	2.76E-02	9.71E-01	0.14	8.29E-01	9.78E-01
**GP1BA**	-0.78	5.80E-02	1.00E+00	0.15	8.29E-01	9.78E-01
**XLOC_014188**	-1.29	6.93E-01	1.00E+00	-0.32	8.29E-01	9.78E-01
**DLGAP1-AS2**	-0.60	5.57E-01	1.00E+00	0.18	8.29E-01	9.78E-01
**FAR2P2**	0.88	3.10E-01	1.00E+00	-0.17	8.29E-01	9.78E-01
**HSPG2**	-0.01	9.79E-01	1.00E+00	0.68	8.29E-01	9.78E-01
**AC093627.4**	0.23	6.78E-01	1.00E+00	0.19	8.29E-01	9.78E-01
**ZNF835**	0.74	3.61E-01	1.00E+00	-0.46	8.29E-01	9.78E-01
**ST14**	-0.45	1.93E-01	1.00E+00	0.13	8.29E-01	9.78E-01
**CDKN1C**	0.24	6.22E-01	1.00E+00	0.13	8.29E-01	9.78E-01
**TMEM256P2**	1.48	6.66E-01	1.00E+00	-0.29	8.29E-01	9.78E-01
**UTF1**	0.43	8.87E-01	1.00E+00	0.43	8.29E-01	9.78E-01
**G25511**	0.79	4.43E-01	1.00E+00	-0.14	8.29E-01	9.78E-01

**XLOC_014369**	0.05	9.69E-01	1.00E+00	-0.21	8.29E-01	9.78E-01
**RPL23P2**	0.87	5.93E-01	1.00E+00	-0.21	8.29E-01	9.79E-01
**B4GALT2**	-0.23	2.48E-01	1.00E+00	-0.09	8.30E-01	9.79E-01
**LIPN**	-0.23	7.65E-01	1.00E+00	0.15	8.30E-01	9.79E-01
**CRISPLD1**	-0.18	7.88E-01	1.00E+00	-0.11	8.30E-01	9.79E-01
**METTL26**	-0.36	9.12E-02	1.00E+00	0.06	8.30E-01	9.79E-01
**PRDM5**	-0.12	7.39E-01	1.00E+00	0.09	8.30E-01	9.79E-01
**NOL7**	0.55	1.25E-01	1.00E+00	0.12	8.30E-01	9.79E-01
**EARS2**	-0.01	9.56E-01	1.00E+00	-0.04	8.30E-01	9.79E-01
**RPS2P44**	-0.32	9.26E-01	1.00E+00	-0.31	8.30E-01	9.79E-01
**AC234775.3**	-0.29	7.64E-01	1.00E+00	0.19	8.30E-01	9.79E-01
**AL008729.1**	0.00	9.98E-01	1.00E+00	-0.50	8.30E-01	9.79E-01
**NPR1**	1.07	8.77E-02	1.00E+00	0.16	8.30E-01	9.79E-01
**ELL3**	1.98	8.68E-02	1.00E+00	-0.20	8.30E-01	9.79E-01
**NRL**	-0.77	1.61E-01	1.00E+00	0.17	8.30E-01	9.79E-01
**JUP**	-0.22	6.20E-01	1.00E+00	-0.11	8.30E-01	9.79E-01
**XLOC_001680**	-0.37	8.99E-01	1.00E+00	0.22	8.30E-01	9.79E-01
**MAML3**	-0.41	2.55E-01	1.00E+00	0.08	8.31E-01	9.79E-01
**PRKAA1**	0.07	7.78E-01	1.00E+00	-0.04	8.31E-01	9.79E-01
**NDOR1**	-0.24	4.36E-01	1.00E+00	0.13	8.31E-01	9.79E-01
**AL451074.2**	1.03	4.24E-01	1.00E+00	-0.23	8.31E-01	9.79E-01
**TIMMDC1**	-0.05	7.70E-01	1.00E+00	-0.05	8.31E-01	9.79E-01
**PLAA**	0.00	9.86E-01	1.00E+00	0.05	8.31E-01	9.79E-01
**HEPACAM**	2.85	1.66E-01	1.00E+00	-0.68	8.31E-01	9.79E-01
**BOK**	0.57	2.01E-01	1.00E+00	-0.10	8.31E-01	9.79E-01
**RNF43**	-0.39	2.56E-01	1.00E+00	0.12	8.31E-01	9.79E-01
**RIOX1**	-0.11	6.64E-01	1.00E+00	-0.10	8.31E-01	9.79E-01
**AC092118.1**	-0.01	9.91E-01	1.00E+00	0.18	8.31E-01	9.79E-01
**TMEM269**	-0.78	3.54E-01	1.00E+00	-0.11	8.31E-01	9.79E-01
**ABCA4**	-0.92	8.83E-02	1.00E+00	0.17	8.31E-01	9.79E-01
**CRACR2A**	0.13	8.77E-01	1.00E+00	0.15	8.31E-01	9.80E-01
**ZNF280D**	0.18	5.54E-01	1.00E+00	0.06	8.31E-01	9.80E-01

**CDC42EP1**	-0.27	4.15E-01	1.00E+00	0.09	8.32E-01	9.80E-01
**IDH3A**	-0.23	3.12E-01	1.00E+00	0.05	8.32E-01	9.80E-01
**BPTF**	-0.12	6.06E-01	1.00E+00	0.06	8.32E-01	9.80E-01
**TUBA1A**	-0.34	2.01E-01	1.00E+00	-0.05	8.32E-01	9.80E-01
**WDR59**	-0.15	4.73E-01	1.00E+00	-0.09	8.32E-01	9.80E-01
**SAFB2**	-0.12	6.12E-01	1.00E+00	0.08	8.32E-01	9.80E-01
**GXYLT2**	0.18	7.13E-01	1.00E+00	-0.11	8.32E-01	9.80E-01
**NSRP1**	0.07	7.57E-01	1.00E+00	-0.06	8.32E-01	9.80E-01
**AL031118.1**	1.08	7.96E-02	1.00E+00	0.20	8.32E-01	9.80E-01
**IGFBP7-AS1**	-2.58	2.88E-01	1.00E+00	0.24	8.32E-01	9.80E-01
**C1DP1**	-0.80	8.18E-01	1.00E+00	0.24	8.32E-01	9.80E-01
**CAMTA2**	-0.16	5.23E-01	1.00E+00	0.09	8.32E-01	9.80E-01
**NONOP2**	0.27	6.77E-01	1.00E+00	-0.13	8.32E-01	9.80E-01
**DDX11L2**	-3.08	4.57E-02	1.00E+00	-0.23	8.32E-01	9.80E-01
**GRPEL2**	-0.25	3.78E-01	1.00E+00	0.07	8.32E-01	9.80E-01
**TRIM39**	0.33	1.36E-01	1.00E+00	-0.09	8.32E-01	9.80E-01
**FAM198B**	0.23	6.81E-01	1.00E+00	0.10	8.32E-01	9.80E-01
**FNBP1**	0.05	8.75E-01	1.00E+00	0.09	8.32E-01	9.80E-01
**EFCC1**	0.04	9.07E-01	1.00E+00	-0.18	8.32E-01	9.80E-01
**ANXA1**	0.41	8.04E-02	1.00E+00	0.07	8.32E-01	9.80E-01
**AC090617.5**	-0.09	8.72E-01	1.00E+00	-0.09	8.32E-01	9.80E-01
**ABCC5**	-0.09	7.05E-01	1.00E+00	0.06	8.32E-01	9.80E-01
**AADAC**	0.81	1.80E-01	1.00E+00	0.13	8.32E-01	9.80E-01
**ATG14**	-0.14	5.54E-01	1.00E+00	-0.06	8.32E-01	9.80E-01
**MED9**	0.05	8.52E-01	1.00E+00	-0.05	8.33E-01	9.80E-01
**COLCA1**	1.02	3.86E-01	1.00E+00	0.39	8.33E-01	9.80E-01
**RANP1**	0.27	8.27E-01	1.00E+00	0.09	8.33E-01	9.80E-01
**ZNF330**	0.16	5.14E-01	1.00E+00	0.04	8.33E-01	9.80E-01
**UPF3AP2**	0.27	7.13E-01	1.00E+00	-0.07	8.33E-01	9.80E-01
**COPG1**	-0.02	8.93E-01	1.00E+00	-0.07	8.33E-01	9.80E-01
**PCSK2**	-0.19	6.25E-01	1.00E+00	0.12	8.33E-01	9.80E-01
**FTL**	0.32	4.68E-01	1.00E+00	-0.11	8.33E-01	9.80E-01

**GIT1**	-0.27	2.11E-01	1.00E+00	-0.10	8.33E-01	9.80E-01
**CFAP74**	-1.46	3.04E-01	1.00E+00	0.23	8.33E-01	9.80E-01
**XG**	0.32	3.80E-01	1.00E+00	-0.08	8.33E-01	9.80E-01
**ZBTB21**	-0.25	3.89E-01	1.00E+00	0.05	8.33E-01	9.80E-01
**TTLL1**	-0.20	5.45E-01	1.00E+00	-0.05	8.33E-01	9.80E-01
**KRT8P15**	-0.93	7.85E-01	1.00E+00	-0.68	8.33E-01	9.80E-01
**TMEM139**	0.89	2.50E-01	1.00E+00	0.11	8.33E-01	9.80E-01
**LINC01102**	-3.52	2.32E-01	1.00E+00	0.67	8.33E-01	9.80E-01
**TMEM102**	-0.30	3.42E-01	1.00E+00	-0.14	8.33E-01	9.80E-01
**PRKX**	0.36	1.70E-01	1.00E+00	0.10	8.33E-01	9.80E-01
**FLG**	-0.10	8.39E-01	1.00E+00	0.16	8.33E-01	9.80E-01
**ACER1**	0.24	6.53E-01	1.00E+00	-0.16	8.33E-01	9.80E-01
**G34424**	0.02	9.87E-01	1.00E+00	0.16	8.33E-01	9.80E-01
**SPG11**	-0.28	2.28E-01	1.00E+00	-0.05	8.34E-01	9.80E-01
**HOXA11-AS**	-1.42	1.86E-01	1.00E+00	-0.26	8.34E-01	9.80E-01
**AP002812.2**	0.64	8.04E-01	1.00E+00	0.34	8.34E-01	9.80E-01
**CYP27B1**	-0.87	3.31E-01	1.00E+00	0.66	8.34E-01	9.80E-01
**TXNDC11**	-0.21	3.30E-01	1.00E+00	0.10	8.34E-01	9.80E-01
**ZNF570**	0.04	8.87E-01	1.00E+00	0.06	8.34E-01	9.80E-01
**TPSB2**	0.67	2.36E-01	1.00E+00	0.12	8.34E-01	9.80E-01
**HINT3**	0.21	3.14E-01	1.00E+00	-0.05	8.34E-01	9.80E-01
**NRIP1**	-0.10	7.35E-01	1.00E+00	-0.10	8.35E-01	9.81E-01
**ZFAND5**	0.00	9.93E-01	1.00E+00	-0.09	8.35E-01	9.81E-01
**COPS7B**	0.02	9.17E-01	1.00E+00	-0.04	8.35E-01	9.81E-01
**AC108704.2**	0.30	8.56E-01	1.00E+00	-0.21	8.35E-01	9.81E-01
**DIS3**	0.15	3.89E-01	1.00E+00	0.05	8.35E-01	9.81E-01
**PIFO**	0.06	8.87E-01	1.00E+00	0.10	8.35E-01	9.81E-01
**SRRM2**	-0.60	2.55E-02	9.29E-01	-0.11	8.35E-01	9.81E-01
**KRTAP1-3**	-19.72	4.78E-09	2.45E-06	-0.66	8.35E-01	9.81E-01
**CNN2P1**	3.95	1.12E-01	1.00E+00	-0.32	8.35E-01	9.81E-01
**AKAP8**	-0.47	4.79E-02	1.00E+00	0.06	8.35E-01	9.81E-01
**RIMS2**	-2.27	1.01E-02	6.26E-01	-0.21	8.35E-01	9.81E-01

**AP002784.2**	-0.56	7.11E-01	1.00E+00	0.09	8.35E-01	9.81E-01
**DIDO1**	-0.25	2.63E-01	1.00E+00	-0.05	8.35E-01	9.81E-01
**RPS26P47**	1.95	1.43E-01	1.00E+00	0.14	8.35E-01	9.81E-01
**RP4-565E6.1**	0.70	4.63E-01	1.00E+00	0.24	8.35E-01	9.81E-01
**RAP1B**	0.29	1.37E-01	1.00E+00	-0.05	8.35E-01	9.81E-01
**GPR75**	0.11	8.62E-01	1.00E+00	0.14	8.35E-01	9.81E-01
**FUNDC2**	0.14	5.12E-01	1.00E+00	-0.04	8.36E-01	9.81E-01
**COL19A1**	-1.98	1.79E-01	1.00E+00	-0.26	8.36E-01	9.81E-01
**SNHG14**	0.38	4.30E-01	1.00E+00	-0.10	8.36E-01	9.81E-01
**AC073254.1**	0.87	9.77E-02	1.00E+00	-0.15	8.36E-01	9.81E-01
**GTF2IRD1**	-0.51	1.32E-01	1.00E+00	0.09	8.36E-01	9.81E-01
**STAT5A**	0.45	1.97E-01	1.00E+00	-0.13	8.36E-01	9.81E-01
**AC046176.1**	-0.49	6.13E-01	1.00E+00	-0.09	8.36E-01	9.81E-01
**NHLRC1**	-0.14	6.16E-01	1.00E+00	0.06	8.36E-01	9.81E-01
**TP53**	-0.22	4.18E-01	1.00E+00	-0.05	8.36E-01	9.81E-01
**ETNK1**	-0.04	9.03E-01	1.00E+00	-0.06	8.36E-01	9.81E-01
**ARHGAP32**	-0.23	4.38E-01	1.00E+00	0.07	8.36E-01	9.81E-01
**AC107032.1**	-0.92	3.54E-01	1.00E+00	-0.14	8.36E-01	9.81E-01
**TDRD3**	0.04	8.40E-01	1.00E+00	-0.04	8.36E-01	9.81E-01
**MLEC**	0.62	9.42E-03	6.06E-01	-0.08	8.36E-01	9.81E-01
**G34423**	-1.18	8.34E-02	1.00E+00	-0.23	8.36E-01	9.81E-01
**CHST8**	-0.78	2.15E-01	1.00E+00	-0.57	8.36E-01	9.81E-01
**GLS2**	0.85	6.90E-01	1.00E+00	-0.18	8.36E-01	9.81E-01
**PTDSS1**	-0.23	2.27E-01	1.00E+00	0.04	8.36E-01	9.81E-01
**MEIS3P1**	0.14	7.53E-01	1.00E+00	-0.51	8.36E-01	9.81E-01
**FAHD2A**	0.18	4.31E-01	1.00E+00	-0.04	8.36E-01	9.81E-01
**AGBL4**	-0.26	7.82E-01	1.00E+00	-0.18	8.36E-01	9.81E-01
**CR2**	2.83	4.04E-01	1.00E+00	0.64	8.36E-01	9.81E-01
**PALM3**	0.11	8.39E-01	1.00E+00	-0.26	8.36E-01	9.81E-01
**C17orf97**	0.54	2.47E-01	1.00E+00	-0.12	8.36E-01	9.81E-01
**PHLDA3**	0.10	7.56E-01	1.00E+00	0.12	8.36E-01	9.81E-01
**G30143**	-1.88	1.61E-01	1.00E+00	-0.25	8.36E-01	9.81E-01

**TCF7**	-0.39	9.69E-02	1.00E+00	-0.11	8.36E-01	9.81E-01
**CMA1**	0.70	2.43E-01	1.00E+00	0.13	8.36E-01	9.81E-01
**TECRP1**	0.12	8.26E-01	1.00E+00	-0.13	8.36E-01	9.81E-01
**MT3**	0.20	9.51E-01	1.00E+00	-0.22	8.36E-01	9.81E-01
**POT1-AS1**	-1.83	5.25E-01	1.00E+00	0.20	8.37E-01	9.81E-01
**FIBP**	-0.26	1.40E-01	1.00E+00	-0.05	8.37E-01	9.81E-01
**IGHE**	0.60	8.17E-01	1.00E+00	-0.40	8.37E-01	9.81E-01
**ARHGAP28**	-0.43	3.51E-01	1.00E+00	-0.08	8.37E-01	9.81E-01
**AGMO**	0.82	2.54E-01	1.00E+00	-0.17	8.37E-01	9.81E-01
**LMLN**	-0.09	8.36E-01	1.00E+00	-0.07	8.37E-01	9.81E-01
**AC109635.5**	1.32	4.93E-01	1.00E+00	0.27	8.37E-01	9.81E-01
**NT5M**	0.07	8.92E-01	1.00E+00	-0.14	8.37E-01	9.81E-01
**G6313**	0.72	1.12E-01	1.00E+00	0.15	8.37E-01	9.81E-01
**IFT140**	-0.02	9.39E-01	1.00E+00	0.12	8.37E-01	9.81E-01
**HCAR2**	0.17	7.58E-01	1.00E+00	0.06	8.37E-01	9.81E-01
**AC106869.1**	0.32	7.26E-01	1.00E+00	0.21	8.37E-01	9.81E-01
**PCGF1**	-0.02	9.00E-01	1.00E+00	0.04	8.37E-01	9.81E-01
**TIA1**	-0.24	4.19E-01	1.00E+00	0.05	8.38E-01	9.81E-01
**THAP9-AS1**	-0.26	3.49E-01	1.00E+00	-0.06	8.38E-01	9.81E-01
**SLC25A24**	0.31	3.24E-01	1.00E+00	-0.05	8.38E-01	9.81E-01
**AL590705.3**	-0.77	4.57E-01	1.00E+00	-0.11	8.38E-01	9.81E-01
**PSMD11**	0.00	9.88E-01	1.00E+00	-0.05	8.38E-01	9.81E-01
**AC099778.1**	-0.76	3.02E-01	1.00E+00	0.17	8.38E-01	9.81E-01
**CKS1BP2**	-2.87	3.99E-01	1.00E+00	-0.12	8.38E-01	9.81E-01
**MOCS1**	0.25	4.96E-01	1.00E+00	-0.12	8.38E-01	9.81E-01
**AL022097.1**	-1.39	4.96E-01	1.00E+00	-0.26	8.38E-01	9.81E-01
**AP000350.6**	2.62	3.19E-01	1.00E+00	-0.33	8.38E-01	9.81E-01
**RBBP5**	0.26	3.47E-01	1.00E+00	0.04	8.38E-01	9.81E-01
**C1orf134**	0.26	5.21E-01	1.00E+00	0.10	8.38E-01	9.81E-01
**AC156455.1**	-1.13	2.97E-01	1.00E+00	0.19	8.38E-01	9.81E-01
**SLC25A13**	-0.01	9.63E-01	1.00E+00	0.04	8.38E-01	9.81E-01
**PRPF40A**	-0.06	8.14E-01	1.00E+00	-0.05	8.38E-01	9.81E-01

**TMEM185A**	0.09	6.49E-01	1.00E+00	0.06	8.38E-01	9.81E-01
**HEATR1**	0.08	7.43E-01	1.00E+00	0.09	8.38E-01	9.81E-01
**NCCRP1**	-0.15	8.03E-01	1.00E+00	-0.15	8.38E-01	9.81E-01
**AL139393.2**	0.18	5.93E-01	1.00E+00	0.14	8.38E-01	9.81E-01
**C5orf56**	0.04	9.46E-01	1.00E+00	0.10	8.38E-01	9.81E-01
**AC007537.1**	2.32	1.22E-01	1.00E+00	0.14	8.38E-01	9.81E-01
**AC100814.1**	-0.72	6.16E-01	1.00E+00	-0.17	8.38E-01	9.81E-01
**NRBF2**	0.06	7.76E-01	1.00E+00	-0.06	8.38E-01	9.81E-01
**CLOCK**	0.03	9.15E-01	1.00E+00	-0.04	8.38E-01	9.81E-01
**AC004943.2**	0.12	8.00E-01	1.00E+00	0.10	8.38E-01	9.81E-01
**PRKAG3**	-0.26	8.79E-01	1.00E+00	-0.31	8.38E-01	9.81E-01
**STARD13-AS**	1.91	1.53E-01	1.00E+00	-0.14	8.38E-01	9.81E-01
**GAS6**	0.08	7.76E-01	1.00E+00	-0.08	8.38E-01	9.81E-01
**ANO6**	0.47	1.33E-01	1.00E+00	-0.08	8.38E-01	9.81E-01
**BTBD7P1**	-1.07	4.22E-01	1.00E+00	0.33	8.38E-01	9.81E-01
**CACNA1H**	-0.55	6.06E-02	1.00E+00	-0.16	8.38E-01	9.81E-01
**AC080013.4**	0.67	2.26E-01	1.00E+00	-0.13	8.39E-01	9.81E-01
**TSPOAP1-AS1**	1.27	3.06E-01	1.00E+00	-0.22	8.39E-01	9.81E-01
**BBX**	0.04	8.67E-01	1.00E+00	-0.03	8.39E-01	9.81E-01
**CDKN3**	0.14	7.33E-01	1.00E+00	-0.10	8.39E-01	9.81E-01
**TMC3**	-0.22	8.90E-01	1.00E+00	0.25	8.39E-01	9.81E-01
**DVL2**	-0.17	4.08E-01	1.00E+00	-0.08	8.39E-01	9.81E-01
**AL021878.2**	0.82	5.73E-01	1.00E+00	0.20	8.39E-01	9.81E-01
**AC078962.2**	-0.26	8.06E-01	1.00E+00	-0.17	8.39E-01	9.81E-01
**AC008443.4**	-2.59	4.49E-01	1.00E+00	-0.15	8.39E-01	9.81E-01
**LINC02227**	2.53	4.56E-01	1.00E+00	0.23	8.39E-01	9.81E-01
**AC098847.1**	0.21	7.60E-01	1.00E+00	0.18	8.39E-01	9.81E-01
**AL035661.1**	-0.80	8.18E-01	1.00E+00	-0.18	8.39E-01	9.81E-01
**RPS6KA2**	0.36	2.05E-01	1.00E+00	-0.09	8.39E-01	9.81E-01
**SNORD104**	1.42	3.83E-01	1.00E+00	0.22	8.39E-01	9.81E-01
**AC005165.1**	-1.88	5.71E-01	1.00E+00	-0.22	8.39E-01	9.81E-01
**TMEM217**	-0.03	9.56E-01	1.00E+00	0.14	8.39E-01	9.81E-01

**AC025857.2**	-2.22	1.90E-01	1.00E+00	0.16	8.39E-01	9.81E-01
**RNF32**	0.00	9.95E-01	1.00E+00	0.10	8.39E-01	9.81E-01
**TMC1**	-0.15	8.40E-01	1.00E+00	-0.16	8.39E-01	9.81E-01
**C5orf22**	-0.26	3.24E-01	1.00E+00	0.06	8.39E-01	9.81E-01
**NUP205**	-0.02	9.27E-01	1.00E+00	-0.07	8.39E-01	9.81E-01
**MLKL**	-0.07	8.80E-01	1.00E+00	-0.49	8.39E-01	9.81E-01
**ESPN**	-0.62	2.25E-01	1.00E+00	-0.14	8.39E-01	9.81E-01
**DHRS4L1**	-1.28	4.12E-01	1.00E+00	-0.18	8.39E-01	9.81E-01
**CLIC4**	0.25	5.39E-01	1.00E+00	0.07	8.40E-01	9.81E-01
**ZNF426**	-0.16	4.41E-01	1.00E+00	0.04	8.40E-01	9.81E-01
**USP28**	0.30	2.35E-01	1.00E+00	-0.08	8.40E-01	9.81E-01
**INSYN1-AS1**	0.65	5.55E-01	1.00E+00	0.27	8.40E-01	9.81E-01
**AC008243.1**	-0.13	9.69E-01	1.00E+00	0.24	8.40E-01	9.81E-01
**FXN**	-0.22	4.99E-01	1.00E+00	-0.05	8.40E-01	9.81E-01
**KCNH3**	-1.07	1.28E-01	1.00E+00	0.28	8.40E-01	9.81E-01
**IGFL2**	0.05	9.16E-01	1.00E+00	0.15	8.40E-01	9.81E-01
**EML1**	-0.05	8.47E-01	1.00E+00	0.08	8.40E-01	9.81E-01
**AMN**	-0.84	9.03E-02	1.00E+00	-0.19	8.40E-01	9.81E-01
**GFRA3**	-0.34	5.15E-01	1.00E+00	0.10	8.40E-01	9.81E-01
**SH3GL1**	-0.18	6.06E-01	1.00E+00	0.08	8.40E-01	9.81E-01
**RPL26P30**	0.02	9.89E-01	1.00E+00	0.14	8.40E-01	9.81E-01
**CCNL2**	-0.13	7.01E-01	1.00E+00	-0.07	8.40E-01	9.81E-01
**AC008494.3**	-1.51	3.66E-01	1.00E+00	-0.14	8.40E-01	9.81E-01
**TOMM40**	-0.37	1.51E-01	1.00E+00	-0.11	8.40E-01	9.81E-01
**BLCAP**	0.23	2.01E-01	1.00E+00	-0.04	8.40E-01	9.81E-01
**BIRC2**	0.22	3.50E-01	1.00E+00	0.03	8.40E-01	9.81E-01
**HPS1**	-0.11	6.10E-01	1.00E+00	0.07	8.40E-01	9.81E-01
**HOXB5**	0.75	3.41E-01	1.00E+00	0.14	8.40E-01	9.81E-01
**OLAH**	0.68	6.91E-01	1.00E+00	-0.24	8.40E-01	9.81E-01
**AC090136.3**	0.39	8.00E-01	1.00E+00	0.25	8.40E-01	9.81E-01
**C22orf15**	1.30	3.96E-01	1.00E+00	-0.31	8.41E-01	9.81E-01
**FOXO3**	-0.05	8.85E-01	1.00E+00	-0.06	8.41E-01	9.81E-01

**AL353704.1**	0.97	2.90E-01	1.00E+00	0.21	8.41E-01	9.81E-01
**FPGT-TNNI3K**	0.62	6.95E-01	1.00E+00	-0.55	8.41E-01	9.81E-01
**PLEKHH3**	-0.35	1.92E-01	1.00E+00	0.13	8.41E-01	9.81E-01
**EIF3J**	0.08	7.05E-01	1.00E+00	0.05	8.41E-01	9.81E-01
**ENTPD1-AS1**	-0.14	6.83E-01	1.00E+00	0.06	8.41E-01	9.81E-01
**TRIM47**	-0.55	2.55E-02	9.29E-01	0.09	8.41E-01	9.81E-01
**ABCB6**	0.82	8.00E-01	1.00E+00	0.15	8.41E-01	9.81E-01
**LINC01504**	-0.12	8.64E-01	1.00E+00	0.15	8.41E-01	9.81E-01
**P2RY13**	0.56	3.03E-01	1.00E+00	0.10	8.41E-01	9.81E-01
**TSPEAR**	-1.25	1.13E-01	1.00E+00	-0.21	8.41E-01	9.81E-01
**BX679664.3**	0.03	9.42E-01	1.00E+00	-0.08	8.41E-01	9.81E-01
**AC004241.1**	0.89	3.93E-01	1.00E+00	-0.11	8.41E-01	9.81E-01
**AC009245.1**	-0.22	7.23E-01	1.00E+00	0.17	8.41E-01	9.81E-01
**FCER2**	0.00	9.98E-01	1.00E+00	0.56	8.41E-01	9.81E-01
**DDX19A**	0.27	2.78E-01	1.00E+00	0.03	8.41E-01	9.81E-01
**JMJD1C-AS1**	1.32	2.68E-01	1.00E+00	-0.15	8.41E-01	9.81E-01
**CLCC1**	0.31	2.76E-01	1.00E+00	-0.05	8.41E-01	9.81E-01
**FAM162B**	0.32	5.31E-01	1.00E+00	0.12	8.42E-01	9.81E-01
**TBC1D25**	-0.17	4.82E-01	1.00E+00	-0.06	8.42E-01	9.81E-01
**STEAP2**	0.69	9.41E-02	1.00E+00	-0.08	8.42E-01	9.81E-01
**OVOL1**	-0.91	2.18E-01	1.00E+00	-0.15	8.42E-01	9.81E-01
**LINC00908**	0.87	3.28E-02	1.00E+00	0.14	8.42E-01	9.81E-01
**MRPS24**	0.44	7.79E-01	1.00E+00	0.17	8.42E-01	9.81E-01
**ALDH1A1**	0.68	1.90E-01	1.00E+00	0.10	8.42E-01	9.81E-01
**RN7SL208P**	0.02	9.88E-01	1.00E+00	0.17	8.42E-01	9.81E-01
**AP000808.2**	-0.16	8.84E-01	1.00E+00	-0.25	8.42E-01	9.81E-01
**TLCD2**	0.09	8.73E-01	1.00E+00	0.09	8.42E-01	9.81E-01
**CERNA1**	-1.19	4.04E-01	1.00E+00	0.16	8.42E-01	9.81E-01
**BTAF1**	0.09	8.06E-01	1.00E+00	0.06	8.42E-01	9.81E-01
**APEX2**	-0.14	5.66E-01	1.00E+00	0.04	8.42E-01	9.81E-01
**HMGN2P15**	0.03	9.74E-01	1.00E+00	0.15	8.42E-01	9.81E-01
**NUDT19**	-0.16	4.79E-01	1.00E+00	0.07	8.42E-01	9.81E-01

**RP11-292B8.1**	-0.02	9.69E-01	1.00E+00	0.07	8.42E-01	9.81E-01
**BICDL1**	-0.14	7.60E-01	1.00E+00	-0.12	8.42E-01	9.81E-01
**TBL1XR1**	0.42	5.96E-02	1.00E+00	-0.08	8.42E-01	9.81E-01
**VSIR**	0.13	6.04E-01	1.00E+00	0.05	8.42E-01	9.81E-01
**SERP2**	-0.15	7.53E-01	1.00E+00	-0.14	8.42E-01	9.81E-01
**AC105942.1**	0.08	8.84E-01	1.00E+00	-0.17	8.42E-01	9.81E-01
**CD27-AS1**	0.75	5.44E-02	1.00E+00	-0.06	8.42E-01	9.81E-01
**PLEKHN1**	-0.09	8.53E-01	1.00E+00	0.12	8.42E-01	9.81E-01
**TYW5**	0.55	9.74E-02	1.00E+00	0.06	8.43E-01	9.81E-01
**HAR1A**	0.86	1.72E-01	1.00E+00	0.21	8.43E-01	9.81E-01
**STX17-AS1**	-0.14	9.51E-01	1.00E+00	-0.15	8.43E-01	9.81E-01
**TOB1**	-0.40	2.75E-01	1.00E+00	0.09	8.43E-01	9.81E-01
**POLG2**	0.30	5.22E-01	1.00E+00	0.09	8.43E-01	9.81E-01
**ZNF134**	0.03	8.82E-01	1.00E+00	-0.04	8.43E-01	9.81E-01
**AC092364.2**	1.25	7.19E-01	1.00E+00	-0.33	8.43E-01	9.81E-01
**CEP55**	-0.11	7.52E-01	1.00E+00	-0.15	8.43E-01	9.81E-01
**G26738**	0.36	8.50E-01	1.00E+00	0.31	8.43E-01	9.81E-01
**PMPCB**	0.03	8.97E-01	1.00E+00	-0.04	8.43E-01	9.81E-01
**G43621**	-1.56	6.20E-01	1.00E+00	-0.64	8.43E-01	9.81E-01
**BSG**	-0.08	6.90E-01	1.00E+00	-0.03	8.43E-01	9.81E-01
**UBA1**	-0.13	4.49E-01	1.00E+00	0.07	8.43E-01	9.81E-01
**CCDC107**	0.27	4.66E-01	1.00E+00	-0.10	8.43E-01	9.81E-01
**KANK2**	0.12	7.84E-01	1.00E+00	0.10	8.43E-01	9.81E-01
**ALDH3B2**	-0.54	2.97E-01	1.00E+00	0.12	8.43E-01	9.81E-01
**ZNF546**	1.02	1.92E-01	1.00E+00	0.13	8.43E-01	9.81E-01
**ANAPC7**	-0.12	5.24E-01	1.00E+00	-0.03	8.43E-01	9.81E-01
**NXF3**	-2.70	1.69E-01	1.00E+00	-0.33	8.43E-01	9.81E-01
**SERPINA5**	0.41	5.81E-01	1.00E+00	-0.15	8.43E-01	9.81E-01
**G42897**	1.17	2.66E-01	1.00E+00	0.12	8.43E-01	9.81E-01
**CPNE2**	0.21	6.14E-01	1.00E+00	0.11	8.43E-01	9.81E-01
**ZNF836**	0.05	8.86E-01	1.00E+00	-0.10	8.43E-01	9.81E-01
**HYAL1**	-0.08	7.88E-01	1.00E+00	-0.05	8.43E-01	9.81E-01

**RP9P**	0.27	3.92E-01	1.00E+00	0.07	8.44E-01	9.81E-01
**IVD**	0.41	4.44E-01	1.00E+00	0.08	8.44E-01	9.81E-01
**LINC00894**	0.47	7.11E-01	1.00E+00	0.18	8.44E-01	9.81E-01
**AC009812.1**	-0.86	2.26E-01	1.00E+00	0.12	8.44E-01	9.81E-01
**AL513321.2**	-0.42	6.14E-01	1.00E+00	-0.22	8.44E-01	9.81E-01
**ARL9**	-0.37	1.63E-01	1.00E+00	0.10	8.44E-01	9.81E-01
**XLOC_001061**	2.12	1.70E-01	1.00E+00	-0.22	8.44E-01	9.81E-01
**AGPAT3**	0.13	6.49E-01	1.00E+00	0.09	8.44E-01	9.81E-01
**DSTNP1**	0.46	4.97E-01	1.00E+00	0.10	8.44E-01	9.81E-01
**WDR45B**	0.13	5.30E-01	1.00E+00	0.03	8.44E-01	9.81E-01
**AL117335.1**	0.47	4.90E-01	1.00E+00	-0.16	8.44E-01	9.81E-01
**AL353596.1**	1.43	2.26E-01	1.00E+00	0.31	8.44E-01	9.81E-01
**ARHGAP31-AS1**	0.95	3.36E-01	1.00E+00	-0.17	8.44E-01	9.81E-01
**LINC01719**	0.49	4.07E-01	1.00E+00	0.20	8.44E-01	9.81E-01
**LRRN1**	-0.63	5.57E-02	1.00E+00	-0.10	8.44E-01	9.81E-01
**TAF1A-AS1**	2.04	2.68E-01	1.00E+00	0.21	8.44E-01	9.81E-01
**NWD2**	-0.03	9.64E-01	1.00E+00	0.18	8.44E-01	9.81E-01
**TTC21B**	0.51	1.19E-01	1.00E+00	-0.06	8.44E-01	9.81E-01
**AL136040.1**	0.10	8.68E-01	1.00E+00	0.12	8.44E-01	9.81E-01
**EMC4**	-0.08	6.11E-01	1.00E+00	-0.06	8.44E-01	9.81E-01
**PPWD1**	0.28	1.70E-01	1.00E+00	-0.05	8.45E-01	9.81E-01
**AL022344.2**	-0.04	9.29E-01	1.00E+00	0.17	8.45E-01	9.81E-01
**APOBEC2**	-0.56	5.97E-01	1.00E+00	-0.18	8.45E-01	9.81E-01
**GAP43**	0.35	9.12E-01	1.00E+00	0.36	8.45E-01	9.81E-01
**UTP18**	-0.09	7.98E-01	1.00E+00	0.05	8.45E-01	9.81E-01
**RPL29P11**	0.38	3.32E-01	1.00E+00	-0.09	8.45E-01	9.81E-01
**AC002059.2**	-2.17	3.26E-01	1.00E+00	-0.19	8.45E-01	9.81E-01
**GRIK5**	-0.15	7.78E-01	1.00E+00	0.22	8.45E-01	9.81E-01
**LRRC37A3**	-0.33	3.68E-01	1.00E+00	0.11	8.45E-01	9.81E-01
**RASGRP1**	-0.39	4.07E-01	1.00E+00	-0.12	8.45E-01	9.81E-01
**JAM3**	0.09	7.99E-01	1.00E+00	0.09	8.45E-01	9.81E-01
**PIK3C3**	0.10	6.42E-01	1.00E+00	0.04	8.45E-01	9.81E-01

**ASAH2B**	0.35	3.48E-01	1.00E+00	0.06	8.45E-01	9.81E-01
**ZNF137P**	-0.15	8.22E-01	1.00E+00	0.22	8.45E-01	9.81E-01
**HOXC13-AS**	0.23	5.97E-01	1.00E+00	-0.10	8.45E-01	9.81E-01
**CTBP1-DT**	-0.37	7.74E-02	1.00E+00	-0.09	8.45E-01	9.81E-01
**KHDRBS3**	-0.21	5.88E-01	1.00E+00	-0.13	8.45E-01	9.81E-01
**AZI2**	0.04	8.64E-01	1.00E+00	-0.05	8.45E-01	9.81E-01
**LINC02026**	-0.43	9.02E-01	1.00E+00	0.19	8.45E-01	9.81E-01
**LRRC37A11P**	0.07	8.37E-01	1.00E+00	0.13	8.45E-01	9.81E-01
**ADAMTS15**	0.78	3.15E-01	1.00E+00	0.13	8.45E-01	9.81E-01
**DLC1**	0.12	7.13E-01	1.00E+00	0.10	8.45E-01	9.81E-01
**JRKL**	0.10	7.57E-01	1.00E+00	0.05	8.45E-01	9.81E-01
**LRCH4**	-0.07	7.13E-01	1.00E+00	0.19	8.45E-01	9.81E-01
**GPAT2P1**	-3.40	9.88E-02	1.00E+00	-0.19	8.45E-01	9.81E-01
**PRKD2**	-0.35	1.77E-01	1.00E+00	0.09	8.46E-01	9.81E-01
**KLHL26**	-0.34	1.38E-01	1.00E+00	0.09	8.46E-01	9.81E-01
**NUP188**	-0.07	7.06E-01	1.00E+00	-0.08	8.46E-01	9.81E-01
**HNRNPH2**	-0.09	6.86E-01	1.00E+00	-0.03	8.46E-01	9.81E-01
**AC093495.1**	2.54	7.74E-04	1.37E-01	-0.22	8.46E-01	9.81E-01
**BACH2**	0.05	9.00E-01	1.00E+00	0.09	8.46E-01	9.81E-01
**SEC24A**	0.21	3.18E-01	1.00E+00	0.05	8.46E-01	9.81E-01
**ZNF239**	0.65	3.40E-01	1.00E+00	-0.12	8.46E-01	9.81E-01
**AL359232.1**	0.03	9.76E-01	1.00E+00	-0.12	8.46E-01	9.81E-01
**SLC25A6**	-1.83	5.95E-01	1.00E+00	-0.12	8.46E-01	9.81E-01
**COPE**	-0.26	2.63E-01	1.00E+00	0.06	8.46E-01	9.81E-01
**G25497**	0.35	8.53E-01	1.00E+00	-0.21	8.46E-01	9.81E-01
**QTRT1**	-0.39	1.04E-01	1.00E+00	-0.04	8.46E-01	9.81E-01
**ANKRD13B**	-0.50	1.90E-01	1.00E+00	0.10	8.46E-01	9.81E-01
**UBE2C**	-0.06	8.57E-01	1.00E+00	-0.10	8.46E-01	9.81E-01
**AC126755.1**	0.22	7.03E-01	1.00E+00	0.28	8.46E-01	9.81E-01
**AC024075.2**	0.05	9.29E-01	1.00E+00	0.10	8.46E-01	9.81E-01
**RASSF1**	0.24	3.70E-01	1.00E+00	0.05	8.46E-01	9.81E-01
**AC005162.2**	-2.98	1.04E-01	1.00E+00	0.28	8.46E-01	9.81E-01

**ARFGAP3**	-0.01	9.68E-01	1.00E+00	0.04	8.46E-01	9.81E-01
**TAF1C**	-0.11	6.61E-01	1.00E+00	0.11	8.46E-01	9.81E-01
**TNNT2**	0.31	6.18E-01	1.00E+00	0.12	8.46E-01	9.81E-01
**AL359094.1**	-0.28	8.96E-01	1.00E+00	0.17	8.46E-01	9.81E-01
**AC025449.1**	0.18	8.32E-01	1.00E+00	0.11	8.46E-01	9.81E-01
**AC116535.1**	-0.37	7.44E-01	1.00E+00	0.11	8.46E-01	9.81E-01
**AL031283.1**	-3.07	2.65E-01	1.00E+00	0.26	8.46E-01	9.81E-01
**RHOU**	0.19	5.54E-01	1.00E+00	0.12	8.47E-01	9.81E-01
**SMARCA1**	0.06	8.56E-01	1.00E+00	-0.05	8.47E-01	9.81E-01
**AC008752.3**	1.62	2.54E-01	1.00E+00	0.24	8.47E-01	9.81E-01
**ZNF703**	-0.34	3.88E-01	1.00E+00	0.10	8.47E-01	9.81E-01
**VWA5A**	0.17	3.98E-01	1.00E+00	0.05	8.47E-01	9.81E-01
**AC106886.4**	-0.19	7.97E-01	1.00E+00	0.22	8.47E-01	9.81E-01
**NCKIPSD**	0.10	6.04E-01	1.00E+00	0.07	8.47E-01	9.81E-01
**RPL7P23**	0.21	7.13E-01	1.00E+00	0.18	8.47E-01	9.81E-01
**UBXN2B**	0.14	5.18E-01	1.00E+00	-0.05	8.47E-01	9.81E-01
**AL158207.1**	-0.25	7.16E-01	1.00E+00	0.11	8.47E-01	9.81E-01
**TCAM1P**	0.37	7.43E-01	1.00E+00	-0.62	8.47E-01	9.81E-01
**AL049647.1**	0.27	8.77E-01	1.00E+00	0.23	8.47E-01	9.81E-01
**SLC44A4**	-0.39	6.03E-01	1.00E+00	-0.11	8.47E-01	9.81E-01
**SERPINB7**	0.53	3.35E-01	1.00E+00	0.14	8.47E-01	9.81E-01
**IGHV3-73**	1.49	6.64E-01	1.00E+00	0.62	8.47E-01	9.81E-01
**IMPDH2**	-0.27	3.43E-01	1.00E+00	0.04	8.47E-01	9.81E-01
**INMT**	0.56	4.00E-01	1.00E+00	-0.09	8.47E-01	9.81E-01
**OGFOD1**	-0.15	3.85E-01	1.00E+00	0.03	8.47E-01	9.81E-01
**VIT**	0.45	2.53E-01	1.00E+00	0.09	8.48E-01	9.81E-01
**SLC35B4**	0.30	3.98E-01	1.00E+00	0.07	8.48E-01	9.81E-01
**AC107959.1**	0.79	6.18E-01	1.00E+00	-0.12	8.48E-01	9.81E-01
**KRTAP5-5**	0.82	7.58E-01	1.00E+00	-0.62	8.48E-01	9.81E-01
**AK1**	-0.04	9.13E-01	1.00E+00	0.08	8.48E-01	9.81E-01
**AL138689.1**	-3.69	1.34E-01	1.00E+00	-0.24	8.48E-01	9.81E-01
**KRTAP5-7**	-1.48	5.52E-01	1.00E+00	-0.62	8.48E-01	9.81E-01

**CORO1C**	0.14	5.85E-01	1.00E+00	-0.05	8.48E-01	9.81E-01
**LINC00921**	-0.06	9.06E-01	1.00E+00	-0.11	8.48E-01	9.81E-01
**ULK3**	0.10	7.64E-01	1.00E+00	-0.07	8.48E-01	9.81E-01
**IL22RA1**	-0.06	9.24E-01	1.00E+00	0.13	8.48E-01	9.81E-01
**AC018868.1**	0.02	9.92E-01	1.00E+00	-0.15	8.48E-01	9.81E-01
**MRGPRF-AS1**	-0.01	9.91E-01	1.00E+00	0.23	8.48E-01	9.81E-01
**XPO1**	0.05	8.30E-01	1.00E+00	-0.04	8.48E-01	9.81E-01
**ADORA2B**	0.06	8.69E-01	1.00E+00	-0.10	8.48E-01	9.81E-01
**AC011498.7**	-0.18	7.99E-01	1.00E+00	-0.20	8.48E-01	9.81E-01
**MTHFD1P1**	1.07	6.72E-01	1.00E+00	-0.18	8.48E-01	9.81E-01
**HIP1R**	0.05	8.72E-01	1.00E+00	0.10	8.48E-01	9.81E-01
**RASSF8-AS1**	0.27	5.46E-01	1.00E+00	0.12	8.48E-01	9.81E-01
**AP003392.5**	0.12	9.58E-01	1.00E+00	0.25	8.48E-01	9.81E-01
**CERS3**	0.19	7.01E-01	1.00E+00	0.14	8.48E-01	9.81E-01
**RAX**	-0.34	6.35E-01	1.00E+00	0.61	8.48E-01	9.81E-01
**AC087741.1**	0.42	6.80E-01	1.00E+00	0.16	8.48E-01	9.81E-01
**AC027607.1**	0.32	9.15E-01	1.00E+00	0.23	8.48E-01	9.81E-01
**SLC26A4**	-0.70	3.99E-01	1.00E+00	-0.40	8.48E-01	9.81E-01
**AC010326.4**	0.16	8.78E-01	1.00E+00	0.13	8.48E-01	9.81E-01
**NLRX1**	-0.40	1.22E-01	1.00E+00	0.10	8.48E-01	9.81E-01
**XLOC_005573**	-1.21	2.26E-01	1.00E+00	0.21	8.48E-01	9.81E-01
**FGD5-AS1**	0.17	3.83E-01	1.00E+00	-0.05	8.48E-01	9.81E-01
**STK32B**	0.02	9.67E-01	1.00E+00	0.11	8.48E-01	9.81E-01
**SMIM29**	0.00	9.91E-01	1.00E+00	0.06	8.48E-01	9.81E-01
**GAL3ST4**	0.07	7.93E-01	1.00E+00	0.06	8.48E-01	9.81E-01
**CEP290**	0.00	9.95E-01	1.00E+00	-0.06	8.48E-01	9.81E-01
**RP2**	0.29	3.98E-01	1.00E+00	0.05	8.48E-01	9.81E-01
**AC064801.2**	-0.17	9.51E-01	1.00E+00	0.61	8.48E-01	9.81E-01
**TIAM1**	-0.10	7.78E-01	1.00E+00	0.06	8.48E-01	9.81E-01
**XLOC_007377**	1.63	1.80E-01	1.00E+00	-0.18	8.48E-01	9.81E-01
**UTP20**	0.05	8.21E-01	1.00E+00	0.08	8.48E-01	9.81E-01
**IL6R-AS1**	-3.14	2.32E-03	2.43E-01	0.22	8.49E-01	9.81E-01

**GUCD1**	0.05	7.38E-01	1.00E+00	-0.09	8.49E-01	9.81E-01
**SLC25A30-AS1**	0.59	6.71E-01	1.00E+00	0.24	8.49E-01	9.81E-01
**BAIAP2L1**	0.24	2.71E-01	1.00E+00	0.08	8.49E-01	9.81E-01
**PCBP1-AS1**	-0.10	7.57E-01	1.00E+00	0.06	8.49E-01	9.81E-01
**AC008991.1**	-0.46	8.04E-01	1.00E+00	0.23	8.49E-01	9.81E-01
**AC100800.1**	-1.47	6.70E-01	1.00E+00	0.26	8.49E-01	9.81E-01
**DMXL1**	-0.01	9.63E-01	1.00E+00	-0.03	8.49E-01	9.81E-01
**FAM184A**	0.69	1.59E-01	1.00E+00	0.15	8.49E-01	9.81E-01
**RSL1D1**	-0.10	6.66E-01	1.00E+00	-0.06	8.49E-01	9.81E-01
**AL365259.1**	-1.07	5.09E-01	1.00E+00	0.22	8.49E-01	9.81E-01
**CENPCP1**	1.76	6.07E-01	1.00E+00	0.15	8.49E-01	9.81E-01
**BANF1P1**	-1.42	6.73E-01	1.00E+00	0.23	8.49E-01	9.81E-01
**AC083837.1**	-0.69	5.70E-01	1.00E+00	-0.22	8.49E-01	9.81E-01
**AL136454.1**	-0.25	8.18E-01	1.00E+00	-0.15	8.49E-01	9.81E-01
**RGL2**	0.08	7.09E-01	1.00E+00	0.05	8.49E-01	9.81E-01
**AP000763.2**	0.00	1.00E+00	NA	-0.61	8.49E-01	9.81E-01
**RFC3**	0.13	6.54E-01	1.00E+00	0.05	8.49E-01	9.81E-01
**CHD1L**	0.04	8.53E-01	1.00E+00	-0.03	8.49E-01	9.81E-01
**AC106798.1**	1.18	3.14E-01	1.00E+00	0.19	8.49E-01	9.81E-01
**PLA2G3**	-0.09	8.80E-01	1.00E+00	0.15	8.49E-01	9.81E-01
**ITGA2**	-0.35	8.53E-02	1.00E+00	0.08	8.49E-01	9.81E-01
**SAMD14**	-0.58	3.12E-01	1.00E+00	0.60	8.49E-01	9.81E-01
**DNA2**	-0.24	6.63E-01	1.00E+00	0.12	8.49E-01	9.81E-01
**BIN1**	0.42	4.53E-01	1.00E+00	0.10	8.49E-01	9.81E-01
**SRSF1**	0.00	9.89E-01	1.00E+00	-0.05	8.50E-01	9.81E-01
**AC105001.1**	-0.53	6.50E-01	1.00E+00	0.19	8.50E-01	9.81E-01
**AL391152.1**	-0.46	8.26E-01	1.00E+00	0.61	8.50E-01	9.81E-01
**ERP27**	0.93	1.78E-01	1.00E+00	-0.12	8.50E-01	9.81E-01
**NAPB**	-0.80	8.63E-02	1.00E+00	-0.10	8.50E-01	9.81E-01
**KLK1**	0.46	3.18E-01	1.00E+00	-0.12	8.50E-01	9.81E-01
**FAAP20**	-0.09	7.41E-01	1.00E+00	-0.07	8.50E-01	9.81E-01
**FIBCD1**	-1.16	2.37E-01	1.00E+00	0.28	8.50E-01	9.81E-01

**TGIF2**	0.04	8.65E-01	1.00E+00	-0.08	8.50E-01	9.81E-01
**SMIM10L2A**	0.01	9.86E-01	1.00E+00	-0.13	8.50E-01	9.81E-01
**AHNAK**	0.06	7.95E-01	1.00E+00	-0.08	8.50E-01	9.81E-01
**RPUSD1**	-0.52	8.56E-02	1.00E+00	0.07	8.50E-01	9.81E-01
**TBX3**	-0.01	9.88E-01	1.00E+00	-0.09	8.50E-01	9.81E-01
**YY2**	0.30	5.06E-01	1.00E+00	0.07	8.50E-01	9.81E-01
**ACSM2A**	-1.80	2.38E-01	1.00E+00	-0.31	8.50E-01	9.81E-01
**MOB3A**	-0.07	7.40E-01	1.00E+00	-0.07	8.50E-01	9.81E-01
**XLOC_008005**	-1.25	5.54E-01	1.00E+00	0.25	8.50E-01	9.81E-01
**ACSF2**	0.42	1.95E-01	1.00E+00	0.05	8.50E-01	9.81E-01
**PTBP1**	-0.34	1.69E-01	1.00E+00	-0.08	8.50E-01	9.81E-01
**HOOK2**	0.24	4.25E-01	1.00E+00	0.04	8.50E-01	9.81E-01
**BTF3P12**	NA	NA	NA	-0.22	8.50E-01	9.81E-01
**G38166**	-2.07	1.13E-01	1.00E+00	0.20	8.50E-01	9.81E-01
**PANX2**	-0.79	2.42E-01	1.00E+00	0.60	8.50E-01	9.81E-01
**ALG10**	0.25	5.01E-01	1.00E+00	-0.07	8.50E-01	9.81E-01
**EXOC3L1**	-0.57	5.10E-02	1.00E+00	0.08	8.50E-01	9.81E-01
**MRPL55**	-0.08	7.45E-01	1.00E+00	-0.06	8.50E-01	9.81E-01
**AL513477.1**	-0.81	7.65E-02	1.00E+00	-0.20	8.50E-01	9.81E-01
**IL12B**	-3.10	3.61E-01	1.00E+00	0.60	8.51E-01	9.81E-01
**TRIM3**	0.10	6.32E-01	1.00E+00	0.10	8.51E-01	9.81E-01
**TMLHE**	-0.16	5.06E-01	1.00E+00	-0.04	8.51E-01	9.81E-01
**AL139353.2**	-0.55	6.85E-01	1.00E+00	-0.13	8.51E-01	9.81E-01
**TRAF1**	-0.23	6.43E-01	1.00E+00	0.13	8.51E-01	9.81E-01
**ADGRG1**	-0.35	1.54E-01	1.00E+00	-0.07	8.51E-01	9.81E-01
**ALMS1**	-0.08	7.04E-01	1.00E+00	0.08	8.51E-01	9.81E-01
**UBE3C**	0.09	5.96E-01	1.00E+00	0.06	8.51E-01	9.81E-01
**RSL24D1P1**	NA	NA	NA	0.16	8.51E-01	9.81E-01
**OR7E121P**	1.17	4.61E-01	1.00E+00	0.21	8.51E-01	9.81E-01
**AL162293.1**	NA	NA	NA	-0.14	8.51E-01	9.81E-01
**IRX3**	-0.31	5.13E-01	1.00E+00	-0.10	8.51E-01	9.81E-01
**C17orf113**	-0.27	4.76E-01	1.00E+00	-0.15	8.51E-01	9.81E-01

**CEBPD**	-0.37	4.75E-01	1.00E+00	-0.14	8.51E-01	9.81E-01
**RITA1**	-0.20	4.19E-01	1.00E+00	0.03	8.51E-01	9.81E-01
**COL4A5**	-0.35	1.93E-01	1.00E+00	-0.07	8.51E-01	9.81E-01
**WWP2**	-0.22	2.99E-01	1.00E+00	0.07	8.51E-01	9.81E-01
**EGR1**	-1.58	3.40E-02	1.00E+00	0.11	8.51E-01	9.81E-01
**RPL35AP21**	NA	NA	NA	0.17	8.51E-01	9.81E-01
**BEND7**	-0.13	8.40E-01	1.00E+00	-0.13	8.51E-01	9.81E-01
**PNMA8A**	-0.01	9.80E-01	1.00E+00	0.14	8.51E-01	9.81E-01
**AC104260.2**	-0.09	9.05E-01	1.00E+00	-0.23	8.51E-01	9.81E-01
**AUTS2**	-0.39	1.06E-01	1.00E+00	-0.08	8.51E-01	9.81E-01
**DPYSL2**	0.38	2.89E-01	1.00E+00	-0.07	8.52E-01	9.82E-01
**RFWD3**	-0.28	1.93E-01	1.00E+00	0.06	8.52E-01	9.82E-01
**AL035425.1**	-1.45	1.57E-01	1.00E+00	0.24	8.52E-01	9.82E-01
**DHFR**	-0.39	2.34E-01	1.00E+00	-0.10	8.52E-01	9.82E-01
**LINC00173**	1.48	3.15E-01	1.00E+00	0.23	8.52E-01	9.82E-01
**AL845472.2**	-0.41	5.40E-01	1.00E+00	-0.12	8.52E-01	9.82E-01
**CLNS1A**	0.05	7.86E-01	1.00E+00	0.04	8.52E-01	9.82E-01
**KIAA1257**	-0.54	2.28E-01	1.00E+00	-0.10	8.52E-01	9.82E-01
**BCDIN3D**	0.54	1.35E-01	1.00E+00	-0.07	8.52E-01	9.82E-01
**MUC4**	-1.90	2.61E-01	1.00E+00	-0.25	8.52E-01	9.82E-01
**NOP14**	-0.35	2.21E-01	1.00E+00	-0.08	8.52E-01	9.82E-01
**AC022440.1**	-0.71	7.14E-01	1.00E+00	-0.60	8.52E-01	9.82E-01
**SGSM3**	-1.27	7.07E-01	1.00E+00	0.13	8.52E-01	9.82E-01
**CSPG4P8**	-2.44	4.43E-02	1.00E+00	-0.21	8.52E-01	9.82E-01
**G4754**	1.05	5.30E-02	1.00E+00	0.08	8.52E-01	9.82E-01
**GOT2P3**	-1.84	5.95E-01	1.00E+00	0.17	8.52E-01	9.82E-01
**PDZD9**	0.66	7.03E-01	1.00E+00	-0.31	8.52E-01	9.82E-01
**HIST1H4C**	-1.84	3.87E-02	1.00E+00	-0.58	8.52E-01	9.82E-01
**FTO**	-0.04	8.76E-01	1.00E+00	-0.06	8.53E-01	9.82E-01
**DPT**	1.66	4.00E-03	3.52E-01	-0.11	8.53E-01	9.82E-01
**TMED7**	0.14	5.52E-01	1.00E+00	-0.04	8.53E-01	9.82E-01
**NR2F1-AS1**	0.40	5.34E-01	1.00E+00	-0.13	8.53E-01	9.82E-01

**KRT40**	0.11	9.57E-01	1.00E+00	-0.59	8.53E-01	9.82E-01
**TADA2A**	-0.43	6.67E-02	1.00E+00	0.07	8.53E-01	9.82E-01
**RASEF**	0.37	4.10E-01	1.00E+00	0.09	8.53E-01	9.82E-01
**AL355001.2**	-0.52	9.29E-02	1.00E+00	0.10	8.53E-01	9.82E-01
**TMEM144**	-0.38	1.16E-01	1.00E+00	-0.05	8.53E-01	9.82E-01
**DNAJC6**	-1.03	3.83E-02	1.00E+00	-0.12	8.53E-01	9.82E-01
**G34428**	-0.69	3.78E-01	1.00E+00	-0.15	8.53E-01	9.82E-01
**AC005730.2**	-0.96	4.68E-01	1.00E+00	-0.24	8.53E-01	9.82E-01
**RPL36A**	-1.29	1.21E-01	1.00E+00	-0.08	8.53E-01	9.82E-01
**KAT6A**	-0.08	7.36E-01	1.00E+00	-0.08	8.53E-01	9.82E-01
**G5880**	-1.45	5.40E-01	1.00E+00	-0.35	8.53E-01	9.82E-01
**AL451074.6**	-0.20	9.53E-01	1.00E+00	-0.29	8.53E-01	9.82E-01
**AC008966.2**	1.65	4.67E-01	1.00E+00	0.23	8.53E-01	9.82E-01
**KRTAP10-7**	-0.56	8.70E-01	1.00E+00	-0.59	8.53E-01	9.82E-01
**TDRD7**	0.23	5.82E-01	1.00E+00	0.12	8.54E-01	9.82E-01
**DNAJB9**	-0.11	6.86E-01	1.00E+00	-0.06	8.54E-01	9.82E-01
**SMN1**	0.26	3.72E-01	1.00E+00	-0.04	8.54E-01	9.82E-01
**TKT**	0.00	9.99E-01	1.00E+00	0.07	8.54E-01	9.82E-01
**CCDC6**	-0.01	9.56E-01	1.00E+00	-0.04	8.54E-01	9.82E-01
**C3orf79**	-0.48	8.79E-01	1.00E+00	-0.28	8.54E-01	9.82E-01
**C1QBP**	-0.30	1.04E-01	1.00E+00	-0.04	8.54E-01	9.82E-01
**C8orf31**	0.89	3.31E-01	1.00E+00	0.52	8.54E-01	9.82E-01
**UBBP4**	0.46	4.06E-01	1.00E+00	-0.10	8.54E-01	9.82E-01
**PRR5L**	0.32	5.44E-01	1.00E+00	0.14	8.54E-01	9.82E-01
**KRTAP4-4**	-29.80	1.11E-17	8.83E-15	-0.59	8.54E-01	9.82E-01
**FBN3**	0.05	9.67E-01	1.00E+00	0.34	8.54E-01	9.82E-01
**PDCD1**	-2.18	3.28E-02	1.00E+00	0.58	8.54E-01	9.82E-01
**ZNF180**	0.09	7.38E-01	1.00E+00	0.05	8.54E-01	9.82E-01
**ST6GALNAC3**	0.77	1.10E-01	1.00E+00	0.10	8.54E-01	9.82E-01
**AL391097.2**	0.25	9.27E-01	1.00E+00	-0.20	8.54E-01	9.82E-01
**ARPC3**	-0.12	5.79E-01	1.00E+00	0.03	8.54E-01	9.82E-01
**G2196**	-1.19	2.32E-01	1.00E+00	-0.17	8.54E-01	9.82E-01

**PRELID1P1**	1.02	7.67E-01	1.00E+00	-0.11	8.54E-01	9.82E-01
**DSP**	-0.26	5.50E-01	1.00E+00	-0.10	8.54E-01	9.82E-01
**OPHN1**	0.29	3.86E-01	1.00E+00	-0.09	8.54E-01	9.82E-01
**HASPIN**	-0.42	5.25E-01	1.00E+00	0.15	8.54E-01	9.82E-01
**TSR1**	-0.09	6.88E-01	1.00E+00	0.04	8.54E-01	9.82E-01
**NOP53**	-0.21	5.13E-01	1.00E+00	0.14	8.55E-01	9.82E-01
**PRR26**	0.30	6.74E-01	1.00E+00	0.42	8.55E-01	9.82E-01
**G42804**	-2.08	1.92E-01	1.00E+00	-0.25	8.55E-01	9.82E-01
**EXOC2**	-0.09	6.27E-01	1.00E+00	0.03	8.55E-01	9.82E-01
**KLHL21**	-0.13	6.55E-01	1.00E+00	0.07	8.55E-01	9.82E-01
**SUDS3P1**	0.01	9.96E-01	1.00E+00	-0.09	8.55E-01	9.82E-01
**C5orf38**	-0.21	5.81E-01	1.00E+00	0.11	8.55E-01	9.82E-01
**AF064858.1**	-0.31	7.08E-01	1.00E+00	-0.13	8.55E-01	9.82E-01
**NRGN**	-1.40	4.79E-02	1.00E+00	0.12	8.55E-01	9.82E-01
**AC104667.2**	0.21	8.80E-01	1.00E+00	0.18	8.55E-01	9.82E-01
**IQCH**	-0.29	5.74E-01	1.00E+00	-0.12	8.55E-01	9.82E-01
**EML5**	0.88	2.05E-01	1.00E+00	0.21	8.55E-01	9.82E-01
**ACTR3**	0.15	6.10E-01	1.00E+00	0.03	8.55E-01	9.82E-01
**HAS2-AS1**	-0.79	3.91E-01	1.00E+00	-0.13	8.55E-01	9.82E-01
**GPALPP1**	-0.12	4.98E-01	1.00E+00	-0.03	8.55E-01	9.82E-01
**CASP1P2**	-0.50	5.03E-01	1.00E+00	-0.13	8.55E-01	9.82E-01
**CCDC144A**	-3.24	2.97E-02	9.97E-01	-0.23	8.55E-01	9.82E-01
**RPS26P13**	0.25	9.42E-01	1.00E+00	0.16	8.55E-01	9.82E-01
**LTF**	-1.99	9.95E-02	1.00E+00	-0.57	8.55E-01	9.82E-01
**IL1RN**	0.17	6.72E-01	1.00E+00	-0.09	8.55E-01	9.82E-01
**AP005264.7**	-1.18	4.29E-01	1.00E+00	0.20	8.56E-01	9.82E-01
**LRRC29**	0.00	9.98E-01	1.00E+00	0.07	8.56E-01	9.82E-01
**LINC00484**	-0.83	5.24E-01	1.00E+00	0.10	8.56E-01	9.82E-01
**SERPINB10**	-0.12	8.56E-01	1.00E+00	0.15	8.56E-01	9.82E-01
**ZNF688**	-0.11	6.39E-01	1.00E+00	-0.10	8.56E-01	9.82E-01
**BRD3OS**	-0.12	6.95E-01	1.00E+00	0.05	8.56E-01	9.82E-01
**GARNL3**	-0.04	9.21E-01	1.00E+00	-0.08	8.56E-01	9.82E-01

**GPRC5D-AS1**	0.35	4.04E-01	1.00E+00	0.10	8.56E-01	9.82E-01
**ANO9**	-0.18	7.54E-01	1.00E+00	-0.11	8.56E-01	9.82E-01
**FAM214A**	0.19	4.60E-01	1.00E+00	0.04	8.56E-01	9.82E-01
**LINC02257**	-1.52	6.58E-01	1.00E+00	0.31	8.56E-01	9.82E-01
**AC018628.2**	-0.10	9.77E-01	1.00E+00	0.17	8.56E-01	9.82E-01
**ADGRB3**	-0.39	5.29E-01	1.00E+00	0.13	8.56E-01	9.82E-01
**PALLD**	-0.26	4.68E-01	1.00E+00	0.05	8.56E-01	9.82E-01
**OR7C1**	0.32	7.27E-01	1.00E+00	0.14	8.56E-01	9.82E-01
**EREG**	-0.27	7.42E-01	1.00E+00	-0.13	8.56E-01	9.82E-01
**TMEM185B**	0.09	7.23E-01	1.00E+00	-0.10	8.56E-01	9.82E-01
**XLOC_009185**	-1.35	9.47E-02	1.00E+00	-0.12	8.56E-01	9.82E-01
**LINC01547**	-0.55	9.67E-02	1.00E+00	-0.07	8.56E-01	9.82E-01
**AL353662.1**	3.43	3.01E-01	1.00E+00	-0.31	8.56E-01	9.82E-01
**G39423**	0.36	7.16E-01	1.00E+00	0.17	8.56E-01	9.82E-01
**HEXIM2**	0.06	9.24E-01	1.00E+00	0.07	8.56E-01	9.82E-01
**SPDYE1**	0.81	5.81E-01	1.00E+00	0.14	8.56E-01	9.82E-01
**RSPO1**	0.14	6.22E-01	1.00E+00	-0.11	8.56E-01	9.82E-01
**AL513175.1**	-0.06	9.86E-01	1.00E+00	-0.13	8.56E-01	9.82E-01
**FABP6**	0.23	8.28E-01	1.00E+00	0.16	8.56E-01	9.82E-01
**AC136632.1**	0.06	9.31E-01	1.00E+00	0.09	8.57E-01	9.82E-01
**CKAP5**	-0.14	3.67E-01	1.00E+00	-0.04	8.57E-01	9.82E-01
**AC117489.1**	0.14	8.82E-01	1.00E+00	0.20	8.57E-01	9.82E-01
**AC127502.2**	0.03	9.55E-01	1.00E+00	0.13	8.57E-01	9.82E-01
**HIST2H2BD**	-0.63	5.88E-01	1.00E+00	-0.10	8.57E-01	9.82E-01
**AC100801.1**	1.58	6.44E-01	1.00E+00	0.22	8.57E-01	9.82E-01
**G3148**	-2.68	2.78E-01	1.00E+00	-0.24	8.57E-01	9.82E-01
**PRPSAP2**	-0.02	9.20E-01	1.00E+00	0.04	8.57E-01	9.82E-01
**BACH1**	-0.08	8.08E-01	1.00E+00	0.05	8.57E-01	9.82E-01
**STOML3**	-1.67	3.44E-01	1.00E+00	0.30	8.57E-01	9.82E-01
**LRRC37A17P**	0.29	5.07E-01	1.00E+00	-0.08	8.57E-01	9.82E-01
**EIF2AK4**	-0.03	8.76E-01	1.00E+00	0.06	8.57E-01	9.82E-01
**FZD1**	0.04	9.19E-01	1.00E+00	0.09	8.57E-01	9.82E-01

**MYO16**	0.58	4.84E-01	1.00E+00	0.16	8.57E-01	9.82E-01
**CLCN1**	-1.52	3.11E-01	1.00E+00	0.21	8.57E-01	9.82E-01
**GYPC**	0.50	2.59E-01	1.00E+00	0.08	8.57E-01	9.82E-01
**ZNF665**	0.03	9.28E-01	1.00E+00	-0.08	8.57E-01	9.82E-01
**BLMH**	-0.25	6.57E-01	1.00E+00	-0.09	8.57E-01	9.82E-01
**FLNC**	-0.40	4.95E-01	1.00E+00	0.13	8.57E-01	9.82E-01
**ZNF714**	-0.17	6.62E-01	1.00E+00	0.07	8.57E-01	9.82E-01
**RANGAP1**	-0.26	4.59E-01	1.00E+00	0.08	8.57E-01	9.82E-01
**MIR203**	-0.51	5.93E-01	1.00E+00	0.24	8.57E-01	9.82E-01
**RP11-111G23.1**	0.09	8.93E-01	1.00E+00	0.10	8.57E-01	9.82E-01
**CPSF3**	0.15	5.03E-01	1.00E+00	-0.04	8.57E-01	9.82E-01
**MZF1-AS1**	-0.65	2.43E-01	1.00E+00	-0.08	8.57E-01	9.82E-01
**ABCC4**	-0.54	1.73E-01	1.00E+00	-0.07	8.57E-01	9.82E-01
**MRPS34**	-0.18	4.57E-01	1.00E+00	-0.04	8.58E-01	9.82E-01
**AC025678.3**	-2.49	1.91E-01	1.00E+00	0.14	8.58E-01	9.82E-01
**AC007743.1**	0.77	2.44E-01	1.00E+00	0.14	8.58E-01	9.82E-01
**NDC80**	-0.17	6.23E-01	1.00E+00	0.07	8.58E-01	9.82E-01
**AC135178.5**	-1.11	2.89E-01	1.00E+00	0.16	8.58E-01	9.82E-01
**AL035587.1**	-0.71	3.44E-01	1.00E+00	0.11	8.58E-01	9.82E-01
**KRTAP19-3**	-0.76	8.28E-01	1.00E+00	0.57	8.58E-01	9.82E-01
**ANK3**	-0.10	7.54E-01	1.00E+00	-0.07	8.58E-01	9.82E-01
**PPM1A**	0.05	8.24E-01	1.00E+00	0.04	8.58E-01	9.82E-01
**LSM2**	-0.18	2.93E-01	1.00E+00	-0.04	8.58E-01	9.82E-01
**TOMM20**	0.04	8.34E-01	1.00E+00	-0.02	8.58E-01	9.82E-01
**ZNF681**	0.48	2.38E-01	1.00E+00	-0.10	8.58E-01	9.82E-01
**FOXF1**	-0.65	4.66E-01	1.00E+00	0.56	8.58E-01	9.82E-01
**MSL3P1**	-2.84	1.12E-01	1.00E+00	0.16	8.58E-01	9.82E-01
**AC092597.1**	-0.33	8.68E-01	1.00E+00	0.18	8.58E-01	9.82E-01
**GLB1L2**	0.14	6.82E-01	1.00E+00	0.06	8.58E-01	9.82E-01
**AC116351.2**	0.00	9.95E-01	1.00E+00	0.18	8.58E-01	9.82E-01
**AL031717.1**	-0.68	7.65E-01	1.00E+00	-0.26	8.58E-01	9.82E-01
**CASK**	-0.06	7.94E-01	1.00E+00	-0.07	8.58E-01	9.82E-01

**KCNS3**	0.12	5.73E-01	1.00E+00	-0.05	8.58E-01	9.82E-01
**XLOC_014104**	-1.94	1.31E-02	7.22E-01	-0.12	8.58E-01	9.82E-01
**GUSBP5**	0.28	6.87E-01	1.00E+00	-0.09	8.58E-01	9.82E-01
**PAEP**	-1.69	3.24E-01	1.00E+00	0.26	8.58E-01	9.82E-01
**AC093535.1**	1.96	2.25E-01	1.00E+00	-0.15	8.58E-01	9.82E-01
**MIR210**	-1.13	3.81E-01	1.00E+00	-0.25	8.59E-01	9.82E-01
**FITM2**	0.23	3.90E-01	1.00E+00	0.06	8.59E-01	9.82E-01
**SNHG5**	0.01	9.92E-01	1.00E+00	0.08	8.59E-01	9.82E-01
**NUP35**	0.15	5.60E-01	1.00E+00	-0.06	8.59E-01	9.82E-01
**RALBP1**	0.18	5.11E-01	1.00E+00	0.05	8.59E-01	9.82E-01
**SNCA**	0.04	8.83E-01	1.00E+00	-0.07	8.59E-01	9.82E-01
**KIAA1211L**	-0.35	1.43E-01	1.00E+00	-0.12	8.59E-01	9.82E-01
**ABCA10**	0.65	4.09E-01	1.00E+00	-0.11	8.59E-01	9.82E-01
**CDH19**	0.23	6.37E-01	1.00E+00	0.08	8.59E-01	9.82E-01
**TMCO4**	-0.22	5.38E-01	1.00E+00	0.09	8.59E-01	9.82E-01
**PAFAH1B2**	0.23	2.92E-01	1.00E+00	-0.05	8.59E-01	9.82E-01
**GGH**	0.12	7.78E-01	1.00E+00	-0.07	8.59E-01	9.82E-01
**AC020978.7**	-0.30	9.25E-01	1.00E+00	-0.23	8.59E-01	9.82E-01
**NCK2**	-0.21	2.99E-01	1.00E+00	0.08	8.59E-01	9.82E-01
**XLOC_007348**	0.65	1.85E-01	1.00E+00	-0.13	8.59E-01	9.82E-01
**AC133552.5**	-0.21	6.27E-01	1.00E+00	0.07	8.59E-01	9.82E-01
**SNORA84**	NA	NA	NA	-0.19	8.59E-01	9.82E-01
**THRB-AS1**	0.24	8.18E-01	1.00E+00	-0.12	8.59E-01	9.82E-01
**CD44**	-0.25	3.04E-01	1.00E+00	0.04	8.59E-01	9.82E-01
**AC092902.5**	2.11	2.27E-01	1.00E+00	0.15	8.59E-01	9.82E-01
**ADCY4**	0.04	9.27E-01	1.00E+00	-0.12	8.59E-01	9.82E-01
**C19orf54**	-0.33	2.03E-01	1.00E+00	0.07	8.59E-01	9.82E-01
**NUP43**	0.15	6.73E-01	1.00E+00	-0.06	8.59E-01	9.82E-01
**TTC36**	0.86	5.24E-01	1.00E+00	0.19	8.59E-01	9.82E-01
**ZNF775**	-0.23	5.58E-01	1.00E+00	0.16	8.60E-01	9.82E-01
**NOP16**	-0.35	3.46E-01	1.00E+00	0.06	8.60E-01	9.82E-01
**SCD5**	-0.13	7.46E-01	1.00E+00	-0.07	8.60E-01	9.82E-01

**AC243960.3**	-3.61	1.33E-02	7.22E-01	0.41	8.60E-01	9.82E-01
**MYBL2**	-0.65	6.30E-02	1.00E+00	0.12	8.60E-01	9.82E-01
**AC139795.1**	-0.16	7.06E-01	1.00E+00	0.11	8.60E-01	9.82E-01
**RBMS1**	0.09	6.50E-01	1.00E+00	-0.04	8.60E-01	9.82E-01
**YWHAQP6**	1.39	6.86E-01	1.00E+00	-0.18	8.60E-01	9.82E-01
**AC136475.3**	-1.85	1.76E-01	1.00E+00	0.19	8.60E-01	9.82E-01
**GPBP1L1**	0.10	6.57E-01	1.00E+00	-0.03	8.60E-01	9.82E-01
**AL138689.2**	-0.80	7.75E-01	1.00E+00	-0.19	8.60E-01	9.82E-01
**KRTAP4-11**	-1.17	6.78E-01	1.00E+00	-0.57	8.60E-01	9.82E-01
**POU6F1**	-0.43	1.13E-01	1.00E+00	0.09	8.60E-01	9.82E-01
**GAS6-DT**	0.17	7.04E-01	1.00E+00	-0.07	8.60E-01	9.82E-01
**SOX13**	0.09	8.36E-01	1.00E+00	0.13	8.60E-01	9.82E-01
**MDM4**	0.08	8.16E-01	1.00E+00	0.05	8.60E-01	9.82E-01
**G42644**	1.18	4.65E-01	1.00E+00	-0.17	8.60E-01	9.82E-01
**C3orf52**	0.38	3.92E-01	1.00E+00	0.11	8.60E-01	9.82E-01
**TTC9B**	-3.77	1.01E-04	2.46E-02	0.18	8.60E-01	9.82E-01
**AC073621.1**	0.49	6.84E-01	1.00E+00	-0.23	8.60E-01	9.82E-01
**AKR1E2**	0.02	9.60E-01	1.00E+00	0.10	8.60E-01	9.82E-01
**OTUD4**	0.10	6.98E-01	1.00E+00	0.06	8.60E-01	9.82E-01
**CDC42EP2**	0.56	8.71E-01	1.00E+00	-0.19	8.61E-01	9.82E-01
**GABRB2**	0.02	9.79E-01	1.00E+00	-0.54	8.61E-01	9.82E-01
**PPFIA1**	0.11	5.98E-01	1.00E+00	0.05	8.61E-01	9.82E-01
**RHOQP2**	-0.92	3.30E-01	1.00E+00	-0.13	8.61E-01	9.82E-01
**CUTC**	0.14	6.36E-01	1.00E+00	-0.05	8.61E-01	9.82E-01
**ARHGEF6**	1.07	8.68E-02	1.00E+00	0.10	8.61E-01	9.82E-01
**CSRNP1**	-0.92	2.00E-01	1.00E+00	-0.10	8.61E-01	9.82E-01
**NRAV**	0.32	1.17E-01	1.00E+00	0.04	8.61E-01	9.82E-01
**AC007920.2**	-0.03	9.74E-01	1.00E+00	0.16	8.61E-01	9.82E-01
**SPATA20**	-0.38	4.66E-01	1.00E+00	0.06	8.61E-01	9.82E-01
**LACTB2-AS1**	0.63	8.54E-01	1.00E+00	-0.17	8.61E-01	9.82E-01
**KANSL3**	0.20	5.08E-01	1.00E+00	0.07	8.61E-01	9.82E-01
**SGCE**	0.60	1.52E-01	1.00E+00	-0.07	8.61E-01	9.82E-01

**DNAH17**	-1.04	1.01E-01	1.00E+00	0.13	8.61E-01	9.82E-01
**RNF225**	-0.53	4.33E-01	1.00E+00	-0.55	8.61E-01	9.82E-01
**EFCAB14**	0.08	5.74E-01	1.00E+00	0.03	8.61E-01	9.82E-01
**AC024145.1**	0.26	7.97E-01	1.00E+00	0.13	8.61E-01	9.82E-01
**NAB1**	0.37	2.94E-01	1.00E+00	0.04	8.61E-01	9.83E-01
**BLOC1S1-RDH5**	-1.36	3.21E-01	1.00E+00	0.13	8.61E-01	9.83E-01
**SMPD4**	-0.34	1.89E-01	1.00E+00	0.07	8.61E-01	9.83E-01
**NSUN3**	-0.04	8.81E-01	1.00E+00	-0.04	8.61E-01	9.83E-01
**HEATR5B**	0.18	4.80E-01	1.00E+00	0.06	8.61E-01	9.83E-01
**PRRT3**	-0.31	2.69E-01	1.00E+00	-0.06	8.61E-01	9.83E-01
**ASPSCR1**	-0.33	2.42E-01	1.00E+00	-0.08	8.61E-01	9.83E-01
**AC027559.1**	1.22	2.55E-01	1.00E+00	-0.13	8.61E-01	9.83E-01
**AC104574.2**	0.76	8.27E-01	1.00E+00	0.21	8.62E-01	9.83E-01
**CIAO2B**	-0.10	5.44E-01	1.00E+00	0.04	8.62E-01	9.83E-01
**VPS26C**	0.16	3.80E-01	1.00E+00	-0.03	8.62E-01	9.83E-01
**RPS26P8**	1.42	2.68E-01	1.00E+00	0.15	8.62E-01	9.83E-01
**SUB1P1**	-1.08	3.25E-01	1.00E+00	0.13	8.62E-01	9.83E-01
**AC108134.3**	0.69	2.09E-01	1.00E+00	0.16	8.62E-01	9.83E-01
**LINC00958**	-0.24	6.83E-01	1.00E+00	-0.11	8.62E-01	9.83E-01
**GGA2**	0.10	7.04E-01	1.00E+00	-0.06	8.62E-01	9.83E-01
**HEY2**	0.06	9.13E-01	1.00E+00	0.09	8.62E-01	9.83E-01
**IDE**	0.04	9.48E-01	1.00E+00	-0.06	8.62E-01	9.83E-01
**AC116565.1**	-1.16	1.75E-02	8.33E-01	0.17	8.62E-01	9.83E-01
**MMACHC**	-0.18	5.85E-01	1.00E+00	0.08	8.62E-01	9.83E-01
**AL136981.2**	-0.36	6.84E-01	1.00E+00	-0.15	8.62E-01	9.83E-01
**LY6D**	-0.19	6.84E-01	1.00E+00	-0.12	8.62E-01	9.83E-01
**SRP14**	0.13	3.69E-01	1.00E+00	-0.04	8.62E-01	9.83E-01
**C1orf116**	0.16	5.17E-01	1.00E+00	-0.09	8.62E-01	9.83E-01
**PITPNA-AS1**	-0.18	9.33E-01	1.00E+00	-0.14	8.62E-01	9.83E-01
**BPI**	-1.27	7.07E-01	1.00E+00	-0.34	8.63E-01	9.83E-01
**GSG1**	1.59	3.90E-01	1.00E+00	0.13	8.63E-01	9.83E-01
**YWHAZ**	-0.14	6.46E-01	1.00E+00	-0.03	8.63E-01	9.83E-01

**G24891**	-0.45	4.33E-01	1.00E+00	0.11	8.63E-01	9.83E-01
**XLOC_003061**	0.78	3.53E-01	1.00E+00	0.40	8.63E-01	9.83E-01
**TRIAP1**	0.12	6.71E-01	1.00E+00	-0.06	8.63E-01	9.83E-01
**LINC02407**	0.78	7.44E-01	1.00E+00	0.24	8.63E-01	9.83E-01
**RFC2**	-0.20	4.14E-01	1.00E+00	-0.04	8.63E-01	9.83E-01
**XLOC_001532**	-0.44	6.31E-01	1.00E+00	-0.18	8.63E-01	9.83E-01
**DDX10**	-0.11	6.59E-01	1.00E+00	-0.04	8.63E-01	9.83E-01
**SLURP1**	-0.17	7.66E-01	1.00E+00	0.14	8.63E-01	9.83E-01
**CORO2B**	-0.04	9.21E-01	1.00E+00	-0.10	8.63E-01	9.83E-01
**SLC2A12**	-0.08	8.06E-01	1.00E+00	-0.11	8.63E-01	9.83E-01
**RPS20P22**	1.44	3.49E-01	1.00E+00	0.18	8.63E-01	9.83E-01
**DNAJC3**	0.06	7.53E-01	1.00E+00	-0.04	8.63E-01	9.83E-01
**XLOC_001460**	2.49	4.64E-01	1.00E+00	0.35	8.63E-01	9.83E-01
**ZFHX2-AS1**	1.04	5.07E-01	1.00E+00	0.14	8.63E-01	9.83E-01
**XLOC_002433**	0.26	8.70E-01	1.00E+00	0.20	8.63E-01	9.83E-01
**RPL17P36**	-0.09	9.72E-01	1.00E+00	-0.14	8.63E-01	9.83E-01
**PTPN2**	0.06	7.35E-01	1.00E+00	-0.03	8.63E-01	9.83E-01
**CSNK2A3**	-0.03	9.82E-01	1.00E+00	0.07	8.63E-01	9.83E-01
**KLHL34**	0.19	7.78E-01	1.00E+00	0.20	8.63E-01	9.83E-01
**AC090616.6**	-0.71	5.41E-01	1.00E+00	-0.14	8.64E-01	9.83E-01
**AC009506.1**	-0.12	7.48E-01	1.00E+00	0.05	8.64E-01	9.83E-01
**CUL1**	-0.11	5.83E-01	1.00E+00	-0.02	8.64E-01	9.83E-01
**AC090673.1**	-0.41	8.13E-01	1.00E+00	-0.17	8.64E-01	9.83E-01
**C16orf89**	0.57	5.78E-01	1.00E+00	-0.41	8.64E-01	9.83E-01
**AL117379.1**	0.63	4.71E-01	1.00E+00	-0.16	8.64E-01	9.84E-01
**AC009065.8**	0.14	9.02E-01	1.00E+00	-0.16	8.64E-01	9.84E-01
**G10911**	-1.01	2.72E-01	1.00E+00	0.26	8.64E-01	9.84E-01
**RAMAC**	0.20	5.48E-01	1.00E+00	0.07	8.64E-01	9.84E-01
**AL031283.3**	-2.67	2.24E-01	1.00E+00	0.23	8.64E-01	9.84E-01
**PCDHB4**	-0.19	6.96E-01	1.00E+00	-0.10	8.64E-01	9.84E-01
**PLPP2**	-0.13	7.49E-01	1.00E+00	-0.06	8.65E-01	9.84E-01
**FMO1**	-0.39	4.11E-01	1.00E+00	-0.09	8.65E-01	9.84E-01

**AL158163.1**	0.33	7.52E-01	1.00E+00	-0.13	8.65E-01	9.84E-01
**LGALSL-DT**	0.08	9.11E-01	1.00E+00	0.14	8.65E-01	9.84E-01
**SEMA4F**	0.33	4.08E-01	1.00E+00	0.11	8.65E-01	9.84E-01
**ATP2A3**	0.28	7.29E-01	1.00E+00	-0.12	8.65E-01	9.84E-01
**OVCH2**	-0.27	7.88E-01	1.00E+00	0.14	8.65E-01	9.84E-01
**MAB21L4**	-0.54	2.40E-01	1.00E+00	0.11	8.65E-01	9.84E-01
**CFAP97D1**	-0.10	9.73E-01	1.00E+00	-0.33	8.65E-01	9.84E-01
**TMEM154**	0.17	7.08E-01	1.00E+00	-0.09	8.65E-01	9.84E-01
**PRKD3**	0.54	2.12E-02	8.94E-01	-0.06	8.65E-01	9.84E-01
**TRIM58**	0.18	8.00E-01	1.00E+00	-0.15	8.65E-01	9.84E-01
**AL096870.2**	-2.28	1.52E-01	1.00E+00	0.12	8.65E-01	9.84E-01
**ASGR2**	0.19	7.28E-01	1.00E+00	0.08	8.65E-01	9.84E-01
**TONSL**	-0.58	3.86E-02	1.00E+00	0.10	8.65E-01	9.84E-01
**AUP1**	-0.21	3.71E-01	1.00E+00	-0.03	8.65E-01	9.84E-01
**AC010889.1**	0.15	9.65E-01	1.00E+00	0.32	8.65E-01	9.84E-01
**OCEL1**	0.20	5.73E-01	1.00E+00	0.05	8.65E-01	9.84E-01
**MAP1B**	0.16	6.81E-01	1.00E+00	-0.06	8.65E-01	9.84E-01
**RSL24D1**	0.13	5.79E-01	1.00E+00	-0.05	8.66E-01	9.84E-01
**AP3B2**	-1.91	2.78E-05	7.87E-03	-0.13	8.66E-01	9.84E-01
**LINC02518**	-1.12	3.87E-01	1.00E+00	0.18	8.66E-01	9.84E-01
**TCP1**	0.00	9.96E-01	1.00E+00	-0.03	8.66E-01	9.84E-01
**DNAJC14**	0.00	9.90E-01	1.00E+00	-0.05	8.66E-01	9.84E-01
**RPN2**	-0.05	7.44E-01	1.00E+00	0.02	8.66E-01	9.84E-01
**LINC00639**	0.80	3.10E-01	1.00E+00	0.21	8.66E-01	9.84E-01
**KMT2A**	-0.11	6.33E-01	1.00E+00	0.06	8.66E-01	9.84E-01
**P4HB**	-0.07	7.26E-01	1.00E+00	0.04	8.66E-01	9.84E-01
**NBEA**	0.25	6.58E-01	1.00E+00	0.09	8.66E-01	9.84E-01
**AL137077.2**	0.78	2.73E-01	1.00E+00	-0.16	8.66E-01	9.84E-01
**AC005383.1**	-0.19	8.68E-01	1.00E+00	0.20	8.66E-01	9.84E-01
**AL360270.1**	0.12	8.95E-01	1.00E+00	0.15	8.66E-01	9.84E-01
**RIF1**	-0.10	6.75E-01	1.00E+00	-0.04	8.66E-01	9.84E-01
**KLC2**	-0.57	9.33E-03	6.05E-01	-0.09	8.66E-01	9.84E-01

**WDR70**	0.06	7.57E-01	1.00E+00	-0.02	8.66E-01	9.84E-01
**AC068338.2**	0.23	6.23E-01	1.00E+00	-0.08	8.66E-01	9.84E-01
**PCDHGA8**	-1.05	1.57E-01	1.00E+00	0.15	8.66E-01	9.84E-01
**TMCC3**	0.01	9.75E-01	1.00E+00	0.06	8.66E-01	9.84E-01
**ARMC4**	0.29	7.58E-01	1.00E+00	0.45	8.66E-01	9.84E-01
**ZNF524**	0.05	8.35E-01	1.00E+00	0.04	8.66E-01	9.84E-01
**KCTD13**	-0.54	6.03E-02	1.00E+00	-0.06	8.67E-01	9.84E-01
**DOK7**	-2.06	4.85E-02	1.00E+00	0.17	8.67E-01	9.84E-01
**SYNJ1**	-0.28	5.16E-01	1.00E+00	0.09	8.67E-01	9.84E-01
**GNPNAT1**	-0.19	3.83E-01	1.00E+00	-0.04	8.67E-01	9.84E-01
**LINC02202**	0.86	9.78E-02	1.00E+00	0.09	8.67E-01	9.84E-01
**TTLL4**	-0.41	8.02E-02	1.00E+00	0.09	8.67E-01	9.84E-01
**ABI2**	0.14	6.20E-01	1.00E+00	-0.03	8.67E-01	9.84E-01
**MBIP**	0.03	8.98E-01	1.00E+00	-0.06	8.67E-01	9.84E-01
**COL25A1**	-0.14	8.16E-01	1.00E+00	0.13	8.67E-01	9.84E-01
**HAGLR**	-0.19	7.23E-01	1.00E+00	0.12	8.67E-01	9.84E-01
**NDUFS8**	0.02	9.49E-01	1.00E+00	-0.05	8.67E-01	9.84E-01
**PRR14L**	-0.13	5.08E-01	1.00E+00	-0.04	8.67E-01	9.84E-01
**RELN**	0.41	4.45E-01	1.00E+00	0.11	8.67E-01	9.84E-01
**CRYZ**	-0.13	6.28E-01	1.00E+00	0.05	8.67E-01	9.84E-01
**AC005072.1**	0.92	6.30E-01	1.00E+00	-0.17	8.67E-01	9.84E-01
**AC020571.1**	1.86	1.37E-01	1.00E+00	0.17	8.67E-01	9.84E-01
**AC100830.1**	-0.47	6.52E-01	1.00E+00	-0.19	8.67E-01	9.84E-01
**CENPL**	-0.11	6.62E-01	1.00E+00	-0.06	8.67E-01	9.84E-01
**CCDC84**	0.10	8.27E-01	1.00E+00	-0.05	8.67E-01	9.84E-01
**XLOC_009389**	-0.96	1.80E-01	1.00E+00	0.14	8.67E-01	9.84E-01
**AC096720.2**	-0.32	9.09E-01	1.00E+00	0.20	8.67E-01	9.84E-01
**COPZ1**	0.00	9.99E-01	1.00E+00	0.02	8.67E-01	9.84E-01
**AC009090.6**	-0.44	7.40E-01	1.00E+00	-0.16	8.67E-01	9.84E-01
**ZBTB1**	0.13	5.54E-01	1.00E+00	-0.03	8.68E-01	9.84E-01
**AC007728.3**	-0.28	8.87E-01	1.00E+00	-0.20	8.68E-01	9.84E-01
**NR2F2-AS1**	0.67	2.39E-01	1.00E+00	-0.10	8.68E-01	9.84E-01

**BCAS4**	-0.37	4.35E-01	1.00E+00	0.05	8.68E-01	9.84E-01
**ABLIM3**	0.67	1.96E-01	1.00E+00	-0.09	8.68E-01	9.84E-01
**ATP9B**	-0.02	9.27E-01	1.00E+00	0.03	8.68E-01	9.84E-01
**SP1**	-0.04	8.40E-01	1.00E+00	0.06	8.68E-01	9.84E-01
**KRT85**	-3.53	2.79E-01	1.00E+00	-0.52	8.68E-01	9.84E-01
**UCHL1**	0.59	4.56E-01	1.00E+00	-0.10	8.68E-01	9.84E-01
**AL121772.1**	-0.13	8.89E-01	1.00E+00	-0.18	8.68E-01	9.84E-01
**RBM38**	-0.05	8.52E-01	1.00E+00	0.09	8.68E-01	9.84E-01
**NUDC**	-0.04	8.41E-01	1.00E+00	0.02	8.68E-01	9.84E-01
**NCAPD3**	0.28	2.50E-01	1.00E+00	-0.05	8.68E-01	9.84E-01
**PNMA8B**	0.03	9.15E-01	1.00E+00	0.11	8.68E-01	9.84E-01
**HR**	-0.11	7.88E-01	1.00E+00	-0.13	8.68E-01	9.84E-01
**TTLL6**	-3.36	6.82E-02	1.00E+00	-0.31	8.68E-01	9.84E-01
**PADI3**	-2.33	3.92E-05	1.06E-02	0.17	8.68E-01	9.84E-01
**XLOC_006338**	-2.50	1.00E-01	1.00E+00	0.23	8.68E-01	9.84E-01
**SETD6**	-0.28	3.34E-01	1.00E+00	-0.04	8.68E-01	9.84E-01
**MYBBP1A**	-0.48	5.29E-02	1.00E+00	0.08	8.68E-01	9.84E-01
**RNF26**	-0.10	6.75E-01	1.00E+00	-0.06	8.68E-01	9.84E-01
**NFATC1**	-0.74	4.74E-03	3.98E-01	0.08	8.68E-01	9.84E-01
**RPS4XP6**	-0.56	8.73E-01	1.00E+00	-0.14	8.68E-01	9.84E-01
**PLAC9**	0.20	5.99E-01	1.00E+00	0.08	8.68E-01	9.84E-01
**AL078645.1**	0.32	9.28E-01	1.00E+00	0.23	8.68E-01	9.84E-01
**DKK2**	-0.33	5.93E-01	1.00E+00	0.08	8.69E-01	9.84E-01
**KLHL24**	0.17	6.06E-01	1.00E+00	-0.05	8.69E-01	9.84E-01
**UCKL1**	-0.08	7.14E-01	1.00E+00	0.02	8.69E-01	9.84E-01
**ARHGAP23**	-0.32	4.17E-01	1.00E+00	-0.10	8.69E-01	9.84E-01
**LIPE**	4.03	6.28E-03	4.75E-01	-0.52	8.69E-01	9.84E-01
**ADAT3**	-0.34	4.55E-01	1.00E+00	0.10	8.69E-01	9.84E-01
**GPATCH1**	0.13	5.23E-01	1.00E+00	0.05	8.69E-01	9.84E-01
**AP001282.1**	0.84	8.06E-01	1.00E+00	-0.10	8.69E-01	9.84E-01
**XLOC_005155**	0.34	9.23E-01	1.00E+00	-0.24	8.69E-01	9.84E-01
**HSPBAP1**	-0.23	5.81E-01	1.00E+00	0.05	8.69E-01	9.84E-01

**NCOA6**	-0.29	2.58E-01	1.00E+00	0.07	8.69E-01	9.84E-01
**XLOC_013093**	0.10	9.47E-01	1.00E+00	-0.22	8.69E-01	9.84E-01
**ZNF674**	0.35	4.15E-01	1.00E+00	-0.09	8.69E-01	9.84E-01
**LZTS3**	-0.10	8.56E-01	1.00E+00	0.15	8.69E-01	9.84E-01
**RSRP1**	-0.36	5.39E-01	1.00E+00	0.07	8.69E-01	9.84E-01
**SDAD1P1**	-0.44	1.98E-01	1.00E+00	-0.06	8.69E-01	9.84E-01
**LPAR1**	0.51	2.13E-01	1.00E+00	-0.06	8.69E-01	9.84E-01
**G8227**	-1.00	1.68E-01	1.00E+00	0.11	8.69E-01	9.84E-01
**LINC00683**	-0.09	9.19E-01	1.00E+00	0.18	8.69E-01	9.84E-01
**NAA25**	0.22	4.49E-01	1.00E+00	-0.04	8.70E-01	9.84E-01
**AL596087.1**	0.02	9.88E-01	1.00E+00	0.13	8.70E-01	9.84E-01
**AC027279.4**	2.64	4.38E-01	1.00E+00	0.53	8.70E-01	9.84E-01
**YWHABP2**	-1.04	6.66E-01	1.00E+00	0.13	8.70E-01	9.84E-01
**ADAMTSL2**	-0.23	5.91E-01	1.00E+00	0.48	8.70E-01	9.84E-01
**KDM4B**	-0.16	4.85E-01	1.00E+00	-0.09	8.70E-01	9.84E-01
**WDR12**	-0.05	8.50E-01	1.00E+00	0.03	8.70E-01	9.84E-01
**HIST1H2AI**	0.66	6.90E-01	1.00E+00	-0.13	8.70E-01	9.84E-01
**IGLV3-16**	NA	NA	NA	0.53	8.70E-01	9.84E-01
**CASC4**	0.39	2.91E-01	1.00E+00	-0.05	8.70E-01	9.84E-01
**RPP21**	1.13	7.44E-01	1.00E+00	0.18	8.70E-01	9.84E-01
**AC090152.1**	-0.36	5.20E-01	1.00E+00	-0.10	8.70E-01	9.84E-01
**TMCO3**	0.31	4.14E-01	1.00E+00	-0.05	8.70E-01	9.84E-01
**ZNF597**	0.43	1.34E-01	1.00E+00	0.06	8.70E-01	9.85E-01
**REL**	-0.49	1.75E-01	1.00E+00	0.07	8.70E-01	9.85E-01
**IGHJ4**	0.56	8.71E-01	1.00E+00	0.53	8.70E-01	9.85E-01
**TMEM150A**	0.29	3.58E-01	1.00E+00	0.06	8.70E-01	9.85E-01
**ESR2**	0.86	2.87E-01	1.00E+00	-0.11	8.70E-01	9.85E-01
**SULT1A2**	-1.52	3.01E-01	1.00E+00	-0.13	8.70E-01	9.85E-01
**IPO5**	0.17	5.15E-01	1.00E+00	0.05	8.70E-01	9.85E-01
**AC008038.1**	-0.22	5.25E-01	1.00E+00	-0.08	8.70E-01	9.85E-01
**RCN1**	0.22	5.34E-01	1.00E+00	0.05	8.70E-01	9.85E-01
**MITF**	-0.05	8.90E-01	1.00E+00	-0.05	8.71E-01	9.85E-01

**KCCAT333**	1.14	2.53E-01	1.00E+00	0.19	8.71E-01	9.85E-01
**ANKRD53**	0.72	2.93E-01	1.00E+00	0.30	8.71E-01	9.85E-01
**AC007938.3**	-0.77	6.03E-01	1.00E+00	0.14	8.71E-01	9.85E-01
**CTNNBIP1**	0.08	8.56E-01	1.00E+00	0.07	8.71E-01	9.85E-01
**G9256**	-2.13	7.67E-02	1.00E+00	0.16	8.71E-01	9.85E-01
**AC096537.1**	-1.40	6.84E-01	1.00E+00	-0.28	8.71E-01	9.85E-01
**SPTLC1**	-0.07	7.40E-01	1.00E+00	0.03	8.71E-01	9.85E-01
**PRXL2B**	-0.37	5.35E-02	1.00E+00	0.06	8.71E-01	9.85E-01
**ZNF669**	0.24	3.49E-01	1.00E+00	-0.05	8.71E-01	9.85E-01
**PYM1**	-0.19	5.19E-01	1.00E+00	-0.05	8.71E-01	9.85E-01
**SLC1A3**	-0.02	9.49E-01	1.00E+00	0.09	8.71E-01	9.85E-01
**PSME3**	-0.12	5.63E-01	1.00E+00	0.02	8.71E-01	9.85E-01
**ZNF185**	-0.23	5.41E-01	1.00E+00	-0.08	8.71E-01	9.85E-01
**ISPD**	-0.70	5.20E-02	1.00E+00	-0.06	8.71E-01	9.85E-01
**KCTD21-AS1**	0.55	4.65E-01	1.00E+00	-0.10	8.72E-01	9.85E-01
**PEX11G**	-0.29	3.92E-01	1.00E+00	0.05	8.72E-01	9.85E-01
**AQP7P4**	2.23	1.93E-01	1.00E+00	-0.48	8.72E-01	9.85E-01
**XLOC_000529**	5.12	9.52E-03	6.06E-01	0.51	8.72E-01	9.85E-01
**AFAP1L1**	0.11	7.95E-01	1.00E+00	-0.10	8.72E-01	9.85E-01
**SRSF6**	-0.26	2.45E-01	1.00E+00	0.05	8.72E-01	9.85E-01
**LINC02288**	2.93	5.59E-02	1.00E+00	0.18	8.72E-01	9.85E-01
**ZMYND12**	-0.66	2.81E-01	1.00E+00	0.09	8.72E-01	9.85E-01
**RP11-497H16.6**	-0.90	7.32E-01	1.00E+00	-0.20	8.72E-01	9.85E-01
**AC145124.1**	-0.40	5.45E-01	1.00E+00	-0.09	8.72E-01	9.85E-01
**RAD23B**	0.05	8.66E-01	1.00E+00	0.08	8.72E-01	9.85E-01
**HDGF**	-0.11	6.80E-01	1.00E+00	0.08	8.72E-01	9.85E-01
**TRAF2**	0.01	9.67E-01	1.00E+00	0.07	8.72E-01	9.85E-01
**EDNRB**	0.67	6.26E-02	1.00E+00	0.06	8.72E-01	9.85E-01
**RBM26**	0.01	9.57E-01	1.00E+00	-0.05	8.72E-01	9.85E-01
**FAM181B**	-0.27	5.41E-01	1.00E+00	0.16	8.72E-01	9.85E-01
**CHD5**	-1.98	5.45E-01	1.00E+00	0.51	8.72E-01	9.85E-01
**KANK4**	1.77	5.59E-02	1.00E+00	-0.13	8.72E-01	9.85E-01

**ABO**	0.93	2.51E-02	9.28E-01	-0.09	8.72E-01	9.85E-01
**RIMBP3**	0.48	6.82E-01	1.00E+00	-0.26	8.73E-01	9.85E-01
**PHBP20**	1.37	6.91E-01	1.00E+00	-0.18	8.73E-01	9.85E-01
**PPIE**	0.17	2.77E-01	1.00E+00	0.05	8.73E-01	9.85E-01
**HSD52**	0.65	5.07E-01	1.00E+00	-0.30	8.73E-01	9.85E-01
**RIMKLBP2**	1.62	5.71E-01	1.00E+00	0.19	8.73E-01	9.85E-01
**TOR1A**	0.10	5.45E-01	1.00E+00	-0.03	8.73E-01	9.85E-01
**MATK**	-1.09	6.08E-03	4.63E-01	0.18	8.73E-01	9.85E-01
**AADACL2-AS1**	-0.20	8.45E-01	1.00E+00	0.15	8.73E-01	9.85E-01
**HAUS2**	0.01	9.60E-01	1.00E+00	-0.02	8.73E-01	9.85E-01
**C4BPB**	-2.40	2.50E-01	1.00E+00	-0.18	8.73E-01	9.85E-01
**UGT2B11**	-1.15	4.96E-01	1.00E+00	-0.48	8.73E-01	9.85E-01
**DGKG**	0.05	9.19E-01	1.00E+00	0.13	8.73E-01	9.85E-01
**SLC16A14**	-0.36	4.05E-01	1.00E+00	-0.06	8.73E-01	9.85E-01
**ANXA2P2**	0.23	3.59E-01	1.00E+00	-0.09	8.73E-01	9.85E-01
**MIR4263**	-0.33	8.33E-01	1.00E+00	-0.15	8.73E-01	9.85E-01
**ARAP2**	-0.01	9.85E-01	1.00E+00	0.07	8.73E-01	9.85E-01
**PAK6**	0.03	9.35E-01	1.00E+00	-0.08	8.73E-01	9.85E-01
**KIF14**	0.75	1.48E-01	1.00E+00	0.14	8.73E-01	9.85E-01
**PPIAP73**	-1.27	6.00E-01	1.00E+00	0.21	8.73E-01	9.85E-01
**UNC45B**	1.46	4.95E-01	1.00E+00	0.23	8.73E-01	9.85E-01
**GMPPB**	-0.14	5.32E-01	1.00E+00	-0.04	8.73E-01	9.85E-01
**ARHGEF12**	-0.22	2.95E-01	1.00E+00	-0.07	8.73E-01	9.85E-01
**AVIL**	0.07	8.69E-01	1.00E+00	-0.07	8.73E-01	9.85E-01
**D2HGDH**	0.06	8.49E-01	1.00E+00	-0.09	8.73E-01	9.85E-01
**EEF1AKMT3**	-0.05	8.35E-01	1.00E+00	-0.05	8.73E-01	9.85E-01
**AC087289.2**	-1.91	2.36E-01	1.00E+00	0.21	8.73E-01	9.85E-01
**AC008972.1**	0.84	8.03E-01	1.00E+00	0.20	8.74E-01	9.85E-01
**EDC4**	-0.08	7.64E-01	1.00E+00	-0.12	8.74E-01	9.85E-01
**ALDH7A1**	-0.04	8.18E-01	1.00E+00	-0.02	8.74E-01	9.85E-01
**TIMM23B**	-0.08	8.63E-01	1.00E+00	0.04	8.74E-01	9.85E-01
**ARHGAP31**	-0.25	1.77E-01	1.00E+00	0.07	8.74E-01	9.85E-01

**CARMIL1**	-0.13	5.94E-01	1.00E+00	0.05	8.74E-01	9.85E-01
**TBPL1**	0.13	5.26E-01	1.00E+00	-0.05	8.74E-01	9.85E-01
**RPS3P6**	1.27	7.13E-01	1.00E+00	-0.12	8.74E-01	9.85E-01
**CDK9**	-0.15	4.94E-01	1.00E+00	-0.08	8.74E-01	9.85E-01
**KLF15**	0.45	5.39E-01	1.00E+00	0.17	8.74E-01	9.85E-01
**RB1**	-0.18	4.87E-01	1.00E+00	-0.03	8.74E-01	9.85E-01
**GDA**	-4.85	1.62E-04	3.78E-02	-0.50	8.74E-01	9.85E-01
**XLOC_006303**	-2.11	7.86E-02	1.00E+00	-0.17	8.74E-01	9.85E-01
**EFNB1**	-0.13	6.31E-01	1.00E+00	0.08	8.74E-01	9.85E-01
**G30008**	0.01	9.95E-01	1.00E+00	0.25	8.74E-01	9.85E-01
**HSPA9P1**	NA	NA	NA	0.11	8.74E-01	9.85E-01
**EIF5A**	-0.07	7.89E-01	1.00E+00	0.05	8.74E-01	9.85E-01
**KRT80**	0.14	7.85E-01	1.00E+00	-0.12	8.74E-01	9.85E-01
**AC015712.2**	0.38	3.81E-01	1.00E+00	-0.08	8.74E-01	9.85E-01
**NEK1**	0.04	8.95E-01	1.00E+00	0.03	8.74E-01	9.85E-01
**AC004232.3**	1.57	1.44E-01	1.00E+00	-0.18	8.74E-01	9.85E-01
**SPIDR**	-0.15	5.59E-01	1.00E+00	-0.03	8.74E-01	9.85E-01
**TELO2**	-0.32	1.98E-01	1.00E+00	0.08	8.74E-01	9.85E-01
**AC110792.3**	3.04	1.06E-01	1.00E+00	-0.19	8.74E-01	9.85E-01
**CXADR**	-0.15	6.74E-01	1.00E+00	-0.10	8.74E-01	9.85E-01
**ACOT8**	0.22	3.36E-01	1.00E+00	0.04	8.75E-01	9.85E-01
**RPS26P31**	-0.49	7.98E-01	1.00E+00	0.16	8.75E-01	9.85E-01
**QKI**	0.04	8.33E-01	1.00E+00	-0.05	8.75E-01	9.85E-01
**AC073073.2**	0.88	1.65E-01	1.00E+00	-0.18	8.75E-01	9.85E-01
**SPATA5L1**	-0.18	5.88E-01	1.00E+00	-0.08	8.75E-01	9.85E-01
**AC021074.1**	3.76	7.15E-02	1.00E+00	0.11	8.75E-01	9.85E-01
**R3HCC1**	-0.14	5.85E-01	1.00E+00	-0.04	8.75E-01	9.85E-01
**PLCG1-AS1**	-0.90	4.25E-01	1.00E+00	-0.13	8.75E-01	9.85E-01
**GMDS**	-0.33	2.74E-01	1.00E+00	0.04	8.75E-01	9.85E-01
**XLOC_009765**	-0.19	8.48E-01	1.00E+00	-0.12	8.75E-01	9.85E-01
**CSDE1**	0.13	4.21E-01	1.00E+00	-0.03	8.75E-01	9.85E-01
**WRNIP1**	0.01	9.73E-01	1.00E+00	-0.08	8.75E-01	9.85E-01

**LDHAP2**	-2.60	2.17E-01	1.00E+00	-0.13	8.75E-01	9.85E-01
**SNRNP70**	-0.29	2.27E-01	1.00E+00	0.13	8.75E-01	9.85E-01
**ANAPC15**	-0.31	4.46E-01	1.00E+00	-0.08	8.75E-01	9.85E-01
**MIR210HG**	0.23	5.16E-01	1.00E+00	-0.07	8.75E-01	9.85E-01
**ADRA1B**	0.54	5.19E-01	1.00E+00	-0.24	8.75E-01	9.85E-01
**NCBP2-AS2**	-0.28	2.94E-01	1.00E+00	-0.05	8.75E-01	9.85E-01
**ZNF324**	-0.06	7.68E-01	1.00E+00	-0.07	8.75E-01	9.85E-01
**NAA20**	-0.17	4.72E-01	1.00E+00	-0.05	8.75E-01	9.85E-01
**AC131649.2**	-0.83	3.96E-01	1.00E+00	0.15	8.75E-01	9.85E-01
**POFUT1**	0.26	2.59E-01	1.00E+00	0.07	8.75E-01	9.85E-01
**FECH**	0.16	4.75E-01	1.00E+00	0.03	8.75E-01	9.85E-01
**NDUFB4P11**	-3.02	4.15E-02	1.00E+00	-0.19	8.75E-01	9.85E-01
**TMPRSS3**	0.65	2.83E-01	1.00E+00	0.15	8.75E-01	9.85E-01
**APRT**	-0.17	5.49E-01	1.00E+00	0.05	8.75E-01	9.85E-01
**ZBTB11-AS1**	0.68	9.31E-02	1.00E+00	0.08	8.75E-01	9.85E-01
**PF4V1**	-1.18	7.32E-01	1.00E+00	0.27	8.75E-01	9.85E-01
**G26553**	0.97	1.77E-01	1.00E+00	0.18	8.75E-01	9.85E-01
**MRPS22**	-0.04	8.25E-01	1.00E+00	-0.03	8.75E-01	9.85E-01
**ZKSCAN7-AS1**	-1.72	2.55E-01	1.00E+00	0.15	8.75E-01	9.85E-01
**RPS28P7**	0.07	8.29E-01	1.00E+00	-0.06	8.76E-01	9.85E-01
**PSENEN**	0.10	7.80E-01	1.00E+00	-0.07	8.76E-01	9.85E-01
**AL139424.3**	-0.67	2.58E-01	1.00E+00	-0.14	8.76E-01	9.85E-01
**PAXBP1-AS1**	-0.47	4.97E-01	1.00E+00	0.11	8.76E-01	9.85E-01
**GLYATL1P1**	-0.79	6.02E-01	1.00E+00	0.16	8.76E-01	9.85E-01
**IL17B**	-1.17	9.21E-02	1.00E+00	0.09	8.76E-01	9.85E-01
**VPS50**	0.33	2.93E-01	1.00E+00	-0.05	8.76E-01	9.85E-01
**G2592**	-0.52	6.79E-01	1.00E+00	0.15	8.76E-01	9.85E-01
**Z98884.1**	-3.44	1.39E-01	1.00E+00	0.13	8.76E-01	9.85E-01
**XLOC_011365**	0.32	8.88E-01	1.00E+00	-0.18	8.76E-01	9.85E-01
**NIPAL1**	0.30	5.79E-01	1.00E+00	0.08	8.76E-01	9.85E-01
**ESRRB**	-4.67	2.78E-03	2.73E-01	-0.18	8.76E-01	9.85E-01
**ASAP2**	-0.19	4.50E-01	1.00E+00	-0.06	8.76E-01	9.85E-01

**AL135786.1**	1.19	2.89E-01	1.00E+00	-0.17	8.76E-01	9.85E-01
**AC141586.1**	-0.73	9.16E-02	1.00E+00	-0.06	8.76E-01	9.85E-01
**SEC24C**	0.02	9.26E-01	1.00E+00	0.08	8.76E-01	9.85E-01
**CCDC177**	-2.35	5.22E-02	1.00E+00	0.22	8.76E-01	9.85E-01
**SHISA3**	1.19	1.89E-01	1.00E+00	-0.13	8.76E-01	9.85E-01
**C16orf58**	-0.29	1.21E-01	1.00E+00	0.07	8.76E-01	9.85E-01
**AL121917.1**	-0.87	1.72E-01	1.00E+00	-0.12	8.76E-01	9.85E-01
**SSPN**	0.50	2.39E-01	1.00E+00	-0.04	8.76E-01	9.85E-01
**AC103810.3**	-0.30	7.11E-01	1.00E+00	0.49	8.77E-01	9.85E-01
**MAST4-IT1**	NA	NA	NA	-0.35	8.77E-01	9.85E-01
**ZNF292**	-0.11	5.97E-01	1.00E+00	-0.05	8.77E-01	9.85E-01
**NBPF11**	-0.23	7.09E-01	1.00E+00	-0.06	8.77E-01	9.85E-01
**NFIL3**	-0.27	5.70E-01	1.00E+00	-0.09	8.77E-01	9.85E-01
**AP000437.1**	0.36	7.80E-01	1.00E+00	-0.11	8.77E-01	9.85E-01
**TATDN1P1**	NA	NA	NA	0.11	8.77E-01	9.85E-01
**AL031719.2**	-1.04	4.96E-01	1.00E+00	-0.11	8.77E-01	9.85E-01
**MARCKSL1**	-0.45	2.35E-01	1.00E+00	0.08	8.77E-01	9.85E-01
**GRID2IP**	0.13	7.81E-01	1.00E+00	-0.08	8.77E-01	9.85E-01
**TCHHL1**	-5.34	1.10E-01	1.00E+00	-0.49	8.77E-01	9.85E-01
**CHML**	-0.61	3.12E-02	1.00E+00	-0.07	8.77E-01	9.85E-01
**IGKJ4**	1.34	6.98E-01	1.00E+00	0.50	8.77E-01	9.85E-01
**AHCTF1**	0.03	8.90E-01	1.00E+00	-0.06	8.77E-01	9.85E-01
**SON**	-0.28	1.71E-01	1.00E+00	-0.04	8.77E-01	9.85E-01
**ATP6V1B2**	0.27	2.38E-01	1.00E+00	0.05	8.77E-01	9.85E-01
**NPTX1**	-0.77	3.23E-01	1.00E+00	-0.17	8.77E-01	9.85E-01
**MAP3K10**	-0.70	9.36E-03	6.05E-01	-0.48	8.77E-01	9.85E-01
**SMPD5**	-0.92	3.61E-01	1.00E+00	0.20	8.77E-01	9.85E-01
**AC005082.1**	0.62	3.53E-01	1.00E+00	0.11	8.77E-01	9.85E-01
**POLR2D**	0.06	7.33E-01	1.00E+00	-0.04	8.77E-01	9.85E-01
**C15orf56**	0.15	8.72E-01	1.00E+00	0.14	8.77E-01	9.85E-01
**DIP2A**	-0.12	5.39E-01	1.00E+00	0.05	8.77E-01	9.85E-01
**AC007834.1**	1.12	2.60E-01	1.00E+00	-0.16	8.77E-01	9.85E-01

**OSMR-AS1**	0.17	7.54E-01	1.00E+00	-0.08	8.77E-01	9.85E-01
**ZDHHC17**	0.23	4.22E-01	1.00E+00	-0.05	8.77E-01	9.85E-01
**NPRL2**	-0.06	8.22E-01	1.00E+00	0.05	8.77E-01	9.85E-01
**VSTM5**	-1.35	5.49E-02	1.00E+00	-0.17	8.77E-01	9.85E-01
**PCDHGA11**	-0.30	3.87E-01	1.00E+00	-0.08	8.78E-01	9.85E-01
**DLEC1**	1.15	1.63E-01	1.00E+00	-0.18	8.78E-01	9.85E-01
**TFG**	-0.11	5.67E-01	1.00E+00	0.03	8.78E-01	9.85E-01
**G3707**	0.09	8.98E-01	1.00E+00	0.11	8.78E-01	9.85E-01
**XLOC_005889**	0.67	5.47E-01	1.00E+00	0.14	8.78E-01	9.85E-01
**SHOC2**	0.04	8.65E-01	1.00E+00	-0.04	8.78E-01	9.85E-01
**EEFSEC**	-0.27	2.43E-01	1.00E+00	0.03	8.78E-01	9.85E-01
**NDUFB1P1**	0.25	9.42E-01	1.00E+00	-0.12	8.78E-01	9.85E-01
**BTG3**	0.18	4.25E-01	1.00E+00	-0.04	8.78E-01	9.85E-01
**ZNF385C**	-0.24	6.96E-01	1.00E+00	-0.14	8.78E-01	9.85E-01
**MTMR9**	0.29	2.59E-01	1.00E+00	-0.03	8.78E-01	9.85E-01
**DSCAML1**	-0.29	7.63E-01	1.00E+00	0.22	8.78E-01	9.85E-01
**PCDHA10**	-0.09	9.40E-01	1.00E+00	-0.12	8.78E-01	9.85E-01
**ARL4D**	-0.71	1.70E-01	1.00E+00	0.05	8.78E-01	9.85E-01
**DUSP23**	-0.28	1.51E-01	1.00E+00	-0.04	8.78E-01	9.85E-01
**KRT17P3**	0.00	1.00E+00	NA	-0.18	8.78E-01	9.85E-01
**MESTIT1**	0.79	7.67E-01	1.00E+00	0.16	8.78E-01	9.85E-01
**AC078881.1**	-0.48	4.61E-01	1.00E+00	-0.08	8.78E-01	9.85E-01
**RNF115**	-0.04	8.59E-01	1.00E+00	0.05	8.78E-01	9.85E-01
**ZNF337**	-0.32	7.44E-01	1.00E+00	0.07	8.78E-01	9.85E-01
**RPL41P5**	-0.48	3.85E-01	1.00E+00	0.07	8.78E-01	9.85E-01
**XLOC_002876**	0.35	9.19E-01	1.00E+00	-0.29	8.78E-01	9.85E-01
**AC007191.1**	-0.06	9.25E-01	1.00E+00	0.14	8.78E-01	9.85E-01
**RAPSN**	-2.59	7.68E-02	1.00E+00	0.15	8.78E-01	9.85E-01
**IFNK**	-2.46	4.70E-01	1.00E+00	0.49	8.79E-01	9.85E-01
**AC016596.1**	0.18	6.85E-01	1.00E+00	0.08	8.79E-01	9.85E-01
**DPH2**	-0.23	3.03E-01	1.00E+00	0.03	8.79E-01	9.85E-01
**AL135910.1**	0.26	6.44E-01	1.00E+00	0.08	8.79E-01	9.85E-01

**MBTPS1**	-0.01	9.44E-01	1.00E+00	0.04	8.79E-01	9.85E-01
**TRMU**	0.25	4.00E-01	1.00E+00	0.03	8.79E-01	9.85E-01
**LINC00337**	-2.97	1.63E-01	1.00E+00	0.22	8.79E-01	9.85E-01
**AC010890.1**	-0.61	5.75E-01	1.00E+00	-0.15	8.79E-01	9.85E-01
**CRLF1**	-0.94	1.95E-02	8.74E-01	-0.45	8.79E-01	9.85E-01
**ACER3**	-0.10	7.81E-01	1.00E+00	-0.05	8.79E-01	9.85E-01
**AKT1**	-0.17	4.33E-01	1.00E+00	-0.09	8.79E-01	9.85E-01
**ZNF354A**	0.05	8.76E-01	1.00E+00	-0.03	8.79E-01	9.85E-01
**BAD**	0.31	3.94E-01	1.00E+00	0.08	8.79E-01	9.85E-01
**FAM171A1**	0.26	5.34E-01	1.00E+00	0.09	8.79E-01	9.85E-01
**MYOZ1**	-0.67	3.63E-01	1.00E+00	0.10	8.79E-01	9.85E-01
**RPL38**	-0.03	9.21E-01	1.00E+00	0.03	8.79E-01	9.85E-01
**KCNH2**	-0.35	7.41E-01	1.00E+00	-0.15	8.80E-01	9.85E-01
**LINC01750**	-0.69	1.30E-01	1.00E+00	-0.08	8.80E-01	9.86E-01
**AC140134.1**	-0.04	9.56E-01	1.00E+00	-0.14	8.80E-01	9.86E-01
**AC016712.1**	NA	NA	NA	-0.14	8.80E-01	9.86E-01
**HERC3**	0.40	9.49E-02	1.00E+00	0.02	8.80E-01	9.86E-01
**TMX4**	0.35	2.56E-01	1.00E+00	0.04	8.80E-01	9.86E-01
**AL162595.1**	-0.18	8.51E-01	1.00E+00	0.11	8.80E-01	9.86E-01
**RPL34P6**	-0.54	8.76E-01	1.00E+00	0.15	8.80E-01	9.86E-01
**RPS21**	-0.08	7.97E-01	1.00E+00	0.04	8.80E-01	9.86E-01
**TRBV4-2**	-2.42	2.71E-01	1.00E+00	0.18	8.80E-01	9.86E-01
**IL18**	0.07	8.75E-01	1.00E+00	-0.08	8.80E-01	9.86E-01
**SMNDC1**	0.25	2.71E-01	1.00E+00	-0.03	8.80E-01	9.86E-01
**ENTHD1**	-2.23	1.69E-01	1.00E+00	0.19	8.80E-01	9.86E-01
**LINC00472**	0.76	3.40E-01	1.00E+00	-0.13	8.80E-01	9.86E-01
**PCNT**	-0.36	2.02E-01	1.00E+00	0.05	8.80E-01	9.86E-01
**LINC02519**	1.97	1.26E-01	1.00E+00	-0.28	8.80E-01	9.86E-01
**XLOC_006964**	-1.23	4.07E-01	1.00E+00	0.19	8.80E-01	9.86E-01
**PLCD4**	0.13	6.98E-01	1.00E+00	0.08	8.80E-01	9.86E-01
**HOXB-AS3**	0.42	6.01E-01	1.00E+00	0.10	8.80E-01	9.86E-01
**LRBA**	-0.06	8.16E-01	1.00E+00	0.06	8.80E-01	9.86E-01

**AC087203.3**	-1.77	1.56E-01	1.00E+00	-0.14	8.81E-01	9.86E-01
**AL121574.1**	1.11	7.45E-01	1.00E+00	0.19	8.81E-01	9.86E-01
**HDAC2-AS2**	0.68	6.41E-01	1.00E+00	-0.13	8.81E-01	9.86E-01
**CDNF**	-0.55	2.23E-01	1.00E+00	0.08	8.81E-01	9.86E-01
**AC012254.3**	-1.52	6.60E-01	1.00E+00	0.19	8.81E-01	9.86E-01
**CPPED1**	0.00	9.93E-01	1.00E+00	0.03	8.81E-01	9.86E-01
**LCE5A**	0.85	2.89E-01	1.00E+00	-0.14	8.81E-01	9.86E-01
**CPTP**	-0.23	3.03E-01	1.00E+00	0.09	8.81E-01	9.86E-01
**TMEM233**	-2.31	6.79E-02	1.00E+00	-0.12	8.81E-01	9.86E-01
**CCDC66**	-0.01	9.81E-01	1.00E+00	-0.05	8.81E-01	9.86E-01
**UBE2FP3**	0.75	4.80E-01	1.00E+00	-0.16	8.81E-01	9.86E-01
**AC004596.1**	-1.99	2.69E-01	1.00E+00	0.18	8.81E-01	9.86E-01
**ZNF146**	-0.03	8.63E-01	1.00E+00	-0.04	8.81E-01	9.86E-01
**LINC00843**	-0.52	5.61E-01	1.00E+00	0.04	8.81E-01	9.86E-01
**AC107074.1**	0.86	3.27E-01	1.00E+00	-0.13	8.81E-01	9.86E-01
**RPL34P31**	NA	NA	NA	-0.13	8.81E-01	9.86E-01
**RNASEK**	-2.38	7.50E-02	1.00E+00	0.08	8.81E-01	9.86E-01
**AC010173.1**	0.42	5.68E-01	1.00E+00	0.19	8.81E-01	9.86E-01
**KIF5B**	0.12	5.77E-01	1.00E+00	-0.02	8.82E-01	9.86E-01
**PAGR1**	0.36	2.65E-01	1.00E+00	0.07	8.82E-01	9.86E-01
**REEP2**	-0.45	3.46E-01	1.00E+00	-0.11	8.82E-01	9.86E-01
**SMPD4P1**	-3.46	1.86E-01	1.00E+00	-0.22	8.82E-01	9.86E-01
**RAB1A**	0.10	5.06E-01	1.00E+00	0.02	8.82E-01	9.86E-01
**EVPL**	-0.17	7.36E-01	1.00E+00	-0.11	8.82E-01	9.86E-01
**AL024498.1**	1.34	2.03E-01	1.00E+00	0.13	8.82E-01	9.86E-01
**P3H2-AS1**	-0.67	5.03E-01	1.00E+00	-0.15	8.82E-01	9.86E-01
**ATP6V1D**	0.13	6.09E-01	1.00E+00	-0.04	8.82E-01	9.86E-01
**TMEM104**	-0.27	2.98E-01	1.00E+00	0.08	8.82E-01	9.86E-01
**LDHAP5**	NA	NA	NA	0.10	8.82E-01	9.86E-01
**TMEM121**	-0.54	1.09E-01	1.00E+00	0.12	8.82E-01	9.86E-01
**NTNG2**	-0.18	5.95E-01	1.00E+00	0.44	8.82E-01	9.86E-01
**AFTPH**	0.13	6.20E-01	1.00E+00	-0.04	8.82E-01	9.86E-01

**PGAM4**	-0.48	7.66E-01	1.00E+00	0.08	8.82E-01	9.86E-01
**XLOC_005079**	0.27	7.49E-01	1.00E+00	-0.15	8.82E-01	9.86E-01
**KRT81**	-1.54	6.30E-01	1.00E+00	-0.47	8.82E-01	9.86E-01
**AC092747.2**	1.92	4.65E-01	1.00E+00	-0.11	8.82E-01	9.86E-01
**TATDN2P2**	0.50	2.72E-01	1.00E+00	0.12	8.82E-01	9.86E-01
**AC096708.3**	-1.76	6.09E-01	1.00E+00	0.34	8.82E-01	9.86E-01
**G30001**	0.59	4.26E-01	1.00E+00	0.14	8.82E-01	9.86E-01
**U73166.1**	1.36	3.84E-01	1.00E+00	0.17	8.82E-01	9.86E-01
**PFKL**	0.01	9.47E-01	1.00E+00	0.05	8.82E-01	9.86E-01
**PLAC1**	-0.05	9.66E-01	1.00E+00	0.16	8.82E-01	9.86E-01
**LOH12CR2**	0.08	9.01E-01	1.00E+00	-0.08	8.82E-01	9.86E-01
**FBXO43**	0.60	3.91E-01	1.00E+00	-0.15	8.82E-01	9.86E-01
**MAPK8IP1P2**	-4.00	1.79E-01	1.00E+00	0.48	8.82E-01	9.86E-01
**AC110769.2**	0.32	8.65E-01	1.00E+00	0.16	8.82E-01	9.86E-01
**NUDT6**	0.11	7.66E-01	1.00E+00	-0.05	8.83E-01	9.86E-01
**FOXP3**	-0.57	2.97E-01	1.00E+00	0.46	8.83E-01	9.86E-01
**CPSF2**	-0.07	7.54E-01	1.00E+00	-0.03	8.83E-01	9.86E-01
**AC090587.2**	-1.73	3.64E-03	3.27E-01	0.11	8.83E-01	9.86E-01
**PPP1R13L**	-0.40	3.69E-01	1.00E+00	0.07	8.83E-01	9.86E-01
**CLEC14A**	0.22	6.36E-01	1.00E+00	0.07	8.83E-01	9.86E-01
**EIF4HP1**	-0.22	6.83E-01	1.00E+00	-0.06	8.83E-01	9.86E-01
**IKZF5**	0.06	7.89E-01	1.00E+00	-0.05	8.83E-01	9.86E-01
**CAPN10**	-0.15	6.02E-01	1.00E+00	0.10	8.83E-01	9.86E-01
**LIX1L**	0.31	4.32E-01	1.00E+00	-0.05	8.83E-01	9.86E-01
**TICRR**	-0.46	2.28E-01	1.00E+00	0.18	8.83E-01	9.86E-01
**FLII**	-0.14	4.15E-01	1.00E+00	0.05	8.83E-01	9.86E-01
**XLOC_005778**	1.83	3.02E-01	1.00E+00	-0.12	8.83E-01	9.86E-01
**AC120049.1**	-0.03	9.44E-01	1.00E+00	0.12	8.83E-01	9.86E-01
**NSF**	-0.12	5.46E-01	1.00E+00	-0.03	8.83E-01	9.86E-01
**LINC01116**	0.11	7.56E-01	1.00E+00	-0.06	8.83E-01	9.86E-01
**PPP1R32**	-0.39	5.22E-01	1.00E+00	0.09	8.83E-01	9.86E-01
**ZNF34**	0.31	2.37E-01	1.00E+00	-0.05	8.83E-01	9.86E-01

**PMS2P3**	-0.74	1.99E-01	1.00E+00	0.11	8.83E-01	9.86E-01
**KIDINS220**	-0.06	7.15E-01	1.00E+00	0.05	8.83E-01	9.86E-01
**AC004554.2**	-0.96	4.67E-01	1.00E+00	-0.17	8.83E-01	9.86E-01
**TBC1D13**	0.42	1.62E-01	1.00E+00	0.07	8.83E-01	9.86E-01
**MRPL14**	0.01	9.63E-01	1.00E+00	0.04	8.83E-01	9.86E-01
**AL512598.2**	NA	NA	NA	0.15	8.83E-01	9.86E-01
**VWA2**	-0.10	7.56E-01	1.00E+00	0.07	8.83E-01	9.86E-01
**DCTN2**	-0.19	4.28E-01	1.00E+00	-0.02	8.83E-01	9.86E-01
**AL627230.4**	0.19	8.59E-01	1.00E+00	0.09	8.83E-01	9.86E-01
**PNISR**	0.06	7.77E-01	1.00E+00	0.03	8.84E-01	9.86E-01
**CUX2**	1.19	2.69E-01	1.00E+00	0.46	8.84E-01	9.86E-01
**LNX1-AS1**	-1.52	6.60E-01	1.00E+00	-0.17	8.84E-01	9.86E-01
**AC133785.1**	-0.10	8.60E-01	1.00E+00	0.15	8.84E-01	9.86E-01
**RPL37P2**	-0.63	4.63E-01	1.00E+00	0.12	8.84E-01	9.86E-01
**XYLT2**	-0.11	5.66E-01	1.00E+00	0.04	8.84E-01	9.86E-01
**GCSAM**	-0.20	6.05E-01	1.00E+00	-0.06	8.84E-01	9.86E-01
**MTMR11**	0.10	7.63E-01	1.00E+00	0.04	8.84E-01	9.86E-01
**CYP46A1**	0.45	6.56E-01	1.00E+00	-0.10	8.84E-01	9.86E-01
**RBM8B**	-0.59	7.57E-01	1.00E+00	-0.08	8.84E-01	9.86E-01
**VKORC1**	0.43	4.97E-01	1.00E+00	-0.08	8.84E-01	9.86E-01
**RPS6KL1**	-0.64	2.17E-01	1.00E+00	-0.10	8.84E-01	9.86E-01
**SUGT1P1**	-1.06	2.02E-01	1.00E+00	0.15	8.84E-01	9.86E-01
**WDR77**	0.17	5.64E-01	1.00E+00	0.03	8.84E-01	9.86E-01
**AL132656.3**	-0.56	8.73E-01	1.00E+00	0.14	8.84E-01	9.86E-01
**CCR6**	1.27	6.83E-01	1.00E+00	-0.14	8.84E-01	9.86E-01
**AC008555.8**	-0.75	5.09E-01	1.00E+00	0.12	8.84E-01	9.86E-01
**CCM2L**	0.29	5.61E-01	1.00E+00	-0.13	8.84E-01	9.86E-01
**AC074194.1**	-3.07	2.41E-01	1.00E+00	0.16	8.84E-01	9.86E-01
**TRIM72**	-0.81	3.48E-01	1.00E+00	-0.43	8.84E-01	9.86E-01
**PCF11**	-0.07	8.40E-01	1.00E+00	-0.03	8.84E-01	9.86E-01
**CHGA**	-4.39	2.14E-12	1.47E-09	-0.17	8.85E-01	9.86E-01
**AC090772.4**	1.37	3.19E-01	1.00E+00	-0.12	8.85E-01	9.86E-01

**AC003071.1**	-0.05	9.88E-01	1.00E+00	-0.12	8.85E-01	9.86E-01
**NAT1**	0.47	2.08E-01	1.00E+00	0.05	8.85E-01	9.86E-01
**NAMPTP1**	-1.32	2.14E-01	1.00E+00	0.07	8.85E-01	9.86E-01
**IMP4**	-0.07	6.21E-01	1.00E+00	0.03	8.85E-01	9.86E-01
**ZNF575**	0.15	7.22E-01	1.00E+00	-0.07	8.85E-01	9.86E-01
**DDX10P1**	1.47	5.15E-01	1.00E+00	-0.19	8.85E-01	9.86E-01
**EDARADD**	-0.42	2.40E-01	1.00E+00	-0.08	8.85E-01	9.86E-01
**AL021937.2**	1.25	3.44E-01	1.00E+00	0.10	8.85E-01	9.86E-01
**DEF6**	-0.15	6.05E-01	1.00E+00	0.06	8.85E-01	9.86E-01
**ZNF710-AS1**	-0.01	9.82E-01	1.00E+00	0.06	8.85E-01	9.86E-01
**ZNF766**	0.02	9.28E-01	1.00E+00	-0.05	8.85E-01	9.86E-01
**TCP10L**	0.09	9.38E-01	1.00E+00	0.12	8.85E-01	9.86E-01
**LRRC55**	0.00	9.96E-01	1.00E+00	0.22	8.85E-01	9.86E-01
**AC044849.1**	-1.06	6.77E-01	1.00E+00	0.12	8.85E-01	9.86E-01
**RPL3P7**	-3.06	2.59E-01	1.00E+00	-0.12	8.85E-01	9.86E-01
**AC005840.2**	-1.05	4.26E-01	1.00E+00	0.16	8.85E-01	9.86E-01
**AC133644.2**	1.79	2.65E-01	1.00E+00	-0.16	8.85E-01	9.86E-01
**ZNF57**	-0.21	6.84E-01	1.00E+00	0.08	8.85E-01	9.86E-01
**KIAA0586**	0.00	9.99E-01	1.00E+00	0.03	8.85E-01	9.86E-01
**TMEM132A**	-0.38	4.83E-01	1.00E+00	0.45	8.85E-01	9.86E-01
**PABPC1P3**	-1.81	3.13E-01	1.00E+00	0.08	8.85E-01	9.86E-01
**HMGN1P37**	0.38	8.83E-01	1.00E+00	-0.13	8.85E-01	9.86E-01
**PITHD1**	0.01	9.58E-01	1.00E+00	0.03	8.85E-01	9.86E-01
**IFT88**	0.22	4.38E-01	1.00E+00	-0.04	8.85E-01	9.86E-01
**CCDC190**	-1.96	5.64E-01	1.00E+00	-0.26	8.85E-01	9.86E-01
**ANKRD12**	0.05	8.63E-01	1.00E+00	-0.05	8.85E-01	9.86E-01
**SLC41A1**	0.03	9.09E-01	1.00E+00	0.06	8.85E-01	9.86E-01
**C17orf49**	0.48	6.33E-01	1.00E+00	-0.09	8.85E-01	9.86E-01
**NPLOC4**	0.13	5.52E-01	1.00E+00	0.07	8.85E-01	9.86E-01
**GGNBP2**	0.01	9.52E-01	1.00E+00	-0.02	8.86E-01	9.86E-01
**AL031777.3**	-2.69	7.23E-02	1.00E+00	0.18	8.86E-01	9.86E-01
**TMEM129**	0.14	5.94E-01	1.00E+00	-0.08	8.86E-01	9.86E-01

**SREBF2-AS1**	0.26	6.13E-01	1.00E+00	0.05	8.86E-01	9.86E-01
**EIF3K**	-0.20	3.85E-01	1.00E+00	-0.02	8.86E-01	9.86E-01
**EBLN3P**	0.25	3.02E-01	1.00E+00	0.04	8.86E-01	9.86E-01
**RTF1**	-0.03	8.66E-01	1.00E+00	0.04	8.86E-01	9.86E-01
**VAX2**	-0.21	7.75E-01	1.00E+00	-0.13	8.86E-01	9.86E-01
**HPD**	0.19	8.69E-01	1.00E+00	-0.13	8.86E-01	9.86E-01
**XLOC_007528**	-0.41	8.51E-01	1.00E+00	0.16	8.86E-01	9.86E-01
**KLHL38**	-0.28	7.37E-01	1.00E+00	-0.17	8.86E-01	9.86E-01
**AC099494.1**	1.23	7.18E-01	1.00E+00	-0.20	8.86E-01	9.86E-01
**ANO4**	-0.54	5.71E-01	1.00E+00	-0.14	8.86E-01	9.86E-01
**SLC16A8**	0.43	4.67E-01	1.00E+00	-0.09	8.86E-01	9.86E-01
**BTF3P10**	0.62	8.57E-01	1.00E+00	0.10	8.86E-01	9.86E-01
**XLOC_012997**	0.58	3.27E-01	1.00E+00	0.10	8.86E-01	9.86E-01
**CES4A**	0.08	9.02E-01	1.00E+00	-0.07	8.86E-01	9.86E-01
**CRY1**	0.70	8.12E-03	5.56E-01	0.04	8.86E-01	9.86E-01
**AC023512.1**	1.11	7.45E-01	1.00E+00	0.13	8.86E-01	9.86E-01
**METTL22**	0.30	3.12E-01	1.00E+00	0.04	8.86E-01	9.86E-01
**DAP**	-0.22	3.32E-01	1.00E+00	-0.03	8.86E-01	9.86E-01
**SEMA6C**	0.13	6.65E-01	1.00E+00	0.12	8.86E-01	9.86E-01
**G30917**	-1.29	3.52E-01	1.00E+00	0.15	8.86E-01	9.86E-01
**ESR1**	0.49	4.58E-01	1.00E+00	0.07	8.87E-01	9.86E-01
**ANXA11**	0.12	5.86E-01	1.00E+00	-0.06	8.87E-01	9.86E-01
**RNF24**	0.08	8.18E-01	1.00E+00	-0.03	8.87E-01	9.86E-01
**AL162311.3**	-0.25	7.72E-01	1.00E+00	-0.12	8.87E-01	9.86E-01
**IL17RC**	-0.10	7.08E-01	1.00E+00	-0.08	8.87E-01	9.86E-01
**NAA35**	0.15	4.56E-01	1.00E+00	0.03	8.87E-01	9.86E-01
**AC087392.5**	-0.08	9.24E-01	1.00E+00	0.13	8.87E-01	9.86E-01
**PARK7**	0.14	5.91E-01	1.00E+00	0.03	8.87E-01	9.86E-01
**PTP4A3**	-0.64	1.19E-02	6.77E-01	0.06	8.87E-01	9.86E-01
**AC025754.2**	-4.25	7.38E-02	1.00E+00	0.14	8.87E-01	9.86E-01
**GTF2E1**	-0.06	8.28E-01	1.00E+00	-0.03	8.87E-01	9.86E-01
**SND1**	-0.14	4.42E-01	1.00E+00	0.04	8.87E-01	9.86E-01

**AC087392.1**	-1.10	2.10E-01	1.00E+00	-0.09	8.87E-01	9.86E-01
**FBXO46**	-0.08	7.55E-01	1.00E+00	-0.08	8.87E-01	9.86E-01
**AC135803.1**	1.04	7.57E-01	1.00E+00	0.23	8.87E-01	9.86E-01
**FMO6P**	0.88	7.23E-01	1.00E+00	-0.16	8.87E-01	9.86E-01
**KMT2C**	-0.26	2.48E-01	1.00E+00	-0.04	8.87E-01	9.86E-01
**MPP2**	-0.06	9.01E-01	1.00E+00	0.11	8.87E-01	9.86E-01
**AP000851.2**	-1.13	6.56E-01	1.00E+00	0.18	8.87E-01	9.86E-01
**AC142381.3**	-0.28	6.73E-01	1.00E+00	-0.18	8.87E-01	9.86E-01
**FAM185BP**	-0.22	7.17E-01	1.00E+00	0.08	8.87E-01	9.86E-01
**AL162274.3**	-0.12	9.03E-01	1.00E+00	0.19	8.87E-01	9.86E-01
**CNTRL**	-0.03	9.13E-01	1.00E+00	-0.05	8.88E-01	9.86E-01
**AL139393.1**	-0.66	7.13E-01	1.00E+00	0.24	8.88E-01	9.86E-01
**IP6K3**	-0.18	9.02E-01	1.00E+00	-0.21	8.88E-01	9.86E-01
**SHANK3**	-0.55	1.03E-01	1.00E+00	0.44	8.88E-01	9.86E-01
**SAMD5**	-0.18	7.40E-01	1.00E+00	-0.10	8.88E-01	9.86E-01
**AC079848.1**	0.30	5.79E-01	1.00E+00	0.16	8.88E-01	9.86E-01
**GRHL2**	-0.29	3.46E-01	1.00E+00	-0.08	8.88E-01	9.86E-01
**G23688**	-0.57	6.94E-01	1.00E+00	-0.45	8.88E-01	9.86E-01
**AC078883.1**	-3.22	2.41E-02	9.18E-01	-0.07	8.88E-01	9.86E-01
**RPS3A**	-0.05	8.92E-01	1.00E+00	0.06	8.88E-01	9.86E-01
**KLHL2**	0.38	3.62E-01	1.00E+00	-0.08	8.88E-01	9.86E-01
**G11276**	-0.27	9.08E-01	1.00E+00	-0.17	8.88E-01	9.86E-01
**BBIP1**	0.04	8.78E-01	1.00E+00	0.03	8.88E-01	9.86E-01
**CSRNP2**	-0.19	4.63E-01	1.00E+00	0.07	8.88E-01	9.86E-01
**ISY1**	0.03	8.76E-01	1.00E+00	0.02	8.88E-01	9.86E-01
**AC023632.2**	-0.85	1.68E-01	1.00E+00	0.09	8.88E-01	9.86E-01
**RPLP1**	-0.21	5.17E-01	1.00E+00	-0.04	8.88E-01	9.86E-01
**AC008555.1**	0.47	5.29E-01	1.00E+00	0.19	8.88E-01	9.86E-01
**ARHGEF16**	-0.52	4.64E-02	1.00E+00	-0.09	8.88E-01	9.86E-01
**AC026704.1**	0.33	8.96E-01	1.00E+00	-0.14	8.88E-01	9.86E-01
**KRTAP1-5**	-2.82	2.12E-03	2.39E-01	-0.44	8.88E-01	9.86E-01
**G11342**	-3.57	6.68E-02	1.00E+00	-0.11	8.88E-01	9.86E-01

**PUS7L**	-0.03	9.16E-01	1.00E+00	0.04	8.88E-01	9.86E-01
**EIF3M**	-0.07	7.40E-01	1.00E+00	-0.03	8.88E-01	9.86E-01
**XLOC_012999**	2.14	1.88E-01	1.00E+00	0.45	8.89E-01	9.86E-01
**PSMB2**	-0.17	4.08E-01	1.00E+00	-0.02	8.89E-01	9.86E-01
**AL354707.1**	0.18	7.92E-01	1.00E+00	-0.11	8.89E-01	9.86E-01
**AC020910.5**	-0.41	7.10E-01	1.00E+00	-0.09	8.89E-01	9.86E-01
**LINC01814**	-1.14	5.55E-01	1.00E+00	0.14	8.89E-01	9.86E-01
**NDN**	0.49	2.64E-01	1.00E+00	0.10	8.89E-01	9.86E-01
**ATAD5**	-0.23	5.12E-01	1.00E+00	0.06	8.89E-01	9.86E-01
**ENOSF1**	0.49	2.21E-01	1.00E+00	0.04	8.89E-01	9.86E-01
**PLP2**	-0.07	8.37E-01	1.00E+00	-0.05	8.89E-01	9.86E-01
**S100A11**	-0.11	6.86E-01	1.00E+00	-0.03	8.89E-01	9.86E-01
**PARD3B**	0.16	6.70E-01	1.00E+00	0.05	8.89E-01	9.86E-01
**PASK**	-0.15	5.84E-01	1.00E+00	0.08	8.89E-01	9.86E-01
**C15orf41**	-0.22	3.57E-01	1.00E+00	0.05	8.89E-01	9.86E-01
**ASPA**	0.32	4.60E-01	1.00E+00	0.06	8.89E-01	9.86E-01
**XLOC_011152**	-0.23	7.90E-01	1.00E+00	0.14	8.89E-01	9.86E-01
**NCOA5**	-0.13	5.94E-01	1.00E+00	0.05	8.89E-01	9.86E-01
**KRTAP10-2**	-2.67	8.58E-03	5.70E-01	-0.44	8.89E-01	9.86E-01
**SMIM35**	1.55	4.91E-01	1.00E+00	-0.23	8.89E-01	9.86E-01
**FGFR1OP**	0.28	5.68E-01	1.00E+00	0.05	8.89E-01	9.86E-01
**AC098859.1**	NA	NA	NA	0.13	8.89E-01	9.86E-01
**SOCS2**	0.14	6.38E-01	1.00E+00	0.07	8.90E-01	9.86E-01
**FAM102B**	0.06	8.64E-01	1.00E+00	0.06	8.90E-01	9.86E-01
**AL450063.1**	0.90	7.96E-01	1.00E+00	-0.11	8.90E-01	9.86E-01
**CCDC120**	-0.55	2.25E-01	1.00E+00	0.08	8.90E-01	9.86E-01
**AC134043.2**	0.76	2.67E-01	1.00E+00	-0.09	8.90E-01	9.86E-01
**CTSF**	0.53	1.15E-01	1.00E+00	-0.07	8.90E-01	9.86E-01
**AC137936.1**	-4.04	1.02E-01	1.00E+00	0.44	8.90E-01	9.86E-01
**LYVE1**	0.20	7.39E-01	1.00E+00	-0.08	8.90E-01	9.86E-01
**DNAJC2**	0.02	9.20E-01	1.00E+00	-0.03	8.90E-01	9.86E-01
**CASC19**	1.67	2.36E-02	9.15E-01	0.09	8.90E-01	9.86E-01

**MIER1**	0.05	8.36E-01	1.00E+00	-0.03	8.90E-01	9.86E-01
**SYCP2**	-0.29	5.96E-01	1.00E+00	0.08	8.90E-01	9.86E-01
**PTPRZ1**	-0.38	3.07E-01	1.00E+00	-0.08	8.90E-01	9.86E-01
**JAKMIP2**	-0.48	3.53E-01	1.00E+00	0.10	8.90E-01	9.86E-01
**ERLIN1**	0.12	6.06E-01	1.00E+00	-0.02	8.91E-01	9.86E-01
**TTC7B**	0.38	3.51E-01	1.00E+00	0.09	8.91E-01	9.86E-01
**VPS13C**	-0.09	6.59E-01	1.00E+00	0.04	8.91E-01	9.86E-01
**CAP1P2**	NA	NA	NA	-0.08	8.91E-01	9.86E-01
**KRI1**	-0.31	3.30E-01	1.00E+00	-0.05	8.91E-01	9.86E-01
**CAMKV**	-3.84	1.16E-03	1.79E-01	-0.43	8.91E-01	9.86E-01
**LCE1B**	0.56	4.18E-01	1.00E+00	-0.12	8.91E-01	9.86E-01
**DHH**	0.76	2.75E-01	1.00E+00	-0.12	8.91E-01	9.86E-01
**TRAPPC13**	0.17	5.63E-01	1.00E+00	0.03	8.91E-01	9.86E-01
**AC105345.2**	NA	NA	NA	-0.18	8.91E-01	9.86E-01
**AL441988.1**	0.11	9.60E-01	1.00E+00	-0.13	8.91E-01	9.86E-01
**NKAIN3**	0.26	8.39E-01	1.00E+00	-0.12	8.91E-01	9.86E-01
**ZNF286B**	0.27	4.07E-01	1.00E+00	-0.05	8.91E-01	9.86E-01
**LINC02344**	-0.58	8.68E-01	1.00E+00	-0.27	8.91E-01	9.86E-01
**FAM72B**	0.32	4.50E-01	1.00E+00	0.06	8.91E-01	9.86E-01
**NTN4**	0.58	2.82E-01	1.00E+00	0.08	8.91E-01	9.86E-01
**LCMT1**	-0.05	8.46E-01	1.00E+00	-0.03	8.91E-01	9.86E-01
**ATP6V0E2-AS1**	0.44	6.58E-01	1.00E+00	-0.15	8.91E-01	9.86E-01
**SNX17**	0.11	6.10E-01	1.00E+00	-0.02	8.91E-01	9.86E-01
**CLMN**	0.79	1.30E-01	1.00E+00	-0.08	8.91E-01	9.86E-01
**PPP1R3D**	-0.22	3.81E-01	1.00E+00	-0.08	8.91E-01	9.86E-01
**RGMB**	-0.08	8.10E-01	1.00E+00	-0.05	8.91E-01	9.86E-01
**DHX34**	-0.46	1.39E-01	1.00E+00	0.08	8.91E-01	9.86E-01
**NOC3L**	0.21	6.21E-01	1.00E+00	0.04	8.92E-01	9.86E-01
**C17orf107**	0.00	9.97E-01	1.00E+00	0.08	8.92E-01	9.86E-01
**HLA-DQA1**	0.05	9.49E-01	1.00E+00	-0.08	8.92E-01	9.86E-01
**TCF23**	-0.36	7.87E-01	1.00E+00	0.18	8.92E-01	9.86E-01
**GPC5-AS2**	-2.97	2.62E-01	1.00E+00	-0.43	8.92E-01	9.86E-01

**SHTN1**	-0.31	2.57E-01	1.00E+00	0.04	8.92E-01	9.86E-01
**MFAP3L**	-0.07	7.93E-01	1.00E+00	-0.05	8.92E-01	9.86E-01
**RPL23AP18**	-1.60	6.12E-01	1.00E+00	0.16	8.92E-01	9.86E-01
**TAS1R3**	0.49	5.69E-01	1.00E+00	0.16	8.92E-01	9.86E-01
**C6orf47**	0.05	8.02E-01	1.00E+00	0.04	8.92E-01	9.86E-01
**GALNT12**	0.21	6.85E-01	1.00E+00	-0.08	8.92E-01	9.86E-01
**NDUFAF1**	0.23	1.98E-01	1.00E+00	0.04	8.92E-01	9.86E-01
**RPL3P2**	-0.07	9.42E-01	1.00E+00	0.12	8.92E-01	9.86E-01
**PRKAB1**	0.01	9.82E-01	1.00E+00	0.05	8.92E-01	9.86E-01
**RASA2**	0.18	5.89E-01	1.00E+00	0.04	8.92E-01	9.86E-01
**LINC00937**	0.57	6.76E-01	1.00E+00	0.10	8.92E-01	9.86E-01
**AC130469.1**	0.54	8.72E-01	1.00E+00	0.10	8.92E-01	9.86E-01
**AL138899.1**	-1.07	6.07E-01	1.00E+00	-0.16	8.92E-01	9.86E-01
**ZNF235**	0.52	2.17E-01	1.00E+00	0.06	8.92E-01	9.86E-01
**AGO1**	0.10	6.65E-01	1.00E+00	-0.03	8.92E-01	9.86E-01
**FAM110A**	0.00	9.92E-01	1.00E+00	0.08	8.92E-01	9.86E-01
**BEX2**	-0.17	6.01E-01	1.00E+00	-0.06	8.92E-01	9.86E-01
**PRDM15**	-0.42	1.48E-01	1.00E+00	0.06	8.92E-01	9.86E-01
**AC006305.1**	-1.01	5.41E-01	1.00E+00	0.16	8.92E-01	9.86E-01
**REXO4**	0.13	5.60E-01	1.00E+00	0.06	8.92E-01	9.86E-01
**SPTBN4**	-0.66	2.52E-01	1.00E+00	-0.13	8.92E-01	9.86E-01
**NKX3-1**	-0.87	1.42E-01	1.00E+00	-0.07	8.92E-01	9.86E-01
**RDM1**	-1.70	2.38E-01	1.00E+00	-0.13	8.92E-01	9.86E-01
**PHKG2**	-0.31	1.39E-01	1.00E+00	-0.02	8.92E-01	9.86E-01
**KRT8P33**	-0.96	2.53E-01	1.00E+00	0.12	8.93E-01	9.86E-01
**AC007114.1**	-0.17	6.22E-01	1.00E+00	-0.05	8.93E-01	9.86E-01
**WDR24**	0.05	8.66E-01	1.00E+00	-0.06	8.93E-01	9.86E-01
**CAPS2**	0.01	9.84E-01	1.00E+00	0.05	8.93E-01	9.86E-01
**CITED4**	-0.42	2.91E-01	1.00E+00	-0.12	8.93E-01	9.86E-01
**DDX55**	-0.13	5.98E-01	1.00E+00	-0.04	8.93E-01	9.86E-01
**LINC02315**	-1.19	4.03E-01	1.00E+00	-0.32	8.93E-01	9.86E-01
**RPS26P11**	0.59	7.22E-01	1.00E+00	0.11	8.93E-01	9.86E-01

**GOLGA8M**	-0.02	9.94E-01	1.00E+00	0.16	8.93E-01	9.86E-01
**AP002449.1**	0.03	9.91E-01	1.00E+00	-0.13	8.93E-01	9.86E-01
**XLOC_002840**	-2.93	2.86E-01	1.00E+00	-0.12	8.93E-01	9.86E-01
**XLOC_004680**	-1.08	6.27E-01	1.00E+00	-0.17	8.93E-01	9.86E-01
**10-Mar**	-2.15	3.57E-01	1.00E+00	0.19	8.93E-01	9.86E-01
**FRAT1**	-0.16	5.69E-01	1.00E+00	0.10	8.93E-01	9.86E-01
**XLOC_013955**	-0.55	8.60E-01	1.00E+00	0.20	8.93E-01	9.86E-01
**UGT8**	-0.68	2.77E-01	1.00E+00	0.10	8.93E-01	9.86E-01
**CTC1**	0.21	4.58E-01	1.00E+00	-0.05	8.93E-01	9.86E-01
**MYO7B**	0.71	2.43E-01	1.00E+00	-0.06	8.93E-01	9.86E-01
**LRRC41**	-0.01	9.60E-01	1.00E+00	0.03	8.94E-01	9.86E-01
**ANKRD54**	0.20	4.17E-01	1.00E+00	-0.04	8.94E-01	9.86E-01
**BNIP3**	0.31	1.72E-01	1.00E+00	0.05	8.94E-01	9.86E-01
**XLOC_002606**	2.81	3.73E-01	1.00E+00	0.16	8.94E-01	9.86E-01
**AC008758.6**	-1.10	5.97E-01	1.00E+00	0.14	8.94E-01	9.86E-01
**AMBN**	-6.46	6.36E-02	1.00E+00	0.37	8.94E-01	9.86E-01
**AL023583.1**	-2.70	3.54E-02	1.00E+00	-0.10	8.94E-01	9.86E-01
**RHBG**	-0.24	6.56E-01	1.00E+00	-0.10	8.94E-01	9.86E-01
**DMRT2**	1.09	3.63E-02	1.00E+00	-0.12	8.94E-01	9.86E-01
**NELFA**	-0.15	5.88E-01	1.00E+00	-0.04	8.94E-01	9.86E-01
**VN1R1**	0.29	7.70E-01	1.00E+00	-0.12	8.94E-01	9.86E-01
**EMID1**	-0.15	8.01E-01	1.00E+00	0.40	8.94E-01	9.86E-01
**GSTA1**	-0.48	6.29E-01	1.00E+00	0.11	8.94E-01	9.86E-01
**TMEM136**	0.58	2.92E-01	1.00E+00	0.07	8.94E-01	9.86E-01
**AL365205.1**	-0.65	7.08E-01	1.00E+00	0.09	8.94E-01	9.86E-01
**RAD54L2**	0.13	5.89E-01	1.00E+00	0.02	8.94E-01	9.86E-01
**RPL6P27**	-0.33	3.96E-01	1.00E+00	-0.06	8.94E-01	9.86E-01
**MYL6P5**	NA	NA	NA	0.10	8.94E-01	9.86E-01
**RAB19**	-2.32	1.25E-01	1.00E+00	0.16	8.94E-01	9.86E-01
**ATG9A**	-0.22	3.52E-01	1.00E+00	-0.06	8.94E-01	9.86E-01
**DNMBP**	-0.04	9.05E-01	1.00E+00	-0.05	8.94E-01	9.86E-01
**AL590556.1**	0.56	8.71E-01	1.00E+00	0.42	8.94E-01	9.86E-01

**PROSER3**	-0.68	6.57E-02	1.00E+00	-0.05	8.94E-01	9.86E-01
**MINDY2**	0.32	2.64E-01	1.00E+00	0.03	8.94E-01	9.86E-01
**ADGRE3**	4.00	2.33E-01	1.00E+00	0.30	8.94E-01	9.86E-01
**SEC14L5**	0.28	6.34E-01	1.00E+00	0.09	8.94E-01	9.86E-01
**G27376**	-1.98	5.62E-01	1.00E+00	-0.22	8.94E-01	9.86E-01
**PHLDA1**	0.14	7.32E-01	1.00E+00	0.04	8.94E-01	9.86E-01
**AOC2**	1.41	1.10E-01	1.00E+00	0.32	8.94E-01	9.86E-01
**CHRNB2**	-1.51	7.76E-02	1.00E+00	0.16	8.94E-01	9.86E-01
**MTBP**	-0.59	7.03E-02	1.00E+00	0.07	8.94E-01	9.86E-01
**OTOF**	-0.21	7.76E-01	1.00E+00	0.38	8.94E-01	9.86E-01
**GRM2**	-1.38	3.08E-02	1.00E+00	-0.22	8.95E-01	9.86E-01
**AC009509.1**	-0.25	8.98E-01	1.00E+00	0.42	8.95E-01	9.86E-01
**STAU1**	-0.11	4.93E-01	1.00E+00	0.03	8.95E-01	9.86E-01
**HMGCR**	-0.27	5.36E-01	1.00E+00	0.05	8.95E-01	9.86E-01
**MMGT1**	0.27	1.51E-01	1.00E+00	0.03	8.95E-01	9.86E-01
**G30504**	-0.91	3.64E-01	1.00E+00	-0.11	8.95E-01	9.86E-01
**BCO1**	-1.35	5.99E-02	1.00E+00	0.08	8.95E-01	9.86E-01
**CTSZ**	0.13	7.03E-01	1.00E+00	0.41	8.95E-01	9.86E-01
**TMEM86B**	-1.09	2.17E-01	1.00E+00	-0.10	8.95E-01	9.86E-01
**G42645**	0.62	3.46E-01	1.00E+00	0.17	8.95E-01	9.86E-01
**SCN3B**	-0.60	2.26E-01	1.00E+00	0.04	8.95E-01	9.86E-01
**AC055713.1**	-0.01	9.89E-01	1.00E+00	0.07	8.95E-01	9.86E-01
**PREX2**	0.46	2.70E-01	1.00E+00	-0.08	8.95E-01	9.86E-01
**RADIL**	0.36	3.63E-01	1.00E+00	-0.09	8.95E-01	9.86E-01
**MIR3682**	1.63	3.56E-01	1.00E+00	-0.09	8.95E-01	9.86E-01
**B3GNTL1**	-0.05	9.31E-01	1.00E+00	0.03	8.95E-01	9.86E-01
**AL031599.1**	0.87	1.89E-01	1.00E+00	-0.14	8.95E-01	9.86E-01
**CYP1A1**	1.80	1.87E-01	1.00E+00	0.41	8.95E-01	9.86E-01
**CDH24**	-0.35	4.17E-01	1.00E+00	0.14	8.95E-01	9.86E-01
**ABRAXAS2**	0.07	6.83E-01	1.00E+00	-0.03	8.95E-01	9.86E-01
**RCC2**	-0.04	8.92E-01	1.00E+00	0.06	8.95E-01	9.86E-01
**AL157392.2**	-0.62	7.72E-01	1.00E+00	0.21	8.95E-01	9.86E-01

**AC124283.2**	-0.95	3.69E-01	1.00E+00	0.13	8.95E-01	9.86E-01
**AL451064.1**	-1.19	7.32E-01	1.00E+00	-0.20	8.95E-01	9.86E-01
**CLTB**	0.25	4.39E-01	1.00E+00	-0.06	8.95E-01	9.86E-01
**AC004987.2**	-0.78	2.92E-01	1.00E+00	0.04	8.95E-01	9.86E-01
**AC129502.1**	2.76	3.10E-01	1.00E+00	-0.13	8.95E-01	9.86E-01
**RPS27AP12**	-1.28	7.07E-01	1.00E+00	0.14	8.95E-01	9.86E-01
**TRABD2B**	-0.02	9.68E-01	1.00E+00	-0.33	8.95E-01	9.86E-01
**AC009948.2**	-1.14	7.38E-01	1.00E+00	-0.08	8.95E-01	9.86E-01
**AL021368.2**	0.31	5.01E-01	1.00E+00	0.07	8.95E-01	9.86E-01
**ZNF699**	0.04	9.45E-01	1.00E+00	0.08	8.95E-01	9.86E-01
**HRK**	-1.04	3.49E-01	1.00E+00	0.12	8.95E-01	9.86E-01
**AC091271.1**	0.56	1.97E-01	1.00E+00	-0.06	8.96E-01	9.86E-01
**PPP1R3G**	0.22	5.51E-01	1.00E+00	-0.08	8.96E-01	9.86E-01
**COL9A2**	-0.30	6.09E-01	1.00E+00	-0.39	8.96E-01	9.86E-01
**KHDC1**	-2.34	9.92E-02	1.00E+00	-0.09	8.96E-01	9.86E-01
**SLC26A6**	-0.24	4.04E-01	1.00E+00	-0.06	8.96E-01	9.86E-01
**RDX**	0.10	7.03E-01	1.00E+00	-0.03	8.96E-01	9.86E-01
**COL4A6**	-0.47	6.84E-02	1.00E+00	-0.06	8.96E-01	9.86E-01
**AL049767.1**	-0.10	9.41E-01	1.00E+00	-0.10	8.96E-01	9.86E-01
**AC087521.3**	-0.40	7.30E-01	1.00E+00	0.12	8.96E-01	9.86E-01
**AC023818.1**	-1.19	4.61E-01	1.00E+00	0.09	8.96E-01	9.86E-01
**PPP1R3F**	-0.33	2.77E-01	1.00E+00	-0.08	8.96E-01	9.86E-01
**NRAP**	-0.96	4.07E-01	1.00E+00	-0.18	8.96E-01	9.86E-01
**STC1**	-0.28	5.52E-01	1.00E+00	0.06	8.96E-01	9.86E-01
**PKN2**	0.17	5.23E-01	1.00E+00	-0.02	8.96E-01	9.86E-01
**AL139022.1**	-1.09	4.04E-01	1.00E+00	-0.12	8.96E-01	9.86E-01
**AC062029.1**	0.15	7.94E-01	1.00E+00	-0.07	8.96E-01	9.86E-01
**PDP2**	0.34	2.22E-01	1.00E+00	0.05	8.96E-01	9.86E-01
**HS1BP3**	0.01	9.53E-01	1.00E+00	0.06	8.96E-01	9.86E-01
**PPP1R3E**	-0.25	2.55E-01	1.00E+00	-0.06	8.96E-01	9.86E-01
**AL671883.2**	-1.95	5.67E-01	1.00E+00	0.12	8.96E-01	9.86E-01
**HMGB1P8**	0.56	8.71E-01	1.00E+00	-0.08	8.96E-01	9.86E-01

**GAPDHP63**	NA	NA	NA	-0.11	8.96E-01	9.86E-01
**GLYCTK**	0.04	9.53E-01	1.00E+00	0.08	8.96E-01	9.86E-01
**SRSF4**	0.13	2.88E-01	1.00E+00	0.03	8.96E-01	9.86E-01
**SMPD2**	-0.15	7.12E-01	1.00E+00	-0.04	8.96E-01	9.86E-01
**CXXC5**	-0.10	5.48E-01	1.00E+00	-0.06	8.96E-01	9.86E-01
**LINC01239**	0.57	8.58E-01	1.00E+00	-0.15	8.96E-01	9.86E-01
**ARHGEF4**	-0.60	1.07E-01	1.00E+00	0.07	8.96E-01	9.86E-01
**COPA**	0.09	5.25E-01	1.00E+00	0.03	8.96E-01	9.86E-01
**AL390066.1**	0.78	5.80E-01	1.00E+00	-0.21	8.96E-01	9.86E-01
**AC008438.1**	-0.56	2.03E-01	1.00E+00	-0.05	8.96E-01	9.86E-01
**RPL12P4**	-0.35	3.62E-01	1.00E+00	0.04	8.96E-01	9.86E-01
**NSUN7**	-0.06	9.05E-01	1.00E+00	0.07	8.97E-01	9.86E-01
**AC097059.1**	-3.40	2.22E-02	8.97E-01	0.10	8.97E-01	9.86E-01
**XLOC_009819**	0.08	9.63E-01	1.00E+00	-0.39	8.97E-01	9.86E-01
**ZBTB3**	-0.13	7.33E-01	1.00E+00	0.05	8.97E-01	9.86E-01
**RPL22P1**	0.04	9.24E-01	1.00E+00	0.06	8.97E-01	9.86E-01
**TIPIN**	-0.08	8.07E-01	1.00E+00	0.04	8.97E-01	9.86E-01
**ADH1A**	1.51	6.62E-01	1.00E+00	0.11	8.97E-01	9.86E-01
**KCNA5**	-0.99	1.87E-01	1.00E+00	-0.39	8.97E-01	9.86E-01
**MAST3**	-0.31	2.13E-01	1.00E+00	-0.04	8.97E-01	9.86E-01
**HOXA-AS2**	-1.20	4.40E-02	1.00E+00	0.09	8.97E-01	9.86E-01
**HSP90B1**	0.00	9.80E-01	1.00E+00	0.03	8.97E-01	9.86E-01
**BMS1P15**	0.16	9.03E-01	1.00E+00	-0.09	8.97E-01	9.86E-01
**CCDC192**	-0.42	6.64E-01	1.00E+00	-0.09	8.97E-01	9.86E-01
**AL109983.1**	NA	NA	NA	0.17	8.97E-01	9.86E-01
**AL035413.1**	-1.52	1.73E-01	1.00E+00	0.08	8.97E-01	9.86E-01
**CEP170P1**	0.68	4.94E-01	1.00E+00	-0.07	8.97E-01	9.86E-01
**ACADS**	0.44	3.61E-01	1.00E+00	-0.07	8.97E-01	9.86E-01
**ARHGAP8**	0.89	3.60E-01	1.00E+00	0.11	8.97E-01	9.86E-01
**AC139495.1**	-0.23	8.69E-01	1.00E+00	0.13	8.97E-01	9.86E-01
**LINC01023**	-0.15	9.66E-01	1.00E+00	-0.10	8.97E-01	9.86E-01
**TBX1**	-0.65	1.32E-01	1.00E+00	-0.29	8.97E-01	9.86E-01

**ALDH1B1**	-0.35	5.16E-01	1.00E+00	0.06	8.97E-01	9.86E-01
**SCN11A**	0.02	9.74E-01	1.00E+00	0.29	8.97E-01	9.86E-01
**PLSCR4**	0.49	1.66E-01	1.00E+00	-0.05	8.97E-01	9.86E-01
**PET117**	1.40	4.33E-01	1.00E+00	0.11	8.98E-01	9.86E-01
**RPL7P9**	-0.03	9.47E-01	1.00E+00	0.09	8.98E-01	9.86E-01
**AC107027.3**	0.40	1.54E-01	1.00E+00	-0.07	8.98E-01	9.86E-01
**LINC00114**	NA	NA	NA	-0.37	8.98E-01	9.86E-01
**HSPA8P4**	1.31	6.98E-01	1.00E+00	0.13	8.98E-01	9.86E-01
**GATAD1**	0.32	1.79E-01	1.00E+00	-0.05	8.98E-01	9.86E-01
**ANKS6**	0.40	2.55E-01	1.00E+00	0.07	8.98E-01	9.86E-01
**AC113174.1**	-1.22	5.82E-01	1.00E+00	0.08	8.98E-01	9.86E-01
**AL139100.1**	-0.90	6.29E-01	1.00E+00	0.40	8.98E-01	9.86E-01
**CDH13**	-0.29	3.47E-01	1.00E+00	0.04	8.98E-01	9.86E-01
**TLDC2**	1.06	4.37E-01	1.00E+00	0.12	8.98E-01	9.86E-01
**GLB1L3**	0.25	2.74E-01	1.00E+00	-0.06	8.98E-01	9.86E-01
**AC009061.1**	0.04	9.61E-01	1.00E+00	-0.14	8.98E-01	9.86E-01
**IBA57**	0.06	8.32E-01	1.00E+00	0.04	8.98E-01	9.86E-01
**XLOC_003111**	0.83	2.51E-01	1.00E+00	-0.08	8.98E-01	9.86E-01
**SLC49A3**	0.10	7.83E-01	1.00E+00	0.12	8.98E-01	9.86E-01
**KRTAP5-1**	-2.88	5.71E-02	1.00E+00	-0.41	8.98E-01	9.86E-01
**ZNF702P**	0.43	5.78E-01	1.00E+00	-0.07	8.98E-01	9.86E-01
**ZNF725P**	1.29	6.59E-01	1.00E+00	0.41	8.98E-01	9.86E-01
**AC009318.2**	-0.65	5.95E-01	1.00E+00	0.10	8.98E-01	9.86E-01
**GNAQ**	-0.01	9.84E-01	1.00E+00	0.05	8.98E-01	9.86E-01
**GRIK1**	-0.15	8.11E-01	1.00E+00	0.09	8.99E-01	9.86E-01
**C2orf74**	0.92	7.17E-01	1.00E+00	-0.10	8.99E-01	9.86E-01
**AC012313.5**	0.48	3.71E-01	1.00E+00	-0.11	8.99E-01	9.86E-01
**PFKFB3**	0.55	3.33E-01	1.00E+00	-0.06	8.99E-01	9.86E-01
**GYS1**	0.02	9.20E-01	1.00E+00	-0.06	8.99E-01	9.86E-01
**RNF17**	-3.61	2.29E-01	1.00E+00	0.20	8.99E-01	9.86E-01
**SLC9C1**	1.00	2.86E-01	1.00E+00	-0.10	8.99E-01	9.86E-01
**GEMIN2**	0.46	1.45E-01	1.00E+00	-0.04	8.99E-01	9.86E-01

**KDM8**	-0.35	2.98E-01	1.00E+00	0.07	8.99E-01	9.86E-01
**TP63**	-0.12	7.42E-01	1.00E+00	-0.07	8.99E-01	9.86E-01
**ZC3H15**	0.09	6.67E-01	1.00E+00	-0.02	8.99E-01	9.86E-01
**ABCG5**	0.57	7.42E-01	1.00E+00	0.14	8.99E-01	9.86E-01
**PSMB3**	-0.29	1.37E-01	1.00E+00	-0.03	8.99E-01	9.86E-01
**POT1**	0.33	2.04E-01	1.00E+00	-0.03	8.99E-01	9.86E-01
**B4GALNT4**	-0.81	1.27E-01	1.00E+00	-0.40	8.99E-01	9.86E-01
**AC092053.2**	-0.39	8.05E-01	1.00E+00	-0.14	8.99E-01	9.86E-01
**AC080037.1**	-1.91	3.51E-01	1.00E+00	-0.16	8.99E-01	9.86E-01
**LRRC61**	0.04	9.05E-01	1.00E+00	0.06	8.99E-01	9.86E-01
**KANSL2**	0.09	6.50E-01	1.00E+00	0.03	8.99E-01	9.86E-01
**ZNF334**	0.49	5.62E-01	1.00E+00	0.08	8.99E-01	9.86E-01
**AP5Z1**	0.01	9.72E-01	1.00E+00	-0.05	8.99E-01	9.86E-01
**SEPT7P9**	-0.25	8.66E-01	1.00E+00	-0.09	8.99E-01	9.86E-01
**PLPP6**	-0.33	3.13E-01	1.00E+00	-0.03	8.99E-01	9.86E-01
**PDLIM5**	0.13	5.21E-01	1.00E+00	0.02	9.00E-01	9.86E-01
**OSBPL2**	0.15	5.95E-01	1.00E+00	0.02	9.00E-01	9.86E-01
**RNF181**	0.10	5.73E-01	1.00E+00	0.04	9.00E-01	9.86E-01
**AL513123.1**	-1.55	6.55E-01	1.00E+00	0.24	9.00E-01	9.86E-01
**RAPGEF3**	0.08	8.60E-01	1.00E+00	0.07	9.00E-01	9.86E-01
**AC092634.3**	2.63	1.13E-01	1.00E+00	-0.13	9.00E-01	9.86E-01
**AL358472.1**	NA	NA	NA	0.40	9.00E-01	9.86E-01
**AC135178.3**	0.55	3.89E-01	1.00E+00	0.08	9.00E-01	9.86E-01
**GART**	0.03	8.95E-01	1.00E+00	0.03	9.00E-01	9.86E-01
**PPIAP2**	-2.48	4.68E-01	1.00E+00	0.19	9.00E-01	9.86E-01
**OTC**	-0.23	9.31E-01	1.00E+00	0.23	9.00E-01	9.86E-01
**PSMD2**	0.04	8.53E-01	1.00E+00	0.03	9.00E-01	9.86E-01
**AC110749.1**	-1.60	5.52E-01	1.00E+00	-0.08	9.00E-01	9.86E-01
**SLC10A3**	-0.17	5.70E-01	1.00E+00	-0.03	9.00E-01	9.86E-01
**XLOC_006476**	0.28	8.14E-01	1.00E+00	0.14	9.00E-01	9.86E-01
**STAC**	-0.12	8.43E-01	1.00E+00	0.07	9.00E-01	9.86E-01
**TRAP1**	-0.15	4.50E-01	1.00E+00	-0.03	9.00E-01	9.86E-01

**MAX**	0.20	2.29E-01	1.00E+00	0.04	9.00E-01	9.86E-01
**ZPR1**	0.04	7.99E-01	1.00E+00	-0.04	9.01E-01	9.86E-01
**PICK1**	0.00	9.84E-01	1.00E+00	-0.10	9.01E-01	9.86E-01
**XLOC_014263**	-1.78	3.83E-01	1.00E+00	0.18	9.01E-01	9.86E-01
**DDAH1**	0.25	5.08E-01	1.00E+00	-0.04	9.01E-01	9.86E-01
**AC025419.1**	0.85	7.58E-01	1.00E+00	-0.14	9.01E-01	9.86E-01
**DCLRE1C**	0.23	4.82E-01	1.00E+00	0.04	9.01E-01	9.87E-01
**TAF5**	-0.15	7.27E-01	1.00E+00	-0.06	9.01E-01	9.87E-01
**CU639417.3**	-0.54	4.67E-01	1.00E+00	-0.08	9.01E-01	9.87E-01
**DENND1B**	-0.13	6.81E-01	1.00E+00	-0.05	9.01E-01	9.87E-01
**WARS**	-0.32	3.71E-01	1.00E+00	0.07	9.01E-01	9.87E-01
**AC008155.1**	-1.95	5.70E-01	1.00E+00	0.11	9.01E-01	9.87E-01
**CDK1**	0.34	4.50E-01	1.00E+00	0.04	9.01E-01	9.87E-01
**MMP28**	0.27	4.90E-01	1.00E+00	0.04	9.01E-01	9.87E-01
**DPM3**	0.23	2.53E-01	1.00E+00	-0.02	9.01E-01	9.87E-01
**LAD1**	-0.26	5.73E-01	1.00E+00	0.08	9.01E-01	9.87E-01
**AC016757.1**	0.79	3.62E-01	1.00E+00	0.13	9.01E-01	9.87E-01
**SH3RF2**	0.06	8.77E-01	1.00E+00	-0.06	9.01E-01	9.87E-01
**IL17RE**	-0.07	8.44E-01	1.00E+00	0.07	9.01E-01	9.87E-01
**AC097375.3**	1.31	3.17E-01	1.00E+00	-0.39	9.02E-01	9.87E-01
**HIPK4**	0.15	8.01E-01	1.00E+00	0.17	9.02E-01	9.87E-01
**TNKS2**	0.15	5.09E-01	1.00E+00	0.06	9.02E-01	9.87E-01
**SRCAP**	-0.32	4.40E-01	1.00E+00	0.07	9.02E-01	9.87E-01
**TP73-AS1**	-0.15	6.14E-01	1.00E+00	0.07	9.02E-01	9.87E-01
**CLTRN**	-0.44	6.24E-01	1.00E+00	-0.08	9.02E-01	9.87E-01
**CFAP61**	0.13	8.72E-01	1.00E+00	-0.08	9.02E-01	9.87E-01
**STAG1**	-0.19	3.92E-01	1.00E+00	-0.03	9.02E-01	9.87E-01
**ZNF311**	0.12	7.65E-01	1.00E+00	0.09	9.02E-01	9.87E-01
**MARVELD2**	0.09	7.23E-01	1.00E+00	-0.05	9.02E-01	9.87E-01
**FAM183A**	-0.24	7.05E-01	1.00E+00	-0.08	9.02E-01	9.87E-01
**EPN3**	-0.19	6.62E-01	1.00E+00	-0.10	9.02E-01	9.87E-01
**C1orf198**	-0.02	9.37E-01	1.00E+00	0.03	9.02E-01	9.87E-01

**RAB3D**	0.02	9.50E-01	1.00E+00	0.04	9.02E-01	9.87E-01
**NSG1**	0.10	8.07E-01	1.00E+00	0.07	9.02E-01	9.87E-01
**LGR6**	-0.48	2.58E-01	1.00E+00	0.07	9.02E-01	9.87E-01
**AL137145.2**	-0.09	9.75E-01	1.00E+00	0.13	9.02E-01	9.87E-01
**AC012174.1**	1.27	7.13E-01	1.00E+00	-0.21	9.02E-01	9.87E-01
**ABCB5**	0.54	6.22E-01	1.00E+00	0.14	9.02E-01	9.87E-01
**TUBA1B**	-0.10	6.63E-01	1.00E+00	0.04	9.02E-01	9.87E-01
**RPSAP6**	NA	NA	NA	0.23	9.02E-01	9.87E-01
**NCOA3**	-0.02	9.41E-01	1.00E+00	0.03	9.03E-01	9.87E-01
**AL031708.1**	0.84	6.17E-01	1.00E+00	-0.14	9.03E-01	9.87E-01
**ATG4A**	0.21	3.07E-01	1.00E+00	0.03	9.03E-01	9.87E-01
**CETP**	0.33	6.44E-01	1.00E+00	0.10	9.03E-01	9.87E-01
**ZNF497**	-0.60	2.56E-01	1.00E+00	-0.11	9.03E-01	9.87E-01
**PCDHB6**	-0.37	3.51E-01	1.00E+00	0.08	9.03E-01	9.87E-01
**HSPB1**	-0.26	4.67E-01	1.00E+00	-0.05	9.03E-01	9.87E-01
**FANCD2**	-0.29	4.28E-01	1.00E+00	-0.05	9.03E-01	9.87E-01
**G41963**	-0.61	5.60E-01	1.00E+00	0.10	9.03E-01	9.87E-01
**AC090229.1**	0.05	9.59E-01	1.00E+00	0.10	9.03E-01	9.87E-01
**HINFP**	-0.27	2.80E-01	1.00E+00	0.03	9.03E-01	9.87E-01
**AC008760.1**	-1.55	3.69E-01	1.00E+00	0.14	9.03E-01	9.87E-01
**AC131235.1**	-1.73	2.42E-01	1.00E+00	-0.08	9.03E-01	9.87E-01
**SUPT16H**	-0.03	8.89E-01	1.00E+00	-0.01	9.03E-01	9.87E-01
**LINC02601**	1.21	6.28E-01	1.00E+00	0.12	9.03E-01	9.87E-01
**ACD**	-0.16	4.58E-01	1.00E+00	0.03	9.03E-01	9.87E-01
**ADH1B**	1.54	5.15E-03	4.18E-01	0.09	9.03E-01	9.87E-01
**ASH1L**	-0.11	5.38E-01	1.00E+00	0.03	9.03E-01	9.87E-01
**AC005042.2**	0.12	9.10E-01	1.00E+00	0.13	9.03E-01	9.87E-01
**G25761**	-2.68	2.63E-01	1.00E+00	0.18	9.03E-01	9.87E-01
**TENT5C**	-0.11	6.85E-01	1.00E+00	-0.05	9.03E-01	9.87E-01
**SCUBE1**	0.93	1.38E-01	1.00E+00	-0.11	9.04E-01	9.87E-01
**MTUS1**	0.19	5.75E-01	1.00E+00	0.03	9.04E-01	9.87E-01
**XLOC_002952**	-2.63	8.62E-02	1.00E+00	-0.10	9.04E-01	9.87E-01

**G26933**	-1.07	3.56E-01	1.00E+00	-0.09	9.04E-01	9.87E-01
**FAM19A5**	0.61	9.82E-02	1.00E+00	0.09	9.04E-01	9.87E-01
**TERB2**	-0.15	9.67E-01	1.00E+00	0.25	9.04E-01	9.87E-01
**RRM2P3**	0.34	7.50E-01	1.00E+00	-0.13	9.04E-01	9.87E-01
**AL139384.1**	-0.24	5.27E-01	1.00E+00	-0.05	9.04E-01	9.87E-01
**AC007790.1**	-1.17	7.37E-01	1.00E+00	0.33	9.04E-01	9.87E-01
**AC027309.2**	-0.91	4.37E-01	1.00E+00	-0.10	9.04E-01	9.87E-01
**GPR156**	-1.49	2.11E-02	8.94E-01	0.10	9.04E-01	9.87E-01
**LINC02560**	0.27	5.39E-01	1.00E+00	0.07	9.04E-01	9.87E-01
**ABHD4**	0.19	4.81E-01	1.00E+00	0.04	9.04E-01	9.87E-01
**FLOT2**	-0.01	9.80E-01	1.00E+00	-0.05	9.04E-01	9.87E-01
**TAF7L**	-0.60	5.84E-01	1.00E+00	0.13	9.04E-01	9.87E-01
**RPSAP69**	0.57	6.40E-01	1.00E+00	-0.12	9.04E-01	9.87E-01
**ZNF607**	0.83	3.21E-02	1.00E+00	0.06	9.04E-01	9.87E-01
**AC040977.1**	-0.03	9.32E-01	1.00E+00	-0.06	9.05E-01	9.87E-01
**UBQLN4**	-0.22	2.26E-01	1.00E+00	0.07	9.05E-01	9.87E-01
**ZNF439**	0.15	7.19E-01	1.00E+00	-0.03	9.05E-01	9.87E-01
**FRK**	0.11	7.50E-01	1.00E+00	0.06	9.05E-01	9.87E-01
**HSPD1P3**	-1.10	7.53E-01	1.00E+00	0.39	9.05E-01	9.87E-01
**GRIN3B**	-2.26	1.01E-01	1.00E+00	0.38	9.05E-01	9.87E-01
**AL023803.2**	0.54	6.69E-01	1.00E+00	-0.15	9.05E-01	9.87E-01
**LINC00570**	-2.99	8.04E-02	1.00E+00	0.38	9.05E-01	9.87E-01
**AC004877.2**	-0.36	7.23E-01	1.00E+00	-0.09	9.05E-01	9.87E-01
**KCNB1**	1.12	1.50E-01	1.00E+00	-0.10	9.05E-01	9.87E-01
**PPP1CA**	-0.28	2.40E-01	1.00E+00	0.03	9.05E-01	9.87E-01
**RBFOX3**	-2.29	1.03E-01	1.00E+00	0.16	9.05E-01	9.87E-01
**PARG**	-0.03	9.00E-01	1.00E+00	-0.02	9.05E-01	9.87E-01
**PYROXD2**	0.75	3.42E-01	1.00E+00	0.05	9.05E-01	9.87E-01
**RAB29**	0.20	6.25E-01	1.00E+00	0.05	9.05E-01	9.87E-01
**CACNA1G**	0.36	5.83E-01	1.00E+00	0.37	9.05E-01	9.87E-01
**TMEM151A**	-3.55	2.15E-03	2.39E-01	-0.14	9.05E-01	9.87E-01
**RSPO4**	-0.54	5.66E-01	1.00E+00	0.37	9.05E-01	9.87E-01

**PCDHA3**	1.20	2.18E-01	1.00E+00	-0.36	9.05E-01	9.87E-01
**TPM2**	-0.61	2.94E-01	1.00E+00	-0.05	9.05E-01	9.87E-01
**NUDT16L1**	-0.23	3.11E-01	1.00E+00	-0.03	9.05E-01	9.87E-01
**ARMH3**	-0.05	7.90E-01	1.00E+00	0.03	9.05E-01	9.87E-01
**C8orf88**	-0.58	1.21E-01	1.00E+00	-0.05	9.05E-01	9.87E-01
**AL591379.1**	-0.87	8.00E-01	1.00E+00	0.17	9.05E-01	9.87E-01
**AC002558.3**	-0.20	8.70E-01	1.00E+00	0.09	9.05E-01	9.87E-01
**PMS2P7**	-0.42	5.88E-01	1.00E+00	-0.06	9.06E-01	9.87E-01
**TRDC**	0.15	8.93E-01	1.00E+00	-0.08	9.06E-01	9.87E-01
**SH3GL3**	-0.83	2.37E-01	1.00E+00	-0.12	9.06E-01	9.87E-01
**NIN**	-0.25	3.69E-01	1.00E+00	0.05	9.06E-01	9.87E-01
**AP003084.1**	0.46	5.95E-01	1.00E+00	-0.12	9.06E-01	9.87E-01
**SPR**	0.08	8.31E-01	1.00E+00	-0.04	9.06E-01	9.87E-01
**METTL2B**	-0.05	8.51E-01	1.00E+00	0.04	9.06E-01	9.87E-01
**TRIM35**	0.15	7.35E-01	1.00E+00	-0.04	9.06E-01	9.87E-01
**SORT1**	0.16	5.40E-01	1.00E+00	-0.03	9.06E-01	9.87E-01
**PDXDC1**	0.22	3.75E-01	1.00E+00	-0.03	9.06E-01	9.87E-01
**CYB5R2**	-0.02	9.31E-01	1.00E+00	-0.04	9.06E-01	9.87E-01
**ARHGAP27P2**	-2.17	8.96E-02	1.00E+00	-0.15	9.06E-01	9.87E-01
**PTPN5**	-1.08	3.02E-01	1.00E+00	-0.14	9.06E-01	9.87E-01
**KRTAP9-7**	-6.41	6.55E-02	1.00E+00	0.38	9.06E-01	9.87E-01
**ZNF483**	-0.05	9.42E-01	1.00E+00	0.08	9.06E-01	9.87E-01
**LINC01229**	0.67	3.73E-01	1.00E+00	-0.09	9.06E-01	9.87E-01
**USP6NL**	-0.15	5.97E-01	1.00E+00	-0.04	9.06E-01	9.87E-01
**TMPO-AS1**	-0.13	8.24E-01	1.00E+00	-0.09	9.06E-01	9.87E-01
**LINC01527**	3.16	3.50E-01	1.00E+00	0.18	9.06E-01	9.87E-01
**ACO2**	-0.10	6.78E-01	1.00E+00	-0.03	9.06E-01	9.87E-01
**MT1M**	0.00	9.93E-01	1.00E+00	0.04	9.06E-01	9.87E-01
**SIM1**	1.23	1.05E-02	6.33E-01	-0.28	9.06E-01	9.87E-01
**GALNT7**	0.53	4.66E-01	1.00E+00	0.05	9.06E-01	9.87E-01
**CFTR**	-0.28	6.61E-01	1.00E+00	-0.08	9.06E-01	9.87E-01
**ZCCHC7**	0.10	5.70E-01	1.00E+00	-0.02	9.06E-01	9.87E-01

**BMP4**	0.28	2.47E-01	1.00E+00	0.04	9.07E-01	9.87E-01
**BX537318.2**	-1.03	5.22E-01	1.00E+00	0.27	9.07E-01	9.87E-01
**AC098864.1**	0.59	6.73E-01	1.00E+00	0.12	9.07E-01	9.87E-01
**DDIT3**	-0.10	8.15E-01	1.00E+00	0.03	9.07E-01	9.87E-01
**TXNP4**	-0.72	8.35E-01	1.00E+00	-0.10	9.07E-01	9.87E-01
**HCFC2**	0.22	5.60E-01	1.00E+00	0.03	9.07E-01	9.87E-01
**KCNJ12**	0.07	9.04E-01	1.00E+00	0.06	9.07E-01	9.87E-01
**AC092755.2**	1.81	5.94E-01	1.00E+00	-0.14	9.07E-01	9.87E-01
**AC007038.2**	-2.35	3.26E-01	1.00E+00	-0.11	9.07E-01	9.87E-01
**IRF6**	-0.09	7.69E-01	1.00E+00	-0.05	9.07E-01	9.87E-01
**PIMREG**	0.23	5.71E-01	1.00E+00	0.06	9.07E-01	9.87E-01
**AL109618.1**	0.93	5.85E-01	1.00E+00	-0.06	9.07E-01	9.87E-01
**JAM2**	-0.07	8.71E-01	1.00E+00	-0.06	9.07E-01	9.87E-01
**MDP1**	NA	NA	NA	-0.13	9.07E-01	9.87E-01
**SPOUT1**	-0.01	9.74E-01	1.00E+00	0.02	9.07E-01	9.87E-01
**RECK**	1.00	1.99E-02	8.80E-01	0.05	9.07E-01	9.87E-01
**PARD6B**	0.44	2.29E-01	1.00E+00	-0.06	9.07E-01	9.87E-01
**HS6ST2**	-0.01	9.89E-01	1.00E+00	0.13	9.07E-01	9.87E-01
**MAMDC2-AS1**	0.72	4.70E-01	1.00E+00	-0.08	9.07E-01	9.87E-01
**AC132812.1**	0.06	9.41E-01	1.00E+00	0.09	9.07E-01	9.87E-01
**CYP4X1**	0.14	8.18E-01	1.00E+00	0.06	9.07E-01	9.87E-01
**G19270**	1.00	3.50E-01	1.00E+00	-0.37	9.07E-01	9.87E-01
**BTG3-AS1**	0.39	4.57E-01	1.00E+00	0.06	9.07E-01	9.87E-01
**BCL11B**	-0.34	4.08E-01	1.00E+00	-0.06	9.07E-01	9.87E-01
**KRT83**	-2.94	3.78E-01	1.00E+00	-0.37	9.07E-01	9.87E-01
**DTD2**	0.20	4.02E-01	1.00E+00	-0.04	9.07E-01	9.87E-01
**C10orf113**	0.44	8.99E-01	1.00E+00	-0.20	9.08E-01	9.87E-01
**B3GALT6**	-0.14	5.13E-01	1.00E+00	0.07	9.08E-01	9.87E-01
**FNDC4**	0.41	5.19E-01	1.00E+00	-0.08	9.08E-01	9.87E-01
**POSTN**	-0.33	2.30E-01	1.00E+00	-0.05	9.08E-01	9.87E-01
**SAP30L-AS1**	-0.73	4.41E-01	1.00E+00	-0.05	9.08E-01	9.87E-01
**UROD**	0.00	9.87E-01	1.00E+00	-0.03	9.08E-01	9.87E-01

**KCNE5**	-2.43	2.16E-01	1.00E+00	0.11	9.08E-01	9.87E-01
**G17127**	-0.10	9.03E-01	1.00E+00	-0.04	9.08E-01	9.87E-01
**Z83826.1**	0.74	4.51E-01	1.00E+00	-0.10	9.08E-01	9.87E-01
**AL357507.1**	-2.49	4.24E-01	1.00E+00	0.12	9.08E-01	9.87E-01
**CDX4**	0.95	1.87E-01	1.00E+00	-0.12	9.08E-01	9.87E-01
**AP002414.2**	0.11	9.21E-01	1.00E+00	-0.16	9.08E-01	9.87E-01
**ADAM11**	-0.97	1.32E-01	1.00E+00	0.09	9.08E-01	9.87E-01
**ABCB8**	-0.23	3.18E-01	1.00E+00	0.04	9.08E-01	9.87E-01
**CMTR2**	0.34	2.93E-01	1.00E+00	0.03	9.08E-01	9.87E-01
**HOXB7**	-0.03	9.47E-01	1.00E+00	0.04	9.08E-01	9.87E-01
**ZFP57**	-3.77	7.96E-04	1.40E-01	0.21	9.08E-01	9.87E-01
**PDZRN3**	-0.07	9.00E-01	1.00E+00	0.06	9.08E-01	9.87E-01
**NCR3LG1**	-0.05	9.33E-01	1.00E+00	0.07	9.08E-01	9.87E-01
**RTL8C**	-0.07	7.87E-01	1.00E+00	-0.03	9.08E-01	9.87E-01
**AC073072.1**	0.34	9.23E-01	1.00E+00	-0.37	9.08E-01	9.87E-01
**AC055854.1**	-1.34	1.34E-01	1.00E+00	0.09	9.09E-01	9.87E-01
**RILP**	0.02	9.55E-01	1.00E+00	-0.08	9.09E-01	9.87E-01
**ESRP2**	-0.26	4.97E-01	1.00E+00	-0.07	9.09E-01	9.87E-01
**EIF2A**	-0.21	4.09E-01	1.00E+00	-0.03	9.09E-01	9.87E-01
**ZNF236**	-0.18	5.59E-01	1.00E+00	0.03	9.09E-01	9.87E-01
**RPUSD4**	0.08	7.09E-01	1.00E+00	-0.02	9.09E-01	9.87E-01
**BBOX1**	-0.18	6.35E-01	1.00E+00	-0.07	9.09E-01	9.87E-01
**ZNF878**	-2.34	4.06E-01	1.00E+00	0.19	9.09E-01	9.87E-01
**CTAGE15**	-0.24	8.72E-01	1.00E+00	0.10	9.09E-01	9.87E-01
**ERC1**	-0.23	2.67E-01	1.00E+00	-0.04	9.09E-01	9.87E-01
**AC019131.2**	-0.74	2.10E-01	1.00E+00	-0.06	9.09E-01	9.87E-01
**AL355607.2**	-0.88	7.29E-01	1.00E+00	0.09	9.09E-01	9.87E-01
**AC010203.1**	0.06	9.78E-01	1.00E+00	0.14	9.09E-01	9.87E-01
**ZNF81**	0.01	9.57E-01	1.00E+00	-0.04	9.09E-01	9.87E-01
**GLI1**	0.14	8.05E-01	1.00E+00	-0.10	9.09E-01	9.87E-01
**BNIPL**	-0.02	9.70E-01	1.00E+00	0.07	9.09E-01	9.87E-01
**SP2-AS1**	-0.36	3.95E-01	1.00E+00	0.05	9.09E-01	9.87E-01

**CCDC30**	0.19	5.26E-01	1.00E+00	-0.04	9.09E-01	9.87E-01
**AC125257.1**	0.62	8.74E-02	1.00E+00	0.05	9.09E-01	9.87E-01
**TEX2**	-0.09	5.95E-01	1.00E+00	-0.03	9.09E-01	9.87E-01
**RNF38**	0.13	5.16E-01	1.00E+00	0.03	9.09E-01	9.87E-01
**RPL23AP53**	-0.27	4.59E-01	1.00E+00	0.03	9.09E-01	9.87E-01
**DESI2**	0.21	3.98E-01	1.00E+00	0.04	9.09E-01	9.87E-01
**SARNP**	0.37	8.11E-01	1.00E+00	-0.12	9.09E-01	9.87E-01
**EGF**	0.36	5.56E-01	1.00E+00	-0.10	9.09E-01	9.87E-01
**PGAP2**	-0.22	4.59E-01	1.00E+00	-0.04	9.09E-01	9.87E-01
**LFNG**	-0.05	8.55E-01	1.00E+00	0.05	9.10E-01	9.87E-01
**ANGPTL4**	0.80	1.93E-01	1.00E+00	-0.06	9.10E-01	9.88E-01
**SATB2**	-0.04	8.97E-01	1.00E+00	-0.05	9.10E-01	9.88E-01
**G34301**	0.93	3.25E-01	1.00E+00	-0.13	9.10E-01	9.88E-01
**NAP1L4**	0.18	2.49E-01	1.00E+00	-0.02	9.10E-01	9.88E-01
**AC135352.1**	-2.14	2.21E-01	1.00E+00	0.13	9.10E-01	9.88E-01
**AMOTL2**	0.05	8.12E-01	1.00E+00	0.05	9.10E-01	9.88E-01
**GALNT9**	-0.56	8.67E-01	1.00E+00	0.22	9.10E-01	9.88E-01
**ZNF670**	-0.17	7.10E-01	1.00E+00	-0.05	9.10E-01	9.88E-01
**UBE2L5**	0.31	6.46E-01	1.00E+00	0.07	9.10E-01	9.88E-01
**OR2A1-AS1**	0.30	6.49E-01	1.00E+00	-0.07	9.10E-01	9.88E-01
**ZBED3**	-0.14	7.43E-01	1.00E+00	-0.07	9.10E-01	9.88E-01
**AQP8**	-0.90	4.65E-01	1.00E+00	0.17	9.10E-01	9.88E-01
**MAPK13**	-0.04	9.10E-01	1.00E+00	0.04	9.10E-01	9.88E-01
**HPS3**	-0.17	4.38E-01	1.00E+00	-0.04	9.10E-01	9.88E-01
**COA1**	-0.06	7.64E-01	1.00E+00	-0.02	9.10E-01	9.88E-01
**AHSA2P**	0.68	2.19E-01	1.00E+00	-0.07	9.11E-01	9.88E-01
**CASTOR1**	0.53	7.98E-01	1.00E+00	0.08	9.11E-01	9.88E-01
**GOLGA6L9**	-0.65	3.32E-01	1.00E+00	0.09	9.11E-01	9.88E-01
**SRY**	1.28	2.35E-01	1.00E+00	-0.14	9.11E-01	9.88E-01
**USP27X-AS1**	0.65	1.01E-01	1.00E+00	0.07	9.11E-01	9.88E-01
**HECTD2**	-0.10	7.82E-01	1.00E+00	-0.04	9.11E-01	9.88E-01
**TMUB1**	-0.07	7.25E-01	1.00E+00	-0.08	9.11E-01	9.88E-01

**AC103703.1**	-1.25	7.13E-01	1.00E+00	-0.15	9.11E-01	9.88E-01
**CLLU1OS**	-1.24	7.19E-01	1.00E+00	0.23	9.11E-01	9.88E-01
**ST13P15**	-0.27	8.96E-01	1.00E+00	0.06	9.11E-01	9.88E-01
**SRGAP2C**	-0.01	9.79E-01	1.00E+00	-0.06	9.11E-01	9.88E-01
**CYP2D6**	-2.18	6.37E-02	1.00E+00	-0.08	9.11E-01	9.88E-01
**EXOC3-AS1**	-0.24	3.62E-01	1.00E+00	0.05	9.11E-01	9.88E-01
**CDR2**	0.18	2.95E-01	1.00E+00	0.05	9.11E-01	9.88E-01
**TAF8**	-0.01	9.73E-01	1.00E+00	0.02	9.11E-01	9.88E-01
**AC004000.1**	-0.57	7.47E-01	1.00E+00	0.14	9.11E-01	9.88E-01
**AL034417.4**	1.39	3.19E-01	1.00E+00	0.11	9.11E-01	9.88E-01
**NDUFA5P11**	NA	NA	NA	-0.12	9.11E-01	9.88E-01
**SINHCAFP2**	NA	NA	NA	0.09	9.12E-01	9.88E-01
**WHAMMP2**	0.84	3.63E-01	1.00E+00	0.08	9.12E-01	9.88E-01
**HYAL4**	-1.36	6.12E-02	1.00E+00	-0.28	9.12E-01	9.88E-01
**LRFN2**	0.63	6.36E-01	1.00E+00	-0.17	9.12E-01	9.88E-01
**PRKCZ-AS1**	-0.75	2.98E-01	1.00E+00	0.11	9.12E-01	9.88E-01
**MGC16275**	0.13	8.39E-01	1.00E+00	-0.12	9.12E-01	9.88E-01
**FBXO5**	-0.01	9.70E-01	1.00E+00	0.03	9.12E-01	9.88E-01
**PKMP3**	0.62	6.15E-01	1.00E+00	0.12	9.12E-01	9.88E-01
**ORC2**	0.03	9.19E-01	1.00E+00	-0.03	9.12E-01	9.88E-01
**PKMP1**	NA	NA	NA	0.07	9.12E-01	9.88E-01
**CD59**	0.42	2.93E-01	1.00E+00	-0.03	9.12E-01	9.88E-01
**SLC46A2**	0.12	8.65E-01	1.00E+00	-0.07	9.12E-01	9.88E-01
**RPS3AP47**	0.05	9.51E-01	1.00E+00	-0.09	9.12E-01	9.88E-01
**PGM5-AS1**	1.62	3.38E-02	1.00E+00	-0.07	9.12E-01	9.88E-01
**MRPL12**	-0.40	4.45E-01	1.00E+00	0.05	9.12E-01	9.88E-01
**ZNF189**	0.14	5.29E-01	1.00E+00	0.03	9.13E-01	9.88E-01
**BHLHE41**	-0.79	1.28E-01	1.00E+00	-0.06	9.13E-01	9.88E-01
**KLHL12**	0.37	6.82E-02	1.00E+00	0.02	9.13E-01	9.88E-01
**LRIG3**	-0.18	4.63E-01	1.00E+00	-0.02	9.13E-01	9.88E-01
**VAC14**	-0.30	1.08E-01	1.00E+00	0.06	9.13E-01	9.88E-01
**ITGB3**	0.31	5.06E-01	1.00E+00	0.09	9.13E-01	9.88E-01

**AC078817.1**	-1.79	5.88E-01	1.00E+00	0.34	9.13E-01	9.88E-01
**NKIRAS2**	-0.14	4.96E-01	1.00E+00	-0.02	9.13E-01	9.88E-01
**GATA1**	1.64	1.72E-01	1.00E+00	0.15	9.13E-01	9.88E-01
**G8320**	-3.78	5.10E-03	4.18E-01	0.11	9.13E-01	9.88E-01
**AC025459.1**	-1.76	6.09E-01	1.00E+00	0.09	9.13E-01	9.89E-01
**DNAH9**	-0.46	3.47E-01	1.00E+00	0.05	9.13E-01	9.89E-01
**HGH1**	0.05	9.28E-01	1.00E+00	-0.10	9.13E-01	9.89E-01
**G24464**	-0.30	6.94E-01	1.00E+00	-0.10	9.13E-01	9.89E-01
**OTOP3**	-0.24	8.77E-01	1.00E+00	0.10	9.13E-01	9.89E-01
**PHKA2**	-0.24	3.72E-01	1.00E+00	-0.05	9.13E-01	9.89E-01
**FAXC**	-0.33	6.37E-01	1.00E+00	0.08	9.13E-01	9.89E-01
**VWA8-AS1**	-2.43	2.62E-01	1.00E+00	-0.14	9.13E-01	9.89E-01
**CABLES2**	-0.44	1.10E-01	1.00E+00	-0.08	9.13E-01	9.89E-01
**INSC**	-0.14	8.02E-01	1.00E+00	0.16	9.13E-01	9.89E-01
**PRELID3B**	0.04	8.74E-01	1.00E+00	0.04	9.14E-01	9.89E-01
**G7052**	2.50	5.02E-02	1.00E+00	-0.10	9.14E-01	9.89E-01
**FAM25BP**	1.06	7.31E-01	1.00E+00	-0.09	9.14E-01	9.89E-01
**AC079148.1**	0.25	8.30E-01	1.00E+00	-0.14	9.14E-01	9.89E-01
**TNNT3**	-1.56	1.59E-03	2.22E-01	-0.06	9.14E-01	9.89E-01
**MHENCR**	-0.48	2.43E-01	1.00E+00	-0.04	9.14E-01	9.89E-01
**GPR19**	0.56	5.80E-01	1.00E+00	0.09	9.14E-01	9.89E-01
**XLOC_011034**	NA	NA	NA	-0.08	9.14E-01	9.89E-01
**TCERG1**	-0.31	2.12E-01	1.00E+00	-0.02	9.14E-01	9.89E-01
**MAF1**	0.08	7.02E-01	1.00E+00	0.04	9.14E-01	9.89E-01
**AP001999.1**	1.46	2.07E-01	1.00E+00	-0.20	9.14E-01	9.89E-01
**RHPN1**	-1.31	1.29E-04	3.03E-02	0.09	9.14E-01	9.89E-01
**TNK1**	-0.31	1.92E-01	1.00E+00	0.06	9.14E-01	9.89E-01
**EPHX1**	0.65	2.29E-01	1.00E+00	0.04	9.14E-01	9.89E-01
**GPRIN2**	0.06	9.08E-01	1.00E+00	0.07	9.14E-01	9.89E-01
**PKP3**	-0.28	5.86E-01	1.00E+00	-0.07	9.14E-01	9.89E-01
**TMEM254-AS1**	-0.05	8.88E-01	1.00E+00	0.05	9.14E-01	9.89E-01
**KCTD8**	1.71	1.49E-01	1.00E+00	0.11	9.14E-01	9.89E-01

**AC104971.1**	-2.14	3.22E-01	1.00E+00	0.13	9.14E-01	9.89E-01
**RNPEPL1**	-0.02	9.36E-01	1.00E+00	-0.06	9.15E-01	9.89E-01
**AC006441.1**	0.76	7.25E-01	1.00E+00	-0.10	9.15E-01	9.89E-01
**SEC24B-AS1**	0.65	2.26E-01	1.00E+00	-0.07	9.15E-01	9.89E-01
**ARNT**	0.33	1.47E-01	1.00E+00	-0.02	9.15E-01	9.89E-01
**AC008764.8**	-1.93	9.93E-02	1.00E+00	0.05	9.15E-01	9.89E-01
**NRF1**	-0.13	5.34E-01	1.00E+00	0.04	9.15E-01	9.89E-01
**AC108673.3**	-0.01	9.94E-01	1.00E+00	0.06	9.15E-01	9.89E-01
**GAB3**	0.19	6.93E-01	1.00E+00	0.07	9.15E-01	9.89E-01
**G2112**	-0.25	8.39E-01	1.00E+00	-0.09	9.15E-01	9.89E-01
**EIF2B2**	-0.29	8.77E-02	1.00E+00	0.02	9.15E-01	9.89E-01
**AC093162.2**	-0.16	7.12E-01	1.00E+00	0.08	9.15E-01	9.89E-01
**KIF25-AS1**	-0.59	7.02E-01	1.00E+00	0.17	9.15E-01	9.89E-01
**AC007496.2**	0.90	4.93E-01	1.00E+00	-0.12	9.15E-01	9.89E-01
**MAP2K6**	0.16	4.34E-01	1.00E+00	0.03	9.15E-01	9.89E-01
**AL646090.2**	-1.42	5.65E-01	1.00E+00	0.12	9.15E-01	9.89E-01
**FXR2**	-0.01	9.61E-01	1.00E+00	-0.06	9.15E-01	9.89E-01
**MBLAC1**	-0.08	8.30E-01	1.00E+00	-0.10	9.15E-01	9.89E-01
**ZFP2**	0.46	3.00E-01	1.00E+00	-0.06	9.15E-01	9.89E-01
**AL357079.1**	0.47	6.73E-01	1.00E+00	-0.11	9.15E-01	9.89E-01
**GTF3C4**	-0.02	9.22E-01	1.00E+00	-0.03	9.16E-01	9.89E-01
**5-Mar**	0.20	3.82E-01	1.00E+00	-0.02	9.16E-01	9.89E-01
**LTN1**	0.04	9.01E-01	1.00E+00	-0.02	9.16E-01	9.89E-01
**AC084876.2**	-1.43	3.09E-01	1.00E+00	-0.13	9.16E-01	9.89E-01
**IRX1**	0.02	9.63E-01	1.00E+00	-0.07	9.16E-01	9.89E-01
**RAN**	-0.11	6.36E-01	1.00E+00	-0.02	9.16E-01	9.89E-01
**CBLN2**	-0.85	7.66E-01	1.00E+00	-0.11	9.16E-01	9.89E-01
**COX20P1**	5.78	2.49E-02	9.28E-01	0.33	9.16E-01	9.89E-01
**SLC39A8**	-0.14	7.93E-01	1.00E+00	-0.05	9.16E-01	9.89E-01
**AHI1**	0.37	1.40E-01	1.00E+00	-0.02	9.16E-01	9.89E-01
**PCK2**	-0.20	4.20E-01	1.00E+00	0.05	9.16E-01	9.89E-01
**Z83847.1**	-3.59	2.75E-01	1.00E+00	-0.11	9.16E-01	9.89E-01

**POLN**	0.28	7.16E-01	1.00E+00	0.10	9.16E-01	9.89E-01
**SNAPC5**	0.06	7.68E-01	1.00E+00	-0.02	9.16E-01	9.89E-01
**MUC20**	-1.83	1.38E-01	1.00E+00	0.08	9.16E-01	9.89E-01
**G30851**	1.15	8.48E-02	1.00E+00	0.29	9.16E-01	9.89E-01
**PLCB1**	-0.01	9.82E-01	1.00E+00	-0.06	9.16E-01	9.89E-01
**VPS33B-DT**	-0.54	2.86E-01	1.00E+00	-0.06	9.16E-01	9.89E-01
**MARVELD3**	0.21	5.06E-01	1.00E+00	-0.07	9.16E-01	9.89E-01
**PYCR2**	0.13	6.27E-01	1.00E+00	-0.03	9.16E-01	9.89E-01
**AC009560.1**	-0.20	9.31E-01	1.00E+00	-0.07	9.16E-01	9.89E-01
**TRNP1**	-0.06	7.88E-01	1.00E+00	-0.06	9.16E-01	9.89E-01
**TINAGL1**	0.07	8.86E-01	1.00E+00	-0.09	9.16E-01	9.89E-01
**SDC4**	0.01	9.80E-01	1.00E+00	0.04	9.16E-01	9.89E-01
**ASB6**	-0.27	2.83E-01	1.00E+00	0.03	9.16E-01	9.89E-01
**MRPL27**	-0.08	7.40E-01	1.00E+00	0.03	9.16E-01	9.89E-01
**TAPBP**	-0.34	3.08E-01	1.00E+00	0.03	9.17E-01	9.89E-01
**GTPBP3**	-0.16	5.57E-01	1.00E+00	0.03	9.17E-01	9.89E-01
**DPP7**	-0.10	6.16E-01	1.00E+00	-0.02	9.17E-01	9.89E-01
**AP002992.1**	-3.29	1.99E-01	1.00E+00	0.18	9.17E-01	9.89E-01
**G10909**	0.67	4.75E-01	1.00E+00	-0.08	9.17E-01	9.89E-01
**KLK7**	-0.02	9.70E-01	1.00E+00	0.08	9.17E-01	9.89E-01
**AL450998.2**	-1.24	4.33E-01	1.00E+00	0.10	9.17E-01	9.89E-01
**RERGL**	-0.11	8.72E-01	1.00E+00	-0.07	9.17E-01	9.89E-01
**PMPCA**	-0.05	7.90E-01	1.00E+00	-0.02	9.17E-01	9.89E-01
**KLHL5**	0.07	8.86E-01	1.00E+00	-0.06	9.17E-01	9.89E-01
**SCAF11**	-0.16	4.18E-01	1.00E+00	-0.02	9.17E-01	9.89E-01
**SERPINB5**	-0.13	7.98E-01	1.00E+00	0.07	9.17E-01	9.89E-01
**SPDYE12P**	-0.14	8.15E-01	1.00E+00	-0.06	9.17E-01	9.89E-01
**AC024568.1**	1.81	2.65E-01	1.00E+00	0.10	9.17E-01	9.89E-01
**AL139274.2**	0.22	7.29E-01	1.00E+00	0.07	9.17E-01	9.89E-01
**ZC3H7A**	0.18	4.25E-01	1.00E+00	-0.03	9.17E-01	9.89E-01
**USP43**	-0.52	1.85E-01	1.00E+00	0.08	9.17E-01	9.89E-01
**POLR3C**	0.03	8.71E-01	1.00E+00	-0.02	9.17E-01	9.89E-01

**CAND2**	0.01	9.88E-01	1.00E+00	0.10	9.17E-01	9.89E-01
**RPS27AP1**	NA	NA	NA	0.32	9.17E-01	9.89E-01
**CAPNS2**	-0.07	8.41E-01	1.00E+00	-0.06	9.17E-01	9.89E-01
**NAIF1**	0.05	8.30E-01	1.00E+00	0.05	9.17E-01	9.89E-01
**AC243960.2**	-2.58	1.33E-01	1.00E+00	-0.04	9.17E-01	9.89E-01
**RAD51AP1**	-0.21	5.37E-01	1.00E+00	0.04	9.17E-01	9.89E-01
**TEX21P**	0.66	5.61E-01	1.00E+00	0.07	9.17E-01	9.89E-01
**NDUFS1**	-0.06	7.38E-01	1.00E+00	-0.02	9.17E-01	9.89E-01
**SLC4A1AP**	-0.17	3.50E-01	1.00E+00	0.02	9.17E-01	9.89E-01
**SLC19A1**	0.13	7.84E-01	1.00E+00	0.06	9.17E-01	9.89E-01
**XLOC_014362**	-0.83	7.32E-01	1.00E+00	0.12	9.17E-01	9.89E-01
**AC124068.2**	0.72	2.37E-01	1.00E+00	-0.08	9.17E-01	9.89E-01
**AL078581.3**	-1.06	5.19E-01	1.00E+00	0.09	9.17E-01	9.89E-01
**FOXL2NB**	0.42	7.26E-01	1.00E+00	-0.15	9.18E-01	9.89E-01
**KCNK10**	0.25	7.77E-01	1.00E+00	-0.14	9.18E-01	9.89E-01
**CCDC137**	-0.16	5.73E-01	1.00E+00	0.04	9.18E-01	9.89E-01
**PFN1**	-0.10	5.93E-01	1.00E+00	-0.03	9.18E-01	9.89E-01
**GOLGA6A**	0.97	7.78E-01	1.00E+00	-0.20	9.18E-01	9.89E-01
**TOP2A**	0.13	6.13E-01	1.00E+00	-0.06	9.18E-01	9.89E-01
**LINC00941**	-0.51	5.79E-01	1.00E+00	-0.10	9.18E-01	9.89E-01
**ARID4B**	0.14	5.92E-01	1.00E+00	0.02	9.18E-01	9.89E-01
**SNX10**	0.98	2.82E-01	1.00E+00	0.07	9.18E-01	9.89E-01
**CYS1**	0.14	7.96E-01	1.00E+00	-0.08	9.18E-01	9.89E-01
**GPR20**	0.53	4.83E-01	1.00E+00	-0.11	9.18E-01	9.89E-01
**PTH1R**	0.38	4.13E-01	1.00E+00	-0.08	9.18E-01	9.89E-01
**FZD4**	1.90	2.49E-02	9.28E-01	0.07	9.18E-01	9.89E-01
**PLEKHS1**	0.04	9.72E-01	1.00E+00	-0.31	9.18E-01	9.89E-01
**PHF14**	0.03	8.87E-01	1.00E+00	0.04	9.18E-01	9.89E-01
**RBM15**	-0.05	8.51E-01	1.00E+00	-0.04	9.18E-01	9.89E-01
**HNRNPA3P3**	0.13	9.12E-01	1.00E+00	0.06	9.18E-01	9.89E-01
**SLC8A2**	-2.13	7.95E-03	5.48E-01	0.11	9.18E-01	9.89E-01
**HIST1H4H**	0.21	5.94E-01	1.00E+00	-0.04	9.18E-01	9.89E-01

**RPL21P44**	-1.24	3.18E-01	1.00E+00	-0.08	9.18E-01	9.89E-01
**NSA2**	0.00	9.98E-01	1.00E+00	0.03	9.18E-01	9.89E-01
**GRXCR2**	-0.19	8.32E-01	1.00E+00	-0.10	9.18E-01	9.89E-01
**SLC9B2**	-0.23	4.31E-01	1.00E+00	-0.04	9.18E-01	9.89E-01
**TRIM73**	-2.08	7.41E-02	1.00E+00	0.07	9.19E-01	9.89E-01
**UBE2D3P2**	-1.68	6.27E-01	1.00E+00	0.07	9.19E-01	9.89E-01
**DTNBP1**	0.12	6.12E-01	1.00E+00	0.04	9.19E-01	9.89E-01
**GUCA1B**	-0.46	4.73E-01	1.00E+00	-0.11	9.19E-01	9.89E-01
**NAA16**	0.03	9.13E-01	1.00E+00	0.03	9.19E-01	9.89E-01
**AQP10**	-0.52	6.14E-01	1.00E+00	-0.13	9.19E-01	9.89E-01
**XLOC_009836**	1.96	5.66E-01	1.00E+00	-0.15	9.19E-01	9.89E-01
**XLOC_008479**	0.69	7.99E-01	1.00E+00	-0.15	9.19E-01	9.89E-01
**C1QTNF9B**	3.11	2.17E-01	1.00E+00	0.11	9.19E-01	9.89E-01
**MLC1**	-0.99	7.11E-03	5.13E-01	0.08	9.19E-01	9.89E-01
**JARID2**	-0.60	8.00E-02	1.00E+00	0.04	9.19E-01	9.89E-01
**KIR3DL1**	-0.12	9.73E-01	1.00E+00	-0.20	9.19E-01	9.89E-01
**COG5**	0.02	9.17E-01	1.00E+00	-0.02	9.19E-01	9.89E-01
**RPL10P9**	-0.20	5.07E-01	1.00E+00	-0.05	9.19E-01	9.89E-01
**ACTN2**	-1.07	3.12E-01	1.00E+00	-0.28	9.19E-01	9.89E-01
**DHRS4**	-0.16	4.91E-01	1.00E+00	-0.02	9.19E-01	9.89E-01
**AC008026.1**	-0.84	6.00E-01	1.00E+00	0.07	9.19E-01	9.89E-01
**DNAAF5**	-0.13	5.65E-01	1.00E+00	0.04	9.19E-01	9.89E-01
**AC116614.1**	5.30	1.48E-02	7.62E-01	0.13	9.19E-01	9.89E-01
**EIF2S2**	0.02	8.91E-01	1.00E+00	0.03	9.19E-01	9.89E-01
**STEAP1**	0.64	2.24E-01	1.00E+00	-0.06	9.19E-01	9.89E-01
**POLR2J**	-0.25	3.52E-01	1.00E+00	-0.02	9.19E-01	9.89E-01
**AC244021.1**	0.42	5.64E-01	1.00E+00	-0.11	9.19E-01	9.89E-01
**AL133216.2**	-0.38	3.62E-01	1.00E+00	0.07	9.19E-01	9.89E-01
**XLOC_004420**	-0.37	3.02E-01	1.00E+00	-0.13	9.19E-01	9.89E-01
**BRPF1**	-0.42	9.26E-02	1.00E+00	-0.07	9.19E-01	9.89E-01
**INTS12**	-0.26	2.19E-01	1.00E+00	0.04	9.19E-01	9.89E-01
**AL512324.3**	1.77	2.20E-01	1.00E+00	0.10	9.20E-01	9.89E-01

**SLC30A6**	0.33	2.05E-01	1.00E+00	-0.02	9.20E-01	9.89E-01
**PRC1**	-1.99	4.21E-01	1.00E+00	0.08	9.20E-01	9.89E-01
**ZNF157**	1.59	1.27E-01	1.00E+00	0.13	9.20E-01	9.89E-01
**AC091825.1**	-0.13	8.03E-01	1.00E+00	-0.06	9.20E-01	9.89E-01
**AL162171.1**	-0.01	9.82E-01	1.00E+00	-0.04	9.20E-01	9.89E-01
**EHMT2**	-0.10	6.53E-01	1.00E+00	0.05	9.20E-01	9.89E-01
**TSPEAR-AS2**	-1.38	5.46E-01	1.00E+00	0.20	9.20E-01	9.89E-01
**AC008083.2**	-2.58	2.57E-01	1.00E+00	0.09	9.20E-01	9.89E-01
**SNN**	0.12	7.34E-01	1.00E+00	0.05	9.20E-01	9.89E-01
**CPB1**	-0.34	7.21E-01	1.00E+00	-0.08	9.20E-01	9.89E-01
**SNHG22**	0.34	9.15E-01	1.00E+00	0.08	9.20E-01	9.89E-01
**AFP**	3.24	2.29E-01	1.00E+00	-0.20	9.20E-01	9.89E-01
**AIRE**	0.05	9.31E-01	1.00E+00	0.10	9.20E-01	9.89E-01
**LSMEM2**	0.35	7.76E-01	1.00E+00	0.13	9.21E-01	9.89E-01
**BUD23**	-0.12	5.09E-01	1.00E+00	-0.02	9.21E-01	9.89E-01
**AC093535.2**	0.04	9.40E-01	1.00E+00	-0.05	9.21E-01	9.89E-01
**HNRNPRP1**	NA	NA	NA	0.07	9.21E-01	9.89E-01
**KRT31**	-0.52	8.69E-01	1.00E+00	-0.09	9.21E-01	9.89E-01
**CYB561**	0.08	6.85E-01	1.00E+00	-0.02	9.21E-01	9.89E-01
**SRD5A3-AS1**	0.04	9.74E-01	1.00E+00	0.08	9.21E-01	9.89E-01
**UHMK1**	0.33	3.19E-01	1.00E+00	0.03	9.21E-01	9.89E-01
**CLCN7**	-0.17	3.74E-01	1.00E+00	0.31	9.21E-01	9.89E-01
**FICD**	-0.13	7.70E-01	1.00E+00	0.03	9.21E-01	9.89E-01
**AC079174.2**	-0.36	6.95E-01	1.00E+00	-0.08	9.21E-01	9.89E-01
**G40298**	1.37	2.91E-01	1.00E+00	0.13	9.21E-01	9.89E-01
**Z99943.2**	0.97	7.78E-01	1.00E+00	-0.07	9.21E-01	9.89E-01
**TMEM30B**	0.03	8.93E-01	1.00E+00	-0.04	9.21E-01	9.89E-01
**DR1**	0.32	2.46E-01	1.00E+00	-0.02	9.21E-01	9.89E-01
**TRIM44**	0.10	7.08E-01	1.00E+00	0.04	9.21E-01	9.89E-01
**LRRC9**	-3.82	2.20E-01	1.00E+00	-0.32	9.21E-01	9.89E-01
**WBP1LP2**	-1.58	2.00E-01	1.00E+00	-0.08	9.21E-01	9.89E-01
**TGFBRAP1**	-0.25	2.28E-01	1.00E+00	-0.03	9.21E-01	9.89E-01

**MAP2K3**	-0.24	4.83E-01	1.00E+00	-0.03	9.21E-01	9.89E-01
**HNRNPH1**	-0.20	2.19E-01	1.00E+00	0.02	9.21E-01	9.89E-01
**ADAMTS17**	-0.28	3.73E-01	1.00E+00	-0.07	9.21E-01	9.89E-01
**SLCO5A1**	-0.21	4.99E-01	1.00E+00	-0.10	9.21E-01	9.89E-01
**PGR-AS1**	1.02	7.67E-01	1.00E+00	0.12	9.21E-01	9.89E-01
**AP001432.1**	-0.66	7.17E-01	1.00E+00	0.13	9.22E-01	9.89E-01
**AC093323.1**	0.13	6.32E-01	1.00E+00	-0.05	9.22E-01	9.89E-01
**RNF112**	0.10	8.54E-01	1.00E+00	0.12	9.22E-01	9.89E-01
**KPNA1**	0.09	6.65E-01	1.00E+00	0.02	9.22E-01	9.89E-01
**VDAC1P2**	2.46	4.70E-01	1.00E+00	-0.08	9.22E-01	9.89E-01
**CENPM**	-0.32	4.81E-01	1.00E+00	0.05	9.22E-01	9.89E-01
**KRT86**	-1.42	6.57E-01	1.00E+00	0.11	9.22E-01	9.89E-01
**CLNK**	-1.27	2.16E-01	1.00E+00	-0.09	9.22E-01	9.89E-01
**ATF3**	-2.11	6.26E-02	1.00E+00	0.06	9.22E-01	9.89E-01
**USP54**	-0.01	9.81E-01	1.00E+00	0.04	9.22E-01	9.89E-01
**XLOC_003400**	-0.69	3.16E-01	1.00E+00	0.11	9.22E-01	9.89E-01
**FBXW4P1**	-0.51	4.80E-01	1.00E+00	-0.09	9.22E-01	9.89E-01
**RASL12**	-0.26	5.64E-01	1.00E+00	-0.07	9.22E-01	9.89E-01
**NR2F1**	-0.28	5.54E-01	1.00E+00	0.08	9.22E-01	9.89E-01
**SCYGR6**	-8.50	1.46E-02	7.60E-01	0.31	9.22E-01	9.89E-01
**AL022326.2**	0.40	6.56E-01	1.00E+00	-0.07	9.22E-01	9.89E-01
**LINC02035**	0.30	4.01E-01	1.00E+00	0.05	9.23E-01	9.89E-01
**C11orf49**	-0.16	6.35E-01	1.00E+00	-0.03	9.23E-01	9.89E-01
**BRD7**	-0.11	6.65E-01	1.00E+00	-0.03	9.23E-01	9.89E-01
**CLCN4**	0.26	6.09E-01	1.00E+00	0.06	9.23E-01	9.89E-01
**EFCAB12**	1.66	2.29E-01	1.00E+00	-0.08	9.23E-01	9.89E-01
**HOXB1**	1.21	2.12E-01	1.00E+00	0.09	9.23E-01	9.89E-01
**DEFB4B**	0.00	1.00E+00	NA	-0.31	9.23E-01	9.89E-01
**OIP5**	0.08	8.43E-01	1.00E+00	0.05	9.23E-01	9.89E-01
**AC084116.1**	-1.24	7.15E-01	1.00E+00	0.31	9.23E-01	9.89E-01
**CHST7**	0.15	6.83E-01	1.00E+00	0.06	9.23E-01	9.89E-01
**ZNF410**	0.41	7.06E-01	1.00E+00	-0.06	9.23E-01	9.89E-01

**TNFRSF14-AS1**	1.67	4.01E-01	1.00E+00	0.08	9.23E-01	9.89E-01
**C1QL1**	-1.17	6.48E-02	1.00E+00	0.10	9.23E-01	9.89E-01
**G28347**	1.28	2.59E-01	1.00E+00	-0.11	9.23E-01	9.89E-01
**AC129507.1**	-0.48	5.68E-01	1.00E+00	-0.05	9.23E-01	9.89E-01
**NBR1**	-0.04	7.96E-01	1.00E+00	-0.02	9.23E-01	9.89E-01
**AC130689.1**	-0.82	3.46E-01	1.00E+00	0.06	9.23E-01	9.89E-01
**PUM3**	-0.18	5.52E-01	1.00E+00	-0.02	9.23E-01	9.89E-01
**BRAT1**	-0.11	5.66E-01	1.00E+00	-0.04	9.23E-01	9.89E-01
**WRN**	0.05	8.57E-01	1.00E+00	-0.04	9.23E-01	9.89E-01
**RHD**	0.02	9.73E-01	1.00E+00	0.09	9.23E-01	9.89E-01
**TNPO3**	0.14	4.00E-01	1.00E+00	0.01	9.23E-01	9.89E-01
**AP003080.1**	0.11	9.15E-01	1.00E+00	-0.16	9.23E-01	9.89E-01
**TSPYL5**	0.05	8.55E-01	1.00E+00	-0.05	9.23E-01	9.89E-01
**SYT7**	-0.41	5.00E-01	1.00E+00	0.06	9.23E-01	9.89E-01
**AL645929.2**	1.10	4.39E-01	1.00E+00	0.11	9.23E-01	9.89E-01
**AL355312.5**	0.79	1.62E-01	1.00E+00	-0.07	9.23E-01	9.89E-01
**SLC9A7**	-0.15	7.49E-01	1.00E+00	-0.05	9.23E-01	9.89E-01
**CLEC6A**	-2.78	4.14E-01	1.00E+00	0.31	9.23E-01	9.89E-01
**HOXD-AS2**	0.81	7.99E-02	1.00E+00	-0.10	9.23E-01	9.89E-01
**XLOC_012406**	1.27	2.63E-01	1.00E+00	-0.07	9.23E-01	9.89E-01
**HSPH1**	-0.10	7.67E-01	1.00E+00	0.03	9.24E-01	9.89E-01
**INTS3**	-0.10	6.68E-01	1.00E+00	-0.04	9.24E-01	9.89E-01
**DCTN3**	-0.12	5.22E-01	1.00E+00	0.02	9.24E-01	9.89E-01
**CEP97**	-0.13	6.49E-01	1.00E+00	-0.03	9.24E-01	9.89E-01
**BARD1**	0.05	8.76E-01	1.00E+00	-0.05	9.24E-01	9.89E-01
**SHE**	0.15	7.19E-01	1.00E+00	0.07	9.24E-01	9.89E-01
**RAB27A**	-0.13	5.26E-01	1.00E+00	0.02	9.24E-01	9.89E-01
**CPB2**	1.27	7.13E-01	1.00E+00	-0.15	9.24E-01	9.89E-01
**FGFR4**	-0.74	2.10E-01	1.00E+00	-0.09	9.24E-01	9.89E-01
**TCN1**	-1.95	1.14E-01	1.00E+00	0.30	9.24E-01	9.89E-01
**ACSF3**	-0.22	3.53E-01	1.00E+00	0.02	9.24E-01	9.89E-01
**AL117341.1**	-0.56	8.73E-01	1.00E+00	0.12	9.24E-01	9.89E-01

**HLA-DRB5**	-0.52	4.11E-01	1.00E+00	0.05	9.24E-01	9.89E-01
**CCNB2**	0.19	5.57E-01	1.00E+00	0.05	9.24E-01	9.89E-01
**ZNF503-AS1**	-1.13	5.24E-02	1.00E+00	0.06	9.24E-01	9.90E-01
**THEM5**	0.13	7.93E-01	1.00E+00	-0.06	9.24E-01	9.90E-01
**PKP4-AS1**	0.31	6.54E-01	1.00E+00	-0.05	9.24E-01	9.90E-01
**XPC**	-0.04	8.45E-01	1.00E+00	0.02	9.24E-01	9.90E-01
**KBTBD12**	0.42	5.44E-01	1.00E+00	-0.11	9.24E-01	9.90E-01
**AC005332.2**	0.34	8.05E-01	1.00E+00	-0.15	9.25E-01	9.90E-01
**AC139792.1**	-0.05	9.77E-01	1.00E+00	-0.12	9.25E-01	9.90E-01
**SDCBPP3**	NA	NA	NA	-0.09	9.25E-01	9.90E-01
**AC009126.1**	-0.54	1.46E-01	1.00E+00	0.05	9.25E-01	9.90E-01
**ZFYVE1**	0.12	5.33E-01	1.00E+00	0.03	9.25E-01	9.90E-01
**TOGARAM1**	0.27	1.75E-01	1.00E+00	0.02	9.25E-01	9.90E-01
**DVL3**	-0.25	1.86E-01	1.00E+00	-0.05	9.25E-01	9.90E-01
**AC007262.2**	-0.57	5.48E-01	1.00E+00	-0.07	9.25E-01	9.90E-01
**DAW1**	-2.33	2.04E-01	1.00E+00	-0.12	9.25E-01	9.90E-01
**AC068631.3**	-0.71	8.37E-01	1.00E+00	-0.07	9.25E-01	9.90E-01
**PLEKHB1**	-0.15	8.37E-01	1.00E+00	-0.05	9.25E-01	9.90E-01
**NFX1**	-0.05	7.59E-01	1.00E+00	0.02	9.25E-01	9.90E-01
**PIK3R3**	0.41	4.35E-01	1.00E+00	0.04	9.25E-01	9.90E-01
**HBA2**	1.33	3.06E-02	1.00E+00	-0.29	9.25E-01	9.90E-01
**PTPRK**	-0.27	1.61E-01	1.00E+00	-0.02	9.25E-01	9.90E-01
**SAYSD1**	0.00	9.99E-01	1.00E+00	0.02	9.25E-01	9.90E-01
**AC073367.1**	0.01	9.95E-01	1.00E+00	-0.10	9.25E-01	9.90E-01
**RPL7P32**	0.41	6.17E-01	1.00E+00	0.08	9.25E-01	9.90E-01
**COX4I1**	-0.07	7.45E-01	1.00E+00	0.02	9.25E-01	9.90E-01
**AC104794.2**	-0.18	6.11E-01	1.00E+00	0.03	9.25E-01	9.90E-01
**TAB3**	-0.02	9.35E-01	1.00E+00	-0.04	9.25E-01	9.90E-01
**UBE2E1-AS1**	-1.35	5.76E-01	1.00E+00	-0.15	9.25E-01	9.90E-01
**ATXN7**	-0.17	4.07E-01	1.00E+00	-0.03	9.25E-01	9.90E-01
**DNM2**	-0.10	6.72E-01	1.00E+00	0.05	9.25E-01	9.90E-01
**RPL7L1P8**	2.26	5.07E-01	1.00E+00	-0.12	9.25E-01	9.90E-01

**ARL10**	0.05	8.81E-01	1.00E+00	-0.06	9.25E-01	9.90E-01
**EDN2**	-1.94	2.52E-02	9.28E-01	-0.07	9.25E-01	9.90E-01
**AL133245.1**	1.95	3.17E-01	1.00E+00	-0.09	9.25E-01	9.90E-01
**CTAG2**	0.00	1.00E+00	NA	-0.30	9.25E-01	9.90E-01
**LOR**	0.24	6.92E-01	1.00E+00	0.29	9.26E-01	9.90E-01
**SH2B2**	-0.19	6.64E-01	1.00E+00	-0.29	9.26E-01	9.90E-01
**AC079907.1**	-0.06	9.86E-01	1.00E+00	0.12	9.26E-01	9.90E-01
**EEPD1**	0.33	2.95E-01	1.00E+00	0.04	9.26E-01	9.90E-01
**AL049836.1**	0.04	9.78E-01	1.00E+00	0.13	9.26E-01	9.90E-01
**AL157904.1**	-2.71	2.81E-01	1.00E+00	0.12	9.26E-01	9.90E-01
**HK1**	-0.15	4.76E-01	1.00E+00	-0.03	9.26E-01	9.90E-01
**FLT1**	0.05	9.23E-01	1.00E+00	-0.05	9.26E-01	9.90E-01
**PAK1IP1**	-0.13	5.48E-01	1.00E+00	-0.03	9.26E-01	9.90E-01
**CRB3**	-0.08	8.46E-01	1.00E+00	0.05	9.26E-01	9.90E-01
**AC002310.6**	-0.15	6.17E-01	1.00E+00	0.29	9.26E-01	9.90E-01
**CHD7**	-0.18	5.65E-01	1.00E+00	-0.03	9.26E-01	9.90E-01
**G24514**	-0.99	3.34E-01	1.00E+00	-0.06	9.26E-01	9.90E-01
**ATG4B**	-0.10	6.09E-01	1.00E+00	0.01	9.26E-01	9.90E-01
**DRAIC**	-0.34	7.46E-01	1.00E+00	-0.08	9.26E-01	9.90E-01
**AC005261.1**	0.43	3.51E-01	1.00E+00	0.03	9.26E-01	9.90E-01
**GIGYF2**	-0.02	8.90E-01	1.00E+00	-0.02	9.26E-01	9.90E-01
**WHRN**	-0.60	2.25E-02	9.03E-01	0.05	9.27E-01	9.90E-01
**FAM229A**	-1.23	9.01E-02	1.00E+00	-0.06	9.27E-01	9.90E-01
**DYNC1LI1**	0.08	7.82E-01	1.00E+00	-0.03	9.27E-01	9.90E-01
**FGF10**	0.96	2.05E-01	1.00E+00	0.18	9.27E-01	9.90E-01
**AF131216.3**	-1.23	5.36E-01	1.00E+00	-0.09	9.27E-01	9.90E-01
**AL355574.1**	-0.69	2.93E-01	1.00E+00	-0.07	9.27E-01	9.90E-01
**AC011979.1**	-0.31	8.63E-01	1.00E+00	-0.07	9.27E-01	9.90E-01
**XLOC_001751**	-0.24	5.93E-01	1.00E+00	-0.07	9.27E-01	9.90E-01
**PGA5**	2.74	8.81E-02	1.00E+00	-0.13	9.27E-01	9.90E-01
**AC002070.1**	-1.79	2.91E-01	1.00E+00	-0.11	9.27E-01	9.90E-01
**TIAM2**	-0.05	8.92E-01	1.00E+00	-0.04	9.27E-01	9.90E-01

**ZNF746**	-0.37	1.14E-01	1.00E+00	0.06	9.27E-01	9.90E-01
**SIRT7**	-0.09	7.69E-01	1.00E+00	-0.05	9.27E-01	9.90E-01
**G3383**	0.13	7.00E-01	1.00E+00	-0.05	9.27E-01	9.90E-01
**AL356235.1**	0.66	3.79E-01	1.00E+00	0.06	9.27E-01	9.90E-01
**PSORS1C2**	0.22	7.46E-01	1.00E+00	0.07	9.27E-01	9.90E-01
**AC068870.2**	-0.21	6.86E-01	1.00E+00	-0.05	9.27E-01	9.90E-01
**DTWD1**	0.10	7.10E-01	1.00E+00	0.03	9.27E-01	9.90E-01
**AC022400.1**	1.22	3.70E-01	1.00E+00	0.08	9.27E-01	9.90E-01
**EBF3**	0.11	7.93E-01	1.00E+00	0.07	9.27E-01	9.90E-01
**UBE4B**	0.09	7.70E-01	1.00E+00	0.02	9.27E-01	9.90E-01
**ZNF574**	-0.17	4.94E-01	1.00E+00	0.02	9.28E-01	9.90E-01
**AC004241.3**	1.26	6.56E-01	1.00E+00	-0.05	9.28E-01	9.90E-01
**CPAMD8**	1.26	1.05E-01	1.00E+00	0.07	9.28E-01	9.90E-01
**XLOC_000206**	-1.18	7.82E-02	1.00E+00	0.07	9.28E-01	9.90E-01
**SLC27A1**	0.18	5.81E-01	1.00E+00	0.05	9.28E-01	9.90E-01
**G3793**	0.38	7.04E-01	1.00E+00	-0.11	9.28E-01	9.90E-01
**G23133**	0.70	3.04E-01	1.00E+00	-0.06	9.28E-01	9.90E-01
**MS4A14**	0.41	7.31E-01	1.00E+00	-0.11	9.28E-01	9.90E-01
**C11orf65**	-0.08	9.37E-01	1.00E+00	0.06	9.28E-01	9.90E-01
**SLC8A3**	0.13	8.23E-01	1.00E+00	-0.11	9.28E-01	9.90E-01
**GNB1**	0.05	7.19E-01	1.00E+00	0.04	9.28E-01	9.90E-01
**WNT3A**	-1.08	2.59E-03	2.61E-01	-0.06	9.28E-01	9.90E-01
**VANGL2**	-0.25	3.40E-01	1.00E+00	-0.06	9.28E-01	9.90E-01
**CFAP69**	0.35	5.28E-01	1.00E+00	0.06	9.28E-01	9.90E-01
**C19orf44**	-0.17	5.73E-01	1.00E+00	0.07	9.28E-01	9.90E-01
**CALCOCO1**	-0.23	4.15E-01	1.00E+00	-0.02	9.28E-01	9.90E-01
**DUOX2**	0.48	2.11E-01	1.00E+00	-0.28	9.28E-01	9.90E-01
**AC083805.1**	0.11	9.75E-01	1.00E+00	0.11	9.28E-01	9.90E-01
**FAM110C**	-0.11	7.66E-01	1.00E+00	0.06	9.28E-01	9.90E-01
**AC105020.5**	-1.46	1.82E-01	1.00E+00	0.12	9.28E-01	9.90E-01
**MAPK8IP2**	-1.07	4.79E-02	1.00E+00	-0.06	9.28E-01	9.90E-01
**WSB2**	0.27	7.55E-02	1.00E+00	-0.04	9.28E-01	9.90E-01

**MCTP1**	0.04	9.25E-01	1.00E+00	-0.05	9.28E-01	9.90E-01
**CLDN16**	0.74	6.67E-02	1.00E+00	0.06	9.28E-01	9.90E-01
**AC126283.1**	2.77	3.18E-01	1.00E+00	-0.10	9.29E-01	9.90E-01
**CLDN4**	0.06	8.93E-01	1.00E+00	0.04	9.29E-01	9.90E-01
**AC005856.1**	0.16	8.93E-01	1.00E+00	0.07	9.29E-01	9.90E-01
**TRPV4**	-0.17	6.70E-01	1.00E+00	-0.04	9.29E-01	9.90E-01
**CAMK2N1**	-0.29	6.31E-01	1.00E+00	-0.04	9.29E-01	9.90E-01
**XLOC_005798**	2.80	1.49E-01	1.00E+00	-0.28	9.29E-01	9.90E-01
**FBXW9**	-0.34	3.65E-01	1.00E+00	-0.02	9.29E-01	9.90E-01
**PDZRN4**	0.00	9.97E-01	1.00E+00	-0.09	9.29E-01	9.90E-01
**AC008514.1**	0.41	7.26E-01	1.00E+00	-0.07	9.29E-01	9.90E-01
**CECR2**	-1.01	1.21E-01	1.00E+00	0.08	9.29E-01	9.90E-01
**TMEM214**	-0.07	6.41E-01	1.00E+00	-0.04	9.29E-01	9.90E-01
**RXRA**	0.00	9.97E-01	1.00E+00	0.04	9.29E-01	9.90E-01
**PTCH1**	0.01	9.84E-01	1.00E+00	-0.05	9.29E-01	9.90E-01
**AC025048.6**	0.27	8.92E-01	1.00E+00	-0.06	9.29E-01	9.90E-01
**DUBR**	0.08	8.25E-01	1.00E+00	-0.03	9.29E-01	9.90E-01
**SLC22A5**	0.40	2.04E-01	1.00E+00	-0.03	9.29E-01	9.90E-01
**AFAP1L2**	-0.51	8.53E-02	1.00E+00	-0.03	9.29E-01	9.90E-01
**IFNG**	-2.43	4.80E-01	1.00E+00	-0.28	9.29E-01	9.90E-01
**CILP2**	-0.43	8.53E-01	1.00E+00	-0.28	9.29E-01	9.90E-01
**AC243960.1**	-3.28	1.85E-02	8.55E-01	0.06	9.29E-01	9.90E-01
**LCE2B**	0.87	2.33E-01	1.00E+00	-0.07	9.29E-01	9.90E-01
**THSD1**	-0.24	3.77E-01	1.00E+00	-0.05	9.29E-01	9.90E-01
**SNHG4**	-1.55	6.93E-03	5.08E-01	-0.06	9.29E-01	9.90E-01
**AL049557.2**	-0.90	7.83E-01	1.00E+00	-0.10	9.30E-01	9.90E-01
**RPUSD3**	0.06	7.61E-01	1.00E+00	0.02	9.30E-01	9.90E-01
**KCTD10**	0.15	4.74E-01	1.00E+00	-0.03	9.30E-01	9.90E-01
**AC107871.1**	-0.37	7.45E-01	1.00E+00	-0.10	9.30E-01	9.90E-01
**SLC9A3R1**	-0.25	2.59E-01	1.00E+00	-0.02	9.30E-01	9.90E-01
**FAM21B**	-0.11	9.51E-01	1.00E+00	-0.21	9.30E-01	9.90E-01
**XLOC_008109**	3.81	6.61E-02	1.00E+00	0.09	9.30E-01	9.90E-01

**ERVE-1**	-0.44	1.61E-01	1.00E+00	-0.05	9.30E-01	9.90E-01
**EEF1A1P22**	-0.53	8.05E-01	1.00E+00	-0.07	9.30E-01	9.90E-01
**TMEM69**	-0.04	8.78E-01	1.00E+00	0.02	9.30E-01	9.90E-01
**ADO**	-0.13	4.55E-01	1.00E+00	0.03	9.30E-01	9.90E-01
**G5901**	-1.33	4.99E-01	1.00E+00	-0.12	9.30E-01	9.90E-01
**SPAG7**	-0.19	2.36E-01	1.00E+00	0.02	9.30E-01	9.90E-01
**APTR**	0.28	3.03E-01	1.00E+00	-0.03	9.30E-01	9.90E-01
**RNF39**	-0.20	6.80E-01	1.00E+00	-0.05	9.30E-01	9.90E-01
**FBXO28**	-0.04	9.05E-01	1.00E+00	0.02	9.30E-01	9.90E-01
**TMEM123**	0.14	5.72E-01	1.00E+00	-0.02	9.30E-01	9.90E-01
**AC025682.1**	0.69	5.23E-01	1.00E+00	0.05	9.30E-01	9.90E-01
**TNFSF15**	0.12	8.87E-01	1.00E+00	-0.07	9.30E-01	9.90E-01
**LINC01266**	-0.96	4.09E-01	1.00E+00	-0.09	9.30E-01	9.90E-01
**AC112206.2**	0.06	9.70E-01	1.00E+00	-0.09	9.30E-01	9.90E-01
**AL049775.2**	-0.93	6.22E-01	1.00E+00	-0.10	9.30E-01	9.90E-01
**ZNF658**	0.25	4.66E-01	1.00E+00	-0.03	9.30E-01	9.90E-01
**CEP120**	0.01	9.83E-01	1.00E+00	-0.05	9.30E-01	9.90E-01
**ZNF112**	0.67	5.54E-02	1.00E+00	0.03	9.30E-01	9.90E-01
**CAPN1**	-0.16	5.31E-01	1.00E+00	0.04	9.30E-01	9.90E-01
**TBRG4**	0.13	5.06E-01	1.00E+00	0.02	9.30E-01	9.90E-01
**PAN3-AS1**	0.63	3.05E-01	1.00E+00	0.08	9.30E-01	9.90E-01
**AL357140.2**	-0.95	6.33E-01	1.00E+00	0.05	9.30E-01	9.90E-01
**IQGAP1**	-0.25	1.90E-01	1.00E+00	-0.02	9.30E-01	9.90E-01
**IRS2**	-0.26	5.83E-01	1.00E+00	0.04	9.30E-01	9.90E-01
**RPL7P26**	-0.10	9.76E-01	1.00E+00	0.08	9.30E-01	9.90E-01
**ATG4D**	-0.05	8.40E-01	1.00E+00	-0.05	9.30E-01	9.90E-01
**LINC01127**	0.35	5.59E-01	1.00E+00	-0.27	9.31E-01	9.90E-01
**MECOM**	0.39	4.33E-01	1.00E+00	-0.05	9.31E-01	9.90E-01
**YTHDF3**	0.03	8.55E-01	1.00E+00	0.02	9.31E-01	9.90E-01
**ZNF280B**	-0.75	3.68E-01	1.00E+00	-0.07	9.31E-01	9.90E-01
**AC116562.4**	-1.58	2.23E-01	1.00E+00	0.07	9.31E-01	9.90E-01
**CRYM**	-0.85	1.08E-02	6.42E-01	0.03	9.31E-01	9.90E-01

**AC073284.1**	1.57	4.27E-01	1.00E+00	-0.08	9.31E-01	9.90E-01
**PSMC2**	0.01	9.65E-01	1.00E+00	0.02	9.31E-01	9.90E-01
**RIPK4**	-0.33	1.58E-01	1.00E+00	0.05	9.31E-01	9.90E-01
**XLOC_003615**	-1.53	1.27E-01	1.00E+00	0.07	9.31E-01	9.90E-01
**MED19**	0.08	7.15E-01	1.00E+00	0.03	9.31E-01	9.90E-01
**SIK3**	-0.05	8.31E-01	1.00E+00	0.05	9.31E-01	9.90E-01
**XLOC_010704**	-1.81	3.45E-01	1.00E+00	0.11	9.31E-01	9.90E-01
**MIEF1**	0.12	5.87E-01	1.00E+00	0.02	9.31E-01	9.90E-01
**GTF3C1**	-0.14	5.95E-01	1.00E+00	0.03	9.31E-01	9.90E-01
**AP001922.3**	1.27	5.01E-01	1.00E+00	-0.12	9.31E-01	9.90E-01
**FMOD**	0.54	2.45E-01	1.00E+00	0.04	9.31E-01	9.90E-01
**DCLK3**	-0.31	4.46E-01	1.00E+00	0.09	9.31E-01	9.90E-01
**THAP5**	-0.05	8.51E-01	1.00E+00	-0.04	9.31E-01	9.90E-01
**XLOC_002888**	-1.09	2.63E-01	1.00E+00	-0.09	9.31E-01	9.90E-01
**LINC01873**	-1.52	3.71E-01	1.00E+00	-0.09	9.31E-01	9.90E-01
**TPT1P9**	-1.40	6.84E-01	1.00E+00	0.07	9.31E-01	9.90E-01
**CD151**	0.27	5.28E-01	1.00E+00	-0.03	9.31E-01	9.90E-01
**ZCCHC24**	0.50	3.30E-01	1.00E+00	0.05	9.31E-01	9.90E-01
**TPPP2**	0.55	7.47E-01	1.00E+00	0.16	9.31E-01	9.90E-01
**BABAM2**	-0.31	1.66E-01	1.00E+00	0.01	9.31E-01	9.90E-01
**RNA5SP151**	1.50	6.61E-01	1.00E+00	-0.27	9.32E-01	9.90E-01
**OR2A7**	-0.57	3.13E-01	1.00E+00	0.05	9.32E-01	9.90E-01
**EML4**	-0.16	5.94E-01	1.00E+00	0.02	9.32E-01	9.90E-01
**CDH1**	-0.13	6.63E-01	1.00E+00	0.04	9.32E-01	9.90E-01
**TMEM134**	-0.12	7.22E-01	1.00E+00	-0.04	9.32E-01	9.90E-01
**ZDHHC1**	-0.12	6.25E-01	1.00E+00	-0.07	9.32E-01	9.90E-01
**ZNF526**	0.00	9.86E-01	1.00E+00	0.03	9.32E-01	9.90E-01
**ARHGAP11A**	0.20	5.18E-01	1.00E+00	0.04	9.32E-01	9.90E-01
**HES7**	-0.53	6.51E-01	1.00E+00	-0.10	9.32E-01	9.90E-01
**PLEKHA8P1**	0.41	5.21E-01	1.00E+00	-0.05	9.32E-01	9.90E-01
**PC**	0.98	1.82E-01	1.00E+00	-0.06	9.32E-01	9.90E-01
**RNF31**	-0.29	2.74E-01	1.00E+00	0.06	9.32E-01	9.90E-01

**PKD2L2**	-3.76	2.83E-02	9.75E-01	-0.09	9.32E-01	9.90E-01
**CIZ1**	-0.26	3.59E-01	1.00E+00	0.03	9.32E-01	9.90E-01
**TBC1D17**	-0.21	3.78E-01	1.00E+00	0.03	9.32E-01	9.90E-01
**DSG1**	0.08	8.72E-01	1.00E+00	0.05	9.32E-01	9.90E-01
**FOXK1**	0.24	5.82E-01	1.00E+00	0.03	9.32E-01	9.90E-01
**JAKMIP3**	-0.28	6.64E-01	1.00E+00	-0.08	9.32E-01	9.90E-01
**CDC14B**	0.25	4.72E-01	1.00E+00	0.03	9.32E-01	9.90E-01
**C9orf84**	-0.51	6.47E-01	1.00E+00	-0.08	9.32E-01	9.90E-01
**XLOC_000105**	-0.28	6.47E-01	1.00E+00	0.27	9.32E-01	9.90E-01
**MYLIP**	0.12	6.30E-01	1.00E+00	0.04	9.32E-01	9.90E-01
**AC005532.1**	0.50	7.12E-01	1.00E+00	0.08	9.32E-01	9.90E-01
**C2CD4D**	0.03	9.51E-01	1.00E+00	0.07	9.32E-01	9.90E-01
**PKNOX1**	-0.14	3.62E-01	1.00E+00	-0.02	9.32E-01	9.90E-01
**AF127577.2**	-1.33	6.98E-01	1.00E+00	-0.09	9.32E-01	9.90E-01
**LIMS2**	0.05	9.17E-01	1.00E+00	-0.04	9.32E-01	9.90E-01
**MRAS**	1.09	1.69E-01	1.00E+00	-0.05	9.33E-01	9.90E-01
**FKBP2**	-0.02	9.20E-01	1.00E+00	-0.01	9.33E-01	9.90E-01
**NFRKB**	-0.21	4.08E-01	1.00E+00	-0.03	9.33E-01	9.90E-01
**STOML2**	-0.09	6.23E-01	1.00E+00	0.01	9.33E-01	9.90E-01
**TMPRSS11F**	0.72	4.40E-01	1.00E+00	0.07	9.33E-01	9.90E-01
**AC097382.2**	3.41	2.30E-01	1.00E+00	0.10	9.33E-01	9.90E-01
**BAIAP2L2**	-0.93	5.31E-02	1.00E+00	0.04	9.33E-01	9.90E-01
**SLC8A1-AS1**	-0.61	7.46E-01	1.00E+00	0.06	9.33E-01	9.90E-01
**MARCO**	0.20	7.82E-01	1.00E+00	0.25	9.33E-01	9.90E-01
**ZPLD1**	1.36	6.21E-01	1.00E+00	-0.12	9.33E-01	9.90E-01
**AC037459.3**	-0.25	7.77E-01	1.00E+00	-0.08	9.33E-01	9.90E-01
**PIM3**	-0.59	1.48E-01	1.00E+00	-0.04	9.33E-01	9.90E-01
**POLR1E**	0.08	7.02E-01	1.00E+00	-0.02	9.33E-01	9.90E-01
**AC018529.3**	-1.26	7.16E-01	1.00E+00	0.11	9.33E-01	9.90E-01
**ALAS1**	0.03	8.97E-01	1.00E+00	0.02	9.33E-01	9.90E-01
**GOLGA8B**	-0.62	3.83E-01	1.00E+00	0.05	9.33E-01	9.90E-01
**AC007014.2**	0.95	6.08E-01	1.00E+00	0.06	9.33E-01	9.90E-01

**AC107918.4**	-0.01	9.94E-01	1.00E+00	0.05	9.33E-01	9.90E-01
**AC015921.1**	-2.35	1.13E-01	1.00E+00	-0.11	9.33E-01	9.90E-01
**AL603910.1**	-1.03	4.19E-01	1.00E+00	0.07	9.33E-01	9.90E-01
**AP001282.2**	-1.73	5.80E-01	1.00E+00	-0.05	9.33E-01	9.90E-01
**AC007786.1**	-0.94	5.58E-01	1.00E+00	-0.08	9.34E-01	9.90E-01
**AC073585.1**	-0.44	7.76E-01	1.00E+00	0.12	9.34E-01	9.90E-01
**AC069544.1**	-0.08	9.10E-01	1.00E+00	0.04	9.34E-01	9.90E-01
**OSBPL11**	0.04	8.73E-01	1.00E+00	-0.03	9.34E-01	9.90E-01
**RALY**	-0.16	3.93E-01	1.00E+00	0.03	9.34E-01	9.90E-01
**ANKRD6**	-0.43	2.86E-01	1.00E+00	-0.05	9.34E-01	9.90E-01
**MAD1L1**	-0.32	1.74E-01	1.00E+00	0.01	9.34E-01	9.90E-01
**KRTAP10-9**	-12.88	1.76E-04	4.07E-02	-0.27	9.34E-01	9.90E-01
**AP001992.1**	1.05	4.87E-01	1.00E+00	-0.11	9.34E-01	9.90E-01
**AC068831.7**	-0.65	6.31E-01	1.00E+00	-0.21	9.34E-01	9.90E-01
**AL137009.1**	0.54	6.97E-01	1.00E+00	-0.07	9.34E-01	9.90E-01
**OXSM**	-0.02	9.38E-01	1.00E+00	0.02	9.34E-01	9.90E-01
**AL161891.1**	0.60	3.30E-01	1.00E+00	0.06	9.34E-01	9.90E-01
**C1orf123**	-0.01	9.59E-01	1.00E+00	-0.02	9.34E-01	9.90E-01
**ALOX12P2**	-2.02	1.31E-01	1.00E+00	0.08	9.34E-01	9.90E-01
**LINC00540**	-0.19	9.14E-01	1.00E+00	0.10	9.34E-01	9.90E-01
**PPP1R3B**	0.18	5.42E-01	1.00E+00	-0.03	9.34E-01	9.90E-01
**LRRC37A16P**	0.33	4.30E-01	1.00E+00	-0.02	9.34E-01	9.90E-01
**TARDBP**	0.12	5.76E-01	1.00E+00	0.02	9.34E-01	9.90E-01
**AC003973.3**	1.15	4.16E-01	1.00E+00	0.09	9.34E-01	9.90E-01
**ITPRIPL1**	0.57	3.23E-01	1.00E+00	0.05	9.35E-01	9.90E-01
**FIGNL1**	0.10	6.91E-01	1.00E+00	-0.04	9.35E-01	9.90E-01
**RPSAP15**	-0.01	9.81E-01	1.00E+00	0.03	9.35E-01	9.90E-01
**MMEL1**	0.75	4.47E-01	1.00E+00	0.07	9.35E-01	9.90E-01
**NTRK2**	0.39	4.14E-01	1.00E+00	-0.04	9.35E-01	9.90E-01
**TMEM33**	0.17	4.64E-01	1.00E+00	-0.03	9.35E-01	9.90E-01
**KCTD17**	0.01	9.87E-01	1.00E+00	-0.05	9.35E-01	9.90E-01
**LEF1-AS1**	-2.26	1.09E-01	1.00E+00	-0.07	9.35E-01	9.90E-01

**USP3**	-0.07	7.43E-01	1.00E+00	-0.02	9.35E-01	9.90E-01
**RWDD3**	0.41	6.06E-01	1.00E+00	-0.05	9.35E-01	9.90E-01
**GTSF1**	-3.32	8.83E-02	1.00E+00	-0.06	9.35E-01	9.90E-01
**DENND2C**	0.24	5.73E-01	1.00E+00	0.03	9.35E-01	9.90E-01
**POLD2**	-0.17	4.02E-01	1.00E+00	-0.02	9.35E-01	9.90E-01
**HSPBP1**	-0.27	3.06E-01	1.00E+00	0.02	9.35E-01	9.90E-01
**RASD2**	-0.20	7.43E-01	1.00E+00	-0.05	9.35E-01	9.90E-01
**EVC2**	0.26	3.90E-01	1.00E+00	0.05	9.35E-01	9.90E-01
**SMC5-AS1**	-1.76	9.49E-02	1.00E+00	-0.05	9.35E-01	9.90E-01
**CAMSAP2**	-0.04	8.91E-01	1.00E+00	0.03	9.35E-01	9.90E-01
**ALKBH4**	0.06	7.62E-01	1.00E+00	0.03	9.35E-01	9.90E-01
**E2F1**	-0.09	7.08E-01	1.00E+00	0.06	9.35E-01	9.90E-01
**AL078590.3**	-1.05	1.14E-01	1.00E+00	-0.05	9.35E-01	9.90E-01
**TCEAL4**	0.03	8.78E-01	1.00E+00	-0.02	9.36E-01	9.90E-01
**STK17A**	0.00	9.96E-01	1.00E+00	-0.05	9.36E-01	9.90E-01
**ZMYND10**	0.18	7.88E-01	1.00E+00	-0.06	9.36E-01	9.90E-01
**SREK1IP1**	0.26	3.58E-01	1.00E+00	0.02	9.36E-01	9.90E-01
**GALM**	0.12	7.42E-01	1.00E+00	-0.03	9.36E-01	9.90E-01
**PHACTR4**	-0.19	5.73E-01	1.00E+00	0.02	9.36E-01	9.90E-01
**AC103718.1**	0.09	9.13E-01	1.00E+00	-0.07	9.36E-01	9.90E-01
**SH2D4A**	0.14	6.69E-01	1.00E+00	0.02	9.36E-01	9.90E-01
**CCDC138**	0.06	8.59E-01	1.00E+00	0.04	9.36E-01	9.90E-01
**TPRN**	-0.08	8.32E-01	1.00E+00	-0.03	9.36E-01	9.90E-01
**FYN**	0.09	7.47E-01	1.00E+00	-0.04	9.36E-01	9.90E-01
**AC023790.2**	0.18	8.93E-01	1.00E+00	0.08	9.36E-01	9.90E-01
**PI4KA**	-0.39	5.49E-02	1.00E+00	0.04	9.36E-01	9.90E-01
**BMPER**	-0.13	8.64E-01	1.00E+00	0.16	9.36E-01	9.90E-01
**TUBBP5**	-0.51	6.05E-01	1.00E+00	-0.06	9.36E-01	9.90E-01
**IMPG2**	-1.98	2.44E-01	1.00E+00	-0.13	9.36E-01	9.90E-01
**FBXO42**	0.12	5.67E-01	1.00E+00	-0.02	9.36E-01	9.91E-01
**KRT73**	-2.55	4.24E-01	1.00E+00	0.10	9.37E-01	9.91E-01
**F2R**	0.14	6.17E-01	1.00E+00	-0.02	9.37E-01	9.91E-01

**AP003031.1**	0.55	3.47E-01	1.00E+00	0.04	9.37E-01	9.91E-01
**WSCD1**	0.32	4.06E-01	1.00E+00	0.06	9.37E-01	9.91E-01
**COBL**	0.03	8.98E-01	1.00E+00	-0.04	9.37E-01	9.91E-01
**SLC17A5**	0.05	8.53E-01	1.00E+00	0.02	9.37E-01	9.91E-01
**KANK3**	0.53	3.45E-01	1.00E+00	-0.06	9.37E-01	9.91E-01
**STARD7-AS1**	-0.02	9.53E-01	1.00E+00	0.04	9.37E-01	9.91E-01
**DIAPH1**	-0.06	7.99E-01	1.00E+00	0.03	9.37E-01	9.91E-01
**TCF4**	0.26	3.83E-01	1.00E+00	-0.02	9.37E-01	9.91E-01
**SLC4A11**	-0.25	6.35E-01	1.00E+00	0.05	9.37E-01	9.91E-01
**AC084824.4**	-3.44	5.68E-02	1.00E+00	0.09	9.37E-01	9.91E-01
**TTTY15**	-0.91	2.83E-01	1.00E+00	0.12	9.37E-01	9.91E-01
**SIRT3**	-0.01	9.63E-01	1.00E+00	-0.01	9.37E-01	9.91E-01
**EIF4BP6**	-0.03	9.61E-01	1.00E+00	0.02	9.37E-01	9.91E-01
**AC005899.6**	-0.09	9.41E-01	1.00E+00	0.07	9.37E-01	9.91E-01
**C19orf70**	-0.18	2.65E-01	1.00E+00	-0.02	9.37E-01	9.91E-01
**KAT8**	-0.15	3.28E-01	1.00E+00	-0.04	9.38E-01	9.91E-01
**CRB2**	-1.00	2.14E-01	1.00E+00	-0.25	9.38E-01	9.91E-01
**AC120114.1**	-1.88	1.19E-01	1.00E+00	-0.07	9.38E-01	9.91E-01
**COL6A6**	-0.09	9.04E-01	1.00E+00	-0.25	9.38E-01	9.91E-01
**RPS15AP11**	-0.33	8.60E-01	1.00E+00	0.05	9.38E-01	9.91E-01
**HHIPL1**	-0.12	7.60E-01	1.00E+00	0.23	9.38E-01	9.91E-01
**CYB561D1**	0.52	1.79E-01	1.00E+00	-0.02	9.38E-01	9.91E-01
**ATAD3A**	-0.67	3.42E-02	1.00E+00	-0.04	9.38E-01	9.91E-01
**DTL**	-0.44	2.06E-01	1.00E+00	-0.02	9.38E-01	9.91E-01
**BX537318.1**	-0.28	6.69E-01	1.00E+00	-0.04	9.38E-01	9.91E-01
**MRGBP**	-0.18	4.70E-01	1.00E+00	0.05	9.38E-01	9.91E-01
**FGD5P1**	-0.62	3.60E-01	1.00E+00	-0.11	9.38E-01	9.91E-01
**LCN12**	-4.16	5.13E-02	1.00E+00	0.12	9.38E-01	9.91E-01
**ABHD17A**	-0.09	7.63E-01	1.00E+00	-0.08	9.38E-01	9.91E-01
**RPS13P2**	-1.40	1.61E-01	1.00E+00	-0.06	9.38E-01	9.91E-01
**AL450384.2**	-1.69	1.43E-01	1.00E+00	0.05	9.38E-01	9.91E-01
**PCDH19**	0.24	5.78E-01	1.00E+00	-0.06	9.38E-01	9.91E-01

**AC009053.1**	0.09	8.50E-01	1.00E+00	0.04	9.38E-01	9.91E-01
**G37033**	-3.42	6.14E-02	1.00E+00	0.09	9.38E-01	9.91E-01
**AC005225.4**	0.38	5.85E-01	1.00E+00	-0.06	9.38E-01	9.91E-01
**MST1P2**	-0.19	7.60E-01	1.00E+00	-0.06	9.38E-01	9.91E-01
**AC245060.5**	0.80	3.05E-01	1.00E+00	0.05	9.38E-01	9.91E-01
**LEMD3**	0.00	9.89E-01	1.00E+00	-0.03	9.39E-01	9.91E-01
**GRAMD4**	-0.05	7.99E-01	1.00E+00	-0.03	9.39E-01	9.91E-01
**FAM83G**	-0.28	5.40E-01	1.00E+00	0.05	9.39E-01	9.91E-01
**PAPPA2**	0.27	8.07E-01	1.00E+00	0.07	9.39E-01	9.91E-01
**SEC63**	0.04	7.82E-01	1.00E+00	0.01	9.39E-01	9.91E-01
**AC092902.2**	0.75	2.60E-01	1.00E+00	0.04	9.39E-01	9.91E-01
**HIST1H3E**	0.73	2.82E-01	1.00E+00	0.06	9.39E-01	9.91E-01
**AL391058.1**	0.55	6.71E-01	1.00E+00	-0.04	9.39E-01	9.91E-01
**UBQLNL**	0.41	7.86E-01	1.00E+00	-0.13	9.39E-01	9.91E-01
**TRAM1**	0.18	3.79E-01	1.00E+00	0.02	9.39E-01	9.91E-01
**CAST**	0.06	7.07E-01	1.00E+00	0.01	9.39E-01	9.91E-01
**AL354761.1**	0.34	6.76E-01	1.00E+00	-0.07	9.39E-01	9.91E-01
**AGK**	-0.22	3.03E-01	1.00E+00	-0.01	9.39E-01	9.91E-01
**UBE2Q1**	0.07	7.44E-01	1.00E+00	-0.03	9.39E-01	9.91E-01
**USE1**	-0.02	8.90E-01	1.00E+00	0.02	9.39E-01	9.91E-01
**BRD1**	-0.22	4.86E-01	1.00E+00	0.03	9.39E-01	9.91E-01
**AL133367.1**	0.26	6.21E-01	1.00E+00	-0.06	9.39E-01	9.91E-01
**RNF123**	-0.15	4.32E-01	1.00E+00	0.04	9.39E-01	9.91E-01
**AL158152.2**	1.08	3.46E-01	1.00E+00	-0.05	9.39E-01	9.91E-01
**ZFHX4-AS1**	-1.85	4.89E-01	1.00E+00	0.24	9.39E-01	9.91E-01
**RCC1L**	0.26	2.58E-01	1.00E+00	-0.05	9.39E-01	9.91E-01
**NYNRIN**	-0.23	4.33E-01	1.00E+00	0.04	9.39E-01	9.91E-01
**PID1**	0.75	2.11E-02	8.94E-01	0.03	9.39E-01	9.91E-01
**SAMD1**	-0.22	3.61E-01	1.00E+00	-0.04	9.39E-01	9.91E-01
**AKAP7**	0.41	1.51E-01	1.00E+00	-0.04	9.39E-01	9.91E-01
**CCAR2**	-0.10	6.13E-01	1.00E+00	-0.03	9.39E-01	9.91E-01
**GALNT17**	-0.21	7.55E-01	1.00E+00	-0.17	9.40E-01	9.91E-01

**AC015813.6**	0.68	9.23E-02	1.00E+00	-0.04	9.40E-01	9.91E-01
**SLC6A9**	-0.03	9.46E-01	1.00E+00	0.03	9.40E-01	9.91E-01
**AC112907.3**	0.56	5.80E-01	1.00E+00	-0.08	9.40E-01	9.91E-01
**ARMCX1**	0.30	3.17E-01	1.00E+00	-0.02	9.40E-01	9.91E-01
**PLRG1**	0.04	8.12E-01	1.00E+00	0.01	9.40E-01	9.91E-01
**PROSER1**	-0.16	5.26E-01	1.00E+00	-0.02	9.40E-01	9.91E-01
**TBC1D5**	-0.02	9.40E-01	1.00E+00	0.02	9.40E-01	9.91E-01
**AC091163.1**	-0.32	8.21E-01	1.00E+00	-0.11	9.40E-01	9.91E-01
**GPS1**	-0.27	1.57E-01	1.00E+00	-0.01	9.40E-01	9.91E-01
**XLOC_006168**	-0.91	2.51E-01	1.00E+00	0.07	9.40E-01	9.91E-01
**AL357054.2**	-3.59	1.63E-01	1.00E+00	0.07	9.40E-01	9.91E-01
**C16orf72**	0.03	8.74E-01	1.00E+00	-0.03	9.40E-01	9.91E-01
**CHIC2**	0.00	9.97E-01	1.00E+00	-0.02	9.40E-01	9.91E-01
**AC123768.3**	-0.75	7.99E-01	1.00E+00	-0.05	9.40E-01	9.91E-01
**MYO5B**	-0.47	3.00E-01	1.00E+00	0.04	9.40E-01	9.91E-01
**ZFP64**	-0.14	6.13E-01	1.00E+00	-0.02	9.40E-01	9.91E-01
**SHF**	-0.24	4.59E-01	1.00E+00	0.05	9.40E-01	9.91E-01
**AC099518.6**	0.11	8.66E-01	1.00E+00	0.06	9.40E-01	9.91E-01
**ESRRA**	-0.19	4.70E-01	1.00E+00	0.05	9.40E-01	9.91E-01
**TRMT112**	0.05	7.42E-01	1.00E+00	-0.02	9.40E-01	9.91E-01
**CAPN14**	-0.83	2.87E-01	1.00E+00	0.08	9.40E-01	9.91E-01
**SNRPA**	-0.10	6.46E-01	1.00E+00	0.01	9.41E-01	9.91E-01
**C8orf58**	-0.01	9.83E-01	1.00E+00	-0.03	9.41E-01	9.91E-01
**ARL17A**	-0.03	9.77E-01	1.00E+00	-0.04	9.41E-01	9.91E-01
**MAPT**	-0.15	6.46E-01	1.00E+00	0.03	9.41E-01	9.91E-01
**DOCK9**	0.00	9.94E-01	1.00E+00	0.02	9.41E-01	9.91E-01
**MSANTD3**	-0.16	5.06E-01	1.00E+00	-0.02	9.41E-01	9.91E-01
**PIGQ**	-0.07	7.73E-01	1.00E+00	0.03	9.41E-01	9.91E-01
**AC022916.1**	0.01	9.90E-01	1.00E+00	0.02	9.41E-01	9.91E-01
**AL031429.2**	1.08	3.52E-01	1.00E+00	-0.06	9.41E-01	9.91E-01
**RP11-353N4.8**	-1.16	7.39E-01	1.00E+00	0.07	9.41E-01	9.91E-01
**SIX1**	3.95	2.75E-02	9.69E-01	-0.06	9.41E-01	9.91E-01

**BCAS3**	-0.35	1.49E-01	1.00E+00	-0.03	9.41E-01	9.91E-01
**YWHAZP5**	0.14	9.67E-01	1.00E+00	0.05	9.41E-01	9.91E-01
**AL162431.2**	0.00	9.98E-01	1.00E+00	-0.07	9.41E-01	9.91E-01
**G10803**	-1.52	6.60E-01	1.00E+00	0.24	9.41E-01	9.91E-01
**AL137782.1**	-2.12	4.31E-01	1.00E+00	0.06	9.41E-01	9.91E-01
**ALAD**	0.08	7.57E-01	1.00E+00	-0.02	9.41E-01	9.91E-01
**LINC01415**	-0.31	5.57E-01	1.00E+00	0.05	9.41E-01	9.91E-01
**GFI1B**	-0.12	9.27E-01	1.00E+00	-0.06	9.41E-01	9.91E-01
**R3HDM4**	-0.42	1.07E-01	1.00E+00	-0.05	9.41E-01	9.91E-01
**CENPN**	-0.42	2.66E-01	1.00E+00	0.02	9.41E-01	9.91E-01
**SNRPC**	-0.10	6.11E-01	1.00E+00	-0.02	9.41E-01	9.91E-01
**CFD**	1.05	2.70E-02	9.59E-01	-0.04	9.42E-01	9.91E-01
**DUSP26**	-3.41	2.10E-03	2.39E-01	-0.10	9.42E-01	9.91E-01
**AP000808.1**	-0.03	9.50E-01	1.00E+00	-0.08	9.42E-01	9.91E-01
**C9orf85**	0.05	8.25E-01	1.00E+00	0.02	9.42E-01	9.91E-01
**GPC6**	0.88	2.55E-01	1.00E+00	-0.04	9.42E-01	9.91E-01
**TFPI**	0.77	3.70E-02	1.00E+00	-0.03	9.42E-01	9.91E-01
**G35948**	0.81	4.92E-01	1.00E+00	0.06	9.42E-01	9.91E-01
**SLC5A5**	-3.74	7.98E-02	1.00E+00	0.23	9.42E-01	9.91E-01
**PIN1**	-0.13	5.72E-01	1.00E+00	-0.02	9.42E-01	9.91E-01
**AC093583.1**	0.17	9.60E-01	1.00E+00	-0.13	9.42E-01	9.91E-01
**AP005018.2**	-2.44	4.47E-01	1.00E+00	-0.07	9.42E-01	9.91E-01
**LDLRAD4-AS1**	-1.22	1.79E-01	1.00E+00	0.08	9.42E-01	9.91E-01
**CYP2A6**	2.83	1.10E-01	1.00E+00	0.10	9.42E-01	9.91E-01
**KRT3**	-0.03	9.65E-01	1.00E+00	-0.23	9.42E-01	9.91E-01
**NR2F6**	0.14	5.37E-01	1.00E+00	0.04	9.42E-01	9.91E-01
**DCUN1D3**	0.39	1.47E-01	1.00E+00	-0.04	9.42E-01	9.91E-01
**TNS2**	0.05	9.08E-01	1.00E+00	0.05	9.42E-01	9.91E-01
**AC002075.2**	1.74	5.13E-01	1.00E+00	0.06	9.42E-01	9.91E-01
**MBTD1**	0.24	3.15E-01	1.00E+00	0.03	9.42E-01	9.91E-01
**GP6**	0.44	7.10E-01	1.00E+00	0.07	9.42E-01	9.91E-01
**PARP2**	-0.13	6.38E-01	1.00E+00	-0.02	9.42E-01	9.91E-01

**UQCRBP1**	-1.62	6.37E-01	1.00E+00	0.08	9.42E-01	9.91E-01
**C17orf99**	-1.07	3.13E-01	1.00E+00	0.06	9.43E-01	9.91E-01
**C21orf91**	-0.05	9.12E-01	1.00E+00	0.02	9.43E-01	9.91E-01
**MST1R**	-0.01	9.73E-01	1.00E+00	-0.04	9.43E-01	9.91E-01
**LINC01508**	-2.49	1.57E-01	1.00E+00	-0.09	9.43E-01	9.91E-01
**NUTM2D**	-0.13	8.52E-01	1.00E+00	0.22	9.43E-01	9.91E-01
**ACTA2**	-0.05	9.30E-01	1.00E+00	0.03	9.43E-01	9.91E-01
**LINC01353**	-0.27	8.68E-01	1.00E+00	0.07	9.43E-01	9.91E-01
**GSDMB**	-1.19	1.93E-01	1.00E+00	0.06	9.43E-01	9.91E-01
**AC104389.4**	0.82	6.03E-01	1.00E+00	0.07	9.43E-01	9.91E-01
**SERPINA4**	-2.28	5.01E-01	1.00E+00	0.14	9.43E-01	9.91E-01
**FLJ12825**	-0.81	4.52E-01	1.00E+00	0.07	9.43E-01	9.91E-01
**HSPA5**	-0.66	8.96E-03	5.88E-01	0.02	9.43E-01	9.91E-01
**AC020912.1**	-0.79	5.75E-01	1.00E+00	-0.11	9.43E-01	9.91E-01
**AP000240.1**	0.68	3.56E-01	1.00E+00	-0.06	9.43E-01	9.91E-01
**G2136**	1.05	3.16E-01	1.00E+00	0.09	9.43E-01	9.91E-01
**AL136985.3**	-1.79	6.00E-01	1.00E+00	-0.23	9.43E-01	9.91E-01
**RPL7AP11**	-0.10	9.59E-01	1.00E+00	0.04	9.43E-01	9.91E-01
**NR3C1**	0.19	2.67E-01	1.00E+00	-0.01	9.43E-01	9.91E-01
**CEACAM1**	-0.12	8.77E-01	1.00E+00	-0.03	9.43E-01	9.91E-01
**COL17A1**	-0.12	6.65E-01	1.00E+00	-0.04	9.43E-01	9.91E-01
**CADPS2**	0.05	9.13E-01	1.00E+00	0.05	9.43E-01	9.91E-01
**ADTRP**	-0.32	5.25E-01	1.00E+00	-0.04	9.43E-01	9.91E-01
**SSBP2**	0.03	8.69E-01	1.00E+00	-0.02	9.43E-01	9.91E-01
**F13A1**	0.12	8.04E-01	1.00E+00	0.04	9.43E-01	9.91E-01
**VSIG10L2**	0.32	5.64E-01	1.00E+00	-0.06	9.43E-01	9.91E-01
**UCN2**	-1.49	1.17E-02	6.70E-01	0.06	9.43E-01	9.91E-01
**IQCK**	-0.60	1.80E-01	1.00E+00	0.02	9.43E-01	9.91E-01
**RPL12P10**	0.46	6.65E-01	1.00E+00	-0.12	9.44E-01	9.91E-01
**AC108866.1**	0.89	5.89E-01	1.00E+00	0.06	9.44E-01	9.91E-01
**AC008781.2**	-0.03	9.92E-01	1.00E+00	-0.09	9.44E-01	9.91E-01
**KRTAP29-1**	-3.45	3.00E-01	1.00E+00	-0.23	9.44E-01	9.91E-01

**PDPK1**	0.28	1.03E-01	1.00E+00	0.02	9.44E-01	9.91E-01
**AC012467.2**	0.74	2.02E-01	1.00E+00	0.05	9.44E-01	9.91E-01
**MANF**	-0.09	7.02E-01	1.00E+00	0.02	9.44E-01	9.91E-01
**MYBPC1**	1.02	1.17E-01	1.00E+00	-0.06	9.44E-01	9.91E-01
**G8112**	-1.05	1.81E-01	1.00E+00	0.04	9.44E-01	9.91E-01
**LINC00649**	-0.41	4.49E-01	1.00E+00	-0.03	9.45E-01	9.92E-01
**FTH1P20**	-1.15	4.15E-01	1.00E+00	-0.05	9.45E-01	9.92E-01
**MCM3**	-0.23	1.22E-01	1.00E+00	0.01	9.45E-01	9.92E-01
**PRSS12**	-0.07	8.05E-01	1.00E+00	-0.04	9.45E-01	9.92E-01
**KCTD5**	0.05	8.71E-01	1.00E+00	-0.03	9.45E-01	9.92E-01
**C3orf18**	0.07	8.95E-01	1.00E+00	0.03	9.45E-01	9.92E-01
**POLG**	0.12	5.55E-01	1.00E+00	0.04	9.45E-01	9.92E-01
**CHCHD5**	-0.16	4.73E-01	1.00E+00	0.02	9.45E-01	9.92E-01
**AP001542.1**	-0.96	3.11E-01	1.00E+00	-0.07	9.45E-01	9.92E-01
**AC093673.1**	-0.13	8.04E-01	1.00E+00	0.05	9.45E-01	9.92E-01
**WDR5**	0.33	1.96E-01	1.00E+00	0.02	9.45E-01	9.92E-01
**G40462**	-1.78	1.11E-01	1.00E+00	-0.16	9.45E-01	9.92E-01
**PRR5**	0.51	2.00E-01	1.00E+00	-0.04	9.45E-01	9.92E-01
**LAIR2**	-0.92	7.84E-01	1.00E+00	0.08	9.45E-01	9.92E-01
**TUBA3D**	-0.83	5.38E-01	1.00E+00	-0.07	9.45E-01	9.92E-01
**PPCDC**	0.09	7.85E-01	1.00E+00	0.01	9.45E-01	9.92E-01
**ZNF786**	-0.38	2.22E-01	1.00E+00	0.02	9.45E-01	9.92E-01
**UGCG**	0.27	5.98E-01	1.00E+00	0.02	9.46E-01	9.92E-01
**CRYBG1**	0.28	4.53E-01	1.00E+00	0.03	9.46E-01	9.92E-01
**SNX19**	0.44	4.48E-02	1.00E+00	0.03	9.46E-01	9.92E-01
**MARK3**	-0.03	8.87E-01	1.00E+00	0.04	9.46E-01	9.92E-01
**TNFRSF8**	-0.65	3.19E-01	1.00E+00	-0.05	9.46E-01	9.92E-01
**AC103563.7**	-2.25	1.85E-01	1.00E+00	0.09	9.46E-01	9.92E-01
**PRSS57**	-1.44	2.79E-01	1.00E+00	0.10	9.46E-01	9.92E-01
**SMG1P3**	-0.06	8.90E-01	1.00E+00	0.04	9.46E-01	9.92E-01
**PURB**	-0.11	6.61E-01	1.00E+00	-0.03	9.46E-01	9.92E-01
**CEP72**	0.13	7.28E-01	1.00E+00	0.03	9.46E-01	9.92E-01

**AC007969.1**	-0.08	8.38E-01	1.00E+00	0.05	9.46E-01	9.92E-01
**G22256**	0.59	6.62E-01	1.00E+00	-0.05	9.46E-01	9.92E-01
**SCRG1**	-0.47	5.21E-01	1.00E+00	-0.04	9.46E-01	9.92E-01
**NBPF20**	-1.22	7.51E-02	1.00E+00	0.03	9.46E-01	9.92E-01
**CEP89**	-0.25	1.34E-01	1.00E+00	0.01	9.46E-01	9.92E-01
**AC090360.1**	-0.03	9.86E-01	1.00E+00	-0.08	9.46E-01	9.92E-01
**CPNE8**	0.64	1.42E-01	1.00E+00	-0.02	9.46E-01	9.92E-01
**MORN3**	0.18	5.76E-01	1.00E+00	-0.03	9.46E-01	9.92E-01
**AC012306.2**	-0.45	1.56E-01	1.00E+00	0.02	9.46E-01	9.92E-01
**ATP1A2**	0.65	3.61E-01	1.00E+00	0.05	9.46E-01	9.92E-01
**IL1RL2**	0.22	6.19E-01	1.00E+00	-0.03	9.46E-01	9.92E-01
**AC007342.7**	-0.84	2.14E-01	1.00E+00	0.05	9.46E-01	9.92E-01
**AL445248.1**	1.21	7.24E-01	1.00E+00	0.06	9.46E-01	9.92E-01
**TECRL**	2.09	2.18E-01	1.00E+00	-0.22	9.46E-01	9.92E-01
**KCNMB1**	0.27	6.50E-01	1.00E+00	-0.02	9.46E-01	9.92E-01
**DDX18**	0.20	2.03E-01	1.00E+00	0.01	9.46E-01	9.92E-01
**TAGLN2**	-0.12	6.08E-01	1.00E+00	-0.01	9.46E-01	9.92E-01
**HBB**	0.43	7.37E-01	1.00E+00	-0.21	9.46E-01	9.92E-01
**XLOC_011942**	2.37	2.20E-01	1.00E+00	-0.19	9.46E-01	9.92E-01
**DUS1L**	-0.18	3.90E-01	1.00E+00	-0.04	9.46E-01	9.92E-01
**CLDN2**	0.21	8.17E-01	1.00E+00	0.21	9.46E-01	9.92E-01
**PTPRA**	-0.05	7.95E-01	1.00E+00	0.03	9.46E-01	9.92E-01
**TMLHE-AS1**	-2.45	2.23E-01	1.00E+00	-0.07	9.47E-01	9.92E-01
**ATP5S**	0.04	8.32E-01	1.00E+00	-0.02	9.47E-01	9.92E-01
**TBL3**	-0.26	3.32E-01	1.00E+00	0.01	9.47E-01	9.92E-01
**IGFBPL1**	-3.21	2.20E-01	1.00E+00	-0.11	9.47E-01	9.92E-01
**TPGS1**	-0.30	3.15E-01	1.00E+00	-0.06	9.47E-01	9.92E-01
**G11773**	-0.74	3.74E-01	1.00E+00	0.05	9.47E-01	9.92E-01
**AC123912.4**	0.36	6.81E-01	1.00E+00	0.07	9.47E-01	9.92E-01
**ATP2A1-AS1**	-2.04	4.77E-01	1.00E+00	-0.06	9.47E-01	9.92E-01
**DOC2B**	-0.01	9.88E-01	1.00E+00	-0.21	9.47E-01	9.92E-01
**LINC02356**	-2.91	5.07E-02	1.00E+00	-0.06	9.47E-01	9.92E-01

**AGGF1P1**	0.05	9.85E-01	1.00E+00	-0.13	9.47E-01	9.92E-01
**SPAST**	0.16	5.24E-01	1.00E+00	0.01	9.47E-01	9.92E-01
**SMUG1-AS1**	-0.26	6.64E-01	1.00E+00	-0.05	9.47E-01	9.92E-01
**LINC00398**	0.09	9.39E-01	1.00E+00	-0.08	9.47E-01	9.92E-01
**G11316**	-1.93	4.11E-01	1.00E+00	-0.12	9.47E-01	9.92E-01
**SLC51A**	0.08	7.88E-01	1.00E+00	-0.03	9.47E-01	9.92E-01
**FTH1P3**	-0.06	9.86E-01	1.00E+00	-0.04	9.47E-01	9.92E-01
**CDH16**	-1.58	3.98E-01	1.00E+00	-0.11	9.47E-01	9.92E-01
**TWF1P1**	-0.77	3.75E-01	1.00E+00	0.03	9.47E-01	9.92E-01
**PTGDR2**	1.92	4.37E-03	3.74E-01	0.08	9.47E-01	9.92E-01
**HOMER1**	-0.47	1.77E-01	1.00E+00	-0.03	9.48E-01	9.92E-01
**CCNB1**	0.32	1.75E-01	1.00E+00	-0.02	9.48E-01	9.92E-01
**AC079140.5**	-1.80	3.54E-01	1.00E+00	-0.07	9.48E-01	9.92E-01
**NKTR**	0.35	4.75E-01	1.00E+00	-0.02	9.48E-01	9.92E-01
**PFN4**	0.38	6.98E-01	1.00E+00	-0.06	9.48E-01	9.92E-01
**AC069547.1**	-0.90	3.72E-02	1.00E+00	-0.03	9.48E-01	9.92E-01
**RASL10A**	-0.26	7.64E-01	1.00E+00	0.07	9.48E-01	9.92E-01
**TSTD2**	-0.09	7.62E-01	1.00E+00	-0.02	9.48E-01	9.92E-01
**DERL1**	0.46	5.42E-02	1.00E+00	0.01	9.48E-01	9.92E-01
**AC024940.1**	0.44	5.90E-01	1.00E+00	0.05	9.48E-01	9.92E-01
**SMC6**	-0.16	4.79E-01	1.00E+00	0.01	9.48E-01	9.92E-01
**SPRYD4**	-0.23	3.23E-01	1.00E+00	-0.01	9.48E-01	9.92E-01
**KCNQ4**	-0.90	6.24E-02	1.00E+00	-0.06	9.48E-01	9.92E-01
**PDZD7**	0.39	3.49E-01	1.00E+00	-0.05	9.48E-01	9.92E-01
**KRT8**	-0.06	9.45E-01	1.00E+00	0.04	9.48E-01	9.92E-01
**C1orf52**	0.03	8.76E-01	1.00E+00	0.01	9.48E-01	9.92E-01
**DEPP1**	-0.23	5.58E-01	1.00E+00	-0.04	9.48E-01	9.92E-01
**ESYT2**	0.16	3.25E-01	1.00E+00	-0.03	9.48E-01	9.92E-01
**DEGS2**	-0.02	9.72E-01	1.00E+00	0.04	9.48E-01	9.92E-01
**RABL2B**	-0.10	7.29E-01	1.00E+00	0.01	9.48E-01	9.92E-01
**AL162731.1**	-1.86	3.05E-01	1.00E+00	0.07	9.48E-01	9.92E-01
**EHD4-AS1**	-0.25	8.80E-01	1.00E+00	0.06	9.48E-01	9.92E-01

**G26247**	0.63	6.67E-01	1.00E+00	-0.07	9.48E-01	9.92E-01
**TJP1**	-0.12	5.03E-01	1.00E+00	-0.01	9.49E-01	9.92E-01
**YTHDF2P1**	-0.18	9.59E-01	1.00E+00	0.03	9.49E-01	9.92E-01
**XLOC_014405**	0.12	9.33E-01	1.00E+00	-0.09	9.49E-01	9.92E-01
**LANCL2**	-0.15	6.05E-01	1.00E+00	-0.01	9.49E-01	9.92E-01
**SPACA9**	-0.15	5.40E-01	1.00E+00	-0.02	9.49E-01	9.92E-01
**ULK4P3**	2.54	2.17E-01	1.00E+00	0.07	9.49E-01	9.92E-01
**DKFZp779M0652**	1.97	4.56E-02	1.00E+00	-0.11	9.49E-01	9.92E-01
**RAB5A**	-0.20	4.70E-01	1.00E+00	-0.01	9.49E-01	9.92E-01
**RND1**	-2.37	4.64E-02	1.00E+00	-0.06	9.49E-01	9.92E-01
**RPL22**	0.12	5.13E-01	1.00E+00	-0.01	9.49E-01	9.92E-01
**LRP2**	-4.21	5.28E-05	1.40E-02	0.20	9.49E-01	9.92E-01
**XLOC_011309**	1.36	5.27E-01	1.00E+00	-0.10	9.49E-01	9.92E-01
**PCID2**	0.24	3.09E-01	1.00E+00	-0.02	9.49E-01	9.92E-01
**CDHR4**	-3.51	9.95E-03	6.19E-01	0.08	9.49E-01	9.92E-01
**AF064858.3**	-1.76	1.19E-01	1.00E+00	0.05	9.49E-01	9.92E-01
**ETV7**	-0.04	9.40E-01	1.00E+00	0.03	9.49E-01	9.92E-01
**AC015883.1**	1.37	2.73E-01	1.00E+00	-0.08	9.49E-01	9.92E-01
**FAM35DP**	-0.98	3.18E-01	1.00E+00	-0.03	9.49E-01	9.92E-01
**GPBP1**	0.11	4.91E-01	1.00E+00	-0.01	9.50E-01	9.92E-01
**CATSPERG**	-0.24	5.97E-01	1.00E+00	0.03	9.50E-01	9.93E-01
**RPS14P8**	-2.07	5.39E-01	1.00E+00	0.20	9.50E-01	9.93E-01
**VILL**	0.23	2.86E-01	1.00E+00	-0.03	9.50E-01	9.93E-01
**GUSBP9**	0.59	5.42E-01	1.00E+00	0.06	9.50E-01	9.93E-01
**XLOC_000318**	1.17	2.31E-01	1.00E+00	-0.16	9.50E-01	9.93E-01
**AC108488.1**	0.61	5.72E-01	1.00E+00	-0.04	9.50E-01	9.93E-01
**HCG25**	-2.32	1.76E-01	1.00E+00	-0.05	9.50E-01	9.93E-01
**ZNF736P9Y**	0.61	6.81E-01	1.00E+00	0.12	9.50E-01	9.93E-01
**PTGR2**	0.87	6.79E-02	1.00E+00	-0.03	9.50E-01	9.93E-01
**XLOC_008126**	0.35	8.50E-01	1.00E+00	-0.07	9.50E-01	9.93E-01
**RPL23AP2**	1.50	6.62E-01	1.00E+00	0.05	9.50E-01	9.93E-01
**TRAF6**	0.05	7.95E-01	1.00E+00	-0.02	9.50E-01	9.93E-01

**XLOC_011015**	1.57	3.98E-01	1.00E+00	0.06	9.50E-01	9.93E-01
**AL137847.2**	3.15	1.49E-01	1.00E+00	-0.06	9.50E-01	9.93E-01
**TRIOBP**	-0.03	9.12E-01	1.00E+00	-0.03	9.50E-01	9.93E-01
**POLDIP3**	0.14	4.62E-01	1.00E+00	-0.01	9.50E-01	9.93E-01
**LINC00571**	-0.05	9.54E-01	1.00E+00	-0.06	9.50E-01	9.93E-01
**RPL13P2**	NA	NA	NA	0.05	9.50E-01	9.93E-01
**G36172**	-0.38	7.91E-01	1.00E+00	-0.04	9.51E-01	9.93E-01
**AL356258.1**	4.08	1.51E-01	1.00E+00	0.20	9.51E-01	9.93E-01
**TATDN2P3**	2.15	2.16E-02	8.94E-01	-0.08	9.51E-01	9.93E-01
**SEC62**	0.29	2.97E-01	1.00E+00	-0.01	9.51E-01	9.93E-01
**DCAF8**	0.20	4.05E-01	1.00E+00	0.01	9.51E-01	9.93E-01
**CCDC142**	0.04	9.23E-01	1.00E+00	0.04	9.51E-01	9.93E-01
**XLOC_006198**	-0.79	8.19E-01	1.00E+00	0.09	9.51E-01	9.93E-01
**UVRAG**	0.28	2.55E-01	1.00E+00	0.02	9.51E-01	9.93E-01
**PCOTH**	0.44	8.99E-01	1.00E+00	-0.06	9.51E-01	9.93E-01
**F2RL3**	-0.51	4.77E-01	1.00E+00	-0.05	9.51E-01	9.93E-01
**CLCN6**	0.24	5.17E-01	1.00E+00	0.03	9.51E-01	9.93E-01
**REPS2**	-0.01	9.76E-01	1.00E+00	0.03	9.51E-01	9.93E-01
**PCDHGA4**	-0.22	6.60E-01	1.00E+00	-0.06	9.51E-01	9.93E-01
**MYH1**	3.62	1.01E-01	1.00E+00	-0.09	9.51E-01	9.93E-01
**XLOC_013282**	-0.02	9.70E-01	1.00E+00	-0.04	9.51E-01	9.93E-01
**AC069185.1**	-0.12	8.77E-01	1.00E+00	0.04	9.51E-01	9.93E-01
**RPL34-AS1**	0.85	2.19E-01	1.00E+00	0.04	9.52E-01	9.93E-01
**PPL**	-0.16	7.27E-01	1.00E+00	0.03	9.52E-01	9.93E-01
**AC005775.1**	-2.23	2.65E-01	1.00E+00	0.07	9.52E-01	9.93E-01
**ATP8B4**	0.84	2.55E-01	1.00E+00	-0.04	9.52E-01	9.93E-01
**AL118505.1**	0.04	9.45E-01	1.00E+00	-0.05	9.52E-01	9.93E-01
**LDB2**	0.27	6.00E-01	1.00E+00	-0.03	9.52E-01	9.93E-01
**AC087477.2**	-0.16	8.67E-01	1.00E+00	-0.05	9.52E-01	9.93E-01
**NAA10**	-0.01	9.83E-01	1.00E+00	0.01	9.52E-01	9.93E-01
**TEDC1**	-0.31	3.78E-01	1.00E+00	-0.04	9.52E-01	9.93E-01
**MIR762HG**	-2.49	1.23E-01	1.00E+00	0.03	9.52E-01	9.93E-01

**NDUFS7**	-0.41	4.02E-01	1.00E+00	-0.04	9.52E-01	9.93E-01
**G11672**	-0.26	7.32E-01	1.00E+00	-0.10	9.52E-01	9.93E-01
**MFAP1**	0.11	5.41E-01	1.00E+00	-0.01	9.52E-01	9.93E-01
**AC025569.1**	0.28	8.86E-01	1.00E+00	0.06	9.52E-01	9.93E-01
**HNRNPR**	-0.04	8.04E-01	1.00E+00	0.01	9.52E-01	9.93E-01
**RHOD**	0.01	9.83E-01	1.00E+00	0.02	9.52E-01	9.93E-01
**CCP110**	0.08	7.80E-01	1.00E+00	-0.01	9.52E-01	9.93E-01
**ZNF764**	-0.06	8.09E-01	1.00E+00	-0.03	9.52E-01	9.93E-01
**RASD1**	0.28	6.37E-01	1.00E+00	0.04	9.52E-01	9.93E-01
**FAM160A1-DT**	0.71	1.99E-01	1.00E+00	-0.05	9.52E-01	9.93E-01
**CTXN1**	-1.12	1.08E-02	6.40E-01	0.05	9.52E-01	9.93E-01
**TMEM62**	0.27	3.86E-01	1.00E+00	-0.03	9.52E-01	9.93E-01
**ANLN**	-0.05	8.93E-01	1.00E+00	0.03	9.52E-01	9.93E-01
**NELL1**	1.43	3.38E-01	1.00E+00	-0.19	9.52E-01	9.93E-01
**LINC00852**	0.29	6.23E-01	1.00E+00	0.03	9.52E-01	9.93E-01
**AL390195.2**	0.38	5.81E-01	1.00E+00	-0.05	9.52E-01	9.93E-01
**AC098934.2**	-1.65	1.50E-01	1.00E+00	-0.07	9.53E-01	9.93E-01
**AC099687.1**	0.65	6.88E-01	1.00E+00	0.06	9.53E-01	9.93E-01
**AL360270.2**	0.39	7.55E-01	1.00E+00	0.06	9.53E-01	9.93E-01
**PCP4L1**	-0.44	4.51E-01	1.00E+00	-0.03	9.53E-01	9.93E-01
**GPRC5A**	-0.22	6.94E-01	1.00E+00	0.03	9.53E-01	9.93E-01
**AC105052.2**	-0.85	5.57E-01	1.00E+00	-0.06	9.53E-01	9.93E-01
**FBXL19**	-0.55	1.35E-01	1.00E+00	0.19	9.53E-01	9.93E-01
**AC009831.3**	2.47	1.86E-01	1.00E+00	-0.06	9.53E-01	9.93E-01
**NCOR1**	-0.19	3.28E-01	1.00E+00	-0.01	9.53E-01	9.93E-01
**SPTBN2**	-0.34	4.52E-01	1.00E+00	0.04	9.53E-01	9.93E-01
**CAVIN1**	0.21	5.64E-01	1.00E+00	0.03	9.53E-01	9.93E-01
**EFCAB13**	-0.82	2.41E-01	1.00E+00	0.03	9.53E-01	9.93E-01
**PRPH**	0.35	6.77E-01	1.00E+00	-0.04	9.53E-01	9.93E-01
**THSD7A**	0.17	7.10E-01	1.00E+00	-0.03	9.53E-01	9.93E-01
**KDM4A-AS1**	0.20	8.78E-01	1.00E+00	-0.05	9.53E-01	9.93E-01
**ZDHHC9**	0.16	6.19E-01	1.00E+00	-0.01	9.53E-01	9.93E-01

**RPL23AP78**	NA	NA	NA	0.08	9.53E-01	9.93E-01
**Z93930.3**	-3.03	2.37E-02	9.15E-01	-0.05	9.53E-01	9.93E-01
**ZNF551**	0.36	1.54E-01	1.00E+00	0.02	9.53E-01	9.93E-01
**LENG8-AS1**	-0.57	2.97E-01	1.00E+00	-0.04	9.53E-01	9.93E-01
**PLVAP**	0.03	9.60E-01	1.00E+00	-0.03	9.53E-01	9.93E-01
**POLR2J2**	-0.31	8.00E-01	1.00E+00	-0.03	9.53E-01	9.93E-01
**METTL14**	-0.04	8.41E-01	1.00E+00	0.01	9.53E-01	9.93E-01
**NOTUM**	0.04	9.13E-01	1.00E+00	0.05	9.53E-01	9.93E-01
**XRCC2**	-0.70	8.85E-02	1.00E+00	0.05	9.54E-01	9.93E-01
**TBX21**	-0.70	5.34E-01	1.00E+00	0.18	9.54E-01	9.93E-01
**ZNF417**	0.11	7.82E-01	1.00E+00	0.02	9.54E-01	9.93E-01
**KNSTRN**	-0.18	4.11E-01	1.00E+00	0.02	9.54E-01	9.93E-01
**LINC01836**	0.31	6.35E-01	1.00E+00	-0.05	9.54E-01	9.93E-01
**G4388**	0.75	2.65E-01	1.00E+00	-0.05	9.54E-01	9.93E-01
**HUNK**	-0.30	3.70E-01	1.00E+00	-0.03	9.54E-01	9.93E-01
**LOXL4**	0.52	2.52E-01	1.00E+00	0.03	9.54E-01	9.93E-01
**ZBTB22**	-0.16	5.61E-01	1.00E+00	0.03	9.54E-01	9.93E-01
**ANKK1**	0.00	9.99E-01	1.00E+00	0.05	9.54E-01	9.93E-01
**PSMD1**	0.00	9.92E-01	1.00E+00	-0.01	9.54E-01	9.93E-01
**AC008464.1**	2.85	7.79E-02	1.00E+00	0.11	9.54E-01	9.93E-01
**MRPL9**	0.07	7.25E-01	1.00E+00	-0.01	9.54E-01	9.93E-01
**ZNF263**	-0.04	8.14E-01	1.00E+00	-0.03	9.54E-01	9.93E-01
**AC244093.4**	-1.44	5.34E-01	1.00E+00	-0.09	9.54E-01	9.93E-01
**PRLR**	0.41	1.02E-01	1.00E+00	0.03	9.54E-01	9.93E-01
**SLC44A5**	0.49	2.70E-01	1.00E+00	0.05	9.54E-01	9.93E-01
**LNPEP**	-0.02	9.19E-01	1.00E+00	0.02	9.54E-01	9.93E-01
**METAP1D**	-0.65	1.17E-01	1.00E+00	-0.01	9.54E-01	9.93E-01
**TMEM145**	-2.16	7.16E-03	5.13E-01	0.09	9.54E-01	9.93E-01
**AC007773.1**	-2.40	1.97E-01	1.00E+00	0.05	9.54E-01	9.93E-01
**CNR1**	0.10	8.34E-01	1.00E+00	-0.05	9.54E-01	9.93E-01
**AP1S3**	-0.56	2.30E-01	1.00E+00	-0.03	9.55E-01	9.93E-01
**ALS2CL**	-0.32	3.38E-01	1.00E+00	-0.02	9.55E-01	9.93E-01

**PRRC1**	-0.09	6.48E-01	1.00E+00	-0.02	9.55E-01	9.93E-01
**GLDN**	0.41	2.76E-01	1.00E+00	0.03	9.55E-01	9.93E-01
**KCNE3**	0.22	7.34E-01	1.00E+00	0.03	9.55E-01	9.93E-01
**GIT2**	-0.03	9.00E-01	1.00E+00	-0.01	9.55E-01	9.93E-01
**G6796**	-2.41	6.08E-02	1.00E+00	0.04	9.55E-01	9.93E-01
**DUSP9**	-0.04	9.51E-01	1.00E+00	-0.07	9.55E-01	9.93E-01
**PRPS1P2**	0.56	5.47E-01	1.00E+00	-0.04	9.55E-01	9.93E-01
**IFRD1**	0.45	8.11E-02	1.00E+00	-0.01	9.55E-01	9.93E-01
**CDCA5**	-0.21	5.17E-01	1.00E+00	0.03	9.55E-01	9.94E-01
**RPS20P2**	NA	NA	NA	-0.05	9.55E-01	9.94E-01
**ARL2**	-0.04	8.67E-01	1.00E+00	-0.02	9.55E-01	9.94E-01
**CLU**	0.61	2.08E-01	1.00E+00	0.02	9.56E-01	9.94E-01
**TMCC2**	-0.67	1.02E-02	6.27E-01	0.03	9.56E-01	9.94E-01
**G5173**	0.10	9.32E-01	1.00E+00	-0.10	9.56E-01	9.94E-01
**TMSB10P1**	0.56	8.71E-01	1.00E+00	0.04	9.56E-01	9.94E-01
**RPL7P22**	0.66	8.49E-01	1.00E+00	0.06	9.56E-01	9.94E-01
**MAP2K2**	-0.17	4.49E-01	1.00E+00	0.02	9.56E-01	9.94E-01
**VAV3**	0.06	8.61E-01	1.00E+00	-0.03	9.56E-01	9.94E-01
**LRRC3B**	-1.50	2.09E-01	1.00E+00	0.09	9.56E-01	9.94E-01
**CNTD2**	2.13	1.60E-01	1.00E+00	0.05	9.56E-01	9.94E-01
**SFXN2**	-0.60	9.86E-02	1.00E+00	0.02	9.56E-01	9.94E-01
**AL139384.2**	0.48	4.53E-01	1.00E+00	-0.07	9.56E-01	9.94E-01
**MZF1**	-0.37	2.57E-01	1.00E+00	-0.02	9.56E-01	9.94E-01
**AL121821.1**	-1.11	5.06E-01	1.00E+00	0.06	9.56E-01	9.94E-01
**AL357078.1**	0.68	5.94E-01	1.00E+00	-0.05	9.56E-01	9.94E-01
**XLOC_008522**	-0.88	2.79E-01	1.00E+00	0.06	9.56E-01	9.94E-01
**KCNJ9**	-5.36	1.51E-06	5.44E-04	0.08	9.56E-01	9.94E-01
**SEMA3B-AS1**	-1.74	2.03E-01	1.00E+00	-0.06	9.56E-01	9.94E-01
**AL354733.2**	-0.94	7.73E-01	1.00E+00	0.06	9.56E-01	9.94E-01
**G3945**	-0.70	4.40E-01	1.00E+00	-0.04	9.57E-01	9.94E-01
**AL133338.2**	-0.96	7.37E-01	1.00E+00	-0.03	9.57E-01	9.94E-01
**AC025181.2**	0.03	9.56E-01	1.00E+00	-0.03	9.57E-01	9.94E-01

**TXNDC12**	0.32	3.70E-01	1.00E+00	-0.03	9.57E-01	9.94E-01
**LACTB**	0.04	8.72E-01	1.00E+00	-0.03	9.57E-01	9.94E-01
**FBXO4**	0.09	7.02E-01	1.00E+00	-0.01	9.57E-01	9.94E-01
**AC022613.1**	-2.90	9.00E-02	1.00E+00	0.07	9.57E-01	9.94E-01
**KDM3A**	-0.10	7.49E-01	1.00E+00	-0.02	9.57E-01	9.94E-01
**LRRC19**	-1.38	5.57E-01	1.00E+00	-0.07	9.57E-01	9.94E-01
**G10908**	-2.02	2.13E-01	1.00E+00	0.05	9.57E-01	9.94E-01
**MAP2K5**	-0.12	6.66E-01	1.00E+00	0.01	9.57E-01	9.94E-01
**PSMD9**	-0.65	1.32E-01	1.00E+00	-0.02	9.57E-01	9.94E-01
**CKAP2L**	-0.27	5.34E-01	1.00E+00	0.03	9.57E-01	9.94E-01
**ARFGEF2**	0.42	1.51E-02	7.65E-01	0.02	9.57E-01	9.94E-01
**ZNF155**	0.12	7.51E-01	1.00E+00	0.02	9.57E-01	9.94E-01
**PUM2**	0.08	6.72E-01	1.00E+00	-0.02	9.57E-01	9.94E-01
**LINC01411**	-1.75	2.70E-01	1.00E+00	0.06	9.57E-01	9.94E-01
**UBE2D3P1**	-0.22	9.49E-01	1.00E+00	-0.03	9.57E-01	9.94E-01
**POLR2J4**	-0.65	8.00E-02	1.00E+00	-0.02	9.57E-01	9.94E-01
**IGFL1P1**	-0.10	9.23E-01	1.00E+00	-0.05	9.57E-01	9.94E-01
**ZNF384**	-0.28	9.47E-02	1.00E+00	-0.03	9.57E-01	9.94E-01
**STK32A**	0.58	4.58E-01	1.00E+00	0.03	9.58E-01	9.94E-01
**DDX12P**	-0.97	6.99E-02	1.00E+00	0.05	9.58E-01	9.94E-01
**SCUBE3**	-0.69	3.36E-01	1.00E+00	-0.04	9.58E-01	9.94E-01
**FALEC**	0.42	5.65E-01	1.00E+00	-0.04	9.58E-01	9.94E-01
**CLASP1**	-0.10	6.09E-01	1.00E+00	-0.02	9.58E-01	9.94E-01
**AC005962.1**	-0.29	8.43E-01	1.00E+00	0.06	9.58E-01	9.94E-01
**SLC16A13**	0.09	8.24E-01	1.00E+00	0.03	9.58E-01	9.94E-01
**TSPAN33**	-0.02	9.38E-01	1.00E+00	-0.04	9.58E-01	9.94E-01
**LINC01960**	1.08	1.16E-01	1.00E+00	0.05	9.58E-01	9.94E-01
**TBX5-AS1**	1.62	4.23E-02	1.00E+00	0.15	9.58E-01	9.94E-01
**SLC19A2**	0.13	7.83E-01	1.00E+00	0.02	9.58E-01	9.94E-01
**PPM1G**	-0.39	8.49E-02	1.00E+00	-0.01	9.58E-01	9.94E-01
**NKX1-2**	-0.27	6.50E-01	1.00E+00	-0.06	9.58E-01	9.94E-01
**MRI1**	-0.65	1.00E-01	1.00E+00	0.02	9.58E-01	9.94E-01

**AC036108.3**	-0.73	4.90E-01	1.00E+00	0.07	9.58E-01	9.94E-01
**RPL35A**	-0.04	8.93E-01	1.00E+00	0.01	9.58E-01	9.94E-01
**METTL7B**	3.85	1.02E-01	1.00E+00	0.05	9.58E-01	9.94E-01
**AC104695.2**	-1.66	1.56E-01	1.00E+00	0.04	9.58E-01	9.94E-01
**ARHGAP27P1-BPTFP1-KPNA2P3**	-1.24	6.54E-01	1.00E+00	-0.04	9.58E-01	9.94E-01
**NHLH1**	-0.59	6.28E-01	1.00E+00	-0.05	9.58E-01	9.94E-01
**BEND5**	0.20	6.63E-01	1.00E+00	0.02	9.58E-01	9.94E-01
**AK5**	-1.17	2.89E-02	9.88E-01	-0.03	9.58E-01	9.94E-01
**SNAP29**	0.00	9.96E-01	1.00E+00	-0.01	9.58E-01	9.94E-01
**AL365356.3**	-2.79	2.32E-03	2.43E-01	-0.05	9.59E-01	9.94E-01
**FHIT**	0.37	3.26E-01	1.00E+00	0.02	9.59E-01	9.94E-01
**ANKLE2**	-0.11	6.03E-01	1.00E+00	-0.02	9.59E-01	9.94E-01
**AC093014.1**	0.73	8.33E-01	1.00E+00	0.04	9.59E-01	9.94E-01
**KRT1**	-0.08	8.53E-01	1.00E+00	-0.04	9.59E-01	9.94E-01
**POLR1D**	0.02	9.20E-01	1.00E+00	-0.01	9.59E-01	9.94E-01
**AC108860.2**	-0.89	2.82E-01	1.00E+00	-0.04	9.59E-01	9.94E-01
**G18696**	1.71	2.40E-01	1.00E+00	-0.06	9.59E-01	9.94E-01
**SP5**	0.79	1.39E-01	1.00E+00	-0.04	9.59E-01	9.94E-01
**ALDH18A1**	-0.05	7.81E-01	1.00E+00	0.01	9.59E-01	9.94E-01
**ATP8B5P**	1.55	5.43E-01	1.00E+00	-0.04	9.59E-01	9.94E-01
**SSBP3-AS1**	0.08	9.37E-01	1.00E+00	0.04	9.59E-01	9.94E-01
**DCAF12L2**	0.51	4.26E-01	1.00E+00	0.06	9.59E-01	9.94E-01
**SAP130**	-0.38	1.53E-01	1.00E+00	-0.02	9.59E-01	9.94E-01
**ARHGAP10**	0.26	1.27E-01	1.00E+00	0.01	9.59E-01	9.94E-01
**P2RY14**	-0.11	7.87E-01	1.00E+00	-0.02	9.59E-01	9.94E-01
**KCTD6**	-0.01	9.84E-01	1.00E+00	-0.02	9.59E-01	9.94E-01
**IQCN**	-0.95	5.06E-02	1.00E+00	-0.04	9.59E-01	9.94E-01
**MCRIP2**	0.13	7.97E-01	1.00E+00	0.03	9.59E-01	9.94E-01
**GAS5-AS1**	-0.13	8.83E-01	1.00E+00	-0.03	9.59E-01	9.94E-01
**RAB25**	-0.06	8.75E-01	1.00E+00	0.03	9.59E-01	9.94E-01
**AC098851.1**	-0.36	8.99E-01	1.00E+00	-0.06	9.60E-01	9.94E-01
**SERPINB12**	0.06	9.35E-01	1.00E+00	0.03	9.60E-01	9.94E-01

**AC096921.2**	-0.17	7.98E-01	1.00E+00	0.05	9.60E-01	9.94E-01
**G26459**	-1.47	1.62E-01	1.00E+00	0.04	9.60E-01	9.94E-01
**ZNF484**	-0.19	6.11E-01	1.00E+00	0.02	9.60E-01	9.94E-01
**UBE2T**	-0.07	8.42E-01	1.00E+00	0.02	9.60E-01	9.94E-01
**XLOC_001188**	-1.03	2.41E-01	1.00E+00	-0.03	9.60E-01	9.94E-01
**BHLHE40**	-0.80	3.55E-02	1.00E+00	-0.02	9.60E-01	9.94E-01
**INTS9**	-0.06	7.49E-01	1.00E+00	-0.01	9.60E-01	9.94E-01
**AC104260.3**	-0.77	7.77E-01	1.00E+00	-0.07	9.60E-01	9.94E-01
**AL021408.1**	1.36	1.97E-01	1.00E+00	-0.15	9.60E-01	9.94E-01
**NUP37**	0.18	4.46E-01	1.00E+00	0.01	9.60E-01	9.94E-01
**RASSF7**	-0.37	3.66E-01	1.00E+00	-0.02	9.60E-01	9.94E-01
**LRRC17**	0.65	1.57E-01	1.00E+00	0.02	9.60E-01	9.94E-01
**ZNF493**	-0.11	8.11E-01	1.00E+00	0.02	9.60E-01	9.94E-01
**LMBRD2**	-0.02	9.51E-01	1.00E+00	-0.01	9.60E-01	9.94E-01
**ANKZF1**	0.29	3.95E-01	1.00E+00	0.02	9.60E-01	9.94E-01
**MGMT**	0.01	9.77E-01	1.00E+00	0.01	9.60E-01	9.94E-01
**ARHGAP44**	-0.16	7.14E-01	1.00E+00	-0.03	9.61E-01	9.94E-01
**XLOC_001446**	-0.23	6.60E-01	1.00E+00	-0.04	9.61E-01	9.94E-01
**AL359918.1**	-0.58	8.67E-01	1.00E+00	-0.03	9.61E-01	9.94E-01
**FAM86C2P**	-0.63	3.03E-02	1.00E+00	-0.02	9.61E-01	9.94E-01
**AC009495.3**	1.74	5.51E-01	1.00E+00	-0.06	9.61E-01	9.94E-01
**VAMP1**	-0.48	3.54E-01	1.00E+00	0.03	9.61E-01	9.94E-01
**LINC00861**	3.38	1.31E-01	1.00E+00	-0.05	9.61E-01	9.94E-01
**HDHD2**	-0.85	2.90E-01	1.00E+00	-0.03	9.61E-01	9.94E-01
**AC090409.2**	0.90	7.96E-01	1.00E+00	-0.12	9.61E-01	9.94E-01
**PEF1**	0.05	7.82E-01	1.00E+00	0.01	9.61E-01	9.94E-01
**AC005332.4**	-0.49	9.47E-02	1.00E+00	-0.04	9.61E-01	9.94E-01
**FERMT2**	0.60	3.00E-01	1.00E+00	-0.02	9.61E-01	9.94E-01
**DEPTOR**	0.20	5.48E-01	1.00E+00	0.02	9.61E-01	9.94E-01
**DIRAS3**	-0.21	6.69E-01	1.00E+00	0.04	9.61E-01	9.94E-01
**SCNN1A**	-0.19	3.69E-01	1.00E+00	-0.02	9.61E-01	9.94E-01
**AGAP2-AS1**	-1.15	5.17E-01	1.00E+00	0.04	9.61E-01	9.94E-01

**AC099560.2**	0.02	9.68E-01	1.00E+00	-0.03	9.61E-01	9.94E-01
**PDZD8**	-0.22	1.90E-01	1.00E+00	-0.02	9.61E-01	9.94E-01
**FAM57A**	0.26	4.49E-01	1.00E+00	0.02	9.61E-01	9.94E-01
**AC111188.1**	2.03	1.90E-01	1.00E+00	0.07	9.61E-01	9.94E-01
**FCHSD2**	-0.06	8.17E-01	1.00E+00	-0.02	9.61E-01	9.94E-01
**PCYT1A**	0.18	3.70E-01	1.00E+00	0.01	9.61E-01	9.94E-01
**WDR76**	-0.13	5.50E-01	1.00E+00	0.01	9.61E-01	9.94E-01
**AC018816.1**	-0.02	9.89E-01	1.00E+00	-0.02	9.61E-01	9.94E-01
**PNMA6A**	-1.04	2.21E-01	1.00E+00	-0.06	9.61E-01	9.94E-01
**RFX3**	-0.10	7.42E-01	1.00E+00	0.02	9.62E-01	9.95E-01
**GDAP1**	-0.32	3.24E-01	1.00E+00	-0.02	9.62E-01	9.95E-01
**TMEM150C**	0.30	4.81E-01	1.00E+00	-0.02	9.62E-01	9.95E-01
**FOXM1**	-0.65	1.53E-01	1.00E+00	0.03	9.62E-01	9.95E-01
**NUP160**	0.23	3.07E-01	1.00E+00	0.01	9.62E-01	9.95E-01
**WNT11**	0.25	4.77E-01	1.00E+00	-0.12	9.62E-01	9.95E-01
**GRK4**	-0.01	9.74E-01	1.00E+00	-0.03	9.62E-01	9.95E-01
**TCHP**	0.05	8.85E-01	1.00E+00	-0.02	9.62E-01	9.95E-01
**ZNF582-AS1**	0.23	8.28E-01	1.00E+00	0.03	9.62E-01	9.95E-01
**DAPL1**	0.05	9.15E-01	1.00E+00	-0.03	9.62E-01	9.95E-01
**AL590326.1**	0.33	6.85E-01	1.00E+00	0.06	9.62E-01	9.95E-01
**PCDHB18P**	-0.56	2.37E-01	1.00E+00	-0.04	9.63E-01	9.95E-01
**NDUFB7**	-0.04	8.44E-01	1.00E+00	0.01	9.63E-01	9.95E-01
**AC093525.7**	1.49	3.36E-01	1.00E+00	0.08	9.63E-01	9.95E-01
**XLOC_005252**	-0.35	7.65E-01	1.00E+00	0.04	9.63E-01	9.95E-01
**CERS1**	-0.84	4.31E-01	1.00E+00	0.07	9.63E-01	9.95E-01
**TUBGCP6**	-0.48	2.24E-02	9.00E-01	-0.03	9.63E-01	9.95E-01
**FAM102A**	-0.20	4.41E-01	1.00E+00	0.02	9.63E-01	9.95E-01
**LEMD2**	0.05	8.02E-01	1.00E+00	0.03	9.63E-01	9.95E-01
**RBPJ**	0.46	6.25E-02	1.00E+00	-0.01	9.63E-01	9.95E-01
**AL078621.1**	0.84	6.80E-01	1.00E+00	-0.03	9.63E-01	9.95E-01
**CCDC96**	-0.03	9.11E-01	1.00E+00	0.03	9.63E-01	9.95E-01
**BARX1**	-3.01	4.01E-02	1.00E+00	-0.08	9.63E-01	9.95E-01

**AC012213.3**	-1.20	7.25E-01	1.00E+00	0.13	9.63E-01	9.95E-01
**WFDC5**	0.36	4.23E-01	1.00E+00	0.03	9.63E-01	9.95E-01
**LARP7**	0.05	8.53E-01	1.00E+00	0.01	9.63E-01	9.95E-01
**CARD10**	0.22	5.90E-01	1.00E+00	0.02	9.63E-01	9.95E-01
**NUP153**	-0.20	3.69E-01	1.00E+00	0.02	9.64E-01	9.95E-01
**AP5S1**	0.12	7.40E-01	1.00E+00	-0.01	9.64E-01	9.95E-01
**KEAP1**	-0.04	8.24E-01	1.00E+00	0.01	9.64E-01	9.95E-01
**DIRAS1**	-0.72	1.55E-01	1.00E+00	-0.03	9.64E-01	9.95E-01
**XLOC_009067**	-0.53	5.30E-01	1.00E+00	-0.05	9.64E-01	9.95E-01
**CASC15**	-0.36	5.30E-01	1.00E+00	0.02	9.64E-01	9.95E-01
**OXTR**	-2.30	1.44E-02	7.56E-01	-0.05	9.64E-01	9.95E-01
**CPSF4**	0.30	1.98E-01	1.00E+00	-0.02	9.64E-01	9.95E-01
**AL589880.1**	-0.17	8.70E-01	1.00E+00	0.03	9.64E-01	9.95E-01
**TRAPPC12-AS1**	-0.58	4.75E-01	1.00E+00	-0.04	9.64E-01	9.95E-01
**ZBTB7A**	-0.24	4.18E-01	1.00E+00	0.03	9.64E-01	9.95E-01
**C8orf59**	-1.07	9.85E-02	1.00E+00	0.02	9.64E-01	9.95E-01
**AL670729.1**	-2.05	1.29E-01	1.00E+00	-0.03	9.64E-01	9.95E-01
**AC108477.1**	-1.35	7.47E-02	1.00E+00	0.05	9.64E-01	9.95E-01
**OFD1**	0.01	9.75E-01	1.00E+00	0.01	9.64E-01	9.95E-01
**PMP22**	0.41	2.40E-01	1.00E+00	0.01	9.64E-01	9.95E-01
**AC016924.1**	2.54	7.42E-02	1.00E+00	-0.05	9.64E-01	9.95E-01
**AC005838.2**	0.48	5.64E-01	1.00E+00	0.04	9.64E-01	9.95E-01
**GRM4**	-0.84	4.05E-01	1.00E+00	0.06	9.64E-01	9.95E-01
**RPS10P3**	-0.86	2.85E-01	1.00E+00	0.03	9.64E-01	9.95E-01
**G35821**	0.09	8.80E-01	1.00E+00	-0.03	9.64E-01	9.95E-01
**HPX**	-0.55	7.07E-01	1.00E+00	-0.07	9.64E-01	9.95E-01
**ITGB7**	-0.23	6.41E-01	1.00E+00	-0.13	9.64E-01	9.95E-01
**WNT5B**	-0.33	3.49E-01	1.00E+00	0.02	9.64E-01	9.95E-01
**SAMD13**	-0.47	5.41E-01	1.00E+00	-0.04	9.64E-01	9.95E-01
**MAP1LC3B2**	-0.44	6.52E-01	1.00E+00	0.02	9.64E-01	9.95E-01
**AC010654.1**	-0.56	5.98E-01	1.00E+00	0.03	9.65E-01	9.95E-01
**XLOC_010730**	5.36	9.35E-02	1.00E+00	-0.05	9.65E-01	9.96E-01

**REPIN1**	-0.11	6.78E-01	1.00E+00	-0.03	9.65E-01	9.96E-01
**UBAP2**	-0.27	3.02E-01	1.00E+00	0.01	9.65E-01	9.96E-01
**AC019080.1**	0.43	3.16E-01	1.00E+00	0.02	9.65E-01	9.96E-01
**C2CD2L**	-0.26	4.08E-01	1.00E+00	0.02	9.65E-01	9.96E-01
**ALG12**	-0.10	6.78E-01	1.00E+00	0.01	9.65E-01	9.96E-01
**FOXO3B**	-0.42	2.57E-01	1.00E+00	-0.04	9.65E-01	9.96E-01
**AL033543.1**	0.14	9.15E-01	1.00E+00	-0.03	9.65E-01	9.96E-01
**DDAH2**	0.38	2.57E-01	1.00E+00	0.01	9.65E-01	9.96E-01
**CHRNE**	-0.34	6.94E-01	1.00E+00	0.02	9.65E-01	9.96E-01
**G26207**	-2.58	1.13E-01	1.00E+00	0.05	9.65E-01	9.96E-01
**DHRS7**	-0.03	9.00E-01	1.00E+00	-0.01	9.65E-01	9.96E-01
**DPY19L1**	0.20	5.40E-01	1.00E+00	0.02	9.65E-01	9.96E-01
**SURF4**	0.25	2.17E-01	1.00E+00	0.01	9.65E-01	9.96E-01
**AL132657.1**	-0.53	6.86E-01	1.00E+00	0.04	9.65E-01	9.96E-01
**CBLL1**	-0.12	5.02E-01	1.00E+00	0.01	9.66E-01	9.96E-01
**TMEM184C**	0.29	6.39E-02	1.00E+00	-0.01	9.66E-01	9.96E-01
**TMEM63C**	0.05	9.41E-01	1.00E+00	-0.13	9.66E-01	9.96E-01
**TSC2**	-0.25	3.76E-01	1.00E+00	0.02	9.66E-01	9.96E-01
**RPL4P4**	-0.15	7.47E-01	1.00E+00	0.03	9.66E-01	9.96E-01
**LRRC75A**	-0.70	2.26E-01	1.00E+00	0.04	9.66E-01	9.96E-01
**INPP5F**	-0.01	9.74E-01	1.00E+00	-0.01	9.66E-01	9.96E-01
**BMP3**	1.01	8.13E-02	1.00E+00	-0.10	9.66E-01	9.96E-01
**IL19**	-1.28	7.10E-01	1.00E+00	-0.14	9.66E-01	9.96E-01
**NXPH3**	0.32	5.10E-01	1.00E+00	0.03	9.66E-01	9.96E-01
**G10060**	-0.21	8.08E-01	1.00E+00	-0.04	9.66E-01	9.96E-01
**SPINT2**	0.04	8.87E-01	1.00E+00	0.02	9.66E-01	9.96E-01
**SNAI3-AS1**	-0.11	8.60E-01	1.00E+00	-0.03	9.66E-01	9.96E-01
**SNTB1**	0.11	7.16E-01	1.00E+00	-0.02	9.66E-01	9.96E-01
**AC131254.1**	-1.97	5.67E-01	1.00E+00	0.13	9.66E-01	9.96E-01
**TBX4**	0.64	5.45E-01	1.00E+00	-0.13	9.66E-01	9.96E-01
**CCT2**	0.05	8.21E-01	1.00E+00	0.01	9.66E-01	9.96E-01
**ENOX1**	-0.17	7.25E-01	1.00E+00	0.03	9.66E-01	9.96E-01

**TMEM125**	-0.02	9.63E-01	1.00E+00	-0.02	9.66E-01	9.96E-01
**SLC52A2**	-0.08	7.66E-01	1.00E+00	-0.02	9.66E-01	9.96E-01
**SYT12**	-0.81	1.73E-01	1.00E+00	0.04	9.66E-01	9.96E-01
**ZCWPW1**	-0.12	6.62E-01	1.00E+00	0.01	9.66E-01	9.96E-01
**TUBG1**	-0.11	5.98E-01	1.00E+00	-0.01	9.66E-01	9.96E-01
**RBM12B**	-0.04	9.06E-01	1.00E+00	0.02	9.66E-01	9.96E-01
**OSBPL10**	0.08	7.17E-01	1.00E+00	0.02	9.67E-01	9.96E-01
**TIGD2**	0.09	7.74E-01	1.00E+00	-0.01	9.67E-01	9.96E-01
**AC002116.2**	-1.64	4.54E-01	1.00E+00	-0.03	9.67E-01	9.96E-01
**FNDC5**	0.40	6.30E-01	1.00E+00	0.03	9.67E-01	9.96E-01
**FAM192A**	-0.05	7.73E-01	1.00E+00	-0.01	9.67E-01	9.96E-01
**CRELD2**	-0.25	3.06E-01	1.00E+00	0.01	9.67E-01	9.96E-01
**PPARGC1B**	-0.04	8.98E-01	1.00E+00	0.02	9.67E-01	9.96E-01
**RPS15AP1**	0.09	9.44E-01	1.00E+00	0.03	9.67E-01	9.96E-01
**MT1DP**	-2.88	2.60E-01	1.00E+00	0.05	9.67E-01	9.96E-01
**RPL7P44**	NA	NA	NA	-0.04	9.67E-01	9.96E-01
**G32188**	-0.05	9.36E-01	1.00E+00	0.05	9.67E-01	9.96E-01
**LINC02362**	0.38	9.11E-01	1.00E+00	-0.07	9.67E-01	9.96E-01
**ERBB2**	-0.04	8.80E-01	1.00E+00	-0.02	9.67E-01	9.96E-01
**KDM4C**	-0.08	7.78E-01	1.00E+00	-0.01	9.67E-01	9.96E-01
**AC138035.1**	-4.08	8.32E-02	1.00E+00	-0.05	9.67E-01	9.96E-01
**AC018462.1**	0.59	5.31E-01	1.00E+00	-0.04	9.67E-01	9.96E-01
**E2F4**	-0.26	3.25E-01	1.00E+00	0.02	9.67E-01	9.96E-01
**SYT5**	-1.09	3.75E-01	1.00E+00	-0.06	9.68E-01	9.96E-01
**RPL39**	-0.13	6.67E-01	1.00E+00	-0.01	9.68E-01	9.96E-01
**THOC3**	-0.06	7.95E-01	1.00E+00	0.01	9.68E-01	9.96E-01
**ADCY5**	0.58	4.44E-01	1.00E+00	-0.03	9.68E-01	9.96E-01
**RBMS3**	0.53	2.98E-01	1.00E+00	0.02	9.68E-01	9.96E-01
**ZNF415**	0.32	5.58E-01	1.00E+00	-0.02	9.68E-01	9.96E-01
**PSMB7**	-0.14	4.50E-01	1.00E+00	-0.01	9.68E-01	9.96E-01
**CCDC140**	-1.03	2.56E-01	1.00E+00	0.05	9.68E-01	9.96E-01
**CC2D2A**	0.29	4.40E-01	1.00E+00	-0.02	9.68E-01	9.96E-01

**WLS**	0.10	6.34E-01	1.00E+00	-0.01	9.68E-01	9.96E-01
**PRTG**	-0.26	7.01E-01	1.00E+00	-0.03	9.68E-01	9.96E-01
**AC004980.2**	-0.21	8.55E-01	1.00E+00	-0.05	9.68E-01	9.96E-01
**FAM91A1**	0.41	1.00E-01	1.00E+00	0.01	9.68E-01	9.96E-01
**MYCT1**	0.50	4.01E-01	1.00E+00	-0.02	9.68E-01	9.96E-01
**TMEM259**	-0.04	8.32E-01	1.00E+00	-0.03	9.68E-01	9.96E-01
**RFTN2**	0.38	4.36E-01	1.00E+00	-0.02	9.68E-01	9.96E-01
**RPS6P25**	0.58	8.66E-01	1.00E+00	0.04	9.68E-01	9.96E-01
**DYDC2**	-2.81	3.53E-02	1.00E+00	-0.05	9.68E-01	9.96E-01
**IDH3G**	-0.27	1.97E-01	1.00E+00	0.01	9.68E-01	9.96E-01
**ERI1**	0.30	3.43E-01	1.00E+00	-0.01	9.68E-01	9.96E-01
**AL591848.3**	-1.02	6.06E-01	1.00E+00	-0.06	9.69E-01	9.96E-01
**TRPC4AP**	0.30	2.35E-01	1.00E+00	0.01	9.69E-01	9.96E-01
**GPR87**	0.39	2.94E-01	1.00E+00	0.03	9.69E-01	9.96E-01
**MEOX2**	0.47	3.58E-01	1.00E+00	-0.02	9.69E-01	9.96E-01
**SLC6A4**	1.74	2.34E-02	9.15E-01	0.03	9.69E-01	9.96E-01
**HEMK1**	-0.38	7.45E-02	1.00E+00	0.01	9.69E-01	9.96E-01
**AL451042.1**	-0.26	8.18E-01	1.00E+00	0.03	9.69E-01	9.96E-01
**SPG7**	0.08	6.80E-01	1.00E+00	-0.02	9.69E-01	9.96E-01
**A2MP1**	-0.39	8.59E-01	1.00E+00	0.05	9.69E-01	9.96E-01
**C1orf35**	-0.03	8.96E-01	1.00E+00	0.01	9.69E-01	9.96E-01
**ATP10B**	-0.26	6.54E-01	1.00E+00	0.03	9.69E-01	9.96E-01
**HSPB6**	1.63	6.19E-02	1.00E+00	-0.03	9.69E-01	9.96E-01
**SALL2**	0.08	8.38E-01	1.00E+00	0.03	9.69E-01	9.96E-01
**AC072022.2**	0.30	8.09E-01	1.00E+00	0.03	9.69E-01	9.96E-01
**RPL23P8**	-0.68	8.44E-01	1.00E+00	0.03	9.69E-01	9.96E-01
**GABARAPL1**	0.28	4.88E-01	1.00E+00	0.01	9.69E-01	9.96E-01
**KIF3A**	-0.36	1.61E-01	1.00E+00	-0.01	9.69E-01	9.96E-01
**ZNF48**	-0.32	1.66E-01	1.00E+00	0.02	9.69E-01	9.96E-01
**TBX5**	1.15	1.76E-01	1.00E+00	-0.12	9.69E-01	9.96E-01
**G18244**	0.07	8.81E-01	1.00E+00	0.02	9.69E-01	9.96E-01
**WDFY3-AS2**	0.66	6.38E-02	1.00E+00	0.02	9.69E-01	9.96E-01

**RC3H2**	0.05	7.99E-01	1.00E+00	0.01	9.69E-01	9.96E-01
**MTMR10**	0.09	6.83E-01	1.00E+00	-0.01	9.69E-01	9.96E-01
**MTND5P11**	-0.23	9.47E-01	1.00E+00	-0.02	9.70E-01	9.96E-01
**DOP1A**	0.02	9.41E-01	1.00E+00	0.01	9.70E-01	9.96E-01
**LINC00431**	-3.68	3.90E-02	1.00E+00	-0.05	9.70E-01	9.96E-01
**LINC00997**	0.07	9.08E-01	1.00E+00	-0.03	9.70E-01	9.96E-01
**CDKN2AIP**	0.25	2.70E-01	1.00E+00	-0.01	9.70E-01	9.96E-01
**GRIN1**	-1.35	4.38E-02	1.00E+00	-0.03	9.70E-01	9.96E-01
**CASC8**	-1.01	4.21E-01	1.00E+00	-0.03	9.70E-01	9.96E-01
**TMEM50A**	0.16	3.63E-01	1.00E+00	0.01	9.70E-01	9.96E-01
**KIF11**	-0.06	8.69E-01	1.00E+00	0.02	9.70E-01	9.96E-01
**PSMA6P1**	0.60	6.34E-01	1.00E+00	-0.02	9.70E-01	9.96E-01
**BCL10**	-0.06	7.43E-01	1.00E+00	-0.01	9.70E-01	9.96E-01
**AVPR1A**	0.22	6.72E-01	1.00E+00	-0.03	9.70E-01	9.96E-01
**SPRTN**	-0.09	7.46E-01	1.00E+00	0.01	9.70E-01	9.96E-01
**GPRIN1**	-1.45	2.36E-02	9.15E-01	-0.12	9.70E-01	9.96E-01
**YBX1P1**	-0.39	3.45E-01	1.00E+00	-0.02	9.70E-01	9.96E-01
**EMC8**	0.02	9.09E-01	1.00E+00	0.01	9.70E-01	9.96E-01
**KBTBD11**	0.05	9.15E-01	1.00E+00	0.08	9.70E-01	9.96E-01
**ZFP69**	-0.25	4.68E-01	1.00E+00	0.02	9.70E-01	9.96E-01
**STEAP3-AS1**	-2.05	5.52E-01	1.00E+00	-0.05	9.70E-01	9.96E-01
**DENND6A-DT**	-2.12	5.36E-01	1.00E+00	0.12	9.70E-01	9.96E-01
**RP6-206I17.2**	3.55	9.07E-02	1.00E+00	-0.03	9.70E-01	9.96E-01
**WDHD1**	-0.17	6.00E-01	1.00E+00	0.01	9.71E-01	9.96E-01
**GRM3**	-1.70	2.63E-01	1.00E+00	-0.03	9.71E-01	9.96E-01
**LINC02449**	-0.98	5.42E-01	1.00E+00	-0.04	9.71E-01	9.96E-01
**TMEM225B**	-2.81	1.02E-01	1.00E+00	-0.02	9.71E-01	9.96E-01
**EZH2**	-0.20	5.83E-01	1.00E+00	-0.02	9.71E-01	9.96E-01
**LGALS8**	-0.03	9.12E-01	1.00E+00	0.01	9.71E-01	9.96E-01
**ABHD12**	-0.02	9.31E-01	1.00E+00	0.01	9.71E-01	9.96E-01
**AC135050.6**	-0.05	8.94E-01	1.00E+00	-0.02	9.71E-01	9.96E-01
**PKP2**	0.18	6.76E-01	1.00E+00	-0.02	9.71E-01	9.96E-01

**VCL**	-0.18	4.42E-01	1.00E+00	0.01	9.71E-01	9.96E-01
**ERMARD**	0.12	7.26E-01	1.00E+00	-0.01	9.71E-01	9.96E-01
**ATP10D**	0.00	9.92E-01	1.00E+00	-0.01	9.71E-01	9.96E-01
**SPATA6**	0.16	5.43E-01	1.00E+00	-0.01	9.71E-01	9.96E-01
**RBM33**	-0.18	5.36E-01	1.00E+00	-0.01	9.71E-01	9.96E-01
**CCDC69**	0.75	1.70E-01	1.00E+00	-0.03	9.71E-01	9.96E-01
**TTYH1**	-0.08	9.03E-01	1.00E+00	-0.02	9.71E-01	9.96E-01
**RPS4XP8**	NA	NA	NA	0.11	9.71E-01	9.96E-01
**MSANTD4**	0.24	5.09E-01	1.00E+00	-0.01	9.71E-01	9.96E-01
**ZDBF2**	0.08	8.11E-01	1.00E+00	-0.02	9.71E-01	9.97E-01
**USP9Y**	0.25	6.81E-01	1.00E+00	0.03	9.71E-01	9.97E-01
**CHAF1B**	-0.27	2.19E-01	1.00E+00	0.01	9.71E-01	9.97E-01
**L2HGDH**	0.03	9.40E-01	1.00E+00	-0.01	9.71E-01	9.97E-01
**GEMIN6**	0.15	5.78E-01	1.00E+00	0.01	9.71E-01	9.97E-01
**AC020915.3**	0.34	5.94E-01	1.00E+00	0.02	9.71E-01	9.97E-01
**EXOC8**	-0.28	2.60E-01	1.00E+00	-0.01	9.72E-01	9.97E-01
**SRL**	0.10	8.80E-01	1.00E+00	-0.03	9.72E-01	9.97E-01
**BX649597.1**	-2.02	2.46E-01	1.00E+00	-0.04	9.72E-01	9.97E-01
**AL672207.1**	-0.49	7.07E-01	1.00E+00	-0.02	9.72E-01	9.97E-01
**AC107464.3**	1.47	3.10E-01	1.00E+00	0.04	9.72E-01	9.97E-01
**CYSLTR1**	0.59	3.51E-01	1.00E+00	0.01	9.72E-01	9.97E-01
**ITGBL1**	0.56	2.78E-01	1.00E+00	-0.02	9.72E-01	9.97E-01
**AC007950.2**	-0.07	9.85E-01	1.00E+00	-0.05	9.72E-01	9.97E-01
**CYTH3**	0.09	7.12E-01	1.00E+00	0.02	9.72E-01	9.97E-01
**CBARP**	-0.08	8.80E-01	1.00E+00	-0.11	9.72E-01	9.97E-01
**HMBS**	-0.30	2.16E-01	1.00E+00	0.01	9.72E-01	9.97E-01
**ITPKA**	-1.68	1.45E-02	7.58E-01	0.04	9.72E-01	9.97E-01
**GSTA8P**	1.53	2.07E-01	1.00E+00	-0.04	9.72E-01	9.97E-01
**TCAF1P1**	-0.03	9.30E-01	1.00E+00	-0.02	9.72E-01	9.97E-01
**CDC42BPG**	-0.25	4.85E-01	1.00E+00	0.03	9.72E-01	9.97E-01
**SNRPD1**	-0.07	7.19E-01	1.00E+00	-0.01	9.72E-01	9.97E-01
**INSL3**	-2.52	2.76E-01	1.00E+00	0.05	9.72E-01	9.97E-01

**MAP10**	0.86	4.68E-03	3.94E-01	-0.02	9.72E-01	9.97E-01
**AC007240.2**	-1.36	5.91E-01	1.00E+00	-0.04	9.72E-01	9.97E-01
**CPNE4**	-1.81	4.23E-03	3.66E-01	-0.05	9.73E-01	9.97E-01
**EXOC7**	-0.15	5.26E-01	1.00E+00	-0.01	9.73E-01	9.97E-01
**HMGCS1**	-0.49	2.96E-01	1.00E+00	-0.02	9.73E-01	9.97E-01
**AC026401.3**	-0.30	3.45E-01	1.00E+00	-0.02	9.73E-01	9.97E-01
**MTF2**	0.08	7.31E-01	1.00E+00	-0.01	9.73E-01	9.97E-01
**ATP8B1**	0.17	3.83E-01	1.00E+00	-0.01	9.73E-01	9.97E-01
**AL590235.1**	1.20	4.18E-01	1.00E+00	-0.03	9.73E-01	9.97E-01
**SLC2A4RG**	-0.15	4.48E-01	1.00E+00	-0.02	9.73E-01	9.97E-01
**AC124947.1**	1.03	4.15E-01	1.00E+00	0.03	9.73E-01	9.97E-01
**PALM2**	0.43	6.41E-01	1.00E+00	0.03	9.73E-01	9.97E-01
**TPBGL**	-3.64	2.71E-04	6.07E-02	-0.04	9.73E-01	9.97E-01
**HID1**	0.07	9.00E-01	1.00E+00	-0.01	9.73E-01	9.97E-01
**AC004540.2**	-3.19	3.12E-02	1.00E+00	-0.03	9.73E-01	9.97E-01
**LURAP1L**	-0.11	6.92E-01	1.00E+00	-0.01	9.73E-01	9.97E-01
**AC087045.2**	-0.54	8.29E-01	1.00E+00	-0.04	9.73E-01	9.97E-01
**SMG1P7**	0.71	5.17E-01	1.00E+00	0.04	9.73E-01	9.97E-01
**LBHD2**	2.70	1.28E-01	1.00E+00	-0.04	9.73E-01	9.97E-01
**LINC01300**	-0.05	9.54E-01	1.00E+00	-0.03	9.73E-01	9.97E-01
**SRSF5**	-0.51	2.15E-01	1.00E+00	0.01	9.73E-01	9.97E-01
**AMIGO1**	0.40	3.43E-01	1.00E+00	0.02	9.73E-01	9.97E-01
**FAM71E1**	-0.34	5.79E-01	1.00E+00	0.02	9.73E-01	9.97E-01
**C1orf61**	-6.38	5.98E-02	1.00E+00	-0.04	9.73E-01	9.97E-01
**LRFN1**	-0.83	3.12E-02	1.00E+00	-0.03	9.73E-01	9.97E-01
**FAM50B**	0.10	6.74E-01	1.00E+00	0.01	9.73E-01	9.97E-01
**GCGR**	-0.52	5.72E-01	1.00E+00	0.04	9.73E-01	9.97E-01
**NOS3**	-0.15	6.88E-01	1.00E+00	0.03	9.73E-01	9.97E-01
**ZNF107**	-0.05	8.97E-01	1.00E+00	0.01	9.74E-01	9.97E-01
**CENPI**	0.72	2.41E-01	1.00E+00	0.03	9.74E-01	9.97E-01
**G19311**	-2.84	3.76E-01	1.00E+00	-0.04	9.74E-01	9.97E-01
**HOXA1**	-0.35	2.94E-01	1.00E+00	-0.02	9.74E-01	9.97E-01

**ETV6**	-0.22	3.05E-01	1.00E+00	0.01	9.74E-01	9.97E-01
**AC006329.1**	0.19	7.43E-01	1.00E+00	-0.02	9.74E-01	9.97E-01
**GAL3ST1**	-0.74	2.62E-01	1.00E+00	-0.03	9.74E-01	9.97E-01
**AL049552.1**	1.41	3.80E-01	1.00E+00	-0.10	9.74E-01	9.97E-01
**RAB23**	-0.01	9.82E-01	1.00E+00	0.01	9.74E-01	9.97E-01
**EXOSC4**	0.06	8.01E-01	1.00E+00	-0.01	9.74E-01	9.97E-01
**TNXB**	0.38	4.03E-01	1.00E+00	0.03	9.74E-01	9.97E-01
**APBB1**	-0.11	7.50E-01	1.00E+00	0.01	9.74E-01	9.97E-01
**AC106886.5**	0.20	7.39E-01	1.00E+00	0.02	9.74E-01	9.97E-01
**AC010422.1**	0.31	7.89E-01	1.00E+00	-0.03	9.74E-01	9.97E-01
**KRTAP2-3**	-6.16	7.65E-02	1.00E+00	-0.10	9.74E-01	9.97E-01
**LINC02159**	-5.67	2.80E-03	2.73E-01	0.10	9.74E-01	9.97E-01
**G41275**	-0.30	6.18E-01	1.00E+00	0.02	9.74E-01	9.97E-01
**ELAVL2**	-2.07	1.50E-02	7.62E-01	0.04	9.74E-01	9.97E-01
**LHX2**	-1.16	4.62E-01	1.00E+00	-0.10	9.74E-01	9.97E-01
**AC027279.1**	-0.81	6.50E-01	1.00E+00	0.02	9.74E-01	9.97E-01
**KCNK7**	0.15	7.58E-01	1.00E+00	-0.02	9.74E-01	9.97E-01
**AC006213.6**	-2.09	1.61E-01	1.00E+00	0.04	9.74E-01	9.97E-01
**ALOX12**	-0.27	6.21E-01	1.00E+00	-0.01	9.75E-01	9.97E-01
**TPK1**	0.54	1.20E-01	1.00E+00	0.02	9.75E-01	9.97E-01
**AC138089.1**	-2.49	2.66E-01	1.00E+00	0.04	9.75E-01	9.97E-01
**YWHAQ**	-0.15	3.90E-01	1.00E+00	-0.01	9.75E-01	9.97E-01
**LINC00278**	0.27	8.39E-01	1.00E+00	0.07	9.75E-01	9.97E-01
**RBMXL1**	-0.06	7.82E-01	1.00E+00	-0.01	9.75E-01	9.97E-01
**LCE2D**	1.12	1.26E-01	1.00E+00	0.02	9.75E-01	9.97E-01
**AC007342.4**	0.78	4.59E-01	1.00E+00	0.03	9.75E-01	9.97E-01
**LINC01589**	1.27	3.55E-01	1.00E+00	-0.04	9.75E-01	9.97E-01
**DDX11L10**	0.67	6.78E-01	1.00E+00	-0.03	9.75E-01	9.97E-01
**KRTAP10-10**	-6.29	7.06E-02	1.00E+00	-0.10	9.75E-01	9.97E-01
**TMEM127**	-0.06	6.99E-01	1.00E+00	-0.02	9.75E-01	9.97E-01
**TMPRSS5**	-0.67	3.86E-01	1.00E+00	-0.02	9.75E-01	9.97E-01
**CSPG4P12**	-0.04	9.49E-01	1.00E+00	0.03	9.75E-01	9.97E-01

**AGAP12P**	-1.13	4.54E-01	1.00E+00	-0.03	9.75E-01	9.97E-01
**PLEKHG6**	0.12	6.64E-01	1.00E+00	-0.02	9.75E-01	9.97E-01
**HLA-DQB2**	-1.25	1.45E-02	7.60E-01	0.02	9.75E-01	9.97E-01
**GJB4**	0.30	6.04E-01	1.00E+00	0.02	9.75E-01	9.97E-01
**PGM5P1**	0.60	5.79E-01	1.00E+00	-0.02	9.75E-01	9.97E-01
**INTS7**	-0.06	8.39E-01	1.00E+00	0.01	9.75E-01	9.97E-01
**MCM4**	-0.37	1.55E-01	1.00E+00	0.01	9.75E-01	9.97E-01
**ZMYM4**	0.29	4.31E-01	1.00E+00	0.01	9.75E-01	9.97E-01
**STARD3NL**	0.72	5.60E-02	1.00E+00	-0.01	9.75E-01	9.97E-01
**AC004801.6**	-0.67	5.46E-01	1.00E+00	0.03	9.75E-01	9.97E-01
**AC126474.1**	1.20	2.83E-01	1.00E+00	-0.03	9.75E-01	9.97E-01
**ADAMTS1**	-0.76	1.28E-01	1.00E+00	-0.02	9.75E-01	9.97E-01
**LINC00881**	-1.26	7.16E-01	1.00E+00	-0.07	9.76E-01	9.97E-01
**NECTIN4**	0.19	6.31E-01	1.00E+00	0.01	9.76E-01	9.97E-01
**CCDC40**	-1.08	4.26E-02	1.00E+00	0.02	9.76E-01	9.97E-01
**AL117332.1**	-1.07	8.03E-02	1.00E+00	0.02	9.76E-01	9.97E-01
**AP000866.1**	0.85	1.61E-01	1.00E+00	-0.03	9.76E-01	9.97E-01
**LINC01140**	1.38	1.73E-01	1.00E+00	-0.02	9.76E-01	9.97E-01
**MT-CO1**	0.06	8.92E-01	1.00E+00	-0.02	9.76E-01	9.97E-01
**GOLGB1**	-0.37	1.25E-02	6.97E-01	0.01	9.76E-01	9.97E-01
**AL137784.2**	1.96	5.66E-01	1.00E+00	0.03	9.76E-01	9.97E-01
**TACO1**	-0.15	4.94E-01	1.00E+00	-0.01	9.76E-01	9.97E-01
**A2M**	0.01	9.89E-01	1.00E+00	0.02	9.76E-01	9.97E-01
**ZNF800**	-0.13	7.08E-01	1.00E+00	-0.01	9.76E-01	9.97E-01
**PPM1H**	0.28	6.53E-01	1.00E+00	-0.02	9.76E-01	9.97E-01
**AC011297.1**	-0.01	9.96E-01	1.00E+00	0.06	9.76E-01	9.97E-01
**ATP6V0A1**	0.02	9.42E-01	1.00E+00	0.01	9.76E-01	9.97E-01
**ASAP1-IT2**	0.73	5.63E-01	1.00E+00	-0.02	9.76E-01	9.97E-01
**KCNIP1**	-0.25	6.94E-01	1.00E+00	-0.06	9.76E-01	9.97E-01
**MYO1H**	1.57	3.66E-01	1.00E+00	0.03	9.76E-01	9.97E-01
**C7orf26**	-0.16	4.30E-01	1.00E+00	0.01	9.76E-01	9.97E-01
**LINC01395**	-3.83	1.58E-02	7.86E-01	-0.03	9.76E-01	9.97E-01

**TMEM45A**	0.12	8.02E-01	1.00E+00	0.02	9.76E-01	9.97E-01
**AGPS**	0.19	4.61E-01	1.00E+00	0.01	9.76E-01	9.97E-01
**POMK**	-0.14	7.02E-01	1.00E+00	0.01	9.76E-01	9.97E-01
**SLCO4A1-AS1**	-0.21	7.14E-01	1.00E+00	0.02	9.76E-01	9.97E-01
**NELFB**	-0.20	3.71E-01	1.00E+00	-0.02	9.76E-01	9.97E-01
**AL137786.1**	-0.19	9.08E-01	1.00E+00	0.04	9.77E-01	9.97E-01
**TTC9**	0.25	5.29E-01	1.00E+00	0.02	9.77E-01	9.97E-01
**MYOZ2**	-1.35	4.17E-01	1.00E+00	0.09	9.77E-01	9.97E-01
**MKNK2**	0.03	9.22E-01	1.00E+00	-0.02	9.77E-01	9.97E-01
**PABPC3**	0.26	8.61E-01	1.00E+00	-0.02	9.77E-01	9.97E-01
**FOSB**	-2.67	5.02E-02	1.00E+00	-0.02	9.77E-01	9.97E-01
**C10orf25**	0.68	2.25E-01	1.00E+00	0.03	9.77E-01	9.97E-01
**PLEKHA6**	0.41	4.44E-01	1.00E+00	-0.02	9.77E-01	9.97E-01
**ACKR1**	-0.52	1.85E-01	1.00E+00	0.01	9.77E-01	9.97E-01
**H6PD**	-0.05	8.21E-01	1.00E+00	0.01	9.77E-01	9.97E-01
**AL049555.1**	-0.08	8.44E-01	1.00E+00	0.02	9.77E-01	9.97E-01
**ITGB5**	0.07	7.66E-01	1.00E+00	0.01	9.77E-01	9.97E-01
**AC008481.2**	-0.23	8.44E-01	1.00E+00	0.04	9.77E-01	9.97E-01
**POLR2F**	-0.14	4.46E-01	1.00E+00	0.01	9.77E-01	9.97E-01
**TSKS**	0.32	5.19E-01	1.00E+00	-0.03	9.77E-01	9.97E-01
**MRPL41**	-0.28	2.12E-01	1.00E+00	-0.01	9.77E-01	9.97E-01
**ATP6AP1L**	0.32	5.64E-01	1.00E+00	0.01	9.77E-01	9.97E-01
**CRCP**	0.09	6.85E-01	1.00E+00	-0.01	9.77E-01	9.97E-01
**CNOT10**	0.02	9.40E-01	1.00E+00	0.01	9.77E-01	9.97E-01
**MT-ND1**	0.01	9.90E-01	1.00E+00	0.01	9.77E-01	9.97E-01
**KLHDC8B**	0.41	7.28E-02	1.00E+00	0.01	9.77E-01	9.97E-01
**AL133485.2**	-1.91	4.70E-01	1.00E+00	0.04	9.77E-01	9.97E-01
**SERPINB6**	-0.12	7.39E-01	1.00E+00	0.01	9.77E-01	9.97E-01
**XLOC_005369**	0.83	6.86E-01	1.00E+00	-0.03	9.77E-01	9.97E-01
**AL033384.1**	-0.07	8.97E-01	1.00E+00	-0.02	9.77E-01	9.97E-01
**PTGES2**	-0.06	8.07E-01	1.00E+00	-0.02	9.77E-01	9.97E-01
**ZNF304**	0.14	5.27E-01	1.00E+00	-0.01	9.77E-01	9.97E-01

**GABRG3**	-0.34	7.47E-01	1.00E+00	-0.03	9.78E-01	9.97E-01
**ANKRD50**	-0.45	6.15E-02	1.00E+00	-0.01	9.78E-01	9.97E-01
**AL023881.1**	-2.22	1.98E-01	1.00E+00	0.04	9.78E-01	9.97E-01
**METTL8**	-0.37	1.95E-01	1.00E+00	-0.01	9.78E-01	9.97E-01
**AL391834.2**	-0.05	9.52E-01	1.00E+00	0.01	9.78E-01	9.97E-01
**AC012360.3**	-0.69	2.14E-01	1.00E+00	0.02	9.78E-01	9.97E-01
**TM2D2**	0.24	3.02E-01	1.00E+00	0.01	9.78E-01	9.97E-01
**PDE8A**	0.03	9.16E-01	1.00E+00	-0.01	9.78E-01	9.97E-01
**AC079250.1**	-0.26	6.18E-01	1.00E+00	-0.02	9.78E-01	9.97E-01
**PGGT1B**	0.00	9.91E-01	1.00E+00	0.01	9.78E-01	9.97E-01
**EPHA4**	-0.36	1.48E-01	1.00E+00	-0.01	9.78E-01	9.97E-01
**ADORA2BP1**	0.55	5.85E-01	1.00E+00	-0.03	9.78E-01	9.97E-01
**AC083880.1**	-0.14	9.08E-01	1.00E+00	-0.02	9.78E-01	9.97E-01
**ISOC2**	-0.37	1.83E-01	1.00E+00	0.01	9.78E-01	9.97E-01
**HMGA1P4**	-0.37	7.30E-01	1.00E+00	-0.02	9.78E-01	9.97E-01
**AC116312.1**	0.69	6.73E-01	1.00E+00	0.02	9.78E-01	9.97E-01
**KLHL17**	-0.41	3.56E-01	1.00E+00	0.02	9.78E-01	9.97E-01
**KRTAP1-1**	-1.14	7.40E-01	1.00E+00	-0.09	9.78E-01	9.97E-01
**ADAM15**	-0.11	5.42E-01	1.00E+00	-0.01	9.78E-01	9.97E-01
**CMAS**	0.05	8.69E-01	1.00E+00	-0.01	9.78E-01	9.97E-01
**FIP1L1**	0.05	8.38E-01	1.00E+00	0.01	9.79E-01	9.97E-01
**ENOX2**	0.04	8.42E-01	1.00E+00	0.01	9.79E-01	9.97E-01
**ARVCF**	-0.26	3.44E-01	1.00E+00	-0.02	9.79E-01	9.97E-01
**BCLAF1**	-0.40	1.01E-01	1.00E+00	0.01	9.79E-01	9.97E-01
**MRPL49**	0.17	3.39E-01	1.00E+00	0.00	9.79E-01	9.97E-01
**HSD17B7P2**	-0.04	9.73E-01	1.00E+00	0.01	9.79E-01	9.97E-01
**SNRK**	0.00	9.99E-01	1.00E+00	0.01	9.79E-01	9.97E-01
**AC005014.3**	-1.00	2.78E-01	1.00E+00	0.02	9.79E-01	9.97E-01
**GAS2**	-0.29	6.90E-01	1.00E+00	0.01	9.79E-01	9.97E-01
**LIN7B**	0.29	5.99E-01	1.00E+00	-0.01	9.79E-01	9.97E-01
**FIGN**	0.88	8.50E-02	1.00E+00	0.02	9.79E-01	9.97E-01
**XLOC_002561**	-0.46	4.27E-01	1.00E+00	0.02	9.79E-01	9.97E-01

**CYFIP1**	-0.13	4.75E-01	1.00E+00	0.01	9.79E-01	9.97E-01
**TEK**	0.26	6.21E-01	1.00E+00	0.01	9.79E-01	9.97E-01
**G40091**	-30.00	3.42E-18	5.54E-15	-0.08	9.79E-01	9.97E-01
**HMGN1P7**	-2.81	1.27E-01	1.00E+00	0.03	9.79E-01	9.97E-01
**AC108718.1**	-0.76	2.33E-01	1.00E+00	0.02	9.80E-01	9.97E-01
**SNORA11**	-1.33	3.47E-01	1.00E+00	0.02	9.80E-01	9.97E-01
**AL357033.1**	0.20	7.11E-01	1.00E+00	0.02	9.80E-01	9.97E-01
**KDM5B**	-0.02	9.38E-01	1.00E+00	-0.01	9.80E-01	9.97E-01
**CKAP2**	-0.06	8.27E-01	1.00E+00	0.01	9.80E-01	9.97E-01
**LSM3P3**	NA	NA	NA	0.03	9.80E-01	9.97E-01
**VAMP2**	-0.14	5.82E-01	1.00E+00	0.01	9.80E-01	9.97E-01
**LINC01058**	0.43	5.55E-01	1.00E+00	0.02	9.80E-01	9.97E-01
**AC002546.1**	3.00	1.03E-02	6.31E-01	-0.08	9.80E-01	9.97E-01
**GNL2**	-0.02	9.16E-01	1.00E+00	0.01	9.80E-01	9.97E-01
**AF165147.1**	-1.62	7.65E-02	1.00E+00	-0.02	9.80E-01	9.97E-01
**SKA2**	0.01	9.67E-01	1.00E+00	0.00	9.80E-01	9.97E-01
**ADAMTS9-AS2**	-0.49	2.73E-01	1.00E+00	0.01	9.80E-01	9.97E-01
**LINC00294**	-0.01	9.88E-01	1.00E+00	-0.01	9.80E-01	9.97E-01
**LINC02178**	-0.08	9.37E-01	1.00E+00	-0.02	9.80E-01	9.97E-01
**FAM71D**	-2.17	4.21E-01	1.00E+00	0.02	9.80E-01	9.97E-01
**G38514**	1.23	3.97E-02	1.00E+00	0.02	9.80E-01	9.97E-01
**DCP2**	0.50	6.89E-02	1.00E+00	0.01	9.81E-01	9.97E-01
**XLOC_010457**	-3.00	1.89E-01	1.00E+00	-0.04	9.81E-01	9.97E-01
**GLUD1P3**	-1.39	2.13E-03	2.39E-01	-0.01	9.81E-01	9.97E-01
**TRIR**	0.02	9.04E-01	1.00E+00	-0.02	9.81E-01	9.97E-01
**RSBN1**	0.04	8.04E-01	1.00E+00	-0.01	9.81E-01	9.97E-01
**COQ8B**	-0.15	2.92E-01	1.00E+00	0.01	9.81E-01	9.97E-01
**TRIM65**	-0.13	4.76E-01	1.00E+00	0.01	9.81E-01	9.97E-01
**EYA1**	0.47	7.09E-01	1.00E+00	-0.02	9.81E-01	9.97E-01
**ATOH8**	0.78	5.28E-02	1.00E+00	-0.02	9.81E-01	9.97E-01
**DINOL**	-3.78	1.69E-01	1.00E+00	-0.03	9.81E-01	9.97E-01
**TM4SF18**	0.04	9.48E-01	1.00E+00	0.01	9.81E-01	9.97E-01

**FAM83E**	-0.03	9.62E-01	1.00E+00	-0.07	9.81E-01	9.97E-01
**TCEAL2**	-0.18	7.43E-01	1.00E+00	-0.01	9.81E-01	9.97E-01
**DDR2**	0.89	1.73E-01	1.00E+00	0.01	9.81E-01	9.97E-01
**ELFN1-AS1**	0.90	7.96E-01	1.00E+00	0.05	9.81E-01	9.97E-01
**RPS26P15**	1.13	5.94E-01	1.00E+00	-0.02	9.81E-01	9.97E-01
**CHTF18**	-0.25	3.98E-01	1.00E+00	0.01	9.81E-01	9.97E-01
**XLOC_008694**	-0.12	8.22E-01	1.00E+00	0.01	9.81E-01	9.97E-01
**ZFP1**	0.05	8.64E-01	1.00E+00	0.01	9.81E-01	9.97E-01
**JDP2**	0.00	9.91E-01	1.00E+00	0.01	9.82E-01	9.97E-01
**STX17**	0.09	7.54E-01	1.00E+00	0.00	9.82E-01	9.97E-01
**DUSP8P5**	-0.76	8.84E-02	1.00E+00	0.03	9.82E-01	9.97E-01
**ZNF280C**	0.01	9.84E-01	1.00E+00	-0.01	9.82E-01	9.97E-01
**AC103974.1**	0.04	9.51E-01	1.00E+00	-0.02	9.82E-01	9.97E-01
**FOXRED1**	-0.14	5.39E-01	1.00E+00	0.00	9.82E-01	9.97E-01
**SCN4A**	1.58	1.25E-01	1.00E+00	-0.02	9.82E-01	9.97E-01
**RAD23A**	0.02	9.02E-01	1.00E+00	0.00	9.82E-01	9.97E-01
**KIAA0100**	0.16	4.32E-01	1.00E+00	0.02	9.82E-01	9.97E-01
**TGM4**	-1.95	2.00E-01	1.00E+00	-0.02	9.82E-01	9.97E-01
**BTG2**	-0.84	1.89E-01	1.00E+00	-0.01	9.82E-01	9.97E-01
**KRT82**	-7.78	9.90E-04	1.63E-01	0.04	9.82E-01	9.97E-01
**SDHC**	0.05	7.37E-01	1.00E+00	-0.01	9.82E-01	9.97E-01
**LINC02066**	-0.54	7.41E-01	1.00E+00	-0.03	9.82E-01	9.97E-01
**PQLC1**	-0.03	8.85E-01	1.00E+00	0.01	9.82E-01	9.97E-01
**TNFRSF11A**	-0.02	9.50E-01	1.00E+00	0.01	9.82E-01	9.97E-01
**XLOC_004822**	2.29	1.10E-01	1.00E+00	-0.02	9.82E-01	9.97E-01
**SYP**	-1.07	5.94E-02	1.00E+00	-0.02	9.82E-01	9.97E-01
**PCDHGA7**	-0.51	2.12E-01	1.00E+00	-0.02	9.82E-01	9.97E-01
**AC068888.2**	1.78	1.41E-02	7.48E-01	0.02	9.82E-01	9.97E-01
**CASKIN1**	-1.57	3.07E-04	6.69E-02	-0.07	9.82E-01	9.97E-01
**MRPL32**	0.30	1.24E-01	1.00E+00	0.01	9.82E-01	9.97E-01
**GRK3**	1.08	5.23E-02	1.00E+00	-0.01	9.82E-01	9.97E-01
**AL158071.2**	1.52	4.89E-01	1.00E+00	-0.03	9.83E-01	9.97E-01

**TRAPPC10**	-0.27	2.21E-01	1.00E+00	-0.01	9.83E-01	9.97E-01
**ALDH16A1**	-0.13	5.37E-01	1.00E+00	-0.01	9.83E-01	9.97E-01
**G2899**	1.01	1.60E-01	1.00E+00	-0.02	9.83E-01	9.97E-01
**CIAO3**	0.21	2.99E-01	1.00E+00	-0.01	9.83E-01	9.97E-01
**HNRNPK**	0.07	6.66E-01	1.00E+00	0.00	9.83E-01	9.97E-01
**GTF2IP13**	-0.67	4.29E-01	1.00E+00	-0.02	9.83E-01	9.97E-01
**ADRA1A**	0.58	4.82E-01	1.00E+00	-0.02	9.83E-01	9.97E-01
**AL133370.1**	-0.87	6.39E-01	1.00E+00	0.03	9.83E-01	9.97E-01
**CTU1**	-0.17	5.65E-01	1.00E+00	-0.02	9.83E-01	9.97E-01
**ACTG1P17**	-1.43	6.74E-01	1.00E+00	-0.02	9.83E-01	9.97E-01
**OCLNP1**	-0.14	9.38E-01	1.00E+00	-0.02	9.83E-01	9.97E-01
**ZNF558**	-0.01	9.76E-01	1.00E+00	0.01	9.83E-01	9.97E-01
**C1orf112**	-0.15	6.50E-01	1.00E+00	-0.01	9.83E-01	9.97E-01
**PRIM2**	0.23	5.10E-01	1.00E+00	-0.01	9.83E-01	9.97E-01
**VRK2**	-0.14	6.27E-01	1.00E+00	0.00	9.83E-01	9.97E-01
**PLAGL1**	0.22	4.99E-01	1.00E+00	0.01	9.83E-01	9.97E-01
**CAPN10-DT**	0.37	4.88E-01	1.00E+00	-0.02	9.83E-01	9.97E-01
**AL603840.1**	-0.47	3.50E-01	1.00E+00	0.01	9.83E-01	9.97E-01
**LINC01347**	-0.80	4.89E-01	1.00E+00	-0.02	9.83E-01	9.97E-01
**AC006026.3**	0.98	3.16E-01	1.00E+00	0.02	9.83E-01	9.97E-01
**KCNQ1OT1**	-0.17	7.46E-01	1.00E+00	-0.01	9.83E-01	9.97E-01
**ZNF883**	1.74	9.47E-03	6.06E-01	-0.04	9.83E-01	9.97E-01
**ZNF100**	0.64	1.05E-01	1.00E+00	0.01	9.83E-01	9.97E-01
**DCT**	0.15	7.69E-01	1.00E+00	-0.02	9.83E-01	9.97E-01
**LITAF**	0.21	3.86E-01	1.00E+00	0.00	9.83E-01	9.97E-01
**FOXRED2**	-0.42	1.07E-01	1.00E+00	0.01	9.84E-01	9.97E-01
**AL021807.1**	-1.88	2.17E-01	1.00E+00	-0.03	9.84E-01	9.97E-01
**LRMDA**	0.36	5.20E-01	1.00E+00	0.01	9.84E-01	9.97E-01
**RELCH**	-0.07	8.44E-01	1.00E+00	-0.01	9.84E-01	9.97E-01
**EPS8**	0.21	5.79E-01	1.00E+00	0.01	9.84E-01	9.97E-01
**RORB**	0.90	2.16E-01	1.00E+00	0.01	9.84E-01	9.97E-01
**AC092117.1**	-0.35	5.78E-01	1.00E+00	0.02	9.84E-01	9.97E-01

**AL079342.1**	0.56	8.71E-01	1.00E+00	0.02	9.84E-01	9.97E-01
**MMRN2**	0.01	9.80E-01	1.00E+00	0.01	9.84E-01	9.97E-01
**GUCY2C**	-0.09	8.74E-01	1.00E+00	-0.01	9.84E-01	9.97E-01
**SUPT7L**	-0.19	3.92E-01	1.00E+00	0.00	9.84E-01	9.97E-01
**TMEM130**	-2.29	2.53E-02	9.29E-01	0.02	9.84E-01	9.97E-01
**CEP41**	0.20	4.82E-01	1.00E+00	-0.01	9.84E-01	9.97E-01
**MBTPS2**	0.10	6.92E-01	1.00E+00	0.00	9.84E-01	9.97E-01
**AL157893.1**	0.89	3.97E-02	1.00E+00	0.02	9.84E-01	9.97E-01
**PRPF40B**	-0.53	4.35E-02	1.00E+00	0.01	9.84E-01	9.97E-01
**KBTBD2**	-0.04	8.45E-01	1.00E+00	0.01	9.84E-01	9.97E-01
**ORMDL2**	0.24	3.20E-01	1.00E+00	0.01	9.84E-01	9.97E-01
**USP6**	-0.05	9.64E-01	1.00E+00	-0.02	9.84E-01	9.97E-01
**RARS**	0.10	6.07E-01	1.00E+00	0.00	9.84E-01	9.97E-01
**AP000936.3**	1.36	3.42E-01	1.00E+00	-0.02	9.84E-01	9.97E-01
**ZNF536**	0.25	7.02E-01	1.00E+00	-0.02	9.84E-01	9.97E-01
**AC005034.5**	-0.92	3.29E-01	1.00E+00	-0.02	9.84E-01	9.97E-01
**TTC8**	-0.07	7.36E-01	1.00E+00	-0.01	9.84E-01	9.97E-01
**BREA2**	1.65	1.38E-01	1.00E+00	0.02	9.84E-01	9.97E-01
**LINC02458**	0.77	7.11E-01	1.00E+00	-0.04	9.84E-01	9.97E-01
**HBG1**	0.00	1.00E+00	NA	0.06	9.84E-01	9.97E-01
**IGF2BP2-AS1**	-0.08	9.82E-01	1.00E+00	0.06	9.84E-01	9.97E-01
**AC069148.1**	-0.16	9.23E-01	1.00E+00	-0.02	9.84E-01	9.97E-01
**SLC35F3**	0.06	9.35E-01	1.00E+00	0.05	9.84E-01	9.97E-01
**PCLAF**	0.06	8.87E-01	1.00E+00	0.01	9.84E-01	9.97E-01
**PCDHB7**	0.09	8.73E-01	1.00E+00	0.01	9.85E-01	9.98E-01
**PHF8**	0.10	6.33E-01	1.00E+00	-0.01	9.85E-01	9.98E-01
**RECQL**	-0.20	3.75E-01	1.00E+00	0.00	9.85E-01	9.98E-01
**UQCRHL**	-0.57	4.49E-01	1.00E+00	0.01	9.85E-01	9.98E-01
**GIPC1**	0.00	9.92E-01	1.00E+00	0.01	9.85E-01	9.98E-01
**CFAP54**	0.55	6.52E-01	1.00E+00	-0.05	9.85E-01	9.98E-01
**KRT84**	-2.46	1.98E-01	1.00E+00	0.02	9.85E-01	9.98E-01
**USP4**	0.08	6.63E-01	1.00E+00	0.00	9.85E-01	9.98E-01

**AP005432.2**	-0.51	5.57E-01	1.00E+00	-0.02	9.85E-01	9.98E-01
**HIVEP3**	-0.45	1.11E-01	1.00E+00	-0.01	9.85E-01	9.98E-01
**AC055813.1**	-2.66	3.12E-01	1.00E+00	0.02	9.85E-01	9.98E-01
**UQCC1**	-0.21	2.91E-01	1.00E+00	0.00	9.85E-01	9.98E-01
**CLIC5**	0.13	8.37E-01	1.00E+00	0.01	9.85E-01	9.98E-01
**RNASEH2CP1**	0.65	4.45E-01	1.00E+00	0.06	9.85E-01	9.98E-01
**KCNG3**	-1.64	3.86E-02	1.00E+00	-0.02	9.85E-01	9.98E-01
**CROT**	-0.01	9.51E-01	1.00E+00	-0.01	9.85E-01	9.98E-01
**SLMAP**	-0.16	4.99E-01	1.00E+00	0.00	9.86E-01	9.98E-01
**VSIG10L**	-0.34	2.38E-01	1.00E+00	0.01	9.86E-01	9.98E-01
**LINC00343**	1.54	6.52E-01	1.00E+00	-0.03	9.86E-01	9.98E-01
**RETREG2**	-0.01	9.73E-01	1.00E+00	0.01	9.86E-01	9.98E-01
**NRBP2**	-0.22	4.54E-01	1.00E+00	-0.01	9.86E-01	9.98E-01
**ART5**	0.82	2.51E-01	1.00E+00	-0.02	9.86E-01	9.98E-01
**GCK**	-0.37	4.75E-01	1.00E+00	-0.01	9.86E-01	9.98E-01
**AL049870.3**	-0.10	9.47E-01	1.00E+00	0.02	9.86E-01	9.98E-01
**LINC01354**	-0.12	9.13E-01	1.00E+00	-0.02	9.86E-01	9.98E-01
**AC012254.5**	-0.52	8.80E-01	1.00E+00	-0.06	9.86E-01	9.98E-01
**ACTG1P20**	-1.76	5.51E-01	1.00E+00	-0.01	9.86E-01	9.98E-01
**ACTB**	-0.47	2.51E-02	9.28E-01	0.00	9.86E-01	9.98E-01
**AC005682.1**	-0.39	2.89E-01	1.00E+00	-0.02	9.86E-01	9.98E-01
**PFAS**	0.13	7.42E-01	1.00E+00	-0.01	9.86E-01	9.98E-01
**IFT20**	0.09	6.95E-01	1.00E+00	-0.01	9.86E-01	9.98E-01
**PPFIA2**	-0.63	4.33E-01	1.00E+00	-0.01	9.86E-01	9.98E-01
**IL22RA2**	-0.36	6.46E-01	1.00E+00	0.02	9.86E-01	9.98E-01
**KCNN1**	-0.38	6.09E-01	1.00E+00	-0.02	9.86E-01	9.98E-01
**PRPF18**	0.01	9.76E-01	1.00E+00	0.00	9.86E-01	9.98E-01
**ALOX12-AS1**	-0.93	8.37E-02	1.00E+00	0.01	9.86E-01	9.98E-01
**CTDSPL2**	0.12	5.56E-01	1.00E+00	0.00	9.87E-01	9.98E-01
**MAGI1**	0.13	4.94E-01	1.00E+00	0.01	9.87E-01	9.98E-01
**ACHE**	-0.23	6.24E-01	1.00E+00	-0.04	9.87E-01	9.98E-01
**ZNF518B**	0.20	3.72E-01	1.00E+00	0.00	9.87E-01	9.98E-01

**CDCP1**	-0.18	5.86E-01	1.00E+00	-0.01	9.87E-01	9.98E-01
**KLHL25**	0.00	9.94E-01	1.00E+00	0.01	9.87E-01	9.98E-01
**SNHG8**	0.18	5.56E-01	1.00E+00	-0.01	9.87E-01	9.98E-01
**AAGAB**	0.09	5.54E-01	1.00E+00	0.00	9.87E-01	9.98E-01
**KCTD20**	0.30	1.74E-01	1.00E+00	0.00	9.87E-01	9.98E-01
**NKD1**	0.04	9.42E-01	1.00E+00	0.01	9.87E-01	9.98E-01
**KDF1**	0.01	9.77E-01	1.00E+00	0.01	9.87E-01	9.98E-01
**AC012313.6**	0.39	5.27E-01	1.00E+00	-0.01	9.87E-01	9.98E-01
**AL031673.1**	-2.48	3.85E-02	1.00E+00	-0.02	9.87E-01	9.98E-01
**GS1-24F4.2**	-0.07	9.56E-01	1.00E+00	-0.02	9.87E-01	9.98E-01
**PLEKHG4**	-0.45	1.87E-01	1.00E+00	0.01	9.87E-01	9.98E-01
**ZNF285B**	-1.18	6.01E-01	1.00E+00	-0.02	9.87E-01	9.98E-01
**AL031123.3**	-1.30	1.54E-01	1.00E+00	-0.02	9.87E-01	9.98E-01
**LRCH3**	0.09	6.92E-01	1.00E+00	0.01	9.87E-01	9.98E-01
**AP000866.4**	0.50	5.35E-01	1.00E+00	-0.02	9.87E-01	9.98E-01
**COPS5**	0.13	4.90E-01	1.00E+00	0.00	9.88E-01	9.98E-01
**SLC1A1**	0.05	9.34E-01	1.00E+00	0.01	9.88E-01	9.98E-01
**CCDC36**	-0.21	8.19E-01	1.00E+00	0.01	9.88E-01	9.98E-01
**G40216**	0.72	4.45E-01	1.00E+00	0.02	9.88E-01	9.98E-01
**AC138932.1**	-0.73	1.94E-01	1.00E+00	-0.05	9.88E-01	9.98E-01
**YBEY**	0.03	9.26E-01	1.00E+00	0.00	9.88E-01	9.98E-01
**CTXND1**	-0.32	4.39E-01	1.00E+00	0.01	9.88E-01	9.98E-01
**USP19**	-0.06	7.20E-01	1.00E+00	0.01	9.88E-01	9.98E-01
**RPL35P2**	-1.05	1.88E-01	1.00E+00	0.01	9.88E-01	9.98E-01
**DYNLL1P1**	-2.16	2.58E-01	1.00E+00	-0.01	9.88E-01	9.98E-01
**PRR9**	-4.26	1.98E-01	1.00E+00	0.05	9.88E-01	9.98E-01
**AL353583.1**	-2.35	2.09E-01	1.00E+00	-0.01	9.88E-01	9.98E-01
**XLOC_002030**	-1.55	1.15E-01	1.00E+00	0.01	9.88E-01	9.98E-01
**G36985**	-3.02	1.65E-01	1.00E+00	0.01	9.88E-01	9.98E-01
**ARSD**	-0.52	3.20E-02	1.00E+00	0.00	9.88E-01	9.98E-01
**AC012085.2**	0.08	9.56E-01	1.00E+00	-0.03	9.88E-01	9.98E-01
**PMFBP1**	-0.69	3.45E-01	1.00E+00	-0.01	9.88E-01	9.98E-01

**AC002451.1**	0.87	4.98E-01	1.00E+00	0.01	9.88E-01	9.98E-01
**CLMP**	1.27	4.09E-02	1.00E+00	-0.01	9.88E-01	9.98E-01
**TADA2B**	-0.05	7.20E-01	1.00E+00	-0.01	9.88E-01	9.98E-01
**RHBDL3**	-0.63	3.13E-01	1.00E+00	-0.04	9.88E-01	9.98E-01
**ZNF653**	-0.67	1.99E-02	8.80E-01	-0.01	9.88E-01	9.98E-01
**TBL1Y**	-0.16	8.83E-01	1.00E+00	0.03	9.88E-01	9.98E-01
**USP48**	0.12	4.63E-01	1.00E+00	0.00	9.88E-01	9.98E-01
**NKPD1**	-0.02	9.75E-01	1.00E+00	-0.01	9.88E-01	9.98E-01
**NAV2**	-0.34	5.36E-01	1.00E+00	0.01	9.88E-01	9.98E-01
**GTF2IRD1P1**	-0.85	4.83E-01	1.00E+00	-0.01	9.88E-01	9.98E-01
**METTL4**	0.06	8.47E-01	1.00E+00	0.01	9.89E-01	9.98E-01
**XLOC_008033**	0.57	6.22E-01	1.00E+00	-0.01	9.89E-01	9.98E-01
**ANO5**	0.19	8.79E-01	1.00E+00	-0.01	9.89E-01	9.98E-01
**ENDOV**	0.17	3.92E-01	1.00E+00	0.00	9.89E-01	9.98E-01
**AL606834.1**	0.72	2.01E-01	1.00E+00	-0.01	9.89E-01	9.98E-01
**ACTBL2**	0.58	8.60E-01	1.00E+00	0.05	9.89E-01	9.98E-01
**AL031719.1**	1.24	3.90E-01	1.00E+00	0.01	9.89E-01	9.98E-01
**TMEM161A**	-0.44	8.51E-02	1.00E+00	0.00	9.89E-01	9.98E-01
**PHYKPL**	0.34	2.33E-01	1.00E+00	0.00	9.89E-01	9.98E-01
**ETV4**	-0.40	1.61E-01	1.00E+00	0.01	9.89E-01	9.98E-01
**AC002558.1**	-1.59	2.64E-01	1.00E+00	-0.02	9.89E-01	9.98E-01
**WNT6**	0.02	9.71E-01	1.00E+00	0.04	9.89E-01	9.98E-01
**CHMP7**	0.22	2.22E-01	1.00E+00	0.00	9.89E-01	9.98E-01
**NPHP4**	-0.15	6.44E-01	1.00E+00	-0.01	9.89E-01	9.98E-01
**UPK3B**	1.21	7.46E-02	1.00E+00	0.01	9.89E-01	9.98E-01
**FIZ1**	-0.25	2.88E-01	1.00E+00	-0.01	9.89E-01	9.98E-01
**XLOC_007486**	2.73	2.12E-01	1.00E+00	0.04	9.89E-01	9.98E-01
**PSMG3**	-0.31	2.99E-01	1.00E+00	0.00	9.89E-01	9.98E-01
**AC005330.1**	-0.16	9.14E-01	1.00E+00	0.03	9.89E-01	9.98E-01
**IL20RB**	0.39	2.91E-01	1.00E+00	-0.01	9.89E-01	9.98E-01
**TPCN2**	-0.06	7.36E-01	1.00E+00	-0.01	9.89E-01	9.98E-01
**UHRF1BP1L**	-0.08	7.48E-01	1.00E+00	0.00	9.90E-01	9.98E-01

**ITGB1**	0.46	2.15E-01	1.00E+00	0.01	9.90E-01	9.98E-01
**XLOC_002653**	-0.38	7.29E-01	1.00E+00	-0.01	9.90E-01	9.98E-01
**SET**	-0.07	6.84E-01	1.00E+00	0.00	9.90E-01	9.98E-01
**MSH6**	-0.14	5.29E-01	1.00E+00	0.00	9.90E-01	9.98E-01
**AC027575.2**	-2.73	2.66E-01	1.00E+00	-0.01	9.90E-01	9.98E-01
**RABAC1**	0.06	9.39E-01	1.00E+00	0.01	9.90E-01	9.98E-01
**SLC35E4**	-0.08	7.36E-01	1.00E+00	0.01	9.90E-01	9.98E-01
**ZNF492**	0.09	9.09E-01	1.00E+00	0.01	9.90E-01	9.98E-01
**G5552**	0.05	9.76E-01	1.00E+00	0.01	9.90E-01	9.98E-01
**PIGS**	0.23	1.71E-01	1.00E+00	0.00	9.90E-01	9.98E-01
**ERH**	0.14	3.95E-01	1.00E+00	0.00	9.90E-01	9.98E-01
**ALX4**	-0.15	8.47E-01	1.00E+00	-0.01	9.90E-01	9.98E-01
**GJB3**	0.00	9.97E-01	1.00E+00	-0.01	9.90E-01	9.98E-01
**VAC14-AS1**	1.56	2.00E-01	1.00E+00	0.01	9.90E-01	9.98E-01
**RPS29P16**	0.56	8.71E-01	1.00E+00	0.01	9.90E-01	9.98E-01
**XLOC_009198**	-3.23	4.32E-03	3.72E-01	0.01	9.90E-01	9.98E-01
**G40866**	-2.03	5.49E-01	1.00E+00	0.01	9.90E-01	9.98E-01
**ATRX**	0.03	8.62E-01	1.00E+00	0.00	9.90E-01	9.98E-01
**C4orf46**	0.45	1.51E-01	1.00E+00	0.01	9.90E-01	9.98E-01
**AC120053.1**	0.80	3.32E-01	1.00E+00	-0.01	9.90E-01	9.98E-01
**PCNPP5**	0.98	7.78E-01	1.00E+00	-0.01	9.90E-01	9.98E-01
**ANKRD34A**	-0.30	6.79E-01	1.00E+00	-0.01	9.90E-01	9.98E-01
**TYR**	0.16	7.92E-01	1.00E+00	-0.01	9.90E-01	9.98E-01
**LINC01252**	-0.18	8.86E-01	1.00E+00	0.01	9.90E-01	9.98E-01
**LRCH1**	0.09	7.58E-01	1.00E+00	0.00	9.90E-01	9.98E-01
**CYP27C1**	-0.08	8.46E-01	1.00E+00	-0.01	9.90E-01	9.98E-01
**CAPN5**	-0.09	8.43E-01	1.00E+00	0.01	9.90E-01	9.98E-01
**AC062004.1**	0.02	9.91E-01	1.00E+00	0.04	9.91E-01	9.98E-01
**KRTAP11-1**	-6.92	3.48E-02	1.00E+00	-0.04	9.91E-01	9.98E-01
**CDAN1**	-0.02	9.32E-01	1.00E+00	-0.01	9.91E-01	9.98E-01
**TXNDC17**	-0.17	3.48E-01	1.00E+00	0.00	9.91E-01	9.98E-01
**KLHL41**	-1.41	4.47E-01	1.00E+00	0.02	9.91E-01	9.98E-01

**TXLNA**	-0.05	8.16E-01	1.00E+00	0.00	9.91E-01	9.98E-01
**DCAF1**	0.17	5.15E-01	1.00E+00	0.00	9.91E-01	9.98E-01
**BEST3**	-0.44	7.21E-01	1.00E+00	0.01	9.91E-01	9.98E-01
**ETF1**	0.09	6.63E-01	1.00E+00	0.00	9.91E-01	9.98E-01
**HOTAIR**	-0.18	7.58E-01	1.00E+00	0.01	9.91E-01	9.98E-01
**LY6G6C**	0.33	5.26E-01	1.00E+00	0.01	9.91E-01	9.98E-01
**PLXNA2**	-0.07	8.37E-01	1.00E+00	0.00	9.91E-01	9.98E-01
**RPL12P12**	-3.22	1.17E-01	1.00E+00	-0.01	9.91E-01	9.98E-01
**SUSD3**	-0.37	3.92E-01	1.00E+00	-0.01	9.91E-01	9.98E-01
**GTF2F2**	0.14	7.12E-01	1.00E+00	0.00	9.91E-01	9.98E-01
**AC004990.1**	1.07	6.54E-01	1.00E+00	-0.01	9.91E-01	9.98E-01
**MPP5**	0.25	3.14E-01	1.00E+00	0.00	9.91E-01	9.98E-01
**PDGFD**	0.85	9.11E-02	1.00E+00	-0.01	9.91E-01	9.98E-01
**G31329**	0.10	9.07E-01	1.00E+00	0.01	9.92E-01	9.98E-01
**AC018521.6**	-0.20	7.87E-01	1.00E+00	0.01	9.92E-01	9.98E-01
**CST6**	0.03	9.54E-01	1.00E+00	-0.01	9.92E-01	9.98E-01
**PEPD**	0.17	2.84E-01	1.00E+00	0.00	9.92E-01	9.98E-01
**SUPT16HP1**	1.10	7.50E-01	1.00E+00	-0.01	9.92E-01	9.98E-01
**Z69706.1**	-0.39	7.52E-01	1.00E+00	0.01	9.92E-01	9.98E-01
**TRMT6**	-0.18	5.04E-01	1.00E+00	0.00	9.92E-01	9.98E-01
**MTRR**	0.17	3.74E-01	1.00E+00	0.00	9.92E-01	9.98E-01
**XLOC_014010**	-0.56	8.73E-01	1.00E+00	0.01	9.92E-01	9.98E-01
**FTH1P15**	-10.56	2.06E-03	2.39E-01	0.01	9.92E-01	9.99E-01
**PSMA4**	-0.02	9.00E-01	1.00E+00	0.00	9.92E-01	9.99E-01
**POP7**	-0.14	5.72E-01	1.00E+00	0.00	9.92E-01	9.99E-01
**AP000911.1**	-0.94	9.41E-02	1.00E+00	0.00	9.92E-01	9.99E-01
**PFDN2**	-0.06	7.46E-01	1.00E+00	0.00	9.92E-01	9.99E-01
**SDC1**	-0.15	7.10E-01	1.00E+00	0.00	9.92E-01	9.99E-01
**B4GALT4**	0.21	3.82E-01	1.00E+00	0.00	9.92E-01	9.99E-01
**RPL17P43**	-0.48	8.87E-01	1.00E+00	-0.01	9.92E-01	9.99E-01
**SOCS2-AS1**	-0.02	9.78E-01	1.00E+00	0.01	9.92E-01	9.99E-01
**NR2E3**	2.29	4.14E-01	1.00E+00	0.01	9.93E-01	9.99E-01

**AC144530.1**	-1.79	2.23E-01	1.00E+00	0.01	9.93E-01	9.99E-01
**TMX3**	0.30	3.46E-01	1.00E+00	0.00	9.93E-01	9.99E-01
**AP003486.1**	0.67	1.48E-01	1.00E+00	0.00	9.93E-01	9.99E-01
**ATP11B**	0.22	5.62E-01	1.00E+00	0.00	9.93E-01	9.99E-01
**CHP1**	-0.04	8.30E-01	1.00E+00	0.00	9.93E-01	9.99E-01
**PTPRM**	0.04	9.19E-01	1.00E+00	0.00	9.93E-01	9.99E-01
**COL26A1**	-1.09	1.53E-01	1.00E+00	-0.01	9.93E-01	9.99E-01
**RSPRY1**	0.04	8.58E-01	1.00E+00	0.00	9.93E-01	9.99E-01
**DCAF16**	-0.16	4.83E-01	1.00E+00	0.00	9.93E-01	9.99E-01
**UGDH-AS1**	-0.07	8.83E-01	1.00E+00	0.00	9.93E-01	9.99E-01
**AC093620.1**	0.58	5.79E-01	1.00E+00	0.01	9.93E-01	9.99E-01
**STX1B**	-1.17	8.40E-02	1.00E+00	0.01	9.93E-01	9.99E-01
**AL161669.3**	-5.08	1.24E-01	1.00E+00	-0.01	9.93E-01	9.99E-01
**TAX1BP3**	1.49	2.74E-02	9.66E-01	0.01	9.93E-01	9.99E-01
**GINS4**	-0.65	3.95E-02	1.00E+00	-0.01	9.93E-01	9.99E-01
**WHAMM**	0.02	9.36E-01	1.00E+00	0.00	9.93E-01	9.99E-01
**CYBRD1**	0.67	1.67E-01	1.00E+00	0.00	9.94E-01	9.99E-01
**HRH4**	-0.88	5.74E-01	1.00E+00	-0.02	9.94E-01	9.99E-01
**ELOB**	-0.10	5.50E-01	1.00E+00	0.00	9.94E-01	9.99E-01
**FRMD8**	-0.22	4.55E-01	1.00E+00	0.00	9.94E-01	9.99E-01
**UBE2O**	-0.26	2.25E-01	1.00E+00	0.00	9.94E-01	9.99E-01
**TBCEL**	0.26	4.04E-01	1.00E+00	0.00	9.94E-01	9.99E-01
**RNFT2**	-0.82	2.80E-01	1.00E+00	0.00	9.94E-01	9.99E-01
**ZNF506**	-0.10	6.77E-01	1.00E+00	0.00	9.94E-01	9.99E-01
**WDR92**	0.22	4.80E-01	1.00E+00	0.00	9.94E-01	9.99E-01
**R3HDM1**	-0.24	2.47E-01	1.00E+00	0.00	9.94E-01	9.99E-01
**RPL5**	-0.18	5.80E-01	1.00E+00	0.00	9.94E-01	9.99E-01
**NCAM1**	-0.16	7.46E-01	1.00E+00	0.00	9.94E-01	9.99E-01
**PIKFYVE**	0.30	1.49E-01	1.00E+00	0.00	9.94E-01	9.99E-01
**AC093591.3**	0.73	5.21E-01	1.00E+00	0.01	9.94E-01	9.99E-01
**AC084859.1**	-0.49	4.64E-01	1.00E+00	-0.01	9.94E-01	9.99E-01
**MOCS3**	0.18	4.91E-01	1.00E+00	0.00	9.94E-01	9.99E-01

**FEM1B**	0.13	5.96E-01	1.00E+00	0.00	9.94E-01	9.99E-01
**KLF14**	1.48	4.94E-02	1.00E+00	-0.01	9.94E-01	9.99E-01
**STX19**	0.15	6.80E-01	1.00E+00	0.00	9.94E-01	9.99E-01
**CFAP44**	1.63	5.42E-02	1.00E+00	0.01	9.94E-01	9.99E-01
**KLF11**	-0.06	8.49E-01	1.00E+00	0.00	9.94E-01	9.99E-01
**SHISA9**	-0.14	8.67E-01	1.00E+00	-0.01	9.94E-01	9.99E-01
**KDM6A**	-0.07	6.69E-01	1.00E+00	0.00	9.94E-01	9.99E-01
**XLOC_008116**	0.39	8.20E-01	1.00E+00	0.01	9.94E-01	9.99E-01
**BEGAIN**	-0.91	1.12E-01	1.00E+00	0.01	9.94E-01	9.99E-01
**BEAN1**	0.02	9.64E-01	1.00E+00	-0.01	9.94E-01	9.99E-01
**CEP85L**	0.27	4.55E-01	1.00E+00	0.00	9.94E-01	9.99E-01
**LINC01291**	0.28	7.65E-01	1.00E+00	0.01	9.95E-01	9.99E-01
**RASA4B**	-0.30	7.09E-01	1.00E+00	0.01	9.95E-01	9.99E-01
**NUDT17**	0.27	5.85E-01	1.00E+00	0.00	9.95E-01	9.99E-01
**AAMP**	-0.02	8.82E-01	1.00E+00	0.00	9.95E-01	9.99E-01
**AC010207.1**	-3.51	5.97E-03	4.59E-01	0.01	9.95E-01	9.99E-01
**ZNF750**	-0.58	2.73E-01	1.00E+00	0.00	9.95E-01	9.99E-01
**GPR157**	-0.16	6.77E-01	1.00E+00	0.00	9.95E-01	9.99E-01
**LINC01863**	-2.44	2.78E-01	1.00E+00	0.00	9.95E-01	9.99E-01
**LINC01934**	-1.88	1.77E-01	1.00E+00	-0.01	9.95E-01	9.99E-01
**TMEM250**	0.05	8.45E-01	1.00E+00	0.00	9.95E-01	9.99E-01
**1-Dec**	-2.93	7.24E-02	1.00E+00	-0.01	9.95E-01	9.99E-01
**AL136164.3**	0.08	9.45E-01	1.00E+00	-0.01	9.95E-01	9.99E-01
**XLOC_013981**	-2.14	2.28E-01	1.00E+00	0.01	9.95E-01	9.99E-01
**ME3**	0.18	7.81E-01	1.00E+00	0.00	9.95E-01	9.99E-01
**MLF2**	-0.15	4.25E-01	1.00E+00	0.00	9.95E-01	9.99E-01
**AL035456.1**	1.44	5.18E-01	1.00E+00	0.00	9.95E-01	9.99E-01
**GLUL**	0.27	4.04E-01	1.00E+00	0.00	9.95E-01	9.99E-01
**CTF1**	0.55	2.19E-01	1.00E+00	0.00	9.96E-01	9.99E-01
**RHCE**	0.45	4.22E-01	1.00E+00	0.00	9.96E-01	9.99E-01
**NECAB2**	-3.55	3.61E-02	1.00E+00	0.01	9.96E-01	9.99E-01
**SMARCE1P5**	-0.86	8.05E-01	1.00E+00	0.00	9.96E-01	9.99E-01

**FOXL2**	0.50	5.66E-01	1.00E+00	0.02	9.96E-01	9.99E-01
**AF001548.3**	0.35	8.16E-01	1.00E+00	0.01	9.96E-01	9.99E-01
**XKR5**	-0.85	4.78E-01	1.00E+00	-0.01	9.96E-01	9.99E-01
**ATP2B2**	-1.13	1.07E-01	1.00E+00	0.00	9.96E-01	9.99E-01
**SOX11**	-4.02	1.09E-02	6.43E-01	0.00	9.96E-01	9.99E-01
**SNRNP25**	-0.43	9.36E-02	1.00E+00	0.00	9.96E-01	9.99E-01
**G34551**	-0.73	2.10E-01	1.00E+00	0.00	9.96E-01	9.99E-01
**ATP5MC1P6**	-3.61	4.00E-02	1.00E+00	0.00	9.96E-01	9.99E-01
**LINC00954**	2.33	8.51E-02	1.00E+00	0.01	9.96E-01	9.99E-01
**NUP62CL**	-0.27	6.57E-01	1.00E+00	0.00	9.96E-01	9.99E-01
**NTM**	0.52	2.43E-02	9.22E-01	0.00	9.96E-01	9.99E-01
**CDON**	0.12	7.51E-01	1.00E+00	0.00	9.96E-01	9.99E-01
**ZNF200**	0.39	2.81E-01	1.00E+00	0.00	9.96E-01	9.99E-01
**ELOA**	-0.06	8.23E-01	1.00E+00	0.00	9.97E-01	9.99E-01
**TULP3**	0.01	9.65E-01	1.00E+00	0.00	9.97E-01	9.99E-01
**HP**	1.74	4.10E-01	1.00E+00	0.01	9.97E-01	9.99E-01
**C9orf66**	0.11	8.82E-01	1.00E+00	0.00	9.97E-01	9.99E-01
**CEP192**	-0.03	8.92E-01	1.00E+00	0.00	9.97E-01	9.99E-01
**SMIM10**	-0.04	9.37E-01	1.00E+00	0.00	9.97E-01	9.99E-01
**AC004947.1**	-0.18	7.50E-01	1.00E+00	0.00	9.97E-01	9.99E-01
**HLA-DQB1**	-0.71	2.00E-01	1.00E+00	0.00	9.97E-01	1.00E+00
**SNORD17**	-1.84	5.92E-01	1.00E+00	0.00	9.97E-01	1.00E+00
**STAT5B**	0.33	2.13E-01	1.00E+00	0.00	9.97E-01	1.00E+00
**AC023157.2**	NA	NA	NA	0.00	9.97E-01	1.00E+00
**G35261**	-0.34	7.67E-01	1.00E+00	0.00	9.97E-01	1.00E+00
**MUTYH**	0.01	9.81E-01	1.00E+00	0.00	9.97E-01	1.00E+00
**KDM5D**	0.19	7.21E-01	1.00E+00	0.00	9.97E-01	1.00E+00
**AC072061.1**	0.11	8.37E-01	1.00E+00	0.00	9.97E-01	1.00E+00
**TP53AIP1**	-0.47	2.42E-01	1.00E+00	0.00	9.97E-01	1.00E+00
**TMEM273**	-0.07	8.89E-01	1.00E+00	0.00	9.97E-01	1.00E+00
**AL121983.2**	-1.82	1.34E-01	1.00E+00	0.00	9.97E-01	1.00E+00
**KIF15**	0.19	5.20E-01	1.00E+00	0.00	9.97E-01	1.00E+00

**ALG1L**	0.12	8.13E-01	1.00E+00	0.00	9.97E-01	1.00E+00
**G28274**	-1.13	3.07E-01	1.00E+00	0.00	9.98E-01	1.00E+00
**LBP**	2.63	4.34E-01	1.00E+00	0.00	9.98E-01	1.00E+00
**MLXIPL**	1.18	1.96E-01	1.00E+00	0.00	9.98E-01	1.00E+00
**NFE4**	-0.64	8.51E-01	1.00E+00	0.01	9.98E-01	1.00E+00
**ZDHHC5**	-0.18	5.56E-01	1.00E+00	0.00	9.98E-01	1.00E+00
**FBXO30**	-0.40	4.63E-02	1.00E+00	0.00	9.98E-01	1.00E+00
**UNK**	-0.07	7.45E-01	1.00E+00	0.00	9.98E-01	1.00E+00
**ETV2**	0.16	6.86E-01	1.00E+00	0.00	9.98E-01	1.00E+00
**INTS5**	-0.10	6.45E-01	1.00E+00	0.00	9.98E-01	1.00E+00
**FLAD1**	-0.19	4.51E-01	1.00E+00	0.00	9.98E-01	1.00E+00
**VRK3**	0.11	5.78E-01	1.00E+00	0.00	9.98E-01	1.00E+00
**ETV5**	0.40	3.35E-01	1.00E+00	0.00	9.98E-01	1.00E+00
**LYRM4-AS1**	-1.05	3.45E-02	1.00E+00	0.00	9.98E-01	1.00E+00
**G11315**	-1.79	4.90E-01	1.00E+00	0.01	9.98E-01	1.00E+00
**HNRNPKP4**	-0.92	6.25E-01	1.00E+00	0.00	9.98E-01	1.00E+00
**CDH23**	0.57	1.40E-01	1.00E+00	0.00	9.98E-01	1.00E+00
**LRRC37A4P**	-0.28	6.64E-01	1.00E+00	0.00	9.98E-01	1.00E+00
**AC113935.1**	-0.08	8.45E-01	1.00E+00	0.00	9.98E-01	1.00E+00
**TCAF2C**	-3.35	4.73E-02	1.00E+00	0.00	9.98E-01	1.00E+00
**VPS41**	0.12	4.91E-01	1.00E+00	0.00	9.98E-01	1.00E+00
**STK11**	-0.18	4.57E-01	1.00E+00	0.00	9.98E-01	1.00E+00
**AC011472.1**	0.23	8.06E-01	1.00E+00	0.00	9.98E-01	1.00E+00
**IQCC**	0.19	5.89E-01	1.00E+00	0.00	9.98E-01	1.00E+00
**BX088645.3**	-0.36	7.56E-01	1.00E+00	0.00	9.99E-01	1.00E+00
**POLH-AS1**	-0.58	7.14E-01	1.00E+00	0.00	9.99E-01	1.00E+00
**FZD10**	-0.20	5.39E-01	1.00E+00	0.00	9.99E-01	1.00E+00
**TGM5**	0.43	3.57E-01	1.00E+00	0.00	9.99E-01	1.00E+00
**GPC3**	0.78	1.47E-01	1.00E+00	0.00	9.99E-01	1.00E+00
**SLC17A7**	-0.70	2.50E-01	1.00E+00	0.00	9.99E-01	1.00E+00
**AL136038.5**	-1.61	6.37E-01	1.00E+00	0.00	9.99E-01	1.00E+00
**CLEC1A**	0.39	4.28E-01	1.00E+00	0.00	9.99E-01	1.00E+00

**RCBTB2**	0.05	8.93E-01	1.00E+00	0.00	9.99E-01	1.00E+00
**NTHL1**	0.11	7.14E-01	1.00E+00	0.00	9.99E-01	1.00E+00
**KCNK15**	0.86	1.86E-01	1.00E+00	0.00	9.99E-01	1.00E+00
**AC022613.2**	0.15	7.26E-01	1.00E+00	0.00	9.99E-01	1.00E+00
**FREM3**	-3.37	2.37E-01	1.00E+00	0.00	9.99E-01	1.00E+00
**MT-TR**	NA	NA	NA	0.00	9.99E-01	1.00E+00
**RTEL1-TNFRSF6B**	-1.76	2.26E-01	1.00E+00	0.00	9.99E-01	1.00E+00
**RPS6KA1**	-0.32	1.60E-01	1.00E+00	0.00	9.99E-01	1.00E+00
**NDFIP1**	0.29	3.74E-01	1.00E+00	0.00	9.99E-01	1.00E+00
**TNNT1**	0.24	5.23E-01	1.00E+00	0.00	1.00E+00	1.00E+00
**AC099336.2**	0.24	6.77E-01	1.00E+00	0.00	1.00E+00	1.00E+00
**CDK6**	0.15	5.63E-01	1.00E+00	0.00	1.00E+00	1.00E+00
**NMNAT1**	-0.28	2.68E-01	1.00E+00	0.00	1.00E+00	1.00E+00
**ZNF76**	-0.19	3.47E-01	1.00E+00	0.00	1.00E+00	1.00E+00
**AL365214.2**	3.36	2.37E-03	2.46E-01	0.00	1.00E+00	1.00E+00
**G12797**	0.79	4.40E-01	1.00E+00	0.00	1.00E+00	1.00E+00
**COL4A3BP**	0.35	6.60E-02	1.00E+00	0.00	1.00E+00	1.00E+00
**XLOC_001153**	0.75	7.85E-01	1.00E+00	0.00	1.00E+00	1.00E+00
**EMILIN3**	0.27	5.83E-01	1.00E+00	0.00	1.00E+00	1.00E+00
**CAPZA1**	0.25	4.16E-01	1.00E+00	0.00	1.00E+00	1.00E+00
**TTTY14**	0.04	9.57E-01	1.00E+00	0.00	1.00E+00	1.00E+00
**CRYBG2**	-0.36	3.78E-01	1.00E+00	0.00	1.00E+00	1.00E+00
**TXLNG**	0.31	2.45E-01	1.00E+00	0.00	1.00E+00	1.00E+00
**NBAS**	-0.28	1.50E-01	1.00E+00	0.00	1.00E+00	1.00E+00

Supplementary Table 2: Significantly differentially expressed genes under each condition (Healthy Control - HC, Lupus - L, Non-scarring - NS, and Scarring - S) for each stimulation (online access).

Supplementary Table 3: Negative (Neg) and Positive (Pos) Effect Sizes (ES) across condition comparisons (HC vs. L and S vs. NS) for each stimulation (online access).

Supplementary Table 4: Summary statistics for the differential expression analysis between scarring and non-scarring lesional skin samples from lupus patients (online access).

Supplementary Table 5: Medications reported from the time of enrollment

	**Non-damage (*n* = 13)**	**Damage (*n* = 8)**
**Medications**		
Oral prednisone	8 (62%)	5 (63%)
Hydroxychloroquine	8 (62%)	5 (63%)
Mycophenolate	3 (23%)	4 (50%)
Methotrexate	2 (15%)	0 (0%)
Azathioprine	0 (0%)	1 (13%)
Belimumab	1 (8%)	1 (13%)
Rituximab	2 (15%)	1 (13%)
Tacrolimus topical	0 (0%)	1 (13%)
